# Supplementary material for: Periodic oscillations of atmospheric electric field during snowfall in the Tokyo metropolitan area
Source: Sci Rep. 2021 Jan 26;11:2280. doi: 10.1038/s41598-021-82091-w (PMC7838252; doi:10.1038/s41598-021-82091-w)
Supplement: Supplementary file 8 — Supplementary Information 8. [file 41598_2021_82091_MOESM8_ESM.pdf]

-6.2831855	0.3416000	110.6699982
-6.2831855	0.3904000	111.4099960
-6.2831855	0.4392000	110.5499954
-6.2831855	0.4880000	109.9300003
-6.2831855	0.5368000	107.1699982
-6.2831855	0.5856000	103.2699966
-6.2831855	0.6344000	105.2600021
-6.2831855	0.6832000	106.4799957
-6.2831855	0.7320000	109.0499954
-6.2831855	0.7808000	110.6899948
-6.2831855	0.8296000	108.7999954
-6.2831855	0.8784000	105.6800003
-6.2831855	0.9272000	109.2500000
-6.2831855	0.9760000	109.7999954
-6.2831855	1.0247999	110.1999969
-6.2831855	1.0735999	108.9599991
-6.2831855	1.1223999	108.5000000
-6.2831855	1.1711999	103.9300003
-6.2831855	1.2200000	105.9700012
-6.2831855	1.2688000	106.9099960
-6.2831855	1.3176000	102.6500015
-6.2831855	1.3664000	107.9899979
-6.2831855	1.4152000	108.5800018
-6.2831855	1.4640000	110.6100006
-6.2831855	1.5128000	110.3799973
-6.2831855	1.5616000	106.5400009
-6.2831855	1.6104000	104.7600021
-6.2831855	1.6592000	110.4000015
-6.2831855	1.7079999	111.9199982
-6.2831855	1.7567999	114.8699951
-6.2831855	1.8055999	114.4399948
-6.2831855	1.8543999	118.9199982
-6.2831855	1.9031999	121.2500000
-6.2831855	1.9519999	118.4199982
-6.2831855	2.0007999	107.7999954
-6.2831855	2.0495999	102.1399994
-6.2831855	2.0983999	103.2299957
-6.2831855	2.1471999	106.9000015
-6.2831855	2.1959999	107.5299988
-6.2831855	2.2447999	107.3600006
-6.2831855	2.2935998	105.5800018
-6.2831855	2.3423998	108.6500015
-6.2831855	2.3912001	105.7799988
-6.2831855	2.4400001	104.2900009
-6.2831855	2.4888000	105.4199982
-6.2831855	2.5376000	103.4000015
-6.2831855	2.5864000	105.4099960
-6.2831855	2.6352000	107.2699966
-6.2831855	2.6840000	106.3399963
-6.2831855	2.7328000	103.9099960
-6.2831855	2.7816000	102.0000000
-6.2831855	2.8304000	105.7500000
-6.2831855	2.8792000	105.7799988
-6.2831855	2.9280000	106.4499969
-6.2831855	2.9768000	105.4700012
-6.2831855	3.0256000	102.7500000
-6.2831855	3.0743999	104.7099991
-6.2831855	3.1231999	107.9300003

-6.2831855	3.1719999	107.1399994
-6.2831855	3.2207999	105.6399994
-6.2831855	3.2695999	106.7999954
-6.2831855	3.3183999	106.4899979
-6.2831855	3.3671999	106.4499969
-6.2831855	3.4159999	101.9399948
-6.2831855	3.4647999	104.9199982
-6.2831855	3.5135999	103.8799973
-6.2831855	3.5623999	105.2399979
-6.2831855	3.6111999	104.3099976
-6.2831855	3.6599998	102.8099976
-6.2831855	3.7087998	101.7500000
-6.2831855	3.7575998	101.0299988
-6.2831855	3.8063998	104.9399948
-6.2831855	3.8551998	104.0800018
-6.2831855	3.9039998	102.8499985
-6.2831855	3.9527998	104.9000015
-6.2831855	4.0016003	104.8699951
-6.2831855	4.0503998	106.1100006
-6.2831855	4.0991998	105.7999954
-6.2831855	4.1479998	104.6500015
-6.2831855	4.1967998	102.3799973
-6.2831855	4.2455997	103.6500015
-6.2831855	4.2943997	106.6699982
-6.2831855	4.3431997	111.3399963
-6.2831855	4.3919997	110.5800018
-6.2831855	4.4407997	112.1800003
-6.2831855	4.4895997	118.1399994
-6.2831855	4.5383997	122.3899994
-6.2831855	4.5871997	123.0000000
-6.2831855	4.6359997	120.1100006
-6.2831855	4.6847997	115.0299988
-6.2831855	4.7336001	112.2099991
-6.2831855	4.7824001	110.3600006
-6.2831855	4.8312001	108.8699951
-6.2831855	4.8800001	111.4700012
-6.2831855	4.9288001	112.8600006
-6.2831855	4.9776001	103.5599976
-6.2831855	5.0264001	109.0699997
-6.2831855	5.0752001	114.2200012
-6.2831855	5.1240001	112.9099960
-6.2831855	5.1728001	108.1100006
-6.2831855	5.2216001	103.0599976
-6.2831855	5.2704000	106.5199966
-6.2831855	5.3192000	109.0899963
-6.2831855	5.3680000	109.0899963
-6.2831855	5.4168000	109.3099976
-6.2831855	5.4656000	108.5699997
-6.2831855	5.5144000	110.2600021
-6.2831855	5.5632000	106.7299957
-6.2831855	5.6120000	109.6100006
-6.2831855	5.6608000	109.1999969
-6.2831855	5.7096000	107.3399963
-6.2831855	5.7584000	109.4300003
-6.2831855	5.8072000	106.9099960
-6.2831855	5.8559999	108.3399963
-6.2831855	5.9047999	105.9599991
-6.2831855	5.9535999	108.7500000

-6.2831855	6.0023999	111.0100021
-6.2831855	6.0511999	110.0699997
-6.2831855	6.0999999	106.6999969
-6.2831855	6.1487999	107.7999954
-6.2831855	6.1975999	106.8899994
-6.2831855	6.2463999	102.8499985
-6.2831855	6.2951999	101.6899948
-6.2831855	6.3439999	105.3699951
-6.2831855	6.3927999	107.9099960
-6.2831855	6.4415998	110.7399979
-6.2831855	6.4903998	112.9499969
-6.2831855	6.5391998	109.7900009
-6.2831855	6.5879998	109.7200012
-6.2831855	6.6367998	110.6899948
-6.2831855	6.6855998	106.0599976
-6.2831855	6.7343998	106.8899994
-6.2831855	6.7831998	108.9499969
-6.2831855	6.8319998	108.2900009
-6.2831855	6.8807998	105.5100021
-6.2831855	6.9295998	99.9099960
-6.2831855	6.9783998	101.2399979
-6.2831855	7.0271997	102.7799988
-6.2831855	7.0759997	103.3600006
-6.2831855	7.1247997	99.5699997
-6.2831855	7.1735997	102.2099991
-6.2831855	7.2223997	108.5400009
-6.2831855	7.2711997	109.5199966
-6.2831855	7.3199997	106.2699966
-6.2831855	7.3687997	105.0999985
-6.2831855	7.4175997	106.5699997
-6.2831855	7.4663997	104.9000015
-6.2831855	7.5151997	100.0599976
-6.2831855	7.5639997	101.2799988
-6.2831855	7.6127996	105.6500015
-6.2831855	7.6615996	105.6299973
-6.2831855	7.7103996	104.6399994
-6.2831855	7.7591996	104.9199982
-6.2831855	7.8079996	100.2600021
-6.2831855	7.8567996	104.7999954
-6.2831855	7.9056001	106.3899994
-6.2831855	7.9544001	108.0499954
-6.2831855	8.0032005	104.6500015
-6.2831855	8.0520000	105.7999954
-6.2831855	8.1008005	105.4799957
-6.2831855	8.1496000	106.3199997
-6.2831855	8.1984005	104.0999985
-6.2831855	8.2472000	101.6699982
-6.2831855	8.2960005	101.4199982
-6.2831855	8.3448000	105.3899994
-6.2831855	8.3936005	104.7500000
-6.2831855	8.4424000	101.1199951
-6.2831855	8.4912004	99.0199966
-6.2831855	8.5400000	102.0999985
-6.2831855	8.5888004	104.7900009
-6.2831855	8.6375999	105.4300003
-6.2831855	8.6864004	106.4799957
-6.2831855	8.7351999	105.8199997
-6.2831855	8.7840004	103.4499969

-6.2831855	8.8327999	102.1199951
-6.2831855	8.8816004	102.0299988
-6.2831855	8.9303999	101.4499969
-6.2831855	8.9792004	97.6699982
-6.2831855	9.0279999	104.2600021
-6.2831855	9.0768003	105.1599960
-6.2831855	9.1255999	104.7299957
-6.2831855	9.1744003	104.8300018
-6.2831855	9.2231998	108.5800018
-6.2831855	9.2720003	108.8699951
-6.2831855	9.3207998	105.9000015
-6.2831855	9.3696003	103.4199982
-6.2831855	9.4184008	103.5899963
-6.2831855	9.4672003	105.5199966
-6.2831855	9.5160007	105.2099991
-6.2831855	9.5648003	102.9399948
-6.2831855	9.6136007	102.6699982
-6.2831855	9.6624002	102.0100021
-6.2831855	9.7112007	100.8799973
-6.2831855	9.7600002	98.2699966
-6.2831855	9.8088007	102.5599976
-6.2831855	9.8576002	104.7099991
-6.2831855	9.9064007	103.8300018
-6.2831855	9.9552002	103.4099960
-6.2831855	10.0040007	105.1100006
-6.2831855	10.0528002	103.8600006
-6.2831855	10.1016006	100.0400009
-6.2831855	10.1504002	103.9700012
-6.2831855	10.1992006	105.0899963
-6.2831855	10.2480001	104.6299973
-6.2831855	10.2968006	106.8799973
-6.2831855	10.3456001	106.4199982
-6.2831855	10.3944006	105.9599991
-6.2831855	10.4432001	103.4099960
-6.2831855	10.4920006	101.5299988
-6.2831855	10.5408001	106.9300003
-6.2831855	10.5896006	106.0800018
-6.2831855	10.6384001	104.9899979
-6.2831855	10.6872005	99.8699951
-6.2831855	10.7360001	101.8899994
-6.2831855	10.7848005	103.6699982
-6.2831855	10.8336000	101.1800003
-6.2831855	10.8824005	104.8899994
-6.2831855	10.9312000	106.3099976
-6.2831855	10.9800005	104.4300003
-6.2831855	11.0288000	105.6500015
-6.2831855	11.0776005	103.5599976
-6.2831855	11.1264000	102.6999969
-6.2831855	11.1752005	102.3799973
-6.2831855	11.2240000	103.7699966
-6.2831855	11.2728004	106.9499969
-6.2831855	11.3216000	105.1899948
-6.2831855	11.3704004	106.0699997
-6.2831855	11.4191999	106.4499969
-6.2831855	11.4680004	105.4300003
-6.2831855	11.5167999	102.8399963
-6.2831855	11.5656004	102.7999954
-6.2831855	11.6143999	104.3399963

-6.2831855	11.6632004	106.1399994
-6.2831855	11.7119999	108.4899979
-6.2831855	11.7608004	107.6699982
-6.2831855	11.8095999	104.9899979
-6.2831855	11.8584003	103.9099960
-6.2831855	11.9071999	104.3399963
-6.2831855	11.9560003	104.2600021
-6.2831855	12.0047998	104.2399979
-6.2831855	12.0536003	106.7900009
-6.2831855	12.1023998	106.1199951
-6.2831855	12.1512003	102.0599976
-6.2831855	12.1999998	105.5899963
-6.2831855	12.2488003	103.4899979
-6.2831855	12.2975998	104.4399948
-6.2831855	12.3464003	105.2299957
-6.2831855	12.3951998	104.4700012
-6.2831855	12.4440002	99.6999969
-6.2831855	12.4927998	104.1699982
-6.2831855	12.5416002	104.3199997
-6.2831855	12.5904007	103.8499985
-6.2831855	12.6392002	105.4499969
-6.2831855	12.6880007	104.4700012
-6.2831855	12.7368002	101.5000000
-6.2831855	12.7856007	103.3899994
-6.2831855	12.8344002	102.6599960
-6.2831855	12.8832006	102.0899963
-6.2831855	12.9320002	105.2999954
-6.2831855	12.9808006	102.4499969
-6.2831855	13.0296001	104.1100006
-6.2831855	13.0784006	104.1299973
-6.2831855	13.1272001	98.0100021
-6.2831855	13.1760006	102.5800018
-6.2831855	13.2248001	103.6500015
-6.2831855	13.2736006	101.5499954
-6.2831855	13.3224001	104.0100021
-6.2831855	13.3712006	105.6500015
-6.2831855	13.4200001	102.7500000
-6.2831855	13.4688005	106.2099991
-6.2831855	13.5176001	105.1299973
-6.2831855	13.5664005	102.0000000
-6.2831855	13.6152000	101.8199997
-6.2831855	13.6640005	97.7799988
-6.2831855	13.7128000	102.4700012
-6.2831855	13.7616005	103.0699997
-6.2831855	13.8104000	103.3099976
-6.2831855	13.8592005	100.4000015
-6.2831855	13.9080000	103.8600006
-6.2831855	13.9568005	104.7699966
-6.2831855	14.0056000	103.4300003
-6.2831855	14.0544004	100.1699982
-6.2831855	14.1032000	103.7099991
-6.2831855	14.1520004	105.2099991
-6.2831855	14.2007999	105.2600021
-6.2831855	14.2496004	103.3099976
-6.2831855	14.2983999	104.2999954
-6.2831855	14.3472004	98.9899979
-6.2831855	14.3959999	103.5800018
-6.2831855	14.4448004	105.1800003

-6.2831855	14.4935999	104.1699982
-6.2831855	14.5424004	103.1500015
-6.2831855	14.5911999	102.7699966
-6.2831855	14.6400003	103.3300018
-6.2831855	14.6887999	102.6100006
-6.2831855	14.7376003	101.9300003
-6.2831855	14.7863998	101.9899979
-6.2831855	14.8352003	101.3600006
-6.2831855	14.8839998	103.9399948
-6.2831855	14.9328003	106.9700012
-6.2831855	14.9815998	107.9799957
-6.2831855	15.0304003	105.7099991
-6.2770495	0.3416000	111.7399979
-6.2770495	0.3904000	111.5800018
-6.2770495	0.4392000	110.7600021
-6.2770495	0.4880000	109.8499985
-6.2770495	0.5368000	105.8899994
-6.2770495	0.5856000	103.5499954
-6.2770495	0.6344000	104.2999954
-6.2770495	0.6832000	106.0599976
-6.2770495	0.7320000	108.5000000
-6.2770495	0.7808000	110.3300018
-6.2770495	0.8296000	108.6899948
-6.2770495	0.8784000	106.2900009
-6.2770495	0.9272000	108.7099991
-6.2770495	0.9760000	109.2500000
-6.2770495	1.0247999	109.8199997
-6.2770495	1.0735999	109.0199966
-6.2770495	1.1223999	109.1599960
-6.2770495	1.1711999	103.9599991
-6.2770495	1.2200000	106.0800018
-6.2770495	1.2688000	107.0699997
-6.2770495	1.3176000	104.0599976
-6.2770495	1.3664000	108.1199951
-6.2770495	1.4152000	108.7299957
-6.2770495	1.4640000	109.7500000
-6.2770495	1.5128000	109.5100021
-6.2770495	1.5616000	106.0899963
-6.2770495	1.6104000	105.0499954
-6.2770495	1.6592000	110.8300018
-6.2770495	1.7079999	111.1800003
-6.2770495	1.7567999	114.8199997
-6.2770495	1.8055999	114.2699966
-6.2770495	1.8543999	118.2900009
-6.2770495	1.9031999	120.5899963
-6.2770495	1.9519999	117.9099960
-6.2770495	2.0007999	107.2999954
-6.2770495	2.0495999	103.3399963
-6.2770495	2.0983999	103.8799973
-6.2770495	2.1471999	105.6399994
-6.2770495	2.1959999	106.0899963
-6.2770495	2.2447999	106.5499954
-6.2770495	2.2935998	105.8399963
-6.2770495	2.3423998	108.4099960
-6.2770495	2.3912001	104.9199982
-6.2770495	2.4400001	104.3499985
-6.2770495	2.4888000	105.5499954
-6.2770495	2.5376000	104.2299957

-6.2770495	2.5864000	105.0100021
-6.2770495	2.6352000	106.7799988
-6.2770495	2.6840000	105.4899979
-6.2770495	2.7328000	102.1800003
-6.2770495	2.7816000	100.4799957
-6.2770495	2.8304000	105.3899994
-6.2770495	2.8792000	105.6199951
-6.2770495	2.9280000	106.2500000
-6.2770495	2.9768000	105.1299973
-6.2770495	3.0256000	102.4399948
-6.2770495	3.0743999	104.9199982
-6.2770495	3.1231999	108.5000000
-6.2770495	3.1719999	108.6899948
-6.2770495	3.2207999	107.0599976
-6.2770495	3.2695999	107.7399979
-6.2770495	3.3183999	107.2900009
-6.2770495	3.3671999	106.5000000
-6.2770495	3.4159999	101.8799973
-6.2770495	3.4647999	104.5299988
-6.2770495	3.5135999	103.2500000
-6.2770495	3.5623999	105.3399963
-6.2770495	3.6111999	104.2999954
-6.2770495	3.6599998	102.3499985
-6.2770495	3.7087998	101.3799973
-6.2770495	3.7575998	100.8399963
-6.2770495	3.8063998	103.8499985
-6.2770495	3.8551998	103.5899963
-6.2770495	3.9039998	102.7699966
-6.2770495	3.9527998	104.4399948
-6.2770495	4.0016003	104.5999985
-6.2770495	4.0503998	106.0299988
-6.2770495	4.0991998	106.3799973
-6.2770495	4.1479998	105.6299973
-6.2770495	4.1967998	103.4000015
-6.2770495	4.2455997	103.5100021
-6.2770495	4.2943997	105.4899979
-6.2770495	4.3431997	109.7799988
-6.2770495	4.3919997	107.5999985
-6.2770495	4.4407997	111.6999969
-6.2770495	4.4895997	118.5499954
-6.2770495	4.5383997	122.4199982
-6.2770495	4.5871997	122.3699951
-6.2770495	4.6359997	119.6199951
-6.2770495	4.6847997	115.8300018
-6.2770495	4.7336001	112.6399994
-6.2770495	4.7824001	109.8399963
-6.2770495	4.8312001	109.8199997
-6.2770495	4.8800001	112.7900009
-6.2770495	4.9288001	113.3699951
-6.2770495	4.9776001	104.6299973
-6.2770495	5.0264001	107.6599960
-6.2770495	5.0752001	114.0100021
-6.2770495	5.1240001	113.2099991
-6.2770495	5.1728001	109.0999985
-6.2770495	5.2216001	104.0699997
-6.2770495	5.2704000	106.9799957
-6.2770495	5.3192000	109.8300018
-6.2770495	5.3680000	109.4300003

-6.2770495	5.4168000	108.7799988
-6.2770495	5.4656000	108.0100021
-6.2770495	5.5144000	109.6199951
-6.2770495	5.5632000	107.2699966
-6.2770495	5.6120000	109.7699966
-6.2770495	5.6608000	108.3300018
-6.2770495	5.7096000	106.2200012
-6.2770495	5.7584000	108.2299957
-6.2770495	5.8072000	106.0599976
-6.2770495	5.8559999	108.5599976
-6.2770495	5.9047999	106.1399994
-6.2770495	5.9535999	108.2900009
-6.2770495	6.0023999	110.5199966
-6.2770495	6.0511999	109.8799973
-6.2770495	6.0999999	106.8699951
-6.2770495	6.1487999	105.8799973
-6.2770495	6.1975999	104.7699966
-6.2770495	6.2463999	102.9399948
-6.2770495	6.2951999	102.5299988
-6.2770495	6.3439999	104.2699966
-6.2770495	6.3927999	107.2500000
-6.2770495	6.4415998	110.6199951
-6.2770495	6.4903998	113.3799973
-6.2770495	6.5391998	109.8300018
-6.2770495	6.5879998	108.8099976
-6.2770495	6.6367998	110.2299957
-6.2770495	6.6855998	107.0999985
-6.2770495	6.7343998	107.5800018
-6.2770495	6.7831998	109.3199997
-6.2770495	6.8319998	108.5599976
-6.2770495	6.8807998	105.5599976
-6.2770495	6.9295998	99.0899963
-6.2770495	6.9783998	100.7600021
-6.2770495	7.0271997	102.3399963
-6.2770495	7.0759997	103.7900009
-6.2770495	7.1247997	101.4199982
-6.2770495	7.1735997	102.8699951
-6.2770495	7.2223997	107.6199951
-6.2770495	7.2711997	108.7900009
-6.2770495	7.3199997	106.1699982
-6.2770495	7.3687997	104.7799988
-6.2770495	7.4175997	106.0299988
-6.2770495	7.4663997	103.7900009
-6.2770495	7.5151997	97.6599960
-6.2770495	7.5639997	100.3899994
-6.2770495	7.6127996	104.9499969
-6.2770495	7.6615996	105.0499954
-6.2770495	7.7103996	105.0000000
-6.2770495	7.7591996	105.4399948
-6.2770495	7.8079996	100.1899948
-6.2770495	7.8567996	104.8600006
-6.2770495	7.9056001	106.2600021
-6.2770495	7.9544001	107.0299988
-6.2770495	8.0032005	103.2099991
-6.2770495	8.0520000	106.1500015
-6.2770495	8.1008005	105.6800003
-6.2770495	8.1496000	107.0800018
-6.2770495	8.1984005	105.3699951

-6.2770495	8.2472000	102.5699997
-6.2770495	8.2960005	100.7399979
-6.2770495	8.3448000	104.4899979
-6.2770495	8.3936005	104.8699951
-6.2770495	8.4424000	102.1399994
-6.2770495	8.4912004	99.0199966
-6.2770495	8.5400000	101.5499954
-6.2770495	8.5888004	104.0899963
-6.2770495	8.6375999	104.8699951
-6.2770495	8.6864004	106.9300003
-6.2770495	8.7351999	106.3199997
-6.2770495	8.7840004	103.0899963
-6.2770495	8.8327999	101.6500015
-6.2770495	8.8816004	102.4899979
-6.2770495	8.9303999	101.5899963
-6.2770495	8.9792004	98.0499954
-6.2770495	9.0279999	104.1399994
-6.2770495	9.0768003	104.4300003
-6.2770495	9.1255999	102.7699966
-6.2770495	9.1744003	104.0100021
-6.2770495	9.2231998	108.0899963
-6.2770495	9.2720003	108.1699982
-6.2770495	9.3207998	105.2099991
-6.2770495	9.3696003	103.6399994
-6.2770495	9.4184008	104.0999985
-6.2770495	9.4672003	105.6399994
-6.2770495	9.5160007	104.8600006
-6.2770495	9.5648003	103.0400009
-6.2770495	9.6136007	102.0100021
-6.2770495	9.6624002	101.7500000
-6.2770495	9.7112007	100.9199982
-6.2770495	9.7600002	99.4799957
-6.2770495	9.8088007	103.1999969
-6.2770495	9.8576002	105.3699951
-6.2770495	9.9064007	104.4799957
-6.2770495	9.9552002	102.8799973
-6.2770495	10.0040007	104.6999969
-6.2770495	10.0528002	103.7399979
-6.2770495	10.1016006	101.0400009
-6.2770495	10.1504002	103.8899994
-6.2770495	10.1992006	103.9099960
-6.2770495	10.2480001	103.6399994
-6.2770495	10.2968006	106.3499985
-6.2770495	10.3456001	106.0999985
-6.2770495	10.3944006	105.6399994
-6.2770495	10.4432001	102.6800003
-6.2770495	10.4920006	101.0400009
-6.2770495	10.5408001	106.2500000
-6.2770495	10.5896006	106.4399948
-6.2770495	10.6384001	105.1299973
-6.2770495	10.6872005	98.0100021
-6.2770495	10.7360001	101.8499985
-6.2770495	10.7848005	102.7299957
-6.2770495	10.8336000	100.6199951
-6.2770495	10.8824005	104.3399963
-6.2770495	10.9312000	106.7299957
-6.2770495	10.9800005	104.9799957
-6.2770495	11.0288000	105.6999969

-6.2770495	11.0776005	103.3300018
-6.2770495	11.1264000	102.7399979
-6.2770495	11.1752005	102.4799957
-6.2770495	11.2240000	103.2099991
-6.2770495	11.2728004	105.7500000
-6.2770495	11.3216000	103.6699982
-6.2770495	11.3704004	105.2299957
-6.2770495	11.4191999	105.9700012
-6.2770495	11.4680004	104.5400009
-6.2770495	11.5167999	101.4700012
-6.2770495	11.5656004	100.0100021
-6.2770495	11.6143999	103.3699951
-6.2770495	11.6632004	104.7999954
-6.2770495	11.7119999	107.7200012
-6.2770495	11.7608004	106.9899979
-6.2770495	11.8095999	104.5000000
-6.2770495	11.8584003	104.7900009
-6.2770495	11.9071999	103.9300003
-6.2770495	11.9560003	103.5400009
-6.2770495	12.0047998	104.7699966
-6.2770495	12.0536003	106.7399979
-6.2770495	12.1023998	107.0899963
-6.2770495	12.1512003	103.7399979
-6.2770495	12.1999998	105.3300018
-6.2770495	12.2488003	103.6399994
-6.2770495	12.2975998	104.9799957
-6.2770495	12.3464003	104.7699966
-6.2770495	12.3951998	104.2399979
-6.2770495	12.4440002	99.8099976
-6.2770495	12.4927998	102.9599991
-6.2770495	12.5416002	103.8099976
-6.2770495	12.5904007	104.0599976
-6.2770495	12.6392002	105.4499969
-6.2770495	12.6880007	104.2399979
-6.2770495	12.7368002	102.1699982
-6.2770495	12.7856007	102.5199966
-6.2770495	12.8344002	102.0800018
-6.2770495	12.8832006	102.0800018
-6.2770495	12.9320002	105.7699966
-6.2770495	12.9808006	103.3300018
-6.2770495	13.0296001	103.6699982
-6.2770495	13.0784006	103.6800003
-6.2770495	13.1272001	99.4700012
-6.2770495	13.1760006	102.8199997
-6.2770495	13.2248001	103.3399963
-6.2770495	13.2736006	101.1399994
-6.2770495	13.3224001	103.6999969
-6.2770495	13.3712006	105.2500000
-6.2770495	13.4200001	103.2999954
-6.2770495	13.4688005	106.4199982
-6.2770495	13.5176001	104.9000015
-6.2770495	13.5664005	102.1399994
-6.2770495	13.6152000	101.2299957
-6.2770495	13.6640005	97.5899963
-6.2770495	13.7128000	102.2299957
-6.2770495	13.7616005	103.6899948
-6.2770495	13.8104000	103.8399963
-6.2770495	13.8592005	100.9700012

-6.2770495	13.9080000	104.1399994
-6.2770495	13.9568005	104.5899963
-6.2770495	14.0056000	103.6199951
-6.2770495	14.0544004	101.1699982
-6.2770495	14.1032000	104.2900009
-6.2770495	14.1520004	105.3199997
-6.2770495	14.2007999	105.3799973
-6.2770495	14.2496004	102.5100021
-6.2770495	14.2983999	104.0599976
-6.2770495	14.3472004	99.8899994
-6.2770495	14.3959999	103.4300003
-6.2770495	14.4448004	105.3499985
-6.2770495	14.4935999	105.0299988
-6.2770495	14.5424004	103.1699982
-6.2770495	14.5911999	102.4099960
-6.2770495	14.6400003	102.9199982
-6.2770495	14.6887999	102.1199951
-6.2770495	14.7376003	102.8300018
-6.2770495	14.7863998	103.2099991
-6.2770495	14.8352003	102.4499969
-6.2770495	14.8839998	104.8499985
-6.2770495	14.9328003	107.0299988
-6.2770495	14.9815998	107.6999969
-6.2770495	15.0304003	105.9599991
-6.2709136	0.3416000	112.4199982
-6.2709136	0.3904000	111.7099991
-6.2709136	0.4392000	111.0800018
-6.2709136	0.4880000	109.8899994
-6.2709136	0.5368000	105.0899963
-6.2709136	0.5856000	103.9399948
-6.2709136	0.6344000	103.1299973
-6.2709136	0.6832000	105.4899979
-6.2709136	0.7320000	107.2699966
-6.2709136	0.7808000	109.6800003
-6.2709136	0.8296000	108.4099960
-6.2709136	0.8784000	106.3099976
-6.2709136	0.9272000	107.5599976
-6.2709136	0.9760000	108.6699982
-6.2709136	1.0247999	109.6299973
-6.2709136	1.0735999	109.1399994
-6.2709136	1.1223999	109.6100006
-6.2709136	1.1711999	103.7799988
-6.2709136	1.2200000	105.9300003
-6.2709136	1.2688000	107.6100006
-6.2709136	1.3176000	105.8899994
-6.2709136	1.3664000	108.6399994
-6.2709136	1.4152000	108.9199982
-6.2709136	1.4640000	108.6800003
-6.2709136	1.5128000	108.7399979
-6.2709136	1.5616000	106.1999969
-6.2709136	1.6104000	105.0400009
-6.2709136	1.6592000	111.2099991
-6.2709136	1.7079999	111.8600006
-6.2709136	1.7567999	115.2600021
-6.2709136	1.8055999	114.2900009
-6.2709136	1.8543999	118.1899948
-6.2709136	1.9031999	120.5800018
-6.2709136	1.9519999	117.7099991

-6.2709136	2.0007999	106.8499985
-6.2709136	2.0495999	104.3799973
-6.2709136	2.0983999	103.8300018
-6.2709136	2.1471999	104.4899979
-6.2709136	2.1959999	105.0299988
-6.2709136	2.2447999	105.4499969
-6.2709136	2.2935998	105.0199966
-6.2709136	2.3423998	108.0400009
-6.2709136	2.3912001	104.5800018
-6.2709136	2.4400001	104.6199951
-6.2709136	2.4888000	105.5499954
-6.2709136	2.5376000	104.8799973
-6.2709136	2.5864000	104.0599976
-6.2709136	2.6352000	105.7999954
-6.2709136	2.6840000	104.4000015
-6.2709136	2.7328000	101.7500000
-6.2709136	2.7816000	99.7699966
-6.2709136	2.8304000	105.1199951
-6.2709136	2.8792000	104.6299973
-6.2709136	2.9280000	105.0400009
-6.2709136	2.9768000	104.5599976
-6.2709136	3.0256000	102.2799988
-6.2709136	3.0743999	105.3300018
-6.2709136	3.1231999	108.7900009
-6.2709136	3.1719999	109.1199951
-6.2709136	3.2207999	107.7699966
-6.2709136	3.2695999	108.1500015
-6.2709136	3.3183999	107.7399979
-6.2709136	3.3671999	106.7399979
-6.2709136	3.4159999	102.5400009
-6.2709136	3.4647999	104.7200012
-6.2709136	3.5135999	103.8199997
-6.2709136	3.5623999	105.6299973
-6.2709136	3.6111999	104.5299988
-6.2709136	3.6599998	101.9499969
-6.2709136	3.7087998	101.0100021
-6.2709136	3.7575998	101.1800003
-6.2709136	3.8063998	102.9499969
-6.2709136	3.8551998	103.2099991
-6.2709136	3.9039998	102.2799988
-6.2709136	3.9527998	103.7399979
-6.2709136	4.0016003	104.3899994
-6.2709136	4.0503998	105.9700012
-6.2709136	4.0991998	106.6500015
-6.2709136	4.1479998	106.5899963
-6.2709136	4.1967998	104.4599991
-6.2709136	4.2455997	103.6100006
-6.2709136	4.2943997	104.7600021
-6.2709136	4.3431997	108.7900009
-6.2709136	4.3919997	106.0699997
-6.2709136	4.4407997	111.2600021
-6.2709136	4.4895997	118.4300003
-6.2709136	4.5383997	121.9899979
-6.2709136	4.5871997	121.3799973
-6.2709136	4.6359997	118.7299957
-6.2709136	4.6847997	116.3300018
-6.2709136	4.7336001	112.7299957
-6.2709136	4.7824001	108.6500015

-6.2709136	4.8312001	111.1399994
-6.2709136	4.8800001	114.6500015
-6.2709136	4.9288001	114.3799973
-6.2709136	4.9776001	106.3399963
-6.2709136	5.0264001	108.1199951
-6.2709136	5.0752001	114.0100021
-6.2709136	5.1240001	113.2799988
-6.2709136	5.1728001	109.6399994
-6.2709136	5.2216001	104.5999985
-6.2709136	5.2704000	107.6100006
-6.2709136	5.3192000	110.8699951
-6.2709136	5.3680000	109.7600021
-6.2709136	5.4168000	107.9599991
-6.2709136	5.4656000	107.5899963
-6.2709136	5.5144000	109.0499954
-6.2709136	5.5632000	107.3300018
-6.2709136	5.6120000	110.1100006
-6.2709136	5.6608000	108.4899979
-6.2709136	5.7096000	106.1800003
-6.2709136	5.7584000	106.5299988
-6.2709136	5.8072000	105.8300018
-6.2709136	5.8559999	108.3099976
-6.2709136	5.9047999	106.1399994
-6.2709136	5.9535999	108.6800003
-6.2709136	6.0023999	110.2200012
-6.2709136	6.0511999	109.5800018
-6.2709136	6.0999999	107.0899963
-6.2709136	6.1487999	104.9000015
-6.2709136	6.1975999	102.8300018
-6.2709136	6.2463999	103.1999969
-6.2709136	6.2951999	103.8899994
-6.2709136	6.3439999	102.7900009
-6.2709136	6.3927999	106.5299988
-6.2709136	6.4415998	110.6299973
-6.2709136	6.4903998	113.1999969
-6.2709136	6.5391998	109.2500000
-6.2709136	6.5879998	108.5499954
-6.2709136	6.6367998	109.1899948
-6.2709136	6.6855998	107.1599960
-6.2709136	6.7343998	107.5499954
-6.2709136	6.7831998	108.9199982
-6.2709136	6.8319998	108.1399994
-6.2709136	6.8807998	104.7200012
-6.2709136	6.9295998	98.7099991
-6.2709136	6.9783998	100.5999985
-6.2709136	7.0271997	102.3300018
-6.2709136	7.0759997	104.3300018
-6.2709136	7.1247997	102.7200012
-6.2709136	7.1735997	102.8699951
-6.2709136	7.2223997	106.4000015
-6.2709136	7.2711997	108.0000000
-6.2709136	7.3199997	105.9099960
-6.2709136	7.3687997	104.5800018
-6.2709136	7.4175997	105.7200012
-6.2709136	7.4663997	103.8099976
-6.2709136	7.5151997	96.6599960
-6.2709136	7.5639997	100.7500000
-6.2709136	7.6127996	104.9799957

-6.2709136	7.6615996	104.9000015
-6.2709136	7.7103996	105.5699997
-6.2709136	7.7591996	105.6399994
-6.2709136	7.8079996	100.0899963
-6.2709136	7.8567996	105.0899963
-6.2709136	7.9056001	105.9000015
-6.2709136	7.9544001	105.8399963
-6.2709136	8.0032005	102.8799973
-6.2709136	8.0520000	105.8399963
-6.2709136	8.1008005	105.3199997
-6.2709136	8.1496000	107.0499954
-6.2709136	8.1984005	106.0800018
-6.2709136	8.2472000	103.7600021
-6.2709136	8.2960005	101.5000000
-6.2709136	8.3448000	103.4199982
-6.2709136	8.3936005	104.3099976
-6.2709136	8.4424000	103.0899963
-6.2709136	8.4912004	100.4399948
-6.2709136	8.5400000	102.4300003
-6.2709136	8.5888004	104.2399979
-6.2709136	8.6375999	104.4599991
-6.2709136	8.6864004	106.9099960
-6.2709136	8.7351999	106.3600006
-6.2709136	8.7840004	102.8399963
-6.2709136	8.8327999	101.4000015
-6.2709136	8.8816004	102.5100021
-6.2709136	8.9303999	102.1599960
-6.2709136	8.9792004	99.1899948
-6.2709136	9.0279999	104.6100006
-6.2709136	9.0768003	104.5299988
-6.2709136	9.1255999	101.7399979
-6.2709136	9.1744003	103.0100021
-6.2709136	9.2231998	106.8699951
-6.2709136	9.2720003	107.0800018
-6.2709136	9.3207998	104.3699951
-6.2709136	9.3696003	102.0699997
-6.2709136	9.4184008	104.1999969
-6.2709136	9.4672003	104.8300018
-6.2709136	9.5160007	103.9899979
-6.2709136	9.5648003	103.1199951
-6.2709136	9.6136007	101.6399994
-6.2709136	9.6624002	102.0899963
-6.2709136	9.7112007	101.1599960
-6.2709136	9.7600002	100.1599960
-6.2709136	9.8088007	103.5400009
-6.2709136	9.8576002	105.7799988
-6.2709136	9.9064007	104.9599991
-6.2709136	9.9552002	102.5999985
-6.2709136	10.0040007	104.5599976
-6.2709136	10.0528002	103.7200012
-6.2709136	10.1016006	102.4300003
-6.2709136	10.1504002	104.0800018
-6.2709136	10.1992006	102.5400009
-6.2709136	10.2480001	102.1500015
-6.2709136	10.2968006	105.7699966
-6.2709136	10.3456001	105.5199966
-6.2709136	10.3944006	105.1100006
-6.2709136	10.4432001	102.4599991

-6.2709136	10.4920006	101.3199997
-6.2709136	10.5408001	106.1699982
-6.2709136	10.5896006	106.8199997
-6.2709136	10.6384001	104.7099991
-6.2709136	10.6872005	96.0899963
-6.2709136	10.7360001	101.9799957
-6.2709136	10.7848005	102.0800018
-6.2709136	10.8336000	100.4099960
-6.2709136	10.8824005	104.2500000
-6.2709136	10.9312000	107.0999985
-6.2709136	10.9800005	105.4099960
-6.2709136	11.0288000	105.5999985
-6.2709136	11.0776005	102.9199982
-6.2709136	11.1264000	102.6500015
-6.2709136	11.1752005	102.6599960
-6.2709136	11.2240000	102.1699982
-6.2709136	11.2728004	104.2900009
-6.2709136	11.3216000	102.7200012
-6.2709136	11.3704004	105.0000000
-6.2709136	11.4191999	105.7999954
-6.2709136	11.4680004	103.8600006
-6.2709136	11.5167999	101.2799988
-6.2709136	11.5656004	99.2699966
-6.2709136	11.6143999	102.7699966
-6.2709136	11.6632004	103.3399963
-6.2709136	11.7119999	106.5800018
-6.2709136	11.7608004	105.6699982
-6.2709136	11.8095999	103.0800018
-6.2709136	11.8584003	103.7500000
-6.2709136	11.9071999	103.4899979
-6.2709136	11.9560003	103.0699997
-6.2709136	12.0047998	105.0199966
-6.2709136	12.0536003	106.1399994
-6.2709136	12.1023998	107.0899963
-6.2709136	12.1512003	104.7500000
-6.2709136	12.1999998	104.8499985
-6.2709136	12.2488003	103.7600021
-6.2709136	12.2975998	105.3899994
-6.2709136	12.3464003	104.5599976
-6.2709136	12.3951998	104.2200012
-6.2709136	12.4440002	100.2500000
-6.2709136	12.4927998	102.0999985
-6.2709136	12.5416002	103.0699997
-6.2709136	12.5904007	104.1399994
-6.2709136	12.6392002	105.4199982
-6.2709136	12.6880007	104.4000015
-6.2709136	12.7368002	103.5199966
-6.2709136	12.7856007	103.1599960
-6.2709136	12.8344002	103.3300018
-6.2709136	12.8832006	103.4199982
-6.2709136	12.9320002	106.5999985
-6.2709136	12.9808006	103.7699966
-6.2709136	13.0296001	103.0100021
-6.2709136	13.0784006	103.4499969
-6.2709136	13.1272001	101.0499954
-6.2709136	13.1760006	103.0199966
-6.2709136	13.2248001	103.0400009
-6.2709136	13.2736006	100.8499985

-6.2709136	13.3224001	103.3899994
-6.2709136	13.3712006	104.8499985
-6.2709136	13.4200001	103.9199982
-6.2709136	13.4688005	106.6299973
-6.2709136	13.5176001	104.8699951
-6.2709136	13.5664005	102.4799957
-6.2709136	13.6152000	100.9000015
-6.2709136	13.6640005	98.2600021
-6.2709136	13.7128000	102.2900009
-6.2709136	13.7616005	104.3600006
-6.2709136	13.8104000	104.4099960
-6.2709136	13.8592005	102.0499954
-6.2709136	13.9080000	104.5999985
-6.2709136	13.9568005	104.5800018
-6.2709136	14.0056000	103.9499969
-6.2709136	14.0544004	101.5999985
-6.2709136	14.1032000	104.0999985
-6.2709136	14.1520004	104.8499985
-6.2709136	14.2007999	105.2399979
-6.2709136	14.2496004	101.8099976
-6.2709136	14.2983999	104.2200012
-6.2709136	14.3472004	101.0299988
-6.2709136	14.3959999	102.6399994
-6.2709136	14.4448004	104.6599960
-6.2709136	14.4935999	105.4099960
-6.2709136	14.5424004	103.1899948
-6.2709136	14.5911999	102.4499969
-6.2709136	14.6400003	102.4700012
-6.2709136	14.6887999	101.3699951
-6.2709136	14.7376003	103.5999985
-6.2709136	14.7863998	104.2200012
-6.2709136	14.8352003	103.4399948
-6.2709136	14.8839998	105.5999985
-6.2709136	14.9328003	107.0499954
-6.2709136	14.9815998	107.2799988
-6.2709136	15.0304003	106.2399979
-6.2647777	0.3416000	112.4099960
-6.2647777	0.3904000	110.9199982
-6.2647777	0.4392000	111.0400009
-6.2647777	0.4880000	109.6599960
-6.2647777	0.5368000	103.8899994
-6.2647777	0.5856000	103.6199951
-6.2647777	0.6344000	102.1500015
-6.2647777	0.6832000	104.7200012
-6.2647777	0.7320000	105.6999969
-6.2647777	0.7808000	108.8699951
-6.2647777	0.8296000	108.1800003
-6.2647777	0.8784000	106.4799957
-6.2647777	0.9272000	106.5299988
-6.2647777	0.9760000	108.1699982
-6.2647777	1.0247999	110.0000000
-6.2647777	1.0735999	110.0999985
-6.2647777	1.1223999	109.9000015
-6.2647777	1.1711999	103.0800018
-6.2647777	1.2200000	106.1299973
-6.2647777	1.2688000	108.1899948
-6.2647777	1.3176000	107.5100021
-6.2647777	1.3664000	108.6100006

-6.2647777	1.4152000	108.4799957
-6.2647777	1.4640000	107.3799973
-6.2647777	1.5128000	108.1999969
-6.2647777	1.5616000	106.4000015
-6.2647777	1.6104000	105.1899948
-6.2647777	1.6592000	111.3199997
-6.2647777	1.7079999	112.2500000
-6.2647777	1.7567999	115.2799988
-6.2647777	1.8055999	114.0299988
-6.2647777	1.8543999	118.7200012
-6.2647777	1.9031999	121.1199951
-6.2647777	1.9519999	117.8399963
-6.2647777	2.0007999	106.9000015
-6.2647777	2.0495999	105.6199951
-6.2647777	2.0983999	104.3600006
-6.2647777	2.1471999	104.0100021
-6.2647777	2.1959999	105.2999954
-6.2647777	2.2447999	105.2099991
-6.2647777	2.2935998	104.8300018
-6.2647777	2.3423998	107.6699982
-6.2647777	2.3912001	104.8199997
-6.2647777	2.4400001	105.5999985
-6.2647777	2.4888000	106.4399948
-6.2647777	2.5376000	105.5999985
-6.2647777	2.5864000	103.0699997
-6.2647777	2.6352000	105.3600006
-6.2647777	2.6840000	104.3199997
-6.2647777	2.7328000	101.2200012
-6.2647777	2.7816000	98.1599960
-6.2647777	2.8304000	104.0400009
-6.2647777	2.8792000	103.3300018
-6.2647777	2.9280000	103.5999985
-6.2647777	2.9768000	103.8600006
-6.2647777	3.0256000	102.3399963
-6.2647777	3.0743999	106.1500015
-6.2647777	3.1231999	108.6800003
-6.2647777	3.1719999	108.9099960
-6.2647777	3.2207999	107.9399948
-6.2647777	3.2695999	108.2399979
-6.2647777	3.3183999	107.6500015
-6.2647777	3.3671999	106.5400009
-6.2647777	3.4159999	103.2900009
-6.2647777	3.4647999	104.6599960
-6.2647777	3.5135999	103.9599991
-6.2647777	3.5623999	105.7799988
-6.2647777	3.6111999	105.1399994
-6.2647777	3.6599998	101.8600006
-6.2647777	3.7087998	101.0899963
-6.2647777	3.7575998	101.7500000
-6.2647777	3.8063998	102.1699982
-6.2647777	3.8551998	102.7200012
-6.2647777	3.9039998	102.3300018
-6.2647777	3.9527998	103.6199951
-6.2647777	4.0016003	104.6299973
-6.2647777	4.0503998	106.2299957
-6.2647777	4.0991998	106.5499954
-6.2647777	4.1479998	106.6599960
-6.2647777	4.1967998	104.4399948

-6.2647777	4.2455997	103.4599991
-6.2647777	4.2943997	104.9899979
-6.2647777	4.3431997	108.3199997
-6.2647777	4.3919997	104.9899979
-6.2647777	4.4407997	110.7399979
-6.2647777	4.4895997	118.0299988
-6.2647777	4.5383997	121.3600006
-6.2647777	4.5871997	120.3699951
-6.2647777	4.6359997	118.0699997
-6.2647777	4.6847997	116.6699982
-6.2647777	4.7336001	113.0199966
-6.2647777	4.7824001	108.0100021
-6.2647777	4.8312001	112.1899948
-6.2647777	4.8800001	115.5699997
-6.2647777	4.9288001	114.8499985
-6.2647777	4.9776001	108.0400009
-6.2647777	5.0264001	107.7600021
-6.2647777	5.0752001	113.5100021
-6.2647777	5.1240001	112.9599991
-6.2647777	5.1728001	109.8399963
-6.2647777	5.2216001	104.3199997
-6.2647777	5.2704000	106.7999954
-6.2647777	5.3192000	110.4899979
-6.2647777	5.3680000	109.6399994
-6.2647777	5.4168000	107.0999985
-6.2647777	5.4656000	106.7399979
-6.2647777	5.5144000	108.5000000
-6.2647777	5.5632000	107.9700012
-6.2647777	5.6120000	109.6299973
-6.2647777	5.6608000	108.3600006
-6.2647777	5.7096000	105.7600021
-6.2647777	5.7584000	104.9099960
-6.2647777	5.8072000	106.3199997
-6.2647777	5.8559999	108.2299957
-6.2647777	5.9047999	106.9099960
-6.2647777	5.9535999	108.3399963
-6.2647777	6.0023999	109.6899948
-6.2647777	6.0511999	109.1999969
-6.2647777	6.0999999	107.0999985
-6.2647777	6.1487999	103.9000015
-6.2647777	6.1975999	101.6199951
-6.2647777	6.2463999	103.8399963
-6.2647777	6.2951999	105.0699997
-6.2647777	6.3439999	100.9599991
-6.2647777	6.3927999	106.0499954
-6.2647777	6.4415998	110.5000000
-6.2647777	6.4903998	112.3300018
-6.2647777	6.5391998	108.3199997
-6.2647777	6.5879998	108.1599960
-6.2647777	6.6367998	108.0000000
-6.2647777	6.6855998	106.1500015
-6.2647777	6.7343998	107.1399994
-6.2647777	6.7831998	108.0999985
-6.2647777	6.8319998	107.2999954
-6.2647777	6.8807998	104.1299973
-6.2647777	6.9295998	99.2900009
-6.2647777	6.9783998	101.5199966
-6.2647777	7.0271997	102.9799957

-6.2647777	7.0759997	104.7099991
-6.2647777	7.1247997	103.1100006
-6.2647777	7.1735997	102.6299973
-6.2647777	7.2223997	104.7699966
-6.2647777	7.2711997	106.7799988
-6.2647777	7.3199997	105.6299973
-6.2647777	7.3687997	105.5699997
-6.2647777	7.4175997	106.2900009
-6.2647777	7.4663997	104.4599991
-6.2647777	7.5151997	97.1199951
-6.2647777	7.5639997	100.4700012
-6.2647777	7.6127996	104.1399994
-6.2647777	7.6615996	104.1399994
-6.2647777	7.7103996	105.6500015
-6.2647777	7.7591996	105.7200012
-6.2647777	7.8079996	99.8199997
-6.2647777	7.8567996	104.9199982
-6.2647777	7.9056001	104.9199982
-6.2647777	7.9544001	104.2600021
-6.2647777	8.0032005	102.0400009
-6.2647777	8.0520000	104.9899979
-6.2647777	8.1008005	105.4099960
-6.2647777	8.1496000	106.7900009
-6.2647777	8.1984005	106.5800018
-6.2647777	8.2472000	104.8199997
-6.2647777	8.2960005	101.9300003
-6.2647777	8.3448000	102.1500015
-6.2647777	8.3936005	103.6399994
-6.2647777	8.4424000	103.9499969
-6.2647777	8.4912004	101.5599976
-6.2647777	8.5400000	102.9799957
-6.2647777	8.5888004	104.1699982
-6.2647777	8.6375999	103.3899994
-6.2647777	8.6864004	106.5999985
-6.2647777	8.7351999	106.4000015
-6.2647777	8.7840004	103.2699966
-6.2647777	8.8327999	101.9499969
-6.2647777	8.8816004	102.6100006
-6.2647777	8.9303999	102.2699966
-6.2647777	8.9792004	100.6500015
-6.2647777	9.0279999	105.0699997
-6.2647777	9.0768003	104.2900009
-6.2647777	9.1255999	101.0000000
-6.2647777	9.1744003	102.1699982
-6.2647777	9.2231998	105.6899948
-6.2647777	9.2720003	106.2299957
-6.2647777	9.3207998	103.5699997
-6.2647777	9.3696003	99.6199951
-6.2647777	9.4184008	104.0800018
-6.2647777	9.4672003	103.8600006
-6.2647777	9.5160007	102.9899979
-6.2647777	9.5648003	102.9899979
-6.2647777	9.6136007	101.7099991
-6.2647777	9.6624002	103.0899963
-6.2647777	9.7112007	102.1699982
-6.2647777	9.7600002	101.3799973
-6.2647777	9.8088007	103.2299957
-6.2647777	9.8576002	105.5100021

-6.2647777	9.9064007	104.9499969
-6.2647777	9.9552002	102.1899948
-6.2647777	10.0040007	104.5699997
-6.2647777	10.0528002	104.8699951
-6.2647777	10.1016006	103.6599960
-6.2647777	10.1504002	103.7699966
-6.2647777	10.1992006	101.7500000
-6.2647777	10.2480001	102.1599960
-6.2647777	10.2968006	105.8199997
-6.2647777	10.3456001	104.5499954
-6.2647777	10.3944006	104.0599976
-6.2647777	10.4432001	102.4599991
-6.2647777	10.4920006	101.5699997
-6.2647777	10.5408001	105.8899994
-6.2647777	10.5896006	106.2500000
-6.2647777	10.6384001	104.0000000
-6.2647777	10.6872005	96.3799973
-6.2647777	10.7360001	102.1699982
-6.2647777	10.7848005	102.2900009
-6.2647777	10.8336000	100.8199997
-6.2647777	10.8824005	104.2099991
-6.2647777	10.9312000	107.1399994
-6.2647777	10.9800005	105.2099991
-6.2647777	11.0288000	104.7299957
-6.2647777	11.0776005	102.4799957
-6.2647777	11.1264000	102.2799988
-6.2647777	11.1752005	102.7299957
-6.2647777	11.2240000	101.0299988
-6.2647777	11.2728004	102.6800003
-6.2647777	11.3216000	101.5899963
-6.2647777	11.3704004	104.2900009
-6.2647777	11.4191999	105.1299973
-6.2647777	11.4680004	103.0899963
-6.2647777	11.5167999	101.2299957
-6.2647777	11.5656004	98.5800018
-6.2647777	11.6143999	102.2200012
-6.2647777	11.6632004	101.3099976
-6.2647777	11.7119999	105.1999969
-6.2647777	11.7608004	104.5599976
-6.2647777	11.8095999	102.1199951
-6.2647777	11.8584003	102.9599991
-6.2647777	11.9071999	103.2999954
-6.2647777	11.9560003	102.5599976
-6.2647777	12.0047998	104.5800018
-6.2647777	12.0536003	104.7900009
-6.2647777	12.1023998	106.7200012
-6.2647777	12.1512003	104.8699951
-6.2647777	12.1999998	104.0800018
-6.2647777	12.2488003	103.7699966
-6.2647777	12.2975998	105.1699982
-6.2647777	12.3464003	103.6500015
-6.2647777	12.3951998	104.5000000
-6.2647777	12.4440002	101.7200012
-6.2647777	12.4927998	101.9599991
-6.2647777	12.5416002	103.4700012
-6.2647777	12.5904007	104.4300003
-6.2647777	12.6392002	105.9499969
-6.2647777	12.6880007	105.3199997

-6.2647777	12.7368002	104.9399948
-6.2647777	12.7856007	103.5999985
-6.2647777	12.8344002	104.0499954
-6.2647777	12.8832006	103.9399948
-6.2647777	12.9320002	106.6800003
-6.2647777	12.9808006	104.0100021
-6.2647777	13.0296001	102.4199982
-6.2647777	13.0784006	103.0899963
-6.2647777	13.1272001	102.1399994
-6.2647777	13.1760006	102.7500000
-6.2647777	13.2248001	102.2799988
-6.2647777	13.2736006	100.3199997
-6.2647777	13.3224001	103.1699982
-6.2647777	13.3712006	104.5899963
-6.2647777	13.4200001	104.3399963
-6.2647777	13.4688005	106.7699966
-6.2647777	13.5176001	104.8399963
-6.2647777	13.5664005	102.4000015
-6.2647777	13.6152000	100.5499954
-6.2647777	13.6640005	98.3699951
-6.2647777	13.7128000	102.2799988
-6.2647777	13.7616005	104.6999969
-6.2647777	13.8104000	104.6999969
-6.2647777	13.8592005	102.8799973
-6.2647777	13.9080000	105.0000000
-6.2647777	13.9568005	104.9700012
-6.2647777	14.0056000	103.6100006
-6.2647777	14.0544004	102.0299988
-6.2647777	14.1032000	104.2600021
-6.2647777	14.1520004	104.5699997
-6.2647777	14.2007999	105.0999985
-6.2647777	14.2496004	101.7900009
-6.2647777	14.2983999	104.3399963
-6.2647777	14.3472004	102.0599976
-6.2647777	14.3959999	102.0100021
-6.2647777	14.4448004	104.1999969
-6.2647777	14.4935999	106.3699951
-6.2647777	14.5424004	104.4300003
-6.2647777	14.5911999	103.1299973
-6.2647777	14.6400003	102.6699982
-6.2647777	14.6887999	101.5000000
-6.2647777	14.7376003	104.6599960
-6.2647777	14.7863998	105.0499954
-6.2647777	14.8352003	103.8899994
-6.2647777	14.8839998	105.3099976
-6.2647777	14.9328003	106.2699966
-6.2647777	14.9815998	106.1699982
-6.2647777	15.0304003	106.4000015
-6.2586417	0.3416000	111.8199997
-6.2586417	0.3904000	109.7799988
-6.2586417	0.4392000	110.9899979
-6.2586417	0.4880000	109.3600006
-6.2586417	0.5368000	102.6800003
-6.2586417	0.5856000	103.3300018
-6.2586417	0.6344000	101.1899948
-6.2586417	0.6832000	104.4000015
-6.2586417	0.7320000	104.5899963
-6.2586417	0.7808000	108.1800003

-6.2586417	0.8296000	108.0800018
-6.2586417	0.8784000	106.6800003
-6.2586417	0.9272000	105.8199997
-6.2586417	0.9760000	108.2799988
-6.2586417	1.0247999	110.9700012
-6.2586417	1.0735999	111.2999954
-6.2586417	1.1223999	110.2600021
-6.2586417	1.1711999	103.7500000
-6.2586417	1.2200000	106.0499954
-6.2586417	1.2688000	107.6899948
-6.2586417	1.3176000	107.9499969
-6.2586417	1.3664000	108.3199997
-6.2586417	1.4152000	107.0699997
-6.2586417	1.4640000	105.3199997
-6.2586417	1.5128000	107.5800018
-6.2586417	1.5616000	106.6800003
-6.2586417	1.6104000	106.8699951
-6.2586417	1.6592000	112.3099976
-6.2586417	1.7079999	113.6399994
-6.2586417	1.7567999	115.5999985
-6.2586417	1.8055999	113.4199982
-6.2586417	1.8543999	118.6100006
-6.2586417	1.9031999	120.9700012
-6.2586417	1.9519999	117.3799973
-6.2586417	2.0007999	105.9700012
-6.2586417	2.0495999	105.7900009
-6.2586417	2.0983999	104.7200012
-6.2586417	2.1471999	104.5100021
-6.2586417	2.1959999	105.8600006
-6.2586417	2.2447999	104.9599991
-6.2586417	2.2935998	104.2699966
-6.2586417	2.3423998	107.2200012
-6.2586417	2.3912001	105.3899994
-6.2586417	2.4400001	106.2399979
-6.2586417	2.4888000	106.9000015
-6.2586417	2.5376000	106.4000015
-6.2586417	2.5864000	103.1399994
-6.2586417	2.6352000	104.8699951
-6.2586417	2.6840000	103.9099960
-6.2586417	2.7328000	100.5499954
-6.2586417	2.7816000	97.9899979
-6.2586417	2.8304000	102.6399994
-6.2586417	2.8792000	102.2200012
-6.2586417	2.9280000	102.7299957
-6.2586417	2.9768000	103.9199982
-6.2586417	3.0256000	101.9499969
-6.2586417	3.0743999	105.8099976
-6.2586417	3.1231999	107.3300018
-6.2586417	3.1719999	108.1299973
-6.2586417	3.2207999	107.8699951
-6.2586417	3.2695999	108.6800003
-6.2586417	3.3183999	108.3899994
-6.2586417	3.3671999	106.5100021
-6.2586417	3.4159999	103.3899994
-6.2586417	3.4647999	103.6399994
-6.2586417	3.5135999	103.9499969
-6.2586417	3.5623999	105.6599960
-6.2586417	3.6111999	105.0499954

-6.2586417	3.6599998	101.2600021
-6.2586417	3.7087998	101.4499969
-6.2586417	3.7575998	102.3799973
-6.2586417	3.8063998	101.5299988
-6.2586417	3.8551998	102.2500000
-6.2586417	3.9039998	102.5999985
-6.2586417	3.9527998	104.6299973
-6.2586417	4.0016003	105.7799988
-6.2586417	4.0503998	106.4499969
-6.2586417	4.0991998	105.7799988
-6.2586417	4.1479998	105.3799973
-6.2586417	4.1967998	103.5800018
-6.2586417	4.2455997	102.9799957
-6.2586417	4.2943997	105.2900009
-6.2586417	4.3431997	108.3600006
-6.2586417	4.3919997	104.5800018
-6.2586417	4.4407997	109.6999969
-6.2586417	4.4895997	117.5899963
-6.2586417	4.5383997	120.9000015
-6.2586417	4.5871997	119.3399963
-6.2586417	4.6359997	116.8199997
-6.2586417	4.6847997	116.6100006
-6.2586417	4.7336001	112.6500015
-6.2586417	4.7824001	107.6299973
-6.2586417	4.8312001	113.0499954
-6.2586417	4.8800001	116.2099991
-6.2586417	4.9288001	114.8600006
-6.2586417	4.9776001	109.2600021
-6.2586417	5.0264001	108.2799988
-6.2586417	5.0752001	113.1199951
-6.2586417	5.1240001	112.4899979
-6.2586417	5.1728001	110.0699997
-6.2586417	5.2216001	105.4899979
-6.2586417	5.2704000	106.7500000
-6.2586417	5.3192000	110.1500015
-6.2586417	5.3680000	109.7799988
-6.2586417	5.4168000	106.9700012
-6.2586417	5.4656000	106.5299988
-6.2586417	5.5144000	108.0599976
-6.2586417	5.5632000	107.8199997
-6.2586417	5.6120000	109.1699982
-6.2586417	5.6608000	108.7500000
-6.2586417	5.7096000	106.0800018
-6.2586417	5.7584000	103.4700012
-6.2586417	5.8072000	106.6599960
-6.2586417	5.8559999	107.7600021
-6.2586417	5.9047999	106.9199982
-6.2586417	5.9535999	107.2600021
-6.2586417	6.0023999	108.2299957
-6.2586417	6.0511999	108.6899948
-6.2586417	6.0999999	107.2999954
-6.2586417	6.1487999	103.4799957
-6.2586417	6.1975999	100.9000015
-6.2586417	6.2463999	104.5599976
-6.2586417	6.2951999	105.4700012
-6.2586417	6.3439999	99.6199951
-6.2586417	6.3927999	105.9199982
-6.2586417	6.4415998	110.1199951

-6.2586417	6.4903998	110.9399948
-6.2586417	6.5391998	106.5999985
-6.2586417	6.5879998	107.2500000
-6.2586417	6.6367998	106.8099976
-6.2586417	6.6855998	105.0499954
-6.2586417	6.7343998	106.1599960
-6.2586417	6.7831998	106.8300018
-6.2586417	6.8319998	105.8799973
-6.2586417	6.8807998	103.0699997
-6.2586417	6.9295998	99.7399979
-6.2586417	6.9783998	103.0000000
-6.2586417	7.0271997	103.6800003
-6.2586417	7.0759997	105.0400009
-6.2586417	7.1247997	103.5599976
-6.2586417	7.1735997	103.2299957
-6.2586417	7.2223997	103.5400009
-6.2586417	7.2711997	105.6599960
-6.2586417	7.3199997	105.3600006
-6.2586417	7.3687997	105.6800003
-6.2586417	7.4175997	106.0400009
-6.2586417	7.4663997	104.6699982
-6.2586417	7.5151997	97.5899963
-6.2586417	7.5639997	100.6399994
-6.2586417	7.6127996	103.6199951
-6.2586417	7.6615996	103.5199966
-6.2586417	7.7103996	105.5400009
-6.2586417	7.7591996	105.3799973
-6.2586417	7.8079996	99.3799973
-6.2586417	7.8567996	104.9199982
-6.2586417	7.9056001	104.3399963
-6.2586417	7.9544001	103.5299988
-6.2586417	8.0032005	100.8899994
-6.2586417	8.0520000	103.4399948
-6.2586417	8.1008005	105.1100006
-6.2586417	8.1496000	106.5299988
-6.2586417	8.1984005	106.6899948
-6.2586417	8.2472000	105.5100021
-6.2586417	8.2960005	102.0400009
-6.2586417	8.3448000	100.9499969
-6.2586417	8.3936005	102.8699951
-6.2586417	8.4424000	104.2500000
-6.2586417	8.4912004	102.7200012
-6.2586417	8.5400000	103.6100006
-6.2586417	8.5888004	103.8300018
-6.2586417	8.6375999	102.1100006
-6.2586417	8.6864004	105.4499969
-6.2586417	8.7351999	105.6999969
-6.2586417	8.7840004	103.3499985
-6.2586417	8.8327999	102.1899948
-6.2586417	8.8816004	103.0299988
-6.2586417	8.9303999	102.9499969
-6.2586417	8.9792004	101.8099976
-6.2586417	9.0279999	105.4499969
-6.2586417	9.0768003	104.4499969
-6.2586417	9.1255999	101.3499985
-6.2586417	9.1744003	102.3600006
-6.2586417	9.2231998	104.8899994
-6.2586417	9.2720003	105.4199982

-6.2586417	9.3207998	102.7900009
-6.2586417	9.3696003	97.2399979
-6.2586417	9.4184008	103.5000000
-6.2586417	9.4672003	102.2699966
-6.2586417	9.5160007	101.7399979
-6.2586417	9.5648003	102.8499985
-6.2586417	9.6136007	102.0499954
-6.2586417	9.6624002	104.2200012
-6.2586417	9.7112007	103.1299973
-6.2586417	9.7600002	102.4199982
-6.2586417	9.8088007	103.0499954
-6.2586417	9.8576002	105.2600021
-6.2586417	9.9064007	104.8600006
-6.2586417	9.9552002	102.6599960
-6.2586417	10.0040007	104.8899994
-6.2586417	10.0528002	105.2900009
-6.2586417	10.1016006	103.8799973
-6.2586417	10.1504002	103.4599991
-6.2586417	10.1992006	102.0199966
-6.2586417	10.2480001	102.2900009
-6.2586417	10.2968006	105.2200012
-6.2586417	10.3456001	103.1800003
-6.2586417	10.3944006	102.8899994
-6.2586417	10.4432001	102.7699966
-6.2586417	10.4920006	102.0699997
-6.2586417	10.5408001	105.3199997
-6.2586417	10.5896006	105.2600021
-6.2586417	10.6384001	102.9899979
-6.2586417	10.6872005	96.8399963
-6.2586417	10.7360001	101.5699997
-6.2586417	10.7848005	102.2799988
-6.2586417	10.8336000	101.3600006
-6.2586417	10.8824005	103.9599991
-6.2586417	10.9312000	106.4799957
-6.2586417	10.9800005	104.4700012
-6.2586417	11.0288000	103.8600006
-6.2586417	11.0776005	102.0999985
-6.2586417	11.1264000	101.8899994
-6.2586417	11.1752005	102.7799988
-6.2586417	11.2240000	100.9399948
-6.2586417	11.2728004	101.5100021
-6.2586417	11.3216000	101.8899994
-6.2586417	11.3704004	103.7399979
-6.2586417	11.4191999	103.9799957
-6.2586417	11.4680004	102.3399963
-6.2586417	11.5167999	101.5599976
-6.2586417	11.5656004	98.1599960
-6.2586417	11.6143999	101.8499985
-6.2586417	11.6632004	101.2200012
-6.2586417	11.7119999	104.2699966
-6.2586417	11.7608004	103.6399994
-6.2586417	11.8095999	101.5499954
-6.2586417	11.8584003	103.0000000
-6.2586417	11.9071999	103.4300003
-6.2586417	11.9560003	102.0899963
-6.2586417	12.0047998	103.1999969
-6.2586417	12.0536003	103.8300018
-6.2586417	12.1023998	106.0199966

-6.2586417	12.1512003	104.7600021
-6.2586417	12.1999998	103.7799988
-6.2586417	12.2488003	103.8300018
-6.2586417	12.2975998	104.7699966
-6.2586417	12.3464003	102.2099991
-6.2586417	12.3951998	104.1299973
-6.2586417	12.4440002	103.1399994
-6.2586417	12.4927998	101.2799988
-6.2586417	12.5416002	103.5599976
-6.2586417	12.5904007	105.3199997
-6.2586417	12.6392002	106.1599960
-6.2586417	12.6880007	105.8799973
-6.2586417	12.7368002	105.9399948
-6.2586417	12.7856007	104.1299973
-6.2586417	12.8344002	104.5499954
-6.2586417	12.8832006	103.8499985
-6.2586417	12.9320002	106.2799988
-6.2586417	12.9808006	104.6199951
-6.2586417	13.0296001	102.4099960
-6.2586417	13.0784006	102.6599960
-6.2586417	13.1272001	102.4300003
-6.2586417	13.1760006	101.0800018
-6.2586417	13.2248001	101.3799973
-6.2586417	13.2736006	100.8499985
-6.2586417	13.3224001	103.0000000
-6.2586417	13.3712006	104.1500015
-6.2586417	13.4200001	104.5899963
-6.2586417	13.4688005	106.7299957
-6.2586417	13.5176001	104.7699966
-6.2586417	13.5664005	102.4599991
-6.2586417	13.6152000	100.8300018
-6.2586417	13.6640005	98.7999954
-6.2586417	13.7128000	102.2099991
-6.2586417	13.7616005	104.3499985
-6.2586417	13.8104000	104.5199966
-6.2586417	13.8592005	103.1699982
-6.2586417	13.9080000	104.0199966
-6.2586417	13.9568005	104.0599976
-6.2586417	14.0056000	101.4499969
-6.2586417	14.0544004	101.5699997
-6.2586417	14.1032000	104.4000015
-6.2586417	14.1520004	104.0699997
-6.2586417	14.2007999	104.1899948
-6.2586417	14.2496004	101.3799973
-6.2586417	14.2983999	104.5599976
-6.2586417	14.3472004	103.1100006
-6.2586417	14.3959999	102.1399994
-6.2586417	14.4448004	104.3600006
-6.2586417	14.4935999	107.4099960
-6.2586417	14.5424004	105.4000015
-6.2586417	14.5911999	103.6399994
-6.2586417	14.6400003	102.5800018
-6.2586417	14.6887999	101.5199966
-6.2586417	14.7376003	105.4899979
-6.2586417	14.7863998	105.6599960
-6.2586417	14.8352003	104.4599991
-6.2586417	14.8839998	105.2399979
-6.2586417	14.9328003	105.9399948

-6.2586417	14.9815998	106.2799988
-6.2586417	15.0304003	106.9000015
-6.2525058	0.3416000	111.1599960
-6.2525058	0.3904000	108.6199951
-6.2525058	0.4392000	110.6699982
-6.2525058	0.4880000	108.9599991
-6.2525058	0.5368000	101.5899963
-6.2525058	0.5856000	101.8399963
-6.2525058	0.6344000	100.4199982
-6.2525058	0.6832000	104.5599976
-6.2525058	0.7320000	103.7299957
-6.2525058	0.7808000	108.0299988
-6.2525058	0.8296000	108.7799988
-6.2525058	0.8784000	107.2200012
-6.2525058	0.9272000	105.9599991
-6.2525058	0.9760000	109.2799988
-6.2525058	1.0247999	111.6399994
-6.2525058	1.0735999	111.3300018
-6.2525058	1.1223999	110.0299988
-6.2525058	1.1711999	103.9799957
-6.2525058	1.2200000	105.5800018
-6.2525058	1.2688000	106.3099976
-6.2525058	1.3176000	107.9399948
-6.2525058	1.3664000	107.4300003
-6.2525058	1.4152000	105.8199997
-6.2525058	1.4640000	104.0999985
-6.2525058	1.5128000	106.5699997
-6.2525058	1.5616000	106.5599976
-6.2525058	1.6104000	108.0199966
-6.2525058	1.6592000	112.5400009
-6.2525058	1.7079999	114.6899948
-6.2525058	1.7567999	115.4799957
-6.2525058	1.8055999	113.6599960
-6.2525058	1.8543999	118.4399948
-6.2525058	1.9031999	120.6699982
-6.2525058	1.9519999	116.8600006
-6.2525058	2.0007999	105.1100006
-6.2525058	2.0495999	105.9599991
-6.2525058	2.0983999	104.6500015
-6.2525058	2.1471999	105.3300018
-6.2525058	2.1959999	107.4000015
-6.2525058	2.2447999	106.3099976
-6.2525058	2.2935998	104.7999954
-6.2525058	2.3423998	107.1599960
-6.2525058	2.3912001	105.5199966
-6.2525058	2.4400001	106.2799988
-6.2525058	2.4888000	107.0800018
-6.2525058	2.5376000	106.8899994
-6.2525058	2.5864000	102.7299957
-6.2525058	2.6352000	104.4899979
-6.2525058	2.6840000	104.4199982
-6.2525058	2.7328000	101.6999969
-6.2525058	2.7816000	98.6899948
-6.2525058	2.8304000	102.3300018
-6.2525058	2.8792000	102.4000015
-6.2525058	2.9280000	102.3499985
-6.2525058	2.9768000	104.3499985
-6.2525058	3.0256000	102.6999969

-6.2525058	3.0743999	104.8300018
-6.2525058	3.1231999	105.0899963
-6.2525058	3.1719999	106.7699966
-6.2525058	3.2207999	107.5199966
-6.2525058	3.2695999	108.4599991
-6.2525058	3.3183999	108.4399948
-6.2525058	3.3671999	106.2999954
-6.2525058	3.4159999	103.7799988
-6.2525058	3.4647999	102.9000015
-6.2525058	3.5135999	104.2200012
-6.2525058	3.5623999	105.6500015
-6.2525058	3.6111999	105.5199966
-6.2525058	3.6599998	101.8899994
-6.2525058	3.7087998	101.4899979
-6.2525058	3.7575998	103.2399979
-6.2525058	3.8063998	101.6999969
-6.2525058	3.8551998	102.1100006
-6.2525058	3.9039998	102.1899948
-6.2525058	3.9527998	104.8099976
-6.2525058	4.0016003	105.7699966
-6.2525058	4.0503998	106.2299957
-6.2525058	4.0991998	105.2600021
-6.2525058	4.1479998	104.3399963
-6.2525058	4.1967998	102.9000015
-6.2525058	4.2455997	102.7299957
-6.2525058	4.2943997	105.8799973
-6.2525058	4.3431997	109.0800018
-6.2525058	4.3919997	104.7699966
-6.2525058	4.4407997	109.8300018
-6.2525058	4.4895997	117.5699997
-6.2525058	4.5383997	120.3799973
-6.2525058	4.5871997	118.2799988
-6.2525058	4.6359997	115.9899979
-6.2525058	4.6847997	116.1399994
-6.2525058	4.7336001	112.2299957
-6.2525058	4.7824001	107.0699997
-6.2525058	4.8312001	113.8199997
-6.2525058	4.8800001	116.7699966
-6.2525058	4.9288001	114.6100006
-6.2525058	4.9776001	109.0100021
-6.2525058	5.0264001	107.9399948
-6.2525058	5.0752001	112.1800003
-6.2525058	5.1240001	111.8300018
-6.2525058	5.1728001	109.8799973
-6.2525058	5.2216001	106.2200012
-6.2525058	5.2704000	107.1999969
-6.2525058	5.3192000	110.2299957
-6.2525058	5.3680000	110.1800003
-6.2525058	5.4168000	107.0699997
-6.2525058	5.4656000	106.3799973
-6.2525058	5.5144000	108.0199966
-6.2525058	5.5632000	107.6399994
-6.2525058	5.6120000	108.6100006
-6.2525058	5.6608000	109.0999985
-6.2525058	5.7096000	106.4899979
-6.2525058	5.7584000	101.6699982
-6.2525058	5.8072000	106.5000000
-6.2525058	5.8559999	106.9399948

-6.2525058	5.9047999	106.9300003
-6.2525058	5.9535999	106.0299988
-6.2525058	6.0023999	106.4899979
-6.2525058	6.0511999	107.7900009
-6.2525058	6.0999999	106.9199982
-6.2525058	6.1487999	103.5400009
-6.2525058	6.1975999	100.4099960
-6.2525058	6.2463999	105.0699997
-6.2525058	6.2951999	106.0199966
-6.2525058	6.3439999	100.1699982
-6.2525058	6.3927999	106.6399994
-6.2525058	6.4415998	110.5699997
-6.2525058	6.4903998	110.2099991
-6.2525058	6.5391998	106.5299988
-6.2525058	6.5879998	107.0000000
-6.2525058	6.6367998	105.7500000
-6.2525058	6.6855998	103.4499969
-6.2525058	6.7343998	104.8300018
-6.2525058	6.7831998	105.6699982
-6.2525058	6.8319998	104.4899979
-6.2525058	6.8807998	101.9700012
-6.2525058	6.9295998	101.3799973
-6.2525058	6.9783998	104.4000015
-6.2525058	7.0271997	105.0000000
-6.2525058	7.0759997	105.3799973
-6.2525058	7.1247997	103.9799957
-6.2525058	7.1735997	104.1999969
-6.2525058	7.2223997	102.5899963
-6.2525058	7.2711997	104.7600021
-6.2525058	7.3199997	105.6500015
-6.2525058	7.3687997	105.9599991
-6.2525058	7.4175997	105.8099976
-6.2525058	7.4663997	105.0199966
-6.2525058	7.5151997	99.5699997
-6.2525058	7.5639997	100.9499969
-6.2525058	7.6127996	103.3699951
-6.2525058	7.6615996	103.7799988
-6.2525058	7.7103996	105.1100006
-6.2525058	7.7591996	104.7399979
-6.2525058	7.8079996	101.0899963
-6.2525058	7.8567996	104.7900009
-6.2525058	7.9056001	103.5800018
-6.2525058	7.9544001	103.1100006
-6.2525058	8.0032005	100.4199982
-6.2525058	8.0520000	102.2699966
-6.2525058	8.1008005	104.4599991
-6.2525058	8.1496000	106.0499954
-6.2525058	8.1984005	106.5199966
-6.2525058	8.2472000	105.7699966
-6.2525058	8.2960005	102.4399948
-6.2525058	8.3448000	101.1999969
-6.2525058	8.3936005	102.9199982
-6.2525058	8.4424000	105.2900009
-6.2525058	8.4912004	104.0800018
-6.2525058	8.5400000	104.2399979
-6.2525058	8.5888004	103.9000015
-6.2525058	8.6375999	101.2299957
-6.2525058	8.6864004	104.0499954

-6.2525058	8.7351999	104.3499985
-6.2525058	8.7840004	102.8499985
-6.2525058	8.8327999	102.0499954
-6.2525058	8.8816004	103.5800018
-6.2525058	8.9303999	104.0999985
-6.2525058	8.9792004	103.8600006
-6.2525058	9.0279999	105.9099960
-6.2525058	9.0768003	104.2099991
-6.2525058	9.1255999	101.3099976
-6.2525058	9.1744003	101.5599976
-6.2525058	9.2231998	103.5199966
-6.2525058	9.2720003	104.2200012
-6.2525058	9.3207998	102.0100021
-6.2525058	9.3696003	94.5800018
-6.2525058	9.4184008	102.2500000
-6.2525058	9.4672003	100.8399963
-6.2525058	9.5160007	101.1800003
-6.2525058	9.5648003	102.4000015
-6.2525058	9.6136007	102.1899948
-6.2525058	9.6624002	105.1599960
-6.2525058	9.7112007	104.2099991
-6.2525058	9.7600002	103.4700012
-6.2525058	9.8088007	103.4199982
-6.2525058	9.8576002	105.5400009
-6.2525058	9.9064007	104.2600021
-6.2525058	9.9552002	102.5699997
-6.2525058	10.0040007	104.1699982
-6.2525058	10.0528002	105.2099991
-6.2525058	10.1016006	103.9099960
-6.2525058	10.1504002	103.0199966
-6.2525058	10.1992006	102.6399994
-6.2525058	10.2480001	103.3799973
-6.2525058	10.2968006	105.1999969
-6.2525058	10.3456001	102.3899994
-6.2525058	10.3944006	101.7900009
-6.2525058	10.4432001	103.6399994
-6.2525058	10.4920006	103.1299973
-6.2525058	10.5408001	104.9399948
-6.2525058	10.5896006	104.6399994
-6.2525058	10.6384001	102.2299957
-6.2525058	10.6872005	97.2200012
-6.2525058	10.7360001	101.5199966
-6.2525058	10.7848005	102.3300018
-6.2525058	10.8336000	101.2900009
-6.2525058	10.8824005	103.3699951
-6.2525058	10.9312000	105.2500000
-6.2525058	10.9800005	103.5899963
-6.2525058	11.0288000	103.3199997
-6.2525058	11.0776005	102.1199951
-6.2525058	11.1264000	101.4000015
-6.2525058	11.1752005	103.2799988
-6.2525058	11.2240000	101.9000015
-6.2525058	11.2728004	101.2500000
-6.2525058	11.3216000	102.4799957
-6.2525058	11.3704004	103.0699997
-6.2525058	11.4191999	102.9399948
-6.2525058	11.4680004	101.5800018
-6.2525058	11.5167999	102.0199966

-6.2525058	11.5656004	98.6699982
-6.2525058	11.6143999	101.6399994
-6.2525058	11.6632004	101.4599991
-6.2525058	11.7119999	103.6199951
-6.2525058	11.7608004	103.0699997
-6.2525058	11.8095999	101.4899979
-6.2525058	11.8584003	102.9399948
-6.2525058	11.9071999	103.4399948
-6.2525058	11.9560003	101.9700012
-6.2525058	12.0047998	101.7600021
-6.2525058	12.0536003	102.3699951
-6.2525058	12.1023998	104.1199951
-6.2525058	12.1512003	103.7999954
-6.2525058	12.1999998	103.6500015
-6.2525058	12.2488003	104.1899948
-6.2525058	12.2975998	104.2200012
-6.2525058	12.3464003	100.8099976
-6.2525058	12.3951998	104.5299988
-6.2525058	12.4440002	104.5800018
-6.2525058	12.4927998	100.9300003
-6.2525058	12.5416002	103.4399948
-6.2525058	12.5904007	105.4599991
-6.2525058	12.6392002	106.1800003
-6.2525058	12.6880007	106.7099991
-6.2525058	12.7368002	106.7600021
-6.2525058	12.7856007	104.4199982
-6.2525058	12.8344002	104.8099976
-6.2525058	12.8832006	103.5199966
-6.2525058	12.9320002	104.2600021
-6.2525058	12.9808006	104.0199966
-6.2525058	13.0296001	102.7999954
-6.2525058	13.0784006	102.6699982
-6.2525058	13.1272001	102.3499985
-6.2525058	13.1760006	99.3600006
-6.2525058	13.2248001	100.3899994
-6.2525058	13.2736006	101.1100006
-6.2525058	13.3224001	102.3799973
-6.2525058	13.3712006	103.5599976
-6.2525058	13.4200001	104.4700012
-6.2525058	13.4688005	105.5999985
-6.2525058	13.5176001	103.9499969
-6.2525058	13.5664005	101.4399948
-6.2525058	13.6152000	100.8600006
-6.2525058	13.6640005	100.0199966
-6.2525058	13.7128000	102.9300003
-6.2525058	13.7616005	105.0800018
-6.2525058	13.8104000	104.7900009
-6.2525058	13.8592005	103.1899948
-6.2525058	13.9080000	103.1999969
-6.2525058	13.9568005	103.0599976
-6.2525058	14.0056000	98.7099991
-6.2525058	14.0544004	102.1100006
-6.2525058	14.1032000	104.4300003
-6.2525058	14.1520004	103.5299988
-6.2525058	14.2007999	103.5599976
-6.2525058	14.2496004	101.7799988
-6.2525058	14.2983999	104.8699951
-6.2525058	14.3472004	103.9700012

-6.2525058	14.3959999	102.2399979
-6.2525058	14.4448004	104.2399979
-6.2525058	14.4935999	107.7099991
-6.2525058	14.5424004	105.5400009
-6.2525058	14.5911999	103.9199982
-6.2525058	14.6400003	102.0400009
-6.2525058	14.6887999	102.4799957
-6.2525058	14.7376003	106.1299973
-6.2525058	14.7863998	105.7799988
-6.2525058	14.8352003	104.1800003
-6.2525058	14.8839998	104.8399963
-6.2525058	14.9328003	105.0899963
-6.2525058	14.9815998	105.8399963
-6.2525058	15.0304003	106.2500000
-6.2463698	0.3416000	110.0299988
-6.2463698	0.3904000	107.2500000
-6.2463698	0.4392000	110.1800003
-6.2463698	0.4880000	108.8799973
-6.2463698	0.5368000	101.7999954
-6.2463698	0.5856000	100.5299988
-6.2463698	0.6344000	101.7299957
-6.2463698	0.6832000	104.8199997
-6.2463698	0.7320000	102.6800003
-6.2463698	0.7808000	107.6999969
-6.2463698	0.8296000	108.9099960
-6.2463698	0.8784000	107.2600021
-6.2463698	0.9272000	105.9599991
-6.2463698	0.9760000	109.8899994
-6.2463698	1.0247999	111.5899963
-6.2463698	1.0735999	110.2999954
-6.2463698	1.1223999	109.1899948
-6.2463698	1.1711999	103.6999969
-6.2463698	1.2200000	105.0800018
-6.2463698	1.2688000	105.1800003
-6.2463698	1.3176000	107.4799957
-6.2463698	1.3664000	106.8799973
-6.2463698	1.4152000	105.6399994
-6.2463698	1.4640000	103.7500000
-6.2463698	1.5128000	106.6100006
-6.2463698	1.5616000	107.4399948
-6.2463698	1.6104000	109.4000015
-6.2463698	1.6592000	112.1999969
-6.2463698	1.7079999	114.9799957
-6.2463698	1.7567999	114.8799973
-6.2463698	1.8055999	113.8399963
-6.2463698	1.8543999	118.3399963
-6.2463698	1.9031999	120.1100006
-6.2463698	1.9519999	115.7600021
-6.2463698	2.0007999	104.6800003
-6.2463698	2.0495999	106.1399994
-6.2463698	2.0983999	104.4099960
-6.2463698	2.1471999	106.5299988
-6.2463698	2.1959999	108.7399979
-6.2463698	2.2447999	107.2099991
-6.2463698	2.2935998	104.4099960
-6.2463698	2.3423998	106.9099960
-6.2463698	2.3912001	105.6800003
-6.2463698	2.4400001	106.4099960

-6.2463698	2.4888000	107.3899994
-6.2463698	2.5376000	107.0000000
-6.2463698	2.5864000	102.6199951
-6.2463698	2.6352000	105.1999969
-6.2463698	2.6840000	105.2999954
-6.2463698	2.7328000	102.8699951
-6.2463698	2.7816000	99.6399994
-6.2463698	2.8304000	101.9499969
-6.2463698	2.8792000	102.3499985
-6.2463698	2.9280000	102.2200012
-6.2463698	2.9768000	105.0100021
-6.2463698	3.0256000	103.4499969
-6.2463698	3.0743999	104.3099976
-6.2463698	3.1231999	103.5699997
-6.2463698	3.1719999	106.1100006
-6.2463698	3.2207999	107.4799957
-6.2463698	3.2695999	108.1199951
-6.2463698	3.3183999	108.3399963
-6.2463698	3.3671999	106.1500015
-6.2463698	3.4159999	103.4700012
-6.2463698	3.4647999	102.5999985
-6.2463698	3.5135999	104.7299957
-6.2463698	3.5623999	105.3399963
-6.2463698	3.6111999	105.2999954
-6.2463698	3.6599998	101.5699997
-6.2463698	3.7087998	100.6899948
-6.2463698	3.7575998	103.7200012
-6.2463698	3.8063998	102.3199997
-6.2463698	3.8551998	102.2200012
-6.2463698	3.9039998	101.9099960
-6.2463698	3.9527998	104.8499985
-6.2463698	4.0016003	105.2299957
-6.2463698	4.0503998	105.7299957
-6.2463698	4.0991998	104.8199997
-6.2463698	4.1479998	103.6199951
-6.2463698	4.1967998	102.3799973
-6.2463698	4.2455997	102.9499969
-6.2463698	4.2943997	107.1999969
-6.2463698	4.3431997	110.7999954
-6.2463698	4.3919997	107.8399963
-6.2463698	4.4407997	111.1299973
-6.2463698	4.4895997	117.6199951
-6.2463698	4.5383997	119.9499969
-6.2463698	4.5871997	117.5499954
-6.2463698	4.6359997	115.6100006
-6.2463698	4.6847997	116.0499954
-6.2463698	4.7336001	111.8899994
-6.2463698	4.7824001	106.0599976
-6.2463698	4.8312001	113.4199982
-6.2463698	4.8800001	116.2999954
-6.2463698	4.9288001	113.7299957
-6.2463698	4.9776001	108.4700012
-6.2463698	5.0264001	107.9799957
-6.2463698	5.0752001	111.7500000
-6.2463698	5.1240001	110.7099991
-6.2463698	5.1728001	108.8099976
-6.2463698	5.2216001	105.5899963
-6.2463698	5.2704000	107.4000015

-6.2463698	5.3192000	110.2900009
-6.2463698	5.3680000	109.8899994
-6.2463698	5.4168000	106.6699982
-6.2463698	5.4656000	106.2500000
-6.2463698	5.5144000	108.1599960
-6.2463698	5.5632000	107.5499954
-6.2463698	5.6120000	108.1199951
-6.2463698	5.6608000	109.5499954
-6.2463698	5.7096000	107.4300003
-6.2463698	5.7584000	101.7999954
-6.2463698	5.8072000	107.2299957
-6.2463698	5.8559999	106.4000015
-6.2463698	5.9047999	106.4799957
-6.2463698	5.9535999	104.9300003
-6.2463698	6.0023999	105.1199951
-6.2463698	6.0511999	107.1299973
-6.2463698	6.0999999	106.2399979
-6.2463698	6.1487999	104.2900009
-6.2463698	6.1975999	101.5999985
-6.2463698	6.2463999	104.8499985
-6.2463698	6.2951999	105.4300003
-6.2463698	6.3439999	100.5000000
-6.2463698	6.3927999	107.5000000
-6.2463698	6.4415998	111.1299973
-6.2463698	6.4903998	109.5400009
-6.2463698	6.5391998	106.4599991
-6.2463698	6.5879998	106.9599991
-6.2463698	6.6367998	105.2399979
-6.2463698	6.6855998	102.5699997
-6.2463698	6.7343998	103.7099991
-6.2463698	6.7831998	104.6599960
-6.2463698	6.8319998	103.6199951
-6.2463698	6.8807998	100.8499985
-6.2463698	6.9295998	102.4300003
-6.2463698	6.9783998	105.4599991
-6.2463698	7.0271997	106.0100021
-6.2463698	7.0759997	105.4599991
-6.2463698	7.1247997	103.4199982
-6.2463698	7.1735997	104.1800003
-6.2463698	7.2223997	101.9099960
-6.2463698	7.2711997	104.3899994
-6.2463698	7.3199997	106.2900009
-6.2463698	7.3687997	105.7699966
-6.2463698	7.4175997	106.3099976
-6.2463698	7.4663997	105.6899948
-6.2463698	7.5151997	101.3300018
-6.2463698	7.5639997	101.8099976
-6.2463698	7.6127996	103.4700012
-6.2463698	7.6615996	103.9399948
-6.2463698	7.7103996	104.9499969
-6.2463698	7.7591996	105.0100021
-6.2463698	7.8079996	102.6299973
-6.2463698	7.8567996	104.8799973
-6.2463698	7.9056001	103.0000000
-6.2463698	7.9544001	102.8399963
-6.2463698	8.0032005	99.9199982
-6.2463698	8.0520000	100.8600006
-6.2463698	8.1008005	103.9199982

-6.2463698	8.1496000	105.3199997
-6.2463698	8.1984005	105.9300003
-6.2463698	8.2472000	105.8099976
-6.2463698	8.2960005	103.0199966
-6.2463698	8.3448000	101.6399994
-6.2463698	8.3936005	102.7600021
-6.2463698	8.4424000	105.0699997
-6.2463698	8.4912004	104.1999969
-6.2463698	8.5400000	103.7399979
-6.2463698	8.5888004	102.8300018
-6.2463698	8.6375999	100.5199966
-6.2463698	8.6864004	103.4799957
-6.2463698	8.7351999	103.8300018
-6.2463698	8.7840004	102.5000000
-6.2463698	8.8327999	101.9099960
-6.2463698	8.8816004	103.5599976
-6.2463698	8.9303999	103.9799957
-6.2463698	8.9792004	104.3899994
-6.2463698	9.0279999	105.6299973
-6.2463698	9.0768003	103.5299988
-6.2463698	9.1255999	101.1699982
-6.2463698	9.1744003	101.5599976
-6.2463698	9.2231998	102.6999969
-6.2463698	9.2720003	103.8499985
-6.2463698	9.3207998	101.7299957
-6.2463698	9.3696003	92.0400009
-6.2463698	9.4184008	100.8799973
-6.2463698	9.4672003	99.3199997
-6.2463698	9.5160007	100.9499969
-6.2463698	9.5648003	102.4099960
-6.2463698	9.6136007	103.5499954
-6.2463698	9.6624002	106.5599976
-6.2463698	9.7112007	105.3799973
-6.2463698	9.7600002	103.6999969
-6.2463698	9.8088007	103.0100021
-6.2463698	9.8576002	105.3600006
-6.2463698	9.9064007	104.2600021
-6.2463698	9.9552002	103.4899979
-6.2463698	10.0040007	104.2299957
-6.2463698	10.0528002	105.3799973
-6.2463698	10.1016006	103.9499969
-6.2463698	10.1504002	102.5100021
-6.2463698	10.1992006	102.8399963
-6.2463698	10.2480001	104.4000015
-6.2463698	10.2968006	105.1899948
-6.2463698	10.3456001	101.7999954
-6.2463698	10.3944006	100.9499969
-6.2463698	10.4432001	104.7099991
-6.2463698	10.4920006	104.3300018
-6.2463698	10.5408001	104.5800018
-6.2463698	10.5896006	103.9599991
-6.2463698	10.6384001	101.8099976
-6.2463698	10.6872005	98.3300018
-6.2463698	10.7360001	102.1399994
-6.2463698	10.7848005	102.8699951
-6.2463698	10.8336000	101.7500000
-6.2463698	10.8824005	102.6800003
-6.2463698	10.9312000	104.2399979

-6.2463698	10.9800005	103.0699997
-6.2463698	11.0288000	103.2799988
-6.2463698	11.0776005	102.7500000
-6.2463698	11.1264000	101.5899963
-6.2463698	11.1752005	103.7799988
-6.2463698	11.2240000	102.6599960
-6.2463698	11.2728004	101.8499985
-6.2463698	11.3216000	103.3099976
-6.2463698	11.3704004	102.6299973
-6.2463698	11.4191999	102.7200012
-6.2463698	11.4680004	102.0499954
-6.2463698	11.5167999	103.4000015
-6.2463698	11.5656004	100.8300018
-6.2463698	11.6143999	102.0499954
-6.2463698	11.6632004	102.6100006
-6.2463698	11.7119999	103.8499985
-6.2463698	11.7608004	102.7900009
-6.2463698	11.8095999	101.5899963
-6.2463698	11.8584003	103.2500000
-6.2463698	11.9071999	103.7600021
-6.2463698	11.9560003	102.0599976
-6.2463698	12.0047998	100.4799957
-6.2463698	12.0536003	102.3600006
-6.2463698	12.1023998	102.9099960
-6.2463698	12.1512003	102.6599960
-6.2463698	12.1999998	103.2399979
-6.2463698	12.2488003	104.5299988
-6.2463698	12.2975998	103.5199966
-6.2463698	12.3464003	100.5299988
-6.2463698	12.3951998	105.1100006
-6.2463698	12.4440002	105.8099976
-6.2463698	12.4927998	101.9499969
-6.2463698	12.5416002	103.4099960
-6.2463698	12.5904007	105.5000000
-6.2463698	12.6392002	106.7699966
-6.2463698	12.6880007	107.7699966
-6.2463698	12.7368002	107.5800018
-6.2463698	12.7856007	105.5299988
-6.2463698	12.8344002	105.1100006
-6.2463698	12.8832006	102.5599976
-6.2463698	12.9320002	102.0499954
-6.2463698	12.9808006	103.6199951
-6.2463698	13.0296001	103.5299988
-6.2463698	13.0784006	102.7099991
-6.2463698	13.1272001	101.4499969
-6.2463698	13.1760006	98.0299988
-6.2463698	13.2248001	100.3799973
-6.2463698	13.2736006	101.2399979
-6.2463698	13.3224001	101.7900009
-6.2463698	13.3712006	103.4700012
-6.2463698	13.4200001	104.4700012
-6.2463698	13.4688005	104.6999969
-6.2463698	13.5176001	103.9700012
-6.2463698	13.5664005	101.1199951
-6.2463698	13.6152000	102.1399994
-6.2463698	13.6640005	101.7900009
-6.2463698	13.7128000	104.0599976
-6.2463698	13.7616005	105.5000000

-6.2463698	13.8104000	105.0599976
-6.2463698	13.8592005	103.8899994
-6.2463698	13.9080000	102.6399994
-6.2463698	13.9568005	102.6500015
-6.2463698	14.0056000	97.2900009
-6.2463698	14.0544004	102.2099991
-6.2463698	14.1032000	104.7399979
-6.2463698	14.1520004	103.8099976
-6.2463698	14.2007999	103.4300003
-6.2463698	14.2496004	102.9000015
-6.2463698	14.2983999	105.3799973
-6.2463698	14.3472004	103.8199997
-6.2463698	14.3959999	101.9700012
-6.2463698	14.4448004	103.7900009
-6.2463698	14.4935999	107.4599991
-6.2463698	14.5424004	105.2200012
-6.2463698	14.5911999	103.9499969
-6.2463698	14.6400003	101.9099960
-6.2463698	14.6887999	102.9399948
-6.2463698	14.7376003	106.1500015
-6.2463698	14.7863998	105.5199966
-6.2463698	14.8352003	103.5400009
-6.2463698	14.8839998	104.4199982
-6.2463698	14.9328003	104.2699966
-6.2463698	14.9815998	105.2299957
-6.2463698	15.0304003	105.9799957
-6.2402339	0.3416000	109.2399979
-6.2402339	0.3904000	106.1899948
-6.2402339	0.4392000	109.6299973
-6.2402339	0.4880000	108.6299973
-6.2402339	0.5368000	101.8799973
-6.2402339	0.5856000	99.9499969
-6.2402339	0.6344000	103.6599960
-6.2402339	0.6832000	104.9499969
-6.2402339	0.7320000	102.5400009
-6.2402339	0.7808000	107.1999969
-6.2402339	0.8296000	108.3499985
-6.2402339	0.8784000	107.3899994
-6.2402339	0.9272000	106.2699966
-6.2402339	0.9760000	110.1899948
-6.2402339	1.0247999	110.8499985
-6.2402339	1.0735999	108.4099960
-6.2402339	1.1223999	108.3399963
-6.2402339	1.1711999	103.7299957
-6.2402339	1.2200000	105.5499954
-6.2402339	1.2688000	104.0899963
-6.2402339	1.3176000	106.7699966
-6.2402339	1.3664000	106.0199966
-6.2402339	1.4152000	105.3799973
-6.2402339	1.4640000	103.4799957
-6.2402339	1.5128000	106.0899963
-6.2402339	1.5616000	107.6599960
-6.2402339	1.6104000	110.4899979
-6.2402339	1.6592000	112.1299973
-6.2402339	1.7079999	114.9399948
-6.2402339	1.7567999	114.1899948
-6.2402339	1.8055999	113.7799988
-6.2402339	1.8543999	117.8600006

-6.2402339	1.9031999	119.2299957
-6.2402339	1.9519999	114.5400009
-6.2402339	2.0007999	104.4799957
-6.2402339	2.0495999	106.5499954
-6.2402339	2.0983999	105.4499969
-6.2402339	2.1471999	107.9399948
-6.2402339	2.1959999	110.0800018
-6.2402339	2.2447999	108.1999969
-6.2402339	2.2935998	104.9700012
-6.2402339	2.3423998	106.2399979
-6.2402339	2.3912001	105.2200012
-6.2402339	2.4400001	106.0100021
-6.2402339	2.4888000	107.3399963
-6.2402339	2.5376000	107.3099976
-6.2402339	2.5864000	103.8399963
-6.2402339	2.6352000	106.5100021
-6.2402339	2.6840000	106.2200012
-6.2402339	2.7328000	103.2299957
-6.2402339	2.7816000	100.1500015
-6.2402339	2.8304000	101.7399979
-6.2402339	2.8792000	103.3600006
-6.2402339	2.9280000	103.8199997
-6.2402339	2.9768000	106.3099976
-6.2402339	3.0256000	104.3799973
-6.2402339	3.0743999	103.7200012
-6.2402339	3.1231999	101.7799988
-6.2402339	3.1719999	105.3099976
-6.2402339	3.2207999	107.3499985
-6.2402339	3.2695999	107.6500015
-6.2402339	3.3183999	108.4000015
-6.2402339	3.3671999	105.9499969
-6.2402339	3.4159999	102.6999969
-6.2402339	3.4647999	102.5000000
-6.2402339	3.5135999	104.5299988
-6.2402339	3.5623999	104.9099960
-6.2402339	3.6111999	104.7500000
-6.2402339	3.6599998	100.8799973
-6.2402339	3.7087998	99.5199966
-6.2402339	3.7575998	104.1999969
-6.2402339	3.8063998	103.0899963
-6.2402339	3.8551998	102.2200012
-6.2402339	3.9039998	101.8899994
-6.2402339	3.9527998	104.9700012
-6.2402339	4.0016003	104.4300003
-6.2402339	4.0503998	104.9799957
-6.2402339	4.0991998	103.8799973
-6.2402339	4.1479998	102.5599976
-6.2402339	4.1967998	101.6899948
-6.2402339	4.2455997	102.8499985
-6.2402339	4.2943997	108.0400009
-6.2402339	4.3431997	111.9199982
-6.2402339	4.3919997	109.8600006
-6.2402339	4.4407997	111.8399963
-6.2402339	4.4895997	117.3699951
-6.2402339	4.5383997	119.3899994
-6.2402339	4.5871997	116.6399994
-6.2402339	4.6359997	114.3199997
-6.2402339	4.6847997	115.9199982

-6.2402339	4.7336001	111.9899979
-6.2402339	4.7824001	106.2600021
-6.2402339	4.8312001	112.9099960
-6.2402339	4.8800001	114.9599991
-6.2402339	4.9288001	111.9300003
-6.2402339	4.9776001	107.1999969
-6.2402339	5.0264001	107.1199951
-6.2402339	5.0752001	111.3199997
-6.2402339	5.1240001	110.1299973
-6.2402339	5.1728001	108.2900009
-6.2402339	5.2216001	105.8399963
-6.2402339	5.2704000	107.5100021
-6.2402339	5.3192000	110.1800003
-6.2402339	5.3680000	109.6899948
-6.2402339	5.4168000	106.7200012
-6.2402339	5.4656000	105.1299973
-6.2402339	5.5144000	108.0899963
-6.2402339	5.5632000	108.0000000
-6.2402339	5.6120000	107.1299973
-6.2402339	5.6608000	109.1800003
-6.2402339	5.7096000	107.9199982
-6.2402339	5.7584000	103.5100021
-6.2402339	5.8072000	107.9300003
-6.2402339	5.8559999	106.7399979
-6.2402339	5.9047999	106.5999985
-6.2402339	5.9535999	103.5699997
-6.2402339	6.0023999	103.7799988
-6.2402339	6.0511999	106.5199966
-6.2402339	6.0999999	104.8699951
-6.2402339	6.1487999	104.0800018
-6.2402339	6.1975999	102.5299988
-6.2402339	6.2463999	105.0999985
-6.2402339	6.2951999	104.7699966
-6.2402339	6.3439999	101.4399948
-6.2402339	6.3927999	108.5699997
-6.2402339	6.4415998	111.2799988
-6.2402339	6.4903998	109.1399994
-6.2402339	6.5391998	106.4300003
-6.2402339	6.5879998	106.7900009
-6.2402339	6.6367998	105.3899994
-6.2402339	6.6855998	102.2099991
-6.2402339	6.7343998	103.5199966
-6.2402339	6.7831998	104.4499969
-6.2402339	6.8319998	103.5100021
-6.2402339	6.8807998	101.1800003
-6.2402339	6.9295998	102.8799973
-6.2402339	6.9783998	106.5899963
-6.2402339	7.0271997	107.0599976
-6.2402339	7.0759997	105.5899963
-6.2402339	7.1247997	103.1399994
-6.2402339	7.1735997	104.1999969
-6.2402339	7.2223997	101.5699997
-6.2402339	7.2711997	104.1800003
-6.2402339	7.3199997	106.4300003
-6.2402339	7.3687997	105.0899963
-6.2402339	7.4175997	106.8600006
-6.2402339	7.4663997	105.9700012
-6.2402339	7.5151997	102.8199997

-6.2402339	7.5639997	102.6500015
-6.2402339	7.6127996	103.3799973
-6.2402339	7.6615996	103.8199997
-6.2402339	7.7103996	104.6199951
-6.2402339	7.7591996	105.3399963
-6.2402339	7.8079996	104.2900009
-6.2402339	7.8567996	105.0299988
-6.2402339	7.9056001	102.5000000
-6.2402339	7.9544001	103.7299957
-6.2402339	8.0032005	100.4799957
-6.2402339	8.0520000	101.0100021
-6.2402339	8.1008005	104.2900009
-6.2402339	8.1496000	106.0100021
-6.2402339	8.1984005	106.4599991
-6.2402339	8.2472000	105.5899963
-6.2402339	8.2960005	102.4700012
-6.2402339	8.3448000	102.5400009
-6.2402339	8.3936005	103.3199997
-6.2402339	8.4424000	105.0699997
-6.2402339	8.4912004	103.5499954
-6.2402339	8.5400000	102.6800003
-6.2402339	8.5888004	101.5599976
-6.2402339	8.6375999	99.9000015
-6.2402339	8.6864004	103.3699951
-6.2402339	8.7351999	103.6199951
-6.2402339	8.7840004	101.9700012
-6.2402339	8.8327999	101.3399963
-6.2402339	8.8816004	103.4199982
-6.2402339	8.9303999	103.4799957
-6.2402339	8.9792004	104.3499985
-6.2402339	9.0279999	104.8799973
-6.2402339	9.0768003	102.6800003
-6.2402339	9.1255999	101.1800003
-6.2402339	9.1744003	102.0100021
-6.2402339	9.2231998	102.3600006
-6.2402339	9.2720003	103.5100021
-6.2402339	9.3207998	101.9300003
-6.2402339	9.3696003	92.4300003
-6.2402339	9.4184008	100.3899994
-6.2402339	9.4672003	99.6100006
-6.2402339	9.5160007	101.1699982
-6.2402339	9.5648003	102.6399994
-6.2402339	9.6136007	104.8499985
-6.2402339	9.6624002	107.5299988
-6.2402339	9.7112007	106.2099991
-6.2402339	9.7600002	104.6199951
-6.2402339	9.8088007	102.8399963
-6.2402339	9.8576002	105.0599976
-6.2402339	9.9064007	103.9399948
-6.2402339	9.9552002	103.7200012
-6.2402339	10.0040007	103.8699951
-6.2402339	10.0528002	104.5899963
-6.2402339	10.1016006	103.2600021
-6.2402339	10.1504002	101.5100021
-6.2402339	10.1992006	103.3799973
-6.2402339	10.2480001	105.7699966
-6.2402339	10.2968006	105.1999969
-6.2402339	10.3456001	101.5400009

-6.2402339	10.3944006	99.9399948
-6.2402339	10.4432001	105.1399994
-6.2402339	10.4920006	105.0299988
-6.2402339	10.5408001	103.6100006
-6.2402339	10.5896006	103.3799973
-6.2402339	10.6384001	102.1399994
-6.2402339	10.6872005	99.6800003
-6.2402339	10.7360001	102.7099991
-6.2402339	10.7848005	103.1500015
-6.2402339	10.8336000	102.2699966
-6.2402339	10.8824005	102.0199966
-6.2402339	10.9312000	103.1399994
-6.2402339	10.9800005	102.5400009
-6.2402339	11.0288000	103.1199951
-6.2402339	11.0776005	103.5599976
-6.2402339	11.1264000	102.5800018
-6.2402339	11.1752005	104.8399963
-6.2402339	11.2240000	104.1599960
-6.2402339	11.2728004	103.0299988
-6.2402339	11.3216000	104.1100006
-6.2402339	11.3704004	102.6299973
-6.2402339	11.4191999	102.6100006
-6.2402339	11.4680004	102.3899994
-6.2402339	11.5167999	103.8600006
-6.2402339	11.5656004	101.5299988
-6.2402339	11.6143999	102.1199951
-6.2402339	11.6632004	103.7900009
-6.2402339	11.7119999	104.3300018
-6.2402339	11.7608004	102.8799973
-6.2402339	11.8095999	102.4399948
-6.2402339	11.8584003	104.4099960
-6.2402339	11.9071999	104.5999985
-6.2402339	11.9560003	101.8399963
-6.2402339	12.0047998	99.2900009
-6.2402339	12.0536003	102.5000000
-6.2402339	12.1023998	102.2600021
-6.2402339	12.1512003	102.0199966
-6.2402339	12.1999998	102.9799957
-6.2402339	12.2488003	104.7699966
-6.2402339	12.2975998	103.0000000
-6.2402339	12.3464003	100.0499954
-6.2402339	12.3951998	105.3300018
-6.2402339	12.4440002	106.8899994
-6.2402339	12.4927998	103.4599991
-6.2402339	12.5416002	103.9599991
-6.2402339	12.5904007	105.4000015
-6.2402339	12.6392002	106.8899994
-6.2402339	12.6880007	108.4000015
-6.2402339	12.7368002	107.9000015
-6.2402339	12.7856007	105.6899948
-6.2402339	12.8344002	104.5199966
-6.2402339	12.8832006	101.4499969
-6.2402339	12.9320002	99.9599991
-6.2402339	12.9808006	103.2900009
-6.2402339	13.0296001	103.9499969
-6.2402339	13.0784006	102.6899948
-6.2402339	13.1272001	100.6399994
-6.2402339	13.1760006	96.6999969

-6.2402339	13.2248001	100.6500015
-6.2402339	13.2736006	101.9300003
-6.2402339	13.3224001	101.5599976
-6.2402339	13.3712006	103.4199982
-6.2402339	13.4200001	103.9799957
-6.2402339	13.4688005	103.3799973
-6.2402339	13.5176001	103.7299957
-6.2402339	13.5664005	100.7200012
-6.2402339	13.6152000	103.1699982
-6.2402339	13.6640005	102.7999954
-6.2402339	13.7128000	104.6399994
-6.2402339	13.7616005	105.6500015
-6.2402339	13.8104000	105.3899994
-6.2402339	13.8592005	104.1899948
-6.2402339	13.9080000	101.3600006
-6.2402339	13.9568005	101.5400009
-6.2402339	14.0056000	95.5599976
-6.2402339	14.0544004	102.0000000
-6.2402339	14.1032000	105.0000000
-6.2402339	14.1520004	104.0000000
-6.2402339	14.2007999	102.8600006
-6.2402339	14.2496004	103.5899963
-6.2402339	14.2983999	105.4499969
-6.2402339	14.3472004	103.4899979
-6.2402339	14.3959999	102.1399994
-6.2402339	14.4448004	104.0400009
-6.2402339	14.4935999	107.2900009
-6.2402339	14.5424004	104.7699966
-6.2402339	14.5911999	102.9300003
-6.2402339	14.6400003	101.9899979
-6.2402339	14.6887999	103.1699982
-6.2402339	14.7376003	106.0599976
-6.2402339	14.7863998	105.3600006
-6.2402339	14.8352003	102.9499969
-6.2402339	14.8839998	103.9799957
-6.2402339	14.9328003	103.2099991
-6.2402339	14.9815998	104.2900009
-6.2402339	15.0304003	105.4599991
-6.2340980	0.3416000	108.4199982
-6.2340980	0.3904000	106.6999969
-6.2340980	0.4392000	109.3899994
-6.2340980	0.4880000	108.2299957
-6.2340980	0.5368000	102.1599960
-6.2340980	0.5856000	100.5199966
-6.2340980	0.6344000	104.8300018
-6.2340980	0.6832000	105.0000000
-6.2340980	0.7320000	102.8799973
-6.2340980	0.7808000	107.1500015
-6.2340980	0.8296000	108.1899948
-6.2340980	0.8784000	107.9099960
-6.2340980	0.9272000	106.9799957
-6.2340980	0.9760000	110.5899963
-6.2340980	1.0247999	109.9700012
-6.2340980	1.0735999	106.3600006
-6.2340980	1.1223999	108.3099976
-6.2340980	1.1711999	105.6299973
-6.2340980	1.2200000	105.7299957
-6.2340980	1.2688000	102.2799988

-6.2340980	1.3176000	106.0400009
-6.2340980	1.3664000	105.6399994
-6.2340980	1.4152000	105.7900009
-6.2340980	1.4640000	103.7200012
-6.2340980	1.5128000	105.8300018
-6.2340980	1.5616000	107.7600021
-6.2340980	1.6104000	111.4399948
-6.2340980	1.6592000	112.0800018
-6.2340980	1.7079999	115.0000000
-6.2340980	1.7567999	113.7200012
-6.2340980	1.8055999	113.8699951
-6.2340980	1.8543999	117.3499985
-6.2340980	1.9031999	118.4599991
-6.2340980	1.9519999	113.5800018
-6.2340980	2.0007999	104.5000000
-6.2340980	2.0495999	107.2099991
-6.2340980	2.0983999	106.2900009
-6.2340980	2.1471999	108.9499969
-6.2340980	2.1959999	110.9399948
-6.2340980	2.2447999	108.8799973
-6.2340980	2.2935998	105.0999985
-6.2340980	2.3423998	105.7699966
-6.2340980	2.3912001	104.3499985
-6.2340980	2.4400001	105.3399963
-6.2340980	2.4888000	107.1599960
-6.2340980	2.5376000	108.0800018
-6.2340980	2.5864000	105.2600021
-6.2340980	2.6352000	107.4499969
-6.2340980	2.6840000	106.6599960
-6.2340980	2.7328000	103.4499969
-6.2340980	2.7816000	101.4399948
-6.2340980	2.8304000	102.0999985
-6.2340980	2.8792000	104.5199966
-6.2340980	2.9280000	105.5199966
-6.2340980	2.9768000	107.7200012
-6.2340980	3.0256000	105.1599960
-6.2340980	3.0743999	103.0400009
-6.2340980	3.1231999	100.2699966
-6.2340980	3.1719999	104.5499954
-6.2340980	3.2207999	107.0400009
-6.2340980	3.2695999	107.0800018
-6.2340980	3.3183999	108.5400009
-6.2340980	3.3671999	106.1199951
-6.2340980	3.4159999	102.3799973
-6.2340980	3.4647999	102.9399948
-6.2340980	3.5135999	104.9399948
-6.2340980	3.5623999	104.8499985
-6.2340980	3.6111999	104.0899963
-6.2340980	3.6599998	100.1299973
-6.2340980	3.7087998	98.2600021
-6.2340980	3.7575998	104.4799957
-6.2340980	3.8063998	104.1500015
-6.2340980	3.8551998	102.2799988
-6.2340980	3.9039998	101.4799957
-6.2340980	3.9527998	104.7999954
-6.2340980	4.0016003	103.4399948
-6.2340980	4.0503998	104.0899963
-6.2340980	4.0991998	102.5599976

-6.2340980	4.1479998	101.5299988
-6.2340980	4.1967998	102.0199966
-6.2340980	4.2455997	102.5199966
-6.2340980	4.2943997	108.0400009
-6.2340980	4.3431997	112.5800018
-6.2340980	4.3919997	111.4000015
-6.2340980	4.4407997	112.2200012
-6.2340980	4.4895997	117.0100021
-6.2340980	4.5383997	118.5599976
-6.2340980	4.5871997	115.7500000
-6.2340980	4.6359997	112.9000015
-6.2340980	4.6847997	115.3600006
-6.2340980	4.7336001	111.4300003
-6.2340980	4.7824001	105.8799973
-6.2340980	4.8312001	112.0599976
-6.2340980	4.8800001	113.5400009
-6.2340980	4.9288001	110.3499985
-6.2340980	4.9776001	106.3099976
-6.2340980	5.0264001	107.2799988
-6.2340980	5.0752001	111.1399994
-6.2340980	5.1240001	109.5400009
-6.2340980	5.1728001	107.8099976
-6.2340980	5.2216001	106.6100006
-6.2340980	5.2704000	108.0999985
-6.2340980	5.3192000	109.8899994
-6.2340980	5.3680000	109.2799988
-6.2340980	5.4168000	106.7600021
-6.2340980	5.4656000	104.1899948
-6.2340980	5.5144000	107.8799973
-6.2340980	5.5632000	108.1999969
-6.2340980	5.6120000	107.5800018
-6.2340980	5.6608000	109.7799988
-6.2340980	5.7096000	109.2799988
-6.2340980	5.7584000	105.4000015
-6.2340980	5.8072000	108.3099976
-6.2340980	5.8559999	106.7099991
-6.2340980	5.9047999	105.6599960
-6.2340980	5.9535999	103.6299973
-6.2340980	6.0023999	102.9399948
-6.2340980	6.0511999	106.3099976
-6.2340980	6.0999999	104.4300003
-6.2340980	6.1487999	104.9599991
-6.2340980	6.1975999	103.6599960
-6.2340980	6.2463999	105.2799988
-6.2340980	6.2951999	104.0699997
-6.2340980	6.3439999	102.4700012
-6.2340980	6.3927999	109.5199966
-6.2340980	6.4415998	111.5899963
-6.2340980	6.4903998	109.9099960
-6.2340980	6.5391998	106.6899948
-6.2340980	6.5879998	106.4000015
-6.2340980	6.6367998	105.6299973
-6.2340980	6.6855998	102.4700012
-6.2340980	6.7343998	103.4399948
-6.2340980	6.7831998	104.2099991
-6.2340980	6.8319998	103.6599960
-6.2340980	6.8807998	101.3799973
-6.2340980	6.9295998	102.6299973

-6.2340980	6.9783998	106.7900009
-6.2340980	7.0271997	107.5299988
-6.2340980	7.0759997	105.9300003
-6.2340980	7.1247997	103.4700012
-6.2340980	7.1735997	104.2699966
-6.2340980	7.2223997	101.5199966
-6.2340980	7.2711997	104.5299988
-6.2340980	7.3199997	106.2500000
-6.2340980	7.3687997	103.0100021
-6.2340980	7.4175997	106.4700012
-6.2340980	7.4663997	105.2099991
-6.2340980	7.5151997	103.3199997
-6.2340980	7.5639997	103.8600006
-6.2340980	7.6127996	104.0400009
-6.2340980	7.6615996	104.2699966
-6.2340980	7.7103996	104.3899994
-6.2340980	7.7591996	105.2299957
-6.2340980	7.8079996	105.7699966
-6.2340980	7.8567996	105.2900009
-6.2340980	7.9056001	102.2799988
-6.2340980	7.9544001	104.3099976
-6.2340980	8.0032005	101.0599976
-6.2340980	8.0520000	100.8699951
-6.2340980	8.1008005	104.1399994
-6.2340980	8.1496000	106.5800018
-6.2340980	8.1984005	106.6800003
-6.2340980	8.2472000	105.1100006
-6.2340980	8.2960005	102.4300003
-6.2340980	8.3448000	103.7699966
-6.2340980	8.3936005	103.6800003
-6.2340980	8.4424000	104.5999985
-6.2340980	8.4912004	102.4300003
-6.2340980	8.5400000	101.5899963
-6.2340980	8.5888004	100.5599976
-6.2340980	8.6375999	100.3699951
-6.2340980	8.6864004	103.7600021
-6.2340980	8.7351999	103.3799973
-6.2340980	8.7840004	101.4899979
-6.2340980	8.8327999	100.8300018
-6.2340980	8.8816004	103.0800018
-6.2340980	8.9303999	103.4700012
-6.2340980	8.9792004	104.0699997
-6.2340980	9.0279999	103.7999954
-6.2340980	9.0768003	101.8399963
-6.2340980	9.1255999	101.1100006
-6.2340980	9.1744003	102.3799973
-6.2340980	9.2231998	101.8499985
-6.2340980	9.2720003	102.8600006
-6.2340980	9.3207998	102.1899948
-6.2340980	9.3696003	94.3799973
-6.2340980	9.4184008	99.9099960
-6.2340980	9.4672003	99.4300003
-6.2340980	9.5160007	100.7099991
-6.2340980	9.5648003	102.4700012
-6.2340980	9.6136007	105.1699982
-6.2340980	9.6624002	107.7999954
-6.2340980	9.7112007	106.3399963
-6.2340980	9.7600002	104.9700012

-6.2340980	9.8088007	103.3399963
-6.2340980	9.8576002	105.0499954
-6.2340980	9.9064007	103.9000015
-6.2340980	9.9552002	103.9700012
-6.2340980	10.0040007	103.4399948
-6.2340980	10.0528002	103.2500000
-6.2340980	10.1016006	102.6100006
-6.2340980	10.1504002	101.1699982
-6.2340980	10.1992006	103.7900009
-6.2340980	10.2480001	106.3399963
-6.2340980	10.2968006	104.8699951
-6.2340980	10.3456001	101.9300003
-6.2340980	10.3944006	99.1999969
-6.2340980	10.4432001	105.0499954
-6.2340980	10.4920006	105.7600021
-6.2340980	10.5408001	103.1100006
-6.2340980	10.5896006	103.3799973
-6.2340980	10.6384001	102.8199997
-6.2340980	10.6872005	100.1199951
-6.2340980	10.7360001	102.4899979
-6.2340980	10.7848005	102.1299973
-6.2340980	10.8336000	102.2299957
-6.2340980	10.8824005	101.3499985
-6.2340980	10.9312000	101.8899994
-6.2340980	10.9800005	102.0899963
-6.2340980	11.0288000	103.2799988
-6.2340980	11.0776005	104.4199982
-6.2340980	11.1264000	104.1500015
-6.2340980	11.1752005	106.0000000
-6.2340980	11.2240000	105.6899948
-6.2340980	11.2728004	104.2200012
-6.2340980	11.3216000	104.9899979
-6.2340980	11.3704004	102.8499985
-6.2340980	11.4191999	102.2299957
-6.2340980	11.4680004	102.1800003
-6.2340980	11.5167999	104.0499954
-6.2340980	11.5656004	102.2799988
-6.2340980	11.6143999	102.8300018
-6.2340980	11.6632004	105.4000015
-6.2340980	11.7119999	104.9199982
-6.2340980	11.7608004	102.8399963
-6.2340980	11.8095999	103.0800018
-6.2340980	11.8584003	105.3499985
-6.2340980	11.9071999	104.7099991
-6.2340980	11.9560003	101.5599976
-6.2340980	12.0047998	98.5899963
-6.2340980	12.0536003	102.8499985
-6.2340980	12.1023998	101.8199997
-6.2340980	12.1512003	102.0999985
-6.2340980	12.1999998	103.6500015
-6.2340980	12.2488003	105.5899963
-6.2340980	12.2975998	102.8699951
-6.2340980	12.3464003	100.1100006
-6.2340980	12.3951998	105.2799988
-6.2340980	12.4440002	107.1899948
-6.2340980	12.4927998	104.1199951
-6.2340980	12.5416002	103.7399979
-6.2340980	12.5904007	104.7299957

-6.2340980	12.6392002	106.5499954
-6.2340980	12.6880007	108.5699997
-6.2340980	12.7368002	108.2900009
-6.2340980	12.7856007	106.0599976
-6.2340980	12.8344002	104.4000015
-6.2340980	12.8832006	101.5000000
-6.2340980	12.9320002	98.9199982
-6.2340980	12.9808006	102.7900009
-6.2340980	13.0296001	103.9000015
-6.2340980	13.0784006	103.0199966
-6.2340980	13.1272001	100.5899963
-6.2340980	13.1760006	96.0400009
-6.2340980	13.2248001	100.7500000
-6.2340980	13.2736006	102.5699997
-6.2340980	13.3224001	101.3399963
-6.2340980	13.3712006	102.8600006
-6.2340980	13.4200001	103.1299973
-6.2340980	13.4688005	102.0899963
-6.2340980	13.5176001	103.4700012
-6.2340980	13.5664005	99.9700012
-6.2340980	13.6152000	103.9799957
-6.2340980	13.6640005	103.8899994
-6.2340980	13.7128000	104.2900009
-6.2340980	13.7616005	105.6500015
-6.2340980	13.8104000	105.5000000
-6.2340980	13.8592005	104.0499954
-6.2340980	13.9080000	99.5599976
-6.2340980	13.9568005	99.9099960
-6.2340980	14.0056000	94.4599991
-6.2340980	14.0544004	101.6899948
-6.2340980	14.1032000	104.7799988
-6.2340980	14.1520004	104.0599976
-6.2340980	14.2007999	102.5499954
-6.2340980	14.2496004	104.2299957
-6.2340980	14.2983999	105.4700012
-6.2340980	14.3472004	103.2200012
-6.2340980	14.3959999	102.4599991
-6.2340980	14.4448004	104.1699982
-6.2340980	14.4935999	106.6999969
-6.2340980	14.5424004	103.6500015
-6.2340980	14.5911999	101.5699997
-6.2340980	14.6400003	101.6500015
-6.2340980	14.6887999	102.6399994
-6.2340980	14.7376003	105.4399948
-6.2340980	14.7863998	104.9700012
-6.2340980	14.8352003	102.5100021
-6.2340980	14.8839998	103.8799973
-6.2340980	14.9328003	102.5499954
-6.2340980	14.9815998	104.0899963
-6.2340980	15.0304003	105.2500000
-6.2279620	0.3416000	107.6199951
-6.2279620	0.3904000	106.9499969
-6.2279620	0.4392000	108.8399963
-6.2279620	0.4880000	107.6500015
-6.2279620	0.5368000	103.1599960
-6.2279620	0.5856000	101.4099960
-6.2279620	0.6344000	105.8499985
-6.2279620	0.6832000	104.6999969

-6.2279620	0.7320000	103.3099976
-6.2279620	0.7808000	107.2799988
-6.2279620	0.8296000	107.9499969
-6.2279620	0.8784000	108.1100006
-6.2279620	0.9272000	107.5699997
-6.2279620	0.9760000	110.7099991
-6.2279620	1.0247999	108.7900009
-6.2279620	1.0735999	104.0999985
-6.2279620	1.1223999	107.6999969
-6.2279620	1.1711999	106.2299957
-6.2279620	1.2200000	105.9700012
-6.2279620	1.2688000	101.1899948
-6.2279620	1.3176000	105.7999954
-6.2279620	1.3664000	105.3099976
-6.2279620	1.4152000	106.6299973
-6.2279620	1.4640000	105.4099960
-6.2279620	1.5128000	105.8799973
-6.2279620	1.5616000	107.4899979
-6.2279620	1.6104000	111.4199982
-6.2279620	1.6592000	111.3399963
-6.2279620	1.7079999	114.1899948
-6.2279620	1.7567999	113.0400009
-6.2279620	1.8055999	114.5400009
-6.2279620	1.8543999	117.8799973
-6.2279620	1.9031999	118.5199966
-6.2279620	1.9519999	112.9099960
-6.2279620	2.0007999	106.0599976
-6.2279620	2.0495999	108.4199982
-6.2279620	2.0983999	107.2299957
-6.2279620	2.1471999	109.4399948
-6.2279620	2.1959999	111.4700012
-6.2279620	2.2447999	109.4300003
-6.2279620	2.2935998	105.6699982
-6.2279620	2.3423998	105.3600006
-6.2279620	2.3912001	103.1500015
-6.2279620	2.4400001	104.8799973
-6.2279620	2.4888000	107.0299988
-6.2279620	2.5376000	108.7099991
-6.2279620	2.5864000	106.1999969
-6.2279620	2.6352000	108.5699997
-6.2279620	2.6840000	107.6699982
-6.2279620	2.7328000	104.1699982
-6.2279620	2.7816000	102.4000015
-6.2279620	2.8304000	103.1800003
-6.2279620	2.8792000	105.4099960
-6.2279620	2.9280000	106.5199966
-6.2279620	2.9768000	108.5699997
-6.2279620	3.0256000	106.5499954
-6.2279620	3.0743999	103.6100006
-6.2279620	3.1231999	100.6599960
-6.2279620	3.1719999	104.1399994
-6.2279620	3.2207999	106.0999985
-6.2279620	3.2695999	105.5000000
-6.2279620	3.3183999	107.5299988
-6.2279620	3.3671999	106.0699997
-6.2279620	3.4159999	102.6500015
-6.2279620	3.4647999	104.2500000
-6.2279620	3.5135999	105.6800003

-6.2279620	3.5623999	105.2600021
-6.2279620	3.6111999	104.3799973
-6.2279620	3.6599998	101.0899963
-6.2279620	3.7087998	97.4399948
-6.2279620	3.7575998	104.4099960
-6.2279620	3.8063998	104.8300018
-6.2279620	3.8551998	101.9499969
-6.2279620	3.9039998	100.7900009
-6.2279620	3.9527998	103.5599976
-6.2279620	4.0016003	101.5100021
-6.2279620	4.0503998	102.8300018
-6.2279620	4.0991998	101.3600006
-6.2279620	4.1479998	101.7999954
-6.2279620	4.1967998	102.7900009
-6.2279620	4.2455997	102.1899948
-6.2279620	4.2943997	108.1399994
-6.2279620	4.3431997	113.2500000
-6.2279620	4.3919997	112.6599960
-6.2279620	4.4407997	112.4399948
-6.2279620	4.4895997	116.3899994
-6.2279620	4.5383997	117.6199951
-6.2279620	4.5871997	115.3099976
-6.2279620	4.6359997	111.7799988
-6.2279620	4.6847997	114.6800003
-6.2279620	4.7336001	110.6699982
-6.2279620	4.7824001	105.2900009
-6.2279620	4.8312001	111.3799973
-6.2279620	4.8800001	111.9599991
-6.2279620	4.9288001	108.5299988
-6.2279620	4.9776001	105.8699951
-6.2279620	5.0264001	107.0699997
-6.2279620	5.0752001	110.6699982
-6.2279620	5.1240001	108.7099991
-6.2279620	5.1728001	106.8300018
-6.2279620	5.2216001	106.1299973
-6.2279620	5.2704000	108.0499954
-6.2279620	5.3192000	109.4099960
-6.2279620	5.3680000	108.7299957
-6.2279620	5.4168000	106.4700012
-6.2279620	5.4656000	103.3600006
-6.2279620	5.5144000	107.7999954
-6.2279620	5.5632000	108.2799988
-6.2279620	5.6120000	107.8499985
-6.2279620	5.6608000	109.4099960
-6.2279620	5.7096000	109.6100006
-6.2279620	5.7584000	107.1299973
-6.2279620	5.8072000	108.9700012
-6.2279620	5.8559999	107.2399979
-6.2279620	5.9047999	105.0000000
-6.2279620	5.9535999	103.8699951
-6.2279620	6.0023999	103.8899994
-6.2279620	6.0511999	106.5800018
-6.2279620	6.0999999	103.9700012
-6.2279620	6.1487999	104.6599960
-6.2279620	6.1975999	104.5599976
-6.2279620	6.2463999	105.5800018
-6.2279620	6.2951999	103.0000000
-6.2279620	6.3439999	102.6699982

-6.2279620	6.3927999	109.9700012
-6.2279620	6.4415998	111.8399963
-6.2279620	6.4903998	111.0699997
-6.2279620	6.5391998	107.7999954
-6.2279620	6.5879998	106.4499969
-6.2279620	6.6367998	106.2799988
-6.2279620	6.6855998	103.1199951
-6.2279620	6.7343998	103.7799988
-6.2279620	6.7831998	104.2500000
-6.2279620	6.8319998	103.9899979
-6.2279620	6.8807998	102.0899963
-6.2279620	6.9295998	102.7200012
-6.2279620	6.9783998	106.5100021
-6.2279620	7.0271997	107.8600006
-6.2279620	7.0759997	106.2900009
-6.2279620	7.1247997	104.0699997
-6.2279620	7.1735997	104.1800003
-6.2279620	7.2223997	101.6999969
-6.2279620	7.2711997	105.0100021
-6.2279620	7.3199997	105.6100006
-6.2279620	7.3687997	101.4000015
-6.2279620	7.4175997	106.2999954
-6.2279620	7.4663997	104.2900009
-6.2279620	7.5151997	104.0599976
-6.2279620	7.5639997	104.9099960
-6.2279620	7.6127996	104.5400009
-6.2279620	7.6615996	104.5400009
-6.2279620	7.7103996	104.2799988
-6.2279620	7.7591996	105.0499954
-6.2279620	7.8079996	106.9899979
-6.2279620	7.8567996	105.5400009
-6.2279620	7.9056001	101.8899994
-6.2279620	7.9544001	104.0899963
-6.2279620	8.0032005	101.9599991
-6.2279620	8.0520000	101.5800018
-6.2279620	8.1008005	104.9000015
-6.2279620	8.1496000	107.2399979
-6.2279620	8.1984005	107.0100021
-6.2279620	8.2472000	104.8799973
-6.2279620	8.2960005	102.5499954
-6.2279620	8.3448000	105.0199966
-6.2279620	8.3936005	104.0199966
-6.2279620	8.4424000	104.3799973
-6.2279620	8.4912004	102.0100021
-6.2279620	8.5400000	101.0899963
-6.2279620	8.5888004	99.9399948
-6.2279620	8.6375999	101.3899994
-6.2279620	8.6864004	104.6399994
-6.2279620	8.7351999	103.5100021
-6.2279620	8.7840004	101.4199982
-6.2279620	8.8327999	100.5599976
-6.2279620	8.8816004	102.3499985
-6.2279620	8.9303999	102.9700012
-6.2279620	8.9792004	104.0100021
-6.2279620	9.0279999	102.6899948
-6.2279620	9.0768003	101.3600006
-6.2279620	9.1255999	100.9300003
-6.2279620	9.1744003	102.6999969

-6.2279620	9.2231998	101.5000000
-6.2279620	9.2720003	101.9099960
-6.2279620	9.3207998	102.7999954
-6.2279620	9.3696003	96.7299957
-6.2279620	9.4184008	100.1500015
-6.2279620	9.4672003	100.0999985
-6.2279620	9.5160007	100.2900009
-6.2279620	9.5648003	101.9599991
-6.2279620	9.6136007	105.1699982
-6.2279620	9.6624002	107.6100006
-6.2279620	9.7112007	105.9099960
-6.2279620	9.7600002	104.8300018
-6.2279620	9.8088007	103.6100006
-6.2279620	9.8576002	105.0800018
-6.2279620	9.9064007	104.5400009
-6.2279620	9.9552002	104.2799988
-6.2279620	10.0040007	103.0100021
-6.2279620	10.0528002	102.1100006
-6.2279620	10.1016006	102.0699997
-6.2279620	10.1504002	100.5599976
-6.2279620	10.1992006	103.3300018
-6.2279620	10.2480001	106.1800003
-6.2279620	10.2968006	104.5699997
-6.2279620	10.3456001	102.1399994
-6.2279620	10.3944006	98.7500000
-6.2279620	10.4432001	104.9899979
-6.2279620	10.4920006	106.4199982
-6.2279620	10.5408001	102.7600021
-6.2279620	10.5896006	103.4199982
-6.2279620	10.6384001	103.3799973
-6.2279620	10.6872005	100.5100021
-6.2279620	10.7360001	103.0299988
-6.2279620	10.7848005	101.4700012
-6.2279620	10.8336000	102.4199982
-6.2279620	10.8824005	101.0899963
-6.2279620	10.9312000	101.1199951
-6.2279620	10.9800005	101.8099976
-6.2279620	11.0288000	103.5199966
-6.2279620	11.0776005	104.9799957
-6.2279620	11.1264000	105.5400009
-6.2279620	11.1752005	106.8899994
-6.2279620	11.2240000	106.1699982
-6.2279620	11.2728004	104.8399963
-6.2279620	11.3216000	104.7200012
-6.2279620	11.3704004	103.4599991
-6.2279620	11.4191999	103.0000000
-6.2279620	11.4680004	102.4399948
-6.2279620	11.5167999	103.6599960
-6.2279620	11.5656004	102.5299988
-6.2279620	11.6143999	103.2299957
-6.2279620	11.6632004	106.1699982
-6.2279620	11.7119999	105.5999985
-6.2279620	11.7608004	103.0699997
-6.2279620	11.8095999	103.4899979
-6.2279620	11.8584003	105.2399979
-6.2279620	11.9071999	104.3399963
-6.2279620	11.9560003	101.4099960
-6.2279620	12.0047998	100.4599991

-6.2279620	12.0536003	103.5599976
-6.2279620	12.1023998	101.9399948
-6.2279620	12.1512003	102.4000015
-6.2279620	12.1999998	104.0400009
-6.2279620	12.2488003	106.2600021
-6.2279620	12.2975998	103.0899963
-6.2279620	12.3464003	101.0599976
-6.2279620	12.3951998	106.1599960
-6.2279620	12.4440002	107.6800003
-6.2279620	12.4927998	105.3099976
-6.2279620	12.5416002	103.9099960
-6.2279620	12.5904007	104.0800018
-6.2279620	12.6392002	106.2999954
-6.2279620	12.6880007	108.5699997
-6.2279620	12.7368002	107.9700012
-6.2279620	12.7856007	105.8499985
-6.2279620	12.8344002	104.2699966
-6.2279620	12.8832006	101.7299957
-6.2279620	12.9320002	97.6800003
-6.2279620	12.9808006	101.5599976
-6.2279620	13.0296001	103.2399979
-6.2279620	13.0784006	103.3699951
-6.2279620	13.1272001	101.1999969
-6.2279620	13.1760006	97.4199982
-6.2279620	13.2248001	101.2999954
-6.2279620	13.2736006	102.8099976
-6.2279620	13.3224001	101.4000015
-6.2279620	13.3712006	102.6399994
-6.2279620	13.4200001	102.0299988
-6.2279620	13.4688005	100.7099991
-6.2279620	13.5176001	103.5499954
-6.2279620	13.5664005	100.0999985
-6.2279620	13.6152000	103.9799957
-6.2279620	13.6640005	104.5100021
-6.2279620	13.7128000	103.9599991
-6.2279620	13.7616005	105.9399948
-6.2279620	13.8104000	104.7900009
-6.2279620	13.8592005	104.0000000
-6.2279620	13.9080000	99.8799973
-6.2279620	13.9568005	99.6199951
-6.2279620	14.0056000	94.9799957
-6.2279620	14.0544004	101.7399979
-6.2279620	14.1032000	104.4700012
-6.2279620	14.1520004	104.1199951
-6.2279620	14.2007999	102.8300018
-6.2279620	14.2496004	104.8699951
-6.2279620	14.2983999	105.2799988
-6.2279620	14.3472004	102.8099976
-6.2279620	14.3959999	102.5400009
-6.2279620	14.4448004	104.0800018
-6.2279620	14.4935999	105.9199982
-6.2279620	14.5424004	102.5499954
-6.2279620	14.5911999	100.5699997
-6.2279620	14.6400003	101.2399979
-6.2279620	14.6887999	102.2799988
-6.2279620	14.7376003	105.4899979
-6.2279620	14.7863998	104.8600006
-6.2279620	14.8352003	101.8899994

-6.2279620	14.8839998	103.7799988
-6.2279620	14.9328003	102.1399994
-6.2279620	14.9815998	103.3799973
-6.2279620	15.0304003	104.8899994
-6.2218261	0.3416000	106.5000000
-6.2218261	0.3904000	107.1599960
-6.2218261	0.4392000	108.4000015
-6.2218261	0.4880000	106.5699997
-6.2218261	0.5368000	103.8099976
-6.2218261	0.5856000	103.2600021
-6.2218261	0.6344000	106.8300018
-6.2218261	0.6832000	104.5599976
-6.2218261	0.7320000	104.1299973
-6.2218261	0.7808000	107.3499985
-6.2218261	0.8296000	107.4499969
-6.2218261	0.8784000	108.1500015
-6.2218261	0.9272000	108.1599960
-6.2218261	0.9760000	110.6399994
-6.2218261	1.0247999	108.3300018
-6.2218261	1.0735999	103.1899948
-6.2218261	1.1223999	107.5299988
-6.2218261	1.1711999	107.0599976
-6.2218261	1.2200000	106.5100021
-6.2218261	1.2688000	101.7399979
-6.2218261	1.3176000	105.9300003
-6.2218261	1.3664000	105.8499985
-6.2218261	1.4152000	107.5000000
-6.2218261	1.4640000	106.9000015
-6.2218261	1.5128000	106.9599991
-6.2218261	1.5616000	108.0999985
-6.2218261	1.6104000	111.3199997
-6.2218261	1.6592000	110.5400009
-6.2218261	1.7079999	113.2900009
-6.2218261	1.7567999	112.7299957
-6.2218261	1.8055999	114.3699951
-6.2218261	1.8543999	118.5499954
-6.2218261	1.9031999	118.7500000
-6.2218261	1.9519999	112.2600021
-6.2218261	2.0007999	107.5199966
-6.2218261	2.0495999	109.5800018
-6.2218261	2.0983999	108.3600006
-6.2218261	2.1471999	109.1500015
-6.2218261	2.1959999	111.1899948
-6.2218261	2.2447999	109.3300018
-6.2218261	2.2935998	105.4799957
-6.2218261	2.3423998	104.6500015
-6.2218261	2.3912001	102.6100006
-6.2218261	2.4400001	105.1100006
-6.2218261	2.4888000	107.0199966
-6.2218261	2.5376000	109.0299988
-6.2218261	2.5864000	107.2699966
-6.2218261	2.6352000	109.2500000
-6.2218261	2.6840000	107.4199982
-6.2218261	2.7328000	103.1500015
-6.2218261	2.7816000	102.7999954
-6.2218261	2.8304000	103.8099976
-6.2218261	2.8792000	105.7799988
-6.2218261	2.9280000	107.2600021

-6.2218261	2.9768000	109.0299988
-6.2218261	3.0256000	107.2699966
-6.2218261	3.0743999	104.7699966
-6.2218261	3.1231999	101.6999969
-6.2218261	3.1719999	103.8300018
-6.2218261	3.2207999	105.3499985
-6.2218261	3.2695999	104.8300018
-6.2218261	3.3183999	107.0000000
-6.2218261	3.3671999	106.1500015
-6.2218261	3.4159999	102.4399948
-6.2218261	3.4647999	104.8499985
-6.2218261	3.5135999	105.7999954
-6.2218261	3.5623999	105.6399994
-6.2218261	3.6111999	104.1899948
-6.2218261	3.6599998	101.2500000
-6.2218261	3.7087998	97.6899948
-6.2218261	3.7575998	103.5999985
-6.2218261	3.8063998	104.5199966
-6.2218261	3.8551998	101.3499985
-6.2218261	3.9039998	100.7200012
-6.2218261	3.9527998	102.5999985
-6.2218261	4.0016003	100.1399994
-6.2218261	4.0503998	102.1500015
-6.2218261	4.0991998	100.0000000
-6.2218261	4.1479998	101.6299973
-6.2218261	4.1967998	103.0400009
-6.2218261	4.2455997	101.7399979
-6.2218261	4.2943997	108.2799988
-6.2218261	4.3431997	113.4300003
-6.2218261	4.3919997	113.3899994
-6.2218261	4.4407997	111.7099991
-6.2218261	4.4895997	115.1599960
-6.2218261	4.5383997	116.8099976
-6.2218261	4.5871997	115.6999969
-6.2218261	4.6359997	111.3199997
-6.2218261	4.6847997	114.7699966
-6.2218261	4.7336001	110.1599960
-6.2218261	4.7824001	105.8399963
-6.2218261	4.8312001	110.7500000
-6.2218261	4.8800001	109.8699951
-6.2218261	4.9288001	107.0699997
-6.2218261	4.9776001	106.1999969
-6.2218261	5.0264001	108.4300003
-6.2218261	5.0752001	111.4300003
-6.2218261	5.1240001	108.5100021
-6.2218261	5.1728001	106.5800018
-6.2218261	5.2216001	106.0299988
-6.2218261	5.2704000	108.1100006
-6.2218261	5.3192000	109.3399963
-6.2218261	5.3680000	108.7200012
-6.2218261	5.4168000	106.3699951
-6.2218261	5.4656000	102.4499969
-6.2218261	5.5144000	107.5299988
-6.2218261	5.5632000	108.3199997
-6.2218261	5.6120000	107.9000015
-6.2218261	5.6608000	108.7600021
-6.2218261	5.7096000	109.4700012
-6.2218261	5.7584000	108.8399963

-6.2218261	5.8072000	109.5499954
-6.2218261	5.8559999	107.3600006
-6.2218261	5.9047999	104.2299957
-6.2218261	5.9535999	104.0999985
-6.2218261	6.0023999	105.1699982
-6.2218261	6.0511999	107.0699997
-6.2218261	6.0999999	103.2799988
-6.2218261	6.1487999	104.1800003
-6.2218261	6.1975999	105.8399963
-6.2218261	6.2463999	106.1100006
-6.2218261	6.2951999	101.9199982
-6.2218261	6.3439999	101.5400009
-6.2218261	6.3927999	109.2099991
-6.2218261	6.4415998	110.9599991
-6.2218261	6.4903998	111.5199966
-6.2218261	6.5391998	108.0199966
-6.2218261	6.5879998	106.1500015
-6.2218261	6.6367998	106.5899963
-6.2218261	6.6855998	103.6599960
-6.2218261	6.7343998	103.7399979
-6.2218261	6.7831998	103.8799973
-6.2218261	6.8319998	103.8699951
-6.2218261	6.8807998	101.9000015
-6.2218261	6.9295998	101.5499954
-6.2218261	6.9783998	105.3399963
-6.2218261	7.0271997	107.4000015
-6.2218261	7.0759997	106.4499969
-6.2218261	7.1247997	104.7500000
-6.2218261	7.1735997	104.0499954
-6.2218261	7.2223997	102.5699997
-6.2218261	7.2711997	105.7600021
-6.2218261	7.3199997	104.6299973
-6.2218261	7.3687997	99.5699997
-6.2218261	7.4175997	105.9000015
-6.2218261	7.4663997	103.3600006
-6.2218261	7.5151997	104.1500015
-6.2218261	7.5639997	105.8399963
-6.2218261	7.6127996	105.2600021
-6.2218261	7.6615996	104.6699982
-6.2218261	7.7103996	104.3799973
-6.2218261	7.7591996	105.0599976
-6.2218261	7.8079996	106.8199997
-6.2218261	7.8567996	105.7699966
-6.2218261	7.9056001	101.8199997
-6.2218261	7.9544001	103.9499969
-6.2218261	8.0032005	102.3699951
-6.2218261	8.0520000	102.1699982
-6.2218261	8.1008005	105.5000000
-6.2218261	8.1496000	107.8499985
-6.2218261	8.1984005	107.6199951
-6.2218261	8.2472000	105.3099976
-6.2218261	8.2960005	102.6599960
-6.2218261	8.3448000	104.6500015
-6.2218261	8.3936005	103.1599960
-6.2218261	8.4424000	103.2799988
-6.2218261	8.4912004	101.2699966
-6.2218261	8.5400000	100.6199951
-6.2218261	8.5888004	99.3399963

-6.2218261	8.6375999	102.1899948
-6.2218261	8.6864004	105.0100021
-6.2218261	8.7351999	103.0599976
-6.2218261	8.7840004	101.0599976
-6.2218261	8.8327999	100.7099991
-6.2218261	8.8816004	101.3600006
-6.2218261	8.9303999	101.8099976
-6.2218261	8.9792004	103.1699982
-6.2218261	9.0279999	101.7299957
-6.2218261	9.0768003	101.3399963
-6.2218261	9.1255999	100.8600006
-6.2218261	9.1744003	103.1999969
-6.2218261	9.2231998	102.0299988
-6.2218261	9.2720003	101.8799973
-6.2218261	9.3207998	103.6800003
-6.2218261	9.3696003	99.4799957
-6.2218261	9.4184008	100.7699966
-6.2218261	9.4672003	100.3099976
-6.2218261	9.5160007	99.0299988
-6.2218261	9.5648003	100.9599991
-6.2218261	9.6136007	104.9199982
-6.2218261	9.6624002	107.3699951
-6.2218261	9.7112007	105.6500015
-6.2218261	9.7600002	104.4599991
-6.2218261	9.8088007	103.2099991
-6.2218261	9.8576002	104.6399994
-6.2218261	9.9064007	105.0999985
-6.2218261	9.9552002	104.8899994
-6.2218261	10.0040007	103.3300018
-6.2218261	10.0528002	100.9499969
-6.2218261	10.1016006	101.7999954
-6.2218261	10.1504002	100.9300003
-6.2218261	10.1992006	102.9899979
-6.2218261	10.2480001	105.0800018
-6.2218261	10.2968006	103.9799957
-6.2218261	10.3456001	102.3899994
-6.2218261	10.3944006	98.9700012
-6.2218261	10.4432001	104.5000000
-6.2218261	10.4920006	106.0800018
-6.2218261	10.5408001	102.2900009
-6.2218261	10.5896006	103.3399963
-6.2218261	10.6384001	104.0299988
-6.2218261	10.6872005	102.1500015
-6.2218261	10.7360001	103.6800003
-6.2218261	10.7848005	101.4700012
-6.2218261	10.8336000	102.5000000
-6.2218261	10.8824005	100.6999969
-6.2218261	10.9312000	101.0499954
-6.2218261	10.9800005	101.7200012
-6.2218261	11.0288000	103.4899979
-6.2218261	11.0776005	105.5299988
-6.2218261	11.1264000	106.9099960
-6.2218261	11.1752005	107.1800003
-6.2218261	11.2240000	105.5299988
-6.2218261	11.2728004	104.9399948
-6.2218261	11.3216000	104.3499985
-6.2218261	11.3704004	104.0699997
-6.2218261	11.4191999	103.4399948

-6.2218261	11.4680004	102.6599960
-6.2218261	11.5167999	103.2600021
-6.2218261	11.5656004	102.2500000
-6.2218261	11.6143999	103.4599991
-6.2218261	11.6632004	106.8799973
-6.2218261	11.7119999	106.3300018
-6.2218261	11.7608004	103.4399948
-6.2218261	11.8095999	104.1800003
-6.2218261	11.8584003	105.6899948
-6.2218261	11.9071999	104.6699982
-6.2218261	11.9560003	101.8600006
-6.2218261	12.0047998	102.2299957
-6.2218261	12.0536003	104.3199997
-6.2218261	12.1023998	102.8699951
-6.2218261	12.1512003	103.4799957
-6.2218261	12.1999998	104.0800018
-6.2218261	12.2488003	106.5599976
-6.2218261	12.2975998	103.0000000
-6.2218261	12.3464003	102.0299988
-6.2218261	12.3951998	106.9899979
-6.2218261	12.4440002	107.9199982
-6.2218261	12.4927998	105.9700012
-6.2218261	12.5416002	104.0100021
-6.2218261	12.5904007	103.6399994
-6.2218261	12.6392002	106.1899948
-6.2218261	12.6880007	108.3399963
-6.2218261	12.7368002	107.5999985
-6.2218261	12.7856007	105.8199997
-6.2218261	12.8344002	104.3600006
-6.2218261	12.8832006	102.4000015
-6.2218261	12.9320002	98.8600006
-6.2218261	12.9808006	101.2299957
-6.2218261	13.0296001	102.3300018
-6.2218261	13.0784006	102.8699951
-6.2218261	13.1272001	101.5100021
-6.2218261	13.1760006	98.9499969
-6.2218261	13.2248001	101.6399994
-6.2218261	13.2736006	102.8799973
-6.2218261	13.3224001	101.5899963
-6.2218261	13.3712006	101.9199982
-6.2218261	13.4200001	100.9000015
-6.2218261	13.4688005	100.4399948
-6.2218261	13.5176001	104.1899948
-6.2218261	13.5664005	101.3899994
-6.2218261	13.6152000	103.0400009
-6.2218261	13.6640005	103.8899994
-6.2218261	13.7128000	102.8300018
-6.2218261	13.7616005	105.5100021
-6.2218261	13.8104000	103.7500000
-6.2218261	13.8592005	103.9799957
-6.2218261	13.9080000	100.2399979
-6.2218261	13.9568005	99.5899963
-6.2218261	14.0056000	96.5999985
-6.2218261	14.0544004	101.0999985
-6.2218261	14.1032000	103.3699951
-6.2218261	14.1520004	103.8300018
-6.2218261	14.2007999	103.2600021
-6.2218261	14.2496004	105.5199966

-6.2218261	14.2983999	104.8199997
-6.2218261	14.3472004	102.1999969
-6.2218261	14.3959999	102.5699997
-6.2218261	14.4448004	104.0699997
-6.2218261	14.4935999	105.4499969
-6.2218261	14.5424004	102.6899948
-6.2218261	14.5911999	99.5899963
-6.2218261	14.6400003	101.2500000
-6.2218261	14.6887999	101.6699982
-6.2218261	14.7376003	105.7200012
-6.2218261	14.7863998	105.1899948
-6.2218261	14.8352003	101.7900009
-6.2218261	14.8839998	103.5999985
-6.2218261	14.9328003	101.6500015
-6.2218261	14.9815998	102.4499969
-6.2218261	15.0304003	104.6899948
-6.2156901	0.3416000	105.8199997
-6.2156901	0.3904000	107.5299988
-6.2156901	0.4392000	107.7699966
-6.2156901	0.4880000	105.0699997
-6.2156901	0.5368000	103.9099960
-6.2156901	0.5856000	104.4199982
-6.2156901	0.6344000	106.2299957
-6.2156901	0.6832000	104.3899994
-6.2156901	0.7320000	105.3499985
-6.2156901	0.7808000	107.9599991
-6.2156901	0.8296000	107.5800018
-6.2156901	0.8784000	108.7500000
-6.2156901	0.9272000	108.8899994
-6.2156901	0.9760000	110.7799988
-6.2156901	1.0247999	108.8199997
-6.2156901	1.0735999	104.0000000
-6.2156901	1.1223999	107.8600006
-6.2156901	1.1711999	108.0100021
-6.2156901	1.2200000	107.1800003
-6.2156901	1.2688000	102.3399963
-6.2156901	1.3176000	106.2799988
-6.2156901	1.3664000	105.9199982
-6.2156901	1.4152000	107.7799988
-6.2156901	1.4640000	108.0299988
-6.2156901	1.5128000	107.2799988
-6.2156901	1.5616000	107.7799988
-6.2156901	1.6104000	110.7200012
-6.2156901	1.6592000	109.5100021
-6.2156901	1.7079999	112.5400009
-6.2156901	1.7567999	112.2600021
-6.2156901	1.8055999	114.0299988
-6.2156901	1.8543999	119.2600021
-6.2156901	1.9031999	119.1999969
-6.2156901	1.9519999	112.6999969
-6.2156901	2.0007999	108.6399994
-6.2156901	2.0495999	110.3399963
-6.2156901	2.0983999	109.1399994
-6.2156901	2.1471999	108.5400009
-6.2156901	2.1959999	110.8600006
-6.2156901	2.2447999	109.2600021
-6.2156901	2.2935998	104.3499985
-6.2156901	2.3423998	103.6699982

-6.2156901	2.3912001	102.4700012
-6.2156901	2.4400001	105.0400009
-6.2156901	2.4888000	106.5100021
-6.2156901	2.5376000	109.1800003
-6.2156901	2.5864000	107.9799957
-6.2156901	2.6352000	108.9700012
-6.2156901	2.6840000	106.7900009
-6.2156901	2.7328000	102.1599960
-6.2156901	2.7816000	103.3199997
-6.2156901	2.8304000	104.3899994
-6.2156901	2.8792000	105.8600006
-6.2156901	2.9280000	107.6399994
-6.2156901	2.9768000	109.2799988
-6.2156901	3.0256000	107.5499954
-6.2156901	3.0743999	105.5699997
-6.2156901	3.1231999	102.3600006
-6.2156901	3.1719999	102.7699966
-6.2156901	3.2207999	103.0899963
-6.2156901	3.2695999	104.1399994
-6.2156901	3.3183999	106.4499969
-6.2156901	3.3671999	106.0599976
-6.2156901	3.4159999	103.0299988
-6.2156901	3.4647999	105.1699982
-6.2156901	3.5135999	105.7200012
-6.2156901	3.5623999	106.0699997
-6.2156901	3.6111999	104.5299988
-6.2156901	3.6599998	102.3300018
-6.2156901	3.7087998	98.8600006
-6.2156901	3.7575998	103.0199966
-6.2156901	3.8063998	104.1699982
-6.2156901	3.8551998	101.3600006
-6.2156901	3.9039998	100.7699966
-6.2156901	3.9527998	102.0299988
-6.2156901	4.0016003	100.0899963
-6.2156901	4.0503998	102.1699982
-6.2156901	4.0991998	98.7500000
-6.2156901	4.1479998	101.2500000
-6.2156901	4.1967998	102.7799988
-6.2156901	4.2455997	101.2500000
-6.2156901	4.2943997	108.1800003
-6.2156901	4.3431997	113.0800018
-6.2156901	4.3919997	112.6299973
-6.2156901	4.4407997	110.1800003
-6.2156901	4.4895997	113.6199951
-6.2156901	4.5383997	115.7399979
-6.2156901	4.5871997	116.2900009
-6.2156901	4.6359997	111.2500000
-6.2156901	4.6847997	114.7600021
-6.2156901	4.7336001	109.8499985
-6.2156901	4.7824001	107.0999985
-6.2156901	4.8312001	110.6299973
-6.2156901	4.8800001	108.2399979
-6.2156901	4.9288001	106.1800003
-6.2156901	4.9776001	106.8300018
-6.2156901	5.0264001	109.5699997
-6.2156901	5.0752001	112.1299973
-6.2156901	5.1240001	109.5699997
-6.2156901	5.1728001	106.9899979

-6.2156901	5.2216001	106.5100021
-6.2156901	5.2704000	108.1399994
-6.2156901	5.3192000	109.1399994
-6.2156901	5.3680000	108.8399963
-6.2156901	5.4168000	106.3499985
-6.2156901	5.4656000	101.3399963
-6.2156901	5.5144000	107.0000000
-6.2156901	5.5632000	107.9799957
-6.2156901	5.6120000	107.8300018
-6.2156901	5.6608000	108.0400009
-6.2156901	5.7096000	108.9099960
-6.2156901	5.7584000	109.0899963
-6.2156901	5.8072000	109.2299957
-6.2156901	5.8559999	107.0599976
-6.2156901	5.9047999	103.5499954
-6.2156901	5.9535999	104.0499954
-6.2156901	6.0023999	105.9099960
-6.2156901	6.0511999	107.4199982
-6.2156901	6.0999999	102.9899979
-6.2156901	6.1487999	103.5299988
-6.2156901	6.1975999	106.4499969
-6.2156901	6.2463999	107.0999985
-6.2156901	6.2951999	102.6699982
-6.2156901	6.3439999	100.4199982
-6.2156901	6.3927999	108.2200012
-6.2156901	6.4415998	109.7500000
-6.2156901	6.4903998	111.6500015
-6.2156901	6.5391998	107.8499985
-6.2156901	6.5879998	106.0699997
-6.2156901	6.6367998	106.5199966
-6.2156901	6.6855998	103.9099960
-6.2156901	6.7343998	103.5800018
-6.2156901	6.7831998	103.3799973
-6.2156901	6.8319998	103.9700012
-6.2156901	6.8807998	102.1299973
-6.2156901	6.9295998	100.6100006
-6.2156901	6.9783998	103.9099960
-6.2156901	7.0271997	106.0199966
-6.2156901	7.0759997	106.2699966
-6.2156901	7.1247997	105.2900009
-6.2156901	7.1735997	103.8600006
-6.2156901	7.2223997	102.8699951
-6.2156901	7.2711997	105.8300018
-6.2156901	7.3199997	103.2699966
-6.2156901	7.3687997	98.7099991
-6.2156901	7.4175997	105.2299957
-6.2156901	7.4663997	102.4199982
-6.2156901	7.5151997	104.7999954
-6.2156901	7.5639997	107.2699966
-6.2156901	7.6127996	106.0699997
-6.2156901	7.6615996	104.7200012
-6.2156901	7.7103996	104.4199982
-6.2156901	7.7591996	104.6100006
-6.2156901	7.8079996	105.9300003
-6.2156901	7.8567996	105.8099976
-6.2156901	7.9056001	101.7500000
-6.2156901	7.9544001	103.8799973
-6.2156901	8.0032005	102.9399948

-6.2156901	8.0520000	103.2999954
-6.2156901	8.1008005	106.0999985
-6.2156901	8.1496000	108.2200012
-6.2156901	8.1984005	107.6800003
-6.2156901	8.2472000	105.3199997
-6.2156901	8.2960005	102.6899948
-6.2156901	8.3448000	104.3399963
-6.2156901	8.3936005	102.7699966
-6.2156901	8.4424000	103.4599991
-6.2156901	8.4912004	101.8799973
-6.2156901	8.5400000	101.1100006
-6.2156901	8.5888004	99.5400009
-6.2156901	8.6375999	102.8300018
-6.2156901	8.6864004	104.9499969
-6.2156901	8.7351999	102.2999954
-6.2156901	8.7840004	101.3300018
-6.2156901	8.8327999	101.4000015
-6.2156901	8.8816004	100.8899994
-6.2156901	8.9303999	101.9499969
-6.2156901	8.9792004	103.3699951
-6.2156901	9.0279999	102.0699997
-6.2156901	9.0768003	102.5000000
-6.2156901	9.1255999	102.2200012
-6.2156901	9.1744003	103.7399979
-6.2156901	9.2231998	102.4899979
-6.2156901	9.2720003	101.3699951
-6.2156901	9.3207998	103.9199982
-6.2156901	9.3696003	102.0699997
-6.2156901	9.4184008	101.3399963
-6.2156901	9.4672003	100.5800018
-6.2156901	9.5160007	98.1299973
-6.2156901	9.5648003	100.8600006
-6.2156901	9.6136007	104.2500000
-6.2156901	9.6624002	106.8499985
-6.2156901	9.7112007	104.9899979
-6.2156901	9.7600002	103.7799988
-6.2156901	9.8088007	103.2099991
-6.2156901	9.8576002	104.8699951
-6.2156901	9.9064007	105.3199997
-6.2156901	9.9552002	104.7999954
-6.2156901	10.0040007	102.9899979
-6.2156901	10.0528002	99.3899994
-6.2156901	10.1016006	101.0899963
-6.2156901	10.1504002	101.1800003
-6.2156901	10.1992006	103.5000000
-6.2156901	10.2480001	103.9700012
-6.2156901	10.2968006	103.2399979
-6.2156901	10.3456001	102.4799957
-6.2156901	10.3944006	99.6500015
-6.2156901	10.4432001	103.6699982
-6.2156901	10.4920006	105.5100021
-6.2156901	10.5408001	102.2099991
-6.2156901	10.5896006	103.7799988
-6.2156901	10.6384001	104.4199982
-6.2156901	10.6872005	103.1299973
-6.2156901	10.7360001	103.7600021
-6.2156901	10.7848005	101.0699997
-6.2156901	10.8336000	102.2399979

-6.2156901	10.8824005	101.0100021
-6.2156901	10.9312000	100.9300003
-6.2156901	10.9800005	101.6599960
-6.2156901	11.0288000	103.2600021
-6.2156901	11.0776005	105.1100006
-6.2156901	11.1264000	107.3499985
-6.2156901	11.1752005	106.9499969
-6.2156901	11.2240000	105.2999954
-6.2156901	11.2728004	104.7799988
-6.2156901	11.3216000	103.3600006
-6.2156901	11.3704004	104.9700012
-6.2156901	11.4191999	103.9199982
-6.2156901	11.4680004	102.4000015
-6.2156901	11.5167999	102.6800003
-6.2156901	11.5656004	101.7699966
-6.2156901	11.6143999	103.2600021
-6.2156901	11.6632004	106.9300003
-6.2156901	11.7119999	106.7200012
-6.2156901	11.7608004	104.0199966
-6.2156901	11.8095999	104.5899963
-6.2156901	11.8584003	105.4000015
-6.2156901	11.9071999	104.6599960
-6.2156901	11.9560003	102.0400009
-6.2156901	12.0047998	103.5699997
-6.2156901	12.0536003	104.6399994
-6.2156901	12.1023998	103.8699951
-6.2156901	12.1512003	104.6399994
-6.2156901	12.1999998	104.9199982
-6.2156901	12.2488003	106.4499969
-6.2156901	12.2975998	102.5199966
-6.2156901	12.3464003	102.5800018
-6.2156901	12.3951998	106.9099960
-6.2156901	12.4440002	107.3199997
-6.2156901	12.4927998	105.6599960
-6.2156901	12.5416002	104.1999969
-6.2156901	12.5904007	103.2099991
-6.2156901	12.6392002	105.6999969
-6.2156901	12.6880007	107.3699951
-6.2156901	12.7368002	106.7399979
-6.2156901	12.7856007	105.3899994
-6.2156901	12.8344002	104.5899963
-6.2156901	12.8832006	103.2699966
-6.2156901	12.9320002	100.2500000
-6.2156901	12.9808006	100.7600021
-6.2156901	13.0296001	101.5699997
-6.2156901	13.0784006	103.1599960
-6.2156901	13.1272001	103.2299957
-6.2156901	13.1760006	101.1100006
-6.2156901	13.2248001	101.9199982
-6.2156901	13.2736006	103.4199982
-6.2156901	13.3224001	102.0899963
-6.2156901	13.3712006	101.1299973
-6.2156901	13.4200001	100.1699982
-6.2156901	13.4688005	101.0199966
-6.2156901	13.5176001	104.9799957
-6.2156901	13.5664005	102.5999985
-6.2156901	13.6152000	101.4300003
-6.2156901	13.6640005	102.7600021

-6.2156901	13.7128000	100.9599991
-6.2156901	13.7616005	104.8300018
-6.2156901	13.8104000	102.2699966
-6.2156901	13.8592005	103.0299988
-6.2156901	13.9080000	100.2600021
-6.2156901	13.9568005	99.3499985
-6.2156901	14.0056000	97.7099991
-6.2156901	14.0544004	100.2900009
-6.2156901	14.1032000	101.8699951
-6.2156901	14.1520004	102.5899963
-6.2156901	14.2007999	102.6999969
-6.2156901	14.2496004	105.3899994
-6.2156901	14.2983999	103.7200012
-6.2156901	14.3472004	101.3099976
-6.2156901	14.3959999	102.8300018
-6.2156901	14.4448004	104.4899979
-6.2156901	14.4935999	105.2399979
-6.2156901	14.5424004	102.7699966
-6.2156901	14.5911999	98.3499985
-6.2156901	14.6400003	101.0899963
-6.2156901	14.6887999	102.1199951
-6.2156901	14.7376003	106.1199951
-6.2156901	14.7863998	105.3499985
-6.2156901	14.8352003	102.3699951
-6.2156901	14.8839998	103.4499969
-6.2156901	14.9328003	100.3499985
-6.2156901	14.9815998	101.8199997
-6.2156901	15.0304003	104.3699951
-6.2095542	0.3416000	104.5699997
-6.2095542	0.3904000	108.0599976
-6.2095542	0.4392000	107.6399994
-6.2095542	0.4880000	104.1800003
-6.2095542	0.5368000	104.2900009
-6.2095542	0.5856000	105.1299973
-6.2095542	0.6344000	105.2900009
-6.2095542	0.6832000	104.5999985
-6.2095542	0.7320000	106.3799973
-6.2095542	0.7808000	108.7699966
-6.2095542	0.8296000	108.5199966
-6.2095542	0.8784000	109.2200012
-6.2095542	0.9272000	109.3199997
-6.2095542	0.9760000	110.9300003
-6.2095542	1.0247999	109.6699982
-6.2095542	1.0735999	105.9300003
-6.2095542	1.1223999	108.3399963
-6.2095542	1.1711999	108.7799988
-6.2095542	1.2200000	107.4199982
-6.2095542	1.2688000	102.8099976
-6.2095542	1.3176000	106.3899994
-6.2095542	1.3664000	106.0899963
-6.2095542	1.4152000	108.2099991
-6.2095542	1.4640000	109.0699997
-6.2095542	1.5128000	108.4399948
-6.2095542	1.5616000	107.8199997
-6.2095542	1.6104000	110.2699966
-6.2095542	1.6592000	108.9199982
-6.2095542	1.7079999	112.0899963
-6.2095542	1.7567999	112.1399994

-6.2095542	1.8055999	113.5999985
-6.2095542	1.8543999	119.6800003
-6.2095542	1.9031999	119.3699951
-6.2095542	1.9519999	112.9499969
-6.2095542	2.0007999	109.9000015
-6.2095542	2.0495999	111.3399963
-6.2095542	2.0983999	109.5299988
-6.2095542	2.1471999	107.6100006
-6.2095542	2.1959999	110.1399994
-6.2095542	2.2447999	108.9099960
-6.2095542	2.2935998	103.0999985
-6.2095542	2.3423998	103.8499985
-6.2095542	2.3912001	103.0999985
-6.2095542	2.4400001	105.7900009
-6.2095542	2.4888000	106.5100021
-6.2095542	2.5376000	108.9499969
-6.2095542	2.5864000	107.8600006
-6.2095542	2.6352000	108.3399963
-6.2095542	2.6840000	106.3600006
-6.2095542	2.7328000	101.6399994
-6.2095542	2.7816000	104.3499985
-6.2095542	2.8304000	105.0000000
-6.2095542	2.8792000	105.6299973
-6.2095542	2.9280000	106.9599991
-6.2095542	2.9768000	108.9899979
-6.2095542	3.0256000	107.9199982
-6.2095542	3.0743999	106.3300018
-6.2095542	3.1231999	103.5499954
-6.2095542	3.1719999	102.4399948
-6.2095542	3.2207999	101.1399994
-6.2095542	3.2695999	103.1399994
-6.2095542	3.3183999	105.6299973
-6.2095542	3.3671999	105.9700012
-6.2095542	3.4159999	103.9199982
-6.2095542	3.4647999	105.4199982
-6.2095542	3.5135999	105.7900009
-6.2095542	3.5623999	106.4300003
-6.2095542	3.6111999	104.9899979
-6.2095542	3.6599998	104.0100021
-6.2095542	3.7087998	101.0699997
-6.2095542	3.7575998	102.2600021
-6.2095542	3.8063998	103.5499954
-6.2095542	3.8551998	101.9199982
-6.2095542	3.9039998	101.7500000
-6.2095542	3.9527998	101.8899994
-6.2095542	4.0016003	100.5800018
-6.2095542	4.0503998	102.5000000
-6.2095542	4.0991998	98.9099960
-6.2095542	4.1479998	101.2799988
-6.2095542	4.1967998	102.8099976
-6.2095542	4.2455997	101.1699982
-6.2095542	4.2943997	108.2399979
-6.2095542	4.3431997	112.9000015
-6.2095542	4.3919997	112.1800003
-6.2095542	4.4407997	109.6100006
-6.2095542	4.4895997	112.4799957
-6.2095542	4.5383997	114.8199997
-6.2095542	4.5871997	117.1299973

-6.2095542	4.6359997	112.7699966
-6.2095542	4.6847997	114.5599976
-6.2095542	4.7336001	109.0199966
-6.2095542	4.7824001	107.1199951
-6.2095542	4.8312001	110.1500015
-6.2095542	4.8800001	107.4599991
-6.2095542	4.9288001	106.4099960
-6.2095542	4.9776001	108.1699982
-6.2095542	5.0264001	110.3699951
-6.2095542	5.0752001	112.1699982
-6.2095542	5.1240001	109.7099991
-6.2095542	5.1728001	107.2799988
-6.2095542	5.2216001	106.6599960
-6.2095542	5.2704000	108.0299988
-6.2095542	5.3192000	108.6299973
-6.2095542	5.3680000	108.7799988
-6.2095542	5.4168000	106.2200012
-6.2095542	5.4656000	102.0499954
-6.2095542	5.5144000	106.4799957
-6.2095542	5.5632000	107.1899948
-6.2095542	5.6120000	108.3099976
-6.2095542	5.6608000	107.7799988
-6.2095542	5.7096000	108.4300003
-6.2095542	5.7584000	108.4000015
-6.2095542	5.8072000	108.1899948
-6.2095542	5.8559999	105.5499954
-6.2095542	5.9047999	101.2200012
-6.2095542	5.9535999	103.0899963
-6.2095542	6.0023999	106.0499954
-6.2095542	6.0511999	107.8300018
-6.2095542	6.0999999	103.8300018
-6.2095542	6.1487999	103.3699951
-6.2095542	6.1975999	106.8600006
-6.2095542	6.2463999	107.6899948
-6.2095542	6.2951999	103.3899994
-6.2095542	6.3439999	99.0299988
-6.2095542	6.3927999	106.9499969
-6.2095542	6.4415998	108.8300018
-6.2095542	6.4903998	111.4599991
-6.2095542	6.5391998	108.4599991
-6.2095542	6.5879998	105.9899979
-6.2095542	6.6367998	106.1199951
-6.2095542	6.6855998	104.0100021
-6.2095542	6.7343998	103.2600021
-6.2095542	6.7831998	103.2099991
-6.2095542	6.8319998	103.8899994
-6.2095542	6.8807998	101.5400009
-6.2095542	6.9295998	100.4099960
-6.2095542	6.9783998	102.5000000
-6.2095542	7.0271997	104.5100021
-6.2095542	7.0759997	105.6100006
-6.2095542	7.1247997	105.8799973
-6.2095542	7.1735997	103.7999954
-6.2095542	7.2223997	102.8300018
-6.2095542	7.2711997	105.7200012
-6.2095542	7.3199997	102.2900009
-6.2095542	7.3687997	98.4000015
-6.2095542	7.4175997	104.4599991

-6.2095542	7.4663997	101.3199997
-6.2095542	7.5151997	104.4199982
-6.2095542	7.5639997	108.2299957
-6.2095542	7.6127996	106.8300018
-6.2095542	7.6615996	104.7699966
-6.2095542	7.7103996	104.1899948
-6.2095542	7.7591996	103.7500000
-6.2095542	7.8079996	104.6599960
-6.2095542	7.8567996	105.6599960
-6.2095542	7.9056001	102.0599976
-6.2095542	7.9544001	102.3699951
-6.2095542	8.0032005	101.9300003
-6.2095542	8.0520000	103.3600006
-6.2095542	8.1008005	106.0000000
-6.2095542	8.1496000	107.5199966
-6.2095542	8.1984005	107.0499954
-6.2095542	8.2472000	105.6299973
-6.2095542	8.2960005	103.5400009
-6.2095542	8.3448000	103.8799973
-6.2095542	8.3936005	102.6999969
-6.2095542	8.4424000	103.3600006
-6.2095542	8.4912004	102.7200012
-6.2095542	8.5400000	101.8399963
-6.2095542	8.5888004	99.7299957
-6.2095542	8.6375999	103.4499969
-6.2095542	8.6864004	104.6699982
-6.2095542	8.7351999	101.0199966
-6.2095542	8.7840004	101.2699966
-6.2095542	8.8327999	102.0800018
-6.2095542	8.8816004	100.5699997
-6.2095542	8.9303999	102.2500000
-6.2095542	8.9792004	103.5899963
-6.2095542	9.0279999	102.1800003
-6.2095542	9.0768003	103.4399948
-6.2095542	9.1255999	103.1699982
-6.2095542	9.1744003	103.8699951
-6.2095542	9.2231998	102.7399979
-6.2095542	9.2720003	101.1199951
-6.2095542	9.3207998	103.9000015
-6.2095542	9.3696003	103.3600006
-6.2095542	9.4184008	101.1500015
-6.2095542	9.4672003	99.9199982
-6.2095542	9.5160007	97.7900009
-6.2095542	9.5648003	100.4399948
-6.2095542	9.6136007	103.2099991
-6.2095542	9.6624002	106.2600021
-6.2095542	9.7112007	104.4599991
-6.2095542	9.7600002	102.6999969
-6.2095542	9.8088007	102.8199997
-6.2095542	9.8576002	104.6199951
-6.2095542	9.9064007	105.1100006
-6.2095542	9.9552002	104.2500000
-6.2095542	10.0040007	102.3099976
-6.2095542	10.0528002	98.8099976
-6.2095542	10.1016006	100.5800018
-6.2095542	10.1504002	101.7099991
-6.2095542	10.1992006	103.5999985
-6.2095542	10.2480001	102.4799957

-6.2095542	10.2968006	102.8699951
-6.2095542	10.3456001	103.3300018
-6.2095542	10.3944006	101.2699966
-6.2095542	10.4432001	103.5100021
-6.2095542	10.4920006	105.2200012
-6.2095542	10.5408001	103.0899963
-6.2095542	10.5896006	104.5299988
-6.2095542	10.6384001	104.2699966
-6.2095542	10.6872005	103.2699966
-6.2095542	10.7360001	103.4300003
-6.2095542	10.7848005	100.8199997
-6.2095542	10.8336000	101.9700012
-6.2095542	10.8824005	101.2299957
-6.2095542	10.9312000	101.2299957
-6.2095542	10.9800005	101.6299973
-6.2095542	11.0288000	103.2900009
-6.2095542	11.0776005	104.4599991
-6.2095542	11.1264000	106.5999985
-6.2095542	11.1752005	106.0599976
-6.2095542	11.2240000	104.5000000
-6.2095542	11.2728004	104.1199951
-6.2095542	11.3216000	102.2399979
-6.2095542	11.3704004	105.4899979
-6.2095542	11.4191999	104.5599976
-6.2095542	11.4680004	102.7500000
-6.2095542	11.5167999	102.8199997
-6.2095542	11.5656004	102.0599976
-6.2095542	11.6143999	103.5299988
-6.2095542	11.6632004	107.2799988
-6.2095542	11.7119999	107.2299957
-6.2095542	11.7608004	104.8499985
-6.2095542	11.8095999	105.0299988
-6.2095542	11.8584003	104.7799988
-6.2095542	11.9071999	104.1399994
-6.2095542	11.9560003	103.1199951
-6.2095542	12.0047998	105.1699982
-6.2095542	12.0536003	105.2200012
-6.2095542	12.1023998	105.1999969
-6.2095542	12.1512003	105.7200012
-6.2095542	12.1999998	105.5400009
-6.2095542	12.2488003	105.7299957
-6.2095542	12.2975998	101.5999985
-6.2095542	12.3464003	102.9399948
-6.2095542	12.3951998	106.8699951
-6.2095542	12.4440002	106.7299957
-6.2095542	12.4927998	104.7200012
-6.2095542	12.5416002	104.3399963
-6.2095542	12.5904007	102.8099976
-6.2095542	12.6392002	105.3899994
-6.2095542	12.6880007	106.5100021
-6.2095542	12.7368002	106.0199966
-6.2095542	12.7856007	105.1800003
-6.2095542	12.8344002	104.8300018
-6.2095542	12.8832006	103.3899994
-6.2095542	12.9320002	101.2500000
-6.2095542	12.9808006	100.4799957
-6.2095542	13.0296001	101.5999985
-6.2095542	13.0784006	103.6800003

-6.2095542	13.1272001	104.8499985
-6.2095542	13.1760006	103.0000000
-6.2095542	13.2248001	100.9799957
-6.2095542	13.2736006	103.3499985
-6.2095542	13.3224001	102.5199966
-6.2095542	13.3712006	100.3499985
-6.2095542	13.4200001	100.1800003
-6.2095542	13.4688005	102.2399979
-6.2095542	13.5176001	106.0199966
-6.2095542	13.5664005	104.0499954
-6.2095542	13.6152000	100.1199951
-6.2095542	13.6640005	101.5499954
-6.2095542	13.7128000	98.9199982
-6.2095542	13.7616005	103.7399979
-6.2095542	13.8104000	100.4700012
-6.2095542	13.8592005	102.0199966
-6.2095542	13.9080000	100.7999954
-6.2095542	13.9568005	100.3300018
-6.2095542	14.0056000	99.4000015
-6.2095542	14.0544004	100.0800018
-6.2095542	14.1032000	101.0299988
-6.2095542	14.1520004	102.0599976
-6.2095542	14.2007999	102.9700012
-6.2095542	14.2496004	105.5000000
-6.2095542	14.2983999	102.8300018
-6.2095542	14.3472004	100.8600006
-6.2095542	14.3959999	102.8799973
-6.2095542	14.4448004	104.7099991
-6.2095542	14.4935999	104.8799973
-6.2095542	14.5424004	102.5999985
-6.2095542	14.5911999	98.6899948
-6.2095542	14.6400003	100.5499954
-6.2095542	14.6887999	102.5499954
-6.2095542	14.7376003	106.5400009
-6.2095542	14.7863998	105.5299988
-6.2095542	14.8352003	103.0000000
-6.2095542	14.8839998	103.4499969
-6.2095542	14.9328003	99.6599960
-6.2095542	14.9815998	102.0100021
-6.2095542	15.0304003	104.4000015
-6.2034183	0.3416000	104.2500000
-6.2034183	0.3904000	107.4899979
-6.2034183	0.4392000	107.1299973
-6.2034183	0.4880000	103.6899948
-6.2034183	0.5368000	104.8899994
-6.2034183	0.5856000	105.0599976
-6.2034183	0.6344000	105.2299957
-6.2034183	0.6832000	105.0499954
-6.2034183	0.7320000	106.3499985
-6.2034183	0.7808000	108.8899994
-6.2034183	0.8296000	108.8099976
-6.2034183	0.8784000	109.4199982
-6.2034183	0.9272000	109.9099960
-6.2034183	0.9760000	110.8399963
-6.2034183	1.0247999	110.0999985
-6.2034183	1.0735999	107.0599976
-6.2034183	1.1223999	108.0000000
-6.2034183	1.1711999	108.3499985

-6.2034183	1.2200000	107.7699966
-6.2034183	1.2688000	104.5100021
-6.2034183	1.3176000	106.8600006
-6.2034183	1.3664000	106.2900009
-6.2034183	1.4152000	108.3899994
-6.2034183	1.4640000	109.7299957
-6.2034183	1.5128000	109.3699951
-6.2034183	1.5616000	107.9000015
-6.2034183	1.6104000	109.9799957
-6.2034183	1.6592000	108.4599991
-6.2034183	1.7079999	111.3799973
-6.2034183	1.7567999	111.7600021
-6.2034183	1.8055999	113.2900009
-6.2034183	1.8543999	119.7500000
-6.2034183	1.9031999	119.0699997
-6.2034183	1.9519999	112.7999954
-6.2034183	2.0007999	110.3300018
-6.2034183	2.0495999	111.3899994
-6.2034183	2.0983999	109.6199951
-6.2034183	2.1471999	107.0000000
-6.2034183	2.1959999	108.9799957
-6.2034183	2.2447999	108.0999985
-6.2034183	2.2935998	102.2500000
-6.2034183	2.3423998	104.2399979
-6.2034183	2.3912001	104.4700012
-6.2034183	2.4400001	106.6899948
-6.2034183	2.4888000	105.8899994
-6.2034183	2.5376000	108.0299988
-6.2034183	2.5864000	107.2099991
-6.2034183	2.6352000	107.4700012
-6.2034183	2.6840000	106.0299988
-6.2034183	2.7328000	102.2299957
-6.2034183	2.7816000	105.2699966
-6.2034183	2.8304000	105.6399994
-6.2034183	2.8792000	105.3699951
-6.2034183	2.9280000	106.1800003
-6.2034183	2.9768000	108.7500000
-6.2034183	3.0256000	108.2600021
-6.2034183	3.0743999	106.7799988
-6.2034183	3.1231999	103.9599991
-6.2034183	3.1719999	102.0800018
-6.2034183	3.2207999	99.0199966
-6.2034183	3.2695999	102.0599976
-6.2034183	3.3183999	105.0800018
-6.2034183	3.3671999	106.3199997
-6.2034183	3.4159999	104.7200012
-6.2034183	3.4647999	105.4499969
-6.2034183	3.5135999	105.5299988
-6.2034183	3.5623999	106.4799957
-6.2034183	3.6111999	105.2399979
-6.2034183	3.6599998	104.7099991
-6.2034183	3.7087998	102.9399948
-6.2034183	3.7575998	101.4499969
-6.2034183	3.8063998	102.7900009
-6.2034183	3.8551998	102.5299988
-6.2034183	3.9039998	102.9700012
-6.2034183	3.9527998	101.9899979
-6.2034183	4.0016003	101.5599976

-6.2034183	4.0503998	103.1800003
-6.2034183	4.0991998	100.1599960
-6.2034183	4.1479998	102.2500000
-6.2034183	4.1967998	102.6800003
-6.2034183	4.2455997	101.7099991
-6.2034183	4.2943997	108.6800003
-6.2034183	4.3431997	112.9199982
-6.2034183	4.3919997	111.9799957
-6.2034183	4.4407997	109.5000000
-6.2034183	4.4895997	112.0499954
-6.2034183	4.5383997	114.9300003
-6.2034183	4.5871997	118.2099991
-6.2034183	4.6359997	114.3699951
-6.2034183	4.6847997	114.7900009
-6.2034183	4.7336001	109.4199982
-6.2034183	4.7824001	107.6299973
-6.2034183	4.8312001	110.3099976
-6.2034183	4.8800001	107.1399994
-6.2034183	4.9288001	106.7399979
-6.2034183	4.9776001	109.0999985
-6.2034183	5.0264001	110.6500015
-6.2034183	5.0752001	112.2099991
-6.2034183	5.1240001	110.0499954
-6.2034183	5.1728001	107.5499954
-6.2034183	5.2216001	106.6899948
-6.2034183	5.2704000	107.9899979
-6.2034183	5.3192000	108.7099991
-6.2034183	5.3680000	109.0499954
-6.2034183	5.4168000	106.3899994
-6.2034183	5.4656000	102.9399948
-6.2034183	5.5144000	106.1299973
-6.2034183	5.5632000	106.5400009
-6.2034183	5.6120000	108.0400009
-6.2034183	5.6608000	106.8799973
-6.2034183	5.7096000	107.2699966
-6.2034183	5.7584000	107.3300018
-6.2034183	5.8072000	107.0499954
-6.2034183	5.8559999	104.5699997
-6.2034183	5.9047999	100.6399994
-6.2034183	5.9535999	101.5299988
-6.2034183	6.0023999	105.2699966
-6.2034183	6.0511999	107.3799973
-6.2034183	6.0999999	103.7699966
-6.2034183	6.1487999	102.4799957
-6.2034183	6.1975999	107.0800018
-6.2034183	6.2463999	108.2500000
-6.2034183	6.2951999	103.9199982
-6.2034183	6.3439999	98.6699982
-6.2034183	6.3927999	106.3099976
-6.2034183	6.4415998	108.4899979
-6.2034183	6.4903998	110.5400009
-6.2034183	6.5391998	108.6999969
-6.2034183	6.5879998	106.3899994
-6.2034183	6.6367998	105.7900009
-6.2034183	6.6855998	103.7600021
-6.2034183	6.7343998	103.3199997
-6.2034183	6.7831998	103.5599976
-6.2034183	6.8319998	104.2200012

-6.2034183	6.8807998	101.4399948
-6.2034183	6.9295998	100.8300018
-6.2034183	6.9783998	101.8199997
-6.2034183	7.0271997	103.4199982
-6.2034183	7.0759997	104.7999954
-6.2034183	7.1247997	106.0599976
-6.2034183	7.1735997	103.8899994
-6.2034183	7.2223997	103.0999985
-6.2034183	7.2711997	105.2999954
-6.2034183	7.3199997	101.4099960
-6.2034183	7.3687997	98.4700012
-6.2034183	7.4175997	104.0299988
-6.2034183	7.4663997	101.1399994
-6.2034183	7.5151997	103.8499985
-6.2034183	7.5639997	108.7699966
-6.2034183	7.6127996	107.5199966
-6.2034183	7.6615996	104.9799957
-6.2034183	7.7103996	104.1399994
-6.2034183	7.7591996	102.9700012
-6.2034183	7.8079996	102.8799973
-6.2034183	7.8567996	105.1500015
-6.2034183	7.9056001	102.3199997
-6.2034183	7.9544001	101.4599991
-6.2034183	8.0032005	101.0800018
-6.2034183	8.0520000	103.8699951
-6.2034183	8.1008005	106.3399963
-6.2034183	8.1496000	107.0999985
-6.2034183	8.1984005	106.2799988
-6.2034183	8.2472000	105.7399979
-6.2034183	8.2960005	103.2399979
-6.2034183	8.3448000	103.0299988
-6.2034183	8.3936005	102.5699997
-6.2034183	8.4424000	103.3499985
-6.2034183	8.4912004	103.9099960
-6.2034183	8.5400000	102.6999969
-6.2034183	8.5888004	99.7900009
-6.2034183	8.6375999	103.4000015
-6.2034183	8.6864004	104.0899963
-6.2034183	8.7351999	99.9799957
-6.2034183	8.7840004	100.8199997
-6.2034183	8.8327999	102.0400009
-6.2034183	8.8816004	100.5699997
-6.2034183	8.9303999	102.3699951
-6.2034183	8.9792004	103.5699997
-6.2034183	9.0279999	102.1500015
-6.2034183	9.0768003	104.5299988
-6.2034183	9.1255999	104.2099991
-6.2034183	9.1744003	104.2099991
-6.2034183	9.2231998	103.5299988
-6.2034183	9.2720003	101.0699997
-6.2034183	9.3207998	103.9099960
-6.2034183	9.3696003	104.0899963
-6.2034183	9.4184008	101.6100006
-6.2034183	9.4672003	100.4799957
-6.2034183	9.5160007	98.9199982
-6.2034183	9.5648003	100.5999985
-6.2034183	9.6136007	102.5400009
-6.2034183	9.6624002	105.7900009

-6.2034183	9.7112007	104.0199966
-6.2034183	9.7600002	101.8300018
-6.2034183	9.8088007	102.3899994
-6.2034183	9.8576002	104.2200012
-6.2034183	9.9064007	104.7799988
-6.2034183	9.9552002	104.2299957
-6.2034183	10.0040007	102.0000000
-6.2034183	10.0528002	98.3300018
-6.2034183	10.1016006	100.0999985
-6.2034183	10.1504002	101.5100021
-6.2034183	10.1992006	103.4599991
-6.2034183	10.2480001	101.0899963
-6.2034183	10.2968006	102.5999985
-6.2034183	10.3456001	103.4499969
-6.2034183	10.3944006	102.7200012
-6.2034183	10.4432001	103.6899948
-6.2034183	10.4920006	104.5899963
-6.2034183	10.5408001	103.4799957
-6.2034183	10.5896006	104.9799957
-6.2034183	10.6384001	104.3899994
-6.2034183	10.6872005	103.5599976
-6.2034183	10.7360001	103.9300003
-6.2034183	10.7848005	101.8099976
-6.2034183	10.8336000	101.9499969
-6.2034183	10.8824005	101.1299973
-6.2034183	10.9312000	101.6899948
-6.2034183	10.9800005	101.5400009
-6.2034183	11.0288000	103.2200012
-6.2034183	11.0776005	103.6800003
-6.2034183	11.1264000	105.5000000
-6.2034183	11.1752005	105.0199966
-6.2034183	11.2240000	103.8699951
-6.2034183	11.2728004	104.0100021
-6.2034183	11.3216000	101.4199982
-6.2034183	11.3704004	106.0800018
-6.2034183	11.4191999	105.6800003
-6.2034183	11.4680004	103.9199982
-6.2034183	11.5167999	103.4300003
-6.2034183	11.5656004	102.6299973
-6.2034183	11.6143999	103.3300018
-6.2034183	11.6632004	107.1100006
-6.2034183	11.7119999	107.4499969
-6.2034183	11.7608004	105.6500015
-6.2034183	11.8095999	105.5199966
-6.2034183	11.8584003	104.2600021
-6.2034183	11.9071999	104.1800003
-6.2034183	11.9560003	104.2500000
-6.2034183	12.0047998	106.7600021
-6.2034183	12.0536003	105.8699951
-6.2034183	12.1023998	106.3799973
-6.2034183	12.1512003	106.3199997
-6.2034183	12.1999998	105.2799988
-6.2034183	12.2488003	104.4000015
-6.2034183	12.2975998	100.7200012
-6.2034183	12.3464003	103.3699951
-6.2034183	12.3951998	106.7999954
-6.2034183	12.4440002	106.5699997
-6.2034183	12.4927998	104.7900009

-6.2034183	12.5416002	104.8799973
-6.2034183	12.5904007	103.2799988
-6.2034183	12.6392002	105.4199982
-6.2034183	12.6880007	105.5899963
-6.2034183	12.7368002	104.7299957
-6.2034183	12.7856007	104.6699982
-6.2034183	12.8344002	104.7399979
-6.2034183	12.8832006	102.8499985
-6.2034183	12.9320002	101.9599991
-6.2034183	12.9808006	101.0599976
-6.2034183	13.0296001	101.9000015
-6.2034183	13.0784006	103.5000000
-6.2034183	13.1272001	106.0100021
-6.2034183	13.1760006	104.8499985
-6.2034183	13.2248001	101.3099976
-6.2034183	13.2736006	103.6500015
-6.2034183	13.3224001	102.9000015
-6.2034183	13.3712006	99.8600006
-6.2034183	13.4200001	100.3799973
-6.2034183	13.4688005	103.2799988
-6.2034183	13.5176001	106.8600006
-6.2034183	13.5664005	105.5400009
-6.2034183	13.6152000	99.1399994
-6.2034183	13.6640005	100.5599976
-6.2034183	13.7128000	98.1800003
-6.2034183	13.7616005	102.6599960
-6.2034183	13.8104000	99.1399994
-6.2034183	13.8592005	101.9300003
-6.2034183	13.9080000	101.9399948
-6.2034183	13.9568005	101.3199997
-6.2034183	14.0056000	100.8099976
-6.2034183	14.0544004	99.9199982
-6.2034183	14.1032000	100.6999969
-6.2034183	14.1520004	101.8099976
-6.2034183	14.2007999	102.9799957
-6.2034183	14.2496004	105.3099976
-6.2034183	14.2983999	102.4899979
-6.2034183	14.3472004	100.6999969
-6.2034183	14.3959999	102.7299957
-6.2034183	14.4448004	104.6299973
-6.2034183	14.4935999	104.2999954
-6.2034183	14.5424004	102.0800018
-6.2034183	14.5911999	98.7299957
-6.2034183	14.6400003	100.4199982
-6.2034183	14.6887999	103.0400009
-6.2034183	14.7376003	106.8499985
-6.2034183	14.7863998	105.8399963
-6.2034183	14.8352003	103.5299988
-6.2034183	14.8839998	103.1800003
-6.2034183	14.9328003	98.6899948
-6.2034183	14.9815998	101.9899979
-6.2034183	15.0304003	104.3600006
-6.1972828	0.3416000	104.3899994
-6.1972828	0.3904000	107.5499954
-6.1972828	0.4392000	107.2200012
-6.1972828	0.4880000	103.8399963
-6.1972828	0.5368000	105.2299957
-6.1972828	0.5856000	104.9899979

-6.1972828	0.6344000	105.5299988
-6.1972828	0.6832000	106.0800018
-6.1972828	0.7320000	106.6199951
-6.1972828	0.7808000	108.9899979
-6.1972828	0.8296000	109.3300018
-6.1972828	0.8784000	110.1100006
-6.1972828	0.9272000	109.8199997
-6.1972828	0.9760000	110.4000015
-6.1972828	1.0247999	110.3300018
-6.1972828	1.0735999	108.3399963
-6.1972828	1.1223999	108.4799957
-6.1972828	1.1711999	108.6399994
-6.1972828	1.2200000	108.3099976
-6.1972828	1.2688000	106.4899979
-6.1972828	1.3176000	107.2099991
-6.1972828	1.3664000	106.3899994
-6.1972828	1.4152000	108.3799973
-6.1972828	1.4640000	109.7999954
-6.1972828	1.5128000	110.2200012
-6.1972828	1.5616000	108.5599976
-6.1972828	1.6104000	110.1899948
-6.1972828	1.6592000	108.5400009
-6.1972828	1.7079999	111.0699997
-6.1972828	1.7567999	111.4000015
-6.1972828	1.8055999	112.3300018
-6.1972828	1.8543999	119.4000015
-6.1972828	1.9031999	118.9099960
-6.1972828	1.9519999	113.4199982
-6.1972828	2.0007999	110.2500000
-6.1972828	2.0495999	111.1999969
-6.1972828	2.0983999	109.4700012
-6.1972828	2.1471999	106.4399948
-6.1972828	2.1959999	107.7099991
-6.1972828	2.2447999	107.3499985
-6.1972828	2.2935998	101.8099976
-6.1972828	2.3423998	105.2500000
-6.1972828	2.3912001	105.7200012
-6.1972828	2.4400001	107.3899994
-6.1972828	2.4888000	105.5899963
-6.1972828	2.5376000	106.9000015
-6.1972828	2.5864000	106.0299988
-6.1972828	2.6352000	105.9799957
-6.1972828	2.6840000	105.0299988
-6.1972828	2.7328000	102.1999969
-6.1972828	2.7816000	105.6899948
-6.1972828	2.8304000	105.9000015
-6.1972828	2.8792000	105.3399963
-6.1972828	2.9280000	105.9300003
-6.1972828	2.9768000	108.8499985
-6.1972828	3.0256000	108.3799973
-6.1972828	3.0743999	106.5299988
-6.1972828	3.1231999	103.5400009
-6.1972828	3.1719999	102.2999954
-6.1972828	3.2207999	98.0999985
-6.1972828	3.2695999	102.0800018
-6.1972828	3.3183999	105.6199951
-6.1972828	3.3671999	107.0100021
-6.1972828	3.4159999	105.3699951

-6.1972828	3.4647999	105.0599976
-6.1972828	3.5135999	104.8199997
-6.1972828	3.5623999	105.9499969
-6.1972828	3.6111999	105.2600021
-6.1972828	3.6599998	104.5299988
-6.1972828	3.7087998	104.4300003
-6.1972828	3.7575998	101.1800003
-6.1972828	3.8063998	102.4000015
-6.1972828	3.8551998	103.5199966
-6.1972828	3.9039998	103.9399948
-6.1972828	3.9527998	102.6100006
-6.1972828	4.0016003	103.4300003
-6.1972828	4.0503998	104.5400009
-6.1972828	4.0991998	101.8300018
-6.1972828	4.1479998	102.6100006
-6.1972828	4.1967998	102.4199982
-6.1972828	4.2455997	102.1899948
-6.1972828	4.2943997	108.8499985
-6.1972828	4.3431997	112.5199966
-6.1972828	4.3919997	110.9700012
-6.1972828	4.4407997	109.4599991
-6.1972828	4.4895997	111.8499985
-6.1972828	4.5383997	114.9599991
-6.1972828	4.5871997	118.9499969
-6.1972828	4.6359997	116.2299957
-6.1972828	4.6847997	115.5499954
-6.1972828	4.7336001	110.6100006
-6.1972828	4.7824001	108.2799988
-6.1972828	4.8312001	111.0100021
-6.1972828	4.8800001	108.0199966
-6.1972828	4.9288001	108.2799988
-6.1972828	4.9776001	110.0400009
-6.1972828	5.0264001	110.9399948
-6.1972828	5.0752001	112.6899948
-6.1972828	5.1240001	110.9499969
-6.1972828	5.1728001	108.4099960
-6.1972828	5.2216001	107.2799988
-6.1972828	5.2704000	107.9899979
-6.1972828	5.3192000	108.4000015
-6.1972828	5.3680000	108.9199982
-6.1972828	5.4168000	106.3699951
-6.1972828	5.4656000	103.9300003
-6.1972828	5.5144000	105.8099976
-6.1972828	5.5632000	106.1100006
-6.1972828	5.6120000	108.0899963
-6.1972828	5.6608000	107.0400009
-6.1972828	5.7096000	106.3699951
-6.1972828	5.7584000	106.1500015
-6.1972828	5.8072000	105.2799988
-6.1972828	5.8559999	103.2099991
-6.1972828	5.9047999	100.5899963
-6.1972828	5.9535999	100.8799973
-6.1972828	6.0023999	103.4300003
-6.1972828	6.0511999	106.5000000
-6.1972828	6.0999999	103.4799957
-6.1972828	6.1487999	103.1199951
-6.1972828	6.1975999	107.3399963
-6.1972828	6.2463999	108.4099960

-6.1972828	6.2951999	104.0400009
-6.1972828	6.3439999	98.7799988
-6.1972828	6.3927999	105.7900009
-6.1972828	6.4415998	108.1199951
-6.1972828	6.4903998	109.3899994
-6.1972828	6.5391998	108.3699951
-6.1972828	6.5879998	106.3899994
-6.1972828	6.6367998	105.2099991
-6.1972828	6.6855998	103.2299957
-6.1972828	6.7343998	102.9700012
-6.1972828	6.7831998	103.6399994
-6.1972828	6.8319998	103.9799957
-6.1972828	6.8807998	101.3300018
-6.1972828	6.9295998	101.2600021
-6.1972828	6.9783998	101.1999969
-6.1972828	7.0271997	101.8699951
-6.1972828	7.0759997	103.5000000
-6.1972828	7.1247997	105.6899948
-6.1972828	7.1735997	103.7999954
-6.1972828	7.2223997	103.1399994
-6.1972828	7.2711997	104.8099976
-6.1972828	7.3199997	101.5400009
-6.1972828	7.3687997	98.7600021
-6.1972828	7.4175997	103.4799957
-6.1972828	7.4663997	101.3399963
-6.1972828	7.5151997	102.9300003
-6.1972828	7.5639997	108.5199966
-6.1972828	7.6127996	107.5999985
-6.1972828	7.6615996	104.7900009
-6.1972828	7.7103996	103.1199951
-6.1972828	7.7591996	102.2200012
-6.1972828	7.8079996	101.1599960
-6.1972828	7.8567996	104.3099976
-6.1972828	7.9056001	102.7500000
-6.1972828	7.9544001	101.3499985
-6.1972828	8.0032005	100.1899948
-6.1972828	8.0520000	103.7900009
-6.1972828	8.1008005	105.8099976
-6.1972828	8.1496000	106.5000000
-6.1972828	8.1984005	105.7200012
-6.1972828	8.2472000	106.1199951
-6.1972828	8.2960005	103.1500015
-6.1972828	8.3448000	101.9599991
-6.1972828	8.3936005	102.1599960
-6.1972828	8.4424000	103.0299988
-6.1972828	8.4912004	104.2999954
-6.1972828	8.5400000	102.6500015
-6.1972828	8.5888004	100.2299957
-6.1972828	8.6375999	103.4899979
-6.1972828	8.6864004	103.5400009
-6.1972828	8.7351999	99.2099991
-6.1972828	8.7840004	100.6699982
-6.1972828	8.8327999	102.1299973
-6.1972828	8.8816004	100.8499985
-6.1972828	8.9303999	102.9799957
-6.1972828	8.9792004	103.8699951
-6.1972828	9.0279999	102.8499985
-6.1972828	9.0768003	105.3799973

-6.1972828	9.1255999	105.0100021
-6.1972828	9.1744003	104.5699997
-6.1972828	9.2231998	104.7600021
-6.1972828	9.2720003	102.3699951
-6.1972828	9.3207998	104.0100021
-6.1972828	9.3696003	104.3399963
-6.1972828	9.4184008	101.3099976
-6.1972828	9.4672003	100.4300003
-6.1972828	9.5160007	99.6699982
-6.1972828	9.5648003	100.8699951
-6.1972828	9.6136007	102.0100021
-6.1972828	9.6624002	105.4899979
-6.1972828	9.7112007	103.9899979
-6.1972828	9.7600002	101.1100006
-6.1972828	9.8088007	101.7099991
-6.1972828	9.8576002	103.4899979
-6.1972828	9.9064007	103.6399994
-6.1972828	9.9552002	103.3699951
-6.1972828	10.0040007	101.2500000
-6.1972828	10.0528002	97.2200012
-6.1972828	10.1016006	99.8099976
-6.1972828	10.1504002	101.0699997
-6.1972828	10.1992006	103.4099960
-6.1972828	10.2480001	100.7900009
-6.1972828	10.2968006	103.2299957
-6.1972828	10.3456001	104.3099976
-6.1972828	10.3944006	104.2099991
-6.1972828	10.4432001	103.4499969
-6.1972828	10.4920006	103.7500000
-6.1972828	10.5408001	104.2600021
-6.1972828	10.5896006	105.5400009
-6.1972828	10.6384001	104.2500000
-6.1972828	10.6872005	103.4099960
-6.1972828	10.7360001	103.4899979
-6.1972828	10.7848005	102.3399963
-6.1972828	10.8336000	101.6800003
-6.1972828	10.8824005	100.8600006
-6.1972828	10.9312000	102.5299988
-6.1972828	10.9800005	101.8899994
-6.1972828	11.0288000	102.9700012
-6.1972828	11.0776005	103.0599976
-6.1972828	11.1264000	104.0599976
-6.1972828	11.1752005	103.7399979
-6.1972828	11.2240000	103.7099991
-6.1972828	11.2728004	104.5999985
-6.1972828	11.3216000	101.9599991
-6.1972828	11.3704004	106.5499954
-6.1972828	11.4191999	106.2600021
-6.1972828	11.4680004	104.9000015
-6.1972828	11.5167999	104.4700012
-6.1972828	11.5656004	103.7900009
-6.1972828	11.6143999	103.5999985
-6.1972828	11.6632004	106.6800003
-6.1972828	11.7119999	107.4499969
-6.1972828	11.7608004	106.1599960
-6.1972828	11.8095999	106.0299988
-6.1972828	11.8584003	104.3399963
-6.1972828	11.9071999	103.9099960

-6.1972828	11.9560003	104.8099976
-6.1972828	12.0047998	106.2999954
-6.1972828	12.0536003	105.8499985
-6.1972828	12.1023998	107.1699982
-6.1972828	12.1512003	106.2200012
-6.1972828	12.1999998	104.4399948
-6.1972828	12.2488003	102.7699966
-6.1972828	12.2975998	99.7999954
-6.1972828	12.3464003	103.0000000
-6.1972828	12.3951998	105.8699951
-6.1972828	12.4440002	105.4899979
-6.1972828	12.4927998	104.6199951
-6.1972828	12.5416002	105.1899948
-6.1972828	12.5904007	103.2799988
-6.1972828	12.6392002	105.2600021
-6.1972828	12.6880007	104.8399963
-6.1972828	12.7368002	104.3600006
-6.1972828	12.7856007	105.1699982
-6.1972828	12.8344002	105.2500000
-6.1972828	12.8832006	102.7900009
-6.1972828	12.9320002	103.2600021
-6.1972828	12.9808006	102.4300003
-6.1972828	13.0296001	102.6399994
-6.1972828	13.0784006	103.1100006
-6.1972828	13.1272001	106.6599960
-6.1972828	13.1760006	105.8600006
-6.1972828	13.2248001	100.8499985
-6.1972828	13.2736006	103.4599991
-6.1972828	13.3224001	103.0000000
-6.1972828	13.3712006	99.2999954
-6.1972828	13.4200001	101.4199982
-6.1972828	13.4688005	104.6500015
-6.1972828	13.5176001	107.3499985
-6.1972828	13.5664005	106.2600021
-6.1972828	13.6152000	98.7799988
-6.1972828	13.6640005	99.9000015
-6.1972828	13.7128000	97.8300018
-6.1972828	13.7616005	102.1100006
-6.1972828	13.8104000	99.7299957
-6.1972828	13.8592005	101.7699966
-6.1972828	13.9080000	102.0999985
-6.1972828	13.9568005	101.7600021
-6.1972828	14.0056000	101.1399994
-6.1972828	14.0544004	99.6599960
-6.1972828	14.1032000	100.5000000
-6.1972828	14.1520004	102.0899963
-6.1972828	14.2007999	103.0499954
-6.1972828	14.2496004	104.9599991
-6.1972828	14.2983999	102.3199997
-6.1972828	14.3472004	101.3199997
-6.1972828	14.3959999	102.8499985
-6.1972828	14.4448004	104.1899948
-6.1972828	14.4935999	103.9399948
-6.1972828	14.5424004	101.7900009
-6.1972828	14.5911999	98.1999969
-6.1972828	14.6400003	100.7200012
-6.1972828	14.6887999	103.4199982
-6.1972828	14.7376003	106.1899948

-6.1972828	14.7863998	105.0699997
-6.1972828	14.8352003	103.4099960
-6.1972828	14.8839998	102.1599960
-6.1972828	14.9328003	97.3499985
-6.1972828	14.9815998	101.9000015
-6.1972828	15.0304003	104.1399994
-6.1911469	0.3416000	105.0599976
-6.1911469	0.3904000	108.0999985
-6.1911469	0.4392000	107.9399948
-6.1911469	0.4880000	105.0999985
-6.1911469	0.5368000	106.1100006
-6.1911469	0.5856000	104.8099976
-6.1911469	0.6344000	106.0000000
-6.1911469	0.6832000	106.7500000
-6.1911469	0.7320000	105.2600021
-6.1911469	0.7808000	108.4799957
-6.1911469	0.8296000	109.5199966
-6.1911469	0.8784000	110.2200012
-6.1911469	0.9272000	109.3899994
-6.1911469	0.9760000	110.2399979
-6.1911469	1.0247999	110.3799973
-6.1911469	1.0735999	108.9599991
-6.1911469	1.1223999	108.7900009
-6.1911469	1.1711999	108.6899948
-6.1911469	1.2200000	108.1699982
-6.1911469	1.2688000	107.1899948
-6.1911469	1.3176000	107.2900009
-6.1911469	1.3664000	106.1699982
-6.1911469	1.4152000	108.4899979
-6.1911469	1.4640000	109.7999954
-6.1911469	1.5128000	110.1699982
-6.1911469	1.5616000	108.7699966
-6.1911469	1.6104000	110.9000015
-6.1911469	1.6592000	108.8600006
-6.1911469	1.7079999	111.0899963
-6.1911469	1.7567999	110.6599960
-6.1911469	1.8055999	111.9700012
-6.1911469	1.8543999	118.8099976
-6.1911469	1.9031999	118.4000015
-6.1911469	1.9519999	113.6699982
-6.1911469	2.0007999	109.5699997
-6.1911469	2.0495999	110.5100021
-6.1911469	2.0983999	109.0699997
-6.1911469	2.1471999	106.3699951
-6.1911469	2.1959999	106.9000015
-6.1911469	2.2447999	106.5800018
-6.1911469	2.2935998	101.6199951
-6.1911469	2.3423998	106.4799957
-6.1911469	2.3912001	106.6399994
-6.1911469	2.4400001	107.3600006
-6.1911469	2.4888000	105.1299973
-6.1911469	2.5376000	106.0999985
-6.1911469	2.5864000	105.4599991
-6.1911469	2.6352000	105.3899994
-6.1911469	2.6840000	105.1299973
-6.1911469	2.7328000	103.4799957
-6.1911469	2.7816000	106.4099960
-6.1911469	2.8304000	106.2799988

-6.1911469	2.8792000	105.5599976
-6.1911469	2.9280000	105.6299973
-6.1911469	2.9768000	109.1199951
-6.1911469	3.0256000	108.5299988
-6.1911469	3.0743999	106.2099991
-6.1911469	3.1231999	103.4700012
-6.1911469	3.1719999	103.4199982
-6.1911469	3.2207999	97.7299957
-6.1911469	3.2695999	101.6199951
-6.1911469	3.3183999	105.7799988
-6.1911469	3.3671999	107.3499985
-6.1911469	3.4159999	106.0599976
-6.1911469	3.4647999	105.1800003
-6.1911469	3.5135999	104.1800003
-6.1911469	3.5623999	105.1500015
-6.1911469	3.6111999	105.1899948
-6.1911469	3.6599998	104.3399963
-6.1911469	3.7087998	105.3300018
-6.1911469	3.7575998	102.1599960
-6.1911469	3.8063998	103.0100021
-6.1911469	3.8551998	104.8600006
-6.1911469	3.9039998	104.6800003
-6.1911469	3.9527998	103.0000000
-6.1911469	4.0016003	105.3600006
-6.1911469	4.0503998	105.9199982
-6.1911469	4.0991998	103.8399963
-6.1911469	4.1479998	103.0400009
-6.1911469	4.1967998	101.6699982
-6.1911469	4.2455997	102.8199997
-6.1911469	4.2943997	109.3399963
-6.1911469	4.3431997	112.6399994
-6.1911469	4.3919997	110.4399948
-6.1911469	4.4407997	109.8600006
-6.1911469	4.4895997	112.4899979
-6.1911469	4.5383997	115.5000000
-6.1911469	4.5871997	119.2900009
-6.1911469	4.6359997	117.3799973
-6.1911469	4.6847997	115.7500000
-6.1911469	4.7336001	111.5699997
-6.1911469	4.7824001	108.2799988
-6.1911469	4.8312001	111.9399948
-6.1911469	4.8800001	110.1500015
-6.1911469	4.9288001	109.9199982
-6.1911469	4.9776001	109.7999954
-6.1911469	5.0264001	109.6899948
-6.1911469	5.0752001	111.5999985
-6.1911469	5.1240001	110.6500015
-6.1911469	5.1728001	108.6399994
-6.1911469	5.2216001	107.9300003
-6.1911469	5.2704000	107.6699982
-6.1911469	5.3192000	107.5800018
-6.1911469	5.3680000	108.2600021
-6.1911469	5.4168000	105.9099960
-6.1911469	5.4656000	104.5400009
-6.1911469	5.5144000	105.6100006
-6.1911469	5.5632000	105.8399963
-6.1911469	5.6120000	108.1800003
-6.1911469	5.6608000	106.4300003

-6.1911469	5.7096000	105.7999954
-6.1911469	5.7584000	105.5299988
-6.1911469	5.8072000	104.2299957
-6.1911469	5.8559999	102.2399979
-6.1911469	5.9047999	100.4599991
-6.1911469	5.9535999	100.3899994
-6.1911469	6.0023999	101.5699997
-6.1911469	6.0511999	105.7099991
-6.1911469	6.0999999	103.8499985
-6.1911469	6.1487999	103.9000015
-6.1911469	6.1975999	107.0100021
-6.1911469	6.2463999	108.4300003
-6.1911469	6.2951999	104.5400009
-6.1911469	6.3439999	100.1999969
-6.1911469	6.3927999	106.5000000
-6.1911469	6.4415998	108.5899963
-6.1911469	6.4903998	108.5499954
-6.1911469	6.5391998	107.5999985
-6.1911469	6.5879998	106.2799988
-6.1911469	6.6367998	104.8899994
-6.1911469	6.6855998	103.8099976
-6.1911469	6.7343998	103.8499985
-6.1911469	6.7831998	104.4099960
-6.1911469	6.8319998	104.3799973
-6.1911469	6.8807998	102.6800003
-6.1911469	6.9295998	102.3499985
-6.1911469	6.9783998	101.2699966
-6.1911469	7.0271997	100.5199966
-6.1911469	7.0759997	102.2900009
-6.1911469	7.1247997	105.0499954
-6.1911469	7.1735997	103.6299973
-6.1911469	7.2223997	102.7399979
-6.1911469	7.2711997	103.5800018
-6.1911469	7.3199997	101.1399994
-6.1911469	7.3687997	99.1999969
-6.1911469	7.4175997	103.3899994
-6.1911469	7.4663997	101.9599991
-6.1911469	7.5151997	102.0100021
-6.1911469	7.5639997	107.7999954
-6.1911469	7.6127996	107.0800018
-6.1911469	7.6615996	104.2699966
-6.1911469	7.7103996	101.9899979
-6.1911469	7.7591996	101.3799973
-6.1911469	7.8079996	100.0599976
-6.1911469	7.8567996	103.6599960
-6.1911469	7.9056001	102.8899994
-6.1911469	7.9544001	101.2999954
-6.1911469	8.0032005	100.5299988
-6.1911469	8.0520000	103.7399979
-6.1911469	8.1008005	105.1500015
-6.1911469	8.1496000	105.6599960
-6.1911469	8.1984005	104.9099960
-6.1911469	8.2472000	106.3399963
-6.1911469	8.2960005	103.4899979
-6.1911469	8.3448000	102.6699982
-6.1911469	8.3936005	102.5999985
-6.1911469	8.4424000	103.2999954
-6.1911469	8.4912004	104.8300018

-6.1911469	8.5400000	102.4799957
-6.1911469	8.5888004	100.7399979
-6.1911469	8.6375999	103.9099960
-6.1911469	8.6864004	103.8399963
-6.1911469	8.7351999	99.4899979
-6.1911469	8.7840004	101.1899948
-6.1911469	8.8327999	101.8199997
-6.1911469	8.8816004	100.6399994
-6.1911469	8.9303999	103.7099991
-6.1911469	8.9792004	104.5899963
-6.1911469	9.0279999	103.3199997
-6.1911469	9.0768003	105.7600021
-6.1911469	9.1255999	106.0599976
-6.1911469	9.1744003	104.9700012
-6.1911469	9.2231998	105.2699966
-6.1911469	9.2720003	102.8300018
-6.1911469	9.3207998	103.8799973
-6.1911469	9.3696003	104.5400009
-6.1911469	9.4184008	101.0299988
-6.1911469	9.4672003	100.6800003
-6.1911469	9.5160007	100.7799988
-6.1911469	9.5648003	101.3399963
-6.1911469	9.6136007	101.1500015
-6.1911469	9.6624002	105.2099991
-6.1911469	9.7112007	103.8099976
-6.1911469	9.7600002	100.2600021
-6.1911469	9.8088007	101.5299988
-6.1911469	9.8576002	103.0499954
-6.1911469	9.9064007	103.2699966
-6.1911469	9.9552002	103.0499954
-6.1911469	10.0040007	100.5599976
-6.1911469	10.0528002	96.9399948
-6.1911469	10.1016006	99.5299988
-6.1911469	10.1504002	100.0999985
-6.1911469	10.1992006	103.1899948
-6.1911469	10.2480001	101.3499985
-6.1911469	10.2968006	103.5299988
-6.1911469	10.3456001	104.5899963
-6.1911469	10.3944006	104.7600021
-6.1911469	10.4432001	103.2799988
-6.1911469	10.4920006	103.4099960
-6.1911469	10.5408001	105.0100021
-6.1911469	10.5896006	106.1899948
-6.1911469	10.6384001	104.0899963
-6.1911469	10.6872005	102.4099960
-6.1911469	10.7360001	102.5800018
-6.1911469	10.7848005	102.3699951
-6.1911469	10.8336000	101.8399963
-6.1911469	10.8824005	101.2999954
-6.1911469	10.9312000	103.0800018
-6.1911469	10.9800005	102.0100021
-6.1911469	11.0288000	102.8399963
-6.1911469	11.0776005	102.1800003
-6.1911469	11.1264000	102.5800018
-6.1911469	11.1752005	103.1399994
-6.1911469	11.2240000	104.5599976
-6.1911469	11.2728004	105.0400009
-6.1911469	11.3216000	102.1399994

-6.1911469	11.3704004	106.6199951
-6.1911469	11.4191999	106.3600006
-6.1911469	11.4680004	105.6599960
-6.1911469	11.5167999	105.3099976
-6.1911469	11.5656004	104.8799973
-6.1911469	11.6143999	103.6399994
-6.1911469	11.6632004	105.7900009
-6.1911469	11.7119999	107.1599960
-6.1911469	11.7608004	106.3799973
-6.1911469	11.8095999	105.9099960
-6.1911469	11.8584003	103.8799973
-6.1911469	11.9071999	102.8499985
-6.1911469	11.9560003	104.5299988
-6.1911469	12.0047998	106.1399994
-6.1911469	12.0536003	105.9499969
-6.1911469	12.1023998	107.1999969
-6.1911469	12.1512003	105.6199951
-6.1911469	12.1999998	103.7999954
-6.1911469	12.2488003	101.3799973
-6.1911469	12.2975998	99.4899979
-6.1911469	12.3464003	102.3199997
-6.1911469	12.3951998	104.2699966
-6.1911469	12.4440002	104.5400009
-6.1911469	12.4927998	104.8499985
-6.1911469	12.5416002	105.6800003
-6.1911469	12.5904007	103.6199951
-6.1911469	12.6392002	105.1199951
-6.1911469	12.6880007	103.9499969
-6.1911469	12.7368002	103.9199982
-6.1911469	12.7856007	105.4799957
-6.1911469	12.8344002	105.5699997
-6.1911469	12.8832006	102.3899994
-6.1911469	12.9320002	103.5100021
-6.1911469	12.9808006	103.6199951
-6.1911469	13.0296001	103.8799973
-6.1911469	13.0784006	103.8399963
-6.1911469	13.1272001	106.9199982
-6.1911469	13.1760006	106.0100021
-6.1911469	13.2248001	100.6399994
-6.1911469	13.2736006	103.2200012
-6.1911469	13.3224001	103.4300003
-6.1911469	13.3712006	99.8300018
-6.1911469	13.4200001	102.6699982
-6.1911469	13.4688005	105.4700012
-6.1911469	13.5176001	106.7900009
-6.1911469	13.5664005	106.4799957
-6.1911469	13.6152000	99.8899994
-6.1911469	13.6640005	100.1999969
-6.1911469	13.7128000	98.0699997
-6.1911469	13.7616005	101.9000015
-6.1911469	13.8104000	100.4799957
-6.1911469	13.8592005	101.5100021
-6.1911469	13.9080000	101.6800003
-6.1911469	13.9568005	102.1699982
-6.1911469	14.0056000	101.3099976
-6.1911469	14.0544004	99.7200012
-6.1911469	14.1032000	101.1999969
-6.1911469	14.1520004	102.5599976

-6.1911469	14.2007999	102.9599991
-6.1911469	14.2496004	104.3199997
-6.1911469	14.2983999	102.2799988
-6.1911469	14.3472004	102.0400009
-6.1911469	14.3959999	102.7299957
-6.1911469	14.4448004	103.4399948
-6.1911469	14.4935999	103.1800003
-6.1911469	14.5424004	100.5699997
-6.1911469	14.5911999	97.8799973
-6.1911469	14.6400003	100.7299957
-6.1911469	14.6887999	103.2500000
-6.1911469	14.7376003	104.5999985
-6.1911469	14.7863998	104.0299988
-6.1911469	14.8352003	103.4000015
-6.1911469	14.8839998	101.8399963
-6.1911469	14.9328003	97.3799973
-6.1911469	14.9815998	102.3699951
-6.1911469	15.0304003	104.0999985
-6.1850109	0.3416000	106.1999969
-6.1850109	0.3904000	109.1699982
-6.1850109	0.4392000	108.9599991
-6.1850109	0.4880000	106.6800003
-6.1850109	0.5368000	107.1599960
-6.1850109	0.5856000	105.0999985
-6.1850109	0.6344000	106.4300003
-6.1850109	0.6832000	107.2799988
-6.1850109	0.7320000	103.5299988
-6.1850109	0.7808000	107.4199982
-6.1850109	0.8296000	109.1999969
-6.1850109	0.8784000	109.9499969
-6.1850109	0.9272000	108.8699951
-6.1850109	0.9760000	110.0199966
-6.1850109	1.0247999	109.9599991
-6.1850109	1.0735999	108.7500000
-6.1850109	1.1223999	108.9099960
-6.1850109	1.1711999	108.5199966
-6.1850109	1.2200000	107.3300018
-6.1850109	1.2688000	107.4499969
-6.1850109	1.3176000	107.3600006
-6.1850109	1.3664000	106.4199982
-6.1850109	1.4152000	108.7500000
-6.1850109	1.4640000	109.6899948
-6.1850109	1.5128000	110.5699997
-6.1850109	1.5616000	109.5199966
-6.1850109	1.6104000	111.7200012
-6.1850109	1.6592000	109.8600006
-6.1850109	1.7079999	111.5499954
-6.1850109	1.7567999	110.8199997
-6.1850109	1.8055999	111.7999954
-6.1850109	1.8543999	118.4300003
-6.1850109	1.9031999	118.3300018
-6.1850109	1.9519999	113.6199951
-6.1850109	2.0007999	108.7999954
-6.1850109	2.0495999	109.8300018
-6.1850109	2.0983999	108.4199982
-6.1850109	2.1471999	106.0100021
-6.1850109	2.1959999	105.7500000
-6.1850109	2.2447999	105.7699966

-6.1850109	2.2935998	103.3199997
-6.1850109	2.3423998	108.0000000
-6.1850109	2.3912001	107.1999969
-6.1850109	2.4400001	106.9899979
-6.1850109	2.4888000	105.2099991
-6.1850109	2.5376000	105.9499969
-6.1850109	2.5864000	105.7200012
-6.1850109	2.6352000	105.3799973
-6.1850109	2.6840000	105.5599976
-6.1850109	2.7328000	104.9499969
-6.1850109	2.7816000	107.0100021
-6.1850109	2.8304000	106.8099976
-6.1850109	2.8792000	106.0000000
-6.1850109	2.9280000	105.1699982
-6.1850109	2.9768000	109.0499954
-6.1850109	3.0256000	108.8899994
-6.1850109	3.0743999	105.8699951
-6.1850109	3.1231999	103.2299957
-6.1850109	3.1719999	104.9000015
-6.1850109	3.2207999	99.6899948
-6.1850109	3.2695999	101.9599991
-6.1850109	3.3183999	106.4300003
-6.1850109	3.3671999	107.8199997
-6.1850109	3.4159999	106.0000000
-6.1850109	3.4647999	105.4899979
-6.1850109	3.5135999	103.9199982
-6.1850109	3.5623999	104.8799973
-6.1850109	3.6111999	105.3300018
-6.1850109	3.6599998	103.6199951
-6.1850109	3.7087998	105.5499954
-6.1850109	3.7575998	102.8799973
-6.1850109	3.8063998	103.4099960
-6.1850109	3.8551998	105.8099976
-6.1850109	3.9039998	105.4300003
-6.1850109	3.9527998	102.0800018
-6.1850109	4.0016003	106.4000015
-6.1850109	4.0503998	106.6800003
-6.1850109	4.0991998	105.7099991
-6.1850109	4.1479998	103.8099976
-6.1850109	4.1967998	101.4700012
-6.1850109	4.2455997	103.3799973
-6.1850109	4.2943997	109.7799988
-6.1850109	4.3431997	112.6899948
-6.1850109	4.3919997	109.9300003
-6.1850109	4.4407997	110.5999985
-6.1850109	4.4895997	113.0800018
-6.1850109	4.5383997	116.1100006
-6.1850109	4.5871997	119.2299957
-6.1850109	4.6359997	117.6800003
-6.1850109	4.6847997	115.7299957
-6.1850109	4.7336001	112.5199966
-6.1850109	4.7824001	108.0499954
-6.1850109	4.8312001	112.5400009
-6.1850109	4.8800001	112.0699997
-6.1850109	4.9288001	111.3699951
-6.1850109	4.9776001	109.5100021
-6.1850109	5.0264001	108.5899963
-6.1850109	5.0752001	110.3300018

-6.1850109	5.1240001	109.3699951
-6.1850109	5.1728001	108.0899963
-6.1850109	5.2216001	107.9899979
-6.1850109	5.2704000	107.3399963
-6.1850109	5.3192000	106.6299973
-6.1850109	5.3680000	107.2099991
-6.1850109	5.4168000	105.7699966
-6.1850109	5.4656000	105.5800018
-6.1850109	5.5144000	105.6599960
-6.1850109	5.5632000	105.6599960
-6.1850109	5.6120000	108.1299973
-6.1850109	5.6608000	105.3600006
-6.1850109	5.7096000	105.2299957
-6.1850109	5.7584000	105.2999954
-6.1850109	5.8072000	103.1299973
-6.1850109	5.8559999	101.6100006
-6.1850109	5.9047999	100.4799957
-6.1850109	5.9535999	100.2200012
-6.1850109	6.0023999	99.7200012
-6.1850109	6.0511999	104.7699966
-6.1850109	6.0999999	104.0800018
-6.1850109	6.1487999	104.6800003
-6.1850109	6.1975999	106.5100021
-6.1850109	6.2463999	107.6599960
-6.1850109	6.2951999	103.7500000
-6.1850109	6.3439999	101.8499985
-6.1850109	6.3927999	107.3099976
-6.1850109	6.4415998	109.3699951
-6.1850109	6.4903998	108.1699982
-6.1850109	6.5391998	107.1699982
-6.1850109	6.5879998	105.9599991
-6.1850109	6.6367998	104.7099991
-6.1850109	6.6855998	104.0800018
-6.1850109	6.7343998	104.7699966
-6.1850109	6.7831998	105.5800018
-6.1850109	6.8319998	103.8799973
-6.1850109	6.8807998	103.6100006
-6.1850109	6.9295998	103.8699951
-6.1850109	6.9783998	102.2699966
-6.1850109	7.0271997	101.0499954
-6.1850109	7.0759997	101.6699982
-6.1850109	7.1247997	104.5400009
-6.1850109	7.1735997	103.5899963
-6.1850109	7.2223997	102.8399963
-6.1850109	7.2711997	103.0100021
-6.1850109	7.3199997	101.3699951
-6.1850109	7.3687997	99.4899979
-6.1850109	7.4175997	103.3699951
-6.1850109	7.4663997	102.3099976
-6.1850109	7.5151997	100.4300003
-6.1850109	7.5639997	106.3899994
-6.1850109	7.6127996	105.9599991
-6.1850109	7.6615996	104.4899979
-6.1850109	7.7103996	101.4000015
-6.1850109	7.7591996	100.8799973
-6.1850109	7.8079996	99.8300018
-6.1850109	7.8567996	102.9399948
-6.1850109	7.9056001	102.6599960

-6.1850109	7.9544001	101.2500000
-6.1850109	8.0032005	101.2799988
-6.1850109	8.0520000	103.8600006
-6.1850109	8.1008005	104.5400009
-6.1850109	8.1496000	104.9199982
-6.1850109	8.1984005	104.6199951
-6.1850109	8.2472000	106.7399979
-6.1850109	8.2960005	103.7399979
-6.1850109	8.3448000	103.4799957
-6.1850109	8.3936005	102.8699951
-6.1850109	8.4424000	102.7200012
-6.1850109	8.4912004	104.9199982
-6.1850109	8.5400000	102.3499985
-6.1850109	8.5888004	100.6500015
-6.1850109	8.6375999	104.3300018
-6.1850109	8.6864004	104.4599991
-6.1850109	8.7351999	100.3699951
-6.1850109	8.7840004	101.4000015
-6.1850109	8.8327999	100.9099960
-6.1850109	8.8816004	100.5199966
-6.1850109	8.9303999	104.3300018
-6.1850109	8.9792004	104.9499969
-6.1850109	9.0279999	103.0100021
-6.1850109	9.0768003	105.1199951
-6.1850109	9.1255999	106.0800018
-6.1850109	9.1744003	105.4199982
-6.1850109	9.2231998	105.7500000
-6.1850109	9.2720003	103.4300003
-6.1850109	9.3207998	104.0199966
-6.1850109	9.3696003	104.6899948
-6.1850109	9.4184008	100.8199997
-6.1850109	9.4672003	101.2200012
-6.1850109	9.5160007	101.8600006
-6.1850109	9.5648003	101.2299957
-6.1850109	9.6136007	100.0800018
-6.1850109	9.6624002	104.4700012
-6.1850109	9.7112007	103.7999954
-6.1850109	9.7600002	100.2699966
-6.1850109	9.8088007	101.5800018
-6.1850109	9.8576002	102.4899979
-6.1850109	9.9064007	103.0599976
-6.1850109	9.9552002	102.9499969
-6.1850109	10.0040007	100.3099976
-6.1850109	10.0528002	97.5199966
-6.1850109	10.1016006	100.4799957
-6.1850109	10.1504002	99.9799957
-6.1850109	10.1992006	103.0699997
-6.1850109	10.2480001	102.1599960
-6.1850109	10.2968006	103.8699951
-6.1850109	10.3456001	104.7500000
-6.1850109	10.3944006	104.8499985
-6.1850109	10.4432001	102.9000015
-6.1850109	10.4920006	102.5899963
-6.1850109	10.5408001	105.2399979
-6.1850109	10.5896006	106.1599960
-6.1850109	10.6384001	103.7099991
-6.1850109	10.6872005	100.8699951
-6.1850109	10.7360001	102.0599976

-6.1850109	10.7848005	102.5699997
-6.1850109	10.8336000	102.0599976
-6.1850109	10.8824005	101.5899963
-6.1850109	10.9312000	103.6599960
-6.1850109	10.9800005	102.1999969
-6.1850109	11.0288000	103.1500015
-6.1850109	11.0776005	102.0800018
-6.1850109	11.1264000	101.2099991
-6.1850109	11.1752005	103.3099976
-6.1850109	11.2240000	105.3300018
-6.1850109	11.2728004	105.6800003
-6.1850109	11.3216000	103.5299988
-6.1850109	11.3704004	106.8300018
-6.1850109	11.4191999	106.5599976
-6.1850109	11.4680004	106.5299988
-6.1850109	11.5167999	106.2900009
-6.1850109	11.5656004	105.9599991
-6.1850109	11.6143999	104.1699982
-6.1850109	11.6632004	105.2600021
-6.1850109	11.7119999	106.9199982
-6.1850109	11.7608004	106.5499954
-6.1850109	11.8095999	106.1599960
-6.1850109	11.8584003	104.2500000
-6.1850109	11.9071999	102.4700012
-6.1850109	11.9560003	104.7500000
-6.1850109	12.0047998	105.9899979
-6.1850109	12.0536003	106.0499954
-6.1850109	12.1023998	106.6800003
-6.1850109	12.1512003	104.7500000
-6.1850109	12.1999998	102.7799988
-6.1850109	12.2488003	100.7399979
-6.1850109	12.2975998	100.1100006
-6.1850109	12.3464003	102.2799988
-6.1850109	12.3951998	104.2900009
-6.1850109	12.4440002	104.6999969
-6.1850109	12.4927998	105.5400009
-6.1850109	12.5416002	106.0899963
-6.1850109	12.5904007	103.5199966
-6.1850109	12.6392002	105.0599976
-6.1850109	12.6880007	104.1199951
-6.1850109	12.7368002	104.0199966
-6.1850109	12.7856007	106.1500015
-6.1850109	12.8344002	105.8499985
-6.1850109	12.8832006	101.7900009
-6.1850109	12.9320002	102.9899979
-6.1850109	12.9808006	104.5299988
-6.1850109	13.0296001	105.1199951
-6.1850109	13.0784006	103.9700012
-6.1850109	13.1272001	106.0000000
-6.1850109	13.1760006	105.1599960
-6.1850109	13.2248001	99.8499985
-6.1850109	13.2736006	102.6899948
-6.1850109	13.3224001	103.5999985
-6.1850109	13.3712006	100.4799957
-6.1850109	13.4200001	103.7699966
-6.1850109	13.4688005	105.8600006
-6.1850109	13.5176001	105.0599976
-6.1850109	13.5664005	106.3799973

-6.1850109	13.6152000	101.5299988
-6.1850109	13.6640005	100.7999954
-6.1850109	13.7128000	99.6699982
-6.1850109	13.7616005	102.2500000
-6.1850109	13.8104000	101.5999985
-6.1850109	13.8592005	101.8300018
-6.1850109	13.9080000	101.5000000
-6.1850109	13.9568005	102.6999969
-6.1850109	14.0056000	101.6599960
-6.1850109	14.0544004	100.1199951
-6.1850109	14.1032000	102.1399994
-6.1850109	14.1520004	103.5999985
-6.1850109	14.2007999	103.3699951
-6.1850109	14.2496004	103.9300003
-6.1850109	14.2983999	102.7600021
-6.1850109	14.3472004	103.2699966
-6.1850109	14.3959999	102.6800003
-6.1850109	14.4448004	102.2999954
-6.1850109	14.4935999	102.4300003
-6.1850109	14.5424004	99.8899994
-6.1850109	14.5911999	98.0800018
-6.1850109	14.6400003	100.8399963
-6.1850109	14.6887999	102.5599976
-6.1850109	14.7376003	102.9399948
-6.1850109	14.7863998	102.9000015
-6.1850109	14.8352003	102.9199982
-6.1850109	14.8839998	101.2399979
-6.1850109	14.9328003	98.6800003
-6.1850109	14.9815998	102.7799988
-6.1850109	15.0304003	104.3699951
-6.1788750	0.3416000	107.7500000
-6.1788750	0.3904000	109.5100021
-6.1788750	0.4392000	109.2399979
-6.1788750	0.4880000	107.6199951
-6.1788750	0.5368000	107.6999969
-6.1788750	0.5856000	105.1599960
-6.1788750	0.6344000	106.4099960
-6.1788750	0.6832000	107.5400009
-6.1788750	0.7320000	101.6699982
-6.1788750	0.7808000	106.7399979
-6.1788750	0.8296000	109.1800003
-6.1788750	0.8784000	109.9499969
-6.1788750	0.9272000	108.0800018
-6.1788750	0.9760000	109.2099991
-6.1788750	1.0247999	109.0999985
-6.1788750	1.0735999	108.0199966
-6.1788750	1.1223999	108.9899979
-6.1788750	1.1711999	108.0400009
-6.1788750	1.2200000	106.4000015
-6.1788750	1.2688000	107.7399979
-6.1788750	1.3176000	107.2500000
-6.1788750	1.3664000	106.6800003
-6.1788750	1.4152000	108.9799957
-6.1788750	1.4640000	110.0199966
-6.1788750	1.5128000	110.4599991
-6.1788750	1.5616000	109.6500015
-6.1788750	1.6104000	111.9899979
-6.1788750	1.6592000	110.2500000

-6.1788750	1.7079999	111.5599976
-6.1788750	1.7567999	110.5699997
-6.1788750	1.8055999	111.5299988
-6.1788750	1.8543999	118.2799988
-6.1788750	1.9031999	118.4799957
-6.1788750	1.9519999	113.5899963
-6.1788750	2.0007999	107.7399979
-6.1788750	2.0495999	108.7600021
-6.1788750	2.0983999	107.6599960
-6.1788750	2.1471999	105.5100021
-6.1788750	2.1959999	104.1399994
-6.1788750	2.2447999	105.0699997
-6.1788750	2.2935998	104.7699966
-6.1788750	2.3423998	108.1699982
-6.1788750	2.3912001	106.9399948
-6.1788750	2.4400001	105.9499969
-6.1788750	2.4888000	105.1399994
-6.1788750	2.5376000	106.1999969
-6.1788750	2.5864000	106.0899963
-6.1788750	2.6352000	105.4599991
-6.1788750	2.6840000	105.7699966
-6.1788750	2.7328000	105.7999954
-6.1788750	2.7816000	106.7900009
-6.1788750	2.8304000	107.0100021
-6.1788750	2.8792000	106.0599976
-6.1788750	2.9280000	104.8099976
-6.1788750	2.9768000	109.2200012
-6.1788750	3.0256000	109.0699997
-6.1788750	3.0743999	105.7200012
-6.1788750	3.1231999	102.8199997
-6.1788750	3.1719999	105.8499985
-6.1788750	3.2207999	101.5100021
-6.1788750	3.2695999	103.1100006
-6.1788750	3.3183999	107.2699966
-6.1788750	3.3671999	107.9899979
-6.1788750	3.4159999	104.7799988
-6.1788750	3.4647999	105.2900009
-6.1788750	3.5135999	103.7099991
-6.1788750	3.5623999	104.4599991
-6.1788750	3.6111999	105.0899963
-6.1788750	3.6599998	102.1399994
-6.1788750	3.7087998	104.7200012
-6.1788750	3.7575998	103.4599991
-6.1788750	3.8063998	103.7799988
-6.1788750	3.8551998	106.0499954
-6.1788750	3.9039998	105.2099991
-6.1788750	3.9527998	100.6100006
-6.1788750	4.0016003	106.4399948
-6.1788750	4.0503998	106.9599991
-6.1788750	4.0991998	106.6100006
-6.1788750	4.1479998	104.0999985
-6.1788750	4.1967998	100.8899994
-6.1788750	4.2455997	103.6699982
-6.1788750	4.2943997	110.1100006
-6.1788750	4.3431997	112.8300018
-6.1788750	4.3919997	109.2200012
-6.1788750	4.4407997	110.5199966
-6.1788750	4.4895997	113.3899994

-6.1788750	4.5383997	116.6299973
-6.1788750	4.5871997	119.1500015
-6.1788750	4.6359997	117.5800018
-6.1788750	4.6847997	116.4000015
-6.1788750	4.7336001	114.0800018
-6.1788750	4.7824001	108.9199982
-6.1788750	4.8312001	113.3099976
-6.1788750	4.8800001	113.5599976
-6.1788750	4.9288001	112.4000015
-6.1788750	4.9776001	109.0699997
-6.1788750	5.0264001	107.4599991
-6.1788750	5.0752001	109.0499954
-6.1788750	5.1240001	108.3699951
-6.1788750	5.1728001	107.2600021
-6.1788750	5.2216001	107.7600021
-6.1788750	5.2704000	107.6899948
-6.1788750	5.3192000	106.6299973
-6.1788750	5.3680000	106.5599976
-6.1788750	5.4168000	105.8099976
-6.1788750	5.4656000	105.9099960
-6.1788750	5.5144000	106.0699997
-6.1788750	5.5632000	105.7999954
-6.1788750	5.6120000	107.7099991
-6.1788750	5.6608000	103.6800003
-6.1788750	5.7096000	104.1500015
-6.1788750	5.7584000	104.9199982
-6.1788750	5.8072000	103.0199966
-6.1788750	5.8559999	102.3899994
-6.1788750	5.9047999	102.5999985
-6.1788750	5.9535999	101.2099991
-6.1788750	6.0023999	98.9399948
-6.1788750	6.0511999	104.0499954
-6.1788750	6.0999999	104.1699982
-6.1788750	6.1487999	105.3300018
-6.1788750	6.1975999	106.5899963
-6.1788750	6.2463999	107.1399994
-6.1788750	6.2951999	102.9700012
-6.1788750	6.3439999	103.4199982
-6.1788750	6.3927999	108.2799988
-6.1788750	6.4415998	110.0699997
-6.1788750	6.4903998	108.0499954
-6.1788750	6.5391998	106.1800003
-6.1788750	6.5879998	105.3899994
-6.1788750	6.6367998	104.5800018
-6.1788750	6.6855998	104.4899979
-6.1788750	6.7343998	105.3300018
-6.1788750	6.7831998	105.5199966
-6.1788750	6.8319998	103.1199951
-6.1788750	6.8807998	105.1100006
-6.1788750	6.9295998	105.3399963
-6.1788750	6.9783998	102.8899994
-6.1788750	7.0271997	100.9599991
-6.1788750	7.0759997	101.6800003
-6.1788750	7.1247997	103.9199982
-6.1788750	7.1735997	103.6500015
-6.1788750	7.2223997	103.2500000
-6.1788750	7.2711997	102.5800018
-6.1788750	7.3199997	101.6299973

-6.1788750	7.3687997	99.5800018
-6.1788750	7.4175997	103.2399979
-6.1788750	7.4663997	102.7399979
-6.1788750	7.5151997	100.4700012
-6.1788750	7.5639997	105.2600021
-6.1788750	7.6127996	104.7799988
-6.1788750	7.6615996	104.5100021
-6.1788750	7.7103996	101.4300003
-6.1788750	7.7591996	101.2399979
-6.1788750	7.8079996	99.6599960
-6.1788750	7.8567996	101.7799988
-6.1788750	7.9056001	101.5899963
-6.1788750	7.9544001	101.9799957
-6.1788750	8.0032005	102.7299957
-6.1788750	8.0520000	104.3799973
-6.1788750	8.1008005	104.1399994
-6.1788750	8.1496000	104.5199966
-6.1788750	8.1984005	104.2999954
-6.1788750	8.2472000	106.7399979
-6.1788750	8.2960005	103.5199966
-6.1788750	8.3448000	104.1800003
-6.1788750	8.3936005	102.5699997
-6.1788750	8.4424000	102.3499985
-6.1788750	8.4912004	104.7099991
-6.1788750	8.5400000	101.7699966
-6.1788750	8.5888004	100.3899994
-6.1788750	8.6375999	104.3300018
-6.1788750	8.6864004	104.9599991
-6.1788750	8.7351999	102.1899948
-6.1788750	8.7840004	102.3600006
-6.1788750	8.8327999	100.4099960
-6.1788750	8.8816004	100.2500000
-6.1788750	8.9303999	104.7099991
-6.1788750	8.9792004	105.4000015
-6.1788750	9.0279999	103.2999954
-6.1788750	9.0768003	104.9399948
-6.1788750	9.1255999	106.2500000
-6.1788750	9.1744003	105.7900009
-6.1788750	9.2231998	106.0599976
-6.1788750	9.2720003	104.1199951
-6.1788750	9.3207998	103.9899979
-6.1788750	9.3696003	104.6699982
-6.1788750	9.4184008	101.0599976
-6.1788750	9.4672003	101.1599960
-6.1788750	9.5160007	102.3399963
-6.1788750	9.5648003	101.7600021
-6.1788750	9.6136007	99.4899979
-6.1788750	9.6624002	104.1100006
-6.1788750	9.7112007	103.9599991
-6.1788750	9.7600002	100.2699966
-6.1788750	9.8088007	101.9599991
-6.1788750	9.8576002	102.4599991
-6.1788750	9.9064007	103.2500000
-6.1788750	9.9552002	103.1399994
-6.1788750	10.0040007	100.3799973
-6.1788750	10.0528002	98.0299988
-6.1788750	10.1016006	101.9300003
-6.1788750	10.1504002	100.3600006

-6.1788750	10.1992006	103.5499954
-6.1788750	10.2480001	103.3799973
-6.1788750	10.2968006	103.6899948
-6.1788750	10.3456001	104.0499954
-6.1788750	10.3944006	104.2799988
-6.1788750	10.4432001	102.3799973
-6.1788750	10.4920006	101.1399994
-6.1788750	10.5408001	104.6800003
-6.1788750	10.5896006	105.7200012
-6.1788750	10.6384001	103.6999969
-6.1788750	10.6872005	100.4399948
-6.1788750	10.7360001	102.2600021
-6.1788750	10.7848005	102.7500000
-6.1788750	10.8336000	102.0199966
-6.1788750	10.8824005	101.5100021
-6.1788750	10.9312000	103.7799988
-6.1788750	10.9800005	102.9199982
-6.1788750	11.0288000	103.4000015
-6.1788750	11.0776005	102.0400009
-6.1788750	11.1264000	100.4700012
-6.1788750	11.1752005	103.7699966
-6.1788750	11.2240000	106.2399979
-6.1788750	11.2728004	106.2799988
-6.1788750	11.3216000	104.8399963
-6.1788750	11.3704004	106.8600006
-6.1788750	11.4191999	106.2900009
-6.1788750	11.4680004	106.6800003
-6.1788750	11.5167999	106.8300018
-6.1788750	11.5656004	106.9399948
-6.1788750	11.6143999	104.5100021
-6.1788750	11.6632004	104.3699951
-6.1788750	11.7119999	106.4499969
-6.1788750	11.7608004	106.2799988
-6.1788750	11.8095999	106.1100006
-6.1788750	11.8584003	104.6399994
-6.1788750	11.9071999	102.5599976
-6.1788750	11.9560003	104.5800018
-6.1788750	12.0047998	105.7099991
-6.1788750	12.0536003	106.0800018
-6.1788750	12.1023998	105.8600006
-6.1788750	12.1512003	103.8499985
-6.1788750	12.1999998	102.0199966
-6.1788750	12.2488003	101.0699997
-6.1788750	12.2975998	101.4499969
-6.1788750	12.3464003	102.4599991
-6.1788750	12.3951998	103.9099960
-6.1788750	12.4440002	103.5899963
-6.1788750	12.4927998	106.0499954
-6.1788750	12.5416002	106.1699982
-6.1788750	12.5904007	103.4099960
-6.1788750	12.6392002	104.5999985
-6.1788750	12.6880007	103.7699966
-6.1788750	12.7368002	103.9499969
-6.1788750	12.7856007	106.5800018
-6.1788750	12.8344002	106.0599976
-6.1788750	12.8832006	102.0699997
-6.1788750	12.9320002	102.9099960
-6.1788750	12.9808006	105.5199966

-6.1788750	13.0296001	105.7600021
-6.1788750	13.0784006	103.1399994
-6.1788750	13.1272001	105.0299988
-6.1788750	13.1760006	105.1100006
-6.1788750	13.2248001	101.0699997
-6.1788750	13.2736006	102.3899994
-6.1788750	13.3224001	103.3799973
-6.1788750	13.3712006	100.9199982
-6.1788750	13.4200001	103.7500000
-6.1788750	13.4688005	105.1999969
-6.1788750	13.5176001	102.7900009
-6.1788750	13.5664005	105.9000015
-6.1788750	13.6152000	103.1199951
-6.1788750	13.6640005	102.3199997
-6.1788750	13.7128000	102.2399979
-6.1788750	13.7616005	103.6500015
-6.1788750	13.8104000	102.9099960
-6.1788750	13.8592005	102.3699951
-6.1788750	13.9080000	100.7500000
-6.1788750	13.9568005	102.7099991
-6.1788750	14.0056000	101.9799957
-6.1788750	14.0544004	100.7500000
-6.1788750	14.1032000	102.2099991
-6.1788750	14.1520004	103.7799988
-6.1788750	14.2007999	103.3300018
-6.1788750	14.2496004	103.2999954
-6.1788750	14.2983999	103.0699997
-6.1788750	14.3472004	104.3099976
-6.1788750	14.3959999	102.6999969
-6.1788750	14.4448004	100.7200012
-6.1788750	14.4935999	101.6699982
-6.1788750	14.5424004	99.3099976
-6.1788750	14.5911999	97.9199982
-6.1788750	14.6400003	101.4799957
-6.1788750	14.6887999	102.4799957
-6.1788750	14.7376003	101.5000000
-6.1788750	14.7863998	101.5800018
-6.1788750	14.8352003	102.3399963
-6.1788750	14.8839998	100.7399979
-6.1788750	14.9328003	99.6899948
-6.1788750	14.9815998	102.6100006
-6.1788750	15.0304003	104.6399994
-6.1727390	0.3416000	109.1500015
-6.1727390	0.3904000	110.5599976
-6.1727390	0.4392000	110.0000000
-6.1727390	0.4880000	108.2799988
-6.1727390	0.5368000	107.7399979
-6.1727390	0.5856000	105.6899948
-6.1727390	0.6344000	106.1899948
-6.1727390	0.6832000	107.6800003
-6.1727390	0.7320000	101.2399979
-6.1727390	0.7808000	106.5599976
-6.1727390	0.8296000	109.1699982
-6.1727390	0.8784000	109.4799957
-6.1727390	0.9272000	106.5400009
-6.1727390	0.9760000	108.0899963
-6.1727390	1.0247999	108.2500000
-6.1727390	1.0735999	108.2200012

-6.1727390	1.1223999	109.6199951
-6.1727390	1.1711999	107.7900009
-6.1727390	1.2200000	105.2799988
-6.1727390	1.2688000	106.5100021
-6.1727390	1.3176000	106.1500015
-6.1727390	1.3664000	106.0899963
-6.1727390	1.4152000	108.7699966
-6.1727390	1.4640000	110.0499954
-6.1727390	1.5128000	110.0999985
-6.1727390	1.5616000	110.1599960
-6.1727390	1.6104000	112.2099991
-6.1727390	1.6592000	110.5999985
-6.1727390	1.7079999	111.9099960
-6.1727390	1.7567999	110.7099991
-6.1727390	1.8055999	110.9899979
-6.1727390	1.8543999	117.7299957
-6.1727390	1.9031999	118.1199951
-6.1727390	1.9519999	112.8699951
-6.1727390	2.0007999	106.7600021
-6.1727390	2.0495999	108.1500015
-6.1727390	2.0983999	106.9099960
-6.1727390	2.1471999	104.4399948
-6.1727390	2.1959999	102.8099976
-6.1727390	2.2447999	104.8300018
-6.1727390	2.2935998	105.9099960
-6.1727390	2.3423998	107.6599960
-6.1727390	2.3912001	106.1100006
-6.1727390	2.4400001	104.8699951
-6.1727390	2.4888000	105.4300003
-6.1727390	2.5376000	106.6599960
-6.1727390	2.5864000	106.6999969
-6.1727390	2.6352000	105.5299988
-6.1727390	2.6840000	105.1199951
-6.1727390	2.7328000	105.9799957
-6.1727390	2.7816000	106.8199997
-6.1727390	2.8304000	107.2699966
-6.1727390	2.8792000	106.1500015
-6.1727390	2.9280000	104.6399994
-6.1727390	2.9768000	109.0100021
-6.1727390	3.0256000	108.8899994
-6.1727390	3.0743999	105.7299957
-6.1727390	3.1231999	103.9399948
-6.1727390	3.1719999	107.3399963
-6.1727390	3.2207999	104.0999985
-6.1727390	3.2695999	104.5400009
-6.1727390	3.3183999	107.6800003
-6.1727390	3.3671999	107.0100021
-6.1727390	3.4159999	103.1899948
-6.1727390	3.4647999	105.1500015
-6.1727390	3.5135999	103.8699951
-6.1727390	3.5623999	104.5999985
-6.1727390	3.6111999	105.0899963
-6.1727390	3.6599998	101.8899994
-6.1727390	3.7087998	104.2099991
-6.1727390	3.7575998	104.1999969
-6.1727390	3.8063998	104.0400009
-6.1727390	3.8551998	106.1399994
-6.1727390	3.9039998	104.7600021

-6.1727390	3.9527998	98.5599976
-6.1727390	4.0016003	105.7500000
-6.1727390	4.0503998	107.0100021
-6.1727390	4.0991998	107.1299973
-6.1727390	4.1479998	103.9399948
-6.1727390	4.1967998	100.1599960
-6.1727390	4.2455997	103.3099976
-6.1727390	4.2943997	110.2299957
-6.1727390	4.3431997	112.9499969
-6.1727390	4.3919997	108.5299988
-6.1727390	4.4407997	110.2699966
-6.1727390	4.4895997	113.2299957
-6.1727390	4.5383997	116.3799973
-6.1727390	4.5871997	118.7799988
-6.1727390	4.6359997	117.3600006
-6.1727390	4.6847997	116.2999954
-6.1727390	4.7336001	114.6599960
-6.1727390	4.7824001	109.4599991
-6.1727390	4.8312001	113.4300003
-6.1727390	4.8800001	114.7399979
-6.1727390	4.9288001	113.4199982
-6.1727390	4.9776001	108.7600021
-6.1727390	5.0264001	106.4700012
-6.1727390	5.0752001	107.6100006
-6.1727390	5.1240001	107.1299973
-6.1727390	5.1728001	106.7699966
-6.1727390	5.2216001	107.5599976
-6.1727390	5.2704000	108.0599976
-6.1727390	5.3192000	106.8199997
-6.1727390	5.3680000	106.1299973
-6.1727390	5.4168000	105.2699966
-6.1727390	5.4656000	105.8099976
-6.1727390	5.5144000	105.8899994
-6.1727390	5.5632000	105.7999954
-6.1727390	5.6120000	106.7799988
-6.1727390	5.6608000	101.6800003
-6.1727390	5.7096000	103.5000000
-6.1727390	5.7584000	105.1199951
-6.1727390	5.8072000	102.8199997
-6.1727390	5.8559999	102.5400009
-6.1727390	5.9047999	103.5499954
-6.1727390	5.9535999	102.5100021
-6.1727390	6.0023999	99.5400009
-6.1727390	6.0511999	104.4700012
-6.1727390	6.0999999	104.8300018
-6.1727390	6.1487999	106.0000000
-6.1727390	6.1975999	106.4000015
-6.1727390	6.2463999	106.0899963
-6.1727390	6.2951999	102.4000015
-6.1727390	6.3439999	103.9799957
-6.1727390	6.3927999	108.4000015
-6.1727390	6.4415998	110.4799957
-6.1727390	6.4903998	108.5899963
-6.1727390	6.5391998	105.7699966
-6.1727390	6.5879998	104.8499985
-6.1727390	6.6367998	104.2900009
-6.1727390	6.6855998	104.5599976
-6.1727390	6.7343998	105.4499969

-6.1727390	6.7831998	104.9099960
-6.1727390	6.8319998	101.4799957
-6.1727390	6.8807998	105.8300018
-6.1727390	6.9295998	106.0199966
-6.1727390	6.9783998	103.1299973
-6.1727390	7.0271997	100.7500000
-6.1727390	7.0759997	101.5499954
-6.1727390	7.1247997	102.9899979
-6.1727390	7.1735997	103.5299988
-6.1727390	7.2223997	103.4300003
-6.1727390	7.2711997	102.1500015
-6.1727390	7.3199997	101.8199997
-6.1727390	7.3687997	100.6500015
-6.1727390	7.4175997	102.6599960
-6.1727390	7.4663997	102.8399963
-6.1727390	7.5151997	101.0499954
-6.1727390	7.5639997	103.5800018
-6.1727390	7.6127996	103.1800003
-6.1727390	7.6615996	104.1299973
-6.1727390	7.7103996	101.2299957
-6.1727390	7.7591996	101.9700012
-6.1727390	7.8079996	100.3899994
-6.1727390	7.8567996	100.9700012
-6.1727390	7.9056001	100.8399963
-6.1727390	7.9544001	102.8499985
-6.1727390	8.0032005	104.3099976
-6.1727390	8.0520000	104.7799988
-6.1727390	8.1008005	103.5299988
-6.1727390	8.1496000	104.0699997
-6.1727390	8.1984005	104.8099976
-6.1727390	8.2472000	107.1199951
-6.1727390	8.2960005	104.4300003
-6.1727390	8.3448000	104.7900009
-6.1727390	8.3936005	102.1500015
-6.1727390	8.4424000	101.6500015
-6.1727390	8.4912004	104.0899963
-6.1727390	8.5400000	100.5800018
-6.1727390	8.5888004	99.6100006
-6.1727390	8.6375999	104.4199982
-6.1727390	8.6864004	105.4599991
-6.1727390	8.7351999	103.7200012
-6.1727390	8.7840004	103.3600006
-6.1727390	8.8327999	99.6100006
-6.1727390	8.8816004	100.1100006
-6.1727390	8.9303999	105.2099991
-6.1727390	8.9792004	106.2699966
-6.1727390	9.0279999	103.9199982
-6.1727390	9.0768003	104.7999954
-6.1727390	9.1255999	106.6800003
-6.1727390	9.1744003	106.0599976
-6.1727390	9.2231998	105.5299988
-6.1727390	9.2720003	104.9000015
-6.1727390	9.3207998	104.1399994
-6.1727390	9.3696003	104.5400009
-6.1727390	9.4184008	100.9700012
-6.1727390	9.4672003	100.6399994
-6.1727390	9.5160007	102.2099991
-6.1727390	9.5648003	102.0100021

-6.1727390	9.6136007	99.7099991
-6.1727390	9.6624002	104.0999985
-6.1727390	9.7112007	104.5499954
-6.1727390	9.7600002	100.8799973
-6.1727390	9.8088007	102.7299957
-6.1727390	9.8576002	102.3699951
-6.1727390	9.9064007	103.2500000
-6.1727390	9.9552002	102.7299957
-6.1727390	10.0040007	99.7399979
-6.1727390	10.0528002	98.2999954
-6.1727390	10.1016006	103.5000000
-6.1727390	10.1504002	101.5000000
-6.1727390	10.1992006	103.4000015
-6.1727390	10.2480001	103.6699982
-6.1727390	10.2968006	103.0499954
-6.1727390	10.3456001	103.9599991
-6.1727390	10.3944006	103.9300003
-6.1727390	10.4432001	101.5499954
-6.1727390	10.4920006	99.8699951
-6.1727390	10.5408001	103.9199982
-6.1727390	10.5896006	105.0999985
-6.1727390	10.6384001	103.1299973
-6.1727390	10.6872005	99.9499969
-6.1727390	10.7360001	102.3399963
-6.1727390	10.7848005	102.2699966
-6.1727390	10.8336000	102.4099960
-6.1727390	10.8824005	102.4000015
-6.1727390	10.9312000	104.0299988
-6.1727390	10.9800005	103.8899994
-6.1727390	11.0288000	103.5699997
-6.1727390	11.0776005	101.7999954
-6.1727390	11.1264000	99.1699982
-6.1727390	11.1752005	104.4199982
-6.1727390	11.2240000	107.0999985
-6.1727390	11.2728004	106.3399963
-6.1727390	11.3216000	105.7999954
-6.1727390	11.3704004	106.7699966
-6.1727390	11.4191999	105.6299973
-6.1727390	11.4680004	106.2999954
-6.1727390	11.5167999	106.8199997
-6.1727390	11.5656004	107.2299957
-6.1727390	11.6143999	104.9199982
-6.1727390	11.6632004	103.2699966
-6.1727390	11.7119999	105.9700012
-6.1727390	11.7608004	106.0599976
-6.1727390	11.8095999	105.9199982
-6.1727390	11.8584003	104.8199997
-6.1727390	11.9071999	102.5400009
-6.1727390	11.9560003	104.1999969
-6.1727390	12.0047998	104.5299988
-6.1727390	12.0536003	105.6199951
-6.1727390	12.1023998	105.1399994
-6.1727390	12.1512003	103.2299957
-6.1727390	12.1999998	101.7299957
-6.1727390	12.2488003	101.3300018
-6.1727390	12.2975998	102.6399994
-6.1727390	12.3464003	102.3099976
-6.1727390	12.3951998	102.8300018

-6.1727390	12.4440002	101.6399994
-6.1727390	12.4927998	105.7699966
-6.1727390	12.5416002	105.9899979
-6.1727390	12.5904007	102.6299973
-6.1727390	12.6392002	103.7099991
-6.1727390	12.6880007	103.7299957
-6.1727390	12.7368002	104.8600006
-6.1727390	12.7856007	107.3899994
-6.1727390	12.8344002	106.3099976
-6.1727390	12.8832006	102.1399994
-6.1727390	12.9320002	102.4799957
-6.1727390	12.9808006	105.7299957
-6.1727390	13.0296001	106.0000000
-6.1727390	13.0784006	102.3799973
-6.1727390	13.1272001	103.4300003
-6.1727390	13.1760006	104.1599960
-6.1727390	13.2248001	100.9499969
-6.1727390	13.2736006	101.7299957
-6.1727390	13.3224001	103.3499985
-6.1727390	13.3712006	101.9300003
-6.1727390	13.4200001	104.0400009
-6.1727390	13.4688005	104.9300003
-6.1727390	13.5176001	100.8699951
-6.1727390	13.5664005	105.3300018
-6.1727390	13.6152000	104.3099976
-6.1727390	13.6640005	103.6899948
-6.1727390	13.7128000	104.4300003
-6.1727390	13.7616005	105.2299957
-6.1727390	13.8104000	103.7099991
-6.1727390	13.8592005	102.1100006
-6.1727390	13.9080000	99.8399963
-6.1727390	13.9568005	102.7299957
-6.1727390	14.0056000	101.7799988
-6.1727390	14.0544004	101.0400009
-6.1727390	14.1032000	100.8199997
-6.1727390	14.1520004	103.4700012
-6.1727390	14.2007999	103.4700012
-6.1727390	14.2496004	102.9300003
-6.1727390	14.2983999	102.9700012
-6.1727390	14.3472004	105.0899963
-6.1727390	14.3959999	102.9199982
-6.1727390	14.4448004	99.8699951
-6.1727390	14.4935999	101.3499985
-6.1727390	14.5424004	99.0899963
-6.1727390	14.5911999	98.1599960
-6.1727390	14.6400003	102.0800018
-6.1727390	14.6887999	102.8499985
-6.1727390	14.7376003	100.3199997
-6.1727390	14.7863998	99.9599991
-6.1727390	14.8352003	101.7399979
-6.1727390	14.8839998	100.2399979
-6.1727390	14.9328003	101.0699997
-6.1727390	14.9815998	102.4499969
-6.1727390	15.0304003	104.7799988
-6.1666031	0.3416000	110.1899948
-6.1666031	0.3904000	110.8099976
-6.1666031	0.4392000	110.0499954
-6.1666031	0.4880000	108.3399963

-6.1666031	0.5368000	107.2699966
-6.1666031	0.5856000	105.7999954
-6.1666031	0.6344000	105.7200012
-6.1666031	0.6832000	107.4700012
-6.1666031	0.7320000	100.6100006
-6.1666031	0.7808000	106.3499985
-6.1666031	0.8296000	108.6999969
-6.1666031	0.8784000	108.3699951
-6.1666031	0.9272000	106.0499954
-6.1666031	0.9760000	107.4700012
-6.1666031	1.0247999	107.6100006
-6.1666031	1.0735999	108.3699951
-6.1666031	1.1223999	109.8300018
-6.1666031	1.1711999	106.7900009
-6.1666031	1.2200000	103.9899979
-6.1666031	1.2688000	104.3799973
-6.1666031	1.3176000	104.9599991
-6.1666031	1.3664000	105.7900009
-6.1666031	1.4152000	108.9599991
-6.1666031	1.4640000	109.9700012
-6.1666031	1.5128000	109.3799973
-6.1666031	1.5616000	109.9300003
-6.1666031	1.6104000	111.6599960
-6.1666031	1.6592000	110.3399963
-6.1666031	1.7079999	112.1299973
-6.1666031	1.7567999	110.9399948
-6.1666031	1.8055999	111.1100006
-6.1666031	1.8543999	117.4399948
-6.1666031	1.9031999	117.8099976
-6.1666031	1.9519999	112.2200012
-6.1666031	2.0007999	105.2500000
-6.1666031	2.0495999	107.3499985
-6.1666031	2.0983999	106.4000015
-6.1666031	2.1471999	103.6500015
-6.1666031	2.1959999	101.5800018
-6.1666031	2.2447999	104.6999969
-6.1666031	2.2935998	106.4799957
-6.1666031	2.3423998	106.7500000
-6.1666031	2.3912001	106.0899963
-6.1666031	2.4400001	103.9199982
-6.1666031	2.4888000	105.4899979
-6.1666031	2.5376000	107.5400009
-6.1666031	2.5864000	107.8899994
-6.1666031	2.6352000	106.1500015
-6.1666031	2.6840000	104.8799973
-6.1666031	2.7328000	106.4300003
-6.1666031	2.7816000	106.8600006
-6.1666031	2.8304000	107.3600006
-6.1666031	2.8792000	106.0699997
-6.1666031	2.9280000	104.2500000
-6.1666031	2.9768000	108.5499954
-6.1666031	3.0256000	108.5899963
-6.1666031	3.0743999	106.0800018
-6.1666031	3.1231999	105.1599960
-6.1666031	3.1719999	108.2999954
-6.1666031	3.2207999	106.0800018
-6.1666031	3.2695999	105.5100021
-6.1666031	3.3183999	107.4700012

-6.1666031	3.3671999	105.3199997
-6.1666031	3.4159999	101.4199982
-6.1666031	3.4647999	105.1999969
-6.1666031	3.5135999	104.2299957
-6.1666031	3.5623999	104.7399979
-6.1666031	3.6111999	104.8600006
-6.1666031	3.6599998	101.4899979
-6.1666031	3.7087998	103.5199966
-6.1666031	3.7575998	104.6599960
-6.1666031	3.8063998	104.5999985
-6.1666031	3.8551998	106.3199997
-6.1666031	3.9039998	104.4799957
-6.1666031	3.9527998	96.2299957
-6.1666031	4.0016003	104.2399979
-6.1666031	4.0503998	106.2999954
-6.1666031	4.0991998	107.0499954
-6.1666031	4.1479998	104.2500000
-6.1666031	4.1967998	100.0499954
-6.1666031	4.2455997	103.4000015
-6.1666031	4.2943997	110.1299973
-6.1666031	4.3431997	113.4399948
-6.1666031	4.3919997	109.4700012
-6.1666031	4.4407997	110.2200012
-6.1666031	4.4895997	113.5899963
-6.1666031	4.5383997	116.9799957
-6.1666031	4.5871997	118.8600006
-6.1666031	4.6359997	117.0599976
-6.1666031	4.6847997	116.0199966
-6.1666031	4.7336001	114.7699966
-6.1666031	4.7824001	110.0299988
-6.1666031	4.8312001	112.8600006
-6.1666031	4.8800001	114.8600006
-6.1666031	4.9288001	113.4399948
-6.1666031	4.9776001	108.1299973
-6.1666031	5.0264001	105.4499969
-6.1666031	5.0752001	105.8799973
-6.1666031	5.1240001	105.5299988
-6.1666031	5.1728001	105.9099960
-6.1666031	5.2216001	107.0299988
-6.1666031	5.2704000	108.5400009
-6.1666031	5.3192000	107.6299973
-6.1666031	5.3680000	106.2099991
-6.1666031	5.4168000	105.2999954
-6.1666031	5.4656000	105.1899948
-6.1666031	5.5144000	105.2299957
-6.1666031	5.5632000	105.3799973
-6.1666031	5.6120000	105.2500000
-6.1666031	5.6608000	99.0400009
-6.1666031	5.7096000	103.4899979
-6.1666031	5.7584000	105.8600006
-6.1666031	5.8072000	103.7200012
-6.1666031	5.8559999	102.8600006
-6.1666031	5.9047999	103.9000015
-6.1666031	5.9535999	103.1699982
-6.1666031	6.0023999	99.9799957
-6.1666031	6.0511999	104.8899994
-6.1666031	6.0999999	105.7999954
-6.1666031	6.1487999	106.1399994

-6.1666031	6.1975999	106.2200012
-6.1666031	6.2463999	105.6100006
-6.1666031	6.2951999	102.9700012
-6.1666031	6.3439999	104.6100006
-6.1666031	6.3927999	108.5499954
-6.1666031	6.4415998	110.9300003
-6.1666031	6.4903998	109.1999969
-6.1666031	6.5391998	105.6199951
-6.1666031	6.5879998	104.2799988
-6.1666031	6.6367998	103.8699951
-6.1666031	6.6855998	104.7299957
-6.1666031	6.7343998	105.8099976
-6.1666031	6.7831998	103.7699966
-6.1666031	6.8319998	100.6500015
-6.1666031	6.8807998	106.4199982
-6.1666031	6.9295998	106.4899979
-6.1666031	6.9783998	103.5499954
-6.1666031	7.0271997	101.5400009
-6.1666031	7.0759997	102.5199966
-6.1666031	7.1247997	102.7900009
-6.1666031	7.1735997	103.6199951
-6.1666031	7.2223997	103.7900009
-6.1666031	7.2711997	102.0800018
-6.1666031	7.3199997	101.4700012
-6.1666031	7.3687997	101.6599960
-6.1666031	7.4175997	102.9799957
-6.1666031	7.4663997	103.5100021
-6.1666031	7.5151997	102.1899948
-6.1666031	7.5639997	102.6100006
-6.1666031	7.6127996	101.8600006
-6.1666031	7.6615996	104.2999954
-6.1666031	7.7103996	102.6699982
-6.1666031	7.7591996	102.1800003
-6.1666031	7.8079996	100.8399963
-6.1666031	7.8567996	100.6100006
-6.1666031	7.9056001	100.2299957
-6.1666031	7.9544001	103.6199951
-6.1666031	8.0032005	106.0800018
-6.1666031	8.0520000	105.5699997
-6.1666031	8.1008005	103.7200012
-6.1666031	8.1496000	103.9599991
-6.1666031	8.1984005	104.7900009
-6.1666031	8.2472000	106.4000015
-6.1666031	8.2960005	104.8300018
-6.1666031	8.3448000	105.5100021
-6.1666031	8.3936005	101.9599991
-6.1666031	8.4424000	100.9799957
-6.1666031	8.4912004	104.1500015
-6.1666031	8.5400000	100.8899994
-6.1666031	8.5888004	98.9599991
-6.1666031	8.6375999	104.1899948
-6.1666031	8.6864004	105.9099960
-6.1666031	8.7351999	105.1100006
-6.1666031	8.7840004	103.4499969
-6.1666031	8.8327999	98.6699982
-6.1666031	8.8816004	99.8799973
-6.1666031	8.9303999	105.1299973
-6.1666031	8.9792004	106.1699982

-6.1666031	9.0279999	103.8600006
-6.1666031	9.0768003	104.6999969
-6.1666031	9.1255999	107.0199966
-6.1666031	9.1744003	105.7999954
-6.1666031	9.2231998	104.3199997
-6.1666031	9.2720003	104.9499969
-6.1666031	9.3207998	104.3799973
-6.1666031	9.3696003	104.9899979
-6.1666031	9.4184008	101.7600021
-6.1666031	9.4672003	100.8899994
-6.1666031	9.5160007	103.0499954
-6.1666031	9.5648003	102.3399963
-6.1666031	9.6136007	99.0899963
-6.1666031	9.6624002	103.5800018
-6.1666031	9.7112007	104.7200012
-6.1666031	9.7600002	101.3499985
-6.1666031	9.8088007	103.7699966
-6.1666031	9.8576002	102.9000015
-6.1666031	9.9064007	104.3499985
-6.1666031	9.9552002	103.3799973
-6.1666031	10.0040007	99.7299957
-6.1666031	10.0528002	100.1399994
-6.1666031	10.1016006	105.2299957
-6.1666031	10.1504002	103.3899994
-6.1666031	10.1992006	103.5199966
-6.1666031	10.2480001	103.1899948
-6.1666031	10.2968006	101.7200012
-6.1666031	10.3456001	103.4099960
-6.1666031	10.3944006	103.5999985
-6.1666031	10.4432001	101.5999985
-6.1666031	10.4920006	99.0400009
-6.1666031	10.5408001	102.8199997
-6.1666031	10.5896006	104.3899994
-6.1666031	10.6384001	102.9399948
-6.1666031	10.6872005	99.7200012
-6.1666031	10.7360001	103.0199966
-6.1666031	10.7848005	102.0499954
-6.1666031	10.8336000	103.3499985
-6.1666031	10.8824005	103.4000015
-6.1666031	10.9312000	103.3600006
-6.1666031	10.9800005	104.0699997
-6.1666031	11.0288000	103.3699951
-6.1666031	11.0776005	101.5000000
-6.1666031	11.1264000	98.2399979
-6.1666031	11.1752005	105.3099976
-6.1666031	11.2240000	107.7299957
-6.1666031	11.2728004	105.7600021
-6.1666031	11.3216000	105.2699966
-6.1666031	11.3704004	106.3399963
-6.1666031	11.4191999	104.8300018
-6.1666031	11.4680004	105.6500015
-6.1666031	11.5167999	106.3600006
-6.1666031	11.5656004	107.1399994
-6.1666031	11.6143999	105.2099991
-6.1666031	11.6632004	102.2699966
-6.1666031	11.7119999	104.8799973
-6.1666031	11.7608004	105.0199966
-6.1666031	11.8095999	104.8899994

-6.1666031	11.8584003	104.6199951
-6.1666031	11.9071999	103.1199951
-6.1666031	11.9560003	103.9799957
-6.1666031	12.0047998	103.9599991
-6.1666031	12.0536003	105.5100021
-6.1666031	12.1023998	104.4599991
-6.1666031	12.1512003	103.5899963
-6.1666031	12.1999998	102.6199951
-6.1666031	12.2488003	102.1800003
-6.1666031	12.2975998	104.0800018
-6.1666031	12.3464003	103.0000000
-6.1666031	12.3951998	102.4000015
-6.1666031	12.4440002	100.8300018
-6.1666031	12.4927998	105.2600021
-6.1666031	12.5416002	106.0199966
-6.1666031	12.5904007	102.2699966
-6.1666031	12.6392002	102.4300003
-6.1666031	12.6880007	103.6399994
-6.1666031	12.7368002	105.2200012
-6.1666031	12.7856007	107.2900009
-6.1666031	12.8344002	105.7600021
-6.1666031	12.8832006	101.5999985
-6.1666031	12.9320002	101.9700012
-6.1666031	12.9808006	105.4399948
-6.1666031	13.0296001	105.8099976
-6.1666031	13.0784006	101.8699951
-6.1666031	13.1272001	101.8099976
-6.1666031	13.1760006	102.9399948
-6.1666031	13.2248001	101.4799957
-6.1666031	13.2736006	101.2999954
-6.1666031	13.3224001	103.1599960
-6.1666031	13.3712006	103.2699966
-6.1666031	13.4200001	104.0400009
-6.1666031	13.4688005	104.3699951
-6.1666031	13.5176001	98.9300003
-6.1666031	13.5664005	104.5400009
-6.1666031	13.6152000	104.9099960
-6.1666031	13.6640005	104.4799957
-6.1666031	13.7128000	105.4700012
-6.1666031	13.7616005	105.6899948
-6.1666031	13.8104000	103.1100006
-6.1666031	13.8592005	101.6699982
-6.1666031	13.9080000	98.4700012
-6.1666031	13.9568005	102.4700012
-6.1666031	14.0056000	102.2699966
-6.1666031	14.0544004	101.2399979
-6.1666031	14.1032000	99.1800003
-6.1666031	14.1520004	102.6100006
-6.1666031	14.2007999	103.2699966
-6.1666031	14.2496004	102.6399994
-6.1666031	14.2983999	103.0400009
-6.1666031	14.3472004	105.2799988
-6.1666031	14.3959999	102.6800003
-6.1666031	14.4448004	99.6100006
-6.1666031	14.4935999	100.8300018
-6.1666031	14.5424004	98.5999985
-6.1666031	14.5911999	98.9700012
-6.1666031	14.6400003	102.4899979

-6.1666031	14.6887999	103.0100021
-6.1666031	14.7376003	99.8099976
-6.1666031	14.7863998	99.8199997
-6.1666031	14.8352003	102.1199951
-6.1666031	14.8839998	100.7299957
-6.1666031	14.9328003	102.5299988
-6.1666031	14.9815998	102.3699951
-6.1666031	15.0304003	104.9899979
-6.1604671	0.3416000	110.6899948
-6.1604671	0.3904000	110.2299957
-6.1604671	0.4392000	109.5299988
-6.1604671	0.4880000	107.7500000
-6.1604671	0.5368000	106.1199951
-6.1604671	0.5856000	105.4799957
-6.1604671	0.6344000	104.9300003
-6.1604671	0.6832000	107.3600006
-6.1604671	0.7320000	102.5599976
-6.1604671	0.7808000	106.9899979
-6.1604671	0.8296000	108.2600021
-6.1604671	0.8784000	107.7999954
-6.1604671	0.9272000	105.7299957
-6.1604671	0.9760000	106.4799957
-6.1604671	1.0247999	106.7399979
-6.1604671	1.0735999	108.4199982
-6.1604671	1.1223999	109.7900009
-6.1604671	1.1711999	105.7999954
-6.1604671	1.2200000	103.3399963
-6.1604671	1.2688000	102.7200012
-6.1604671	1.3176000	103.8099976
-6.1604671	1.3664000	105.2999954
-6.1604671	1.4152000	108.5100021
-6.1604671	1.4640000	109.1999969
-6.1604671	1.5128000	108.5699997
-6.1604671	1.5616000	109.4300003
-6.1604671	1.6104000	110.4899979
-6.1604671	1.6592000	109.7600021
-6.1604671	1.7079999	112.2299957
-6.1604671	1.7567999	111.7399979
-6.1604671	1.8055999	111.0599976
-6.1604671	1.8543999	117.3600006
-6.1604671	1.9031999	117.7999954
-6.1604671	1.9519999	111.9799957
-6.1604671	2.0007999	104.3699951
-6.1604671	2.0495999	107.0000000
-6.1604671	2.0983999	106.2600021
-6.1604671	2.1471999	103.0100021
-6.1604671	2.1959999	100.6800003
-6.1604671	2.2447999	104.8199997
-6.1604671	2.2935998	106.7299957
-6.1604671	2.3423998	105.3899994
-6.1604671	2.3912001	106.0899963
-6.1604671	2.4400001	103.6899948
-6.1604671	2.4888000	105.9300003
-6.1604671	2.5376000	108.4799957
-6.1604671	2.5864000	108.6899948
-6.1604671	2.6352000	106.3899994
-6.1604671	2.6840000	104.3600006
-6.1604671	2.7328000	106.2799988

-6.1604671	2.7816000	106.5499954
-6.1604671	2.8304000	107.2600021
-6.1604671	2.8792000	105.7699966
-6.1604671	2.9280000	104.4199982
-6.1604671	2.9768000	108.0999985
-6.1604671	3.0256000	107.7500000
-6.1604671	3.0743999	106.1599960
-6.1604671	3.1231999	105.9000015
-6.1604671	3.1719999	108.4899979
-6.1604671	3.2207999	107.0699997
-6.1604671	3.2695999	106.6399994
-6.1604671	3.3183999	107.1599960
-6.1604671	3.3671999	103.7600021
-6.1604671	3.4159999	100.1299973
-6.1604671	3.4647999	105.0100021
-6.1604671	3.5135999	105.2999954
-6.1604671	3.5623999	105.0999985
-6.1604671	3.6111999	104.7799988
-6.1604671	3.6599998	101.4899979
-6.1604671	3.7087998	103.4000015
-6.1604671	3.7575998	104.4799957
-6.1604671	3.8063998	104.4399948
-6.1604671	3.8551998	106.2099991
-6.1604671	3.9039998	104.3300018
-6.1604671	3.9527998	94.6100006
-6.1604671	4.0016003	102.6199951
-6.1604671	4.0503998	105.2900009
-6.1604671	4.0991998	106.8199997
-6.1604671	4.1479998	105.0499954
-6.1604671	4.1967998	101.2500000
-6.1604671	4.2455997	103.5899963
-6.1604671	4.2943997	109.5999985
-6.1604671	4.3431997	113.3399963
-6.1604671	4.3919997	109.8499985
-6.1604671	4.4407997	110.0000000
-6.1604671	4.4895997	113.2699966
-6.1604671	4.5383997	117.1299973
-6.1604671	4.5871997	118.9099960
-6.1604671	4.6359997	116.9599991
-6.1604671	4.6847997	115.8099976
-6.1604671	4.7336001	114.7500000
-6.1604671	4.7824001	110.2399979
-6.1604671	4.8312001	111.7999954
-6.1604671	4.8800001	114.2399979
-6.1604671	4.9288001	113.5400009
-6.1604671	4.9776001	108.8499985
-6.1604671	5.0264001	105.0699997
-6.1604671	5.0752001	105.5499954
-6.1604671	5.1240001	105.3899994
-6.1604671	5.1728001	105.5699997
-6.1604671	5.2216001	106.0899963
-6.1604671	5.2704000	108.6299973
-6.1604671	5.3192000	107.9499969
-6.1604671	5.3680000	105.7600021
-6.1604671	5.4168000	105.0699997
-6.1604671	5.4656000	104.7600021
-6.1604671	5.5144000	103.8600006
-6.1604671	5.5632000	104.5899963

-6.1604671	5.6120000	103.8799973
-6.1604671	5.6608000	98.3300018
-6.1604671	5.7096000	103.2600021
-6.1604671	5.7584000	105.7399979
-6.1604671	5.8072000	103.6999969
-6.1604671	5.8559999	103.6899948
-6.1604671	5.9047999	104.4099960
-6.1604671	5.9535999	103.7900009
-6.1604671	6.0023999	100.2600021
-6.1604671	6.0511999	105.0999985
-6.1604671	6.0999999	106.3099976
-6.1604671	6.1487999	106.2200012
-6.1604671	6.1975999	106.4799957
-6.1604671	6.2463999	105.2299957
-6.1604671	6.2951999	103.2399979
-6.1604671	6.3439999	104.2600021
-6.1604671	6.3927999	107.4899979
-6.1604671	6.4415998	110.4199982
-6.1604671	6.4903998	109.4700012
-6.1604671	6.5391998	106.2699966
-6.1604671	6.5879998	104.2299957
-6.1604671	6.6367998	104.1199951
-6.1604671	6.6855998	104.5999985
-6.1604671	6.7343998	105.4099960
-6.1604671	6.7831998	102.1699982
-6.1604671	6.8319998	99.4599991
-6.1604671	6.8807998	106.2399979
-6.1604671	6.9295998	106.8399963
-6.1604671	6.9783998	104.4599991
-6.1604671	7.0271997	102.4599991
-6.1604671	7.0759997	103.4000015
-6.1604671	7.1247997	102.9099960
-6.1604671	7.1735997	103.7699966
-6.1604671	7.2223997	104.4599991
-6.1604671	7.2711997	103.2399979
-6.1604671	7.3199997	102.4899979
-6.1604671	7.3687997	102.6599960
-6.1604671	7.4175997	102.9399948
-6.1604671	7.4663997	103.7600021
-6.1604671	7.5151997	103.1599960
-6.1604671	7.5639997	102.0599976
-6.1604671	7.6127996	100.7500000
-6.1604671	7.6615996	104.3199997
-6.1604671	7.7103996	104.2500000
-6.1604671	7.7591996	102.7399979
-6.1604671	7.8079996	101.1599960
-6.1604671	7.8567996	100.5100021
-6.1604671	7.9056001	100.0999985
-6.1604671	7.9544001	104.1999969
-6.1604671	8.0032005	106.8699951
-6.1604671	8.0520000	106.2299957
-6.1604671	8.1008005	104.1599960
-6.1604671	8.1496000	104.1599960
-6.1604671	8.1984005	104.9599991
-6.1604671	8.2472000	105.5599976
-6.1604671	8.2960005	104.6199951
-6.1604671	8.3448000	105.1599960
-6.1604671	8.3936005	101.7699966

-6.1604671	8.4424000	100.1399994
-6.1604671	8.4912004	104.3099976
-6.1604671	8.5400000	101.8499985
-6.1604671	8.5888004	98.2299957
-6.1604671	8.6375999	103.4899979
-6.1604671	8.6864004	105.8499985
-6.1604671	8.7351999	106.0299988
-6.1604671	8.7840004	103.3499985
-6.1604671	8.8327999	98.3300018
-6.1604671	8.8816004	100.6199951
-6.1604671	8.9303999	104.9300003
-6.1604671	8.9792004	105.3899994
-6.1604671	9.0279999	103.5199966
-6.1604671	9.0768003	105.0000000
-6.1604671	9.1255999	107.2399979
-6.1604671	9.1744003	105.5199966
-6.1604671	9.2231998	102.9899979
-6.1604671	9.2720003	105.2200012
-6.1604671	9.3207998	104.4099960
-6.1604671	9.3696003	104.8300018
-6.1604671	9.4184008	101.9199982
-6.1604671	9.4672003	100.8699951
-6.1604671	9.5160007	103.5899963
-6.1604671	9.5648003	102.8799973
-6.1604671	9.6136007	99.5000000
-6.1604671	9.6624002	103.0100021
-6.1604671	9.7112007	104.3399963
-6.1604671	9.7600002	102.8600006
-6.1604671	9.8088007	104.7699966
-6.1604671	9.8576002	103.2699966
-6.1604671	9.9064007	104.7999954
-6.1604671	9.9552002	103.5000000
-6.1604671	10.0040007	99.6899948
-6.1604671	10.0528002	101.4599991
-6.1604671	10.1016006	106.4199982
-6.1604671	10.1504002	104.7900009
-6.1604671	10.1992006	103.2999954
-6.1604671	10.2480001	102.3799973
-6.1604671	10.2968006	101.1899948
-6.1604671	10.3456001	103.1899948
-6.1604671	10.3944006	103.9199982
-6.1604671	10.4432001	101.7799988
-6.1604671	10.4920006	98.2699966
-6.1604671	10.5408001	102.0800018
-6.1604671	10.5896006	103.1299973
-6.1604671	10.6384001	102.6100006
-6.1604671	10.6872005	99.5299988
-6.1604671	10.7360001	104.1100006
-6.1604671	10.7848005	102.0299988
-6.1604671	10.8336000	103.8099976
-6.1604671	10.8824005	103.7799988
-6.1604671	10.9312000	102.5899963
-6.1604671	10.9800005	104.5299988
-6.1604671	11.0288000	103.0999985
-6.1604671	11.0776005	101.2799988
-6.1604671	11.1264000	97.1699982
-6.1604671	11.1752005	105.2600021
-6.1604671	11.2240000	107.0899963

-6.1604671	11.2728004	105.3300018
-6.1604671	11.3216000	105.2500000
-6.1604671	11.3704004	106.7299957
-6.1604671	11.4191999	105.6500015
-6.1604671	11.4680004	105.6100006
-6.1604671	11.5167999	106.0800018
-6.1604671	11.5656004	106.9199982
-6.1604671	11.6143999	105.5800018
-6.1604671	11.6632004	101.8300018
-6.1604671	11.7119999	104.1299973
-6.1604671	11.7608004	104.4499969
-6.1604671	11.8095999	104.5800018
-6.1604671	11.8584003	104.9499969
-6.1604671	11.9071999	103.9599991
-6.1604671	11.9560003	103.9000015
-6.1604671	12.0047998	103.4399948
-6.1604671	12.0536003	105.7799988
-6.1604671	12.1023998	104.4599991
-6.1604671	12.1512003	104.1199951
-6.1604671	12.1999998	103.6500015
-6.1604671	12.2488003	103.0499954
-6.1604671	12.2975998	105.3799973
-6.1604671	12.3464003	104.3799973
-6.1604671	12.3951998	102.8199997
-6.1604671	12.4440002	100.1599960
-6.1604671	12.4927998	104.1399994
-6.1604671	12.5416002	104.8600006
-6.1604671	12.5904007	100.9799957
-6.1604671	12.6392002	100.9099960
-6.1604671	12.6880007	103.6699982
-6.1604671	12.7368002	105.3499985
-6.1604671	12.7856007	106.6599960
-6.1604671	12.8344002	104.3199997
-6.1604671	12.8832006	100.9300003
-6.1604671	12.9320002	101.2500000
-6.1604671	12.9808006	104.7099991
-6.1604671	13.0296001	104.9000015
-6.1604671	13.0784006	100.6399994
-6.1604671	13.1272001	101.2699966
-6.1604671	13.1760006	103.0800018
-6.1604671	13.2248001	102.3499985
-6.1604671	13.2736006	101.6500015
-6.1604671	13.3224001	103.1500015
-6.1604671	13.3712006	104.1299973
-6.1604671	13.4200001	103.7900009
-6.1604671	13.4688005	103.7999954
-6.1604671	13.5176001	98.5599976
-6.1604671	13.5664005	103.6699982
-6.1604671	13.6152000	104.9499969
-6.1604671	13.6640005	105.1399994
-6.1604671	13.7128000	106.2399979
-6.1604671	13.7616005	105.6800003
-6.1604671	13.8104000	101.9399948
-6.1604671	13.8592005	100.8899994
-6.1604671	13.9080000	97.7999954
-6.1604671	13.9568005	102.2799988
-6.1604671	14.0056000	102.4599991
-6.1604671	14.0544004	101.4099960

-6.1604671	14.1032000	97.4300003
-6.1604671	14.1520004	101.7299957
-6.1604671	14.2007999	102.9899979
-6.1604671	14.2496004	102.2099991
-6.1604671	14.2983999	103.7500000
-6.1604671	14.3472004	105.9700012
-6.1604671	14.3959999	102.6800003
-6.1604671	14.4448004	99.0299988
-6.1604671	14.4935999	100.7900009
-6.1604671	14.5424004	99.3899994
-6.1604671	14.5911999	100.4300003
-6.1604671	14.6400003	103.3699951
-6.1604671	14.6887999	103.5899963
-6.1604671	14.7376003	100.4300003
-6.1604671	14.7863998	99.5400009
-6.1604671	14.8352003	102.0899963
-6.1604671	14.8839998	100.8199997
-6.1604671	14.9328003	103.2500000
-6.1604671	14.9815998	102.7399979
-6.1604671	15.0304003	105.3300018
-6.1543312	0.3416000	110.0299988
-6.1543312	0.3904000	108.9399948
-6.1543312	0.4392000	108.6699982
-6.1543312	0.4880000	107.0000000
-6.1543312	0.5368000	104.9199982
-6.1543312	0.5856000	105.3600006
-6.1543312	0.6344000	104.7500000
-6.1543312	0.6832000	107.4399948
-6.1543312	0.7320000	104.5400009
-6.1543312	0.7808000	107.7399979
-6.1543312	0.8296000	108.0199966
-6.1543312	0.8784000	107.2600021
-6.1543312	0.9272000	105.5699997
-6.1543312	0.9760000	105.8699951
-6.1543312	1.0247999	106.1800003
-6.1543312	1.0735999	108.6899948
-6.1543312	1.1223999	110.0599976
-6.1543312	1.1711999	105.4099960
-6.1543312	1.2200000	103.6999969
-6.1543312	1.2688000	101.7200012
-6.1543312	1.3176000	102.7500000
-6.1543312	1.3664000	105.0100021
-6.1543312	1.4152000	108.1299973
-6.1543312	1.4640000	108.6399994
-6.1543312	1.5128000	107.4599991
-6.1543312	1.5616000	108.2299957
-6.1543312	1.6104000	109.3399963
-6.1543312	1.6592000	109.4399948
-6.1543312	1.7079999	112.3899994
-6.1543312	1.7567999	111.7799988
-6.1543312	1.8055999	110.4199982
-6.1543312	1.8543999	117.0400009
-6.1543312	1.9031999	117.5999985
-6.1543312	1.9519999	111.8099976
-6.1543312	2.0007999	104.1399994
-6.1543312	2.0495999	106.8399963
-6.1543312	2.0983999	106.7399979
-6.1543312	2.1471999	102.9899979

-6.1543312	2.1959999	100.4199982
-6.1543312	2.2447999	104.8799973
-6.1543312	2.2935998	105.7699966
-6.1543312	2.3423998	103.6599960
-6.1543312	2.3912001	106.0100021
-6.1543312	2.4400001	104.1800003
-6.1543312	2.4888000	106.0100021
-6.1543312	2.5376000	108.6999969
-6.1543312	2.5864000	108.5400009
-6.1543312	2.6352000	105.9300003
-6.1543312	2.6840000	102.4899979
-6.1543312	2.7328000	105.2699966
-6.1543312	2.7816000	105.6199951
-6.1543312	2.8304000	106.5199966
-6.1543312	2.8792000	105.0400009
-6.1543312	2.9280000	104.8499985
-6.1543312	2.9768000	108.3099976
-6.1543312	3.0256000	107.3399963
-6.1543312	3.0743999	106.6399994
-6.1543312	3.1231999	106.5800018
-6.1543312	3.1719999	108.5999985
-6.1543312	3.2207999	106.8399963
-6.1543312	3.2695999	106.8399963
-6.1543312	3.3183999	106.4700012
-6.1543312	3.3671999	102.5899963
-6.1543312	3.4159999	99.8699951
-6.1543312	3.4647999	104.8399963
-6.1543312	3.5135999	105.7399979
-6.1543312	3.5623999	104.8899994
-6.1543312	3.6111999	104.3099976
-6.1543312	3.6599998	101.3499985
-6.1543312	3.7087998	103.0800018
-6.1543312	3.7575998	103.7600021
-6.1543312	3.8063998	103.7799988
-6.1543312	3.8551998	105.7200012
-6.1543312	3.9039998	104.0299988
-6.1543312	3.9527998	95.6299973
-6.1543312	4.0016003	101.5899963
-6.1543312	4.0503998	104.5599976
-6.1543312	4.0991998	106.4899979
-6.1543312	4.1479998	105.8399963
-6.1543312	4.1967998	102.3600006
-6.1543312	4.2455997	103.4599991
-6.1543312	4.2943997	109.0100021
-6.1543312	4.3431997	113.3300018
-6.1543312	4.3919997	110.0400009
-6.1543312	4.4407997	109.6100006
-6.1543312	4.4895997	113.0800018
-6.1543312	4.5383997	117.0499954
-6.1543312	4.5871997	118.8899994
-6.1543312	4.6359997	117.2399979
-6.1543312	4.6847997	115.4499969
-6.1543312	4.7336001	114.2600021
-6.1543312	4.7824001	110.2799988
-6.1543312	4.8312001	110.7500000
-6.1543312	4.8800001	113.3699951
-6.1543312	4.9288001	112.9899979
-6.1543312	4.9776001	109.1800003

-6.1543312	5.0264001	104.4599991
-6.1543312	5.0752001	105.0400009
-6.1543312	5.1240001	105.4399948
-6.1543312	5.1728001	105.7999954
-6.1543312	5.2216001	105.6800003
-6.1543312	5.2704000	108.7900009
-6.1543312	5.3192000	108.5100021
-6.1543312	5.3680000	106.1100006
-6.1543312	5.4168000	104.8899994
-6.1543312	5.4656000	104.3300018
-6.1543312	5.5144000	102.6599960
-6.1543312	5.5632000	104.7099991
-6.1543312	5.6120000	103.0699997
-6.1543312	5.6608000	98.3799973
-6.1543312	5.7096000	103.3499985
-6.1543312	5.7584000	106.1599960
-6.1543312	5.8072000	104.7999954
-6.1543312	5.8559999	104.4000015
-6.1543312	5.9047999	104.7399979
-6.1543312	5.9535999	104.2399979
-6.1543312	6.0023999	101.2999954
-6.1543312	6.0511999	105.7699966
-6.1543312	6.0999999	107.0000000
-6.1543312	6.1487999	106.5400009
-6.1543312	6.1975999	107.2299957
-6.1543312	6.2463999	105.5299988
-6.1543312	6.2951999	103.9399948
-6.1543312	6.3439999	103.8499985
-6.1543312	6.3927999	106.6500015
-6.1543312	6.4415998	109.8399963
-6.1543312	6.4903998	109.2799988
-6.1543312	6.5391998	106.6500015
-6.1543312	6.5879998	104.7099991
-6.1543312	6.6367998	104.6699982
-6.1543312	6.6855998	104.8899994
-6.1543312	6.7343998	104.9300003
-6.1543312	6.7831998	99.9099960
-6.1543312	6.8319998	99.2999954
-6.1543312	6.8807998	106.2500000
-6.1543312	6.9295998	106.6599960
-6.1543312	6.9783998	104.4899979
-6.1543312	7.0271997	102.1100006
-6.1543312	7.0759997	103.9599991
-6.1543312	7.1247997	102.9399948
-6.1543312	7.1735997	103.8099976
-6.1543312	7.2223997	104.9499969
-6.1543312	7.2711997	104.0800018
-6.1543312	7.3199997	103.0400009
-6.1543312	7.3687997	103.2699966
-6.1543312	7.4175997	102.6199951
-6.1543312	7.4663997	103.7600021
-6.1543312	7.5151997	104.2699966
-6.1543312	7.5639997	101.6500015
-6.1543312	7.6127996	100.5599976
-6.1543312	7.6615996	103.7399979
-6.1543312	7.7103996	105.0800018
-6.1543312	7.7591996	102.7399979
-6.1543312	7.8079996	100.8399963

-6.1543312	7.8567996	100.5999985
-6.1543312	7.9056001	100.6599960
-6.1543312	7.9544001	104.5599976
-6.1543312	8.0032005	106.9799957
-6.1543312	8.0520000	106.3699951
-6.1543312	8.1008005	104.1100006
-6.1543312	8.1496000	104.4199982
-6.1543312	8.1984005	104.8799973
-6.1543312	8.2472000	104.6500015
-6.1543312	8.2960005	104.2699966
-6.1543312	8.3448000	104.3199997
-6.1543312	8.3936005	101.4099960
-6.1543312	8.4424000	99.6899948
-6.1543312	8.4912004	104.3300018
-6.1543312	8.5400000	102.5899963
-6.1543312	8.5888004	98.8899994
-6.1543312	8.6375999	102.8699951
-6.1543312	8.6864004	105.5400009
-6.1543312	8.7351999	106.5499954
-6.1543312	8.7840004	103.4599991
-6.1543312	8.8327999	98.7099991
-6.1543312	8.8816004	101.1599960
-6.1543312	8.9303999	104.1299973
-6.1543312	8.9792004	104.1500015
-6.1543312	9.0279999	103.3300018
-6.1543312	9.0768003	105.9799957
-6.1543312	9.1255999	107.7399979
-6.1543312	9.1744003	105.0699997
-6.1543312	9.2231998	101.4000015
-6.1543312	9.2720003	105.5199966
-6.1543312	9.3207998	105.1899948
-6.1543312	9.3696003	105.2200012
-6.1543312	9.4184008	102.3600006
-6.1543312	9.4672003	100.7500000
-6.1543312	9.5160007	103.6699982
-6.1543312	9.5648003	103.0199966
-6.1543312	9.6136007	100.0299988
-6.1543312	9.6624002	103.3399963
-6.1543312	9.7112007	104.2500000
-6.1543312	9.7600002	103.7600021
-6.1543312	9.8088007	105.1199951
-6.1543312	9.8576002	103.1100006
-6.1543312	9.9064007	105.0599976
-6.1543312	9.9552002	103.5999985
-6.1543312	10.0040007	99.6399994
-6.1543312	10.0528002	101.7200012
-6.1543312	10.1016006	106.5800018
-6.1543312	10.1504002	105.5999985
-6.1543312	10.1992006	102.5100021
-6.1543312	10.2480001	101.3399963
-6.1543312	10.2968006	101.0499954
-6.1543312	10.3456001	103.4799957
-6.1543312	10.3944006	104.5999985
-6.1543312	10.4432001	102.6800003
-6.1543312	10.4920006	98.6100006
-6.1543312	10.5408001	101.9499969
-6.1543312	10.5896006	102.6999969
-6.1543312	10.6384001	102.6500015

-6.1543312	10.6872005	101.1199951
-6.1543312	10.7360001	104.9599991
-6.1543312	10.7848005	101.4199982
-6.1543312	10.8336000	104.2999954
-6.1543312	10.8824005	104.2200012
-6.1543312	10.9312000	102.1100006
-6.1543312	10.9800005	104.6699982
-6.1543312	11.0288000	101.3300018
-6.1543312	11.0776005	101.1500015
-6.1543312	11.1264000	97.2799988
-6.1543312	11.1752005	105.2500000
-6.1543312	11.2240000	106.5899963
-6.1543312	11.2728004	104.7999954
-6.1543312	11.3216000	104.6100006
-6.1543312	11.3704004	106.7399979
-6.1543312	11.4191999	106.1599960
-6.1543312	11.4680004	105.7299957
-6.1543312	11.5167999	105.4099960
-6.1543312	11.5656004	105.9199982
-6.1543312	11.6143999	105.0899963
-6.1543312	11.6632004	101.3300018
-6.1543312	11.7119999	103.4499969
-6.1543312	11.7608004	103.5699997
-6.1543312	11.8095999	103.6999969
-6.1543312	11.8584003	104.7900009
-6.1543312	11.9071999	104.2299957
-6.1543312	11.9560003	103.5000000
-6.1543312	12.0047998	103.2900009
-6.1543312	12.0536003	106.1699982
-6.1543312	12.1023998	105.4300003
-6.1543312	12.1512003	104.9099960
-6.1543312	12.1999998	104.5999985
-6.1543312	12.2488003	103.8600006
-6.1543312	12.2975998	106.5199966
-6.1543312	12.3464003	105.2699966
-6.1543312	12.3951998	102.0699997
-6.1543312	12.4440002	98.4799957
-6.1543312	12.4927998	102.8099976
-6.1543312	12.5416002	104.0299988
-6.1543312	12.5904007	100.7600021
-6.1543312	12.6392002	99.7699966
-6.1543312	12.6880007	103.4199982
-6.1543312	12.7368002	105.5100021
-6.1543312	12.7856007	105.5899963
-6.1543312	12.8344002	102.8199997
-6.1543312	12.8832006	100.8600006
-6.1543312	12.9320002	101.8099976
-6.1543312	12.9808006	104.5800018
-6.1543312	13.0296001	103.9300003
-6.1543312	13.0784006	99.3600006
-6.1543312	13.1272001	100.5599976
-6.1543312	13.1760006	102.9300003
-6.1543312	13.2248001	103.5899963
-6.1543312	13.2736006	102.4199982
-6.1543312	13.3224001	103.4700012
-6.1543312	13.3712006	104.9399948
-6.1543312	13.4200001	103.3600006
-6.1543312	13.4688005	103.1800003

-6.1543312	13.5176001	99.5999985
-6.1543312	13.5664005	102.9700012
-6.1543312	13.6152000	104.6500015
-6.1543312	13.6640005	105.8199997
-6.1543312	13.7128000	106.2299957
-6.1543312	13.7616005	105.1500015
-6.1543312	13.8104000	100.7999954
-6.1543312	13.8592005	99.9899979
-6.1543312	13.9080000	96.6299973
-6.1543312	13.9568005	101.7299957
-6.1543312	14.0056000	102.5899963
-6.1543312	14.0544004	101.6299973
-6.1543312	14.1032000	95.8799973
-6.1543312	14.1520004	100.7799988
-6.1543312	14.2007999	103.1699982
-6.1543312	14.2496004	102.1800003
-6.1543312	14.2983999	104.4099960
-6.1543312	14.3472004	106.4300003
-6.1543312	14.3959999	102.6399994
-6.1543312	14.4448004	98.7600021
-6.1543312	14.4935999	101.1500015
-6.1543312	14.5424004	100.1899948
-6.1543312	14.5911999	101.6699982
-6.1543312	14.6400003	103.7299957
-6.1543312	14.6887999	104.1299973
-6.1543312	14.7376003	101.2999954
-6.1543312	14.7863998	99.9599991
-6.1543312	14.8352003	102.4000015
-6.1543312	14.8839998	101.5299988
-6.1543312	14.9328003	103.4899979
-6.1543312	14.9815998	103.1399994
-6.1543312	15.0304003	105.2600021
-6.1481953	0.3416000	109.2900009
-6.1481953	0.3904000	108.2699966
-6.1481953	0.4392000	108.2999954
-6.1481953	0.4880000	106.4399948
-6.1481953	0.5368000	103.8499985
-6.1481953	0.5856000	105.3099976
-6.1481953	0.6344000	105.1199951
-6.1481953	0.6832000	107.2099991
-6.1481953	0.7320000	106.3099976
-6.1481953	0.7808000	107.8899994
-6.1481953	0.8296000	106.9300003
-6.1481953	0.8784000	106.4300003
-6.1481953	0.9272000	106.1399994
-6.1481953	0.9760000	106.6299973
-6.1481953	1.0247999	106.6500015
-6.1481953	1.0735999	109.2099991
-6.1481953	1.1223999	110.1399994
-6.1481953	1.1711999	105.1500015
-6.1481953	1.2200000	104.0000000
-6.1481953	1.2688000	100.8799973
-6.1481953	1.3176000	102.8799973
-6.1481953	1.3664000	104.9000015
-6.1481953	1.4152000	107.4000015
-6.1481953	1.4640000	107.5199966
-6.1481953	1.5128000	106.7099991
-6.1481953	1.5616000	108.1199951

-6.1481953	1.6104000	109.0999985
-6.1481953	1.6592000	109.1100006
-6.1481953	1.7079999	112.0100021
-6.1481953	1.7567999	111.7900009
-6.1481953	1.8055999	110.6500015
-6.1481953	1.8543999	117.0599976
-6.1481953	1.9031999	117.7099991
-6.1481953	1.9519999	112.1299973
-6.1481953	2.0007999	104.0100021
-6.1481953	2.0495999	106.8399963
-6.1481953	2.0983999	107.4099960
-6.1481953	2.1471999	103.5499954
-6.1481953	2.1959999	101.3099976
-6.1481953	2.2447999	105.1100006
-6.1481953	2.2935998	105.1199951
-6.1481953	2.3423998	103.1599960
-6.1481953	2.3912001	106.4899979
-6.1481953	2.4400001	105.0000000
-6.1481953	2.4888000	106.3399963
-6.1481953	2.5376000	108.4799957
-6.1481953	2.5864000	108.2799988
-6.1481953	2.6352000	105.7299957
-6.1481953	2.6840000	101.0699997
-6.1481953	2.7328000	105.0100021
-6.1481953	2.7816000	105.5299988
-6.1481953	2.8304000	105.9099960
-6.1481953	2.8792000	104.5100021
-6.1481953	2.9280000	105.4199982
-6.1481953	2.9768000	107.8099976
-6.1481953	3.0256000	106.3399963
-6.1481953	3.0743999	106.8499985
-6.1481953	3.1231999	107.1800003
-6.1481953	3.1719999	108.5899963
-6.1481953	3.2207999	106.6399994
-6.1481953	3.2695999	106.8899994
-6.1481953	3.3183999	105.9700012
-6.1481953	3.3671999	101.8600006
-6.1481953	3.4159999	101.2600021
-6.1481953	3.4647999	105.0499954
-6.1481953	3.5135999	105.9799957
-6.1481953	3.5623999	104.6800003
-6.1481953	3.6111999	104.0899963
-6.1481953	3.6599998	102.0100021
-6.1481953	3.7087998	103.0699997
-6.1481953	3.7575998	103.6599960
-6.1481953	3.8063998	103.8699951
-6.1481953	3.8551998	105.6899948
-6.1481953	3.9039998	103.1999969
-6.1481953	3.9527998	96.1999969
-6.1481953	4.0016003	101.7600021
-6.1481953	4.0503998	104.7500000
-6.1481953	4.0991998	106.4899979
-6.1481953	4.1479998	106.9300003
-6.1481953	4.1967998	103.5299988
-6.1481953	4.2455997	103.3199997
-6.1481953	4.2943997	108.7799988
-6.1481953	4.3431997	113.4300003
-6.1481953	4.3919997	110.7999954

-6.1481953	4.4407997	110.0699997
-6.1481953	4.4895997	113.0800018
-6.1481953	4.5383997	116.9899979
-6.1481953	4.5871997	118.7999954
-6.1481953	4.6359997	117.0699997
-6.1481953	4.6847997	114.7600021
-6.1481953	4.7336001	113.5299988
-6.1481953	4.7824001	109.7600021
-6.1481953	4.8312001	110.2600021
-6.1481953	4.8800001	113.0499954
-6.1481953	4.9288001	112.6399994
-6.1481953	4.9776001	109.7099991
-6.1481953	5.0264001	104.6899948
-6.1481953	5.0752001	105.5199966
-6.1481953	5.1240001	106.4399948
-6.1481953	5.1728001	106.5699997
-6.1481953	5.2216001	105.5800018
-6.1481953	5.2704000	107.9300003
-6.1481953	5.3192000	107.9099960
-6.1481953	5.3680000	105.6399994
-6.1481953	5.4168000	104.1599960
-6.1481953	5.4656000	103.4000015
-6.1481953	5.5144000	100.6500015
-6.1481953	5.5632000	104.1399994
-6.1481953	5.6120000	102.2200012
-6.1481953	5.6608000	99.3899994
-6.1481953	5.7096000	104.5199966
-6.1481953	5.7584000	107.2200012
-6.1481953	5.8072000	105.2200012
-6.1481953	5.8559999	104.0699997
-6.1481953	5.9047999	104.5000000
-6.1481953	5.9535999	104.4300003
-6.1481953	6.0023999	101.8399963
-6.1481953	6.0511999	106.2900009
-6.1481953	6.0999999	107.3499985
-6.1481953	6.1487999	107.1100006
-6.1481953	6.1975999	107.5800018
-6.1481953	6.2463999	105.3499985
-6.1481953	6.2951999	104.6599960
-6.1481953	6.3439999	103.7699966
-6.1481953	6.3927999	105.5899963
-6.1481953	6.4415998	109.2399979
-6.1481953	6.4903998	109.1399994
-6.1481953	6.5391998	106.9199982
-6.1481953	6.5879998	104.9300003
-6.1481953	6.6367998	104.7099991
-6.1481953	6.6855998	104.9000015
-6.1481953	6.7343998	104.7299957
-6.1481953	6.7831998	99.0699997
-6.1481953	6.8319998	99.5800018
-6.1481953	6.8807998	105.6800003
-6.1481953	6.9295998	106.1599960
-6.1481953	6.9783998	105.1299973
-6.1481953	7.0271997	102.6100006
-6.1481953	7.0759997	104.4700012
-6.1481953	7.1247997	103.0400009
-6.1481953	7.1735997	103.6299973
-6.1481953	7.2223997	105.5100021

-6.1481953	7.2711997	104.9899979
-6.1481953	7.3199997	103.3399963
-6.1481953	7.3687997	103.2699966
-6.1481953	7.4175997	101.8799973
-6.1481953	7.4663997	103.4300003
-6.1481953	7.5151997	104.2399979
-6.1481953	7.5639997	100.9799957
-6.1481953	7.6127996	100.5899963
-6.1481953	7.6615996	103.5899963
-6.1481953	7.7103996	105.4300003
-6.1481953	7.7591996	101.9799957
-6.1481953	7.8079996	100.5400009
-6.1481953	7.8567996	100.7299957
-6.1481953	7.9056001	102.2500000
-6.1481953	7.9544001	104.9799957
-6.1481953	8.0032005	106.6699982
-6.1481953	8.0520000	106.4199982
-6.1481953	8.1008005	104.2500000
-6.1481953	8.1496000	104.8099976
-6.1481953	8.1984005	105.0100021
-6.1481953	8.2472000	103.8099976
-6.1481953	8.2960005	103.9000015
-6.1481953	8.3448000	103.7099991
-6.1481953	8.3936005	101.5299988
-6.1481953	8.4424000	99.4899979
-6.1481953	8.4912004	104.5899963
-6.1481953	8.5400000	103.7999954
-6.1481953	8.5888004	100.1399994
-6.1481953	8.6375999	103.2699966
-6.1481953	8.6864004	105.5999985
-6.1481953	8.7351999	106.4499969
-6.1481953	8.7840004	102.9399948
-6.1481953	8.8327999	98.3499985
-6.1481953	8.8816004	101.9399948
-6.1481953	8.9303999	103.5499954
-6.1481953	8.9792004	103.0999985
-6.1481953	9.0279999	103.0999985
-6.1481953	9.0768003	106.1199951
-6.1481953	9.1255999	107.9099960
-6.1481953	9.1744003	105.2600021
-6.1481953	9.2231998	100.7200012
-6.1481953	9.2720003	105.5400009
-6.1481953	9.3207998	106.0599976
-6.1481953	9.3696003	105.0599976
-6.1481953	9.4184008	102.5299988
-6.1481953	9.4672003	101.8099976
-6.1481953	9.5160007	103.6999969
-6.1481953	9.5648003	102.7600021
-6.1481953	9.6136007	100.6599960
-6.1481953	9.6624002	103.6100006
-6.1481953	9.7112007	104.0499954
-6.1481953	9.7600002	104.8399963
-6.1481953	9.8088007	105.7299957
-6.1481953	9.8576002	103.5499954
-6.1481953	9.9064007	106.0499954
-6.1481953	9.9552002	104.7399979
-6.1481953	10.0040007	100.5499954
-6.1481953	10.0528002	102.0800018

-6.1481953	10.1016006	105.8899994
-6.1481953	10.1504002	105.6399994
-6.1481953	10.1992006	101.3300018
-6.1481953	10.2480001	100.1899948
-6.1481953	10.2968006	101.6999969
-6.1481953	10.3456001	104.1199951
-6.1481953	10.3944006	105.2399979
-6.1481953	10.4432001	103.5599976
-6.1481953	10.4920006	100.0199966
-6.1481953	10.5408001	102.2900009
-6.1481953	10.5896006	102.6899948
-6.1481953	10.6384001	102.7699966
-6.1481953	10.6872005	102.2099991
-6.1481953	10.7360001	105.2600021
-6.1481953	10.7848005	100.7299957
-6.1481953	10.8336000	104.8199997
-6.1481953	10.8824005	104.6399994
-6.1481953	10.9312000	101.6100006
-6.1481953	10.9800005	103.6399994
-6.1481953	11.0288000	99.1299973
-6.1481953	11.0776005	100.8799973
-6.1481953	11.1264000	97.7500000
-6.1481953	11.1752005	105.1599960
-6.1481953	11.2240000	106.1100006
-6.1481953	11.2728004	104.2299957
-6.1481953	11.3216000	103.9599991
-6.1481953	11.3704004	107.0499954
-6.1481953	11.4191999	106.9899979
-6.1481953	11.4680004	106.1299973
-6.1481953	11.5167999	105.0599976
-6.1481953	11.5656004	104.6299973
-6.1481953	11.6143999	104.3699951
-6.1481953	11.6632004	100.4099960
-6.1481953	11.7119999	102.6399994
-6.1481953	11.7608004	103.3099976
-6.1481953	11.8095999	103.1699982
-6.1481953	11.8584003	104.5800018
-6.1481953	11.9071999	104.0400009
-6.1481953	11.9560003	102.3399963
-6.1481953	12.0047998	102.6399994
-6.1481953	12.0536003	106.0699997
-6.1481953	12.1023998	105.9399948
-6.1481953	12.1512003	105.4499969
-6.1481953	12.1999998	105.1899948
-6.1481953	12.2488003	104.1800003
-6.1481953	12.2975998	107.5499954
-6.1481953	12.3464003	106.3699951
-6.1481953	12.3951998	102.0100021
-6.1481953	12.4440002	99.0299988
-6.1481953	12.4927998	102.4499969
-6.1481953	12.5416002	103.7600021
-6.1481953	12.5904007	101.0499954
-6.1481953	12.6392002	99.6199951
-6.1481953	12.6880007	104.4099960
-6.1481953	12.7368002	106.0699997
-6.1481953	12.7856007	104.7200012
-6.1481953	12.8344002	101.5000000
-6.1481953	12.8832006	100.3699951

-6.1481953	12.9320002	102.0199966
-6.1481953	12.9808006	104.1500015
-6.1481953	13.0296001	103.1899948
-6.1481953	13.0784006	99.5000000
-6.1481953	13.1272001	100.0599976
-6.1481953	13.1760006	102.1399994
-6.1481953	13.2248001	103.4799957
-6.1481953	13.2736006	102.9199982
-6.1481953	13.3224001	104.0599976
-6.1481953	13.3712006	105.8799973
-6.1481953	13.4200001	104.0599976
-6.1481953	13.4688005	103.9700012
-6.1481953	13.5176001	101.5199966
-6.1481953	13.5664005	102.6500015
-6.1481953	13.6152000	104.0100021
-6.1481953	13.6640005	105.5599976
-6.1481953	13.7128000	105.3199997
-6.1481953	13.7616005	103.9300003
-6.1481953	13.8104000	99.8199997
-6.1481953	13.8592005	99.1800003
-6.1481953	13.9080000	96.8600006
-6.1481953	13.9568005	101.0400009
-6.1481953	14.0056000	102.1500015
-6.1481953	14.0544004	101.5699997
-6.1481953	14.1032000	95.4000015
-6.1481953	14.1520004	100.5000000
-6.1481953	14.2007999	103.3499985
-6.1481953	14.2496004	102.2500000
-6.1481953	14.2983999	105.0899963
-6.1481953	14.3472004	106.3899994
-6.1481953	14.3959999	102.4799957
-6.1481953	14.4448004	99.2299957
-6.1481953	14.4935999	101.6299973
-6.1481953	14.5424004	101.7200012
-6.1481953	14.5911999	102.9599991
-6.1481953	14.6400003	104.0199966
-6.1481953	14.6887999	104.4499969
-6.1481953	14.7376003	102.0100021
-6.1481953	14.7863998	100.3399963
-6.1481953	14.8352003	102.5400009
-6.1481953	14.8839998	101.6399994
-6.1481953	14.9328003	103.4099960
-6.1481953	14.9815998	103.5499954
-6.1481953	15.0304003	104.7900009
-6.1420593	0.3416000	108.0199966
-6.1420593	0.3904000	107.2999954
-6.1420593	0.4392000	107.6199951
-6.1420593	0.4880000	105.8799973
-6.1420593	0.5368000	103.2799988
-6.1420593	0.5856000	104.9599991
-6.1420593	0.6344000	104.5199966
-6.1420593	0.6832000	107.1399994
-6.1420593	0.7320000	107.7299957
-6.1420593	0.7808000	108.0000000
-6.1420593	0.8296000	106.2799988
-6.1420593	0.8784000	106.5100021
-6.1420593	0.9272000	107.5299988
-6.1420593	0.9760000	108.0100021

-6.1420593	1.0247999	107.5699997
-6.1420593	1.0735999	109.1100006
-6.1420593	1.1223999	109.7500000
-6.1420593	1.1711999	104.5000000
-6.1420593	1.2200000	104.6399994
-6.1420593	1.2688000	101.5699997
-6.1420593	1.3176000	104.1500015
-6.1420593	1.3664000	105.7999954
-6.1420593	1.4152000	107.1800003
-6.1420593	1.4640000	106.7999954
-6.1420593	1.5128000	105.8799973
-6.1420593	1.5616000	107.0400009
-6.1420593	1.6104000	108.2699966
-6.1420593	1.6592000	108.2600021
-6.1420593	1.7079999	111.2799988
-6.1420593	1.7567999	111.5100021
-6.1420593	1.8055999	111.4700012
-6.1420593	1.8543999	118.3099976
-6.1420593	1.9031999	119.3099976
-6.1420593	1.9519999	114.2200012
-6.1420593	2.0007999	104.6800003
-6.1420593	2.0495999	107.0800018
-6.1420593	2.0983999	108.3399963
-6.1420593	2.1471999	104.7200012
-6.1420593	2.1959999	102.1800003
-6.1420593	2.2447999	105.2200012
-6.1420593	2.2935998	104.5299988
-6.1420593	2.3423998	103.2099991
-6.1420593	2.3912001	106.8199997
-6.1420593	2.4400001	105.1699982
-6.1420593	2.4888000	106.9000015
-6.1420593	2.5376000	108.4899979
-6.1420593	2.5864000	107.9099960
-6.1420593	2.6352000	106.0499954
-6.1420593	2.6840000	101.0299988
-6.1420593	2.7328000	104.4399948
-6.1420593	2.7816000	104.9300003
-6.1420593	2.8304000	105.4599991
-6.1420593	2.8792000	104.2799988
-6.1420593	2.9280000	105.9599991
-6.1420593	2.9768000	107.7799988
-6.1420593	3.0256000	105.7699966
-6.1420593	3.0743999	106.8399963
-6.1420593	3.1231999	106.6100006
-6.1420593	3.1719999	107.6999969
-6.1420593	3.2207999	106.0000000
-6.1420593	3.2695999	106.4199982
-6.1420593	3.3183999	105.8099976
-6.1420593	3.3671999	102.3799973
-6.1420593	3.4159999	103.0499954
-6.1420593	3.4647999	105.1999969
-6.1420593	3.5135999	105.6399994
-6.1420593	3.5623999	103.9700012
-6.1420593	3.6111999	103.9300003
-6.1420593	3.6599998	102.5699997
-6.1420593	3.7087998	103.0499954
-6.1420593	3.7575998	103.5400009
-6.1420593	3.8063998	103.8699951

-6.1420593	3.8551998	105.5400009
-6.1420593	3.9039998	102.7999954
-6.1420593	3.9527998	97.7500000
-6.1420593	4.0016003	102.2099991
-6.1420593	4.0503998	104.8099976
-6.1420593	4.0991998	106.1399994
-6.1420593	4.1479998	107.2500000
-6.1420593	4.1967998	105.1199951
-6.1420593	4.2455997	103.8899994
-6.1420593	4.2943997	108.4300003
-6.1420593	4.3431997	113.1800003
-6.1420593	4.3919997	111.5100021
-6.1420593	4.4407997	110.5800018
-6.1420593	4.4895997	113.0400009
-6.1420593	4.5383997	117.1599960
-6.1420593	4.5871997	119.0899963
-6.1420593	4.6359997	117.1899948
-6.1420593	4.6847997	115.2299957
-6.1420593	4.7336001	113.6699982
-6.1420593	4.7824001	110.1699982
-6.1420593	4.8312001	110.7299957
-6.1420593	4.8800001	113.0599976
-6.1420593	4.9288001	112.3099976
-6.1420593	4.9776001	110.2799988
-6.1420593	5.0264001	105.3600006
-6.1420593	5.0752001	106.7200012
-6.1420593	5.1240001	108.0999985
-6.1420593	5.1728001	107.3799973
-6.1420593	5.2216001	105.5499954
-6.1420593	5.2704000	106.8399963
-6.1420593	5.3192000	106.8399963
-6.1420593	5.3680000	105.0999985
-6.1420593	5.4168000	104.4300003
-6.1420593	5.4656000	103.2999954
-6.1420593	5.5144000	99.8899994
-6.1420593	5.5632000	103.5699997
-6.1420593	5.6120000	101.7399979
-6.1420593	5.6608000	100.6100006
-6.1420593	5.7096000	105.0299988
-6.1420593	5.7584000	107.7399979
-6.1420593	5.8072000	105.8699951
-6.1420593	5.8559999	104.4899979
-6.1420593	5.9047999	104.8799973
-6.1420593	5.9535999	104.7699966
-6.1420593	6.0023999	102.0999985
-6.1420593	6.0511999	106.5800018
-6.1420593	6.0999999	107.7099991
-6.1420593	6.1487999	107.7099991
-6.1420593	6.1975999	107.9099960
-6.1420593	6.2463999	105.6399994
-6.1420593	6.2951999	105.3699951
-6.1420593	6.3439999	104.0599976
-6.1420593	6.3927999	105.2799988
-6.1420593	6.4415998	108.4700012
-6.1420593	6.4903998	108.2900009
-6.1420593	6.5391998	106.6999969
-6.1420593	6.5879998	104.5699997
-6.1420593	6.6367998	104.1299973

-6.1420593	6.6855998	105.2099991
-6.1420593	6.7343998	104.7200012
-6.1420593	6.7831998	98.4899979
-6.1420593	6.8319998	100.5100021
-6.1420593	6.8807998	105.7500000
-6.1420593	6.9295998	106.5000000
-6.1420593	6.9783998	105.7399979
-6.1420593	7.0271997	102.3600006
-6.1420593	7.0759997	105.2099991
-6.1420593	7.1247997	103.7900009
-6.1420593	7.1735997	103.3199997
-6.1420593	7.2223997	105.6100006
-6.1420593	7.2711997	105.7099991
-6.1420593	7.3199997	103.8499985
-6.1420593	7.3687997	103.1500015
-6.1420593	7.4175997	102.7600021
-6.1420593	7.4663997	103.5599976
-6.1420593	7.5151997	104.2099991
-6.1420593	7.5639997	101.7999954
-6.1420593	7.6127996	100.9000015
-6.1420593	7.6615996	103.3699951
-6.1420593	7.7103996	105.7600021
-6.1420593	7.7591996	101.1999969
-6.1420593	7.8079996	100.1299973
-6.1420593	7.8567996	100.9700012
-6.1420593	7.9056001	103.4399948
-6.1420593	7.9544001	105.2200012
-6.1420593	8.0032005	106.2099991
-6.1420593	8.0520000	105.8499985
-6.1420593	8.1008005	103.7200012
-6.1420593	8.1496000	104.5400009
-6.1420593	8.1984005	104.5400009
-6.1420593	8.2472000	102.9899979
-6.1420593	8.2960005	103.5400009
-6.1420593	8.3448000	103.5400009
-6.1420593	8.3936005	102.1599960
-6.1420593	8.4424000	100.1800003
-6.1420593	8.4912004	105.0499954
-6.1420593	8.5400000	105.3600006
-6.1420593	8.5888004	102.2600021
-6.1420593	8.6375999	103.7699966
-6.1420593	8.6864004	105.4499969
-6.1420593	8.7351999	106.3499985
-6.1420593	8.7840004	102.5999985
-6.1420593	8.8327999	99.9499969
-6.1420593	8.8816004	103.0999985
-6.1420593	8.9303999	103.1999969
-6.1420593	8.9792004	102.0899963
-6.1420593	9.0279999	102.9199982
-6.1420593	9.0768003	105.5199966
-6.1420593	9.1255999	107.5299988
-6.1420593	9.1744003	105.3199997
-6.1420593	9.2231998	100.7200012
-6.1420593	9.2720003	104.8199997
-6.1420593	9.3207998	106.4499969
-6.1420593	9.3696003	104.4199982
-6.1420593	9.4184008	101.4599991
-6.1420593	9.4672003	102.2500000

-6.1420593	9.5160007	103.5100021
-6.1420593	9.5648003	103.0299988
-6.1420593	9.6136007	101.0499954
-6.1420593	9.6624002	103.9099960
-6.1420593	9.7112007	103.7500000
-6.1420593	9.7600002	105.5000000
-6.1420593	9.8088007	105.5199966
-6.1420593	9.8576002	103.6500015
-6.1420593	9.9064007	106.8899994
-6.1420593	9.9552002	106.0599976
-6.1420593	10.0040007	102.2600021
-6.1420593	10.0528002	102.8899994
-6.1420593	10.1016006	105.1100006
-6.1420593	10.1504002	105.3899994
-6.1420593	10.1992006	101.4000015
-6.1420593	10.2480001	100.2999954
-6.1420593	10.2968006	102.6599960
-6.1420593	10.3456001	104.4499969
-6.1420593	10.3944006	105.6399994
-6.1420593	10.4432001	104.6800003
-6.1420593	10.4920006	101.5100021
-6.1420593	10.5408001	103.1599960
-6.1420593	10.5896006	102.4399948
-6.1420593	10.6384001	102.1199951
-6.1420593	10.6872005	102.8300018
-6.1420593	10.7360001	105.0400009
-6.1420593	10.7848005	100.1399994
-6.1420593	10.8336000	104.7200012
-6.1420593	10.8824005	104.5299988
-6.1420593	10.9312000	101.6500015
-6.1420593	10.9800005	102.8399963
-6.1420593	11.0288000	96.9899979
-6.1420593	11.0776005	100.1800003
-6.1420593	11.1264000	99.0299988
-6.1420593	11.1752005	104.9499969
-6.1420593	11.2240000	105.5599976
-6.1420593	11.2728004	104.0400009
-6.1420593	11.3216000	103.7500000
-6.1420593	11.3704004	107.5699997
-6.1420593	11.4191999	108.1399994
-6.1420593	11.4680004	106.7699966
-6.1420593	11.5167999	105.1699982
-6.1420593	11.5656004	104.3499985
-6.1420593	11.6143999	104.1999969
-6.1420593	11.6632004	100.8399963
-6.1420593	11.7119999	102.2999954
-6.1420593	11.7608004	102.7799988
-6.1420593	11.8095999	102.4300003
-6.1420593	11.8584003	104.6299973
-6.1420593	11.9071999	103.6299973
-6.1420593	11.9560003	101.3199997
-6.1420593	12.0047998	103.2299957
-6.1420593	12.0536003	106.4899979
-6.1420593	12.1023998	105.9099960
-6.1420593	12.1512003	104.0400009
-6.1420593	12.1999998	105.1500015
-6.1420593	12.2488003	105.1599960
-6.1420593	12.2975998	108.4700012

-6.1420593	12.3464003	107.3799973
-6.1420593	12.3951998	102.9199982
-6.1420593	12.4440002	99.9099960
-6.1420593	12.4927998	102.1699982
-6.1420593	12.5416002	103.2500000
-6.1420593	12.5904007	101.2500000
-6.1420593	12.6392002	99.8799973
-6.1420593	12.6880007	105.1399994
-6.1420593	12.7368002	105.8499985
-6.1420593	12.7856007	103.3799973
-6.1420593	12.8344002	100.1599960
-6.1420593	12.8832006	100.1299973
-6.1420593	12.9320002	101.8399963
-6.1420593	12.9808006	103.7299957
-6.1420593	13.0296001	102.7399979
-6.1420593	13.0784006	100.2500000
-6.1420593	13.1272001	100.4499969
-6.1420593	13.1760006	101.3099976
-6.1420593	13.2248001	103.6299973
-6.1420593	13.2736006	102.7799988
-6.1420593	13.3224001	103.7500000
-6.1420593	13.3712006	106.0599976
-6.1420593	13.4200001	104.3899994
-6.1420593	13.4688005	104.3899994
-6.1420593	13.5176001	102.9700012
-6.1420593	13.5664005	102.2600021
-6.1420593	13.6152000	103.4499969
-6.1420593	13.6640005	105.1899948
-6.1420593	13.7128000	104.1699982
-6.1420593	13.7616005	102.2699966
-6.1420593	13.8104000	99.3499985
-6.1420593	13.8592005	98.7500000
-6.1420593	13.9080000	96.8600006
-6.1420593	13.9568005	100.7799988
-6.1420593	14.0056000	102.7299957
-6.1420593	14.0544004	102.4599991
-6.1420593	14.1032000	97.5299988
-6.1420593	14.1520004	100.8699951
-6.1420593	14.2007999	103.3600006
-6.1420593	14.2496004	102.0599976
-6.1420593	14.2983999	105.8600006
-6.1420593	14.3472004	106.5499954
-6.1420593	14.3959999	102.2099991
-6.1420593	14.4448004	99.0699997
-6.1420593	14.4935999	101.8600006
-6.1420593	14.5424004	102.8799973
-6.1420593	14.5911999	103.6599960
-6.1420593	14.6400003	104.0499954
-6.1420593	14.6887999	104.3600006
-6.1420593	14.7376003	102.6399994
-6.1420593	14.7863998	101.1299973
-6.1420593	14.8352003	102.8799973
-6.1420593	14.8839998	102.4899979
-6.1420593	14.9328003	103.4499969
-6.1420593	14.9815998	103.9799957
-6.1420593	15.0304003	104.3300018
-6.1359234	0.3416000	106.7699966
-6.1359234	0.3904000	106.9199982

-6.1359234	0.4392000	107.5999985
-6.1359234	0.4880000	105.8099976
-6.1359234	0.5368000	102.7900009
-6.1359234	0.5856000	105.0000000
-6.1359234	0.6344000	103.7600021
-6.1359234	0.6832000	106.7500000
-6.1359234	0.7320000	108.9000015
-6.1359234	0.7808000	108.0400009
-6.1359234	0.8296000	106.0000000
-6.1359234	0.8784000	107.1399994
-6.1359234	0.9272000	108.6199951
-6.1359234	0.9760000	109.1500015
-6.1359234	1.0247999	108.5100021
-6.1359234	1.0735999	109.0000000
-6.1359234	1.1223999	109.2799988
-6.1359234	1.1711999	104.5599976
-6.1359234	1.2200000	105.3600006
-6.1359234	1.2688000	103.0999985
-6.1359234	1.3176000	104.9499969
-6.1359234	1.3664000	105.6299973
-6.1359234	1.4152000	105.8099976
-6.1359234	1.4640000	105.7500000
-6.1359234	1.5128000	105.0699997
-6.1359234	1.5616000	105.8699951
-6.1359234	1.6104000	107.6100006
-6.1359234	1.6592000	107.9499969
-6.1359234	1.7079999	110.4300003
-6.1359234	1.7567999	110.9300003
-6.1359234	1.8055999	111.6599960
-6.1359234	1.8543999	119.0800018
-6.1359234	1.9031999	120.4300003
-6.1359234	1.9519999	115.6800003
-6.1359234	2.0007999	105.7799988
-6.1359234	2.0495999	107.3699951
-6.1359234	2.0983999	108.9099960
-6.1359234	2.1471999	105.5199966
-6.1359234	2.1959999	103.7500000
-6.1359234	2.2447999	105.4399948
-6.1359234	2.2935998	104.2699966
-6.1359234	2.3423998	103.0299988
-6.1359234	2.3912001	106.4199982
-6.1359234	2.4400001	105.3499985
-6.1359234	2.4888000	107.5999985
-6.1359234	2.5376000	108.2999954
-6.1359234	2.5864000	107.0199966
-6.1359234	2.6352000	105.3600006
-6.1359234	2.6840000	99.9899979
-6.1359234	2.7328000	103.1999969
-6.1359234	2.7816000	104.1100006
-6.1359234	2.8304000	105.0599976
-6.1359234	2.8792000	104.3199997
-6.1359234	2.9280000	106.5199966
-6.1359234	2.9768000	107.0499954
-6.1359234	3.0256000	104.9000015
-6.1359234	3.0743999	106.4700012
-6.1359234	3.1231999	105.4399948
-6.1359234	3.1719999	106.7699966
-6.1359234	3.2207999	105.2299957

-6.1359234	3.2695999	105.9700012
-6.1359234	3.3183999	105.8699951
-6.1359234	3.3671999	103.1199951
-6.1359234	3.4159999	104.5599976
-6.1359234	3.4647999	105.0100021
-6.1359234	3.5135999	105.2099991
-6.1359234	3.5623999	103.9499969
-6.1359234	3.6111999	104.1299973
-6.1359234	3.6599998	103.1699982
-6.1359234	3.7087998	102.8199997
-6.1359234	3.7575998	103.0000000
-6.1359234	3.8063998	103.3499985
-6.1359234	3.8551998	105.1800003
-6.1359234	3.9039998	102.8699951
-6.1359234	3.9527998	100.1599960
-6.1359234	4.0016003	103.2200012
-6.1359234	4.0503998	104.8099976
-6.1359234	4.0991998	105.7699966
-6.1359234	4.1479998	106.5599976
-6.1359234	4.1967998	105.8799973
-6.1359234	4.2455997	104.1199951
-6.1359234	4.2943997	108.6299973
-6.1359234	4.3431997	112.7999954
-6.1359234	4.3919997	111.3300018
-6.1359234	4.4407997	110.2399979
-6.1359234	4.4895997	112.0299988
-6.1359234	4.5383997	116.6999969
-6.1359234	4.5871997	119.1399994
-6.1359234	4.6359997	117.2399979
-6.1359234	4.6847997	115.6599960
-6.1359234	4.7336001	114.0199966
-6.1359234	4.7824001	110.6299973
-6.1359234	4.8312001	111.2999954
-6.1359234	4.8800001	113.3099976
-6.1359234	4.9288001	112.2900009
-6.1359234	4.9776001	110.9000015
-6.1359234	5.0264001	106.0999985
-6.1359234	5.0752001	108.3300018
-6.1359234	5.1240001	109.9899979
-6.1359234	5.1728001	108.3799973
-6.1359234	5.2216001	105.6999969
-6.1359234	5.2704000	105.6199951
-6.1359234	5.3192000	105.2200012
-6.1359234	5.3680000	104.2900009
-6.1359234	5.4168000	104.5699997
-6.1359234	5.4656000	103.9000015
-6.1359234	5.5144000	99.5899963
-6.1359234	5.5632000	103.0599976
-6.1359234	5.6120000	101.9099960
-6.1359234	5.6608000	102.5599976
-6.1359234	5.7096000	104.9000015
-6.1359234	5.7584000	107.0800018
-6.1359234	5.8072000	105.4099960
-6.1359234	5.8559999	104.6599960
-6.1359234	5.9047999	105.4700012
-6.1359234	5.9535999	105.4099960
-6.1359234	6.0023999	103.2699966
-6.1359234	6.0511999	106.9300003

-6.1359234	6.0999999	107.7600021
-6.1359234	6.1487999	107.7399979
-6.1359234	6.1975999	107.9599991
-6.1359234	6.2463999	105.4499969
-6.1359234	6.2951999	105.3499985
-6.1359234	6.3439999	104.3699951
-6.1359234	6.3927999	104.8399963
-6.1359234	6.4415998	107.7600021
-6.1359234	6.4903998	107.6699982
-6.1359234	6.5391998	106.9099960
-6.1359234	6.5879998	105.1800003
-6.1359234	6.6367998	104.0499954
-6.1359234	6.6855998	105.7600021
-6.1359234	6.7343998	105.0100021
-6.1359234	6.7831998	99.0999985
-6.1359234	6.8319998	101.8099976
-6.1359234	6.8807998	105.9599991
-6.1359234	6.9295998	106.8699951
-6.1359234	6.9783998	106.2900009
-6.1359234	7.0271997	101.4700012
-6.1359234	7.0759997	105.0199966
-6.1359234	7.1247997	104.0199966
-6.1359234	7.1735997	102.7900009
-6.1359234	7.2223997	105.5100021
-6.1359234	7.2711997	106.1999969
-6.1359234	7.3199997	104.5400009
-6.1359234	7.3687997	102.1699982
-6.1359234	7.4175997	102.6899948
-6.1359234	7.4663997	102.8300018
-6.1359234	7.5151997	103.6999969
-6.1359234	7.5639997	102.6599960
-6.1359234	7.6127996	102.0299988
-6.1359234	7.6615996	103.0299988
-6.1359234	7.7103996	104.7699966
-6.1359234	7.7591996	101.7099991
-6.1359234	7.8079996	100.7299957
-6.1359234	7.8567996	101.0199966
-6.1359234	7.9056001	104.0699997
-6.1359234	7.9544001	104.9499969
-6.1359234	8.0032005	105.1500015
-6.1359234	8.0520000	104.9300003
-6.1359234	8.1008005	102.9399948
-6.1359234	8.1496000	104.0899963
-6.1359234	8.1984005	104.6899948
-6.1359234	8.2472000	103.7900009
-6.1359234	8.2960005	103.9499969
-6.1359234	8.3448000	103.6899948
-6.1359234	8.3936005	103.0699997
-6.1359234	8.4424000	101.2500000
-6.1359234	8.4912004	104.9700012
-6.1359234	8.5400000	105.9000015
-6.1359234	8.5888004	103.8600006
-6.1359234	8.6375999	104.1800003
-6.1359234	8.6864004	105.2299957
-6.1359234	8.7351999	106.0100021
-6.1359234	8.7840004	102.9399948
-6.1359234	8.8327999	101.0100021
-6.1359234	8.8816004	103.8499985

-6.1359234	8.9303999	103.1100006
-6.1359234	8.9792004	102.1500015
-6.1359234	9.0279999	102.8799973
-6.1359234	9.0768003	104.4899979
-6.1359234	9.1255999	107.0699997
-6.1359234	9.1744003	105.6599960
-6.1359234	9.2231998	100.7900009
-6.1359234	9.2720003	103.7600021
-6.1359234	9.3207998	106.3499985
-6.1359234	9.3696003	103.4300003
-6.1359234	9.4184008	100.2600021
-6.1359234	9.4672003	101.8699951
-6.1359234	9.5160007	102.5499954
-6.1359234	9.5648003	103.1100006
-6.1359234	9.6136007	102.7699966
-6.1359234	9.6624002	104.5299988
-6.1359234	9.7112007	102.9799957
-6.1359234	9.7600002	106.1299973
-6.1359234	9.8088007	105.2399979
-6.1359234	9.8576002	102.9499969
-6.1359234	9.9064007	107.0400009
-6.1359234	9.9552002	106.7900009
-6.1359234	10.0040007	103.5199966
-6.1359234	10.0528002	102.8099976
-6.1359234	10.1016006	104.1299973
-6.1359234	10.1504002	104.4499969
-6.1359234	10.1992006	101.2799988
-6.1359234	10.2480001	100.9899979
-6.1359234	10.2968006	103.9899979
-6.1359234	10.3456001	105.0000000
-6.1359234	10.3944006	105.9499969
-6.1359234	10.4432001	105.2099991
-6.1359234	10.4920006	102.5999985
-6.1359234	10.5408001	103.9000015
-6.1359234	10.5896006	102.0899963
-6.1359234	10.6384001	101.4599991
-6.1359234	10.6872005	103.7299957
-6.1359234	10.7360001	104.8199997
-6.1359234	10.7848005	100.2500000
-6.1359234	10.8336000	104.2900009
-6.1359234	10.8824005	104.2799988
-6.1359234	10.9312000	102.2200012
-6.1359234	10.9800005	102.3199997
-6.1359234	11.0288000	95.8399963
-6.1359234	11.0776005	99.9799957
-6.1359234	11.1264000	100.3899994
-6.1359234	11.1752005	104.3600006
-6.1359234	11.2240000	104.6899948
-6.1359234	11.2728004	103.1399994
-6.1359234	11.3216000	104.5999985
-6.1359234	11.3704004	108.3499985
-6.1359234	11.4191999	109.0800018
-6.1359234	11.4680004	107.1100006
-6.1359234	11.5167999	105.1699982
-6.1359234	11.5656004	103.9899979
-6.1359234	11.6143999	103.5199966
-6.1359234	11.6632004	101.7299957
-6.1359234	11.7119999	103.3899994

-6.1359234	11.7608004	103.9300003
-6.1359234	11.8095999	103.1500015
-6.1359234	11.8584003	105.2399979
-6.1359234	11.9071999	103.1299973
-6.1359234	11.9560003	100.8499985
-6.1359234	12.0047998	103.7299957
-6.1359234	12.0536003	105.9799957
-6.1359234	12.1023998	106.0800018
-6.1359234	12.1512003	102.6899948
-6.1359234	12.1999998	104.7699966
-6.1359234	12.2488003	105.7799988
-6.1359234	12.2975998	108.7699966
-6.1359234	12.3464003	107.4799957
-6.1359234	12.3951998	103.0699997
-6.1359234	12.4440002	100.3499985
-6.1359234	12.4927998	102.4199982
-6.1359234	12.5416002	102.3499985
-6.1359234	12.5904007	100.9700012
-6.1359234	12.6392002	101.3699951
-6.1359234	12.6880007	105.8699951
-6.1359234	12.7368002	105.5000000
-6.1359234	12.7856007	102.2799988
-6.1359234	12.8344002	99.4799957
-6.1359234	12.8832006	100.8300018
-6.1359234	12.9320002	101.7299957
-6.1359234	12.9808006	103.5999985
-6.1359234	13.0296001	102.6800003
-6.1359234	13.0784006	100.6800003
-6.1359234	13.1272001	101.1599960
-6.1359234	13.1760006	100.8499985
-6.1359234	13.2248001	103.4700012
-6.1359234	13.2736006	102.5199966
-6.1359234	13.3224001	103.3600006
-6.1359234	13.3712006	105.9199982
-6.1359234	13.4200001	104.7699966
-6.1359234	13.4688005	105.0800018
-6.1359234	13.5176001	104.3899994
-6.1359234	13.5664005	102.4000015
-6.1359234	13.6152000	103.5999985
-6.1359234	13.6640005	105.5800018
-6.1359234	13.7128000	104.4599991
-6.1359234	13.7616005	101.9799957
-6.1359234	13.8104000	99.8499985
-6.1359234	13.8592005	99.1500015
-6.1359234	13.9080000	97.8199997
-6.1359234	13.9568005	101.1100006
-6.1359234	14.0056000	103.0899963
-6.1359234	14.0544004	103.2900009
-6.1359234	14.1032000	99.5800018
-6.1359234	14.1520004	101.3699951
-6.1359234	14.2007999	103.6699982
-6.1359234	14.2496004	102.4700012
-6.1359234	14.2983999	106.3099976
-6.1359234	14.3472004	106.4599991
-6.1359234	14.3959999	102.0599976
-6.1359234	14.4448004	98.1299973
-6.1359234	14.4935999	102.1100006
-6.1359234	14.5424004	104.0599976

-6.1359234	14.5911999	104.0899963
-6.1359234	14.6400003	103.7900009
-6.1359234	14.6887999	104.7299957
-6.1359234	14.7376003	103.2799988
-6.1359234	14.7863998	101.1699982
-6.1359234	14.8352003	102.6699982
-6.1359234	14.8839998	103.0400009
-6.1359234	14.9328003	103.5699997
-6.1359234	14.9815998	104.2200012
-6.1359234	15.0304003	103.7600021
-6.1297874	0.3416000	105.9899979
-6.1297874	0.3904000	107.2500000
-6.1297874	0.4392000	108.0000000
-6.1297874	0.4880000	106.2500000
-6.1297874	0.5368000	103.0199966
-6.1297874	0.5856000	104.9499969
-6.1297874	0.6344000	102.8899994
-6.1297874	0.6832000	106.0800018
-6.1297874	0.7320000	108.8499985
-6.1297874	0.7808000	108.0499954
-6.1297874	0.8296000	106.2399979
-6.1297874	0.8784000	107.6299973
-6.1297874	0.9272000	109.5499954
-6.1297874	0.9760000	110.4099960
-6.1297874	1.0247999	109.7200012
-6.1297874	1.0735999	109.2900009
-6.1297874	1.1223999	109.2999954
-6.1297874	1.1711999	104.9599991
-6.1297874	1.2200000	106.0199966
-6.1297874	1.2688000	104.6599960
-6.1297874	1.3176000	105.9599991
-6.1297874	1.3664000	105.4599991
-6.1297874	1.4152000	104.7500000
-6.1297874	1.4640000	105.3600006
-6.1297874	1.5128000	104.4899979
-6.1297874	1.5616000	104.1100006
-6.1297874	1.6104000	106.9199982
-6.1297874	1.6592000	107.0800018
-6.1297874	1.7079999	109.2600021
-6.1297874	1.7567999	109.8600006
-6.1297874	1.8055999	112.1199951
-6.1297874	1.8543999	119.7600021
-6.1297874	1.9031999	121.2999954
-6.1297874	1.9519999	116.7500000
-6.1297874	2.0007999	106.6500015
-6.1297874	2.0495999	107.8099976
-6.1297874	2.0983999	109.2799988
-6.1297874	2.1471999	105.9700012
-6.1297874	2.1959999	104.7699966
-6.1297874	2.2447999	105.3699951
-6.1297874	2.2935998	104.5999985
-6.1297874	2.3423998	103.5499954
-6.1297874	2.3912001	106.0100021
-6.1297874	2.4400001	105.4000015
-6.1297874	2.4888000	108.0299988
-6.1297874	2.5376000	107.6899948
-6.1297874	2.5864000	105.4899979
-6.1297874	2.6352000	104.3799973

-6.1297874	2.6840000	98.6399994
-6.1297874	2.7328000	101.8199997
-6.1297874	2.7816000	103.2399979
-6.1297874	2.8304000	104.4199982
-6.1297874	2.8792000	104.0800018
-6.1297874	2.9280000	106.6999969
-6.1297874	2.9768000	106.2799988
-6.1297874	3.0256000	104.9300003
-6.1297874	3.0743999	106.0400009
-6.1297874	3.1231999	103.5100021
-6.1297874	3.1719999	105.6299973
-6.1297874	3.2207999	105.2099991
-6.1297874	3.2695999	105.2799988
-6.1297874	3.3183999	106.2399979
-6.1297874	3.3671999	103.8399963
-6.1297874	3.4159999	105.5699997
-6.1297874	3.4647999	105.1299973
-6.1297874	3.5135999	104.4499969
-6.1297874	3.5623999	103.7099991
-6.1297874	3.6111999	104.3399963
-6.1297874	3.6599998	103.3799973
-6.1297874	3.7087998	102.0400009
-6.1297874	3.7575998	102.6299973
-6.1297874	3.8063998	102.9799957
-6.1297874	3.8551998	104.5000000
-6.1297874	3.9039998	102.8399963
-6.1297874	3.9527998	102.3799973
-6.1297874	4.0016003	104.3199997
-6.1297874	4.0503998	104.7799988
-6.1297874	4.0991998	105.0400009
-6.1297874	4.1479998	105.1999969
-6.1297874	4.1967998	105.6999969
-6.1297874	4.2455997	104.1899948
-6.1297874	4.2943997	108.6999969
-6.1297874	4.3431997	112.4099960
-6.1297874	4.3919997	111.2600021
-6.1297874	4.4407997	110.0199966
-6.1297874	4.4895997	111.2900009
-6.1297874	4.5383997	116.0899963
-6.1297874	4.5871997	119.0199966
-6.1297874	4.6359997	117.2099991
-6.1297874	4.6847997	116.2299957
-6.1297874	4.7336001	114.5999985
-6.1297874	4.7824001	111.2900009
-6.1297874	4.8312001	112.0000000
-6.1297874	4.8800001	113.6699982
-6.1297874	4.9288001	111.8300018
-6.1297874	4.9776001	110.5899963
-6.1297874	5.0264001	107.2500000
-6.1297874	5.0752001	109.4399948
-6.1297874	5.1240001	111.2299957
-6.1297874	5.1728001	109.2799988
-6.1297874	5.2216001	106.3499985
-6.1297874	5.2704000	105.1100006
-6.1297874	5.3192000	104.4099960
-6.1297874	5.3680000	104.6800003
-6.1297874	5.4168000	105.4899979
-6.1297874	5.4656000	104.8799973

-6.1297874	5.5144000	100.7099991
-6.1297874	5.5632000	102.7299957
-6.1297874	5.6120000	102.2399979
-6.1297874	5.6608000	103.6500015
-6.1297874	5.7096000	104.9199982
-6.1297874	5.7584000	106.9799957
-6.1297874	5.8072000	105.7200012
-6.1297874	5.8559999	104.4099960
-6.1297874	5.9047999	106.1800003
-6.1297874	5.9535999	106.3899994
-6.1297874	6.0023999	104.6800003
-6.1297874	6.0511999	107.5199966
-6.1297874	6.0999999	108.1399994
-6.1297874	6.1487999	107.7900009
-6.1297874	6.1975999	107.4799957
-6.1297874	6.2463999	104.6299973
-6.1297874	6.2951999	104.7399979
-6.1297874	6.3439999	104.8699951
-6.1297874	6.3927999	105.4000015
-6.1297874	6.4415998	107.9599991
-6.1297874	6.4903998	107.3899994
-6.1297874	6.5391998	106.5299988
-6.1297874	6.5879998	105.3799973
-6.1297874	6.6367998	103.2799988
-6.1297874	6.6855998	105.6800003
-6.1297874	6.7343998	105.1800003
-6.1297874	6.7831998	99.9499969
-6.1297874	6.8319998	103.3499985
-6.1297874	6.8807998	106.0199966
-6.1297874	6.9295998	106.6199951
-6.1297874	6.9783998	104.8199997
-6.1297874	7.0271997	100.3300018
-6.1297874	7.0759997	104.9399948
-6.1297874	7.1247997	103.9899979
-6.1297874	7.1735997	101.8099976
-6.1297874	7.2223997	104.6800003
-6.1297874	7.2711997	105.2799988
-6.1297874	7.3199997	104.1899948
-6.1297874	7.3687997	101.1500015
-6.1297874	7.4175997	102.5299988
-6.1297874	7.4663997	102.1299973
-6.1297874	7.5151997	103.4099960
-6.1297874	7.5639997	103.5199966
-6.1297874	7.6127996	103.3499985
-6.1297874	7.6615996	103.0899963
-6.1297874	7.7103996	103.3899994
-6.1297874	7.7591996	101.7699966
-6.1297874	7.8079996	101.3300018
-6.1297874	7.8567996	100.3300018
-6.1297874	7.9056001	103.7799988
-6.1297874	7.9544001	104.4000015
-6.1297874	8.0032005	104.1199951
-6.1297874	8.0520000	104.1100006
-6.1297874	8.1008005	102.2500000
-6.1297874	8.1496000	103.5999985
-6.1297874	8.1984005	104.5899963
-6.1297874	8.2472000	104.3699951
-6.1297874	8.2960005	104.8399963

-6.1297874	8.3448000	104.5999985
-6.1297874	8.3936005	103.9700012
-6.1297874	8.4424000	102.3499985
-6.1297874	8.4912004	104.8799973
-6.1297874	8.5400000	105.6800003
-6.1297874	8.5888004	104.8799973
-6.1297874	8.6375999	104.3499985
-6.1297874	8.6864004	104.7799988
-6.1297874	8.7351999	105.3199997
-6.1297874	8.7840004	103.0499954
-6.1297874	8.8327999	101.5599976
-6.1297874	8.8816004	103.9099960
-6.1297874	8.9303999	102.8699951
-6.1297874	8.9792004	102.3499985
-6.1297874	9.0279999	103.3799973
-6.1297874	9.0768003	103.4899979
-6.1297874	9.1255999	106.5000000
-6.1297874	9.1744003	105.8300018
-6.1297874	9.2231998	101.5599976
-6.1297874	9.2720003	102.5400009
-6.1297874	9.3207998	106.2900009
-6.1297874	9.3696003	103.0299988
-6.1297874	9.4184008	99.5999985
-6.1297874	9.4672003	102.1599960
-6.1297874	9.5160007	102.3499985
-6.1297874	9.5648003	103.0999985
-6.1297874	9.6136007	103.5100021
-6.1297874	9.6624002	105.1999969
-6.1297874	9.7112007	103.3099976
-6.1297874	9.7600002	106.0699997
-6.1297874	9.8088007	104.2999954
-6.1297874	9.8576002	101.9700012
-6.1297874	9.9064007	107.2099991
-6.1297874	9.9552002	107.5599976
-6.1297874	10.0040007	104.5999985
-6.1297874	10.0528002	102.0999985
-6.1297874	10.1016006	103.3399963
-6.1297874	10.1504002	104.1199951
-6.1297874	10.1992006	101.5800018
-6.1297874	10.2480001	101.4899979
-6.1297874	10.2968006	104.8199997
-6.1297874	10.3456001	105.6100006
-6.1297874	10.3944006	106.5000000
-6.1297874	10.4432001	105.9000015
-6.1297874	10.4920006	103.5999985
-6.1297874	10.5408001	104.4300003
-6.1297874	10.5896006	102.7399979
-6.1297874	10.6384001	100.4899979
-6.1297874	10.6872005	104.5000000
-6.1297874	10.7360001	104.5599976
-6.1297874	10.7848005	100.6599960
-6.1297874	10.8336000	104.5899963
-6.1297874	10.8824005	104.6199951
-6.1297874	10.9312000	103.0499954
-6.1297874	10.9800005	101.6599960
-6.1297874	11.0288000	95.2099991
-6.1297874	11.0776005	100.0499954
-6.1297874	11.1264000	101.8600006

-6.1297874	11.1752005	104.6800003
-6.1297874	11.2240000	105.1999969
-6.1297874	11.2728004	102.5199966
-6.1297874	11.3216000	104.5499954
-6.1297874	11.3704004	108.1199951
-6.1297874	11.4191999	108.8099976
-6.1297874	11.4680004	107.0999985
-6.1297874	11.5167999	105.2900009
-6.1297874	11.5656004	104.0499954
-6.1297874	11.6143999	103.0699997
-6.1297874	11.6632004	102.3300018
-6.1297874	11.7119999	104.0000000
-6.1297874	11.7608004	104.5800018
-6.1297874	11.8095999	103.3099976
-6.1297874	11.8584003	105.3300018
-6.1297874	11.9071999	103.1999969
-6.1297874	11.9560003	101.0899963
-6.1297874	12.0047998	104.0400009
-6.1297874	12.0536003	104.8399963
-6.1297874	12.1023998	105.9899979
-6.1297874	12.1512003	101.5599976
-6.1297874	12.1999998	104.1699982
-6.1297874	12.2488003	106.1500015
-6.1297874	12.2975998	108.7200012
-6.1297874	12.3464003	106.8499985
-6.1297874	12.3951998	102.6800003
-6.1297874	12.4440002	100.6299973
-6.1297874	12.4927998	103.2799988
-6.1297874	12.5416002	103.1100006
-6.1297874	12.5904007	101.7200012
-6.1297874	12.6392002	103.2200012
-6.1297874	12.6880007	106.8799973
-6.1297874	12.7368002	105.7799988
-6.1297874	12.7856007	102.1500015
-6.1297874	12.8344002	100.4499969
-6.1297874	12.8832006	101.7600021
-6.1297874	12.9320002	101.7699966
-6.1297874	12.9808006	103.8199997
-6.1297874	13.0296001	102.9300003
-6.1297874	13.0784006	101.6399994
-6.1297874	13.1272001	101.5000000
-6.1297874	13.1760006	99.6800003
-6.1297874	13.2248001	103.1999969
-6.1297874	13.2736006	102.0299988
-6.1297874	13.3224001	102.6899948
-6.1297874	13.3712006	105.6899948
-6.1297874	13.4200001	105.2600021
-6.1297874	13.4688005	105.5800018
-6.1297874	13.5176001	105.2699966
-6.1297874	13.5664005	103.1599960
-6.1297874	13.6152000	103.7699966
-6.1297874	13.6640005	105.5999985
-6.1297874	13.7128000	104.4000015
-6.1297874	13.7616005	101.4099960
-6.1297874	13.8104000	100.3099976
-6.1297874	13.8592005	99.9300003
-6.1297874	13.9080000	98.7799988
-6.1297874	13.9568005	101.3899994

-6.1297874	14.0056000	103.4599991
-6.1297874	14.0544004	104.1800003
-6.1297874	14.1032000	101.7600021
-6.1297874	14.1520004	101.9099960
-6.1297874	14.2007999	103.9199982
-6.1297874	14.2496004	103.1899948
-6.1297874	14.2983999	106.4099960
-6.1297874	14.3472004	105.9399948
-6.1297874	14.3959999	102.0999985
-6.1297874	14.4448004	98.6100006
-6.1297874	14.4935999	102.5999985
-6.1297874	14.5424004	104.4399948
-6.1297874	14.5911999	103.7399979
-6.1297874	14.6400003	102.9300003
-6.1297874	14.6887999	104.9899979
-6.1297874	14.7376003	104.0899963
-6.1297874	14.7863998	101.8499985
-6.1297874	14.8352003	102.4899979
-6.1297874	14.8839998	103.3499985
-6.1297874	14.9328003	103.3699951
-6.1297874	14.9815998	103.6800003
-6.1297874	15.0304003	102.6800003
-6.1236515	0.3416000	106.4000015
-6.1236515	0.3904000	108.0899963
-6.1236515	0.4392000	108.5299988
-6.1236515	0.4880000	106.4700012
-6.1236515	0.5368000	102.6299973
-6.1236515	0.5856000	104.5499954
-6.1236515	0.6344000	102.1399994
-6.1236515	0.6832000	105.4799957
-6.1236515	0.7320000	108.7600021
-6.1236515	0.7808000	108.1699982
-6.1236515	0.8296000	106.6800003
-6.1236515	0.8784000	108.3399963
-6.1236515	0.9272000	110.4599991
-6.1236515	0.9760000	111.5100021
-6.1236515	1.0247999	110.6999969
-6.1236515	1.0735999	109.0000000
-6.1236515	1.1223999	108.5800018
-6.1236515	1.1711999	104.7600021
-6.1236515	1.2200000	106.0000000
-6.1236515	1.2688000	105.7699966
-6.1236515	1.3176000	106.8499985
-6.1236515	1.3664000	105.1699982
-6.1236515	1.4152000	103.6399994
-6.1236515	1.4640000	104.4499969
-6.1236515	1.5128000	103.6800003
-6.1236515	1.5616000	102.9799957
-6.1236515	1.6104000	106.0699997
-6.1236515	1.6592000	105.5299988
-6.1236515	1.7079999	108.0000000
-6.1236515	1.7567999	109.4300003
-6.1236515	1.8055999	113.1199951
-6.1236515	1.8543999	120.3899994
-6.1236515	1.9031999	122.0299988
-6.1236515	1.9519999	117.4000015
-6.1236515	2.0007999	107.1100006
-6.1236515	2.0495999	108.2999954

-6.1236515	2.0983999	109.2399979
-6.1236515	2.1471999	106.0400009
-6.1236515	2.1959999	105.5000000
-6.1236515	2.2447999	105.6699982
-6.1236515	2.2935998	105.1199951
-6.1236515	2.3423998	104.3600006
-6.1236515	2.3912001	105.6999969
-6.1236515	2.4400001	104.8600006
-6.1236515	2.4888000	108.4700012
-6.1236515	2.5376000	107.4199982
-6.1236515	2.5864000	104.6699982
-6.1236515	2.6352000	104.1699982
-6.1236515	2.6840000	99.2799988
-6.1236515	2.7328000	101.3300018
-6.1236515	2.7816000	102.7999954
-6.1236515	2.8304000	104.5299988
-6.1236515	2.8792000	104.4199982
-6.1236515	2.9280000	106.6500015
-6.1236515	2.9768000	105.0599976
-6.1236515	3.0256000	104.5499954
-6.1236515	3.0743999	105.1599960
-6.1236515	3.1231999	101.8300018
-6.1236515	3.1719999	104.2999954
-6.1236515	3.2207999	105.2399979
-6.1236515	3.2695999	105.3099976
-6.1236515	3.3183999	106.9899979
-6.1236515	3.3671999	104.1899948
-6.1236515	3.4159999	105.7999954
-6.1236515	3.4647999	105.1500015
-6.1236515	3.5135999	104.0400009
-6.1236515	3.5623999	103.4799957
-6.1236515	3.6111999	104.4399948
-6.1236515	3.6599998	103.5499954
-6.1236515	3.7087998	101.7099991
-6.1236515	3.7575998	103.4099960
-6.1236515	3.8063998	103.7799988
-6.1236515	3.8551998	104.3199997
-6.1236515	3.9039998	102.9700012
-6.1236515	3.9527998	102.9499969
-6.1236515	4.0016003	104.5499954
-6.1236515	4.0503998	104.0899963
-6.1236515	4.0991998	104.4599991
-6.1236515	4.1479998	103.6199951
-6.1236515	4.1967998	105.3600006
-6.1236515	4.2455997	104.4700012
-6.1236515	4.2943997	108.7099991
-6.1236515	4.3431997	112.1500015
-6.1236515	4.3919997	111.7900009
-6.1236515	4.4407997	109.6999969
-6.1236515	4.4895997	110.2399979
-6.1236515	4.5383997	115.8499985
-6.1236515	4.5871997	119.3199997
-6.1236515	4.6359997	117.4599991
-6.1236515	4.6847997	117.2299957
-6.1236515	4.7336001	115.7099991
-6.1236515	4.7824001	112.0999985
-6.1236515	4.8312001	112.5800018
-6.1236515	4.8800001	113.9499969

-6.1236515	4.9288001	111.7699966
-6.1236515	4.9776001	110.4799957
-6.1236515	5.0264001	108.2099991
-6.1236515	5.0752001	110.6399994
-6.1236515	5.1240001	111.7200012
-6.1236515	5.1728001	109.7799988
-6.1236515	5.2216001	106.7299957
-6.1236515	5.2704000	103.9599991
-6.1236515	5.3192000	103.2200012
-6.1236515	5.3680000	104.7399979
-6.1236515	5.4168000	106.1999969
-6.1236515	5.4656000	105.4099960
-6.1236515	5.5144000	101.2200012
-6.1236515	5.5632000	101.4399948
-6.1236515	5.6120000	102.2099991
-6.1236515	5.6608000	104.6699982
-6.1236515	5.7096000	104.6500015
-6.1236515	5.7584000	106.3600006
-6.1236515	5.8072000	105.5499954
-6.1236515	5.8559999	104.0999985
-6.1236515	5.9047999	106.8300018
-6.1236515	5.9535999	107.6299973
-6.1236515	6.0023999	106.1500015
-6.1236515	6.0511999	107.9399948
-6.1236515	6.0999999	108.2699966
-6.1236515	6.1487999	107.5499954
-6.1236515	6.1975999	106.6999969
-6.1236515	6.2463999	103.6999969
-6.1236515	6.2951999	104.3899994
-6.1236515	6.3439999	105.0000000
-6.1236515	6.3927999	105.5000000
-6.1236515	6.4415998	107.6899948
-6.1236515	6.4903998	106.9899979
-6.1236515	6.5391998	106.0999985
-6.1236515	6.5879998	105.1899948
-6.1236515	6.6367998	102.6199951
-6.1236515	6.6855998	105.7999954
-6.1236515	6.7343998	105.6100006
-6.1236515	6.7831998	101.5400009
-6.1236515	6.8319998	104.6399994
-6.1236515	6.8807998	105.5699997
-6.1236515	6.9295998	106.2999954
-6.1236515	6.9783998	103.9599991
-6.1236515	7.0271997	99.9899979
-6.1236515	7.0759997	105.0400009
-6.1236515	7.1247997	104.0499954
-6.1236515	7.1735997	100.9399948
-6.1236515	7.2223997	103.8799973
-6.1236515	7.2711997	104.5899963
-6.1236515	7.3199997	104.2299957
-6.1236515	7.3687997	100.4000015
-6.1236515	7.4175997	102.4399948
-6.1236515	7.4663997	101.6100006
-6.1236515	7.5151997	103.0000000
-6.1236515	7.5639997	104.5100021
-6.1236515	7.6127996	104.3699951
-6.1236515	7.6615996	103.2799988
-6.1236515	7.7103996	102.1100006

-6.1236515	7.7591996	101.7399979
-6.1236515	7.8079996	102.0199966
-6.1236515	7.8567996	99.6500015
-6.1236515	7.9056001	103.2500000
-6.1236515	7.9544001	104.5000000
-6.1236515	8.0032005	103.7399979
-6.1236515	8.0520000	103.4300003
-6.1236515	8.1008005	102.0199966
-6.1236515	8.1496000	103.2299957
-6.1236515	8.1984005	104.0999985
-6.1236515	8.2472000	104.7200012
-6.1236515	8.2960005	105.7099991
-6.1236515	8.3448000	105.7299957
-6.1236515	8.3936005	104.6800003
-6.1236515	8.4424000	103.0000000
-6.1236515	8.4912004	104.6999969
-6.1236515	8.5400000	105.4700012
-6.1236515	8.5888004	105.2900009
-6.1236515	8.6375999	104.4499969
-6.1236515	8.6864004	104.2500000
-6.1236515	8.7351999	104.3899994
-6.1236515	8.7840004	102.7099991
-6.1236515	8.8327999	101.4599991
-6.1236515	8.8816004	103.6999969
-6.1236515	8.9303999	102.6100006
-6.1236515	8.9792004	102.6800003
-6.1236515	9.0279999	103.8799973
-6.1236515	9.0768003	102.3399963
-6.1236515	9.1255999	106.0100021
-6.1236515	9.1744003	106.0899963
-6.1236515	9.2231998	102.3699951
-6.1236515	9.2720003	100.8199997
-6.1236515	9.3207998	105.3799973
-6.1236515	9.3696003	102.1199951
-6.1236515	9.4184008	98.7200012
-6.1236515	9.4672003	102.4300003
-6.1236515	9.5160007	102.0299988
-6.1236515	9.5648003	103.5699997
-6.1236515	9.6136007	104.1899948
-6.1236515	9.6624002	105.1199951
-6.1236515	9.7112007	103.3399963
-6.1236515	9.7600002	106.1599960
-6.1236515	9.8088007	103.6199951
-6.1236515	9.8576002	101.0999985
-6.1236515	9.9064007	106.9599991
-6.1236515	9.9552002	108.2399979
-6.1236515	10.0040007	105.6800003
-6.1236515	10.0528002	102.3300018
-6.1236515	10.1016006	102.9399948
-6.1236515	10.1504002	103.6999969
-6.1236515	10.1992006	102.2699966
-6.1236515	10.2480001	102.4399948
-6.1236515	10.2968006	105.5100021
-6.1236515	10.3456001	105.7600021
-6.1236515	10.3944006	106.7099991
-6.1236515	10.4432001	106.2799988
-6.1236515	10.4920006	103.7799988
-6.1236515	10.5408001	103.9799957

-6.1236515	10.5896006	102.7200012
-6.1236515	10.6384001	99.6500015
-6.1236515	10.6872005	104.5000000
-6.1236515	10.7360001	104.7399979
-6.1236515	10.7848005	102.2999954
-6.1236515	10.8336000	105.1599960
-6.1236515	10.8824005	104.9700012
-6.1236515	10.9312000	103.7200012
-6.1236515	10.9800005	101.3799973
-6.1236515	11.0288000	97.2600021
-6.1236515	11.0776005	100.1800003
-6.1236515	11.1264000	102.5899963
-6.1236515	11.1752005	104.6599960
-6.1236515	11.2240000	105.6999969
-6.1236515	11.2728004	102.0400009
-6.1236515	11.3216000	104.5400009
-6.1236515	11.3704004	107.6599960
-6.1236515	11.4191999	108.5000000
-6.1236515	11.4680004	106.8600006
-6.1236515	11.5167999	105.4700012
-6.1236515	11.5656004	104.6999969
-6.1236515	11.6143999	103.5400009
-6.1236515	11.6632004	102.9599991
-6.1236515	11.7119999	104.2999954
-6.1236515	11.7608004	105.2699966
-6.1236515	11.8095999	103.9899979
-6.1236515	11.8584003	105.4399948
-6.1236515	11.9071999	103.6399994
-6.1236515	11.9560003	101.7500000
-6.1236515	12.0047998	103.6899948
-6.1236515	12.0536003	102.9000015
-6.1236515	12.1023998	104.6800003
-6.1236515	12.1512003	100.1399994
-6.1236515	12.1999998	103.9599991
-6.1236515	12.2488003	106.4000015
-6.1236515	12.2975998	108.3099976
-6.1236515	12.3464003	106.1500015
-6.1236515	12.3951998	102.8399963
-6.1236515	12.4440002	101.7099991
-6.1236515	12.4927998	104.0100021
-6.1236515	12.5416002	103.3499985
-6.1236515	12.5904007	101.6500015
-6.1236515	12.6392002	104.8399963
-6.1236515	12.6880007	107.9599991
-6.1236515	12.7368002	106.0400009
-6.1236515	12.7856007	101.9199982
-6.1236515	12.8344002	101.2999954
-6.1236515	12.8832006	102.1800003
-6.1236515	12.9320002	100.6199951
-6.1236515	12.9808006	103.4499969
-6.1236515	13.0296001	103.0499954
-6.1236515	13.0784006	102.7699966
-6.1236515	13.1272001	102.2299957
-6.1236515	13.1760006	99.5599976
-6.1236515	13.2248001	103.1699982
-6.1236515	13.2736006	101.6800003
-6.1236515	13.3224001	102.2500000
-6.1236515	13.3712006	105.3199997

-6.1236515	13.4200001	105.4300003
-6.1236515	13.4688005	105.5999985
-6.1236515	13.5176001	104.7900009
-6.1236515	13.5664005	103.6899948
-6.1236515	13.6152000	103.7299957
-6.1236515	13.6640005	105.1199951
-6.1236515	13.7128000	104.2099991
-6.1236515	13.7616005	101.0899963
-6.1236515	13.8104000	100.9399948
-6.1236515	13.8592005	100.9499969
-6.1236515	13.9080000	100.4399948
-6.1236515	13.9568005	102.2699966
-6.1236515	14.0056000	103.5499954
-6.1236515	14.0544004	104.7399979
-6.1236515	14.1032000	103.8499985
-6.1236515	14.1520004	102.9899979
-6.1236515	14.2007999	104.0400009
-6.1236515	14.2496004	103.4399948
-6.1236515	14.2983999	106.1399994
-6.1236515	14.3472004	105.3199997
-6.1236515	14.3959999	102.4499969
-6.1236515	14.4448004	98.9199982
-6.1236515	14.4935999	102.3199997
-6.1236515	14.5424004	104.4199982
-6.1236515	14.5911999	103.4000015
-6.1236515	14.6400003	102.1699982
-6.1236515	14.6887999	105.0999985
-6.1236515	14.7376003	104.3300018
-6.1236515	14.7863998	102.4099960
-6.1236515	14.8352003	101.9799957
-6.1236515	14.8839998	102.7699966
-6.1236515	14.9328003	102.9799957
-6.1236515	14.9815998	102.7099991
-6.1236515	15.0304003	101.7299957
-6.1175156	0.3416000	107.2799988
-6.1175156	0.3904000	109.1199951
-6.1175156	0.4392000	109.2900009
-6.1175156	0.4880000	106.9499969
-6.1175156	0.5368000	102.5299988
-6.1175156	0.5856000	104.3600006
-6.1175156	0.6344000	101.4399948
-6.1175156	0.6832000	105.3199997
-6.1175156	0.7320000	108.4799957
-6.1175156	0.7808000	108.4099960
-6.1175156	0.8296000	107.6100006
-6.1175156	0.8784000	109.1800003
-6.1175156	0.9272000	110.6299973
-6.1175156	0.9760000	111.3699951
-6.1175156	1.0247999	110.6299973
-6.1175156	1.0735999	108.4399948
-6.1175156	1.1223999	108.2500000
-6.1175156	1.1711999	105.0899963
-6.1175156	1.2200000	105.8499985
-6.1175156	1.2688000	106.5899963
-6.1175156	1.3176000	106.7600021
-6.1175156	1.3664000	104.5899963
-6.1175156	1.4152000	103.2200012
-6.1175156	1.4640000	104.1399994

-6.1175156	1.5128000	102.7399979
-6.1175156	1.5616000	101.4000015
-6.1175156	1.6104000	104.9000015
-6.1175156	1.6592000	104.0800018
-6.1175156	1.7079999	107.9599991
-6.1175156	1.7567999	109.4099960
-6.1175156	1.8055999	113.5199966
-6.1175156	1.8543999	120.6899948
-6.1175156	1.9031999	122.4300003
-6.1175156	1.9519999	117.6299973
-6.1175156	2.0007999	107.3099976
-6.1175156	2.0495999	107.8199997
-6.1175156	2.0983999	108.2999954
-6.1175156	2.1471999	105.0400009
-6.1175156	2.1959999	105.5699997
-6.1175156	2.2447999	105.8099976
-6.1175156	2.2935998	105.3600006
-6.1175156	2.3423998	104.5299988
-6.1175156	2.3912001	105.1699982
-6.1175156	2.4400001	104.1800003
-6.1175156	2.4888000	108.9399948
-6.1175156	2.5376000	108.0000000
-6.1175156	2.5864000	104.0800018
-6.1175156	2.6352000	103.4000015
-6.1175156	2.6840000	99.5100021
-6.1175156	2.7328000	100.4899979
-6.1175156	2.7816000	102.2999954
-6.1175156	2.8304000	104.9899979
-6.1175156	2.8792000	104.7299957
-6.1175156	2.9280000	106.6800003
-6.1175156	2.9768000	104.9000015
-6.1175156	3.0256000	105.0800018
-6.1175156	3.0743999	104.4199982
-6.1175156	3.1231999	100.2799988
-6.1175156	3.1719999	103.3300018
-6.1175156	3.2207999	104.9799957
-6.1175156	3.2695999	104.6599960
-6.1175156	3.3183999	107.1399994
-6.1175156	3.3671999	104.0499954
-6.1175156	3.4159999	105.2099991
-6.1175156	3.4647999	105.1699982
-6.1175156	3.5135999	103.8300018
-6.1175156	3.5623999	103.3099976
-6.1175156	3.6111999	104.0999985
-6.1175156	3.6599998	103.2099991
-6.1175156	3.7087998	102.1500015
-6.1175156	3.7575998	103.9300003
-6.1175156	3.8063998	104.0100021
-6.1175156	3.8551998	103.7799988
-6.1175156	3.9039998	104.0199966
-6.1175156	3.9527998	104.7099991
-6.1175156	4.0016003	104.3600006
-6.1175156	4.0503998	102.7600021
-6.1175156	4.0991998	103.5699997
-6.1175156	4.1479998	101.7999954
-6.1175156	4.1967998	105.0899963
-6.1175156	4.2455997	104.1999969
-6.1175156	4.2943997	108.0499954

-6.1175156	4.3431997	111.3899994
-6.1175156	4.3919997	111.5400009
-6.1175156	4.4407997	108.5000000
-6.1175156	4.4895997	108.7500000
-6.1175156	4.5383997	115.5999985
-6.1175156	4.5871997	119.7399979
-6.1175156	4.6359997	118.1299973
-6.1175156	4.6847997	117.3199997
-6.1175156	4.7336001	116.1800003
-6.1175156	4.7824001	112.5499954
-6.1175156	4.8312001	112.7299957
-6.1175156	4.8800001	113.5999985
-6.1175156	4.9288001	110.7500000
-6.1175156	4.9776001	109.8899994
-6.1175156	5.0264001	108.7299957
-6.1175156	5.0752001	111.3499985
-6.1175156	5.1240001	111.0800018
-6.1175156	5.1728001	109.7600021
-6.1175156	5.2216001	107.1199951
-6.1175156	5.2704000	103.9000015
-6.1175156	5.3192000	102.8600006
-6.1175156	5.3680000	105.8099976
-6.1175156	5.4168000	107.7600021
-6.1175156	5.4656000	106.8099976
-6.1175156	5.5144000	102.8300018
-6.1175156	5.5632000	101.0699997
-6.1175156	5.6120000	102.8499985
-6.1175156	5.6608000	105.3099976
-6.1175156	5.7096000	103.2699966
-6.1175156	5.7584000	104.4700012
-6.1175156	5.8072000	104.6500015
-6.1175156	5.8559999	103.7099991
-6.1175156	5.9047999	106.8600006
-6.1175156	5.9535999	108.2999954
-6.1175156	6.0023999	106.9799957
-6.1175156	6.0511999	107.7799988
-6.1175156	6.0999999	108.3799973
-6.1175156	6.1487999	107.2099991
-6.1175156	6.1975999	106.1399994
-6.1175156	6.2463999	102.7099991
-6.1175156	6.2951999	103.3499985
-6.1175156	6.3439999	104.5899963
-6.1175156	6.3927999	105.8699951
-6.1175156	6.4415998	107.4000015
-6.1175156	6.4903998	106.2799988
-6.1175156	6.5391998	105.6299973
-6.1175156	6.5879998	105.5299988
-6.1175156	6.6367998	102.8499985
-6.1175156	6.6855998	106.0499954
-6.1175156	6.7343998	105.8099976
-6.1175156	6.7831998	102.5299988
-6.1175156	6.8319998	105.6399994
-6.1175156	6.8807998	105.4499969
-6.1175156	6.9295998	106.1899948
-6.1175156	6.9783998	102.8499985
-6.1175156	7.0271997	99.8600006
-6.1175156	7.0759997	104.8799973
-6.1175156	7.1247997	104.0199966

-6.1175156	7.1735997	100.6899948
-6.1175156	7.2223997	102.8300018
-6.1175156	7.2711997	103.5800018
-6.1175156	7.3199997	104.7099991
-6.1175156	7.3687997	101.0599976
-6.1175156	7.4175997	103.3899994
-6.1175156	7.4663997	101.3799973
-6.1175156	7.5151997	102.6999969
-6.1175156	7.5639997	105.2299957
-6.1175156	7.6127996	105.1999969
-6.1175156	7.6615996	103.1999969
-6.1175156	7.7103996	101.1599960
-6.1175156	7.7591996	102.5899963
-6.1175156	7.8079996	103.2099991
-6.1175156	7.8567996	99.8600006
-6.1175156	7.9056001	102.1899948
-6.1175156	7.9544001	104.6899948
-6.1175156	8.0032005	104.1599960
-6.1175156	8.0520000	102.8699951
-6.1175156	8.1008005	101.5299988
-6.1175156	8.1496000	102.5999985
-6.1175156	8.1984005	103.4799957
-6.1175156	8.2472000	105.3300018
-6.1175156	8.2960005	106.6500015
-6.1175156	8.3448000	106.2299957
-6.1175156	8.3936005	104.5299988
-6.1175156	8.4424000	103.2699966
-6.1175156	8.4912004	104.3699951
-6.1175156	8.5400000	105.0100021
-6.1175156	8.5888004	105.2900009
-6.1175156	8.6375999	103.8899994
-6.1175156	8.6864004	103.5599976
-6.1175156	8.7351999	103.8799973
-6.1175156	8.7840004	103.1800003
-6.1175156	8.8327999	102.7299957
-6.1175156	8.8816004	103.8300018
-6.1175156	8.9303999	102.5199966
-6.1175156	8.9792004	102.9399948
-6.1175156	9.0279999	104.4700012
-6.1175156	9.0768003	102.2999954
-6.1175156	9.1255999	105.9099960
-6.1175156	9.1744003	105.8600006
-6.1175156	9.2231998	102.4899979
-6.1175156	9.2720003	99.4199982
-6.1175156	9.3207998	104.5499954
-6.1175156	9.3696003	102.3499985
-6.1175156	9.4184008	98.0100021
-6.1175156	9.4672003	101.6500015
-6.1175156	9.5160007	101.4599991
-6.1175156	9.5648003	103.5899963
-6.1175156	9.6136007	104.1299973
-6.1175156	9.6624002	104.6100006
-6.1175156	9.7112007	102.7200012
-6.1175156	9.7600002	106.0199966
-6.1175156	9.8088007	102.8199997
-6.1175156	9.8576002	99.7600021
-6.1175156	9.9064007	105.5199966
-6.1175156	9.9552002	107.7200012

-6.1175156	10.0040007	105.6199951
-6.1175156	10.0528002	102.0199966
-6.1175156	10.1016006	103.3799973
-6.1175156	10.1504002	103.7799988
-6.1175156	10.1992006	102.7500000
-6.1175156	10.2480001	102.5499954
-6.1175156	10.2968006	104.9000015
-6.1175156	10.3456001	105.3300018
-6.1175156	10.3944006	106.7299957
-6.1175156	10.4432001	106.5100021
-6.1175156	10.4920006	103.6399994
-6.1175156	10.5408001	103.4799957
-6.1175156	10.5896006	102.5199966
-6.1175156	10.6384001	98.8199997
-6.1175156	10.6872005	104.5400009
-6.1175156	10.7360001	105.0599976
-6.1175156	10.7848005	104.0499954
-6.1175156	10.8336000	105.8199997
-6.1175156	10.8824005	105.7799988
-6.1175156	10.9312000	104.4799957
-6.1175156	10.9800005	101.9899979
-6.1175156	11.0288000	99.6500015
-6.1175156	11.0776005	100.7299957
-6.1175156	11.1264000	103.0999985
-6.1175156	11.1752005	104.5299988
-6.1175156	11.2240000	105.9399948
-6.1175156	11.2728004	101.5000000
-6.1175156	11.3216000	104.1899948
-6.1175156	11.3704004	106.6800003
-6.1175156	11.4191999	107.9899979
-6.1175156	11.4680004	106.6399994
-6.1175156	11.5167999	105.5699997
-6.1175156	11.5656004	105.3600006
-6.1175156	11.6143999	104.0199966
-6.1175156	11.6632004	104.4199982
-6.1175156	11.7119999	104.7399979
-6.1175156	11.7608004	105.2999954
-6.1175156	11.8095999	104.2699966
-6.1175156	11.8584003	105.3899994
-6.1175156	11.9071999	104.1899948
-6.1175156	11.9560003	103.2500000
-6.1175156	12.0047998	103.5100021
-6.1175156	12.0536003	101.2099991
-6.1175156	12.1023998	104.0100021
-6.1175156	12.1512003	98.9899979
-6.1175156	12.1999998	103.6599960
-6.1175156	12.2488003	106.8099976
-6.1175156	12.2975998	107.6100006
-6.1175156	12.3464003	105.0800018
-6.1175156	12.3951998	102.2500000
-6.1175156	12.4440002	101.5000000
-6.1175156	12.4927998	104.1399994
-6.1175156	12.5416002	102.9700012
-6.1175156	12.5904007	100.9700012
-6.1175156	12.6392002	105.5999985
-6.1175156	12.6880007	107.8399963
-6.1175156	12.7368002	106.0899963
-6.1175156	12.7856007	102.2299957

-6.1175156	12.8344002	102.2799988
-6.1175156	12.8832006	102.5199966
-6.1175156	12.9320002	99.7799988
-6.1175156	12.9808006	103.0100021
-6.1175156	13.0296001	102.6199951
-6.1175156	13.0784006	102.9399948
-6.1175156	13.1272001	102.7399979
-6.1175156	13.1760006	99.5599976
-6.1175156	13.2248001	103.5400009
-6.1175156	13.2736006	101.4000015
-6.1175156	13.3224001	101.6399994
-6.1175156	13.3712006	105.2099991
-6.1175156	13.4200001	105.7299957
-6.1175156	13.4688005	104.9599991
-6.1175156	13.5176001	103.6800003
-6.1175156	13.5664005	104.0199966
-6.1175156	13.6152000	104.3300018
-6.1175156	13.6640005	105.1299973
-6.1175156	13.7128000	104.5599976
-6.1175156	13.7616005	101.4499969
-6.1175156	13.8104000	101.9199982
-6.1175156	13.8592005	101.7900009
-6.1175156	13.9080000	100.7200012
-6.1175156	13.9568005	103.3099976
-6.1175156	14.0056000	104.2399979
-6.1175156	14.0544004	105.1399994
-6.1175156	14.1032000	105.0800018
-6.1175156	14.1520004	104.0100021
-6.1175156	14.2007999	104.5000000
-6.1175156	14.2496004	103.8899994
-6.1175156	14.2983999	105.7799988
-6.1175156	14.3472004	105.1800003
-6.1175156	14.3959999	102.7600021
-6.1175156	14.4448004	98.7399979
-6.1175156	14.4935999	102.0299988
-6.1175156	14.5424004	103.7200012
-6.1175156	14.5911999	102.4300003
-6.1175156	14.6400003	100.9000015
-6.1175156	14.6887999	104.9000015
-6.1175156	14.7376003	104.6699982
-6.1175156	14.7863998	103.2999954
-6.1175156	14.8352003	101.8300018
-6.1175156	14.8839998	102.8499985
-6.1175156	14.9328003	103.0100021
-6.1175156	14.9815998	102.3499985
-6.1175156	15.0304003	101.6699982
-6.1113796	0.3416000	108.5699997
-6.1113796	0.3904000	109.8099976
-6.1113796	0.4392000	109.8099976
-6.1113796	0.4880000	107.5100021
-6.1113796	0.5368000	102.3099976
-6.1113796	0.5856000	104.3199997
-6.1113796	0.6344000	102.4300003
-6.1113796	0.6832000	105.5299988
-6.1113796	0.7320000	108.1800003
-6.1113796	0.7808000	108.7500000
-6.1113796	0.8296000	108.1899948
-6.1113796	0.8784000	109.2099991

-6.1113796	0.9272000	110.1100006
-6.1113796	0.9760000	110.8600006
-6.1113796	1.0247999	110.3699951
-6.1113796	1.0735999	107.9799957
-6.1113796	1.1223999	108.2799988
-6.1113796	1.1711999	106.0299988
-6.1113796	1.2200000	105.7399979
-6.1113796	1.2688000	106.8699951
-6.1113796	1.3176000	106.2099991
-6.1113796	1.3664000	103.4399948
-6.1113796	1.4152000	102.7799988
-6.1113796	1.4640000	103.9700012
-6.1113796	1.5128000	102.3600006
-6.1113796	1.5616000	101.0100021
-6.1113796	1.6104000	104.4700012
-6.1113796	1.6592000	103.5899963
-6.1113796	1.7079999	108.5499954
-6.1113796	1.7567999	109.8600006
-6.1113796	1.8055999	113.5800018
-6.1113796	1.8543999	120.1599960
-6.1113796	1.9031999	122.0199966
-6.1113796	1.9519999	117.0000000
-6.1113796	2.0007999	107.1500015
-6.1113796	2.0495999	107.0999985
-6.1113796	2.0983999	107.3199997
-6.1113796	2.1471999	104.0899963
-6.1113796	2.1959999	106.1399994
-6.1113796	2.2447999	106.0899963
-6.1113796	2.2935998	105.5800018
-6.1113796	2.3423998	104.4700012
-6.1113796	2.3912001	104.6899948
-6.1113796	2.4400001	104.4000015
-6.1113796	2.4888000	109.0599976
-6.1113796	2.5376000	108.0299988
-6.1113796	2.5864000	104.1500015
-6.1113796	2.6352000	102.5100021
-6.1113796	2.6840000	99.3399963
-6.1113796	2.7328000	100.1899948
-6.1113796	2.7816000	102.0400009
-6.1113796	2.8304000	105.1199951
-6.1113796	2.8792000	104.8600006
-6.1113796	2.9280000	106.8600006
-6.1113796	2.9768000	104.3600006
-6.1113796	3.0256000	105.4000015
-6.1113796	3.0743999	103.9099960
-6.1113796	3.1231999	98.6899948
-6.1113796	3.1719999	102.8699951
-6.1113796	3.2207999	104.7600021
-6.1113796	3.2695999	104.5000000
-6.1113796	3.3183999	106.7799988
-6.1113796	3.3671999	102.9799957
-6.1113796	3.4159999	104.1199951
-6.1113796	3.4647999	104.9499969
-6.1113796	3.5135999	103.7299957
-6.1113796	3.5623999	102.8600006
-6.1113796	3.6111999	103.3399963
-6.1113796	3.6599998	102.6999969
-6.1113796	3.7087998	102.4499969

-6.1113796	3.7575998	104.5699997
-6.1113796	3.8063998	104.5199966
-6.1113796	3.8551998	103.2099991
-6.1113796	3.9039998	104.8099976
-6.1113796	3.9527998	106.1199951
-6.1113796	4.0016003	104.6399994
-6.1113796	4.0503998	101.5899963
-6.1113796	4.0991998	102.7299957
-6.1113796	4.1479998	101.1100006
-6.1113796	4.1967998	104.3499985
-6.1113796	4.2455997	103.3499985
-6.1113796	4.2943997	107.7799988
-6.1113796	4.3431997	111.1299973
-6.1113796	4.3919997	111.1299973
-6.1113796	4.4407997	107.2699966
-6.1113796	4.4895997	107.5800018
-6.1113796	4.5383997	115.5100021
-6.1113796	4.5871997	119.7699966
-6.1113796	4.6359997	118.6599960
-6.1113796	4.6847997	116.8699951
-6.1113796	4.7336001	116.0800018
-6.1113796	4.7824001	112.1100006
-6.1113796	4.8312001	112.1899948
-6.1113796	4.8800001	112.9199982
-6.1113796	4.9288001	109.3600006
-6.1113796	4.9776001	108.7200012
-6.1113796	5.0264001	108.7200012
-6.1113796	5.0752001	111.4300003
-6.1113796	5.1240001	109.6999969
-6.1113796	5.1728001	109.1699982
-6.1113796	5.2216001	107.4499969
-6.1113796	5.2704000	103.9899979
-6.1113796	5.3192000	102.4700012
-6.1113796	5.3680000	106.4799957
-6.1113796	5.4168000	108.5699997
-6.1113796	5.4656000	107.6599960
-6.1113796	5.5144000	103.7799988
-6.1113796	5.5632000	100.8199997
-6.1113796	5.6120000	103.8399963
-6.1113796	5.6608000	106.2799988
-6.1113796	5.7096000	102.6199951
-6.1113796	5.7584000	102.9300003
-6.1113796	5.8072000	103.6500015
-6.1113796	5.8559999	103.0400009
-6.1113796	5.9047999	106.4499969
-6.1113796	5.9535999	108.3799973
-6.1113796	6.0023999	107.1699982
-6.1113796	6.0511999	107.0499954
-6.1113796	6.0999999	108.2900009
-6.1113796	6.1487999	107.0800018
-6.1113796	6.1975999	105.7799988
-6.1113796	6.2463999	101.9899979
-6.1113796	6.2951999	102.0599976
-6.1113796	6.3439999	104.0899963
-6.1113796	6.3927999	106.1599960
-6.1113796	6.4415998	107.3499985
-6.1113796	6.4903998	106.0100021
-6.1113796	6.5391998	104.9799957

-6.1113796	6.5879998	106.3199997
-6.1113796	6.6367998	104.5499954
-6.1113796	6.6855998	106.4799957
-6.1113796	6.7343998	105.7999954
-6.1113796	6.7831998	102.9300003
-6.1113796	6.8319998	106.2900009
-6.1113796	6.8807998	104.7699966
-6.1113796	6.9295998	105.4599991
-6.1113796	6.9783998	101.7600021
-6.1113796	7.0271997	100.9099960
-6.1113796	7.0759997	104.3300018
-6.1113796	7.1247997	103.6599960
-6.1113796	7.1735997	100.6399994
-6.1113796	7.2223997	102.3499985
-6.1113796	7.2711997	103.0299988
-6.1113796	7.3199997	105.1999969
-6.1113796	7.3687997	101.1899948
-6.1113796	7.4175997	103.2500000
-6.1113796	7.4663997	100.9499969
-6.1113796	7.5151997	102.3499985
-6.1113796	7.5639997	105.4300003
-6.1113796	7.6127996	106.2500000
-6.1113796	7.6615996	103.6699982
-6.1113796	7.7103996	100.4799957
-6.1113796	7.7591996	102.8699951
-6.1113796	7.8079996	103.9300003
-6.1113796	7.8567996	100.1100006
-6.1113796	7.9056001	101.5999985
-6.1113796	7.9544001	104.6899948
-6.1113796	8.0032005	104.4700012
-6.1113796	8.0520000	103.3799973
-6.1113796	8.1008005	102.2200012
-6.1113796	8.1496000	102.7500000
-6.1113796	8.1984005	102.8699951
-6.1113796	8.2472000	105.5400009
-6.1113796	8.2960005	106.7299957
-6.1113796	8.3448000	105.9799957
-6.1113796	8.3936005	103.1899948
-6.1113796	8.4424000	103.0899963
-6.1113796	8.4912004	104.3899994
-6.1113796	8.5400000	105.0599976
-6.1113796	8.5888004	105.1699982
-6.1113796	8.6375999	103.5199966
-6.1113796	8.6864004	103.5499954
-6.1113796	8.7351999	103.5999985
-6.1113796	8.7840004	103.6399994
-6.1113796	8.8327999	103.0400009
-6.1113796	8.8816004	103.4399948
-6.1113796	8.9303999	102.0499954
-6.1113796	8.9792004	102.5199966
-6.1113796	9.0279999	104.8199997
-6.1113796	9.0768003	103.3300018
-6.1113796	9.1255999	106.2799988
-6.1113796	9.1744003	105.8399963
-6.1113796	9.2231998	102.6299973
-6.1113796	9.2720003	99.3699951
-6.1113796	9.3207998	103.8799973
-6.1113796	9.3696003	102.6500015

-6.1113796	9.4184008	98.6199951
-6.1113796	9.4672003	101.0899963
-6.1113796	9.5160007	100.9899979
-6.1113796	9.5648003	103.1500015
-6.1113796	9.6136007	103.2299957
-6.1113796	9.6624002	103.5800018
-6.1113796	9.7112007	101.6599960
-6.1113796	9.7600002	105.5299988
-6.1113796	9.8088007	102.1999969
-6.1113796	9.8576002	98.9799957
-6.1113796	9.9064007	103.9599991
-6.1113796	9.9552002	106.9399948
-6.1113796	10.0040007	104.8699951
-6.1113796	10.0528002	101.2999954
-6.1113796	10.1016006	103.7200012
-6.1113796	10.1504002	103.5800018
-6.1113796	10.1992006	102.5699997
-6.1113796	10.2480001	102.4000015
-6.1113796	10.2968006	104.5800018
-6.1113796	10.3456001	104.9399948
-6.1113796	10.3944006	106.5100021
-6.1113796	10.4432001	106.4799957
-6.1113796	10.4920006	103.2999954
-6.1113796	10.5408001	102.9399948
-6.1113796	10.5896006	103.2399979
-6.1113796	10.6384001	100.0800018
-6.1113796	10.6872005	105.2600021
-6.1113796	10.7360001	105.8799973
-6.1113796	10.7848005	105.9499969
-6.1113796	10.8336000	106.7900009
-6.1113796	10.8824005	106.4799957
-6.1113796	10.9312000	104.2500000
-6.1113796	10.9800005	101.8799973
-6.1113796	11.0288000	101.8899994
-6.1113796	11.0776005	101.7099991
-6.1113796	11.1264000	103.2799988
-6.1113796	11.1752005	103.9300003
-6.1113796	11.2240000	105.8300018
-6.1113796	11.2728004	100.5999985
-6.1113796	11.3216000	103.3499985
-6.1113796	11.3704004	105.5100021
-6.1113796	11.4191999	107.4199982
-6.1113796	11.4680004	106.4000015
-6.1113796	11.5167999	105.5899963
-6.1113796	11.5656004	105.5400009
-6.1113796	11.6143999	103.8099976
-6.1113796	11.6632004	104.6599960
-6.1113796	11.7119999	104.3699951
-6.1113796	11.7608004	105.3899994
-6.1113796	11.8095999	105.2099991
-6.1113796	11.8584003	105.1599960
-6.1113796	11.9071999	104.3199997
-6.1113796	11.9560003	104.2900009
-6.1113796	12.0047998	102.7999954
-6.1113796	12.0536003	99.2299957
-6.1113796	12.1023998	103.9899979
-6.1113796	12.1512003	101.1399994
-6.1113796	12.1999998	104.0000000

-6.1113796	12.2488003	106.8399963
-6.1113796	12.2975998	107.0400009
-6.1113796	12.3464003	104.2900009
-6.1113796	12.3951998	101.6999969
-6.1113796	12.4440002	101.9499969
-6.1113796	12.4927998	104.5899963
-6.1113796	12.5416002	103.0699997
-6.1113796	12.5904007	101.0499954
-6.1113796	12.6392002	106.3699951
-6.1113796	12.6880007	107.9499969
-6.1113796	12.7368002	106.6699982
-6.1113796	12.7856007	103.0000000
-6.1113796	12.8344002	103.3699951
-6.1113796	12.8832006	103.0499954
-6.1113796	12.9320002	100.4000015
-6.1113796	12.9808006	103.4399948
-6.1113796	13.0296001	102.6500015
-6.1113796	13.0784006	102.8600006
-6.1113796	13.1272001	102.9399948
-6.1113796	13.1760006	100.9599991
-6.1113796	13.2248001	104.4700012
-6.1113796	13.2736006	102.4099960
-6.1113796	13.3224001	101.6599960
-6.1113796	13.3712006	105.0999985
-6.1113796	13.4200001	106.0299988
-6.1113796	13.4688005	104.5199966
-6.1113796	13.5176001	103.1399994
-6.1113796	13.5664005	104.5999985
-6.1113796	13.6152000	104.8899994
-6.1113796	13.6640005	105.0000000
-6.1113796	13.7128000	104.8300018
-6.1113796	13.7616005	102.5599976
-6.1113796	13.8104000	102.9399948
-6.1113796	13.8592005	102.7299957
-6.1113796	13.9080000	101.1999969
-6.1113796	13.9568005	103.6899948
-6.1113796	14.0056000	104.0499954
-6.1113796	14.0544004	104.9399948
-6.1113796	14.1032000	105.0100021
-6.1113796	14.1520004	104.4399948
-6.1113796	14.2007999	104.7200012
-6.1113796	14.2496004	104.5100021
-6.1113796	14.2983999	105.2699966
-6.1113796	14.3472004	104.6800003
-6.1113796	14.3959999	103.2399979
-6.1113796	14.4448004	99.0899963
-6.1113796	14.4935999	101.9000015
-6.1113796	14.5424004	103.4899979
-6.1113796	14.5911999	102.2399979
-6.1113796	14.6400003	99.8099976
-6.1113796	14.6887999	104.7999954
-6.1113796	14.7376003	104.9599991
-6.1113796	14.7863998	104.4099960
-6.1113796	14.8352003	102.0999985
-6.1113796	14.8839998	102.4300003
-6.1113796	14.9328003	102.9199982
-6.1113796	14.9815998	102.0000000
-6.1113796	15.0304003	101.4899979

-6.1052437	0.3416000	109.3399963
-6.1052437	0.3904000	110.1199951
-6.1052437	0.4392000	109.8600006
-6.1052437	0.4880000	107.8699951
-6.1052437	0.5368000	103.0499954
-6.1052437	0.5856000	104.0800018
-6.1052437	0.6344000	103.5400009
-6.1052437	0.6832000	106.0400009
-6.1052437	0.7320000	107.9000015
-6.1052437	0.7808000	109.0499954
-6.1052437	0.8296000	108.5800018
-6.1052437	0.8784000	108.8399963
-6.1052437	0.9272000	108.7099991
-6.1052437	0.9760000	110.1399994
-6.1052437	1.0247999	109.7699966
-6.1052437	1.0735999	107.2200012
-6.1052437	1.1223999	108.3899994
-6.1052437	1.1711999	106.5100021
-6.1052437	1.2200000	105.1800003
-6.1052437	1.2688000	106.5499954
-6.1052437	1.3176000	106.0199966
-6.1052437	1.3664000	103.8199997
-6.1052437	1.4152000	104.0100021
-6.1052437	1.4640000	104.6599960
-6.1052437	1.5128000	103.0100021
-6.1052437	1.5616000	101.7900009
-6.1052437	1.6104000	104.7399979
-6.1052437	1.6592000	102.8799973
-6.1052437	1.7079999	108.9300003
-6.1052437	1.7567999	110.2099991
-6.1052437	1.8055999	113.4000015
-6.1052437	1.8543999	119.6299973
-6.1052437	1.9031999	121.5999985
-6.1052437	1.9519999	116.4300003
-6.1052437	2.0007999	107.1500015
-6.1052437	2.0495999	106.6800003
-6.1052437	2.0983999	107.0299988
-6.1052437	2.1471999	103.5499954
-6.1052437	2.1959999	105.9799957
-6.1052437	2.2447999	106.2200012
-6.1052437	2.2935998	105.7600021
-6.1052437	2.3423998	104.8600006
-6.1052437	2.3912001	104.6299973
-6.1052437	2.4400001	104.3699951
-6.1052437	2.4888000	108.8600006
-6.1052437	2.5376000	107.7699966
-6.1052437	2.5864000	104.4499969
-6.1052437	2.6352000	103.0800018
-6.1052437	2.6840000	100.8099976
-6.1052437	2.7328000	101.2200012
-6.1052437	2.7816000	102.5800018
-6.1052437	2.8304000	105.5699997
-6.1052437	2.8792000	104.6399994
-6.1052437	2.9280000	106.8899994
-6.1052437	2.9768000	105.2200012
-6.1052437	3.0256000	106.3399963
-6.1052437	3.0743999	103.9099960
-6.1052437	3.1231999	98.3399963

-6.1052437	3.1719999	102.4000015
-6.1052437	3.2207999	103.6299973
-6.1052437	3.2695999	104.1800003
-6.1052437	3.3183999	106.4700012
-6.1052437	3.3671999	102.8600006
-6.1052437	3.4159999	103.8499985
-6.1052437	3.4647999	105.2200012
-6.1052437	3.5135999	103.7099991
-6.1052437	3.5623999	101.3899994
-6.1052437	3.6111999	101.9199982
-6.1052437	3.6599998	102.1500015
-6.1052437	3.7087998	103.3099976
-6.1052437	3.7575998	105.9599991
-6.1052437	3.8063998	105.3699951
-6.1052437	3.8551998	102.3799973
-6.1052437	3.9039998	104.7900009
-6.1052437	3.9527998	106.2999954
-6.1052437	4.0016003	104.8799973
-6.1052437	4.0503998	101.6599960
-6.1052437	4.0991998	102.8799973
-6.1052437	4.1479998	101.4899979
-6.1052437	4.1967998	103.9899979
-6.1052437	4.2455997	102.4700012
-6.1052437	4.2943997	107.2900009
-6.1052437	4.3431997	110.8699951
-6.1052437	4.3919997	110.8199997
-6.1052437	4.4407997	107.4300003
-6.1052437	4.4895997	107.1599960
-6.1052437	4.5383997	114.9399948
-6.1052437	4.5871997	118.5800018
-6.1052437	4.6359997	117.9099960
-6.1052437	4.6847997	116.0899963
-6.1052437	4.7336001	115.8499985
-6.1052437	4.7824001	111.6100006
-6.1052437	4.8312001	111.5299988
-6.1052437	4.8800001	112.1599960
-6.1052437	4.9288001	107.8099976
-6.1052437	4.9776001	107.5199966
-6.1052437	5.0264001	108.7900009
-6.1052437	5.0752001	111.0000000
-6.1052437	5.1240001	108.0699997
-6.1052437	5.1728001	108.1800003
-6.1052437	5.2216001	107.5599976
-6.1052437	5.2704000	104.6800003
-6.1052437	5.3192000	102.7999954
-6.1052437	5.3680000	107.0999985
-6.1052437	5.4168000	109.0299988
-6.1052437	5.4656000	108.1399994
-6.1052437	5.5144000	104.9300003
-6.1052437	5.5632000	101.1199951
-6.1052437	5.6120000	104.6800003
-6.1052437	5.6608000	106.7799988
-6.1052437	5.7096000	102.1100006
-6.1052437	5.7584000	102.1399994
-6.1052437	5.8072000	103.2399979
-6.1052437	5.8559999	102.5199966
-6.1052437	5.9047999	105.3600006
-6.1052437	5.9535999	108.4099960

-6.1052437	6.0023999	107.1299973
-6.1052437	6.0511999	106.5499954
-6.1052437	6.0999999	108.4499969
-6.1052437	6.1487999	107.5999985
-6.1052437	6.1975999	105.4499969
-6.1052437	6.2463999	101.3899994
-6.1052437	6.2951999	100.7999954
-6.1052437	6.3439999	103.5100021
-6.1052437	6.3927999	106.5899963
-6.1052437	6.4415998	106.6800003
-6.1052437	6.4903998	105.2799988
-6.1052437	6.5391998	103.8499985
-6.1052437	6.5879998	106.1899948
-6.1052437	6.6367998	105.1800003
-6.1052437	6.6855998	105.9000015
-6.1052437	6.7343998	105.2699966
-6.1052437	6.7831998	103.1199951
-6.1052437	6.8319998	106.2099991
-6.1052437	6.8807998	104.0999985
-6.1052437	6.9295998	104.9499969
-6.1052437	6.9783998	100.5699997
-6.1052437	7.0271997	102.1299973
-6.1052437	7.0759997	104.3799973
-6.1052437	7.1247997	103.5100021
-6.1052437	7.1735997	100.7699966
-6.1052437	7.2223997	101.9599991
-6.1052437	7.2711997	102.5699997
-6.1052437	7.3199997	105.8199997
-6.1052437	7.3687997	103.0100021
-6.1052437	7.4175997	103.8899994
-6.1052437	7.4663997	101.1299973
-6.1052437	7.5151997	102.1199951
-6.1052437	7.5639997	105.1599960
-6.1052437	7.6127996	106.8099976
-6.1052437	7.6615996	104.6100006
-6.1052437	7.7103996	101.2299957
-6.1052437	7.7591996	101.7600021
-6.1052437	7.8079996	104.1199951
-6.1052437	7.8567996	100.5499954
-6.1052437	7.9056001	100.6899948
-6.1052437	7.9544001	104.9199982
-6.1052437	8.0032005	105.1500015
-6.1052437	8.0520000	104.0599976
-6.1052437	8.1008005	102.9799957
-6.1052437	8.1496000	103.1699982
-6.1052437	8.1984005	102.2399979
-6.1052437	8.2472000	104.4499969
-6.1052437	8.2960005	106.2699966
-6.1052437	8.3448000	105.3799973
-6.1052437	8.3936005	101.4899979
-6.1052437	8.4424000	102.8499985
-6.1052437	8.4912004	104.6100006
-6.1052437	8.5400000	104.9199982
-6.1052437	8.5888004	104.6800003
-6.1052437	8.6375999	103.2500000
-6.1052437	8.6864004	103.1299973
-6.1052437	8.7351999	103.3699951
-6.1052437	8.7840004	104.1899948

-6.1052437	8.8327999	103.8600006
-6.1052437	8.8816004	103.1699982
-6.1052437	8.9303999	101.1899948
-6.1052437	8.9792004	101.4099960
-6.1052437	9.0279999	105.2600021
-6.1052437	9.0768003	104.7699966
-6.1052437	9.1255999	106.2699966
-6.1052437	9.1744003	105.7200012
-6.1052437	9.2231998	102.9300003
-6.1052437	9.2720003	99.2699966
-6.1052437	9.3207998	103.2799988
-6.1052437	9.3696003	102.5299988
-6.1052437	9.4184008	99.0000000
-6.1052437	9.4672003	101.4099960
-6.1052437	9.5160007	100.9399948
-6.1052437	9.5648003	102.6299973
-6.1052437	9.6136007	101.7900009
-6.1052437	9.6624002	102.9799957
-6.1052437	9.7112007	101.9399948
-6.1052437	9.7600002	104.9300003
-6.1052437	9.8088007	101.7099991
-6.1052437	9.8576002	99.4899979
-6.1052437	9.9064007	102.2200012
-6.1052437	9.9552002	105.4499969
-6.1052437	10.0040007	104.0299988
-6.1052437	10.0528002	100.7099991
-6.1052437	10.1016006	104.0100021
-6.1052437	10.1504002	103.5100021
-6.1052437	10.1992006	102.4399948
-6.1052437	10.2480001	101.2299957
-6.1052437	10.2968006	103.5299988
-6.1052437	10.3456001	104.0199966
-6.1052437	10.3944006	105.9700012
-6.1052437	10.4432001	106.3099976
-6.1052437	10.4920006	103.4399948
-6.1052437	10.5408001	102.9499969
-6.1052437	10.5896006	104.4499969
-6.1052437	10.6384001	101.4399948
-6.1052437	10.6872005	105.7900009
-6.1052437	10.7360001	106.5699997
-6.1052437	10.7848005	107.1500015
-6.1052437	10.8336000	107.4300003
-6.1052437	10.8824005	106.7200012
-6.1052437	10.9312000	103.7200012
-6.1052437	10.9800005	100.8699951
-6.1052437	11.0288000	103.5100021
-6.1052437	11.0776005	102.2500000
-6.1052437	11.1264000	103.1100006
-6.1052437	11.1752005	103.6999969
-6.1052437	11.2240000	106.1299973
-6.1052437	11.2728004	101.5100021
-6.1052437	11.3216000	102.2999954
-6.1052437	11.3704004	104.2600021
-6.1052437	11.4191999	106.8199997
-6.1052437	11.4680004	106.4499969
-6.1052437	11.5167999	106.1199951
-6.1052437	11.5656004	105.9199982
-6.1052437	11.6143999	103.9499969

-6.1052437	11.6632004	104.4799957
-6.1052437	11.7119999	102.8899994
-6.1052437	11.7608004	103.7699966
-6.1052437	11.8095999	105.0599976
-6.1052437	11.8584003	104.3899994
-6.1052437	11.9071999	104.6699982
-6.1052437	11.9560003	105.0000000
-6.1052437	12.0047998	102.0599976
-6.1052437	12.0536003	98.7200012
-6.1052437	12.1023998	103.6500015
-6.1052437	12.1512003	102.6899948
-6.1052437	12.1999998	104.0899963
-6.1052437	12.2488003	106.9799957
-6.1052437	12.2975998	107.0100021
-6.1052437	12.3464003	104.3199997
-6.1052437	12.3951998	102.0699997
-6.1052437	12.4440002	102.3399963
-6.1052437	12.4927998	104.0699997
-6.1052437	12.5416002	103.5299988
-6.1052437	12.5904007	101.7299957
-6.1052437	12.6392002	106.4099960
-6.1052437	12.6880007	107.8799973
-6.1052437	12.7368002	107.6699982
-6.1052437	12.7856007	104.0800018
-6.1052437	12.8344002	104.0199966
-6.1052437	12.8832006	102.7999954
-6.1052437	12.9320002	100.7200012
-6.1052437	12.9808006	103.7999954
-6.1052437	13.0296001	102.8799973
-6.1052437	13.0784006	103.5800018
-6.1052437	13.1272001	103.2799988
-6.1052437	13.1760006	102.2200012
-6.1052437	13.2248001	105.5100021
-6.1052437	13.2736006	103.4300003
-6.1052437	13.3224001	101.9099960
-6.1052437	13.3712006	104.9899979
-6.1052437	13.4200001	106.1199951
-6.1052437	13.4688005	103.8799973
-6.1052437	13.5176001	103.0599976
-6.1052437	13.5664005	105.1699982
-6.1052437	13.6152000	104.9099960
-6.1052437	13.6640005	104.1299973
-6.1052437	13.7128000	104.1299973
-6.1052437	13.7616005	102.7799988
-6.1052437	13.8104000	103.0499954
-6.1052437	13.8592005	103.0100021
-6.1052437	13.9080000	101.0199966
-6.1052437	13.9568005	103.2500000
-6.1052437	14.0056000	103.6399994
-6.1052437	14.0544004	104.9300003
-6.1052437	14.1032000	105.4599991
-6.1052437	14.1520004	105.3099976
-6.1052437	14.2007999	104.9000015
-6.1052437	14.2496004	104.5699997
-6.1052437	14.2983999	104.6999969
-6.1052437	14.3472004	104.5100021
-6.1052437	14.3959999	103.9799957
-6.1052437	14.4448004	100.6599960

-6.1052437	14.4935999	102.0800018
-6.1052437	14.5424004	103.3499985
-6.1052437	14.5911999	102.2500000
-6.1052437	14.6400003	98.9700012
-6.1052437	14.6887999	104.4899979
-6.1052437	14.7376003	105.2699966
-6.1052437	14.7863998	105.7299957
-6.1052437	14.8352003	102.5299988
-6.1052437	14.8839998	101.7799988
-6.1052437	14.9328003	102.3499985
-6.1052437	14.9815998	101.5000000
-6.1052437	15.0304003	101.2900009
-6.0991077	0.3416000	109.7799988
-6.0991077	0.3904000	110.3199997
-6.0991077	0.4392000	109.5599976
-6.0991077	0.4880000	107.6999969
-6.0991077	0.5368000	103.5800018
-6.0991077	0.5856000	103.9399948
-6.0991077	0.6344000	105.0100021
-6.0991077	0.6832000	106.7999954
-6.0991077	0.7320000	107.7900009
-6.0991077	0.7808000	109.1699982
-6.0991077	0.8296000	108.3399963
-6.0991077	0.8784000	108.1299973
-6.0991077	0.9272000	107.1999969
-6.0991077	0.9760000	109.1100006
-6.0991077	1.0247999	108.5699997
-6.0991077	1.0735999	106.1599960
-6.0991077	1.1223999	108.3300018
-6.0991077	1.1711999	106.7900009
-6.0991077	1.2200000	104.9199982
-6.0991077	1.2688000	106.4799957
-6.0991077	1.3176000	105.5699997
-6.0991077	1.3664000	104.6199951
-6.0991077	1.4152000	105.6599960
-6.0991077	1.4640000	105.3399963
-6.0991077	1.5128000	104.0899963
-6.0991077	1.5616000	104.1899948
-6.0991077	1.6104000	105.7299957
-6.0991077	1.6592000	103.4399948
-6.0991077	1.7079999	109.5100021
-6.0991077	1.7567999	110.5599976
-6.0991077	1.8055999	112.7299957
-6.0991077	1.8543999	118.9599991
-6.0991077	1.9031999	121.2600021
-6.0991077	1.9519999	116.0199966
-6.0991077	2.0007999	106.9700012
-6.0991077	2.0495999	105.6899948
-6.0991077	2.0983999	106.3600006
-6.0991077	2.1471999	103.4099960
-6.0991077	2.1959999	105.8499985
-6.0991077	2.2447999	105.8799973
-6.0991077	2.2935998	105.3099976
-6.0991077	2.3423998	104.6399994
-6.0991077	2.3912001	104.6299973
-6.0991077	2.4400001	104.2799988
-6.0991077	2.4888000	108.5800018
-6.0991077	2.5376000	107.6399994

-6.0991077	2.5864000	105.2399979
-6.0991077	2.6352000	103.4499969
-6.0991077	2.6840000	101.8499985
-6.0991077	2.7328000	102.0899963
-6.0991077	2.7816000	102.7299957
-6.0991077	2.8304000	106.2299957
-6.0991077	2.8792000	105.2200012
-6.0991077	2.9280000	106.7299957
-6.0991077	2.9768000	105.7699966
-6.0991077	3.0256000	106.7099991
-6.0991077	3.0743999	104.2200012
-6.0991077	3.1231999	99.5400009
-6.0991077	3.1719999	102.7699966
-6.0991077	3.2207999	102.8099976
-6.0991077	3.2695999	104.3300018
-6.0991077	3.3183999	106.2900009
-6.0991077	3.3671999	103.0999985
-6.0991077	3.4159999	104.0000000
-6.0991077	3.4647999	105.3199997
-6.0991077	3.5135999	103.5699997
-6.0991077	3.5623999	100.7099991
-6.0991077	3.6111999	100.8300018
-6.0991077	3.6599998	102.0400009
-6.0991077	3.7087998	104.4899979
-6.0991077	3.7575998	107.1100006
-6.0991077	3.8063998	105.9899979
-6.0991077	3.8551998	101.5100021
-6.0991077	3.9039998	104.5999985
-6.0991077	3.9527998	106.2900009
-6.0991077	4.0016003	105.2299957
-6.0991077	4.0503998	102.0499954
-6.0991077	4.0991998	103.4700012
-6.0991077	4.1479998	102.3399963
-6.0991077	4.1967998	104.0699997
-6.0991077	4.2455997	101.4300003
-6.0991077	4.2943997	106.8899994
-6.0991077	4.3431997	110.5999985
-6.0991077	4.3919997	110.5999985
-6.0991077	4.4407997	107.4899979
-6.0991077	4.4895997	107.0699997
-6.0991077	4.5383997	114.4799957
-6.0991077	4.5871997	117.1899948
-6.0991077	4.6359997	117.1599960
-6.0991077	4.6847997	115.4799957
-6.0991077	4.7336001	115.8099976
-6.0991077	4.7824001	111.3600006
-6.0991077	4.8312001	110.9000015
-6.0991077	4.8800001	111.5000000
-6.0991077	4.9288001	106.6899948
-6.0991077	4.9776001	106.8699951
-6.0991077	5.0264001	108.5699997
-6.0991077	5.0752001	110.5000000
-6.0991077	5.1240001	106.5499954
-6.0991077	5.1728001	107.2099991
-6.0991077	5.2216001	107.4799957
-6.0991077	5.2704000	105.2699966
-6.0991077	5.3192000	103.5199966
-6.0991077	5.3680000	107.4599991

-6.0991077	5.4168000	109.0599976
-6.0991077	5.4656000	107.9499969
-6.0991077	5.5144000	105.3600006
-6.0991077	5.5632000	102.1199951
-6.0991077	5.6120000	105.2699966
-6.0991077	5.6608000	106.9499969
-6.0991077	5.7096000	101.8899994
-6.0991077	5.7584000	101.5999985
-6.0991077	5.8072000	102.7900009
-6.0991077	5.8559999	102.0800018
-6.0991077	5.9047999	103.8199997
-6.0991077	5.9535999	107.6399994
-6.0991077	6.0023999	106.5499954
-6.0991077	6.0511999	105.4799957
-6.0991077	6.0999999	107.9599991
-6.0991077	6.1487999	107.7699966
-6.0991077	6.1975999	105.3300018
-6.0991077	6.2463999	101.2299957
-6.0991077	6.2951999	99.9799957
-6.0991077	6.3439999	102.5800018
-6.0991077	6.3927999	106.2399979
-6.0991077	6.4415998	105.4599991
-6.0991077	6.4903998	104.4000015
-6.0991077	6.5391998	103.0400009
-6.0991077	6.5879998	105.9799957
-6.0991077	6.6367998	105.8699951
-6.0991077	6.6855998	105.6699982
-6.0991077	6.7343998	104.6399994
-6.0991077	6.7831998	102.6199951
-6.0991077	6.8319998	105.5899963
-6.0991077	6.8807998	103.5699997
-6.0991077	6.9295998	104.9899979
-6.0991077	6.9783998	101.9700012
-6.0991077	7.0271997	103.5699997
-6.0991077	7.0759997	104.4399948
-6.0991077	7.1247997	103.9599991
-6.0991077	7.1735997	102.1100006
-6.0991077	7.2223997	102.5199966
-6.0991077	7.2711997	103.3499985
-6.0991077	7.3199997	106.5400009
-6.0991077	7.3687997	104.9199982
-6.0991077	7.4175997	105.1899948
-6.0991077	7.4663997	101.6399994
-6.0991077	7.5151997	101.8699951
-6.0991077	7.5639997	104.9099960
-6.0991077	7.6127996	107.3099976
-6.0991077	7.6615996	105.3099976
-6.0991077	7.7103996	102.0100021
-6.0991077	7.7591996	100.5499954
-6.0991077	7.8079996	103.8899994
-6.0991077	7.8567996	101.6199951
-6.0991077	7.9056001	100.9000015
-6.0991077	7.9544001	105.7299957
-6.0991077	8.0032005	105.9499969
-6.0991077	8.0520000	104.5100021
-6.0991077	8.1008005	103.4700012
-6.0991077	8.1496000	103.3199997
-6.0991077	8.1984005	101.8699951

-6.0991077	8.2472000	103.7500000
-6.0991077	8.2960005	105.5400009
-6.0991077	8.3448000	103.9199982
-6.0991077	8.3936005	99.4799957
-6.0991077	8.4424000	102.6399994
-6.0991077	8.4912004	104.2699966
-6.0991077	8.5400000	105.1500015
-6.0991077	8.5888004	104.9499969
-6.0991077	8.6375999	103.9199982
-6.0991077	8.6864004	103.4199982
-6.0991077	8.7351999	103.9700012
-6.0991077	8.7840004	105.0800018
-6.0991077	8.8327999	105.0299988
-6.0991077	8.8816004	103.4399948
-6.0991077	8.9303999	101.1899948
-6.0991077	8.9792004	101.3499985
-6.0991077	9.0279999	105.6100006
-6.0991077	9.0768003	105.8399963
-6.0991077	9.1255999	105.7999954
-6.0991077	9.1744003	105.9000015
-6.0991077	9.2231998	103.3699951
-6.0991077	9.2720003	100.1899948
-6.0991077	9.3207998	103.3899994
-6.0991077	9.3696003	102.4499969
-6.0991077	9.4184008	99.5599976
-6.0991077	9.4672003	100.8099976
-6.0991077	9.5160007	99.7999954
-6.0991077	9.5648003	101.7600021
-6.0991077	9.6136007	100.8799973
-6.0991077	9.6624002	102.5100021
-6.0991077	9.7112007	101.7200012
-6.0991077	9.7600002	104.2900009
-6.0991077	9.8088007	101.8899994
-6.0991077	9.8576002	100.1100006
-6.0991077	9.9064007	100.3600006
-6.0991077	9.9552002	104.0400009
-6.0991077	10.0040007	103.5299988
-6.0991077	10.0528002	101.5999985
-6.0991077	10.1016006	104.4799957
-6.0991077	10.1504002	103.2799988
-6.0991077	10.1992006	102.0899963
-6.0991077	10.2480001	100.2600021
-6.0991077	10.2968006	101.9700012
-6.0991077	10.3456001	103.4099960
-6.0991077	10.3944006	104.9599991
-6.0991077	10.4432001	105.7399979
-6.0991077	10.4920006	103.4000015
-6.0991077	10.5408001	103.0299988
-6.0991077	10.5896006	105.1399994
-6.0991077	10.6384001	102.9899979
-6.0991077	10.6872005	106.2399979
-6.0991077	10.7360001	107.0400009
-6.0991077	10.7848005	107.7600021
-6.0991077	10.8336000	107.1399994
-6.0991077	10.8824005	106.2399979
-6.0991077	10.9312000	103.1500015
-6.0991077	10.9800005	100.0800018
-6.0991077	11.0288000	104.5100021

-6.0991077	11.0776005	101.9799957
-6.0991077	11.1264000	102.7099991
-6.0991077	11.1752005	102.6999969
-6.0991077	11.2240000	105.3699951
-6.0991077	11.2728004	102.1899948
-6.0991077	11.3216000	102.4899979
-6.0991077	11.3704004	103.7900009
-6.0991077	11.4191999	106.3399963
-6.0991077	11.4680004	106.1999969
-6.0991077	11.5167999	106.0699997
-6.0991077	11.5656004	105.7099991
-6.0991077	11.6143999	103.8399963
-6.0991077	11.6632004	104.2799988
-6.0991077	11.7119999	101.7799988
-6.0991077	11.7608004	102.1699982
-6.0991077	11.8095999	104.9399948
-6.0991077	11.8584003	104.0499954
-6.0991077	11.9071999	104.4499969
-6.0991077	11.9560003	104.7699966
-6.0991077	12.0047998	101.1100006
-6.0991077	12.0536003	98.4399948
-6.0991077	12.1023998	103.2799988
-6.0991077	12.1512003	104.1100006
-6.0991077	12.1999998	104.1599960
-6.0991077	12.2488003	107.0400009
-6.0991077	12.2975998	107.0400009
-6.0991077	12.3464003	104.8799973
-6.0991077	12.3951998	102.5899963
-6.0991077	12.4440002	102.1500015
-6.0991077	12.4927998	103.3399963
-6.0991077	12.5416002	103.2699966
-6.0991077	12.5904007	101.6899948
-6.0991077	12.6392002	105.9899979
-6.0991077	12.6880007	106.9300003
-6.0991077	12.7368002	107.8600006
-6.0991077	12.7856007	104.5800018
-6.0991077	12.8344002	104.0400009
-6.0991077	12.8832006	102.9499969
-6.0991077	12.9320002	101.5199966
-6.0991077	12.9808006	104.3499985
-6.0991077	13.0296001	103.4300003
-6.0991077	13.0784006	103.9300003
-6.0991077	13.1272001	103.5899963
-6.0991077	13.1760006	103.8899994
-6.0991077	13.2248001	106.5100021
-6.0991077	13.2736006	104.2799988
-6.0991077	13.3224001	102.2200012
-6.0991077	13.3712006	104.9000015
-6.0991077	13.4200001	106.0199966
-6.0991077	13.4688005	103.8899994
-6.0991077	13.5176001	102.6599960
-6.0991077	13.5664005	104.8300018
-6.0991077	13.6152000	104.5999985
-6.0991077	13.6640005	103.4099960
-6.0991077	13.7128000	103.9799957
-6.0991077	13.7616005	103.4899979
-6.0991077	13.8104000	103.9199982
-6.0991077	13.8592005	103.4499969

-6.0991077	13.9080000	100.9599991
-6.0991077	13.9568005	102.9499969
-6.0991077	14.0056000	103.8899994
-6.0991077	14.0544004	105.3099976
-6.0991077	14.1032000	105.8199997
-6.0991077	14.1520004	106.0599976
-6.0991077	14.2007999	105.1599960
-6.0991077	14.2496004	104.6500015
-6.0991077	14.2983999	104.0199966
-6.0991077	14.3472004	104.1999969
-6.0991077	14.3959999	103.7200012
-6.0991077	14.4448004	101.1699982
-6.0991077	14.4935999	101.8300018
-6.0991077	14.5424004	103.1999969
-6.0991077	14.5911999	102.4700012
-6.0991077	14.6400003	98.4199982
-6.0991077	14.6887999	103.6899948
-6.0991077	14.7376003	104.8899994
-6.0991077	14.7863998	106.5499954
-6.0991077	14.8352003	103.2900009
-6.0991077	14.8839998	101.6399994
-6.0991077	14.9328003	102.2999954
-6.0991077	14.9815998	101.4300003
-6.0991077	15.0304003	101.4300003
-6.0929718	0.3416000	109.8300018
-6.0929718	0.3904000	110.1100006
-6.0929718	0.4392000	108.7299957
-6.0929718	0.4880000	107.4899979
-6.0929718	0.5368000	104.9199982
-6.0929718	0.5856000	104.1199951
-6.0929718	0.6344000	106.0699997
-6.0929718	0.6832000	107.4399948
-6.0929718	0.7320000	107.8399963
-6.0929718	0.7808000	109.0499954
-6.0929718	0.8296000	107.2399979
-6.0929718	0.8784000	105.7699966
-6.0929718	0.9272000	105.0199966
-6.0929718	0.9760000	107.6599960
-6.0929718	1.0247999	107.3499985
-6.0929718	1.0735999	105.7299957
-6.0929718	1.1223999	108.9799957
-6.0929718	1.1711999	107.2999954
-6.0929718	1.2200000	104.8600006
-6.0929718	1.2688000	106.4300003
-6.0929718	1.3176000	105.2200012
-6.0929718	1.3664000	105.4000015
-6.0929718	1.4152000	107.3499985
-6.0929718	1.4640000	106.6800003
-6.0929718	1.5128000	105.7699966
-6.0929718	1.5616000	106.3199997
-6.0929718	1.6104000	107.2999954
-6.0929718	1.6592000	104.7099991
-6.0929718	1.7079999	110.1299973
-6.0929718	1.7567999	110.5199966
-6.0929718	1.8055999	111.7500000
-6.0929718	1.8543999	118.5800018
-6.0929718	1.9031999	121.0599976
-6.0929718	1.9519999	115.9499969

-6.0929718	2.0007999	106.9399948
-6.0929718	2.0495999	104.4700012
-6.0929718	2.0983999	105.2399979
-6.0929718	2.1471999	103.2200012
-6.0929718	2.1959999	105.7500000
-6.0929718	2.2447999	105.7799988
-6.0929718	2.2935998	105.4799957
-6.0929718	2.3423998	104.5400009
-6.0929718	2.3912001	104.9799957
-6.0929718	2.4400001	104.7699966
-6.0929718	2.4888000	108.4300003
-6.0929718	2.5376000	106.5199966
-6.0929718	2.5864000	105.7500000
-6.0929718	2.6352000	103.8300018
-6.0929718	2.6840000	102.3499985
-6.0929718	2.7328000	102.8199997
-6.0929718	2.7816000	102.8399963
-6.0929718	2.8304000	106.5299988
-6.0929718	2.8792000	105.6699982
-6.0929718	2.9280000	106.8799973
-6.0929718	2.9768000	106.4300003
-6.0929718	3.0256000	106.9499969
-6.0929718	3.0743999	104.5299988
-6.0929718	3.1231999	101.0299988
-6.0929718	3.1719999	103.4599991
-6.0929718	3.2207999	101.8099976
-6.0929718	3.2695999	103.8499985
-6.0929718	3.3183999	105.4199982
-6.0929718	3.3671999	102.8300018
-6.0929718	3.4159999	103.4399948
-6.0929718	3.4647999	104.8699951
-6.0929718	3.5135999	103.4899979
-6.0929718	3.5623999	100.3499985
-6.0929718	3.6111999	100.1399994
-6.0929718	3.6599998	102.5800018
-6.0929718	3.7087998	105.8099976
-6.0929718	3.7575998	107.8199997
-6.0929718	3.8063998	106.0100021
-6.0929718	3.8551998	100.4899979
-6.0929718	3.9039998	104.2999954
-6.0929718	3.9527998	106.4099960
-6.0929718	4.0016003	106.2299957
-6.0929718	4.0503998	103.4899979
-6.0929718	4.0991998	103.8399963
-6.0929718	4.1479998	102.9399948
-6.0929718	4.1967998	103.7999954
-6.0929718	4.2455997	100.7999954
-6.0929718	4.2943997	106.8499985
-6.0929718	4.3431997	110.1199951
-6.0929718	4.3919997	109.4000015
-6.0929718	4.4407997	107.8300018
-6.0929718	4.4895997	107.6599960
-6.0929718	4.5383997	113.6999969
-6.0929718	4.5871997	115.2999954
-6.0929718	4.6359997	115.9700012
-6.0929718	4.6847997	114.6699982
-6.0929718	4.7336001	115.5199966
-6.0929718	4.7824001	110.8699951

-6.0929718	4.8312001	110.3199997
-6.0929718	4.8800001	110.9899979
-6.0929718	4.9288001	106.1100006
-6.0929718	4.9776001	106.4300003
-6.0929718	5.0264001	108.6100006
-6.0929718	5.0752001	110.1500015
-6.0929718	5.1240001	106.2500000
-6.0929718	5.1728001	106.6399994
-6.0929718	5.2216001	107.5499954
-6.0929718	5.2704000	106.8199997
-6.0929718	5.3192000	104.7900009
-6.0929718	5.3680000	107.3499985
-6.0929718	5.4168000	108.9399948
-6.0929718	5.4656000	108.0599976
-6.0929718	5.5144000	106.2299957
-6.0929718	5.5632000	103.4399948
-6.0929718	5.6120000	105.1999969
-6.0929718	5.6608000	106.8199997
-6.0929718	5.7096000	102.2299957
-6.0929718	5.7584000	101.0199966
-6.0929718	5.8072000	101.9099960
-6.0929718	5.8559999	101.5800018
-6.0929718	5.9047999	102.0800018
-6.0929718	5.9535999	106.5699997
-6.0929718	6.0023999	105.5999985
-6.0929718	6.0511999	104.5999985
-6.0929718	6.0999999	107.6199951
-6.0929718	6.1487999	107.7399979
-6.0929718	6.1975999	105.1599960
-6.0929718	6.2463999	101.3600006
-6.0929718	6.2951999	98.8399963
-6.0929718	6.3439999	102.7999954
-6.0929718	6.3927999	106.6399994
-6.0929718	6.4415998	105.0899963
-6.0929718	6.4903998	104.2500000
-6.0929718	6.5391998	102.6999969
-6.0929718	6.5879998	105.7699966
-6.0929718	6.6367998	106.1599960
-6.0929718	6.6855998	104.7799988
-6.0929718	6.7343998	103.5599976
-6.0929718	6.7831998	101.6500015
-6.0929718	6.8319998	104.8799973
-6.0929718	6.8807998	103.8600006
-6.0929718	6.9295998	105.6699982
-6.0929718	6.9783998	103.3499985
-6.0929718	7.0271997	104.6299973
-6.0929718	7.0759997	104.2600021
-6.0929718	7.1247997	104.1299973
-6.0929718	7.1735997	103.2099991
-6.0929718	7.2223997	103.2999954
-6.0929718	7.2711997	104.1500015
-6.0929718	7.3199997	106.8699951
-6.0929718	7.3687997	106.4199982
-6.0929718	7.4175997	106.6399994
-6.0929718	7.4663997	103.0000000
-6.0929718	7.5151997	102.1899948
-6.0929718	7.5639997	104.4799957
-6.0929718	7.6127996	107.5599976

-6.0929718	7.6615996	105.7200012
-6.0929718	7.7103996	103.0100021
-6.0929718	7.7591996	100.0000000
-6.0929718	7.8079996	103.7600021
-6.0929718	7.8567996	102.4599991
-6.0929718	7.9056001	100.9199982
-6.0929718	7.9544001	105.9599991
-6.0929718	8.0032005	106.2299957
-6.0929718	8.0520000	105.0299988
-6.0929718	8.1008005	104.3499985
-6.0929718	8.1496000	103.6100006
-6.0929718	8.1984005	102.3199997
-6.0929718	8.2472000	102.7699966
-6.0929718	8.2960005	104.4099960
-6.0929718	8.3448000	102.5599976
-6.0929718	8.3936005	98.2799988
-6.0929718	8.4424000	103.0400009
-6.0929718	8.4912004	104.2500000
-6.0929718	8.5400000	105.5400009
-6.0929718	8.5888004	105.0699997
-6.0929718	8.6375999	104.4399948
-6.0929718	8.6864004	103.7900009
-6.0929718	8.7351999	104.5699997
-6.0929718	8.7840004	106.0599976
-6.0929718	8.8327999	106.5299988
-6.0929718	8.8816004	104.1999969
-6.0929718	8.9303999	101.6500015
-6.0929718	8.9792004	101.4000015
-6.0929718	9.0279999	105.9199982
-6.0929718	9.0768003	106.6800003
-6.0929718	9.1255999	104.8300018
-6.0929718	9.1744003	105.4599991
-6.0929718	9.2231998	104.2399979
-6.0929718	9.2720003	101.8499985
-6.0929718	9.3207998	103.4599991
-6.0929718	9.3696003	102.2500000
-6.0929718	9.4184008	99.9899979
-6.0929718	9.4672003	100.2099991
-6.0929718	9.5160007	99.6199951
-6.0929718	9.5648003	100.4399948
-6.0929718	9.6136007	99.2399979
-6.0929718	9.6624002	102.3300018
-6.0929718	9.7112007	101.6599960
-6.0929718	9.7600002	103.7399979
-6.0929718	9.8088007	102.4099960
-6.0929718	9.8576002	101.3699951
-6.0929718	9.9064007	99.5299988
-6.0929718	9.9552002	103.1199951
-6.0929718	10.0040007	103.3399963
-6.0929718	10.0528002	102.4199982
-6.0929718	10.1016006	104.7799988
-6.0929718	10.1504002	103.4700012
-6.0929718	10.1992006	101.8499985
-6.0929718	10.2480001	99.0400009
-6.0929718	10.2968006	99.7799988
-6.0929718	10.3456001	102.8399963
-6.0929718	10.3944006	104.3099976
-6.0929718	10.4432001	105.2799988

-6.0929718	10.4920006	103.8499985
-6.0929718	10.5408001	104.2500000
-6.0929718	10.5896006	106.3300018
-6.0929718	10.6384001	104.4599991
-6.0929718	10.6872005	106.2200012
-6.0929718	10.7360001	106.6100006
-6.0929718	10.7848005	107.1199951
-6.0929718	10.8336000	105.9300003
-6.0929718	10.8824005	105.0299988
-6.0929718	10.9312000	102.4499969
-6.0929718	10.9800005	99.0499954
-6.0929718	11.0288000	103.9499969
-6.0929718	11.0776005	101.5499954
-6.0929718	11.1264000	102.4700012
-6.0929718	11.1752005	101.1899948
-6.0929718	11.2240000	104.0899963
-6.0929718	11.2728004	102.0699997
-6.0929718	11.3216000	102.7399979
-6.0929718	11.3704004	104.1399994
-6.0929718	11.4191999	106.0699997
-6.0929718	11.4680004	106.0999985
-6.0929718	11.5167999	106.3799973
-6.0929718	11.5656004	105.4799957
-6.0929718	11.6143999	102.9599991
-6.0929718	11.6632004	104.0699997
-6.0929718	11.7119999	101.0499954
-6.0929718	11.7608004	100.6500015
-6.0929718	11.8095999	104.6299973
-6.0929718	11.8584003	103.9700012
-6.0929718	11.9071999	103.8600006
-6.0929718	11.9560003	104.3300018
-6.0929718	12.0047998	101.1100006
-6.0929718	12.0536003	99.1100006
-6.0929718	12.1023998	103.7699966
-6.0929718	12.1512003	105.7500000
-6.0929718	12.1999998	103.3600006
-6.0929718	12.2488003	106.8600006
-6.0929718	12.2975998	107.2099991
-6.0929718	12.3464003	105.3099976
-6.0929718	12.3951998	102.7200012
-6.0929718	12.4440002	101.3300018
-6.0929718	12.4927998	102.7600021
-6.0929718	12.5416002	103.5000000
-6.0929718	12.5904007	102.0999985
-6.0929718	12.6392002	105.4300003
-6.0929718	12.6880007	105.5400009
-6.0929718	12.7368002	107.6800003
-6.0929718	12.7856007	105.6500015
-6.0929718	12.8344002	104.3499985
-6.0929718	12.8832006	103.4300003
-6.0929718	12.9320002	103.4700012
-6.0929718	12.9808006	105.2299957
-6.0929718	13.0296001	103.8699951
-6.0929718	13.0784006	104.1699982
-6.0929718	13.1272001	104.3600006
-6.0929718	13.1760006	105.2399979
-6.0929718	13.2248001	106.9099960
-6.0929718	13.2736006	104.3499985

-6.0929718	13.3224001	101.6399994
-6.0929718	13.3712006	104.7699966
-6.0929718	13.4200001	106.4099960
-6.0929718	13.4688005	104.4300003
-6.0929718	13.5176001	103.5000000
-6.0929718	13.5664005	104.7900009
-6.0929718	13.6152000	104.5100021
-6.0929718	13.6640005	102.9300003
-6.0929718	13.7128000	103.3899994
-6.0929718	13.7616005	104.1699982
-6.0929718	13.8104000	104.3699951
-6.0929718	13.8592005	103.3099976
-6.0929718	13.9080000	100.2200012
-6.0929718	13.9568005	102.0100021
-6.0929718	14.0056000	104.3099976
-6.0929718	14.0544004	105.8099976
-6.0929718	14.1032000	106.0699997
-6.0929718	14.1520004	106.2699966
-6.0929718	14.2007999	105.0499954
-6.0929718	14.2496004	104.7600021
-6.0929718	14.2983999	103.4799957
-6.0929718	14.3472004	103.7999954
-6.0929718	14.3959999	103.0999985
-6.0929718	14.4448004	102.1100006
-6.0929718	14.4935999	102.5100021
-6.0929718	14.5424004	103.3199997
-6.0929718	14.5911999	102.7299957
-6.0929718	14.6400003	98.8600006
-6.0929718	14.6887999	103.0599976
-6.0929718	14.7376003	105.3499985
-6.0929718	14.7863998	107.4099960
-6.0929718	14.8352003	104.3699951
-6.0929718	14.8839998	102.4599991
-6.0929718	14.9328003	102.8099976
-6.0929718	14.9815998	102.3799973
-6.0929718	15.0304003	101.6599960
-6.0868359	0.3416000	109.1599960
-6.0868359	0.3904000	109.4700012
-6.0868359	0.4392000	107.3099976
-6.0868359	0.4880000	106.8199997
-6.0868359	0.5368000	105.8899994
-6.0868359	0.5856000	104.2900009
-6.0868359	0.6344000	107.3099976
-6.0868359	0.6832000	108.1500015
-6.0868359	0.7320000	108.5100021
-6.0868359	0.7808000	109.0699997
-6.0868359	0.8296000	105.5999985
-6.0868359	0.8784000	103.3499985
-6.0868359	0.9272000	103.6999969
-6.0868359	0.9760000	107.4000015
-6.0868359	1.0247999	106.9799957
-6.0868359	1.0735999	105.2799988
-6.0868359	1.1223999	109.2099991
-6.0868359	1.1711999	107.4000015
-6.0868359	1.2200000	105.0699997
-6.0868359	1.2688000	106.3799973
-6.0868359	1.3176000	104.7299957
-6.0868359	1.3664000	105.9000015

-6.0868359	1.4152000	108.4599991
-6.0868359	1.4640000	107.6299973
-6.0868359	1.5128000	107.3399963
-6.0868359	1.5616000	108.2299957
-6.0868359	1.6104000	108.6500015
-6.0868359	1.6592000	106.2699966
-6.0868359	1.7079999	109.9499969
-6.0868359	1.7567999	109.5400009
-6.0868359	1.8055999	110.3899994
-6.0868359	1.8543999	117.5499954
-6.0868359	1.9031999	120.1399994
-6.0868359	1.9519999	115.3699951
-6.0868359	2.0007999	106.7099991
-6.0868359	2.0495999	104.3600006
-6.0868359	2.0983999	105.0899963
-6.0868359	2.1471999	104.4499969
-6.0868359	2.1959999	106.0499954
-6.0868359	2.2447999	105.8199997
-6.0868359	2.2935998	105.9399948
-6.0868359	2.3423998	104.3199997
-6.0868359	2.3912001	105.4799957
-6.0868359	2.4400001	105.7200012
-6.0868359	2.4888000	108.0699997
-6.0868359	2.5376000	104.7699966
-6.0868359	2.5864000	106.1899948
-6.0868359	2.6352000	104.8699951
-6.0868359	2.6840000	103.0100021
-6.0868359	2.7328000	103.4700012
-6.0868359	2.7816000	102.5299988
-6.0868359	2.8304000	106.4700012
-6.0868359	2.8792000	106.1599960
-6.0868359	2.9280000	106.3199997
-6.0868359	2.9768000	105.8099976
-6.0868359	3.0256000	106.7799988
-6.0868359	3.0743999	105.1599960
-6.0868359	3.1231999	102.5599976
-6.0868359	3.1719999	103.6199951
-6.0868359	3.2207999	101.1599960
-6.0868359	3.2695999	103.8899994
-6.0868359	3.3183999	104.7399979
-6.0868359	3.3671999	102.7299957
-6.0868359	3.4159999	102.9099960
-6.0868359	3.4647999	104.4099960
-6.0868359	3.5135999	103.0899963
-6.0868359	3.5623999	100.3699951
-6.0868359	3.6111999	100.6199951
-6.0868359	3.6599998	103.3899994
-6.0868359	3.7087998	106.5199966
-6.0868359	3.7575998	108.1999969
-6.0868359	3.8063998	105.2399979
-6.0868359	3.8551998	99.9700012
-6.0868359	3.9039998	104.3499985
-6.0868359	3.9527998	106.3399963
-6.0868359	4.0016003	107.2099991
-6.0868359	4.0503998	105.0499954
-6.0868359	4.0991998	104.4899979
-6.0868359	4.1479998	103.7999954
-6.0868359	4.1967998	103.5199966

-6.0868359	4.2455997	101.2500000
-6.0868359	4.2943997	107.5699997
-6.0868359	4.3431997	110.1299973
-6.0868359	4.3919997	108.3699951
-6.0868359	4.4407997	108.4700012
-6.0868359	4.4895997	108.9700012
-6.0868359	4.5383997	113.1299973
-6.0868359	4.5871997	113.4899979
-6.0868359	4.6359997	114.8099976
-6.0868359	4.6847997	115.2699966
-6.0868359	4.7336001	116.2399979
-6.0868359	4.7824001	111.2799988
-6.0868359	4.8312001	109.2799988
-6.0868359	4.8800001	109.4499969
-6.0868359	4.9288001	106.5000000
-6.0868359	4.9776001	105.9499969
-6.0868359	5.0264001	107.9599991
-6.0868359	5.0752001	108.8300018
-6.0868359	5.1240001	106.5100021
-6.0868359	5.1728001	106.2200012
-6.0868359	5.2216001	107.1800003
-6.0868359	5.2704000	107.5899963
-6.0868359	5.3192000	105.5999985
-6.0868359	5.3680000	106.3699951
-6.0868359	5.4168000	107.7399979
-6.0868359	5.4656000	106.9899979
-6.0868359	5.5144000	106.0599976
-6.0868359	5.5632000	103.6599960
-6.0868359	5.6120000	103.6800003
-6.0868359	5.6608000	106.1500015
-6.0868359	5.7096000	103.3199997
-6.0868359	5.7584000	102.0499954
-6.0868359	5.8072000	102.6100006
-6.0868359	5.8559999	101.9399948
-6.0868359	5.9047999	100.9099960
-6.0868359	5.9535999	105.6699982
-6.0868359	6.0023999	105.5699997
-6.0868359	6.0511999	104.5699997
-6.0868359	6.0999999	107.3799973
-6.0868359	6.1487999	107.5499954
-6.0868359	6.1975999	104.8799973
-6.0868359	6.2463999	102.2099991
-6.0868359	6.2951999	98.5499954
-6.0868359	6.3439999	103.0899963
-6.0868359	6.3927999	106.7500000
-6.0868359	6.4415998	105.0000000
-6.0868359	6.4903998	104.5400009
-6.0868359	6.5391998	102.7399979
-6.0868359	6.5879998	105.3300018
-6.0868359	6.6367998	105.7900009
-6.0868359	6.6855998	104.0000000
-6.0868359	6.7343998	102.7699966
-6.0868359	6.7831998	100.1899948
-6.0868359	6.8319998	103.7999954
-6.0868359	6.8807998	104.1100006
-6.0868359	6.9295998	105.9799957
-6.0868359	6.9783998	104.5100021
-6.0868359	7.0271997	105.2099991

-6.0868359	7.0759997	104.0499954
-6.0868359	7.1247997	104.4099960
-6.0868359	7.1735997	103.9700012
-6.0868359	7.2223997	104.0000000
-6.0868359	7.2711997	105.1299973
-6.0868359	7.3199997	106.7399979
-6.0868359	7.3687997	106.7200012
-6.0868359	7.4175997	107.1800003
-6.0868359	7.4663997	103.8600006
-6.0868359	7.5151997	102.9700012
-6.0868359	7.5639997	104.7600021
-6.0868359	7.6127996	107.7099991
-6.0868359	7.6615996	106.0400009
-6.0868359	7.7103996	103.8600006
-6.0868359	7.7591996	98.6599960
-6.0868359	7.8079996	103.2799988
-6.0868359	7.8567996	103.3799973
-6.0868359	7.9056001	101.3699951
-6.0868359	7.9544001	106.0499954
-6.0868359	8.0032005	105.8099976
-6.0868359	8.0520000	105.1699982
-6.0868359	8.1008005	105.1500015
-6.0868359	8.1496000	104.0800018
-6.0868359	8.1984005	102.9300003
-6.0868359	8.2472000	101.7900009
-6.0868359	8.2960005	103.5199966
-6.0868359	8.3448000	102.1199951
-6.0868359	8.3936005	97.6299973
-6.0868359	8.4424000	103.6999969
-6.0868359	8.4912004	104.3499985
-6.0868359	8.5400000	105.5800018
-6.0868359	8.5888004	104.1399994
-6.0868359	8.6375999	104.6999969
-6.0868359	8.6864004	104.2099991
-6.0868359	8.7351999	104.9399948
-6.0868359	8.7840004	106.2500000
-6.0868359	8.8327999	106.7699966
-6.0868359	8.8816004	104.1399994
-6.0868359	8.9303999	101.5999985
-6.0868359	8.9792004	100.5299988
-6.0868359	9.0279999	105.6299973
-6.0868359	9.0768003	106.7999954
-6.0868359	9.1255999	103.7900009
-6.0868359	9.1744003	105.0299988
-6.0868359	9.2231998	104.9099960
-6.0868359	9.2720003	103.1500015
-6.0868359	9.3207998	103.5199966
-6.0868359	9.3696003	101.4599991
-6.0868359	9.4184008	100.6800003
-6.0868359	9.4672003	100.5499954
-6.0868359	9.5160007	100.1699982
-6.0868359	9.5648003	99.8600006
-6.0868359	9.6136007	97.7500000
-6.0868359	9.6624002	102.4599991
-6.0868359	9.7112007	102.5400009
-6.0868359	9.7600002	102.9899979
-6.0868359	9.8088007	102.4199982
-6.0868359	9.8576002	102.4000015

-6.0868359	9.9064007	99.2600021
-6.0868359	9.9552002	102.3600006
-6.0868359	10.0040007	103.6299973
-6.0868359	10.0528002	103.9499969
-6.0868359	10.1016006	104.9799957
-6.0868359	10.1504002	103.5999985
-6.0868359	10.1992006	102.3399963
-6.0868359	10.2480001	99.3899994
-6.0868359	10.2968006	99.0899963
-6.0868359	10.3456001	102.7600021
-6.0868359	10.3944006	103.9799957
-6.0868359	10.4432001	105.0499954
-6.0868359	10.4920006	104.0499954
-6.0868359	10.5408001	105.0299988
-6.0868359	10.5896006	107.0199966
-6.0868359	10.6384001	105.2500000
-6.0868359	10.6872005	105.8699951
-6.0868359	10.7360001	106.4499969
-6.0868359	10.7848005	106.5999985
-6.0868359	10.8336000	104.9399948
-6.0868359	10.8824005	103.7299957
-6.0868359	10.9312000	101.9000015
-6.0868359	10.9800005	98.3199997
-6.0868359	11.0288000	103.9799957
-6.0868359	11.0776005	101.0999985
-6.0868359	11.1264000	102.1699982
-6.0868359	11.1752005	99.6699982
-6.0868359	11.2240000	102.8099976
-6.0868359	11.2728004	102.1299973
-6.0868359	11.3216000	103.7099991
-6.0868359	11.3704004	104.9899979
-6.0868359	11.4191999	105.6800003
-6.0868359	11.4680004	105.7200012
-6.0868359	11.5167999	106.5999985
-6.0868359	11.5656004	105.5599976
-6.0868359	11.6143999	102.5499954
-6.0868359	11.6632004	103.4599991
-6.0868359	11.7119999	100.1699982
-6.0868359	11.7608004	99.5400009
-6.0868359	11.8095999	104.5999985
-6.0868359	11.8584003	104.1899948
-6.0868359	11.9071999	103.3799973
-6.0868359	11.9560003	103.5299988
-6.0868359	12.0047998	101.6699982
-6.0868359	12.0536003	100.2399979
-6.0868359	12.1023998	104.0299988
-6.0868359	12.1512003	107.3799973
-6.0868359	12.1999998	103.5400009
-6.0868359	12.2488003	106.5899963
-6.0868359	12.2975998	107.2600021
-6.0868359	12.3464003	105.7900009
-6.0868359	12.3951998	103.5899963
-6.0868359	12.4440002	100.8799973
-6.0868359	12.4927998	102.0699997
-6.0868359	12.5416002	104.0499954
-6.0868359	12.5904007	102.5800018
-6.0868359	12.6392002	105.2399979
-6.0868359	12.6880007	105.1500015

-6.0868359	12.7368002	107.7299957
-6.0868359	12.7856007	106.2600021
-6.0868359	12.8344002	104.2999954
-6.0868359	12.8832006	104.3799973
-6.0868359	12.9320002	105.4000015
-6.0868359	12.9808006	106.5699997
-6.0868359	13.0296001	105.0699997
-6.0868359	13.0784006	104.6199951
-6.0868359	13.1272001	104.5699997
-6.0868359	13.1760006	106.3199997
-6.0868359	13.2248001	107.4000015
-6.0868359	13.2736006	104.9499969
-6.0868359	13.3224001	101.5599976
-6.0868359	13.3712006	104.5199966
-6.0868359	13.4200001	106.1399994
-6.0868359	13.4688005	104.7500000
-6.0868359	13.5176001	104.5499954
-6.0868359	13.5664005	104.5199966
-6.0868359	13.6152000	103.5199966
-6.0868359	13.6640005	102.1100006
-6.0868359	13.7128000	102.4799957
-6.0868359	13.7616005	103.9899979
-6.0868359	13.8104000	104.0400009
-6.0868359	13.8592005	103.4499969
-6.0868359	13.9080000	100.5699997
-6.0868359	13.9568005	100.9899979
-6.0868359	14.0056000	104.3499985
-6.0868359	14.0544004	106.3199997
-6.0868359	14.1032000	106.5299988
-6.0868359	14.1520004	106.2600021
-6.0868359	14.2007999	104.6800003
-6.0868359	14.2496004	104.6699982
-6.0868359	14.2983999	103.0800018
-6.0868359	14.3472004	102.8899994
-6.0868359	14.3959999	102.3199997
-6.0868359	14.4448004	102.5499954
-6.0868359	14.4935999	103.3099976
-6.0868359	14.5424004	103.5599976
-6.0868359	14.5911999	103.3799973
-6.0868359	14.6400003	99.6999969
-6.0868359	14.6887999	102.7200012
-6.0868359	14.7376003	105.7299957
-6.0868359	14.7863998	107.7900009
-6.0868359	14.8352003	105.0999985
-6.0868359	14.8839998	103.0100021
-6.0868359	14.9328003	103.0400009
-6.0868359	14.9815998	102.8499985
-6.0868359	15.0304003	101.2399979
-6.0806999	0.3416000	108.0499954
-6.0806999	0.3904000	109.2600021
-6.0806999	0.4392000	106.4799957
-6.0806999	0.4880000	106.3899994
-6.0806999	0.5368000	106.5400009
-6.0806999	0.5856000	104.3199997
-6.0806999	0.6344000	107.5699997
-6.0806999	0.6832000	108.2299957
-6.0806999	0.7320000	108.9300003
-6.0806999	0.7808000	109.0000000

-6.0806999	0.8296000	104.5800018
-6.0806999	0.8784000	101.7099991
-6.0806999	0.9272000	102.7999954
-6.0806999	0.9760000	106.8300018
-6.0806999	1.0247999	106.3499985
-6.0806999	1.0735999	105.3699951
-6.0806999	1.1223999	108.9799957
-6.0806999	1.1711999	106.7399979
-6.0806999	1.2200000	104.8399963
-6.0806999	1.2688000	105.9199982
-6.0806999	1.3176000	104.0599976
-6.0806999	1.3664000	106.5800018
-6.0806999	1.4152000	108.6599960
-6.0806999	1.4640000	108.2399979
-6.0806999	1.5128000	108.8199997
-6.0806999	1.5616000	109.9000015
-6.0806999	1.6104000	110.0400009
-6.0806999	1.6592000	107.3600006
-6.0806999	1.7079999	109.4700012
-6.0806999	1.7567999	108.4199982
-6.0806999	1.8055999	109.2699966
-6.0806999	1.8543999	116.8799973
-6.0806999	1.9031999	119.0400009
-6.0806999	1.9519999	114.1500015
-6.0806999	2.0007999	105.5999985
-6.0806999	2.0495999	104.1599960
-6.0806999	2.0983999	104.6800003
-6.0806999	2.1471999	105.2799988
-6.0806999	2.1959999	105.9300003
-6.0806999	2.2447999	105.9199982
-6.0806999	2.2935998	106.0599976
-6.0806999	2.3423998	104.2699966
-6.0806999	2.3912001	106.0699997
-6.0806999	2.4400001	105.9899979
-6.0806999	2.4888000	107.8199997
-6.0806999	2.5376000	103.9000015
-6.0806999	2.5864000	106.1500015
-6.0806999	2.6352000	105.5400009
-6.0806999	2.6840000	103.5599976
-6.0806999	2.7328000	104.0800018
-6.0806999	2.7816000	102.5800018
-6.0806999	2.8304000	106.2600021
-6.0806999	2.8792000	106.4300003
-6.0806999	2.9280000	105.5400009
-6.0806999	2.9768000	105.6100006
-6.0806999	3.0256000	107.0299988
-6.0806999	3.0743999	106.0999985
-6.0806999	3.1231999	104.9000015
-6.0806999	3.1719999	104.0899963
-6.0806999	3.2207999	100.6299973
-6.0806999	3.2695999	104.3199997
-6.0806999	3.3183999	104.9000015
-6.0806999	3.3671999	103.2099991
-6.0806999	3.4159999	102.7600021
-6.0806999	3.4647999	104.3099976
-6.0806999	3.5135999	103.2600021
-6.0806999	3.5623999	101.2600021
-6.0806999	3.6111999	101.8600006

-6.0806999	3.6599998	104.3600006
-6.0806999	3.7087998	107.5299988
-6.0806999	3.7575998	108.4799957
-6.0806999	3.8063998	104.2600021
-6.0806999	3.8551998	99.7999954
-6.0806999	3.9039998	104.4000015
-6.0806999	3.9527998	106.0699997
-6.0806999	4.0016003	107.7099991
-6.0806999	4.0503998	106.5800018
-6.0806999	4.0991998	105.2799988
-6.0806999	4.1479998	104.1299973
-6.0806999	4.1967998	103.2999954
-6.0806999	4.2455997	102.2299957
-6.0806999	4.2943997	108.2999954
-6.0806999	4.3431997	110.2999954
-6.0806999	4.3919997	107.8199997
-6.0806999	4.4407997	109.0599976
-6.0806999	4.4895997	109.9599991
-6.0806999	4.5383997	112.5000000
-6.0806999	4.5871997	112.1399994
-6.0806999	4.6359997	113.4499969
-6.0806999	4.6847997	115.4199982
-6.0806999	4.7336001	116.5699997
-6.0806999	4.7824001	111.4799957
-6.0806999	4.8312001	108.0000000
-6.0806999	4.8800001	107.7799988
-6.0806999	4.9288001	107.2900009
-6.0806999	4.9776001	106.3399963
-6.0806999	5.0264001	107.7099991
-6.0806999	5.0752001	108.0199966
-6.0806999	5.1240001	107.4000015
-6.0806999	5.1728001	106.3600006
-6.0806999	5.2216001	106.7099991
-6.0806999	5.2704000	108.1599960
-6.0806999	5.3192000	106.6899948
-6.0806999	5.3680000	106.0400009
-6.0806999	5.4168000	106.8399963
-6.0806999	5.4656000	105.7399979
-6.0806999	5.5144000	105.3699951
-6.0806999	5.5632000	103.6899948
-6.0806999	5.6120000	101.6699982
-6.0806999	5.6608000	105.3099976
-6.0806999	5.7096000	104.4099960
-6.0806999	5.7584000	103.0999985
-6.0806999	5.8072000	103.4300003
-6.0806999	5.8559999	102.4399948
-6.0806999	5.9047999	99.9899979
-6.0806999	5.9535999	104.6399994
-6.0806999	6.0023999	105.2799988
-6.0806999	6.0511999	104.9599991
-6.0806999	6.0999999	107.3799973
-6.0806999	6.1487999	107.3499985
-6.0806999	6.1975999	104.8499985
-6.0806999	6.2463999	102.9000015
-6.0806999	6.2951999	98.9599991
-6.0806999	6.3439999	103.5800018
-6.0806999	6.3927999	106.4300003
-6.0806999	6.4415998	104.2900009

-6.0806999	6.4903998	104.8499985
-6.0806999	6.5391998	103.7500000
-6.0806999	6.5879998	105.2200012
-6.0806999	6.6367998	105.0400009
-6.0806999	6.6855998	103.3799973
-6.0806999	6.7343998	102.1399994
-6.0806999	6.7831998	99.7600021
-6.0806999	6.8319998	103.0899963
-6.0806999	6.8807998	104.6899948
-6.0806999	6.9295998	106.3199997
-6.0806999	6.9783998	105.5100021
-6.0806999	7.0271997	105.1699982
-6.0806999	7.0759997	104.1800003
-6.0806999	7.1247997	104.7200012
-6.0806999	7.1735997	104.8099976
-6.0806999	7.2223997	104.5599976
-6.0806999	7.2711997	105.6699982
-6.0806999	7.3199997	106.1399994
-6.0806999	7.3687997	106.6899948
-6.0806999	7.4175997	107.4099960
-6.0806999	7.4663997	104.2900009
-6.0806999	7.5151997	103.3899994
-6.0806999	7.5639997	104.5699997
-6.0806999	7.6127996	107.2399979
-6.0806999	7.6615996	105.5499954
-6.0806999	7.7103996	104.1999969
-6.0806999	7.7591996	98.0199966
-6.0806999	7.8079996	103.2299957
-6.0806999	7.8567996	103.9399948
-6.0806999	7.9056001	101.1199951
-6.0806999	7.9544001	105.4300003
-6.0806999	8.0032005	105.5499954
-6.0806999	8.0520000	104.8699951
-6.0806999	8.1008005	105.4199982
-6.0806999	8.1496000	104.4000015
-6.0806999	8.1984005	103.7500000
-6.0806999	8.2472000	101.2200012
-6.0806999	8.2960005	103.5000000
-6.0806999	8.3448000	102.2900009
-6.0806999	8.3936005	98.2900009
-6.0806999	8.4424000	104.2099991
-6.0806999	8.4912004	103.4899979
-6.0806999	8.5400000	104.8600006
-6.0806999	8.5888004	102.6199951
-6.0806999	8.6375999	104.4199982
-6.0806999	8.6864004	104.1800003
-6.0806999	8.7351999	105.3199997
-6.0806999	8.7840004	106.5299988
-6.0806999	8.8327999	106.4799957
-6.0806999	8.8816004	103.9599991
-6.0806999	8.9303999	101.6399994
-6.0806999	8.9792004	100.3899994
-6.0806999	9.0279999	105.5800018
-6.0806999	9.0768003	106.6299973
-6.0806999	9.1255999	102.8300018
-6.0806999	9.1744003	105.0199966
-6.0806999	9.2231998	105.5899963
-6.0806999	9.2720003	103.5199966

-6.0806999	9.3207998	103.4799957
-6.0806999	9.3696003	101.2600021
-6.0806999	9.4184008	101.4899979
-6.0806999	9.4672003	101.2699966
-6.0806999	9.5160007	100.8600006
-6.0806999	9.5648003	99.5299988
-6.0806999	9.6136007	97.2999954
-6.0806999	9.6624002	103.0100021
-6.0806999	9.7112007	103.7500000
-6.0806999	9.7600002	102.6999969
-6.0806999	9.8088007	102.9700012
-6.0806999	9.8576002	103.4099960
-6.0806999	9.9064007	99.7799988
-6.0806999	9.9552002	101.5599976
-6.0806999	10.0040007	104.3300018
-6.0806999	10.0528002	105.3499985
-6.0806999	10.1016006	104.8399963
-6.0806999	10.1504002	103.8600006
-6.0806999	10.1992006	103.1699982
-6.0806999	10.2480001	99.7399979
-6.0806999	10.2968006	98.2900009
-6.0806999	10.3456001	102.7900009
-6.0806999	10.3944006	104.1699982
-6.0806999	10.4432001	105.1199951
-6.0806999	10.4920006	104.9399948
-6.0806999	10.5408001	105.9099960
-6.0806999	10.5896006	107.1699982
-6.0806999	10.6384001	104.7900009
-6.0806999	10.6872005	104.9300003
-6.0806999	10.7360001	106.0999985
-6.0806999	10.7848005	105.3499985
-6.0806999	10.8336000	103.8899994
-6.0806999	10.8824005	102.3899994
-6.0806999	10.9312000	101.7699966
-6.0806999	10.9800005	98.1299973
-6.0806999	11.0288000	104.1699982
-6.0806999	11.0776005	101.3799973
-6.0806999	11.1264000	101.6999969
-6.0806999	11.1752005	98.9700012
-6.0806999	11.2240000	101.7900009
-6.0806999	11.2728004	102.1399994
-6.0806999	11.3216000	104.9700012
-6.0806999	11.3704004	105.9399948
-6.0806999	11.4191999	105.2200012
-6.0806999	11.4680004	105.1800003
-6.0806999	11.5167999	106.2500000
-6.0806999	11.5656004	105.2399979
-6.0806999	11.6143999	101.9499969
-6.0806999	11.6632004	103.1500015
-6.0806999	11.7119999	100.0899963
-6.0806999	11.7608004	98.7500000
-6.0806999	11.8095999	104.1100006
-6.0806999	11.8584003	104.6500015
-6.0806999	11.9071999	103.9300003
-6.0806999	11.9560003	103.3899994
-6.0806999	12.0047998	102.1699982
-6.0806999	12.0536003	101.7299957
-6.0806999	12.1023998	104.0299988

-6.0806999	12.1512003	107.1399994
-6.0806999	12.1999998	103.8600006
-6.0806999	12.2488003	106.6599960
-6.0806999	12.2975998	107.2900009
-6.0806999	12.3464003	105.8899994
-6.0806999	12.3951998	104.0499954
-6.0806999	12.4440002	100.1599960
-6.0806999	12.4927998	101.2699966
-6.0806999	12.5416002	104.3399963
-6.0806999	12.5904007	102.5999985
-6.0806999	12.6392002	104.9499969
-6.0806999	12.6880007	104.4899979
-6.0806999	12.7368002	106.9300003
-6.0806999	12.7856007	106.5100021
-6.0806999	12.8344002	103.9000015
-6.0806999	12.8832006	105.2799988
-6.0806999	12.9320002	106.4700012
-6.0806999	12.9808006	106.9599991
-6.0806999	13.0296001	105.4000015
-6.0806999	13.0784006	104.6899948
-6.0806999	13.1272001	104.3799973
-6.0806999	13.1760006	106.2699966
-6.0806999	13.2248001	107.4300003
-6.0806999	13.2736006	105.0699997
-6.0806999	13.3224001	102.0800018
-6.0806999	13.3712006	104.7500000
-6.0806999	13.4200001	105.9199982
-6.0806999	13.4688005	104.7999954
-6.0806999	13.5176001	104.9899979
-6.0806999	13.5664005	103.8099976
-6.0806999	13.6152000	101.7200012
-6.0806999	13.6640005	101.2799988
-6.0806999	13.7128000	101.2600021
-6.0806999	13.7616005	102.8799973
-6.0806999	13.8104000	103.2900009
-6.0806999	13.8592005	103.2999954
-6.0806999	13.9080000	101.1599960
-6.0806999	13.9568005	100.7999954
-6.0806999	14.0056000	105.2500000
-6.0806999	14.0544004	106.8199997
-6.0806999	14.1032000	107.0199966
-6.0806999	14.1520004	106.7799988
-6.0806999	14.2007999	105.0000000
-6.0806999	14.2496004	104.4099960
-6.0806999	14.2983999	102.1899948
-6.0806999	14.3472004	102.2500000
-6.0806999	14.3959999	101.2200012
-6.0806999	14.4448004	102.4399948
-6.0806999	14.4935999	103.9199982
-6.0806999	14.5424004	103.8799973
-6.0806999	14.5911999	103.6999969
-6.0806999	14.6400003	100.4499969
-6.0806999	14.6887999	102.3899994
-6.0806999	14.7376003	106.2600021
-6.0806999	14.7863998	107.6100006
-6.0806999	14.8352003	105.0000000
-6.0806999	14.8839998	103.2099991
-6.0806999	14.9328003	103.2399979

-6.0806999	14.9815998	103.2099991
-6.0806999	15.0304003	101.0100021
-6.0745640	0.3416000	108.3199997
-6.0745640	0.3904000	109.7699966
-6.0745640	0.4392000	106.0699997
-6.0745640	0.4880000	105.5100021
-6.0745640	0.5368000	106.4899979
-6.0745640	0.5856000	104.7699966
-6.0745640	0.6344000	108.1999969
-6.0745640	0.6832000	108.3699951
-6.0745640	0.7320000	108.4300003
-6.0745640	0.7808000	108.6899948
-6.0745640	0.8296000	103.4899979
-6.0745640	0.8784000	100.3099976
-6.0745640	0.9272000	103.1999969
-6.0745640	0.9760000	106.6500015
-6.0745640	1.0247999	106.2200012
-6.0745640	1.0735999	105.7500000
-6.0745640	1.1223999	108.6500015
-6.0745640	1.1711999	106.0299988
-6.0745640	1.2200000	105.2999954
-6.0745640	1.2688000	105.9199982
-6.0745640	1.3176000	104.1599960
-6.0745640	1.3664000	106.2900009
-6.0745640	1.4152000	107.4099960
-6.0745640	1.4640000	108.0100021
-6.0745640	1.5128000	109.4199982
-6.0745640	1.5616000	110.6899948
-6.0745640	1.6104000	110.8899994
-6.0745640	1.6592000	108.6199951
-6.0745640	1.7079999	109.0899963
-6.0745640	1.7567999	107.5599976
-6.0745640	1.8055999	109.2299957
-6.0745640	1.8543999	116.4399948
-6.0745640	1.9031999	117.6699982
-6.0745640	1.9519999	112.5100021
-6.0745640	2.0007999	104.1899948
-6.0745640	2.0495999	103.6999969
-6.0745640	2.0983999	103.7600021
-6.0745640	2.1471999	105.4799957
-6.0745640	2.1959999	106.1899948
-6.0745640	2.2447999	106.2900009
-6.0745640	2.2935998	106.1699982
-6.0745640	2.3423998	103.5800018
-6.0745640	2.3912001	106.2500000
-6.0745640	2.4400001	106.6999969
-6.0745640	2.4888000	108.0199966
-6.0745640	2.5376000	103.7500000
-6.0745640	2.5864000	106.5800018
-6.0745640	2.6352000	105.6599960
-6.0745640	2.6840000	103.4599991
-6.0745640	2.7328000	104.0000000
-6.0745640	2.7816000	102.0499954
-6.0745640	2.8304000	105.7399979
-6.0745640	2.8792000	107.1399994
-6.0745640	2.9280000	105.3600006
-6.0745640	2.9768000	104.8300018
-6.0745640	3.0256000	106.8399963

-6.0745640	3.0743999	106.5800018
-6.0745640	3.1231999	106.4700012
-6.0745640	3.1719999	104.7399979
-6.0745640	3.2207999	102.1800003
-6.0745640	3.2695999	104.9399948
-6.0745640	3.3183999	104.8399963
-6.0745640	3.3671999	103.1699982
-6.0745640	3.4159999	102.2699966
-6.0745640	3.4647999	103.6800003
-6.0745640	3.5135999	103.0699997
-6.0745640	3.5623999	102.8300018
-6.0745640	3.6111999	103.6599960
-6.0745640	3.6599998	105.4300003
-6.0745640	3.7087998	108.2500000
-6.0745640	3.7575998	108.2999954
-6.0745640	3.8063998	103.1199951
-6.0745640	3.8551998	100.2699966
-6.0745640	3.9039998	105.2299957
-6.0745640	3.9527998	106.7799988
-6.0745640	4.0016003	107.8499985
-6.0745640	4.0503998	107.0599976
-6.0745640	4.0991998	105.2900009
-6.0745640	4.1479998	103.8099976
-6.0745640	4.1967998	102.7299957
-6.0745640	4.2455997	102.8899994
-6.0745640	4.2943997	108.7900009
-6.0745640	4.3431997	110.3600006
-6.0745640	4.3919997	107.2799988
-6.0745640	4.4407997	109.2399979
-6.0745640	4.4895997	111.0000000
-6.0745640	4.5383997	112.4499969
-6.0745640	4.5871997	112.6299973
-6.0745640	4.6359997	114.2299957
-6.0745640	4.6847997	116.1599960
-6.0745640	4.7336001	116.9199982
-6.0745640	4.7824001	111.5499954
-6.0745640	4.8312001	107.2299957
-6.0745640	4.8800001	106.5299988
-6.0745640	4.9288001	108.3699951
-6.0745640	4.9776001	106.8499985
-6.0745640	5.0264001	107.2099991
-6.0745640	5.0752001	107.6399994
-6.0745640	5.1240001	108.4399948
-6.0745640	5.1728001	106.9899979
-6.0745640	5.2216001	106.4300003
-6.0745640	5.2704000	108.1500015
-6.0745640	5.3192000	107.4099960
-6.0745640	5.3680000	105.9700012
-6.0745640	5.4168000	106.2699966
-6.0745640	5.4656000	104.4000015
-6.0745640	5.5144000	104.2500000
-6.0745640	5.5632000	103.2999954
-6.0745640	5.6120000	99.1999969
-6.0745640	5.6608000	103.8600006
-6.0745640	5.7096000	105.5599976
-6.0745640	5.7584000	104.6899948
-6.0745640	5.8072000	104.1699982
-6.0745640	5.8559999	102.9599991

-6.0745640	5.9047999	100.2799988
-6.0745640	5.9535999	103.1699982
-6.0745640	6.0023999	104.4199982
-6.0745640	6.0511999	104.7799988
-6.0745640	6.0999999	106.9599991
-6.0745640	6.1487999	106.7699966
-6.0745640	6.1975999	105.0899963
-6.0745640	6.2463999	103.3300018
-6.0745640	6.2951999	99.5499954
-6.0745640	6.3439999	104.1599960
-6.0745640	6.3927999	106.2299957
-6.0745640	6.4415998	104.6100006
-6.0745640	6.4903998	105.4099960
-6.0745640	6.5391998	104.3099976
-6.0745640	6.5879998	105.2099991
-6.0745640	6.6367998	105.3300018
-6.0745640	6.6855998	103.8399963
-6.0745640	6.7343998	101.7099991
-6.0745640	6.7831998	98.2699966
-6.0745640	6.8319998	102.5199966
-6.0745640	6.8807998	104.8399963
-6.0745640	6.9295998	105.5899963
-6.0745640	6.9783998	105.8499985
-6.0745640	7.0271997	105.3499985
-6.0745640	7.0759997	104.2900009
-6.0745640	7.1247997	105.0400009
-6.0745640	7.1735997	105.5499954
-6.0745640	7.2223997	105.0599976
-6.0745640	7.2711997	105.9599991
-6.0745640	7.3199997	105.0400009
-6.0745640	7.3687997	105.5400009
-6.0745640	7.4175997	106.9599991
-6.0745640	7.4663997	104.1399994
-6.0745640	7.5151997	103.5800018
-6.0745640	7.5639997	104.7600021
-6.0745640	7.6127996	106.7900009
-6.0745640	7.6615996	104.6999969
-6.0745640	7.7103996	103.8899994
-6.0745640	7.7591996	99.1399994
-6.0745640	7.8079996	103.3499985
-6.0745640	7.8567996	104.4799957
-6.0745640	7.9056001	102.0800018
-6.0745640	7.9544001	105.0199966
-6.0745640	8.0032005	105.5400009
-6.0745640	8.0520000	104.8499985
-6.0745640	8.1008005	105.2299957
-6.0745640	8.1496000	104.1199951
-6.0745640	8.1984005	104.4599991
-6.0745640	8.2472000	101.9700012
-6.0745640	8.2960005	103.8199997
-6.0745640	8.3448000	102.3600006
-6.0745640	8.3936005	99.0000000
-6.0745640	8.4424000	104.4499969
-6.0745640	8.4912004	102.5299988
-6.0745640	8.5400000	104.5599976
-6.0745640	8.5888004	101.3199997
-6.0745640	8.6375999	104.2500000
-6.0745640	8.6864004	105.0000000

-6.0745640	8.7351999	105.8799973
-6.0745640	8.7840004	106.6100006
-6.0745640	8.8327999	105.7500000
-6.0745640	8.8816004	104.1199951
-6.0745640	8.9303999	102.2900009
-6.0745640	8.9792004	100.5400009
-6.0745640	9.0279999	105.1999969
-6.0745640	9.0768003	106.1699982
-6.0745640	9.1255999	102.5899963
-6.0745640	9.1744003	104.7600021
-6.0745640	9.2231998	106.3399963
-6.0745640	9.2720003	104.3899994
-6.0745640	9.3207998	103.3799973
-6.0745640	9.3696003	101.5000000
-6.0745640	9.4184008	102.2999954
-6.0745640	9.4672003	101.4099960
-6.0745640	9.5160007	100.5999985
-6.0745640	9.5648003	100.1199951
-6.0745640	9.6136007	97.0800018
-6.0745640	9.6624002	103.2999954
-6.0745640	9.7112007	104.4300003
-6.0745640	9.7600002	102.9199982
-6.0745640	9.8088007	103.1599960
-6.0745640	9.8576002	103.1599960
-6.0745640	9.9064007	100.4199982
-6.0745640	9.9552002	101.8799973
-6.0745640	10.0040007	105.3300018
-6.0745640	10.0528002	106.7500000
-6.0745640	10.1016006	104.9199982
-6.0745640	10.1504002	104.4700012
-6.0745640	10.1992006	104.2299957
-6.0745640	10.2480001	101.4599991
-6.0745640	10.2968006	98.1500015
-6.0745640	10.3456001	102.9599991
-6.0745640	10.3944006	104.4499969
-6.0745640	10.4432001	105.3499985
-6.0745640	10.4920006	105.3399963
-6.0745640	10.5408001	105.6500015
-6.0745640	10.5896006	106.5299988
-6.0745640	10.6384001	104.0000000
-6.0745640	10.6872005	103.9199982
-6.0745640	10.7360001	105.6199951
-6.0745640	10.7848005	104.0999985
-6.0745640	10.8336000	103.2500000
-6.0745640	10.8824005	101.8699951
-6.0745640	10.9312000	101.6800003
-6.0745640	10.9800005	99.7200012
-6.0745640	11.0288000	104.6299973
-6.0745640	11.0776005	101.8600006
-6.0745640	11.1264000	101.5899963
-6.0745640	11.1752005	97.7999954
-6.0745640	11.2240000	100.3899994
-6.0745640	11.2728004	100.9300003
-6.0745640	11.3216000	105.6199951
-6.0745640	11.3704004	106.4399948
-6.0745640	11.4191999	105.2500000
-6.0745640	11.4680004	104.8899994
-6.0745640	11.5167999	105.0100021

-6.0745640	11.5656004	104.5899963
-6.0745640	11.6143999	101.7900009
-6.0745640	11.6632004	102.8699951
-6.0745640	11.7119999	100.4300003
-6.0745640	11.7608004	99.2600021
-6.0745640	11.8095999	103.8499985
-6.0745640	11.8584003	105.6500015
-6.0745640	11.9071999	104.4000015
-6.0745640	11.9560003	103.2500000
-6.0745640	12.0047998	102.7699966
-6.0745640	12.0536003	102.4099960
-6.0745640	12.1023998	104.2500000
-6.0745640	12.1512003	106.8300018
-6.0745640	12.1999998	103.8499985
-6.0745640	12.2488003	106.5999985
-6.0745640	12.2975998	106.9300003
-6.0745640	12.3464003	105.4000015
-6.0745640	12.3951998	104.1199951
-6.0745640	12.4440002	99.6100006
-6.0745640	12.4927998	101.8899994
-6.0745640	12.5416002	104.4599991
-6.0745640	12.5904007	102.1599960
-6.0745640	12.6392002	104.6599960
-6.0745640	12.6880007	103.9300003
-6.0745640	12.7368002	105.7099991
-6.0745640	12.7856007	106.5599976
-6.0745640	12.8344002	103.8499985
-6.0745640	12.8832006	106.1100006
-6.0745640	12.9320002	107.0499954
-6.0745640	12.9808006	107.0699997
-6.0745640	13.0296001	105.3199997
-6.0745640	13.0784006	104.2200012
-6.0745640	13.1272001	103.9700012
-6.0745640	13.1760006	105.9099960
-6.0745640	13.2248001	107.1100006
-6.0745640	13.2736006	105.5499954
-6.0745640	13.3224001	102.9599991
-6.0745640	13.3712006	105.1599960
-6.0745640	13.4200001	105.9399948
-6.0745640	13.4688005	105.0299988
-6.0745640	13.5176001	105.0999985
-6.0745640	13.5664005	103.0699997
-6.0745640	13.6152000	100.0100021
-6.0745640	13.6640005	100.7900009
-6.0745640	13.7128000	100.3499985
-6.0745640	13.7616005	101.5800018
-6.0745640	13.8104000	102.9599991
-6.0745640	13.8592005	103.3899994
-6.0745640	13.9080000	102.0699997
-6.0745640	13.9568005	101.6899948
-6.0745640	14.0056000	106.3499985
-6.0745640	14.0544004	106.8499985
-6.0745640	14.1032000	106.7500000
-6.0745640	14.1520004	106.6100006
-6.0745640	14.2007999	105.0100021
-6.0745640	14.2496004	104.2299957
-6.0745640	14.2983999	101.2699966
-6.0745640	14.3472004	101.1100006

-6.0745640	14.3959999	99.6899948
-6.0745640	14.4448004	101.5599976
-6.0745640	14.4935999	104.2699966
-6.0745640	14.5424004	104.1899948
-6.0745640	14.5911999	103.8899994
-6.0745640	14.6400003	101.7399979
-6.0745640	14.6887999	102.4199982
-6.0745640	14.7376003	106.5299988
-6.0745640	14.7863998	107.0199966
-6.0745640	14.8352003	104.2699966
-6.0745640	14.8839998	103.6899948
-6.0745640	14.9328003	104.5100021
-6.0745640	14.9815998	104.6399994
-6.0745640	15.0304003	101.7699966
-6.0684280	0.3416000	108.9499969
-6.0684280	0.3904000	110.2699966
-6.0684280	0.4392000	105.8799973
-6.0684280	0.4880000	104.7699966
-6.0684280	0.5368000	106.3600006
-6.0684280	0.5856000	105.2600021
-6.0684280	0.6344000	108.7399979
-6.0684280	0.6832000	108.7200012
-6.0684280	0.7320000	107.6100006
-6.0684280	0.7808000	108.1100006
-6.0684280	0.8296000	103.1500015
-6.0684280	0.8784000	99.8099976
-6.0684280	0.9272000	103.9199982
-6.0684280	0.9760000	106.6500015
-6.0684280	1.0247999	106.7900009
-6.0684280	1.0735999	105.9300003
-6.0684280	1.1223999	107.7799988
-6.0684280	1.1711999	105.2299957
-6.0684280	1.2200000	105.1199951
-6.0684280	1.2688000	105.3199997
-6.0684280	1.3176000	104.4199982
-6.0684280	1.3664000	105.5899963
-6.0684280	1.4152000	105.7999954
-6.0684280	1.4640000	107.8600006
-6.0684280	1.5128000	109.3699951
-6.0684280	1.5616000	110.0599976
-6.0684280	1.6104000	111.2600021
-6.0684280	1.6592000	109.7500000
-6.0684280	1.7079999	109.2399979
-6.0684280	1.7567999	107.2099991
-6.0684280	1.8055999	108.9899979
-6.0684280	1.8543999	115.4799957
-6.0684280	1.9031999	116.0499954
-6.0684280	1.9519999	110.9099960
-6.0684280	2.0007999	103.3399963
-6.0684280	2.0495999	103.8099976
-6.0684280	2.0983999	103.7600021
-6.0684280	2.1471999	105.6199951
-6.0684280	2.1959999	106.7699966
-6.0684280	2.2447999	107.7600021
-6.0684280	2.2935998	106.7699966
-6.0684280	2.3423998	103.4399948
-6.0684280	2.3912001	106.6899948
-6.0684280	2.4400001	107.2600021

-6.0684280	2.4888000	107.7999954
-6.0684280	2.5376000	103.3300018
-6.0684280	2.5864000	107.1299973
-6.0684280	2.6352000	106.7299957
-6.0684280	2.6840000	104.7900009
-6.0684280	2.7328000	104.7399979
-6.0684280	2.7816000	102.6500015
-6.0684280	2.8304000	105.1199951
-6.0684280	2.8792000	107.2500000
-6.0684280	2.9280000	105.3600006
-6.0684280	2.9768000	104.2399979
-6.0684280	3.0256000	106.6699982
-6.0684280	3.0743999	106.4799957
-6.0684280	3.1231999	107.0899963
-6.0684280	3.1719999	104.9300003
-6.0684280	3.2207999	103.3699951
-6.0684280	3.2695999	105.1800003
-6.0684280	3.3183999	104.6399994
-6.0684280	3.3671999	103.3199997
-6.0684280	3.4159999	101.7200012
-6.0684280	3.4647999	102.9899979
-6.0684280	3.5135999	102.8399963
-6.0684280	3.5623999	103.6899948
-6.0684280	3.6111999	105.1699982
-6.0684280	3.6599998	106.0400009
-6.0684280	3.7087998	107.9199982
-6.0684280	3.7575998	107.8600006
-6.0684280	3.8063998	102.0599976
-6.0684280	3.8551998	101.5000000
-6.0684280	3.9039998	106.4000015
-6.0684280	3.9527998	107.4799957
-6.0684280	4.0016003	107.7299957
-6.0684280	4.0503998	107.2099991
-6.0684280	4.0991998	104.8300018
-6.0684280	4.1479998	102.0299988
-6.0684280	4.1967998	101.2900009
-6.0684280	4.2455997	103.9099960
-6.0684280	4.2943997	109.4700012
-6.0684280	4.3431997	110.8399963
-6.0684280	4.3919997	107.1199951
-6.0684280	4.4407997	109.5800018
-6.0684280	4.4895997	111.9599991
-6.0684280	4.5383997	112.9499969
-6.0684280	4.5871997	113.7699966
-6.0684280	4.6359997	115.0000000
-6.0684280	4.6847997	116.6699982
-6.0684280	4.7336001	116.9700012
-6.0684280	4.7824001	111.3600006
-6.0684280	4.8312001	106.6500015
-6.0684280	4.8800001	105.2999954
-6.0684280	4.9288001	109.5199966
-6.0684280	4.9776001	107.2999954
-6.0684280	5.0264001	106.8600006
-6.0684280	5.0752001	107.3899994
-6.0684280	5.1240001	109.5299988
-6.0684280	5.1728001	107.6500015
-6.0684280	5.2216001	106.0999985
-6.0684280	5.2704000	107.8399963

-6.0684280	5.3192000	107.3799973
-6.0684280	5.3680000	105.4399948
-6.0684280	5.4168000	105.7699966
-6.0684280	5.4656000	103.9199982
-6.0684280	5.5144000	103.3300018
-6.0684280	5.5632000	102.4399948
-6.0684280	5.6120000	96.9399948
-6.0684280	5.6608000	102.8199997
-6.0684280	5.7096000	106.5100021
-6.0684280	5.7584000	105.7600021
-6.0684280	5.8072000	104.7299957
-6.0684280	5.8559999	103.6599960
-6.0684280	5.9047999	101.0800018
-6.0684280	5.9535999	102.5000000
-6.0684280	6.0023999	104.2500000
-6.0684280	6.0511999	105.2200012
-6.0684280	6.0999999	106.9899979
-6.0684280	6.1487999	106.3699951
-6.0684280	6.1975999	105.3799973
-6.0684280	6.2463999	104.0199966
-6.0684280	6.2951999	100.8300018
-6.0684280	6.3439999	105.1299973
-6.0684280	6.3927999	106.5699997
-6.0684280	6.4415998	105.5599976
-6.0684280	6.4903998	106.5599976
-6.0684280	6.5391998	105.1999969
-6.0684280	6.5879998	105.6699982
-6.0684280	6.6367998	105.8899994
-6.0684280	6.6855998	104.5299988
-6.0684280	6.7343998	101.7399979
-6.0684280	6.7831998	97.4300003
-6.0684280	6.8319998	102.2600021
-6.0684280	6.8807998	105.0999985
-6.0684280	6.9295998	104.9899979
-6.0684280	6.9783998	105.3799973
-6.0684280	7.0271997	105.9300003
-6.0684280	7.0759997	104.7900009
-6.0684280	7.1247997	105.2200012
-6.0684280	7.1735997	105.6199951
-6.0684280	7.2223997	104.9799957
-6.0684280	7.2711997	105.6899948
-6.0684280	7.3199997	104.3300018
-6.0684280	7.3687997	104.0400009
-6.0684280	7.4175997	105.7699966
-6.0684280	7.4663997	103.5000000
-6.0684280	7.5151997	103.7500000
-6.0684280	7.5639997	104.9899979
-6.0684280	7.6127996	106.1599960
-6.0684280	7.6615996	103.8600006
-6.0684280	7.7103996	103.8799973
-6.0684280	7.7591996	100.0599976
-6.0684280	7.8079996	104.0299988
-6.0684280	7.8567996	104.7999954
-6.0684280	7.9056001	101.9599991
-6.0684280	7.9544001	103.8799973
-6.0684280	8.0032005	105.5000000
-6.0684280	8.0520000	105.2600021
-6.0684280	8.1008005	104.9700012

-6.0684280	8.1496000	104.0999985
-6.0684280	8.1984005	105.0999985
-6.0684280	8.2472000	102.6899948
-6.0684280	8.2960005	104.3300018
-6.0684280	8.3448000	103.0599976
-6.0684280	8.3936005	100.0000000
-6.0684280	8.4424000	104.5800018
-6.0684280	8.4912004	102.0199966
-6.0684280	8.5400000	104.2699966
-6.0684280	8.5888004	99.9700012
-6.0684280	8.6375999	103.4799957
-6.0684280	8.6864004	105.3199997
-6.0684280	8.7351999	105.7600021
-6.0684280	8.7840004	106.1299973
-6.0684280	8.8327999	104.3499985
-6.0684280	8.8816004	103.7600021
-6.0684280	8.9303999	102.3799973
-6.0684280	8.9792004	99.8600006
-6.0684280	9.0279999	104.5800018
-6.0684280	9.0768003	105.8499985
-6.0684280	9.1255999	103.0000000
-6.0684280	9.1744003	104.0899963
-6.0684280	9.2231998	106.3399963
-6.0684280	9.2720003	104.1100006
-6.0684280	9.3207998	102.0000000
-6.0684280	9.3696003	101.3199997
-6.0684280	9.4184008	102.7099991
-6.0684280	9.4672003	102.1800003
-6.0684280	9.5160007	101.4000015
-6.0684280	9.5648003	101.5699997
-6.0684280	9.6136007	97.3399963
-6.0684280	9.6624002	103.5100021
-6.0684280	9.7112007	105.1599960
-6.0684280	9.7600002	103.2900009
-6.0684280	9.8088007	103.4799957
-6.0684280	9.8576002	103.3600006
-6.0684280	9.9064007	101.4099960
-6.0684280	9.9552002	102.1399994
-6.0684280	10.0040007	105.8699951
-6.0684280	10.0528002	107.0499954
-6.0684280	10.1016006	104.8300018
-6.0684280	10.1504002	105.0100021
-6.0684280	10.1992006	105.5000000
-6.0684280	10.2480001	103.4000015
-6.0684280	10.2968006	99.3499985
-6.0684280	10.3456001	102.6899948
-6.0684280	10.3944006	104.6800003
-6.0684280	10.4432001	105.6599960
-6.0684280	10.4920006	105.7900009
-6.0684280	10.5408001	105.1299973
-6.0684280	10.5896006	105.7799988
-6.0684280	10.6384001	103.4099960
-6.0684280	10.6872005	102.7900009
-6.0684280	10.7360001	104.1800003
-6.0684280	10.7848005	102.9599991
-6.0684280	10.8336000	103.3099976
-6.0684280	10.8824005	101.8499985
-6.0684280	10.9312000	101.1800003

-6.0684280	10.9800005	101.0100021
-6.0684280	11.0288000	105.4499969
-6.0684280	11.0776005	103.2600021
-6.0684280	11.1264000	101.5100021
-6.0684280	11.1752005	97.1399994
-6.0684280	11.2240000	99.5000000
-6.0684280	11.2728004	99.4700012
-6.0684280	11.3216000	105.0299988
-6.0684280	11.3704004	105.9899979
-6.0684280	11.4191999	105.1699982
-6.0684280	11.4680004	104.8399963
-6.0684280	11.5167999	104.2799988
-6.0684280	11.5656004	104.3799973
-6.0684280	11.6143999	101.8099976
-6.0684280	11.6632004	102.9199982
-6.0684280	11.7119999	100.6999969
-6.0684280	11.7608004	99.1599960
-6.0684280	11.8095999	103.3199997
-6.0684280	11.8584003	106.1399994
-6.0684280	11.9071999	104.7200012
-6.0684280	11.9560003	103.6899948
-6.0684280	12.0047998	103.9700012
-6.0684280	12.0536003	103.4399948
-6.0684280	12.1023998	104.4300003
-6.0684280	12.1512003	106.2200012
-6.0684280	12.1999998	103.6399994
-6.0684280	12.2488003	106.6299973
-6.0684280	12.2975998	106.4300003
-6.0684280	12.3464003	104.2399979
-6.0684280	12.3951998	103.9499969
-6.0684280	12.4440002	100.0699997
-6.0684280	12.4927998	102.4700012
-6.0684280	12.5416002	104.7600021
-6.0684280	12.5904007	102.3399963
-6.0684280	12.6392002	104.6100006
-6.0684280	12.6880007	104.0299988
-6.0684280	12.7368002	105.1100006
-6.0684280	12.7856007	106.7200012
-6.0684280	12.8344002	103.3699951
-6.0684280	12.8832006	106.3600006
-6.0684280	12.9320002	107.0499954
-6.0684280	12.9808006	106.5999985
-6.0684280	13.0296001	104.8499985
-6.0684280	13.0784006	104.0999985
-6.0684280	13.1272001	103.5199966
-6.0684280	13.1760006	105.0999985
-6.0684280	13.2248001	106.2500000
-6.0684280	13.2736006	105.8799973
-6.0684280	13.3224001	103.7099991
-6.0684280	13.3712006	105.1999969
-6.0684280	13.4200001	106.0800018
-6.0684280	13.4688005	104.8199997
-6.0684280	13.5176001	105.3899994
-6.0684280	13.5664005	103.0400009
-6.0684280	13.6152000	98.1999969
-6.0684280	13.6640005	100.4300003
-6.0684280	13.7128000	99.3199997
-6.0684280	13.7616005	100.5999985

-6.0684280	13.8104000	102.1399994
-6.0684280	13.8592005	103.2600021
-6.0684280	13.9080000	102.7699966
-6.0684280	13.9568005	102.7900009
-6.0684280	14.0056000	106.7500000
-6.0684280	14.0544004	105.9099960
-6.0684280	14.1032000	105.6599960
-6.0684280	14.1520004	106.2600021
-6.0684280	14.2007999	104.7200012
-6.0684280	14.2496004	103.8399963
-6.0684280	14.2983999	100.3600006
-6.0684280	14.3472004	100.3499985
-6.0684280	14.3959999	99.6599960
-6.0684280	14.4448004	100.9499969
-6.0684280	14.4935999	104.4700012
-6.0684280	14.5424004	104.1500015
-6.0684280	14.5911999	103.8399963
-6.0684280	14.6400003	103.2600021
-6.0684280	14.6887999	103.5000000
-6.0684280	14.7376003	106.9899979
-6.0684280	14.7863998	106.2500000
-6.0684280	14.8352003	102.8099976
-6.0684280	14.8839998	103.3300018
-6.0684280	14.9328003	104.8899994
-6.0684280	14.9815998	105.3300018
-6.0684280	15.0304003	102.3799973
-6.0622921	0.3416000	109.0999985
-6.0622921	0.3904000	110.9899979
-6.0622921	0.4392000	106.9599991
-6.0622921	0.4880000	104.7900009
-6.0622921	0.5368000	106.0800018
-6.0622921	0.5856000	105.3199997
-6.0622921	0.6344000	108.5100021
-6.0622921	0.6832000	108.8300018
-6.0622921	0.7320000	106.9099960
-6.0622921	0.7808000	108.0499954
-6.0622921	0.8296000	103.5499954
-6.0622921	0.8784000	101.9199982
-6.0622921	0.9272000	105.5800018
-6.0622921	0.9760000	107.0599976
-6.0622921	1.0247999	107.1800003
-6.0622921	1.0735999	105.4399948
-6.0622921	1.1223999	106.1399994
-6.0622921	1.1711999	103.7299957
-6.0622921	1.2200000	104.5699997
-6.0622921	1.2688000	104.6800003
-6.0622921	1.3176000	104.5800018
-6.0622921	1.3664000	104.9000015
-6.0622921	1.4152000	104.1599960
-6.0622921	1.4640000	106.9799957
-6.0622921	1.5128000	108.6699982
-6.0622921	1.5616000	109.3399963
-6.0622921	1.6104000	110.7900009
-6.0622921	1.6592000	110.2600021
-6.0622921	1.7079999	109.1199951
-6.0622921	1.7567999	106.8300018
-6.0622921	1.8055999	109.2099991
-6.0622921	1.8543999	114.4700012

-6.0622921	1.9031999	114.5199966
-6.0622921	1.9519999	109.5699997
-6.0622921	2.0007999	102.3799973
-6.0622921	2.0495999	103.9399948
-6.0622921	2.0983999	104.6599960
-6.0622921	2.1471999	106.0699997
-6.0622921	2.1959999	107.4300003
-6.0622921	2.2447999	108.0999985
-6.0622921	2.2935998	106.5599976
-6.0622921	2.3423998	102.8099976
-6.0622921	2.3912001	106.6699982
-6.0622921	2.4400001	107.3899994
-6.0622921	2.4888000	107.5100021
-6.0622921	2.5376000	104.1899948
-6.0622921	2.5864000	107.6199951
-6.0622921	2.6352000	107.3499985
-6.0622921	2.6840000	105.4700012
-6.0622921	2.7328000	105.5199966
-6.0622921	2.7816000	103.8899994
-6.0622921	2.8304000	104.6399994
-6.0622921	2.8792000	106.2799988
-6.0622921	2.9280000	104.7900009
-6.0622921	2.9768000	103.3899994
-6.0622921	3.0256000	106.6100006
-6.0622921	3.0743999	106.7299957
-6.0622921	3.1231999	107.4799957
-6.0622921	3.1719999	105.0000000
-6.0622921	3.2207999	104.7500000
-6.0622921	3.2695999	105.7299957
-6.0622921	3.3183999	104.9300003
-6.0622921	3.3671999	104.0899963
-6.0622921	3.4159999	102.4199982
-6.0622921	3.4647999	103.3600006
-6.0622921	3.5135999	102.4599991
-6.0622921	3.5623999	103.9899979
-6.0622921	3.6111999	105.9899979
-6.0622921	3.6599998	105.8899994
-6.0622921	3.7087998	107.0899963
-6.0622921	3.7575998	107.0299988
-6.0622921	3.8063998	100.6599960
-6.0622921	3.8551998	102.4700012
-6.0622921	3.9039998	107.3499985
-6.0622921	3.9527998	108.2200012
-6.0622921	4.0016003	107.6699982
-6.0622921	4.0503998	107.3199997
-6.0622921	4.0991998	105.2900009
-6.0622921	4.1479998	101.0999985
-6.0622921	4.1967998	100.7399979
-6.0622921	4.2455997	104.0699997
-6.0622921	4.2943997	109.4199982
-6.0622921	4.3431997	111.3099976
-6.0622921	4.3919997	108.0800018
-6.0622921	4.4407997	109.9399948
-6.0622921	4.4895997	112.4399948
-6.0622921	4.5383997	113.7699966
-6.0622921	4.5871997	115.2799988
-6.0622921	4.6359997	115.7399979
-6.0622921	4.6847997	116.8199997

-6.0622921	4.7336001	116.6899948
-6.0622921	4.7824001	111.0000000
-6.0622921	4.8312001	106.0999985
-6.0622921	4.8800001	104.5299988
-6.0622921	4.9288001	110.5000000
-6.0622921	4.9776001	107.6999969
-6.0622921	5.0264001	106.8099976
-6.0622921	5.0752001	107.4000015
-6.0622921	5.1240001	110.4499969
-6.0622921	5.1728001	108.2299957
-6.0622921	5.2216001	106.0899963
-6.0622921	5.2704000	107.4000015
-6.0622921	5.3192000	107.2399979
-6.0622921	5.3680000	105.4700012
-6.0622921	5.4168000	105.2699966
-6.0622921	5.4656000	103.1899948
-6.0622921	5.5144000	102.3799973
-6.0622921	5.5632000	101.9099960
-6.0622921	5.6120000	96.3399963
-6.0622921	5.6608000	101.9599991
-6.0622921	5.7096000	106.8600006
-6.0622921	5.7584000	106.6899948
-6.0622921	5.8072000	105.7900009
-6.0622921	5.8559999	104.7099991
-6.0622921	5.9047999	102.3600006
-6.0622921	5.9535999	101.9700012
-6.0622921	6.0023999	103.7799988
-6.0622921	6.0511999	104.8199997
-6.0622921	6.0999999	106.2900009
-6.0622921	6.1487999	106.0499954
-6.0622921	6.1975999	105.5599976
-6.0622921	6.2463999	104.1500015
-6.0622921	6.2951999	102.4000015
-6.0622921	6.3439999	104.9000015
-6.0622921	6.3927999	106.3399963
-6.0622921	6.4415998	106.2699966
-6.0622921	6.4903998	107.4799957
-6.0622921	6.5391998	105.7999954
-6.0622921	6.5879998	106.2200012
-6.0622921	6.6367998	106.4000015
-6.0622921	6.6855998	105.8399963
-6.0622921	6.7343998	102.5899963
-6.0622921	6.7831998	97.2900009
-6.0622921	6.8319998	102.0699997
-6.0622921	6.8807998	104.8699951
-6.0622921	6.9295998	103.5299988
-6.0622921	6.9783998	104.3799973
-6.0622921	7.0271997	106.2799988
-6.0622921	7.0759997	105.2799988
-6.0622921	7.1247997	105.6100006
-6.0622921	7.1735997	105.7900009
-6.0622921	7.2223997	104.4000015
-6.0622921	7.2711997	105.2200012
-6.0622921	7.3199997	103.9000015
-6.0622921	7.3687997	103.0199966
-6.0622921	7.4175997	104.5299988
-6.0622921	7.4663997	102.0199966
-6.0622921	7.5151997	103.5199966

-6.0622921	7.5639997	105.4099960
-6.0622921	7.6127996	105.3899994
-6.0622921	7.6615996	103.4099960
-6.0622921	7.7103996	103.9099960
-6.0622921	7.7591996	100.6899948
-6.0622921	7.8079996	104.7299957
-6.0622921	7.8567996	105.2600021
-6.0622921	7.9056001	101.8199997
-6.0622921	7.9544001	102.7500000
-6.0622921	8.0032005	105.2799988
-6.0622921	8.0520000	105.3899994
-6.0622921	8.1008005	104.3799973
-6.0622921	8.1496000	103.9899979
-6.0622921	8.1984005	105.5999985
-6.0622921	8.2472000	104.7399979
-6.0622921	8.2960005	106.0100021
-6.0622921	8.3448000	103.8600006
-6.0622921	8.3936005	100.1399994
-6.0622921	8.4424000	104.1100006
-6.0622921	8.4912004	101.1199951
-6.0622921	8.5400000	103.7799988
-6.0622921	8.5888004	98.5499954
-6.0622921	8.6375999	103.0000000
-6.0622921	8.6864004	105.8199997
-6.0622921	8.7351999	105.9499969
-6.0622921	8.7840004	105.3600006
-6.0622921	8.8327999	102.4899979
-6.0622921	8.8816004	102.6800003
-6.0622921	8.9303999	102.0299988
-6.0622921	8.9792004	99.2900009
-6.0622921	9.0279999	103.9899979
-6.0622921	9.0768003	105.3899994
-6.0622921	9.1255999	103.8300018
-6.0622921	9.1744003	104.3399963
-6.0622921	9.2231998	106.1599960
-6.0622921	9.2720003	103.6100006
-6.0622921	9.3207998	101.1299973
-6.0622921	9.3696003	101.5499954
-6.0622921	9.4184008	103.3799973
-6.0622921	9.4672003	103.0699997
-6.0622921	9.5160007	101.3300018
-6.0622921	9.5648003	103.3399963
-6.0622921	9.6136007	98.7399979
-6.0622921	9.6624002	103.9000015
-6.0622921	9.7112007	105.8799973
-6.0622921	9.7600002	103.6399994
-6.0622921	9.8088007	103.4300003
-6.0622921	9.8576002	103.2399979
-6.0622921	9.9064007	102.0699997
-6.0622921	9.9552002	101.9000015
-6.0622921	10.0040007	105.4799957
-6.0622921	10.0528002	106.5299988
-6.0622921	10.1016006	105.0800018
-6.0622921	10.1504002	105.1800003
-6.0622921	10.1992006	106.4099960
-6.0622921	10.2480001	105.4000015
-6.0622921	10.2968006	101.4300003
-6.0622921	10.3456001	102.4199982

-6.0622921	10.3944006	104.4799957
-6.0622921	10.4432001	105.5699997
-6.0622921	10.4920006	105.6299973
-6.0622921	10.5408001	103.8699951
-6.0622921	10.5896006	104.8699951
-6.0622921	10.6384001	103.1500015
-6.0622921	10.6872005	102.4700012
-6.0622921	10.7360001	103.8300018
-6.0622921	10.7848005	102.4799957
-6.0622921	10.8336000	104.1899948
-6.0622921	10.8824005	102.5899963
-6.0622921	10.9312000	100.8799973
-6.0622921	10.9800005	102.6699982
-6.0622921	11.0288000	106.5599976
-6.0622921	11.0776005	104.6100006
-6.0622921	11.1264000	101.4099960
-6.0622921	11.1752005	97.9099960
-6.0622921	11.2240000	99.3300018
-6.0622921	11.2728004	98.9499969
-6.0622921	11.3216000	103.7500000
-6.0622921	11.3704004	104.8199997
-6.0622921	11.4191999	104.7900009
-6.0622921	11.4680004	104.4700012
-6.0622921	11.5167999	103.0699997
-6.0622921	11.5656004	104.0100021
-6.0622921	11.6143999	102.4199982
-6.0622921	11.6632004	103.0800018
-6.0622921	11.7119999	101.2099991
-6.0622921	11.7608004	99.7699966
-6.0622921	11.8095999	103.3099976
-6.0622921	11.8584003	106.5400009
-6.0622921	11.9071999	105.3399963
-6.0622921	11.9560003	104.0599976
-6.0622921	12.0047998	104.3499985
-6.0622921	12.0536003	103.5999985
-6.0622921	12.1023998	104.1800003
-6.0622921	12.1512003	105.2600021
-6.0622921	12.1999998	104.1299973
-6.0622921	12.2488003	106.5199966
-6.0622921	12.2975998	105.6999969
-6.0622921	12.3464003	103.4899979
-6.0622921	12.3951998	103.9700012
-6.0622921	12.4440002	101.1100006
-6.0622921	12.4927998	103.2200012
-6.0622921	12.5416002	104.7600021
-6.0622921	12.5904007	102.2699966
-6.0622921	12.6392002	104.8399963
-6.0622921	12.6880007	104.4700012
-6.0622921	12.7368002	104.6100006
-6.0622921	12.7856007	106.3399963
-6.0622921	12.8344002	102.5000000
-6.0622921	12.8832006	106.4499969
-6.0622921	12.9320002	106.5199966
-6.0622921	12.9808006	106.2200012
-6.0622921	13.0296001	104.8899994
-6.0622921	13.0784006	104.1299973
-6.0622921	13.1272001	102.5599976
-6.0622921	13.1760006	104.2699966

-6.0622921	13.2248001	105.9599991
-6.0622921	13.2736006	106.8899994
-6.0622921	13.3224001	105.2900009
-6.0622921	13.3712006	105.4599991
-6.0622921	13.4200001	105.6800003
-6.0622921	13.4688005	104.3199997
-6.0622921	13.5176001	105.0999985
-6.0622921	13.5664005	102.8300018
-6.0622921	13.6152000	96.3799973
-6.0622921	13.6640005	100.3499985
-6.0622921	13.7128000	99.5199966
-6.0622921	13.7616005	99.5699997
-6.0622921	13.8104000	101.5599976
-6.0622921	13.8592005	103.4399948
-6.0622921	13.9080000	104.0499954
-6.0622921	13.9568005	104.5299988
-6.0622921	14.0056000	107.0100021
-6.0622921	14.0544004	104.5299988
-6.0622921	14.1032000	104.3799973
-6.0622921	14.1520004	105.7799988
-6.0622921	14.2007999	104.6500015
-6.0622921	14.2496004	103.8899994
-6.0622921	14.2983999	99.7699966
-6.0622921	14.3472004	99.8199997
-6.0622921	14.3959999	99.4000015
-6.0622921	14.4448004	99.7099991
-6.0622921	14.4935999	103.5899963
-6.0622921	14.5424004	104.0699997
-6.0622921	14.5911999	103.5899963
-6.0622921	14.6400003	104.1199951
-6.0622921	14.6887999	104.8899994
-6.0622921	14.7376003	106.9000015
-6.0622921	14.7863998	104.7200012
-6.0622921	14.8352003	101.1899948
-6.0622921	14.8839998	102.7699966
-6.0622921	14.9328003	104.8099976
-6.0622921	14.9815998	105.0599976
-6.0622921	15.0304003	102.6699982
-6.0561562	0.3416000	109.8199997
-6.0561562	0.3904000	111.9199982
-6.0561562	0.4392000	108.4000015
-6.0561562	0.4880000	105.1999969
-6.0561562	0.5368000	106.1999969
-6.0561562	0.5856000	105.3399963
-6.0561562	0.6344000	107.9399948
-6.0561562	0.6832000	108.6100006
-6.0561562	0.7320000	105.8600006
-6.0561562	0.7808000	108.2500000
-6.0561562	0.8296000	104.9199982
-6.0561562	0.8784000	104.4199982
-6.0561562	0.9272000	106.9399948
-6.0561562	0.9760000	106.8499985
-6.0561562	1.0247999	107.2600021
-6.0561562	1.0735999	105.3699951
-6.0561562	1.1223999	104.4899979
-6.0561562	1.1711999	102.2900009
-6.0561562	1.2200000	103.7699966
-6.0561562	1.2688000	103.9599991

-6.0561562	1.3176000	105.2900009
-6.0561562	1.3664000	104.9300003
-6.0561562	1.4152000	102.4700012
-6.0561562	1.4640000	105.9300003
-6.0561562	1.5128000	107.8300018
-6.0561562	1.5616000	108.4000015
-6.0561562	1.6104000	109.9599991
-6.0561562	1.6592000	110.4000015
-6.0561562	1.7079999	109.3699951
-6.0561562	1.7567999	107.6800003
-6.0561562	1.8055999	109.8300018
-6.0561562	1.8543999	114.3899994
-6.0561562	1.9031999	114.2099991
-6.0561562	1.9519999	109.1800003
-6.0561562	2.0007999	102.3399963
-6.0561562	2.0495999	104.3600006
-6.0561562	2.0983999	105.2099991
-6.0561562	2.1471999	105.3899994
-6.0561562	2.1959999	107.6599960
-6.0561562	2.2447999	107.4300003
-6.0561562	2.2935998	106.0199966
-6.0561562	2.3423998	102.8799973
-6.0561562	2.3912001	106.6999969
-6.0561562	2.4400001	106.8300018
-6.0561562	2.4888000	107.0599976
-6.0561562	2.5376000	105.1599960
-6.0561562	2.5864000	107.9599991
-6.0561562	2.6352000	107.3099976
-6.0561562	2.6840000	105.2399979
-6.0561562	2.7328000	106.5100021
-6.0561562	2.7816000	105.2999954
-6.0561562	2.8304000	104.0800018
-6.0561562	2.8792000	105.2799988
-6.0561562	2.9280000	105.2999954
-6.0561562	2.9768000	103.8799973
-6.0561562	3.0256000	106.5999985
-6.0561562	3.0743999	106.4399948
-6.0561562	3.1231999	107.2999954
-6.0561562	3.1719999	105.9199982
-6.0561562	3.2207999	106.0800018
-6.0561562	3.2695999	106.2200012
-6.0561562	3.3183999	105.5800018
-6.0561562	3.3671999	104.8300018
-6.0561562	3.4159999	102.8099976
-6.0561562	3.4647999	103.1800003
-6.0561562	3.5135999	102.5699997
-6.0561562	3.5623999	103.8499985
-6.0561562	3.6111999	105.8399963
-6.0561562	3.6599998	105.6999969
-6.0561562	3.7087998	105.9199982
-6.0561562	3.7575998	106.4099960
-6.0561562	3.8063998	101.0699997
-6.0561562	3.8551998	103.4799957
-6.0561562	3.9039998	107.5599976
-6.0561562	3.9527998	108.2900009
-6.0561562	4.0016003	107.6699982
-6.0561562	4.0503998	107.5699997
-6.0561562	4.0991998	105.7299957

-6.0561562	4.1479998	100.2900009
-6.0561562	4.1967998	100.6699982
-6.0561562	4.2455997	103.9700012
-6.0561562	4.2943997	109.0599976
-6.0561562	4.3431997	111.5999985
-6.0561562	4.3919997	109.6800003
-6.0561562	4.4407997	110.5999985
-6.0561562	4.4895997	112.7099991
-6.0561562	4.5383997	114.5699997
-6.0561562	4.5871997	116.6599960
-6.0561562	4.6359997	116.3600006
-6.0561562	4.6847997	116.5800018
-6.0561562	4.7336001	115.7399979
-6.0561562	4.7824001	109.8300018
-6.0561562	4.8312001	106.3799973
-6.0561562	4.8800001	106.3399963
-6.0561562	4.9288001	111.3699951
-6.0561562	4.9776001	108.4099960
-6.0561562	5.0264001	107.6800003
-6.0561562	5.0752001	108.8399963
-6.0561562	5.1240001	111.0199966
-6.0561562	5.1728001	108.6699982
-6.0561562	5.2216001	106.2900009
-6.0561562	5.2704000	107.1999969
-6.0561562	5.3192000	107.7399979
-6.0561562	5.3680000	106.3600006
-6.0561562	5.4168000	105.2399979
-6.0561562	5.4656000	103.0899963
-6.0561562	5.5144000	102.2299957
-6.0561562	5.5632000	102.7099991
-6.0561562	5.6120000	97.5000000
-6.0561562	5.6608000	101.8199997
-6.0561562	5.7096000	106.8899994
-6.0561562	5.7584000	106.4499969
-6.0561562	5.8072000	106.0599976
-6.0561562	5.8559999	104.8499985
-6.0561562	5.9047999	103.1800003
-6.0561562	5.9535999	101.9000015
-6.0561562	6.0023999	102.5800018
-6.0561562	6.0511999	103.8899994
-6.0561562	6.0999999	105.5299988
-6.0561562	6.1487999	105.5699997
-6.0561562	6.1975999	104.8399963
-6.0561562	6.2463999	103.9799957
-6.0561562	6.2951999	104.0199966
-6.0561562	6.3439999	104.9000015
-6.0561562	6.3927999	106.2900009
-6.0561562	6.4415998	106.8199997
-6.0561562	6.4903998	108.2299957
-6.0561562	6.5391998	106.1800003
-6.0561562	6.5879998	106.7500000
-6.0561562	6.6367998	107.0199966
-6.0561562	6.6855998	106.5800018
-6.0561562	6.7343998	102.7999954
-6.0561562	6.7831998	97.7900009
-6.0561562	6.8319998	102.7699966
-6.0561562	6.8807998	104.7299957
-6.0561562	6.9295998	102.2699966

-6.0561562	6.9783998	103.4300003
-6.0561562	7.0271997	106.6800003
-6.0561562	7.0759997	106.1299973
-6.0561562	7.1247997	106.0400009
-6.0561562	7.1735997	105.3699951
-6.0561562	7.2223997	104.3799973
-6.0561562	7.2711997	104.7299957
-6.0561562	7.3199997	103.1599960
-6.0561562	7.3687997	102.0800018
-6.0561562	7.4175997	103.8600006
-6.0561562	7.4663997	101.6599960
-6.0561562	7.5151997	103.1599960
-6.0561562	7.5639997	105.4000015
-6.0561562	7.6127996	104.7600021
-6.0561562	7.6615996	103.2699966
-6.0561562	7.7103996	104.0599976
-6.0561562	7.7591996	101.4799957
-6.0561562	7.8079996	105.0400009
-6.0561562	7.8567996	104.7600021
-6.0561562	7.9056001	101.0899963
-6.0561562	7.9544001	101.3600006
-6.0561562	8.0032005	105.0299988
-6.0561562	8.0520000	105.7099991
-6.0561562	8.1008005	104.0000000
-6.0561562	8.1496000	103.9099960
-6.0561562	8.1984005	105.9700012
-6.0561562	8.2472000	106.3699951
-6.0561562	8.2960005	107.1699982
-6.0561562	8.3448000	104.0499954
-6.0561562	8.3936005	100.5400009
-6.0561562	8.4424000	103.2099991
-6.0561562	8.4912004	100.0400009
-6.0561562	8.5400000	103.9700012
-6.0561562	8.5888004	98.9899979
-6.0561562	8.6375999	103.0100021
-6.0561562	8.6864004	106.3699951
-6.0561562	8.7351999	106.2500000
-6.0561562	8.7840004	104.9899979
-6.0561562	8.8327999	101.2299957
-6.0561562	8.8816004	102.1899948
-6.0561562	8.9303999	102.2200012
-6.0561562	8.9792004	100.2200012
-6.0561562	9.0279999	103.6800003
-6.0561562	9.0768003	104.8699951
-6.0561562	9.1255999	104.6299973
-6.0561562	9.1744003	104.8199997
-6.0561562	9.2231998	106.5400009
-6.0561562	9.2720003	103.5699997
-6.0561562	9.3207998	100.3799973
-6.0561562	9.3696003	101.7999954
-6.0561562	9.4184008	103.5499954
-6.0561562	9.4672003	103.3699951
-6.0561562	9.5160007	100.8799973
-6.0561562	9.5648003	104.8399963
-6.0561562	9.6136007	101.0999985
-6.0561562	9.6624002	104.3899994
-6.0561562	9.7112007	106.0899963
-6.0561562	9.7600002	103.9799957

-6.0561562	9.8088007	103.6299973
-6.0561562	9.8576002	103.2099991
-6.0561562	9.9064007	102.9199982
-6.0561562	9.9552002	102.0699997
-6.0561562	10.0040007	104.6500015
-6.0561562	10.0528002	105.1299973
-6.0561562	10.1016006	104.9199982
-6.0561562	10.1504002	105.3399963
-6.0561562	10.1992006	106.8199997
-6.0561562	10.2480001	106.3699951
-6.0561562	10.2968006	102.6999969
-6.0561562	10.3456001	102.2600021
-6.0561562	10.3944006	104.4899979
-6.0561562	10.4432001	105.3099976
-6.0561562	10.4920006	105.4000015
-6.0561562	10.5408001	103.0999985
-6.0561562	10.5896006	104.5000000
-6.0561562	10.6384001	103.3499985
-6.0561562	10.6872005	102.8600006
-6.0561562	10.7360001	103.3499985
-6.0561562	10.7848005	101.7900009
-6.0561562	10.8336000	104.8099976
-6.0561562	10.8824005	103.2799988
-6.0561562	10.9312000	100.8099976
-6.0561562	10.9800005	104.1100006
-6.0561562	11.0288000	107.0899963
-6.0561562	11.0776005	105.5999985
-6.0561562	11.1264000	100.9399948
-6.0561562	11.1752005	98.8099976
-6.0561562	11.2240000	99.7600021
-6.0561562	11.2728004	98.9700012
-6.0561562	11.3216000	102.0400009
-6.0561562	11.3704004	103.4199982
-6.0561562	11.4191999	104.5599976
-6.0561562	11.4680004	104.0899963
-6.0561562	11.5167999	101.6699982
-6.0561562	11.5656004	103.3300018
-6.0561562	11.6143999	102.4599991
-6.0561562	11.6632004	103.5100021
-6.0561562	11.7119999	102.1199951
-6.0561562	11.7608004	100.2299957
-6.0561562	11.8095999	103.1599960
-6.0561562	11.8584003	106.4099960
-6.0561562	11.9071999	105.7999954
-6.0561562	11.9560003	104.7299957
-6.0561562	12.0047998	104.5000000
-6.0561562	12.0536003	103.1100006
-6.0561562	12.1023998	103.5299988
-6.0561562	12.1512003	104.1599960
-6.0561562	12.1999998	103.8600006
-6.0561562	12.2488003	106.4000015
-6.0561562	12.2975998	105.1999969
-6.0561562	12.3464003	102.7799988
-6.0561562	12.3951998	103.7399979
-6.0561562	12.4440002	102.0299988
-6.0561562	12.4927998	103.8899994
-6.0561562	12.5416002	104.8399963
-6.0561562	12.5904007	102.7799988

-6.0561562	12.6392002	104.9899979
-6.0561562	12.6880007	104.2399979
-6.0561562	12.7368002	103.7299957
-6.0561562	12.7856007	105.8899994
-6.0561562	12.8344002	102.1699982
-6.0561562	12.8832006	105.7799988
-6.0561562	12.9320002	105.3300018
-6.0561562	12.9808006	105.1500015
-6.0561562	13.0296001	104.4499969
-6.0561562	13.0784006	104.3899994
-6.0561562	13.1272001	102.8199997
-6.0561562	13.1760006	103.7699966
-6.0561562	13.2248001	105.1100006
-6.0561562	13.2736006	107.1199951
-6.0561562	13.3224001	106.2200012
-6.0561562	13.3712006	105.5899963
-6.0561562	13.4200001	105.6899948
-6.0561562	13.4688005	104.3300018
-6.0561562	13.5176001	104.7600021
-6.0561562	13.5664005	103.0400009
-6.0561562	13.6152000	95.4499969
-6.0561562	13.6640005	100.4799957
-6.0561562	13.7128000	99.6899948
-6.0561562	13.7616005	98.7999954
-6.0561562	13.8104000	101.3300018
-6.0561562	13.8592005	103.3399963
-6.0561562	13.9080000	104.6999969
-6.0561562	13.9568005	106.1399994
-6.0561562	14.0056000	106.5699997
-6.0561562	14.0544004	103.0999985
-6.0561562	14.1032000	103.2799988
-6.0561562	14.1520004	105.3300018
-6.0561562	14.2007999	104.3199997
-6.0561562	14.2496004	103.7600021
-6.0561562	14.2983999	99.5299988
-6.0561562	14.3472004	100.3799973
-6.0561562	14.3959999	99.7799988
-6.0561562	14.4448004	99.0999985
-6.0561562	14.4935999	102.7200012
-6.0561562	14.5424004	104.0800018
-6.0561562	14.5911999	103.2099991
-6.0561562	14.6400003	104.3099976
-6.0561562	14.6887999	105.6299973
-6.0561562	14.7376003	106.5800018
-6.0561562	14.7863998	103.4799957
-6.0561562	14.8352003	99.5599976
-6.0561562	14.8839998	102.1899948
-6.0561562	14.9328003	104.9899979
-6.0561562	14.9815998	104.8499985
-6.0561562	15.0304003	103.0000000
-6.0500202	0.3416000	110.4599991
-6.0500202	0.3904000	112.4899979
-6.0500202	0.4392000	109.2500000
-6.0500202	0.4880000	105.0899963
-6.0500202	0.5368000	106.5199966
-6.0500202	0.5856000	105.6500015
-6.0500202	0.6344000	107.5000000
-6.0500202	0.6832000	108.5100021

-6.0500202	0.7320000	104.8699951
-6.0500202	0.7808000	108.4000015
-6.0500202	0.8296000	105.8600006
-6.0500202	0.8784000	106.5000000
-6.0500202	0.9272000	108.2299957
-6.0500202	0.9760000	107.4700012
-6.0500202	1.0247999	107.8600006
-6.0500202	1.0735999	104.8399963
-6.0500202	1.1223999	103.5299988
-6.0500202	1.1711999	101.8300018
-6.0500202	1.2200000	103.5699997
-6.0500202	1.2688000	103.7999954
-6.0500202	1.3176000	106.5499954
-6.0500202	1.3664000	105.3899994
-6.0500202	1.4152000	101.7099991
-6.0500202	1.4640000	105.3499985
-6.0500202	1.5128000	106.9099960
-6.0500202	1.5616000	107.1500015
-6.0500202	1.6104000	108.7699966
-6.0500202	1.6592000	110.3399963
-6.0500202	1.7079999	109.4199982
-6.0500202	1.7567999	108.0599976
-6.0500202	1.8055999	109.9199982
-6.0500202	1.8543999	114.0499954
-6.0500202	1.9031999	114.1800003
-6.0500202	1.9519999	109.6699982
-6.0500202	2.0007999	103.4499969
-6.0500202	2.0495999	104.6699982
-6.0500202	2.0983999	105.9499969
-6.0500202	2.1471999	104.7799988
-6.0500202	2.1959999	107.6999969
-6.0500202	2.2447999	106.2900009
-6.0500202	2.2935998	106.4700012
-6.0500202	2.3423998	102.9499969
-6.0500202	2.3912001	106.7099991
-6.0500202	2.4400001	106.9399948
-6.0500202	2.4888000	106.4599991
-6.0500202	2.5376000	105.0899963
-6.0500202	2.5864000	107.9399948
-6.0500202	2.6352000	107.0599976
-6.0500202	2.6840000	104.5100021
-6.0500202	2.7328000	106.9599991
-6.0500202	2.7816000	106.2699966
-6.0500202	2.8304000	103.9000015
-6.0500202	2.8792000	103.5599976
-6.0500202	2.9280000	105.4399948
-6.0500202	2.9768000	103.5699997
-6.0500202	3.0256000	106.0800018
-6.0500202	3.0743999	105.5299988
-6.0500202	3.1231999	106.4599991
-6.0500202	3.1719999	106.4000015
-6.0500202	3.2207999	107.2200012
-6.0500202	3.2695999	106.2600021
-6.0500202	3.3183999	105.8300018
-6.0500202	3.3671999	105.4499969
-6.0500202	3.4159999	103.2900009
-6.0500202	3.4647999	103.1599960
-6.0500202	3.5135999	102.4599991

-6.0500202	3.5623999	103.1399994
-6.0500202	3.6111999	105.0800018
-6.0500202	3.6599998	105.5499954
-6.0500202	3.7087998	104.6399994
-6.0500202	3.7575998	105.7699966
-6.0500202	3.8063998	101.7600021
-6.0500202	3.8551998	104.3300018
-6.0500202	3.9039998	107.5699997
-6.0500202	3.9527998	108.1100006
-6.0500202	4.0016003	107.3399963
-6.0500202	4.0503998	107.4899979
-6.0500202	4.0991998	106.0000000
-6.0500202	4.1479998	99.3600006
-6.0500202	4.1967998	100.0199966
-6.0500202	4.2455997	103.2799988
-6.0500202	4.2943997	108.1299973
-6.0500202	4.3431997	111.2500000
-6.0500202	4.3919997	110.3499985
-6.0500202	4.4407997	110.9300003
-6.0500202	4.4895997	112.7900009
-6.0500202	4.5383997	114.8899994
-6.0500202	4.5871997	117.4799957
-6.0500202	4.6359997	117.2200012
-6.0500202	4.6847997	117.1100006
-6.0500202	4.7336001	115.7299957
-6.0500202	4.7824001	109.5299988
-6.0500202	4.8312001	107.4099960
-6.0500202	4.8800001	108.5299988
-6.0500202	4.9288001	112.0699997
-6.0500202	4.9776001	108.5899963
-6.0500202	5.0264001	108.3300018
-6.0500202	5.0752001	109.6199951
-6.0500202	5.1240001	110.8899994
-6.0500202	5.1728001	108.6199951
-6.0500202	5.2216001	106.1299973
-6.0500202	5.2704000	107.2099991
-6.0500202	5.3192000	108.1399994
-6.0500202	5.3680000	106.6699982
-6.0500202	5.4168000	104.8600006
-6.0500202	5.4656000	103.3499985
-6.0500202	5.5144000	103.1299973
-6.0500202	5.5632000	103.8699951
-6.0500202	5.6120000	98.8199997
-6.0500202	5.6608000	101.7299957
-6.0500202	5.7096000	106.4899979
-6.0500202	5.7584000	105.6699982
-6.0500202	5.8072000	105.5599976
-6.0500202	5.8559999	104.4899979
-6.0500202	5.9047999	103.9199982
-6.0500202	5.9535999	102.5999985
-6.0500202	6.0023999	102.4199982
-6.0500202	6.0511999	103.0000000
-6.0500202	6.0999999	104.5100021
-6.0500202	6.1487999	104.7999954
-6.0500202	6.1975999	103.2799988
-6.0500202	6.2463999	103.5599976
-6.0500202	6.2951999	104.8899994
-6.0500202	6.3439999	104.2799988

-6.0500202	6.3927999	106.1399994
-6.0500202	6.4415998	108.0899963
-6.0500202	6.4903998	108.9499969
-6.0500202	6.5391998	105.9599991
-6.0500202	6.5879998	106.5000000
-6.0500202	6.6367998	107.0199966
-6.0500202	6.6855998	106.9000015
-6.0500202	6.7343998	103.3600006
-6.0500202	6.7831998	98.0599976
-6.0500202	6.8319998	102.5800018
-6.0500202	6.8807998	104.6299973
-6.0500202	6.9295998	101.4099960
-6.0500202	6.9783998	102.7200012
-6.0500202	7.0271997	107.1399994
-6.0500202	7.0759997	106.5599976
-6.0500202	7.1247997	106.6699982
-6.0500202	7.1735997	104.8399963
-6.0500202	7.2223997	104.4099960
-6.0500202	7.2711997	104.5400009
-6.0500202	7.3199997	102.5199966
-6.0500202	7.3687997	101.6299973
-6.0500202	7.4175997	103.5000000
-6.0500202	7.4663997	101.9000015
-6.0500202	7.5151997	103.3199997
-6.0500202	7.5639997	105.2299957
-6.0500202	7.6127996	104.4099960
-6.0500202	7.6615996	103.5899963
-6.0500202	7.7103996	104.4300003
-6.0500202	7.7591996	101.7299957
-6.0500202	7.8079996	105.0499954
-6.0500202	7.8567996	104.1599960
-6.0500202	7.9056001	100.5800018
-6.0500202	7.9544001	101.5699997
-6.0500202	8.0032005	104.7999954
-6.0500202	8.0520000	106.0100021
-6.0500202	8.1008005	104.0199966
-6.0500202	8.1496000	104.0999985
-6.0500202	8.1984005	106.4099960
-6.0500202	8.2472000	107.7200012
-6.0500202	8.2960005	107.6399994
-6.0500202	8.3448000	103.6699982
-6.0500202	8.3936005	100.6800003
-6.0500202	8.4424000	102.5899963
-6.0500202	8.4912004	100.1800003
-6.0500202	8.5400000	104.5499954
-6.0500202	8.5888004	99.7600021
-6.0500202	8.6375999	103.4399948
-6.0500202	8.6864004	107.0699997
-6.0500202	8.7351999	106.4700012
-6.0500202	8.7840004	104.5100021
-6.0500202	8.8327999	100.5899963
-6.0500202	8.8816004	101.9599991
-6.0500202	8.9303999	102.5800018
-6.0500202	8.9792004	101.1299973
-6.0500202	9.0279999	103.2799988
-6.0500202	9.0768003	104.0400009
-6.0500202	9.1255999	104.8899994
-6.0500202	9.1744003	104.5899963

-6.0500202	9.2231998	106.6399994
-6.0500202	9.2720003	103.9000015
-6.0500202	9.3207998	100.2099991
-6.0500202	9.3696003	102.0800018
-6.0500202	9.4184008	103.5000000
-6.0500202	9.4672003	103.5299988
-6.0500202	9.5160007	100.0400009
-6.0500202	9.5648003	105.8600006
-6.0500202	9.6136007	102.5199966
-6.0500202	9.6624002	104.5400009
-6.0500202	9.7112007	106.0800018
-6.0500202	9.7600002	103.9799957
-6.0500202	9.8088007	103.4799957
-6.0500202	9.8576002	102.9799957
-6.0500202	9.9064007	103.2799988
-6.0500202	9.9552002	103.0000000
-6.0500202	10.0040007	104.0000000
-6.0500202	10.0528002	104.3300018
-6.0500202	10.1016006	105.4399948
-6.0500202	10.1504002	105.8699951
-6.0500202	10.1992006	107.2299957
-6.0500202	10.2480001	107.3099976
-6.0500202	10.2968006	103.8499985
-6.0500202	10.3456001	102.1199951
-6.0500202	10.3944006	104.5899963
-6.0500202	10.4432001	105.2900009
-6.0500202	10.4920006	104.6999969
-6.0500202	10.5408001	101.9399948
-6.0500202	10.5896006	103.7999954
-6.0500202	10.6384001	103.1100006
-6.0500202	10.6872005	103.5100021
-6.0500202	10.7360001	102.7500000
-6.0500202	10.7848005	102.3300018
-6.0500202	10.8336000	105.2600021
-6.0500202	10.8824005	103.9599991
-6.0500202	10.9312000	100.9099960
-6.0500202	10.9800005	105.3499985
-6.0500202	11.0288000	107.5299988
-6.0500202	11.0776005	105.7500000
-6.0500202	11.1264000	100.5100021
-6.0500202	11.1752005	99.2500000
-6.0500202	11.2240000	100.3300018
-6.0500202	11.2728004	99.2099991
-6.0500202	11.3216000	99.7799988
-6.0500202	11.3704004	101.8399963
-6.0500202	11.4191999	104.3699951
-6.0500202	11.4680004	103.8799973
-6.0500202	11.5167999	101.0299988
-6.0500202	11.5656004	103.2500000
-6.0500202	11.6143999	103.0100021
-6.0500202	11.6632004	104.0499954
-6.0500202	11.7119999	102.7399979
-6.0500202	11.7608004	100.6199951
-6.0500202	11.8095999	103.1999969
-6.0500202	11.8584003	106.1299973
-6.0500202	11.9071999	105.7399979
-6.0500202	11.9560003	105.3300018
-6.0500202	12.0047998	104.8899994

-6.0500202	12.0536003	102.3399963
-6.0500202	12.1023998	102.9199982
-6.0500202	12.1512003	103.5699997
-6.0500202	12.1999998	103.1599960
-6.0500202	12.2488003	105.3799973
-6.0500202	12.2975998	104.3099976
-6.0500202	12.3464003	102.1399994
-6.0500202	12.3951998	103.1999969
-6.0500202	12.4440002	102.5299988
-6.0500202	12.4927998	104.5199966
-6.0500202	12.5416002	105.1899948
-6.0500202	12.5904007	103.6899948
-6.0500202	12.6392002	105.2799988
-6.0500202	12.6880007	104.3099976
-6.0500202	12.7368002	103.8699951
-6.0500202	12.7856007	105.9399948
-6.0500202	12.8344002	102.7999954
-6.0500202	12.8832006	105.1500015
-6.0500202	12.9320002	104.1999969
-6.0500202	12.9808006	104.6899948
-6.0500202	13.0296001	104.8600006
-6.0500202	13.0784006	105.1699982
-6.0500202	13.1272001	103.4599991
-6.0500202	13.1760006	104.0100021
-6.0500202	13.2248001	104.6599960
-6.0500202	13.2736006	107.4799957
-6.0500202	13.3224001	106.7299957
-6.0500202	13.3712006	105.2500000
-6.0500202	13.4200001	105.6399994
-6.0500202	13.4688005	104.5000000
-6.0500202	13.5176001	104.6299973
-6.0500202	13.5664005	103.6500015
-6.0500202	13.6152000	96.3199997
-6.0500202	13.6640005	101.1199951
-6.0500202	13.7128000	100.6899948
-6.0500202	13.7616005	98.8799973
-6.0500202	13.8104000	101.3499985
-6.0500202	13.8592005	103.5499954
-6.0500202	13.9080000	105.2299957
-6.0500202	13.9568005	107.0299988
-6.0500202	14.0056000	105.3699951
-6.0500202	14.0544004	101.6299973
-6.0500202	14.1032000	102.1500015
-6.0500202	14.1520004	104.7299957
-6.0500202	14.2007999	103.6299973
-6.0500202	14.2496004	103.6899948
-6.0500202	14.2983999	99.9799957
-6.0500202	14.3472004	100.4000015
-6.0500202	14.3959999	100.0699997
-6.0500202	14.4448004	98.8799973
-6.0500202	14.4935999	102.2900009
-6.0500202	14.5424004	103.8099976
-6.0500202	14.5911999	102.6899948
-6.0500202	14.6400003	104.6100006
-6.0500202	14.6887999	106.4300003
-6.0500202	14.7376003	106.2999954
-6.0500202	14.7863998	102.5499954
-6.0500202	14.8352003	98.2299957

-6.0500202	14.8839998	102.4399948
-6.0500202	14.9328003	105.2900009
-6.0500202	14.9815998	104.8799973
-6.0500202	15.0304003	103.6699982
-6.0438843	0.3416000	110.2600021
-6.0438843	0.3904000	112.3199997
-6.0438843	0.4392000	109.5899963
-6.0438843	0.4880000	105.7600021
-6.0438843	0.5368000	106.5899963
-6.0438843	0.5856000	105.3099976
-6.0438843	0.6344000	106.6399994
-6.0438843	0.6832000	108.3799973
-6.0438843	0.7320000	105.6199951
-6.0438843	0.7808000	108.6100006
-6.0438843	0.8296000	106.8899994
-6.0438843	0.8784000	108.4700012
-6.0438843	0.9272000	108.9599991
-6.0438843	0.9760000	107.7900009
-6.0438843	1.0247999	108.0499954
-6.0438843	1.0735999	104.6999969
-6.0438843	1.1223999	102.9399948
-6.0438843	1.1711999	101.9899979
-6.0438843	1.2200000	102.9099960
-6.0438843	1.2688000	103.9199982
-6.0438843	1.3176000	107.4499969
-6.0438843	1.3664000	106.4499969
-6.0438843	1.4152000	101.6500015
-6.0438843	1.4640000	104.8399963
-6.0438843	1.5128000	106.0999985
-6.0438843	1.5616000	105.9199982
-6.0438843	1.6104000	107.3799973
-6.0438843	1.6592000	110.2500000
-6.0438843	1.7079999	110.0299988
-6.0438843	1.7567999	108.3899994
-6.0438843	1.8055999	109.1500015
-6.0438843	1.8543999	113.2600021
-6.0438843	1.9031999	114.4499969
-6.0438843	1.9519999	110.7299957
-6.0438843	2.0007999	104.6999969
-6.0438843	2.0495999	104.4599991
-6.0438843	2.0983999	106.4199982
-6.0438843	2.1471999	104.5400009
-6.0438843	2.1959999	106.9499969
-6.0438843	2.2447999	104.2799988
-6.0438843	2.2935998	106.6199951
-6.0438843	2.3423998	103.6999969
-6.0438843	2.3912001	106.9399948
-6.0438843	2.4400001	106.5899963
-6.0438843	2.4888000	105.4899979
-6.0438843	2.5376000	104.5499954
-6.0438843	2.5864000	107.4099960
-6.0438843	2.6352000	106.6800003
-6.0438843	2.6840000	103.8699951
-6.0438843	2.7328000	107.5999985
-6.0438843	2.7816000	107.1999969
-6.0438843	2.8304000	103.3499985
-6.0438843	2.8792000	101.2699966
-6.0438843	2.9280000	104.9099960

-6.0438843	2.9768000	102.9799957
-6.0438843	3.0256000	105.4700012
-6.0438843	3.0743999	104.2200012
-6.0438843	3.1231999	105.6899948
-6.0438843	3.1719999	106.6699982
-6.0438843	3.2207999	107.1999969
-6.0438843	3.2695999	106.4599991
-6.0438843	3.3183999	106.3399963
-6.0438843	3.3671999	105.9599991
-6.0438843	3.4159999	103.6299973
-6.0438843	3.4647999	103.1999969
-6.0438843	3.5135999	102.6899948
-6.0438843	3.5623999	102.6999969
-6.0438843	3.6111999	104.4399948
-6.0438843	3.6599998	105.5499954
-6.0438843	3.7087998	103.5000000
-6.0438843	3.7575998	104.8399963
-6.0438843	3.8063998	102.2600021
-6.0438843	3.8551998	104.9899979
-6.0438843	3.9039998	107.3399963
-6.0438843	3.9527998	107.6199951
-6.0438843	4.0016003	107.2799988
-6.0438843	4.0503998	107.6999969
-6.0438843	4.0991998	106.5699997
-6.0438843	4.1479998	99.2099991
-6.0438843	4.1967998	100.1399994
-6.0438843	4.2455997	103.2500000
-6.0438843	4.2943997	107.3799973
-6.0438843	4.3431997	111.0100021
-6.0438843	4.3919997	111.0100021
-6.0438843	4.4407997	110.7500000
-6.0438843	4.4895997	112.5299988
-6.0438843	4.5383997	115.5100021
-6.0438843	4.5871997	117.6299973
-6.0438843	4.6359997	116.8600006
-6.0438843	4.6847997	117.0100021
-6.0438843	4.7336001	115.2200012
-6.0438843	4.7824001	109.1100006
-6.0438843	4.8312001	108.2799988
-6.0438843	4.8800001	110.1999969
-6.0438843	4.9288001	112.1399994
-6.0438843	4.9776001	108.6299973
-6.0438843	5.0264001	109.2200012
-6.0438843	5.0752001	110.1800003
-6.0438843	5.1240001	110.4199982
-6.0438843	5.1728001	108.0599976
-6.0438843	5.2216001	105.9499969
-6.0438843	5.2704000	107.3099976
-6.0438843	5.3192000	108.4000015
-6.0438843	5.3680000	106.4799957
-6.0438843	5.4168000	103.9799957
-6.0438843	5.4656000	103.3600006
-6.0438843	5.5144000	104.1899948
-6.0438843	5.5632000	105.3300018
-6.0438843	5.6120000	101.0199966
-6.0438843	5.6608000	102.9399948
-6.0438843	5.7096000	106.3099976
-6.0438843	5.7584000	104.4700012

-6.0438843	5.8072000	104.9499969
-6.0438843	5.8559999	103.8399963
-6.0438843	5.9047999	103.9199982
-6.0438843	5.9535999	103.3399963
-6.0438843	6.0023999	102.1699982
-6.0438843	6.0511999	102.6599960
-6.0438843	6.0999999	104.0499954
-6.0438843	6.1487999	104.3399963
-6.0438843	6.1975999	102.0199966
-6.0438843	6.2463999	103.8799973
-6.0438843	6.2951999	105.5100021
-6.0438843	6.3439999	103.0999985
-6.0438843	6.3927999	105.4099960
-6.0438843	6.4415998	108.7099991
-6.0438843	6.4903998	109.6699982
-6.0438843	6.5391998	106.8099976
-6.0438843	6.5879998	106.5699997
-6.0438843	6.6367998	106.5400009
-6.0438843	6.6855998	106.4399948
-6.0438843	6.7343998	103.5400009
-6.0438843	6.7831998	99.7799988
-6.0438843	6.8319998	102.1899948
-6.0438843	6.8807998	105.1899948
-6.0438843	6.9295998	102.2399979
-6.0438843	6.9783998	103.4599991
-6.0438843	7.0271997	107.5199966
-6.0438843	7.0759997	106.7299957
-6.0438843	7.1247997	107.0999985
-6.0438843	7.1735997	103.7699966
-6.0438843	7.2223997	104.1299973
-6.0438843	7.2711997	104.1999969
-6.0438843	7.3199997	102.0999985
-6.0438843	7.3687997	101.4899979
-6.0438843	7.4175997	103.2999954
-6.0438843	7.4663997	102.2399979
-6.0438843	7.5151997	103.8499985
-6.0438843	7.5639997	104.7900009
-6.0438843	7.6127996	103.6500015
-6.0438843	7.6615996	103.5299988
-6.0438843	7.7103996	104.5800018
-6.0438843	7.7591996	101.3799973
-6.0438843	7.8079996	104.5800018
-6.0438843	7.8567996	103.2799988
-6.0438843	7.9056001	100.0100021
-6.0438843	7.9544001	101.5000000
-6.0438843	8.0032005	104.0299988
-6.0438843	8.0520000	105.3099976
-6.0438843	8.1008005	104.3099976
-6.0438843	8.1496000	104.3499985
-6.0438843	8.1984005	106.1299973
-6.0438843	8.2472000	107.6800003
-6.0438843	8.2960005	107.3300018
-6.0438843	8.3448000	103.5499954
-6.0438843	8.3936005	100.8399963
-6.0438843	8.4424000	102.0299988
-6.0438843	8.4912004	99.9700012
-6.0438843	8.5400000	105.1100006
-6.0438843	8.5888004	101.3699951

-6.0438843	8.6375999	103.6399994
-6.0438843	8.6864004	106.8099976
-6.0438843	8.7351999	106.2600021
-6.0438843	8.7840004	103.7900009
-6.0438843	8.8327999	100.4599991
-6.0438843	8.8816004	101.7500000
-6.0438843	8.9303999	102.8199997
-6.0438843	8.9792004	102.0499954
-6.0438843	9.0279999	103.3399963
-6.0438843	9.0768003	103.7699966
-6.0438843	9.1255999	105.3799973
-6.0438843	9.1744003	105.1800003
-6.0438843	9.2231998	106.5800018
-6.0438843	9.2720003	104.1299973
-6.0438843	9.3207998	100.6699982
-6.0438843	9.3696003	102.7500000
-6.0438843	9.4184008	103.4799957
-6.0438843	9.4672003	103.0899963
-6.0438843	9.5160007	100.5299988
-6.0438843	9.5648003	105.8799973
-6.0438843	9.6136007	103.4199982
-6.0438843	9.6624002	104.2500000
-6.0438843	9.7112007	105.7799988
-6.0438843	9.7600002	103.8600006
-6.0438843	9.8088007	103.7500000
-6.0438843	9.8576002	103.3499985
-6.0438843	9.9064007	103.3899994
-6.0438843	9.9552002	102.9899979
-6.0438843	10.0040007	103.2900009
-6.0438843	10.0528002	103.3300018
-6.0438843	10.1016006	105.5999985
-6.0438843	10.1504002	106.0299988
-6.0438843	10.1992006	106.7999954
-6.0438843	10.2480001	107.4300003
-6.0438843	10.2968006	105.0100021
-6.0438843	10.3456001	102.5899963
-6.0438843	10.3944006	105.2600021
-6.0438843	10.4432001	105.5400009
-6.0438843	10.4920006	104.0899963
-6.0438843	10.5408001	101.7900009
-6.0438843	10.5896006	103.1199951
-6.0438843	10.6384001	102.9000015
-6.0438843	10.6872005	104.2699966
-6.0438843	10.7360001	102.2399979
-6.0438843	10.7848005	102.6199951
-6.0438843	10.8336000	105.1599960
-6.0438843	10.8824005	104.0800018
-6.0438843	10.9312000	101.4899979
-6.0438843	10.9800005	105.7799988
-6.0438843	11.0288000	107.5800018
-6.0438843	11.0776005	105.5800018
-6.0438843	11.1264000	100.0199966
-6.0438843	11.1752005	100.5999985
-6.0438843	11.2240000	101.5199966
-6.0438843	11.2728004	100.1100006
-6.0438843	11.3216000	98.0199966
-6.0438843	11.3704004	101.0800018
-6.0438843	11.4191999	104.1899948

-6.0438843	11.4680004	103.8300018
-6.0438843	11.5167999	101.6100006
-6.0438843	11.5656004	103.5599976
-6.0438843	11.6143999	103.0599976
-6.0438843	11.6632004	104.3799973
-6.0438843	11.7119999	103.0599976
-6.0438843	11.7608004	100.7699966
-6.0438843	11.8095999	103.8799973
-6.0438843	11.8584003	105.3699951
-6.0438843	11.9071999	105.1899948
-6.0438843	11.9560003	105.7999954
-6.0438843	12.0047998	105.3099976
-6.0438843	12.0536003	102.0899963
-6.0438843	12.1023998	102.4099960
-6.0438843	12.1512003	103.4099960
-6.0438843	12.1999998	103.0800018
-6.0438843	12.2488003	104.1399994
-6.0438843	12.2975998	103.3699951
-6.0438843	12.3464003	101.6399994
-6.0438843	12.3951998	101.7799988
-6.0438843	12.4440002	102.5000000
-6.0438843	12.4927998	104.1800003
-6.0438843	12.5416002	105.4300003
-6.0438843	12.5904007	104.6999969
-6.0438843	12.6392002	105.7399979
-6.0438843	12.6880007	104.8399963
-6.0438843	12.7368002	104.5100021
-6.0438843	12.7856007	105.8300018
-6.0438843	12.8344002	103.1299973
-6.0438843	12.8832006	105.0899963
-6.0438843	12.9320002	104.1800003
-6.0438843	12.9808006	104.5400009
-6.0438843	13.0296001	105.0499954
-6.0438843	13.0784006	106.0599976
-6.0438843	13.1272001	104.1199951
-6.0438843	13.1760006	104.3499985
-6.0438843	13.2248001	104.6599960
-6.0438843	13.2736006	107.3899994
-6.0438843	13.3224001	106.8600006
-6.0438843	13.3712006	104.7500000
-6.0438843	13.4200001	105.2699966
-6.0438843	13.4688005	104.3899994
-6.0438843	13.5176001	104.6299973
-6.0438843	13.5664005	103.9799957
-6.0438843	13.6152000	97.5999985
-6.0438843	13.6640005	102.2999954
-6.0438843	13.7128000	101.7900009
-6.0438843	13.7616005	99.5899963
-6.0438843	13.8104000	101.5100021
-6.0438843	13.8592005	103.7399979
-6.0438843	13.9080000	105.5499954
-6.0438843	13.9568005	107.0199966
-6.0438843	14.0056000	103.7299957
-6.0438843	14.0544004	100.8199997
-6.0438843	14.1032000	101.6999969
-6.0438843	14.1520004	104.3600006
-6.0438843	14.2007999	102.6899948
-6.0438843	14.2496004	103.5299988

-6.0438843	14.2983999	101.2099991
-6.0438843	14.3472004	101.4399948
-6.0438843	14.3959999	100.4199982
-6.0438843	14.4448004	98.7999954
-6.0438843	14.4935999	102.1199951
-6.0438843	14.5424004	103.9399948
-6.0438843	14.5911999	102.6100006
-6.0438843	14.6400003	104.6800003
-6.0438843	14.6887999	107.0400009
-6.0438843	14.7376003	106.1699982
-6.0438843	14.7863998	102.0400009
-6.0438843	14.8352003	98.1100006
-6.0438843	14.8839998	102.6399994
-6.0438843	14.9328003	104.8300018
-6.0438843	14.9815998	103.9099960
-6.0438843	15.0304003	102.9499969
-6.0377483	0.3416000	109.5000000
-6.0377483	0.3904000	111.8199997
-6.0377483	0.4392000	109.8199997
-6.0377483	0.4880000	106.8199997
-6.0377483	0.5368000	107.0299988
-6.0377483	0.5856000	105.6399994
-6.0377483	0.6344000	105.9099960
-6.0377483	0.6832000	107.9899979
-6.0377483	0.7320000	105.8300018
-6.0377483	0.7808000	108.9099960
-6.0377483	0.8296000	107.3300018
-6.0377483	0.8784000	109.4199982
-6.0377483	0.9272000	109.3699951
-6.0377483	0.9760000	108.2799988
-6.0377483	1.0247999	107.6399994
-6.0377483	1.0735999	104.8899994
-6.0377483	1.1223999	103.1699982
-6.0377483	1.1711999	102.5400009
-6.0377483	1.2200000	102.8799973
-6.0377483	1.2688000	104.5199966
-6.0377483	1.3176000	107.7299957
-6.0377483	1.3664000	107.5999985
-6.0377483	1.4152000	101.6699982
-6.0377483	1.4640000	104.3499985
-6.0377483	1.5128000	105.9399948
-6.0377483	1.5616000	105.6699982
-6.0377483	1.6104000	105.9199982
-6.0377483	1.6592000	109.7200012
-6.0377483	1.7079999	110.2799988
-6.0377483	1.7567999	108.5199966
-6.0377483	1.8055999	108.8899994
-6.0377483	1.8543999	113.2600021
-6.0377483	1.9031999	114.8799973
-6.0377483	1.9519999	111.7399979
-6.0377483	2.0007999	105.3099976
-6.0377483	2.0495999	103.7099991
-6.0377483	2.0983999	106.1599960
-6.0377483	2.1471999	104.0000000
-6.0377483	2.1959999	106.4300003
-6.0377483	2.2447999	101.9799957
-6.0377483	2.2935998	106.8399963
-6.0377483	2.3423998	104.0999985

-6.0377483	2.3912001	106.6100006
-6.0377483	2.4400001	105.8099976
-6.0377483	2.4888000	105.4700012
-6.0377483	2.5376000	104.9000015
-6.0377483	2.5864000	107.1699982
-6.0377483	2.6352000	105.6899948
-6.0377483	2.6840000	102.0999985
-6.0377483	2.7328000	107.3899994
-6.0377483	2.7816000	107.1100006
-6.0377483	2.8304000	102.6699982
-6.0377483	2.8792000	98.9799957
-6.0377483	2.9280000	104.2699966
-6.0377483	2.9768000	102.5199966
-6.0377483	3.0256000	105.0100021
-6.0377483	3.0743999	103.5499954
-6.0377483	3.1231999	104.2299957
-6.0377483	3.1719999	106.6299973
-6.0377483	3.2207999	106.6100006
-6.0377483	3.2695999	106.3600006
-6.0377483	3.3183999	106.4099960
-6.0377483	3.3671999	105.4899979
-6.0377483	3.4159999	103.7999954
-6.0377483	3.4647999	103.2500000
-6.0377483	3.5135999	102.8499985
-6.0377483	3.5623999	102.4000015
-6.0377483	3.6111999	103.7999954
-6.0377483	3.6599998	105.8600006
-6.0377483	3.7087998	103.8099976
-6.0377483	3.7575998	104.5499954
-6.0377483	3.8063998	103.2600021
-6.0377483	3.8551998	105.2999954
-6.0377483	3.9039998	106.5599976
-6.0377483	3.9527998	106.9499969
-6.0377483	4.0016003	107.2200012
-6.0377483	4.0503998	107.9700012
-6.0377483	4.0991998	107.4300003
-6.0377483	4.1479998	101.3699951
-6.0377483	4.1967998	101.3199997
-6.0377483	4.2455997	103.0699997
-6.0377483	4.2943997	106.6500015
-6.0377483	4.3431997	110.7999954
-6.0377483	4.3919997	111.4799957
-6.0377483	4.4407997	109.9599991
-6.0377483	4.4895997	111.5000000
-6.0377483	4.5383997	115.4000015
-6.0377483	4.5871997	117.5899963
-6.0377483	4.6359997	116.8099976
-6.0377483	4.6847997	116.8699951
-6.0377483	4.7336001	114.6599960
-6.0377483	4.7824001	108.7399979
-6.0377483	4.8312001	109.1100006
-6.0377483	4.8800001	111.6899948
-6.0377483	4.9288001	111.3600006
-6.0377483	4.9776001	107.6599960
-6.0377483	5.0264001	109.6299973
-6.0377483	5.0752001	110.5000000
-6.0377483	5.1240001	109.4300003
-6.0377483	5.1728001	107.0499954

-6.0377483	5.2216001	105.6999969
-6.0377483	5.2704000	107.5000000
-6.0377483	5.3192000	108.7699966
-6.0377483	5.3680000	106.4399948
-6.0377483	5.4168000	102.9000015
-6.0377483	5.4656000	102.5199966
-6.0377483	5.5144000	104.7699966
-6.0377483	5.5632000	106.5400009
-6.0377483	5.6120000	103.1599960
-6.0377483	5.6608000	104.1399994
-6.0377483	5.7096000	106.0499954
-6.0377483	5.7584000	103.6100006
-6.0377483	5.8072000	104.3600006
-6.0377483	5.8559999	103.0100021
-6.0377483	5.9047999	103.9499969
-6.0377483	5.9535999	103.9199982
-6.0377483	6.0023999	101.6399994
-6.0377483	6.0511999	102.2500000
-6.0377483	6.0999999	103.9199982
-6.0377483	6.1487999	104.4399948
-6.0377483	6.1975999	101.0000000
-6.0377483	6.2463999	103.9899979
-6.0377483	6.2951999	105.6299973
-6.0377483	6.3439999	102.1399994
-6.0377483	6.3927999	104.5299988
-6.0377483	6.4415998	108.5800018
-6.0377483	6.4903998	109.6699982
-6.0377483	6.5391998	107.1699982
-6.0377483	6.5879998	105.8699951
-6.0377483	6.6367998	105.6500015
-6.0377483	6.6855998	105.6199951
-6.0377483	6.7343998	103.1899948
-6.0377483	6.7831998	100.9700012
-6.0377483	6.8319998	101.6500015
-6.0377483	6.8807998	105.5599976
-6.0377483	6.9295998	102.6899948
-6.0377483	6.9783998	103.6500015
-6.0377483	7.0271997	106.9799957
-6.0377483	7.0759997	105.8899994
-6.0377483	7.1247997	106.4099960
-6.0377483	7.1735997	102.3099976
-6.0377483	7.2223997	103.9700012
-6.0377483	7.2711997	103.8600006
-6.0377483	7.3199997	100.4499969
-6.0377483	7.3687997	101.5800018
-6.0377483	7.4175997	103.2299957
-6.0377483	7.4663997	101.9499969
-6.0377483	7.5151997	103.9499969
-6.0377483	7.5639997	104.1599960
-6.0377483	7.6127996	103.1299973
-6.0377483	7.6615996	103.7200012
-6.0377483	7.7103996	104.8300018
-6.0377483	7.7591996	102.1199951
-6.0377483	7.8079996	103.9499969
-6.0377483	7.8567996	102.9000015
-6.0377483	7.9056001	100.6999969
-6.0377483	7.9544001	102.3699951
-6.0377483	8.0032005	103.3899994

-6.0377483	8.0520000	104.4000015
-6.0377483	8.1008005	105.3199997
-6.0377483	8.1496000	104.9599991
-6.0377483	8.1984005	105.8499985
-6.0377483	8.2472000	107.4099960
-6.0377483	8.2960005	106.7900009
-6.0377483	8.3448000	103.2200012
-6.0377483	8.3936005	100.9399948
-6.0377483	8.4424000	101.4199982
-6.0377483	8.4912004	100.4799957
-6.0377483	8.5400000	105.6899948
-6.0377483	8.5888004	102.6699982
-6.0377483	8.6375999	103.7900009
-6.0377483	8.6864004	106.4799957
-6.0377483	8.7351999	106.0599976
-6.0377483	8.7840004	103.1399994
-6.0377483	8.8327999	100.5499954
-6.0377483	8.8816004	101.5699997
-6.0377483	8.9303999	103.1999969
-6.0377483	8.9792004	103.3899994
-6.0377483	9.0279999	103.8199997
-6.0377483	9.0768003	103.7099991
-6.0377483	9.1255999	105.7500000
-6.0377483	9.1744003	105.7999954
-6.0377483	9.2231998	106.6100006
-6.0377483	9.2720003	104.8399963
-6.0377483	9.3207998	102.4700012
-6.0377483	9.3696003	103.6100006
-6.0377483	9.4184008	103.5800018
-6.0377483	9.4672003	102.6800003
-6.0377483	9.5160007	100.5499954
-6.0377483	9.5648003	105.2200012
-6.0377483	9.6136007	103.6699982
-6.0377483	9.6624002	103.9099960
-6.0377483	9.7112007	104.8199997
-6.0377483	9.7600002	103.5800018
-6.0377483	9.8088007	103.4899979
-6.0377483	9.8576002	103.1100006
-6.0377483	9.9064007	102.7200012
-6.0377483	9.9552002	102.4599991
-6.0377483	10.0040007	102.3799973
-6.0377483	10.0528002	102.6199951
-6.0377483	10.1016006	105.3399963
-6.0377483	10.1504002	105.9799957
-6.0377483	10.1992006	106.2399979
-6.0377483	10.2480001	107.0499954
-6.0377483	10.2968006	105.2299957
-6.0377483	10.3456001	103.3699951
-6.0377483	10.3944006	105.7699966
-6.0377483	10.4432001	105.6299973
-6.0377483	10.4920006	103.5800018
-6.0377483	10.5408001	102.1299973
-6.0377483	10.5896006	102.8099976
-6.0377483	10.6384001	102.9799957
-6.0377483	10.6872005	105.5400009
-6.0377483	10.7360001	103.1699982
-6.0377483	10.7848005	102.9300003
-6.0377483	10.8336000	104.6399994

-6.0377483	10.8824005	103.7799988
-6.0377483	10.9312000	102.2799988
-6.0377483	10.9800005	105.9899979
-6.0377483	11.0288000	107.2500000
-6.0377483	11.0776005	105.0699997
-6.0377483	11.1264000	99.8199997
-6.0377483	11.1752005	101.7600021
-6.0377483	11.2240000	102.5800018
-6.0377483	11.2728004	101.2299957
-6.0377483	11.3216000	96.6599960
-6.0377483	11.3704004	101.3300018
-6.0377483	11.4191999	104.4499969
-6.0377483	11.4680004	103.9000015
-6.0377483	11.5167999	102.1899948
-6.0377483	11.5656004	103.8300018
-6.0377483	11.6143999	102.9399948
-6.0377483	11.6632004	104.1299973
-6.0377483	11.7119999	103.8099976
-6.0377483	11.7608004	102.6999969
-6.0377483	11.8095999	105.3399963
-6.0377483	11.8584003	105.2799988
-6.0377483	11.9071999	105.3600006
-6.0377483	11.9560003	106.3600006
-6.0377483	12.0047998	105.3199997
-6.0377483	12.0536003	101.2699966
-6.0377483	12.1023998	102.0400009
-6.0377483	12.1512003	103.6699982
-6.0377483	12.1999998	103.5899963
-6.0377483	12.2488003	103.3799973
-6.0377483	12.2975998	103.0999985
-6.0377483	12.3464003	102.0400009
-6.0377483	12.3951998	100.6699982
-6.0377483	12.4440002	101.7900009
-6.0377483	12.4927998	103.6599960
-6.0377483	12.5416002	105.0899963
-6.0377483	12.5904007	104.7999954
-6.0377483	12.6392002	105.9000015
-6.0377483	12.6880007	105.2699966
-6.0377483	12.7368002	104.9099960
-6.0377483	12.7856007	105.7200012
-6.0377483	12.8344002	104.0699997
-6.0377483	12.8832006	105.3899994
-6.0377483	12.9320002	104.4799957
-6.0377483	12.9808006	104.9199982
-6.0377483	13.0296001	105.3099976
-6.0377483	13.0784006	106.6299973
-6.0377483	13.1272001	104.6800003
-6.0377483	13.1760006	104.6800003
-6.0377483	13.2248001	104.2699966
-6.0377483	13.2736006	106.9000015
-6.0377483	13.3224001	106.8199997
-6.0377483	13.3712006	104.9300003
-6.0377483	13.4200001	104.8499985
-6.0377483	13.4688005	103.8799973
-6.0377483	13.5176001	104.5199966
-6.0377483	13.5664005	104.0699997
-6.0377483	13.6152000	99.7900009
-6.0377483	13.6640005	103.3799973

-6.0377483	13.7128000	102.7399979
-6.0377483	13.7616005	99.7900009
-6.0377483	13.8104000	102.0100021
-6.0377483	13.8592005	103.8600006
-6.0377483	13.9080000	105.6899948
-6.0377483	13.9568005	106.6399994
-6.0377483	14.0056000	102.1899948
-6.0377483	14.0544004	100.3499985
-6.0377483	14.1032000	102.4700012
-6.0377483	14.1520004	104.4599991
-6.0377483	14.2007999	102.1999969
-6.0377483	14.2496004	103.2099991
-6.0377483	14.2983999	102.2399979
-6.0377483	14.3472004	102.0800018
-6.0377483	14.3959999	99.5199966
-6.0377483	14.4448004	98.6399994
-6.0377483	14.4935999	102.3600006
-6.0377483	14.5424004	104.7399979
-6.0377483	14.5911999	102.8799973
-6.0377483	14.6400003	104.3099976
-6.0377483	14.6887999	106.8300018
-6.0377483	14.7376003	105.9000015
-6.0377483	14.7863998	101.7999954
-6.0377483	14.8352003	98.6199951
-6.0377483	14.8839998	103.6100006
-6.0377483	14.9328003	105.1699982
-6.0377483	14.9815998	103.8300018
-6.0377483	15.0304003	102.4499969
-6.0316124	0.3416000	109.1699982
-6.0316124	0.3904000	111.2299957
-6.0316124	0.4392000	109.1399994
-6.0316124	0.4880000	107.0199966
-6.0316124	0.5368000	107.1399994
-6.0316124	0.5856000	106.0800018
-6.0316124	0.6344000	106.4300003
-6.0316124	0.6832000	107.9899979
-6.0316124	0.7320000	105.6800003
-6.0316124	0.7808000	108.7900009
-6.0316124	0.8296000	108.2200012
-6.0316124	0.8784000	109.5100021
-6.0316124	0.9272000	109.3399963
-6.0316124	0.9760000	108.3600006
-6.0316124	1.0247999	107.4599991
-6.0316124	1.0735999	105.6100006
-6.0316124	1.1223999	104.0299988
-6.0316124	1.1711999	104.1800003
-6.0316124	1.2200000	103.2699966
-6.0316124	1.2688000	104.9499969
-6.0316124	1.3176000	107.8899994
-6.0316124	1.3664000	108.3199997
-6.0316124	1.4152000	101.2799988
-6.0316124	1.4640000	104.4099960
-6.0316124	1.5128000	106.4399948
-6.0316124	1.5616000	106.0999985
-6.0316124	1.6104000	105.3399963
-6.0316124	1.6592000	109.3199997
-6.0316124	1.7079999	110.4799957
-6.0316124	1.7567999	108.7399979

-6.0316124	1.8055999	108.4899979
-6.0316124	1.8543999	112.8399963
-6.0316124	1.9031999	114.6699982
-6.0316124	1.9519999	112.1599960
-6.0316124	2.0007999	106.0000000
-6.0316124	2.0495999	103.2200012
-6.0316124	2.0983999	105.6399994
-6.0316124	2.1471999	103.2999954
-6.0316124	2.1959999	105.9099960
-6.0316124	2.2447999	100.9799957
-6.0316124	2.2935998	107.5899963
-6.0316124	2.3423998	105.6800003
-6.0316124	2.3912001	106.5999985
-6.0316124	2.4400001	105.1199951
-6.0316124	2.4888000	105.5499954
-6.0316124	2.5376000	105.2200012
-6.0316124	2.5864000	106.9499969
-6.0316124	2.6352000	104.6500015
-6.0316124	2.6840000	101.0400009
-6.0316124	2.7328000	107.1800003
-6.0316124	2.7816000	106.5699997
-6.0316124	2.8304000	101.9399948
-6.0316124	2.8792000	98.2099991
-6.0316124	2.9280000	104.2099991
-6.0316124	2.9768000	102.4599991
-6.0316124	3.0256000	104.6800003
-6.0316124	3.0743999	102.4799957
-6.0316124	3.1231999	101.9799957
-6.0316124	3.1719999	106.5899963
-6.0316124	3.2207999	105.9799957
-6.0316124	3.2695999	105.8799973
-6.0316124	3.3183999	106.3799973
-6.0316124	3.3671999	105.1100006
-6.0316124	3.4159999	103.7399979
-6.0316124	3.4647999	103.0499954
-6.0316124	3.5135999	103.3699951
-6.0316124	3.5623999	102.7399979
-6.0316124	3.6111999	103.1399994
-6.0316124	3.6599998	106.6399994
-6.0316124	3.7087998	104.3899994
-6.0316124	3.7575998	104.4899979
-6.0316124	3.8063998	104.2200012
-6.0316124	3.8551998	105.7399979
-6.0316124	3.9039998	106.0499954
-6.0316124	3.9527998	106.2399979
-6.0316124	4.0016003	106.7699966
-6.0316124	4.0503998	107.7799988
-6.0316124	4.0991998	107.5400009
-6.0316124	4.1479998	102.8600006
-6.0316124	4.1967998	101.8600006
-6.0316124	4.2455997	103.3199997
-6.0316124	4.2943997	106.4899979
-6.0316124	4.3431997	110.5899963
-6.0316124	4.3919997	111.1100006
-6.0316124	4.4407997	108.5599976
-6.0316124	4.4895997	110.4599991
-6.0316124	4.5383997	115.0000000
-6.0316124	4.5871997	116.5100021

-6.0316124	4.6359997	116.8199997
-6.0316124	4.6847997	117.2799988
-6.0316124	4.7336001	114.7399979
-6.0316124	4.7824001	108.9799957
-6.0316124	4.8312001	109.9599991
-6.0316124	4.8800001	112.0000000
-6.0316124	4.9288001	110.2299957
-6.0316124	4.9776001	106.8399963
-6.0316124	5.0264001	109.6599960
-6.0316124	5.0752001	110.1599960
-6.0316124	5.1240001	107.9700012
-6.0316124	5.1728001	105.9099960
-6.0316124	5.2216001	104.5000000
-6.0316124	5.2704000	107.2299957
-6.0316124	5.3192000	108.9599991
-6.0316124	5.3680000	106.2600021
-6.0316124	5.4168000	102.2399979
-6.0316124	5.4656000	102.2099991
-6.0316124	5.5144000	105.4300003
-6.0316124	5.5632000	107.3699951
-6.0316124	5.6120000	104.2299957
-6.0316124	5.6608000	105.2900009
-6.0316124	5.7096000	106.2799988
-6.0316124	5.7584000	103.2399979
-6.0316124	5.8072000	103.5400009
-6.0316124	5.8559999	101.9799957
-6.0316124	5.9047999	103.9000015
-6.0316124	5.9535999	104.7999954
-6.0316124	6.0023999	101.8499985
-6.0316124	6.0511999	102.9199982
-6.0316124	6.0999999	104.4199982
-6.0316124	6.1487999	104.4700012
-6.0316124	6.1975999	99.9899979
-6.0316124	6.2463999	104.1699982
-6.0316124	6.2951999	105.1899948
-6.0316124	6.3439999	101.8899994
-6.0316124	6.3927999	103.7900009
-6.0316124	6.4415998	108.2500000
-6.0316124	6.4903998	109.3699951
-6.0316124	6.5391998	106.9700012
-6.0316124	6.5879998	104.6599960
-6.0316124	6.6367998	104.7900009
-6.0316124	6.6855998	104.2900009
-6.0316124	6.7343998	102.0299988
-6.0316124	6.7831998	101.4099960
-6.0316124	6.8319998	100.8600006
-6.0316124	6.8807998	106.0599976
-6.0316124	6.9295998	103.8699951
-6.0316124	6.9783998	104.2600021
-6.0316124	7.0271997	106.6199951
-6.0316124	7.0759997	104.9399948
-6.0316124	7.1247997	105.5400009
-6.0316124	7.1735997	101.0100021
-6.0316124	7.2223997	103.8099976
-6.0316124	7.2711997	103.4499969
-6.0316124	7.3199997	98.7200012
-6.0316124	7.3687997	101.4199982
-6.0316124	7.4175997	102.2799988

-6.0316124	7.4663997	101.8899994
-6.0316124	7.5151997	104.2399979
-6.0316124	7.5639997	103.4300003
-6.0316124	7.6127996	102.9700012
-6.0316124	7.6615996	103.6999969
-6.0316124	7.7103996	104.9399948
-6.0316124	7.7591996	102.7600021
-6.0316124	7.8079996	103.2999954
-6.0316124	7.8567996	102.5599976
-6.0316124	7.9056001	101.5599976
-6.0316124	7.9544001	103.3899994
-6.0316124	8.0032005	103.1899948
-6.0316124	8.0520000	103.7699966
-6.0316124	8.1008005	106.0199966
-6.0316124	8.1496000	105.1800003
-6.0316124	8.1984005	105.4199982
-6.0316124	8.2472000	106.1599960
-6.0316124	8.2960005	105.2399979
-6.0316124	8.3448000	102.3499985
-6.0316124	8.3936005	101.3300018
-6.0316124	8.4424000	101.2399979
-6.0316124	8.4912004	101.2399979
-6.0316124	8.5400000	105.9000015
-6.0316124	8.5888004	103.6599960
-6.0316124	8.6375999	103.6599960
-6.0316124	8.6864004	105.7999954
-6.0316124	8.7351999	105.0000000
-6.0316124	8.7840004	102.9899979
-6.0316124	8.8327999	101.0000000
-6.0316124	8.8816004	102.0000000
-6.0316124	8.9303999	104.1599960
-6.0316124	8.9792004	104.6299973
-6.0316124	9.0279999	104.4499969
-6.0316124	9.0768003	104.0899963
-6.0316124	9.1255999	106.0599976
-6.0316124	9.1744003	106.2999954
-6.0316124	9.2231998	106.7799988
-6.0316124	9.2720003	105.4599991
-6.0316124	9.3207998	103.8099976
-6.0316124	9.3696003	103.7699966
-6.0316124	9.4184008	103.3399963
-6.0316124	9.4672003	102.2600021
-6.0316124	9.5160007	101.3499985
-6.0316124	9.5648003	104.2200012
-6.0316124	9.6136007	103.8699951
-6.0316124	9.6624002	103.5800018
-6.0316124	9.7112007	103.7299957
-6.0316124	9.7600002	103.1899948
-6.0316124	9.8088007	103.7699966
-6.0316124	9.8576002	103.0599976
-6.0316124	9.9064007	101.7299957
-6.0316124	9.9552002	101.9000015
-6.0316124	10.0040007	102.1100006
-6.0316124	10.0528002	102.1599960
-6.0316124	10.1016006	104.7200012
-6.0316124	10.1504002	106.0499954
-6.0316124	10.1992006	105.8399963
-6.0316124	10.2480001	106.6199951

-6.0316124	10.2968006	105.3499985
-6.0316124	10.3456001	104.5599976
-6.0316124	10.3944006	106.2699966
-6.0316124	10.4432001	105.7600021
-6.0316124	10.4920006	103.5899963
-6.0316124	10.5408001	102.8499985
-6.0316124	10.5896006	102.2299957
-6.0316124	10.6384001	102.5699997
-6.0316124	10.6872005	106.3499985
-6.0316124	10.7360001	103.6199951
-6.0316124	10.7848005	103.3399963
-6.0316124	10.8336000	104.1599960
-6.0316124	10.8824005	103.2799988
-6.0316124	10.9312000	102.9300003
-6.0316124	10.9800005	105.7600021
-6.0316124	11.0288000	106.5499954
-6.0316124	11.0776005	104.4300003
-6.0316124	11.1264000	99.4099960
-6.0316124	11.1752005	101.5000000
-6.0316124	11.2240000	102.5899963
-6.0316124	11.2728004	101.6100006
-6.0316124	11.3216000	96.2200012
-6.0316124	11.3704004	101.7799988
-6.0316124	11.4191999	104.9199982
-6.0316124	11.4680004	104.1399994
-6.0316124	11.5167999	102.8799973
-6.0316124	11.5656004	104.2099991
-6.0316124	11.6143999	102.5100021
-6.0316124	11.6632004	103.3600006
-6.0316124	11.7119999	104.0299988
-6.0316124	11.7608004	103.8399963
-6.0316124	11.8095999	106.1699982
-6.0316124	11.8584003	104.8399963
-6.0316124	11.9071999	105.2799988
-6.0316124	11.9560003	106.8799973
-6.0316124	12.0047998	105.7900009
-6.0316124	12.0536003	101.6100006
-6.0316124	12.1023998	102.1699982
-6.0316124	12.1512003	103.9499969
-6.0316124	12.1999998	103.4899979
-6.0316124	12.2488003	102.7399979
-6.0316124	12.2975998	102.6999969
-6.0316124	12.3464003	101.7699966
-6.0316124	12.3951998	99.8399963
-6.0316124	12.4440002	101.0699997
-6.0316124	12.4927998	103.0699997
-6.0316124	12.5416002	104.7900009
-6.0316124	12.5904007	104.3099976
-6.0316124	12.6392002	105.5599976
-6.0316124	12.6880007	105.5499954
-6.0316124	12.7368002	105.4899979
-6.0316124	12.7856007	105.7799988
-6.0316124	12.8344002	105.3099976
-6.0316124	12.8832006	105.4300003
-6.0316124	12.9320002	104.2999954
-6.0316124	12.9808006	104.5100021
-6.0316124	13.0296001	104.6399994
-6.0316124	13.0784006	107.1399994

-6.0316124	13.1272001	105.3600006
-6.0316124	13.1760006	104.1800003
-6.0316124	13.2248001	102.9099960
-6.0316124	13.2736006	105.7200012
-6.0316124	13.3224001	106.3699951
-6.0316124	13.3712006	105.0899963
-6.0316124	13.4200001	104.8699951
-6.0316124	13.4688005	103.4099960
-6.0316124	13.5176001	104.4700012
-6.0316124	13.5664005	104.3699951
-6.0316124	13.6152000	102.0999985
-6.0316124	13.6640005	104.2200012
-6.0316124	13.7128000	103.0999985
-6.0316124	13.7616005	100.2799988
-6.0316124	13.8104000	102.6899948
-6.0316124	13.8592005	104.2299957
-6.0316124	13.9080000	105.3300018
-6.0316124	13.9568005	105.3300018
-6.0316124	14.0056000	100.1999969
-6.0316124	14.0544004	100.1100006
-6.0316124	14.1032000	103.1100006
-6.0316124	14.1520004	104.5499954
-6.0316124	14.2007999	101.4700012
-6.0316124	14.2496004	102.3799973
-6.0316124	14.2983999	102.6399994
-6.0316124	14.3472004	102.8799973
-6.0316124	14.3959999	99.6100006
-6.0316124	14.4448004	99.1699982
-6.0316124	14.4935999	103.1899948
-6.0316124	14.5424004	105.3099976
-6.0316124	14.5911999	103.1199951
-6.0316124	14.6400003	103.9799957
-6.0316124	14.6887999	106.0100021
-6.0316124	14.7376003	105.4399948
-6.0316124	14.7863998	101.8899994
-6.0316124	14.8352003	99.0199966
-6.0316124	14.8839998	104.2699966
-6.0316124	14.9328003	105.4899979
-6.0316124	14.9815998	104.2299957
-6.0316124	15.0304003	102.2999954
-6.0254765	0.3416000	108.6699982
-6.0254765	0.3904000	110.6199951
-6.0254765	0.4392000	108.4499969
-6.0254765	0.4880000	106.5999985
-6.0254765	0.5368000	106.8600006
-6.0254765	0.5856000	106.3699951
-6.0254765	0.6344000	106.6999969
-6.0254765	0.6832000	108.0999985
-6.0254765	0.7320000	105.9399948
-6.0254765	0.7808000	108.5499954
-6.0254765	0.8296000	108.7699966
-6.0254765	0.8784000	109.2999954
-6.0254765	0.9272000	109.1299973
-6.0254765	0.9760000	108.4899979
-6.0254765	1.0247999	107.3199997
-6.0254765	1.0735999	106.2099991
-6.0254765	1.1223999	105.3899994
-6.0254765	1.1711999	106.0999985

-6.0254765	1.2200000	104.0999985
-6.0254765	1.2688000	105.8399963
-6.0254765	1.3176000	107.9799957
-6.0254765	1.3664000	108.9799957
-6.0254765	1.4152000	101.4099960
-6.0254765	1.4640000	104.7600021
-6.0254765	1.5128000	107.0499954
-6.0254765	1.5616000	106.7799988
-6.0254765	1.6104000	105.4899979
-6.0254765	1.6592000	109.2699966
-6.0254765	1.7079999	110.6399994
-6.0254765	1.7567999	108.5699997
-6.0254765	1.8055999	108.3499985
-6.0254765	1.8543999	112.0499954
-6.0254765	1.9031999	113.8799973
-6.0254765	1.9519999	112.0999985
-6.0254765	2.0007999	105.5299988
-6.0254765	2.0495999	102.1500015
-6.0254765	2.0983999	105.2399979
-6.0254765	2.1471999	103.4899979
-6.0254765	2.1959999	105.5800018
-6.0254765	2.2447999	101.2099991
-6.0254765	2.2935998	107.9799957
-6.0254765	2.3423998	106.4599991
-6.0254765	2.3912001	106.1399994
-6.0254765	2.4400001	103.9300003
-6.0254765	2.4888000	105.2099991
-6.0254765	2.5376000	105.1800003
-6.0254765	2.5864000	106.9000015
-6.0254765	2.6352000	104.4300003
-6.0254765	2.6840000	101.7399979
-6.0254765	2.7328000	106.6399994
-6.0254765	2.7816000	105.6599960
-6.0254765	2.8304000	100.9899979
-6.0254765	2.8792000	97.4099960
-6.0254765	2.9280000	103.3799973
-6.0254765	2.9768000	101.9700012
-6.0254765	3.0256000	104.4799957
-6.0254765	3.0743999	101.5499954
-6.0254765	3.1231999	99.9399948
-6.0254765	3.1719999	105.8199997
-6.0254765	3.2207999	104.9300003
-6.0254765	3.2695999	105.1399994
-6.0254765	3.3183999	106.1399994
-6.0254765	3.3671999	104.9099960
-6.0254765	3.4159999	103.8099976
-6.0254765	3.4647999	103.6899948
-6.0254765	3.5135999	103.6199951
-6.0254765	3.5623999	103.1299973
-6.0254765	3.6111999	102.9700012
-6.0254765	3.6599998	107.2399979
-6.0254765	3.7087998	105.4700012
-6.0254765	3.7575998	104.3600006
-6.0254765	3.8063998	104.3199997
-6.0254765	3.8551998	106.1100006
-6.0254765	3.9039998	105.8600006
-6.0254765	3.9527998	105.8399963
-6.0254765	4.0016003	105.9700012

-6.0254765	4.0503998	106.7900009
-6.0254765	4.0991998	106.7500000
-6.0254765	4.1479998	103.4199982
-6.0254765	4.1967998	101.4000015
-6.0254765	4.2455997	102.5899963
-6.0254765	4.2943997	106.2699966
-6.0254765	4.3431997	110.2399979
-6.0254765	4.3919997	109.6500015
-6.0254765	4.4407997	106.9300003
-6.0254765	4.4895997	109.4000015
-6.0254765	4.5383997	114.2999954
-6.0254765	4.5871997	115.3600006
-6.0254765	4.6359997	116.8099976
-6.0254765	4.6847997	117.5699997
-6.0254765	4.7336001	115.2299957
-6.0254765	4.7824001	110.0999985
-6.0254765	4.8312001	110.7200012
-6.0254765	4.8800001	111.1899948
-6.0254765	4.9288001	109.0699997
-6.0254765	4.9776001	105.9199982
-6.0254765	5.0264001	109.1599960
-6.0254765	5.0752001	109.4300003
-6.0254765	5.1240001	107.0299988
-6.0254765	5.1728001	104.7600021
-6.0254765	5.2216001	102.9199982
-6.0254765	5.2704000	106.8899994
-6.0254765	5.3192000	108.0400009
-6.0254765	5.3680000	105.1399994
-6.0254765	5.4168000	102.3399963
-6.0254765	5.4656000	102.7500000
-6.0254765	5.5144000	106.2299957
-6.0254765	5.5632000	107.5999985
-6.0254765	5.6120000	104.3899994
-6.0254765	5.6608000	106.0499954
-6.0254765	5.7096000	106.5199966
-6.0254765	5.7584000	103.6599960
-6.0254765	5.8072000	103.1699982
-6.0254765	5.8559999	102.1500015
-6.0254765	5.9047999	104.4799957
-6.0254765	5.9535999	105.6500015
-6.0254765	6.0023999	102.9700012
-6.0254765	6.0511999	103.9799957
-6.0254765	6.0999999	104.9799957
-6.0254765	6.1487999	104.6999969
-6.0254765	6.1975999	100.5199966
-6.0254765	6.2463999	104.0100021
-6.0254765	6.2951999	104.4399948
-6.0254765	6.3439999	101.8199997
-6.0254765	6.3927999	102.9799957
-6.0254765	6.4415998	107.6399994
-6.0254765	6.4903998	109.0400009
-6.0254765	6.5391998	107.0100021
-6.0254765	6.5879998	103.8499985
-6.0254765	6.6367998	104.3300018
-6.0254765	6.6855998	103.7699966
-6.0254765	6.7343998	101.6299973
-6.0254765	6.7831998	101.5899963
-6.0254765	6.8319998	99.3199997

-6.0254765	6.8807998	106.3699951
-6.0254765	6.9295998	105.3300018
-6.0254765	6.9783998	105.2699966
-6.0254765	7.0271997	106.3300018
-6.0254765	7.0759997	103.5499954
-6.0254765	7.1247997	104.4099960
-6.0254765	7.1735997	100.6500015
-6.0254765	7.2223997	103.1800003
-6.0254765	7.2711997	103.2699966
-6.0254765	7.3199997	97.1999969
-6.0254765	7.3687997	101.9000015
-6.0254765	7.4175997	101.1599960
-6.0254765	7.4663997	101.2900009
-6.0254765	7.5151997	104.5400009
-6.0254765	7.5639997	102.9799957
-6.0254765	7.6127996	103.1299973
-6.0254765	7.6615996	103.8799973
-6.0254765	7.7103996	105.2999954
-6.0254765	7.7591996	103.9199982
-6.0254765	7.8079996	103.4000015
-6.0254765	7.8567996	103.0100021
-6.0254765	7.9056001	102.7999954
-6.0254765	7.9544001	104.5800018
-6.0254765	8.0032005	102.9899979
-6.0254765	8.0520000	103.1199951
-6.0254765	8.1008005	106.6399994
-6.0254765	8.1496000	105.1899948
-6.0254765	8.1984005	104.8699951
-6.0254765	8.2472000	105.2299957
-6.0254765	8.2960005	103.5999985
-6.0254765	8.3448000	101.5599976
-6.0254765	8.3936005	101.6399994
-6.0254765	8.4424000	101.0599976
-6.0254765	8.4912004	102.1999969
-6.0254765	8.5400000	105.8499985
-6.0254765	8.5888004	103.7299957
-6.0254765	8.6375999	103.1599960
-6.0254765	8.6864004	104.5199966
-6.0254765	8.7351999	103.7799988
-6.0254765	8.7840004	102.8399963
-6.0254765	8.8327999	101.3699951
-6.0254765	8.8816004	102.4000015
-6.0254765	8.9303999	104.8399963
-6.0254765	8.9792004	105.3199997
-6.0254765	9.0279999	105.4700012
-6.0254765	9.0768003	104.9199982
-6.0254765	9.1255999	105.8399963
-6.0254765	9.1744003	106.5199966
-6.0254765	9.2231998	106.7200012
-6.0254765	9.2720003	105.8600006
-6.0254765	9.3207998	104.9000015
-6.0254765	9.3696003	103.7099991
-6.0254765	9.4184008	103.5000000
-6.0254765	9.4672003	101.9199982
-6.0254765	9.5160007	101.9399948
-6.0254765	9.5648003	102.9099960
-6.0254765	9.6136007	103.2900009
-6.0254765	9.6624002	103.1500015

-6.0254765	9.7112007	102.8499985
-6.0254765	9.7600002	103.3399963
-6.0254765	9.8088007	103.9399948
-6.0254765	9.8576002	103.1100006
-6.0254765	9.9064007	100.7099991
-6.0254765	9.9552002	101.2900009
-6.0254765	10.0040007	102.2799988
-6.0254765	10.0528002	101.7399979
-6.0254765	10.1016006	104.0499954
-6.0254765	10.1504002	105.5400009
-6.0254765	10.1992006	104.6800003
-6.0254765	10.2480001	106.2699966
-6.0254765	10.2968006	105.6299973
-6.0254765	10.3456001	105.5699997
-6.0254765	10.3944006	106.2299957
-6.0254765	10.4432001	105.2600021
-6.0254765	10.4920006	103.3600006
-6.0254765	10.5408001	103.0999985
-6.0254765	10.5896006	101.3199997
-6.0254765	10.6384001	102.1500015
-6.0254765	10.6872005	106.3899994
-6.0254765	10.7360001	103.6899948
-6.0254765	10.7848005	103.7299957
-6.0254765	10.8336000	103.8699951
-6.0254765	10.8824005	103.0400009
-6.0254765	10.9312000	103.2399979
-6.0254765	10.9800005	103.9899979
-6.0254765	11.0288000	105.0999985
-6.0254765	11.0776005	103.9199982
-6.0254765	11.1264000	99.6199951
-6.0254765	11.1752005	101.3499985
-6.0254765	11.2240000	102.3399963
-6.0254765	11.2728004	101.6399994
-6.0254765	11.3216000	95.6999969
-6.0254765	11.3704004	102.1299973
-6.0254765	11.4191999	105.2699966
-6.0254765	11.4680004	104.6199951
-6.0254765	11.5167999	103.8499985
-6.0254765	11.5656004	104.6299973
-6.0254765	11.6143999	102.8099976
-6.0254765	11.6632004	102.6199951
-6.0254765	11.7119999	104.0699997
-6.0254765	11.7608004	104.6399994
-6.0254765	11.8095999	107.0100021
-6.0254765	11.8584003	105.1800003
-6.0254765	11.9071999	105.4099960
-6.0254765	11.9560003	107.0199966
-6.0254765	12.0047998	106.1800003
-6.0254765	12.0536003	102.6599960
-6.0254765	12.1023998	103.1399994
-6.0254765	12.1512003	104.5699997
-6.0254765	12.1999998	103.5999985
-6.0254765	12.2488003	101.9399948
-6.0254765	12.2975998	102.1599960
-6.0254765	12.3464003	101.4099960
-6.0254765	12.3951998	98.6599960
-6.0254765	12.4440002	99.9000015
-6.0254765	12.4927998	102.8199997

-6.0254765	12.5416002	104.3699951
-6.0254765	12.5904007	103.5100021
-6.0254765	12.6392002	105.1999969
-6.0254765	12.6880007	105.8499985
-6.0254765	12.7368002	105.9799957
-6.0254765	12.7856007	105.6100006
-6.0254765	12.8344002	106.3399963
-6.0254765	12.8832006	106.3499985
-6.0254765	12.9320002	105.1100006
-6.0254765	12.9808006	104.5499954
-6.0254765	13.0296001	103.9099960
-6.0254765	13.0784006	107.3099976
-6.0254765	13.1272001	105.4199982
-6.0254765	13.1760006	103.6599960
-6.0254765	13.2248001	101.6999969
-6.0254765	13.2736006	104.6199951
-6.0254765	13.3224001	106.0599976
-6.0254765	13.3712006	104.8399963
-6.0254765	13.4200001	104.0800018
-6.0254765	13.4688005	102.0999985
-6.0254765	13.5176001	103.4399948
-6.0254765	13.5664005	103.9199982
-6.0254765	13.6152000	103.6699982
-6.0254765	13.6640005	104.7299957
-6.0254765	13.7128000	103.5499954
-6.0254765	13.7616005	101.2200012
-6.0254765	13.8104000	103.5899963
-6.0254765	13.8592005	104.5000000
-6.0254765	13.9080000	104.7699966
-6.0254765	13.9568005	104.0499954
-6.0254765	14.0056000	98.9099960
-6.0254765	14.0544004	100.0899963
-6.0254765	14.1032000	103.7200012
-6.0254765	14.1520004	104.5999985
-6.0254765	14.2007999	101.0000000
-6.0254765	14.2496004	101.9499969
-6.0254765	14.2983999	102.5800018
-6.0254765	14.3472004	102.8199997
-6.0254765	14.3959999	99.2900009
-6.0254765	14.4448004	100.3699951
-6.0254765	14.4935999	103.6199951
-6.0254765	14.5424004	105.4499969
-6.0254765	14.5911999	103.5699997
-6.0254765	14.6400003	104.7799988
-6.0254765	14.6887999	106.0299988
-6.0254765	14.7376003	105.0400009
-6.0254765	14.7863998	101.7999954
-6.0254765	14.8352003	99.6899948
-6.0254765	14.8839998	104.8199997
-6.0254765	14.9328003	105.5800018
-6.0254765	14.9815998	104.4300003
-6.0254765	15.0304003	101.9399948
-6.0193410	0.3416000	108.4899979
-6.0193410	0.3904000	109.5800018
-6.0193410	0.4392000	107.9300003
-6.0193410	0.4880000	106.7399979
-6.0193410	0.5368000	106.8600006
-6.0193410	0.5856000	106.1999969

-6.0193410	0.6344000	106.8300018
-6.0193410	0.6832000	108.4099960
-6.0193410	0.7320000	106.3600006
-6.0193410	0.7808000	108.3199997
-6.0193410	0.8296000	109.3300018
-6.0193410	0.8784000	108.9700012
-6.0193410	0.9272000	107.7200012
-6.0193410	0.9760000	107.9700012
-6.0193410	1.0247999	106.9899979
-6.0193410	1.0735999	107.0699997
-6.0193410	1.1223999	106.2799988
-6.0193410	1.1711999	107.0499954
-6.0193410	1.2200000	104.1899948
-6.0193410	1.2688000	106.4099960
-6.0193410	1.3176000	107.6500015
-6.0193410	1.3664000	109.2799988
-6.0193410	1.4152000	102.4799957
-6.0193410	1.4640000	104.9199982
-6.0193410	1.5128000	107.4799957
-6.0193410	1.5616000	107.1899948
-6.0193410	1.6104000	106.0800018
-6.0193410	1.6592000	109.9499969
-6.0193410	1.7079999	111.0000000
-6.0193410	1.7567999	108.6500015
-6.0193410	1.8055999	108.6599960
-6.0193410	1.8543999	111.4599991
-6.0193410	1.9031999	112.6999969
-6.0193410	1.9519999	111.6899948
-6.0193410	2.0007999	104.9000015
-6.0193410	2.0495999	101.5699997
-6.0193410	2.0983999	104.9499969
-6.0193410	2.1471999	103.6699982
-6.0193410	2.1959999	105.0599976
-6.0193410	2.2447999	101.7399979
-6.0193410	2.2935998	107.5100021
-6.0193410	2.3423998	106.6899948
-6.0193410	2.3912001	106.0699997
-6.0193410	2.4400001	102.3799973
-6.0193410	2.4888000	104.5000000
-6.0193410	2.5376000	105.9499969
-6.0193410	2.5864000	107.6500015
-6.0193410	2.6352000	105.3899994
-6.0193410	2.6840000	103.5699997
-6.0193410	2.7328000	106.8499985
-6.0193410	2.7816000	104.8799973
-6.0193410	2.8304000	99.6599960
-6.0193410	2.8792000	97.8099976
-6.0193410	2.9280000	102.8199997
-6.0193410	2.9768000	102.5400009
-6.0193410	3.0256000	104.6999969
-6.0193410	3.0743999	101.2200012
-6.0193410	3.1231999	98.3699951
-6.0193410	3.1719999	105.4599991
-6.0193410	3.2207999	104.1699982
-6.0193410	3.2695999	104.2500000
-6.0193410	3.3183999	105.8799973
-6.0193410	3.3671999	104.7399979
-6.0193410	3.4159999	103.7600021

-6.0193410	3.4647999	103.9399948
-6.0193410	3.5135999	103.3899994
-6.0193410	3.5623999	102.8699951
-6.0193410	3.6111999	102.9300003
-6.0193410	3.6599998	106.7399979
-6.0193410	3.7087998	106.2500000
-6.0193410	3.7575998	104.3899994
-6.0193410	3.8063998	104.3799973
-6.0193410	3.8551998	106.4700012
-6.0193410	3.9039998	105.9099960
-6.0193410	3.9527998	105.8499985
-6.0193410	4.0016003	105.7500000
-6.0193410	4.0503998	105.7999954
-6.0193410	4.0991998	105.7099991
-6.0193410	4.1479998	104.4099960
-6.0193410	4.1967998	102.5299988
-6.0193410	4.2455997	102.5299988
-6.0193410	4.2943997	106.5499954
-6.0193410	4.3431997	110.3899994
-6.0193410	4.3919997	109.1299973
-6.0193410	4.4407997	105.8699951
-6.0193410	4.4895997	109.1299973
-6.0193410	4.5383997	114.4399948
-6.0193410	4.5871997	114.6699982
-6.0193410	4.6359997	116.8499985
-6.0193410	4.6847997	117.5400009
-6.0193410	4.7336001	115.1500015
-6.0193410	4.7824001	110.4300003
-6.0193410	4.8312001	110.8199997
-6.0193410	4.8800001	110.2600021
-6.0193410	4.9288001	108.1399994
-6.0193410	4.9776001	104.9000015
-6.0193410	5.0264001	108.5599976
-6.0193410	5.0752001	108.9700012
-6.0193410	5.1240001	106.6199951
-6.0193410	5.1728001	104.1999969
-6.0193410	5.2216001	102.3499985
-6.0193410	5.2704000	106.5899963
-6.0193410	5.3192000	107.0999985
-6.0193410	5.3680000	104.1199951
-6.0193410	5.4168000	102.3699951
-6.0193410	5.4656000	102.8499985
-6.0193410	5.5144000	106.2399979
-6.0193410	5.5632000	107.5800018
-6.0193410	5.6120000	105.3899994
-6.0193410	5.6608000	107.1100006
-6.0193410	5.7096000	107.2900009
-6.0193410	5.7584000	104.8499985
-6.0193410	5.8072000	103.8600006
-6.0193410	5.8559999	102.4899979
-6.0193410	5.9047999	104.7900009
-6.0193410	5.9535999	106.0400009
-6.0193410	6.0023999	103.6999969
-6.0193410	6.0511999	104.9499969
-6.0193410	6.0999999	105.5699997
-6.0193410	6.1487999	105.1100006
-6.0193410	6.1975999	102.0499954
-6.0193410	6.2463999	103.8799973

-6.0193410	6.2951999	104.1299973
-6.0193410	6.3439999	102.4700012
-6.0193410	6.3927999	102.7699966
-6.0193410	6.4415998	106.6699982
-6.0193410	6.4903998	108.3600006
-6.0193410	6.5391998	106.1199951
-6.0193410	6.5879998	103.0999985
-6.0193410	6.6367998	103.7500000
-6.0193410	6.6855998	103.4199982
-6.0193410	6.7343998	101.2399979
-6.0193410	6.7831998	101.7999954
-6.0193410	6.8319998	99.3399963
-6.0193410	6.8807998	105.9499969
-6.0193410	6.9295998	106.2799988
-6.0193410	6.9783998	106.1399994
-6.0193410	7.0271997	105.9499969
-6.0193410	7.0759997	103.0599976
-6.0193410	7.1247997	103.2099991
-6.0193410	7.1735997	100.3899994
-6.0193410	7.2223997	102.5499954
-6.0193410	7.2711997	103.0400009
-6.0193410	7.3199997	96.4899979
-6.0193410	7.3687997	102.4300003
-6.0193410	7.4175997	100.2099991
-6.0193410	7.4663997	100.2099991
-6.0193410	7.5151997	104.4700012
-6.0193410	7.5639997	103.6699982
-6.0193410	7.6127996	103.6999969
-6.0193410	7.6615996	104.3199997
-6.0193410	7.7103996	106.0899963
-6.0193410	7.7591996	105.4899979
-6.0193410	7.8079996	103.6899948
-6.0193410	7.8567996	103.7399979
-6.0193410	7.9056001	103.9099960
-6.0193410	7.9544001	104.6599960
-6.0193410	8.0032005	102.3899994
-6.0193410	8.0520000	102.2699966
-6.0193410	8.1008005	106.7299957
-6.0193410	8.1496000	104.7399979
-6.0193410	8.1984005	103.7399979
-6.0193410	8.2472000	104.2600021
-6.0193410	8.2960005	101.9300003
-6.0193410	8.3448000	101.9000015
-6.0193410	8.3936005	102.2799988
-6.0193410	8.4424000	101.0699997
-6.0193410	8.4912004	102.7799988
-6.0193410	8.5400000	105.8899994
-6.0193410	8.5888004	104.0599976
-6.0193410	8.6375999	102.6899948
-6.0193410	8.6864004	103.1399994
-6.0193410	8.7351999	102.2799988
-6.0193410	8.7840004	102.1500015
-6.0193410	8.8327999	100.8499985
-6.0193410	8.8816004	102.4099960
-6.0193410	8.9303999	105.2799988
-6.0193410	8.9792004	105.8300018
-6.0193410	9.0279999	106.5000000
-6.0193410	9.0768003	105.7699966

-6.0193410	9.1255999	105.5400009
-6.0193410	9.1744003	106.7399979
-6.0193410	9.2231998	106.5199966
-6.0193410	9.2720003	106.0100021
-6.0193410	9.3207998	105.4199982
-6.0193410	9.3696003	103.4499969
-6.0193410	9.4184008	103.8199997
-6.0193410	9.4672003	102.0800018
-6.0193410	9.5160007	103.0800018
-6.0193410	9.5648003	101.9300003
-6.0193410	9.6136007	103.2699966
-6.0193410	9.6624002	102.7799988
-6.0193410	9.7112007	101.3099976
-6.0193410	9.7600002	103.5800018
-6.0193410	9.8088007	104.2399979
-6.0193410	9.8576002	103.4399948
-6.0193410	9.9064007	100.0199966
-6.0193410	9.9552002	100.0699997
-6.0193410	10.0040007	101.9499969
-6.0193410	10.0528002	100.8099976
-6.0193410	10.1016006	103.1699982
-6.0193410	10.1504002	104.7900009
-6.0193410	10.1992006	103.1500015
-6.0193410	10.2480001	105.6599960
-6.0193410	10.2968006	105.6800003
-6.0193410	10.3456001	106.2200012
-6.0193410	10.3944006	105.2699966
-6.0193410	10.4432001	104.3499985
-6.0193410	10.4920006	103.1599960
-6.0193410	10.5408001	103.1599960
-6.0193410	10.5896006	100.8099976
-6.0193410	10.6384001	102.7299957
-6.0193410	10.6872005	106.1599960
-6.0193410	10.7360001	103.7500000
-6.0193410	10.7848005	103.5199966
-6.0193410	10.8336000	103.4000015
-6.0193410	10.8824005	102.6599960
-6.0193410	10.9312000	103.4499969
-6.0193410	10.9800005	102.3399963
-6.0193410	11.0288000	103.8300018
-6.0193410	11.0776005	103.9199982
-6.0193410	11.1264000	100.5599976
-6.0193410	11.1752005	101.5999985
-6.0193410	11.2240000	102.4099960
-6.0193410	11.2728004	102.0599976
-6.0193410	11.3216000	96.0100021
-6.0193410	11.3704004	102.6299973
-6.0193410	11.4191999	105.6999969
-6.0193410	11.4680004	105.0400009
-6.0193410	11.5167999	104.4700012
-6.0193410	11.5656004	104.7500000
-6.0193410	11.6143999	102.8300018
-6.0193410	11.6632004	100.6399994
-6.0193410	11.7119999	103.6899948
-6.0193410	11.7608004	104.9700012
-6.0193410	11.8095999	107.0199966
-6.0193410	11.8584003	105.3399963
-6.0193410	11.9071999	105.6399994

-6.0193410	11.9560003	107.1599960
-6.0193410	12.0047998	106.5999985
-6.0193410	12.0536003	103.9700012
-6.0193410	12.1023998	104.4499969
-6.0193410	12.1512003	105.0599976
-6.0193410	12.1999998	103.5299988
-6.0193410	12.2488003	102.0000000
-6.0193410	12.2975998	102.0899963
-6.0193410	12.3464003	101.4000015
-6.0193410	12.3951998	98.2200012
-6.0193410	12.4440002	99.1899948
-6.0193410	12.4927998	102.6299973
-6.0193410	12.5416002	103.6599960
-6.0193410	12.5904007	102.1699982
-6.0193410	12.6392002	104.1699982
-6.0193410	12.6880007	105.2999954
-6.0193410	12.7368002	105.6899948
-6.0193410	12.7856007	104.7799988
-6.0193410	12.8344002	106.4199982
-6.0193410	12.8832006	106.7299957
-6.0193410	12.9320002	105.5100021
-6.0193410	12.9808006	103.9199982
-6.0193410	13.0296001	102.3099976
-6.0193410	13.0784006	106.5199966
-6.0193410	13.1272001	105.3899994
-6.0193410	13.1760006	102.3499985
-6.0193410	13.2248001	99.3300018
-6.0193410	13.2736006	102.9499969
-6.0193410	13.3224001	105.4000015
-6.0193410	13.3712006	104.4399948
-6.0193410	13.4200001	103.2500000
-6.0193410	13.4688005	100.7699966
-6.0193410	13.5176001	102.6399994
-6.0193410	13.5664005	103.4000015
-6.0193410	13.6152000	104.2699966
-6.0193410	13.6640005	104.5899963
-6.0193410	13.7128000	102.8600006
-6.0193410	13.7616005	101.1500015
-6.0193410	13.8104000	103.8099976
-6.0193410	13.8592005	104.5400009
-6.0193410	13.9080000	104.2399979
-6.0193410	13.9568005	103.1299973
-6.0193410	14.0056000	97.9099960
-6.0193410	14.0544004	100.1699982
-6.0193410	14.1032000	104.0499954
-6.0193410	14.1520004	104.4899979
-6.0193410	14.2007999	100.4000015
-6.0193410	14.2496004	101.6299973
-6.0193410	14.2983999	102.7900009
-6.0193410	14.3472004	103.5599976
-6.0193410	14.3959999	99.2799988
-6.0193410	14.4448004	101.4199982
-6.0193410	14.4935999	103.5499954
-6.0193410	14.5424004	105.5800018
-6.0193410	14.5911999	104.3300018
-6.0193410	14.6400003	105.3199997
-6.0193410	14.6887999	105.2299957
-6.0193410	14.7376003	104.0499954

-6.0193410	14.7863998	101.3099976
-6.0193410	14.8352003	100.5000000
-6.0193410	14.8839998	104.4799957
-6.0193410	14.9328003	105.0499954
-6.0193410	14.9815998	104.5400009
-6.0193410	15.0304003	101.4199982
-6.0132051	0.3416000	108.3499985
-6.0132051	0.3904000	108.8300018
-6.0132051	0.4392000	107.6199951
-6.0132051	0.4880000	105.7099991
-6.0132051	0.5368000	107.0100021
-6.0132051	0.5856000	106.3699951
-6.0132051	0.6344000	106.8099976
-6.0132051	0.6832000	107.9399948
-6.0132051	0.7320000	105.8099976
-6.0132051	0.7808000	108.3899994
-6.0132051	0.8296000	109.9700012
-6.0132051	0.8784000	108.7699966
-6.0132051	0.9272000	106.4799957
-6.0132051	0.9760000	107.7399979
-6.0132051	1.0247999	106.7200012
-6.0132051	1.0735999	107.0499954
-6.0132051	1.1223999	106.6800003
-6.0132051	1.1711999	107.6999969
-6.0132051	1.2200000	105.0499954
-6.0132051	1.2688000	106.5199966
-6.0132051	1.3176000	106.7699966
-6.0132051	1.3664000	109.0899963
-6.0132051	1.4152000	103.6599960
-6.0132051	1.4640000	104.9399948
-6.0132051	1.5128000	107.8099976
-6.0132051	1.5616000	107.7500000
-6.0132051	1.6104000	107.3099976
-6.0132051	1.6592000	110.6500015
-6.0132051	1.7079999	110.8300018
-6.0132051	1.7567999	108.5999985
-6.0132051	1.8055999	109.2500000
-6.0132051	1.8543999	111.7399979
-6.0132051	1.9031999	112.2200012
-6.0132051	1.9519999	111.5400009
-6.0132051	2.0007999	103.7699966
-6.0132051	2.0495999	102.2500000
-6.0132051	2.0983999	105.3899994
-6.0132051	2.1471999	104.1999969
-6.0132051	2.1959999	105.5599976
-6.0132051	2.2447999	103.0100021
-6.0132051	2.2935998	107.3199997
-6.0132051	2.3423998	106.7999954
-6.0132051	2.3912001	105.9899979
-6.0132051	2.4400001	101.0199966
-6.0132051	2.4888000	103.9899979
-6.0132051	2.5376000	106.7799988
-6.0132051	2.5864000	108.4499969
-6.0132051	2.6352000	105.9899979
-6.0132051	2.6840000	104.7500000
-6.0132051	2.7328000	105.9300003
-6.0132051	2.7816000	104.2900009
-6.0132051	2.8304000	99.3300018

-6.0132051	2.8792000	98.6800003
-6.0132051	2.9280000	102.1899948
-6.0132051	2.9768000	103.0299988
-6.0132051	3.0256000	105.3199997
-6.0132051	3.0743999	101.7399979
-6.0132051	3.1231999	96.8300018
-6.0132051	3.1719999	105.5499954
-6.0132051	3.2207999	103.6399994
-6.0132051	3.2695999	102.4599991
-6.0132051	3.3183999	105.3199997
-6.0132051	3.3671999	104.4000015
-6.0132051	3.4159999	103.3899994
-6.0132051	3.4647999	104.3300018
-6.0132051	3.5135999	102.9399948
-6.0132051	3.5623999	101.5299988
-6.0132051	3.6111999	102.0899963
-6.0132051	3.6599998	105.6800003
-6.0132051	3.7087998	106.3399963
-6.0132051	3.7575998	104.9599991
-6.0132051	3.8063998	104.7500000
-6.0132051	3.8551998	106.7900009
-6.0132051	3.9039998	105.8699951
-6.0132051	3.9527998	105.8399963
-6.0132051	4.0016003	105.5699997
-6.0132051	4.0503998	104.8799973
-6.0132051	4.0991998	104.5599976
-6.0132051	4.1479998	104.8799973
-6.0132051	4.1967998	103.2699966
-6.0132051	4.2455997	102.2799988
-6.0132051	4.2943997	106.9499969
-6.0132051	4.3431997	110.3199997
-6.0132051	4.3919997	107.8099976
-6.0132051	4.4407997	105.0100021
-6.0132051	4.4895997	109.1100006
-6.0132051	4.5383997	114.3600006
-6.0132051	4.5871997	114.1599960
-6.0132051	4.6359997	117.4399948
-6.0132051	4.6847997	118.0299988
-6.0132051	4.7336001	115.6999969
-6.0132051	4.7824001	111.0899963
-6.0132051	4.8312001	110.8699951
-6.0132051	4.8800001	109.7099991
-6.0132051	4.9288001	108.1199951
-6.0132051	4.9776001	104.4300003
-6.0132051	5.0264001	108.0199966
-6.0132051	5.0752001	108.6599960
-6.0132051	5.1240001	106.6899948
-6.0132051	5.1728001	104.0800018
-6.0132051	5.2216001	101.7799988
-6.0132051	5.2704000	106.3300018
-6.0132051	5.3192000	105.5999985
-6.0132051	5.3680000	103.7099991
-6.0132051	5.4168000	103.1899948
-6.0132051	5.4656000	103.6999969
-6.0132051	5.5144000	106.5299988
-6.0132051	5.5632000	107.4599991
-6.0132051	5.6120000	105.7799988
-6.0132051	5.6608000	107.1999969

-6.0132051	5.7096000	107.5899963
-6.0132051	5.7584000	105.7600021
-6.0132051	5.8072000	104.4899979
-6.0132051	5.8559999	103.3499985
-6.0132051	5.9047999	105.7500000
-6.0132051	5.9535999	107.3099976
-6.0132051	6.0023999	105.2799988
-6.0132051	6.0511999	105.6599960
-6.0132051	6.0999999	105.8699951
-6.0132051	6.1487999	105.4300003
-6.0132051	6.1975999	103.0400009
-6.0132051	6.2463999	103.0100021
-6.0132051	6.2951999	103.8499985
-6.0132051	6.3439999	103.3899994
-6.0132051	6.3927999	102.8899994
-6.0132051	6.4415998	105.6100006
-6.0132051	6.4903998	107.4599991
-6.0132051	6.5391998	104.2999954
-6.0132051	6.5879998	101.8600006
-6.0132051	6.6367998	103.2999954
-6.0132051	6.6855998	103.6399994
-6.0132051	6.7343998	101.7600021
-6.0132051	6.7831998	101.6299973
-6.0132051	6.8319998	99.8799973
-6.0132051	6.8807998	105.0499954
-6.0132051	6.9295998	106.1800003
-6.0132051	6.9783998	106.0299988
-6.0132051	7.0271997	104.9000015
-6.0132051	7.0759997	102.0499954
-6.0132051	7.1247997	101.6699982
-6.0132051	7.1735997	100.8099976
-6.0132051	7.2223997	102.1699982
-6.0132051	7.2711997	102.9300003
-6.0132051	7.3199997	95.9000015
-6.0132051	7.3687997	102.9099960
-6.0132051	7.4175997	99.3799973
-6.0132051	7.4663997	98.8699951
-6.0132051	7.5151997	103.9899979
-6.0132051	7.5639997	104.4899979
-6.0132051	7.6127996	104.7099991
-6.0132051	7.6615996	105.0299988
-6.0132051	7.7103996	106.6199951
-6.0132051	7.7591996	106.4499969
-6.0132051	7.8079996	104.4300003
-6.0132051	7.8567996	104.7699966
-6.0132051	7.9056001	104.6899948
-6.0132051	7.9544001	105.0100021
-6.0132051	8.0032005	102.2500000
-6.0132051	8.0520000	102.8300018
-6.0132051	8.1008005	106.6100006
-6.0132051	8.1496000	104.8399963
-6.0132051	8.1984005	103.4799957
-6.0132051	8.2472000	103.9099960
-6.0132051	8.2960005	101.2600021
-6.0132051	8.3448000	102.3699951
-6.0132051	8.3936005	102.9599991
-6.0132051	8.4424000	101.0599976
-6.0132051	8.4912004	103.9499969

-6.0132051	8.5400000	105.8099976
-6.0132051	8.5888004	103.7099991
-6.0132051	8.6375999	102.2399979
-6.0132051	8.6864004	102.5100021
-6.0132051	8.7351999	101.5800018
-6.0132051	8.7840004	101.9499969
-6.0132051	8.8327999	100.3199997
-6.0132051	8.8816004	102.1899948
-6.0132051	8.9303999	105.5800018
-6.0132051	8.9792004	106.1599960
-6.0132051	9.0279999	107.3499985
-6.0132051	9.0768003	106.4199982
-6.0132051	9.1255999	104.8199997
-6.0132051	9.1744003	106.0999985
-6.0132051	9.2231998	106.1999969
-6.0132051	9.2720003	105.6800003
-6.0132051	9.3207998	105.2399979
-6.0132051	9.3696003	102.4799957
-6.0132051	9.4184008	104.0699997
-6.0132051	9.4672003	103.4599991
-6.0132051	9.5160007	103.7200012
-6.0132051	9.5648003	101.6399994
-6.0132051	9.6136007	103.6999969
-6.0132051	9.6624002	102.8099976
-6.0132051	9.7112007	99.6699982
-6.0132051	9.7600002	102.9399948
-6.0132051	9.8088007	104.0000000
-6.0132051	9.8576002	103.7900009
-6.0132051	9.9064007	100.1500015
-6.0132051	9.9552002	100.0199966
-6.0132051	10.0040007	101.6399994
-6.0132051	10.0528002	100.0400009
-6.0132051	10.1016006	102.6399994
-6.0132051	10.1504002	104.3699951
-6.0132051	10.1992006	102.8199997
-6.0132051	10.2480001	105.1100006
-6.0132051	10.2968006	105.0000000
-6.0132051	10.3456001	105.9300003
-6.0132051	10.3944006	103.7399979
-6.0132051	10.4432001	103.4499969
-6.0132051	10.4920006	103.6899948
-6.0132051	10.5408001	103.3699951
-6.0132051	10.5896006	101.2500000
-6.0132051	10.6384001	103.2200012
-6.0132051	10.6872005	104.6999969
-6.0132051	10.7360001	102.8799973
-6.0132051	10.7848005	103.7600021
-6.0132051	10.8336000	103.7500000
-6.0132051	10.8824005	102.7399979
-6.0132051	10.9312000	103.3099976
-6.0132051	10.9800005	100.5499954
-6.0132051	11.0288000	102.9399948
-6.0132051	11.0776005	104.2200012
-6.0132051	11.1264000	100.7299957
-6.0132051	11.1752005	101.4300003
-6.0132051	11.2240000	101.7600021
-6.0132051	11.2728004	102.1899948
-6.0132051	11.3216000	96.5699997

-6.0132051	11.3704004	102.7600021
-6.0132051	11.4191999	105.7500000
-6.0132051	11.4680004	104.9499969
-6.0132051	11.5167999	104.2799988
-6.0132051	11.5656004	104.6800003
-6.0132051	11.6143999	103.2299957
-6.0132051	11.6632004	99.2699966
-6.0132051	11.7119999	103.8499985
-6.0132051	11.7608004	105.1100006
-6.0132051	11.8095999	105.9499969
-6.0132051	11.8584003	105.6299973
-6.0132051	11.9071999	106.1299973
-6.0132051	11.9560003	106.1899948
-6.0132051	12.0047998	106.9899979
-6.0132051	12.0536003	105.2200012
-6.0132051	12.1023998	105.5100021
-6.0132051	12.1512003	104.2500000
-6.0132051	12.1999998	102.9899979
-6.0132051	12.2488003	102.6699982
-6.0132051	12.2975998	102.5899963
-6.0132051	12.3464003	101.6299973
-6.0132051	12.3951998	99.2200012
-6.0132051	12.4440002	98.9499969
-6.0132051	12.4927998	102.8799973
-6.0132051	12.5416002	103.3600006
-6.0132051	12.5904007	100.9700012
-6.0132051	12.6392002	102.9099960
-6.0132051	12.6880007	103.8300018
-6.0132051	12.7368002	104.6800003
-6.0132051	12.7856007	104.0800018
-6.0132051	12.8344002	106.7399979
-6.0132051	12.8832006	106.7099991
-6.0132051	12.9320002	105.3300018
-6.0132051	12.9808006	103.3399963
-6.0132051	13.0296001	101.3199997
-6.0132051	13.0784006	105.0599976
-6.0132051	13.1272001	104.9099960
-6.0132051	13.1760006	101.2999954
-6.0132051	13.2248001	96.6699982
-6.0132051	13.2736006	101.6100006
-6.0132051	13.3224001	104.5999985
-6.0132051	13.3712006	103.9399948
-6.0132051	13.4200001	102.7099991
-6.0132051	13.4688005	99.6999969
-6.0132051	13.5176001	101.7699966
-6.0132051	13.5664005	103.5000000
-6.0132051	13.6152000	104.5899963
-6.0132051	13.6640005	103.6599960
-6.0132051	13.7128000	101.5299988
-6.0132051	13.7616005	100.6199951
-6.0132051	13.8104000	103.5100021
-6.0132051	13.8592005	104.4099960
-6.0132051	13.9080000	103.7399979
-6.0132051	13.9568005	102.4199982
-6.0132051	14.0056000	97.4599991
-6.0132051	14.0544004	99.8799973
-6.0132051	14.1032000	103.8399963
-6.0132051	14.1520004	104.3600006

-6.0132051	14.2007999	100.6699982
-6.0132051	14.2496004	101.8499985
-6.0132051	14.2983999	102.6399994
-6.0132051	14.3472004	103.7099991
-6.0132051	14.3959999	99.4499969
-6.0132051	14.4448004	102.3399963
-6.0132051	14.4935999	103.1399994
-6.0132051	14.5424004	104.6800003
-6.0132051	14.5911999	104.0199966
-6.0132051	14.6400003	104.0299988
-6.0132051	14.6887999	103.9499969
-6.0132051	14.7376003	102.8799973
-6.0132051	14.7863998	100.5699997
-6.0132051	14.8352003	100.7900009
-6.0132051	14.8839998	104.2600021
-6.0132051	14.9328003	104.6899948
-6.0132051	14.9815998	104.4599991
-6.0132051	15.0304003	101.8499985
-6.0070691	0.3416000	108.5899963
-6.0070691	0.3904000	108.3499985
-6.0070691	0.4392000	107.2799988
-6.0070691	0.4880000	104.2900009
-6.0070691	0.5368000	106.6999969
-6.0070691	0.5856000	105.6699982
-6.0070691	0.6344000	106.2799988
-6.0070691	0.6832000	107.3600006
-6.0070691	0.7320000	106.5100021
-6.0070691	0.7808000	108.6899948
-6.0070691	0.8296000	110.7200012
-6.0070691	0.8784000	109.1399994
-6.0070691	0.9272000	105.3899994
-6.0070691	0.9760000	107.4000015
-6.0070691	1.0247999	107.0999985
-6.0070691	1.0735999	106.9300003
-6.0070691	1.1223999	106.6399994
-6.0070691	1.1711999	108.1999969
-6.0070691	1.2200000	105.5499954
-6.0070691	1.2688000	106.3600006
-6.0070691	1.3176000	106.7799988
-6.0070691	1.3664000	108.5800018
-6.0070691	1.4152000	104.7999954
-6.0070691	1.4640000	105.0299988
-6.0070691	1.5128000	107.9199982
-6.0070691	1.5616000	107.9399948
-6.0070691	1.6104000	108.7399979
-6.0070691	1.6592000	111.0199966
-6.0070691	1.7079999	110.1500015
-6.0070691	1.7567999	108.2399979
-6.0070691	1.8055999	109.9000015
-6.0070691	1.8543999	112.2500000
-6.0070691	1.9031999	112.2999954
-6.0070691	1.9519999	111.7399979
-6.0070691	2.0007999	103.2999954
-6.0070691	2.0495999	103.0499954
-6.0070691	2.0983999	105.8499985
-6.0070691	2.1471999	105.0000000
-6.0070691	2.1959999	105.2099991
-6.0070691	2.2447999	103.5899963

-6.0070691	2.2935998	106.4099960
-6.0070691	2.3423998	106.7099991
-6.0070691	2.3912001	106.2699966
-6.0070691	2.4400001	100.8099976
-6.0070691	2.4888000	103.0999985
-6.0070691	2.5376000	106.7900009
-6.0070691	2.5864000	108.4300003
-6.0070691	2.6352000	106.4700012
-6.0070691	2.6840000	106.0299988
-6.0070691	2.7328000	104.9399948
-6.0070691	2.7816000	104.0999985
-6.0070691	2.8304000	99.9700012
-6.0070691	2.8792000	100.1699982
-6.0070691	2.9280000	102.1500015
-6.0070691	2.9768000	103.7699966
-6.0070691	3.0256000	105.8799973
-6.0070691	3.0743999	102.0999985
-6.0070691	3.1231999	97.1299973
-6.0070691	3.1719999	106.2600021
-6.0070691	3.2207999	103.9499969
-6.0070691	3.2695999	100.8099976
-6.0070691	3.3183999	105.2799988
-6.0070691	3.3671999	105.1500015
-6.0070691	3.4159999	103.1999969
-6.0070691	3.4647999	104.8899994
-6.0070691	3.5135999	102.7699966
-6.0070691	3.5623999	100.0000000
-6.0070691	3.6111999	100.6500015
-6.0070691	3.6599998	104.0499954
-6.0070691	3.7087998	105.7500000
-6.0070691	3.7575998	105.2799988
-6.0070691	3.8063998	104.6500015
-6.0070691	3.8551998	106.9000015
-6.0070691	3.9039998	106.4799957
-6.0070691	3.9527998	106.1399994
-6.0070691	4.0016003	105.2999954
-6.0070691	4.0503998	103.9499969
-6.0070691	4.0991998	103.6299973
-6.0070691	4.1479998	104.1699982
-6.0070691	4.1967998	103.2200012
-6.0070691	4.2455997	102.0899963
-6.0070691	4.2943997	107.3499985
-6.0070691	4.3431997	109.9399948
-6.0070691	4.3919997	106.2399979
-6.0070691	4.4407997	104.7799988
-6.0070691	4.4895997	109.5100021
-6.0070691	4.5383997	114.2999954
-6.0070691	4.5871997	113.1100006
-6.0070691	4.6359997	117.1100006
-6.0070691	4.6847997	118.4199982
-6.0070691	4.7336001	116.3199997
-6.0070691	4.7824001	111.8899994
-6.0070691	4.8312001	110.7099991
-6.0070691	4.8800001	109.5800018
-6.0070691	4.9288001	109.3399963
-6.0070691	4.9776001	105.3499985
-6.0070691	5.0264001	107.2500000
-6.0070691	5.0752001	107.9499969

-6.0070691	5.1240001	107.0100021
-6.0070691	5.1728001	104.2600021
-6.0070691	5.2216001	101.3099976
-6.0070691	5.2704000	105.8600006
-6.0070691	5.3192000	103.8499985
-6.0070691	5.3680000	103.2500000
-6.0070691	5.4168000	104.0800018
-6.0070691	5.4656000	105.0000000
-6.0070691	5.5144000	107.0999985
-6.0070691	5.5632000	107.6100006
-6.0070691	5.6120000	106.1800003
-6.0070691	5.6608000	107.2799988
-6.0070691	5.7096000	108.0100021
-6.0070691	5.7584000	106.4300003
-6.0070691	5.8072000	104.5800018
-6.0070691	5.8559999	104.0499954
-6.0070691	5.9047999	106.2500000
-6.0070691	5.9535999	108.0800018
-6.0070691	6.0023999	106.5299988
-6.0070691	6.0511999	106.0000000
-6.0070691	6.0999999	105.4899979
-6.0070691	6.1487999	104.3699951
-6.0070691	6.1975999	103.5599976
-6.0070691	6.2463999	102.0800018
-6.0070691	6.2951999	103.1599960
-6.0070691	6.3439999	103.9700012
-6.0070691	6.3927999	103.2399979
-6.0070691	6.4415998	105.0400009
-6.0070691	6.4903998	106.5499954
-6.0070691	6.5391998	101.7999954
-6.0070691	6.5879998	101.2299957
-6.0070691	6.6367998	103.0800018
-6.0070691	6.6855998	104.0400009
-6.0070691	6.7343998	102.8399963
-6.0070691	6.7831998	102.3199997
-6.0070691	6.8319998	100.7699966
-6.0070691	6.8807998	103.9399948
-6.0070691	6.9295998	105.6999969
-6.0070691	6.9783998	106.1500015
-6.0070691	7.0271997	104.4700012
-6.0070691	7.0759997	101.5199966
-6.0070691	7.1247997	101.1599960
-6.0070691	7.1735997	101.4799957
-6.0070691	7.2223997	101.5800018
-6.0070691	7.2711997	102.6699982
-6.0070691	7.3199997	97.2600021
-6.0070691	7.3687997	103.4000015
-6.0070691	7.4175997	99.1199951
-6.0070691	7.4663997	99.6199951
-6.0070691	7.5151997	104.2699966
-6.0070691	7.5639997	105.1599960
-6.0070691	7.6127996	105.7500000
-6.0070691	7.6615996	105.6299973
-6.0070691	7.7103996	106.7999954
-6.0070691	7.7591996	106.5199966
-6.0070691	7.8079996	104.7399979
-6.0070691	7.8567996	104.1199951
-6.0070691	7.9056001	103.5899963

-6.0070691	7.9544001	104.3399963
-6.0070691	8.0032005	101.8399963
-6.0070691	8.0520000	103.5199966
-6.0070691	8.1008005	105.8799973
-6.0070691	8.1496000	104.4700012
-6.0070691	8.1984005	102.9799957
-6.0070691	8.2472000	103.2799988
-6.0070691	8.2960005	100.4000015
-6.0070691	8.3448000	102.6899948
-6.0070691	8.3936005	103.6199951
-6.0070691	8.4424000	101.6199951
-6.0070691	8.4912004	104.4499969
-6.0070691	8.5400000	105.3199997
-6.0070691	8.5888004	103.4099960
-6.0070691	8.6375999	102.2500000
-6.0070691	8.6864004	102.3099976
-6.0070691	8.7351999	101.1100006
-6.0070691	8.7840004	102.0199966
-6.0070691	8.8327999	100.1599960
-6.0070691	8.8816004	102.1199951
-6.0070691	8.9303999	105.5999985
-6.0070691	8.9792004	106.0299988
-6.0070691	9.0279999	107.4799957
-6.0070691	9.0768003	106.4599991
-6.0070691	9.1255999	104.0800018
-6.0070691	9.1744003	105.8399963
-6.0070691	9.2231998	106.0199966
-6.0070691	9.2720003	105.3199997
-6.0070691	9.3207998	104.5699997
-6.0070691	9.3696003	101.7699966
-6.0070691	9.4184008	104.6199951
-6.0070691	9.4672003	104.8099976
-6.0070691	9.5160007	104.3399963
-6.0070691	9.5648003	101.1599960
-6.0070691	9.6136007	104.1999969
-6.0070691	9.6624002	103.0599976
-6.0070691	9.7112007	98.2099991
-6.0070691	9.7600002	102.3899994
-6.0070691	9.8088007	103.9700012
-6.0070691	9.8576002	104.1100006
-6.0070691	9.9064007	101.1299973
-6.0070691	9.9552002	101.0699997
-6.0070691	10.0040007	102.3499985
-6.0070691	10.0528002	99.5100021
-6.0070691	10.1016006	102.6999969
-6.0070691	10.1504002	103.9799957
-6.0070691	10.1992006	102.1500015
-6.0070691	10.2480001	104.8199997
-6.0070691	10.2968006	104.6100006
-6.0070691	10.3456001	105.5199966
-6.0070691	10.3944006	102.7799988
-6.0070691	10.4432001	102.6500015
-6.0070691	10.4920006	103.5400009
-6.0070691	10.5408001	102.9399948
-6.0070691	10.5896006	101.4300003
-6.0070691	10.6384001	103.1699982
-6.0070691	10.6872005	102.9000015
-6.0070691	10.7360001	102.0899963

-6.0070691	10.7848005	104.1599960
-6.0070691	10.8336000	104.1800003
-6.0070691	10.8824005	103.2799988
-6.0070691	10.9312000	103.2699966
-6.0070691	10.9800005	99.4399948
-6.0070691	11.0288000	102.5199966
-6.0070691	11.0776005	104.5800018
-6.0070691	11.1264000	100.3199997
-6.0070691	11.1752005	101.3399963
-6.0070691	11.2240000	101.0499954
-6.0070691	11.2728004	102.3199997
-6.0070691	11.3216000	97.7999954
-6.0070691	11.3704004	102.9499969
-6.0070691	11.4191999	105.7099991
-6.0070691	11.4680004	104.5499954
-6.0070691	11.5167999	103.6299973
-6.0070691	11.5656004	104.2999954
-6.0070691	11.6143999	103.6599960
-6.0070691	11.6632004	98.5000000
-6.0070691	11.7119999	103.4199982
-6.0070691	11.7608004	104.0800018
-6.0070691	11.8095999	104.3399963
-6.0070691	11.8584003	105.5299988
-6.0070691	11.9071999	106.0299988
-6.0070691	11.9560003	104.7699966
-6.0070691	12.0047998	107.3799973
-6.0070691	12.0536003	106.7399979
-6.0070691	12.1023998	106.2399979
-6.0070691	12.1512003	103.0800018
-6.0070691	12.1999998	102.0299988
-6.0070691	12.2488003	102.9099960
-6.0070691	12.2975998	102.6500015
-6.0070691	12.3464003	101.5400009
-6.0070691	12.3951998	100.2999954
-6.0070691	12.4440002	99.2600021
-6.0070691	12.4927998	103.3600006
-6.0070691	12.5416002	103.8399963
-6.0070691	12.5904007	100.8199997
-6.0070691	12.6392002	101.7399979
-6.0070691	12.6880007	102.4799957
-6.0070691	12.7368002	103.6899948
-6.0070691	12.7856007	103.3199997
-6.0070691	12.8344002	106.4399948
-6.0070691	12.8832006	106.1199951
-6.0070691	12.9320002	104.5499954
-6.0070691	12.9808006	102.1599960
-6.0070691	13.0296001	100.5000000
-6.0070691	13.0784006	103.0199966
-6.0070691	13.1272001	104.1500015
-6.0070691	13.1760006	100.4399948
-6.0070691	13.2248001	96.0199966
-6.0070691	13.2736006	101.3199997
-6.0070691	13.3224001	104.2299957
-6.0070691	13.3712006	103.0800018
-6.0070691	13.4200001	102.3099976
-6.0070691	13.4688005	99.5100021
-6.0070691	13.5176001	100.7900009
-6.0070691	13.5664005	103.3799973

-6.0070691	13.6152000	104.1800003
-6.0070691	13.6640005	102.2799988
-6.0070691	13.7128000	99.8799973
-6.0070691	13.7616005	100.5199966
-6.0070691	13.8104000	103.2900009
-6.0070691	13.8592005	104.6699982
-6.0070691	13.9080000	103.5699997
-6.0070691	13.9568005	101.8899994
-6.0070691	14.0056000	97.1299973
-6.0070691	14.0544004	99.7600021
-6.0070691	14.1032000	102.9899979
-6.0070691	14.1520004	103.7200012
-6.0070691	14.2007999	100.9099960
-6.0070691	14.2496004	102.6500015
-6.0070691	14.2983999	102.9899979
-6.0070691	14.3472004	104.2999954
-6.0070691	14.3959999	100.8399963
-6.0070691	14.4448004	103.4899979
-6.0070691	14.4935999	102.4899979
-6.0070691	14.5424004	103.3199997
-6.0070691	14.5911999	103.2799988
-6.0070691	14.6400003	102.4399948
-6.0070691	14.6887999	102.5400009
-6.0070691	14.7376003	101.5899963
-6.0070691	14.7863998	99.9099960
-6.0070691	14.8352003	101.2500000
-6.0070691	14.8839998	103.9199982
-6.0070691	14.9328003	104.0400009
-6.0070691	14.9815998	103.7999954
-6.0070691	15.0304003	101.9499969
-6.0009332	0.3416000	108.5499954
-6.0009332	0.3904000	107.6100006
-6.0009332	0.4392000	106.8600006
-6.0009332	0.4880000	103.0899963
-6.0009332	0.5368000	106.3499985
-6.0009332	0.5856000	105.1399994
-6.0009332	0.6344000	105.3799973
-6.0009332	0.6832000	106.2900009
-6.0009332	0.7320000	107.3600006
-6.0009332	0.7808000	109.1699982
-6.0009332	0.8296000	110.4300003
-6.0009332	0.8784000	108.4799957
-6.0009332	0.9272000	104.5100021
-6.0009332	0.9760000	107.6800003
-6.0009332	1.0247999	107.3899994
-6.0009332	1.0735999	106.4000015
-6.0009332	1.1223999	106.6899948
-6.0009332	1.1711999	108.1500015
-6.0009332	1.2200000	106.1199951
-6.0009332	1.2688000	106.8799973
-6.0009332	1.3176000	107.0599976
-6.0009332	1.3664000	108.5000000
-6.0009332	1.4152000	106.2600021
-6.0009332	1.4640000	105.2600021
-6.0009332	1.5128000	107.9799957
-6.0009332	1.5616000	107.1800003
-6.0009332	1.6104000	109.2399979
-6.0009332	1.6592000	111.3300018

-6.0009332	1.7079999	109.7299957
-6.0009332	1.7567999	107.8799973
-6.0009332	1.8055999	110.2799988
-6.0009332	1.8543999	113.1299973
-6.0009332	1.9031999	113.0299988
-6.0009332	1.9519999	112.1199951
-6.0009332	2.0007999	102.4599991
-6.0009332	2.0495999	103.5400009
-6.0009332	2.0983999	106.0299988
-6.0009332	2.1471999	105.4799957
-6.0009332	2.1959999	104.6999969
-6.0009332	2.2447999	103.6699982
-6.0009332	2.2935998	105.0199966
-6.0009332	2.3423998	105.2399979
-6.0009332	2.3912001	106.2600021
-6.0009332	2.4400001	100.6500015
-6.0009332	2.4888000	102.0199966
-6.0009332	2.5376000	106.0199966
-6.0009332	2.5864000	108.2600021
-6.0009332	2.6352000	106.8799973
-6.0009332	2.6840000	107.1599960
-6.0009332	2.7328000	103.9599991
-6.0009332	2.7816000	103.9899979
-6.0009332	2.8304000	100.7799988
-6.0009332	2.8792000	101.3399963
-6.0009332	2.9280000	101.6800003
-6.0009332	2.9768000	104.1199951
-6.0009332	3.0256000	105.9399948
-6.0009332	3.0743999	102.8099976
-6.0009332	3.1231999	99.0299988
-6.0009332	3.1719999	107.1500015
-6.0009332	3.2207999	104.5199966
-6.0009332	3.2695999	99.9300003
-6.0009332	3.3183999	105.3699951
-6.0009332	3.3671999	105.7799988
-6.0009332	3.4159999	102.6100006
-6.0009332	3.4647999	104.6999969
-6.0009332	3.5135999	102.1299973
-6.0009332	3.5623999	98.1100006
-6.0009332	3.6111999	100.4099960
-6.0009332	3.6599998	102.2999954
-6.0009332	3.7087998	105.0800018
-6.0009332	3.7575998	105.6199951
-6.0009332	3.8063998	105.4899979
-6.0009332	3.8551998	107.3600006
-6.0009332	3.9039998	107.0000000
-6.0009332	3.9527998	106.2299957
-6.0009332	4.0016003	104.6399994
-6.0009332	4.0503998	102.6100006
-6.0009332	4.0991998	102.6199951
-6.0009332	4.1479998	103.5899963
-6.0009332	4.1967998	103.2999954
-6.0009332	4.2455997	101.6999969
-6.0009332	4.2943997	107.5400009
-6.0009332	4.3431997	109.7399979
-6.0009332	4.3919997	105.1699982
-6.0009332	4.4407997	105.1500015
-6.0009332	4.4895997	110.2099991

-6.0009332	4.5383997	114.7600021
-6.0009332	4.5871997	114.0599976
-6.0009332	4.6359997	117.0999985
-6.0009332	4.6847997	118.4499969
-6.0009332	4.7336001	116.5699997
-6.0009332	4.7824001	112.4099960
-6.0009332	4.8312001	110.7699966
-6.0009332	4.8800001	111.0400009
-6.0009332	4.9288001	110.8199997
-6.0009332	4.9776001	105.9599991
-6.0009332	5.0264001	106.5299988
-6.0009332	5.0752001	107.9199982
-6.0009332	5.1240001	107.7299957
-6.0009332	5.1728001	104.8099976
-6.0009332	5.2216001	101.8499985
-6.0009332	5.2704000	105.4700012
-6.0009332	5.3192000	102.2099991
-6.0009332	5.3680000	102.7299957
-6.0009332	5.4168000	103.9700012
-6.0009332	5.4656000	105.3499985
-6.0009332	5.5144000	107.4300003
-6.0009332	5.5632000	107.8099976
-6.0009332	5.6120000	106.7999954
-6.0009332	5.6608000	107.4300003
-6.0009332	5.7096000	108.0899963
-6.0009332	5.7584000	106.6199951
-6.0009332	5.8072000	104.2900009
-6.0009332	5.8559999	104.5100021
-6.0009332	5.9047999	106.3399963
-6.0009332	5.9535999	108.1800003
-6.0009332	6.0023999	107.0299988
-6.0009332	6.0511999	105.8600006
-6.0009332	6.0999999	105.5299988
-6.0009332	6.1487999	103.8699951
-6.0009332	6.1975999	104.3499985
-6.0009332	6.2463999	101.4899979
-6.0009332	6.2951999	103.3799973
-6.0009332	6.3439999	104.5899963
-6.0009332	6.3927999	103.5599976
-6.0009332	6.4415998	104.2399979
-6.0009332	6.4903998	106.0599976
-6.0009332	6.5391998	100.5999985
-6.0009332	6.5879998	101.5400009
-6.0009332	6.6367998	103.4000015
-6.0009332	6.6855998	104.9599991
-6.0009332	6.7343998	104.2900009
-6.0009332	6.7831998	103.2399979
-6.0009332	6.8319998	101.7399979
-6.0009332	6.8807998	101.8099976
-6.0009332	6.9295998	104.4499969
-6.0009332	6.9783998	106.0899963
-6.0009332	7.0271997	104.0699997
-6.0009332	7.0759997	101.2999954
-6.0009332	7.1247997	100.8199997
-6.0009332	7.1735997	102.0800018
-6.0009332	7.2223997	101.5499954
-6.0009332	7.2711997	102.3199997
-6.0009332	7.3199997	98.2600021

-6.0009332	7.3687997	103.4499969
-6.0009332	7.4175997	100.5000000
-6.0009332	7.4663997	100.5299988
-6.0009332	7.5151997	104.6299973
-6.0009332	7.5639997	105.8499985
-6.0009332	7.6127996	105.9199982
-6.0009332	7.6615996	106.7799988
-6.0009332	7.7103996	107.4300003
-6.0009332	7.7591996	106.9899979
-6.0009332	7.8079996	104.9599991
-6.0009332	7.8567996	103.5199966
-6.0009332	7.9056001	102.6100006
-6.0009332	7.9544001	103.8099976
-6.0009332	8.0032005	101.5999985
-6.0009332	8.0520000	104.2799988
-6.0009332	8.1008005	105.3300018
-6.0009332	8.1496000	104.4799957
-6.0009332	8.1984005	102.7099991
-6.0009332	8.2472000	103.0299988
-6.0009332	8.2960005	100.1100006
-6.0009332	8.3448000	103.6299973
-6.0009332	8.3936005	104.6800003
-6.0009332	8.4424000	102.0100021
-6.0009332	8.4912004	104.7399979
-6.0009332	8.5400000	105.3899994
-6.0009332	8.5888004	103.5199966
-6.0009332	8.6375999	102.5800018
-6.0009332	8.6864004	102.4599991
-6.0009332	8.7351999	102.1399994
-6.0009332	8.7840004	102.4099960
-6.0009332	8.8327999	100.2999954
-6.0009332	8.8816004	102.1899948
-6.0009332	8.9303999	105.1999969
-6.0009332	8.9792004	105.9399948
-6.0009332	9.0279999	107.3300018
-6.0009332	9.0768003	106.2999954
-6.0009332	9.1255999	103.2299957
-6.0009332	9.1744003	104.9599991
-6.0009332	9.2231998	105.2399979
-6.0009332	9.2720003	104.5800018
-6.0009332	9.3207998	104.0899963
-6.0009332	9.3696003	102.1500015
-6.0009332	9.4184008	105.6299973
-6.0009332	9.4672003	105.5800018
-6.0009332	9.5160007	104.5100021
-6.0009332	9.5648003	101.3199997
-6.0009332	9.6136007	104.2600021
-6.0009332	9.6624002	103.1500015
-6.0009332	9.7112007	96.9000015
-6.0009332	9.7600002	102.0199966
-6.0009332	9.8088007	103.3799973
-6.0009332	9.8576002	103.2900009
-6.0009332	9.9064007	102.0299988
-6.0009332	9.9552002	102.2699966
-6.0009332	10.0040007	103.1599960
-6.0009332	10.0528002	99.7699966
-6.0009332	10.1016006	102.8499985
-6.0009332	10.1504002	103.5899963

-6.0009332	10.1992006	101.3499985
-6.0009332	10.2480001	104.3199997
-6.0009332	10.2968006	103.9599991
-6.0009332	10.3456001	104.7099991
-6.0009332	10.3944006	101.7500000
-6.0009332	10.4432001	102.1800003
-6.0009332	10.4920006	103.2399979
-6.0009332	10.5408001	101.9199982
-6.0009332	10.5896006	101.7099991
-6.0009332	10.6384001	103.4000015
-6.0009332	10.6872005	101.1500015
-6.0009332	10.7360001	101.2399979
-6.0009332	10.7848005	104.3499985
-6.0009332	10.8336000	104.1800003
-6.0009332	10.8824005	103.2500000
-6.0009332	10.9312000	103.0299988
-6.0009332	10.9800005	98.0000000
-6.0009332	11.0288000	101.9099960
-6.0009332	11.0776005	104.6100006
-6.0009332	11.1264000	100.1500015
-6.0009332	11.1752005	102.4599991
-6.0009332	11.2240000	101.4599991
-6.0009332	11.2728004	103.0999985
-6.0009332	11.3216000	99.2200012
-6.0009332	11.3704004	103.4000015
-6.0009332	11.4191999	105.5599976
-6.0009332	11.4680004	104.0599976
-6.0009332	11.5167999	103.2600021
-6.0009332	11.5656004	104.0899963
-6.0009332	11.6143999	104.3799973
-6.0009332	11.6632004	98.4499969
-6.0009332	11.7119999	103.2500000
-6.0009332	11.7608004	103.2399979
-6.0009332	11.8095999	102.4399948
-6.0009332	11.8584003	105.9300003
-6.0009332	11.9071999	106.4199982
-6.0009332	11.9560003	103.6299973
-6.0009332	12.0047998	107.6500015
-6.0009332	12.0536003	107.4399948
-6.0009332	12.1023998	106.6299973
-6.0009332	12.1512003	102.3499985
-6.0009332	12.1999998	102.2600021
-6.0009332	12.2488003	103.7600021
-6.0009332	12.2975998	103.4899979
-6.0009332	12.3464003	102.4000015
-6.0009332	12.3951998	101.1100006
-6.0009332	12.4440002	98.5599976
-6.0009332	12.4927998	103.5000000
-6.0009332	12.5416002	104.5599976
-6.0009332	12.5904007	102.0000000
-6.0009332	12.6392002	100.9399948
-6.0009332	12.6880007	101.0899963
-6.0009332	12.7368002	102.6999969
-6.0009332	12.7856007	103.0299988
-6.0009332	12.8344002	106.0400009
-6.0009332	12.8832006	105.7500000
-6.0009332	12.9320002	104.3499985
-6.0009332	12.9808006	101.6800003

-6.0009332	13.0296001	100.6899948
-6.0009332	13.0784006	100.7600021
-6.0009332	13.1272001	103.0599976
-6.0009332	13.1760006	100.9099960
-6.0009332	13.2248001	96.1299973
-6.0009332	13.2736006	101.6800003
-6.0009332	13.3224001	103.9499969
-6.0009332	13.3712006	102.0199966
-6.0009332	13.4200001	101.6100006
-6.0009332	13.4688005	99.5899963
-6.0009332	13.5176001	99.6399994
-6.0009332	13.5664005	102.9199982
-6.0009332	13.6152000	103.3799973
-6.0009332	13.6640005	100.7500000
-6.0009332	13.7128000	98.1500015
-6.0009332	13.7616005	99.7500000
-6.0009332	13.8104000	102.0800018
-6.0009332	13.8592005	104.3499985
-6.0009332	13.9080000	103.8099976
-6.0009332	13.9568005	102.2900009
-6.0009332	14.0056000	97.9599991
-6.0009332	14.0544004	100.1599960
-6.0009332	14.1032000	102.6199951
-6.0009332	14.1520004	103.3699951
-6.0009332	14.2007999	101.3099976
-6.0009332	14.2496004	103.5400009
-6.0009332	14.2983999	103.8600006
-6.0009332	14.3472004	104.3099976
-6.0009332	14.3959999	101.0599976
-6.0009332	14.4448004	104.2600021
-6.0009332	14.4935999	102.3399963
-6.0009332	14.5424004	101.8699951
-6.0009332	14.5911999	102.8899994
-6.0009332	14.6400003	100.6800003
-6.0009332	14.6887999	101.1899948
-6.0009332	14.7376003	101.0299988
-6.0009332	14.7863998	100.0599976
-6.0009332	14.8352003	102.0199966
-6.0009332	14.8839998	103.6100006
-6.0009332	14.9328003	103.5899963
-6.0009332	14.9815998	103.3600006
-6.0009332	15.0304003	102.4899979
-5.9947972	0.3416000	107.9499969
-5.9947972	0.3904000	106.1699982
-5.9947972	0.4392000	105.8199997
-5.9947972	0.4880000	102.5599976
-5.9947972	0.5368000	105.6599960
-5.9947972	0.5856000	102.9899979
-5.9947972	0.6344000	103.8899994
-5.9947972	0.6832000	104.7900009
-5.9947972	0.7320000	107.4599991
-5.9947972	0.7808000	109.1199951
-5.9947972	0.8296000	109.6800003
-5.9947972	0.8784000	107.7500000
-5.9947972	0.9272000	103.7299957
-5.9947972	0.9760000	108.0699997
-5.9947972	1.0247999	107.7200012
-5.9947972	1.0735999	105.8600006

-5.9947972	1.1223999	106.4899979
-5.9947972	1.1711999	107.6899948
-5.9947972	1.2200000	106.3199997
-5.9947972	1.2688000	106.9599991
-5.9947972	1.3176000	106.6599960
-5.9947972	1.3664000	108.0400009
-5.9947972	1.4152000	107.5699997
-5.9947972	1.4640000	105.7399979
-5.9947972	1.5128000	108.2500000
-5.9947972	1.5616000	106.5100021
-5.9947972	1.6104000	109.5499954
-5.9947972	1.6592000	111.3199997
-5.9947972	1.7079999	108.3300018
-5.9947972	1.7567999	107.1800003
-5.9947972	1.8055999	110.5000000
-5.9947972	1.8543999	114.4799957
-5.9947972	1.9031999	114.3399963
-5.9947972	1.9519999	113.0299988
-5.9947972	2.0007999	102.8699951
-5.9947972	2.0495999	104.1299973
-5.9947972	2.0983999	106.3699951
-5.9947972	2.1471999	105.5299988
-5.9947972	2.1959999	104.0000000
-5.9947972	2.2447999	103.5299988
-5.9947972	2.2935998	104.1800003
-5.9947972	2.3423998	102.8199997
-5.9947972	2.3912001	106.4799957
-5.9947972	2.4400001	102.1599960
-5.9947972	2.4888000	101.8899994
-5.9947972	2.5376000	105.6299973
-5.9947972	2.5864000	107.7500000
-5.9947972	2.6352000	106.6500015
-5.9947972	2.6840000	107.4099960
-5.9947972	2.7328000	103.5699997
-5.9947972	2.7816000	104.2699966
-5.9947972	2.8304000	102.1999969
-5.9947972	2.8792000	102.5899963
-5.9947972	2.9280000	101.6800003
-5.9947972	2.9768000	104.4199982
-5.9947972	3.0256000	105.9199982
-5.9947972	3.0743999	104.0199966
-5.9947972	3.1231999	102.0199966
-5.9947972	3.1719999	108.5100021
-5.9947972	3.2207999	105.7799988
-5.9947972	3.2695999	99.6500015
-5.9947972	3.3183999	105.6999969
-5.9947972	3.3671999	106.6800003
-5.9947972	3.4159999	103.1100006
-5.9947972	3.4647999	103.6399994
-5.9947972	3.5135999	101.5699997
-5.9947972	3.5623999	96.5999985
-5.9947972	3.6111999	99.9199982
-5.9947972	3.6599998	100.7299957
-5.9947972	3.7087998	104.2099991
-5.9947972	3.7575998	106.1800003
-5.9947972	3.8063998	106.2699966
-5.9947972	3.8551998	107.5699997
-5.9947972	3.9039998	107.4300003

-5.9947972	3.9527998	106.4499969
-5.9947972	4.0016003	103.7799988
-5.9947972	4.0503998	102.0699997
-5.9947972	4.0991998	102.8300018
-5.9947972	4.1479998	103.8899994
-5.9947972	4.1967998	103.6699982
-5.9947972	4.2455997	102.2500000
-5.9947972	4.2943997	107.7099991
-5.9947972	4.3431997	109.8099976
-5.9947972	4.3919997	105.1399994
-5.9947972	4.4407997	105.4000015
-5.9947972	4.4895997	110.4300003
-5.9947972	4.5383997	115.5299988
-5.9947972	4.5871997	115.2699966
-5.9947972	4.6359997	117.1999969
-5.9947972	4.6847997	118.4099960
-5.9947972	4.7336001	116.3300018
-5.9947972	4.7824001	111.5899963
-5.9947972	4.8312001	109.9700012
-5.9947972	4.8800001	112.0599976
-5.9947972	4.9288001	112.0400009
-5.9947972	4.9776001	106.2600021
-5.9947972	5.0264001	106.0000000
-5.9947972	5.0752001	107.5100021
-5.9947972	5.1240001	107.9099960
-5.9947972	5.1728001	105.9300003
-5.9947972	5.2216001	103.6500015
-5.9947972	5.2704000	105.5599976
-5.9947972	5.3192000	101.9799957
-5.9947972	5.3680000	103.2299957
-5.9947972	5.4168000	103.0100021
-5.9947972	5.4656000	104.7699966
-5.9947972	5.5144000	107.3399963
-5.9947972	5.5632000	107.9799957
-5.9947972	5.6120000	107.4199982
-5.9947972	5.6608000	107.6599960
-5.9947972	5.7096000	108.0599976
-5.9947972	5.7584000	106.1800003
-5.9947972	5.8072000	103.3699951
-5.9947972	5.8559999	104.0400009
-5.9947972	5.9047999	105.9700012
-5.9947972	5.9535999	108.0899963
-5.9947972	6.0023999	107.2200012
-5.9947972	6.0511999	105.7200012
-5.9947972	6.0999999	105.5899963
-5.9947972	6.1487999	103.3099976
-5.9947972	6.1975999	104.1599960
-5.9947972	6.2463999	101.3199997
-5.9947972	6.2951999	103.4499969
-5.9947972	6.3439999	104.7500000
-5.9947972	6.3927999	103.7200012
-5.9947972	6.4415998	103.8600006
-5.9947972	6.4903998	105.5599976
-5.9947972	6.5391998	99.1100006
-5.9947972	6.5879998	101.3899994
-5.9947972	6.6367998	102.3499985
-5.9947972	6.6855998	105.4000015
-5.9947972	6.7343998	105.4700012

-5.9947972	6.7831998	104.2399979
-5.9947972	6.8319998	102.9099960
-5.9947972	6.8807998	99.3699951
-5.9947972	6.9295998	102.8899994
-5.9947972	6.9783998	105.6800003
-5.9947972	7.0271997	103.5199966
-5.9947972	7.0759997	101.3799973
-5.9947972	7.1247997	100.7099991
-5.9947972	7.1735997	102.0999985
-5.9947972	7.2223997	101.6199951
-5.9947972	7.2711997	101.5199966
-5.9947972	7.3199997	100.1699982
-5.9947972	7.3687997	103.3399963
-5.9947972	7.4175997	101.7399979
-5.9947972	7.4663997	100.9300003
-5.9947972	7.5151997	104.4199982
-5.9947972	7.5639997	106.2299957
-5.9947972	7.6127996	105.8799973
-5.9947972	7.6615996	107.5599976
-5.9947972	7.7103996	107.4599991
-5.9947972	7.7591996	106.4700012
-5.9947972	7.8079996	104.6299973
-5.9947972	7.8567996	102.3399963
-5.9947972	7.9056001	101.2600021
-5.9947972	7.9544001	103.2200012
-5.9947972	8.0032005	101.7999954
-5.9947972	8.0520000	105.2500000
-5.9947972	8.1008005	104.7299957
-5.9947972	8.1496000	104.4899979
-5.9947972	8.1984005	102.1100006
-5.9947972	8.2472000	102.6599960
-5.9947972	8.2960005	100.5499954
-5.9947972	8.3448000	104.0699997
-5.9947972	8.3936005	105.5699997
-5.9947972	8.4424000	103.5100021
-5.9947972	8.4912004	105.2099991
-5.9947972	8.5400000	105.5400009
-5.9947972	8.5888004	103.8499985
-5.9947972	8.6375999	103.2699966
-5.9947972	8.6864004	103.1699982
-5.9947972	8.7351999	102.6399994
-5.9947972	8.7840004	102.2900009
-5.9947972	8.8327999	100.2500000
-5.9947972	8.8816004	102.3499985
-5.9947972	8.9303999	105.0899963
-5.9947972	8.9792004	105.7099991
-5.9947972	9.0279999	106.6100006
-5.9947972	9.0768003	105.8300018
-5.9947972	9.1255999	103.4000015
-5.9947972	9.1744003	104.2500000
-5.9947972	9.2231998	104.2299957
-5.9947972	9.2720003	103.4499969
-5.9947972	9.3207998	103.3699951
-5.9947972	9.3696003	102.9199982
-5.9947972	9.4184008	106.2799988
-5.9947972	9.4672003	106.0499954
-5.9947972	9.5160007	104.7799988
-5.9947972	9.5648003	101.7799988

-5.9947972	9.6136007	104.1699982
-5.9947972	9.6624002	103.0100021
-5.9947972	9.7112007	95.6599960
-5.9947972	9.7600002	101.7699966
-5.9947972	9.8088007	103.5699997
-5.9947972	9.8576002	102.5599976
-5.9947972	9.9064007	102.4899979
-5.9947972	9.9552002	103.1199951
-5.9947972	10.0040007	103.7999954
-5.9947972	10.0528002	100.9300003
-5.9947972	10.1016006	103.3600006
-5.9947972	10.1504002	104.0199966
-5.9947972	10.1992006	101.8600006
-5.9947972	10.2480001	104.0599976
-5.9947972	10.2968006	103.0999985
-5.9947972	10.3456001	103.7099991
-5.9947972	10.3944006	101.1100006
-5.9947972	10.4432001	102.9099960
-5.9947972	10.4920006	103.5699997
-5.9947972	10.5408001	101.2999954
-5.9947972	10.5896006	102.1500015
-5.9947972	10.6384001	103.7299957
-5.9947972	10.6872005	99.9399948
-5.9947972	10.7360001	100.6899948
-5.9947972	10.7848005	104.2999954
-5.9947972	10.8336000	103.6800003
-5.9947972	10.8824005	102.5999985
-5.9947972	10.9312000	102.7200012
-5.9947972	10.9800005	98.9700012
-5.9947972	11.0288000	102.7600021
-5.9947972	11.0776005	104.8300018
-5.9947972	11.1264000	99.6999969
-5.9947972	11.1752005	103.2900009
-5.9947972	11.2240000	101.6599960
-5.9947972	11.2728004	104.0499954
-5.9947972	11.3216000	101.3099976
-5.9947972	11.3704004	104.1800003
-5.9947972	11.4191999	105.4799957
-5.9947972	11.4680004	102.9300003
-5.9947972	11.5167999	102.3499985
-5.9947972	11.5656004	104.0000000
-5.9947972	11.6143999	104.5699997
-5.9947972	11.6632004	98.0599976
-5.9947972	11.7119999	103.0599976
-5.9947972	11.7608004	103.1599960
-5.9947972	11.8095999	100.4300003
-5.9947972	11.8584003	105.5499954
-5.9947972	11.9071999	106.4399948
-5.9947972	11.9560003	102.6999969
-5.9947972	12.0047998	107.8399963
-5.9947972	12.0536003	107.5000000
-5.9947972	12.1023998	105.8499985
-5.9947972	12.1512003	100.5400009
-5.9947972	12.1999998	102.0899963
-5.9947972	12.2488003	104.6699982
-5.9947972	12.2975998	104.3199997
-5.9947972	12.3464003	103.4799957
-5.9947972	12.3951998	102.5699997

-5.9947972	12.4440002	99.0199966
-5.9947972	12.4927998	103.4199982
-5.9947972	12.5416002	104.4199982
-5.9947972	12.5904007	102.7999954
-5.9947972	12.6392002	100.2600021
-5.9947972	12.6880007	99.9199982
-5.9947972	12.7368002	101.5999985
-5.9947972	12.7856007	103.0000000
-5.9947972	12.8344002	105.5699997
-5.9947972	12.8832006	104.6100006
-5.9947972	12.9320002	103.3600006
-5.9947972	12.9808006	101.3600006
-5.9947972	13.0296001	101.4199982
-5.9947972	13.0784006	98.9399948
-5.9947972	13.1272001	102.1299973
-5.9947972	13.1760006	101.1100006
-5.9947972	13.2248001	96.5400009
-5.9947972	13.2736006	102.0599976
-5.9947972	13.3224001	103.3399963
-5.9947972	13.3712006	101.1399994
-5.9947972	13.4200001	101.0899963
-5.9947972	13.4688005	100.6299973
-5.9947972	13.5176001	100.4599991
-5.9947972	13.5664005	103.5499954
-5.9947972	13.6152000	103.4499969
-5.9947972	13.6640005	100.2099991
-5.9947972	13.7128000	96.9399948
-5.9947972	13.7616005	98.7099991
-5.9947972	13.8104000	100.4899979
-5.9947972	13.8592005	104.3399963
-5.9947972	13.9080000	104.6100006
-5.9947972	13.9568005	103.0100021
-5.9947972	14.0056000	99.6199951
-5.9947972	14.0544004	100.4499969
-5.9947972	14.1032000	101.9899979
-5.9947972	14.1520004	102.6699982
-5.9947972	14.2007999	101.7900009
-5.9947972	14.2496004	104.4099960
-5.9947972	14.2983999	105.0699997
-5.9947972	14.3472004	104.7600021
-5.9947972	14.3959999	101.5999985
-5.9947972	14.4448004	104.4700012
-5.9947972	14.4935999	102.5499954
-5.9947972	14.5424004	101.0800018
-5.9947972	14.5911999	102.3899994
-5.9947972	14.6400003	98.1399994
-5.9947972	14.6887999	99.4099960
-5.9947972	14.7376003	100.6399994
-5.9947972	14.7863998	100.2500000
-5.9947972	14.8352003	102.8499985
-5.9947972	14.8839998	103.6800003
-5.9947972	14.9328003	103.3899994
-5.9947972	14.9815998	103.0699997
-5.9947972	15.0304003	102.9199982
-5.9886613	0.3416000	107.2399979
-5.9886613	0.3904000	105.6800003
-5.9886613	0.4392000	105.1100006
-5.9886613	0.4880000	101.9799957

-5.9886613	0.5368000	104.7600021
-5.9886613	0.5856000	101.2799988
-5.9886613	0.6344000	102.7999954
-5.9886613	0.6832000	103.1999969
-5.9886613	0.7320000	107.0599976
-5.9886613	0.7808000	108.8699951
-5.9886613	0.8296000	108.6199951
-5.9886613	0.8784000	107.0100021
-5.9886613	0.9272000	105.1100006
-5.9886613	0.9760000	108.9199982
-5.9886613	1.0247999	108.0100021
-5.9886613	1.0735999	105.1599960
-5.9886613	1.1223999	106.4199982
-5.9886613	1.1711999	107.8699951
-5.9886613	1.2200000	107.2600021
-5.9886613	1.2688000	107.1599960
-5.9886613	1.3176000	106.3799973
-5.9886613	1.3664000	107.5599976
-5.9886613	1.4152000	107.8600006
-5.9886613	1.4640000	106.1500015
-5.9886613	1.5128000	108.5999985
-5.9886613	1.5616000	106.4399948
-5.9886613	1.6104000	109.6599960
-5.9886613	1.6592000	110.7200012
-5.9886613	1.7079999	106.7799988
-5.9886613	1.7567999	106.5699997
-5.9886613	1.8055999	110.4499969
-5.9886613	1.8543999	115.7099991
-5.9886613	1.9031999	116.3699951
-5.9886613	1.9519999	114.1500015
-5.9886613	2.0007999	102.8499985
-5.9886613	2.0495999	104.7099991
-5.9886613	2.0983999	106.6199951
-5.9886613	2.1471999	105.6100006
-5.9886613	2.1959999	103.7600021
-5.9886613	2.2447999	103.4700012
-5.9886613	2.2935998	103.7200012
-5.9886613	2.3423998	101.3099976
-5.9886613	2.3912001	106.3899994
-5.9886613	2.4400001	102.8600006
-5.9886613	2.4888000	102.9700012
-5.9886613	2.5376000	105.0199966
-5.9886613	2.5864000	106.5999985
-5.9886613	2.6352000	105.3300018
-5.9886613	2.6840000	106.1699982
-5.9886613	2.7328000	102.2099991
-5.9886613	2.7816000	104.5999985
-5.9886613	2.8304000	104.4700012
-5.9886613	2.8792000	104.2900009
-5.9886613	2.9280000	102.1800003
-5.9886613	2.9768000	103.1299973
-5.9886613	3.0256000	105.3899994
-5.9886613	3.0743999	104.8600006
-5.9886613	3.1231999	104.3300018
-5.9886613	3.1719999	108.7699966
-5.9886613	3.2207999	106.4499969
-5.9886613	3.2695999	99.6599960
-5.9886613	3.3183999	105.9599991

-5.9886613	3.3671999	106.7999954
-5.9886613	3.4159999	103.0199966
-5.9886613	3.4647999	102.2699966
-5.9886613	3.5135999	101.3799973
-5.9886613	3.5623999	96.5599976
-5.9886613	3.6111999	99.6599960
-5.9886613	3.6599998	100.8699951
-5.9886613	3.7087998	103.5599976
-5.9886613	3.7575998	106.3799973
-5.9886613	3.8063998	106.8099976
-5.9886613	3.8551998	107.6999969
-5.9886613	3.9039998	107.5800018
-5.9886613	3.9527998	106.1500015
-5.9886613	4.0016003	102.4700012
-5.9886613	4.0503998	102.3099976
-5.9886613	4.0991998	103.8899994
-5.9886613	4.1479998	104.0299988
-5.9886613	4.1967998	102.9700012
-5.9886613	4.2455997	102.4099960
-5.9886613	4.2943997	107.8099976
-5.9886613	4.3431997	109.5800018
-5.9886613	4.3919997	104.7699966
-5.9886613	4.4407997	105.2299957
-5.9886613	4.4895997	109.5699997
-5.9886613	4.5383997	115.5199966
-5.9886613	4.5871997	116.4099960
-5.9886613	4.6359997	117.1999969
-5.9886613	4.6847997	118.1599960
-5.9886613	4.7336001	116.1299973
-5.9886613	4.7824001	111.0599976
-5.9886613	4.8312001	109.2699966
-5.9886613	4.8800001	112.5400009
-5.9886613	4.9288001	112.6100006
-5.9886613	4.9776001	107.0800018
-5.9886613	5.0264001	105.4899979
-5.9886613	5.0752001	107.1899948
-5.9886613	5.1240001	107.6699982
-5.9886613	5.1728001	106.1599960
-5.9886613	5.2216001	104.2099991
-5.9886613	5.2704000	105.3399963
-5.9886613	5.3192000	101.3600006
-5.9886613	5.3680000	103.7900009
-5.9886613	5.4168000	102.7200012
-5.9886613	5.4656000	104.7999954
-5.9886613	5.5144000	107.4499969
-5.9886613	5.5632000	108.2200012
-5.9886613	5.6120000	107.2999954
-5.9886613	5.6608000	107.4099960
-5.9886613	5.7096000	107.6100006
-5.9886613	5.7584000	105.3300018
-5.9886613	5.8072000	101.6999969
-5.9886613	5.8559999	104.1100006
-5.9886613	5.9047999	106.1800003
-5.9886613	5.9535999	108.0400009
-5.9886613	6.0023999	107.1999969
-5.9886613	6.0511999	105.5599976
-5.9886613	6.0999999	105.6699982
-5.9886613	6.1487999	102.4899979

-5.9886613	6.1975999	102.6999969
-5.9886613	6.2463999	101.2900009
-5.9886613	6.2951999	103.2399979
-5.9886613	6.3439999	104.4000015
-5.9886613	6.3927999	103.5699997
-5.9886613	6.4415998	103.8199997
-5.9886613	6.4903998	105.5499954
-5.9886613	6.5391998	99.2200012
-5.9886613	6.5879998	101.9199982
-5.9886613	6.6367998	102.1500015
-5.9886613	6.6855998	105.9399948
-5.9886613	6.7343998	105.8799973
-5.9886613	6.7831998	104.5000000
-5.9886613	6.8319998	102.9000015
-5.9886613	6.8807998	97.9899979
-5.9886613	6.9295998	101.6199951
-5.9886613	6.9783998	105.2099991
-5.9886613	7.0271997	103.0699997
-5.9886613	7.0759997	100.5299988
-5.9886613	7.1247997	100.1500015
-5.9886613	7.1735997	101.9899979
-5.9886613	7.2223997	101.7500000
-5.9886613	7.2711997	100.8899994
-5.9886613	7.3199997	101.7900009
-5.9886613	7.3687997	103.2099991
-5.9886613	7.4175997	102.9700012
-5.9886613	7.4663997	101.6699982
-5.9886613	7.5151997	104.2399979
-5.9886613	7.5639997	105.6100006
-5.9886613	7.6127996	105.2399979
-5.9886613	7.6615996	107.8600006
-5.9886613	7.7103996	106.9799957
-5.9886613	7.7591996	105.4000015
-5.9886613	7.8079996	104.0199966
-5.9886613	7.8567996	101.2200012
-5.9886613	7.9056001	100.3899994
-5.9886613	7.9544001	103.0999985
-5.9886613	8.0032005	102.8300018
-5.9886613	8.0520000	106.6299973
-5.9886613	8.1008005	104.4300003
-5.9886613	8.1496000	104.7299957
-5.9886613	8.1984005	102.4599991
-5.9886613	8.2472000	102.7099991
-5.9886613	8.2960005	101.7699966
-5.9886613	8.3448000	103.6299973
-5.9886613	8.3936005	105.9700012
-5.9886613	8.4424000	104.6100006
-5.9886613	8.4912004	105.2099991
-5.9886613	8.5400000	104.9799957
-5.9886613	8.5888004	103.1399994
-5.9886613	8.6375999	103.2900009
-5.9886613	8.6864004	103.4199982
-5.9886613	8.7351999	103.1899948
-5.9886613	8.7840004	102.9300003
-5.9886613	8.8327999	101.3699951
-5.9886613	8.8816004	102.8099976
-5.9886613	8.9303999	104.5599976
-5.9886613	8.9792004	104.8600006

-5.9886613	9.0279999	105.4199982
-5.9886613	9.0768003	105.5499954
-5.9886613	9.1255999	104.1800003
-5.9886613	9.1744003	103.6899948
-5.9886613	9.2231998	103.4700012
-5.9886613	9.2720003	102.4199982
-5.9886613	9.3207998	102.8899994
-5.9886613	9.3696003	103.4300003
-5.9886613	9.4184008	105.7500000
-5.9886613	9.4672003	105.7600021
-5.9886613	9.5160007	104.6299973
-5.9886613	9.5648003	102.2699966
-5.9886613	9.6136007	103.9300003
-5.9886613	9.6624002	102.9899979
-5.9886613	9.7112007	96.1800003
-5.9886613	9.7600002	101.8300018
-5.9886613	9.8088007	103.6500015
-5.9886613	9.8576002	101.1999969
-5.9886613	9.9064007	103.0199966
-5.9886613	9.9552002	104.7399979
-5.9886613	10.0040007	105.2099991
-5.9886613	10.0528002	102.6199951
-5.9886613	10.1016006	103.6899948
-5.9886613	10.1504002	103.8899994
-5.9886613	10.1992006	102.0699997
-5.9886613	10.2480001	103.8199997
-5.9886613	10.2968006	102.6599960
-5.9886613	10.3456001	103.0899963
-5.9886613	10.3944006	101.5199966
-5.9886613	10.4432001	103.5599976
-5.9886613	10.4920006	103.7999954
-5.9886613	10.5408001	101.4099960
-5.9886613	10.5896006	102.7799988
-5.9886613	10.6384001	103.9099960
-5.9886613	10.6872005	99.6299973
-5.9886613	10.7360001	100.7900009
-5.9886613	10.7848005	104.5699997
-5.9886613	10.8336000	103.9199982
-5.9886613	10.8824005	102.4700012
-5.9886613	10.9312000	102.5499954
-5.9886613	10.9800005	99.8600006
-5.9886613	11.0288000	103.3499985
-5.9886613	11.0776005	104.8600006
-5.9886613	11.1264000	98.9399948
-5.9886613	11.1752005	104.0800018
-5.9886613	11.2240000	101.6399994
-5.9886613	11.2728004	104.7099991
-5.9886613	11.3216000	103.2399979
-5.9886613	11.3704004	104.5999985
-5.9886613	11.4191999	105.2200012
-5.9886613	11.4680004	102.0000000
-5.9886613	11.5167999	101.7200012
-5.9886613	11.5656004	103.6999969
-5.9886613	11.6143999	104.8699951
-5.9886613	11.6632004	99.8499985
-5.9886613	11.7119999	103.2299957
-5.9886613	11.7608004	103.7900009
-5.9886613	11.8095999	99.6599960

-5.9886613	11.8584003	105.2600021
-5.9886613	11.9071999	106.0699997
-5.9886613	11.9560003	101.1100006
-5.9886613	12.0047998	107.1199951
-5.9886613	12.0536003	106.5999985
-5.9886613	12.1023998	104.3399963
-5.9886613	12.1512003	98.5199966
-5.9886613	12.1999998	102.5100021
-5.9886613	12.2488003	105.6100006
-5.9886613	12.2975998	105.1299973
-5.9886613	12.3464003	104.4199982
-5.9886613	12.3951998	103.8099976
-5.9886613	12.4440002	99.0199966
-5.9886613	12.4927998	102.5299988
-5.9886613	12.5416002	103.9799957
-5.9886613	12.5904007	103.7299957
-5.9886613	12.6392002	100.3099976
-5.9886613	12.6880007	99.8799973
-5.9886613	12.7368002	100.9199982
-5.9886613	12.7856007	103.0299988
-5.9886613	12.8344002	104.8399963
-5.9886613	12.8832006	103.7799988
-5.9886613	12.9320002	103.0000000
-5.9886613	12.9808006	101.6500015
-5.9886613	13.0296001	102.6999969
-5.9886613	13.0784006	98.5599976
-5.9886613	13.1272001	101.7999954
-5.9886613	13.1760006	102.1399994
-5.9886613	13.2248001	98.4899979
-5.9886613	13.2736006	103.0400009
-5.9886613	13.3224001	103.1999969
-5.9886613	13.3712006	101.3199997
-5.9886613	13.4200001	100.8099976
-5.9886613	13.4688005	101.6999969
-5.9886613	13.5176001	101.1599960
-5.9886613	13.5664005	104.1599960
-5.9886613	13.6152000	103.3600006
-5.9886613	13.6640005	99.6399994
-5.9886613	13.7128000	96.6199951
-5.9886613	13.7616005	98.6899948
-5.9886613	13.8104000	100.2500000
-5.9886613	13.8592005	104.5499954
-5.9886613	13.9080000	105.2500000
-5.9886613	13.9568005	103.7099991
-5.9886613	14.0056000	101.5800018
-5.9886613	14.0544004	100.9499969
-5.9886613	14.1032000	101.7600021
-5.9886613	14.1520004	102.0000000
-5.9886613	14.2007999	102.4899979
-5.9886613	14.2496004	105.1899948
-5.9886613	14.2983999	105.2299957
-5.9886613	14.3472004	104.6500015
-5.9886613	14.3959999	101.9599991
-5.9886613	14.4448004	104.4399948
-5.9886613	14.4935999	102.8600006
-5.9886613	14.5424004	100.6500015
-5.9886613	14.5911999	101.9599991
-5.9886613	14.6400003	95.9799957

-5.9886613	14.6887999	98.9700012
-5.9886613	14.7376003	101.1699982
-5.9886613	14.7863998	101.3399963
-5.9886613	14.8352003	104.1999969
-5.9886613	14.8839998	104.7900009
-5.9886613	14.9328003	104.1500015
-5.9886613	14.9815998	102.5599976
-5.9886613	15.0304003	102.8899994
-5.9825253	0.3416000	106.4399948
-5.9825253	0.3904000	104.8899994
-5.9825253	0.4392000	104.8099976
-5.9825253	0.4880000	103.0800018
-5.9825253	0.5368000	104.4000015
-5.9825253	0.5856000	99.6299973
-5.9825253	0.6344000	102.1500015
-5.9825253	0.6832000	102.7399979
-5.9825253	0.7320000	107.0299988
-5.9825253	0.7808000	108.6100006
-5.9825253	0.8296000	107.4599991
-5.9825253	0.8784000	106.0999985
-5.9825253	0.9272000	105.8899994
-5.9825253	0.9760000	109.5999985
-5.9825253	1.0247999	108.9300003
-5.9825253	1.0735999	105.5299988
-5.9825253	1.1223999	106.6199951
-5.9825253	1.1711999	107.3600006
-5.9825253	1.2200000	107.3199997
-5.9825253	1.2688000	107.2399979
-5.9825253	1.3176000	106.4099960
-5.9825253	1.3664000	107.2900009
-5.9825253	1.4152000	107.8899994
-5.9825253	1.4640000	107.5400009
-5.9825253	1.5128000	109.1199951
-5.9825253	1.5616000	106.2999954
-5.9825253	1.6104000	109.0999985
-5.9825253	1.6592000	109.3099976
-5.9825253	1.7079999	105.3499985
-5.9825253	1.7567999	106.1500015
-5.9825253	1.8055999	110.2999954
-5.9825253	1.8543999	116.2900009
-5.9825253	1.9031999	117.5999985
-5.9825253	1.9519999	114.7600021
-5.9825253	2.0007999	103.8300018
-5.9825253	2.0495999	104.4300003
-5.9825253	2.0983999	106.3099976
-5.9825253	2.1471999	105.1299973
-5.9825253	2.1959999	103.0400009
-5.9825253	2.2447999	103.5400009
-5.9825253	2.2935998	102.4300003
-5.9825253	2.3423998	99.3399963
-5.9825253	2.3912001	106.2399979
-5.9825253	2.4400001	103.7699966
-5.9825253	2.4888000	104.1100006
-5.9825253	2.5376000	104.4899979
-5.9825253	2.5864000	105.5599976
-5.9825253	2.6352000	104.5100021
-5.9825253	2.6840000	105.3899994
-5.9825253	2.7328000	102.3899994

-5.9825253	2.7816000	105.0599976
-5.9825253	2.8304000	106.1199951
-5.9825253	2.8792000	105.3799973
-5.9825253	2.9280000	103.5000000
-5.9825253	2.9768000	102.6500015
-5.9825253	3.0256000	104.6599960
-5.9825253	3.0743999	105.1599960
-5.9825253	3.1231999	106.3799973
-5.9825253	3.1719999	108.2999954
-5.9825253	3.2207999	106.5599976
-5.9825253	3.2695999	100.7799988
-5.9825253	3.3183999	106.5400009
-5.9825253	3.3671999	107.1800003
-5.9825253	3.4159999	103.5800018
-5.9825253	3.4647999	100.6699982
-5.9825253	3.5135999	101.4399948
-5.9825253	3.5623999	97.7900009
-5.9825253	3.6111999	100.2900009
-5.9825253	3.6599998	101.5999985
-5.9825253	3.7087998	104.1100006
-5.9825253	3.7575998	106.3199997
-5.9825253	3.8063998	107.1599960
-5.9825253	3.8551998	107.8099976
-5.9825253	3.9039998	107.6800003
-5.9825253	3.9527998	105.6800003
-5.9825253	4.0016003	100.4399948
-5.9825253	4.0503998	102.1899948
-5.9825253	4.0991998	104.8600006
-5.9825253	4.1479998	104.0699997
-5.9825253	4.1967998	102.0899963
-5.9825253	4.2455997	102.3899994
-5.9825253	4.2943997	107.6899948
-5.9825253	4.3431997	109.6100006
-5.9825253	4.3919997	105.8099976
-5.9825253	4.4407997	105.3699951
-5.9825253	4.4895997	108.4399948
-5.9825253	4.5383997	114.8699951
-5.9825253	4.5871997	116.9300003
-5.9825253	4.6359997	116.4799957
-5.9825253	4.6847997	117.2099991
-5.9825253	4.7336001	115.3199997
-5.9825253	4.7824001	109.9199982
-5.9825253	4.8312001	107.8300018
-5.9825253	4.8800001	112.6500015
-5.9825253	4.9288001	112.5599976
-5.9825253	4.9776001	107.2500000
-5.9825253	5.0264001	104.7900009
-5.9825253	5.0752001	106.8199997
-5.9825253	5.1240001	107.3499985
-5.9825253	5.1728001	105.7099991
-5.9825253	5.2216001	104.7200012
-5.9825253	5.2704000	105.0800018
-5.9825253	5.3192000	101.9099960
-5.9825253	5.3680000	104.3099976
-5.9825253	5.4168000	102.1100006
-5.9825253	5.4656000	104.2699966
-5.9825253	5.5144000	106.9799957
-5.9825253	5.5632000	108.1399994

-5.9825253	5.6120000	107.4099960
-5.9825253	5.6608000	107.8399963
-5.9825253	5.7096000	107.5400009
-5.9825253	5.7584000	104.6500015
-5.9825253	5.8072000	99.7500000
-5.9825253	5.8559999	103.6199951
-5.9825253	5.9047999	105.5000000
-5.9825253	5.9535999	107.0199966
-5.9825253	6.0023999	106.8099976
-5.9825253	6.0511999	105.7500000
-5.9825253	6.0999999	105.7399979
-5.9825253	6.1487999	101.5800018
-5.9825253	6.1975999	101.4700012
-5.9825253	6.2463999	101.5299988
-5.9825253	6.2951999	103.3399963
-5.9825253	6.3439999	104.5999985
-5.9825253	6.3927999	103.4899979
-5.9825253	6.4415998	104.1500015
-5.9825253	6.4903998	105.6299973
-5.9825253	6.5391998	99.1100006
-5.9825253	6.5879998	102.7799988
-5.9825253	6.6367998	101.9300003
-5.9825253	6.6855998	106.1100006
-5.9825253	6.7343998	105.6199951
-5.9825253	6.7831998	104.5100021
-5.9825253	6.8319998	102.8399963
-5.9825253	6.8807998	96.8699951
-5.9825253	6.9295998	100.4399948
-5.9825253	6.9783998	105.0000000
-5.9825253	7.0271997	103.5999985
-5.9825253	7.0759997	100.2500000
-5.9825253	7.1247997	100.1299973
-5.9825253	7.1735997	102.0499954
-5.9825253	7.2223997	102.1800003
-5.9825253	7.2711997	100.4399948
-5.9825253	7.3199997	103.0499954
-5.9825253	7.3687997	102.7500000
-5.9825253	7.4175997	103.8899994
-5.9825253	7.4663997	103.4199982
-5.9825253	7.5151997	104.8300018
-5.9825253	7.5639997	105.1800003
-5.9825253	7.6127996	104.3699951
-5.9825253	7.6615996	108.0599976
-5.9825253	7.7103996	106.8499985
-5.9825253	7.7591996	104.7699966
-5.9825253	7.8079996	103.3099976
-5.9825253	7.8567996	99.7999954
-5.9825253	7.9056001	99.1999969
-5.9825253	7.9544001	102.6399994
-5.9825253	8.0032005	103.5999985
-5.9825253	8.0520000	107.4099960
-5.9825253	8.1008005	104.7799988
-5.9825253	8.1496000	104.6199951
-5.9825253	8.1984005	102.6800003
-5.9825253	8.2472000	103.2500000
-5.9825253	8.2960005	102.7399979
-5.9825253	8.3448000	103.1699982
-5.9825253	8.3936005	106.2999954

-5.9825253	8.4424000	105.9099960
-5.9825253	8.4912004	105.4700012
-5.9825253	8.5400000	104.5400009
-5.9825253	8.5888004	102.4599991
-5.9825253	8.6375999	103.5100021
-5.9825253	8.6864004	103.6500015
-5.9825253	8.7351999	103.4799957
-5.9825253	8.7840004	103.3899994
-5.9825253	8.8327999	102.4300003
-5.9825253	8.8816004	103.2399979
-5.9825253	8.9303999	103.2699966
-5.9825253	8.9792004	103.6100006
-5.9825253	9.0279999	103.8699951
-5.9825253	9.0768003	104.8399963
-5.9825253	9.1255999	104.6699982
-5.9825253	9.1744003	103.5299988
-5.9825253	9.2231998	103.1199951
-5.9825253	9.2720003	101.9499969
-5.9825253	9.3207998	102.0699997
-5.9825253	9.3696003	104.0800018
-5.9825253	9.4184008	105.3799973
-5.9825253	9.4672003	105.1699982
-5.9825253	9.5160007	104.8799973
-5.9825253	9.5648003	102.6299973
-5.9825253	9.6136007	103.4700012
-5.9825253	9.6624002	102.5299988
-5.9825253	9.7112007	97.1800003
-5.9825253	9.7600002	103.2299957
-5.9825253	9.8088007	104.3699951
-5.9825253	9.8576002	99.5800018
-5.9825253	9.9064007	103.1899948
-5.9825253	9.9552002	105.7699966
-5.9825253	10.0040007	105.9799957
-5.9825253	10.0528002	104.0899963
-5.9825253	10.1016006	104.0000000
-5.9825253	10.1504002	103.5800018
-5.9825253	10.1992006	100.8699951
-5.9825253	10.2480001	103.7500000
-5.9825253	10.2968006	103.1399994
-5.9825253	10.3456001	103.1699982
-5.9825253	10.3944006	102.0199966
-5.9825253	10.4432001	103.7699966
-5.9825253	10.4920006	102.9799957
-5.9825253	10.5408001	100.5400009
-5.9825253	10.5896006	102.4300003
-5.9825253	10.6384001	104.3600006
-5.9825253	10.6872005	101.3300018
-5.9825253	10.7360001	102.3300018
-5.9825253	10.7848005	104.4199982
-5.9825253	10.8336000	103.1100006
-5.9825253	10.8824005	101.2500000
-5.9825253	10.9312000	102.0499954
-5.9825253	10.9800005	100.9799957
-5.9825253	11.0288000	103.7500000
-5.9825253	11.0776005	104.7900009
-5.9825253	11.1264000	99.7500000
-5.9825253	11.1752005	105.0699997
-5.9825253	11.2240000	102.6899948

-5.9825253	11.2728004	105.1100006
-5.9825253	11.3216000	104.7399979
-5.9825253	11.3704004	105.1100006
-5.9825253	11.4191999	104.9700012
-5.9825253	11.4680004	100.9899979
-5.9825253	11.5167999	101.5800018
-5.9825253	11.5656004	103.6800003
-5.9825253	11.6143999	104.7999954
-5.9825253	11.6632004	101.0599976
-5.9825253	11.7119999	102.8499985
-5.9825253	11.7608004	104.1100006
-5.9825253	11.8095999	99.8199997
-5.9825253	11.8584003	104.9899979
-5.9825253	11.9071999	105.9599991
-5.9825253	11.9560003	102.0499954
-5.9825253	12.0047998	106.3499985
-5.9825253	12.0536003	105.0599976
-5.9825253	12.1023998	101.5299988
-5.9825253	12.1512003	98.0499954
-5.9825253	12.1999998	103.0299988
-5.9825253	12.2488003	106.0800018
-5.9825253	12.2975998	105.6699982
-5.9825253	12.3464003	105.3899994
-5.9825253	12.3951998	104.5400009
-5.9825253	12.4440002	98.9199982
-5.9825253	12.4927998	101.4000015
-5.9825253	12.5416002	103.1800003
-5.9825253	12.5904007	104.4399948
-5.9825253	12.6392002	100.4099960
-5.9825253	12.6880007	100.5699997
-5.9825253	12.7368002	100.8399963
-5.9825253	12.7856007	103.0800018
-5.9825253	12.8344002	103.6100006
-5.9825253	12.8832006	102.9799957
-5.9825253	12.9320002	102.6999969
-5.9825253	12.9808006	101.6999969
-5.9825253	13.0296001	103.3300018
-5.9825253	13.0784006	99.1899948
-5.9825253	13.1272001	102.3399963
-5.9825253	13.1760006	103.2099991
-5.9825253	13.2248001	100.5699997
-5.9825253	13.2736006	104.1699982
-5.9825253	13.3224001	103.6800003
-5.9825253	13.3712006	101.8300018
-5.9825253	13.4200001	100.2299957
-5.9825253	13.4688005	102.3300018
-5.9825253	13.5176001	101.6299973
-5.9825253	13.5664005	104.3099976
-5.9825253	13.6152000	103.4499969
-5.9825253	13.6640005	99.8499985
-5.9825253	13.7128000	97.5699997
-5.9825253	13.7616005	99.1699982
-5.9825253	13.8104000	100.5599976
-5.9825253	13.8592005	104.8399963
-5.9825253	13.9080000	105.8899994
-5.9825253	13.9568005	104.7999954
-5.9825253	14.0056000	103.5499954
-5.9825253	14.0544004	101.7500000

-5.9825253	14.1032000	101.9899979
-5.9825253	14.1520004	101.4599991
-5.9825253	14.2007999	102.8600006
-5.9825253	14.2496004	105.3600006
-5.9825253	14.2983999	104.9399948
-5.9825253	14.3472004	104.4700012
-5.9825253	14.3959999	102.6399994
-5.9825253	14.4448004	104.5000000
-5.9825253	14.4935999	103.3300018
-5.9825253	14.5424004	100.9300003
-5.9825253	14.5911999	102.4199982
-5.9825253	14.6400003	96.7299957
-5.9825253	14.6887999	99.0800018
-5.9825253	14.7376003	101.7900009
-5.9825253	14.7863998	102.3099976
-5.9825253	14.8352003	105.2500000
-5.9825253	14.8839998	105.5999985
-5.9825253	14.9328003	104.9599991
-5.9825253	14.9815998	103.1500015
-5.9825253	15.0304003	102.8300018
-5.9763894	0.3416000	105.3399963
-5.9763894	0.3904000	103.9399948
-5.9763894	0.4392000	104.1199951
-5.9763894	0.4880000	103.9599991
-5.9763894	0.5368000	104.1999969
-5.9763894	0.5856000	98.4700012
-5.9763894	0.6344000	101.9700012
-5.9763894	0.6832000	102.2200012
-5.9763894	0.7320000	106.0400009
-5.9763894	0.7808000	108.0699997
-5.9763894	0.8296000	106.2699966
-5.9763894	0.8784000	104.6299973
-5.9763894	0.9272000	106.3399963
-5.9763894	0.9760000	109.9599991
-5.9763894	1.0247999	109.2500000
-5.9763894	1.0735999	106.0000000
-5.9763894	1.1223999	106.7900009
-5.9763894	1.1711999	106.5899963
-5.9763894	1.2200000	107.2799988
-5.9763894	1.2688000	107.3600006
-5.9763894	1.3176000	106.1699982
-5.9763894	1.3664000	107.4700012
-5.9763894	1.4152000	107.7600021
-5.9763894	1.4640000	108.5000000
-5.9763894	1.5128000	109.3099976
-5.9763894	1.5616000	106.0699997
-5.9763894	1.6104000	108.6699982
-5.9763894	1.6592000	108.5999985
-5.9763894	1.7079999	104.5299988
-5.9763894	1.7567999	105.7699966
-5.9763894	1.8055999	110.0800018
-5.9763894	1.8543999	116.5800018
-5.9763894	1.9031999	118.4199982
-5.9763894	1.9519999	115.0400009
-5.9763894	2.0007999	104.4399948
-5.9763894	2.0495999	104.5800018
-5.9763894	2.0983999	106.4799957
-5.9763894	2.1471999	104.9700012

-5.9763894	2.1959999	103.1299973
-5.9763894	2.2447999	103.9599991
-5.9763894	2.2935998	101.3699951
-5.9763894	2.3423998	97.6599960
-5.9763894	2.3912001	105.6699982
-5.9763894	2.4400001	103.9399948
-5.9763894	2.4888000	105.3399963
-5.9763894	2.5376000	104.5299988
-5.9763894	2.5864000	105.1100006
-5.9763894	2.6352000	103.6999969
-5.9763894	2.6840000	104.5000000
-5.9763894	2.7328000	103.5800018
-5.9763894	2.7816000	106.1500015
-5.9763894	2.8304000	107.3099976
-5.9763894	2.8792000	105.8399963
-5.9763894	2.9280000	104.1999969
-5.9763894	2.9768000	101.8199997
-5.9763894	3.0256000	103.9799957
-5.9763894	3.0743999	105.5400009
-5.9763894	3.1231999	107.1899948
-5.9763894	3.1719999	107.1899948
-5.9763894	3.2207999	106.1299973
-5.9763894	3.2695999	102.6199951
-5.9763894	3.3183999	107.0100021
-5.9763894	3.3671999	106.7799988
-5.9763894	3.4159999	103.5800018
-5.9763894	3.4647999	98.4899979
-5.9763894	3.5135999	100.8699951
-5.9763894	3.5623999	99.3499985
-5.9763894	3.6111999	101.8199997
-5.9763894	3.6599998	102.6599960
-5.9763894	3.7087998	104.3799973
-5.9763894	3.7575998	105.8799973
-5.9763894	3.8063998	107.5400009
-5.9763894	3.8551998	108.3199997
-5.9763894	3.9039998	107.5699997
-5.9763894	3.9527998	104.9799957
-5.9763894	4.0016003	98.6699982
-5.9763894	4.0503998	102.0000000
-5.9763894	4.0991998	105.5000000
-5.9763894	4.1479998	104.5599976
-5.9763894	4.1967998	101.9899979
-5.9763894	4.2455997	102.0499954
-5.9763894	4.2943997	107.1199951
-5.9763894	4.3431997	109.5800018
-5.9763894	4.3919997	107.0499954
-5.9763894	4.4407997	105.7699966
-5.9763894	4.4895997	108.1100006
-5.9763894	4.5383997	114.8600006
-5.9763894	4.5871997	117.9700012
-5.9763894	4.6359997	116.5999985
-5.9763894	4.6847997	116.2500000
-5.9763894	4.7336001	114.4199982
-5.9763894	4.7824001	108.6999969
-5.9763894	4.8312001	107.2900009
-5.9763894	4.8800001	112.5599976
-5.9763894	4.9288001	111.8699951
-5.9763894	4.9776001	106.8499985

-5.9763894	5.0264001	105.4300003
-5.9763894	5.0752001	107.7699966
-5.9763894	5.1240001	107.6199951
-5.9763894	5.1728001	105.2600021
-5.9763894	5.2216001	105.0299988
-5.9763894	5.2704000	105.0999985
-5.9763894	5.3192000	102.6899948
-5.9763894	5.3680000	104.6699982
-5.9763894	5.4168000	101.4300003
-5.9763894	5.4656000	103.1899948
-5.9763894	5.5144000	106.0899963
-5.9763894	5.5632000	107.9300003
-5.9763894	5.6120000	107.5899963
-5.9763894	5.6608000	108.2200012
-5.9763894	5.7096000	107.3300018
-5.9763894	5.7584000	104.0599976
-5.9763894	5.8072000	98.6899948
-5.9763894	5.8559999	103.4000015
-5.9763894	5.9047999	105.3300018
-5.9763894	5.9535999	106.4399948
-5.9763894	6.0023999	106.8399963
-5.9763894	6.0511999	106.3899994
-5.9763894	6.0999999	106.2699966
-5.9763894	6.1487999	102.0100021
-5.9763894	6.1975999	100.2600021
-5.9763894	6.2463999	101.6800003
-5.9763894	6.2951999	103.8899994
-5.9763894	6.3439999	104.5599976
-5.9763894	6.3927999	102.7399979
-5.9763894	6.4415998	104.1699982
-5.9763894	6.4903998	106.2500000
-5.9763894	6.5391998	100.6399994
-5.9763894	6.5879998	103.4599991
-5.9763894	6.6367998	101.4199982
-5.9763894	6.6855998	105.8699951
-5.9763894	6.7343998	104.9599991
-5.9763894	6.7831998	104.1699982
-5.9763894	6.8319998	102.8099976
-5.9763894	6.8807998	96.1199951
-5.9763894	6.9295998	100.2200012
-5.9763894	6.9783998	104.6800003
-5.9763894	7.0271997	103.8199997
-5.9763894	7.0759997	100.3799973
-5.9763894	7.1247997	100.2999954
-5.9763894	7.1735997	102.3300018
-5.9763894	7.2223997	103.0299988
-5.9763894	7.2711997	100.5100021
-5.9763894	7.3199997	103.3699951
-5.9763894	7.3687997	102.7699966
-5.9763894	7.4175997	104.6199951
-5.9763894	7.4663997	103.9199982
-5.9763894	7.5151997	104.7299957
-5.9763894	7.5639997	105.0599976
-5.9763894	7.6127996	103.0999985
-5.9763894	7.6615996	107.9899979
-5.9763894	7.7103996	106.8600006
-5.9763894	7.7591996	104.3799973
-5.9763894	7.8079996	103.3399963

-5.9763894	7.8567996	100.5899963
-5.9763894	7.9056001	100.4199982
-5.9763894	7.9544001	103.2999954
-5.9763894	8.0032005	104.8300018
-5.9763894	8.0520000	107.9599991
-5.9763894	8.1008005	105.1500015
-5.9763894	8.1496000	104.6100006
-5.9763894	8.1984005	103.2799988
-5.9763894	8.2472000	104.3199997
-5.9763894	8.2960005	103.9799957
-5.9763894	8.3448000	102.6599960
-5.9763894	8.3936005	106.2799988
-5.9763894	8.4424000	106.3699951
-5.9763894	8.4912004	104.5499954
-5.9763894	8.5400000	104.3899994
-5.9763894	8.5888004	101.9399948
-5.9763894	8.6375999	103.0800018
-5.9763894	8.6864004	103.4499969
-5.9763894	8.7351999	103.8199997
-5.9763894	8.7840004	103.9000015
-5.9763894	8.8327999	103.6699982
-5.9763894	8.8816004	104.1399994
-5.9763894	8.9303999	102.5100021
-5.9763894	8.9792004	102.6500015
-5.9763894	9.0279999	102.7200012
-5.9763894	9.0768003	104.5400009
-5.9763894	9.1255999	105.1100006
-5.9763894	9.1744003	103.2500000
-5.9763894	9.2231998	102.9700012
-5.9763894	9.2720003	101.5299988
-5.9763894	9.3207998	101.1299973
-5.9763894	9.3696003	104.1199951
-5.9763894	9.4184008	104.4599991
-5.9763894	9.4672003	103.7699966
-5.9763894	9.5160007	104.8899994
-5.9763894	9.5648003	103.4700012
-5.9763894	9.6136007	102.9799957
-5.9763894	9.6624002	101.9099960
-5.9763894	9.7112007	99.6100006
-5.9763894	9.7600002	104.9000015
-5.9763894	9.8088007	105.3499985
-5.9763894	9.8576002	98.3499985
-5.9763894	9.9064007	103.3600006
-5.9763894	9.9552002	106.1500015
-5.9763894	10.0040007	106.0299988
-5.9763894	10.0528002	105.4199982
-5.9763894	10.1016006	104.4799957
-5.9763894	10.1504002	103.7799988
-5.9763894	10.1992006	99.9099960
-5.9763894	10.2480001	103.5299988
-5.9763894	10.2968006	103.4199982
-5.9763894	10.3456001	102.8699951
-5.9763894	10.3944006	101.8499985
-5.9763894	10.4432001	104.0899963
-5.9763894	10.4920006	103.1599960
-5.9763894	10.5408001	100.2799988
-5.9763894	10.5896006	101.9700012
-5.9763894	10.6384001	104.8099976

-5.9763894	10.6872005	103.0699997
-5.9763894	10.7360001	103.5299988
-5.9763894	10.7848005	104.2500000
-5.9763894	10.8336000	101.8699951
-5.9763894	10.8824005	99.8099976
-5.9763894	10.9312000	101.1100006
-5.9763894	10.9800005	101.6500015
-5.9763894	11.0288000	103.7099991
-5.9763894	11.0776005	104.0400009
-5.9763894	11.1264000	101.5199966
-5.9763894	11.1752005	106.2600021
-5.9763894	11.2240000	103.9099960
-5.9763894	11.2728004	105.6599960
-5.9763894	11.3216000	106.1299973
-5.9763894	11.3704004	105.3799973
-5.9763894	11.4191999	104.6299973
-5.9763894	11.4680004	100.3799973
-5.9763894	11.5167999	102.0400009
-5.9763894	11.5656004	104.0100021
-5.9763894	11.6143999	104.7500000
-5.9763894	11.6632004	102.0699997
-5.9763894	11.7119999	102.5599976
-5.9763894	11.7608004	104.1100006
-5.9763894	11.8095999	99.7799988
-5.9763894	11.8584003	104.7399979
-5.9763894	11.9071999	105.8600006
-5.9763894	11.9560003	102.6899948
-5.9763894	12.0047998	104.9700012
-5.9763894	12.0536003	102.7699966
-5.9763894	12.1023998	98.4799957
-5.9763894	12.1512003	97.6100006
-5.9763894	12.1999998	103.5100021
-5.9763894	12.2488003	106.1999969
-5.9763894	12.2975998	105.6399994
-5.9763894	12.3464003	105.8699951
-5.9763894	12.3951998	104.9099960
-5.9763894	12.4440002	98.5499954
-5.9763894	12.4927998	100.4399948
-5.9763894	12.5416002	102.0199966
-5.9763894	12.5904007	104.7500000
-5.9763894	12.6392002	101.0000000
-5.9763894	12.6880007	101.3600006
-5.9763894	12.7368002	101.1500015
-5.9763894	12.7856007	103.5499954
-5.9763894	12.8344002	102.7699966
-5.9763894	12.8832006	102.5000000
-5.9763894	12.9320002	103.1500015
-5.9763894	12.9808006	102.4499969
-5.9763894	13.0296001	103.2399979
-5.9763894	13.0784006	101.0299988
-5.9763894	13.1272001	103.4599991
-5.9763894	13.1760006	104.1899948
-5.9763894	13.2248001	100.9199982
-5.9763894	13.2736006	103.9000015
-5.9763894	13.3224001	103.7299957
-5.9763894	13.3712006	102.2799988
-5.9763894	13.4200001	99.5199966
-5.9763894	13.4688005	102.5199966

-5.9763894	13.5176001	102.3399963
-5.9763894	13.5664005	104.2799988
-5.9763894	13.6152000	103.9000015
-5.9763894	13.6640005	100.8799973
-5.9763894	13.7128000	99.2399979
-5.9763894	13.7616005	99.5499954
-5.9763894	13.8104000	100.7799988
-5.9763894	13.8592005	104.8300018
-5.9763894	13.9080000	105.6399994
-5.9763894	13.9568005	105.4300003
-5.9763894	14.0056000	105.0100021
-5.9763894	14.0544004	102.6299973
-5.9763894	14.1032000	101.9499969
-5.9763894	14.1520004	101.2200012
-5.9763894	14.2007999	103.3600006
-5.9763894	14.2496004	105.0800018
-5.9763894	14.2983999	103.9099960
-5.9763894	14.3472004	103.3799973
-5.9763894	14.3959999	102.3199997
-5.9763894	14.4448004	104.1899948
-5.9763894	14.4935999	103.7200012
-5.9763894	14.5424004	101.4000015
-5.9763894	14.5911999	103.5000000
-5.9763894	14.6400003	98.6299973
-5.9763894	14.6887999	99.7200012
-5.9763894	14.7376003	102.6599960
-5.9763894	14.7863998	102.8899994
-5.9763894	14.8352003	105.9000015
-5.9763894	14.8839998	106.2900009
-5.9763894	14.9328003	106.0899963
-5.9763894	14.9815998	104.1699982
-5.9763894	15.0304003	102.9399948
-5.9702535	0.3416000	105.0899963
-5.9702535	0.3904000	103.7299957
-5.9702535	0.4392000	103.9799957
-5.9702535	0.4880000	105.1699982
-5.9702535	0.5368000	104.0100021
-5.9702535	0.5856000	96.9599991
-5.9702535	0.6344000	101.4399948
-5.9702535	0.6832000	102.1199951
-5.9702535	0.7320000	105.0899963
-5.9702535	0.7808000	107.5199966
-5.9702535	0.8296000	105.9000015
-5.9702535	0.8784000	104.2099991
-5.9702535	0.9272000	105.9899979
-5.9702535	0.9760000	108.8399963
-5.9702535	1.0247999	109.5899963
-5.9702535	1.0735999	107.2099991
-5.9702535	1.1223999	106.9399948
-5.9702535	1.1711999	105.9899979
-5.9702535	1.2200000	107.2099991
-5.9702535	1.2688000	107.0800018
-5.9702535	1.3176000	105.7699966
-5.9702535	1.3664000	107.5800018
-5.9702535	1.4152000	108.3699951
-5.9702535	1.4640000	109.3300018
-5.9702535	1.5128000	109.1500015
-5.9702535	1.5616000	106.5699997

-5.9702535	1.6104000	108.8699951
-5.9702535	1.6592000	107.6599960
-5.9702535	1.7079999	103.5999985
-5.9702535	1.7567999	105.0999985
-5.9702535	1.8055999	110.1199951
-5.9702535	1.8543999	116.8600006
-5.9702535	1.9031999	119.1599960
-5.9702535	1.9519999	115.6699982
-5.9702535	2.0007999	105.2900009
-5.9702535	2.0495999	104.5999985
-5.9702535	2.0983999	106.7600021
-5.9702535	2.1471999	105.2600021
-5.9702535	2.1959999	103.3099976
-5.9702535	2.2447999	104.2399979
-5.9702535	2.2935998	100.3199997
-5.9702535	2.3423998	96.5699997
-5.9702535	2.3912001	105.0999985
-5.9702535	2.4400001	103.7999954
-5.9702535	2.4888000	106.5000000
-5.9702535	2.5376000	105.0800018
-5.9702535	2.5864000	104.6800003
-5.9702535	2.6352000	103.6999969
-5.9702535	2.6840000	103.4599991
-5.9702535	2.7328000	104.3499985
-5.9702535	2.7816000	106.6399994
-5.9702535	2.8304000	108.2299957
-5.9702535	2.8792000	106.1500015
-5.9702535	2.9280000	105.3099976
-5.9702535	2.9768000	101.4599991
-5.9702535	3.0256000	104.2600021
-5.9702535	3.0743999	105.4099960
-5.9702535	3.1231999	106.9099960
-5.9702535	3.1719999	105.5299988
-5.9702535	3.2207999	104.9799957
-5.9702535	3.2695999	103.8099976
-5.9702535	3.3183999	107.2099991
-5.9702535	3.3671999	106.1899948
-5.9702535	3.4159999	103.7999954
-5.9702535	3.4647999	97.1199951
-5.9702535	3.5135999	100.2500000
-5.9702535	3.5623999	100.6399994
-5.9702535	3.6111999	102.6100006
-5.9702535	3.6599998	103.5000000
-5.9702535	3.7087998	104.6800003
-5.9702535	3.7575998	105.6599960
-5.9702535	3.8063998	107.2600021
-5.9702535	3.8551998	108.6399994
-5.9702535	3.9039998	107.5299988
-5.9702535	3.9527998	104.5100021
-5.9702535	4.0016003	97.4599991
-5.9702535	4.0503998	102.5599976
-5.9702535	4.0991998	106.2799988
-5.9702535	4.1479998	105.0100021
-5.9702535	4.1967998	102.0800018
-5.9702535	4.2455997	102.6199951
-5.9702535	4.2943997	107.1399994
-5.9702535	4.3431997	109.8799973
-5.9702535	4.3919997	108.4599991

-5.9702535	4.4407997	106.1800003
-5.9702535	4.4895997	107.6299973
-5.9702535	4.5383997	114.1800003
-5.9702535	4.5871997	117.5899963
-5.9702535	4.6359997	116.3600006
-5.9702535	4.6847997	115.5699997
-5.9702535	4.7336001	113.6999969
-5.9702535	4.7824001	107.4700012
-5.9702535	4.8312001	106.3899994
-5.9702535	4.8800001	111.3099976
-5.9702535	4.9288001	110.6100006
-5.9702535	4.9776001	106.4700012
-5.9702535	5.0264001	106.4399948
-5.9702535	5.0752001	108.4300003
-5.9702535	5.1240001	107.5599976
-5.9702535	5.1728001	104.4399948
-5.9702535	5.2216001	105.0400009
-5.9702535	5.2704000	105.0499954
-5.9702535	5.3192000	103.4300003
-5.9702535	5.3680000	105.1500015
-5.9702535	5.4168000	102.0800018
-5.9702535	5.4656000	103.0299988
-5.9702535	5.5144000	105.4899979
-5.9702535	5.5632000	107.8499985
-5.9702535	5.6120000	107.7399979
-5.9702535	5.6608000	108.5000000
-5.9702535	5.7096000	107.1800003
-5.9702535	5.7584000	103.8499985
-5.9702535	5.8072000	98.4599991
-5.9702535	5.8559999	103.5400009
-5.9702535	5.9047999	105.3600006
-5.9702535	5.9535999	106.2399979
-5.9702535	6.0023999	106.4599991
-5.9702535	6.0511999	106.7999954
-5.9702535	6.0999999	105.9599991
-5.9702535	6.1487999	101.7999954
-5.9702535	6.1975999	98.5899963
-5.9702535	6.2463999	101.7699966
-5.9702535	6.2951999	103.7999954
-5.9702535	6.3439999	104.0199966
-5.9702535	6.3927999	101.8300018
-5.9702535	6.4415998	104.6399994
-5.9702535	6.4903998	106.1999969
-5.9702535	6.5391998	100.8799973
-5.9702535	6.5879998	103.5299988
-5.9702535	6.6367998	100.4599991
-5.9702535	6.6855998	105.1699982
-5.9702535	6.7343998	104.2500000
-5.9702535	6.7831998	103.8600006
-5.9702535	6.8319998	103.1199951
-5.9702535	6.8807998	97.5599976
-5.9702535	6.9295998	100.9700012
-5.9702535	6.9783998	104.3600006
-5.9702535	7.0271997	103.8300018
-5.9702535	7.0759997	100.2299957
-5.9702535	7.1247997	100.4499969
-5.9702535	7.1735997	102.5800018
-5.9702535	7.2223997	103.7999954

-5.9702535	7.2711997	100.9300003
-5.9702535	7.3199997	103.6599960
-5.9702535	7.3687997	103.2299957
-5.9702535	7.4175997	103.6199951
-5.9702535	7.4663997	103.8699951
-5.9702535	7.5151997	104.2399979
-5.9702535	7.5639997	104.7399979
-5.9702535	7.6127996	103.4799957
-5.9702535	7.6615996	107.4399948
-5.9702535	7.7103996	105.9499969
-5.9702535	7.7591996	102.9300003
-5.9702535	7.8079996	103.2999954
-5.9702535	7.8567996	100.5400009
-5.9702535	7.9056001	101.2200012
-5.9702535	7.9544001	103.3899994
-5.9702535	8.0032005	105.1299973
-5.9702535	8.0520000	108.0999985
-5.9702535	8.1008005	105.6599960
-5.9702535	8.1496000	104.5899963
-5.9702535	8.1984005	103.4799957
-5.9702535	8.2472000	104.8600006
-5.9702535	8.2960005	104.6899948
-5.9702535	8.3448000	102.1199951
-5.9702535	8.3936005	106.3199997
-5.9702535	8.4424000	106.8099976
-5.9702535	8.4912004	103.7999954
-5.9702535	8.5400000	104.2099991
-5.9702535	8.5888004	101.0400009
-5.9702535	8.6375999	102.6500015
-5.9702535	8.6864004	103.3499985
-5.9702535	8.7351999	103.3399963
-5.9702535	8.7840004	103.7799988
-5.9702535	8.8327999	104.2200012
-5.9702535	8.8816004	104.3899994
-5.9702535	8.9303999	101.6299973
-5.9702535	8.9792004	100.9199982
-5.9702535	9.0279999	101.6399994
-5.9702535	9.0768003	104.4399948
-5.9702535	9.1255999	105.6899948
-5.9702535	9.1744003	102.6899948
-5.9702535	9.2231998	103.0999985
-5.9702535	9.2720003	101.6800003
-5.9702535	9.3207998	100.4099960
-5.9702535	9.3696003	103.4599991
-5.9702535	9.4184008	103.0899963
-5.9702535	9.4672003	102.9799957
-5.9702535	9.5160007	104.8399963
-5.9702535	9.5648003	104.0899963
-5.9702535	9.6136007	102.5899963
-5.9702535	9.6624002	101.1599960
-5.9702535	9.7112007	101.8600006
-5.9702535	9.7600002	106.1699982
-5.9702535	9.8088007	106.7600021
-5.9702535	9.8576002	99.0699997
-5.9702535	9.9064007	104.1500015
-5.9702535	9.9552002	106.5299988
-5.9702535	10.0040007	105.2299957
-5.9702535	10.0528002	105.9300003

-5.9702535	10.1016006	105.2999954
-5.9702535	10.1504002	104.0199966
-5.9702535	10.1992006	99.0400009
-5.9702535	10.2480001	103.4000015
-5.9702535	10.2968006	103.2900009
-5.9702535	10.3456001	102.1800003
-5.9702535	10.3944006	102.0000000
-5.9702535	10.4432001	104.2799988
-5.9702535	10.4920006	103.2900009
-5.9702535	10.5408001	100.4099960
-5.9702535	10.5896006	101.3399963
-5.9702535	10.6384001	104.8499985
-5.9702535	10.6872005	104.6800003
-5.9702535	10.7360001	104.7699966
-5.9702535	10.7848005	103.9799957
-5.9702535	10.8336000	101.0800018
-5.9702535	10.8824005	99.3799973
-5.9702535	10.9312000	100.8600006
-5.9702535	10.9800005	103.5999985
-5.9702535	11.0288000	104.5999985
-5.9702535	11.0776005	103.9399948
-5.9702535	11.1264000	102.0800018
-5.9702535	11.1752005	106.0999985
-5.9702535	11.2240000	104.6399994
-5.9702535	11.2728004	105.7299957
-5.9702535	11.3216000	106.6299973
-5.9702535	11.3704004	105.2399979
-5.9702535	11.4191999	104.3600006
-5.9702535	11.4680004	99.8799973
-5.9702535	11.5167999	102.2999954
-5.9702535	11.5656004	103.9099960
-5.9702535	11.6143999	103.9000015
-5.9702535	11.6632004	102.7399979
-5.9702535	11.7119999	102.3699951
-5.9702535	11.7608004	104.2900009
-5.9702535	11.8095999	100.7500000
-5.9702535	11.8584003	103.8799973
-5.9702535	11.9071999	104.9499969
-5.9702535	11.9560003	102.8099976
-5.9702535	12.0047998	103.4000015
-5.9702535	12.0536003	100.6299973
-5.9702535	12.1023998	95.1899948
-5.9702535	12.1512003	97.0499954
-5.9702535	12.1999998	103.3699951
-5.9702535	12.2488003	105.8099976
-5.9702535	12.2975998	104.9399948
-5.9702535	12.3464003	105.6699982
-5.9702535	12.3951998	105.4499969
-5.9702535	12.4440002	99.7600021
-5.9702535	12.4927998	100.0699997
-5.9702535	12.5416002	100.9000015
-5.9702535	12.5904007	104.6599960
-5.9702535	12.6392002	101.8899994
-5.9702535	12.6880007	102.2999954
-5.9702535	12.7368002	101.4499969
-5.9702535	12.7856007	103.5800018
-5.9702535	12.8344002	101.9099960
-5.9702535	12.8832006	102.3300018

-5.9702535	12.9320002	103.5699997
-5.9702535	12.9808006	103.2399979
-5.9702535	13.0296001	102.5199966
-5.9702535	13.0784006	103.0999985
-5.9702535	13.1272001	104.9399948
-5.9702535	13.1760006	104.4499969
-5.9702535	13.2248001	101.6800003
-5.9702535	13.2736006	103.5100021
-5.9702535	13.3224001	103.8899994
-5.9702535	13.3712006	103.1399994
-5.9702535	13.4200001	99.6100006
-5.9702535	13.4688005	102.6399994
-5.9702535	13.5176001	103.2999954
-5.9702535	13.5664005	104.5800018
-5.9702535	13.6152000	104.8799973
-5.9702535	13.6640005	102.3199997
-5.9702535	13.7128000	101.1899948
-5.9702535	13.7616005	100.6399994
-5.9702535	13.8104000	101.9099960
-5.9702535	13.8592005	105.4499969
-5.9702535	13.9080000	105.2900009
-5.9702535	13.9568005	105.5100021
-5.9702535	14.0056000	105.7200012
-5.9702535	14.0544004	103.2399979
-5.9702535	14.1032000	101.8799973
-5.9702535	14.1520004	100.9700012
-5.9702535	14.2007999	103.9300003
-5.9702535	14.2496004	105.1100006
-5.9702535	14.2983999	102.8699951
-5.9702535	14.3472004	102.7799988
-5.9702535	14.3959999	102.8600006
-5.9702535	14.4448004	104.1399994
-5.9702535	14.4935999	103.8899994
-5.9702535	14.5424004	102.8099976
-5.9702535	14.5911999	104.8300018
-5.9702535	14.6400003	100.9399948
-5.9702535	14.6887999	100.6999969
-5.9702535	14.7376003	102.6299973
-5.9702535	14.7863998	102.6399994
-5.9702535	14.8352003	106.2399979
-5.9702535	14.8839998	107.2299957
-5.9702535	14.9328003	107.2500000
-5.9702535	14.9815998	104.9399948
-5.9702535	15.0304003	102.5999985
-5.9641175	0.3416000	105.6999969
-5.9641175	0.3904000	104.7399979
-5.9641175	0.4392000	104.9799957
-5.9641175	0.4880000	106.6800003
-5.9641175	0.5368000	105.0499954
-5.9641175	0.5856000	98.8600006
-5.9641175	0.6344000	102.6899948
-5.9641175	0.6832000	102.7799988
-5.9641175	0.7320000	104.6100006
-5.9641175	0.7808000	106.9700012
-5.9641175	0.8296000	105.5699997
-5.9641175	0.8784000	103.4599991
-5.9641175	0.9272000	105.3499985
-5.9641175	0.9760000	107.5100021

-5.9641175	1.0247999	109.6599960
-5.9641175	1.0735999	108.0299988
-5.9641175	1.1223999	106.5499954
-5.9641175	1.1711999	105.1699982
-5.9641175	1.2200000	107.0699997
-5.9641175	1.2688000	106.9799957
-5.9641175	1.3176000	106.0800018
-5.9641175	1.3664000	107.6800003
-5.9641175	1.4152000	108.5499954
-5.9641175	1.4640000	109.5599976
-5.9641175	1.5128000	108.5299988
-5.9641175	1.5616000	106.9899979
-5.9641175	1.6104000	108.8499985
-5.9641175	1.6592000	106.8799973
-5.9641175	1.7079999	104.1699982
-5.9641175	1.7567999	104.2999954
-5.9641175	1.8055999	110.0000000
-5.9641175	1.8543999	116.5699997
-5.9641175	1.9031999	119.1899948
-5.9641175	1.9519999	115.6800003
-5.9641175	2.0007999	105.7099991
-5.9641175	2.0495999	104.9300003
-5.9641175	2.0983999	106.9899979
-5.9641175	2.1471999	105.4300003
-5.9641175	2.1959999	103.6800003
-5.9641175	2.2447999	104.2699966
-5.9641175	2.2935998	98.5800018
-5.9641175	2.3423998	97.2500000
-5.9641175	2.3912001	104.2200012
-5.9641175	2.4400001	102.5000000
-5.9641175	2.4888000	107.2699966
-5.9641175	2.5376000	105.6100006
-5.9641175	2.5864000	104.5199966
-5.9641175	2.6352000	103.7699966
-5.9641175	2.6840000	102.0199966
-5.9641175	2.7328000	104.5100021
-5.9641175	2.7816000	107.5299988
-5.9641175	2.8304000	109.2099991
-5.9641175	2.8792000	106.4599991
-5.9641175	2.9280000	105.9199982
-5.9641175	2.9768000	100.7299957
-5.9641175	3.0256000	104.0400009
-5.9641175	3.0743999	104.4499969
-5.9641175	3.1231999	105.6800003
-5.9641175	3.1719999	103.5899963
-5.9641175	3.2207999	102.8199997
-5.9641175	3.2695999	104.2999954
-5.9641175	3.3183999	106.9399948
-5.9641175	3.3671999	104.9899979
-5.9641175	3.4159999	103.0100021
-5.9641175	3.4647999	96.5400009
-5.9641175	3.5135999	99.5999985
-5.9641175	3.5623999	101.6199951
-5.9641175	3.6111999	103.5299988
-5.9641175	3.6599998	104.5000000
-5.9641175	3.7087998	104.9399948
-5.9641175	3.7575998	104.7900009
-5.9641175	3.8063998	106.2299957

-5.9641175	3.8551998	108.6800003
-5.9641175	3.9039998	107.4799957
-5.9641175	3.9527998	103.9499969
-5.9641175	4.0016003	97.4300003
-5.9641175	4.0503998	103.1999969
-5.9641175	4.0991998	106.2200012
-5.9641175	4.1479998	105.4899979
-5.9641175	4.1967998	102.9099960
-5.9641175	4.2455997	103.1199951
-5.9641175	4.2943997	107.3199997
-5.9641175	4.3431997	110.1199951
-5.9641175	4.3919997	109.5499954
-5.9641175	4.4407997	106.8099976
-5.9641175	4.4895997	107.6999969
-5.9641175	4.5383997	112.9399948
-5.9641175	4.5871997	116.3199997
-5.9641175	4.6359997	115.1699982
-5.9641175	4.6847997	114.6199951
-5.9641175	4.7336001	113.3600006
-5.9641175	4.7824001	107.5199966
-5.9641175	4.8312001	106.5499954
-5.9641175	4.8800001	110.5499954
-5.9641175	4.9288001	109.5400009
-5.9641175	4.9776001	106.4199982
-5.9641175	5.0264001	107.4899979
-5.9641175	5.0752001	109.5999985
-5.9641175	5.1240001	108.0599976
-5.9641175	5.1728001	103.1999969
-5.9641175	5.2216001	104.3899994
-5.9641175	5.2704000	104.7500000
-5.9641175	5.3192000	103.3799973
-5.9641175	5.3680000	105.3600006
-5.9641175	5.4168000	103.2399979
-5.9641175	5.4656000	103.0499954
-5.9641175	5.5144000	104.7900009
-5.9641175	5.5632000	107.7399979
-5.9641175	5.6120000	108.1800003
-5.9641175	5.6608000	108.8799973
-5.9641175	5.7096000	107.5299988
-5.9641175	5.7584000	104.2399979
-5.9641175	5.8072000	98.3799973
-5.9641175	5.8559999	103.6800003
-5.9641175	5.9047999	105.4199982
-5.9641175	5.9535999	106.1500015
-5.9641175	6.0023999	105.8499985
-5.9641175	6.0511999	106.8300018
-5.9641175	6.0999999	105.5499954
-5.9641175	6.1487999	101.8499985
-5.9641175	6.1975999	97.6199951
-5.9641175	6.2463999	101.6800003
-5.9641175	6.2951999	103.9700012
-5.9641175	6.3439999	103.9099960
-5.9641175	6.3927999	101.7099991
-5.9641175	6.4415998	105.0299988
-5.9641175	6.4903998	105.9199982
-5.9641175	6.5391998	100.8799973
-5.9641175	6.5879998	103.8600006
-5.9641175	6.6367998	101.7200012

-5.9641175	6.6855998	104.5599976
-5.9641175	6.7343998	103.3099976
-5.9641175	6.7831998	103.1800003
-5.9641175	6.8319998	103.4899979
-5.9641175	6.8807998	99.6699982
-5.9641175	6.9295998	101.8099976
-5.9641175	6.9783998	104.1299973
-5.9641175	7.0271997	103.9099960
-5.9641175	7.0759997	99.9599991
-5.9641175	7.1247997	100.7600021
-5.9641175	7.1735997	103.5000000
-5.9641175	7.2223997	104.8899994
-5.9641175	7.2711997	102.0000000
-5.9641175	7.3199997	103.1599960
-5.9641175	7.3687997	103.1599960
-5.9641175	7.4175997	102.6999969
-5.9641175	7.4663997	104.5299988
-5.9641175	7.5151997	104.4499969
-5.9641175	7.5639997	104.6199951
-5.9641175	7.6127996	103.7699966
-5.9641175	7.6615996	106.6800003
-5.9641175	7.7103996	105.3399963
-5.9641175	7.7591996	102.6500015
-5.9641175	7.8079996	103.7399979
-5.9641175	7.8567996	100.9399948
-5.9641175	7.9056001	102.3699951
-5.9641175	7.9544001	103.5299988
-5.9641175	8.0032005	105.0699997
-5.9641175	8.0520000	108.0400009
-5.9641175	8.1008005	106.2200012
-5.9641175	8.1496000	104.7399979
-5.9641175	8.1984005	104.6999969
-5.9641175	8.2472000	105.3699951
-5.9641175	8.2960005	104.8300018
-5.9641175	8.3448000	102.8399963
-5.9641175	8.3936005	106.3799973
-5.9641175	8.4424000	106.2799988
-5.9641175	8.4912004	102.5899963
-5.9641175	8.5400000	103.6899948
-5.9641175	8.5888004	99.6500015
-5.9641175	8.6375999	101.8499985
-5.9641175	8.6864004	103.3300018
-5.9641175	8.7351999	103.9300003
-5.9641175	8.7840004	104.1999969
-5.9641175	8.8327999	104.9099960
-5.9641175	8.8816004	104.4000015
-5.9641175	8.9303999	100.5999985
-5.9641175	8.9792004	99.5199966
-5.9641175	9.0279999	101.1199951
-5.9641175	9.0768003	104.4499969
-5.9641175	9.1255999	105.2699966
-5.9641175	9.1744003	101.9799957
-5.9641175	9.2231998	103.5100021
-5.9641175	9.2720003	102.0299988
-5.9641175	9.3207998	100.0400009
-5.9641175	9.3696003	102.7799988
-5.9641175	9.4184008	102.1100006
-5.9641175	9.4672003	102.6899948

-5.9641175	9.5160007	104.8699951
-5.9641175	9.5648003	104.6199951
-5.9641175	9.6136007	102.5699997
-5.9641175	9.6624002	100.7099991
-5.9641175	9.7112007	103.8399963
-5.9641175	9.7600002	106.7500000
-5.9641175	9.8088007	107.1199951
-5.9641175	9.8576002	99.3699951
-5.9641175	9.9064007	105.3600006
-5.9641175	9.9552002	107.3699951
-5.9641175	10.0040007	104.6399994
-5.9641175	10.0528002	105.6999969
-5.9641175	10.1016006	106.0800018
-5.9641175	10.1504002	103.9099960
-5.9641175	10.1992006	97.7600021
-5.9641175	10.2480001	102.9700012
-5.9641175	10.2968006	103.0599976
-5.9641175	10.3456001	101.5499954
-5.9641175	10.3944006	101.8399963
-5.9641175	10.4432001	103.4700012
-5.9641175	10.4920006	102.7799988
-5.9641175	10.5408001	99.6599960
-5.9641175	10.5896006	100.9099960
-5.9641175	10.6384001	104.7699966
-5.9641175	10.6872005	105.8799973
-5.9641175	10.7360001	105.4399948
-5.9641175	10.7848005	104.2299957
-5.9641175	10.8336000	100.9000015
-5.9641175	10.8824005	99.4599991
-5.9641175	10.9312000	100.8099976
-5.9641175	10.9800005	104.6399994
-5.9641175	11.0288000	104.9599991
-5.9641175	11.0776005	103.5000000
-5.9641175	11.1264000	102.0499954
-5.9641175	11.1752005	105.9599991
-5.9641175	11.2240000	105.3199997
-5.9641175	11.2728004	105.1299973
-5.9641175	11.3216000	106.1199951
-5.9641175	11.3704004	104.6199951
-5.9641175	11.4191999	104.1399994
-5.9641175	11.4680004	100.3199997
-5.9641175	11.5167999	103.0800018
-5.9641175	11.5656004	103.6500015
-5.9641175	11.6143999	103.0499954
-5.9641175	11.6632004	103.5299988
-5.9641175	11.7119999	102.3600006
-5.9641175	11.7608004	104.0000000
-5.9641175	11.8095999	101.6699982
-5.9641175	11.8584003	103.5800018
-5.9641175	11.9071999	104.3699951
-5.9641175	11.9560003	102.9099960
-5.9641175	12.0047998	101.7399979
-5.9641175	12.0536003	98.5199966
-5.9641175	12.1023998	92.8399963
-5.9641175	12.1512003	97.8600006
-5.9641175	12.1999998	103.7200012
-5.9641175	12.2488003	105.4499969
-5.9641175	12.2975998	103.9700012

-5.9641175	12.3464003	104.7200012
-5.9641175	12.3951998	105.0899963
-5.9641175	12.4440002	99.9399948
-5.9641175	12.4927998	100.0199966
-5.9641175	12.5416002	101.4700012
-5.9641175	12.5904007	104.9700012
-5.9641175	12.6392002	102.3399963
-5.9641175	12.6880007	103.0999985
-5.9641175	12.7368002	102.7200012
-5.9641175	12.7856007	104.0000000
-5.9641175	12.8344002	101.1800003
-5.9641175	12.8832006	102.6399994
-5.9641175	12.9320002	104.0499954
-5.9641175	12.9808006	103.5499954
-5.9641175	13.0296001	101.1999969
-5.9641175	13.0784006	104.2099991
-5.9641175	13.1272001	106.3199997
-5.9641175	13.1760006	105.4000015
-5.9641175	13.2248001	102.9799957
-5.9641175	13.2736006	104.0400009
-5.9641175	13.3224001	105.1100006
-5.9641175	13.3712006	104.4700012
-5.9641175	13.4200001	100.7099991
-5.9641175	13.4688005	102.2399979
-5.9641175	13.5176001	102.9499969
-5.9641175	13.5664005	103.9499969
-5.9641175	13.6152000	104.9499969
-5.9641175	13.6640005	102.9399948
-5.9641175	13.7128000	102.7500000
-5.9641175	13.7616005	101.9000015
-5.9641175	13.8104000	103.5400009
-5.9641175	13.8592005	105.9499969
-5.9641175	13.9080000	103.6800003
-5.9641175	13.9568005	104.9399948
-5.9641175	14.0056000	105.5499954
-5.9641175	14.0544004	103.9899979
-5.9641175	14.1032000	102.0999985
-5.9641175	14.1520004	101.5999985
-5.9641175	14.2007999	104.7699966
-5.9641175	14.2496004	105.2799988
-5.9641175	14.2983999	101.9099960
-5.9641175	14.3472004	102.1699982
-5.9641175	14.3959999	103.4499969
-5.9641175	14.4448004	104.2900009
-5.9641175	14.4935999	104.1999969
-5.9641175	14.5424004	103.9199982
-5.9641175	14.5911999	106.1299973
-5.9641175	14.6400003	103.5299988
-5.9641175	14.6887999	101.5400009
-5.9641175	14.7376003	102.3300018
-5.9641175	14.7863998	102.6800003
-5.9641175	14.8352003	106.4599991
-5.9641175	14.8839998	107.9000015
-5.9641175	14.9328003	108.3499985
-5.9641175	14.9815998	106.0800018
-5.9641175	15.0304003	103.4199982
-5.9579816	0.3416000	106.7399979
-5.9579816	0.3904000	105.6699982

-5.9579816	0.4392000	105.6199951
-5.9579816	0.4880000	107.7299957
-5.9579816	0.5368000	106.1199951
-5.9579816	0.5856000	101.0699997
-5.9579816	0.6344000	103.6399994
-5.9579816	0.6832000	103.0800018
-5.9579816	0.7320000	104.6699982
-5.9579816	0.7808000	106.8099976
-5.9579816	0.8296000	105.8399963
-5.9579816	0.8784000	103.0199966
-5.9579816	0.9272000	103.5699997
-5.9579816	0.9760000	105.8799973
-5.9579816	1.0247999	109.6299973
-5.9579816	1.0735999	108.4199982
-5.9579816	1.1223999	105.7600021
-5.9579816	1.1711999	103.6599960
-5.9579816	1.2200000	106.1599960
-5.9579816	1.2688000	106.4799957
-5.9579816	1.3176000	105.9599991
-5.9579816	1.3664000	107.3600006
-5.9579816	1.4152000	108.2600021
-5.9579816	1.4640000	109.4599991
-5.9579816	1.5128000	107.7600021
-5.9579816	1.5616000	106.9899979
-5.9579816	1.6104000	108.7399979
-5.9579816	1.6592000	106.4000015
-5.9579816	1.7079999	105.2299957
-5.9579816	1.7567999	103.6500015
-5.9579816	1.8055999	109.3300018
-5.9579816	1.8543999	116.2900009
-5.9579816	1.9031999	118.7600021
-5.9579816	1.9519999	115.3600006
-5.9579816	2.0007999	106.5000000
-5.9579816	2.0495999	104.6299973
-5.9579816	2.0983999	106.8199997
-5.9579816	2.1471999	105.4399948
-5.9579816	2.1959999	104.3300018
-5.9579816	2.2447999	104.4199982
-5.9579816	2.2935998	96.3899994
-5.9579816	2.3423998	97.2900009
-5.9579816	2.3912001	103.5199966
-5.9579816	2.4400001	103.2600021
-5.9579816	2.4888000	107.6800003
-5.9579816	2.5376000	106.3300018
-5.9579816	2.5864000	104.8199997
-5.9579816	2.6352000	104.2699966
-5.9579816	2.6840000	101.8899994
-5.9579816	2.7328000	105.3600006
-5.9579816	2.7816000	107.8099976
-5.9579816	2.8304000	109.1100006
-5.9579816	2.8792000	105.7999954
-5.9579816	2.9280000	106.3899994
-5.9579816	2.9768000	102.6100006
-5.9579816	3.0256000	104.7399979
-5.9579816	3.0743999	103.9399948
-5.9579816	3.1231999	104.5999985
-5.9579816	3.1719999	102.7099991
-5.9579816	3.2207999	101.1399994

-5.9579816	3.2695999	104.5299988
-5.9579816	3.3183999	106.2099991
-5.9579816	3.3671999	104.6699982
-5.9579816	3.4159999	102.8099976
-5.9579816	3.4647999	96.5400009
-5.9579816	3.5135999	99.0400009
-5.9579816	3.5623999	101.9499969
-5.9579816	3.6111999	104.3300018
-5.9579816	3.6599998	104.5800018
-5.9579816	3.7087998	104.9300003
-5.9579816	3.7575998	103.7600021
-5.9579816	3.8063998	104.9399948
-5.9579816	3.8551998	108.6299973
-5.9579816	3.9039998	107.5899963
-5.9579816	3.9527998	103.4099960
-5.9579816	4.0016003	97.7099991
-5.9579816	4.0503998	103.0999985
-5.9579816	4.0991998	105.6199951
-5.9579816	4.1479998	105.8799973
-5.9579816	4.1967998	103.8300018
-5.9579816	4.2455997	103.5299988
-5.9579816	4.2943997	107.5599976
-5.9579816	4.3431997	110.7299957
-5.9579816	4.3919997	110.7099991
-5.9579816	4.4407997	107.7500000
-5.9579816	4.4895997	108.4000015
-5.9579816	4.5383997	112.0400009
-5.9579816	4.5871997	115.0599976
-5.9579816	4.6359997	114.1399994
-5.9579816	4.6847997	114.0400009
-5.9579816	4.7336001	113.1699982
-5.9579816	4.7824001	107.6899948
-5.9579816	4.8312001	107.0299988
-5.9579816	4.8800001	110.3899994
-5.9579816	4.9288001	109.0800018
-5.9579816	4.9776001	106.0699997
-5.9579816	5.0264001	108.5100021
-5.9579816	5.0752001	110.5800018
-5.9579816	5.1240001	108.5000000
-5.9579816	5.1728001	102.7600021
-5.9579816	5.2216001	104.8099976
-5.9579816	5.2704000	104.7299957
-5.9579816	5.3192000	104.0999985
-5.9579816	5.3680000	105.7900009
-5.9579816	5.4168000	103.9599991
-5.9579816	5.4656000	102.8499985
-5.9579816	5.5144000	104.4000015
-5.9579816	5.5632000	107.3499985
-5.9579816	5.6120000	108.1800003
-5.9579816	5.6608000	109.3799973
-5.9579816	5.7096000	107.9899979
-5.9579816	5.7584000	104.5499954
-5.9579816	5.8072000	98.8399963
-5.9579816	5.8559999	103.3399963
-5.9579816	5.9047999	104.9000015
-5.9579816	5.9535999	105.8899994
-5.9579816	6.0023999	105.2600021
-5.9579816	6.0511999	106.8300018

-5.9579816	6.0999999	105.0899963
-5.9579816	6.1487999	102.2999954
-5.9579816	6.1975999	98.3600006
-5.9579816	6.2463999	101.5000000
-5.9579816	6.2951999	104.0599976
-5.9579816	6.3439999	104.0499954
-5.9579816	6.3927999	101.5999985
-5.9579816	6.4415998	104.9300003
-5.9579816	6.4903998	104.7799988
-5.9579816	6.5391998	99.6299973
-5.9579816	6.5879998	103.7699966
-5.9579816	6.6367998	102.4799957
-5.9579816	6.6855998	103.6299973
-5.9579816	6.7343998	102.9700012
-5.9579816	6.7831998	102.5599976
-5.9579816	6.8319998	104.2600021
-5.9579816	6.8807998	101.6399994
-5.9579816	6.9295998	103.1699982
-5.9579816	6.9783998	104.5699997
-5.9579816	7.0271997	104.4000015
-5.9579816	7.0759997	100.5699997
-5.9579816	7.1247997	101.6299973
-5.9579816	7.1735997	104.3899994
-5.9579816	7.2223997	105.7799988
-5.9579816	7.2711997	102.6399994
-5.9579816	7.3199997	101.6999969
-5.9579816	7.3687997	102.1100006
-5.9579816	7.4175997	101.1699982
-5.9579816	7.4663997	104.6500015
-5.9579816	7.5151997	104.3799973
-5.9579816	7.5639997	104.7600021
-5.9579816	7.6127996	104.0499954
-5.9579816	7.6615996	105.4499969
-5.9579816	7.7103996	104.7500000
-5.9579816	7.7591996	102.7500000
-5.9579816	7.8079996	103.9700012
-5.9579816	7.8567996	101.1800003
-5.9579816	7.9056001	103.4399948
-5.9579816	7.9544001	103.6299973
-5.9579816	8.0032005	104.4899979
-5.9579816	8.0520000	107.6800003
-5.9579816	8.1008005	106.7699966
-5.9579816	8.1496000	104.8699951
-5.9579816	8.1984005	105.3099976
-5.9579816	8.2472000	105.6299973
-5.9579816	8.2960005	104.7999954
-5.9579816	8.3448000	103.8300018
-5.9579816	8.3936005	106.2200012
-5.9579816	8.4424000	105.5499954
-5.9579816	8.4912004	101.5800018
-5.9579816	8.5400000	104.0499954
-5.9579816	8.5888004	99.8300018
-5.9579816	8.6375999	102.2200012
-5.9579816	8.6864004	104.2900009
-5.9579816	8.7351999	104.7399979
-5.9579816	8.7840004	104.1699982
-5.9579816	8.8327999	105.0800018
-5.9579816	8.8816004	104.5299988

-5.9579816	8.9303999	100.5100021
-5.9579816	8.9792004	98.8699951
-5.9579816	9.0279999	101.0100021
-5.9579816	9.0768003	104.3600006
-5.9579816	9.1255999	104.6299973
-5.9579816	9.1744003	101.3799973
-5.9579816	9.2231998	103.7600021
-5.9579816	9.2720003	102.1999969
-5.9579816	9.3207998	99.5199966
-5.9579816	9.3696003	102.0800018
-5.9579816	9.4184008	102.2600021
-5.9579816	9.4672003	102.4599991
-5.9579816	9.5160007	104.4300003
-5.9579816	9.5648003	104.7799988
-5.9579816	9.6136007	102.5699997
-5.9579816	9.6624002	100.7200012
-5.9579816	9.7112007	104.9799957
-5.9579816	9.7600002	106.9000015
-5.9579816	9.8088007	107.1500015
-5.9579816	9.8576002	100.5000000
-5.9579816	9.9064007	106.2500000
-5.9579816	9.9552002	107.4899979
-5.9579816	10.0040007	103.4499969
-5.9579816	10.0528002	104.8899994
-5.9579816	10.1016006	106.7200012
-5.9579816	10.1504002	104.8600006
-5.9579816	10.1992006	97.4700012
-5.9579816	10.2480001	102.5699997
-5.9579816	10.2968006	102.3399963
-5.9579816	10.3456001	100.6999969
-5.9579816	10.3944006	101.4599991
-5.9579816	10.4432001	103.0699997
-5.9579816	10.4920006	102.0000000
-5.9579816	10.5408001	98.4499969
-5.9579816	10.5896006	99.9599991
-5.9579816	10.6384001	104.1800003
-5.9579816	10.6872005	106.2699966
-5.9579816	10.7360001	105.3899994
-5.9579816	10.7848005	103.7600021
-5.9579816	10.8336000	100.0400009
-5.9579816	10.8824005	99.4399948
-5.9579816	10.9312000	100.5699997
-5.9579816	10.9800005	105.4899979
-5.9579816	11.0288000	105.4300003
-5.9579816	11.0776005	102.9300003
-5.9579816	11.1264000	101.6899948
-5.9579816	11.1752005	105.3899994
-5.9579816	11.2240000	105.5299988
-5.9579816	11.2728004	104.2299957
-5.9579816	11.3216000	105.3899994
-5.9579816	11.3704004	104.4700012
-5.9579816	11.4191999	104.0000000
-5.9579816	11.4680004	100.4700012
-5.9579816	11.5167999	103.4799957
-5.9579816	11.5656004	103.5999985
-5.9579816	11.6143999	102.0499954
-5.9579816	11.6632004	103.3399963
-5.9579816	11.7119999	102.5699997

-5.9579816	11.7608004	103.1399994
-5.9579816	11.8095999	101.7799988
-5.9579816	11.8584003	103.0400009
-5.9579816	11.9071999	103.7399979
-5.9579816	11.9560003	103.4899979
-5.9579816	12.0047998	101.0499954
-5.9579816	12.0536003	97.4000015
-5.9579816	12.1023998	91.1100006
-5.9579816	12.1512003	98.8799973
-5.9579816	12.1999998	103.2699966
-5.9579816	12.2488003	104.4799957
-5.9579816	12.2975998	102.9099960
-5.9579816	12.3464003	103.9499969
-5.9579816	12.3951998	104.7399979
-5.9579816	12.4440002	100.7399979
-5.9579816	12.4927998	101.4799957
-5.9579816	12.5416002	102.6699982
-5.9579816	12.5904007	105.0599976
-5.9579816	12.6392002	102.3699951
-5.9579816	12.6880007	103.2600021
-5.9579816	12.7368002	103.8799973
-5.9579816	12.7856007	105.0299988
-5.9579816	12.8344002	101.6800003
-5.9579816	12.8832006	103.3199997
-5.9579816	12.9320002	104.1899948
-5.9579816	12.9808006	103.5100021
-5.9579816	13.0296001	99.9199982
-5.9579816	13.0784006	104.4199982
-5.9579816	13.1272001	106.5000000
-5.9579816	13.1760006	105.7099991
-5.9579816	13.2248001	103.6199951
-5.9579816	13.2736006	104.3199997
-5.9579816	13.3224001	105.9199982
-5.9579816	13.3712006	105.0599976
-5.9579816	13.4200001	101.7999954
-5.9579816	13.4688005	101.8799973
-5.9579816	13.5176001	101.7799988
-5.9579816	13.5664005	102.7900009
-5.9579816	13.6152000	105.3399963
-5.9579816	13.6640005	104.5000000
-5.9579816	13.7128000	104.7299957
-5.9579816	13.7616005	103.4199982
-5.9579816	13.8104000	104.5699997
-5.9579816	13.8592005	106.0899963
-5.9579816	13.9080000	101.6199951
-5.9579816	13.9568005	103.3799973
-5.9579816	14.0056000	104.1299973
-5.9579816	14.0544004	104.1100006
-5.9579816	14.1032000	102.2200012
-5.9579816	14.1520004	102.0899963
-5.9579816	14.2007999	105.1399994
-5.9579816	14.2496004	105.1800003
-5.9579816	14.2983999	102.0499954
-5.9579816	14.3472004	102.2299957
-5.9579816	14.3959999	104.3899994
-5.9579816	14.4448004	104.7799988
-5.9579816	14.4935999	104.4300003
-5.9579816	14.5424004	104.1500015

-5.9579816	14.5911999	106.5199966
-5.9579816	14.6400003	105.7600021
-5.9579816	14.6887999	101.8600006
-5.9579816	14.7376003	101.1800003
-5.9579816	14.7863998	102.0100021
-5.9579816	14.8352003	105.6500015
-5.9579816	14.8839998	107.7399979
-5.9579816	14.9328003	108.8699951
-5.9579816	14.9815998	107.4300003
-5.9579816	15.0304003	104.6699982
-5.9518456	0.3416000	107.9399948
-5.9518456	0.3904000	107.3899994
-5.9518456	0.4392000	106.2500000
-5.9518456	0.4880000	107.7299957
-5.9518456	0.5368000	106.4499969
-5.9518456	0.5856000	103.3099976
-5.9518456	0.6344000	104.4399948
-5.9518456	0.6832000	102.5599976
-5.9518456	0.7320000	104.1399994
-5.9518456	0.7808000	106.3499985
-5.9518456	0.8296000	106.4099960
-5.9518456	0.8784000	102.9099960
-5.9518456	0.9272000	102.7799988
-5.9518456	0.9760000	104.4499969
-5.9518456	1.0247999	108.9199982
-5.9518456	1.0735999	107.5199966
-5.9518456	1.1223999	104.5599976
-5.9518456	1.1711999	103.0299988
-5.9518456	1.2200000	105.9799957
-5.9518456	1.2688000	106.5400009
-5.9518456	1.3176000	106.1599960
-5.9518456	1.3664000	106.9899979
-5.9518456	1.4152000	107.8099976
-5.9518456	1.4640000	108.6199951
-5.9518456	1.5128000	106.9399948
-5.9518456	1.5616000	107.1899948
-5.9518456	1.6104000	109.2200012
-5.9518456	1.6592000	107.3899994
-5.9518456	1.7079999	106.8199997
-5.9518456	1.7567999	103.5299988
-5.9518456	1.8055999	109.0299988
-5.9518456	1.8543999	116.3799973
-5.9518456	1.9031999	118.7200012
-5.9518456	1.9519999	115.3199997
-5.9518456	2.0007999	106.9499969
-5.9518456	2.0495999	104.8099976
-5.9518456	2.0983999	106.8899994
-5.9518456	2.1471999	106.0599976
-5.9518456	2.1959999	105.3199997
-5.9518456	2.2447999	104.4700012
-5.9518456	2.2935998	95.8699951
-5.9518456	2.3423998	97.9000015
-5.9518456	2.3912001	102.8099976
-5.9518456	2.4400001	103.3399963
-5.9518456	2.4888000	107.6699982
-5.9518456	2.5376000	106.6299973
-5.9518456	2.5864000	105.0000000
-5.9518456	2.6352000	104.7200012

-5.9518456	2.6840000	102.0499954
-5.9518456	2.7328000	105.9899979
-5.9518456	2.7816000	108.0499954
-5.9518456	2.8304000	109.2600021
-5.9518456	2.8792000	106.3099976
-5.9518456	2.9280000	106.1800003
-5.9518456	2.9768000	103.8199997
-5.9518456	3.0256000	105.5299988
-5.9518456	3.0743999	103.5400009
-5.9518456	3.1231999	103.7600021
-5.9518456	3.1719999	102.3499985
-5.9518456	3.2207999	100.1500015
-5.9518456	3.2695999	104.3899994
-5.9518456	3.3183999	105.1199951
-5.9518456	3.3671999	104.0800018
-5.9518456	3.4159999	102.5000000
-5.9518456	3.4647999	97.5699997
-5.9518456	3.5135999	98.5999985
-5.9518456	3.5623999	101.4199982
-5.9518456	3.6111999	104.5499954
-5.9518456	3.6599998	104.0000000
-5.9518456	3.7087998	103.3799973
-5.9518456	3.7575998	102.4599991
-5.9518456	3.8063998	103.2099991
-5.9518456	3.8551998	108.2399979
-5.9518456	3.9039998	107.6899948
-5.9518456	3.9527998	103.1299973
-5.9518456	4.0016003	99.2399979
-5.9518456	4.0503998	103.0599976
-5.9518456	4.0991998	104.6899948
-5.9518456	4.1479998	105.9700012
-5.9518456	4.1967998	104.5899963
-5.9518456	4.2455997	103.9399948
-5.9518456	4.2943997	107.7399979
-5.9518456	4.3431997	110.9499969
-5.9518456	4.3919997	111.0199966
-5.9518456	4.4407997	108.3399963
-5.9518456	4.4895997	109.3699951
-5.9518456	4.5383997	112.1999969
-5.9518456	4.5871997	114.5199966
-5.9518456	4.6359997	113.7799988
-5.9518456	4.6847997	114.1899948
-5.9518456	4.7336001	113.5000000
-5.9518456	4.7824001	108.3399963
-5.9518456	4.8312001	108.6199951
-5.9518456	4.8800001	110.4099960
-5.9518456	4.9288001	108.6399994
-5.9518456	4.9776001	106.0100021
-5.9518456	5.0264001	109.3899994
-5.9518456	5.0752001	110.9199982
-5.9518456	5.1240001	108.4700012
-5.9518456	5.1728001	102.9799957
-5.9518456	5.2216001	105.2299957
-5.9518456	5.2704000	104.8799973
-5.9518456	5.3192000	104.5899963
-5.9518456	5.3680000	105.9700012
-5.9518456	5.4168000	104.4700012
-5.9518456	5.4656000	101.7799988

-5.9518456	5.5144000	104.2299957
-5.9518456	5.5632000	107.2399979
-5.9518456	5.6120000	108.0000000
-5.9518456	5.6608000	109.2699966
-5.9518456	5.7096000	108.0899963
-5.9518456	5.7584000	104.7200012
-5.9518456	5.8072000	100.4499969
-5.9518456	5.8559999	103.6800003
-5.9518456	5.9047999	105.1800003
-5.9518456	5.9535999	106.4700012
-5.9518456	6.0023999	104.6999969
-5.9518456	6.0511999	106.6299973
-5.9518456	6.0999999	104.9599991
-5.9518456	6.1487999	102.7799988
-5.9518456	6.1975999	98.7500000
-5.9518456	6.2463999	101.4599991
-5.9518456	6.2951999	103.8399963
-5.9518456	6.3439999	103.2200012
-5.9518456	6.3927999	101.6500015
-5.9518456	6.4415998	104.6800003
-5.9518456	6.4903998	103.8399963
-5.9518456	6.5391998	99.7200012
-5.9518456	6.5879998	103.4799957
-5.9518456	6.6367998	103.1999969
-5.9518456	6.6855998	103.0199966
-5.9518456	6.7343998	102.8699951
-5.9518456	6.7831998	101.6899948
-5.9518456	6.8319998	104.5299988
-5.9518456	6.8807998	103.4300003
-5.9518456	6.9295998	104.7600021
-5.9518456	6.9783998	105.0299988
-5.9518456	7.0271997	104.5000000
-5.9518456	7.0759997	101.5499954
-5.9518456	7.1247997	102.3399963
-5.9518456	7.1735997	104.7500000
-5.9518456	7.2223997	106.6299973
-5.9518456	7.2711997	103.7600021
-5.9518456	7.3199997	100.9199982
-5.9518456	7.3687997	101.5199966
-5.9518456	7.4175997	99.7699966
-5.9518456	7.4663997	104.1899948
-5.9518456	7.5151997	103.5100021
-5.9518456	7.5639997	104.5499954
-5.9518456	7.6127996	104.7200012
-5.9518456	7.6615996	104.7200012
-5.9518456	7.7103996	104.2900009
-5.9518456	7.7591996	102.4499969
-5.9518456	7.8079996	104.1800003
-5.9518456	7.8567996	101.8899994
-5.9518456	7.9056001	104.9199982
-5.9518456	7.9544001	104.1399994
-5.9518456	8.0032005	103.9799957
-5.9518456	8.0520000	107.0899963
-5.9518456	8.1008005	106.7900009
-5.9518456	8.1496000	105.2999954
-5.9518456	8.1984005	105.9199982
-5.9518456	8.2472000	105.3600006
-5.9518456	8.2960005	104.4000015

-5.9518456	8.3448000	104.4099960
-5.9518456	8.3936005	105.9000015
-5.9518456	8.4424000	103.9799957
-5.9518456	8.4912004	99.9199982
-5.9518456	8.5400000	104.2699966
-5.9518456	8.5888004	100.0299988
-5.9518456	8.6375999	101.8499985
-5.9518456	8.6864004	104.5299988
-5.9518456	8.7351999	104.9199982
-5.9518456	8.7840004	103.6699982
-5.9518456	8.8327999	105.1999969
-5.9518456	8.8816004	105.2299957
-5.9518456	8.9303999	101.9599991
-5.9518456	8.9792004	99.1599960
-5.9518456	9.0279999	101.1199951
-5.9518456	9.0768003	104.4099960
-5.9518456	9.1255999	104.0699997
-5.9518456	9.1744003	100.9300003
-5.9518456	9.2231998	103.8499985
-5.9518456	9.2720003	102.5299988
-5.9518456	9.3207998	100.5800018
-5.9518456	9.3696003	101.8899994
-5.9518456	9.4184008	102.4099960
-5.9518456	9.4672003	101.9899979
-5.9518456	9.5160007	103.3799973
-5.9518456	9.5648003	104.2399979
-5.9518456	9.6136007	102.4399948
-5.9518456	9.6624002	101.5000000
-5.9518456	9.7112007	105.7900009
-5.9518456	9.7600002	106.0699997
-5.9518456	9.8088007	106.6699982
-5.9518456	9.8576002	101.9000015
-5.9518456	9.9064007	107.0400009
-5.9518456	9.9552002	107.5000000
-5.9518456	10.0040007	102.9700012
-5.9518456	10.0528002	103.6299973
-5.9518456	10.1016006	106.6999969
-5.9518456	10.1504002	105.4000015
-5.9518456	10.1992006	98.5899963
-5.9518456	10.2480001	101.5800018
-5.9518456	10.2968006	100.7500000
-5.9518456	10.3456001	99.5499954
-5.9518456	10.3944006	101.3600006
-5.9518456	10.4432001	103.0899963
-5.9518456	10.4920006	102.0800018
-5.9518456	10.5408001	98.3199997
-5.9518456	10.5896006	99.7500000
-5.9518456	10.6384001	103.6800003
-5.9518456	10.6872005	105.9300003
-5.9518456	10.7360001	104.8199997
-5.9518456	10.7848005	103.8099976
-5.9518456	10.8336000	100.2299957
-5.9518456	10.8824005	100.8199997
-5.9518456	10.9312000	101.0000000
-5.9518456	10.9800005	106.0499954
-5.9518456	11.0288000	105.4599991
-5.9518456	11.0776005	102.3499985
-5.9518456	11.1264000	100.7299957

-5.9518456	11.1752005	104.6299973
-5.9518456	11.2240000	105.0499954
-5.9518456	11.2728004	103.7600021
-5.9518456	11.3216000	105.0100021
-5.9518456	11.3704004	104.4599991
-5.9518456	11.4191999	104.3899994
-5.9518456	11.4680004	101.2900009
-5.9518456	11.5167999	103.5699997
-5.9518456	11.5656004	103.0100021
-5.9518456	11.6143999	101.0599976
-5.9518456	11.6632004	103.0100021
-5.9518456	11.7119999	102.9300003
-5.9518456	11.7608004	102.9000015
-5.9518456	11.8095999	101.9000015
-5.9518456	11.8584003	102.5100021
-5.9518456	11.9071999	102.6500015
-5.9518456	11.9560003	102.6899948
-5.9518456	12.0047998	100.4000015
-5.9518456	12.0536003	97.0299988
-5.9518456	12.1023998	92.3099976
-5.9518456	12.1512003	99.9000015
-5.9518456	12.1999998	102.9000015
-5.9518456	12.2488003	103.3799973
-5.9518456	12.2975998	101.7200012
-5.9518456	12.3464003	102.9099960
-5.9518456	12.3951998	104.1299973
-5.9518456	12.4440002	101.5000000
-5.9518456	12.4927998	102.8300018
-5.9518456	12.5416002	103.4700012
-5.9518456	12.5904007	104.4899979
-5.9518456	12.6392002	102.1999969
-5.9518456	12.6880007	103.5299988
-5.9518456	12.7368002	105.1500015
-5.9518456	12.7856007	105.9799957
-5.9518456	12.8344002	102.6999969
-5.9518456	12.8832006	103.9099960
-5.9518456	12.9320002	104.0299988
-5.9518456	12.9808006	103.5599976
-5.9518456	13.0296001	99.2200012
-5.9518456	13.0784006	104.0000000
-5.9518456	13.1272001	106.0199966
-5.9518456	13.1760006	105.7900009
-5.9518456	13.2248001	104.4399948
-5.9518456	13.2736006	104.3399963
-5.9518456	13.3224001	106.1100006
-5.9518456	13.3712006	104.9499969
-5.9518456	13.4200001	102.7900009
-5.9518456	13.4688005	101.7299957
-5.9518456	13.5176001	100.5000000
-5.9518456	13.5664005	101.3699951
-5.9518456	13.6152000	104.9300003
-5.9518456	13.6640005	105.1999969
-5.9518456	13.7128000	105.9000015
-5.9518456	13.7616005	104.8699951
-5.9518456	13.8104000	105.1199951
-5.9518456	13.8592005	106.1299973
-5.9518456	13.9080000	99.5199966
-5.9518456	13.9568005	101.5699997

-5.9518456	14.0056000	102.4599991
-5.9518456	14.0544004	103.6299973
-5.9518456	14.1032000	101.7699966
-5.9518456	14.1520004	102.4700012
-5.9518456	14.2007999	104.7600021
-5.9518456	14.2496004	104.7099991
-5.9518456	14.2983999	102.4000015
-5.9518456	14.3472004	102.5100021
-5.9518456	14.3959999	104.9099960
-5.9518456	14.4448004	105.0000000
-5.9518456	14.4935999	104.5199966
-5.9518456	14.5424004	104.6399994
-5.9518456	14.5911999	106.7099991
-5.9518456	14.6400003	106.4300003
-5.9518456	14.6887999	101.9499969
-5.9518456	14.7376003	100.1399994
-5.9518456	14.7863998	101.6299973
-5.9518456	14.8352003	105.0199966
-5.9518456	14.8839998	107.6399994
-5.9518456	14.9328003	109.1999969
-5.9518456	14.9815998	108.1100006
-5.9518456	15.0304003	105.5199966
-5.9457097	0.3416000	109.6399994
-5.9457097	0.3904000	109.2099991
-5.9457097	0.4392000	107.4799957
-5.9457097	0.4880000	107.9399948
-5.9457097	0.5368000	106.0000000
-5.9457097	0.5856000	105.3799973
-5.9457097	0.6344000	105.4700012
-5.9457097	0.6832000	102.5999985
-5.9457097	0.7320000	104.1299973
-5.9457097	0.7808000	106.0199966
-5.9457097	0.8296000	107.0899963
-5.9457097	0.8784000	103.9599991
-5.9457097	0.9272000	103.0999985
-5.9457097	0.9760000	103.6999969
-5.9457097	1.0247999	108.5999985
-5.9457097	1.0735999	106.8300018
-5.9457097	1.1223999	103.9799957
-5.9457097	1.1711999	102.6800003
-5.9457097	1.2200000	105.7200012
-5.9457097	1.2688000	106.5400009
-5.9457097	1.3176000	106.1899948
-5.9457097	1.3664000	106.3499985
-5.9457097	1.4152000	107.6399994
-5.9457097	1.4640000	108.1399994
-5.9457097	1.5128000	106.4799957
-5.9457097	1.5616000	107.6399994
-5.9457097	1.6104000	109.3699951
-5.9457097	1.6592000	108.1699982
-5.9457097	1.7079999	108.6299973
-5.9457097	1.7567999	104.1599960
-5.9457097	1.8055999	108.1399994
-5.9457097	1.8543999	116.1100006
-5.9457097	1.9031999	118.4599991
-5.9457097	1.9519999	115.1500015
-5.9457097	2.0007999	107.2200012
-5.9457097	2.0495999	104.7099991

-5.9457097	2.0983999	106.7699966
-5.9457097	2.1471999	106.1500015
-5.9457097	2.1959999	105.5400009
-5.9457097	2.2447999	104.6599960
-5.9457097	2.2935998	95.9300003
-5.9457097	2.3423998	98.0999985
-5.9457097	2.3912001	102.2999954
-5.9457097	2.4400001	103.4099960
-5.9457097	2.4888000	107.1399994
-5.9457097	2.5376000	106.2699966
-5.9457097	2.5864000	104.9700012
-5.9457097	2.6352000	105.4899979
-5.9457097	2.6840000	102.9599991
-5.9457097	2.7328000	105.7500000
-5.9457097	2.7816000	107.0299988
-5.9457097	2.8304000	108.5699997
-5.9457097	2.8792000	106.7900009
-5.9457097	2.9280000	106.1800003
-5.9457097	2.9768000	105.1500015
-5.9457097	3.0256000	106.3300018
-5.9457097	3.0743999	102.8899994
-5.9457097	3.1231999	103.1500015
-5.9457097	3.1719999	101.8199997
-5.9457097	3.2207999	99.3499985
-5.9457097	3.2695999	103.9599991
-5.9457097	3.3183999	104.0599976
-5.9457097	3.3671999	103.7799988
-5.9457097	3.4159999	102.9099960
-5.9457097	3.4647999	99.6699982
-5.9457097	3.5135999	99.7999954
-5.9457097	3.5623999	101.4799957
-5.9457097	3.6111999	104.8499985
-5.9457097	3.6599998	104.0499954
-5.9457097	3.7087998	102.5499954
-5.9457097	3.7575998	101.8300018
-5.9457097	3.8063998	101.7900009
-5.9457097	3.8551998	107.4700012
-5.9457097	3.9039998	107.4799957
-5.9457097	3.9527998	102.8899994
-5.9457097	4.0016003	100.6199951
-5.9457097	4.0503998	102.9799957
-5.9457097	4.0991998	103.7600021
-5.9457097	4.1479998	105.5499954
-5.9457097	4.1967998	104.7200012
-5.9457097	4.2455997	104.2699966
-5.9457097	4.2943997	108.2600021
-5.9457097	4.3431997	111.2600021
-5.9457097	4.3919997	111.1199951
-5.9457097	4.4407997	108.0000000
-5.9457097	4.4895997	109.2999954
-5.9457097	4.5383997	111.6699982
-5.9457097	4.5871997	113.4499969
-5.9457097	4.6359997	112.6800003
-5.9457097	4.6847997	114.4099960
-5.9457097	4.7336001	113.9300003
-5.9457097	4.7824001	108.5999985
-5.9457097	4.8312001	109.1599960
-5.9457097	4.8800001	109.9199982

-5.9457097	4.9288001	108.0199966
-5.9457097	4.9776001	105.9899979
-5.9457097	5.0264001	109.4099960
-5.9457097	5.0752001	110.3899994
-5.9457097	5.1240001	107.9000015
-5.9457097	5.1728001	103.1500015
-5.9457097	5.2216001	105.8300018
-5.9457097	5.2704000	104.8499985
-5.9457097	5.3192000	104.7999954
-5.9457097	5.3680000	106.4199982
-5.9457097	5.4168000	105.4000015
-5.9457097	5.4656000	101.5800018
-5.9457097	5.5144000	104.5999985
-5.9457097	5.5632000	107.0800018
-5.9457097	5.6120000	107.4099960
-5.9457097	5.6608000	108.8799973
-5.9457097	5.7096000	108.2299957
-5.9457097	5.7584000	104.8399963
-5.9457097	5.8072000	101.3499985
-5.9457097	5.8559999	103.6599960
-5.9457097	5.9047999	105.0400009
-5.9457097	5.9535999	106.9099960
-5.9457097	6.0023999	104.1199951
-5.9457097	6.0511999	106.1800003
-5.9457097	6.0999999	104.4700012
-5.9457097	6.1487999	103.0499954
-5.9457097	6.1975999	99.9499969
-5.9457097	6.2463999	102.0899963
-5.9457097	6.2951999	103.8699951
-5.9457097	6.3439999	102.9399948
-5.9457097	6.3927999	102.2699966
-5.9457097	6.4415998	104.5100021
-5.9457097	6.4903998	102.4300003
-5.9457097	6.5391998	98.9599991
-5.9457097	6.5879998	103.3300018
-5.9457097	6.6367998	103.9700012
-5.9457097	6.6855998	102.8300018
-5.9457097	6.7343998	103.0400009
-5.9457097	6.7831998	100.7999954
-5.9457097	6.8319998	104.5499954
-5.9457097	6.8807998	104.9799957
-5.9457097	6.9295998	106.0100021
-5.9457097	6.9783998	105.9700012
-5.9457097	7.0271997	105.1500015
-5.9457097	7.0759997	102.4599991
-5.9457097	7.1247997	102.5499954
-5.9457097	7.1735997	104.7099991
-5.9457097	7.2223997	107.1199951
-5.9457097	7.2711997	104.6299973
-5.9457097	7.3199997	100.3899994
-5.9457097	7.3687997	100.7200012
-5.9457097	7.4175997	98.3699951
-5.9457097	7.4663997	103.8899994
-5.9457097	7.5151997	102.9799957
-5.9457097	7.5639997	103.7999954
-5.9457097	7.6127996	105.7399979
-5.9457097	7.6615996	104.2999954
-5.9457097	7.7103996	103.7699966

-5.9457097	7.7591996	102.3300018
-5.9457097	7.8079996	104.1199951
-5.9457097	7.8567996	101.3499985
-5.9457097	7.9056001	104.3799973
-5.9457097	7.9544001	103.2600021
-5.9457097	8.0032005	102.1899948
-5.9457097	8.0520000	106.1800003
-5.9457097	8.1008005	106.8199997
-5.9457097	8.1496000	105.8600006
-5.9457097	8.1984005	106.4300003
-5.9457097	8.2472000	105.1599960
-5.9457097	8.2960005	104.4399948
-5.9457097	8.3448000	105.4300003
-5.9457097	8.3936005	106.1100006
-5.9457097	8.4424000	103.0800018
-5.9457097	8.4912004	100.4000015
-5.9457097	8.5400000	104.7500000
-5.9457097	8.5888004	100.3099976
-5.9457097	8.6375999	101.9899979
-5.9457097	8.6864004	104.7500000
-5.9457097	8.7351999	104.7099991
-5.9457097	8.7840004	103.2500000
-5.9457097	8.8327999	104.9799957
-5.9457097	8.8816004	105.1999969
-5.9457097	8.9303999	102.8399963
-5.9457097	8.9792004	99.3799973
-5.9457097	9.0279999	101.7999954
-5.9457097	9.0768003	104.9000015
-5.9457097	9.1255999	103.2600021
-5.9457097	9.1744003	100.0999985
-5.9457097	9.2231998	103.8099976
-5.9457097	9.2720003	102.7399979
-5.9457097	9.3207998	101.6699982
-5.9457097	9.3696003	102.2699966
-5.9457097	9.4184008	102.9899979
-5.9457097	9.4672003	102.7099991
-5.9457097	9.5160007	102.6800003
-5.9457097	9.5648003	103.3499985
-5.9457097	9.6136007	102.2399979
-5.9457097	9.6624002	102.0899963
-5.9457097	9.7112007	105.2799988
-5.9457097	9.7600002	104.5999985
-5.9457097	9.8088007	105.9099960
-5.9457097	9.8576002	103.7099991
-5.9457097	9.9064007	107.4000015
-5.9457097	9.9552002	106.9099960
-5.9457097	10.0040007	102.3199997
-5.9457097	10.0528002	102.0100021
-5.9457097	10.1016006	106.0899963
-5.9457097	10.1504002	105.4199982
-5.9457097	10.1992006	99.8699951
-5.9457097	10.2480001	100.7500000
-5.9457097	10.2968006	99.2200012
-5.9457097	10.3456001	98.7799988
-5.9457097	10.3944006	101.7399979
-5.9457097	10.4432001	103.1399994
-5.9457097	10.4920006	101.5800018
-5.9457097	10.5408001	97.5899963

-5.9457097	10.5896006	100.0100021
-5.9457097	10.6384001	103.9300003
-5.9457097	10.6872005	105.6199951
-5.9457097	10.7360001	104.0800018
-5.9457097	10.7848005	103.9799957
-5.9457097	10.8336000	101.4300003
-5.9457097	10.8824005	102.4300003
-5.9457097	10.9312000	102.9799957
-5.9457097	10.9800005	106.8600006
-5.9457097	11.0288000	105.7099991
-5.9457097	11.0776005	103.0599976
-5.9457097	11.1264000	99.5000000
-5.9457097	11.1752005	103.7099991
-5.9457097	11.2240000	104.4700012
-5.9457097	11.2728004	103.2799988
-5.9457097	11.3216000	104.1500015
-5.9457097	11.3704004	104.1500015
-5.9457097	11.4191999	104.7799988
-5.9457097	11.4680004	101.9499969
-5.9457097	11.5167999	103.2999954
-5.9457097	11.5656004	102.3899994
-5.9457097	11.6143999	100.2500000
-5.9457097	11.6632004	102.6500015
-5.9457097	11.7119999	103.4799957
-5.9457097	11.7608004	103.2200012
-5.9457097	11.8095999	102.4499969
-5.9457097	11.8584003	101.5699997
-5.9457097	11.9071999	101.2600021
-5.9457097	11.9560003	102.2699966
-5.9457097	12.0047998	100.7600021
-5.9457097	12.0536003	98.3499985
-5.9457097	12.1023998	94.7399979
-5.9457097	12.1512003	101.1800003
-5.9457097	12.1999998	102.7699966
-5.9457097	12.2488003	103.0999985
-5.9457097	12.2975998	101.4799957
-5.9457097	12.3464003	102.5299988
-5.9457097	12.3951998	103.9399948
-5.9457097	12.4440002	102.6199951
-5.9457097	12.4927998	103.9599991
-5.9457097	12.5416002	104.2500000
-5.9457097	12.5904007	103.9399948
-5.9457097	12.6392002	102.1599960
-5.9457097	12.6880007	103.5699997
-5.9457097	12.7368002	105.3899994
-5.9457097	12.7856007	106.3199997
-5.9457097	12.8344002	103.4199982
-5.9457097	12.8832006	104.1399994
-5.9457097	12.9320002	103.4099960
-5.9457097	12.9808006	103.1399994
-5.9457097	13.0296001	99.8600006
-5.9457097	13.0784006	103.2900009
-5.9457097	13.1272001	105.4399948
-5.9457097	13.1760006	105.9899979
-5.9457097	13.2248001	105.9099960
-5.9457097	13.2736006	105.8099976
-5.9457097	13.3224001	106.6500015
-5.9457097	13.3712006	104.8300018

-5.9457097	13.4200001	103.5599976
-5.9457097	13.4688005	101.7500000
-5.9457097	13.5176001	99.4899979
-5.9457097	13.5664005	100.7600021
-5.9457097	13.6152000	104.5000000
-5.9457097	13.6640005	105.7200012
-5.9457097	13.7128000	106.8699951
-5.9457097	13.7616005	106.1999969
-5.9457097	13.8104000	105.2099991
-5.9457097	13.8592005	105.6199951
-5.9457097	13.9080000	98.3600006
-5.9457097	13.9568005	100.0000000
-5.9457097	14.0056000	101.1800003
-5.9457097	14.0544004	103.2399979
-5.9457097	14.1032000	102.1500015
-5.9457097	14.1520004	103.0599976
-5.9457097	14.2007999	104.2999954
-5.9457097	14.2496004	104.6399994
-5.9457097	14.2983999	103.6299973
-5.9457097	14.3472004	103.6299973
-5.9457097	14.3959999	105.5499954
-5.9457097	14.4448004	105.5199966
-5.9457097	14.4935999	104.7200012
-5.9457097	14.5424004	104.9700012
-5.9457097	14.5911999	106.4000015
-5.9457097	14.6400003	106.3499985
-5.9457097	14.6887999	102.0899963
-5.9457097	14.7376003	99.4300003
-5.9457097	14.7863998	101.8399963
-5.9457097	14.8352003	104.4700012
-5.9457097	14.8839998	107.2799988
-5.9457097	14.9328003	108.9000015
-5.9457097	14.9815998	108.5800018
-5.9457097	15.0304003	106.5599976
-5.9395738	0.3416000	110.5299988
-5.9395738	0.3904000	110.1399994
-5.9395738	0.4392000	107.9000015
-5.9395738	0.4880000	107.4499969
-5.9395738	0.5368000	105.5299988
-5.9395738	0.5856000	107.7699966
-5.9395738	0.6344000	106.8300018
-5.9395738	0.6832000	102.6199951
-5.9395738	0.7320000	104.2399979
-5.9395738	0.7808000	105.6800003
-5.9395738	0.8296000	107.5199966
-5.9395738	0.8784000	105.2399979
-5.9395738	0.9272000	104.9899979
-5.9395738	0.9760000	105.1599960
-5.9395738	1.0247999	108.4700012
-5.9395738	1.0735999	105.7500000
-5.9395738	1.1223999	103.2500000
-5.9395738	1.1711999	101.8499985
-5.9395738	1.2200000	105.3099976
-5.9395738	1.2688000	106.4300003
-5.9395738	1.3176000	106.0899963
-5.9395738	1.3664000	105.5199966
-5.9395738	1.4152000	106.7500000
-5.9395738	1.4640000	107.5899963

-5.9395738	1.5128000	106.4199982
-5.9395738	1.5616000	108.0199966
-5.9395738	1.6104000	109.5100021
-5.9395738	1.6592000	109.6899948
-5.9395738	1.7079999	110.6299973
-5.9395738	1.7567999	105.4899979
-5.9395738	1.8055999	106.8899994
-5.9395738	1.8543999	115.7099991
-5.9395738	1.9031999	118.1100006
-5.9395738	1.9519999	114.7999954
-5.9395738	2.0007999	107.9499969
-5.9395738	2.0495999	105.5499954
-5.9395738	2.0983999	106.9899979
-5.9395738	2.1471999	105.9799957
-5.9395738	2.1959999	105.9300003
-5.9395738	2.2447999	105.2099991
-5.9395738	2.2935998	96.8300018
-5.9395738	2.3423998	98.8600006
-5.9395738	2.3912001	102.3799973
-5.9395738	2.4400001	104.1599960
-5.9395738	2.4888000	107.2900009
-5.9395738	2.5376000	106.3399963
-5.9395738	2.5864000	105.5699997
-5.9395738	2.6352000	105.8099976
-5.9395738	2.6840000	103.7999954
-5.9395738	2.7328000	105.8399963
-5.9395738	2.7816000	106.7600021
-5.9395738	2.8304000	108.0699997
-5.9395738	2.8792000	107.2299957
-5.9395738	2.9280000	105.9599991
-5.9395738	2.9768000	105.8600006
-5.9395738	3.0256000	106.2699966
-5.9395738	3.0743999	102.5000000
-5.9395738	3.1231999	102.3399963
-5.9395738	3.1719999	101.5100021
-5.9395738	3.2207999	99.1899948
-5.9395738	3.2695999	103.6399994
-5.9395738	3.3183999	102.4099960
-5.9395738	3.3671999	103.1599960
-5.9395738	3.4159999	103.1899948
-5.9395738	3.4647999	101.5899963
-5.9395738	3.5135999	101.5199966
-5.9395738	3.5623999	102.4899979
-5.9395738	3.6111999	105.4700012
-5.9395738	3.6599998	104.6299973
-5.9395738	3.7087998	102.0599976
-5.9395738	3.7575998	101.2200012
-5.9395738	3.8063998	100.7399979
-5.9395738	3.8551998	106.4000015
-5.9395738	3.9039998	107.0599976
-5.9395738	3.9527998	102.5699997
-5.9395738	4.0016003	101.8099976
-5.9395738	4.0503998	102.0000000
-5.9395738	4.0991998	102.7600021
-5.9395738	4.1479998	105.4000015
-5.9395738	4.1967998	105.0400009
-5.9395738	4.2455997	104.1100006
-5.9395738	4.2943997	108.3300018

-5.9395738	4.3431997	111.0999985
-5.9395738	4.3919997	110.7500000
-5.9395738	4.4407997	107.4099960
-5.9395738	4.4895997	109.2099991
-5.9395738	4.5383997	111.8799973
-5.9395738	4.5871997	113.1899948
-5.9395738	4.6359997	111.9599991
-5.9395738	4.6847997	114.7600021
-5.9395738	4.7336001	114.3199997
-5.9395738	4.7824001	108.6500015
-5.9395738	4.8312001	109.2399979
-5.9395738	4.8800001	109.2299957
-5.9395738	4.9288001	107.6999969
-5.9395738	4.9776001	105.7399979
-5.9395738	5.0264001	108.8699951
-5.9395738	5.0752001	109.6599960
-5.9395738	5.1240001	107.1299973
-5.9395738	5.1728001	102.9399948
-5.9395738	5.2216001	105.6599960
-5.9395738	5.2704000	105.1299973
-5.9395738	5.3192000	104.9199982
-5.9395738	5.3680000	106.5599976
-5.9395738	5.4168000	105.9300003
-5.9395738	5.4656000	101.2099991
-5.9395738	5.5144000	104.6800003
-5.9395738	5.5632000	106.6999969
-5.9395738	5.6120000	106.8300018
-5.9395738	5.6608000	108.6500015
-5.9395738	5.7096000	108.5599976
-5.9395738	5.7584000	104.7699966
-5.9395738	5.8072000	101.8799973
-5.9395738	5.8559999	103.8699951
-5.9395738	5.9047999	105.3899994
-5.9395738	5.9535999	107.3600006
-5.9395738	6.0023999	104.6999969
-5.9395738	6.0511999	106.2399979
-5.9395738	6.0999999	104.8799973
-5.9395738	6.1487999	103.9000015
-5.9395738	6.1975999	101.4700012
-5.9395738	6.2463999	102.2699966
-5.9395738	6.2951999	103.8699951
-5.9395738	6.3439999	102.9199982
-5.9395738	6.3927999	103.4899979
-5.9395738	6.4415998	104.4599991
-5.9395738	6.4903998	101.0899963
-5.9395738	6.5391998	98.8399963
-5.9395738	6.5879998	102.7299957
-5.9395738	6.6367998	104.6800003
-5.9395738	6.6855998	102.6999969
-5.9395738	6.7343998	103.0299988
-5.9395738	6.7831998	99.9000015
-5.9395738	6.8319998	104.2399979
-5.9395738	6.8807998	105.4399948
-5.9395738	6.9295998	106.9399948
-5.9395738	6.9783998	107.1100006
-5.9395738	7.0271997	105.9499969
-5.9395738	7.0759997	103.0000000
-5.9395738	7.1247997	102.5000000

-5.9395738	7.1735997	104.5299988
-5.9395738	7.2223997	107.3099976
-5.9395738	7.2711997	105.3399963
-5.9395738	7.3199997	100.6599960
-5.9395738	7.3687997	100.4599991
-5.9395738	7.4175997	98.9300003
-5.9395738	7.4663997	103.9799957
-5.9395738	7.5151997	102.3099976
-5.9395738	7.5639997	103.3799973
-5.9395738	7.6127996	105.4700012
-5.9395738	7.6615996	103.8699951
-5.9395738	7.7103996	104.1299973
-5.9395738	7.7591996	103.5999985
-5.9395738	7.8079996	104.4799957
-5.9395738	7.8567996	102.3899994
-5.9395738	7.9056001	104.2399979
-5.9395738	7.9544001	102.9199982
-5.9395738	8.0032005	101.1999969
-5.9395738	8.0520000	105.7900009
-5.9395738	8.1008005	107.0199966
-5.9395738	8.1496000	106.4000015
-5.9395738	8.1984005	106.9300003
-5.9395738	8.2472000	105.0199966
-5.9395738	8.2960005	104.5000000
-5.9395738	8.3448000	106.1100006
-5.9395738	8.3936005	105.5299988
-5.9395738	8.4424000	102.4300003
-5.9395738	8.4912004	101.1899948
-5.9395738	8.5400000	105.2500000
-5.9395738	8.5888004	101.2799988
-5.9395738	8.6375999	101.7999954
-5.9395738	8.6864004	104.5499954
-5.9395738	8.7351999	104.4700012
-5.9395738	8.7840004	102.7900009
-5.9395738	8.8327999	104.0800018
-5.9395738	8.8816004	105.2299957
-5.9395738	8.9303999	103.9499969
-5.9395738	8.9792004	100.5899963
-5.9395738	9.0279999	102.4899979
-5.9395738	9.0768003	105.0899963
-5.9395738	9.1255999	102.1999969
-5.9395738	9.1744003	100.5100021
-5.9395738	9.2231998	104.0899963
-5.9395738	9.2720003	102.5599976
-5.9395738	9.3207998	101.9700012
-5.9395738	9.3696003	102.3199997
-5.9395738	9.4184008	103.5699997
-5.9395738	9.4672003	103.1800003
-5.9395738	9.5160007	101.5499954
-5.9395738	9.5648003	102.2600021
-5.9395738	9.6136007	102.1800003
-5.9395738	9.6624002	102.4499969
-5.9395738	9.7112007	104.7799988
-5.9395738	9.7600002	103.5400009
-5.9395738	9.8088007	104.8300018
-5.9395738	9.8576002	104.5800018
-5.9395738	9.9064007	107.5999985
-5.9395738	9.9552002	106.6699982

-5.9395738	10.0040007	103.0199966
-5.9395738	10.0528002	100.6999969
-5.9395738	10.1016006	104.9099960
-5.9395738	10.1504002	105.0100021
-5.9395738	10.1992006	100.8699951
-5.9395738	10.2480001	99.9399948
-5.9395738	10.2968006	98.3699951
-5.9395738	10.3456001	98.8799973
-5.9395738	10.3944006	102.4300003
-5.9395738	10.4432001	103.1999969
-5.9395738	10.4920006	100.7799988
-5.9395738	10.5408001	96.6899948
-5.9395738	10.5896006	100.6500015
-5.9395738	10.6384001	104.2200012
-5.9395738	10.6872005	104.3799973
-5.9395738	10.7360001	102.6299973
-5.9395738	10.7848005	103.8899994
-5.9395738	10.8336000	102.3799973
-5.9395738	10.8824005	103.8600006
-5.9395738	10.9312000	104.3300018
-5.9395738	10.9800005	105.9899979
-5.9395738	11.0288000	105.3399963
-5.9395738	11.0776005	103.8499985
-5.9395738	11.1264000	100.2299957
-5.9395738	11.1752005	103.8899994
-5.9395738	11.2240000	103.5299988
-5.9395738	11.2728004	103.1699982
-5.9395738	11.3216000	103.9700012
-5.9395738	11.3704004	104.0299988
-5.9395738	11.4191999	104.6100006
-5.9395738	11.4680004	102.8300018
-5.9395738	11.5167999	103.0999985
-5.9395738	11.5656004	102.0400009
-5.9395738	11.6143999	100.4499969
-5.9395738	11.6632004	102.0899963
-5.9395738	11.7119999	103.4599991
-5.9395738	11.7608004	102.6800003
-5.9395738	11.8095999	102.3699951
-5.9395738	11.8584003	101.4300003
-5.9395738	11.9071999	100.6500015
-5.9395738	11.9560003	102.3099976
-5.9395738	12.0047998	101.3699951
-5.9395738	12.0536003	99.8499985
-5.9395738	12.1023998	97.8399963
-5.9395738	12.1512003	102.6399994
-5.9395738	12.1999998	102.9099960
-5.9395738	12.2488003	103.0800018
-5.9395738	12.2975998	101.5499954
-5.9395738	12.3464003	102.3199997
-5.9395738	12.3951998	103.5599976
-5.9395738	12.4440002	102.7900009
-5.9395738	12.4927998	104.7500000
-5.9395738	12.5416002	104.8199997
-5.9395738	12.5904007	102.9399948
-5.9395738	12.6392002	101.3099976
-5.9395738	12.6880007	103.5000000
-5.9395738	12.7368002	105.2999954
-5.9395738	12.7856007	106.2900009

-5.9395738	12.8344002	104.1199951
-5.9395738	12.8832006	104.1899948
-5.9395738	12.9320002	102.8499985
-5.9395738	12.9808006	102.8099976
-5.9395738	13.0296001	100.6899948
-5.9395738	13.0784006	102.6800003
-5.9395738	13.1272001	105.0400009
-5.9395738	13.1760006	106.3399963
-5.9395738	13.2248001	106.2299957
-5.9395738	13.2736006	106.5000000
-5.9395738	13.3224001	106.0800018
-5.9395738	13.3712006	104.1999969
-5.9395738	13.4200001	103.6899948
-5.9395738	13.4688005	101.3899994
-5.9395738	13.5176001	97.9199982
-5.9395738	13.5664005	99.8799973
-5.9395738	13.6152000	103.7299957
-5.9395738	13.6640005	105.8199997
-5.9395738	13.7128000	107.3499985
-5.9395738	13.7616005	107.1199951
-5.9395738	13.8104000	104.9099960
-5.9395738	13.8592005	104.7500000
-5.9395738	13.9080000	97.3799973
-5.9395738	13.9568005	99.2600021
-5.9395738	14.0056000	100.1899948
-5.9395738	14.0544004	102.7200012
-5.9395738	14.1032000	102.2799988
-5.9395738	14.1520004	103.1699982
-5.9395738	14.2007999	103.5499954
-5.9395738	14.2496004	104.3399963
-5.9395738	14.2983999	104.9700012
-5.9395738	14.3472004	105.0000000
-5.9395738	14.3959999	105.9000015
-5.9395738	14.4448004	105.6599960
-5.9395738	14.4935999	104.5999985
-5.9395738	14.5424004	104.5899963
-5.9395738	14.5911999	105.5599976
-5.9395738	14.6400003	106.0599976
-5.9395738	14.6887999	102.5999985
-5.9395738	14.7376003	99.7099991
-5.9395738	14.7863998	102.5400009
-5.9395738	14.8352003	104.2500000
-5.9395738	14.8839998	106.5499954
-5.9395738	14.9328003	107.4199982
-5.9395738	14.9815998	108.3899994
-5.9395738	15.0304003	107.0800018
-5.9334378	0.3416000	110.6999969
-5.9334378	0.3904000	110.2500000
-5.9334378	0.4392000	107.7699966
-5.9334378	0.4880000	106.5000000
-5.9334378	0.5368000	104.1599960
-5.9334378	0.5856000	107.3199997
-5.9334378	0.6344000	107.0400009
-5.9334378	0.6832000	101.5999985
-5.9334378	0.7320000	104.4199982
-5.9334378	0.7808000	106.2999954
-5.9334378	0.8296000	108.5499954
-5.9334378	0.8784000	107.2299957

-5.9334378	0.9272000	107.0599976
-5.9334378	0.9760000	106.4599991
-5.9334378	1.0247999	108.2399979
-5.9334378	1.0735999	104.7799988
-5.9334378	1.1223999	103.3899994
-5.9334378	1.1711999	101.6800003
-5.9334378	1.2200000	105.0199966
-5.9334378	1.2688000	106.5100021
-5.9334378	1.3176000	106.1699982
-5.9334378	1.3664000	105.1500015
-5.9334378	1.4152000	106.0899963
-5.9334378	1.4640000	107.1299973
-5.9334378	1.5128000	106.9599991
-5.9334378	1.5616000	108.7900009
-5.9334378	1.6104000	109.9399948
-5.9334378	1.6592000	111.0499954
-5.9334378	1.7079999	112.0100021
-5.9334378	1.7567999	107.3600006
-5.9334378	1.8055999	105.5899963
-5.9334378	1.8543999	115.5199966
-5.9334378	1.9031999	118.0999985
-5.9334378	1.9519999	114.6999969
-5.9334378	2.0007999	108.2299957
-5.9334378	2.0495999	105.8199997
-5.9334378	2.0983999	106.8099976
-5.9334378	2.1471999	105.6599960
-5.9334378	2.1959999	106.4199982
-5.9334378	2.2447999	105.5899963
-5.9334378	2.2935998	98.9399948
-5.9334378	2.3423998	99.9000015
-5.9334378	2.3912001	103.1199951
-5.9334378	2.4400001	105.3199997
-5.9334378	2.4888000	107.4799957
-5.9334378	2.5376000	106.3399963
-5.9334378	2.5864000	105.7399979
-5.9334378	2.6352000	106.1500015
-5.9334378	2.6840000	105.1500015
-5.9334378	2.7328000	106.0899963
-5.9334378	2.7816000	105.7600021
-5.9334378	2.8304000	107.2399979
-5.9334378	2.8792000	107.8099976
-5.9334378	2.9280000	106.5999985
-5.9334378	2.9768000	107.4199982
-5.9334378	3.0256000	107.0999985
-5.9334378	3.0743999	102.8199997
-5.9334378	3.1231999	101.7200012
-5.9334378	3.1719999	101.3899994
-5.9334378	3.2207999	100.3600006
-5.9334378	3.2695999	103.9000015
-5.9334378	3.3183999	101.1299973
-5.9334378	3.3671999	102.9199982
-5.9334378	3.4159999	103.7200012
-5.9334378	3.4647999	102.9300003
-5.9334378	3.5135999	103.3199997
-5.9334378	3.5623999	103.5599976
-5.9334378	3.6111999	105.5199966
-5.9334378	3.6599998	105.0199966
-5.9334378	3.7087998	101.9199982

-5.9334378	3.7575998	101.6399994
-5.9334378	3.8063998	100.9700012
-5.9334378	3.8551998	105.7999954
-5.9334378	3.9039998	106.6899948
-5.9334378	3.9527998	102.5699997
-5.9334378	4.0016003	102.4199982
-5.9334378	4.0503998	100.6699982
-5.9334378	4.0991998	102.7900009
-5.9334378	4.1479998	105.1899948
-5.9334378	4.1967998	105.0000000
-5.9334378	4.2455997	103.8099976
-5.9334378	4.2943997	108.1699982
-5.9334378	4.3431997	110.8699951
-5.9334378	4.3919997	110.4899979
-5.9334378	4.4407997	107.0100021
-5.9334378	4.4895997	109.0199966
-5.9334378	4.5383997	112.4499969
-5.9334378	4.5871997	113.7900009
-5.9334378	4.6359997	112.5299988
-5.9334378	4.6847997	115.5699997
-5.9334378	4.7336001	114.6599960
-5.9334378	4.7824001	108.4799957
-5.9334378	4.8312001	108.8199997
-5.9334378	4.8800001	108.2099991
-5.9334378	4.9288001	107.2200012
-5.9334378	4.9776001	105.5400009
-5.9334378	5.0264001	108.1800003
-5.9334378	5.0752001	108.4199982
-5.9334378	5.1240001	105.8099976
-5.9334378	5.1728001	102.0899963
-5.9334378	5.2216001	105.2299957
-5.9334378	5.2704000	105.3600006
-5.9334378	5.3192000	105.2900009
-5.9334378	5.3680000	106.5499954
-5.9334378	5.4168000	105.8399963
-5.9334378	5.4656000	100.6500015
-5.9334378	5.5144000	104.5999985
-5.9334378	5.5632000	106.1100006
-5.9334378	5.6120000	105.9000015
-5.9334378	5.6608000	108.1699982
-5.9334378	5.7096000	108.4300003
-5.9334378	5.7584000	104.4099960
-5.9334378	5.8072000	102.7200012
-5.9334378	5.8559999	104.5599976
-5.9334378	5.9047999	105.9099960
-5.9334378	5.9535999	107.2500000
-5.9334378	6.0023999	105.2600021
-5.9334378	6.0511999	106.6399994
-5.9334378	6.0999999	105.2399979
-5.9334378	6.1487999	104.4000015
-5.9334378	6.1975999	102.5499954
-5.9334378	6.2463999	102.7399979
-5.9334378	6.2951999	104.0499954
-5.9334378	6.3439999	102.7699966
-5.9334378	6.3927999	103.9399948
-5.9334378	6.4415998	103.8600006
-5.9334378	6.4903998	99.9199982
-5.9334378	6.5391998	99.2699966

-5.9334378	6.5879998	101.9000015
-5.9334378	6.6367998	104.1999969
-5.9334378	6.6855998	102.8699951
-5.9334378	6.7343998	103.5100021
-5.9334378	6.7831998	99.6999969
-5.9334378	6.8319998	103.9700012
-5.9334378	6.8807998	105.5699997
-5.9334378	6.9295998	107.7999954
-5.9334378	6.9783998	108.1899948
-5.9334378	7.0271997	106.1299973
-5.9334378	7.0759997	103.5999985
-5.9334378	7.1247997	102.1899948
-5.9334378	7.1735997	103.4499969
-5.9334378	7.2223997	106.7200012
-5.9334378	7.2711997	105.3399963
-5.9334378	7.3199997	101.0499954
-5.9334378	7.3687997	100.5400009
-5.9334378	7.4175997	99.3699951
-5.9334378	7.4663997	103.6899948
-5.9334378	7.5151997	101.3199997
-5.9334378	7.5639997	102.6399994
-5.9334378	7.6127996	105.0699997
-5.9334378	7.6615996	103.8300018
-5.9334378	7.7103996	104.5599976
-5.9334378	7.7591996	104.0899963
-5.9334378	7.8079996	104.3899994
-5.9334378	7.8567996	103.1599960
-5.9334378	7.9056001	104.0599976
-5.9334378	7.9544001	102.7900009
-5.9334378	8.0032005	100.4399948
-5.9334378	8.0520000	105.4099960
-5.9334378	8.1008005	107.0299988
-5.9334378	8.1496000	106.7500000
-5.9334378	8.1984005	106.7600021
-5.9334378	8.2472000	105.3099976
-5.9334378	8.2960005	105.0999985
-5.9334378	8.3448000	106.0299988
-5.9334378	8.3936005	104.3600006
-5.9334378	8.4424000	101.5199966
-5.9334378	8.4912004	101.3399963
-5.9334378	8.5400000	105.8399963
-5.9334378	8.5888004	103.0699997
-5.9334378	8.6375999	102.0400009
-5.9334378	8.6864004	104.2299957
-5.9334378	8.7351999	103.8300018
-5.9334378	8.7840004	102.0899963
-5.9334378	8.8327999	102.8300018
-5.9334378	8.8816004	104.9300003
-5.9334378	8.9303999	104.6199951
-5.9334378	8.9792004	101.1599960
-5.9334378	9.0279999	102.8899994
-5.9334378	9.0768003	105.2999954
-5.9334378	9.1255999	101.8899994
-5.9334378	9.1744003	101.0199966
-5.9334378	9.2231998	104.3099976
-5.9334378	9.2720003	102.9399948
-5.9334378	9.3207998	102.6800003
-5.9334378	9.3696003	101.4199982

-5.9334378	9.4184008	103.6800003
-5.9334378	9.4672003	103.1299973
-5.9334378	9.5160007	100.5999985
-5.9334378	9.5648003	101.4799957
-5.9334378	9.6136007	101.7600021
-5.9334378	9.6624002	102.8399963
-5.9334378	9.7112007	104.5499954
-5.9334378	9.7600002	102.9599991
-5.9334378	9.8088007	104.5699997
-5.9334378	9.8576002	106.1100006
-5.9334378	9.9064007	107.5499954
-5.9334378	9.9552002	105.6299973
-5.9334378	10.0040007	103.6500015
-5.9334378	10.0528002	100.3600006
-5.9334378	10.1016006	103.6199951
-5.9334378	10.1504002	103.9599991
-5.9334378	10.1992006	101.4300003
-5.9334378	10.2480001	99.4099960
-5.9334378	10.2968006	98.0800018
-5.9334378	10.3456001	99.3499985
-5.9334378	10.3944006	103.6199951
-5.9334378	10.4432001	104.1899948
-5.9334378	10.4920006	100.8499985
-5.9334378	10.5408001	97.6100006
-5.9334378	10.5896006	101.2500000
-5.9334378	10.6384001	104.2999954
-5.9334378	10.6872005	103.5599976
-5.9334378	10.7360001	102.5000000
-5.9334378	10.7848005	103.6999969
-5.9334378	10.8336000	102.8300018
-5.9334378	10.8824005	104.7500000
-5.9334378	10.9312000	105.5100021
-5.9334378	10.9800005	104.8199997
-5.9334378	11.0288000	104.6599960
-5.9334378	11.0776005	104.8199997
-5.9334378	11.1264000	101.7600021
-5.9334378	11.1752005	103.6699982
-5.9334378	11.2240000	102.1199951
-5.9334378	11.2728004	103.4700012
-5.9334378	11.3216000	104.5699997
-5.9334378	11.3704004	104.3099976
-5.9334378	11.4191999	104.1999969
-5.9334378	11.4680004	102.3099976
-5.9334378	11.5167999	102.2500000
-5.9334378	11.5656004	101.4199982
-5.9334378	11.6143999	100.7500000
-5.9334378	11.6632004	101.7200012
-5.9334378	11.7119999	103.3300018
-5.9334378	11.7608004	102.2999954
-5.9334378	11.8095999	102.3399963
-5.9334378	11.8584003	100.6100006
-5.9334378	11.9071999	100.0199966
-5.9334378	11.9560003	102.5000000
-5.9334378	12.0047998	102.3499985
-5.9334378	12.0536003	101.7699966
-5.9334378	12.1023998	100.5400009
-5.9334378	12.1512003	103.5000000
-5.9334378	12.1999998	102.8199997

-5.9334378	12.2488003	103.4300003
-5.9334378	12.2975998	102.4899979
-5.9334378	12.3464003	103.1899948
-5.9334378	12.3951998	103.4700012
-5.9334378	12.4440002	103.5299988
-5.9334378	12.4927998	105.5000000
-5.9334378	12.5416002	104.6199951
-5.9334378	12.5904007	101.7699966
-5.9334378	12.6392002	100.7900009
-5.9334378	12.6880007	103.4599991
-5.9334378	12.7368002	104.1399994
-5.9334378	12.7856007	105.4899979
-5.9334378	12.8344002	104.5400009
-5.9334378	12.8832006	104.0000000
-5.9334378	12.9320002	103.0800018
-5.9334378	12.9808006	103.0100021
-5.9334378	13.0296001	101.8499985
-5.9334378	13.0784006	102.7099991
-5.9334378	13.1272001	104.0800018
-5.9334378	13.1760006	105.6800003
-5.9334378	13.2248001	105.4799957
-5.9334378	13.2736006	106.2799988
-5.9334378	13.3224001	104.9799957
-5.9334378	13.3712006	103.4199982
-5.9334378	13.4200001	102.9199982
-5.9334378	13.4688005	101.1500015
-5.9334378	13.5176001	96.6500015
-5.9334378	13.5664005	99.4700012
-5.9334378	13.6152000	103.4599991
-5.9334378	13.6640005	105.5299988
-5.9334378	13.7128000	107.8099976
-5.9334378	13.7616005	107.7099991
-5.9334378	13.8104000	104.0400009
-5.9334378	13.8592005	103.1599960
-5.9334378	13.9080000	97.8499985
-5.9334378	13.9568005	99.0499954
-5.9334378	14.0056000	99.5499954
-5.9334378	14.0544004	102.0699997
-5.9334378	14.1032000	101.8499985
-5.9334378	14.1520004	102.7500000
-5.9334378	14.2007999	102.2699966
-5.9334378	14.2496004	103.4700012
-5.9334378	14.2983999	105.8699951
-5.9334378	14.3472004	106.0699997
-5.9334378	14.3959999	105.9399948
-5.9334378	14.4448004	105.5699997
-5.9334378	14.4935999	104.0899963
-5.9334378	14.5424004	103.8799973
-5.9334378	14.5911999	104.2799988
-5.9334378	14.6400003	105.5100021
-5.9334378	14.6887999	103.4499969
-5.9334378	14.7376003	100.3899994
-5.9334378	14.7863998	103.0800018
-5.9334378	14.8352003	104.0299988
-5.9334378	14.8839998	105.5899963
-5.9334378	14.9328003	105.8699951
-5.9334378	14.9815998	107.8399963
-5.9334378	15.0304003	106.6599960

-5.9273019	0.3416000	110.5400009
-5.9273019	0.3904000	110.0599976
-5.9273019	0.4392000	107.7099991
-5.9273019	0.4880000	105.9199982
-5.9273019	0.5368000	102.5100021
-5.9273019	0.5856000	105.9700012
-5.9273019	0.6344000	107.0599976
-5.9273019	0.6832000	101.2500000
-5.9273019	0.7320000	104.6699982
-5.9273019	0.7808000	106.6299973
-5.9273019	0.8296000	109.0699997
-5.9273019	0.8784000	109.0000000
-5.9273019	0.9272000	109.1100006
-5.9273019	0.9760000	107.7699966
-5.9273019	1.0247999	108.2299957
-5.9273019	1.0735999	104.7900009
-5.9273019	1.1223999	104.3600006
-5.9273019	1.1711999	102.4399948
-5.9273019	1.2200000	105.2699966
-5.9273019	1.2688000	106.7200012
-5.9273019	1.3176000	106.5499954
-5.9273019	1.3664000	105.2600021
-5.9273019	1.4152000	106.1999969
-5.9273019	1.4640000	107.2299957
-5.9273019	1.5128000	107.8699951
-5.9273019	1.5616000	109.8899994
-5.9273019	1.6104000	110.2699966
-5.9273019	1.6592000	111.9399948
-5.9273019	1.7079999	112.7900009
-5.9273019	1.7567999	108.9199982
-5.9273019	1.8055999	105.0299988
-5.9273019	1.8543999	115.3499985
-5.9273019	1.9031999	117.9799957
-5.9273019	1.9519999	114.5000000
-5.9273019	2.0007999	108.6199951
-5.9273019	2.0495999	106.8499985
-5.9273019	2.0983999	107.2600021
-5.9273019	2.1471999	105.2500000
-5.9273019	2.1959999	106.4599991
-5.9273019	2.2447999	105.8799973
-5.9273019	2.2935998	101.7299957
-5.9273019	2.3423998	101.1599960
-5.9273019	2.3912001	103.6299973
-5.9273019	2.4400001	105.2799988
-5.9273019	2.4888000	107.4099960
-5.9273019	2.5376000	106.2099991
-5.9273019	2.5864000	105.7799988
-5.9273019	2.6352000	106.4099960
-5.9273019	2.6840000	105.9700012
-5.9273019	2.7328000	106.2099991
-5.9273019	2.7816000	105.5000000
-5.9273019	2.8304000	106.9700012
-5.9273019	2.8792000	108.3799973
-5.9273019	2.9280000	107.0100021
-5.9273019	2.9768000	107.2399979
-5.9273019	3.0256000	107.0199966
-5.9273019	3.0743999	102.8199997
-5.9273019	3.1231999	101.8399963

-5.9273019	3.1719999	101.0100021
-5.9273019	3.2207999	101.5899963
-5.9273019	3.2695999	104.4199982
-5.9273019	3.3183999	101.0400009
-5.9273019	3.3671999	102.8499985
-5.9273019	3.4159999	104.4499969
-5.9273019	3.4647999	104.3600006
-5.9273019	3.5135999	105.0299988
-5.9273019	3.5623999	104.0899963
-5.9273019	3.6111999	105.2699966
-5.9273019	3.6599998	104.9799957
-5.9273019	3.7087998	101.9799957
-5.9273019	3.7575998	102.7399979
-5.9273019	3.8063998	101.6999969
-5.9273019	3.8551998	105.2099991
-5.9273019	3.9039998	106.2399979
-5.9273019	3.9527998	103.3099976
-5.9273019	4.0016003	102.9899979
-5.9273019	4.0503998	99.9700012
-5.9273019	4.0991998	103.0100021
-5.9273019	4.1479998	105.0400009
-5.9273019	4.1967998	104.9099960
-5.9273019	4.2455997	103.7399979
-5.9273019	4.2943997	108.0000000
-5.9273019	4.3431997	110.5699997
-5.9273019	4.3919997	110.2999954
-5.9273019	4.4407997	106.7200012
-5.9273019	4.4895997	109.3399963
-5.9273019	4.5383997	112.9700012
-5.9273019	4.5871997	113.6299973
-5.9273019	4.6359997	112.5800018
-5.9273019	4.6847997	116.0199966
-5.9273019	4.7336001	114.7399979
-5.9273019	4.7824001	108.1199951
-5.9273019	4.8312001	108.1800003
-5.9273019	4.8800001	107.1999969
-5.9273019	4.9288001	106.5999985
-5.9273019	4.9776001	105.4899979
-5.9273019	5.0264001	107.7999954
-5.9273019	5.0752001	107.3499985
-5.9273019	5.1240001	105.0100021
-5.9273019	5.1728001	101.0699997
-5.9273019	5.2216001	103.6899948
-5.9273019	5.2704000	105.3899994
-5.9273019	5.3192000	105.0999985
-5.9273019	5.3680000	106.1199951
-5.9273019	5.4168000	105.4700012
-5.9273019	5.4656000	99.8300018
-5.9273019	5.5144000	104.0999985
-5.9273019	5.5632000	105.9199982
-5.9273019	5.6120000	105.8300018
-5.9273019	5.6608000	107.7399979
-5.9273019	5.7096000	108.3199997
-5.9273019	5.7584000	104.4000015
-5.9273019	5.8072000	103.5100021
-5.9273019	5.8559999	105.3099976
-5.9273019	5.9047999	106.6699982
-5.9273019	5.9535999	107.3099976

-5.9273019	6.0023999	106.0199966
-5.9273019	6.0511999	106.8199997
-5.9273019	6.0999999	105.5499954
-5.9273019	6.1487999	104.6800003
-5.9273019	6.1975999	102.2699966
-5.9273019	6.2463999	103.5299988
-5.9273019	6.2951999	104.6699982
-5.9273019	6.3439999	102.7900009
-5.9273019	6.3927999	104.4599991
-5.9273019	6.4415998	103.5800018
-5.9273019	6.4903998	99.9399948
-5.9273019	6.5391998	100.8499985
-5.9273019	6.5879998	101.1899948
-5.9273019	6.6367998	103.8399963
-5.9273019	6.6855998	103.4099960
-5.9273019	6.7343998	103.8399963
-5.9273019	6.7831998	100.1699982
-5.9273019	6.8319998	103.6699982
-5.9273019	6.8807998	105.3199997
-5.9273019	6.9295998	107.9000015
-5.9273019	6.9783998	108.4099960
-5.9273019	7.0271997	105.5899963
-5.9273019	7.0759997	103.5100021
-5.9273019	7.1247997	101.6899948
-5.9273019	7.1735997	102.5000000
-5.9273019	7.2223997	106.1899948
-5.9273019	7.2711997	105.4899979
-5.9273019	7.3199997	102.5000000
-5.9273019	7.3687997	102.1699982
-5.9273019	7.4175997	100.5699997
-5.9273019	7.4663997	104.0100021
-5.9273019	7.5151997	100.1699982
-5.9273019	7.5639997	101.6899948
-5.9273019	7.6127996	104.9799957
-5.9273019	7.6615996	104.9300003
-5.9273019	7.7103996	105.0499954
-5.9273019	7.7591996	104.3799973
-5.9273019	7.8079996	105.0800018
-5.9273019	7.8567996	104.1999969
-5.9273019	7.9056001	103.7600021
-5.9273019	7.9544001	102.3600006
-5.9273019	8.0032005	99.9199982
-5.9273019	8.0520000	105.1599960
-5.9273019	8.1008005	106.3199997
-5.9273019	8.1496000	106.7999954
-5.9273019	8.1984005	106.5499954
-5.9273019	8.2472000	105.5400009
-5.9273019	8.2960005	105.4599991
-5.9273019	8.3448000	105.7600021
-5.9273019	8.3936005	103.2999954
-5.9273019	8.4424000	101.0899963
-5.9273019	8.4912004	101.5699997
-5.9273019	8.5400000	105.7299957
-5.9273019	8.5888004	103.5199966
-5.9273019	8.6375999	101.2999954
-5.9273019	8.6864004	103.2200012
-5.9273019	8.7351999	103.2900009
-5.9273019	8.7840004	101.9300003

-5.9273019	8.8327999	101.4799957
-5.9273019	8.8816004	104.3099976
-5.9273019	8.9303999	104.9799957
-5.9273019	8.9792004	101.8099976
-5.9273019	9.0279999	103.3699951
-5.9273019	9.0768003	105.4000015
-5.9273019	9.1255999	101.3199997
-5.9273019	9.1744003	100.9899979
-5.9273019	9.2231998	104.3499985
-5.9273019	9.2720003	103.6800003
-5.9273019	9.3207998	103.6899948
-5.9273019	9.3696003	100.9499969
-5.9273019	9.4184008	103.4799957
-5.9273019	9.4672003	103.5499954
-5.9273019	9.5160007	100.6699982
-5.9273019	9.5648003	100.9399948
-5.9273019	9.6136007	101.5299988
-5.9273019	9.6624002	103.0299988
-5.9273019	9.7112007	104.3399963
-5.9273019	9.7600002	102.5400009
-5.9273019	9.8088007	104.7399979
-5.9273019	9.8576002	107.2399979
-5.9273019	9.9064007	107.5000000
-5.9273019	9.9552002	104.5599976
-5.9273019	10.0040007	104.1899948
-5.9273019	10.0528002	100.2200012
-5.9273019	10.1016006	102.5299988
-5.9273019	10.1504002	102.2500000
-5.9273019	10.1992006	101.4099960
-5.9273019	10.2480001	98.9899979
-5.9273019	10.2968006	98.0400009
-5.9273019	10.3456001	99.5699997
-5.9273019	10.3944006	104.2399979
-5.9273019	10.4432001	104.7299957
-5.9273019	10.4920006	101.5599976
-5.9273019	10.5408001	98.7999954
-5.9273019	10.5896006	102.4199982
-5.9273019	10.6384001	104.5499954
-5.9273019	10.6872005	102.6599960
-5.9273019	10.7360001	102.4599991
-5.9273019	10.7848005	104.0899963
-5.9273019	10.8336000	103.7900009
-5.9273019	10.8824005	105.6899948
-5.9273019	10.9312000	106.8899994
-5.9273019	10.9800005	104.0800018
-5.9273019	11.0288000	104.1100006
-5.9273019	11.0776005	105.7399979
-5.9273019	11.1264000	103.2799988
-5.9273019	11.1752005	103.5999985
-5.9273019	11.2240000	100.6800003
-5.9273019	11.2728004	103.9199982
-5.9273019	11.3216000	105.3099976
-5.9273019	11.3704004	104.9899979
-5.9273019	11.4191999	103.8399963
-5.9273019	11.4680004	101.9300003
-5.9273019	11.5167999	101.8099976
-5.9273019	11.5656004	101.1899948
-5.9273019	11.6143999	101.5899963

-5.9273019	11.6632004	101.5400009
-5.9273019	11.7119999	103.1500015
-5.9273019	11.7608004	102.3699951
-5.9273019	11.8095999	103.0100021
-5.9273019	11.8584003	100.6199951
-5.9273019	11.9071999	100.3300018
-5.9273019	11.9560003	102.9199982
-5.9273019	12.0047998	103.3399963
-5.9273019	12.0536003	103.7299957
-5.9273019	12.1023998	103.3600006
-5.9273019	12.1512003	104.7600021
-5.9273019	12.1999998	103.8399963
-5.9273019	12.2488003	104.3600006
-5.9273019	12.2975998	103.2099991
-5.9273019	12.3464003	103.5000000
-5.9273019	12.3951998	103.1199951
-5.9273019	12.4440002	103.7799988
-5.9273019	12.4927998	105.3899994
-5.9273019	12.5416002	104.0499954
-5.9273019	12.5904007	101.2399979
-5.9273019	12.6392002	100.6599960
-5.9273019	12.6880007	103.7799988
-5.9273019	12.7368002	103.4300003
-5.9273019	12.7856007	104.4000015
-5.9273019	12.8344002	103.8899994
-5.9273019	12.8832006	103.1299973
-5.9273019	12.9320002	103.3399963
-5.9273019	12.9808006	103.4899979
-5.9273019	13.0296001	102.8199997
-5.9273019	13.0784006	102.9599991
-5.9273019	13.1272001	104.0999985
-5.9273019	13.1760006	104.9399948
-5.9273019	13.2248001	104.3099976
-5.9273019	13.2736006	105.8499985
-5.9273019	13.3224001	103.8399963
-5.9273019	13.3712006	102.6800003
-5.9273019	13.4200001	101.9899979
-5.9273019	13.4688005	101.0699997
-5.9273019	13.5176001	97.0599976
-5.9273019	13.5664005	99.7699966
-5.9273019	13.6152000	102.6299973
-5.9273019	13.6640005	104.3699951
-5.9273019	13.7128000	107.5499954
-5.9273019	13.7616005	107.6199951
-5.9273019	13.8104000	102.6399994
-5.9273019	13.8592005	101.7200012
-5.9273019	13.9080000	99.0000000
-5.9273019	13.9568005	99.5000000
-5.9273019	14.0056000	100.3399963
-5.9273019	14.0544004	102.0999985
-5.9273019	14.1032000	101.1999969
-5.9273019	14.1520004	102.2099991
-5.9273019	14.2007999	101.3600006
-5.9273019	14.2496004	103.1599960
-5.9273019	14.2983999	106.6500015
-5.9273019	14.3472004	106.8600006
-5.9273019	14.3959999	105.7399979
-5.9273019	14.4448004	105.2900009

-5.9273019	14.4935999	103.5800018
-5.9273019	14.5424004	104.0100021
-5.9273019	14.5911999	104.2600021
-5.9273019	14.6400003	105.4199982
-5.9273019	14.6887999	104.5100021
-5.9273019	14.7376003	101.9399948
-5.9273019	14.7863998	103.9399948
-5.9273019	14.8352003	104.3899994
-5.9273019	14.8839998	104.6699982
-5.9273019	14.9328003	104.1599960
-5.9273019	14.9815998	107.0000000
-5.9273019	15.0304003	105.8600006
-5.9211659	0.3416000	109.8099976
-5.9211659	0.3904000	108.6699982
-5.9211659	0.4392000	106.9899979
-5.9211659	0.4880000	105.1899948
-5.9211659	0.5368000	101.3799973
-5.9211659	0.5856000	104.6299973
-5.9211659	0.6344000	107.2900009
-5.9211659	0.6832000	102.2900009
-5.9211659	0.7320000	106.0299988
-5.9211659	0.7808000	107.4199982
-5.9211659	0.8296000	109.0599976
-5.9211659	0.8784000	110.0199966
-5.9211659	0.9272000	110.3899994
-5.9211659	0.9760000	108.9000015
-5.9211659	1.0247999	108.5199966
-5.9211659	1.0735999	106.1199951
-5.9211659	1.1223999	106.1100006
-5.9211659	1.1711999	102.9000015
-5.9211659	1.2200000	104.7500000
-5.9211659	1.2688000	105.7699966
-5.9211659	1.3176000	105.9099960
-5.9211659	1.3664000	105.2699966
-5.9211659	1.4152000	106.8099976
-5.9211659	1.4640000	107.8399963
-5.9211659	1.5128000	109.2600021
-5.9211659	1.5616000	110.7399979
-5.9211659	1.6104000	109.4899979
-5.9211659	1.6592000	111.6999969
-5.9211659	1.7079999	112.7500000
-5.9211659	1.7567999	109.6299973
-5.9211659	1.8055999	104.1199951
-5.9211659	1.8543999	115.0999985
-5.9211659	1.9031999	117.8499985
-5.9211659	1.9519999	114.3199997
-5.9211659	2.0007999	108.6599960
-5.9211659	2.0495999	107.4499969
-5.9211659	2.0983999	107.7200012
-5.9211659	2.1471999	104.5100021
-5.9211659	2.1959999	106.4099960
-5.9211659	2.2447999	106.1599960
-5.9211659	2.2935998	103.4799957
-5.9211659	2.3423998	101.6199951
-5.9211659	2.3912001	103.7799988
-5.9211659	2.4400001	105.8600006
-5.9211659	2.4888000	107.4700012
-5.9211659	2.5376000	106.1299973

-5.9211659	2.5864000	105.5800018
-5.9211659	2.6352000	106.7299957
-5.9211659	2.6840000	106.7399979
-5.9211659	2.7328000	106.0100021
-5.9211659	2.7816000	104.9700012
-5.9211659	2.8304000	106.3600006
-5.9211659	2.8792000	108.1100006
-5.9211659	2.9280000	107.7600021
-5.9211659	2.9768000	107.2999954
-5.9211659	3.0256000	106.8499985
-5.9211659	3.0743999	103.3499985
-5.9211659	3.1231999	102.0599976
-5.9211659	3.1719999	100.4799957
-5.9211659	3.2207999	102.7799988
-5.9211659	3.2695999	104.9199982
-5.9211659	3.3183999	100.6299973
-5.9211659	3.3671999	102.7900009
-5.9211659	3.4159999	105.0299988
-5.9211659	3.4647999	104.8699951
-5.9211659	3.5135999	106.0599976
-5.9211659	3.5623999	104.9399948
-5.9211659	3.6111999	105.3300018
-5.9211659	3.6599998	105.7799988
-5.9211659	3.7087998	103.9000015
-5.9211659	3.7575998	104.4599991
-5.9211659	3.8063998	103.5100021
-5.9211659	3.8551998	105.1100006
-5.9211659	3.9039998	105.5699997
-5.9211659	3.9527998	103.1999969
-5.9211659	4.0016003	102.8300018
-5.9211659	4.0503998	99.9599991
-5.9211659	4.0991998	103.9899979
-5.9211659	4.1479998	105.6699982
-5.9211659	4.1967998	105.3499985
-5.9211659	4.2455997	103.4099960
-5.9211659	4.2943997	108.1699982
-5.9211659	4.3431997	110.8799973
-5.9211659	4.3919997	110.4099960
-5.9211659	4.4407997	106.4000015
-5.9211659	4.4895997	109.1199951
-5.9211659	4.5383997	112.7699966
-5.9211659	4.5871997	112.9499969
-5.9211659	4.6359997	112.2399979
-5.9211659	4.6847997	116.2999954
-5.9211659	4.7336001	114.9799957
-5.9211659	4.7824001	108.0699997
-5.9211659	4.8312001	107.1999969
-5.9211659	4.8800001	106.4300003
-5.9211659	4.9288001	106.4300003
-5.9211659	4.9776001	105.4399948
-5.9211659	5.0264001	107.6100006
-5.9211659	5.0752001	107.0299988
-5.9211659	5.1240001	104.6299973
-5.9211659	5.1728001	100.4799957
-5.9211659	5.2216001	102.1100006
-5.9211659	5.2704000	105.3300018
-5.9211659	5.3192000	104.8699951
-5.9211659	5.3680000	105.7200012

-5.9211659	5.4168000	104.9199982
-5.9211659	5.4656000	100.7900009
-5.9211659	5.5144000	103.8699951
-5.9211659	5.5632000	105.5800018
-5.9211659	5.6120000	105.4499969
-5.9211659	5.6608000	107.5499954
-5.9211659	5.7096000	108.5899963
-5.9211659	5.7584000	104.8399963
-5.9211659	5.8072000	103.1899948
-5.9211659	5.8559999	105.1299973
-5.9211659	5.9047999	106.7399979
-5.9211659	5.9535999	106.5699997
-5.9211659	6.0023999	106.2299957
-5.9211659	6.0511999	106.4000015
-5.9211659	6.0999999	104.9599991
-5.9211659	6.1487999	105.0699997
-5.9211659	6.1975999	102.9499969
-5.9211659	6.2463999	104.6699982
-5.9211659	6.2951999	105.7900009
-5.9211659	6.3439999	103.7600021
-5.9211659	6.3927999	104.9199982
-5.9211659	6.4415998	103.4000015
-5.9211659	6.4903998	99.6399994
-5.9211659	6.5391998	101.7500000
-5.9211659	6.5879998	101.1299973
-5.9211659	6.6367998	103.6899948
-5.9211659	6.6855998	103.8699951
-5.9211659	6.7343998	104.2900009
-5.9211659	6.7831998	101.2099991
-5.9211659	6.8319998	103.6899948
-5.9211659	6.8807998	105.1100006
-5.9211659	6.9295998	107.7600021
-5.9211659	6.9783998	107.9000015
-5.9211659	7.0271997	104.5000000
-5.9211659	7.0759997	102.9300003
-5.9211659	7.1247997	101.5899963
-5.9211659	7.1735997	102.1399994
-5.9211659	7.2223997	105.0499954
-5.9211659	7.2711997	104.7900009
-5.9211659	7.3199997	103.5400009
-5.9211659	7.3687997	103.7999954
-5.9211659	7.4175997	102.5000000
-5.9211659	7.4663997	104.4000015
-5.9211659	7.5151997	99.1599960
-5.9211659	7.5639997	100.5499954
-5.9211659	7.6127996	103.8499985
-5.9211659	7.6615996	104.9599991
-5.9211659	7.7103996	105.1899948
-5.9211659	7.7591996	105.0299988
-5.9211659	7.8079996	105.5499954
-5.9211659	7.8567996	104.6899948
-5.9211659	7.9056001	102.9799957
-5.9211659	7.9544001	101.1399994
-5.9211659	8.0032005	98.6699982
-5.9211659	8.0520000	104.4499969
-5.9211659	8.1008005	105.3499985
-5.9211659	8.1496000	106.0800018
-5.9211659	8.1984005	105.6599960

-5.9211659	8.2472000	105.3699951
-5.9211659	8.2960005	105.7999954
-5.9211659	8.3448000	105.9300003
-5.9211659	8.3936005	102.7099991
-5.9211659	8.4424000	101.9799957
-5.9211659	8.4912004	102.1599960
-5.9211659	8.5400000	105.7200012
-5.9211659	8.5888004	104.2200012
-5.9211659	8.6375999	102.0800018
-5.9211659	8.6864004	103.1899948
-5.9211659	8.7351999	103.5400009
-5.9211659	8.7840004	102.3099976
-5.9211659	8.8327999	100.0000000
-5.9211659	8.8816004	103.0699997
-5.9211659	8.9303999	104.4899979
-5.9211659	8.9792004	102.1899948
-5.9211659	9.0279999	104.0800018
-5.9211659	9.0768003	105.6100006
-5.9211659	9.1255999	101.1299973
-5.9211659	9.1744003	100.6899948
-5.9211659	9.2231998	104.1699982
-5.9211659	9.2720003	103.6800003
-5.9211659	9.3207998	103.4899979
-5.9211659	9.3696003	99.9199982
-5.9211659	9.4184008	102.8300018
-5.9211659	9.4672003	103.6599960
-5.9211659	9.5160007	101.0100021
-5.9211659	9.5648003	101.1899948
-5.9211659	9.6136007	101.3099976
-5.9211659	9.6624002	102.5899963
-5.9211659	9.7112007	104.3300018
-5.9211659	9.7600002	102.6699982
-5.9211659	9.8088007	105.0699997
-5.9211659	9.8576002	107.7200012
-5.9211659	9.9064007	107.0299988
-5.9211659	9.9552002	103.5000000
-5.9211659	10.0040007	104.7099991
-5.9211659	10.0528002	101.2699966
-5.9211659	10.1016006	102.5199966
-5.9211659	10.1504002	101.3799973
-5.9211659	10.1992006	100.7699966
-5.9211659	10.2480001	99.4399948
-5.9211659	10.2968006	99.1100006
-5.9211659	10.3456001	100.3899994
-5.9211659	10.3944006	104.6699982
-5.9211659	10.4432001	104.9000015
-5.9211659	10.4920006	101.8099976
-5.9211659	10.5408001	99.0400009
-5.9211659	10.5896006	103.2399979
-5.9211659	10.6384001	104.3099976
-5.9211659	10.6872005	101.0599976
-5.9211659	10.7360001	102.4399948
-5.9211659	10.7848005	104.4000015
-5.9211659	10.8336000	104.3799973
-5.9211659	10.8824005	105.7200012
-5.9211659	10.9312000	107.4399948
-5.9211659	10.9800005	103.4799957
-5.9211659	11.0288000	104.0999985

-5.9211659	11.0776005	106.6899948
-5.9211659	11.1264000	104.5499954
-5.9211659	11.1752005	103.4300003
-5.9211659	11.2240000	99.5100021
-5.9211659	11.2728004	104.4399948
-5.9211659	11.3216000	105.9599991
-5.9211659	11.3704004	105.3899994
-5.9211659	11.4191999	103.2799988
-5.9211659	11.4680004	101.6800003
-5.9211659	11.5167999	101.6199951
-5.9211659	11.5656004	100.9700012
-5.9211659	11.6143999	102.2699966
-5.9211659	11.6632004	102.0699997
-5.9211659	11.7119999	103.3499985
-5.9211659	11.7608004	102.0599976
-5.9211659	11.8095999	103.1699982
-5.9211659	11.8584003	100.6199951
-5.9211659	11.9071999	101.0199966
-5.9211659	11.9560003	103.4700012
-5.9211659	12.0047998	104.2200012
-5.9211659	12.0536003	105.0100021
-5.9211659	12.1023998	104.0699997
-5.9211659	12.1512003	104.9799957
-5.9211659	12.1999998	104.3899994
-5.9211659	12.2488003	105.5199966
-5.9211659	12.2975998	104.0000000
-5.9211659	12.3464003	103.9300003
-5.9211659	12.3951998	103.0299988
-5.9211659	12.4440002	104.0100021
-5.9211659	12.4927998	105.1899948
-5.9211659	12.5416002	104.1399994
-5.9211659	12.5904007	102.0899963
-5.9211659	12.6392002	101.2299957
-5.9211659	12.6880007	103.6699982
-5.9211659	12.7368002	102.2999954
-5.9211659	12.7856007	103.5899963
-5.9211659	12.8344002	103.3499985
-5.9211659	12.8832006	102.5599976
-5.9211659	12.9320002	103.8300018
-5.9211659	12.9808006	103.7299957
-5.9211659	13.0296001	103.6999969
-5.9211659	13.0784006	103.6199951
-5.9211659	13.1272001	104.3199997
-5.9211659	13.1760006	104.2399979
-5.9211659	13.2248001	103.0000000
-5.9211659	13.2736006	105.4499969
-5.9211659	13.3224001	102.9199982
-5.9211659	13.3712006	102.8399963
-5.9211659	13.4200001	101.4899979
-5.9211659	13.4688005	101.3399963
-5.9211659	13.5176001	98.1800003
-5.9211659	13.5664005	99.8199997
-5.9211659	13.6152000	102.6399994
-5.9211659	13.6640005	103.5899963
-5.9211659	13.7128000	106.6800003
-5.9211659	13.7616005	106.8300018
-5.9211659	13.8104000	101.3600006
-5.9211659	13.8592005	100.3600006

-5.9211659	13.9080000	100.1299973
-5.9211659	13.9568005	99.6599960
-5.9211659	14.0056000	101.0499954
-5.9211659	14.0544004	102.6399994
-5.9211659	14.1032000	100.8399963
-5.9211659	14.1520004	101.8199997
-5.9211659	14.2007999	101.6899948
-5.9211659	14.2496004	103.9300003
-5.9211659	14.2983999	107.0899963
-5.9211659	14.3472004	107.3099976
-5.9211659	14.3959999	105.5000000
-5.9211659	14.4448004	104.7500000
-5.9211659	14.4935999	103.2399979
-5.9211659	14.5424004	103.8199997
-5.9211659	14.5911999	104.1899948
-5.9211659	14.6400003	105.6399994
-5.9211659	14.6887999	105.4599991
-5.9211659	14.7376003	103.4300003
-5.9211659	14.7863998	104.8499985
-5.9211659	14.8352003	104.8099976
-5.9211659	14.8839998	103.8199997
-5.9211659	14.9328003	102.5999985
-5.9211659	14.9815998	106.0899963
-5.9211659	15.0304003	105.0699997
-5.9150300	0.3416000	108.6800003
-5.9150300	0.3904000	107.3399963
-5.9150300	0.4392000	106.2999954
-5.9150300	0.4880000	104.7999954
-5.9150300	0.5368000	101.4300003
-5.9150300	0.5856000	102.9799957
-5.9150300	0.6344000	106.7600021
-5.9150300	0.6832000	102.6899948
-5.9150300	0.7320000	107.3499985
-5.9150300	0.7808000	108.1100006
-5.9150300	0.8296000	108.7500000
-5.9150300	0.8784000	109.9399948
-5.9150300	0.9272000	110.7299957
-5.9150300	0.9760000	109.6800003
-5.9150300	1.0247999	108.3699951
-5.9150300	1.0735999	106.3799973
-5.9150300	1.1223999	106.7799988
-5.9150300	1.1711999	103.1899948
-5.9150300	1.2200000	104.6800003
-5.9150300	1.2688000	105.0000000
-5.9150300	1.3176000	105.3300018
-5.9150300	1.3664000	105.5599976
-5.9150300	1.4152000	106.9899979
-5.9150300	1.4640000	107.6899948
-5.9150300	1.5128000	109.9000015
-5.9150300	1.5616000	111.1899948
-5.9150300	1.6104000	108.8199997
-5.9150300	1.6592000	111.1800003
-5.9150300	1.7079999	112.2099991
-5.9150300	1.7567999	109.6999969
-5.9150300	1.8055999	104.1699982
-5.9150300	1.8543999	115.2200012
-5.9150300	1.9031999	117.8099976
-5.9150300	1.9519999	113.9899979

-5.9150300	2.0007999	108.7699966
-5.9150300	2.0495999	107.5800018
-5.9150300	2.0983999	107.1500015
-5.9150300	2.1471999	103.5499954
-5.9150300	2.1959999	107.0699997
-5.9150300	2.2447999	106.3300018
-5.9150300	2.2935998	104.7299957
-5.9150300	2.3423998	103.1999969
-5.9150300	2.3912001	104.7399979
-5.9150300	2.4400001	106.9000015
-5.9150300	2.4888000	108.0100021
-5.9150300	2.5376000	106.5899963
-5.9150300	2.5864000	105.6500015
-5.9150300	2.6352000	106.9700012
-5.9150300	2.6840000	106.8699951
-5.9150300	2.7328000	106.1500015
-5.9150300	2.7816000	105.3600006
-5.9150300	2.8304000	106.5699997
-5.9150300	2.8792000	108.1199951
-5.9150300	2.9280000	108.4899979
-5.9150300	2.9768000	107.4099960
-5.9150300	3.0256000	106.6899948
-5.9150300	3.0743999	104.0100021
-5.9150300	3.1231999	102.4499969
-5.9150300	3.1719999	101.2099991
-5.9150300	3.2207999	104.2799988
-5.9150300	3.2695999	105.3199997
-5.9150300	3.3183999	100.2299957
-5.9150300	3.3671999	102.6599960
-5.9150300	3.4159999	105.0699997
-5.9150300	3.4647999	104.3600006
-5.9150300	3.5135999	105.5100021
-5.9150300	3.5623999	104.8399963
-5.9150300	3.6111999	104.5599976
-5.9150300	3.6599998	105.6999969
-5.9150300	3.7087998	105.1999969
-5.9150300	3.7575998	105.5800018
-5.9150300	3.8063998	104.8399963
-5.9150300	3.8551998	105.0400009
-5.9150300	3.9039998	105.2099991
-5.9150300	3.9527998	102.9499969
-5.9150300	4.0016003	102.5400009
-5.9150300	4.0503998	100.3300018
-5.9150300	4.0991998	105.3199997
-5.9150300	4.1479998	106.5699997
-5.9150300	4.1967998	105.8399963
-5.9150300	4.2455997	103.6399994
-5.9150300	4.2943997	108.0299988
-5.9150300	4.3431997	111.2399979
-5.9150300	4.3919997	110.8899994
-5.9150300	4.4407997	107.0499954
-5.9150300	4.4895997	109.2799988
-5.9150300	4.5383997	113.3300018
-5.9150300	4.5871997	113.5199966
-5.9150300	4.6359997	113.3199997
-5.9150300	4.6847997	116.9899979
-5.9150300	4.7336001	115.4799957
-5.9150300	4.7824001	108.7500000

-5.9150300	4.8312001	106.1399994
-5.9150300	4.8800001	105.9599991
-5.9150300	4.9288001	106.3199997
-5.9150300	4.9776001	105.0400009
-5.9150300	5.0264001	107.0800018
-5.9150300	5.0752001	106.3799973
-5.9150300	5.1240001	104.3300018
-5.9150300	5.1728001	100.3399963
-5.9150300	5.2216001	100.8899994
-5.9150300	5.2704000	105.8600006
-5.9150300	5.3192000	105.3899994
-5.9150300	5.3680000	105.2099991
-5.9150300	5.4168000	104.1800003
-5.9150300	5.4656000	101.5800018
-5.9150300	5.5144000	103.8899994
-5.9150300	5.5632000	105.5599976
-5.9150300	5.6120000	105.5899963
-5.9150300	5.6608000	107.5400009
-5.9150300	5.7096000	108.6800003
-5.9150300	5.7584000	105.4599991
-5.9150300	5.8072000	103.4899979
-5.9150300	5.8559999	104.8499985
-5.9150300	5.9047999	106.9199982
-5.9150300	5.9535999	105.6299973
-5.9150300	6.0023999	105.2500000
-5.9150300	6.0511999	106.0000000
-5.9150300	6.0999999	104.8600006
-5.9150300	6.1487999	105.2299957
-5.9150300	6.1975999	102.8099976
-5.9150300	6.2463999	105.1899948
-5.9150300	6.2951999	106.5599976
-5.9150300	6.3439999	104.5800018
-5.9150300	6.3927999	105.4399948
-5.9150300	6.4415998	103.6699982
-5.9150300	6.4903998	99.9700012
-5.9150300	6.5391998	102.7799988
-5.9150300	6.5879998	101.1100006
-5.9150300	6.6367998	103.8699951
-5.9150300	6.6855998	104.2200012
-5.9150300	6.7343998	104.4099960
-5.9150300	6.7831998	101.9199982
-5.9150300	6.8319998	103.6699982
-5.9150300	6.8807998	105.2399979
-5.9150300	6.9295998	107.5199966
-5.9150300	6.9783998	106.5299988
-5.9150300	7.0271997	102.4399948
-5.9150300	7.0759997	101.9899979
-5.9150300	7.1247997	101.6800003
-5.9150300	7.1735997	101.7099991
-5.9150300	7.2223997	103.7699966
-5.9150300	7.2711997	103.8899994
-5.9150300	7.3199997	104.0999985
-5.9150300	7.3687997	105.0299988
-5.9150300	7.4175997	104.1999969
-5.9150300	7.4663997	104.7200012
-5.9150300	7.5151997	98.2399979
-5.9150300	7.5639997	99.8899994
-5.9150300	7.6127996	102.4000015

-5.9150300	7.6615996	104.7600021
-5.9150300	7.7103996	105.6299973
-5.9150300	7.7591996	105.2600021
-5.9150300	7.8079996	105.6100006
-5.9150300	7.8567996	105.3399963
-5.9150300	7.9056001	103.7200012
-5.9150300	7.9544001	101.5199966
-5.9150300	8.0032005	99.5499954
-5.9150300	8.0520000	104.2200012
-5.9150300	8.1008005	104.2500000
-5.9150300	8.1496000	105.0699997
-5.9150300	8.1984005	104.6699982
-5.9150300	8.2472000	105.0100021
-5.9150300	8.2960005	105.7699966
-5.9150300	8.3448000	105.8600006
-5.9150300	8.3936005	101.9799957
-5.9150300	8.4424000	102.7799988
-5.9150300	8.4912004	102.2099991
-5.9150300	8.5400000	105.4199982
-5.9150300	8.5888004	104.2500000
-5.9150300	8.6375999	102.8799973
-5.9150300	8.6864004	103.7500000
-5.9150300	8.7351999	104.2900009
-5.9150300	8.7840004	102.3600006
-5.9150300	8.8327999	98.6399994
-5.9150300	8.8816004	102.4300003
-5.9150300	8.9303999	104.5000000
-5.9150300	8.9792004	102.4399948
-5.9150300	9.0279999	104.2600021
-5.9150300	9.0768003	105.5800018
-5.9150300	9.1255999	101.7299957
-5.9150300	9.1744003	101.1999969
-5.9150300	9.2231998	103.9000015
-5.9150300	9.2720003	103.5100021
-5.9150300	9.3207998	103.2099991
-5.9150300	9.3696003	98.8799973
-5.9150300	9.4184008	102.0499954
-5.9150300	9.4672003	103.3099976
-5.9150300	9.5160007	101.2099991
-5.9150300	9.5648003	101.7999954
-5.9150300	9.6136007	101.3600006
-5.9150300	9.6624002	102.4399948
-5.9150300	9.7112007	104.6999969
-5.9150300	9.7600002	103.0599976
-5.9150300	9.8088007	105.3699951
-5.9150300	9.8576002	107.7399979
-5.9150300	9.9064007	106.9799957
-5.9150300	9.9552002	103.8699951
-5.9150300	10.0040007	105.6100006
-5.9150300	10.0528002	102.6399994
-5.9150300	10.1016006	102.8699951
-5.9150300	10.1504002	100.7900009
-5.9150300	10.1992006	100.4000015
-5.9150300	10.2480001	99.9899979
-5.9150300	10.2968006	100.5199966
-5.9150300	10.3456001	101.6500015
-5.9150300	10.3944006	105.2299957
-5.9150300	10.4432001	104.9700012

-5.9150300	10.4920006	102.1800003
-5.9150300	10.5408001	99.6899948
-5.9150300	10.5896006	103.7500000
-5.9150300	10.6384001	103.4399948
-5.9150300	10.6872005	99.7200012
-5.9150300	10.7360001	102.2999954
-5.9150300	10.7848005	104.2200012
-5.9150300	10.8336000	104.1800003
-5.9150300	10.8824005	104.8199997
-5.9150300	10.9312000	106.2399979
-5.9150300	10.9800005	101.8399963
-5.9150300	11.0288000	103.7299957
-5.9150300	11.0776005	106.7299957
-5.9150300	11.1264000	105.3099976
-5.9150300	11.1752005	102.9499969
-5.9150300	11.2240000	98.0199966
-5.9150300	11.2728004	104.5599976
-5.9150300	11.3216000	106.4399948
-5.9150300	11.3704004	105.8099976
-5.9150300	11.4191999	102.6299973
-5.9150300	11.4680004	101.4899979
-5.9150300	11.5167999	101.7099991
-5.9150300	11.5656004	100.7699966
-5.9150300	11.6143999	103.0100021
-5.9150300	11.6632004	102.9700012
-5.9150300	11.7119999	103.6599960
-5.9150300	11.7608004	102.0499954
-5.9150300	11.8095999	103.5400009
-5.9150300	11.8584003	101.6199951
-5.9150300	11.9071999	102.4499969
-5.9150300	11.9560003	104.2999954
-5.9150300	12.0047998	105.0899963
-5.9150300	12.0536003	105.3600006
-5.9150300	12.1023998	104.0699997
-5.9150300	12.1512003	105.1599960
-5.9150300	12.1999998	104.8499985
-5.9150300	12.2488003	106.3199997
-5.9150300	12.2975998	104.5800018
-5.9150300	12.3464003	103.7699966
-5.9150300	12.3951998	102.6599960
-5.9150300	12.4440002	103.7699966
-5.9150300	12.4927998	104.8199997
-5.9150300	12.5416002	103.8499985
-5.9150300	12.5904007	102.6999969
-5.9150300	12.6392002	101.1999969
-5.9150300	12.6880007	103.6699982
-5.9150300	12.7368002	102.4899979
-5.9150300	12.7856007	103.3300018
-5.9150300	12.8344002	103.5000000
-5.9150300	12.8832006	103.1100006
-5.9150300	12.9320002	104.6699982
-5.9150300	12.9808006	104.2999954
-5.9150300	13.0296001	103.8699951
-5.9150300	13.0784006	103.7399979
-5.9150300	13.1272001	103.9799957
-5.9150300	13.1760006	103.0499954
-5.9150300	13.2248001	101.4199982
-5.9150300	13.2736006	104.4300003

-5.9150300	13.3224001	101.8399963
-5.9150300	13.3712006	103.0400009
-5.9150300	13.4200001	101.4899979
-5.9150300	13.4688005	101.5899963
-5.9150300	13.5176001	99.0100021
-5.9150300	13.5664005	99.1599960
-5.9150300	13.6152000	102.1599960
-5.9150300	13.6640005	102.3699951
-5.9150300	13.7128000	105.1599960
-5.9150300	13.7616005	105.9000015
-5.9150300	13.8104000	100.5100021
-5.9150300	13.8592005	99.9000015
-5.9150300	13.9080000	101.0000000
-5.9150300	13.9568005	99.8899994
-5.9150300	14.0056000	102.0499954
-5.9150300	14.0544004	102.6899948
-5.9150300	14.1032000	99.3699951
-5.9150300	14.1520004	100.4199982
-5.9150300	14.2007999	101.5899963
-5.9150300	14.2496004	104.1299973
-5.9150300	14.2983999	106.5199966
-5.9150300	14.3472004	107.6299973
-5.9150300	14.3959999	105.7600021
-5.9150300	14.4448004	103.9099960
-5.9150300	14.4935999	102.7299957
-5.9150300	14.5424004	103.5400009
-5.9150300	14.5911999	103.9799957
-5.9150300	14.6400003	105.2399979
-5.9150300	14.6887999	105.0299988
-5.9150300	14.7376003	104.0800018
-5.9150300	14.7863998	105.3300018
-5.9150300	14.8352003	105.0800018
-5.9150300	14.8839998	103.0400009
-5.9150300	14.9328003	101.0199966
-5.9150300	14.9815998	104.7399979
-5.9150300	15.0304003	103.5800018
-5.9088941	0.3416000	107.8399963
-5.9088941	0.3904000	106.4499969
-5.9088941	0.4392000	106.2699966
-5.9088941	0.4880000	105.1299973
-5.9088941	0.5368000	102.0299988
-5.9088941	0.5856000	101.4700012
-5.9088941	0.6344000	106.2699966
-5.9088941	0.6832000	102.4799957
-5.9088941	0.7320000	108.4799957
-5.9088941	0.7808000	108.8399963
-5.9088941	0.8296000	108.4700012
-5.9088941	0.8784000	109.3799973
-5.9088941	0.9272000	110.1800003
-5.9088941	0.9760000	109.4599991
-5.9088941	1.0247999	108.1100006
-5.9088941	1.0735999	106.0499954
-5.9088941	1.1223999	107.2900009
-5.9088941	1.1711999	104.5100021
-5.9088941	1.2200000	104.8099976
-5.9088941	1.2688000	104.5699997
-5.9088941	1.3176000	105.0999985
-5.9088941	1.3664000	106.0400009

-5.9088941	1.4152000	107.3699951
-5.9088941	1.4640000	107.5699997
-5.9088941	1.5128000	110.0800018
-5.9088941	1.5616000	111.2799988
-5.9088941	1.6104000	108.1199951
-5.9088941	1.6592000	110.5199966
-5.9088941	1.7079999	111.6100006
-5.9088941	1.7567999	109.5299988
-5.9088941	1.8055999	104.9499969
-5.9088941	1.8543999	115.4700012
-5.9088941	1.9031999	117.6199951
-5.9088941	1.9519999	113.4599991
-5.9088941	2.0007999	108.4899979
-5.9088941	2.0495999	107.1199951
-5.9088941	2.0983999	106.4300003
-5.9088941	2.1471999	103.2299957
-5.9088941	2.1959999	107.7799988
-5.9088941	2.2447999	106.2099991
-5.9088941	2.2935998	105.0699997
-5.9088941	2.3423998	104.0999985
-5.9088941	2.3912001	105.1299973
-5.9088941	2.4400001	107.2399979
-5.9088941	2.4888000	107.5699997
-5.9088941	2.5376000	106.3199997
-5.9088941	2.5864000	104.9399948
-5.9088941	2.6352000	106.6599960
-5.9088941	2.6840000	106.7099991
-5.9088941	2.7328000	106.2900009
-5.9088941	2.7816000	105.5100021
-5.9088941	2.8304000	106.6299973
-5.9088941	2.8792000	108.0000000
-5.9088941	2.9280000	109.1399994
-5.9088941	2.9768000	107.7600021
-5.9088941	3.0256000	106.8799973
-5.9088941	3.0743999	104.9399948
-5.9088941	3.1231999	103.5199966
-5.9088941	3.1719999	102.4499969
-5.9088941	3.2207999	105.7699966
-5.9088941	3.2695999	106.0100021
-5.9088941	3.3183999	101.3499985
-5.9088941	3.3671999	103.2299957
-5.9088941	3.4159999	104.5699997
-5.9088941	3.4647999	103.1199951
-5.9088941	3.5135999	104.3699951
-5.9088941	3.5623999	104.0000000
-5.9088941	3.6111999	103.4700012
-5.9088941	3.6599998	105.1800003
-5.9088941	3.7087998	106.0100021
-5.9088941	3.7575998	106.0100021
-5.9088941	3.8063998	104.9799957
-5.9088941	3.8551998	104.2799988
-5.9088941	3.9039998	104.6899948
-5.9088941	3.9527998	102.9499969
-5.9088941	4.0016003	103.0000000
-5.9088941	4.0503998	102.0699997
-5.9088941	4.0991998	106.6500015
-5.9088941	4.1479998	106.7900009
-5.9088941	4.1967998	105.6100006

-5.9088941	4.2455997	103.3099976
-5.9088941	4.2943997	107.4799957
-5.9088941	4.3431997	111.2399979
-5.9088941	4.3919997	110.8499985
-5.9088941	4.4407997	107.7299957
-5.9088941	4.4895997	109.1899948
-5.9088941	4.5383997	113.1199951
-5.9088941	4.5871997	113.4099960
-5.9088941	4.6359997	114.0100021
-5.9088941	4.6847997	117.0400009
-5.9088941	4.7336001	115.4399948
-5.9088941	4.7824001	108.8899994
-5.9088941	4.8312001	105.9099960
-5.9088941	4.8800001	106.6199951
-5.9088941	4.9288001	106.8399963
-5.9088941	4.9776001	105.4199982
-5.9088941	5.0264001	107.0100021
-5.9088941	5.0752001	105.8399963
-5.9088941	5.1240001	104.0299988
-5.9088941	5.1728001	101.0599976
-5.9088941	5.2216001	101.1699982
-5.9088941	5.2704000	106.3799973
-5.9088941	5.3192000	105.9000015
-5.9088941	5.3680000	105.1199951
-5.9088941	5.4168000	103.4300003
-5.9088941	5.4656000	102.2600021
-5.9088941	5.5144000	103.7200012
-5.9088941	5.5632000	105.4499969
-5.9088941	5.6120000	105.5899963
-5.9088941	5.6608000	107.2399979
-5.9088941	5.7096000	108.5499954
-5.9088941	5.7584000	106.2900009
-5.9088941	5.8072000	103.6399994
-5.9088941	5.8559999	104.1199951
-5.9088941	5.9047999	106.6299973
-5.9088941	5.9535999	104.7699966
-5.9088941	6.0023999	103.9799957
-5.9088941	6.0511999	105.1800003
-5.9088941	6.0999999	104.1299973
-5.9088941	6.1487999	105.1800003
-5.9088941	6.1975999	102.9300003
-5.9088941	6.2463999	106.0199966
-5.9088941	6.2951999	107.4099960
-5.9088941	6.3439999	105.4199982
-5.9088941	6.3927999	105.6100006
-5.9088941	6.4415998	103.7799988
-5.9088941	6.4903998	101.4000015
-5.9088941	6.5391998	103.8300018
-5.9088941	6.5879998	101.6399994
-5.9088941	6.6367998	104.3099976
-5.9088941	6.6855998	105.0899963
-5.9088941	6.7343998	105.1699982
-5.9088941	6.7831998	102.9700012
-5.9088941	6.8319998	103.6199951
-5.9088941	6.8807998	104.6100006
-5.9088941	6.9295998	106.7200012
-5.9088941	6.9783998	105.0199966
-5.9088941	7.0271997	100.6500015

-5.9088941	7.0759997	102.2500000
-5.9088941	7.1247997	102.1199951
-5.9088941	7.1735997	101.3099976
-5.9088941	7.2223997	102.4700012
-5.9088941	7.2711997	102.8799973
-5.9088941	7.3199997	104.1599960
-5.9088941	7.3687997	105.9899979
-5.9088941	7.4175997	105.3499985
-5.9088941	7.4663997	105.5499954
-5.9088941	7.5151997	98.2999954
-5.9088941	7.5639997	98.9599991
-5.9088941	7.6127996	101.7500000
-5.9088941	7.6615996	104.7200012
-5.9088941	7.7103996	105.7099991
-5.9088941	7.7591996	104.8099976
-5.9088941	7.8079996	105.4099960
-5.9088941	7.8567996	105.2900009
-5.9088941	7.9056001	104.5000000
-5.9088941	7.9544001	102.0599976
-5.9088941	8.0032005	100.7299957
-5.9088941	8.0520000	103.7500000
-5.9088941	8.1008005	102.7999954
-5.9088941	8.1496000	103.7999954
-5.9088941	8.1984005	103.7600021
-5.9088941	8.2472000	104.9799957
-5.9088941	8.2960005	106.2099991
-5.9088941	8.3448000	106.2200012
-5.9088941	8.3936005	102.0599976
-5.9088941	8.4424000	102.5699997
-5.9088941	8.4912004	101.7900009
-5.9088941	8.5400000	104.9399948
-5.9088941	8.5888004	103.2999954
-5.9088941	8.6375999	103.4000015
-5.9088941	8.6864004	104.0800018
-5.9088941	8.7351999	104.9000015
-5.9088941	8.7840004	103.0699997
-5.9088941	8.8327999	99.6500015
-5.9088941	8.8816004	102.5999985
-5.9088941	8.9303999	104.7299957
-5.9088941	8.9792004	102.9599991
-5.9088941	9.0279999	103.4700012
-5.9088941	9.0768003	105.6599960
-5.9088941	9.1255999	102.8099976
-5.9088941	9.1744003	101.1399994
-5.9088941	9.2231998	103.2099991
-5.9088941	9.2720003	103.2099991
-5.9088941	9.3207998	104.0100021
-5.9088941	9.3696003	98.6100006
-5.9088941	9.4184008	101.0499954
-5.9088941	9.4672003	103.2099991
-5.9088941	9.5160007	102.3300018
-5.9088941	9.5648003	102.3799973
-5.9088941	9.6136007	100.7299957
-5.9088941	9.6624002	102.8399963
-5.9088941	9.7112007	105.3699951
-5.9088941	9.7600002	103.5299988
-5.9088941	9.8088007	105.4599991
-5.9088941	9.8576002	106.3699951

-5.9088941	9.9064007	105.9899979
-5.9088941	9.9552002	103.3199997
-5.9088941	10.0040007	105.6199951
-5.9088941	10.0528002	104.0599976
-5.9088941	10.1016006	103.4599991
-5.9088941	10.1504002	100.4799957
-5.9088941	10.1992006	100.5100021
-5.9088941	10.2480001	100.7600021
-5.9088941	10.2968006	101.7600021
-5.9088941	10.3456001	102.7900009
-5.9088941	10.3944006	105.6699982
-5.9088941	10.4432001	105.3499985
-5.9088941	10.4920006	103.4300003
-5.9088941	10.5408001	101.0499954
-5.9088941	10.5896006	104.1899948
-5.9088941	10.6384001	102.9899979
-5.9088941	10.6872005	100.3499985
-5.9088941	10.7360001	103.4700012
-5.9088941	10.7848005	104.4199982
-5.9088941	10.8336000	103.9099960
-5.9088941	10.8824005	103.6399994
-5.9088941	10.9312000	105.3600006
-5.9088941	10.9800005	101.7600021
-5.9088941	11.0288000	103.3799973
-5.9088941	11.0776005	106.2999954
-5.9088941	11.1264000	105.1699982
-5.9088941	11.1752005	101.9399948
-5.9088941	11.2240000	97.2600021
-5.9088941	11.2728004	104.4099960
-5.9088941	11.3216000	106.5199966
-5.9088941	11.3704004	106.0999985
-5.9088941	11.4191999	102.7500000
-5.9088941	11.4680004	101.0100021
-5.9088941	11.5167999	101.7799988
-5.9088941	11.5656004	100.4899979
-5.9088941	11.6143999	103.1999969
-5.9088941	11.6632004	104.2200012
-5.9088941	11.7119999	104.5699997
-5.9088941	11.7608004	103.1699982
-5.9088941	11.8095999	103.9499969
-5.9088941	11.8584003	101.8799973
-5.9088941	11.9071999	103.4199982
-5.9088941	11.9560003	104.7699966
-5.9088941	12.0047998	105.7900009
-5.9088941	12.0536003	105.3099976
-5.9088941	12.1023998	103.7399979
-5.9088941	12.1512003	105.2699966
-5.9088941	12.1999998	104.9700012
-5.9088941	12.2488003	106.6699982
-5.9088941	12.2975998	104.9300003
-5.9088941	12.3464003	103.8199997
-5.9088941	12.3951998	102.3099976
-5.9088941	12.4440002	103.7200012
-5.9088941	12.4927998	104.4599991
-5.9088941	12.5416002	103.4599991
-5.9088941	12.5904007	103.5899963
-5.9088941	12.6392002	102.1699982
-5.9088941	12.6880007	103.8499985

-5.9088941	12.7368002	103.0299988
-5.9088941	12.7856007	103.9300003
-5.9088941	12.8344002	103.7200012
-5.9088941	12.8832006	103.2999954
-5.9088941	12.9320002	104.7600021
-5.9088941	12.9808006	104.2299957
-5.9088941	13.0296001	103.9599991
-5.9088941	13.0784006	103.7399979
-5.9088941	13.1272001	103.4499969
-5.9088941	13.1760006	102.0199966
-5.9088941	13.2248001	100.9300003
-5.9088941	13.2736006	104.0800018
-5.9088941	13.3224001	102.0800018
-5.9088941	13.3712006	103.5599976
-5.9088941	13.4200001	101.7699966
-5.9088941	13.4688005	102.5599976
-5.9088941	13.5176001	100.7900009
-5.9088941	13.5664005	98.7600021
-5.9088941	13.6152000	101.5699997
-5.9088941	13.6640005	100.8199997
-5.9088941	13.7128000	103.7200012
-5.9088941	13.7616005	104.9499969
-5.9088941	13.8104000	100.2299957
-5.9088941	13.8592005	99.2799988
-5.9088941	13.9080000	101.5299988
-5.9088941	13.9568005	99.5000000
-5.9088941	14.0056000	102.8799973
-5.9088941	14.0544004	102.6800003
-5.9088941	14.1032000	97.9199982
-5.9088941	14.1520004	99.5999985
-5.9088941	14.2007999	101.7999954
-5.9088941	14.2496004	104.3699951
-5.9088941	14.2983999	105.5000000
-5.9088941	14.3472004	107.0999985
-5.9088941	14.3959999	105.5699997
-5.9088941	14.4448004	103.1800003
-5.9088941	14.4935999	102.2099991
-5.9088941	14.5424004	103.0199966
-5.9088941	14.5911999	103.7999954
-5.9088941	14.6400003	104.7099991
-5.9088941	14.6887999	104.1500015
-5.9088941	14.7376003	104.2999954
-5.9088941	14.7863998	105.4399948
-5.9088941	14.8352003	105.0299988
-5.9088941	14.8839998	102.5199966
-5.9088941	14.9328003	101.1199951
-5.9088941	14.9815998	103.6500015
-5.9088941	15.0304003	102.6299973
-5.9027581	0.3416000	107.1199951
-5.9027581	0.3904000	105.5699997
-5.9027581	0.4392000	105.8099976
-5.9027581	0.4880000	104.7399979
-5.9027581	0.5368000	102.7699966
-5.9027581	0.5856000	102.0100021
-5.9027581	0.6344000	105.5299988
-5.9027581	0.6832000	103.4599991
-5.9027581	0.7320000	108.9499969
-5.9027581	0.7808000	108.6199951

-5.9027581	0.8296000	107.6599960
-5.9027581	0.8784000	108.3699951
-5.9027581	0.9272000	109.6699982
-5.9027581	0.9760000	109.5699997
-5.9027581	1.0247999	108.2799988
-5.9027581	1.0735999	106.1699982
-5.9027581	1.1223999	107.1699982
-5.9027581	1.1711999	104.7900009
-5.9027581	1.2200000	104.8399963
-5.9027581	1.2688000	103.8799973
-5.9027581	1.3176000	104.1399994
-5.9027581	1.3664000	106.1299973
-5.9027581	1.4152000	108.1399994
-5.9027581	1.4640000	108.1399994
-5.9027581	1.5128000	109.9799957
-5.9027581	1.5616000	110.5199966
-5.9027581	1.6104000	107.0400009
-5.9027581	1.6592000	109.8799973
-5.9027581	1.7079999	110.7799988
-5.9027581	1.7567999	108.9399948
-5.9027581	1.8055999	105.8399963
-5.9027581	1.8543999	115.6899948
-5.9027581	1.9031999	117.2900009
-5.9027581	1.9519999	112.6599960
-5.9027581	2.0007999	108.4599991
-5.9027581	2.0495999	107.0199966
-5.9027581	2.0983999	106.0400009
-5.9027581	2.1471999	103.4700012
-5.9027581	2.1959999	108.2500000
-5.9027581	2.2447999	106.7200012
-5.9027581	2.2935998	104.8199997
-5.9027581	2.3423998	104.2299957
-5.9027581	2.3912001	105.5599976
-5.9027581	2.4400001	107.7399979
-5.9027581	2.4888000	107.0499954
-5.9027581	2.5376000	105.9300003
-5.9027581	2.5864000	105.1199951
-5.9027581	2.6352000	106.6999969
-5.9027581	2.6840000	106.3799973
-5.9027581	2.7328000	106.7900009
-5.9027581	2.7816000	106.0199966
-5.9027581	2.8304000	106.2200012
-5.9027581	2.8792000	107.1500015
-5.9027581	2.9280000	108.9899979
-5.9027581	2.9768000	107.4399948
-5.9027581	3.0256000	106.3099976
-5.9027581	3.0743999	105.3699951
-5.9027581	3.1231999	104.5199966
-5.9027581	3.1719999	103.5599976
-5.9027581	3.2207999	106.3399963
-5.9027581	3.2695999	106.1800003
-5.9027581	3.3183999	101.8899994
-5.9027581	3.3671999	103.5599976
-5.9027581	3.4159999	104.1199951
-5.9027581	3.4647999	102.6999969
-5.9027581	3.5135999	103.2099991
-5.9027581	3.5623999	103.0100021
-5.9027581	3.6111999	103.2299957

-5.9027581	3.6599998	104.6199951
-5.9027581	3.7087998	106.2500000
-5.9027581	3.7575998	105.6199951
-5.9027581	3.8063998	104.3699951
-5.9027581	3.8551998	103.0599976
-5.9027581	3.9039998	103.2900009
-5.9027581	3.9527998	102.1100006
-5.9027581	4.0016003	103.0100021
-5.9027581	4.0503998	103.8899994
-5.9027581	4.0991998	107.3399963
-5.9027581	4.1479998	106.8699951
-5.9027581	4.1967998	105.2600021
-5.9027581	4.2455997	103.2999954
-5.9027581	4.2943997	107.3600006
-5.9027581	4.3431997	111.4399948
-5.9027581	4.3919997	110.7799988
-5.9027581	4.4407997	108.0299988
-5.9027581	4.4895997	108.4799957
-5.9027581	4.5383997	113.1599960
-5.9027581	4.5871997	113.3600006
-5.9027581	4.6359997	114.3099976
-5.9027581	4.6847997	116.9599991
-5.9027581	4.7336001	115.4499969
-5.9027581	4.7824001	108.7699966
-5.9027581	4.8312001	105.6599960
-5.9027581	4.8800001	107.3300018
-5.9027581	4.9288001	107.5800018
-5.9027581	4.9776001	106.0299988
-5.9027581	5.0264001	107.3699951
-5.9027581	5.0752001	105.7799988
-5.9027581	5.1240001	103.8600006
-5.9027581	5.1728001	103.0299988
-5.9027581	5.2216001	101.6800003
-5.9027581	5.2704000	106.6699982
-5.9027581	5.3192000	105.0000000
-5.9027581	5.3680000	104.6199951
-5.9027581	5.4168000	102.8699951
-5.9027581	5.4656000	102.6999969
-5.9027581	5.5144000	104.0299988
-5.9027581	5.5632000	105.6899948
-5.9027581	5.6120000	105.8099976
-5.9027581	5.6608000	107.2299957
-5.9027581	5.7096000	108.4599991
-5.9027581	5.7584000	107.0599976
-5.9027581	5.8072000	103.6800003
-5.9027581	5.8559999	103.0800018
-5.9027581	5.9047999	106.0699997
-5.9027581	5.9535999	104.6699982
-5.9027581	6.0023999	102.8099976
-5.9027581	6.0511999	104.1899948
-5.9027581	6.0999999	103.7399979
-5.9027581	6.1487999	105.5000000
-5.9027581	6.1975999	103.0100021
-5.9027581	6.2463999	106.4000015
-5.9027581	6.2951999	107.7999954
-5.9027581	6.3439999	106.0899963
-5.9027581	6.3927999	106.0499954
-5.9027581	6.4415998	104.8300018

-5.9027581	6.4903998	103.1500015
-5.9027581	6.5391998	104.6299973
-5.9027581	6.5879998	102.0800018
-5.9027581	6.6367998	104.4499969
-5.9027581	6.6855998	105.5599976
-5.9027581	6.7343998	105.6899948
-5.9027581	6.7831998	103.6399994
-5.9027581	6.8319998	103.2099991
-5.9027581	6.8807998	103.6599960
-5.9027581	6.9295998	105.6100006
-5.9027581	6.9783998	103.0000000
-5.9027581	7.0271997	99.7999954
-5.9027581	7.0759997	102.3899994
-5.9027581	7.1247997	102.4899979
-5.9027581	7.1735997	101.4799957
-5.9027581	7.2223997	102.0000000
-5.9027581	7.2711997	102.5000000
-5.9027581	7.3199997	104.0299988
-5.9027581	7.3687997	107.0299988
-5.9027581	7.4175997	106.6299973
-5.9027581	7.4663997	106.4199982
-5.9027581	7.5151997	98.2999954
-5.9027581	7.5639997	98.1399994
-5.9027581	7.6127996	100.6699982
-5.9027581	7.6615996	104.2399979
-5.9027581	7.7103996	105.6199951
-5.9027581	7.7591996	104.6899948
-5.9027581	7.8079996	105.3899994
-5.9027581	7.8567996	105.0100021
-5.9027581	7.9056001	105.0000000
-5.9027581	7.9544001	102.4099960
-5.9027581	8.0032005	101.6399994
-5.9027581	8.0520000	103.3199997
-5.9027581	8.1008005	101.6999969
-5.9027581	8.1496000	102.8899994
-5.9027581	8.1984005	103.0499954
-5.9027581	8.2472000	104.4700012
-5.9027581	8.2960005	106.0599976
-5.9027581	8.3448000	106.4700012
-5.9027581	8.3936005	102.7399979
-5.9027581	8.4424000	102.0599976
-5.9027581	8.4912004	102.8300018
-5.9027581	8.5400000	104.8300018
-5.9027581	8.5888004	101.9599991
-5.9027581	8.6375999	103.5299988
-5.9027581	8.6864004	103.9899979
-5.9027581	8.7351999	105.2699966
-5.9027581	8.7840004	103.7999954
-5.9027581	8.8327999	101.0999985
-5.9027581	8.8816004	103.5100021
-5.9027581	8.9303999	105.6699982
-5.9027581	8.9792004	104.2099991
-5.9027581	9.0279999	102.7500000
-5.9027581	9.0768003	105.7200012
-5.9027581	9.1255999	103.8300018
-5.9027581	9.1744003	101.2600021
-5.9027581	9.2231998	102.3899994
-5.9027581	9.2720003	102.6899948

-5.9027581	9.3207998	104.5599976
-5.9027581	9.3696003	99.6899948
-5.9027581	9.4184008	100.2299957
-5.9027581	9.4672003	103.6100006
-5.9027581	9.5160007	103.2699966
-5.9027581	9.5648003	102.6199951
-5.9027581	9.6136007	100.9700012
-5.9027581	9.6624002	102.7699966
-5.9027581	9.7112007	105.4899979
-5.9027581	9.7600002	103.5299988
-5.9027581	9.8088007	104.9399948
-5.9027581	9.8576002	104.2799988
-5.9027581	9.9064007	105.0000000
-5.9027581	9.9552002	103.3600006
-5.9027581	10.0040007	104.9099960
-5.9027581	10.0528002	104.4300003
-5.9027581	10.1016006	104.1100006
-5.9027581	10.1504002	101.4300003
-5.9027581	10.1992006	101.2200012
-5.9027581	10.2480001	101.4300003
-5.9027581	10.2968006	102.4300003
-5.9027581	10.3456001	103.5800018
-5.9027581	10.3944006	105.7900009
-5.9027581	10.4432001	105.1599960
-5.9027581	10.4920006	104.0599976
-5.9027581	10.5408001	101.3799973
-5.9027581	10.5896006	104.7999954
-5.9027581	10.6384001	103.2299957
-5.9027581	10.6872005	100.9499969
-5.9027581	10.7360001	104.0199966
-5.9027581	10.7848005	104.6699982
-5.9027581	10.8336000	104.2399979
-5.9027581	10.8824005	103.1899948
-5.9027581	10.9312000	104.5499954
-5.9027581	10.9800005	101.6199951
-5.9027581	11.0288000	102.8499985
-5.9027581	11.0776005	105.6599960
-5.9027581	11.1264000	105.0000000
-5.9027581	11.1752005	102.1100006
-5.9027581	11.2240000	97.5899963
-5.9027581	11.2728004	104.2799988
-5.9027581	11.3216000	105.9499969
-5.9027581	11.3704004	105.8799973
-5.9027581	11.4191999	102.9000015
-5.9027581	11.4680004	102.4599991
-5.9027581	11.5167999	103.0699997
-5.9027581	11.5656004	100.9700012
-5.9027581	11.6143999	103.2900009
-5.9027581	11.6632004	104.9799957
-5.9027581	11.7119999	105.2399979
-5.9027581	11.7608004	104.2999954
-5.9027581	11.8095999	104.9700012
-5.9027581	11.8584003	103.3600006
-5.9027581	11.9071999	104.3600006
-5.9027581	11.9560003	104.8699951
-5.9027581	12.0047998	106.0199966
-5.9027581	12.0536003	105.0699997
-5.9027581	12.1023998	103.7900009

-5.9027581	12.1512003	105.0999985
-5.9027581	12.1999998	104.2900009
-5.9027581	12.2488003	106.2200012
-5.9027581	12.2975998	104.6100006
-5.9027581	12.3464003	103.5899963
-5.9027581	12.3951998	102.9599991
-5.9027581	12.4440002	103.6500015
-5.9027581	12.4927998	103.9000015
-5.9027581	12.5416002	102.9399948
-5.9027581	12.5904007	104.5699997
-5.9027581	12.6392002	103.2600021
-5.9027581	12.6880007	103.6500015
-5.9027581	12.7368002	104.0000000
-5.9027581	12.7856007	104.8499985
-5.9027581	12.8344002	104.0499954
-5.9027581	12.8832006	103.7699966
-5.9027581	12.9320002	104.3799973
-5.9027581	12.9808006	103.9099960
-5.9027581	13.0296001	104.1800003
-5.9027581	13.0784006	104.3699951
-5.9027581	13.1272001	103.8899994
-5.9027581	13.1760006	101.6199951
-5.9027581	13.2248001	100.9199982
-5.9027581	13.2736006	103.9000015
-5.9027581	13.3224001	101.7900009
-5.9027581	13.3712006	104.0800018
-5.9027581	13.4200001	102.6699982
-5.9027581	13.4688005	103.3300018
-5.9027581	13.5176001	102.0100021
-5.9027581	13.5664005	98.1299973
-5.9027581	13.6152000	100.7900009
-5.9027581	13.6640005	99.9799957
-5.9027581	13.7128000	101.9599991
-5.9027581	13.7616005	103.9799957
-5.9027581	13.8104000	100.2399979
-5.9027581	13.8592005	99.9499969
-5.9027581	13.9080000	101.8399963
-5.9027581	13.9568005	99.2399979
-5.9027581	14.0056000	103.2399979
-5.9027581	14.0544004	102.6199951
-5.9027581	14.1032000	97.4099960
-5.9027581	14.1520004	98.8699951
-5.9027581	14.2007999	102.4399948
-5.9027581	14.2496004	105.0999985
-5.9027581	14.2983999	104.2699966
-5.9027581	14.3472004	106.1699982
-5.9027581	14.3959999	104.5199966
-5.9027581	14.4448004	101.9799957
-5.9027581	14.4935999	102.1299973
-5.9027581	14.5424004	103.1399994
-5.9027581	14.5911999	104.3099976
-5.9027581	14.6400003	104.2699966
-5.9027581	14.6887999	103.2699966
-5.9027581	14.7376003	104.2999954
-5.9027581	14.7863998	104.9899979
-5.9027581	14.8352003	104.4899979
-5.9027581	14.8839998	102.0699997
-5.9027581	14.9328003	100.6500015

-5.9027581	14.9815998	101.9399948
-5.9027581	15.0304003	101.5999985
-5.8966222	0.3416000	106.0599976
-5.8966222	0.3904000	104.3099976
-5.8966222	0.4392000	105.6299973
-5.8966222	0.4880000	104.5299988
-5.8966222	0.5368000	104.0899963
-5.8966222	0.5856000	103.2600021
-5.8966222	0.6344000	105.2999954
-5.8966222	0.6832000	104.1299973
-5.8966222	0.7320000	108.9300003
-5.8966222	0.7808000	108.0100021
-5.8966222	0.8296000	106.8600006
-5.8966222	0.8784000	107.3300018
-5.8966222	0.9272000	109.1500015
-5.8966222	0.9760000	109.0499954
-5.8966222	1.0247999	107.7399979
-5.8966222	1.0735999	105.1699982
-5.8966222	1.1223999	106.4199982
-5.8966222	1.1711999	104.3099976
-5.8966222	1.2200000	104.8699951
-5.8966222	1.2688000	103.8899994
-5.8966222	1.3176000	103.7099991
-5.8966222	1.3664000	106.4499969
-5.8966222	1.4152000	108.5899963
-5.8966222	1.4640000	107.7900009
-5.8966222	1.5128000	109.1899948
-5.8966222	1.5616000	109.1899948
-5.8966222	1.6104000	105.3499985
-5.8966222	1.6592000	109.2999954
-5.8966222	1.7079999	110.0299988
-5.8966222	1.7567999	107.8899994
-5.8966222	1.8055999	107.1500015
-5.8966222	1.8543999	115.9799957
-5.8966222	1.9031999	117.3300018
-5.8966222	1.9519999	112.6100006
-5.8966222	2.0007999	108.4399948
-5.8966222	2.0495999	106.7399979
-5.8966222	2.0983999	105.0800018
-5.8966222	2.1471999	103.9599991
-5.8966222	2.1959999	108.0400009
-5.8966222	2.2447999	106.9000015
-5.8966222	2.2935998	104.3399963
-5.8966222	2.3423998	104.2200012
-5.8966222	2.3912001	105.9000015
-5.8966222	2.4400001	108.1299973
-5.8966222	2.4888000	106.3099976
-5.8966222	2.5376000	105.2500000
-5.8966222	2.5864000	104.9799957
-5.8966222	2.6352000	106.5800018
-5.8966222	2.6840000	106.1999969
-5.8966222	2.7328000	107.3799973
-5.8966222	2.7816000	106.1500015
-5.8966222	2.8304000	105.5000000
-5.8966222	2.8792000	106.4199982
-5.8966222	2.9280000	108.6199951
-5.8966222	2.9768000	107.3399963
-5.8966222	3.0256000	105.9799957

-5.8966222	3.0743999	105.0199966
-5.8966222	3.1231999	104.7099991
-5.8966222	3.1719999	104.7999954
-5.8966222	3.2207999	106.7099991
-5.8966222	3.2695999	106.0400009
-5.8966222	3.3183999	101.5999985
-5.8966222	3.3671999	103.4599991
-5.8966222	3.4159999	103.5699997
-5.8966222	3.4647999	102.2099991
-5.8966222	3.5135999	101.7600021
-5.8966222	3.5623999	102.8499985
-5.8966222	3.6111999	103.3399963
-5.8966222	3.6599998	104.7900009
-5.8966222	3.7087998	106.4899979
-5.8966222	3.7575998	104.9000015
-5.8966222	3.8063998	103.5800018
-5.8966222	3.8551998	102.2299957
-5.8966222	3.9039998	102.4700012
-5.8966222	3.9527998	101.3600006
-5.8966222	4.0016003	103.3199997
-5.8966222	4.0503998	104.9799957
-5.8966222	4.0991998	107.6599960
-5.8966222	4.1479998	106.7600021
-5.8966222	4.1967998	105.3600006
-5.8966222	4.2455997	103.2099991
-5.8966222	4.2943997	106.9599991
-5.8966222	4.3431997	111.1999969
-5.8966222	4.3919997	110.3300018
-5.8966222	4.4407997	107.7099991
-5.8966222	4.4895997	106.9199982
-5.8966222	4.5383997	112.5999985
-5.8966222	4.5871997	114.4599991
-5.8966222	4.6359997	115.2099991
-5.8966222	4.6847997	117.0899963
-5.8966222	4.7336001	115.6899948
-5.8966222	4.7824001	108.6199951
-5.8966222	4.8312001	105.5000000
-5.8966222	4.8800001	108.0699997
-5.8966222	4.9288001	108.5100021
-5.8966222	4.9776001	106.1599960
-5.8966222	5.0264001	107.2799988
-5.8966222	5.0752001	105.7900009
-5.8966222	5.1240001	103.6699982
-5.8966222	5.1728001	105.0299988
-5.8966222	5.2216001	103.5400009
-5.8966222	5.2704000	107.0899963
-5.8966222	5.3192000	104.2699966
-5.8966222	5.3680000	104.4599991
-5.8966222	5.4168000	102.9000015
-5.8966222	5.4656000	103.4399948
-5.8966222	5.5144000	104.7500000
-5.8966222	5.5632000	106.0000000
-5.8966222	5.6120000	105.3899994
-5.8966222	5.6608000	106.7399979
-5.8966222	5.7096000	107.9799957
-5.8966222	5.7584000	107.4799957
-5.8966222	5.8072000	103.6299973
-5.8966222	5.8559999	101.6699982

-5.8966222	5.9047999	105.0400009
-5.8966222	5.9535999	104.0800018
-5.8966222	6.0023999	101.4300003
-5.8966222	6.0511999	103.8099976
-5.8966222	6.0999999	103.3499985
-5.8966222	6.1487999	105.4499969
-5.8966222	6.1975999	103.6500015
-5.8966222	6.2463999	106.4300003
-5.8966222	6.2951999	107.9799957
-5.8966222	6.3439999	106.6500015
-5.8966222	6.3927999	106.4099960
-5.8966222	6.4415998	105.5100021
-5.8966222	6.4903998	104.0899963
-5.8966222	6.5391998	104.4000015
-5.8966222	6.5879998	102.1199951
-5.8966222	6.6367998	104.2600021
-5.8966222	6.6855998	105.8699951
-5.8966222	6.7343998	106.3499985
-5.8966222	6.7831998	104.5899963
-5.8966222	6.8319998	103.0599976
-5.8966222	6.8807998	102.9300003
-5.8966222	6.9295998	104.8399963
-5.8966222	6.9783998	101.3300018
-5.8966222	7.0271997	99.5800018
-5.8966222	7.0759997	103.1199951
-5.8966222	7.1247997	103.0699997
-5.8966222	7.1735997	101.5199966
-5.8966222	7.2223997	101.6599960
-5.8966222	7.2711997	102.3899994
-5.8966222	7.3199997	103.3799973
-5.8966222	7.3687997	106.9399948
-5.8966222	7.4175997	107.0400009
-5.8966222	7.4663997	106.8099976
-5.8966222	7.5151997	99.6199951
-5.8966222	7.5639997	97.7500000
-5.8966222	7.6127996	99.8300018
-5.8966222	7.6615996	103.7099991
-5.8966222	7.7103996	105.9300003
-5.8966222	7.7591996	104.4499969
-5.8966222	7.8079996	105.0000000
-5.8966222	7.8567996	104.5699997
-5.8966222	7.9056001	105.5499954
-5.8966222	7.9544001	102.7200012
-5.8966222	8.0032005	102.2600021
-5.8966222	8.0520000	102.4499969
-5.8966222	8.1008005	101.0999985
-5.8966222	8.1496000	102.0000000
-5.8966222	8.1984005	102.5299988
-5.8966222	8.2472000	104.0000000
-5.8966222	8.2960005	105.5100021
-5.8966222	8.3448000	106.8399963
-5.8966222	8.3936005	103.7900009
-5.8966222	8.4424000	101.1299973
-5.8966222	8.4912004	103.7699966
-5.8966222	8.5400000	105.1699982
-5.8966222	8.5888004	101.5100021
-5.8966222	8.6375999	104.2399979
-5.8966222	8.6864004	104.1500015

-5.8966222	8.7351999	105.1999969
-5.8966222	8.7840004	104.1699982
-5.8966222	8.8327999	102.7900009
-5.8966222	8.8816004	104.3499985
-5.8966222	8.9303999	106.3399963
-5.8966222	8.9792004	104.5000000
-5.8966222	9.0279999	101.9099960
-5.8966222	9.0768003	105.7200012
-5.8966222	9.1255999	105.0000000
-5.8966222	9.1744003	102.1299973
-5.8966222	9.2231998	101.3099976
-5.8966222	9.2720003	101.8300018
-5.8966222	9.3207998	104.5000000
-5.8966222	9.3696003	100.3199997
-5.8966222	9.4184008	99.8499985
-5.8966222	9.4672003	103.5100021
-5.8966222	9.5160007	103.8399963
-5.8966222	9.5648003	103.4099960
-5.8966222	9.6136007	101.0199966
-5.8966222	9.6624002	102.1999969
-5.8966222	9.7112007	105.0199966
-5.8966222	9.7600002	103.5000000
-5.8966222	9.8088007	104.3499985
-5.8966222	9.8576002	102.1500015
-5.8966222	9.9064007	104.0100021
-5.8966222	9.9552002	103.4700012
-5.8966222	10.0040007	104.2900009
-5.8966222	10.0528002	104.5999985
-5.8966222	10.1016006	104.6299973
-5.8966222	10.1504002	102.0899963
-5.8966222	10.1992006	101.1299973
-5.8966222	10.2480001	101.7699966
-5.8966222	10.2968006	103.2299957
-5.8966222	10.3456001	105.1699982
-5.8966222	10.3944006	106.5999985
-5.8966222	10.4432001	105.2500000
-5.8966222	10.4920006	104.4599991
-5.8966222	10.5408001	101.6100006
-5.8966222	10.5896006	105.1500015
-5.8966222	10.6384001	103.5699997
-5.8966222	10.6872005	101.3300018
-5.8966222	10.7360001	104.2399979
-5.8966222	10.7848005	104.2799988
-5.8966222	10.8336000	103.7900009
-5.8966222	10.8824005	102.0299988
-5.8966222	10.9312000	103.0000000
-5.8966222	10.9800005	100.9199982
-5.8966222	11.0288000	102.1299973
-5.8966222	11.0776005	105.0400009
-5.8966222	11.1264000	104.9099960
-5.8966222	11.1752005	102.5499954
-5.8966222	11.2240000	98.9000015
-5.8966222	11.2728004	104.1299973
-5.8966222	11.3216000	105.1800003
-5.8966222	11.3704004	105.2099991
-5.8966222	11.4191999	103.0800018
-5.8966222	11.4680004	103.8199997
-5.8966222	11.5167999	104.1500015

-5.8966222	11.5656004	101.1299973
-5.8966222	11.6143999	102.9700012
-5.8966222	11.6632004	105.3799973
-5.8966222	11.7119999	105.8799973
-5.8966222	11.7608004	105.2200012
-5.8966222	11.8095999	105.3099976
-5.8966222	11.8584003	103.9399948
-5.8966222	11.9071999	104.7299957
-5.8966222	11.9560003	104.8899994
-5.8966222	12.0047998	106.0800018
-5.8966222	12.0536003	104.5299988
-5.8966222	12.1023998	103.4300003
-5.8966222	12.1512003	104.1800003
-5.8966222	12.1999998	101.6199951
-5.8966222	12.2488003	105.3499985
-5.8966222	12.2975998	104.9399948
-5.8966222	12.3464003	104.0599976
-5.8966222	12.3951998	103.7299957
-5.8966222	12.4440002	103.5899963
-5.8966222	12.4927998	103.3499985
-5.8966222	12.5416002	102.3799973
-5.8966222	12.5904007	105.3399963
-5.8966222	12.6392002	104.1699982
-5.8966222	12.6880007	103.2600021
-5.8966222	12.7368002	104.5699997
-5.8966222	12.7856007	105.7399979
-5.8966222	12.8344002	105.0899963
-5.8966222	12.8832006	104.8600006
-5.8966222	12.9320002	104.0299988
-5.8966222	12.9808006	103.0899963
-5.8966222	13.0296001	104.0599976
-5.8966222	13.0784006	104.6899948
-5.8966222	13.1272001	103.9899979
-5.8966222	13.1760006	101.9399948
-5.8966222	13.2248001	101.6299973
-5.8966222	13.2736006	104.0999985
-5.8966222	13.3224001	102.0199966
-5.8966222	13.3712006	104.8099976
-5.8966222	13.4200001	103.5599976
-5.8966222	13.4688005	103.7500000
-5.8966222	13.5176001	102.2299957
-5.8966222	13.5664005	97.4599991
-5.8966222	13.6152000	100.6800003
-5.8966222	13.6640005	99.7200012
-5.8966222	13.7128000	100.5599976
-5.8966222	13.7616005	103.3099976
-5.8966222	13.8104000	101.0000000
-5.8966222	13.8592005	100.3099976
-5.8966222	13.9080000	101.7999954
-5.8966222	13.9568005	99.8799973
-5.8966222	14.0056000	103.1199951
-5.8966222	14.0544004	102.2099991
-5.8966222	14.1032000	97.5299988
-5.8966222	14.1520004	99.0400009
-5.8966222	14.2007999	103.3899994
-5.8966222	14.2496004	105.5999985
-5.8966222	14.2983999	102.4799957
-5.8966222	14.3472004	104.8499985

-5.8966222	14.3959999	103.3699951
-5.8966222	14.4448004	100.9399948
-5.8966222	14.4935999	102.5100021
-5.8966222	14.5424004	103.1999969
-5.8966222	14.5911999	104.1699982
-5.8966222	14.6400003	103.3499985
-5.8966222	14.6887999	101.8099976
-5.8966222	14.7376003	103.6999969
-5.8966222	14.7863998	104.5899963
-5.8966222	14.8352003	104.1500015
-5.8966222	14.8839998	102.0299988
-5.8966222	14.9328003	100.5000000
-5.8966222	14.9815998	100.3499985
-5.8966222	15.0304003	100.4799957
-5.8904862	0.3416000	105.6699982
-5.8904862	0.3904000	104.0199966
-5.8904862	0.4392000	105.6800003
-5.8904862	0.4880000	104.4000015
-5.8904862	0.5368000	104.8099976
-5.8904862	0.5856000	104.1699982
-5.8904862	0.6344000	104.5800018
-5.8904862	0.6832000	103.2200012
-5.8904862	0.7320000	107.8699951
-5.8904862	0.7808000	106.7200012
-5.8904862	0.8296000	106.6599960
-5.8904862	0.8784000	107.3699951
-5.8904862	0.9272000	108.8199997
-5.8904862	0.9760000	108.4399948
-5.8904862	1.0247999	106.8600006
-5.8904862	1.0735999	103.3099976
-5.8904862	1.1223999	105.1199951
-5.8904862	1.1711999	103.6299973
-5.8904862	1.2200000	104.9499969
-5.8904862	1.2688000	104.8300018
-5.8904862	1.3176000	104.3600006
-5.8904862	1.3664000	107.0199966
-5.8904862	1.4152000	108.5199966
-5.8904862	1.4640000	106.5899963
-5.8904862	1.5128000	107.5699997
-5.8904862	1.5616000	107.4199982
-5.8904862	1.6104000	105.0100021
-5.8904862	1.6592000	109.3499985
-5.8904862	1.7079999	109.7600021
-5.8904862	1.7567999	107.0100021
-5.8904862	1.8055999	107.7500000
-5.8904862	1.8543999	115.7200012
-5.8904862	1.9031999	116.8600006
-5.8904862	1.9519999	112.1299973
-5.8904862	2.0007999	108.6299973
-5.8904862	2.0495999	106.7399979
-5.8904862	2.0983999	103.7200012
-5.8904862	2.1471999	104.5400009
-5.8904862	2.1959999	107.7200012
-5.8904862	2.2447999	106.9899979
-5.8904862	2.2935998	104.1399994
-5.8904862	2.3423998	105.1999969
-5.8904862	2.3912001	107.0999985
-5.8904862	2.4400001	108.6199951

-5.8904862	2.4888000	106.0299988
-5.8904862	2.5376000	105.3099976
-5.8904862	2.5864000	105.7799988
-5.8904862	2.6352000	106.6199951
-5.8904862	2.6840000	105.8699951
-5.8904862	2.7328000	107.7900009
-5.8904862	2.7816000	105.7399979
-5.8904862	2.8304000	104.3799973
-5.8904862	2.8792000	105.7699966
-5.8904862	2.9280000	107.9399948
-5.8904862	2.9768000	107.1500015
-5.8904862	3.0256000	105.5899963
-5.8904862	3.0743999	103.8600006
-5.8904862	3.1231999	104.9799957
-5.8904862	3.1719999	106.3399963
-5.8904862	3.2207999	106.9199982
-5.8904862	3.2695999	105.7900009
-5.8904862	3.3183999	102.5400009
-5.8904862	3.3671999	103.7399979
-5.8904862	3.4159999	102.9599991
-5.8904862	3.4647999	101.6100006
-5.8904862	3.5135999	101.0999985
-5.8904862	3.5623999	102.7399979
-5.8904862	3.6111999	103.3300018
-5.8904862	3.6599998	104.1199951
-5.8904862	3.7087998	105.7799988
-5.8904862	3.7575998	104.1299973
-5.8904862	3.8063998	101.7500000
-5.8904862	3.8551998	101.0800018
-5.8904862	3.9039998	102.1699982
-5.8904862	3.9527998	102.0699997
-5.8904862	4.0016003	104.0000000
-5.8904862	4.0503998	105.5800018
-5.8904862	4.0991998	107.5599976
-5.8904862	4.1479998	106.3099976
-5.8904862	4.1967998	104.6699982
-5.8904862	4.2455997	103.9799957
-5.8904862	4.2943997	106.8099976
-5.8904862	4.3431997	110.8899994
-5.8904862	4.3919997	109.5499954
-5.8904862	4.4407997	107.4599991
-5.8904862	4.4895997	105.9700012
-5.8904862	4.5383997	111.9899979
-5.8904862	4.5871997	115.0000000
-5.8904862	4.6359997	115.3499985
-5.8904862	4.6847997	116.7500000
-5.8904862	4.7336001	115.2999954
-5.8904862	4.7824001	107.5199966
-5.8904862	4.8312001	104.9000015
-5.8904862	4.8800001	108.2900009
-5.8904862	4.9288001	108.8600006
-5.8904862	4.9776001	105.7799988
-5.8904862	5.0264001	106.8899994
-5.8904862	5.0752001	105.5800018
-5.8904862	5.1240001	103.5299988
-5.8904862	5.1728001	106.5599976
-5.8904862	5.2216001	105.2999954
-5.8904862	5.2704000	107.2200012

-5.8904862	5.3192000	102.8399963
-5.8904862	5.3680000	103.7399979
-5.8904862	5.4168000	103.3399963
-5.8904862	5.4656000	103.7299957
-5.8904862	5.5144000	105.3799973
-5.8904862	5.5632000	106.3199997
-5.8904862	5.6120000	105.4199982
-5.8904862	5.6608000	106.0899963
-5.8904862	5.7096000	107.3799973
-5.8904862	5.7584000	107.7799988
-5.8904862	5.8072000	103.9700012
-5.8904862	5.8559999	101.0800018
-5.8904862	5.9047999	104.4799957
-5.8904862	5.9535999	103.9700012
-5.8904862	6.0023999	100.7900009
-5.8904862	6.0511999	104.4199982
-5.8904862	6.0999999	104.1999969
-5.8904862	6.1487999	105.3600006
-5.8904862	6.1975999	103.3399963
-5.8904862	6.2463999	105.3099976
-5.8904862	6.2951999	107.2200012
-5.8904862	6.3439999	106.7099991
-5.8904862	6.3927999	106.7200012
-5.8904862	6.4415998	106.1899948
-5.8904862	6.4903998	105.3899994
-5.8904862	6.5391998	104.3600006
-5.8904862	6.5879998	101.7999954
-5.8904862	6.6367998	103.6399994
-5.8904862	6.6855998	105.4399948
-5.8904862	6.7343998	106.6100006
-5.8904862	6.7831998	105.0299988
-5.8904862	6.8319998	102.9099960
-5.8904862	6.8807998	102.2500000
-5.8904862	6.9295998	104.2999954
-5.8904862	6.9783998	100.8099976
-5.8904862	7.0271997	99.6500015
-5.8904862	7.0759997	103.9599991
-5.8904862	7.1247997	103.2900009
-5.8904862	7.1735997	101.2299957
-5.8904862	7.2223997	102.1699982
-5.8904862	7.2711997	102.8600006
-5.8904862	7.3199997	102.6699982
-5.8904862	7.3687997	106.6500015
-5.8904862	7.4175997	107.0400009
-5.8904862	7.4663997	107.3499985
-5.8904862	7.5151997	101.5999985
-5.8904862	7.5639997	98.1899948
-5.8904862	7.6127996	100.3899994
-5.8904862	7.6615996	104.4300003
-5.8904862	7.7103996	106.5699997
-5.8904862	7.7591996	104.1999969
-5.8904862	7.8079996	104.2999954
-5.8904862	7.8567996	103.8499985
-5.8904862	7.9056001	105.5999985
-5.8904862	7.9544001	103.8399963
-5.8904862	8.0032005	104.1399994
-5.8904862	8.0520000	102.4099960
-5.8904862	8.1008005	100.9899979

-5.8904862	8.1496000	102.0299988
-5.8904862	8.1984005	102.9199982
-5.8904862	8.2472000	104.4199982
-5.8904862	8.2960005	105.5699997
-5.8904862	8.3448000	107.2600021
-5.8904862	8.3936005	104.6100006
-5.8904862	8.4424000	99.3699951
-5.8904862	8.4912004	104.0100021
-5.8904862	8.5400000	104.5299988
-5.8904862	8.5888004	100.3699951
-5.8904862	8.6375999	103.7200012
-5.8904862	8.6864004	103.6399994
-5.8904862	8.7351999	104.6100006
-5.8904862	8.7840004	104.3799973
-5.8904862	8.8327999	104.8099976
-5.8904862	8.8816004	105.5100021
-5.8904862	8.9303999	107.0499954
-5.8904862	8.9792004	104.4700012
-5.8904862	9.0279999	101.2600021
-5.8904862	9.0768003	106.1399994
-5.8904862	9.1255999	106.0699997
-5.8904862	9.1744003	103.0000000
-5.8904862	9.2231998	100.0199966
-5.8904862	9.2720003	101.0299988
-5.8904862	9.3207998	104.4300003
-5.8904862	9.3696003	101.2500000
-5.8904862	9.4184008	100.0999985
-5.8904862	9.4672003	103.7799988
-5.8904862	9.5160007	104.1599960
-5.8904862	9.5648003	103.9099960
-5.8904862	9.6136007	101.6800003
-5.8904862	9.6624002	102.3199997
-5.8904862	9.7112007	104.0800018
-5.8904862	9.7600002	103.0100021
-5.8904862	9.8088007	103.8600006
-5.8904862	9.8576002	101.0699997
-5.8904862	9.9064007	104.0199966
-5.8904862	9.9552002	103.1599960
-5.8904862	10.0040007	103.9199982
-5.8904862	10.0528002	104.5899963
-5.8904862	10.1016006	104.5199966
-5.8904862	10.1504002	102.2799988
-5.8904862	10.1992006	101.5199966
-5.8904862	10.2480001	102.1299973
-5.8904862	10.2968006	103.3899994
-5.8904862	10.3456001	106.1800003
-5.8904862	10.3944006	107.4399948
-5.8904862	10.4432001	105.4099960
-5.8904862	10.4920006	104.7799988
-5.8904862	10.5408001	101.7399979
-5.8904862	10.5896006	105.5400009
-5.8904862	10.6384001	104.1500015
-5.8904862	10.6872005	102.5499954
-5.8904862	10.7360001	104.3799973
-5.8904862	10.7848005	103.5100021
-5.8904862	10.8336000	103.2399979
-5.8904862	10.8824005	101.5400009
-5.8904862	10.9312000	102.5000000

-5.8904862	10.9800005	100.4399948
-5.8904862	11.0288000	101.6800003
-5.8904862	11.0776005	104.4499969
-5.8904862	11.1264000	104.7799988
-5.8904862	11.1752005	102.9599991
-5.8904862	11.2240000	99.8399963
-5.8904862	11.2728004	103.5499954
-5.8904862	11.3216000	104.1800003
-5.8904862	11.3704004	104.3799973
-5.8904862	11.4191999	102.6199951
-5.8904862	11.4680004	104.4899979
-5.8904862	11.5167999	104.9499969
-5.8904862	11.5656004	102.1999969
-5.8904862	11.6143999	103.3899994
-5.8904862	11.6632004	105.6399994
-5.8904862	11.7119999	106.3099976
-5.8904862	11.7608004	106.2200012
-5.8904862	11.8095999	105.9000015
-5.8904862	11.8584003	104.5899963
-5.8904862	11.9071999	105.0199966
-5.8904862	11.9560003	104.7999954
-5.8904862	12.0047998	106.0199966
-5.8904862	12.0536003	104.4799957
-5.8904862	12.1023998	103.9799957
-5.8904862	12.1512003	104.0000000
-5.8904862	12.1999998	99.9199982
-5.8904862	12.2488003	104.9300003
-5.8904862	12.2975998	105.2900009
-5.8904862	12.3464003	104.2699966
-5.8904862	12.3951998	103.8699951
-5.8904862	12.4440002	103.2500000
-5.8904862	12.4927998	102.8499985
-5.8904862	12.5416002	101.8300018
-5.8904862	12.5904007	105.6699982
-5.8904862	12.6392002	104.7500000
-5.8904862	12.6880007	103.0199966
-5.8904862	12.7368002	105.1599960
-5.8904862	12.7856007	106.3099976
-5.8904862	12.8344002	105.6699982
-5.8904862	12.8832006	105.6199951
-5.8904862	12.9320002	103.6999969
-5.8904862	12.9808006	102.2600021
-5.8904862	13.0296001	103.9899979
-5.8904862	13.0784006	104.9599991
-5.8904862	13.1272001	104.3799973
-5.8904862	13.1760006	102.4499969
-5.8904862	13.2248001	102.1899948
-5.8904862	13.2736006	104.1599960
-5.8904862	13.3224001	102.2200012
-5.8904862	13.3712006	105.1299973
-5.8904862	13.4200001	104.1800003
-5.8904862	13.4688005	102.7299957
-5.8904862	13.5176001	101.4899979
-5.8904862	13.5664005	97.6699982
-5.8904862	13.6152000	99.6399994
-5.8904862	13.6640005	99.4099960
-5.8904862	13.7128000	100.3399963
-5.8904862	13.7616005	103.4300003

-5.8904862	13.8104000	102.1199951
-5.8904862	13.8592005	100.8799973
-5.8904862	13.9080000	102.1299973
-5.8904862	13.9568005	100.9700012
-5.8904862	14.0056000	102.7799988
-5.8904862	14.0544004	101.5299988
-5.8904862	14.1032000	98.2399979
-5.8904862	14.1520004	99.3099976
-5.8904862	14.2007999	103.4599991
-5.8904862	14.2496004	105.3699951
-5.8904862	14.2983999	101.2500000
-5.8904862	14.3472004	103.4799957
-5.8904862	14.3959999	102.4700012
-5.8904862	14.4448004	101.1699982
-5.8904862	14.4935999	103.0199966
-5.8904862	14.5424004	103.3300018
-5.8904862	14.5911999	103.9599991
-5.8904862	14.6400003	102.1399994
-5.8904862	14.6887999	100.6399994
-5.8904862	14.7376003	103.1100006
-5.8904862	14.7863998	103.6599960
-5.8904862	14.8352003	103.7299957
-5.8904862	14.8839998	101.9399948
-5.8904862	14.9328003	100.2299957
-5.8904862	14.9815998	98.9300003
-5.8904862	15.0304003	99.7099991
-5.8843503	0.3416000	105.4899979
-5.8843503	0.3904000	103.6899948
-5.8843503	0.4392000	105.5199966
-5.8843503	0.4880000	104.1100006
-5.8843503	0.5368000	105.2799988
-5.8843503	0.5856000	105.5000000
-5.8843503	0.6344000	104.2299957
-5.8843503	0.6832000	102.4799957
-5.8843503	0.7320000	106.6999969
-5.8843503	0.7808000	105.7099991
-5.8843503	0.8296000	107.0199966
-5.8843503	0.8784000	108.1500015
-5.8843503	0.9272000	108.9000015
-5.8843503	0.9760000	107.9899979
-5.8843503	1.0247999	106.6399994
-5.8843503	1.0735999	102.7299957
-5.8843503	1.1223999	104.6500015
-5.8843503	1.1711999	102.7099991
-5.8843503	1.2200000	104.3699951
-5.8843503	1.2688000	105.1399994
-5.8843503	1.3176000	104.9899979
-5.8843503	1.3664000	107.5899963
-5.8843503	1.4152000	108.8799973
-5.8843503	1.4640000	106.4199982
-5.8843503	1.5128000	106.8799973
-5.8843503	1.5616000	105.7999954
-5.8843503	1.6104000	105.1599960
-5.8843503	1.6592000	109.8399963
-5.8843503	1.7079999	109.6299973
-5.8843503	1.7567999	106.0699997
-5.8843503	1.8055999	107.9599991
-5.8843503	1.8543999	115.4899979

-5.8843503	1.9031999	116.7799988
-5.8843503	1.9519999	112.4099960
-5.8843503	2.0007999	109.1599960
-5.8843503	2.0495999	107.7200012
-5.8843503	2.0983999	104.0800018
-5.8843503	2.1471999	105.5000000
-5.8843503	2.1959999	106.2500000
-5.8843503	2.2447999	106.5699997
-5.8843503	2.2935998	103.8099976
-5.8843503	2.3423998	105.2500000
-5.8843503	2.3912001	107.5199966
-5.8843503	2.4400001	108.8300018
-5.8843503	2.4888000	105.8600006
-5.8843503	2.5376000	105.5199966
-5.8843503	2.5864000	106.4099960
-5.8843503	2.6352000	106.5499954
-5.8843503	2.6840000	105.4199982
-5.8843503	2.7328000	107.6299973
-5.8843503	2.7816000	104.9499969
-5.8843503	2.8304000	103.3099976
-5.8843503	2.8792000	105.0999985
-5.8843503	2.9280000	107.1999969
-5.8843503	2.9768000	106.7099991
-5.8843503	3.0256000	104.6399994
-5.8843503	3.0743999	101.9899979
-5.8843503	3.1231999	104.9599991
-5.8843503	3.1719999	106.8799973
-5.8843503	3.2207999	106.3300018
-5.8843503	3.2695999	105.0800018
-5.8843503	3.3183999	103.2799988
-5.8843503	3.3671999	104.2299957
-5.8843503	3.4159999	102.7699966
-5.8843503	3.4647999	101.5899963
-5.8843503	3.5135999	100.9300003
-5.8843503	3.5623999	102.8300018
-5.8843503	3.6111999	103.9799957
-5.8843503	3.6599998	103.7399979
-5.8843503	3.7087998	104.8199997
-5.8843503	3.7575998	103.4199982
-5.8843503	3.8063998	100.1199951
-5.8843503	3.8551998	99.8899994
-5.8843503	3.9039998	101.6800003
-5.8843503	3.9527998	103.2200012
-5.8843503	4.0016003	105.2200012
-5.8843503	4.0503998	106.0400009
-5.8843503	4.0991998	107.2999954
-5.8843503	4.1479998	105.9899979
-5.8843503	4.1967998	104.4899979
-5.8843503	4.2455997	104.5299988
-5.8843503	4.2943997	107.4000015
-5.8843503	4.3431997	110.5999985
-5.8843503	4.3919997	108.2900009
-5.8843503	4.4407997	106.6399994
-5.8843503	4.4895997	105.1599960
-5.8843503	4.5383997	110.7500000
-5.8843503	4.5871997	114.2699966
-5.8843503	4.6359997	114.4899979
-5.8843503	4.6847997	116.2099991

-5.8843503	4.7336001	114.6999969
-5.8843503	4.7824001	106.2399979
-5.8843503	4.8312001	105.3300018
-5.8843503	4.8800001	108.8899994
-5.8843503	4.9288001	109.4700012
-5.8843503	4.9776001	105.7399979
-5.8843503	5.0264001	106.7500000
-5.8843503	5.0752001	105.8499985
-5.8843503	5.1240001	103.3399963
-5.8843503	5.1728001	107.2200012
-5.8843503	5.2216001	105.9099960
-5.8843503	5.2704000	106.6599960
-5.8843503	5.3192000	101.2900009
-5.8843503	5.3680000	103.6899948
-5.8843503	5.4168000	103.8499985
-5.8843503	5.4656000	103.1699982
-5.8843503	5.5144000	105.2600021
-5.8843503	5.5632000	106.1999969
-5.8843503	5.6120000	105.1599960
-5.8843503	5.6608000	105.1999969
-5.8843503	5.7096000	106.7600021
-5.8843503	5.7584000	107.5699997
-5.8843503	5.8072000	103.9000015
-5.8843503	5.8559999	100.7299957
-5.8843503	5.9047999	103.6599960
-5.8843503	5.9535999	104.0100021
-5.8843503	6.0023999	102.2299957
-5.8843503	6.0511999	104.8600006
-5.8843503	6.0999999	104.6199951
-5.8843503	6.1487999	105.3099976
-5.8843503	6.1975999	103.0899963
-5.8843503	6.2463999	104.0999985
-5.8843503	6.2951999	106.1399994
-5.8843503	6.3439999	106.0400009
-5.8843503	6.3927999	106.7200012
-5.8843503	6.4415998	106.4700012
-5.8843503	6.4903998	106.0400009
-5.8843503	6.5391998	103.8899994
-5.8843503	6.5879998	101.4499969
-5.8843503	6.6367998	103.2099991
-5.8843503	6.6855998	105.1800003
-5.8843503	6.7343998	107.0499954
-5.8843503	6.7831998	105.7799988
-5.8843503	6.8319998	102.9000015
-5.8843503	6.8807998	101.1999969
-5.8843503	6.9295998	103.5400009
-5.8843503	6.9783998	100.8899994
-5.8843503	7.0271997	100.4399948
-5.8843503	7.0759997	104.5699997
-5.8843503	7.1247997	103.2200012
-5.8843503	7.1735997	101.0199966
-5.8843503	7.2223997	103.0400009
-5.8843503	7.2711997	103.7500000
-5.8843503	7.3199997	102.3699951
-5.8843503	7.3687997	106.4700012
-5.8843503	7.4175997	106.8099976
-5.8843503	7.4663997	107.7299957
-5.8843503	7.5151997	103.4799957

-5.8843503	7.5639997	99.1299973
-5.8843503	7.6127996	101.4599991
-5.8843503	7.6615996	105.3399963
-5.8843503	7.7103996	106.9300003
-5.8843503	7.7591996	104.1399994
-5.8843503	7.8079996	104.0000000
-5.8843503	7.8567996	103.4599991
-5.8843503	7.9056001	104.7999954
-5.8843503	7.9544001	103.9300003
-5.8843503	8.0032005	104.4399948
-5.8843503	8.0520000	102.4799957
-5.8843503	8.1008005	100.8799973
-5.8843503	8.1496000	102.2200012
-5.8843503	8.1984005	103.4300003
-5.8843503	8.2472000	104.7399979
-5.8843503	8.2960005	105.3799973
-5.8843503	8.3448000	107.1699982
-5.8843503	8.3936005	104.9399948
-5.8843503	8.4424000	97.5000000
-5.8843503	8.4912004	103.8699951
-5.8843503	8.5400000	103.6500015
-5.8843503	8.5888004	100.2200012
-5.8843503	8.6375999	102.8600006
-5.8843503	8.6864004	102.3499985
-5.8843503	8.7351999	103.3699951
-5.8843503	8.7840004	104.5800018
-5.8843503	8.8327999	106.3199997
-5.8843503	8.8816004	105.9899979
-5.8843503	8.9303999	107.0199966
-5.8843503	8.9792004	104.4499969
-5.8843503	9.0279999	100.3399963
-5.8843503	9.0768003	105.7299957
-5.8843503	9.1255999	106.5199966
-5.8843503	9.1744003	103.5400009
-5.8843503	9.2231998	99.0999985
-5.8843503	9.2720003	100.6399994
-5.8843503	9.3207998	104.5199966
-5.8843503	9.3696003	102.0000000
-5.8843503	9.4184008	100.1199951
-5.8843503	9.4672003	103.7299957
-5.8843503	9.5160007	104.0199966
-5.8843503	9.5648003	103.6699982
-5.8843503	9.6136007	100.7999954
-5.8843503	9.6624002	101.8799973
-5.8843503	9.7112007	102.8699951
-5.8843503	9.7600002	102.7799988
-5.8843503	9.8088007	103.5599976
-5.8843503	9.8576002	99.8799973
-5.8843503	9.9064007	103.2099991
-5.8843503	9.9552002	101.4599991
-5.8843503	10.0040007	103.0299988
-5.8843503	10.0528002	104.1899948
-5.8843503	10.1016006	104.1699982
-5.8843503	10.1504002	102.4300003
-5.8843503	10.1992006	101.6100006
-5.8843503	10.2480001	102.6599960
-5.8843503	10.2968006	103.6299973
-5.8843503	10.3456001	106.8499985

-5.8843503	10.3944006	108.0199966
-5.8843503	10.4432001	105.9700012
-5.8843503	10.4920006	105.5800018
-5.8843503	10.5408001	102.0699997
-5.8843503	10.5896006	105.7999954
-5.8843503	10.6384001	104.8199997
-5.8843503	10.6872005	103.3399963
-5.8843503	10.7360001	104.7999954
-5.8843503	10.7848005	103.3899994
-5.8843503	10.8336000	103.5000000
-5.8843503	10.8824005	102.4700012
-5.8843503	10.9312000	102.8600006
-5.8843503	10.9800005	101.5999985
-5.8843503	11.0288000	101.9099960
-5.8843503	11.0776005	104.0999985
-5.8843503	11.1264000	103.7200012
-5.8843503	11.1752005	103.4499969
-5.8843503	11.2240000	100.8899994
-5.8843503	11.2728004	103.1199951
-5.8843503	11.3216000	103.4199982
-5.8843503	11.3704004	103.4199982
-5.8843503	11.4191999	101.8199997
-5.8843503	11.4680004	104.6800003
-5.8843503	11.5167999	104.9899979
-5.8843503	11.5656004	102.5599976
-5.8843503	11.6143999	103.4000015
-5.8843503	11.6632004	105.4899979
-5.8843503	11.7119999	106.3899994
-5.8843503	11.7608004	106.2200012
-5.8843503	11.8095999	105.4399948
-5.8843503	11.8584003	104.0899963
-5.8843503	11.9071999	104.9899979
-5.8843503	11.9560003	104.7900009
-5.8843503	12.0047998	105.4700012
-5.8843503	12.0536003	104.5599976
-5.8843503	12.1023998	104.8300018
-5.8843503	12.1512003	103.8699951
-5.8843503	12.1999998	98.0499954
-5.8843503	12.2488003	104.0699997
-5.8843503	12.2975998	105.1699982
-5.8843503	12.3464003	104.8399963
-5.8843503	12.3951998	103.7099991
-5.8843503	12.4440002	102.5299988
-5.8843503	12.4927998	102.7500000
-5.8843503	12.5416002	102.1899948
-5.8843503	12.5904007	106.2299957
-5.8843503	12.6392002	105.5499954
-5.8843503	12.6880007	102.8199997
-5.8843503	12.7368002	105.0699997
-5.8843503	12.7856007	106.1199951
-5.8843503	12.8344002	105.1599960
-5.8843503	12.8832006	105.3600006
-5.8843503	12.9320002	103.0699997
-5.8843503	12.9808006	100.9099960
-5.8843503	13.0296001	103.7399979
-5.8843503	13.0784006	105.4499969
-5.8843503	13.1272001	105.1699982
-5.8843503	13.1760006	102.7699966

-5.8843503	13.2248001	102.6800003
-5.8843503	13.2736006	104.1500015
-5.8843503	13.3224001	102.3300018
-5.8843503	13.3712006	105.0199966
-5.8843503	13.4200001	104.2200012
-5.8843503	13.4688005	100.5800018
-5.8843503	13.5176001	100.6299973
-5.8843503	13.5664005	98.5899963
-5.8843503	13.6152000	99.2799988
-5.8843503	13.6640005	99.2600021
-5.8843503	13.7128000	100.4000015
-5.8843503	13.7616005	103.3799973
-5.8843503	13.8104000	102.8699951
-5.8843503	13.8592005	101.0299988
-5.8843503	13.9080000	102.1899948
-5.8843503	13.9568005	101.8899994
-5.8843503	14.0056000	102.2500000
-5.8843503	14.0544004	101.5400009
-5.8843503	14.1032000	100.2699966
-5.8843503	14.1520004	101.0999985
-5.8843503	14.2007999	104.0599976
-5.8843503	14.2496004	105.3600006
-5.8843503	14.2983999	101.1299973
-5.8843503	14.3472004	102.4099960
-5.8843503	14.3959999	101.5800018
-5.8843503	14.4448004	101.7699966
-5.8843503	14.4935999	103.8199997
-5.8843503	14.5424004	103.7699966
-5.8843503	14.5911999	104.1999969
-5.8843503	14.6400003	102.0299988
-5.8843503	14.6887999	101.1500015
-5.8843503	14.7376003	103.0999985
-5.8843503	14.7863998	102.5599976
-5.8843503	14.8352003	103.3699951
-5.8843503	14.8839998	102.1599960
-5.8843503	14.9328003	100.5899963
-5.8843503	14.9815998	98.0499954
-5.8843503	15.0304003	99.7200012
-5.8782144	0.3416000	105.0800018
-5.8782144	0.3904000	103.5400009
-5.8782144	0.4392000	105.3899994
-5.8782144	0.4880000	103.6699982
-5.8782144	0.5368000	104.9399948
-5.8782144	0.5856000	106.5800018
-5.8782144	0.6344000	103.9599991
-5.8782144	0.6832000	103.1699982
-5.8782144	0.7320000	105.6199951
-5.8782144	0.7808000	104.4099960
-5.8782144	0.8296000	106.9199982
-5.8782144	0.8784000	108.3600006
-5.8782144	0.9272000	108.4599991
-5.8782144	0.9760000	107.2999954
-5.8782144	1.0247999	106.3799973
-5.8782144	1.0735999	103.0199966
-5.8782144	1.1223999	104.4300003
-5.8782144	1.1711999	101.2200012
-5.8782144	1.2200000	103.3199997
-5.8782144	1.2688000	104.8799973

-5.8782144	1.3176000	105.2900009
-5.8782144	1.3664000	107.6899948
-5.8782144	1.4152000	108.7099991
-5.8782144	1.4640000	106.0299988
-5.8782144	1.5128000	106.4700012
-5.8782144	1.5616000	104.2500000
-5.8782144	1.6104000	104.9300003
-5.8782144	1.6592000	109.6100006
-5.8782144	1.7079999	108.8399963
-5.8782144	1.7567999	104.7399979
-5.8782144	1.8055999	107.5499954
-5.8782144	1.8543999	115.2799988
-5.8782144	1.9031999	117.0299988
-5.8782144	1.9519999	112.9000015
-5.8782144	2.0007999	109.4700012
-5.8782144	2.0495999	108.0999985
-5.8782144	2.0983999	104.0899963
-5.8782144	2.1471999	105.4700012
-5.8782144	2.1959999	104.0699997
-5.8782144	2.2447999	105.4799957
-5.8782144	2.2935998	103.7099991
-5.8782144	2.3423998	105.5000000
-5.8782144	2.3912001	108.1999969
-5.8782144	2.4400001	109.5100021
-5.8782144	2.4888000	106.5999985
-5.8782144	2.5376000	106.0100021
-5.8782144	2.5864000	107.5000000
-5.8782144	2.6352000	107.2999954
-5.8782144	2.6840000	105.6399994
-5.8782144	2.7328000	107.3499985
-5.8782144	2.7816000	103.6100006
-5.8782144	2.8304000	102.4099960
-5.8782144	2.8792000	104.7999954
-5.8782144	2.9280000	106.9700012
-5.8782144	2.9768000	106.8799973
-5.8782144	3.0256000	103.8099976
-5.8782144	3.0743999	100.2600021
-5.8782144	3.1231999	104.9300003
-5.8782144	3.1719999	107.0400009
-5.8782144	3.2207999	105.5999985
-5.8782144	3.2695999	104.2699966
-5.8782144	3.3183999	103.4399948
-5.8782144	3.3671999	104.8199997
-5.8782144	3.4159999	103.7299957
-5.8782144	3.4647999	102.7299957
-5.8782144	3.5135999	101.2799988
-5.8782144	3.5623999	103.2699966
-5.8782144	3.6111999	104.6800003
-5.8782144	3.6599998	103.1899948
-5.8782144	3.7087998	103.7500000
-5.8782144	3.7575998	103.3600006
-5.8782144	3.8063998	100.1800003
-5.8782144	3.8551998	100.4899979
-5.8782144	3.9039998	101.6199951
-5.8782144	3.9527998	104.0199966
-5.8782144	4.0016003	105.6999969
-5.8782144	4.0503998	105.7200012
-5.8782144	4.0991998	107.1899948

-5.8782144	4.1479998	106.4300003
-5.8782144	4.1967998	104.9300003
-5.8782144	4.2455997	105.6299973
-5.8782144	4.2943997	108.2900009
-5.8782144	4.3431997	110.8899994
-5.8782144	4.3919997	108.2999954
-5.8782144	4.4407997	106.2200012
-5.8782144	4.4895997	104.7900009
-5.8782144	4.5383997	109.5899963
-5.8782144	4.5871997	113.5100021
-5.8782144	4.6359997	113.9599991
-5.8782144	4.6847997	116.4599991
-5.8782144	4.7336001	115.0299988
-5.8782144	4.7824001	106.3199997
-5.8782144	4.8312001	105.9599991
-5.8782144	4.8800001	109.2799988
-5.8782144	4.9288001	109.6500015
-5.8782144	4.9776001	105.0400009
-5.8782144	5.0264001	105.9799957
-5.8782144	5.0752001	106.1500015
-5.8782144	5.1240001	103.9300003
-5.8782144	5.1728001	107.4000015
-5.8782144	5.2216001	105.4099960
-5.8782144	5.2704000	105.6599960
-5.8782144	5.3192000	99.6599960
-5.8782144	5.3680000	103.1299973
-5.8782144	5.4168000	103.9199982
-5.8782144	5.4656000	102.4300003
-5.8782144	5.5144000	105.6599960
-5.8782144	5.5632000	106.4899979
-5.8782144	5.6120000	104.9000015
-5.8782144	5.6608000	104.0999985
-5.8782144	5.7096000	106.0100021
-5.8782144	5.7584000	107.2900009
-5.8782144	5.8072000	104.4300003
-5.8782144	5.8559999	100.4399948
-5.8782144	5.9047999	102.9000015
-5.8782144	5.9535999	104.1800003
-5.8782144	6.0023999	103.6199951
-5.8782144	6.0511999	105.4599991
-5.8782144	6.0999999	105.0299988
-5.8782144	6.1487999	105.1500015
-5.8782144	6.1975999	102.6999969
-5.8782144	6.2463999	103.3300018
-5.8782144	6.2951999	105.1599960
-5.8782144	6.3439999	104.9000015
-5.8782144	6.3927999	106.4499969
-5.8782144	6.4415998	106.5899963
-5.8782144	6.4903998	105.9799957
-5.8782144	6.5391998	103.4599991
-5.8782144	6.5879998	101.6800003
-5.8782144	6.6367998	102.6500015
-5.8782144	6.6855998	104.1899948
-5.8782144	6.7343998	106.4599991
-5.8782144	6.7831998	105.9199982
-5.8782144	6.8319998	103.0100021
-5.8782144	6.8807998	101.1599960
-5.8782144	6.9295998	103.1899948

-5.8782144	6.9783998	100.8699951
-5.8782144	7.0271997	100.8799973
-5.8782144	7.0759997	104.3699951
-5.8782144	7.1247997	103.2299957
-5.8782144	7.1735997	101.2200012
-5.8782144	7.2223997	104.3300018
-5.8782144	7.2711997	105.0999985
-5.8782144	7.3199997	102.3600006
-5.8782144	7.3687997	105.4899979
-5.8782144	7.4175997	105.8099976
-5.8782144	7.4663997	107.2399979
-5.8782144	7.5151997	104.5100021
-5.8782144	7.5639997	100.1999969
-5.8782144	7.6127996	102.0800018
-5.8782144	7.6615996	106.1899948
-5.8782144	7.7103996	107.0400009
-5.8782144	7.7591996	103.9000015
-5.8782144	7.8079996	103.6999969
-5.8782144	7.8567996	102.7999954
-5.8782144	7.9056001	103.3099976
-5.8782144	7.9544001	103.6699982
-5.8782144	8.0032005	104.3499985
-5.8782144	8.0520000	102.5199966
-5.8782144	8.1008005	100.7799988
-5.8782144	8.1496000	102.3899994
-5.8782144	8.1984005	103.5100021
-5.8782144	8.2472000	105.1899948
-5.8782144	8.2960005	105.3600006
-5.8782144	8.3448000	106.8199997
-5.8782144	8.3936005	104.8899994
-5.8782144	8.4424000	97.2999954
-5.8782144	8.4912004	103.6999969
-5.8782144	8.5400000	102.6899948
-5.8782144	8.5888004	101.3699951
-5.8782144	8.6375999	102.9499969
-5.8782144	8.6864004	101.7099991
-5.8782144	8.7351999	102.5199966
-5.8782144	8.7840004	104.3300018
-5.8782144	8.8327999	106.2399979
-5.8782144	8.8816004	105.7399979
-5.8782144	8.9303999	106.3199997
-5.8782144	8.9792004	103.3300018
-5.8782144	9.0279999	100.3699951
-5.8782144	9.0768003	104.9899979
-5.8782144	9.1255999	106.4899979
-5.8782144	9.1744003	103.8699951
-5.8782144	9.2231998	98.3499985
-5.8782144	9.2720003	100.9599991
-5.8782144	9.3207998	104.0999985
-5.8782144	9.3696003	102.3499985
-5.8782144	9.4184008	100.7799988
-5.8782144	9.4672003	103.3499985
-5.8782144	9.5160007	102.5699997
-5.8782144	9.5648003	103.0899963
-5.8782144	9.6136007	100.8099976
-5.8782144	9.6624002	101.4899979
-5.8782144	9.7112007	101.5599976
-5.8782144	9.7600002	102.2999954

-5.8782144	9.8088007	103.2500000
-5.8782144	9.8576002	100.7900009
-5.8782144	9.9064007	103.4099960
-5.8782144	9.9552002	100.6199951
-5.8782144	10.0040007	102.3199997
-5.8782144	10.0528002	103.8499985
-5.8782144	10.1016006	103.8399963
-5.8782144	10.1504002	103.1199951
-5.8782144	10.1992006	101.5800018
-5.8782144	10.2480001	102.9199982
-5.8782144	10.2968006	103.6500015
-5.8782144	10.3456001	107.0199966
-5.8782144	10.3944006	108.1999969
-5.8782144	10.4432001	106.0800018
-5.8782144	10.4920006	105.6699982
-5.8782144	10.5408001	101.8600006
-5.8782144	10.5896006	106.0800018
-5.8782144	10.6384001	105.1299973
-5.8782144	10.6872005	102.9300003
-5.8782144	10.7360001	104.4000015
-5.8782144	10.7848005	103.5800018
-5.8782144	10.8336000	104.1399994
-5.8782144	10.8824005	103.5699997
-5.8782144	10.9312000	102.7799988
-5.8782144	10.9800005	101.6199951
-5.8782144	11.0288000	101.9199982
-5.8782144	11.0776005	103.6199951
-5.8782144	11.1264000	102.5899963
-5.8782144	11.1752005	103.8600006
-5.8782144	11.2240000	102.1199951
-5.8782144	11.2728004	103.1100006
-5.8782144	11.3216000	103.0699997
-5.8782144	11.3704004	102.7699966
-5.8782144	11.4191999	101.2099991
-5.8782144	11.4680004	104.6100006
-5.8782144	11.5167999	105.1299973
-5.8782144	11.5656004	103.2200012
-5.8782144	11.6143999	103.7399979
-5.8782144	11.6632004	104.8300018
-5.8782144	11.7119999	105.8899994
-5.8782144	11.7608004	105.6399994
-5.8782144	11.8095999	105.3099976
-5.8782144	11.8584003	104.0899963
-5.8782144	11.9071999	105.1199951
-5.8782144	11.9560003	105.1699982
-5.8782144	12.0047998	104.9899979
-5.8782144	12.0536003	104.7399979
-5.8782144	12.1023998	105.8199997
-5.8782144	12.1512003	103.6199951
-5.8782144	12.1999998	96.1100006
-5.8782144	12.2488003	103.5999985
-5.8782144	12.2975998	105.4099960
-5.8782144	12.3464003	105.3199997
-5.8782144	12.3951998	103.8300018
-5.8782144	12.4440002	101.9399948
-5.8782144	12.4927998	102.3899994
-5.8782144	12.5416002	102.1599960
-5.8782144	12.5904007	106.6399994

-5.8782144	12.6392002	105.9099960
-5.8782144	12.6880007	102.5899963
-5.8782144	12.7368002	104.8699951
-5.8782144	12.7856007	105.3399963
-5.8782144	12.8344002	104.6399994
-5.8782144	12.8832006	105.4399948
-5.8782144	12.9320002	103.3699951
-5.8782144	12.9808006	100.5899963
-5.8782144	13.0296001	104.2099991
-5.8782144	13.0784006	105.9799957
-5.8782144	13.1272001	105.5599976
-5.8782144	13.1760006	102.7299957
-5.8782144	13.2248001	103.0000000
-5.8782144	13.2736006	104.4199982
-5.8782144	13.3224001	102.4799957
-5.8782144	13.3712006	105.0599976
-5.8782144	13.4200001	103.9399948
-5.8782144	13.4688005	97.5599976
-5.8782144	13.5176001	99.0400009
-5.8782144	13.5664005	99.3399963
-5.8782144	13.6152000	99.1999969
-5.8782144	13.6640005	99.9899979
-5.8782144	13.7128000	100.8300018
-5.8782144	13.7616005	103.7799988
-5.8782144	13.8104000	102.9799957
-5.8782144	13.8592005	101.6599960
-5.8782144	13.9080000	102.3799973
-5.8782144	13.9568005	102.6999969
-5.8782144	14.0056000	101.7399979
-5.8782144	14.0544004	101.8199997
-5.8782144	14.1032000	102.5100021
-5.8782144	14.1520004	102.4700012
-5.8782144	14.2007999	104.5599976
-5.8782144	14.2496004	105.2999954
-5.8782144	14.2983999	101.1699982
-5.8782144	14.3472004	101.7999954
-5.8782144	14.3959999	100.9899979
-5.8782144	14.4448004	102.3899994
-5.8782144	14.4935999	104.7799988
-5.8782144	14.5424004	104.7900009
-5.8782144	14.5911999	104.6299973
-5.8782144	14.6400003	101.9799957
-5.8782144	14.6887999	101.5199966
-5.8782144	14.7376003	102.5899963
-5.8782144	14.7863998	101.4700012
-5.8782144	14.8352003	103.2900009
-5.8782144	14.8839998	102.4000015
-5.8782144	14.9328003	100.3799973
-5.8782144	14.9815998	97.9399948
-5.8782144	15.0304003	99.7699966
-5.8720784	0.3416000	104.8300018
-5.8720784	0.3904000	104.0000000
-5.8720784	0.4392000	106.1999969
-5.8720784	0.4880000	104.5800018
-5.8720784	0.5368000	104.4799957
-5.8720784	0.5856000	106.4599991
-5.8720784	0.6344000	104.8499985
-5.8720784	0.6832000	103.6999969

-5.8720784	0.7320000	105.7699966
-5.8720784	0.7808000	103.9700012
-5.8720784	0.8296000	107.2200012
-5.8720784	0.8784000	108.5999985
-5.8720784	0.9272000	107.6699982
-5.8720784	0.9760000	106.2099991
-5.8720784	1.0247999	105.5000000
-5.8720784	1.0735999	102.8600006
-5.8720784	1.1223999	104.5100021
-5.8720784	1.1711999	100.7099991
-5.8720784	1.2200000	102.5199966
-5.8720784	1.2688000	104.5499954
-5.8720784	1.3176000	105.4000015
-5.8720784	1.3664000	107.4099960
-5.8720784	1.4152000	107.5299988
-5.8720784	1.4640000	105.0199966
-5.8720784	1.5128000	106.1100006
-5.8720784	1.5616000	103.5999985
-5.8720784	1.6104000	104.9499969
-5.8720784	1.6592000	109.3699951
-5.8720784	1.7079999	108.3899994
-5.8720784	1.7567999	103.7200012
-5.8720784	1.8055999	106.9300003
-5.8720784	1.8543999	114.9599991
-5.8720784	1.9031999	117.1699982
-5.8720784	1.9519999	113.2699966
-5.8720784	2.0007999	109.4399948
-5.8720784	2.0495999	108.0199966
-5.8720784	2.0983999	104.0499954
-5.8720784	2.1471999	105.2099991
-5.8720784	2.1959999	101.9700012
-5.8720784	2.2447999	104.1999969
-5.8720784	2.2935998	103.8300018
-5.8720784	2.3423998	105.7999954
-5.8720784	2.3912001	108.2999954
-5.8720784	2.4400001	109.6800003
-5.8720784	2.4888000	107.3300018
-5.8720784	2.5376000	106.7900009
-5.8720784	2.5864000	107.9799957
-5.8720784	2.6352000	107.6299973
-5.8720784	2.6840000	105.5000000
-5.8720784	2.7328000	106.5999985
-5.8720784	2.7816000	102.3600006
-5.8720784	2.8304000	102.2900009
-5.8720784	2.8792000	104.9700012
-5.8720784	2.9280000	107.0199966
-5.8720784	2.9768000	107.2399979
-5.8720784	3.0256000	103.7200012
-5.8720784	3.0743999	98.8399963
-5.8720784	3.1231999	104.4199982
-5.8720784	3.1719999	106.6399994
-5.8720784	3.2207999	105.0299988
-5.8720784	3.2695999	103.6100006
-5.8720784	3.3183999	104.1899948
-5.8720784	3.3671999	105.3399963
-5.8720784	3.4159999	104.2500000
-5.8720784	3.4647999	102.9399948
-5.8720784	3.5135999	101.2399979

-5.8720784	3.5623999	103.9399948
-5.8720784	3.6111999	105.0199966
-5.8720784	3.6599998	102.6999969
-5.8720784	3.7087998	103.0699997
-5.8720784	3.7575998	103.5499954
-5.8720784	3.8063998	99.8199997
-5.8720784	3.8551998	100.6699982
-5.8720784	3.9039998	102.4799957
-5.8720784	3.9527998	105.4399948
-5.8720784	4.0016003	106.7099991
-5.8720784	4.0503998	105.6100006
-5.8720784	4.0991998	107.1399994
-5.8720784	4.1479998	106.6500015
-5.8720784	4.1967998	105.0499954
-5.8720784	4.2455997	105.7799988
-5.8720784	4.2943997	108.6199951
-5.8720784	4.3431997	110.7200012
-5.8720784	4.3919997	107.4099960
-5.8720784	4.4407997	105.9199982
-5.8720784	4.4895997	105.7399979
-5.8720784	4.5383997	108.5400009
-5.8720784	4.5871997	112.5499954
-5.8720784	4.6359997	113.1800003
-5.8720784	4.6847997	116.2699966
-5.8720784	4.7336001	114.8899994
-5.8720784	4.7824001	106.3499985
-5.8720784	4.8312001	106.9499969
-5.8720784	4.8800001	110.0000000
-5.8720784	4.9288001	109.5499954
-5.8720784	4.9776001	103.7200012
-5.8720784	5.0264001	104.3399963
-5.8720784	5.0752001	105.1599960
-5.8720784	5.1240001	104.6299973
-5.8720784	5.1728001	107.0499954
-5.8720784	5.2216001	104.7600021
-5.8720784	5.2704000	104.9199982
-5.8720784	5.3192000	100.4300003
-5.8720784	5.3680000	103.4899979
-5.8720784	5.4168000	104.7299957
-5.8720784	5.4656000	102.7900009
-5.8720784	5.5144000	105.8899994
-5.8720784	5.5632000	106.0400009
-5.8720784	5.6120000	104.5400009
-5.8720784	5.6608000	102.9700012
-5.8720784	5.7096000	105.5400009
-5.8720784	5.7584000	107.0199966
-5.8720784	5.8072000	104.4000015
-5.8720784	5.8559999	99.8699951
-5.8720784	5.9047999	102.1100006
-5.8720784	5.9535999	104.0000000
-5.8720784	6.0023999	104.8499985
-5.8720784	6.0511999	105.8300018
-5.8720784	6.0999999	104.9700012
-5.8720784	6.1487999	104.5100021
-5.8720784	6.1975999	102.2500000
-5.8720784	6.2463999	102.6199951
-5.8720784	6.2951999	103.9399948
-5.8720784	6.3439999	103.5199966

-5.8720784	6.3927999	105.9799957
-5.8720784	6.4415998	105.9599991
-5.8720784	6.4903998	105.2799988
-5.8720784	6.5391998	102.8300018
-5.8720784	6.5879998	101.7799988
-5.8720784	6.6367998	102.1899948
-5.8720784	6.6855998	103.2600021
-5.8720784	6.7343998	105.8799973
-5.8720784	6.7831998	106.1999969
-5.8720784	6.8319998	103.8399963
-5.8720784	6.8807998	101.7200012
-5.8720784	6.9295998	103.5000000
-5.8720784	6.9783998	101.9199982
-5.8720784	7.0271997	101.4399948
-5.8720784	7.0759997	104.5000000
-5.8720784	7.1247997	103.3799973
-5.8720784	7.1735997	100.9399948
-5.8720784	7.2223997	104.9099960
-5.8720784	7.2711997	105.8600006
-5.8720784	7.3199997	102.7799988
-5.8720784	7.3687997	104.3899994
-5.8720784	7.4175997	104.8899994
-5.8720784	7.4663997	106.8699951
-5.8720784	7.5151997	105.7299957
-5.8720784	7.5639997	101.4300003
-5.8720784	7.6127996	103.4499969
-5.8720784	7.6615996	107.2999954
-5.8720784	7.7103996	107.1599960
-5.8720784	7.7591996	103.5699997
-5.8720784	7.8079996	103.2699966
-5.8720784	7.8567996	102.3499985
-5.8720784	7.9056001	102.0999985
-5.8720784	7.9544001	103.5299988
-5.8720784	8.0032005	104.6199951
-5.8720784	8.0520000	102.5999985
-5.8720784	8.1008005	100.4499969
-5.8720784	8.1496000	102.4199982
-5.8720784	8.1984005	103.4899979
-5.8720784	8.2472000	105.8300018
-5.8720784	8.2960005	105.7999954
-5.8720784	8.3448000	107.0800018
-5.8720784	8.3936005	105.4499969
-5.8720784	8.4424000	98.9300003
-5.8720784	8.4912004	102.9700012
-5.8720784	8.5400000	101.0299988
-5.8720784	8.5888004	101.9799957
-5.8720784	8.6375999	103.1800003
-5.8720784	8.6864004	101.8399963
-5.8720784	8.7351999	101.9700012
-5.8720784	8.7840004	104.1800003
-5.8720784	8.8327999	105.6199951
-5.8720784	8.8816004	104.5800018
-5.8720784	8.9303999	105.0100021
-5.8720784	8.9792004	101.5000000
-5.8720784	9.0279999	99.6999969
-5.8720784	9.0768003	103.7799988
-5.8720784	9.1255999	106.0599976
-5.8720784	9.1744003	104.0999985

-5.8720784	9.2231998	97.7999954
-5.8720784	9.2720003	100.9099960
-5.8720784	9.3207998	103.5000000
-5.8720784	9.3696003	102.6199951
-5.8720784	9.4184008	101.9799957
-5.8720784	9.4672003	103.1399994
-5.8720784	9.5160007	101.6800003
-5.8720784	9.5648003	103.0000000
-5.8720784	9.6136007	100.0499954
-5.8720784	9.6624002	101.0199966
-5.8720784	9.7112007	100.8199997
-5.8720784	9.7600002	102.4399948
-5.8720784	9.8088007	103.5000000
-5.8720784	9.8576002	102.0400009
-5.8720784	9.9064007	103.6100006
-5.8720784	9.9552002	99.8600006
-5.8720784	10.0040007	102.6500015
-5.8720784	10.0528002	103.9000015
-5.8720784	10.1016006	103.3699951
-5.8720784	10.1504002	103.1899948
-5.8720784	10.1992006	101.4899979
-5.8720784	10.2480001	103.5800018
-5.8720784	10.2968006	104.4499969
-5.8720784	10.3456001	107.5599976
-5.8720784	10.3944006	108.1800003
-5.8720784	10.4432001	105.6699982
-5.8720784	10.4920006	105.6399994
-5.8720784	10.5408001	101.4599991
-5.8720784	10.5896006	105.5100021
-5.8720784	10.6384001	104.8399963
-5.8720784	10.6872005	102.1500015
-5.8720784	10.7360001	103.0199966
-5.8720784	10.7848005	103.1699982
-5.8720784	10.8336000	104.0400009
-5.8720784	10.8824005	104.1699982
-5.8720784	10.9312000	102.7600021
-5.8720784	10.9800005	101.7500000
-5.8720784	11.0288000	102.4799957
-5.8720784	11.0776005	103.4799957
-5.8720784	11.1264000	101.3600006
-5.8720784	11.1752005	103.7699966
-5.8720784	11.2240000	103.0000000
-5.8720784	11.2728004	103.5100021
-5.8720784	11.3216000	103.7699966
-5.8720784	11.3704004	103.2200012
-5.8720784	11.4191999	101.5100021
-5.8720784	11.4680004	103.8300018
-5.8720784	11.5167999	104.7600021
-5.8720784	11.5656004	103.6899948
-5.8720784	11.6143999	104.1500015
-5.8720784	11.6632004	104.5599976
-5.8720784	11.7119999	105.5899963
-5.8720784	11.7608004	104.9300003
-5.8720784	11.8095999	104.4700012
-5.8720784	11.8584003	102.7699966
-5.8720784	11.9071999	105.0699997
-5.8720784	11.9560003	105.7999954
-5.8720784	12.0047998	104.5899963

-5.8720784	12.0536003	104.5999985
-5.8720784	12.1023998	106.5699997
-5.8720784	12.1512003	103.8199997
-5.8720784	12.1999998	95.4199982
-5.8720784	12.2488003	103.5899963
-5.8720784	12.2975998	105.8399963
-5.8720784	12.3464003	105.5800018
-5.8720784	12.3951998	103.0000000
-5.8720784	12.4440002	101.1199951
-5.8720784	12.4927998	102.2299957
-5.8720784	12.5416002	102.4499969
-5.8720784	12.5904007	107.1399994
-5.8720784	12.6392002	106.2600021
-5.8720784	12.6880007	102.9599991
-5.8720784	12.7368002	104.9399948
-5.8720784	12.7856007	104.9099960
-5.8720784	12.8344002	104.2299957
-5.8720784	12.8832006	104.9899979
-5.8720784	12.9320002	103.3600006
-5.8720784	12.9808006	100.0800018
-5.8720784	13.0296001	104.2600021
-5.8720784	13.0784006	105.6399994
-5.8720784	13.1272001	105.1999969
-5.8720784	13.1760006	102.6100006
-5.8720784	13.2248001	103.6500015
-5.8720784	13.2736006	105.1599960
-5.8720784	13.3224001	103.7500000
-5.8720784	13.3712006	105.0400009
-5.8720784	13.4200001	103.5699997
-5.8720784	13.4688005	95.2999954
-5.8720784	13.5176001	99.0999985
-5.8720784	13.5664005	100.7799988
-5.8720784	13.6152000	99.3399963
-5.8720784	13.6640005	100.7900009
-5.8720784	13.7128000	101.6399994
-5.8720784	13.7616005	104.2799988
-5.8720784	13.8104000	103.0699997
-5.8720784	13.8592005	101.8499985
-5.8720784	13.9080000	102.1699982
-5.8720784	13.9568005	103.1999969
-5.8720784	14.0056000	101.5899963
-5.8720784	14.0544004	102.2200012
-5.8720784	14.1032000	103.7099991
-5.8720784	14.1520004	103.8799973
-5.8720784	14.2007999	104.6199951
-5.8720784	14.2496004	104.9399948
-5.8720784	14.2983999	102.0999985
-5.8720784	14.3472004	101.9899979
-5.8720784	14.3959999	101.8199997
-5.8720784	14.4448004	103.4000015
-5.8720784	14.4935999	105.3300018
-5.8720784	14.5424004	105.4499969
-5.8720784	14.5911999	104.7699966
-5.8720784	14.6400003	101.8499985
-5.8720784	14.6887999	101.4199982
-5.8720784	14.7376003	102.5199966
-5.8720784	14.7863998	101.5699997
-5.8720784	14.8352003	104.1899948

-5.8720784	14.8839998	103.1199951
-5.8720784	14.9328003	101.2799988
-5.8720784	14.9815998	99.1299973
-5.8720784	15.0304003	100.2799988
-5.8659425	0.3416000	105.3199997
-5.8659425	0.3904000	105.3300018
-5.8659425	0.4392000	106.0000000
-5.8659425	0.4880000	105.0000000
-5.8659425	0.5368000	103.5699997
-5.8659425	0.5856000	105.7799988
-5.8659425	0.6344000	105.1999969
-5.8659425	0.6832000	104.1599960
-5.8659425	0.7320000	106.3699951
-5.8659425	0.7808000	104.4099960
-5.8659425	0.8296000	107.7999954
-5.8659425	0.8784000	109.0000000
-5.8659425	0.9272000	107.1599960
-5.8659425	0.9760000	106.1199951
-5.8659425	1.0247999	105.4000015
-5.8659425	1.0735999	103.6899948
-5.8659425	1.1223999	104.9000015
-5.8659425	1.1711999	100.8399963
-5.8659425	1.2200000	101.6899948
-5.8659425	1.2688000	103.5800018
-5.8659425	1.3176000	104.6800003
-5.8659425	1.3664000	106.4799957
-5.8659425	1.4152000	106.3399963
-5.8659425	1.4640000	104.9700012
-5.8659425	1.5128000	106.3899994
-5.8659425	1.5616000	103.5000000
-5.8659425	1.6104000	106.3499985
-5.8659425	1.6592000	109.3899994
-5.8659425	1.7079999	107.9199982
-5.8659425	1.7567999	103.8300018
-5.8659425	1.8055999	105.6699982
-5.8659425	1.8543999	114.0100021
-5.8659425	1.9031999	116.9399948
-5.8659425	1.9519999	113.0199966
-5.8659425	2.0007999	108.9000015
-5.8659425	2.0495999	107.2999954
-5.8659425	2.0983999	104.2999954
-5.8659425	2.1471999	105.2200012
-5.8659425	2.1959999	101.3699951
-5.8659425	2.2447999	103.2999954
-5.8659425	2.2935998	103.6800003
-5.8659425	2.3423998	106.4899979
-5.8659425	2.3912001	108.9399948
-5.8659425	2.4400001	110.1100006
-5.8659425	2.4888000	108.0899963
-5.8659425	2.5376000	107.6199951
-5.8659425	2.5864000	108.6299973
-5.8659425	2.6352000	108.0400009
-5.8659425	2.6840000	105.1399994
-5.8659425	2.7328000	105.6599960
-5.8659425	2.7816000	100.9300003
-5.8659425	2.8304000	102.4599991
-5.8659425	2.8792000	105.4799957
-5.8659425	2.9280000	107.5599976

-5.8659425	2.9768000	107.8600006
-5.8659425	3.0256000	103.9300003
-5.8659425	3.0743999	98.7799988
-5.8659425	3.1231999	104.0199966
-5.8659425	3.1719999	105.8799973
-5.8659425	3.2207999	104.0599976
-5.8659425	3.2695999	103.0100021
-5.8659425	3.3183999	104.8799973
-5.8659425	3.3671999	105.6399994
-5.8659425	3.4159999	104.5800018
-5.8659425	3.4647999	103.1199951
-5.8659425	3.5135999	101.7999954
-5.8659425	3.5623999	104.0599976
-5.8659425	3.6111999	105.2099991
-5.8659425	3.6599998	101.8300018
-5.8659425	3.7087998	102.0599976
-5.8659425	3.7575998	103.7900009
-5.8659425	3.8063998	99.8199997
-5.8659425	3.8551998	100.7200012
-5.8659425	3.9039998	102.4099960
-5.8659425	3.9527998	106.2999954
-5.8659425	4.0016003	107.3199997
-5.8659425	4.0503998	106.1800003
-5.8659425	4.0991998	107.3300018
-5.8659425	4.1479998	107.1899948
-5.8659425	4.1967998	104.8499985
-5.8659425	4.2455997	105.9000015
-5.8659425	4.2943997	109.2399979
-5.8659425	4.3431997	111.0199966
-5.8659425	4.3919997	106.9000015
-5.8659425	4.4407997	106.3199997
-5.8659425	4.4895997	107.2900009
-5.8659425	4.5383997	109.0699997
-5.8659425	4.5871997	111.4099960
-5.8659425	4.6359997	112.1500015
-5.8659425	4.6847997	115.9599991
-5.8659425	4.7336001	114.7399979
-5.8659425	4.7824001	106.6599960
-5.8659425	4.8312001	107.6899948
-5.8659425	4.8800001	110.3799973
-5.8659425	4.9288001	108.9399948
-5.8659425	4.9776001	102.9099960
-5.8659425	5.0264001	103.0400009
-5.8659425	5.0752001	104.2900009
-5.8659425	5.1240001	105.4599991
-5.8659425	5.1728001	106.9499969
-5.8659425	5.2216001	103.6800003
-5.8659425	5.2704000	104.2999954
-5.8659425	5.3192000	101.2699966
-5.8659425	5.3680000	103.7399979
-5.8659425	5.4168000	105.2799988
-5.8659425	5.4656000	103.0199966
-5.8659425	5.5144000	105.7299957
-5.8659425	5.5632000	105.0599976
-5.8659425	5.6120000	104.3899994
-5.8659425	5.6608000	102.4399948
-5.8659425	5.7096000	105.2500000
-5.8659425	5.7584000	106.4899979

-5.8659425	5.8072000	104.2200012
-5.8659425	5.8559999	99.9599991
-5.8659425	5.9047999	101.7799988
-5.8659425	5.9535999	104.5599976
-5.8659425	6.0023999	106.1299973
-5.8659425	6.0511999	105.7900009
-5.8659425	6.0999999	105.2399979
-5.8659425	6.1487999	104.6100006
-5.8659425	6.1975999	101.9799957
-5.8659425	6.2463999	102.1699982
-5.8659425	6.2951999	102.6199951
-5.8659425	6.3439999	101.6399994
-5.8659425	6.3927999	105.7799988
-5.8659425	6.4415998	106.1699982
-5.8659425	6.4903998	105.0800018
-5.8659425	6.5391998	102.9700012
-5.8659425	6.5879998	102.3899994
-5.8659425	6.6367998	102.3199997
-5.8659425	6.6855998	102.4300003
-5.8659425	6.7343998	105.0400009
-5.8659425	6.7831998	105.8099976
-5.8659425	6.8319998	104.1800003
-5.8659425	6.8807998	101.4700012
-5.8659425	6.9295998	103.6899948
-5.8659425	6.9783998	103.2799988
-5.8659425	7.0271997	101.9000015
-5.8659425	7.0759997	104.0199966
-5.8659425	7.1247997	103.0299988
-5.8659425	7.1735997	100.8300018
-5.8659425	7.2223997	105.2600021
-5.8659425	7.2711997	106.3199997
-5.8659425	7.3199997	103.5999985
-5.8659425	7.3687997	103.7999954
-5.8659425	7.4175997	105.0199966
-5.8659425	7.4663997	107.2299957
-5.8659425	7.5151997	106.2999954
-5.8659425	7.5639997	102.5699997
-5.8659425	7.6127996	104.6599960
-5.8659425	7.6615996	108.0400009
-5.8659425	7.7103996	107.1199951
-5.8659425	7.7591996	103.8699951
-5.8659425	7.8079996	102.9000015
-5.8659425	7.8567996	101.9899979
-5.8659425	7.9056001	100.5699997
-5.8659425	7.9544001	104.1999969
-5.8659425	8.0032005	105.1999969
-5.8659425	8.0520000	103.4099960
-5.8659425	8.1008005	100.9399948
-5.8659425	8.1496000	102.8099976
-5.8659425	8.1984005	103.4899979
-5.8659425	8.2472000	106.5599976
-5.8659425	8.2960005	106.6999969
-5.8659425	8.3448000	106.9599991
-5.8659425	8.3936005	105.5800018
-5.8659425	8.4424000	100.6699982
-5.8659425	8.4912004	102.2799988
-5.8659425	8.5400000	98.9199982
-5.8659425	8.5888004	102.1399994

-5.8659425	8.6375999	102.9799957
-5.8659425	8.6864004	101.6599960
-5.8659425	8.7351999	101.4899979
-5.8659425	8.7840004	103.6999969
-5.8659425	8.8327999	104.9700012
-5.8659425	8.8816004	104.2600021
-5.8659425	8.9303999	103.9099960
-5.8659425	8.9792004	100.5499954
-5.8659425	9.0279999	99.2299957
-5.8659425	9.0768003	103.2399979
-5.8659425	9.1255999	105.9199982
-5.8659425	9.1744003	104.2099991
-5.8659425	9.2231998	98.6599960
-5.8659425	9.2720003	101.0899963
-5.8659425	9.3207998	103.2900009
-5.8659425	9.3696003	103.0199966
-5.8659425	9.4184008	102.1999969
-5.8659425	9.4672003	103.2299957
-5.8659425	9.5160007	100.6699982
-5.8659425	9.5648003	102.3499985
-5.8659425	9.6136007	99.3099976
-5.8659425	9.6624002	101.1199951
-5.8659425	9.7112007	101.1399994
-5.8659425	9.7600002	103.0199966
-5.8659425	9.8088007	103.5800018
-5.8659425	9.8576002	102.9700012
-5.8659425	9.9064007	103.8300018
-5.8659425	9.9552002	99.2799988
-5.8659425	10.0040007	103.1999969
-5.8659425	10.0528002	104.5599976
-5.8659425	10.1016006	103.5199966
-5.8659425	10.1504002	104.0299988
-5.8659425	10.1992006	102.9000015
-5.8659425	10.2480001	105.0299988
-5.8659425	10.2968006	105.3199997
-5.8659425	10.3456001	106.9099960
-5.8659425	10.3944006	107.6100006
-5.8659425	10.4432001	105.0599976
-5.8659425	10.4920006	105.3499985
-5.8659425	10.5408001	100.7999954
-5.8659425	10.5896006	104.8300018
-5.8659425	10.6384001	104.5400009
-5.8659425	10.6872005	102.4199982
-5.8659425	10.7360001	102.8799973
-5.8659425	10.7848005	103.8899994
-5.8659425	10.8336000	104.9499969
-5.8659425	10.8824005	105.1599960
-5.8659425	10.9312000	103.3199997
-5.8659425	10.9800005	102.3899994
-5.8659425	11.0288000	103.2600021
-5.8659425	11.0776005	103.4000015
-5.8659425	11.1264000	99.9599991
-5.8659425	11.1752005	103.1199951
-5.8659425	11.2240000	103.0699997
-5.8659425	11.2728004	104.0800018
-5.8659425	11.3216000	104.4399948
-5.8659425	11.3704004	103.1899948
-5.8659425	11.4191999	101.5299988

-5.8659425	11.4680004	102.9599991
-5.8659425	11.5167999	104.1599960
-5.8659425	11.5656004	103.9199982
-5.8659425	11.6143999	104.1599960
-5.8659425	11.6632004	104.0000000
-5.8659425	11.7119999	105.1100006
-5.8659425	11.7608004	104.3399963
-5.8659425	11.8095999	104.0400009
-5.8659425	11.8584003	102.0699997
-5.8659425	11.9071999	105.1699982
-5.8659425	11.9560003	106.3899994
-5.8659425	12.0047998	104.2099991
-5.8659425	12.0536003	104.4399948
-5.8659425	12.1023998	107.3600006
-5.8659425	12.1512003	104.4499969
-5.8659425	12.1999998	97.1500015
-5.8659425	12.2488003	103.9099960
-5.8659425	12.2975998	105.9799957
-5.8659425	12.3464003	105.5800018
-5.8659425	12.3951998	102.1899948
-5.8659425	12.4440002	100.5999985
-5.8659425	12.4927998	102.0999985
-5.8659425	12.5416002	103.0100021
-5.8659425	12.5904007	107.5199966
-5.8659425	12.6392002	106.6100006
-5.8659425	12.6880007	103.3600006
-5.8659425	12.7368002	104.9499969
-5.8659425	12.7856007	104.4499969
-5.8659425	12.8344002	103.4799957
-5.8659425	12.8832006	104.2799988
-5.8659425	12.9320002	103.3199997
-5.8659425	12.9808006	100.0699997
-5.8659425	13.0296001	104.8099976
-5.8659425	13.0784006	105.5299988
-5.8659425	13.1272001	104.6399994
-5.8659425	13.1760006	101.9399948
-5.8659425	13.2248001	103.7600021
-5.8659425	13.2736006	105.2699966
-5.8659425	13.3224001	104.1299973
-5.8659425	13.3712006	104.6800003
-5.8659425	13.4200001	103.1599960
-5.8659425	13.4688005	92.8899994
-5.8659425	13.5176001	98.9599991
-5.8659425	13.5664005	102.0199966
-5.8659425	13.6152000	99.2200012
-5.8659425	13.6640005	100.5599976
-5.8659425	13.7128000	101.6999969
-5.8659425	13.7616005	104.2999954
-5.8659425	13.8104000	102.7999954
-5.8659425	13.8592005	102.6999969
-5.8659425	13.9080000	102.1500015
-5.8659425	13.9568005	102.9000015
-5.8659425	14.0056000	101.4199982
-5.8659425	14.0544004	102.8600006
-5.8659425	14.1032000	104.4499969
-5.8659425	14.1520004	104.6100006
-5.8659425	14.2007999	104.2699966
-5.8659425	14.2496004	103.8199997

-5.8659425	14.2983999	102.7500000
-5.8659425	14.3472004	102.1100006
-5.8659425	14.3959999	102.6599960
-5.8659425	14.4448004	104.2500000
-5.8659425	14.4935999	105.1500015
-5.8659425	14.5424004	105.7799988
-5.8659425	14.5911999	104.8300018
-5.8659425	14.6400003	101.3499985
-5.8659425	14.6887999	101.5800018
-5.8659425	14.7376003	102.5400009
-5.8659425	14.7863998	101.2299957
-5.8659425	14.8352003	104.4399948
-5.8659425	14.8839998	103.0199966
-5.8659425	14.9328003	102.0599976
-5.8659425	14.9815998	100.6699982
-5.8659425	15.0304003	100.9899979
-5.8598070	0.3416000	105.7399979
-5.8598070	0.3904000	106.4099960
-5.8598070	0.4392000	106.1100006
-5.8598070	0.4880000	106.0800018
-5.8598070	0.5368000	103.5800018
-5.8598070	0.5856000	105.5000000
-5.8598070	0.6344000	105.7999954
-5.8598070	0.6832000	105.3499985
-5.8598070	0.7320000	107.3899994
-5.8598070	0.7808000	105.6100006
-5.8598070	0.8296000	108.1699982
-5.8598070	0.8784000	108.6800003
-5.8598070	0.9272000	106.8300018
-5.8598070	0.9760000	106.4799957
-5.8598070	1.0247999	105.9899979
-5.8598070	1.0735999	105.1599960
-5.8598070	1.1223999	105.6899948
-5.8598070	1.1711999	101.5699997
-5.8598070	1.2200000	101.7799988
-5.8598070	1.2688000	102.5400009
-5.8598070	1.3176000	103.4000015
-5.8598070	1.3664000	105.3099976
-5.8598070	1.4152000	105.4399948
-5.8598070	1.4640000	105.4899979
-5.8598070	1.5128000	106.7299957
-5.8598070	1.5616000	104.2600021
-5.8598070	1.6104000	107.2399979
-5.8598070	1.6592000	108.9300003
-5.8598070	1.7079999	107.1299973
-5.8598070	1.7567999	104.1699982
-5.8598070	1.8055999	105.7099991
-5.8598070	1.8543999	113.5199966
-5.8598070	1.9031999	116.9300003
-5.8598070	1.9519999	112.7299957
-5.8598070	2.0007999	107.9700012
-5.8598070	2.0495999	106.2600021
-5.8598070	2.0983999	104.9499969
-5.8598070	2.1471999	104.4599991
-5.8598070	2.1959999	100.0000000
-5.8598070	2.2447999	102.0100021
-5.8598070	2.2935998	102.7299957
-5.8598070	2.3423998	106.6599960

-5.8598070	2.3912001	109.1999969
-5.8598070	2.4400001	110.2399979
-5.8598070	2.4888000	108.3300018
-5.8598070	2.5376000	107.9300003
-5.8598070	2.5864000	108.5599976
-5.8598070	2.6352000	107.8199997
-5.8598070	2.6840000	104.0899963
-5.8598070	2.7328000	104.7500000
-5.8598070	2.7816000	100.9000015
-5.8598070	2.8304000	103.8499985
-5.8598070	2.8792000	106.2299957
-5.8598070	2.9280000	108.1500015
-5.8598070	2.9768000	107.8799973
-5.8598070	3.0256000	103.7399979
-5.8598070	3.0743999	99.2999954
-5.8598070	3.1231999	103.0299988
-5.8598070	3.1719999	104.3099976
-5.8598070	3.2207999	102.7799988
-5.8598070	3.2695999	102.6299973
-5.8598070	3.3183999	104.8399963
-5.8598070	3.3671999	105.5800018
-5.8598070	3.4159999	104.7699966
-5.8598070	3.4647999	103.7200012
-5.8598070	3.5135999	101.6899948
-5.8598070	3.5623999	103.9300003
-5.8598070	3.6111999	105.3699951
-5.8598070	3.6599998	101.5899963
-5.8598070	3.7087998	101.1500015
-5.8598070	3.7575998	103.9300003
-5.8598070	3.8063998	100.7900009
-5.8598070	3.8551998	100.6699982
-5.8598070	3.9039998	101.7399979
-5.8598070	3.9527998	106.3099976
-5.8598070	4.0016003	107.7099991
-5.8598070	4.0503998	106.7799988
-5.8598070	4.0991998	107.5100021
-5.8598070	4.1479998	107.7699966
-5.8598070	4.1967998	105.6999969
-5.8598070	4.2455997	105.2900009
-5.8598070	4.2943997	108.9799957
-5.8598070	4.3431997	110.8300018
-5.8598070	4.3919997	106.7500000
-5.8598070	4.4407997	106.6800003
-5.8598070	4.4895997	108.4099960
-5.8598070	4.5383997	110.0100021
-5.8598070	4.5871997	111.6299973
-5.8598070	4.6359997	112.8899994
-5.8598070	4.6847997	116.6100006
-5.8598070	4.7336001	115.3300018
-5.8598070	4.7824001	108.1399994
-5.8598070	4.8312001	109.0199966
-5.8598070	4.8800001	111.0199966
-5.8598070	4.9288001	108.5199966
-5.8598070	4.9776001	103.0499954
-5.8598070	5.0264001	102.5100021
-5.8598070	5.0752001	103.8099976
-5.8598070	5.1240001	106.2200012
-5.8598070	5.1728001	106.5000000

-5.8598070	5.2216001	102.8399963
-5.8598070	5.2704000	104.0199966
-5.8598070	5.3192000	102.6699982
-5.8598070	5.3680000	104.9499969
-5.8598070	5.4168000	105.9300003
-5.8598070	5.4656000	102.9499969
-5.8598070	5.5144000	105.4300003
-5.8598070	5.5632000	104.0899963
-5.8598070	5.6120000	104.2699966
-5.8598070	5.6608000	102.1299973
-5.8598070	5.7096000	104.4199982
-5.8598070	5.7584000	105.4899979
-5.8598070	5.8072000	104.2900009
-5.8598070	5.8559999	100.0199966
-5.8598070	5.9047999	101.4499969
-5.8598070	5.9535999	104.7699966
-5.8598070	6.0023999	106.8099976
-5.8598070	6.0511999	105.0299988
-5.8598070	6.0999999	104.3600006
-5.8598070	6.1487999	103.9000015
-5.8598070	6.1975999	101.6800003
-5.8598070	6.2463999	102.5100021
-5.8598070	6.2951999	102.0499954
-5.8598070	6.3439999	100.2799988
-5.8598070	6.3927999	105.6699982
-5.8598070	6.4415998	106.2099991
-5.8598070	6.4903998	104.7799988
-5.8598070	6.5391998	102.8399963
-5.8598070	6.5879998	102.9099960
-5.8598070	6.6367998	103.0999985
-5.8598070	6.6855998	102.9599991
-5.8598070	6.7343998	104.6599960
-5.8598070	6.7831998	105.8399963
-5.8598070	6.8319998	104.7099991
-5.8598070	6.8807998	101.4899979
-5.8598070	6.9295998	104.0999985
-5.8598070	6.9783998	104.4300003
-5.8598070	7.0271997	103.1199951
-5.8598070	7.0759997	103.4099960
-5.8598070	7.1247997	102.5199966
-5.8598070	7.1735997	100.5899963
-5.8598070	7.2223997	104.9499969
-5.8598070	7.2711997	106.2099991
-5.8598070	7.3199997	104.2299957
-5.8598070	7.3687997	102.7299957
-5.8598070	7.4175997	104.7099991
-5.8598070	7.4663997	107.2999954
-5.8598070	7.5151997	106.4300003
-5.8598070	7.5639997	103.2099991
-5.8598070	7.6127996	105.1199951
-5.8598070	7.6615996	108.1199951
-5.8598070	7.7103996	106.8899994
-5.8598070	7.7591996	104.2600021
-5.8598070	7.8079996	103.6699982
-5.8598070	7.8567996	102.0100021
-5.8598070	7.9056001	100.5100021
-5.8598070	7.9544001	104.7799988
-5.8598070	8.0032005	105.2999954

-5.8598070	8.0520000	104.2399979
-5.8598070	8.1008005	101.1199951
-5.8598070	8.1496000	102.6199951
-5.8598070	8.1984005	102.9300003
-5.8598070	8.2472000	106.5000000
-5.8598070	8.2960005	106.7500000
-5.8598070	8.3448000	106.2399979
-5.8598070	8.3936005	105.2299957
-5.8598070	8.4424000	102.4799957
-5.8598070	8.4912004	102.6699982
-5.8598070	8.5400000	98.6599960
-5.8598070	8.5888004	103.0999985
-5.8598070	8.6375999	102.8399963
-5.8598070	8.6864004	101.0800018
-5.8598070	8.7351999	101.0400009
-5.8598070	8.7840004	103.2699966
-5.8598070	8.8327999	103.9700012
-5.8598070	8.8816004	103.7900009
-5.8598070	8.9303999	102.9899979
-5.8598070	8.9792004	99.5800018
-5.8598070	9.0279999	98.1199951
-5.8598070	9.0768003	102.1500015
-5.8598070	9.1255999	105.6999969
-5.8598070	9.1744003	104.7399979
-5.8598070	9.2231998	100.4899979
-5.8598070	9.2720003	102.3899994
-5.8598070	9.3207998	103.8799973
-5.8598070	9.3696003	103.4000015
-5.8598070	9.4184008	101.8799973
-5.8598070	9.4672003	103.2799988
-5.8598070	9.5160007	99.9000015
-5.8598070	9.5648003	101.6999969
-5.8598070	9.6136007	98.2399979
-5.8598070	9.6624002	100.9499969
-5.8598070	9.7112007	102.3499985
-5.8598070	9.7600002	104.0800018
-5.8598070	9.8088007	103.6699982
-5.8598070	9.8576002	103.2399979
-5.8598070	9.9064007	103.4000015
-5.8598070	9.9552002	99.9399948
-5.8598070	10.0040007	103.2999954
-5.8598070	10.0528002	104.2900009
-5.8598070	10.1016006	103.6399994
-5.8598070	10.1504002	104.6299973
-5.8598070	10.1992006	103.6199951
-5.8598070	10.2480001	106.0000000
-5.8598070	10.2968006	106.0100021
-5.8598070	10.3456001	106.0499954
-5.8598070	10.3944006	106.8099976
-5.8598070	10.4432001	104.7200012
-5.8598070	10.4920006	105.0400009
-5.8598070	10.5408001	101.6599960
-5.8598070	10.5896006	104.3199997
-5.8598070	10.6384001	104.6399994
-5.8598070	10.6872005	102.5499954
-5.8598070	10.7360001	102.7399979
-5.8598070	10.7848005	104.9000015
-5.8598070	10.8336000	105.8099976

-5.8598070	10.8824005	105.1299973
-5.8598070	10.9312000	102.9499969
-5.8598070	10.9800005	102.7299957
-5.8598070	11.0288000	103.8600006
-5.8598070	11.0776005	103.3699951
-5.8598070	11.1264000	98.5999985
-5.8598070	11.1752005	102.7299957
-5.8598070	11.2240000	103.5000000
-5.8598070	11.2728004	104.9300003
-5.8598070	11.3216000	105.1100006
-5.8598070	11.3704004	103.4599991
-5.8598070	11.4191999	102.4199982
-5.8598070	11.4680004	102.3799973
-5.8598070	11.5167999	103.9399948
-5.8598070	11.5656004	103.8799973
-5.8598070	11.6143999	103.7299957
-5.8598070	11.6632004	102.8699951
-5.8598070	11.7119999	104.0899963
-5.8598070	11.7608004	103.5400009
-5.8598070	11.8095999	103.2500000
-5.8598070	11.8584003	100.7799988
-5.8598070	11.9071999	105.0000000
-5.8598070	11.9560003	106.9899979
-5.8598070	12.0047998	104.0299988
-5.8598070	12.0536003	103.7099991
-5.8598070	12.1023998	107.5299988
-5.8598070	12.1512003	104.6399994
-5.8598070	12.1999998	98.1299973
-5.8598070	12.2488003	103.5800018
-5.8598070	12.2975998	106.0999985
-5.8598070	12.3464003	105.9599991
-5.8598070	12.3951998	102.1599960
-5.8598070	12.4440002	100.6399994
-5.8598070	12.4927998	102.6800003
-5.8598070	12.5416002	103.4499969
-5.8598070	12.5904007	107.3399963
-5.8598070	12.6392002	106.6999969
-5.8598070	12.6880007	103.9499969
-5.8598070	12.7368002	105.0599976
-5.8598070	12.7856007	104.1999969
-5.8598070	12.8344002	103.1299973
-5.8598070	12.8832006	103.5999985
-5.8598070	12.9320002	103.0800018
-5.8598070	12.9808006	100.7600021
-5.8598070	13.0296001	105.0999985
-5.8598070	13.0784006	105.1699982
-5.8598070	13.1272001	103.7900009
-5.8598070	13.1760006	101.0599976
-5.8598070	13.2248001	103.7500000
-5.8598070	13.2736006	105.2500000
-5.8598070	13.3224001	104.4199982
-5.8598070	13.3712006	104.3199997
-5.8598070	13.4200001	103.0899963
-5.8598070	13.4688005	92.1899948
-5.8598070	13.5176001	99.7500000
-5.8598070	13.5664005	102.8600006
-5.8598070	13.6152000	100.4399948
-5.8598070	13.6640005	100.5199966

-5.8598070	13.7128000	101.2999954
-5.8598070	13.7616005	104.2299957
-5.8598070	13.8104000	101.9399948
-5.8598070	13.8592005	102.9799957
-5.8598070	13.9080000	101.3600006
-5.8598070	13.9568005	101.9000015
-5.8598070	14.0056000	101.8099976
-5.8598070	14.0544004	103.7699966
-5.8598070	14.1032000	104.4199982
-5.8598070	14.1520004	104.9799957
-5.8598070	14.2007999	104.5000000
-5.8598070	14.2496004	103.1699982
-5.8598070	14.2983999	103.5400009
-5.8598070	14.3472004	103.0800018
-5.8598070	14.3959999	103.6199951
-5.8598070	14.4448004	104.3499985
-5.8598070	14.4935999	104.4000015
-5.8598070	14.5424004	105.7200012
-5.8598070	14.5911999	105.0699997
-5.8598070	14.6400003	101.0100021
-5.8598070	14.6887999	101.1899948
-5.8598070	14.7376003	102.3499985
-5.8598070	14.7863998	101.0400009
-5.8598070	14.8352003	104.1199951
-5.8598070	14.8839998	102.5599976
-5.8598070	14.9328003	102.4700012
-5.8598070	14.9815998	101.8300018
-5.8598070	15.0304003	101.3099976
-5.8536711	0.3416000	106.0000000
-5.8536711	0.3904000	106.8699951
-5.8536711	0.4392000	105.7200012
-5.8536711	0.4880000	106.6599960
-5.8536711	0.5368000	103.1899948
-5.8536711	0.5856000	104.9399948
-5.8536711	0.6344000	106.3699951
-5.8536711	0.6832000	106.6199951
-5.8536711	0.7320000	108.2200012
-5.8536711	0.7808000	106.4799957
-5.8536711	0.8296000	108.1399994
-5.8536711	0.8784000	107.6100006
-5.8536711	0.9272000	105.7600021
-5.8536711	0.9760000	106.2900009
-5.8536711	1.0247999	106.2399979
-5.8536711	1.0735999	106.1999969
-5.8536711	1.1223999	106.1299973
-5.8536711	1.1711999	102.8799973
-5.8536711	1.2200000	102.6699982
-5.8536711	1.2688000	102.7399979
-5.8536711	1.3176000	102.4499969
-5.8536711	1.3664000	104.0400009
-5.8536711	1.4152000	104.3499985
-5.8536711	1.4640000	105.5699997
-5.8536711	1.5128000	106.8499985
-5.8536711	1.5616000	105.3699951
-5.8536711	1.6104000	107.5999985
-5.8536711	1.6592000	107.8199997
-5.8536711	1.7079999	106.0400009
-5.8536711	1.7567999	104.5999985

-5.8536711	1.8055999	105.6199951
-5.8536711	1.8543999	112.9899979
-5.8536711	1.9031999	116.6699982
-5.8536711	1.9519999	112.0000000
-5.8536711	2.0007999	106.7099991
-5.8536711	2.0495999	104.3099976
-5.8536711	2.0983999	104.4199982
-5.8536711	2.1471999	103.6199951
-5.8536711	2.1959999	100.0800018
-5.8536711	2.2447999	101.7299957
-5.8536711	2.2935998	102.5999985
-5.8536711	2.3423998	107.2099991
-5.8536711	2.3912001	109.6699982
-5.8536711	2.4400001	110.3699951
-5.8536711	2.4888000	108.1500015
-5.8536711	2.5376000	107.1199951
-5.8536711	2.5864000	107.9899979
-5.8536711	2.6352000	107.7099991
-5.8536711	2.6840000	103.9399948
-5.8536711	2.7328000	104.7999954
-5.8536711	2.7816000	100.8199997
-5.8536711	2.8304000	104.5899963
-5.8536711	2.8792000	106.5999985
-5.8536711	2.9280000	108.4099960
-5.8536711	2.9768000	107.4499969
-5.8536711	3.0256000	103.9599991
-5.8536711	3.0743999	100.9899979
-5.8536711	3.1231999	101.9399948
-5.8536711	3.1719999	103.0299988
-5.8536711	3.2207999	102.3600006
-5.8536711	3.2695999	102.9300003
-5.8536711	3.3183999	104.9799957
-5.8536711	3.3671999	104.8199997
-5.8536711	3.4159999	104.3799973
-5.8536711	3.4647999	103.3099976
-5.8536711	3.5135999	101.1399994
-5.8536711	3.5623999	103.7299957
-5.8536711	3.6111999	105.4199982
-5.8536711	3.6599998	101.9499969
-5.8536711	3.7087998	101.2399979
-5.8536711	3.7575998	104.3600006
-5.8536711	3.8063998	101.8499985
-5.8536711	3.8551998	100.7200012
-5.8536711	3.9039998	101.3099976
-5.8536711	3.9527998	106.2200012
-5.8536711	4.0016003	107.9099960
-5.8536711	4.0503998	107.3499985
-5.8536711	4.0991998	107.4399948
-5.8536711	4.1479998	108.1699982
-5.8536711	4.1967998	106.3799973
-5.8536711	4.2455997	104.9399948
-5.8536711	4.2943997	108.0000000
-5.8536711	4.3431997	110.1399994
-5.8536711	4.3919997	106.9199982
-5.8536711	4.4407997	107.2099991
-5.8536711	4.4895997	109.5599976
-5.8536711	4.5383997	110.8600006
-5.8536711	4.5871997	112.1500015

-5.8536711	4.6359997	113.7200012
-5.8536711	4.6847997	116.9799957
-5.8536711	4.7336001	115.8699951
-5.8536711	4.7824001	109.5499954
-5.8536711	4.8312001	109.7500000
-5.8536711	4.8800001	111.4499969
-5.8536711	4.9288001	107.9799957
-5.8536711	4.9776001	103.2399979
-5.8536711	5.0264001	102.4700012
-5.8536711	5.0752001	103.5299988
-5.8536711	5.1240001	107.1299973
-5.8536711	5.1728001	106.1599960
-5.8536711	5.2216001	103.2900009
-5.8536711	5.2704000	103.5299988
-5.8536711	5.3192000	103.4700012
-5.8536711	5.3680000	104.9000015
-5.8536711	5.4168000	105.8699951
-5.8536711	5.4656000	103.5899963
-5.8536711	5.5144000	105.5800018
-5.8536711	5.5632000	103.2200012
-5.8536711	5.6120000	104.1599960
-5.8536711	5.6608000	102.4099960
-5.8536711	5.7096000	103.6899948
-5.8536711	5.7584000	105.2799988
-5.8536711	5.8072000	104.7099991
-5.8536711	5.8559999	100.7500000
-5.8536711	5.9047999	101.8600006
-5.8536711	5.9535999	105.3899994
-5.8536711	6.0023999	106.7699966
-5.8536711	6.0511999	104.8300018
-5.8536711	6.0999999	103.6999969
-5.8536711	6.1487999	102.8499985
-5.8536711	6.1975999	101.4799957
-5.8536711	6.2463999	103.1399994
-5.8536711	6.2951999	101.9799957
-5.8536711	6.3439999	99.8399963
-5.8536711	6.3927999	105.5800018
-5.8536711	6.4415998	106.0499954
-5.8536711	6.4903998	104.6100006
-5.8536711	6.5391998	103.1899948
-5.8536711	6.5879998	102.8699951
-5.8536711	6.6367998	103.4099960
-5.8536711	6.6855998	103.4399948
-5.8536711	6.7343998	104.2600021
-5.8536711	6.7831998	105.5999985
-5.8536711	6.8319998	105.2600021
-5.8536711	6.8807998	101.9700012
-5.8536711	6.9295998	104.5000000
-5.8536711	6.9783998	105.8099976
-5.8536711	7.0271997	104.6899948
-5.8536711	7.0759997	103.8199997
-5.8536711	7.1247997	101.8600006
-5.8536711	7.1735997	100.9599991
-5.8536711	7.2223997	104.3499985
-5.8536711	7.2711997	105.2799988
-5.8536711	7.3199997	104.3499985
-5.8536711	7.3687997	101.5699997
-5.8536711	7.4175997	104.6199951

-5.8536711	7.4663997	107.4199982
-5.8536711	7.5151997	106.3699951
-5.8536711	7.5639997	102.9599991
-5.8536711	7.6127996	105.2600021
-5.8536711	7.6615996	108.1299973
-5.8536711	7.7103996	106.6299973
-5.8536711	7.7591996	103.9599991
-5.8536711	7.8079996	104.1800003
-5.8536711	7.8567996	101.8399963
-5.8536711	7.9056001	100.3199997
-5.8536711	7.9544001	105.2699966
-5.8536711	8.0032005	105.9199982
-5.8536711	8.0520000	104.9899979
-5.8536711	8.1008005	101.9399948
-5.8536711	8.1496000	102.4199982
-5.8536711	8.1984005	101.7299957
-5.8536711	8.2472000	106.1699982
-5.8536711	8.2960005	107.0400009
-5.8536711	8.3448000	106.3399963
-5.8536711	8.3936005	105.5899963
-5.8536711	8.4424000	103.9899979
-5.8536711	8.4912004	102.6699982
-5.8536711	8.5400000	98.8600006
-5.8536711	8.5888004	103.5199966
-5.8536711	8.6375999	102.6999969
-5.8536711	8.6864004	101.0599976
-5.8536711	8.7351999	101.3399963
-5.8536711	8.7840004	103.3099976
-5.8536711	8.8327999	103.7200012
-5.8536711	8.8816004	103.7900009
-5.8536711	8.9303999	102.7699966
-5.8536711	8.9792004	98.5100021
-5.8536711	9.0279999	97.1299973
-5.8536711	9.0768003	101.9099960
-5.8536711	9.1255999	105.2099991
-5.8536711	9.1744003	104.9700012
-5.8536711	9.2231998	102.1299973
-5.8536711	9.2720003	103.6500015
-5.8536711	9.3207998	104.4700012
-5.8536711	9.3696003	103.8699951
-5.8536711	9.4184008	101.6699982
-5.8536711	9.4672003	103.1599960
-5.8536711	9.5160007	99.9799957
-5.8536711	9.5648003	102.1800003
-5.8536711	9.6136007	99.9700012
-5.8536711	9.6624002	101.7799988
-5.8536711	9.7112007	103.7500000
-5.8536711	9.7600002	104.3699951
-5.8536711	9.8088007	103.4700012
-5.8536711	9.8576002	103.6699982
-5.8536711	9.9064007	103.9000015
-5.8536711	9.9552002	101.7200012
-5.8536711	10.0040007	103.9799957
-5.8536711	10.0528002	104.4099960
-5.8536711	10.1016006	103.8300018
-5.8536711	10.1504002	104.9599991
-5.8536711	10.1992006	103.9499969
-5.8536711	10.2480001	106.3899994

-5.8536711	10.2968006	106.2399979
-5.8536711	10.3456001	105.0199966
-5.8536711	10.3944006	106.0299988
-5.8536711	10.4432001	103.6399994
-5.8536711	10.4920006	103.8600006
-5.8536711	10.5408001	101.3099976
-5.8536711	10.5896006	103.8199997
-5.8536711	10.6384001	104.6299973
-5.8536711	10.6872005	103.0999985
-5.8536711	10.7360001	102.5000000
-5.8536711	10.7848005	105.3199997
-5.8536711	10.8336000	105.7399979
-5.8536711	10.8824005	104.1699982
-5.8536711	10.9312000	102.4799957
-5.8536711	10.9800005	102.0499954
-5.8536711	11.0288000	103.6599960
-5.8536711	11.0776005	103.3499985
-5.8536711	11.1264000	99.7099991
-5.8536711	11.1752005	102.5899963
-5.8536711	11.2240000	104.1899948
-5.8536711	11.2728004	105.9399948
-5.8536711	11.3216000	105.6999969
-5.8536711	11.3704004	104.0199966
-5.8536711	11.4191999	103.9300003
-5.8536711	11.4680004	102.4700012
-5.8536711	11.5167999	104.5800018
-5.8536711	11.5656004	104.7699966
-5.8536711	11.6143999	103.4799957
-5.8536711	11.6632004	101.6199951
-5.8536711	11.7119999	103.2799988
-5.8536711	11.7608004	103.6199951
-5.8536711	11.8095999	103.4399948
-5.8536711	11.8584003	100.4799957
-5.8536711	11.9071999	104.9300003
-5.8536711	11.9560003	107.2200012
-5.8536711	12.0047998	104.2600021
-5.8536711	12.0536003	102.8699951
-5.8536711	12.1023998	107.1100006
-5.8536711	12.1512003	104.4399948
-5.8536711	12.1999998	99.2900009
-5.8536711	12.2488003	103.9899979
-5.8536711	12.2975998	106.8799973
-5.8536711	12.3464003	106.3199997
-5.8536711	12.3951998	102.7900009
-5.8536711	12.4440002	101.7399979
-5.8536711	12.4927998	103.0299988
-5.8536711	12.5416002	102.7200012
-5.8536711	12.5904007	106.8099976
-5.8536711	12.6392002	106.7600021
-5.8536711	12.6880007	104.4300003
-5.8536711	12.7368002	105.1800003
-5.8536711	12.7856007	104.6999969
-5.8536711	12.8344002	103.7799988
-5.8536711	12.8832006	103.3300018
-5.8536711	12.9320002	103.0800018
-5.8536711	12.9808006	102.1800003
-5.8536711	13.0296001	105.4300003
-5.8536711	13.0784006	104.3300018

-5.8536711	13.1272001	103.0100021
-5.8536711	13.1760006	101.5499954
-5.8536711	13.2248001	104.1899948
-5.8536711	13.2736006	105.4899979
-5.8536711	13.3224001	105.1299973
-5.8536711	13.3712006	104.8199997
-5.8536711	13.4200001	103.2999954
-5.8536711	13.4688005	92.3099976
-5.8536711	13.5176001	100.5100021
-5.8536711	13.5664005	103.0999985
-5.8536711	13.6152000	101.2699966
-5.8536711	13.6640005	100.6500015
-5.8536711	13.7128000	101.4000015
-5.8536711	13.7616005	104.6999969
-5.8536711	13.8104000	101.7600021
-5.8536711	13.8592005	103.6399994
-5.8536711	13.9080000	101.1999969
-5.8536711	13.9568005	101.3899994
-5.8536711	14.0056000	102.0800018
-5.8536711	14.0544004	103.9799957
-5.8536711	14.1032000	103.3600006
-5.8536711	14.1520004	104.1699982
-5.8536711	14.2007999	103.9099960
-5.8536711	14.2496004	102.1299973
-5.8536711	14.2983999	103.3899994
-5.8536711	14.3472004	103.3199997
-5.8536711	14.3959999	104.0699997
-5.8536711	14.4448004	104.1199951
-5.8536711	14.4935999	103.3899994
-5.8536711	14.5424004	105.4799957
-5.8536711	14.5911999	105.0400009
-5.8536711	14.6400003	100.4000015
-5.8536711	14.6887999	100.1699982
-5.8536711	14.7376003	102.4099960
-5.8536711	14.7863998	101.8899994
-5.8536711	14.8352003	104.2200012
-5.8536711	14.8839998	101.8799973
-5.8536711	14.9328003	102.8600006
-5.8536711	14.9815998	103.1599960
-5.8536711	15.0304003	101.2099991
-5.8475351	0.3416000	106.4799957
-5.8475351	0.3904000	107.4199982
-5.8475351	0.4392000	105.3199997
-5.8475351	0.4880000	107.2299957
-5.8475351	0.5368000	103.6699982
-5.8475351	0.5856000	104.1999969
-5.8475351	0.6344000	106.6599960
-5.8475351	0.6832000	106.9799957
-5.8475351	0.7320000	108.8699951
-5.8475351	0.7808000	106.9599991
-5.8475351	0.8296000	108.2399979
-5.8475351	0.8784000	107.2099991
-5.8475351	0.9272000	104.9899979
-5.8475351	0.9760000	105.6399994
-5.8475351	1.0247999	106.3600006
-5.8475351	1.0735999	107.0299988
-5.8475351	1.1223999	106.2200012
-5.8475351	1.1711999	103.8799973

-5.8475351	1.2200000	104.1100006
-5.8475351	1.2688000	103.4000015
-5.8475351	1.3176000	101.3499985
-5.8475351	1.3664000	103.3600006
-5.8475351	1.4152000	103.7799988
-5.8475351	1.4640000	105.7600021
-5.8475351	1.5128000	106.8099976
-5.8475351	1.5616000	106.3099976
-5.8475351	1.6104000	108.0599976
-5.8475351	1.6592000	107.4300003
-5.8475351	1.7079999	105.9300003
-5.8475351	1.7567999	105.2699966
-5.8475351	1.8055999	105.3899994
-5.8475351	1.8543999	112.3799973
-5.8475351	1.9031999	116.2699966
-5.8475351	1.9519999	111.3199997
-5.8475351	2.0007999	105.9700012
-5.8475351	2.0495999	103.4399948
-5.8475351	2.0983999	104.4300003
-5.8475351	2.1471999	103.6399994
-5.8475351	2.1959999	100.8300018
-5.8475351	2.2447999	102.6899948
-5.8475351	2.2935998	102.4599991
-5.8475351	2.3423998	106.9099960
-5.8475351	2.3912001	109.2200012
-5.8475351	2.4400001	109.7699966
-5.8475351	2.4888000	107.5299988
-5.8475351	2.5376000	106.3099976
-5.8475351	2.5864000	107.1899948
-5.8475351	2.6352000	107.0899963
-5.8475351	2.6840000	103.0899963
-5.8475351	2.7328000	104.4300003
-5.8475351	2.7816000	100.9399948
-5.8475351	2.8304000	104.6399994
-5.8475351	2.8792000	106.0599976
-5.8475351	2.9280000	107.8899994
-5.8475351	2.9768000	106.4499969
-5.8475351	3.0256000	103.9000015
-5.8475351	3.0743999	102.2099991
-5.8475351	3.1231999	100.7399979
-5.8475351	3.1719999	102.0199966
-5.8475351	3.2207999	102.0299988
-5.8475351	3.2695999	102.1100006
-5.8475351	3.3183999	104.6500015
-5.8475351	3.3671999	103.5499954
-5.8475351	3.4159999	103.5699997
-5.8475351	3.4647999	102.2099991
-5.8475351	3.5135999	100.1999969
-5.8475351	3.5623999	103.1299973
-5.8475351	3.6111999	105.2799988
-5.8475351	3.6599998	102.6800003
-5.8475351	3.7087998	101.9899979
-5.8475351	3.7575998	104.8499985
-5.8475351	3.8063998	102.0000000
-5.8475351	3.8551998	99.8899994
-5.8475351	3.9039998	101.0199966
-5.8475351	3.9527998	106.3899994
-5.8475351	4.0016003	107.6899948

-5.8475351	4.0503998	107.6500015
-5.8475351	4.0991998	107.2399979
-5.8475351	4.1479998	108.4199982
-5.8475351	4.1967998	107.0100021
-5.8475351	4.2455997	104.5899963
-5.8475351	4.2943997	107.2500000
-5.8475351	4.3431997	108.6199951
-5.8475351	4.3919997	105.7500000
-5.8475351	4.4407997	106.9000015
-5.8475351	4.4895997	110.0199966
-5.8475351	4.5383997	112.1800003
-5.8475351	4.5871997	113.1800003
-5.8475351	4.6359997	114.1100006
-5.8475351	4.6847997	116.5100021
-5.8475351	4.7336001	115.6399994
-5.8475351	4.7824001	110.1800003
-5.8475351	4.8312001	109.7500000
-5.8475351	4.8800001	110.9499969
-5.8475351	4.9288001	106.8899994
-5.8475351	4.9776001	103.5199966
-5.8475351	5.0264001	103.1899948
-5.8475351	5.0752001	103.2799988
-5.8475351	5.1240001	107.2299957
-5.8475351	5.1728001	105.1599960
-5.8475351	5.2216001	103.5400009
-5.8475351	5.2704000	103.4899979
-5.8475351	5.3192000	104.7999954
-5.8475351	5.3680000	105.4199982
-5.8475351	5.4168000	106.5599976
-5.8475351	5.4656000	104.8499985
-5.8475351	5.5144000	105.4000015
-5.8475351	5.5632000	101.4499969
-5.8475351	5.6120000	103.9099960
-5.8475351	5.6608000	103.0199966
-5.8475351	5.7096000	102.4599991
-5.8475351	5.7584000	104.4799957
-5.8475351	5.8072000	104.3899994
-5.8475351	5.8559999	101.7500000
-5.8475351	5.9047999	102.3899994
-5.8475351	5.9535999	105.5999985
-5.8475351	6.0023999	106.4700012
-5.8475351	6.0511999	104.7900009
-5.8475351	6.0999999	103.4499969
-5.8475351	6.1487999	101.7600021
-5.8475351	6.1975999	101.2200012
-5.8475351	6.2463999	103.3799973
-5.8475351	6.2951999	101.9499969
-5.8475351	6.3439999	100.2099991
-5.8475351	6.3927999	105.6899948
-5.8475351	6.4415998	105.5100021
-5.8475351	6.4903998	104.1999969
-5.8475351	6.5391998	103.4399948
-5.8475351	6.5879998	102.3300018
-5.8475351	6.6367998	103.7399979
-5.8475351	6.6855998	104.4399948
-5.8475351	6.7343998	104.7799988
-5.8475351	6.7831998	105.9199982
-5.8475351	6.8319998	105.8499985

-5.8475351	6.8807998	101.5599976
-5.8475351	6.9295998	104.3399963
-5.8475351	6.9783998	106.5699997
-5.8475351	7.0271997	105.6199951
-5.8475351	7.0759997	104.0499954
-5.8475351	7.1247997	101.0999985
-5.8475351	7.1735997	100.9799957
-5.8475351	7.2223997	103.1500015
-5.8475351	7.2711997	103.4599991
-5.8475351	7.3199997	104.2500000
-5.8475351	7.3687997	101.2900009
-5.8475351	7.4175997	104.7200012
-5.8475351	7.4663997	107.7500000
-5.8475351	7.5151997	106.0199966
-5.8475351	7.5639997	102.9599991
-5.8475351	7.6127996	105.6399994
-5.8475351	7.6615996	108.0100021
-5.8475351	7.7103996	106.3899994
-5.8475351	7.7591996	103.5499954
-5.8475351	7.8079996	104.2999954
-5.8475351	7.8567996	101.7699966
-5.8475351	7.9056001	100.7099991
-5.8475351	7.9544001	105.4399948
-5.8475351	8.0032005	105.9199982
-5.8475351	8.0520000	105.4300003
-5.8475351	8.1008005	102.6999969
-5.8475351	8.1496000	102.2500000
-5.8475351	8.1984005	101.6299973
-5.8475351	8.2472000	105.7799988
-5.8475351	8.2960005	106.9099960
-5.8475351	8.3448000	106.3799973
-5.8475351	8.3936005	106.0800018
-5.8475351	8.4424000	104.7799988
-5.8475351	8.4912004	103.2200012
-5.8475351	8.5400000	99.2099991
-5.8475351	8.5888004	103.0199966
-5.8475351	8.6375999	102.0699997
-5.8475351	8.6864004	100.9300003
-5.8475351	8.7351999	101.1100006
-5.8475351	8.7840004	103.4700012
-5.8475351	8.8327999	103.9000015
-5.8475351	8.8816004	104.0899963
-5.8475351	8.9303999	103.3099976
-5.8475351	8.9792004	98.5599976
-5.8475351	9.0279999	97.3300018
-5.8475351	9.0768003	102.2399979
-5.8475351	9.1255999	105.0299988
-5.8475351	9.1744003	105.4499969
-5.8475351	9.2231998	103.7999954
-5.8475351	9.2720003	104.4000015
-5.8475351	9.3207998	104.9199982
-5.8475351	9.3696003	104.5100021
-5.8475351	9.4184008	101.8600006
-5.8475351	9.4672003	103.7299957
-5.8475351	9.5160007	101.9599991
-5.8475351	9.5648003	103.2699966
-5.8475351	9.6136007	101.5999985
-5.8475351	9.6624002	102.4700012

-5.8475351	9.7112007	105.0000000
-5.8475351	9.7600002	104.4799957
-5.8475351	9.8088007	104.0499954
-5.8475351	9.8576002	103.4499969
-5.8475351	9.9064007	104.1699982
-5.8475351	9.9552002	103.2399979
-5.8475351	10.0040007	104.9300003
-5.8475351	10.0528002	103.5699997
-5.8475351	10.1016006	104.2699966
-5.8475351	10.1504002	104.7999954
-5.8475351	10.1992006	103.9000015
-5.8475351	10.2480001	106.8199997
-5.8475351	10.2968006	106.8699951
-5.8475351	10.3456001	103.7299957
-5.8475351	10.3944006	104.9300003
-5.8475351	10.4432001	102.5000000
-5.8475351	10.4920006	102.9700012
-5.8475351	10.5408001	100.9799957
-5.8475351	10.5896006	103.2099991
-5.8475351	10.6384001	104.8799973
-5.8475351	10.6872005	104.0599976
-5.8475351	10.7360001	102.5100021
-5.8475351	10.7848005	105.1199951
-5.8475351	10.8336000	105.1699982
-5.8475351	10.8824005	102.8600006
-5.8475351	10.9312000	102.5999985
-5.8475351	10.9800005	102.2699966
-5.8475351	11.0288000	103.9099960
-5.8475351	11.0776005	103.8399963
-5.8475351	11.1264000	101.1100006
-5.8475351	11.1752005	103.2799988
-5.8475351	11.2240000	104.6999969
-5.8475351	11.2728004	106.5499954
-5.8475351	11.3216000	105.5699997
-5.8475351	11.3704004	104.3799973
-5.8475351	11.4191999	105.0499954
-5.8475351	11.4680004	103.0299988
-5.8475351	11.5167999	104.6800003
-5.8475351	11.5656004	105.0199966
-5.8475351	11.6143999	103.1500015
-5.8475351	11.6632004	101.4199982
-5.8475351	11.7119999	103.2999954
-5.8475351	11.7608004	103.8399963
-5.8475351	11.8095999	103.3099976
-5.8475351	11.8584003	100.2799988
-5.8475351	11.9071999	104.9799957
-5.8475351	11.9560003	107.2699966
-5.8475351	12.0047998	104.6800003
-5.8475351	12.0536003	102.4399948
-5.8475351	12.1023998	106.3300018
-5.8475351	12.1512003	103.8699951
-5.8475351	12.1999998	100.5000000
-5.8475351	12.2488003	104.2900009
-5.8475351	12.2975998	107.1899948
-5.8475351	12.3464003	106.2500000
-5.8475351	12.3951998	103.1199951
-5.8475351	12.4440002	102.6399994
-5.8475351	12.4927998	103.5699997

-5.8475351	12.5416002	102.7200012
-5.8475351	12.5904007	106.1500015
-5.8475351	12.6392002	106.1899948
-5.8475351	12.6880007	104.8499985
-5.8475351	12.7368002	105.5800018
-5.8475351	12.7856007	105.5299988
-5.8475351	12.8344002	104.5400009
-5.8475351	12.8832006	103.1199951
-5.8475351	12.9320002	102.8699951
-5.8475351	12.9808006	102.9000015
-5.8475351	13.0296001	104.7500000
-5.8475351	13.0784006	102.8300018
-5.8475351	13.1272001	102.6999969
-5.8475351	13.1760006	102.4300003
-5.8475351	13.2248001	104.2200012
-5.8475351	13.2736006	105.1100006
-5.8475351	13.3224001	104.9700012
-5.8475351	13.3712006	104.8499985
-5.8475351	13.4200001	103.6999969
-5.8475351	13.4688005	92.9799957
-5.8475351	13.5176001	101.6800003
-5.8475351	13.5664005	103.5800018
-5.8475351	13.6152000	102.1599960
-5.8475351	13.6640005	100.4799957
-5.8475351	13.7128000	101.3099976
-5.8475351	13.7616005	104.8300018
-5.8475351	13.8104000	102.2900009
-5.8475351	13.8592005	103.8199997
-5.8475351	13.9080000	100.9000015
-5.8475351	13.9568005	100.9000015
-5.8475351	14.0056000	102.1199951
-5.8475351	14.0544004	103.6599960
-5.8475351	14.1032000	102.1800003
-5.8475351	14.1520004	104.0199966
-5.8475351	14.2007999	103.3699951
-5.8475351	14.2496004	101.0499954
-5.8475351	14.2983999	103.4300003
-5.8475351	14.3472004	103.8499985
-5.8475351	14.3959999	104.5400009
-5.8475351	14.4448004	103.6699982
-5.8475351	14.4935999	102.1599960
-5.8475351	14.5424004	104.7200012
-5.8475351	14.5911999	104.9499969
-5.8475351	14.6400003	99.8699951
-5.8475351	14.6887999	99.0400009
-5.8475351	14.7376003	103.1100006
-5.8475351	14.7863998	102.9000015
-5.8475351	14.8352003	103.9599991
-5.8475351	14.8839998	100.6399994
-5.8475351	14.9328003	103.0999985
-5.8475351	14.9815998	104.0800018
-5.8475351	15.0304003	100.2299957
-5.8413992	0.3416000	106.9899979
-5.8413992	0.3904000	107.4199982
-5.8413992	0.4392000	104.1999969
-5.8413992	0.4880000	107.1699982
-5.8413992	0.5368000	104.5999985
-5.8413992	0.5856000	103.9300003

-5.8413992	0.6344000	106.2799988
-5.8413992	0.6832000	106.9899979
-5.8413992	0.7320000	108.5899963
-5.8413992	0.7808000	106.6199951
-5.8413992	0.8296000	107.9000015
-5.8413992	0.8784000	106.8199997
-5.8413992	0.9272000	104.7399979
-5.8413992	0.9760000	105.3799973
-5.8413992	1.0247999	106.5800018
-5.8413992	1.0735999	107.5299988
-5.8413992	1.1223999	106.1599960
-5.8413992	1.1711999	104.9199982
-5.8413992	1.2200000	105.2500000
-5.8413992	1.2688000	104.1299973
-5.8413992	1.3176000	101.2600021
-5.8413992	1.3664000	103.3600006
-5.8413992	1.4152000	104.5100021
-5.8413992	1.4640000	106.4499969
-5.8413992	1.5128000	106.8699951
-5.8413992	1.5616000	106.8199997
-5.8413992	1.6104000	108.2500000
-5.8413992	1.6592000	107.1100006
-5.8413992	1.7079999	105.9199982
-5.8413992	1.7567999	106.0400009
-5.8413992	1.8055999	104.6399994
-5.8413992	1.8543999	111.4099960
-5.8413992	1.9031999	115.3499985
-5.8413992	1.9519999	111.2699966
-5.8413992	2.0007999	105.8399963
-5.8413992	2.0495999	103.0899963
-5.8413992	2.0983999	104.4000015
-5.8413992	2.1471999	103.6500015
-5.8413992	2.1959999	101.6699982
-5.8413992	2.2447999	103.2399979
-5.8413992	2.2935998	101.9399948
-5.8413992	2.3423998	106.3399963
-5.8413992	2.3912001	109.0400009
-5.8413992	2.4400001	109.5000000
-5.8413992	2.4888000	107.1599960
-5.8413992	2.5376000	105.5999985
-5.8413992	2.5864000	106.4899979
-5.8413992	2.6352000	106.8699951
-5.8413992	2.6840000	103.3699951
-5.8413992	2.7328000	104.7900009
-5.8413992	2.7816000	101.2399979
-5.8413992	2.8304000	104.7500000
-5.8413992	2.8792000	106.1399994
-5.8413992	2.9280000	107.2299957
-5.8413992	2.9768000	105.2299957
-5.8413992	3.0256000	103.0199966
-5.8413992	3.0743999	102.8199997
-5.8413992	3.1231999	100.2799988
-5.8413992	3.1719999	102.1500015
-5.8413992	3.2207999	102.1999969
-5.8413992	3.2695999	101.7399979
-5.8413992	3.3183999	104.4399948
-5.8413992	3.3671999	102.5800018
-5.8413992	3.4159999	103.3499985

-5.8413992	3.4647999	102.0000000
-5.8413992	3.5135999	99.8899994
-5.8413992	3.5623999	102.2600021
-5.8413992	3.6111999	104.7299957
-5.8413992	3.6599998	103.2399979
-5.8413992	3.7087998	102.5999985
-5.8413992	3.7575998	105.0699997
-5.8413992	3.8063998	103.1399994
-5.8413992	3.8551998	100.4599991
-5.8413992	3.9039998	101.0599976
-5.8413992	3.9527998	106.6699982
-5.8413992	4.0016003	107.1399994
-5.8413992	4.0503998	107.4399948
-5.8413992	4.0991998	106.6299973
-5.8413992	4.1479998	108.9300003
-5.8413992	4.1967998	108.1899948
-5.8413992	4.2455997	105.2999954
-5.8413992	4.2943997	106.3899994
-5.8413992	4.3431997	107.2900009
-5.8413992	4.3919997	105.7699966
-5.8413992	4.4407997	106.4599991
-5.8413992	4.4895997	109.4199982
-5.8413992	4.5383997	112.9399948
-5.8413992	4.5871997	114.0699997
-5.8413992	4.6359997	114.6899948
-5.8413992	4.6847997	116.5000000
-5.8413992	4.7336001	115.8499985
-5.8413992	4.7824001	110.7600021
-5.8413992	4.8312001	109.3099976
-5.8413992	4.8800001	110.1500015
-5.8413992	4.9288001	106.2500000
-5.8413992	4.9776001	104.0800018
-5.8413992	5.0264001	104.4199982
-5.8413992	5.0752001	104.6199951
-5.8413992	5.1240001	107.1999969
-5.8413992	5.1728001	103.9700012
-5.8413992	5.2216001	103.5899963
-5.8413992	5.2704000	103.3600006
-5.8413992	5.3192000	105.2200012
-5.8413992	5.3680000	105.5199966
-5.8413992	5.4168000	106.7500000
-5.8413992	5.4656000	105.6899948
-5.8413992	5.5144000	105.6599960
-5.8413992	5.5632000	101.1899948
-5.8413992	5.6120000	103.7799988
-5.8413992	5.6608000	102.4399948
-5.8413992	5.7096000	100.8899994
-5.8413992	5.7584000	103.8600006
-5.8413992	5.8072000	105.0800018
-5.8413992	5.8559999	103.5000000
-5.8413992	5.9047999	103.5100021
-5.8413992	5.9535999	105.5299988
-5.8413992	6.0023999	106.2699966
-5.8413992	6.0511999	105.0599976
-5.8413992	6.0999999	103.4599991
-5.8413992	6.1487999	101.0000000
-5.8413992	6.1975999	100.9799957
-5.8413992	6.2463999	103.9899979

-5.8413992	6.2951999	102.8799973
-5.8413992	6.3439999	101.2299957
-5.8413992	6.3927999	106.1399994
-5.8413992	6.4415998	105.4000015
-5.8413992	6.4903998	104.2799988
-5.8413992	6.5391998	103.9899979
-5.8413992	6.5879998	102.4000015
-5.8413992	6.6367998	104.9700012
-5.8413992	6.6855998	105.8399963
-5.8413992	6.7343998	105.3199997
-5.8413992	6.7831998	105.9300003
-5.8413992	6.8319998	105.3699951
-5.8413992	6.8807998	100.7399979
-5.8413992	6.9295998	103.5800018
-5.8413992	6.9783998	106.2600021
-5.8413992	7.0271997	105.5699997
-5.8413992	7.0759997	103.6100006
-5.8413992	7.1247997	100.1800003
-5.8413992	7.1735997	101.5499954
-5.8413992	7.2223997	102.2099991
-5.8413992	7.2711997	102.0800018
-5.8413992	7.3199997	103.9799957
-5.8413992	7.3687997	101.0400009
-5.8413992	7.4175997	104.8199997
-5.8413992	7.4663997	108.0599976
-5.8413992	7.5151997	105.6399994
-5.8413992	7.5639997	103.1100006
-5.8413992	7.6127996	106.0699997
-5.8413992	7.6615996	108.0000000
-5.8413992	7.7103996	106.1199951
-5.8413992	7.7591996	102.4899979
-5.8413992	7.8079996	104.2299957
-5.8413992	7.8567996	101.7600021
-5.8413992	7.9056001	100.1299973
-5.8413992	7.9544001	104.6500015
-5.8413992	8.0032005	104.7900009
-5.8413992	8.0520000	105.3799973
-5.8413992	8.1008005	103.3600006
-5.8413992	8.1496000	101.9099960
-5.8413992	8.1984005	101.2900009
-5.8413992	8.2472000	105.3899994
-5.8413992	8.2960005	106.3600006
-5.8413992	8.3448000	105.6899948
-5.8413992	8.3936005	105.7799988
-5.8413992	8.4424000	104.4799957
-5.8413992	8.4912004	103.9000015
-5.8413992	8.5400000	100.3300018
-5.8413992	8.5888004	103.0000000
-5.8413992	8.6375999	101.4099960
-5.8413992	8.6864004	100.3600006
-5.8413992	8.7351999	100.7600021
-5.8413992	8.7840004	103.6699982
-5.8413992	8.8327999	104.5299988
-5.8413992	8.8816004	104.3600006
-5.8413992	8.9303999	104.0100021
-5.8413992	8.9792004	99.0899963
-5.8413992	9.0279999	98.2999954
-5.8413992	9.0768003	103.0699997

-5.8413992	9.1255999	104.9499969
-5.8413992	9.1744003	105.7600021
-5.8413992	9.2231998	105.4499969
-5.8413992	9.2720003	105.1800003
-5.8413992	9.3207998	104.8199997
-5.8413992	9.3696003	103.9199982
-5.8413992	9.4184008	101.0299988
-5.8413992	9.4672003	103.4700012
-5.8413992	9.5160007	103.1299973
-5.8413992	9.5648003	104.0000000
-5.8413992	9.6136007	103.9199982
-5.8413992	9.6624002	104.3199997
-5.8413992	9.7112007	106.1100006
-5.8413992	9.7600002	104.0299988
-5.8413992	9.8088007	104.0699997
-5.8413992	9.8576002	103.5199966
-5.8413992	9.9064007	104.8099976
-5.8413992	9.9552002	104.9899979
-5.8413992	10.0040007	105.2600021
-5.8413992	10.0528002	102.5499954
-5.8413992	10.1016006	104.7999954
-5.8413992	10.1504002	104.3300018
-5.8413992	10.1992006	103.3899994
-5.8413992	10.2480001	106.6399994
-5.8413992	10.2968006	106.5800018
-5.8413992	10.3456001	101.6100006
-5.8413992	10.3944006	103.8699951
-5.8413992	10.4432001	102.1699982
-5.8413992	10.4920006	102.1800003
-5.8413992	10.5408001	100.4000015
-5.8413992	10.5896006	103.6500015
-5.8413992	10.6384001	105.3699951
-5.8413992	10.6872005	105.2999954
-5.8413992	10.7360001	103.9700012
-5.8413992	10.7848005	105.6599960
-5.8413992	10.8336000	105.2299957
-5.8413992	10.8824005	101.7099991
-5.8413992	10.9312000	102.7500000
-5.8413992	10.9800005	102.8699951
-5.8413992	11.0288000	103.9799957
-5.8413992	11.0776005	104.0899963
-5.8413992	11.1264000	102.1899948
-5.8413992	11.1752005	103.5400009
-5.8413992	11.2240000	105.0599976
-5.8413992	11.2728004	106.6399994
-5.8413992	11.3216000	104.5699997
-5.8413992	11.3704004	103.6500015
-5.8413992	11.4191999	105.1899948
-5.8413992	11.4680004	103.3300018
-5.8413992	11.5167999	104.5100021
-5.8413992	11.5656004	105.0000000
-5.8413992	11.6143999	102.6699982
-5.8413992	11.6632004	100.7500000
-5.8413992	11.7119999	103.0499954
-5.8413992	11.7608004	103.9000015
-5.8413992	11.8095999	103.5699997
-5.8413992	11.8584003	101.1999969
-5.8413992	11.9071999	105.0299988

-5.8413992	11.9560003	107.2099991
-5.8413992	12.0047998	104.9199982
-5.8413992	12.0536003	102.3600006
-5.8413992	12.1023998	105.5000000
-5.8413992	12.1512003	103.4399948
-5.8413992	12.1999998	101.7799988
-5.8413992	12.2488003	104.8099976
-5.8413992	12.2975998	107.4399948
-5.8413992	12.3464003	106.3199997
-5.8413992	12.3951998	103.7699966
-5.8413992	12.4440002	103.4700012
-5.8413992	12.4927998	103.9000015
-5.8413992	12.5416002	102.3699951
-5.8413992	12.5904007	105.2200012
-5.8413992	12.6392002	105.5299988
-5.8413992	12.6880007	105.3199997
-5.8413992	12.7368002	105.7099991
-5.8413992	12.7856007	105.7500000
-5.8413992	12.8344002	105.0100021
-5.8413992	12.8832006	103.5499954
-5.8413992	12.9320002	103.1599960
-5.8413992	12.9808006	103.9399948
-5.8413992	13.0296001	104.4799957
-5.8413992	13.0784006	101.8699951
-5.8413992	13.1272001	103.0199966
-5.8413992	13.1760006	103.2299957
-5.8413992	13.2248001	103.4899979
-5.8413992	13.2736006	103.8399963
-5.8413992	13.3224001	103.6399994
-5.8413992	13.3712006	104.5100021
-5.8413992	13.4200001	103.7999954
-5.8413992	13.4688005	93.2900009
-5.8413992	13.5176001	101.9300003
-5.8413992	13.5664005	103.6399994
-5.8413992	13.6152000	103.0499954
-5.8413992	13.6640005	100.4399948
-5.8413992	13.7128000	101.4399948
-5.8413992	13.7616005	105.1299973
-5.8413992	13.8104000	102.7999954
-5.8413992	13.8592005	103.9099960
-5.8413992	13.9080000	100.8499985
-5.8413992	13.9568005	100.1899948
-5.8413992	14.0056000	101.8099976
-5.8413992	14.0544004	103.3699951
-5.8413992	14.1032000	102.2900009
-5.8413992	14.1520004	103.8899994
-5.8413992	14.2007999	103.0499954
-5.8413992	14.2496004	100.0199966
-5.8413992	14.2983999	102.8600006
-5.8413992	14.3472004	103.9899979
-5.8413992	14.3959999	104.5499954
-5.8413992	14.4448004	103.4499969
-5.8413992	14.4935999	101.2600021
-5.8413992	14.5424004	104.0499954
-5.8413992	14.5911999	104.9199982
-5.8413992	14.6400003	99.9199982
-5.8413992	14.6887999	98.8799973
-5.8413992	14.7376003	103.5599976

-5.8413992	14.7863998	103.2399979
-5.8413992	14.8352003	103.2500000
-5.8413992	14.8839998	99.2500000
-5.8413992	14.9328003	102.4499969
-5.8413992	14.9815998	103.6999969
-5.8413992	15.0304003	98.6500015
-5.8352633	0.3416000	107.0499954
-5.8352633	0.3904000	107.0000000
-5.8352633	0.4392000	104.6899948
-5.8352633	0.4880000	107.2399979
-5.8352633	0.5368000	105.4099960
-5.8352633	0.5856000	103.7399979
-5.8352633	0.6344000	106.0499954
-5.8352633	0.6832000	106.9599991
-5.8352633	0.7320000	108.0899963
-5.8352633	0.7808000	106.2299957
-5.8352633	0.8296000	107.9300003
-5.8352633	0.8784000	106.5000000
-5.8352633	0.9272000	104.5599976
-5.8352633	0.9760000	105.1299973
-5.8352633	1.0247999	106.9099960
-5.8352633	1.0735999	107.4700012
-5.8352633	1.1223999	105.5100021
-5.8352633	1.1711999	105.2299957
-5.8352633	1.2200000	105.7900009
-5.8352633	1.2688000	104.5199966
-5.8352633	1.3176000	101.4599991
-5.8352633	1.3664000	103.7500000
-5.8352633	1.4152000	105.0999985
-5.8352633	1.4640000	106.6999969
-5.8352633	1.5128000	106.8099976
-5.8352633	1.5616000	107.0999985
-5.8352633	1.6104000	107.3099976
-5.8352633	1.6592000	106.5299988
-5.8352633	1.7079999	106.2399979
-5.8352633	1.7567999	106.4099960
-5.8352633	1.8055999	104.2600021
-5.8352633	1.8543999	111.5999985
-5.8352633	1.9031999	114.7900009
-5.8352633	1.9519999	111.3300018
-5.8352633	2.0007999	105.9000015
-5.8352633	2.0495999	103.6699982
-5.8352633	2.0983999	104.5299988
-5.8352633	2.1471999	103.2200012
-5.8352633	2.1959999	101.6199951
-5.8352633	2.2447999	103.5000000
-5.8352633	2.2935998	101.8199997
-5.8352633	2.3423998	105.1899948
-5.8352633	2.3912001	107.7299957
-5.8352633	2.4400001	108.6299973
-5.8352633	2.4888000	106.8199997
-5.8352633	2.5376000	104.9499969
-5.8352633	2.5864000	105.6899948
-5.8352633	2.6352000	106.6800003
-5.8352633	2.6840000	103.8600006
-5.8352633	2.7328000	105.2799988
-5.8352633	2.7816000	102.0800018
-5.8352633	2.8304000	104.6100006

-5.8352633	2.8792000	105.3799973
-5.8352633	2.9280000	106.0999985
-5.8352633	2.9768000	103.6500015
-5.8352633	3.0256000	101.7600021
-5.8352633	3.0743999	102.7200012
-5.8352633	3.1231999	100.4700012
-5.8352633	3.1719999	103.0599976
-5.8352633	3.2207999	103.5400009
-5.8352633	3.2695999	101.4099960
-5.8352633	3.3183999	104.4599991
-5.8352633	3.3671999	101.4199982
-5.8352633	3.4159999	103.0899963
-5.8352633	3.4647999	101.6999969
-5.8352633	3.5135999	99.5400009
-5.8352633	3.5623999	102.1800003
-5.8352633	3.6111999	104.5100021
-5.8352633	3.6599998	104.0999985
-5.8352633	3.7087998	103.9399948
-5.8352633	3.7575998	105.0599976
-5.8352633	3.8063998	103.5599976
-5.8352633	3.8551998	100.9000015
-5.8352633	3.9039998	102.0499954
-5.8352633	3.9527998	107.0499954
-5.8352633	4.0016003	106.6899948
-5.8352633	4.0503998	106.9499969
-5.8352633	4.0991998	106.1100006
-5.8352633	4.1479998	109.1399994
-5.8352633	4.1967998	109.0199966
-5.8352633	4.2455997	105.7699966
-5.8352633	4.2943997	105.4700012
-5.8352633	4.3431997	105.8699951
-5.8352633	4.3919997	105.6100006
-5.8352633	4.4407997	105.7200012
-5.8352633	4.4895997	108.5299988
-5.8352633	4.5383997	112.5000000
-5.8352633	4.5871997	114.1500015
-5.8352633	4.6359997	115.0000000
-5.8352633	4.6847997	116.4599991
-5.8352633	4.7336001	115.8799973
-5.8352633	4.7824001	111.1800003
-5.8352633	4.8312001	109.2900009
-5.8352633	4.8800001	109.8300018
-5.8352633	4.9288001	106.3799973
-5.8352633	4.9776001	104.5199966
-5.8352633	5.0264001	105.5199966
-5.8352633	5.0752001	105.7500000
-5.8352633	5.1240001	106.6699982
-5.8352633	5.1728001	102.1100006
-5.8352633	5.2216001	103.1100006
-5.8352633	5.2704000	102.7900009
-5.8352633	5.3192000	105.1899948
-5.8352633	5.3680000	105.3600006
-5.8352633	5.4168000	106.8199997
-5.8352633	5.4656000	106.3199997
-5.8352633	5.5144000	105.5999985
-5.8352633	5.5632000	100.5400009
-5.8352633	5.6120000	103.5899963
-5.8352633	5.6608000	101.7900009

-5.8352633	5.7096000	99.6699982
-5.8352633	5.7584000	103.9499969
-5.8352633	5.8072000	106.0899963
-5.8352633	5.8559999	105.2399979
-5.8352633	5.9047999	105.0800018
-5.8352633	5.9535999	105.5999985
-5.8352633	6.0023999	106.3899994
-5.8352633	6.0511999	105.3300018
-5.8352633	6.0999999	103.0899963
-5.8352633	6.1487999	99.9799957
-5.8352633	6.1975999	100.7099991
-5.8352633	6.2463999	104.5100021
-5.8352633	6.2951999	104.0000000
-5.8352633	6.3439999	102.8799973
-5.8352633	6.3927999	106.0599976
-5.8352633	6.4415998	104.5899963
-5.8352633	6.4903998	104.0899963
-5.8352633	6.5391998	104.2999954
-5.8352633	6.5879998	101.9799957
-5.8352633	6.6367998	105.6500015
-5.8352633	6.6855998	106.9300003
-5.8352633	6.7343998	106.2099991
-5.8352633	6.7831998	106.4099960
-5.8352633	6.8319998	105.2900009
-5.8352633	6.8807998	101.3499985
-5.8352633	6.9295998	102.8699951
-5.8352633	6.9783998	105.5800018
-5.8352633	7.0271997	105.2500000
-5.8352633	7.0759997	103.1100006
-5.8352633	7.1247997	99.6399994
-5.8352633	7.1735997	102.1699982
-5.8352633	7.2223997	101.0999985
-5.8352633	7.2711997	100.6899948
-5.8352633	7.3199997	103.5100021
-5.8352633	7.3687997	100.7699966
-5.8352633	7.4175997	104.1599960
-5.8352633	7.4663997	107.6800003
-5.8352633	7.5151997	105.1999969
-5.8352633	7.5639997	103.5199966
-5.8352633	7.6127996	106.3300018
-5.8352633	7.6615996	107.6100006
-5.8352633	7.7103996	105.4000015
-5.8352633	7.7591996	101.0299988
-5.8352633	7.8079996	103.9099960
-5.8352633	7.8567996	101.4300003
-5.8352633	7.9056001	100.1399994
-5.8352633	7.9544001	103.2999954
-5.8352633	8.0032005	103.0699997
-5.8352633	8.0520000	104.5699997
-5.8352633	8.1008005	103.1999969
-5.8352633	8.1496000	101.4499969
-5.8352633	8.1984005	100.9399948
-5.8352633	8.2472000	104.8899994
-5.8352633	8.2960005	105.8600006
-5.8352633	8.3448000	105.6999969
-5.8352633	8.3936005	105.7900009
-5.8352633	8.4424000	104.8799973
-5.8352633	8.4912004	105.2500000

-5.8352633	8.5400000	102.4399948
-5.8352633	8.5888004	103.2299957
-5.8352633	8.6375999	100.8199997
-5.8352633	8.6864004	99.8199997
-5.8352633	8.7351999	100.7999954
-5.8352633	8.7840004	104.2500000
-5.8352633	8.8327999	104.1999969
-5.8352633	8.8816004	103.7799988
-5.8352633	8.9303999	104.6500015
-5.8352633	8.9792004	100.0400009
-5.8352633	9.0279999	99.3499985
-5.8352633	9.0768003	103.5100021
-5.8352633	9.1255999	104.8899994
-5.8352633	9.1744003	106.0899963
-5.8352633	9.2231998	106.3499985
-5.8352633	9.2720003	106.1800003
-5.8352633	9.3207998	105.1800003
-5.8352633	9.3696003	103.6999969
-5.8352633	9.4184008	100.9300003
-5.8352633	9.4672003	102.8899994
-5.8352633	9.5160007	103.7299957
-5.8352633	9.5648003	104.2399979
-5.8352633	9.6136007	105.7900009
-5.8352633	9.6624002	105.5000000
-5.8352633	9.7112007	106.4000015
-5.8352633	9.7600002	102.5699997
-5.8352633	9.8088007	104.0199966
-5.8352633	9.8576002	103.7299957
-5.8352633	9.9064007	105.1699982
-5.8352633	9.9552002	106.1699982
-5.8352633	10.0040007	105.1699982
-5.8352633	10.0528002	100.8099976
-5.8352633	10.1016006	104.6299973
-5.8352633	10.1504002	103.7500000
-5.8352633	10.1992006	102.7399979
-5.8352633	10.2480001	106.1399994
-5.8352633	10.2968006	106.1599960
-5.8352633	10.3456001	100.7500000
-5.8352633	10.3944006	103.0699997
-5.8352633	10.4432001	102.1299973
-5.8352633	10.4920006	101.9599991
-5.8352633	10.5408001	100.1399994
-5.8352633	10.5896006	104.0000000
-5.8352633	10.6384001	105.4499969
-5.8352633	10.6872005	105.4899979
-5.8352633	10.7360001	104.4199982
-5.8352633	10.7848005	105.3799973
-5.8352633	10.8336000	104.7299957
-5.8352633	10.8824005	100.9000015
-5.8352633	10.9312000	103.2200012
-5.8352633	10.9800005	103.2900009
-5.8352633	11.0288000	103.7900009
-5.8352633	11.0776005	104.4599991
-5.8352633	11.1264000	103.4799957
-5.8352633	11.1752005	104.4199982
-5.8352633	11.2240000	105.7600021
-5.8352633	11.2728004	106.3399963
-5.8352633	11.3216000	102.9399948

-5.8352633	11.3704004	103.2799988
-5.8352633	11.4191999	105.4300003
-5.8352633	11.4680004	103.8799973
-5.8352633	11.5167999	104.4099960
-5.8352633	11.5656004	104.7900009
-5.8352633	11.6143999	102.7099991
-5.8352633	11.6632004	100.5299988
-5.8352633	11.7119999	102.2699966
-5.8352633	11.7608004	103.4199982
-5.8352633	11.8095999	103.3399963
-5.8352633	11.8584003	101.7900009
-5.8352633	11.9071999	104.9499969
-5.8352633	11.9560003	106.7099991
-5.8352633	12.0047998	104.8099976
-5.8352633	12.0536003	102.0599976
-5.8352633	12.1023998	104.2399979
-5.8352633	12.1512003	102.9499969
-5.8352633	12.1999998	101.7399979
-5.8352633	12.2488003	105.0899963
-5.8352633	12.2975998	107.2099991
-5.8352633	12.3464003	106.1299973
-5.8352633	12.3951998	104.7999954
-5.8352633	12.4440002	104.2799988
-5.8352633	12.4927998	104.5800018
-5.8352633	12.5416002	102.2099991
-5.8352633	12.5904007	104.3699951
-5.8352633	12.6392002	104.7600021
-5.8352633	12.6880007	105.5999985
-5.8352633	12.7368002	105.5599976
-5.8352633	12.7856007	105.9599991
-5.8352633	12.8344002	105.5800018
-5.8352633	12.8832006	103.9899979
-5.8352633	12.9320002	103.3600006
-5.8352633	12.9808006	104.7699966
-5.8352633	13.0296001	104.0400009
-5.8352633	13.0784006	100.8899994
-5.8352633	13.1272001	103.4700012
-5.8352633	13.1760006	104.0299988
-5.8352633	13.2248001	103.0599976
-5.8352633	13.2736006	103.2699966
-5.8352633	13.3224001	103.0299988
-5.8352633	13.3712006	104.4700012
-5.8352633	13.4200001	104.1699982
-5.8352633	13.4688005	94.0100021
-5.8352633	13.5176001	102.6800003
-5.8352633	13.5664005	103.7600021
-5.8352633	13.6152000	104.0000000
-5.8352633	13.6640005	101.7500000
-5.8352633	13.7128000	102.1999969
-5.8352633	13.7616005	105.5199966
-5.8352633	13.8104000	103.1999969
-5.8352633	13.8592005	103.8699951
-5.8352633	13.9080000	101.1399994
-5.8352633	13.9568005	99.9399948
-5.8352633	14.0056000	101.6100006
-5.8352633	14.0544004	102.6999969
-5.8352633	14.1032000	102.1599960
-5.8352633	14.1520004	103.4700012

-5.8352633	14.2007999	102.6699982
-5.8352633	14.2496004	99.6800003
-5.8352633	14.2983999	103.0400009
-5.8352633	14.3472004	104.6999969
-5.8352633	14.3959999	104.6599960
-5.8352633	14.4448004	103.2500000
-5.8352633	14.4935999	101.1699982
-5.8352633	14.5424004	103.5699997
-5.8352633	14.5911999	104.2799988
-5.8352633	14.6400003	100.9499969
-5.8352633	14.6887999	98.9499969
-5.8352633	14.7376003	103.9499969
-5.8352633	14.7863998	103.6999969
-5.8352633	14.8352003	102.6800003
-5.8352633	14.8839998	98.6800003
-5.8352633	14.9328003	101.4599991
-5.8352633	14.9815998	102.8699951
-5.8352633	15.0304003	97.4899979
-5.8291273	0.3416000	107.1399994
-5.8291273	0.3904000	106.6999969
-5.8291273	0.4392000	105.0499954
-5.8291273	0.4880000	106.8399963
-5.8291273	0.5368000	105.5499954
-5.8291273	0.5856000	103.3799973
-5.8291273	0.6344000	105.8499985
-5.8291273	0.6832000	107.1500015
-5.8291273	0.7320000	107.9000015
-5.8291273	0.7808000	105.7799988
-5.8291273	0.8296000	107.6500015
-5.8291273	0.8784000	106.0699997
-5.8291273	0.9272000	104.1599960
-5.8291273	0.9760000	104.2600021
-5.8291273	1.0247999	106.4199982
-5.8291273	1.0735999	106.4799957
-5.8291273	1.1223999	104.6699982
-5.8291273	1.1711999	105.2299957
-5.8291273	1.2200000	105.6399994
-5.8291273	1.2688000	104.4300003
-5.8291273	1.3176000	101.7699966
-5.8291273	1.3664000	104.2900009
-5.8291273	1.4152000	105.8399963
-5.8291273	1.4640000	107.0100021
-5.8291273	1.5128000	107.3799973
-5.8291273	1.5616000	107.3699951
-5.8291273	1.6104000	106.4899979
-5.8291273	1.6592000	106.2399979
-5.8291273	1.7079999	106.8799973
-5.8291273	1.7567999	106.9300003
-5.8291273	1.8055999	103.7799988
-5.8291273	1.8543999	111.9599991
-5.8291273	1.9031999	114.4099960
-5.8291273	1.9519999	111.9199982
-5.8291273	2.0007999	106.5699997
-5.8291273	2.0495999	104.5999985
-5.8291273	2.0983999	104.6100006
-5.8291273	2.1471999	103.3099976
-5.8291273	2.1959999	102.3399963
-5.8291273	2.2447999	103.7999954

-5.8291273	2.2935998	102.6100006
-5.8291273	2.3423998	103.9000015
-5.8291273	2.3912001	106.2399979
-5.8291273	2.4400001	107.7699966
-5.8291273	2.4888000	106.6899948
-5.8291273	2.5376000	102.9899979
-5.8291273	2.5864000	104.7099991
-5.8291273	2.6352000	106.7299957
-5.8291273	2.6840000	104.6500015
-5.8291273	2.7328000	105.9499969
-5.8291273	2.7816000	102.2299957
-5.8291273	2.8304000	103.7699966
-5.8291273	2.8792000	104.4599991
-5.8291273	2.9280000	105.2699966
-5.8291273	2.9768000	103.1699982
-5.8291273	3.0256000	101.6899948
-5.8291273	3.0743999	102.7200012
-5.8291273	3.1231999	101.4499969
-5.8291273	3.1719999	104.8699951
-5.8291273	3.2207999	105.4300003
-5.8291273	3.2695999	101.1500015
-5.8291273	3.3183999	104.4700012
-5.8291273	3.3671999	100.4499969
-5.8291273	3.4159999	103.3399963
-5.8291273	3.4647999	101.4599991
-5.8291273	3.5135999	99.7699966
-5.8291273	3.5623999	102.4799957
-5.8291273	3.6111999	104.5499954
-5.8291273	3.6599998	105.0000000
-5.8291273	3.7087998	105.3600006
-5.8291273	3.7575998	104.7600021
-5.8291273	3.8063998	103.0400009
-5.8291273	3.8551998	101.7399979
-5.8291273	3.9039998	103.5199966
-5.8291273	3.9527998	107.3899994
-5.8291273	4.0016003	105.9499969
-5.8291273	4.0503998	106.0599976
-5.8291273	4.0991998	105.2200012
-5.8291273	4.1479998	109.2600021
-5.8291273	4.1967998	109.3600006
-5.8291273	4.2455997	106.6800003
-5.8291273	4.2943997	105.2399979
-5.8291273	4.3431997	104.4599991
-5.8291273	4.3919997	105.0999985
-5.8291273	4.4407997	105.3099976
-5.8291273	4.4895997	107.9499969
-5.8291273	4.5383997	111.7200012
-5.8291273	4.5871997	113.4000015
-5.8291273	4.6359997	114.0899963
-5.8291273	4.6847997	114.9000015
-5.8291273	4.7336001	115.2299957
-5.8291273	4.7824001	111.0100021
-5.8291273	4.8312001	109.0400009
-5.8291273	4.8800001	109.6800003
-5.8291273	4.9288001	106.7099991
-5.8291273	4.9776001	104.0899963
-5.8291273	5.0264001	105.9300003
-5.8291273	5.0752001	106.3300018

-5.8291273	5.1240001	106.2500000
-5.8291273	5.1728001	101.0199966
-5.8291273	5.2216001	102.6699982
-5.8291273	5.2704000	102.2200012
-5.8291273	5.3192000	105.2999954
-5.8291273	5.3680000	104.8499985
-5.8291273	5.4168000	106.4099960
-5.8291273	5.4656000	106.2200012
-5.8291273	5.5144000	105.0899963
-5.8291273	5.5632000	100.1399994
-5.8291273	5.6120000	103.6399994
-5.8291273	5.6608000	101.2999954
-5.8291273	5.7096000	99.0000000
-5.8291273	5.7584000	104.2799988
-5.8291273	5.8072000	106.3899994
-5.8291273	5.8559999	106.7299957
-5.8291273	5.9047999	106.5499954
-5.8291273	5.9535999	106.0100021
-5.8291273	6.0023999	106.5699997
-5.8291273	6.0511999	105.2999954
-5.8291273	6.0999999	103.4399948
-5.8291273	6.1487999	99.9399948
-5.8291273	6.1975999	101.0199966
-5.8291273	6.2463999	105.1299973
-5.8291273	6.2951999	104.8099976
-5.8291273	6.3439999	103.5499954
-5.8291273	6.3927999	105.5199966
-5.8291273	6.4415998	103.7299957
-5.8291273	6.4903998	103.5499954
-5.8291273	6.5391998	104.4300003
-5.8291273	6.5879998	101.4499969
-5.8291273	6.6367998	105.8499985
-5.8291273	6.6855998	107.0599976
-5.8291273	6.7343998	106.6999969
-5.8291273	6.7831998	106.3199997
-5.8291273	6.8319998	104.4300003
-5.8291273	6.8807998	101.5999985
-5.8291273	6.9295998	101.8699951
-5.8291273	6.9783998	104.7099991
-5.8291273	7.0271997	104.5499954
-5.8291273	7.0759997	102.4599991
-5.8291273	7.1247997	99.5899963
-5.8291273	7.1735997	103.1699982
-5.8291273	7.2223997	100.0699997
-5.8291273	7.2711997	99.5199966
-5.8291273	7.3199997	103.0699997
-5.8291273	7.3687997	101.1699982
-5.8291273	7.4175997	103.8499985
-5.8291273	7.4663997	107.0599976
-5.8291273	7.5151997	104.2799988
-5.8291273	7.5639997	103.6800003
-5.8291273	7.6127996	106.2099991
-5.8291273	7.6615996	106.9300003
-5.8291273	7.7103996	104.5599976
-5.8291273	7.7591996	99.2900009
-5.8291273	7.8079996	102.8399963
-5.8291273	7.8567996	101.6299973
-5.8291273	7.9056001	99.3899994

-5.8291273	7.9544001	101.7299957
-5.8291273	8.0032005	101.0299988
-5.8291273	8.0520000	103.5999985
-5.8291273	8.1008005	103.6800003
-5.8291273	8.1496000	101.5100021
-5.8291273	8.1984005	100.6699982
-5.8291273	8.2472000	104.3199997
-5.8291273	8.2960005	105.3899994
-5.8291273	8.3448000	106.0599976
-5.8291273	8.3936005	106.4599991
-5.8291273	8.4424000	105.3799973
-5.8291273	8.4912004	105.9199982
-5.8291273	8.5400000	103.4099960
-5.8291273	8.5888004	102.8499985
-5.8291273	8.6375999	100.5899963
-5.8291273	8.6864004	100.4000015
-5.8291273	8.7351999	101.1699982
-5.8291273	8.7840004	105.0299988
-5.8291273	8.8327999	104.1899948
-5.8291273	8.8816004	103.3799973
-5.8291273	8.9303999	105.2200012
-5.8291273	8.9792004	101.3199997
-5.8291273	9.0279999	100.4300003
-5.8291273	9.0768003	103.8399963
-5.8291273	9.1255999	104.3899994
-5.8291273	9.1744003	105.5499954
-5.8291273	9.2231998	106.2500000
-5.8291273	9.2720003	106.0000000
-5.8291273	9.3207998	104.6399994
-5.8291273	9.3696003	103.2099991
-5.8291273	9.4184008	101.1500015
-5.8291273	9.4672003	102.0699997
-5.8291273	9.5160007	104.0999985
-5.8291273	9.5648003	104.1199951
-5.8291273	9.6136007	107.0800018
-5.8291273	9.6624002	106.1199951
-5.8291273	9.7112007	105.9799957
-5.8291273	9.7600002	100.7699966
-5.8291273	9.8088007	103.7399979
-5.8291273	9.8576002	103.7600021
-5.8291273	9.9064007	105.4199982
-5.8291273	9.9552002	106.4700012
-5.8291273	10.0040007	105.2200012
-5.8291273	10.0528002	100.0800018
-5.8291273	10.1016006	104.6699982
-5.8291273	10.1504002	103.4700012
-5.8291273	10.1992006	102.3799973
-5.8291273	10.2480001	105.5100021
-5.8291273	10.2968006	105.7799988
-5.8291273	10.3456001	100.1599960
-5.8291273	10.3944006	102.1100006
-5.8291273	10.4432001	101.7200012
-5.8291273	10.4920006	102.3199997
-5.8291273	10.5408001	99.1599960
-5.8291273	10.5896006	104.1699982
-5.8291273	10.6384001	104.8899994
-5.8291273	10.6872005	104.3899994
-5.8291273	10.7360001	104.5999985

-5.8291273	10.7848005	104.7099991
-5.8291273	10.8336000	104.3300018
-5.8291273	10.8824005	101.4300003
-5.8291273	10.9312000	104.4499969
-5.8291273	10.9800005	104.0199966
-5.8291273	11.0288000	103.5000000
-5.8291273	11.0776005	104.4300003
-5.8291273	11.1264000	104.0599976
-5.8291273	11.1752005	104.5199966
-5.8291273	11.2240000	106.2299957
-5.8291273	11.2728004	106.2099991
-5.8291273	11.3216000	101.8899994
-5.8291273	11.3704004	103.1399994
-5.8291273	11.4191999	105.1999969
-5.8291273	11.4680004	104.3899994
-5.8291273	11.5167999	104.2600021
-5.8291273	11.5656004	104.3300018
-5.8291273	11.6143999	102.6100006
-5.8291273	11.6632004	100.6500015
-5.8291273	11.7119999	101.6699982
-5.8291273	11.7608004	103.1599960
-5.8291273	11.8095999	103.6800003
-5.8291273	11.8584003	103.2799988
-5.8291273	11.9071999	104.8799973
-5.8291273	11.9560003	106.0599976
-5.8291273	12.0047998	104.5800018
-5.8291273	12.0536003	102.1999969
-5.8291273	12.1023998	102.7999954
-5.8291273	12.1512003	102.6599960
-5.8291273	12.1999998	102.0699997
-5.8291273	12.2488003	105.4899979
-5.8291273	12.2975998	106.7999954
-5.8291273	12.3464003	105.6299973
-5.8291273	12.3951998	105.5699997
-5.8291273	12.4440002	104.9599991
-5.8291273	12.4927998	104.5100021
-5.8291273	12.5416002	101.7099991
-5.8291273	12.5904007	103.8699951
-5.8291273	12.6392002	104.1599960
-5.8291273	12.6880007	105.5199966
-5.8291273	12.7368002	105.1999969
-5.8291273	12.7856007	105.8799973
-5.8291273	12.8344002	105.9199982
-5.8291273	12.8832006	104.7999954
-5.8291273	12.9320002	104.1100006
-5.8291273	12.9808006	105.3300018
-5.8291273	13.0296001	103.5899963
-5.8291273	13.0784006	99.6599960
-5.8291273	13.1272001	103.5699997
-5.8291273	13.1760006	104.7299957
-5.8291273	13.2248001	102.6899948
-5.8291273	13.2736006	103.1999969
-5.8291273	13.3224001	102.6599960
-5.8291273	13.3712006	104.4099960
-5.8291273	13.4200001	104.1199951
-5.8291273	13.4688005	94.7299957
-5.8291273	13.5176001	103.1999969
-5.8291273	13.5664005	104.3899994

-5.8291273	13.6152000	104.0999985
-5.8291273	13.6640005	102.6399994
-5.8291273	13.7128000	102.7500000
-5.8291273	13.7616005	105.4599991
-5.8291273	13.8104000	104.2099991
-5.8291273	13.8592005	104.1199951
-5.8291273	13.9080000	101.1999969
-5.8291273	13.9568005	99.7999954
-5.8291273	14.0056000	100.9499969
-5.8291273	14.0544004	101.3199997
-5.8291273	14.1032000	102.4000015
-5.8291273	14.1520004	102.8499985
-5.8291273	14.2007999	101.8699951
-5.8291273	14.2496004	99.0299988
-5.8291273	14.2983999	102.8199997
-5.8291273	14.3472004	104.5899963
-5.8291273	14.3959999	104.4399948
-5.8291273	14.4448004	102.9399948
-5.8291273	14.4935999	101.7500000
-5.8291273	14.5424004	103.7500000
-5.8291273	14.5911999	103.7999954
-5.8291273	14.6400003	102.5299988
-5.8291273	14.6887999	100.0100021
-5.8291273	14.7376003	104.8199997
-5.8291273	14.7863998	105.0199966
-5.8291273	14.8352003	103.4899979
-5.8291273	14.8839998	99.0599976
-5.8291273	14.9328003	101.5699997
-5.8291273	14.9815998	102.5800018
-5.8291273	15.0304003	96.1899948
-5.8229914	0.3416000	107.2299957
-5.8229914	0.3904000	107.1500015
-5.8229914	0.4392000	106.2200012
-5.8229914	0.4880000	106.9700012
-5.8229914	0.5368000	105.0800018
-5.8229914	0.5856000	102.9199982
-5.8229914	0.6344000	105.9599991
-5.8229914	0.6832000	107.6399994
-5.8229914	0.7320000	108.1500015
-5.8229914	0.7808000	105.6500015
-5.8229914	0.8296000	107.6299973
-5.8229914	0.8784000	106.0800018
-5.8229914	0.9272000	104.2600021
-5.8229914	0.9760000	103.7399979
-5.8229914	1.0247999	105.7799988
-5.8229914	1.0735999	105.3899994
-5.8229914	1.1223999	103.9799957
-5.8229914	1.1711999	104.6299973
-5.8229914	1.2200000	104.5599976
-5.8229914	1.2688000	103.3199997
-5.8229914	1.3176000	101.9099960
-5.8229914	1.3664000	105.0699997
-5.8229914	1.4152000	106.7900009
-5.8229914	1.4640000	106.5800018
-5.8229914	1.5128000	107.5299988
-5.8229914	1.5616000	107.0800018
-5.8229914	1.6104000	105.7399979
-5.8229914	1.6592000	106.3600006

-5.8229914	1.7079999	107.8600006
-5.8229914	1.7567999	107.5800018
-5.8229914	1.8055999	103.1999969
-5.8229914	1.8543999	112.2600021
-5.8229914	1.9031999	114.0599976
-5.8229914	1.9519999	112.6100006
-5.8229914	2.0007999	107.4000015
-5.8229914	2.0495999	106.0400009
-5.8229914	2.0983999	105.2600021
-5.8229914	2.1471999	103.5499954
-5.8229914	2.1959999	102.4199982
-5.8229914	2.2447999	103.4300003
-5.8229914	2.2935998	102.7500000
-5.8229914	2.3423998	102.2500000
-5.8229914	2.3912001	104.4000015
-5.8229914	2.4400001	106.6500015
-5.8229914	2.4888000	106.6999969
-5.8229914	2.5376000	102.4700012
-5.8229914	2.5864000	104.2299957
-5.8229914	2.6352000	106.7099991
-5.8229914	2.6840000	104.9099960
-5.8229914	2.7328000	106.2900009
-5.8229914	2.7816000	102.8499985
-5.8229914	2.8304000	103.3300018
-5.8229914	2.8792000	103.8300018
-5.8229914	2.9280000	104.5400009
-5.8229914	2.9768000	104.0199966
-5.8229914	3.0256000	102.5699997
-5.8229914	3.0743999	102.6999969
-5.8229914	3.1231999	102.9499969
-5.8229914	3.1719999	106.6100006
-5.8229914	3.2207999	106.8199997
-5.8229914	3.2695999	100.2699966
-5.8229914	3.3183999	102.4499969
-5.8229914	3.3671999	100.4599991
-5.8229914	3.4159999	104.1299973
-5.8229914	3.4647999	102.5599976
-5.8229914	3.5135999	100.9399948
-5.8229914	3.5623999	102.9799957
-5.8229914	3.6111999	103.9300003
-5.8229914	3.6599998	104.8699951
-5.8229914	3.7087998	106.0400009
-5.8229914	3.7575998	104.4899979
-5.8229914	3.8063998	102.5699997
-5.8229914	3.8551998	102.7900009
-5.8229914	3.9039998	104.9000015
-5.8229914	3.9527998	107.0000000
-5.8229914	4.0016003	104.4000015
-5.8229914	4.0503998	104.3799973
-5.8229914	4.0991998	104.4099960
-5.8229914	4.1479998	109.1399994
-5.8229914	4.1967998	109.5199966
-5.8229914	4.2455997	107.4300003
-5.8229914	4.2943997	105.5699997
-5.8229914	4.3431997	104.1299973
-5.8229914	4.3919997	104.7099991
-5.8229914	4.4407997	104.5299988
-5.8229914	4.4895997	107.0299988

-5.8229914	4.5383997	111.3600006
-5.8229914	4.5871997	112.9899979
-5.8229914	4.6359997	112.9799957
-5.8229914	4.6847997	113.3399963
-5.8229914	4.7336001	114.4199982
-5.8229914	4.7824001	110.6100006
-5.8229914	4.8312001	108.8600006
-5.8229914	4.8800001	109.5599976
-5.8229914	4.9288001	107.0800018
-5.8229914	4.9776001	103.5100021
-5.8229914	5.0264001	105.3099976
-5.8229914	5.0752001	106.2099991
-5.8229914	5.1240001	105.5100021
-5.8229914	5.1728001	101.0299988
-5.8229914	5.2216001	101.8799973
-5.8229914	5.2704000	102.5299988
-5.8229914	5.3192000	105.0999985
-5.8229914	5.3680000	105.3099976
-5.8229914	5.4168000	107.0899963
-5.8229914	5.4656000	106.6599960
-5.8229914	5.5144000	104.8399963
-5.8229914	5.5632000	100.1599960
-5.8229914	5.6120000	103.9499969
-5.8229914	5.6608000	100.6599960
-5.8229914	5.7096000	98.9899979
-5.8229914	5.7584000	104.1999969
-5.8229914	5.8072000	106.0299988
-5.8229914	5.8559999	107.2900009
-5.8229914	5.9047999	107.1399994
-5.8229914	5.9535999	105.6100006
-5.8229914	6.0023999	106.2500000
-5.8229914	6.0511999	104.8399963
-5.8229914	6.0999999	103.8499985
-5.8229914	6.1487999	100.7500000
-5.8229914	6.1975999	101.7099991
-5.8229914	6.2463999	105.6699982
-5.8229914	6.2951999	105.4700012
-5.8229914	6.3439999	104.2600021
-5.8229914	6.3927999	105.5000000
-5.8229914	6.4415998	103.5499954
-5.8229914	6.4903998	103.4799957
-5.8229914	6.5391998	104.8600006
-5.8229914	6.5879998	102.2699966
-5.8229914	6.6367998	106.5899963
-5.8229914	6.6855998	107.2600021
-5.8229914	6.7343998	107.2999954
-5.8229914	6.7831998	106.4300003
-5.8229914	6.8319998	103.5999985
-5.8229914	6.8807998	101.7900009
-5.8229914	6.9295998	101.4599991
-5.8229914	6.9783998	103.3399963
-5.8229914	7.0271997	103.2699966
-5.8229914	7.0759997	101.1399994
-5.8229914	7.1247997	100.5899963
-5.8229914	7.1735997	103.6199951
-5.8229914	7.2223997	99.0599976
-5.8229914	7.2711997	99.2999954
-5.8229914	7.3199997	103.1299973

-5.8229914	7.3687997	101.1399994
-5.8229914	7.4175997	103.8399963
-5.8229914	7.4663997	106.8499985
-5.8229914	7.5151997	103.5699997
-5.8229914	7.5639997	103.9599991
-5.8229914	7.6127996	105.6299973
-5.8229914	7.6615996	106.0299988
-5.8229914	7.7103996	104.0699997
-5.8229914	7.7591996	99.1999969
-5.8229914	7.8079996	102.2600021
-5.8229914	7.8567996	101.7699966
-5.8229914	7.9056001	99.0199966
-5.8229914	7.9544001	100.4499969
-5.8229914	8.0032005	98.8699951
-5.8229914	8.0520000	102.6899948
-5.8229914	8.1008005	103.7399979
-5.8229914	8.1496000	101.4599991
-5.8229914	8.1984005	101.6599960
-5.8229914	8.2472000	104.6899948
-5.8229914	8.2960005	104.8499985
-5.8229914	8.3448000	105.6599960
-5.8229914	8.3936005	106.5699997
-5.8229914	8.4424000	105.5499954
-5.8229914	8.4912004	106.9700012
-5.8229914	8.5400000	104.2099991
-5.8229914	8.5888004	102.1399994
-5.8229914	8.6375999	100.3699951
-5.8229914	8.6864004	100.9399948
-5.8229914	8.7351999	101.4300003
-5.8229914	8.7840004	105.5699997
-5.8229914	8.8327999	103.6500015
-5.8229914	8.8816004	102.5800018
-5.8229914	8.9303999	105.5000000
-5.8229914	8.9792004	103.0999985
-5.8229914	9.0279999	101.8300018
-5.8229914	9.0768003	104.0100021
-5.8229914	9.1255999	104.4300003
-5.8229914	9.1744003	105.1299973
-5.8229914	9.2231998	105.7999954
-5.8229914	9.2720003	105.0299988
-5.8229914	9.3207998	103.9000015
-5.8229914	9.3696003	102.5299988
-5.8229914	9.4184008	101.2299957
-5.8229914	9.4672003	101.4199982
-5.8229914	9.5160007	103.7099991
-5.8229914	9.5648003	102.9099960
-5.8229914	9.6136007	106.3600006
-5.8229914	9.6624002	105.9599991
-5.8229914	9.7112007	105.8899994
-5.8229914	9.7600002	100.5199966
-5.8229914	9.8088007	103.8899994
-5.8229914	9.8576002	103.3699951
-5.8229914	9.9064007	105.2299957
-5.8229914	9.9552002	106.1599960
-5.8229914	10.0040007	104.9099960
-5.8229914	10.0528002	99.3300018
-5.8229914	10.1016006	104.7699966
-5.8229914	10.1504002	103.3199997

-5.8229914	10.1992006	102.7799988
-5.8229914	10.2480001	105.3600006
-5.8229914	10.2968006	105.3600006
-5.8229914	10.3456001	99.1599960
-5.8229914	10.3944006	100.9499969
-5.8229914	10.4432001	101.9599991
-5.8229914	10.4920006	103.5199966
-5.8229914	10.5408001	100.1899948
-5.8229914	10.5896006	104.8099976
-5.8229914	10.6384001	104.7099991
-5.8229914	10.6872005	103.1299973
-5.8229914	10.7360001	104.7500000
-5.8229914	10.7848005	104.1999969
-5.8229914	10.8336000	104.1899948
-5.8229914	10.8824005	102.0999985
-5.8229914	10.9312000	105.2399979
-5.8229914	10.9800005	104.9599991
-5.8229914	11.0288000	103.7999954
-5.8229914	11.0776005	104.1699982
-5.8229914	11.1264000	102.7299957
-5.8229914	11.1752005	104.5699997
-5.8229914	11.2240000	106.6699982
-5.8229914	11.2728004	106.2399979
-5.8229914	11.3216000	101.3799973
-5.8229914	11.3704004	103.2999954
-5.8229914	11.4191999	104.7299957
-5.8229914	11.4680004	104.1899948
-5.8229914	11.5167999	103.7200012
-5.8229914	11.5656004	103.6999969
-5.8229914	11.6143999	102.7999954
-5.8229914	11.6632004	101.0999985
-5.8229914	11.7119999	100.8799973
-5.8229914	11.7608004	102.6100006
-5.8229914	11.8095999	103.6800003
-5.8229914	11.8584003	104.2799988
-5.8229914	11.9071999	104.8399963
-5.8229914	11.9560003	105.6899948
-5.8229914	12.0047998	104.1299973
-5.8229914	12.0536003	102.1100006
-5.8229914	12.1023998	101.5599976
-5.8229914	12.1512003	102.4899979
-5.8229914	12.1999998	102.4000015
-5.8229914	12.2488003	105.2999954
-5.8229914	12.2975998	105.9300003
-5.8229914	12.3464003	104.6999969
-5.8229914	12.3951998	105.9099960
-5.8229914	12.4440002	105.2799988
-5.8229914	12.4927998	104.6599960
-5.8229914	12.5416002	102.4000015
-5.8229914	12.5904007	103.9300003
-5.8229914	12.6392002	103.4599991
-5.8229914	12.6880007	105.3199997
-5.8229914	12.7368002	104.9799957
-5.8229914	12.7856007	105.6800003
-5.8229914	12.8344002	105.8899994
-5.8229914	12.8832006	105.3699951
-5.8229914	12.9320002	104.3499985
-5.8229914	12.9808006	105.2399979

-5.8229914	13.0296001	103.5000000
-5.8229914	13.0784006	99.7399979
-5.8229914	13.1272001	103.8399963
-5.8229914	13.1760006	104.8699951
-5.8229914	13.2248001	102.2399979
-5.8229914	13.2736006	102.6199951
-5.8229914	13.3224001	101.6299973
-5.8229914	13.3712006	103.4799957
-5.8229914	13.4200001	104.2900009
-5.8229914	13.4688005	96.6199951
-5.8229914	13.5176001	103.9300003
-5.8229914	13.5664005	105.1999969
-5.8229914	13.6152000	104.0000000
-5.8229914	13.6640005	103.6599960
-5.8229914	13.7128000	103.5299988
-5.8229914	13.7616005	105.3699951
-5.8229914	13.8104000	104.4300003
-5.8229914	13.8592005	104.2099991
-5.8229914	13.9080000	101.5400009
-5.8229914	13.9568005	99.9499969
-5.8229914	14.0056000	100.7399979
-5.8229914	14.0544004	100.4399948
-5.8229914	14.1032000	103.1299973
-5.8229914	14.1520004	102.8699951
-5.8229914	14.2007999	101.9799957
-5.8229914	14.2496004	99.3799973
-5.8229914	14.2983999	103.2299957
-5.8229914	14.3472004	104.2900009
-5.8229914	14.3959999	103.6599960
-5.8229914	14.4448004	102.1599960
-5.8229914	14.4935999	102.4799957
-5.8229914	14.5424004	103.7900009
-5.8229914	14.5911999	102.8899994
-5.8229914	14.6400003	103.8899994
-5.8229914	14.6887999	101.5100021
-5.8229914	14.7376003	105.1899948
-5.8229914	14.7863998	105.1500015
-5.8229914	14.8352003	103.8499985
-5.8229914	14.8839998	99.6500015
-5.8229914	14.9328003	101.3300018
-5.8229914	14.9815998	101.7799988
-5.8229914	15.0304003	95.5199966
-5.8168554	0.3416000	106.6999969
-5.8168554	0.3904000	107.0899963
-5.8168554	0.4392000	107.2200012
-5.8168554	0.4880000	107.0800018
-5.8168554	0.5368000	104.3799973
-5.8168554	0.5856000	103.0400009
-5.8168554	0.6344000	106.2799988
-5.8168554	0.6832000	108.3199997
-5.8168554	0.7320000	108.8699951
-5.8168554	0.7808000	106.9099960
-5.8168554	0.8296000	108.2500000
-5.8168554	0.8784000	106.0699997
-5.8168554	0.9272000	104.9300003
-5.8168554	0.9760000	104.3300018
-5.8168554	1.0247999	105.4199982
-5.8168554	1.0735999	104.2699966

-5.8168554	1.1223999	103.9300003
-5.8168554	1.1711999	104.4799957
-5.8168554	1.2200000	103.1299973
-5.8168554	1.2688000	102.2999954
-5.8168554	1.3176000	102.8300018
-5.8168554	1.3664000	105.9199982
-5.8168554	1.4152000	107.6699982
-5.8168554	1.4640000	106.0499954
-5.8168554	1.5128000	107.4499969
-5.8168554	1.5616000	107.1999969
-5.8168554	1.6104000	105.3600006
-5.8168554	1.6592000	106.2299957
-5.8168554	1.7079999	108.4899979
-5.8168554	1.7567999	108.3699951
-5.8168554	1.8055999	102.9499969
-5.8168554	1.8543999	112.1800003
-5.8168554	1.9031999	113.1899948
-5.8168554	1.9519999	112.6599960
-5.8168554	2.0007999	107.4499969
-5.8168554	2.0495999	106.5599976
-5.8168554	2.0983999	105.3799973
-5.8168554	2.1471999	103.5800018
-5.8168554	2.1959999	102.7500000
-5.8168554	2.2447999	102.8399963
-5.8168554	2.2935998	103.4599991
-5.8168554	2.3423998	102.0199966
-5.8168554	2.3912001	103.9799957
-5.8168554	2.4400001	106.3799973
-5.8168554	2.4888000	106.5899963
-5.8168554	2.5376000	100.9799957
-5.8168554	2.5864000	103.5000000
-5.8168554	2.6352000	106.6500015
-5.8168554	2.6840000	105.1899948
-5.8168554	2.7328000	107.2399979
-5.8168554	2.7816000	104.0599976
-5.8168554	2.8304000	103.6699982
-5.8168554	2.8792000	104.2399979
-5.8168554	2.9280000	104.3300018
-5.8168554	2.9768000	104.9399948
-5.8168554	3.0256000	103.7500000
-5.8168554	3.0743999	103.1199951
-5.8168554	3.1231999	104.3300018
-5.8168554	3.1719999	107.9300003
-5.8168554	3.2207999	108.0299988
-5.8168554	3.2695999	101.5899963
-5.8168554	3.3183999	100.9700012
-5.8168554	3.3671999	100.5999985
-5.8168554	3.4159999	104.4499969
-5.8168554	3.4647999	104.0100021
-5.8168554	3.5135999	102.4599991
-5.8168554	3.5623999	103.9700012
-5.8168554	3.6111999	103.8899994
-5.8168554	3.6599998	104.7200012
-5.8168554	3.7087998	106.3399963
-5.8168554	3.7575998	104.0499954
-5.8168554	3.8063998	102.2900009
-5.8168554	3.8551998	103.5699997
-5.8168554	3.9039998	105.5400009

-5.8168554	3.9527998	106.5000000
-5.8168554	4.0016003	103.8899994
-5.8168554	4.0503998	102.5499954
-5.8168554	4.0991998	103.4199982
-5.8168554	4.1479998	108.5599976
-5.8168554	4.1967998	108.9399948
-5.8168554	4.2455997	107.5699997
-5.8168554	4.2943997	105.7099991
-5.8168554	4.3431997	105.1699982
-5.8168554	4.3919997	105.5400009
-5.8168554	4.4407997	104.3399963
-5.8168554	4.4895997	106.9199982
-5.8168554	4.5383997	111.0400009
-5.8168554	4.5871997	112.4899979
-5.8168554	4.6359997	112.5100021
-5.8168554	4.6847997	112.6599960
-5.8168554	4.7336001	114.2299957
-5.8168554	4.7824001	110.5800018
-5.8168554	4.8312001	109.2900009
-5.8168554	4.8800001	110.4499969
-5.8168554	4.9288001	108.3499985
-5.8168554	4.9776001	103.0599976
-5.8168554	5.0264001	104.2200012
-5.8168554	5.0752001	106.1299973
-5.8168554	5.1240001	105.2500000
-5.8168554	5.1728001	101.5699997
-5.8168554	5.2216001	102.1699982
-5.8168554	5.2704000	103.0299988
-5.8168554	5.3192000	104.4799957
-5.8168554	5.3680000	105.1500015
-5.8168554	5.4168000	106.9799957
-5.8168554	5.4656000	106.6699982
-5.8168554	5.5144000	105.0899963
-5.8168554	5.5632000	101.6199951
-5.8168554	5.6120000	104.8399963
-5.8168554	5.6608000	100.2900009
-5.8168554	5.7096000	100.4700012
-5.8168554	5.7584000	105.1299973
-5.8168554	5.8072000	106.3899994
-5.8168554	5.8559999	107.7399979
-5.8168554	5.9047999	107.5599976
-5.8168554	5.9535999	105.5000000
-5.8168554	6.0023999	106.1599960
-5.8168554	6.0511999	104.0599976
-5.8168554	6.0999999	103.6399994
-5.8168554	6.1487999	101.7600021
-5.8168554	6.1975999	102.5100021
-5.8168554	6.2463999	106.2399979
-5.8168554	6.2951999	106.0699997
-5.8168554	6.3439999	104.4499969
-5.8168554	6.3927999	105.0699997
-5.8168554	6.4415998	103.5999985
-5.8168554	6.4903998	103.7699966
-5.8168554	6.5391998	105.3699951
-5.8168554	6.5879998	103.0699997
-5.8168554	6.6367998	107.2600021
-5.8168554	6.6855998	107.2600021
-5.8168554	6.7343998	107.4899979

-5.8168554	6.7831998	105.7799988
-5.8168554	6.8319998	102.5599976
-5.8168554	6.8807998	103.1599960
-5.8168554	6.9295998	102.2699966
-5.8168554	6.9783998	103.2900009
-5.8168554	7.0271997	102.7600021
-5.8168554	7.0759997	99.8300018
-5.8168554	7.1247997	101.0599976
-5.8168554	7.1735997	103.7099991
-5.8168554	7.2223997	98.4000015
-5.8168554	7.2711997	100.0499954
-5.8168554	7.3199997	103.2799988
-5.8168554	7.3687997	100.7900009
-5.8168554	7.4175997	103.9199982
-5.8168554	7.4663997	106.7500000
-5.8168554	7.5151997	103.7600021
-5.8168554	7.5639997	103.4300003
-5.8168554	7.6127996	104.4399948
-5.8168554	7.6615996	105.1699982
-5.8168554	7.7103996	103.9099960
-5.8168554	7.7591996	99.5999985
-5.8168554	7.8079996	102.2999954
-5.8168554	7.8567996	102.0800018
-5.8168554	7.9056001	98.7299957
-5.8168554	7.9544001	99.5699997
-5.8168554	8.0032005	97.4399948
-5.8168554	8.0520000	102.0100021
-5.8168554	8.1008005	103.6599960
-5.8168554	8.1496000	101.7200012
-5.8168554	8.1984005	101.2699966
-5.8168554	8.2472000	104.6699982
-5.8168554	8.2960005	104.1599960
-5.8168554	8.3448000	105.0299988
-5.8168554	8.3936005	106.2999954
-5.8168554	8.4424000	105.4300003
-5.8168554	8.4912004	107.6299973
-5.8168554	8.5400000	105.2399979
-5.8168554	8.5888004	101.8899994
-5.8168554	8.6375999	100.2399979
-5.8168554	8.6864004	100.8499985
-5.8168554	8.7351999	102.4499969
-5.8168554	8.7840004	106.0299988
-5.8168554	8.8327999	102.9799957
-5.8168554	8.8816004	101.7999954
-5.8168554	8.9303999	105.2699966
-5.8168554	8.9792004	103.6199951
-5.8168554	9.0279999	101.7200012
-5.8168554	9.0768003	103.5800018
-5.8168554	9.1255999	103.8499985
-5.8168554	9.1744003	104.5000000
-5.8168554	9.2231998	105.4000015
-5.8168554	9.2720003	104.0800018
-5.8168554	9.3207998	103.4099960
-5.8168554	9.3696003	101.9300003
-5.8168554	9.4184008	100.7099991
-5.8168554	9.4672003	99.7200012
-5.8168554	9.5160007	102.1800003
-5.8168554	9.5648003	102.1599960

-5.8168554	9.6136007	105.4799957
-5.8168554	9.6624002	105.4399948
-5.8168554	9.7112007	105.7900009
-5.8168554	9.7600002	100.8799973
-5.8168554	9.8088007	103.5999985
-5.8168554	9.8576002	102.9199982
-5.8168554	9.9064007	105.1100006
-5.8168554	9.9552002	105.7799988
-5.8168554	10.0040007	104.2500000
-5.8168554	10.0528002	100.1699982
-5.8168554	10.1016006	104.7399979
-5.8168554	10.1504002	103.1699982
-5.8168554	10.1992006	103.6899948
-5.8168554	10.2480001	105.4099960
-5.8168554	10.2968006	104.3899994
-5.8168554	10.3456001	98.9199982
-5.8168554	10.3944006	100.6100006
-5.8168554	10.4432001	102.2900009
-5.8168554	10.4920006	104.3199997
-5.8168554	10.5408001	101.5599976
-5.8168554	10.5896006	105.6899948
-5.8168554	10.6384001	104.2600021
-5.8168554	10.6872005	101.6899948
-5.8168554	10.7360001	104.9099960
-5.8168554	10.7848005	104.2500000
-5.8168554	10.8336000	104.4300003
-5.8168554	10.8824005	103.2799988
-5.8168554	10.9312000	105.9099960
-5.8168554	10.9800005	105.5599976
-5.8168554	11.0288000	103.9499969
-5.8168554	11.0776005	103.7699966
-5.8168554	11.1264000	101.4199982
-5.8168554	11.1752005	103.9000015
-5.8168554	11.2240000	106.2299957
-5.8168554	11.2728004	106.3499985
-5.8168554	11.3216000	101.7900009
-5.8168554	11.3704004	103.4399948
-5.8168554	11.4191999	104.0599976
-5.8168554	11.4680004	104.0499954
-5.8168554	11.5167999	103.5199966
-5.8168554	11.5656004	103.4700012
-5.8168554	11.6143999	102.6199951
-5.8168554	11.6632004	100.6199951
-5.8168554	11.7119999	99.6199951
-5.8168554	11.7608004	102.2200012
-5.8168554	11.8095999	103.7600021
-5.8168554	11.8584003	104.7999954
-5.8168554	11.9071999	104.5999985
-5.8168554	11.9560003	105.0400009
-5.8168554	12.0047998	103.4499969
-5.8168554	12.0536003	102.2099991
-5.8168554	12.1023998	100.7699966
-5.8168554	12.1512003	102.2999954
-5.8168554	12.1999998	102.8600006
-5.8168554	12.2488003	105.1500015
-5.8168554	12.2975998	105.0999985
-5.8168554	12.3464003	104.0599976
-5.8168554	12.3951998	106.4599991

-5.8168554	12.4440002	105.6699982
-5.8168554	12.4927998	104.8799973
-5.8168554	12.5416002	102.8699951
-5.8168554	12.5904007	104.3399963
-5.8168554	12.6392002	103.6800003
-5.8168554	12.6880007	104.9599991
-5.8168554	12.7368002	104.3199997
-5.8168554	12.7856007	104.9899979
-5.8168554	12.8344002	105.8399963
-5.8168554	12.8832006	105.7799988
-5.8168554	12.9320002	104.1199951
-5.8168554	12.9808006	104.9499969
-5.8168554	13.0296001	103.2600021
-5.8168554	13.0784006	100.5699997
-5.8168554	13.1272001	103.8600006
-5.8168554	13.1760006	104.6100006
-5.8168554	13.2248001	101.8199997
-5.8168554	13.2736006	102.1299973
-5.8168554	13.3224001	100.5999985
-5.8168554	13.3712006	102.7099991
-5.8168554	13.4200001	104.4000015
-5.8168554	13.4688005	98.6800003
-5.8168554	13.5176001	104.4099960
-5.8168554	13.5664005	105.5299988
-5.8168554	13.6152000	103.0999985
-5.8168554	13.6640005	104.2699966
-5.8168554	13.7128000	104.1500015
-5.8168554	13.7616005	105.3099976
-5.8168554	13.8104000	104.3099976
-5.8168554	13.8592005	104.3699951
-5.8168554	13.9080000	101.8899994
-5.8168554	13.9568005	100.2500000
-5.8168554	14.0056000	100.8399963
-5.8168554	14.0544004	99.4499969
-5.8168554	14.1032000	103.3099976
-5.8168554	14.1520004	101.6500015
-5.8168554	14.2007999	101.8600006
-5.8168554	14.2496004	99.9799957
-5.8168554	14.2983999	103.6999969
-5.8168554	14.3472004	103.7600021
-5.8168554	14.3959999	102.8499985
-5.8168554	14.4448004	101.5100021
-5.8168554	14.4935999	103.3199997
-5.8168554	14.5424004	104.1999969
-5.8168554	14.5911999	101.6599960
-5.8168554	14.6400003	104.8600006
-5.8168554	14.6887999	103.2799988
-5.8168554	14.7376003	104.8300018
-5.8168554	14.7863998	104.8499985
-5.8168554	14.8352003	104.4700012
-5.8168554	14.8839998	101.0800018
-5.8168554	14.9328003	101.0199966
-5.8168554	14.9815998	100.5999985
-5.8168554	15.0304003	95.4599991
-5.8107195	0.3416000	106.2799988
-5.8107195	0.3904000	106.9199982
-5.8107195	0.4392000	108.3399963
-5.8107195	0.4880000	106.6899948

-5.8107195	0.5368000	102.9599991
-5.8107195	0.5856000	103.1100006
-5.8107195	0.6344000	106.6899948
-5.8107195	0.6832000	108.9099960
-5.8107195	0.7320000	109.5000000
-5.8107195	0.7808000	108.3199997
-5.8107195	0.8296000	109.0199966
-5.8107195	0.8784000	106.2699966
-5.8107195	0.9272000	105.8199997
-5.8107195	0.9760000	105.3899994
-5.8107195	1.0247999	106.1999969
-5.8107195	1.0735999	104.2900009
-5.8107195	1.1223999	104.2099991
-5.8107195	1.1711999	104.3199997
-5.8107195	1.2200000	102.3300018
-5.8107195	1.2688000	101.5599976
-5.8107195	1.3176000	103.2500000
-5.8107195	1.3664000	106.3899994
-5.8107195	1.4152000	107.6100006
-5.8107195	1.4640000	104.9700012
-5.8107195	1.5128000	107.1699982
-5.8107195	1.5616000	107.5599976
-5.8107195	1.6104000	105.4899979
-5.8107195	1.6592000	106.2200012
-5.8107195	1.7079999	108.7999954
-5.8107195	1.7567999	108.8399963
-5.8107195	1.8055999	104.2500000
-5.8107195	1.8543999	112.9000015
-5.8107195	1.9031999	113.5699997
-5.8107195	1.9519999	112.5400009
-5.8107195	2.0007999	107.3499985
-5.8107195	2.0495999	106.5499954
-5.8107195	2.0983999	105.1399994
-5.8107195	2.1471999	103.3799973
-5.8107195	2.1959999	102.9300003
-5.8107195	2.2447999	102.5400009
-5.8107195	2.2935998	104.2399979
-5.8107195	2.3423998	102.2500000
-5.8107195	2.3912001	103.6299973
-5.8107195	2.4400001	106.0000000
-5.8107195	2.4888000	106.1699982
-5.8107195	2.5376000	99.1199951
-5.8107195	2.5864000	103.0299988
-5.8107195	2.6352000	106.2999954
-5.8107195	2.6840000	104.3799973
-5.8107195	2.7328000	107.1999969
-5.8107195	2.7816000	104.5999985
-5.8107195	2.8304000	103.4799957
-5.8107195	2.8792000	104.1599960
-5.8107195	2.9280000	104.3300018
-5.8107195	2.9768000	105.3499985
-5.8107195	3.0256000	105.6299973
-5.8107195	3.0743999	104.4799957
-5.8107195	3.1231999	105.4399948
-5.8107195	3.1719999	108.6699982
-5.8107195	3.2207999	108.7299957
-5.8107195	3.2695999	102.2399979
-5.8107195	3.3183999	99.3300018

-5.8107195	3.3671999	100.9199982
-5.8107195	3.4159999	104.4199982
-5.8107195	3.4647999	104.6599960
-5.8107195	3.5135999	103.5000000
-5.8107195	3.5623999	105.2799988
-5.8107195	3.6111999	104.4099960
-5.8107195	3.6599998	104.3799973
-5.8107195	3.7087998	106.4799957
-5.8107195	3.7575998	103.9000015
-5.8107195	3.8063998	101.6999969
-5.8107195	3.8551998	103.8799973
-5.8107195	3.9039998	105.5800018
-5.8107195	3.9527998	105.4899979
-5.8107195	4.0016003	103.3399963
-5.8107195	4.0503998	100.9099960
-5.8107195	4.0991998	102.7399979
-5.8107195	4.1479998	107.7399979
-5.8107195	4.1967998	107.7399979
-5.8107195	4.2455997	106.8799973
-5.8107195	4.2943997	106.5699997
-5.8107195	4.3431997	106.3600006
-5.8107195	4.3919997	104.8499985
-5.8107195	4.4407997	104.4499969
-5.8107195	4.4895997	107.2600021
-5.8107195	4.5383997	111.0100021
-5.8107195	4.5871997	111.7699966
-5.8107195	4.6359997	111.7500000
-5.8107195	4.6847997	111.7200012
-5.8107195	4.7336001	113.6100006
-5.8107195	4.7824001	110.5499954
-5.8107195	4.8312001	109.6999969
-5.8107195	4.8800001	111.3099976
-5.8107195	4.9288001	109.4700012
-5.8107195	4.9776001	102.9700012
-5.8107195	5.0264001	103.6199951
-5.8107195	5.0752001	105.9899979
-5.8107195	5.1240001	105.7399979
-5.8107195	5.1728001	102.5899963
-5.8107195	5.2216001	103.0100021
-5.8107195	5.2704000	103.7399979
-5.8107195	5.3192000	103.5899963
-5.8107195	5.3680000	104.7999954
-5.8107195	5.4168000	106.4700012
-5.8107195	5.4656000	105.5199966
-5.8107195	5.5144000	104.3300018
-5.8107195	5.5632000	102.0899963
-5.8107195	5.6120000	105.7699966
-5.8107195	5.6608000	101.5000000
-5.8107195	5.7096000	102.2600021
-5.8107195	5.7584000	105.9499969
-5.8107195	5.8072000	105.9499969
-5.8107195	5.8559999	107.6800003
-5.8107195	5.9047999	107.7900009
-5.8107195	5.9535999	106.1399994
-5.8107195	6.0023999	106.1199951
-5.8107195	6.0511999	103.1299973
-5.8107195	6.0999999	102.9199982
-5.8107195	6.1487999	102.7799988

-5.8107195	6.1975999	103.8399963
-5.8107195	6.2463999	106.6800003
-5.8107195	6.2951999	106.1500015
-5.8107195	6.3439999	103.9399948
-5.8107195	6.3927999	103.5499954
-5.8107195	6.4415998	103.1199951
-5.8107195	6.4903998	103.9499969
-5.8107195	6.5391998	105.9399948
-5.8107195	6.5879998	103.6500015
-5.8107195	6.6367998	106.8699951
-5.8107195	6.6855998	106.5800018
-5.8107195	6.7343998	107.3399963
-5.8107195	6.7831998	105.1699982
-5.8107195	6.8319998	102.5800018
-5.8107195	6.8807998	104.6299973
-5.8107195	6.9295998	102.7399979
-5.8107195	6.9783998	104.2600021
-5.8107195	7.0271997	103.4700012
-5.8107195	7.0759997	99.5599976
-5.8107195	7.1247997	101.8499985
-5.8107195	7.1735997	103.6999969
-5.8107195	7.2223997	98.4899979
-5.8107195	7.2711997	100.9700012
-5.8107195	7.3199997	103.2099991
-5.8107195	7.3687997	100.3099976
-5.8107195	7.4175997	103.5800018
-5.8107195	7.4663997	106.1100006
-5.8107195	7.5151997	103.4499969
-5.8107195	7.5639997	102.5599976
-5.8107195	7.6127996	103.2099991
-5.8107195	7.6615996	104.3300018
-5.8107195	7.7103996	103.6999969
-5.8107195	7.7591996	100.6999969
-5.8107195	7.8079996	102.6999969
-5.8107195	7.8567996	102.6399994
-5.8107195	7.9056001	100.0499954
-5.8107195	7.9544001	100.0699997
-5.8107195	8.0032005	97.5299988
-5.8107195	8.0520000	102.1399994
-5.8107195	8.1008005	103.9700012
-5.8107195	8.1496000	102.4799957
-5.8107195	8.1984005	101.6599960
-5.8107195	8.2472000	104.6299973
-5.8107195	8.2960005	103.6999969
-5.8107195	8.3448000	104.8699951
-5.8107195	8.3936005	106.1699982
-5.8107195	8.4424000	104.9599991
-5.8107195	8.4912004	107.5199966
-5.8107195	8.5400000	105.6800003
-5.8107195	8.5888004	101.5599976
-5.8107195	8.6375999	99.4599991
-5.8107195	8.6864004	100.7600021
-5.8107195	8.7351999	103.5000000
-5.8107195	8.7840004	106.3699951
-5.8107195	8.8327999	102.2699966
-5.8107195	8.8816004	102.2600021
-5.8107195	8.9303999	105.0899963
-5.8107195	8.9792004	103.7200012

-5.8107195	9.0279999	101.5800018
-5.8107195	9.0768003	103.1899948
-5.8107195	9.1255999	103.3199997
-5.8107195	9.1744003	103.7600021
-5.8107195	9.2231998	104.8899994
-5.8107195	9.2720003	103.6199951
-5.8107195	9.3207998	104.2099991
-5.8107195	9.3696003	102.7699966
-5.8107195	9.4184008	100.8899994
-5.8107195	9.4672003	98.5100021
-5.8107195	9.5160007	100.5699997
-5.8107195	9.5648003	101.4300003
-5.8107195	9.6136007	104.2699966
-5.8107195	9.6624002	104.5499954
-5.8107195	9.7112007	105.5299988
-5.8107195	9.7600002	101.4799957
-5.8107195	9.8088007	103.8099976
-5.8107195	9.8576002	102.0999985
-5.8107195	9.9064007	104.5499954
-5.8107195	9.9552002	105.0599976
-5.8107195	10.0040007	104.1699982
-5.8107195	10.0528002	101.4099960
-5.8107195	10.1016006	104.7999954
-5.8107195	10.1504002	103.5999985
-5.8107195	10.1992006	104.9799957
-5.8107195	10.2480001	105.6199951
-5.8107195	10.2968006	103.9599991
-5.8107195	10.3456001	99.7500000
-5.8107195	10.3944006	100.8199997
-5.8107195	10.4432001	102.6800003
-5.8107195	10.4920006	105.5000000
-5.8107195	10.5408001	103.6599960
-5.8107195	10.5896006	105.9199982
-5.8107195	10.6384001	103.5899963
-5.8107195	10.6872005	100.0999985
-5.8107195	10.7360001	104.5000000
-5.8107195	10.7848005	104.0400009
-5.8107195	10.8336000	104.1999969
-5.8107195	10.8824005	103.7299957
-5.8107195	10.9312000	106.2099991
-5.8107195	10.9800005	105.9399948
-5.8107195	11.0288000	104.4499969
-5.8107195	11.0776005	103.7699966
-5.8107195	11.1264000	100.4599991
-5.8107195	11.1752005	103.7600021
-5.8107195	11.2240000	105.9300003
-5.8107195	11.2728004	106.5599976
-5.8107195	11.3216000	102.3699951
-5.8107195	11.3704004	104.1399994
-5.8107195	11.4191999	103.7600021
-5.8107195	11.4680004	103.7099991
-5.8107195	11.5167999	103.1100006
-5.8107195	11.5656004	103.4000015
-5.8107195	11.6143999	103.1899948
-5.8107195	11.6632004	101.0999985
-5.8107195	11.7119999	98.6100006
-5.8107195	11.7608004	102.2699966
-5.8107195	11.8095999	103.7099991

-5.8107195	11.8584003	104.9799957
-5.8107195	11.9071999	104.7799988
-5.8107195	11.9560003	104.6299973
-5.8107195	12.0047998	103.8199997
-5.8107195	12.0536003	102.6500015
-5.8107195	12.1023998	99.8699951
-5.8107195	12.1512003	101.2500000
-5.8107195	12.1999998	102.5999985
-5.8107195	12.2488003	104.8300018
-5.8107195	12.2975998	104.2099991
-5.8107195	12.3464003	102.8199997
-5.8107195	12.3951998	106.3899994
-5.8107195	12.4440002	106.1299973
-5.8107195	12.4927998	104.9000015
-5.8107195	12.5416002	102.7299957
-5.8107195	12.5904007	104.2699966
-5.8107195	12.6392002	103.5499954
-5.8107195	12.6880007	103.9799957
-5.8107195	12.7368002	103.2900009
-5.8107195	12.7856007	104.5299988
-5.8107195	12.8344002	106.1699982
-5.8107195	12.8832006	105.9599991
-5.8107195	12.9320002	103.6800003
-5.8107195	12.9808006	104.4599991
-5.8107195	13.0296001	102.6699982
-5.8107195	13.0784006	101.0100021
-5.8107195	13.1272001	103.5100021
-5.8107195	13.1760006	104.2900009
-5.8107195	13.2248001	101.6100006
-5.8107195	13.2736006	102.4899979
-5.8107195	13.3224001	100.6699982
-5.8107195	13.3712006	102.8399963
-5.8107195	13.4200001	104.7200012
-5.8107195	13.4688005	100.3399963
-5.8107195	13.5176001	104.3600006
-5.8107195	13.5664005	105.5599976
-5.8107195	13.6152000	102.0800018
-5.8107195	13.6640005	104.6100006
-5.8107195	13.7128000	104.4899979
-5.8107195	13.7616005	105.0000000
-5.8107195	13.8104000	104.0400009
-5.8107195	13.8592005	104.3699951
-5.8107195	13.9080000	101.9499969
-5.8107195	13.9568005	100.6699982
-5.8107195	14.0056000	100.8099976
-5.8107195	14.0544004	98.0100021
-5.8107195	14.1032000	102.3600006
-5.8107195	14.1520004	99.8199997
-5.8107195	14.2007999	101.3099976
-5.8107195	14.2496004	100.2900009
-5.8107195	14.2983999	103.9300003
-5.8107195	14.3472004	102.9300003
-5.8107195	14.3959999	101.7699966
-5.8107195	14.4448004	100.2500000
-5.8107195	14.4935999	103.4799957
-5.8107195	14.5424004	104.5400009
-5.8107195	14.5911999	100.6199951
-5.8107195	14.6400003	105.2500000

-5.8107195	14.6887999	104.1199951
-5.8107195	14.7376003	104.1599960
-5.8107195	14.7863998	104.3899994
-5.8107195	14.8352003	104.9599991
-5.8107195	14.8839998	102.4700012
-5.8107195	14.9328003	101.5100021
-5.8107195	14.9815998	100.6699982
-5.8107195	15.0304003	96.1999969
-5.8045835	0.3416000	105.7500000
-5.8045835	0.3904000	106.6399994
-5.8045835	0.4392000	108.4499969
-5.8045835	0.4880000	106.3499985
-5.8045835	0.5368000	102.0899963
-5.8045835	0.5856000	103.8699951
-5.8045835	0.6344000	107.3300018
-5.8045835	0.6832000	109.5199966
-5.8045835	0.7320000	110.0999985
-5.8045835	0.7808000	109.2399979
-5.8045835	0.8296000	109.1500015
-5.8045835	0.8784000	106.0499954
-5.8045835	0.9272000	106.1899948
-5.8045835	0.9760000	105.9899979
-5.8045835	1.0247999	106.6999969
-5.8045835	1.0735999	104.6800003
-5.8045835	1.1223999	105.3600006
-5.8045835	1.1711999	104.8399963
-5.8045835	1.2200000	102.3799973
-5.8045835	1.2688000	101.4399948
-5.8045835	1.3176000	104.0100021
-5.8045835	1.3664000	106.9599991
-5.8045835	1.4152000	107.8899994
-5.8045835	1.4640000	104.3300018
-5.8045835	1.5128000	106.9199982
-5.8045835	1.5616000	107.5999985
-5.8045835	1.6104000	106.3300018
-5.8045835	1.6592000	106.6500015
-5.8045835	1.7079999	108.6500015
-5.8045835	1.7567999	108.7999954
-5.8045835	1.8055999	105.1999969
-5.8045835	1.8543999	112.9099960
-5.8045835	1.9031999	113.6299973
-5.8045835	1.9519999	112.1100006
-5.8045835	2.0007999	107.4700012
-5.8045835	2.0495999	106.0100021
-5.8045835	2.0983999	104.7399979
-5.8045835	2.1471999	103.4399948
-5.8045835	2.1959999	103.2200012
-5.8045835	2.2447999	102.4000015
-5.8045835	2.2935998	104.4700012
-5.8045835	2.3423998	102.4000015
-5.8045835	2.3912001	104.5000000
-5.8045835	2.4400001	106.4000015
-5.8045835	2.4888000	105.9700012
-5.8045835	2.5376000	97.5199966
-5.8045835	2.5864000	102.9099960
-5.8045835	2.6352000	105.4399948
-5.8045835	2.6840000	103.0499954
-5.8045835	2.7328000	106.6899948

-5.8045835	2.7816000	104.4799957
-5.8045835	2.8304000	103.1800003
-5.8045835	2.8792000	104.7600021
-5.8045835	2.9280000	104.3399963
-5.8045835	2.9768000	105.7299957
-5.8045835	3.0256000	107.5699997
-5.8045835	3.0743999	105.8600006
-5.8045835	3.1231999	105.9499969
-5.8045835	3.1719999	108.5599976
-5.8045835	3.2207999	108.1999969
-5.8045835	3.2695999	103.0999985
-5.8045835	3.3183999	98.0000000
-5.8045835	3.3671999	102.3799973
-5.8045835	3.4159999	104.1199951
-5.8045835	3.4647999	104.6199951
-5.8045835	3.5135999	103.9099960
-5.8045835	3.5623999	105.8099976
-5.8045835	3.6111999	104.3399963
-5.8045835	3.6599998	103.9099960
-5.8045835	3.7087998	106.3600006
-5.8045835	3.7575998	104.1599960
-5.8045835	3.8063998	101.7699966
-5.8045835	3.8551998	103.7200012
-5.8045835	3.9039998	104.7799988
-5.8045835	3.9527998	104.1999969
-5.8045835	4.0016003	102.9199982
-5.8045835	4.0503998	99.4399948
-5.8045835	4.0991998	102.0699997
-5.8045835	4.1479998	107.0699997
-5.8045835	4.1967998	106.9899979
-5.8045835	4.2455997	106.3600006
-5.8045835	4.2943997	106.7600021
-5.8045835	4.3431997	107.1599960
-5.8045835	4.3919997	103.8499985
-5.8045835	4.4407997	104.2200012
-5.8045835	4.4895997	107.0199966
-5.8045835	4.5383997	110.8300018
-5.8045835	4.5871997	111.4300003
-5.8045835	4.6359997	111.2200012
-5.8045835	4.6847997	110.5599976
-5.8045835	4.7336001	112.9199982
-5.8045835	4.7824001	110.6500015
-5.8045835	4.8312001	109.9300003
-5.8045835	4.8800001	111.6699982
-5.8045835	4.9288001	109.7699966
-5.8045835	4.9776001	102.9499969
-5.8045835	5.0264001	102.9199982
-5.8045835	5.0752001	105.8399963
-5.8045835	5.1240001	106.4899979
-5.8045835	5.1728001	104.0000000
-5.8045835	5.2216001	104.1599960
-5.8045835	5.2704000	104.9399948
-5.8045835	5.3192000	102.9799957
-5.8045835	5.3680000	104.0400009
-5.8045835	5.4168000	105.7600021
-5.8045835	5.4656000	104.5999985
-5.8045835	5.5144000	104.4199982
-5.8045835	5.5632000	104.1299973

-5.8045835	5.6120000	107.0199966
-5.8045835	5.6608000	102.7099991
-5.8045835	5.7096000	103.8699951
-5.8045835	5.7584000	106.1299973
-5.8045835	5.8072000	104.7099991
-5.8045835	5.8559999	107.0499954
-5.8045835	5.9047999	107.3799973
-5.8045835	5.9535999	106.1500015
-5.8045835	6.0023999	105.5000000
-5.8045835	6.0511999	102.0899963
-5.8045835	6.0999999	102.3799973
-5.8045835	6.1487999	103.5800018
-5.8045835	6.1975999	104.5599976
-5.8045835	6.2463999	106.6699982
-5.8045835	6.2951999	105.9499969
-5.8045835	6.3439999	103.2399979
-5.8045835	6.3927999	102.1599960
-5.8045835	6.4415998	102.8899994
-5.8045835	6.4903998	104.2999954
-5.8045835	6.5391998	106.5299988
-5.8045835	6.5879998	104.9099960
-5.8045835	6.6367998	106.5100021
-5.8045835	6.6855998	106.0899963
-5.8045835	6.7343998	106.7999954
-5.8045835	6.7831998	103.9899979
-5.8045835	6.8319998	102.2399979
-5.8045835	6.8807998	105.3199997
-5.8045835	6.9295998	103.1999969
-5.8045835	6.9783998	105.2699966
-5.8045835	7.0271997	104.1299973
-5.8045835	7.0759997	99.6999969
-5.8045835	7.1247997	102.6100006
-5.8045835	7.1735997	103.5899963
-5.8045835	7.2223997	99.1299973
-5.8045835	7.2711997	101.9599991
-5.8045835	7.3199997	102.6899948
-5.8045835	7.3687997	99.9000015
-5.8045835	7.4175997	104.0699997
-5.8045835	7.4663997	105.9899979
-5.8045835	7.5151997	103.1899948
-5.8045835	7.5639997	101.3699951
-5.8045835	7.6127996	102.1299973
-5.8045835	7.6615996	104.0000000
-5.8045835	7.7103996	103.8199997
-5.8045835	7.7591996	101.8799973
-5.8045835	7.8079996	103.4199982
-5.8045835	7.8567996	103.1699982
-5.8045835	7.9056001	100.9000015
-5.8045835	7.9544001	100.4799957
-5.8045835	8.0032005	98.2200012
-5.8045835	8.0520000	102.8600006
-5.8045835	8.1008005	104.7299957
-5.8045835	8.1496000	103.5299988
-5.8045835	8.1984005	102.1999969
-5.8045835	8.2472000	104.7999954
-5.8045835	8.2960005	103.2099991
-5.8045835	8.3448000	104.3300018
-5.8045835	8.3936005	105.8199997

-5.8045835	8.4424000	103.3300018
-5.8045835	8.4912004	106.6599960
-5.8045835	8.5400000	105.6800003
-5.8045835	8.5888004	101.2399979
-5.8045835	8.6375999	98.7999954
-5.8045835	8.6864004	101.0800018
-5.8045835	8.7351999	104.6299973
-5.8045835	8.7840004	106.8099976
-5.8045835	8.8327999	103.0299988
-5.8045835	8.8816004	102.9399948
-5.8045835	8.9303999	104.8499985
-5.8045835	8.9792004	103.1299973
-5.8045835	9.0279999	101.0899963
-5.8045835	9.0768003	102.7999954
-5.8045835	9.1255999	102.4799957
-5.8045835	9.1744003	102.8600006
-5.8045835	9.2231998	103.9599991
-5.8045835	9.2720003	102.4300003
-5.8045835	9.3207998	104.0899963
-5.8045835	9.3696003	102.7900009
-5.8045835	9.4184008	101.2900009
-5.8045835	9.4672003	98.3300018
-5.8045835	9.5160007	100.6399994
-5.8045835	9.5648003	102.0499954
-5.8045835	9.6136007	103.6699982
-5.8045835	9.6624002	104.1699982
-5.8045835	9.7112007	105.6800003
-5.8045835	9.7600002	103.0599976
-5.8045835	9.8088007	104.0899963
-5.8045835	9.8576002	101.4399948
-5.8045835	9.9064007	104.1800003
-5.8045835	9.9552002	104.8899994
-5.8045835	10.0040007	103.7900009
-5.8045835	10.0528002	103.1800003
-5.8045835	10.1016006	105.1699982
-5.8045835	10.1504002	103.6699982
-5.8045835	10.1992006	105.5599976
-5.8045835	10.2480001	105.4799957
-5.8045835	10.2968006	103.5100021
-5.8045835	10.3456001	100.1399994
-5.8045835	10.3944006	100.8699951
-5.8045835	10.4432001	102.0800018
-5.8045835	10.4920006	105.8899994
-5.8045835	10.5408001	105.2600021
-5.8045835	10.5896006	106.0800018
-5.8045835	10.6384001	103.5899963
-5.8045835	10.6872005	99.4700012
-5.8045835	10.7360001	104.4300003
-5.8045835	10.7848005	104.0400009
-5.8045835	10.8336000	104.2999954
-5.8045835	10.8824005	104.3300018
-5.8045835	10.9312000	106.2299957
-5.8045835	10.9800005	106.3799973
-5.8045835	11.0288000	105.1100006
-5.8045835	11.0776005	103.7099991
-5.8045835	11.1264000	99.6599960
-5.8045835	11.1752005	103.3099976
-5.8045835	11.2240000	105.3899994

-5.8045835	11.2728004	107.3099976
-5.8045835	11.3216000	104.5100021
-5.8045835	11.3704004	105.2600021
-5.8045835	11.4191999	103.8399963
-5.8045835	11.4680004	103.4499969
-5.8045835	11.5167999	103.0800018
-5.8045835	11.5656004	104.1999969
-5.8045835	11.6143999	104.0800018
-5.8045835	11.6632004	101.9599991
-5.8045835	11.7119999	98.8399963
-5.8045835	11.7608004	102.6999969
-5.8045835	11.8095999	103.9499969
-5.8045835	11.8584003	105.2600021
-5.8045835	11.9071999	104.9099960
-5.8045835	11.9560003	103.9000015
-5.8045835	12.0047998	104.3399963
-5.8045835	12.0536003	103.2299957
-5.8045835	12.1023998	99.9300003
-5.8045835	12.1512003	100.3899994
-5.8045835	12.1999998	103.1399994
-5.8045835	12.2488003	104.5899963
-5.8045835	12.2975998	103.9199982
-5.8045835	12.3464003	102.0100021
-5.8045835	12.3951998	105.8300018
-5.8045835	12.4440002	106.1800003
-5.8045835	12.4927998	104.4000015
-5.8045835	12.5416002	101.9499969
-5.8045835	12.5904007	103.6899948
-5.8045835	12.6392002	103.3899994
-5.8045835	12.6880007	103.6100006
-5.8045835	12.7368002	103.2099991
-5.8045835	12.7856007	104.2999954
-5.8045835	12.8344002	106.2200012
-5.8045835	12.8832006	105.8600006
-5.8045835	12.9320002	103.5199966
-5.8045835	12.9808006	104.0000000
-5.8045835	13.0296001	101.9199982
-5.8045835	13.0784006	101.4700012
-5.8045835	13.1272001	103.4599991
-5.8045835	13.1760006	104.3199997
-5.8045835	13.2248001	101.2200012
-5.8045835	13.2736006	102.3199997
-5.8045835	13.3224001	100.9099960
-5.8045835	13.3712006	102.8699951
-5.8045835	13.4200001	104.8300018
-5.8045835	13.4688005	102.1899948
-5.8045835	13.5176001	104.1100006
-5.8045835	13.5664005	105.4300003
-5.8045835	13.6152000	101.2500000
-5.8045835	13.6640005	104.2299957
-5.8045835	13.7128000	104.7200012
-5.8045835	13.7616005	105.1100006
-5.8045835	13.8104000	103.9499969
-5.8045835	13.8592005	104.1399994
-5.8045835	13.9080000	101.1999969
-5.8045835	13.9568005	100.2799988
-5.8045835	14.0056000	100.5999985
-5.8045835	14.0544004	96.7099991

-5.8045835	14.1032000	101.8899994
-5.8045835	14.1520004	98.5999985
-5.8045835	14.2007999	101.2299957
-5.8045835	14.2496004	101.2900009
-5.8045835	14.2983999	103.9000015
-5.8045835	14.3472004	101.7999954
-5.8045835	14.3959999	100.8399963
-5.8045835	14.4448004	99.1699982
-5.8045835	14.4935999	102.9199982
-5.8045835	14.5424004	104.7500000
-5.8045835	14.5911999	101.0599976
-5.8045835	14.6400003	105.0999985
-5.8045835	14.6887999	104.6800003
-5.8045835	14.7376003	103.7500000
-5.8045835	14.7863998	104.4000015
-5.8045835	14.8352003	105.9700012
-5.8045835	14.8839998	103.5699997
-5.8045835	14.9328003	102.5899963
-5.8045835	14.9815998	101.1399994
-5.8045835	15.0304003	96.9099960
-5.7984476	0.3416000	104.5299988
-5.7984476	0.3904000	105.7399979
-5.7984476	0.4392000	108.4300003
-5.7984476	0.4880000	106.1299973
-5.7984476	0.5368000	101.6100006
-5.7984476	0.5856000	104.7200012
-5.7984476	0.6344000	108.2600021
-5.7984476	0.6832000	110.0199966
-5.7984476	0.7320000	110.1999969
-5.7984476	0.7808000	109.4700012
-5.7984476	0.8296000	109.2600021
-5.7984476	0.8784000	106.6599960
-5.7984476	0.9272000	107.0599976
-5.7984476	0.9760000	107.3099976
-5.7984476	1.0247999	107.9199982
-5.7984476	1.0735999	106.1500015
-5.7984476	1.1223999	106.2500000
-5.7984476	1.1711999	105.2099991
-5.7984476	1.2200000	102.9300003
-5.7984476	1.2688000	102.5100021
-5.7984476	1.3176000	105.4199982
-5.7984476	1.3664000	107.6100006
-5.7984476	1.4152000	107.8899994
-5.7984476	1.4640000	103.8399963
-5.7984476	1.5128000	106.6299973
-5.7984476	1.5616000	106.7299957
-5.7984476	1.6104000	106.6899948
-5.7984476	1.6592000	106.6999969
-5.7984476	1.7079999	108.1100006
-5.7984476	1.7567999	108.2500000
-5.7984476	1.8055999	105.5499954
-5.7984476	1.8543999	112.6999969
-5.7984476	1.9031999	113.4799957
-5.7984476	1.9519999	111.5400009
-5.7984476	2.0007999	107.4099960
-5.7984476	2.0495999	105.3199997
-5.7984476	2.0983999	104.7799988
-5.7984476	2.1471999	104.4000015

-5.7984476	2.1959999	103.9300003
-5.7984476	2.2447999	103.0400009
-5.7984476	2.2935998	104.7500000
-5.7984476	2.3423998	102.9499969
-5.7984476	2.3912001	105.1500015
-5.7984476	2.4400001	106.5000000
-5.7984476	2.4888000	105.8600006
-5.7984476	2.5376000	98.6299973
-5.7984476	2.5864000	103.0199966
-5.7984476	2.6352000	104.3399963
-5.7984476	2.6840000	102.6399994
-5.7984476	2.7328000	105.9399948
-5.7984476	2.7816000	103.8799973
-5.7984476	2.8304000	102.3699951
-5.7984476	2.8792000	104.9499969
-5.7984476	2.9280000	104.1299973
-5.7984476	2.9768000	105.6699982
-5.7984476	3.0256000	108.7099991
-5.7984476	3.0743999	106.9099960
-5.7984476	3.1231999	106.0199966
-5.7984476	3.1719999	107.8899994
-5.7984476	3.2207999	107.1599960
-5.7984476	3.2695999	103.5699997
-5.7984476	3.3183999	96.7799988
-5.7984476	3.3671999	103.9799957
-5.7984476	3.4159999	103.6199951
-5.7984476	3.4647999	104.2600021
-5.7984476	3.5135999	104.0100021
-5.7984476	3.5623999	105.7099991
-5.7984476	3.6111999	103.5499954
-5.7984476	3.6599998	102.8399963
-5.7984476	3.7087998	105.9000015
-5.7984476	3.7575998	104.5100021
-5.7984476	3.8063998	102.0400009
-5.7984476	3.8551998	103.1100006
-5.7984476	3.9039998	103.7099991
-5.7984476	3.9527998	102.7900009
-5.7984476	4.0016003	102.4199982
-5.7984476	4.0503998	97.8399963
-5.7984476	4.0991998	102.0299988
-5.7984476	4.1479998	106.5199966
-5.7984476	4.1967998	105.9199982
-5.7984476	4.2455997	105.3399963
-5.7984476	4.2943997	106.9700012
-5.7984476	4.3431997	108.7799988
-5.7984476	4.3919997	103.3399963
-5.7984476	4.4407997	103.6800003
-5.7984476	4.4895997	106.5899963
-5.7984476	4.5383997	110.6999969
-5.7984476	4.5871997	111.1999969
-5.7984476	4.6359997	110.9799957
-5.7984476	4.6847997	110.9099960
-5.7984476	4.7336001	112.3799973
-5.7984476	4.7824001	110.4499969
-5.7984476	4.8312001	109.9799957
-5.7984476	4.8800001	111.6599960
-5.7984476	4.9288001	109.6699982
-5.7984476	4.9776001	103.5199966

-5.7984476	5.0264001	102.5400009
-5.7984476	5.0752001	105.7699966
-5.7984476	5.1240001	106.9300003
-5.7984476	5.1728001	104.7500000
-5.7984476	5.2216001	105.0800018
-5.7984476	5.2704000	105.9799957
-5.7984476	5.3192000	102.2699966
-5.7984476	5.3680000	103.4899979
-5.7984476	5.4168000	105.5800018
-5.7984476	5.4656000	104.9599991
-5.7984476	5.5144000	105.4599991
-5.7984476	5.5632000	105.9499969
-5.7984476	5.6120000	107.2999954
-5.7984476	5.6608000	103.3499985
-5.7984476	5.7096000	105.4700012
-5.7984476	5.7584000	105.7799988
-5.7984476	5.8072000	104.2200012
-5.7984476	5.8559999	106.2999954
-5.7984476	5.9047999	106.7200012
-5.7984476	5.9535999	106.1100006
-5.7984476	6.0023999	105.4099960
-5.7984476	6.0511999	102.2799988
-5.7984476	6.0999999	102.2299957
-5.7984476	6.1487999	104.6100006
-5.7984476	6.1975999	105.0999985
-5.7984476	6.2463999	106.3499985
-5.7984476	6.2951999	105.7699966
-5.7984476	6.3439999	103.6100006
-5.7984476	6.3927999	101.3699951
-5.7984476	6.4415998	103.1100006
-5.7984476	6.4903998	105.2699966
-5.7984476	6.5391998	107.2600021
-5.7984476	6.5879998	105.7399979
-5.7984476	6.6367998	106.1800003
-5.7984476	6.6855998	106.1100006
-5.7984476	6.7343998	106.5199966
-5.7984476	6.7831998	103.3799973
-5.7984476	6.8319998	102.7799988
-5.7984476	6.8807998	105.8399963
-5.7984476	6.9295998	103.0499954
-5.7984476	6.9783998	105.8399963
-5.7984476	7.0271997	104.3899994
-5.7984476	7.0759997	99.9799957
-5.7984476	7.1247997	102.9599991
-5.7984476	7.1735997	103.3099976
-5.7984476	7.2223997	100.4099960
-5.7984476	7.2711997	103.1399994
-5.7984476	7.3199997	102.6699982
-5.7984476	7.3687997	99.1800003
-5.7984476	7.4175997	104.0599976
-5.7984476	7.4663997	106.1599960
-5.7984476	7.5151997	103.7200012
-5.7984476	7.5639997	100.6899948
-5.7984476	7.6127996	101.8099976
-5.7984476	7.6615996	104.1699982
-5.7984476	7.7103996	103.9300003
-5.7984476	7.7591996	102.8799973
-5.7984476	7.8079996	104.0899963

-5.7984476	7.8567996	103.3699951
-5.7984476	7.9056001	101.7200012
-5.7984476	7.9544001	101.0499954
-5.7984476	8.0032005	99.4599991
-5.7984476	8.0520000	103.3699951
-5.7984476	8.1008005	104.5599976
-5.7984476	8.1496000	104.0599976
-5.7984476	8.1984005	103.1100006
-5.7984476	8.2472000	106.0400009
-5.7984476	8.2960005	103.7900009
-5.7984476	8.3448000	103.7699966
-5.7984476	8.3936005	105.0899963
-5.7984476	8.4424000	101.8899994
-5.7984476	8.4912004	105.9000015
-5.7984476	8.5400000	105.5199966
-5.7984476	8.5888004	101.3099976
-5.7984476	8.6375999	98.4399948
-5.7984476	8.6864004	100.7600021
-5.7984476	8.7351999	105.6599960
-5.7984476	8.7840004	106.6599960
-5.7984476	8.8327999	103.4000015
-5.7984476	8.8816004	103.3899994
-5.7984476	8.9303999	104.5299988
-5.7984476	8.9792004	102.5999985
-5.7984476	9.0279999	101.4499969
-5.7984476	9.0768003	102.9099960
-5.7984476	9.1255999	102.3300018
-5.7984476	9.1744003	102.6100006
-5.7984476	9.2231998	103.2399979
-5.7984476	9.2720003	101.4099960
-5.7984476	9.3207998	104.4199982
-5.7984476	9.3696003	102.9099960
-5.7984476	9.4184008	101.4700012
-5.7984476	9.4672003	98.1500015
-5.7984476	9.5160007	100.2099991
-5.7984476	9.5648003	103.0599976
-5.7984476	9.6136007	103.7600021
-5.7984476	9.6624002	104.1599960
-5.7984476	9.7112007	105.5699997
-5.7984476	9.7600002	104.2699966
-5.7984476	9.8088007	104.4000015
-5.7984476	9.8576002	101.1899948
-5.7984476	9.9064007	103.4499969
-5.7984476	9.9552002	104.7600021
-5.7984476	10.0040007	103.2699966
-5.7984476	10.0528002	104.2200012
-5.7984476	10.1016006	105.2500000
-5.7984476	10.1504002	103.4499969
-5.7984476	10.1992006	105.8499985
-5.7984476	10.2480001	105.5999985
-5.7984476	10.2968006	103.1800003
-5.7984476	10.3456001	100.4300003
-5.7984476	10.3944006	101.5199966
-5.7984476	10.4432001	101.9300003
-5.7984476	10.4920006	105.5000000
-5.7984476	10.5408001	106.1399994
-5.7984476	10.5896006	106.1599960
-5.7984476	10.6384001	103.9700012

-5.7984476	10.6872005	100.8799973
-5.7984476	10.7360001	104.6899948
-5.7984476	10.7848005	104.3499985
-5.7984476	10.8336000	104.4300003
-5.7984476	10.8824005	104.1599960
-5.7984476	10.9312000	105.6199951
-5.7984476	10.9800005	106.0599976
-5.7984476	11.0288000	105.2999954
-5.7984476	11.0776005	103.9599991
-5.7984476	11.1264000	100.1199951
-5.7984476	11.1752005	102.5499954
-5.7984476	11.2240000	104.2900009
-5.7984476	11.2728004	107.2099991
-5.7984476	11.3216000	105.6599960
-5.7984476	11.3704004	105.6999969
-5.7984476	11.4191999	103.3600006
-5.7984476	11.4680004	102.8300018
-5.7984476	11.5167999	102.6599960
-5.7984476	11.5656004	104.8300018
-5.7984476	11.6143999	105.0699997
-5.7984476	11.6632004	102.9700012
-5.7984476	11.7119999	99.7500000
-5.7984476	11.7608004	102.5100021
-5.7984476	11.8095999	103.6500015
-5.7984476	11.8584003	105.1199951
-5.7984476	11.9071999	105.2500000
-5.7984476	11.9560003	103.2200012
-5.7984476	12.0047998	104.8499985
-5.7984476	12.0536003	104.0699997
-5.7984476	12.1023998	101.1299973
-5.7984476	12.1512003	100.0199966
-5.7984476	12.1999998	103.9199982
-5.7984476	12.2488003	104.6800003
-5.7984476	12.2975998	103.5100021
-5.7984476	12.3464003	101.0800018
-5.7984476	12.3951998	105.1800003
-5.7984476	12.4440002	106.1399994
-5.7984476	12.4927998	104.3799973
-5.7984476	12.5416002	101.9000015
-5.7984476	12.5904007	103.5000000
-5.7984476	12.6392002	103.4599991
-5.7984476	12.6880007	103.5100021
-5.7984476	12.7368002	103.0599976
-5.7984476	12.7856007	104.0899963
-5.7984476	12.8344002	106.1199951
-5.7984476	12.8832006	105.4199982
-5.7984476	12.9320002	102.4099960
-5.7984476	12.9808006	103.3499985
-5.7984476	13.0296001	101.5999985
-5.7984476	13.0784006	102.7900009
-5.7984476	13.1272001	104.0199966
-5.7984476	13.1760006	104.2200012
-5.7984476	13.2248001	101.0800018
-5.7984476	13.2736006	101.9099960
-5.7984476	13.3224001	100.6500015
-5.7984476	13.3712006	102.1500015
-5.7984476	13.4200001	104.6199951
-5.7984476	13.4688005	103.6599960

-5.7984476	13.5176001	103.9300003
-5.7984476	13.5664005	104.9799957
-5.7984476	13.6152000	100.9000015
-5.7984476	13.6640005	104.4799957
-5.7984476	13.7128000	105.3399963
-5.7984476	13.7616005	105.5100021
-5.7984476	13.8104000	103.5499954
-5.7984476	13.8592005	104.0699997
-5.7984476	13.9080000	101.9599991
-5.7984476	13.9568005	101.0999985
-5.7984476	14.0056000	100.8799973
-5.7984476	14.0544004	96.7799988
-5.7984476	14.1032000	101.5199966
-5.7984476	14.1520004	98.0599976
-5.7984476	14.2007999	100.9499969
-5.7984476	14.2496004	102.0599976
-5.7984476	14.2983999	103.7699966
-5.7984476	14.3472004	100.7299957
-5.7984476	14.3959999	99.4000015
-5.7984476	14.4448004	98.2299957
-5.7984476	14.4935999	102.0100021
-5.7984476	14.5424004	104.1899948
-5.7984476	14.5911999	100.8899994
-5.7984476	14.6400003	104.1800003
-5.7984476	14.6887999	104.6699982
-5.7984476	14.7376003	103.3399963
-5.7984476	14.7863998	104.0599976
-5.7984476	14.8352003	106.2999954
-5.7984476	14.8839998	104.0400009
-5.7984476	14.9328003	103.2200012
-5.7984476	14.9815998	101.8099976
-5.7984476	15.0304003	98.4700012
-5.7923117	0.3416000	103.5800018
-5.7923117	0.3904000	105.1399994
-5.7923117	0.4392000	107.8899994
-5.7923117	0.4880000	105.6999969
-5.7923117	0.5368000	102.7900009
-5.7923117	0.5856000	106.2699966
-5.7923117	0.6344000	108.9300003
-5.7923117	0.6832000	110.0599976
-5.7923117	0.7320000	109.6500015
-5.7923117	0.7808000	107.9000015
-5.7923117	0.8296000	108.9099960
-5.7923117	0.8784000	107.1500015
-5.7923117	0.9272000	107.9700012
-5.7923117	0.9760000	108.6299973
-5.7923117	1.0247999	109.0899963
-5.7923117	1.0735999	107.4000015
-5.7923117	1.1223999	107.2600021
-5.7923117	1.1711999	106.3199997
-5.7923117	1.2200000	104.2699966
-5.7923117	1.2688000	103.4899979
-5.7923117	1.3176000	105.9199982
-5.7923117	1.3664000	107.6599960
-5.7923117	1.4152000	107.8099976
-5.7923117	1.4640000	104.9599991
-5.7923117	1.5128000	106.8199997
-5.7923117	1.5616000	106.2699966

-5.7923117	1.6104000	107.1899948
-5.7923117	1.6592000	106.9099960
-5.7923117	1.7079999	107.4399948
-5.7923117	1.7567999	107.4000015
-5.7923117	1.8055999	106.2600021
-5.7923117	1.8543999	112.1599960
-5.7923117	1.9031999	112.9000015
-5.7923117	1.9519999	110.5999985
-5.7923117	2.0007999	107.3399963
-5.7923117	2.0495999	104.7099991
-5.7923117	2.0983999	104.5400009
-5.7923117	2.1471999	105.0800018
-5.7923117	2.1959999	104.8399963
-5.7923117	2.2447999	104.0899963
-5.7923117	2.2935998	105.1599960
-5.7923117	2.3423998	103.5599976
-5.7923117	2.3912001	105.7999954
-5.7923117	2.4400001	106.4899979
-5.7923117	2.4888000	105.0899963
-5.7923117	2.5376000	99.0100021
-5.7923117	2.5864000	103.3600006
-5.7923117	2.6352000	103.0400009
-5.7923117	2.6840000	101.9899979
-5.7923117	2.7328000	104.8899994
-5.7923117	2.7816000	102.8499985
-5.7923117	2.8304000	101.9300003
-5.7923117	2.8792000	105.2600021
-5.7923117	2.9280000	104.5199966
-5.7923117	2.9768000	104.7600021
-5.7923117	3.0256000	108.8499985
-5.7923117	3.0743999	107.5599976
-5.7923117	3.1231999	106.1699982
-5.7923117	3.1719999	107.2999954
-5.7923117	3.2207999	106.2299957
-5.7923117	3.2695999	104.4599991
-5.7923117	3.3183999	99.4399948
-5.7923117	3.3671999	105.2600021
-5.7923117	3.4159999	103.0499954
-5.7923117	3.4647999	103.4899979
-5.7923117	3.5135999	103.6800003
-5.7923117	3.5623999	105.5699997
-5.7923117	3.6111999	103.0999985
-5.7923117	3.6599998	101.9099960
-5.7923117	3.7087998	105.2999954
-5.7923117	3.7575998	104.5100021
-5.7923117	3.8063998	102.2099991
-5.7923117	3.8551998	102.4899979
-5.7923117	3.9039998	102.8399963
-5.7923117	3.9527998	102.5000000
-5.7923117	4.0016003	103.0599976
-5.7923117	4.0503998	97.5100021
-5.7923117	4.0991998	101.4599991
-5.7923117	4.1479998	106.2099991
-5.7923117	4.1967998	104.9199982
-5.7923117	4.2455997	104.2699966
-5.7923117	4.2943997	106.7799988
-5.7923117	4.3431997	109.2999954
-5.7923117	4.3919997	102.1500015

-5.7923117	4.4407997	103.5499954
-5.7923117	4.4895997	106.3899994
-5.7923117	4.5383997	110.2699966
-5.7923117	4.5871997	110.5800018
-5.7923117	4.6359997	110.9700012
-5.7923117	4.6847997	111.3399963
-5.7923117	4.7336001	111.9399948
-5.7923117	4.7824001	110.2200012
-5.7923117	4.8312001	108.8899994
-5.7923117	4.8800001	111.0499954
-5.7923117	4.9288001	109.1299973
-5.7923117	4.9776001	103.9799957
-5.7923117	5.0264001	103.0299988
-5.7923117	5.0752001	106.2299957
-5.7923117	5.1240001	107.9199982
-5.7923117	5.1728001	104.7999954
-5.7923117	5.2216001	105.5599976
-5.7923117	5.2704000	106.7900009
-5.7923117	5.3192000	102.8699951
-5.7923117	5.3680000	102.7900009
-5.7923117	5.4168000	104.7399979
-5.7923117	5.4656000	104.2399979
-5.7923117	5.5144000	105.8099976
-5.7923117	5.5632000	107.1500015
-5.7923117	5.6120000	107.1199951
-5.7923117	5.6608000	103.5499954
-5.7923117	5.7096000	105.6999969
-5.7923117	5.7584000	105.2999954
-5.7923117	5.8072000	104.5100021
-5.7923117	5.8559999	106.3600006
-5.7923117	5.9047999	106.7699966
-5.7923117	5.9535999	106.4899979
-5.7923117	6.0023999	105.5599976
-5.7923117	6.0511999	102.5800018
-5.7923117	6.0999999	102.1599960
-5.7923117	6.1487999	105.2399979
-5.7923117	6.1975999	105.1299973
-5.7923117	6.2463999	106.0599976
-5.7923117	6.2951999	105.6299973
-5.7923117	6.3439999	103.5800018
-5.7923117	6.3927999	100.1100006
-5.7923117	6.4415998	102.9599991
-5.7923117	6.4903998	105.9000015
-5.7923117	6.5391998	107.3499985
-5.7923117	6.5879998	105.2999954
-5.7923117	6.6367998	105.1199951
-5.7923117	6.6855998	105.8099976
-5.7923117	6.7343998	106.1999969
-5.7923117	6.7831998	102.8699951
-5.7923117	6.8319998	103.2299957
-5.7923117	6.8807998	106.0499954
-5.7923117	6.9295998	102.4499969
-5.7923117	6.9783998	105.9000015
-5.7923117	7.0271997	104.3499985
-5.7923117	7.0759997	100.1599960
-5.7923117	7.1247997	102.8300018
-5.7923117	7.1735997	103.4000015
-5.7923117	7.2223997	102.1800003

-5.7923117	7.2711997	103.7500000
-5.7923117	7.3199997	102.3899994
-5.7923117	7.3687997	99.9199982
-5.7923117	7.4175997	103.9499969
-5.7923117	7.4663997	105.9899979
-5.7923117	7.5151997	104.1399994
-5.7923117	7.5639997	100.0699997
-5.7923117	7.6127996	102.3399963
-5.7923117	7.6615996	104.5499954
-5.7923117	7.7103996	103.6800003
-5.7923117	7.7591996	102.7399979
-5.7923117	7.8079996	103.9499969
-5.7923117	7.8567996	103.3499985
-5.7923117	7.9056001	102.0000000
-5.7923117	7.9544001	102.0100021
-5.7923117	8.0032005	101.1299973
-5.7923117	8.0520000	103.7399979
-5.7923117	8.1008005	104.5899963
-5.7923117	8.1496000	105.1999969
-5.7923117	8.1984005	103.2900009
-5.7923117	8.2472000	106.4399948
-5.7923117	8.2960005	104.2099991
-5.7923117	8.3448000	103.3799973
-5.7923117	8.3936005	104.1999969
-5.7923117	8.4424000	101.6999969
-5.7923117	8.4912004	105.3899994
-5.7923117	8.5400000	105.4599991
-5.7923117	8.5888004	101.9700012
-5.7923117	8.6375999	98.6399994
-5.7923117	8.6864004	100.3499985
-5.7923117	8.7351999	106.1899948
-5.7923117	8.7840004	105.9700012
-5.7923117	8.8327999	104.0100021
-5.7923117	8.8816004	104.2399979
-5.7923117	8.9303999	104.2799988
-5.7923117	8.9792004	101.6999969
-5.7923117	9.0279999	101.7500000
-5.7923117	9.0768003	103.1299973
-5.7923117	9.1255999	102.0699997
-5.7923117	9.1744003	102.6800003
-5.7923117	9.2231998	103.1800003
-5.7923117	9.2720003	101.5400009
-5.7923117	9.3207998	104.7399979
-5.7923117	9.3696003	103.0800018
-5.7923117	9.4184008	102.0100021
-5.7923117	9.4672003	99.0400009
-5.7923117	9.5160007	100.7200012
-5.7923117	9.5648003	105.1199951
-5.7923117	9.6136007	104.6800003
-5.7923117	9.6624002	104.1800003
-5.7923117	9.7112007	104.5400009
-5.7923117	9.7600002	103.8799973
-5.7923117	9.8088007	104.0599976
-5.7923117	9.8576002	101.3899994
-5.7923117	9.9064007	102.8499985
-5.7923117	9.9552002	104.7900009
-5.7923117	10.0040007	102.6899948
-5.7923117	10.0528002	104.8699951

-5.7923117	10.1016006	104.9599991
-5.7923117	10.1504002	103.3600006
-5.7923117	10.1992006	105.5400009
-5.7923117	10.2480001	105.3799973
-5.7923117	10.2968006	103.0899963
-5.7923117	10.3456001	101.3499985
-5.7923117	10.3944006	102.0400009
-5.7923117	10.4432001	101.1299973
-5.7923117	10.4920006	104.4000015
-5.7923117	10.5408001	105.8099976
-5.7923117	10.5896006	105.7399979
-5.7923117	10.6384001	103.7999954
-5.7923117	10.6872005	102.2299957
-5.7923117	10.7360001	105.0699997
-5.7923117	10.7848005	104.7399979
-5.7923117	10.8336000	104.8699951
-5.7923117	10.8824005	104.0899963
-5.7923117	10.9312000	105.5800018
-5.7923117	10.9800005	105.8399963
-5.7923117	11.0288000	105.5800018
-5.7923117	11.0776005	104.9899979
-5.7923117	11.1264000	102.0999985
-5.7923117	11.1752005	101.9399948
-5.7923117	11.2240000	102.9700012
-5.7923117	11.2728004	106.8399963
-5.7923117	11.3216000	106.0800018
-5.7923117	11.3704004	105.0100021
-5.7923117	11.4191999	102.8300018
-5.7923117	11.4680004	103.0999985
-5.7923117	11.5167999	102.4700012
-5.7923117	11.5656004	105.3600006
-5.7923117	11.6143999	105.7900009
-5.7923117	11.6632004	103.7799988
-5.7923117	11.7119999	101.0899963
-5.7923117	11.7608004	102.7299957
-5.7923117	11.8095999	103.6899948
-5.7923117	11.8584003	105.0899963
-5.7923117	11.9071999	105.3799973
-5.7923117	11.9560003	102.2600021
-5.7923117	12.0047998	105.2699966
-5.7923117	12.0536003	105.4700012
-5.7923117	12.1023998	102.8899994
-5.7923117	12.1512003	100.1999969
-5.7923117	12.1999998	104.5000000
-5.7923117	12.2488003	104.6800003
-5.7923117	12.2975998	103.2399979
-5.7923117	12.3464003	101.1699982
-5.7923117	12.3951998	104.7799988
-5.7923117	12.4440002	105.5800018
-5.7923117	12.4927998	103.8899994
-5.7923117	12.5416002	101.1399994
-5.7923117	12.5904007	102.5899963
-5.7923117	12.6392002	103.6299973
-5.7923117	12.6880007	103.6599960
-5.7923117	12.7368002	102.5199966
-5.7923117	12.7856007	102.7600021
-5.7923117	12.8344002	105.5299988
-5.7923117	12.8832006	105.1599960

-5.7923117	12.9320002	102.0299988
-5.7923117	12.9808006	102.7200012
-5.7923117	13.0296001	100.8699951
-5.7923117	13.0784006	102.8899994
-5.7923117	13.1272001	103.9000015
-5.7923117	13.1760006	103.7799988
-5.7923117	13.2248001	100.7799988
-5.7923117	13.2736006	101.5299988
-5.7923117	13.3224001	100.6999969
-5.7923117	13.3712006	102.4499969
-5.7923117	13.4200001	104.3699951
-5.7923117	13.4688005	104.2999954
-5.7923117	13.5176001	103.7399979
-5.7923117	13.5664005	104.3799973
-5.7923117	13.6152000	100.9899979
-5.7923117	13.6640005	104.5299988
-5.7923117	13.7128000	105.2900009
-5.7923117	13.7616005	105.3399963
-5.7923117	13.8104000	103.8799973
-5.7923117	13.8592005	103.7600021
-5.7923117	13.9080000	101.2900009
-5.7923117	13.9568005	101.0599976
-5.7923117	14.0056000	100.6899948
-5.7923117	14.0544004	96.5800018
-5.7923117	14.1032000	100.7500000
-5.7923117	14.1520004	97.0499954
-5.7923117	14.2007999	100.4099960
-5.7923117	14.2496004	101.9399948
-5.7923117	14.2983999	103.5400009
-5.7923117	14.3472004	101.1599960
-5.7923117	14.3959999	99.6699982
-5.7923117	14.4448004	98.5999985
-5.7923117	14.4935999	101.1199951
-5.7923117	14.5424004	103.7900009
-5.7923117	14.5911999	101.7200012
-5.7923117	14.6400003	103.3399963
-5.7923117	14.6887999	104.1399994
-5.7923117	14.7376003	103.4000015
-5.7923117	14.7863998	104.6599960
-5.7923117	14.8352003	106.8600006
-5.7923117	14.8839998	104.5800018
-5.7923117	14.9328003	103.9599991
-5.7923117	14.9815998	102.8699951
-5.7923117	15.0304003	99.5400009
-5.7861757	0.3416000	103.5599976
-5.7861757	0.3904000	104.9499969
-5.7861757	0.4392000	107.2999954
-5.7861757	0.4880000	104.9899979
-5.7861757	0.5368000	103.4199982
-5.7861757	0.5856000	107.2999954
-5.7861757	0.6344000	109.1100006
-5.7861757	0.6832000	109.6399994
-5.7861757	0.7320000	108.7099991
-5.7861757	0.7808000	105.3199997
-5.7861757	0.8296000	107.2699966
-5.7861757	0.8784000	107.6500015
-5.7861757	0.9272000	108.7699966
-5.7861757	0.9760000	109.6500015

-5.7861757	1.0247999	109.9099960
-5.7861757	1.0735999	108.0999985
-5.7861757	1.1223999	107.0999985
-5.7861757	1.1711999	106.7200012
-5.7861757	1.2200000	105.4000015
-5.7861757	1.2688000	103.7900009
-5.7861757	1.3176000	105.3499985
-5.7861757	1.3664000	106.9000015
-5.7861757	1.4152000	107.6699982
-5.7861757	1.4640000	106.3499985
-5.7861757	1.5128000	107.6999969
-5.7861757	1.5616000	105.6199951
-5.7861757	1.6104000	107.1599960
-5.7861757	1.6592000	107.0699997
-5.7861757	1.7079999	106.5999985
-5.7861757	1.7567999	106.4499969
-5.7861757	1.8055999	107.3699951
-5.7861757	1.8543999	112.2299957
-5.7861757	1.9031999	112.9700012
-5.7861757	1.9519999	110.2999954
-5.7861757	2.0007999	107.9700012
-5.7861757	2.0495999	104.8300018
-5.7861757	2.0983999	104.6500015
-5.7861757	2.1471999	105.0499954
-5.7861757	2.1959999	105.0699997
-5.7861757	2.2447999	104.8399963
-5.7861757	2.2935998	105.0899963
-5.7861757	2.3423998	103.1699982
-5.7861757	2.3912001	105.5499954
-5.7861757	2.4400001	106.1899948
-5.7861757	2.4888000	104.6199951
-5.7861757	2.5376000	100.9700012
-5.7861757	2.5864000	104.1399994
-5.7861757	2.6352000	102.2399979
-5.7861757	2.6840000	102.5100021
-5.7861757	2.7328000	104.0400009
-5.7861757	2.7816000	101.4000015
-5.7861757	2.8304000	101.2099991
-5.7861757	2.8792000	104.9499969
-5.7861757	2.9280000	104.8899994
-5.7861757	2.9768000	103.9499969
-5.7861757	3.0256000	108.8699951
-5.7861757	3.0743999	107.8199997
-5.7861757	3.1231999	106.2399979
-5.7861757	3.1719999	107.0499954
-5.7861757	3.2207999	105.4700012
-5.7861757	3.2695999	104.1299973
-5.7861757	3.3183999	101.4799957
-5.7861757	3.3671999	106.3499985
-5.7861757	3.4159999	102.7799988
-5.7861757	3.4647999	102.1800003
-5.7861757	3.5135999	102.7900009
-5.7861757	3.5623999	104.7900009
-5.7861757	3.6111999	101.9099960
-5.7861757	3.6599998	100.6999969
-5.7861757	3.7087998	104.7999954
-5.7861757	3.7575998	104.5400009
-5.7861757	3.8063998	102.5699997

-5.7861757	3.8551998	102.4499969
-5.7861757	3.9039998	102.3899994
-5.7861757	3.9527998	102.1500015
-5.7861757	4.0016003	103.1599960
-5.7861757	4.0503998	98.0199966
-5.7861757	4.0991998	100.8499985
-5.7861757	4.1479998	106.0999985
-5.7861757	4.1967998	104.4899979
-5.7861757	4.2455997	103.6899948
-5.7861757	4.2943997	106.7699966
-5.7861757	4.3431997	108.9499969
-5.7861757	4.3919997	100.5000000
-5.7861757	4.4407997	103.8799973
-5.7861757	4.4895997	106.2299957
-5.7861757	4.5383997	109.4700012
-5.7861757	4.5871997	109.5800018
-5.7861757	4.6359997	110.4000015
-5.7861757	4.6847997	111.0400009
-5.7861757	4.7336001	111.0199966
-5.7861757	4.7824001	109.7399979
-5.7861757	4.8312001	107.6800003
-5.7861757	4.8800001	110.3899994
-5.7861757	4.9288001	108.6299973
-5.7861757	4.9776001	104.6699982
-5.7861757	5.0264001	103.0800018
-5.7861757	5.0752001	105.9700012
-5.7861757	5.1240001	108.5400009
-5.7861757	5.1728001	105.7399979
-5.7861757	5.2216001	105.3799973
-5.7861757	5.2704000	106.9899979
-5.7861757	5.3192000	103.3899994
-5.7861757	5.3680000	102.8600006
-5.7861757	5.4168000	104.7200012
-5.7861757	5.4656000	103.4199982
-5.7861757	5.5144000	105.1899948
-5.7861757	5.5632000	107.0100021
-5.7861757	5.6120000	106.2799988
-5.7861757	5.6608000	103.1800003
-5.7861757	5.7096000	105.1100006
-5.7861757	5.7584000	104.1899948
-5.7861757	5.8072000	104.1599960
-5.7861757	5.8559999	106.1800003
-5.7861757	5.9047999	107.1500015
-5.7861757	5.9535999	106.9000015
-5.7861757	6.0023999	105.5199966
-5.7861757	6.0511999	103.0800018
-5.7861757	6.0999999	102.6500015
-5.7861757	6.1487999	105.7200012
-5.7861757	6.1975999	105.0299988
-5.7861757	6.2463999	105.7999954
-5.7861757	6.2951999	105.5100021
-5.7861757	6.3439999	103.7699966
-5.7861757	6.3927999	99.8199997
-5.7861757	6.4415998	103.6399994
-5.7861757	6.4903998	106.5400009
-5.7861757	6.5391998	107.2600021
-5.7861757	6.5879998	104.6399994
-5.7861757	6.6367998	103.7099991

-5.7861757	6.6855998	104.9000015
-5.7861757	6.7343998	105.7099991
-5.7861757	6.7831998	103.2600021
-5.7861757	6.8319998	103.9300003
-5.7861757	6.8807998	104.6999969
-5.7861757	6.9295998	100.6899948
-5.7861757	6.9783998	104.8499985
-5.7861757	7.0271997	104.8199997
-5.7861757	7.0759997	101.0400009
-5.7861757	7.1247997	103.4199982
-5.7861757	7.1735997	103.7600021
-5.7861757	7.2223997	103.8499985
-5.7861757	7.2711997	103.8600006
-5.7861757	7.3199997	101.6399994
-5.7861757	7.3687997	100.9799957
-5.7861757	7.4175997	104.3199997
-5.7861757	7.4663997	106.0199966
-5.7861757	7.5151997	104.4300003
-5.7861757	7.5639997	100.5299988
-5.7861757	7.6127996	103.0199966
-5.7861757	7.6615996	104.8699951
-5.7861757	7.7103996	103.5499954
-5.7861757	7.7591996	102.5899963
-5.7861757	7.8079996	103.1699982
-5.7861757	7.8567996	102.6699982
-5.7861757	7.9056001	102.1100006
-5.7861757	7.9544001	103.4700012
-5.7861757	8.0032005	103.2999954
-5.7861757	8.0520000	104.7099991
-5.7861757	8.1008005	105.1999969
-5.7861757	8.1496000	105.9599991
-5.7861757	8.1984005	104.9099960
-5.7861757	8.2472000	107.2399979
-5.7861757	8.2960005	105.0100021
-5.7861757	8.3448000	103.4499969
-5.7861757	8.3936005	103.5800018
-5.7861757	8.4424000	101.3699951
-5.7861757	8.4912004	104.6199951
-5.7861757	8.5400000	105.1399994
-5.7861757	8.5888004	102.2600021
-5.7861757	8.6375999	98.9000015
-5.7861757	8.6864004	101.3399963
-5.7861757	8.7351999	106.4899979
-5.7861757	8.7840004	105.3399963
-5.7861757	8.8327999	104.4399948
-5.7861757	8.8816004	104.7900009
-5.7861757	8.9303999	104.1899948
-5.7861757	8.9792004	102.1100006
-5.7861757	9.0279999	102.8099976
-5.7861757	9.0768003	104.0299988
-5.7861757	9.1255999	102.6500015
-5.7861757	9.1744003	102.7299957
-5.7861757	9.2231998	102.6100006
-5.7861757	9.2720003	101.4000015
-5.7861757	9.3207998	104.6999969
-5.7861757	9.3696003	102.7600021
-5.7861757	9.4184008	102.4000015
-5.7861757	9.4672003	100.8600006

-5.7861757	9.5160007	101.2999954
-5.7861757	9.5648003	105.9499969
-5.7861757	9.6136007	105.4399948
-5.7861757	9.6624002	104.5999985
-5.7861757	9.7112007	103.6999969
-5.7861757	9.7600002	103.0499954
-5.7861757	9.8088007	103.4000015
-5.7861757	9.8576002	101.1299973
-5.7861757	9.9064007	102.0100021
-5.7861757	9.9552002	104.7399979
-5.7861757	10.0040007	102.2099991
-5.7861757	10.0528002	104.7600021
-5.7861757	10.1016006	104.4300003
-5.7861757	10.1504002	103.0199966
-5.7861757	10.1992006	104.9799957
-5.7861757	10.2480001	105.2200012
-5.7861757	10.2968006	103.7099991
-5.7861757	10.3456001	102.3199997
-5.7861757	10.3944006	102.0999985
-5.7861757	10.4432001	100.5199966
-5.7861757	10.4920006	103.7099991
-5.7861757	10.5408001	105.0699997
-5.7861757	10.5896006	105.1500015
-5.7861757	10.6384001	104.2500000
-5.7861757	10.6872005	103.7299957
-5.7861757	10.7360001	105.4300003
-5.7861757	10.7848005	104.7999954
-5.7861757	10.8336000	104.9599991
-5.7861757	10.8824005	103.6500015
-5.7861757	10.9312000	105.3600006
-5.7861757	10.9800005	105.3399963
-5.7861757	11.0288000	105.7299957
-5.7861757	11.0776005	105.8600006
-5.7861757	11.1264000	103.8399963
-5.7861757	11.1752005	101.5400009
-5.7861757	11.2240000	102.8399963
-5.7861757	11.2728004	106.2900009
-5.7861757	11.3216000	104.9700012
-5.7861757	11.3704004	104.2900009
-5.7861757	11.4191999	102.1299973
-5.7861757	11.4680004	103.2600021
-5.7861757	11.5167999	102.9700012
-5.7861757	11.5656004	106.3099976
-5.7861757	11.6143999	107.2699966
-5.7861757	11.6632004	105.2099991
-5.7861757	11.7119999	102.3399963
-5.7861757	11.7608004	102.6199951
-5.7861757	11.8095999	103.7999954
-5.7861757	11.8584003	105.2200012
-5.7861757	11.9071999	105.1699982
-5.7861757	11.9560003	101.2099991
-5.7861757	12.0047998	105.2900009
-5.7861757	12.0536003	105.9899979
-5.7861757	12.1023998	103.8099976
-5.7861757	12.1512003	100.8799973
-5.7861757	12.1999998	104.6899948
-5.7861757	12.2488003	103.6500015
-5.7861757	12.2975998	103.3799973

-5.7861757	12.3464003	101.6899948
-5.7861757	12.3951998	104.4399948
-5.7861757	12.4440002	104.9499969
-5.7861757	12.4927998	103.3199997
-5.7861757	12.5416002	100.0199966
-5.7861757	12.5904007	101.2699966
-5.7861757	12.6392002	103.5199966
-5.7861757	12.6880007	104.0800018
-5.7861757	12.7368002	102.3199997
-5.7861757	12.7856007	102.0499954
-5.7861757	12.8344002	104.6500015
-5.7861757	12.8832006	104.7999954
-5.7861757	12.9320002	102.0699997
-5.7861757	12.9808006	102.5699997
-5.7861757	13.0296001	101.4799957
-5.7861757	13.0784006	103.1699982
-5.7861757	13.1272001	104.0699997
-5.7861757	13.1760006	103.5299988
-5.7861757	13.2248001	100.5100021
-5.7861757	13.2736006	101.4300003
-5.7861757	13.3224001	101.4499969
-5.7861757	13.3712006	102.4099960
-5.7861757	13.4200001	103.8099976
-5.7861757	13.4688005	104.3600006
-5.7861757	13.5176001	103.5100021
-5.7861757	13.5664005	104.0199966
-5.7861757	13.6152000	102.0999985
-5.7861757	13.6640005	105.0199966
-5.7861757	13.7128000	104.8199997
-5.7861757	13.7616005	105.0199966
-5.7861757	13.8104000	104.0899963
-5.7861757	13.8592005	103.8499985
-5.7861757	13.9080000	100.6299973
-5.7861757	13.9568005	101.0400009
-5.7861757	14.0056000	100.7699966
-5.7861757	14.0544004	97.5899963
-5.7861757	14.1032000	100.8799973
-5.7861757	14.1520004	97.1800003
-5.7861757	14.2007999	100.0000000
-5.7861757	14.2496004	101.3499985
-5.7861757	14.2983999	102.8799973
-5.7861757	14.3472004	101.5000000
-5.7861757	14.3959999	100.1399994
-5.7861757	14.4448004	99.2699966
-5.7861757	14.4935999	100.1299973
-5.7861757	14.5424004	103.2600021
-5.7861757	14.5911999	102.9399948
-5.7861757	14.6400003	103.0400009
-5.7861757	14.6887999	104.0199966
-5.7861757	14.7376003	103.8799973
-5.7861757	14.7863998	105.7999954
-5.7861757	14.8352003	106.9099960
-5.7861757	14.8839998	104.4799957
-5.7861757	14.9328003	104.7200012
-5.7861757	14.9815998	104.2999954
-5.7861757	15.0304003	100.9199982
-5.7800398	0.3416000	103.6500015
-5.7800398	0.3904000	105.0599976

-5.7800398	0.4392000	106.4499969
-5.7800398	0.4880000	103.9700012
-5.7800398	0.5368000	104.8699951
-5.7800398	0.5856000	108.2099991
-5.7800398	0.6344000	108.9599991
-5.7800398	0.6832000	109.6100006
-5.7800398	0.7320000	108.6999969
-5.7800398	0.7808000	103.3499985
-5.7800398	0.8296000	105.2399979
-5.7800398	0.8784000	107.9700012
-5.7800398	0.9272000	109.2500000
-5.7800398	0.9760000	110.1699982
-5.7800398	1.0247999	109.8600006
-5.7800398	1.0735999	107.8399963
-5.7800398	1.1223999	106.6399994
-5.7800398	1.1711999	107.0499954
-5.7800398	1.2200000	106.1100006
-5.7800398	1.2688000	103.3199997
-5.7800398	1.3176000	104.9000015
-5.7800398	1.3664000	106.5599976
-5.7800398	1.4152000	107.9199982
-5.7800398	1.4640000	107.5899963
-5.7800398	1.5128000	108.2699966
-5.7800398	1.5616000	104.6599960
-5.7800398	1.6104000	106.6999969
-5.7800398	1.6592000	106.9599991
-5.7800398	1.7079999	105.7799988
-5.7800398	1.7567999	105.6299973
-5.7800398	1.8055999	107.6299973
-5.7800398	1.8543999	111.8600006
-5.7800398	1.9031999	112.4799957
-5.7800398	1.9519999	109.9399948
-5.7800398	2.0007999	108.3799973
-5.7800398	2.0495999	105.1699982
-5.7800398	2.0983999	104.9799957
-5.7800398	2.1471999	105.3099976
-5.7800398	2.1959999	105.6899948
-5.7800398	2.2447999	105.6500015
-5.7800398	2.2935998	104.9399948
-5.7800398	2.3423998	102.2999954
-5.7800398	2.3912001	105.0499954
-5.7800398	2.4400001	105.7699966
-5.7800398	2.4888000	103.9899979
-5.7800398	2.5376000	102.7799988
-5.7800398	2.5864000	104.6599960
-5.7800398	2.6352000	101.4099960
-5.7800398	2.6840000	103.4799957
-5.7800398	2.7328000	103.8799973
-5.7800398	2.7816000	100.2799988
-5.7800398	2.8304000	100.0899963
-5.7800398	2.8792000	104.1199951
-5.7800398	2.9280000	104.8600006
-5.7800398	2.9768000	104.1800003
-5.7800398	3.0256000	108.4300003
-5.7800398	3.0743999	107.5400009
-5.7800398	3.1231999	105.3399963
-5.7800398	3.1719999	106.4499969
-5.7800398	3.2207999	104.7699966

-5.7800398	3.2695999	104.5100021
-5.7800398	3.3183999	103.5400009
-5.7800398	3.3671999	107.3600006
-5.7800398	3.4159999	102.8399963
-5.7800398	3.4647999	100.6699982
-5.7800398	3.5135999	102.2500000
-5.7800398	3.5623999	103.7900009
-5.7800398	3.6111999	100.2399979
-5.7800398	3.6599998	100.1999969
-5.7800398	3.7087998	104.5800018
-5.7800398	3.7575998	104.8499985
-5.7800398	3.8063998	102.9499969
-5.7800398	3.8551998	102.3099976
-5.7800398	3.9039998	102.3799973
-5.7800398	3.9527998	102.4300003
-5.7800398	4.0016003	103.5299988
-5.7800398	4.0503998	98.7099991
-5.7800398	4.0991998	100.3799973
-5.7800398	4.1479998	105.8899994
-5.7800398	4.1967998	104.1899948
-5.7800398	4.2455997	103.2799988
-5.7800398	4.2943997	106.1899948
-5.7800398	4.3431997	108.6100006
-5.7800398	4.3919997	101.3099976
-5.7800398	4.4407997	104.1100006
-5.7800398	4.4895997	105.9700012
-5.7800398	4.5383997	108.6399994
-5.7800398	4.5871997	108.9499969
-5.7800398	4.6359997	110.2799988
-5.7800398	4.6847997	111.2799988
-5.7800398	4.7336001	110.9799957
-5.7800398	4.7824001	109.3600006
-5.7800398	4.8312001	106.7500000
-5.7800398	4.8800001	109.9899979
-5.7800398	4.9288001	108.0899963
-5.7800398	4.9776001	104.8399963
-5.7800398	5.0264001	102.2999954
-5.7800398	5.0752001	104.7699966
-5.7800398	5.1240001	107.9099960
-5.7800398	5.1728001	105.8300018
-5.7800398	5.2216001	104.5899963
-5.7800398	5.2704000	106.9399948
-5.7800398	5.3192000	104.0899963
-5.7800398	5.3680000	103.0199966
-5.7800398	5.4168000	104.9499969
-5.7800398	5.4656000	103.2099991
-5.7800398	5.5144000	105.0599976
-5.7800398	5.5632000	106.6999969
-5.7800398	5.6120000	105.1899948
-5.7800398	5.6608000	102.9000015
-5.7800398	5.7096000	104.1800003
-5.7800398	5.7584000	102.6800003
-5.7800398	5.8072000	103.9099960
-5.7800398	5.8559999	105.9000015
-5.7800398	5.9047999	107.2099991
-5.7800398	5.9535999	106.6100006
-5.7800398	6.0023999	105.0899963
-5.7800398	6.0511999	103.5800018

-5.7800398	6.0999999	104.0299988
-5.7800398	6.1487999	106.1599960
-5.7800398	6.1975999	104.4199982
-5.7800398	6.2463999	105.0699997
-5.7800398	6.2951999	104.0599976
-5.7800398	6.3439999	104.4799957
-5.7800398	6.3927999	101.2200012
-5.7800398	6.4415998	105.0899963
-5.7800398	6.4903998	107.3099976
-5.7800398	6.5391998	106.9899979
-5.7800398	6.5879998	103.9199982
-5.7800398	6.6367998	103.0800018
-5.7800398	6.6855998	104.5899963
-5.7800398	6.7343998	105.2600021
-5.7800398	6.7831998	103.5100021
-5.7800398	6.8319998	104.1500015
-5.7800398	6.8807998	103.4899979
-5.7800398	6.9295998	100.3199997
-5.7800398	6.9783998	103.1899948
-5.7800398	7.0271997	104.3099976
-5.7800398	7.0759997	101.0599976
-5.7800398	7.1247997	103.8199997
-5.7800398	7.1735997	104.0800018
-5.7800398	7.2223997	104.8600006
-5.7800398	7.2711997	103.8099976
-5.7800398	7.3199997	101.5699997
-5.7800398	7.3687997	102.5100021
-5.7800398	7.4175997	105.2799988
-5.7800398	7.4663997	106.8300018
-5.7800398	7.5151997	105.1899948
-5.7800398	7.5639997	101.2600021
-5.7800398	7.6127996	103.6399994
-5.7800398	7.6615996	105.2099991
-5.7800398	7.7103996	103.8899994
-5.7800398	7.7591996	102.3399963
-5.7800398	7.8079996	102.5599976
-5.7800398	7.8567996	101.8099976
-5.7800398	7.9056001	101.4700012
-5.7800398	7.9544001	104.0299988
-5.7800398	8.0032005	104.6500015
-5.7800398	8.0520000	105.2699966
-5.7800398	8.1008005	105.5400009
-5.7800398	8.1496000	106.2999954
-5.7800398	8.1984005	105.8799973
-5.7800398	8.2472000	107.8199997
-5.7800398	8.2960005	105.4799957
-5.7800398	8.3448000	103.2099991
-5.7800398	8.3936005	103.0400009
-5.7800398	8.4424000	101.6599960
-5.7800398	8.4912004	104.7299957
-5.7800398	8.5400000	104.9199982
-5.7800398	8.5888004	103.0100021
-5.7800398	8.6375999	100.3699951
-5.7800398	8.6864004	102.1800003
-5.7800398	8.7351999	106.3099976
-5.7800398	8.7840004	104.4499969
-5.7800398	8.8327999	104.7500000
-5.7800398	8.8816004	104.6699982

-5.7800398	8.9303999	104.4099960
-5.7800398	8.9792004	103.1399994
-5.7800398	9.0279999	104.0199966
-5.7800398	9.0768003	104.8499985
-5.7800398	9.1255999	103.1699982
-5.7800398	9.1744003	103.0000000
-5.7800398	9.2231998	101.7699966
-5.7800398	9.2720003	100.4099960
-5.7800398	9.3207998	103.7600021
-5.7800398	9.3696003	101.9799957
-5.7800398	9.4184008	103.0199966
-5.7800398	9.4672003	102.7200012
-5.7800398	9.5160007	102.5499954
-5.7800398	9.5648003	106.5800018
-5.7800398	9.6136007	105.6299973
-5.7800398	9.6624002	104.2900009
-5.7800398	9.7112007	103.0400009
-5.7800398	9.7600002	102.3799973
-5.7800398	9.8088007	102.5800018
-5.7800398	9.8576002	101.0299988
-5.7800398	9.9064007	101.0599976
-5.7800398	9.9552002	104.4799957
-5.7800398	10.0040007	101.1699982
-5.7800398	10.0528002	104.2600021
-5.7800398	10.1016006	103.3600006
-5.7800398	10.1504002	102.1699982
-5.7800398	10.1992006	104.1399994
-5.7800398	10.2480001	104.9300003
-5.7800398	10.2968006	104.1399994
-5.7800398	10.3456001	102.7299957
-5.7800398	10.3944006	101.4399948
-5.7800398	10.4432001	99.2299957
-5.7800398	10.4920006	102.7399979
-5.7800398	10.5408001	104.2600021
-5.7800398	10.5896006	104.8499985
-5.7800398	10.6384001	104.3899994
-5.7800398	10.6872005	105.1199951
-5.7800398	10.7360001	105.5999985
-5.7800398	10.7848005	104.5100021
-5.7800398	10.8336000	105.3499985
-5.7800398	10.8824005	104.3899994
-5.7800398	10.9312000	105.5999985
-5.7800398	10.9800005	104.6199951
-5.7800398	11.0288000	105.2999954
-5.7800398	11.0776005	105.8899994
-5.7800398	11.1264000	105.2200012
-5.7800398	11.1752005	100.8300018
-5.7800398	11.2240000	102.3099976
-5.7800398	11.2728004	105.6899948
-5.7800398	11.3216000	103.8899994
-5.7800398	11.3704004	103.2500000
-5.7800398	11.4191999	101.2200012
-5.7800398	11.4680004	103.7999954
-5.7800398	11.5167999	103.8799973
-5.7800398	11.5656004	107.0299988
-5.7800398	11.6143999	107.8799973
-5.7800398	11.6632004	105.7299957
-5.7800398	11.7119999	103.0400009

-5.7800398	11.7608004	102.0499954
-5.7800398	11.8095999	103.9399948
-5.7800398	11.8584003	105.2399979
-5.7800398	11.9071999	104.6699982
-5.7800398	11.9560003	99.8799973
-5.7800398	12.0047998	104.1199951
-5.7800398	12.0536003	105.4899979
-5.7800398	12.1023998	104.6699982
-5.7800398	12.1512003	102.4899979
-5.7800398	12.1999998	105.2399979
-5.7800398	12.2488003	102.6199951
-5.7800398	12.2975998	103.6299973
-5.7800398	12.3464003	103.0699997
-5.7800398	12.3951998	104.8600006
-5.7800398	12.4440002	104.2099991
-5.7800398	12.4927998	102.7999954
-5.7800398	12.5416002	100.0599976
-5.7800398	12.5904007	100.9700012
-5.7800398	12.6392002	103.6699982
-5.7800398	12.6880007	104.9799957
-5.7800398	12.7368002	102.6199951
-5.7800398	12.7856007	101.4399948
-5.7800398	12.8344002	103.3499985
-5.7800398	12.8832006	104.1199951
-5.7800398	12.9320002	101.8799973
-5.7800398	12.9808006	102.4300003
-5.7800398	13.0296001	102.2699966
-5.7800398	13.0784006	103.2399979
-5.7800398	13.1272001	104.1199951
-5.7800398	13.1760006	103.3199997
-5.7800398	13.2248001	100.4300003
-5.7800398	13.2736006	100.5599976
-5.7800398	13.3224001	101.2299957
-5.7800398	13.3712006	101.0299988
-5.7800398	13.4200001	102.6399994
-5.7800398	13.4688005	104.3300018
-5.7800398	13.5176001	103.3399963
-5.7800398	13.5664005	103.3699951
-5.7800398	13.6152000	102.8899994
-5.7800398	13.6640005	104.9499969
-5.7800398	13.7128000	103.4300003
-5.7800398	13.7616005	104.5299988
-5.7800398	13.8104000	104.3799973
-5.7800398	13.8592005	103.9899979
-5.7800398	13.9080000	100.0699997
-5.7800398	13.9568005	101.5599976
-5.7800398	14.0056000	101.4000015
-5.7800398	14.0544004	99.0699997
-5.7800398	14.1032000	101.6899948
-5.7800398	14.1520004	98.3699951
-5.7800398	14.2007999	100.4899979
-5.7800398	14.2496004	101.6199951
-5.7800398	14.2983999	102.9099960
-5.7800398	14.3472004	102.2200012
-5.7800398	14.3959999	100.5899963
-5.7800398	14.4448004	100.4499969
-5.7800398	14.4935999	100.0999985
-5.7800398	14.5424004	102.8399963

-5.7800398	14.5911999	103.9399948
-5.7800398	14.6400003	103.1800003
-5.7800398	14.6887999	104.4499969
-5.7800398	14.7376003	104.4399948
-5.7800398	14.7863998	107.2500000
-5.7800398	14.8352003	107.1699982
-5.7800398	14.8839998	104.3099976
-5.7800398	14.9328003	104.9599991
-5.7800398	14.9815998	104.7600021
-5.7800398	15.0304003	101.8499985
-5.7739038	0.3416000	103.9599991
-5.7739038	0.3904000	105.3699951
-5.7739038	0.4392000	106.0199966
-5.7739038	0.4880000	103.2999954
-5.7739038	0.5368000	106.1500015
-5.7739038	0.5856000	108.6599960
-5.7739038	0.6344000	108.6399994
-5.7739038	0.6832000	109.7200012
-5.7739038	0.7320000	108.4700012
-5.7739038	0.7808000	101.1899948
-5.7739038	0.8296000	103.2799988
-5.7739038	0.8784000	108.6100006
-5.7739038	0.9272000	110.1999969
-5.7739038	0.9760000	110.6699982
-5.7739038	1.0247999	109.0699997
-5.7739038	1.0735999	106.9799957
-5.7739038	1.1223999	105.6299973
-5.7739038	1.1711999	106.9000015
-5.7739038	1.2200000	106.2699966
-5.7739038	1.2688000	102.7500000
-5.7739038	1.3176000	104.2699966
-5.7739038	1.3664000	106.1999969
-5.7739038	1.4152000	108.1500015
-5.7739038	1.4640000	108.5400009
-5.7739038	1.5128000	108.7999954
-5.7739038	1.5616000	104.6100006
-5.7739038	1.6104000	106.1599960
-5.7739038	1.6592000	107.0000000
-5.7739038	1.7079999	105.9599991
-5.7739038	1.7567999	105.7999954
-5.7739038	1.8055999	107.9799957
-5.7739038	1.8543999	111.3399963
-5.7739038	1.9031999	112.0199966
-5.7739038	1.9519999	110.1199951
-5.7739038	2.0007999	108.7099991
-5.7739038	2.0495999	105.3799973
-5.7739038	2.0983999	105.0599976
-5.7739038	2.1471999	105.3499985
-5.7739038	2.1959999	106.3199997
-5.7739038	2.2447999	106.5999985
-5.7739038	2.2935998	105.6699982
-5.7739038	2.3423998	102.4499969
-5.7739038	2.3912001	104.0800018
-5.7739038	2.4400001	105.0499954
-5.7739038	2.4888000	103.2200012
-5.7739038	2.5376000	104.2799988
-5.7739038	2.5864000	105.0699997
-5.7739038	2.6352000	101.8199997

-5.7739038	2.6840000	105.0400009
-5.7739038	2.7328000	104.3699951
-5.7739038	2.7816000	100.2200012
-5.7739038	2.8304000	99.9000015
-5.7739038	2.8792000	103.4799957
-5.7739038	2.9280000	104.9000015
-5.7739038	2.9768000	104.0400009
-5.7739038	3.0256000	107.9499969
-5.7739038	3.0743999	107.4799957
-5.7739038	3.1231999	104.5899963
-5.7739038	3.1719999	106.1500015
-5.7739038	3.2207999	105.0299988
-5.7739038	3.2695999	104.9899979
-5.7739038	3.3183999	105.1199951
-5.7739038	3.3671999	107.4399948
-5.7739038	3.4159999	103.2399979
-5.7739038	3.4647999	100.2900009
-5.7739038	3.5135999	102.4099960
-5.7739038	3.5623999	103.2699966
-5.7739038	3.6111999	99.2699966
-5.7739038	3.6599998	99.6899948
-5.7739038	3.7087998	103.9499969
-5.7739038	3.7575998	104.0199966
-5.7739038	3.8063998	102.8300018
-5.7739038	3.8551998	103.0999985
-5.7739038	3.9039998	102.9399948
-5.7739038	3.9527998	102.7200012
-5.7739038	4.0016003	103.8699951
-5.7739038	4.0503998	99.3600006
-5.7739038	4.0991998	99.9199982
-5.7739038	4.1479998	105.6599960
-5.7739038	4.1967998	103.6899948
-5.7739038	4.2455997	102.4300003
-5.7739038	4.2943997	106.5499954
-5.7739038	4.3431997	108.6899948
-5.7739038	4.3919997	102.6100006
-5.7739038	4.4407997	104.9099960
-5.7739038	4.4895997	106.7500000
-5.7739038	4.5383997	108.4799957
-5.7739038	4.5871997	108.7799988
-5.7739038	4.6359997	110.0299988
-5.7739038	4.6847997	112.0400009
-5.7739038	4.7336001	111.3799973
-5.7739038	4.7824001	108.7900009
-5.7739038	4.8312001	105.5100021
-5.7739038	4.8800001	109.6100006
-5.7739038	4.9288001	107.7500000
-5.7739038	4.9776001	104.5400009
-5.7739038	5.0264001	101.4799957
-5.7739038	5.0752001	103.3899994
-5.7739038	5.1240001	107.0800018
-5.7739038	5.1728001	105.9000015
-5.7739038	5.2216001	104.1899948
-5.7739038	5.2704000	106.5499954
-5.7739038	5.3192000	105.2999954
-5.7739038	5.3680000	104.8300018
-5.7739038	5.4168000	105.7799988
-5.7739038	5.4656000	103.0400009

-5.7739038	5.5144000	104.5699997
-5.7739038	5.5632000	105.2500000
-5.7739038	5.6120000	103.6399994
-5.7739038	5.6608000	102.5599976
-5.7739038	5.7096000	103.2900009
-5.7739038	5.7584000	101.5599976
-5.7739038	5.8072000	104.4599991
-5.7739038	5.8559999	105.9700012
-5.7739038	5.9047999	107.1899948
-5.7739038	5.9535999	106.6699982
-5.7739038	6.0023999	105.4499969
-5.7739038	6.0511999	104.7699966
-5.7739038	6.0999999	105.3699951
-5.7739038	6.1487999	106.4599991
-5.7739038	6.1975999	104.2200012
-5.7739038	6.2463999	104.9599991
-5.7739038	6.2951999	102.6299973
-5.7739038	6.3439999	105.0299988
-5.7739038	6.3927999	102.8300018
-5.7739038	6.4415998	106.3799973
-5.7739038	6.4903998	108.0199966
-5.7739038	6.5391998	106.6899948
-5.7739038	6.5879998	102.6800003
-5.7739038	6.6367998	102.3300018
-5.7739038	6.6855998	104.3099976
-5.7739038	6.7343998	104.7999954
-5.7739038	6.7831998	103.8899994
-5.7739038	6.8319998	104.1899948
-5.7739038	6.8807998	102.2399979
-5.7739038	6.9295998	99.6899948
-5.7739038	6.9783998	101.3199997
-5.7739038	7.0271997	104.0899963
-5.7739038	7.0759997	101.1199951
-5.7739038	7.1247997	103.8199997
-5.7739038	7.1735997	103.2399979
-5.7739038	7.2223997	105.1100006
-5.7739038	7.2711997	103.6899948
-5.7739038	7.3199997	102.0400009
-5.7739038	7.3687997	103.7399979
-5.7739038	7.4175997	105.0400009
-5.7739038	7.4663997	107.0699997
-5.7739038	7.5151997	105.4799957
-5.7739038	7.5639997	101.7900009
-5.7739038	7.6127996	104.0000000
-5.7739038	7.6615996	105.1299973
-5.7739038	7.7103996	103.9899979
-5.7739038	7.7591996	101.8399963
-5.7739038	7.8079996	101.6299973
-5.7739038	7.8567996	101.1899948
-5.7739038	7.9056001	101.2600021
-5.7739038	7.9544001	104.7399979
-5.7739038	8.0032005	106.1100006
-5.7739038	8.0520000	106.0499954
-5.7739038	8.1008005	105.6299973
-5.7739038	8.1496000	105.4799957
-5.7739038	8.1984005	106.4399948
-5.7739038	8.2472000	107.9799957
-5.7739038	8.2960005	105.4199982

-5.7739038	8.3448000	102.9300003
-5.7739038	8.3936005	102.6999969
-5.7739038	8.4424000	102.9599991
-5.7739038	8.4912004	104.9300003
-5.7739038	8.5400000	104.0800018
-5.7739038	8.5888004	103.2799988
-5.7739038	8.6375999	102.0699997
-5.7739038	8.6864004	103.1800003
-5.7739038	8.7351999	106.0699997
-5.7739038	8.7840004	103.5499954
-5.7739038	8.8327999	104.5499954
-5.7739038	8.8816004	104.8300018
-5.7739038	8.9303999	104.6500015
-5.7739038	8.9792004	104.1399994
-5.7739038	9.0279999	105.4199982
-5.7739038	9.0768003	105.4799957
-5.7739038	9.1255999	103.5400009
-5.7739038	9.1744003	102.9199982
-5.7739038	9.2231998	101.3399963
-5.7739038	9.2720003	100.0800018
-5.7739038	9.3207998	102.9599991
-5.7739038	9.3696003	102.0599976
-5.7739038	9.4184008	103.0499954
-5.7739038	9.4672003	103.2600021
-5.7739038	9.5160007	102.1100006
-5.7739038	9.5648003	105.7200012
-5.7739038	9.6136007	104.9199982
-5.7739038	9.6624002	103.6500015
-5.7739038	9.7112007	102.1199951
-5.7739038	9.7600002	101.6100006
-5.7739038	9.8088007	102.3099976
-5.7739038	9.8576002	101.2900009
-5.7739038	9.9064007	100.2699966
-5.7739038	9.9552002	103.8799973
-5.7739038	10.0040007	101.3499985
-5.7739038	10.0528002	103.6800003
-5.7739038	10.1016006	102.2399979
-5.7739038	10.1504002	101.5100021
-5.7739038	10.1992006	103.3600006
-5.7739038	10.2480001	103.8799973
-5.7739038	10.2968006	104.3399963
-5.7739038	10.3456001	102.6100006
-5.7739038	10.3944006	101.0800018
-5.7739038	10.4432001	99.3899994
-5.7739038	10.4920006	102.2799988
-5.7739038	10.5408001	104.0199966
-5.7739038	10.5896006	105.0199966
-5.7739038	10.6384001	104.2900009
-5.7739038	10.6872005	105.8899994
-5.7739038	10.7360001	105.5299988
-5.7739038	10.7848005	103.9899979
-5.7739038	10.8336000	105.3199997
-5.7739038	10.8824005	104.5299988
-5.7739038	10.9312000	104.9899979
-5.7739038	10.9800005	103.6800003
-5.7739038	11.0288000	104.7900009
-5.7739038	11.0776005	105.6800003
-5.7739038	11.1264000	106.1999969

-5.7739038	11.1752005	100.1800003
-5.7739038	11.2240000	101.9000015
-5.7739038	11.2728004	104.5999985
-5.7739038	11.3216000	102.0699997
-5.7739038	11.3704004	101.9199982
-5.7739038	11.4191999	99.8799973
-5.7739038	11.4680004	104.1800003
-5.7739038	11.5167999	104.6599960
-5.7739038	11.5656004	107.2799988
-5.7739038	11.6143999	107.9199982
-5.7739038	11.6632004	105.6899948
-5.7739038	11.7119999	103.3799973
-5.7739038	11.7608004	102.0299988
-5.7739038	11.8095999	104.3300018
-5.7739038	11.8584003	105.1699982
-5.7739038	11.9071999	104.2900009
-5.7739038	11.9560003	99.9499969
-5.7739038	12.0047998	103.1299973
-5.7739038	12.0536003	105.0899963
-5.7739038	12.1023998	104.9700012
-5.7739038	12.1512003	103.5899963
-5.7739038	12.1999998	105.0699997
-5.7739038	12.2488003	101.5299988
-5.7739038	12.2975998	103.2699966
-5.7739038	12.3464003	104.1100006
-5.7739038	12.3951998	105.5299988
-5.7739038	12.4440002	103.9300003
-5.7739038	12.4927998	102.7799988
-5.7739038	12.5416002	100.7900009
-5.7739038	12.5904007	101.4300003
-5.7739038	12.6392002	103.8600006
-5.7739038	12.6880007	104.9199982
-5.7739038	12.7368002	101.8099976
-5.7739038	12.7856007	100.2299957
-5.7739038	12.8344002	102.0400009
-5.7739038	12.8832006	103.8499985
-5.7739038	12.9320002	101.5000000
-5.7739038	12.9808006	101.7200012
-5.7739038	13.0296001	103.2299957
-5.7739038	13.0784006	103.5899963
-5.7739038	13.1272001	104.1199951
-5.7739038	13.1760006	102.8699951
-5.7739038	13.2248001	100.4199982
-5.7739038	13.2736006	100.2099991
-5.7739038	13.3224001	100.6100006
-5.7739038	13.3712006	99.5299988
-5.7739038	13.4200001	101.5699997
-5.7739038	13.4688005	104.1999969
-5.7739038	13.5176001	103.1500015
-5.7739038	13.5664005	102.9199982
-5.7739038	13.6152000	103.3899994
-5.7739038	13.6640005	104.6500015
-5.7739038	13.7128000	101.6699982
-5.7739038	13.7616005	103.4499969
-5.7739038	13.8104000	104.0100021
-5.7739038	13.8592005	103.7500000
-5.7739038	13.9080000	99.7799988
-5.7739038	13.9568005	102.7099991

-5.7739038	14.0056000	102.0800018
-5.7739038	14.0544004	100.7699966
-5.7739038	14.1032000	101.8600006
-5.7739038	14.1520004	98.9899979
-5.7739038	14.2007999	100.4799957
-5.7739038	14.2496004	101.0400009
-5.7739038	14.2983999	103.0199966
-5.7739038	14.3472004	103.1299973
-5.7739038	14.3959999	100.9700012
-5.7739038	14.4448004	101.7099991
-5.7739038	14.4935999	100.6999969
-5.7739038	14.5424004	102.5499954
-5.7739038	14.5911999	104.1500015
-5.7739038	14.6400003	103.5100021
-5.7739038	14.6887999	104.9099960
-5.7739038	14.7376003	104.4599991
-5.7739038	14.7863998	108.1100006
-5.7739038	14.8352003	106.9399948
-5.7739038	14.8839998	104.4000015
-5.7739038	14.9328003	104.8699951
-5.7739038	14.9815998	104.7099991
-5.7739038	15.0304003	101.8199997
-5.7677679	0.3416000	105.4099960
-5.7677679	0.3904000	106.6500015
-5.7677679	0.4392000	105.9000015
-5.7677679	0.4880000	102.3199997
-5.7677679	0.5368000	106.8099976
-5.7677679	0.5856000	108.5800018
-5.7677679	0.6344000	107.6199951
-5.7677679	0.6832000	108.8899994
-5.7677679	0.7320000	108.0100021
-5.7677679	0.7808000	99.6299973
-5.7677679	0.8296000	101.7699966
-5.7677679	0.8784000	108.0199966
-5.7677679	0.9272000	110.0000000
-5.7677679	0.9760000	110.1699982
-5.7677679	1.0247999	107.0999985
-5.7677679	1.0735999	105.1599960
-5.7677679	1.1223999	104.7999954
-5.7677679	1.1711999	106.7699966
-5.7677679	1.2200000	105.9000015
-5.7677679	1.2688000	101.2799988
-5.7677679	1.3176000	103.0199966
-5.7677679	1.3664000	105.7699966
-5.7677679	1.4152000	108.4300003
-5.7677679	1.4640000	108.9399948
-5.7677679	1.5128000	108.7299957
-5.7677679	1.5616000	105.3899994
-5.7677679	1.6104000	105.9099960
-5.7677679	1.6592000	107.4399948
-5.7677679	1.7079999	106.4099960
-5.7677679	1.7567999	106.3199997
-5.7677679	1.8055999	108.5499954
-5.7677679	1.8543999	110.7900009
-5.7677679	1.9031999	112.0400009
-5.7677679	1.9519999	110.4399948
-5.7677679	2.0007999	108.7999954
-5.7677679	2.0495999	105.4000015

-5. 7677679	2. 0983999	104. 8799973
-5. 7677679	2. 1471999	104. 6500015
-5. 7677679	2. 1959999	106. 6100006
-5. 7677679	2. 2447999	107. 3199997
-5. 7677679	2. 2935998	105. 7099991
-5. 7677679	2. 3423998	102. 6699982
-5. 7677679	2. 3912001	103. 9000015
-5. 7677679	2. 4400001	105. 1500015
-5. 7677679	2. 4888000	102. 7799988
-5. 7677679	2. 5376000	104. 2699966
-5. 7677679	2. 5864000	105. 5999985
-5. 7677679	2. 6352000	102. 8899994
-5. 7677679	2. 6840000	105. 9199982
-5. 7677679	2. 7328000	105. 0000000
-5. 7677679	2. 7816000	100. 8600006
-5. 7677679	2. 8304000	100. 2200012
-5. 7677679	2. 8792000	103. 1699982
-5. 7677679	2. 9280000	104. 8300018
-5. 7677679	2. 9768000	103. 7500000
-5. 7677679	3. 0256000	107. 4599991
-5. 7677679	3. 0743999	107. 2399979
-5. 7677679	3. 1231999	103. 6999969
-5. 7677679	3. 1719999	106. 0299988
-5. 7677679	3. 2207999	105. 2600021
-5. 7677679	3. 2695999	104. 6199951
-5. 7677679	3. 3183999	106. 0999985
-5. 7677679	3. 3671999	107. 0299988
-5. 7677679	3. 4159999	103. 2200012
-5. 7677679	3. 4647999	100. 0800018
-5. 7677679	3. 5135999	103. 0899963
-5. 7677679	3. 5623999	103. 5400009
-5. 7677679	3. 6111999	99. 2200012
-5. 7677679	3. 6599998	99. 7999954
-5. 7677679	3. 7087998	103. 3899994
-5. 7677679	3. 7575998	103. 4700012
-5. 7677679	3. 8063998	103. 5999985
-5. 7677679	3. 8551998	104. 3899994
-5. 7677679	3. 9039998	103. 1599960
-5. 7677679	3. 9527998	103. 2500000
-5. 7677679	4. 0016003	104. 7200012
-5. 7677679	4. 0503998	100. 6999969
-5. 7677679	4. 0991998	99. 0000000
-5. 7677679	4. 1479998	104. 9599991
-5. 7677679	4. 1967998	103. 3799973
-5. 7677679	4. 2455997	101. 6100006
-5. 7677679	4. 2943997	106. 0899963
-5. 7677679	4. 3431997	107. 8099976
-5. 7677679	4. 3919997	103. 3199997
-5. 7677679	4. 4407997	105. 7799988
-5. 7677679	4. 4895997	107. 6599960
-5. 7677679	4. 5383997	108. 7099991
-5. 7677679	4. 5871997	108. 8499985
-5. 7677679	4. 6359997	110. 0199966
-5. 7677679	4. 6847997	112. 7099991
-5. 7677679	4. 7336001	112. 4199982
-5. 7677679	4. 7824001	109. 0599976
-5. 7677679	4. 8312001	104. 2799988
-5. 7677679	4. 8800001	109. 4499969

-5.7677679	4.9288001	108.0100021
-5.7677679	4.9776001	104.0699997
-5.7677679	5.0264001	100.2900009
-5.7677679	5.0752001	101.8199997
-5.7677679	5.1240001	106.0000000
-5.7677679	5.1728001	105.9799957
-5.7677679	5.2216001	103.9499969
-5.7677679	5.2704000	105.9499969
-5.7677679	5.3192000	106.1399994
-5.7677679	5.3680000	106.2699966
-5.7677679	5.4168000	106.1500015
-5.7677679	5.4656000	102.1199951
-5.7677679	5.5144000	103.5599976
-5.7677679	5.5632000	103.8199997
-5.7677679	5.6120000	103.0699997
-5.7677679	5.6608000	102.6100006
-5.7677679	5.7096000	102.1299973
-5.7677679	5.7584000	101.4499969
-5.7677679	5.8072000	104.6899948
-5.7677679	5.8559999	106.1299973
-5.7677679	5.9047999	107.3199997
-5.7677679	5.9535999	106.4399948
-5.7677679	6.0023999	105.0899963
-5.7677679	6.0511999	104.9199982
-5.7677679	6.0999999	105.8699951
-5.7677679	6.1487999	105.8399963
-5.7677679	6.1975999	104.0499954
-5.7677679	6.2463999	105.2699966
-5.7677679	6.2951999	101.6699982
-5.7677679	6.3439999	104.9199982
-5.7677679	6.3927999	103.8699951
-5.7677679	6.4415998	106.9899979
-5.7677679	6.4903998	108.0199966
-5.7677679	6.5391998	106.2699966
-5.7677679	6.5879998	102.0899963
-5.7677679	6.6367998	101.0199966
-5.7677679	6.6855998	103.1500015
-5.7677679	6.7343998	103.7099991
-5.7677679	6.7831998	103.5699997
-5.7677679	6.8319998	103.0899963
-5.7677679	6.8807998	100.8699951
-5.7677679	6.9295998	99.3399963
-5.7677679	6.9783998	99.7500000
-5.7677679	7.0271997	104.2799988
-5.7677679	7.0759997	102.2099991
-5.7677679	7.1247997	103.7699966
-5.7677679	7.1735997	101.9399948
-5.7677679	7.2223997	104.6299973
-5.7677679	7.2711997	103.6500015
-5.7677679	7.3199997	102.0899963
-5.7677679	7.3687997	104.8600006
-5.7677679	7.4175997	105.0100021
-5.7677679	7.4663997	106.9199982
-5.7677679	7.5151997	105.2799988
-5.7677679	7.5639997	102.1800003
-5.7677679	7.6127996	104.2299957
-5.7677679	7.6615996	105.0800018
-5.7677679	7.7103996	104.5299988

-5.7677679	7.7591996	101.8300018
-5.7677679	7.8079996	100.9599991
-5.7677679	7.8567996	100.7099991
-5.7677679	7.9056001	101.1699982
-5.7677679	7.9544001	105.2799988
-5.7677679	8.0032005	107.1999969
-5.7677679	8.0520000	107.0599976
-5.7677679	8.1008005	106.3300018
-5.7677679	8.1496000	104.7500000
-5.7677679	8.1984005	106.8600006
-5.7677679	8.2472000	107.6199951
-5.7677679	8.2960005	104.7299957
-5.7677679	8.3448000	102.7299957
-5.7677679	8.3936005	102.8300018
-5.7677679	8.4424000	104.0199966
-5.7677679	8.4912004	104.5299988
-5.7677679	8.5400000	103.5100021
-5.7677679	8.5888004	103.2299957
-5.7677679	8.6375999	103.2099991
-5.7677679	8.6864004	104.2999954
-5.7677679	8.7351999	105.5499954
-5.7677679	8.7840004	103.0499954
-5.7677679	8.8327999	104.3600006
-5.7677679	8.8816004	104.6599960
-5.7677679	8.9303999	104.4300003
-5.7677679	8.9792004	104.8099976
-5.7677679	9.0279999	106.3499985
-5.7677679	9.0768003	105.9799957
-5.7677679	9.1255999	103.7399979
-5.7677679	9.1744003	103.1299973
-5.7677679	9.2231998	101.5699997
-5.7677679	9.2720003	100.0800018
-5.7677679	9.3207998	101.6899948
-5.7677679	9.3696003	101.7099991
-5.7677679	9.4184008	103.4399948
-5.7677679	9.4672003	104.2299957
-5.7677679	9.5160007	103.1100006
-5.7677679	9.5648003	105.0199966
-5.7677679	9.6136007	104.0100021
-5.7677679	9.6624002	103.1399994
-5.7677679	9.7112007	102.2600021
-5.7677679	9.7600002	101.9399948
-5.7677679	9.8088007	102.5199966
-5.7677679	9.8576002	101.8300018
-5.7677679	9.9064007	100.9000015
-5.7677679	9.9552002	103.4499969
-5.7677679	10.0040007	101.1199951
-5.7677679	10.0528002	103.1999969
-5.7677679	10.1016006	101.5199966
-5.7677679	10.1504002	101.3399963
-5.7677679	10.1992006	102.7699966
-5.7677679	10.2480001	102.7699966
-5.7677679	10.2968006	104.4899979
-5.7677679	10.3456001	102.5699997
-5.7677679	10.3944006	100.0699997
-5.7677679	10.4432001	99.2299957
-5.7677679	10.4920006	102.4599991
-5.7677679	10.5408001	104.3199997

-5.7677679	10.5896006	105.0800018
-5.7677679	10.6384001	103.2900009
-5.7677679	10.6872005	105.9499969
-5.7677679	10.7360001	105.6800003
-5.7677679	10.7848005	103.5699997
-5.7677679	10.8336000	105.1100006
-5.7677679	10.8824005	104.6999969
-5.7677679	10.9312000	104.5699997
-5.7677679	10.9800005	103.2699966
-5.7677679	11.0288000	104.5800018
-5.7677679	11.0776005	105.4499969
-5.7677679	11.1264000	106.5800018
-5.7677679	11.1752005	101.5100021
-5.7677679	11.2240000	102.6199951
-5.7677679	11.2728004	104.5800018
-5.7677679	11.3216000	101.2399979
-5.7677679	11.3704004	101.2600021
-5.7677679	11.4191999	99.6899948
-5.7677679	11.4680004	105.0100021
-5.7677679	11.5167999	105.6500015
-5.7677679	11.5656004	107.5899963
-5.7677679	11.6143999	107.9199982
-5.7677679	11.6632004	105.6999969
-5.7677679	11.7119999	103.4899979
-5.7677679	11.7608004	103.0999985
-5.7677679	11.8095999	104.7799988
-5.7677679	11.8584003	105.0000000
-5.7677679	11.9071999	103.9599991
-5.7677679	11.9560003	100.5400009
-5.7677679	12.0047998	102.2200012
-5.7677679	12.0536003	104.1399994
-5.7677679	12.1023998	104.3099976
-5.7677679	12.1512003	104.0400009
-5.7677679	12.1999998	104.4499969
-5.7677679	12.2488003	100.1800003
-5.7677679	12.2975998	103.4899979
-5.7677679	12.3464003	105.7299957
-5.7677679	12.3951998	106.2799988
-5.7677679	12.4440002	103.4300003
-5.7677679	12.4927998	102.2299957
-5.7677679	12.5416002	100.8699951
-5.7677679	12.5904007	101.0299988
-5.7677679	12.6392002	103.2399979
-5.7677679	12.6880007	104.6399994
-5.7677679	12.7368002	101.9899979
-5.7677679	12.7856007	99.6299973
-5.7677679	12.8344002	101.2200012
-5.7677679	12.8832006	103.8799973
-5.7677679	12.9320002	102.2500000
-5.7677679	12.9808006	100.9700012
-5.7677679	13.0296001	103.5000000
-5.7677679	13.0784006	102.8899994
-5.7677679	13.1272001	103.9700012
-5.7677679	13.1760006	103.2999954
-5.7677679	13.2248001	100.7099991
-5.7677679	13.2736006	99.7600021
-5.7677679	13.3224001	100.3799973
-5.7677679	13.3712006	99.5800018

-5.7677679	13.4200001	101.8499985
-5.7677679	13.4688005	103.5499954
-5.7677679	13.5176001	102.4000015
-5.7677679	13.5664005	102.1100006
-5.7677679	13.6152000	103.3899994
-5.7677679	13.6640005	103.9099960
-5.7677679	13.7128000	99.8099976
-5.7677679	13.7616005	102.6699982
-5.7677679	13.8104000	103.8399963
-5.7677679	13.8592005	103.3699951
-5.7677679	13.9080000	98.6800003
-5.7677679	13.9568005	102.9399948
-5.7677679	14.0056000	102.6299973
-5.7677679	14.0544004	101.2500000
-5.7677679	14.1032000	101.7999954
-5.7677679	14.1520004	99.6899948
-5.7677679	14.2007999	101.2900009
-5.7677679	14.2496004	100.9499969
-5.7677679	14.2983999	102.9899979
-5.7677679	14.3472004	104.0599976
-5.7677679	14.3959999	102.1699982
-5.7677679	14.4448004	102.8799973
-5.7677679	14.4935999	101.5899963
-5.7677679	14.5424004	103.0299988
-5.7677679	14.5911999	104.7099991
-5.7677679	14.6400003	104.0400009
-5.7677679	14.6887999	105.3699951
-5.7677679	14.7376003	104.0899963
-5.7677679	14.7863998	108.4799957
-5.7677679	14.8352003	106.2299957
-5.7677679	14.8839998	104.1699982
-5.7677679	14.9328003	104.3600006
-5.7677679	14.9815998	103.9300003
-5.7677679	15.0304003	101.1899948
-5.7616320	0.3416000	106.5599976
-5.7616320	0.3904000	107.3199997
-5.7616320	0.4392000	106.1999969
-5.7616320	0.4880000	101.6899948
-5.7616320	0.5368000	106.5499954
-5.7616320	0.5856000	107.9899979
-5.7616320	0.6344000	106.7099991
-5.7616320	0.6832000	108.2699966
-5.7616320	0.7320000	108.0699997
-5.7616320	0.7808000	100.9700012
-5.7616320	0.8296000	100.4499969
-5.7616320	0.8784000	107.0299988
-5.7616320	0.9272000	109.9899979
-5.7616320	0.9760000	109.9899979
-5.7616320	1.0247999	105.6599960
-5.7616320	1.0735999	103.6999969
-5.7616320	1.1223999	103.2399979
-5.7616320	1.1711999	105.5199966
-5.7616320	1.2200000	104.6100006
-5.7616320	1.2688000	100.4599991
-5.7616320	1.3176000	103.1199951
-5.7616320	1.3664000	105.4700012
-5.7616320	1.4152000	108.7799988
-5.7616320	1.4640000	108.5999985

-5.7616320	1.5128000	108.4700012
-5.7616320	1.5616000	105.6199951
-5.7616320	1.6104000	105.3799973
-5.7616320	1.6592000	107.6899948
-5.7616320	1.7079999	107.1299973
-5.7616320	1.7567999	107.4199982
-5.7616320	1.8055999	108.9499969
-5.7616320	1.8543999	110.5599976
-5.7616320	1.9031999	112.3300018
-5.7616320	1.9519999	110.7299957
-5.7616320	2.0007999	108.4300003
-5.7616320	2.0495999	104.6399994
-5.7616320	2.0983999	104.4099960
-5.7616320	2.1471999	103.9499969
-5.7616320	2.1959999	106.3600006
-5.7616320	2.2447999	107.5800018
-5.7616320	2.2935998	105.5899963
-5.7616320	2.3423998	102.8499985
-5.7616320	2.3912001	103.9099960
-5.7616320	2.4400001	105.1999969
-5.7616320	2.4888000	103.6500015
-5.7616320	2.5376000	104.5999985
-5.7616320	2.5864000	105.9000015
-5.7616320	2.6352000	104.3399963
-5.7616320	2.6840000	106.7999954
-5.7616320	2.7328000	104.9599991
-5.7616320	2.7816000	101.6199951
-5.7616320	2.8304000	100.2200012
-5.7616320	2.8792000	102.7799988
-5.7616320	2.9280000	104.5100021
-5.7616320	2.9768000	103.4499969
-5.7616320	3.0256000	106.7799988
-5.7616320	3.0743999	106.6399994
-5.7616320	3.1231999	102.2699966
-5.7616320	3.1719999	105.6599960
-5.7616320	3.2207999	105.1399994
-5.7616320	3.2695999	103.7900009
-5.7616320	3.3183999	105.0800018
-5.7616320	3.3671999	106.4700012
-5.7616320	3.4159999	102.6100006
-5.7616320	3.4647999	99.6800003
-5.7616320	3.5135999	103.5499954
-5.7616320	3.5623999	103.6899948
-5.7616320	3.6111999	99.8300018
-5.7616320	3.6599998	100.9000015
-5.7616320	3.7087998	103.6800003
-5.7616320	3.7575998	103.4199982
-5.7616320	3.8063998	104.5199966
-5.7616320	3.8551998	105.1899948
-5.7616320	3.9039998	102.9799957
-5.7616320	3.9527998	103.3199997
-5.7616320	4.0016003	104.9799957
-5.7616320	4.0503998	101.0499954
-5.7616320	4.0991998	99.3600006
-5.7616320	4.1479998	104.0000000
-5.7616320	4.1967998	102.7099991
-5.7616320	4.2455997	100.7099991
-5.7616320	4.2943997	105.7600021

-5.7616320	4.3431997	107.6999969
-5.7616320	4.3919997	104.8199997
-5.7616320	4.4407997	106.5299988
-5.7616320	4.4895997	108.4099960
-5.7616320	4.5383997	109.0299988
-5.7616320	4.5871997	109.1500015
-5.7616320	4.6359997	110.1800003
-5.7616320	4.6847997	113.4499969
-5.7616320	4.7336001	113.3699951
-5.7616320	4.7824001	109.2099991
-5.7616320	4.8312001	104.7299957
-5.7616320	4.8800001	110.1299973
-5.7616320	4.9288001	108.7799988
-5.7616320	4.9776001	103.8899994
-5.7616320	5.0264001	99.3600006
-5.7616320	5.0752001	100.3399963
-5.7616320	5.1240001	104.4700012
-5.7616320	5.1728001	105.7099991
-5.7616320	5.2216001	103.9399948
-5.7616320	5.2704000	104.6999969
-5.7616320	5.3192000	106.0299988
-5.7616320	5.3680000	107.1899948
-5.7616320	5.4168000	106.7099991
-5.7616320	5.4656000	101.2799988
-5.7616320	5.5144000	103.1699982
-5.7616320	5.5632000	103.1399994
-5.7616320	5.6120000	103.2200012
-5.7616320	5.6608000	103.2500000
-5.7616320	5.7096000	102.5599976
-5.7616320	5.7584000	101.6599960
-5.7616320	5.8072000	104.0599976
-5.7616320	5.8559999	105.1899948
-5.7616320	5.9047999	107.0800018
-5.7616320	5.9535999	106.5499954
-5.7616320	6.0023999	104.9499969
-5.7616320	6.0511999	105.0599976
-5.7616320	6.0999999	105.7099991
-5.7616320	6.1487999	105.1500015
-5.7616320	6.1975999	104.2500000
-5.7616320	6.2463999	104.9899979
-5.7616320	6.2951999	100.4000015
-5.7616320	6.3439999	104.4499969
-5.7616320	6.3927999	104.1800003
-5.7616320	6.4415998	107.1999969
-5.7616320	6.4903998	107.9300003
-5.7616320	6.5391998	106.2299957
-5.7616320	6.5879998	102.1899948
-5.7616320	6.6367998	100.1699982
-5.7616320	6.6855998	101.6500015
-5.7616320	6.7343998	102.3799973
-5.7616320	6.7831998	103.0800018
-5.7616320	6.8319998	101.8799973
-5.7616320	6.8807998	99.5599976
-5.7616320	6.9295998	99.3799973
-5.7616320	6.9783998	99.5499954
-5.7616320	7.0271997	105.1399994
-5.7616320	7.0759997	103.3799973
-5.7616320	7.1247997	103.3499985

-5.7616320	7.1735997	100.2299957
-5.7616320	7.2223997	103.9099960
-5.7616320	7.2711997	103.9499969
-5.7616320	7.3199997	102.0699997
-5.7616320	7.3687997	104.7900009
-5.7616320	7.4175997	104.3699951
-5.7616320	7.4663997	106.1100006
-5.7616320	7.5151997	105.0100021
-5.7616320	7.5639997	103.3499985
-5.7616320	7.6127996	103.9499969
-5.7616320	7.6615996	104.8799973
-5.7616320	7.7103996	105.1500015
-5.7616320	7.7591996	102.0299988
-5.7616320	7.8079996	101.0699997
-5.7616320	7.8567996	100.7099991
-5.7616320	7.9056001	101.9799957
-5.7616320	7.9544001	105.2999954
-5.7616320	8.0032005	107.7600021
-5.7616320	8.0520000	108.0599976
-5.7616320	8.1008005	106.6800003
-5.7616320	8.1496000	103.2799988
-5.7616320	8.1984005	106.2500000
-5.7616320	8.2472000	107.4599991
-5.7616320	8.2960005	104.5100021
-5.7616320	8.3448000	103.2200012
-5.7616320	8.3936005	103.8399963
-5.7616320	8.4424000	104.2200012
-5.7616320	8.4912004	103.1999969
-5.7616320	8.5400000	102.5299988
-5.7616320	8.5888004	103.5299988
-5.7616320	8.6375999	104.1500015
-5.7616320	8.6864004	105.4700012
-5.7616320	8.7351999	105.7099991
-5.7616320	8.7840004	103.7299957
-5.7616320	8.8327999	104.1899948
-5.7616320	8.8816004	104.1699982
-5.7616320	8.9303999	104.3499985
-5.7616320	8.9792004	105.9099960
-5.7616320	9.0279999	107.4300003
-5.7616320	9.0768003	106.5400009
-5.7616320	9.1255999	104.0699997
-5.7616320	9.1744003	102.9099960
-5.7616320	9.2231998	101.0800018
-5.7616320	9.2720003	99.1399994
-5.7616320	9.3207998	100.6299973
-5.7616320	9.3696003	101.6999969
-5.7616320	9.4184008	103.8799973
-5.7616320	9.4672003	103.7699966
-5.7616320	9.5160007	102.8499985
-5.7616320	9.5648003	103.5400009
-5.7616320	9.6136007	103.2299957
-5.7616320	9.6624002	103.2999954
-5.7616320	9.7112007	103.2399979
-5.7616320	9.7600002	103.2299957
-5.7616320	9.8088007	103.6299973
-5.7616320	9.8576002	102.6800003
-5.7616320	9.9064007	101.5000000
-5.7616320	9.9552002	102.9499969

-5.7616320	10.0040007	101.1599960
-5.7616320	10.0528002	103.0599976
-5.7616320	10.1016006	101.3300018
-5.7616320	10.1504002	101.0299988
-5.7616320	10.1992006	102.3799973
-5.7616320	10.2480001	101.8300018
-5.7616320	10.2968006	104.5100021
-5.7616320	10.3456001	102.3099976
-5.7616320	10.3944006	99.7699966
-5.7616320	10.4432001	99.8499985
-5.7616320	10.4920006	103.1599960
-5.7616320	10.5408001	104.5400009
-5.7616320	10.5896006	104.9599991
-5.7616320	10.6384001	102.5000000
-5.7616320	10.6872005	106.0800018
-5.7616320	10.7360001	105.9000015
-5.7616320	10.7848005	103.4499969
-5.7616320	10.8336000	104.8300018
-5.7616320	10.8824005	104.9099960
-5.7616320	10.9312000	103.8600006
-5.7616320	10.9800005	102.5100021
-5.7616320	11.0288000	103.5699997
-5.7616320	11.0776005	104.0199966
-5.7616320	11.1264000	105.5299988
-5.7616320	11.1752005	102.5800018
-5.7616320	11.2240000	103.6299973
-5.7616320	11.2728004	104.4599991
-5.7616320	11.3216000	100.7799988
-5.7616320	11.3704004	102.1800003
-5.7616320	11.4191999	99.9199982
-5.7616320	11.4680004	105.4899979
-5.7616320	11.5167999	106.4199982
-5.7616320	11.5656004	107.5199966
-5.7616320	11.6143999	107.3199997
-5.7616320	11.6632004	105.5100021
-5.7616320	11.7119999	103.2299957
-5.7616320	11.7608004	103.5999985
-5.7616320	11.8095999	104.7500000
-5.7616320	11.8584003	104.7900009
-5.7616320	11.9071999	103.5699997
-5.7616320	11.9560003	101.1599960
-5.7616320	12.0047998	101.5400009
-5.7616320	12.0536003	102.2299957
-5.7616320	12.1023998	102.7600021
-5.7616320	12.1512003	103.9099960
-5.7616320	12.1999998	104.0999985
-5.7616320	12.2488003	99.7900009
-5.7616320	12.2975998	103.9700012
-5.7616320	12.3464003	106.6800003
-5.7616320	12.3951998	106.5800018
-5.7616320	12.4440002	103.1599960
-5.7616320	12.4927998	102.3199997
-5.7616320	12.5416002	101.5100021
-5.7616320	12.5904007	101.2999954
-5.7616320	12.6392002	102.6199951
-5.7616320	12.6880007	104.2799988
-5.7616320	12.7368002	102.7200012
-5.7616320	12.7856007	100.8399963

-5.7616320	12.8344002	101.1999969
-5.7616320	12.8832006	103.7200012
-5.7616320	12.9320002	102.4099960
-5.7616320	12.9808006	101.0100021
-5.7616320	13.0296001	104.1500015
-5.7616320	13.0784006	102.9599991
-5.7616320	13.1272001	104.6599960
-5.7616320	13.1760006	103.7999954
-5.7616320	13.2248001	101.1199951
-5.7616320	13.2736006	100.0299988
-5.7616320	13.3224001	100.4499969
-5.7616320	13.3712006	98.9899979
-5.7616320	13.4200001	101.7299957
-5.7616320	13.4688005	102.3499985
-5.7616320	13.5176001	101.4799957
-5.7616320	13.5664005	101.7099991
-5.7616320	13.6152000	103.4700012
-5.7616320	13.6640005	103.2399979
-5.7616320	13.7128000	97.9700012
-5.7616320	13.7616005	102.2399979
-5.7616320	13.8104000	103.0599976
-5.7616320	13.8592005	102.9700012
-5.7616320	13.9080000	98.8799973
-5.7616320	13.9568005	103.5000000
-5.7616320	14.0056000	103.2200012
-5.7616320	14.0544004	102.0800018
-5.7616320	14.1032000	102.4099960
-5.7616320	14.1520004	100.8600006
-5.7616320	14.2007999	102.1599960
-5.7616320	14.2496004	101.6299973
-5.7616320	14.2983999	102.7099991
-5.7616320	14.3472004	103.8499985
-5.7616320	14.3959999	102.5999985
-5.7616320	14.4448004	103.6899948
-5.7616320	14.4935999	102.6800003
-5.7616320	14.5424004	103.6800003
-5.7616320	14.5911999	104.8699951
-5.7616320	14.6400003	104.6100006
-5.7616320	14.6887999	105.5899963
-5.7616320	14.7376003	102.9700012
-5.7616320	14.7863998	107.9599991
-5.7616320	14.8352003	104.6100006
-5.7616320	14.8839998	103.5100021
-5.7616320	14.9328003	103.7399979
-5.7616320	14.9815998	102.7600021
-5.7616320	15.0304003	100.0299988
-5.7554960	0.3416000	107.4799957
-5.7554960	0.3904000	107.9599991
-5.7554960	0.4392000	106.5999985
-5.7554960	0.4880000	101.2999954
-5.7554960	0.5368000	106.6299973
-5.7554960	0.5856000	107.4399948
-5.7554960	0.6344000	106.5100021
-5.7554960	0.6832000	108.1599960
-5.7554960	0.7320000	108.2099991
-5.7554960	0.7808000	102.8300018
-5.7554960	0.8296000	100.9000015
-5.7554960	0.8784000	106.0400009

-5.7554960	0.9272000	109.5100021
-5.7554960	0.9760000	109.2900009
-5.7554960	1.0247999	104.0999985
-5.7554960	1.0735999	102.8699951
-5.7554960	1.1223999	103.0400009
-5.7554960	1.1711999	105.1299973
-5.7554960	1.2200000	103.9599991
-5.7554960	1.2688000	100.2600021
-5.7554960	1.3176000	103.9199982
-5.7554960	1.3664000	105.6299973
-5.7554960	1.4152000	109.0400009
-5.7554960	1.4640000	108.1199951
-5.7554960	1.5128000	107.5800018
-5.7554960	1.5616000	105.8399963
-5.7554960	1.6104000	105.1199951
-5.7554960	1.6592000	107.8099976
-5.7554960	1.7079999	107.9300003
-5.7554960	1.7567999	108.2799988
-5.7554960	1.8055999	108.1999969
-5.7554960	1.8543999	109.5699997
-5.7554960	1.9031999	112.1999969
-5.7554960	1.9519999	110.6399994
-5.7554960	2.0007999	107.4399948
-5.7554960	2.0495999	103.4099960
-5.7554960	2.0983999	103.4899979
-5.7554960	2.1471999	103.7999954
-5.7554960	2.1959999	106.2500000
-5.7554960	2.2447999	107.7099991
-5.7554960	2.2935998	105.3899994
-5.7554960	2.3423998	103.6500015
-5.7554960	2.3912001	104.5299988
-5.7554960	2.4400001	105.2600021
-5.7554960	2.4888000	104.2699966
-5.7554960	2.5376000	104.5299988
-5.7554960	2.5864000	106.3399963
-5.7554960	2.6352000	105.4499969
-5.7554960	2.6840000	106.5299988
-5.7554960	2.7328000	104.3499985
-5.7554960	2.7816000	102.6899948
-5.7554960	2.8304000	100.5599976
-5.7554960	2.8792000	102.5800018
-5.7554960	2.9280000	104.0599976
-5.7554960	2.9768000	103.2299957
-5.7554960	3.0256000	106.0899963
-5.7554960	3.0743999	106.0000000
-5.7554960	3.1231999	100.8600006
-5.7554960	3.1719999	104.8399963
-5.7554960	3.2207999	104.6699982
-5.7554960	3.2695999	103.0499954
-5.7554960	3.3183999	104.8499985
-5.7554960	3.3671999	106.4799957
-5.7554960	3.4159999	102.4499969
-5.7554960	3.4647999	100.1199951
-5.7554960	3.5135999	104.5999985
-5.7554960	3.5623999	104.2399979
-5.7554960	3.6111999	101.0499954
-5.7554960	3.6599998	102.0800018
-5.7554960	3.7087998	103.1299973

-5.7554960	3.7575998	102.5699997
-5.7554960	3.8063998	105.0100021
-5.7554960	3.8551998	105.9099960
-5.7554960	3.9039998	103.6599960
-5.7554960	3.9527998	103.9199982
-5.7554960	4.0016003	105.5899963
-5.7554960	4.0503998	101.3600006
-5.7554960	4.0991998	100.0000000
-5.7554960	4.1479998	102.7299957
-5.7554960	4.1967998	101.9399948
-5.7554960	4.2455997	99.5999985
-5.7554960	4.2943997	105.4099960
-5.7554960	4.3431997	107.8699951
-5.7554960	4.3919997	106.3600006
-5.7554960	4.4407997	106.7699966
-5.7554960	4.4895997	108.6999969
-5.7554960	4.5383997	109.5400009
-5.7554960	4.5871997	109.7200012
-5.7554960	4.6359997	110.4099960
-5.7554960	4.6847997	114.6100006
-5.7554960	4.7336001	114.6100006
-5.7554960	4.7824001	109.3300018
-5.7554960	4.8312001	104.5599976
-5.7554960	4.8800001	109.7200012
-5.7554960	4.9288001	108.6699982
-5.7554960	4.9776001	103.2699966
-5.7554960	5.0264001	99.5400009
-5.7554960	5.0752001	100.2399979
-5.7554960	5.1240001	103.3499985
-5.7554960	5.1728001	104.7799988
-5.7554960	5.2216001	103.8799973
-5.7554960	5.2704000	103.4499969
-5.7554960	5.3192000	106.2600021
-5.7554960	5.3680000	107.7500000
-5.7554960	5.4168000	106.7600021
-5.7554960	5.4656000	100.5599976
-5.7554960	5.5144000	103.8699951
-5.7554960	5.5632000	103.5699997
-5.7554960	5.6120000	103.7299957
-5.7554960	5.6608000	103.5800018
-5.7554960	5.7096000	102.3399963
-5.7554960	5.7584000	101.4199982
-5.7554960	5.8072000	103.5699997
-5.7554960	5.8559999	104.4700012
-5.7554960	5.9047999	106.4099960
-5.7554960	5.9535999	106.2299957
-5.7554960	6.0023999	104.5800018
-5.7554960	6.0511999	104.9899979
-5.7554960	6.0999999	105.0999985
-5.7554960	6.1487999	104.0999985
-5.7554960	6.1975999	104.0199966
-5.7554960	6.2463999	103.8099976
-5.7554960	6.2951999	98.5499954
-5.7554960	6.3439999	103.6599960
-5.7554960	6.3927999	103.9000015
-5.7554960	6.4415998	106.6100006
-5.7554960	6.4903998	107.4300003
-5.7554960	6.5391998	106.0699997

-5.7554960	6.5879998	102.4599991
-5.7554960	6.6367998	99.8300018
-5.7554960	6.6855998	100.6299973
-5.7554960	6.7343998	101.0999985
-5.7554960	6.7831998	101.8399963
-5.7554960	6.8319998	100.4799957
-5.7554960	6.8807998	99.8899994
-5.7554960	6.9295998	99.9199982
-5.7554960	6.9783998	100.2399979
-5.7554960	7.0271997	105.3699951
-5.7554960	7.0759997	104.0899963
-5.7554960	7.1247997	102.8300018
-5.7554960	7.1735997	99.2299957
-5.7554960	7.2223997	103.5800018
-5.7554960	7.2711997	104.5400009
-5.7554960	7.3199997	102.9499969
-5.7554960	7.3687997	104.5100021
-5.7554960	7.4175997	103.2900009
-5.7554960	7.4663997	104.9599991
-5.7554960	7.5151997	104.3899994
-5.7554960	7.5639997	104.2099991
-5.7554960	7.6127996	104.5499954
-5.7554960	7.6615996	105.1100006
-5.7554960	7.7103996	105.5899963
-5.7554960	7.7591996	102.0499954
-5.7554960	7.8079996	101.3300018
-5.7554960	7.8567996	101.1899948
-5.7554960	7.9056001	102.5100021
-5.7554960	7.9544001	104.5800018
-5.7554960	8.0032005	106.9700012
-5.7554960	8.0520000	108.1699982
-5.7554960	8.1008005	106.1599960
-5.7554960	8.1496000	102.2900009
-5.7554960	8.1984005	105.1800003
-5.7554960	8.2472000	107.2399979
-5.7554960	8.2960005	104.2600021
-5.7554960	8.3448000	103.8099976
-5.7554960	8.3936005	105.0000000
-5.7554960	8.4424000	104.9199982
-5.7554960	8.4912004	102.1999969
-5.7554960	8.5400000	101.4099960
-5.7554960	8.5888004	103.7099991
-5.7554960	8.6375999	104.7099991
-5.7554960	8.6864004	105.6599960
-5.7554960	8.7351999	105.6199951
-5.7554960	8.7840004	104.2600021
-5.7554960	8.8327999	104.3600006
-5.7554960	8.8816004	104.1500015
-5.7554960	8.9303999	104.2900009
-5.7554960	8.9792004	106.3399963
-5.7554960	9.0279999	108.0199966
-5.7554960	9.0768003	106.9099960
-5.7554960	9.1255999	104.5100021
-5.7554960	9.1744003	103.3799973
-5.7554960	9.2231998	101.7900009
-5.7554960	9.2720003	99.3099976
-5.7554960	9.3207998	100.2500000
-5.7554960	9.3696003	102.2099991

-5.7554960	9.4184008	104.4399948
-5.7554960	9.4672003	102.9000015
-5.7554960	9.5160007	102.7799988
-5.7554960	9.5648003	102.9399948
-5.7554960	9.6136007	102.1399994
-5.7554960	9.6624002	102.9399948
-5.7554960	9.7112007	103.8699951
-5.7554960	9.7600002	104.5699997
-5.7554960	9.8088007	105.0299988
-5.7554960	9.8576002	103.8399963
-5.7554960	9.9064007	102.1800003
-5.7554960	9.9552002	102.4799957
-5.7554960	10.0040007	101.4099960
-5.7554960	10.0528002	103.2799988
-5.7554960	10.1016006	101.5599976
-5.7554960	10.1504002	101.0999985
-5.7554960	10.1992006	102.0400009
-5.7554960	10.2480001	100.7299957
-5.7554960	10.2968006	104.0100021
-5.7554960	10.3456001	100.8399963
-5.7554960	10.3944006	99.7099991
-5.7554960	10.4432001	100.6299973
-5.7554960	10.4920006	103.6500015
-5.7554960	10.5408001	104.4499969
-5.7554960	10.5896006	104.6699982
-5.7554960	10.6384001	101.0999985
-5.7554960	10.6872005	105.8499985
-5.7554960	10.7360001	106.0100021
-5.7554960	10.7848005	103.4199982
-5.7554960	10.8336000	104.6599960
-5.7554960	10.8824005	104.9099960
-5.7554960	10.9312000	103.0999985
-5.7554960	10.9800005	101.8899994
-5.7554960	11.0288000	102.4799957
-5.7554960	11.0776005	102.7099991
-5.7554960	11.1264000	104.6899948
-5.7554960	11.1752005	103.6100006
-5.7554960	11.2240000	103.8300018
-5.7554960	11.2728004	104.5199966
-5.7554960	11.3216000	102.1500015
-5.7554960	11.3704004	103.3799973
-5.7554960	11.4191999	101.1100006
-5.7554960	11.4680004	105.7799988
-5.7554960	11.5167999	106.3600006
-5.7554960	11.5656004	106.7600021
-5.7554960	11.6143999	106.0400009
-5.7554960	11.6632004	104.5400009
-5.7554960	11.7119999	103.2900009
-5.7554960	11.7608004	104.3300018
-5.7554960	11.8095999	104.4199982
-5.7554960	11.8584003	104.6999969
-5.7554960	11.9071999	103.7799988
-5.7554960	11.9560003	102.2099991
-5.7554960	12.0047998	101.2600021
-5.7554960	12.0536003	100.3699951
-5.7554960	12.1023998	101.2500000
-5.7554960	12.1512003	103.6699982
-5.7554960	12.1999998	104.1299973

-5.7554960	12.2488003	100.7299957
-5.7554960	12.2975998	104.0999985
-5.7554960	12.3464003	107.0299988
-5.7554960	12.3951998	106.4399948
-5.7554960	12.4440002	102.2299957
-5.7554960	12.4927998	102.6599960
-5.7554960	12.5416002	102.9199982
-5.7554960	12.5904007	101.9499969
-5.7554960	12.6392002	101.5400009
-5.7554960	12.6880007	103.2299957
-5.7554960	12.7368002	103.0100021
-5.7554960	12.7856007	101.2900009
-5.7554960	12.8344002	101.1999969
-5.7554960	12.8832006	103.5999985
-5.7554960	12.9320002	102.4300003
-5.7554960	12.9808006	100.6800003
-5.7554960	13.0296001	103.8899994
-5.7554960	13.0784006	102.6399994
-5.7554960	13.1272001	105.0899963
-5.7554960	13.1760006	104.2799988
-5.7554960	13.2248001	101.6999969
-5.7554960	13.2736006	100.2099991
-5.7554960	13.3224001	100.7099991
-5.7554960	13.3712006	99.2200012
-5.7554960	13.4200001	102.0000000
-5.7554960	13.4688005	101.5100021
-5.7554960	13.5176001	100.6100006
-5.7554960	13.5664005	100.8499985
-5.7554960	13.6152000	102.7200012
-5.7554960	13.6640005	102.0999985
-5.7554960	13.7128000	96.4399948
-5.7554960	13.7616005	101.4700012
-5.7554960	13.8104000	102.0299988
-5.7554960	13.8592005	102.1399994
-5.7554960	13.9080000	99.1199951
-5.7554960	13.9568005	104.1699982
-5.7554960	14.0056000	103.5999985
-5.7554960	14.0544004	102.2299957
-5.7554960	14.1032000	102.6399994
-5.7554960	14.1520004	101.7900009
-5.7554960	14.2007999	103.4000015
-5.7554960	14.2496004	102.8899994
-5.7554960	14.2983999	102.6599960
-5.7554960	14.3472004	102.8600006
-5.7554960	14.3959999	102.5800018
-5.7554960	14.4448004	104.1699982
-5.7554960	14.4935999	103.7500000
-5.7554960	14.5424004	103.9399948
-5.7554960	14.5911999	104.6299973
-5.7554960	14.6400003	104.6500015
-5.7554960	14.6887999	105.3099976
-5.7554960	14.7376003	102.5400009
-5.7554960	14.7863998	107.2099991
-5.7554960	14.8352003	103.4099960
-5.7554960	14.8839998	103.0800018
-5.7554960	14.9328003	103.1500015
-5.7554960	14.9815998	101.5000000
-5.7554960	15.0304003	98.6599960

-5.7493601	0.3416000	108.2900009
-5.7493601	0.3904000	108.8699951
-5.7493601	0.4392000	107.7500000
-5.7493601	0.4880000	102.5499954
-5.7493601	0.5368000	106.4399948
-5.7493601	0.5856000	107.1399994
-5.7493601	0.6344000	106.9399948
-5.7493601	0.6832000	107.6800003
-5.7493601	0.7320000	107.4199982
-5.7493601	0.7808000	104.0699997
-5.7493601	0.8296000	102.1299973
-5.7493601	0.8784000	105.0199966
-5.7493601	0.9272000	108.8600006
-5.7493601	0.9760000	108.3699951
-5.7493601	1.0247999	102.3699951
-5.7493601	1.0735999	102.5699997
-5.7493601	1.1223999	103.0400009
-5.7493601	1.1711999	104.3699951
-5.7493601	1.2200000	103.4300003
-5.7493601	1.2688000	101.5800018
-5.7493601	1.3176000	104.9300003
-5.7493601	1.3664000	105.5899963
-5.7493601	1.4152000	109.1800003
-5.7493601	1.4640000	108.4199982
-5.7493601	1.5128000	107.3099976
-5.7493601	1.5616000	106.0400009
-5.7493601	1.6104000	104.9399948
-5.7493601	1.6592000	107.9300003
-5.7493601	1.7079999	108.5999985
-5.7493601	1.7567999	108.6500015
-5.7493601	1.8055999	107.5100021
-5.7493601	1.8543999	108.8799973
-5.7493601	1.9031999	111.9700012
-5.7493601	1.9519999	110.4199982
-5.7493601	2.0007999	106.5999985
-5.7493601	2.0495999	102.5000000
-5.7493601	2.0983999	103.0699997
-5.7493601	2.1471999	103.6399994
-5.7493601	2.1959999	105.7799988
-5.7493601	2.2447999	107.5699997
-5.7493601	2.2935998	105.3699951
-5.7493601	2.3423998	104.9099960
-5.7493601	2.3912001	105.2699966
-5.7493601	2.4400001	105.3099976
-5.7493601	2.4888000	105.2399979
-5.7493601	2.5376000	104.7299957
-5.7493601	2.5864000	106.7999954
-5.7493601	2.6352000	106.6999969
-5.7493601	2.6840000	106.2099991
-5.7493601	2.7328000	103.5800018
-5.7493601	2.7816000	104.0999985
-5.7493601	2.8304000	101.9899979
-5.7493601	2.8792000	103.0000000
-5.7493601	2.9280000	103.8300018
-5.7493601	2.9768000	102.6999969
-5.7493601	3.0256000	105.7699966
-5.7493601	3.0743999	105.6699982
-5.7493601	3.1231999	101.4599991

-5.7493601	3.1719999	104.4899979
-5.7493601	3.2207999	104.1599960
-5.7493601	3.2695999	101.8600006
-5.7493601	3.3183999	104.4099960
-5.7493601	3.3671999	105.9499969
-5.7493601	3.4159999	101.5999985
-5.7493601	3.4647999	101.8699951
-5.7493601	3.5135999	105.5400009
-5.7493601	3.5623999	104.9199982
-5.7493601	3.6111999	103.1999969
-5.7493601	3.6599998	103.2399979
-5.7493601	3.7087998	102.0100021
-5.7493601	3.7575998	101.5299988
-5.7493601	3.8063998	105.4799957
-5.7493601	3.8551998	106.2099991
-5.7493601	3.9039998	103.7600021
-5.7493601	3.9527998	103.9599991
-5.7493601	4.0016003	105.7900009
-5.7493601	4.0503998	101.3699951
-5.7493601	4.0991998	100.6599960
-5.7493601	4.1479998	102.0199966
-5.7493601	4.1967998	101.8399963
-5.7493601	4.2455997	99.4599991
-5.7493601	4.2943997	105.4499969
-5.7493601	4.3431997	107.7600021
-5.7493601	4.3919997	105.8099976
-5.7493601	4.4407997	105.8300018
-5.7493601	4.4895997	108.1699982
-5.7493601	4.5383997	110.0400009
-5.7493601	4.5871997	110.2799988
-5.7493601	4.6359997	110.2799988
-5.7493601	4.6847997	115.3199997
-5.7493601	4.7336001	115.2600021
-5.7493601	4.7824001	109.5400009
-5.7493601	4.8312001	104.1299973
-5.7493601	4.8800001	108.8899994
-5.7493601	4.9288001	108.2699966
-5.7493601	4.9776001	102.7399979
-5.7493601	5.0264001	100.1299973
-5.7493601	5.0752001	100.7200012
-5.7493601	5.1240001	102.6100006
-5.7493601	5.1728001	104.5499954
-5.7493601	5.2216001	104.4399948
-5.7493601	5.2704000	102.4099960
-5.7493601	5.3192000	106.3300018
-5.7493601	5.3680000	108.3600006
-5.7493601	5.4168000	107.0199966
-5.7493601	5.4656000	100.6299973
-5.7493601	5.5144000	104.8699951
-5.7493601	5.5632000	104.4099960
-5.7493601	5.6120000	104.3499985
-5.7493601	5.6608000	103.2399979
-5.7493601	5.7096000	102.0299988
-5.7493601	5.7584000	101.1800003
-5.7493601	5.8072000	103.3600006
-5.7493601	5.8559999	104.1999969
-5.7493601	5.9047999	106.1100006
-5.7493601	5.9535999	106.5499954

-5.7493601	6.0023999	104.9700012
-5.7493601	6.0511999	105.1500015
-5.7493601	6.0999999	104.1899948
-5.7493601	6.1487999	103.0100021
-5.7493601	6.1975999	104.0100021
-5.7493601	6.2463999	103.2099991
-5.7493601	6.2951999	99.1599960
-5.7493601	6.3439999	102.8499985
-5.7493601	6.3927999	102.8399963
-5.7493601	6.4415998	105.5000000
-5.7493601	6.4903998	106.6299973
-5.7493601	6.5391998	106.0100021
-5.7493601	6.5879998	103.0699997
-5.7493601	6.6367998	99.1999969
-5.7493601	6.6855998	99.1699982
-5.7493601	6.7343998	100.0699997
-5.7493601	6.7831998	100.9399948
-5.7493601	6.8319998	99.2299957
-5.7493601	6.8807998	100.0100021
-5.7493601	6.9295998	99.0100021
-5.7493601	6.9783998	100.8199997
-5.7493601	7.0271997	105.7699966
-5.7493601	7.0759997	105.0499954
-5.7493601	7.1247997	102.4199982
-5.7493601	7.1735997	98.0000000
-5.7493601	7.2223997	102.8099976
-5.7493601	7.2711997	104.5999985
-5.7493601	7.3199997	103.0800018
-5.7493601	7.3687997	103.7900009
-5.7493601	7.4175997	101.4799957
-5.7493601	7.4663997	103.3199997
-5.7493601	7.5151997	104.0499954
-5.7493601	7.5639997	105.0100021
-5.7493601	7.6127996	104.7500000
-5.7493601	7.6615996	104.5899963
-5.7493601	7.7103996	104.7600021
-5.7493601	7.7591996	102.0400009
-5.7493601	7.8079996	101.3799973
-5.7493601	7.8567996	101.7200012
-5.7493601	7.9056001	103.1100006
-5.7493601	7.9544001	104.3699951
-5.7493601	8.0032005	106.3099976
-5.7493601	8.0520000	108.1299973
-5.7493601	8.1008005	105.2399979
-5.7493601	8.1496000	100.5299988
-5.7493601	8.1984005	103.7699966
-5.7493601	8.2472000	107.0499954
-5.7493601	8.2960005	104.6100006
-5.7493601	8.3448000	104.6399994
-5.7493601	8.3936005	105.7600021
-5.7493601	8.4424000	105.2500000
-5.7493601	8.4912004	100.9599991
-5.7493601	8.5400000	99.9000015
-5.7493601	8.5888004	103.4000015
-5.7493601	8.6375999	104.3199997
-5.7493601	8.6864004	106.1599960
-5.7493601	8.7351999	106.1800003
-5.7493601	8.7840004	105.4899979

-5.7493601	8.8327999	105.0199966
-5.7493601	8.8816004	104.0999985
-5.7493601	8.9303999	103.7099991
-5.7493601	8.9792004	106.2900009
-5.7493601	9.0279999	108.0699997
-5.7493601	9.0768003	106.4499969
-5.7493601	9.1255999	105.0499954
-5.7493601	9.1744003	104.1100006
-5.7493601	9.2231998	102.9399948
-5.7493601	9.2720003	100.0000000
-5.7493601	9.3207998	99.9399948
-5.7493601	9.3696003	102.4199982
-5.7493601	9.4184008	104.6199951
-5.7493601	9.4672003	101.9700012
-5.7493601	9.5160007	102.2099991
-5.7493601	9.5648003	102.3199997
-5.7493601	9.6136007	101.2200012
-5.7493601	9.6624002	103.2699966
-5.7493601	9.7112007	104.5699997
-5.7493601	9.7600002	105.6500015
-5.7493601	9.8088007	106.4099960
-5.7493601	9.8576002	105.0899963
-5.7493601	9.9064007	103.1899948
-5.7493601	9.9552002	102.5000000
-5.7493601	10.0040007	101.9300003
-5.7493601	10.0528002	103.2900009
-5.7493601	10.1016006	102.4399948
-5.7493601	10.1504002	101.5299988
-5.7493601	10.1992006	101.8799973
-5.7493601	10.2480001	99.9700012
-5.7493601	10.2968006	103.6500015
-5.7493601	10.3456001	99.1500015
-5.7493601	10.3944006	99.5100021
-5.7493601	10.4432001	101.8600006
-5.7493601	10.4920006	104.0999985
-5.7493601	10.5408001	103.9599991
-5.7493601	10.5896006	104.2600021
-5.7493601	10.6384001	100.3300018
-5.7493601	10.6872005	105.5100021
-5.7493601	10.7360001	105.9199982
-5.7493601	10.7848005	103.2900009
-5.7493601	10.8336000	104.3899994
-5.7493601	10.8824005	104.3899994
-5.7493601	10.9312000	101.9000015
-5.7493601	10.9800005	100.8799973
-5.7493601	11.0288000	100.9899979
-5.7493601	11.0776005	102.1299973
-5.7493601	11.1264000	104.1599960
-5.7493601	11.1752005	104.4899979
-5.7493601	11.2240000	103.9300003
-5.7493601	11.2728004	104.7500000
-5.7493601	11.3216000	103.6100006
-5.7493601	11.3704004	104.5499954
-5.7493601	11.4191999	102.2200012
-5.7493601	11.4680004	105.9700012
-5.7493601	11.5167999	106.4899979
-5.7493601	11.5656004	106.3799973
-5.7493601	11.6143999	105.1699982

-5.7493601	11.6632004	103.9099960
-5.7493601	11.7119999	104.4799957
-5.7493601	11.7608004	105.4099960
-5.7493601	11.8095999	103.7699966
-5.7493601	11.8584003	104.3399963
-5.7493601	11.9071999	103.8399963
-5.7493601	11.9560003	103.1899948
-5.7493601	12.0047998	101.8199997
-5.7493601	12.0536003	99.9300003
-5.7493601	12.1023998	100.4000015
-5.7493601	12.1512003	103.0599976
-5.7493601	12.1999998	104.0699997
-5.7493601	12.2488003	101.4700012
-5.7493601	12.2975998	104.0599976
-5.7493601	12.3464003	106.9199982
-5.7493601	12.3951998	105.9599991
-5.7493601	12.4440002	101.4599991
-5.7493601	12.4927998	103.3300018
-5.7493601	12.5416002	103.7600021
-5.7493601	12.5904007	101.9199982
-5.7493601	12.6392002	100.9000015
-5.7493601	12.6880007	101.8099976
-5.7493601	12.7368002	103.2799988
-5.7493601	12.7856007	101.9700012
-5.7493601	12.8344002	101.5499954
-5.7493601	12.8832006	102.8600006
-5.7493601	12.9320002	102.6899948
-5.7493601	12.9808006	100.5499954
-5.7493601	13.0296001	103.8399963
-5.7493601	13.0784006	103.1699982
-5.7493601	13.1272001	105.8300018
-5.7493601	13.1760006	104.9399948
-5.7493601	13.2248001	102.6100006
-5.7493601	13.2736006	100.6299973
-5.7493601	13.3224001	101.3799973
-5.7493601	13.3712006	100.7299957
-5.7493601	13.4200001	102.8199997
-5.7493601	13.4688005	101.1999969
-5.7493601	13.5176001	100.4899979
-5.7493601	13.5664005	100.7699966
-5.7493601	13.6152000	102.8899994
-5.7493601	13.6640005	102.1399994
-5.7493601	13.7128000	96.6399994
-5.7493601	13.7616005	100.9399948
-5.7493601	13.8104000	101.0699997
-5.7493601	13.8592005	101.3600006
-5.7493601	13.9080000	99.1999969
-5.7493601	13.9568005	104.4000015
-5.7493601	14.0056000	103.9199982
-5.7493601	14.0544004	102.1699982
-5.7493601	14.1032000	102.1199951
-5.7493601	14.1520004	102.0800018
-5.7493601	14.2007999	104.2399979
-5.7493601	14.2496004	103.6899948
-5.7493601	14.2983999	102.1800003
-5.7493601	14.3472004	101.7999954
-5.7493601	14.3959999	102.8300018
-5.7493601	14.4448004	104.1699982

-5.7493601	14.4935999	104.2699966
-5.7493601	14.5424004	103.7299957
-5.7493601	14.5911999	104.2900009
-5.7493601	14.6400003	104.5699997
-5.7493601	14.6887999	104.8499985
-5.7493601	14.7376003	102.0999985
-5.7493601	14.7863998	106.2200012
-5.7493601	14.8352003	102.1599960
-5.7493601	14.8839998	102.6899948
-5.7493601	14.9328003	102.6699982
-5.7493601	14.9815998	100.9599991
-5.7493601	15.0304003	97.6199951
-5.7432241	0.3416000	109.0400009
-5.7432241	0.3904000	109.6699982
-5.7432241	0.4392000	108.6199951
-5.7432241	0.4880000	103.4000015
-5.7432241	0.5368000	105.9399948
-5.7432241	0.5856000	106.5699997
-5.7432241	0.6344000	107.1199951
-5.7432241	0.6832000	106.9099960
-5.7432241	0.7320000	106.9899979
-5.7432241	0.7808000	105.7699966
-5.7432241	0.8296000	103.5999985
-5.7432241	0.8784000	104.0800018
-5.7432241	0.9272000	107.9399948
-5.7432241	0.9760000	107.3899994
-5.7432241	1.0247999	101.8300018
-5.7432241	1.0735999	102.9599991
-5.7432241	1.1223999	103.1100006
-5.7432241	1.1711999	103.1399994
-5.7432241	1.2200000	103.1399994
-5.7432241	1.2688000	103.0699997
-5.7432241	1.3176000	105.9799957
-5.7432241	1.3664000	105.3099976
-5.7432241	1.4152000	109.1800003
-5.7432241	1.4640000	108.7699966
-5.7432241	1.5128000	107.1199951
-5.7432241	1.5616000	105.6399994
-5.7432241	1.6104000	104.3499985
-5.7432241	1.6592000	106.9199982
-5.7432241	1.7079999	108.1199951
-5.7432241	1.7567999	108.3099976
-5.7432241	1.8055999	107.0699997
-5.7432241	1.8543999	108.7299957
-5.7432241	1.9031999	111.9599991
-5.7432241	1.9519999	110.0800018
-5.7432241	2.0007999	105.4000015
-5.7432241	2.0495999	101.3600006
-5.7432241	2.0983999	102.8600006
-5.7432241	2.1471999	103.9700012
-5.7432241	2.1959999	105.5999985
-5.7432241	2.2447999	107.4099960
-5.7432241	2.2935998	104.7299957
-5.7432241	2.3423998	105.3699951
-5.7432241	2.3912001	106.2500000
-5.7432241	2.4400001	105.5999985
-5.7432241	2.4888000	106.4399948
-5.7432241	2.5376000	105.1699982

-5.7432241	2.5864000	107.0000000
-5.7432241	2.6352000	107.3300018
-5.7432241	2.6840000	105.9300003
-5.7432241	2.7328000	102.7699966
-5.7432241	2.7816000	104.7699966
-5.7432241	2.8304000	102.5800018
-5.7432241	2.8792000	103.1800003
-5.7432241	2.9280000	104.2399979
-5.7432241	2.9768000	103.3399963
-5.7432241	3.0256000	105.4700012
-5.7432241	3.0743999	105.0100021
-5.7432241	3.1231999	101.9899979
-5.7432241	3.1719999	104.0199966
-5.7432241	3.2207999	102.7600021
-5.7432241	3.2695999	100.1199951
-5.7432241	3.3183999	104.1999969
-5.7432241	3.3671999	105.8899994
-5.7432241	3.4159999	101.2099991
-5.7432241	3.4647999	103.2900009
-5.7432241	3.5135999	106.0699997
-5.7432241	3.5623999	105.2099991
-5.7432241	3.6111999	104.9799957
-5.7432241	3.6599998	104.2099991
-5.7432241	3.7087998	100.9799957
-5.7432241	3.7575998	102.0000000
-5.7432241	3.8063998	106.4000015
-5.7432241	3.8551998	106.0899963
-5.7432241	3.9039998	103.5299988
-5.7432241	3.9527998	104.3199997
-5.7432241	4.0016003	105.5599976
-5.7432241	4.0503998	101.0400009
-5.7432241	4.0991998	101.1800003
-5.7432241	4.1479998	101.2600021
-5.7432241	4.1967998	101.8499985
-5.7432241	4.2455997	99.9599991
-5.7432241	4.2943997	104.8799973
-5.7432241	4.3431997	107.0100021
-5.7432241	4.3919997	104.9199982
-5.7432241	4.4407997	104.8099976
-5.7432241	4.4895997	107.1899948
-5.7432241	4.5383997	109.4599991
-5.7432241	4.5871997	110.0100021
-5.7432241	4.6359997	110.4799957
-5.7432241	4.6847997	115.8600006
-5.7432241	4.7336001	115.5599976
-5.7432241	4.7824001	109.5299988
-5.7432241	4.8312001	103.7500000
-5.7432241	4.8800001	108.1199951
-5.7432241	4.9288001	107.8099976
-5.7432241	4.9776001	102.8199997
-5.7432241	5.0264001	101.1500015
-5.7432241	5.0752001	101.6399994
-5.7432241	5.1240001	101.9599991
-5.7432241	5.1728001	103.9599991
-5.7432241	5.2216001	104.6999969
-5.7432241	5.2704000	101.9399948
-5.7432241	5.3192000	105.9300003
-5.7432241	5.3680000	107.6999969

-5.7432241	5.4168000	106.7799988
-5.7432241	5.4656000	100.4000015
-5.7432241	5.5144000	105.7900009
-5.7432241	5.5632000	105.6599960
-5.7432241	5.6120000	105.0599976
-5.7432241	5.6608000	102.6699982
-5.7432241	5.7096000	101.5499954
-5.7432241	5.7584000	100.9599991
-5.7432241	5.8072000	102.8399963
-5.7432241	5.8559999	103.9499969
-5.7432241	5.9047999	106.4000015
-5.7432241	5.9535999	106.6899948
-5.7432241	6.0023999	104.2699966
-5.7432241	6.0511999	104.3099976
-5.7432241	6.0999999	102.8300018
-5.7432241	6.1487999	102.1299973
-5.7432241	6.1975999	104.2699966
-5.7432241	6.2463999	102.7699966
-5.7432241	6.2951999	100.4099960
-5.7432241	6.3439999	102.4599991
-5.7432241	6.3927999	101.5999985
-5.7432241	6.4415998	104.6399994
-5.7432241	6.4903998	105.9300003
-5.7432241	6.5391998	106.0499954
-5.7432241	6.5879998	104.0400009
-5.7432241	6.6367998	99.4399948
-5.7432241	6.6855998	97.8399963
-5.7432241	6.7343998	99.6399994
-5.7432241	6.7831998	100.5999985
-5.7432241	6.8319998	98.7799988
-5.7432241	6.8807998	100.2099991
-5.7432241	6.9295998	98.2600021
-5.7432241	6.9783998	101.5699997
-5.7432241	7.0271997	105.6800003
-5.7432241	7.0759997	105.6299973
-5.7432241	7.1247997	102.6299973
-5.7432241	7.1735997	98.6100006
-5.7432241	7.2223997	102.8300018
-5.7432241	7.2711997	105.1100006
-5.7432241	7.3199997	103.5599976
-5.7432241	7.3687997	103.6500015
-5.7432241	7.4175997	100.5699997
-5.7432241	7.4663997	101.3300018
-5.7432241	7.5151997	103.7699966
-5.7432241	7.5639997	105.7600021
-5.7432241	7.6127996	105.0000000
-5.7432241	7.6615996	104.3899994
-5.7432241	7.7103996	104.1199951
-5.7432241	7.7591996	102.3300018
-5.7432241	7.8079996	101.8199997
-5.7432241	7.8567996	102.2099991
-5.7432241	7.9056001	103.2799988
-5.7432241	7.9544001	103.9099960
-5.7432241	8.0032005	105.3300018
-5.7432241	8.0520000	107.5499954
-5.7432241	8.1008005	104.2399979
-5.7432241	8.1496000	100.2699966
-5.7432241	8.1984005	102.6500015

-5.7432241	8.2472000	107.2299957
-5.7432241	8.2960005	105.5000000
-5.7432241	8.3448000	105.1899948
-5.7432241	8.3936005	105.9700012
-5.7432241	8.4424000	105.0599976
-5.7432241	8.4912004	99.8199997
-5.7432241	8.5400000	98.3899994
-5.7432241	8.5888004	103.5299988
-5.7432241	8.6375999	103.5499954
-5.7432241	8.6864004	105.7399979
-5.7432241	8.7351999	105.9399948
-5.7432241	8.7840004	106.2399979
-5.7432241	8.8327999	105.5100021
-5.7432241	8.8816004	103.4799957
-5.7432241	8.9303999	103.0699997
-5.7432241	8.9792004	106.2399979
-5.7432241	9.0279999	107.9199982
-5.7432241	9.0768003	106.3499985
-5.7432241	9.1255999	106.1500015
-5.7432241	9.1744003	105.5599976
-5.7432241	9.2231998	104.2699966
-5.7432241	9.2720003	100.4899979
-5.7432241	9.3207998	100.1100006
-5.7432241	9.3696003	102.4700012
-5.7432241	9.4184008	105.3099976
-5.7432241	9.4672003	102.0100021
-5.7432241	9.5160007	102.6299973
-5.7432241	9.5648003	102.9599991
-5.7432241	9.6136007	101.9099960
-5.7432241	9.6624002	104.2200012
-5.7432241	9.7112007	105.3199997
-5.7432241	9.7600002	106.1500015
-5.7432241	9.8088007	106.6500015
-5.7432241	9.8576002	105.7600021
-5.7432241	9.9064007	104.2600021
-5.7432241	9.9552002	102.9399948
-5.7432241	10.0040007	101.2500000
-5.7432241	10.0528002	102.8699951
-5.7432241	10.1016006	103.1399994
-5.7432241	10.1504002	101.5699997
-5.7432241	10.1992006	101.6699982
-5.7432241	10.2480001	100.7999954
-5.7432241	10.2968006	103.2399979
-5.7432241	10.3456001	97.9799957
-5.7432241	10.3944006	98.9599991
-5.7432241	10.4432001	102.1599960
-5.7432241	10.4920006	104.4499969
-5.7432241	10.5408001	102.9000015
-5.7432241	10.5896006	103.4099960
-5.7432241	10.6384001	99.5100021
-5.7432241	10.6872005	105.2099991
-5.7432241	10.7360001	105.9799957
-5.7432241	10.7848005	103.2399979
-5.7432241	10.8336000	104.0800018
-5.7432241	10.8824005	104.1100006
-5.7432241	10.9312000	102.1699982
-5.7432241	10.9800005	100.1500015
-5.7432241	11.0288000	99.4899979

-5.7432241	11.0776005	101.7500000
-5.7432241	11.1264000	103.7200012
-5.7432241	11.1752005	105.2200012
-5.7432241	11.2240000	103.7299957
-5.7432241	11.2728004	104.5599976
-5.7432241	11.3216000	104.4899979
-5.7432241	11.3704004	105.3600006
-5.7432241	11.4191999	103.6899948
-5.7432241	11.4680004	106.2099991
-5.7432241	11.5167999	106.5000000
-5.7432241	11.5656004	105.8399963
-5.7432241	11.6143999	103.9300003
-5.7432241	11.6632004	103.4099960
-5.7432241	11.7119999	105.3600006
-5.7432241	11.7608004	105.9599991
-5.7432241	11.8095999	102.9599991
-5.7432241	11.8584003	104.0800018
-5.7432241	11.9071999	103.2200012
-5.7432241	11.9560003	103.2200012
-5.7432241	12.0047998	102.3399963
-5.7432241	12.0536003	99.4099960
-5.7432241	12.1023998	99.4599991
-5.7432241	12.1512003	102.8199997
-5.7432241	12.1999998	104.3300018
-5.7432241	12.2488003	102.1999969
-5.7432241	12.2975998	104.5599976
-5.7432241	12.3464003	106.7500000
-5.7432241	12.3951998	105.1299973
-5.7432241	12.4440002	100.2799988
-5.7432241	12.4927998	103.2900009
-5.7432241	12.5416002	103.3899994
-5.7432241	12.5904007	101.2200012
-5.7432241	12.6392002	100.0699997
-5.7432241	12.6880007	101.2799988
-5.7432241	12.7368002	104.4099960
-5.7432241	12.7856007	103.4000015
-5.7432241	12.8344002	102.6500015
-5.7432241	12.8832006	102.0499954
-5.7432241	12.9320002	102.8600006
-5.7432241	12.9808006	101.5800018
-5.7432241	13.0296001	103.9700012
-5.7432241	13.0784006	103.1599960
-5.7432241	13.1272001	106.0599976
-5.7432241	13.1760006	105.6399994
-5.7432241	13.2248001	103.9399948
-5.7432241	13.2736006	101.2600021
-5.7432241	13.3224001	102.3600006
-5.7432241	13.3712006	102.1699982
-5.7432241	13.4200001	103.1699982
-5.7432241	13.4688005	100.5199966
-5.7432241	13.5176001	100.4499969
-5.7432241	13.5664005	100.8499985
-5.7432241	13.6152000	103.4399948
-5.7432241	13.6640005	102.8199997
-5.7432241	13.7128000	97.1199951
-5.7432241	13.7616005	99.9499969
-5.7432241	13.8104000	101.2099991
-5.7432241	13.8592005	101.5100021

-5.7432241	13.9080000	99.7900009
-5.7432241	13.9568005	103.8099976
-5.7432241	14.0056000	103.8799973
-5.7432241	14.0544004	101.2200012
-5.7432241	14.1032000	102.2099991
-5.7432241	14.1520004	103.0699997
-5.7432241	14.2007999	104.9499969
-5.7432241	14.2496004	104.5199966
-5.7432241	14.2983999	101.6299973
-5.7432241	14.3472004	100.3499985
-5.7432241	14.3959999	102.6500015
-5.7432241	14.4448004	103.7099991
-5.7432241	14.4935999	104.1199951
-5.7432241	14.5424004	103.1999969
-5.7432241	14.5911999	103.6500015
-5.7432241	14.6400003	104.2699966
-5.7432241	14.6887999	104.5599976
-5.7432241	14.7376003	102.1599960
-5.7432241	14.7863998	105.4199982
-5.7432241	14.8352003	101.0800018
-5.7432241	14.8839998	102.0999985
-5.7432241	14.9328003	101.6999969
-5.7432241	14.9815998	100.4199982
-5.7432241	15.0304003	97.7999954
-5.7370882	0.3416000	109.3300018
-5.7370882	0.3904000	109.9099960
-5.7370882	0.4392000	109.1399994
-5.7370882	0.4880000	104.2299957
-5.7370882	0.5368000	105.9799957
-5.7370882	0.5856000	106.5199966
-5.7370882	0.6344000	107.5599976
-5.7370882	0.6832000	107.2099991
-5.7370882	0.7320000	106.9799957
-5.7370882	0.7808000	107.2299957
-5.7370882	0.8296000	104.9799957
-5.7370882	0.8784000	104.1500015
-5.7370882	0.9272000	107.8699951
-5.7370882	0.9760000	107.2500000
-5.7370882	1.0247999	102.7699966
-5.7370882	1.0735999	103.9000015
-5.7370882	1.1223999	103.2600021
-5.7370882	1.1711999	101.7799988
-5.7370882	1.2200000	102.7699966
-5.7370882	1.2688000	104.6100006
-5.7370882	1.3176000	106.9199982
-5.7370882	1.3664000	104.7799988
-5.7370882	1.4152000	109.0999985
-5.7370882	1.4640000	109.5699997
-5.7370882	1.5128000	107.5800018
-5.7370882	1.5616000	105.2299957
-5.7370882	1.6104000	103.9599991
-5.7370882	1.6592000	105.8199997
-5.7370882	1.7079999	107.0100021
-5.7370882	1.7567999	107.5199966
-5.7370882	1.8055999	106.2099991
-5.7370882	1.8543999	108.3699951
-5.7370882	1.9031999	111.5899963
-5.7370882	1.9519999	109.5499954

-5.7370882	2.0007999	104.3499985
-5.7370882	2.0495999	100.7200012
-5.7370882	2.0983999	102.5100021
-5.7370882	2.1471999	104.0400009
-5.7370882	2.1959999	104.7799988
-5.7370882	2.2447999	107.1199951
-5.7370882	2.2935998	105.1299973
-5.7370882	2.3423998	105.5899963
-5.7370882	2.3912001	106.5199966
-5.7370882	2.4400001	105.1100006
-5.7370882	2.4888000	107.0299988
-5.7370882	2.5376000	105.2799988
-5.7370882	2.5864000	107.2399979
-5.7370882	2.6352000	107.7999954
-5.7370882	2.6840000	105.8600006
-5.7370882	2.7328000	102.2099991
-5.7370882	2.7816000	105.4300003
-5.7370882	2.8304000	103.2099991
-5.7370882	2.8792000	103.5000000
-5.7370882	2.9280000	104.5999985
-5.7370882	2.9768000	103.7999954
-5.7370882	3.0256000	105.4599991
-5.7370882	3.0743999	105.0199966
-5.7370882	3.1231999	103.2600021
-5.7370882	3.1719999	103.7900009
-5.7370882	3.2207999	101.8699951
-5.7370882	3.2695999	100.7799988
-5.7370882	3.3183999	105.1299973
-5.7370882	3.3671999	105.6599960
-5.7370882	3.4159999	101.2900009
-5.7370882	3.4647999	104.7099991
-5.7370882	3.5135999	105.7799988
-5.7370882	3.5623999	104.8499985
-5.7370882	3.6111999	106.1699982
-5.7370882	3.6599998	104.0499954
-5.7370882	3.7087998	100.0299988
-5.7370882	3.7575998	101.6800003
-5.7370882	3.8063998	106.1399994
-5.7370882	3.8551998	105.6199951
-5.7370882	3.9039998	104.2799988
-5.7370882	3.9527998	105.0100021
-5.7370882	4.0016003	105.5899963
-5.7370882	4.0503998	101.2399979
-5.7370882	4.0991998	102.2399979
-5.7370882	4.1479998	100.8899994
-5.7370882	4.1967998	102.2399979
-5.7370882	4.2455997	101.1199951
-5.7370882	4.2943997	105.2399979
-5.7370882	4.3431997	106.6299973
-5.7370882	4.3919997	104.4899979
-5.7370882	4.4407997	103.5699997
-5.7370882	4.4895997	106.2399979
-5.7370882	4.5383997	108.5899963
-5.7370882	4.5871997	109.6599960
-5.7370882	4.6359997	110.5800018
-5.7370882	4.6847997	115.9399948
-5.7370882	4.7336001	115.1699982
-5.7370882	4.7824001	108.6999969

-5.7370882	4.8312001	103.5000000
-5.7370882	4.8800001	107.0699997
-5.7370882	4.9288001	105.8399963
-5.7370882	4.9776001	102.3600006
-5.7370882	5.0264001	102.2799988
-5.7370882	5.0752001	103.0299988
-5.7370882	5.1240001	101.4799957
-5.7370882	5.1728001	103.6699982
-5.7370882	5.2216001	104.9799957
-5.7370882	5.2704000	101.5100021
-5.7370882	5.3192000	105.5800018
-5.7370882	5.3680000	107.2299957
-5.7370882	5.4168000	107.0100021
-5.7370882	5.4656000	100.2900009
-5.7370882	5.5144000	106.5899963
-5.7370882	5.5632000	106.9000015
-5.7370882	5.6120000	105.6500015
-5.7370882	5.6608000	102.3300018
-5.7370882	5.7096000	101.7399979
-5.7370882	5.7584000	100.5299988
-5.7370882	5.8072000	102.2299957
-5.7370882	5.8559999	103.4399948
-5.7370882	5.9047999	106.6699982
-5.7370882	5.9535999	107.0199966
-5.7370882	6.0023999	104.4399948
-5.7370882	6.0511999	104.3099976
-5.7370882	6.0999999	102.1100006
-5.7370882	6.1487999	102.0599976
-5.7370882	6.1975999	104.4000015
-5.7370882	6.2463999	102.0800018
-5.7370882	6.2951999	101.5000000
-5.7370882	6.3439999	102.5100021
-5.7370882	6.3927999	100.8699951
-5.7370882	6.4415998	104.3499985
-5.7370882	6.4903998	106.0599976
-5.7370882	6.5391998	106.6399994
-5.7370882	6.5879998	105.2900009
-5.7370882	6.6367998	101.1699982
-5.7370882	6.6855998	97.7999954
-5.7370882	6.7343998	100.1899948
-5.7370882	6.7831998	100.9000015
-5.7370882	6.8319998	99.8699951
-5.7370882	6.8807998	101.4199982
-5.7370882	6.9295998	98.2900009
-5.7370882	6.9783998	102.1100006
-5.7370882	7.0271997	105.0299988
-5.7370882	7.0759997	105.3300018
-5.7370882	7.1247997	103.1399994
-5.7370882	7.1735997	99.3300018
-5.7370882	7.2223997	103.1399994
-5.7370882	7.2711997	105.3600006
-5.7370882	7.3199997	103.7200012
-5.7370882	7.3687997	104.0400009
-5.7370882	7.4175997	100.5400009
-5.7370882	7.4663997	100.1699982
-5.7370882	7.5151997	103.4599991
-5.7370882	7.5639997	105.9700012
-5.7370882	7.6127996	104.3600006

-5.7370882	7.6615996	102.9099960
-5.7370882	7.7103996	103.0699997
-5.7370882	7.7591996	102.7500000
-5.7370882	7.8079996	102.5100021
-5.7370882	7.8567996	102.6800003
-5.7370882	7.9056001	103.7299957
-5.7370882	7.9544001	103.7999954
-5.7370882	8.0032005	104.4799957
-5.7370882	8.0520000	106.5000000
-5.7370882	8.1008005	102.9199982
-5.7370882	8.1496000	100.2999954
-5.7370882	8.1984005	101.1599960
-5.7370882	8.2472000	106.5199966
-5.7370882	8.2960005	105.8899994
-5.7370882	8.3448000	105.6899948
-5.7370882	8.3936005	105.6199951
-5.7370882	8.4424000	104.8699951
-5.7370882	8.4912004	99.9700012
-5.7370882	8.5400000	97.4399948
-5.7370882	8.5888004	104.1500015
-5.7370882	8.6375999	103.1299973
-5.7370882	8.6864004	105.0699997
-5.7370882	8.7351999	105.9799957
-5.7370882	8.7840004	106.8399963
-5.7370882	8.8327999	106.0299988
-5.7370882	8.8816004	103.5899963
-5.7370882	8.9303999	103.3499985
-5.7370882	8.9792004	106.1999969
-5.7370882	9.0279999	107.4599991
-5.7370882	9.0768003	105.8499985
-5.7370882	9.1255999	106.7399979
-5.7370882	9.1744003	105.8300018
-5.7370882	9.2231998	104.3499985
-5.7370882	9.2720003	100.7099991
-5.7370882	9.3207998	100.8499985
-5.7370882	9.3696003	103.4300003
-5.7370882	9.4184008	105.6199951
-5.7370882	9.4672003	101.6800003
-5.7370882	9.5160007	102.2200012
-5.7370882	9.5648003	102.9099960
-5.7370882	9.6136007	102.2999954
-5.7370882	9.6624002	105.0800018
-5.7370882	9.7112007	105.3799973
-5.7370882	9.7600002	105.7399979
-5.7370882	9.8088007	106.1899948
-5.7370882	9.8576002	105.5400009
-5.7370882	9.9064007	104.0899963
-5.7370882	9.9552002	103.3499985
-5.7370882	10.0040007	101.5800018
-5.7370882	10.0528002	102.6100006
-5.7370882	10.1016006	102.9700012
-5.7370882	10.1504002	101.3600006
-5.7370882	10.1992006	101.7200012
-5.7370882	10.2480001	101.8499985
-5.7370882	10.2968006	103.2600021
-5.7370882	10.3456001	97.2200012
-5.7370882	10.3944006	99.4399948
-5.7370882	10.4432001	102.9099960

-5.7370882	10.4920006	104.6599960
-5.7370882	10.5408001	101.8799973
-5.7370882	10.5896006	102.9300003
-5.7370882	10.6384001	100.5800018
-5.7370882	10.6872005	104.6999969
-5.7370882	10.7360001	105.6399994
-5.7370882	10.7848005	102.7699966
-5.7370882	10.8336000	104.0299988
-5.7370882	10.8824005	103.9099960
-5.7370882	10.9312000	102.5199966
-5.7370882	10.9800005	99.7200012
-5.7370882	11.0288000	98.4700012
-5.7370882	11.0776005	101.3600006
-5.7370882	11.1264000	103.5599976
-5.7370882	11.1752005	105.0599976
-5.7370882	11.2240000	103.0699997
-5.7370882	11.2728004	103.8300018
-5.7370882	11.3216000	104.7399979
-5.7370882	11.3704004	105.6899948
-5.7370882	11.4191999	104.5800018
-5.7370882	11.4680004	106.1299973
-5.7370882	11.5167999	105.9899979
-5.7370882	11.5656004	104.8899994
-5.7370882	11.6143999	102.3600006
-5.7370882	11.6632004	102.5000000
-5.7370882	11.7119999	105.8600006
-5.7370882	11.7608004	105.9000015
-5.7370882	11.8095999	102.5400009
-5.7370882	11.8584003	104.1199951
-5.7370882	11.9071999	102.4700012
-5.7370882	11.9560003	102.6599960
-5.7370882	12.0047998	102.9799957
-5.7370882	12.0536003	99.5000000
-5.7370882	12.1023998	99.1399994
-5.7370882	12.1512003	102.9000015
-5.7370882	12.1999998	104.5199966
-5.7370882	12.2488003	102.6599960
-5.7370882	12.2975998	104.8499985
-5.7370882	12.3464003	106.3199997
-5.7370882	12.3951998	104.3600006
-5.7370882	12.4440002	99.5100021
-5.7370882	12.4927998	103.8300018
-5.7370882	12.5416002	103.4700012
-5.7370882	12.5904007	101.1599960
-5.7370882	12.6392002	100.2600021
-5.7370882	12.6880007	101.0199966
-5.7370882	12.7368002	104.4499969
-5.7370882	12.7856007	104.5499954
-5.7370882	12.8344002	103.7699966
-5.7370882	12.8832006	101.1500015
-5.7370882	12.9320002	102.2500000
-5.7370882	12.9808006	102.7099991
-5.7370882	13.0296001	104.7200012
-5.7370882	13.0784006	103.8899994
-5.7370882	13.1272001	105.7699966
-5.7370882	13.1760006	105.2600021
-5.7370882	13.2248001	103.8799973
-5.7370882	13.2736006	100.9300003

-5.7370882	13.3224001	102.7500000
-5.7370882	13.3712006	102.2999954
-5.7370882	13.4200001	102.0800018
-5.7370882	13.4688005	100.5699997
-5.7370882	13.5176001	100.9099960
-5.7370882	13.5664005	100.8899994
-5.7370882	13.6152000	103.7999954
-5.7370882	13.6640005	103.4499969
-5.7370882	13.7128000	97.0400009
-5.7370882	13.7616005	97.8899994
-5.7370882	13.8104000	100.7399979
-5.7370882	13.8592005	101.7999954
-5.7370882	13.9080000	100.5899963
-5.7370882	13.9568005	103.4300003
-5.7370882	14.0056000	103.9899979
-5.7370882	14.0544004	101.1399994
-5.7370882	14.1032000	102.4000015
-5.7370882	14.1520004	103.6299973
-5.7370882	14.2007999	104.9399948
-5.7370882	14.2496004	105.0199966
-5.7370882	14.2983999	101.6299973
-5.7370882	14.3472004	98.2699966
-5.7370882	14.3959999	101.8199997
-5.7370882	14.4448004	103.0999985
-5.7370882	14.4935999	103.5899963
-5.7370882	14.5424004	102.0199966
-5.7370882	14.5911999	102.5800018
-5.7370882	14.6400003	103.4700012
-5.7370882	14.6887999	103.8499985
-5.7370882	14.7376003	102.1999969
-5.7370882	14.7863998	104.8099976
-5.7370882	14.8352003	100.4799957
-5.7370882	14.8839998	102.1500015
-5.7370882	14.9328003	101.2299957
-5.7370882	14.9815998	100.4000015
-5.7370882	15.0304003	98.0199966
-5.7309523	0.3416000	109.0499954
-5.7309523	0.3904000	109.9399948
-5.7309523	0.4392000	109.2900009
-5.7309523	0.4880000	104.8499985
-5.7309523	0.5368000	106.1699982
-5.7309523	0.5856000	106.3300018
-5.7309523	0.6344000	107.9000015
-5.7309523	0.6832000	107.7200012
-5.7309523	0.7320000	107.0899963
-5.7309523	0.7808000	107.4099960
-5.7309523	0.8296000	106.5299988
-5.7309523	0.8784000	104.8699951
-5.7309523	0.9272000	107.5899963
-5.7309523	0.9760000	106.6899948
-5.7309523	1.0247999	103.2399979
-5.7309523	1.0735999	104.6999969
-5.7309523	1.1223999	103.7600021
-5.7309523	1.1711999	101.7500000
-5.7309523	1.2200000	103.9899979
-5.7309523	1.2688000	106.2799988
-5.7309523	1.3176000	107.4399948
-5.7309523	1.3664000	104.9599991

-5.7309523	1.4152000	108.7600021
-5.7309523	1.4640000	109.7099991
-5.7309523	1.5128000	108.1100006
-5.7309523	1.5616000	105.5999985
-5.7309523	1.6104000	103.3600006
-5.7309523	1.6592000	104.5199966
-5.7309523	1.7079999	105.5699997
-5.7309523	1.7567999	106.3300018
-5.7309523	1.8055999	105.3699951
-5.7309523	1.8543999	108.0400009
-5.7309523	1.9031999	111.3099976
-5.7309523	1.9519999	109.3600006
-5.7309523	2.0007999	103.8799973
-5.7309523	2.0495999	101.0899963
-5.7309523	2.0983999	102.6800003
-5.7309523	2.1471999	104.5499954
-5.7309523	2.1959999	104.5100021
-5.7309523	2.2447999	107.3899994
-5.7309523	2.2935998	106.0599976
-5.7309523	2.3423998	106.1399994
-5.7309523	2.3912001	106.7799988
-5.7309523	2.4400001	105.4599991
-5.7309523	2.4888000	106.7500000
-5.7309523	2.5376000	104.9300003
-5.7309523	2.5864000	107.3600006
-5.7309523	2.6352000	107.8799973
-5.7309523	2.6840000	105.6100006
-5.7309523	2.7328000	102.4599991
-5.7309523	2.7816000	105.2999954
-5.7309523	2.8304000	103.5199966
-5.7309523	2.8792000	103.7099991
-5.7309523	2.9280000	104.7999954
-5.7309523	2.9768000	104.4399948
-5.7309523	3.0256000	105.9199982
-5.7309523	3.0743999	105.4799957
-5.7309523	3.1231999	104.8300018
-5.7309523	3.1719999	103.6199951
-5.7309523	3.2207999	101.3600006
-5.7309523	3.2695999	101.8899994
-5.7309523	3.3183999	106.1599960
-5.7309523	3.3671999	105.5400009
-5.7309523	3.4159999	102.4399948
-5.7309523	3.4647999	105.7999954
-5.7309523	3.5135999	105.5400009
-5.7309523	3.5623999	104.4499969
-5.7309523	3.6111999	106.8699951
-5.7309523	3.6599998	103.0899963
-5.7309523	3.7087998	98.1100006
-5.7309523	3.7575998	100.6199951
-5.7309523	3.8063998	105.3199997
-5.7309523	3.8551998	105.5699997
-5.7309523	3.9039998	105.3399963
-5.7309523	3.9527998	105.6100006
-5.7309523	4.0016003	105.8799973
-5.7309523	4.0503998	102.5599976
-5.7309523	4.0991998	102.9499969
-5.7309523	4.1479998	100.9099960
-5.7309523	4.1967998	102.8399963

-5.7309523	4.2455997	102.1599960
-5.7309523	4.2943997	105.5499954
-5.7309523	4.3431997	106.0000000
-5.7309523	4.3919997	104.1299973
-5.7309523	4.4407997	102.5299988
-5.7309523	4.4895997	105.3099976
-5.7309523	4.5383997	107.4599991
-5.7309523	4.5871997	109.2699966
-5.7309523	4.6359997	110.7699966
-5.7309523	4.6847997	116.0599976
-5.7309523	4.7336001	115.1699982
-5.7309523	4.7824001	108.3499985
-5.7309523	4.8312001	102.6299973
-5.7309523	4.8800001	105.6800003
-5.7309523	4.9288001	103.7900009
-5.7309523	4.9776001	101.6699982
-5.7309523	5.0264001	102.7500000
-5.7309523	5.0752001	103.7600021
-5.7309523	5.1240001	100.6500015
-5.7309523	5.1728001	103.7900009
-5.7309523	5.2216001	105.4700012
-5.7309523	5.2704000	101.9899979
-5.7309523	5.3192000	106.0499954
-5.7309523	5.3680000	107.6399994
-5.7309523	5.4168000	107.1800003
-5.7309523	5.4656000	100.7099991
-5.7309523	5.5144000	106.4000015
-5.7309523	5.5632000	107.0800018
-5.7309523	5.6120000	105.2099991
-5.7309523	5.6608000	101.0299988
-5.7309523	5.7096000	100.9000015
-5.7309523	5.7584000	99.9000015
-5.7309523	5.8072000	102.2299957
-5.7309523	5.8559999	103.4199982
-5.7309523	5.9047999	106.7699966
-5.7309523	5.9535999	106.7200012
-5.7309523	6.0023999	103.9199982
-5.7309523	6.0511999	104.0599976
-5.7309523	6.0999999	102.0400009
-5.7309523	6.1487999	101.5499954
-5.7309523	6.1975999	104.1599960
-5.7309523	6.2463999	102.3399963
-5.7309523	6.2951999	102.8199997
-5.7309523	6.3439999	103.2299957
-5.7309523	6.3927999	101.5499954
-5.7309523	6.4415998	104.4599991
-5.7309523	6.4903998	106.0299988
-5.7309523	6.5391998	106.9000015
-5.7309523	6.5879998	105.9099960
-5.7309523	6.6367998	102.6999969
-5.7309523	6.6855998	98.8300018
-5.7309523	6.7343998	101.2399979
-5.7309523	6.7831998	101.4700012
-5.7309523	6.8319998	101.0199966
-5.7309523	6.8807998	102.4099960
-5.7309523	6.9295998	98.0999985
-5.7309523	6.9783998	102.1699982
-5.7309523	7.0271997	104.1199951

-5.7309523	7.0759997	104.8399963
-5.7309523	7.1247997	103.6800003
-5.7309523	7.1735997	100.4000015
-5.7309523	7.2223997	103.5999985
-5.7309523	7.2711997	105.5499954
-5.7309523	7.3199997	104.3600006
-5.7309523	7.3687997	105.1500015
-5.7309523	7.4175997	101.4700012
-5.7309523	7.4663997	100.5899963
-5.7309523	7.5151997	103.7399979
-5.7309523	7.5639997	105.5699997
-5.7309523	7.6127996	103.7500000
-5.7309523	7.6615996	101.4099960
-5.7309523	7.7103996	101.8499985
-5.7309523	7.7591996	103.1500015
-5.7309523	7.8079996	103.0100021
-5.7309523	7.8567996	102.9599991
-5.7309523	7.9056001	103.5199966
-5.7309523	7.9544001	103.6500015
-5.7309523	8.0032005	103.5100021
-5.7309523	8.0520000	105.7600021
-5.7309523	8.1008005	101.9899979
-5.7309523	8.1496000	100.1800003
-5.7309523	8.1984005	101.6699982
-5.7309523	8.2472000	105.9399948
-5.7309523	8.2960005	106.0100021
-5.7309523	8.3448000	105.5199966
-5.7309523	8.3936005	104.4399948
-5.7309523	8.4424000	104.6299973
-5.7309523	8.4912004	100.7699966
-5.7309523	8.5400000	97.0199966
-5.7309523	8.5888004	104.0899963
-5.7309523	8.6375999	101.7999954
-5.7309523	8.6864004	104.0800018
-5.7309523	8.7351999	105.2699966
-5.7309523	8.7840004	105.8399963
-5.7309523	8.8327999	106.3499985
-5.7309523	8.8816004	103.5499954
-5.7309523	8.9303999	102.8899994
-5.7309523	8.9792004	105.1599960
-5.7309523	9.0279999	106.2900009
-5.7309523	9.0768003	104.9700012
-5.7309523	9.1255999	107.0800018
-5.7309523	9.1744003	106.2999954
-5.7309523	9.2231998	104.9700012
-5.7309523	9.2720003	101.8199997
-5.7309523	9.3207998	101.3199997
-5.7309523	9.3696003	103.6999969
-5.7309523	9.4184008	105.7500000
-5.7309523	9.4672003	102.7299957
-5.7309523	9.5160007	102.8799973
-5.7309523	9.5648003	103.2099991
-5.7309523	9.6136007	102.1699982
-5.7309523	9.6624002	105.2600021
-5.7309523	9.7112007	105.0400009
-5.7309523	9.7600002	104.8699951
-5.7309523	9.8088007	104.8600006
-5.7309523	9.8576002	104.4199982

-5.7309523	9.9064007	103.4899979
-5.7309523	9.9552002	103.1999969
-5.7309523	10.0040007	101.4199982
-5.7309523	10.0528002	102.4399948
-5.7309523	10.1016006	102.6999969
-5.7309523	10.1504002	101.2600021
-5.7309523	10.1992006	101.7299957
-5.7309523	10.2480001	102.6699982
-5.7309523	10.2968006	102.8899994
-5.7309523	10.3456001	96.6500015
-5.7309523	10.3944006	99.8099976
-5.7309523	10.4432001	103.1999969
-5.7309523	10.4920006	104.5899963
-5.7309523	10.5408001	101.4300003
-5.7309523	10.5896006	103.1599960
-5.7309523	10.6384001	101.6699982
-5.7309523	10.6872005	103.6500015
-5.7309523	10.7360001	104.8399963
-5.7309523	10.7848005	102.0000000
-5.7309523	10.8336000	104.0899963
-5.7309523	10.8824005	103.8499985
-5.7309523	10.9312000	102.7200012
-5.7309523	10.9800005	99.8399963
-5.7309523	11.0288000	98.2500000
-5.7309523	11.0776005	101.6800003
-5.7309523	11.1264000	104.0299988
-5.7309523	11.1752005	104.9799957
-5.7309523	11.2240000	102.0499954
-5.7309523	11.2728004	102.6899948
-5.7309523	11.3216000	104.2500000
-5.7309523	11.3704004	104.9300003
-5.7309523	11.4191999	105.0499954
-5.7309523	11.4680004	106.0599976
-5.7309523	11.5167999	105.7600021
-5.7309523	11.5656004	104.5499954
-5.7309523	11.6143999	101.8199997
-5.7309523	11.6632004	101.6599960
-5.7309523	11.7119999	106.1299973
-5.7309523	11.7608004	106.1599960
-5.7309523	11.8095999	102.7500000
-5.7309523	11.8584003	103.9399948
-5.7309523	11.9071999	101.5599976
-5.7309523	11.9560003	102.1299973
-5.7309523	12.0047998	103.6800003
-5.7309523	12.0536003	101.0000000
-5.7309523	12.1023998	99.5699997
-5.7309523	12.1512003	102.8300018
-5.7309523	12.1999998	104.4000015
-5.7309523	12.2488003	102.0400009
-5.7309523	12.2975998	104.6299973
-5.7309523	12.3464003	106.0100021
-5.7309523	12.3951998	104.2299957
-5.7309523	12.4440002	99.2699966
-5.7309523	12.4927998	103.9899979
-5.7309523	12.5416002	102.8399963
-5.7309523	12.5904007	101.0400009
-5.7309523	12.6392002	100.7500000
-5.7309523	12.6880007	100.9199982

-5.7309523	12.7368002	104.0999985
-5.7309523	12.7856007	104.3799973
-5.7309523	12.8344002	104.3199997
-5.7309523	12.8832006	100.0299988
-5.7309523	12.9320002	102.1699982
-5.7309523	12.9808006	103.5000000
-5.7309523	13.0296001	105.3300018
-5.7309523	13.0784006	104.2299957
-5.7309523	13.1272001	104.7500000
-5.7309523	13.1760006	104.7799988
-5.7309523	13.2248001	103.6800003
-5.7309523	13.2736006	100.3399963
-5.7309523	13.3224001	103.0299988
-5.7309523	13.3712006	102.4199982
-5.7309523	13.4200001	101.0999985
-5.7309523	13.4688005	101.0699997
-5.7309523	13.5176001	101.4799957
-5.7309523	13.5664005	100.2299957
-5.7309523	13.6152000	103.5100021
-5.7309523	13.6640005	103.9799957
-5.7309523	13.7128000	97.3600006
-5.7309523	13.7616005	95.8399963
-5.7309523	13.8104000	100.5400009
-5.7309523	13.8592005	102.1100006
-5.7309523	13.9080000	100.9499969
-5.7309523	13.9568005	103.0199966
-5.7309523	14.0056000	103.8799973
-5.7309523	14.0544004	99.8899994
-5.7309523	14.1032000	101.6899948
-5.7309523	14.1520004	103.3300018
-5.7309523	14.2007999	104.4199982
-5.7309523	14.2496004	104.8799973
-5.7309523	14.2983999	101.8199997
-5.7309523	14.3472004	96.5699997
-5.7309523	14.3959999	100.7999954
-5.7309523	14.4448004	101.6899948
-5.7309523	14.4935999	102.4899979
-5.7309523	14.5424004	100.3199997
-5.7309523	14.5911999	101.4000015
-5.7309523	14.6400003	102.7699966
-5.7309523	14.6887999	103.7600021
-5.7309523	14.7376003	102.7900009
-5.7309523	14.7863998	104.7999954
-5.7309523	14.8352003	101.2200012
-5.7309523	14.8839998	102.3799973
-5.7309523	14.9328003	100.9000015
-5.7309523	14.9815998	100.7299957
-5.7309523	15.0304003	99.0100021
-5.7248163	0.3416000	108.5899963
-5.7248163	0.3904000	109.6699982
-5.7248163	0.4392000	109.2799988
-5.7248163	0.4880000	105.5400009
-5.7248163	0.5368000	106.5800018
-5.7248163	0.5856000	106.1299973
-5.7248163	0.6344000	107.4099960
-5.7248163	0.6832000	107.6199951
-5.7248163	0.7320000	107.0999985
-5.7248163	0.7808000	107.1399994

-5.7248163	0.8296000	106.9499969
-5.7248163	0.8784000	105.2900009
-5.7248163	0.9272000	107.1100006
-5.7248163	0.9760000	106.6599960
-5.7248163	1.0247999	104.4599991
-5.7248163	1.0735999	105.3600006
-5.7248163	1.1223999	103.6999969
-5.7248163	1.1711999	100.4899979
-5.7248163	1.2200000	104.9000015
-5.7248163	1.2688000	107.7799988
-5.7248163	1.3176000	107.8799973
-5.7248163	1.3664000	105.4599991
-5.7248163	1.4152000	108.6999969
-5.7248163	1.4640000	109.7200012
-5.7248163	1.5128000	108.7500000
-5.7248163	1.5616000	105.8899994
-5.7248163	1.6104000	102.7099991
-5.7248163	1.6592000	103.2200012
-5.7248163	1.7079999	103.9300003
-5.7248163	1.7567999	105.2299957
-5.7248163	1.8055999	105.4899979
-5.7248163	1.8543999	108.3499985
-5.7248163	1.9031999	111.2399979
-5.7248163	1.9519999	109.3199997
-5.7248163	2.0007999	104.0499954
-5.7248163	2.0495999	101.6800003
-5.7248163	2.0983999	103.5400009
-5.7248163	2.1471999	105.5199966
-5.7248163	2.1959999	104.5499954
-5.7248163	2.2447999	107.5599976
-5.7248163	2.2935998	107.2099991
-5.7248163	2.3423998	106.6800003
-5.7248163	2.3912001	106.5400009
-5.7248163	2.4400001	104.9499969
-5.7248163	2.4888000	106.4000015
-5.7248163	2.5376000	103.9599991
-5.7248163	2.5864000	106.9799957
-5.7248163	2.6352000	108.0999985
-5.7248163	2.6840000	106.1100006
-5.7248163	2.7328000	103.4700012
-5.7248163	2.7816000	105.3499985
-5.7248163	2.8304000	103.7699966
-5.7248163	2.8792000	104.0100021
-5.7248163	2.9280000	104.9399948
-5.7248163	2.9768000	104.7699966
-5.7248163	3.0256000	106.2099991
-5.7248163	3.0743999	105.8699951
-5.7248163	3.1231999	105.4700012
-5.7248163	3.1719999	103.4799957
-5.7248163	3.2207999	101.1399994
-5.7248163	3.2695999	102.8499985
-5.7248163	3.3183999	106.5299988
-5.7248163	3.3671999	104.9899979
-5.7248163	3.4159999	103.1500015
-5.7248163	3.4647999	106.1699982
-5.7248163	3.5135999	104.6599960
-5.7248163	3.5623999	103.5499954
-5.7248163	3.6111999	107.0100021

-5.7248163	3.6599998	102.5299988
-5.7248163	3.7087998	97.8899994
-5.7248163	3.7575998	101.3199997
-5.7248163	3.8063998	105.0199966
-5.7248163	3.8551998	104.9899979
-5.7248163	3.9039998	105.1299973
-5.7248163	3.9527998	105.4399948
-5.7248163	4.0016003	105.7200012
-5.7248163	4.0503998	103.4799957
-5.7248163	4.0991998	103.5699997
-5.7248163	4.1479998	101.4599991
-5.7248163	4.1967998	103.4300003
-5.7248163	4.2455997	103.0000000
-5.7248163	4.2943997	105.6800003
-5.7248163	4.3431997	105.3899994
-5.7248163	4.3919997	103.6800003
-5.7248163	4.4407997	100.9899979
-5.7248163	4.4895997	104.3099976
-5.7248163	4.5383997	106.3499985
-5.7248163	4.5871997	109.0000000
-5.7248163	4.6359997	111.2900009
-5.7248163	4.6847997	116.0299988
-5.7248163	4.7336001	114.8699951
-5.7248163	4.7824001	108.0000000
-5.7248163	4.8312001	102.7999954
-5.7248163	4.8800001	105.6699982
-5.7248163	4.9288001	102.4499969
-5.7248163	4.9776001	100.6800003
-5.7248163	5.0264001	103.0800018
-5.7248163	5.0752001	104.0599976
-5.7248163	5.1240001	99.6599960
-5.7248163	5.1728001	103.4099960
-5.7248163	5.2216001	105.9899979
-5.7248163	5.2704000	103.0299988
-5.7248163	5.3192000	106.0800018
-5.7248163	5.3680000	107.2200012
-5.7248163	5.4168000	106.9099960
-5.7248163	5.4656000	100.6500015
-5.7248163	5.5144000	105.5800018
-5.7248163	5.5632000	106.6800003
-5.7248163	5.6120000	104.8199997
-5.7248163	5.6608000	101.3399963
-5.7248163	5.7096000	101.4099960
-5.7248163	5.7584000	100.1699982
-5.7248163	5.8072000	102.1299973
-5.7248163	5.8559999	102.7200012
-5.7248163	5.9047999	106.8199997
-5.7248163	5.9535999	106.5299988
-5.7248163	6.0023999	103.6899948
-5.7248163	6.0511999	104.1599960
-5.7248163	6.0999999	102.3799973
-5.7248163	6.1487999	101.2500000
-5.7248163	6.1975999	104.1999969
-5.7248163	6.2463999	103.5499954
-5.7248163	6.2951999	105.1500015
-5.7248163	6.3439999	104.5599976
-5.7248163	6.3927999	102.4899979
-5.7248163	6.4415998	104.9700012

-5.7248163	6.4903998	106.5100021
-5.7248163	6.5391998	107.0999985
-5.7248163	6.5879998	106.2999954
-5.7248163	6.6367998	104.0199966
-5.7248163	6.6855998	100.2299957
-5.7248163	6.7343998	102.6299973
-5.7248163	6.7831998	102.6699982
-5.7248163	6.8319998	102.3399963
-5.7248163	6.8807998	102.6599960
-5.7248163	6.9295998	98.0400009
-5.7248163	6.9783998	101.8399963
-5.7248163	7.0271997	103.4799957
-5.7248163	7.0759997	104.3699951
-5.7248163	7.1247997	103.5400009
-5.7248163	7.1735997	99.8300018
-5.7248163	7.2223997	103.3699951
-5.7248163	7.2711997	105.5000000
-5.7248163	7.3199997	104.3099976
-5.7248163	7.3687997	105.8899994
-5.7248163	7.4175997	102.3600006
-5.7248163	7.4663997	100.9899979
-5.7248163	7.5151997	103.5499954
-5.7248163	7.5639997	104.7099991
-5.7248163	7.6127996	101.8399963
-5.7248163	7.6615996	98.9700012
-5.7248163	7.7103996	100.0100021
-5.7248163	7.7591996	103.3300018
-5.7248163	7.8079996	103.8099976
-5.7248163	7.8567996	103.3899994
-5.7248163	7.9056001	103.5000000
-5.7248163	7.9544001	103.5800018
-5.7248163	8.0032005	103.4300003
-5.7248163	8.0520000	105.3799973
-5.7248163	8.1008005	101.9300003
-5.7248163	8.1496000	99.6599960
-5.7248163	8.1984005	101.7299957
-5.7248163	8.2472000	104.6599960
-5.7248163	8.2960005	105.8300018
-5.7248163	8.3448000	105.0599976
-5.7248163	8.3936005	103.0199966
-5.7248163	8.4424000	103.5499954
-5.7248163	8.4912004	101.6899948
-5.7248163	8.5400000	97.0400009
-5.7248163	8.5888004	104.1299973
-5.7248163	8.6375999	100.5000000
-5.7248163	8.6864004	103.2500000
-5.7248163	8.7351999	104.7600021
-5.7248163	8.7840004	105.0100021
-5.7248163	8.8327999	106.0999985
-5.7248163	8.8816004	102.4000015
-5.7248163	8.9303999	102.0000000
-5.7248163	8.9792004	104.1500015
-5.7248163	9.0279999	105.2799988
-5.7248163	9.0768003	104.0199966
-5.7248163	9.1255999	107.0199966
-5.7248163	9.1744003	106.1299973
-5.7248163	9.2231998	104.5100021
-5.7248163	9.2720003	101.8600006

-5.7248163	9.3207998	101.2600021
-5.7248163	9.3696003	103.6899948
-5.7248163	9.4184008	105.7799988
-5.7248163	9.4672003	103.6599960
-5.7248163	9.5160007	103.2900009
-5.7248163	9.5648003	103.3300018
-5.7248163	9.6136007	102.7900009
-5.7248163	9.6624002	106.1899948
-5.7248163	9.7112007	105.2500000
-5.7248163	9.7600002	104.0499954
-5.7248163	9.8088007	103.9000015
-5.7248163	9.8576002	103.9199982
-5.7248163	9.9064007	103.0800018
-5.7248163	9.9552002	102.7099991
-5.7248163	10.0040007	100.8899994
-5.7248163	10.0528002	101.6800003
-5.7248163	10.1016006	101.8399963
-5.7248163	10.1504002	100.7099991
-5.7248163	10.1992006	101.4700012
-5.7248163	10.2480001	103.0499954
-5.7248163	10.2968006	102.5899963
-5.7248163	10.3456001	97.6899948
-5.7248163	10.3944006	100.0899963
-5.7248163	10.4432001	103.3899994
-5.7248163	10.4920006	104.7200012
-5.7248163	10.5408001	101.4300003
-5.7248163	10.5896006	103.4799957
-5.7248163	10.6384001	102.6599960
-5.7248163	10.6872005	102.6399994
-5.7248163	10.7360001	103.9799957
-5.7248163	10.7848005	101.0699997
-5.7248163	10.8336000	104.2200012
-5.7248163	10.8824005	104.1199951
-5.7248163	10.9312000	102.5400009
-5.7248163	10.9800005	99.6199951
-5.7248163	11.0288000	98.0000000
-5.7248163	11.0776005	101.9399948
-5.7248163	11.1264000	104.1699982
-5.7248163	11.1752005	104.1299973
-5.7248163	11.2240000	101.4499969
-5.7248163	11.2728004	101.7299957
-5.7248163	11.3216000	103.2200012
-5.7248163	11.3704004	104.1299973
-5.7248163	11.4191999	105.0599976
-5.7248163	11.4680004	106.0100021
-5.7248163	11.5167999	105.6500015
-5.7248163	11.5656004	104.5199966
-5.7248163	11.6143999	101.4000015
-5.7248163	11.6632004	101.6500015
-5.7248163	11.7119999	106.2699966
-5.7248163	11.7608004	106.4099960
-5.7248163	11.8095999	103.1199951
-5.7248163	11.8584003	103.6100006
-5.7248163	11.9071999	100.7299957
-5.7248163	11.9560003	101.7799988
-5.7248163	12.0047998	104.4099960
-5.7248163	12.0536003	102.9499969
-5.7248163	12.1023998	100.3300018

-5.7248163	12.1512003	102.5699997
-5.7248163	12.1999998	103.9599991
-5.7248163	12.2488003	101.1599960
-5.7248163	12.2975998	104.4099960
-5.7248163	12.3464003	105.0800018
-5.7248163	12.3951998	104.2699966
-5.7248163	12.4440002	100.3199997
-5.7248163	12.4927998	103.5199966
-5.7248163	12.5416002	101.7699966
-5.7248163	12.5904007	101.0400009
-5.7248163	12.6392002	101.9899979
-5.7248163	12.6880007	101.6199951
-5.7248163	12.7368002	104.1999969
-5.7248163	12.7856007	105.1599960
-5.7248163	12.8344002	105.2600021
-5.7248163	12.8832006	98.7999954
-5.7248163	12.9320002	101.9700012
-5.7248163	12.9808006	104.7799988
-5.7248163	13.0296001	106.0400009
-5.7248163	13.0784006	104.0699997
-5.7248163	13.1272001	103.5100021
-5.7248163	13.1760006	104.2600021
-5.7248163	13.2248001	103.2999954
-5.7248163	13.2736006	99.7200012
-5.7248163	13.3224001	102.3600006
-5.7248163	13.3712006	102.1399994
-5.7248163	13.4200001	100.0400009
-5.7248163	13.4688005	101.3899994
-5.7248163	13.5176001	101.9399948
-5.7248163	13.5664005	100.3499985
-5.7248163	13.6152000	103.6100006
-5.7248163	13.6640005	104.5499954
-5.7248163	13.7128000	98.4700012
-5.7248163	13.7616005	94.4199982
-5.7248163	13.8104000	100.6199951
-5.7248163	13.8592005	102.6100006
-5.7248163	13.9080000	101.3899994
-5.7248163	13.9568005	102.4300003
-5.7248163	14.0056000	103.7699966
-5.7248163	14.0544004	98.7500000
-5.7248163	14.1032000	101.1699982
-5.7248163	14.1520004	102.9199982
-5.7248163	14.2007999	103.2500000
-5.7248163	14.2496004	104.1100006
-5.7248163	14.2983999	102.0199966
-5.7248163	14.3472004	96.5599976
-5.7248163	14.3959999	100.3499985
-5.7248163	14.4448004	100.3199997
-5.7248163	14.4935999	101.6500015
-5.7248163	14.5424004	99.3799973
-5.7248163	14.5911999	100.6399994
-5.7248163	14.6400003	102.2600021
-5.7248163	14.6887999	103.8499985
-5.7248163	14.7376003	102.8899994
-5.7248163	14.7863998	105.0599976
-5.7248163	14.8352003	102.4399948
-5.7248163	14.8839998	102.8199997
-5.7248163	14.9328003	101.1100006

-5.7248163	14.9815998	101.5800018
-5.7248163	15.0304003	100.6299973
-5.7186804	0.3416000	108.0599976
-5.7186804	0.3904000	109.1100006
-5.7186804	0.4392000	108.6899948
-5.7186804	0.4880000	105.4499969
-5.7186804	0.5368000	106.7600021
-5.7186804	0.5856000	105.2500000
-5.7186804	0.6344000	106.0199966
-5.7186804	0.6832000	107.4599991
-5.7186804	0.7320000	107.2500000
-5.7186804	0.7808000	106.5599976
-5.7186804	0.8296000	107.0400009
-5.7186804	0.8784000	105.5299988
-5.7186804	0.9272000	106.6100006
-5.7186804	0.9760000	106.7399979
-5.7186804	1.0247999	105.7200012
-5.7186804	1.0735999	105.5800018
-5.7186804	1.1223999	103.3699951
-5.7186804	1.1711999	99.5299988
-5.7186804	1.2200000	105.5899963
-5.7186804	1.2688000	108.0599976
-5.7186804	1.3176000	107.6500015
-5.7186804	1.3664000	105.8699951
-5.7186804	1.4152000	108.8499985
-5.7186804	1.4640000	109.3699951
-5.7186804	1.5128000	109.1500015
-5.7186804	1.5616000	106.4399948
-5.7186804	1.6104000	102.5599976
-5.7186804	1.6592000	102.0400009
-5.7186804	1.7079999	102.4700012
-5.7186804	1.7567999	104.8099976
-5.7186804	1.8055999	105.6599960
-5.7186804	1.8543999	108.5699997
-5.7186804	1.9031999	111.5299988
-5.7186804	1.9519999	109.5800018
-5.7186804	2.0007999	104.3499985
-5.7186804	2.0495999	102.5899963
-5.7186804	2.0983999	104.4099960
-5.7186804	2.1471999	106.5400009
-5.7186804	2.1959999	104.7799988
-5.7186804	2.2447999	108.1100006
-5.7186804	2.2935998	108.4199982
-5.7186804	2.3423998	106.9700012
-5.7186804	2.3912001	106.4399948
-5.7186804	2.4400001	104.8300018
-5.7186804	2.4888000	106.2699966
-5.7186804	2.5376000	103.4399948
-5.7186804	2.5864000	106.6899948
-5.7186804	2.6352000	107.5800018
-5.7186804	2.6840000	106.3799973
-5.7186804	2.7328000	104.1399994
-5.7186804	2.7816000	104.5100021
-5.7186804	2.8304000	103.3300018
-5.7186804	2.8792000	104.1199951
-5.7186804	2.9280000	105.4599991
-5.7186804	2.9768000	105.4700012
-5.7186804	3.0256000	106.3199997

-5.7186804	3.0743999	105.8899994
-5.7186804	3.1231999	104.8799973
-5.7186804	3.1719999	103.1699982
-5.7186804	3.2207999	101.0199966
-5.7186804	3.2695999	103.7699966
-5.7186804	3.3183999	106.9300003
-5.7186804	3.3671999	104.7600021
-5.7186804	3.4159999	104.2699966
-5.7186804	3.4647999	105.5499954
-5.7186804	3.5135999	103.6999969
-5.7186804	3.5623999	102.7200012
-5.7186804	3.6111999	106.1599960
-5.7186804	3.6599998	101.1500015
-5.7186804	3.7087998	98.5000000
-5.7186804	3.7575998	101.7799988
-5.7186804	3.8063998	104.7299957
-5.7186804	3.8551998	105.0800018
-5.7186804	3.9039998	105.3699951
-5.7186804	3.9527998	105.2699966
-5.7186804	4.0016003	105.9899979
-5.7186804	4.0503998	105.0400009
-5.7186804	4.0991998	104.4099960
-5.7186804	4.1479998	101.8699951
-5.7186804	4.1967998	103.5299988
-5.7186804	4.2455997	102.7999954
-5.7186804	4.2943997	105.3399963
-5.7186804	4.3431997	105.2699966
-5.7186804	4.3919997	104.8399963
-5.7186804	4.4407997	101.7500000
-5.7186804	4.4895997	104.2600021
-5.7186804	4.5383997	105.4000015
-5.7186804	4.5871997	108.7999954
-5.7186804	4.6359997	112.2799988
-5.7186804	4.6847997	116.1100006
-5.7186804	4.7336001	114.6199951
-5.7186804	4.7824001	107.5999985
-5.7186804	4.8312001	103.1500015
-5.7186804	4.8800001	105.7699966
-5.7186804	4.9288001	101.0599976
-5.7186804	4.9776001	100.0100021
-5.7186804	5.0264001	103.6800003
-5.7186804	5.0752001	104.3499985
-5.7186804	5.1240001	99.4399948
-5.7186804	5.1728001	102.8699951
-5.7186804	5.2216001	106.0999985
-5.7186804	5.2704000	103.7500000
-5.7186804	5.3192000	106.0800018
-5.7186804	5.3680000	106.8499985
-5.7186804	5.4168000	106.7299957
-5.7186804	5.4656000	100.4799957
-5.7186804	5.5144000	104.2200012
-5.7186804	5.5632000	105.6500015
-5.7186804	5.6120000	104.1599960
-5.7186804	5.6608000	101.7200012
-5.7186804	5.7096000	102.5400009
-5.7186804	5.7584000	101.1800003
-5.7186804	5.8072000	101.6500015
-5.7186804	5.8559999	101.4399948

-5.7186804	5.9047999	106.3600006
-5.7186804	5.9535999	105.7399979
-5.7186804	6.0023999	102.8399963
-5.7186804	6.0511999	104.0000000
-5.7186804	6.0999999	102.4399948
-5.7186804	6.1487999	100.3399963
-5.7186804	6.1975999	104.0299988
-5.7186804	6.2463999	104.5100021
-5.7186804	6.2951999	106.3799973
-5.7186804	6.3439999	105.5100021
-5.7186804	6.3927999	103.2399979
-5.7186804	6.4415998	105.7399979
-5.7186804	6.4903998	107.2900009
-5.7186804	6.5391998	107.2500000
-5.7186804	6.5879998	106.1500015
-5.7186804	6.6367998	105.1199951
-5.7186804	6.6855998	101.8799973
-5.7186804	6.7343998	104.1599960
-5.7186804	6.7831998	103.7500000
-5.7186804	6.8319998	103.4700012
-5.7186804	6.8807998	103.0199966
-5.7186804	6.9295998	98.7999954
-5.7186804	6.9783998	101.7799988
-5.7186804	7.0271997	103.2299957
-5.7186804	7.0759997	104.1199951
-5.7186804	7.1247997	103.3199997
-5.7186804	7.1735997	99.5400009
-5.7186804	7.2223997	104.0499954
-5.7186804	7.2711997	106.1500015
-5.7186804	7.3199997	104.2999954
-5.7186804	7.3687997	106.6899948
-5.7186804	7.4175997	104.2900009
-5.7186804	7.4663997	101.6899948
-5.7186804	7.5151997	102.7299957
-5.7186804	7.5639997	102.7200012
-5.7186804	7.6127996	100.1399994
-5.7186804	7.6615996	97.6800003
-5.7186804	7.7103996	100.0499954
-5.7186804	7.7591996	103.8300018
-5.7186804	7.8079996	104.1999969
-5.7186804	7.8567996	103.2299957
-5.7186804	7.9056001	103.1299973
-5.7186804	7.9544001	103.6299973
-5.7186804	8.0032005	103.6800003
-5.7186804	8.0520000	105.3499985
-5.7186804	8.1008005	102.7399979
-5.7186804	8.1496000	100.6899948
-5.7186804	8.1984005	102.4000015
-5.7186804	8.2472000	103.2500000
-5.7186804	8.2960005	105.2200012
-5.7186804	8.3448000	104.4700012
-5.7186804	8.3936005	101.7900009
-5.7186804	8.4424000	102.1599960
-5.7186804	8.4912004	102.0499954
-5.7186804	8.5400000	97.8699951
-5.7186804	8.5888004	104.3300018
-5.7186804	8.6375999	100.5499954
-5.7186804	8.6864004	102.5899963

-5.7186804	8.7351999	104.2900009
-5.7186804	8.7840004	103.8199997
-5.7186804	8.8327999	105.2999954
-5.7186804	8.8816004	101.2799988
-5.7186804	8.9303999	101.4300003
-5.7186804	8.9792004	103.2699966
-5.7186804	9.0279999	104.6399994
-5.7186804	9.0768003	104.2500000
-5.7186804	9.1255999	105.9399948
-5.7186804	9.1744003	105.4399948
-5.7186804	9.2231998	103.9899979
-5.7186804	9.2720003	102.1299973
-5.7186804	9.3207998	101.2399979
-5.7186804	9.3696003	103.2699966
-5.7186804	9.4184008	105.6800003
-5.7186804	9.4672003	104.5699997
-5.7186804	9.5160007	103.8499985
-5.7186804	9.5648003	102.7600021
-5.7186804	9.6136007	103.9499969
-5.7186804	9.6624002	106.6299973
-5.7186804	9.7112007	105.1500015
-5.7186804	9.7600002	103.2699966
-5.7186804	9.8088007	102.4499969
-5.7186804	9.8576002	102.6500015
-5.7186804	9.9064007	102.7299957
-5.7186804	9.9552002	101.6699982
-5.7186804	10.0040007	99.6800003
-5.7186804	10.0528002	101.6199951
-5.7186804	10.1016006	101.0999985
-5.7186804	10.1504002	99.9799957
-5.7186804	10.1992006	101.0800018
-5.7186804	10.2480001	103.2900009
-5.7186804	10.2968006	102.4199982
-5.7186804	10.3456001	99.6500015
-5.7186804	10.3944006	101.9300003
-5.7186804	10.4432001	103.9300003
-5.7186804	10.4920006	104.9499969
-5.7186804	10.5408001	101.4399948
-5.7186804	10.5896006	103.6800003
-5.7186804	10.6384001	103.2200012
-5.7186804	10.6872005	102.1899948
-5.7186804	10.7360001	104.0699997
-5.7186804	10.7848005	101.5899963
-5.7186804	10.8336000	104.6299973
-5.7186804	10.8824005	104.1199951
-5.7186804	10.9312000	102.5299988
-5.7186804	10.9800005	100.2999954
-5.7186804	11.0288000	98.9799957
-5.7186804	11.0776005	102.1999969
-5.7186804	11.1264000	104.3499985
-5.7186804	11.1752005	103.8300018
-5.7186804	11.2240000	101.5800018
-5.7186804	11.2728004	100.5599976
-5.7186804	11.3216000	101.9700012
-5.7186804	11.3704004	103.6699982
-5.7186804	11.4191999	105.6199951
-5.7186804	11.4680004	105.9599991
-5.7186804	11.5167999	104.7600021

-5.7186804	11.5656004	103.7600021
-5.7186804	11.6143999	100.3399963
-5.7186804	11.6632004	101.1100006
-5.7186804	11.7119999	105.2099991
-5.7186804	11.7608004	106.2200012
-5.7186804	11.8095999	103.6699982
-5.7186804	11.8584003	103.1199951
-5.7186804	11.9071999	99.7099991
-5.7186804	11.9560003	101.8899994
-5.7186804	12.0047998	104.7299957
-5.7186804	12.0536003	104.0000000
-5.7186804	12.1023998	100.8199997
-5.7186804	12.1512003	102.6800003
-5.7186804	12.1999998	103.4000015
-5.7186804	12.2488003	100.3600006
-5.7186804	12.2975998	104.4199982
-5.7186804	12.3464003	104.3799973
-5.7186804	12.3951998	104.6199951
-5.7186804	12.4440002	101.7900009
-5.7186804	12.4927998	103.1800003
-5.7186804	12.5416002	100.7900009
-5.7186804	12.5904007	101.2500000
-5.7186804	12.6392002	102.9099960
-5.7186804	12.6880007	103.0599976
-5.7186804	12.7368002	104.0899963
-5.7186804	12.7856007	105.2600021
-5.7186804	12.8344002	106.0599976
-5.7186804	12.8832006	99.5599976
-5.7186804	12.9320002	101.6899948
-5.7186804	12.9808006	105.3199997
-5.7186804	13.0296001	106.0199966
-5.7186804	13.0784006	103.0199966
-5.7186804	13.1272001	102.0100021
-5.7186804	13.1760006	103.6800003
-5.7186804	13.2248001	102.7699966
-5.7186804	13.2736006	99.8899994
-5.7186804	13.3224001	101.6999969
-5.7186804	13.3712006	101.2299957
-5.7186804	13.4200001	99.2900009
-5.7186804	13.4688005	101.4399948
-5.7186804	13.5176001	102.1500015
-5.7186804	13.5664005	100.4700012
-5.7186804	13.6152000	102.8799973
-5.7186804	13.6640005	103.8899994
-5.7186804	13.7128000	98.3099976
-5.7186804	13.7616005	93.2200012
-5.7186804	13.8104000	100.3600006
-5.7186804	13.8592005	103.0400009
-5.7186804	13.9080000	102.3600006
-5.7186804	13.9568005	101.8099976
-5.7186804	14.0056000	103.3499985
-5.7186804	14.0544004	97.7699966
-5.7186804	14.1032000	101.3499985
-5.7186804	14.1520004	102.6500015
-5.7186804	14.2007999	101.7399979
-5.7186804	14.2496004	102.6699982
-5.7186804	14.2983999	101.6399994
-5.7186804	14.3472004	95.9799957

-5.7186804	14.3959999	99.7200012
-5.7186804	14.4448004	99.6299973
-5.7186804	14.4935999	101.0400009
-5.7186804	14.5424004	99.3399963
-5.7186804	14.5911999	100.1999969
-5.7186804	14.6400003	101.4599991
-5.7186804	14.6887999	103.6699982
-5.7186804	14.7376003	103.1399994
-5.7186804	14.7863998	105.4300003
-5.7186804	14.8352003	103.4899979
-5.7186804	14.8839998	103.1299973
-5.7186804	14.9328003	101.3300018
-5.7186804	14.9815998	102.0599976
-5.7186804	15.0304003	101.7999954
-5.7125444	0.3416000	107.3899994
-5.7125444	0.3904000	107.2500000
-5.7125444	0.4392000	107.9599991
-5.7125444	0.4880000	105.3199997
-5.7125444	0.5368000	106.7200012
-5.7125444	0.5856000	104.4700012
-5.7125444	0.6344000	104.9599991
-5.7125444	0.6832000	107.5699997
-5.7125444	0.7320000	107.4199982
-5.7125444	0.7808000	105.9899979
-5.7125444	0.8296000	107.0999985
-5.7125444	0.8784000	105.6299973
-5.7125444	0.9272000	106.1299973
-5.7125444	0.9760000	107.1399994
-5.7125444	1.0247999	106.4199982
-5.7125444	1.0735999	105.2699966
-5.7125444	1.1223999	103.2399979
-5.7125444	1.1711999	99.9000015
-5.7125444	1.2200000	106.1899948
-5.7125444	1.2688000	107.6999969
-5.7125444	1.3176000	107.2500000
-5.7125444	1.3664000	106.5000000
-5.7125444	1.4152000	109.0199966
-5.7125444	1.4640000	108.8899994
-5.7125444	1.5128000	109.3699951
-5.7125444	1.5616000	106.7900009
-5.7125444	1.6104000	102.9099960
-5.7125444	1.6592000	102.3300018
-5.7125444	1.7079999	101.6899948
-5.7125444	1.7567999	104.0999985
-5.7125444	1.8055999	105.4300003
-5.7125444	1.8543999	108.6299973
-5.7125444	1.9031999	111.5899963
-5.7125444	1.9519999	109.5400009
-5.7125444	2.0007999	105.1699982
-5.7125444	2.0495999	104.2999954
-5.7125444	2.0983999	105.4000015
-5.7125444	2.1471999	106.9899979
-5.7125444	2.1959999	104.7200012
-5.7125444	2.2447999	108.5499954
-5.7125444	2.2935998	109.7399979
-5.7125444	2.3423998	107.4499969
-5.7125444	2.3912001	105.9399948
-5.7125444	2.4400001	104.1999969

-5.7125444	2.4888000	105.7299957
-5.7125444	2.5376000	102.9700012
-5.7125444	2.5864000	106.8199997
-5.7125444	2.6352000	106.9799957
-5.7125444	2.6840000	106.2999954
-5.7125444	2.7328000	104.1800003
-5.7125444	2.7816000	103.4300003
-5.7125444	2.8304000	103.0199966
-5.7125444	2.8792000	103.9599991
-5.7125444	2.9280000	104.8600006
-5.7125444	2.9768000	105.0800018
-5.7125444	3.0256000	105.9499969
-5.7125444	3.0743999	106.2600021
-5.7125444	3.1231999	104.4799957
-5.7125444	3.1719999	103.1599960
-5.7125444	3.2207999	101.5599976
-5.7125444	3.2695999	105.2299957
-5.7125444	3.3183999	107.0400009
-5.7125444	3.3671999	103.7600021
-5.7125444	3.4159999	104.4499969
-5.7125444	3.4647999	104.4499969
-5.7125444	3.5135999	102.4199982
-5.7125444	3.5623999	101.7600021
-5.7125444	3.6111999	105.1500015
-5.7125444	3.6599998	100.3499985
-5.7125444	3.7087998	99.9599991
-5.7125444	3.7575998	101.8899994
-5.7125444	3.8063998	104.0400009
-5.7125444	3.8551998	104.7699966
-5.7125444	3.9039998	105.8199997
-5.7125444	3.9527998	105.4700012
-5.7125444	4.0016003	106.5199966
-5.7125444	4.0503998	106.5400009
-5.7125444	4.0991998	105.1800003
-5.7125444	4.1479998	102.2900009
-5.7125444	4.1967998	103.7099991
-5.7125444	4.2455997	102.8600006
-5.7125444	4.2943997	105.8600006
-5.7125444	4.3431997	106.2699966
-5.7125444	4.3919997	106.0999985
-5.7125444	4.4407997	102.5000000
-5.7125444	4.4895997	104.3799973
-5.7125444	4.5383997	105.9700012
-5.7125444	4.5871997	109.4199982
-5.7125444	4.6359997	113.1199951
-5.7125444	4.6847997	116.1999969
-5.7125444	4.7336001	114.6199951
-5.7125444	4.7824001	107.7200012
-5.7125444	4.8312001	103.8499985
-5.7125444	4.8800001	105.8799973
-5.7125444	4.9288001	100.2799988
-5.7125444	4.9776001	99.3600006
-5.7125444	5.0264001	103.7600021
-5.7125444	5.0752001	104.1800003
-5.7125444	5.1240001	99.2399979
-5.7125444	5.1728001	103.1299973
-5.7125444	5.2216001	106.2200012
-5.7125444	5.2704000	103.9300003

-5.7125444	5.3192000	106.1699982
-5.7125444	5.3680000	106.8399963
-5.7125444	5.4168000	106.8300018
-5.7125444	5.4656000	100.7399979
-5.7125444	5.5144000	103.0499954
-5.7125444	5.5632000	104.5599976
-5.7125444	5.6120000	103.9000015
-5.7125444	5.6608000	102.3899994
-5.7125444	5.7096000	103.6899948
-5.7125444	5.7584000	102.2200012
-5.7125444	5.8072000	101.2799988
-5.7125444	5.8559999	99.9700012
-5.7125444	5.9047999	105.2200012
-5.7125444	5.9535999	105.0100021
-5.7125444	6.0023999	103.0800018
-5.7125444	6.0511999	104.6500015
-5.7125444	6.0999999	103.4599991
-5.7125444	6.1487999	99.2999954
-5.7125444	6.1975999	103.7999954
-5.7125444	6.2463999	105.1699982
-5.7125444	6.2951999	106.9399948
-5.7125444	6.3439999	105.6599960
-5.7125444	6.3927999	104.4899979
-5.7125444	6.4415998	106.2299957
-5.7125444	6.4903998	107.6699982
-5.7125444	6.5391998	107.3499985
-5.7125444	6.5879998	105.8099976
-5.7125444	6.6367998	105.4899979
-5.7125444	6.6855998	103.1699982
-5.7125444	6.7343998	105.2500000
-5.7125444	6.7831998	104.3600006
-5.7125444	6.8319998	104.0699997
-5.7125444	6.8807998	103.4899979
-5.7125444	6.9295998	99.8499985
-5.7125444	6.9783998	101.9799957
-5.7125444	7.0271997	103.5999985
-5.7125444	7.0759997	103.6500015
-5.7125444	7.1247997	102.8499985
-5.7125444	7.1735997	98.7399979
-5.7125444	7.2223997	104.2699966
-5.7125444	7.2711997	106.2600021
-5.7125444	7.3199997	103.5899963
-5.7125444	7.3687997	106.7299957
-5.7125444	7.4175997	106.2099991
-5.7125444	7.4663997	103.7600021
-5.7125444	7.5151997	102.7200012
-5.7125444	7.5639997	101.4099960
-5.7125444	7.6127996	98.5199966
-5.7125444	7.6615996	96.3699951
-5.7125444	7.7103996	100.2200012
-5.7125444	7.7591996	103.9300003
-5.7125444	7.8079996	104.3799973
-5.7125444	7.8567996	103.1699982
-5.7125444	7.9056001	102.7799988
-5.7125444	7.9544001	103.9599991
-5.7125444	8.0032005	103.9000015
-5.7125444	8.0520000	105.3300018
-5.7125444	8.1008005	103.3300018

-5.7125444	8.1496000	100.8199997
-5.7125444	8.1984005	102.9000015
-5.7125444	8.2472000	101.2999954
-5.7125444	8.2960005	104.2799988
-5.7125444	8.3448000	104.4300003
-5.7125444	8.3936005	101.0199966
-5.7125444	8.4424000	100.7299957
-5.7125444	8.4912004	101.9899979
-5.7125444	8.5400000	99.7900009
-5.7125444	8.5888004	104.6299973
-5.7125444	8.6375999	100.5499954
-5.7125444	8.6864004	102.9300003
-5.7125444	8.7351999	104.9599991
-5.7125444	8.7840004	103.1699982
-5.7125444	8.8327999	104.3499985
-5.7125444	8.8816004	100.8300018
-5.7125444	8.9303999	101.0299988
-5.7125444	8.9792004	102.3899994
-5.7125444	9.0279999	103.9799957
-5.7125444	9.0768003	104.0400009
-5.7125444	9.1255999	104.2900009
-5.7125444	9.1744003	104.2600021
-5.7125444	9.2231998	102.9700012
-5.7125444	9.2720003	102.3499985
-5.7125444	9.3207998	101.0800018
-5.7125444	9.3696003	102.2099991
-5.7125444	9.4184008	104.9899979
-5.7125444	9.4672003	104.9000015
-5.7125444	9.5160007	103.9700012
-5.7125444	9.5648003	101.5400009
-5.7125444	9.6136007	103.9499969
-5.7125444	9.6624002	106.1599960
-5.7125444	9.7112007	105.2200012
-5.7125444	9.7600002	103.1699982
-5.7125444	9.8088007	102.2699966
-5.7125444	9.8576002	101.7200012
-5.7125444	9.9064007	102.1999969
-5.7125444	9.9552002	100.6999969
-5.7125444	10.0040007	99.6800003
-5.7125444	10.0528002	102.2099991
-5.7125444	10.1016006	100.6199951
-5.7125444	10.1504002	99.8199997
-5.7125444	10.1992006	101.2099991
-5.7125444	10.2480001	103.2699966
-5.7125444	10.2968006	103.2699966
-5.7125444	10.3456001	102.0599976
-5.7125444	10.3944006	103.9899979
-5.7125444	10.4432001	104.8799973
-5.7125444	10.4920006	105.1399994
-5.7125444	10.5408001	101.9700012
-5.7125444	10.5896006	103.9599991
-5.7125444	10.6384001	103.4199982
-5.7125444	10.6872005	101.4099960
-5.7125444	10.7360001	103.4899979
-5.7125444	10.7848005	101.9399948
-5.7125444	10.8336000	105.2399979
-5.7125444	10.8824005	104.0599976
-5.7125444	10.9312000	101.8199997

-5.7125444	10.9800005	100.6800003
-5.7125444	11.0288000	100.1999969
-5.7125444	11.0776005	102.8499985
-5.7125444	11.1264000	104.9000015
-5.7125444	11.1752005	103.6699982
-5.7125444	11.2240000	102.2099991
-5.7125444	11.2728004	100.5599976
-5.7125444	11.3216000	101.3399963
-5.7125444	11.3704004	103.4000015
-5.7125444	11.4191999	104.7900009
-5.7125444	11.4680004	105.5000000
-5.7125444	11.5167999	103.6599960
-5.7125444	11.5656004	103.2900009
-5.7125444	11.6143999	100.4399948
-5.7125444	11.6632004	100.5800018
-5.7125444	11.7119999	104.0699997
-5.7125444	11.7608004	105.9399948
-5.7125444	11.8095999	103.7399979
-5.7125444	11.8584003	102.4799957
-5.7125444	11.9071999	99.9099960
-5.7125444	11.9560003	102.0499954
-5.7125444	12.0047998	104.9399948
-5.7125444	12.0536003	105.3899994
-5.7125444	12.1023998	101.9700012
-5.7125444	12.1512003	103.0499954
-5.7125444	12.1999998	102.9000015
-5.7125444	12.2488003	99.6199951
-5.7125444	12.2975998	104.3399963
-5.7125444	12.3464003	103.8300018
-5.7125444	12.3951998	105.4000015
-5.7125444	12.4440002	103.6399994
-5.7125444	12.4927998	103.2200012
-5.7125444	12.5416002	100.3499985
-5.7125444	12.5904007	102.0800018
-5.7125444	12.6392002	104.5599976
-5.7125444	12.6880007	104.4099960
-5.7125444	12.7368002	103.6299973
-5.7125444	12.7856007	104.7500000
-5.7125444	12.8344002	105.6500015
-5.7125444	12.8832006	99.9499969
-5.7125444	12.9320002	101.5000000
-5.7125444	12.9808006	105.5000000
-5.7125444	13.0296001	106.1699982
-5.7125444	13.0784006	102.5499954
-5.7125444	13.1272001	100.8899994
-5.7125444	13.1760006	102.8799973
-5.7125444	13.2248001	101.2399979
-5.7125444	13.2736006	98.4700012
-5.7125444	13.3224001	100.5100021
-5.7125444	13.3712006	100.6899948
-5.7125444	13.4200001	99.1599960
-5.7125444	13.4688005	101.4700012
-5.7125444	13.5176001	102.3600006
-5.7125444	13.5664005	99.8099976
-5.7125444	13.6152000	101.7399979
-5.7125444	13.6640005	102.9899979
-5.7125444	13.7128000	98.6399994
-5.7125444	13.7616005	93.8600006

-5.7125444	13.8104000	99.5499954
-5.7125444	13.8592005	102.4199982
-5.7125444	13.9080000	102.9300003
-5.7125444	13.9568005	102.7200012
-5.7125444	14.0056000	103.6199951
-5.7125444	14.0544004	98.2600021
-5.7125444	14.1032000	101.4199982
-5.7125444	14.1520004	101.6399994
-5.7125444	14.2007999	100.4599991
-5.7125444	14.2496004	100.8799973
-5.7125444	14.2983999	100.8699951
-5.7125444	14.3472004	95.5199966
-5.7125444	14.3959999	99.5499954
-5.7125444	14.4448004	99.4099960
-5.7125444	14.4935999	101.1800003
-5.7125444	14.5424004	99.6899948
-5.7125444	14.5911999	100.1800003
-5.7125444	14.6400003	100.6800003
-5.7125444	14.6887999	102.9799957
-5.7125444	14.7376003	103.2900009
-5.7125444	14.7863998	106.0100021
-5.7125444	14.8352003	104.1999969
-5.7125444	14.8839998	103.5499954
-5.7125444	14.9328003	102.7099991
-5.7125444	14.9815998	103.1100006
-5.7125444	15.0304003	101.8399963
-5.7064085	0.3416000	106.4099960
-5.7064085	0.3904000	105.1899948
-5.7064085	0.4392000	107.2799988
-5.7064085	0.4880000	105.5699997
-5.7064085	0.5368000	106.5000000
-5.7064085	0.5856000	103.7600021
-5.7064085	0.6344000	104.4300003
-5.7064085	0.6832000	107.4099960
-5.7064085	0.7320000	107.8099976
-5.7064085	0.7808000	105.7799988
-5.7064085	0.8296000	107.2999954
-5.7064085	0.8784000	105.8399963
-5.7064085	0.9272000	106.0699997
-5.7064085	0.9760000	107.4599991
-5.7064085	1.0247999	106.4399948
-5.7064085	1.0735999	105.0400009
-5.7064085	1.1223999	103.2600021
-5.7064085	1.1711999	100.5100021
-5.7064085	1.2200000	107.0599976
-5.7064085	1.2688000	107.2200012
-5.7064085	1.3176000	106.5699997
-5.7064085	1.3664000	107.0199966
-5.7064085	1.4152000	109.0999985
-5.7064085	1.4640000	107.9099960
-5.7064085	1.5128000	108.8799973
-5.7064085	1.5616000	106.9300003
-5.7064085	1.6104000	103.1500015
-5.7064085	1.6592000	102.4700012
-5.7064085	1.7079999	101.9499969
-5.7064085	1.7567999	104.0499954
-5.7064085	1.8055999	105.8399963
-5.7064085	1.8543999	109.0499954

-5.7064085	1.9031999	111.6399994
-5.7064085	1.9519999	109.3799973
-5.7064085	2.0007999	105.8699951
-5.7064085	2.0495999	105.7099991
-5.7064085	2.0983999	106.1100006
-5.7064085	2.1471999	107.3799973
-5.7064085	2.1959999	105.2999954
-5.7064085	2.2447999	108.5800018
-5.7064085	2.2935998	110.0400009
-5.7064085	2.3423998	107.8799973
-5.7064085	2.3912001	105.9099960
-5.7064085	2.4400001	103.7900009
-5.7064085	2.4888000	105.3499985
-5.7064085	2.5376000	103.5899963
-5.7064085	2.5864000	106.4899979
-5.7064085	2.6352000	106.0699997
-5.7064085	2.6840000	106.1399994
-5.7064085	2.7328000	104.0000000
-5.7064085	2.7816000	102.1999969
-5.7064085	2.8304000	103.0499954
-5.7064085	2.8792000	103.7500000
-5.7064085	2.9280000	104.5699997
-5.7064085	2.9768000	105.3899994
-5.7064085	3.0256000	105.4499969
-5.7064085	3.0743999	106.0499954
-5.7064085	3.1231999	103.3699951
-5.7064085	3.1719999	103.0299988
-5.7064085	3.2207999	102.1999969
-5.7064085	3.2695999	105.3199997
-5.7064085	3.3183999	106.4899979
-5.7064085	3.3671999	103.7699966
-5.7064085	3.4159999	104.2699966
-5.7064085	3.4647999	103.4899979
-5.7064085	3.5135999	101.9499969
-5.7064085	3.5623999	101.2500000
-5.7064085	3.6111999	104.4700012
-5.7064085	3.6599998	101.4499969
-5.7064085	3.7087998	101.6399994
-5.7064085	3.7575998	102.5699997
-5.7064085	3.8063998	103.5499954
-5.7064085	3.8551998	104.5299988
-5.7064085	3.9039998	105.7600021
-5.7064085	3.9527998	105.2099991
-5.7064085	4.0016003	106.4700012
-5.7064085	4.0503998	107.1999969
-5.7064085	4.0991998	105.4399948
-5.7064085	4.1479998	102.5400009
-5.7064085	4.1967998	103.1399994
-5.7064085	4.2455997	102.4899979
-5.7064085	4.2943997	106.2299957
-5.7064085	4.3431997	107.5899963
-5.7064085	4.3919997	107.3600006
-5.7064085	4.4407997	103.6199951
-5.7064085	4.4895997	104.3699951
-5.7064085	4.5383997	106.4599991
-5.7064085	4.5871997	109.7699966
-5.7064085	4.6359997	113.4199982
-5.7064085	4.6847997	115.8099976

-5.7064085	4.7336001	114.5000000
-5.7064085	4.7824001	108.0400009
-5.7064085	4.8312001	104.8099976
-5.7064085	4.8800001	106.7699966
-5.7064085	4.9288001	101.9499969
-5.7064085	4.9776001	99.4799957
-5.7064085	5.0264001	103.8799973
-5.7064085	5.0752001	103.6100006
-5.7064085	5.1240001	99.6999969
-5.7064085	5.1728001	103.3799973
-5.7064085	5.2216001	106.4700012
-5.7064085	5.2704000	104.9300003
-5.7064085	5.3192000	106.5999985
-5.7064085	5.3680000	106.6500015
-5.7064085	5.4168000	106.1100006
-5.7064085	5.4656000	101.0000000
-5.7064085	5.5144000	102.0599976
-5.7064085	5.5632000	103.1299973
-5.7064085	5.6120000	103.3099976
-5.7064085	5.6608000	102.8699951
-5.7064085	5.7096000	104.5000000
-5.7064085	5.7584000	103.3300018
-5.7064085	5.8072000	101.1800003
-5.7064085	5.8559999	99.1299973
-5.7064085	5.9047999	103.7999954
-5.7064085	5.9535999	103.9799957
-5.7064085	6.0023999	102.6199951
-5.7064085	6.0511999	104.5699997
-5.7064085	6.0999999	103.9300003
-5.7064085	6.1487999	97.8600006
-5.7064085	6.1975999	103.8199997
-5.7064085	6.2463999	105.5999985
-5.7064085	6.2951999	106.8300018
-5.7064085	6.3439999	105.0499954
-5.7064085	6.3927999	105.1999969
-5.7064085	6.4415998	106.7099991
-5.7064085	6.4903998	107.7399979
-5.7064085	6.5391998	107.0800018
-5.7064085	6.5879998	105.0699997
-5.7064085	6.6367998	104.9700012
-5.7064085	6.6855998	103.7299957
-5.7064085	6.7343998	105.8899994
-5.7064085	6.7831998	104.7999954
-5.7064085	6.8319998	103.5100021
-5.7064085	6.8807998	102.9700012
-5.7064085	6.9295998	100.7900009
-5.7064085	6.9783998	103.0499954
-5.7064085	7.0271997	104.5599976
-5.7064085	7.0759997	103.3099976
-5.7064085	7.1247997	102.4799957
-5.7064085	7.1735997	98.9300003
-5.7064085	7.2223997	104.7500000
-5.7064085	7.2711997	106.6999969
-5.7064085	7.3199997	103.9599991
-5.7064085	7.3687997	106.4799957
-5.7064085	7.4175997	107.2600021
-5.7064085	7.4663997	105.1800003
-5.7064085	7.5151997	103.1800003

-5.7064085	7.5639997	100.9099960
-5.7064085	7.6127996	98.1699982
-5.7064085	7.6615996	97.2299957
-5.7064085	7.7103996	100.4700012
-5.7064085	7.7591996	103.5000000
-5.7064085	7.8079996	104.0499954
-5.7064085	7.8567996	102.8199997
-5.7064085	7.9056001	101.6999969
-5.7064085	7.9544001	103.5699997
-5.7064085	8.0032005	104.0100021
-5.7064085	8.0520000	105.9700012
-5.7064085	8.1008005	104.2299957
-5.7064085	8.1496000	100.7999954
-5.7064085	8.1984005	103.7099991
-5.7064085	8.2472000	100.2399979
-5.7064085	8.2960005	103.9399948
-5.7064085	8.3448000	104.3600006
-5.7064085	8.3936005	100.6500015
-5.7064085	8.4424000	99.4199982
-5.7064085	8.4912004	101.0999985
-5.7064085	8.5400000	100.9099960
-5.7064085	8.5888004	104.0999985
-5.7064085	8.6375999	100.1599960
-5.7064085	8.6864004	103.9599991
-5.7064085	8.7351999	105.5199966
-5.7064085	8.7840004	102.7799988
-5.7064085	8.8327999	103.8099976
-5.7064085	8.8816004	100.0899963
-5.7064085	8.9303999	100.1399994
-5.7064085	8.9792004	101.6999969
-5.7064085	9.0279999	103.5100021
-5.7064085	9.0768003	103.7600021
-5.7064085	9.1255999	102.9300003
-5.7064085	9.1744003	104.2600021
-5.7064085	9.2231998	103.3099976
-5.7064085	9.2720003	103.1500015
-5.7064085	9.3207998	100.9700012
-5.7064085	9.3696003	101.2200012
-5.7064085	9.4184008	105.0199966
-5.7064085	9.4672003	105.6899948
-5.7064085	9.5160007	104.1599960
-5.7064085	9.5648003	101.1199951
-5.7064085	9.6136007	104.4199982
-5.7064085	9.6624002	105.6299973
-5.7064085	9.7112007	105.1599960
-5.7064085	9.7600002	102.5400009
-5.7064085	9.8088007	102.3399963
-5.7064085	9.8576002	101.5299988
-5.7064085	9.9064007	102.1599960
-5.7064085	9.9552002	99.5899963
-5.7064085	10.0040007	99.0899963
-5.7064085	10.0528002	103.0199966
-5.7064085	10.1016006	101.5299988
-5.7064085	10.1504002	99.5000000
-5.7064085	10.1992006	101.1699982
-5.7064085	10.2480001	103.1199951
-5.7064085	10.2968006	103.6199951
-5.7064085	10.3456001	103.5800018

-5.7064085	10.3944006	105.0800018
-5.7064085	10.4432001	105.1500015
-5.7064085	10.4920006	104.8399963
-5.7064085	10.5408001	102.2900009
-5.7064085	10.5896006	103.8300018
-5.7064085	10.6384001	102.1999969
-5.7064085	10.6872005	100.6399994
-5.7064085	10.7360001	102.9899979
-5.7064085	10.7848005	102.8099976
-5.7064085	10.8336000	105.2699966
-5.7064085	10.8824005	103.7399979
-5.7064085	10.9312000	100.9000015
-5.7064085	10.9800005	100.5800018
-5.7064085	11.0288000	101.2399979
-5.7064085	11.0776005	103.7600021
-5.7064085	11.1264000	105.2999954
-5.7064085	11.1752005	103.0999985
-5.7064085	11.2240000	102.5699997
-5.7064085	11.2728004	100.7099991
-5.7064085	11.3216000	101.0400009
-5.7064085	11.3704004	103.4199982
-5.7064085	11.4191999	103.7999954
-5.7064085	11.4680004	105.1999969
-5.7064085	11.5167999	103.3699951
-5.7064085	11.5656004	103.5000000
-5.7064085	11.6143999	100.9000015
-5.7064085	11.6632004	100.1699982
-5.7064085	11.7119999	102.7600021
-5.7064085	11.7608004	105.2399979
-5.7064085	11.8095999	103.4000015
-5.7064085	11.8584003	101.7900009
-5.7064085	11.9071999	100.3600006
-5.7064085	11.9560003	102.8099976
-5.7064085	12.0047998	104.8099976
-5.7064085	12.0536003	106.1699982
-5.7064085	12.1023998	102.2999954
-5.7064085	12.1512003	102.8399963
-5.7064085	12.1999998	102.3199997
-5.7064085	12.2488003	99.4099960
-5.7064085	12.2975998	104.1599960
-5.7064085	12.3464003	102.7799988
-5.7064085	12.3951998	105.6800003
-5.7064085	12.4440002	104.9099960
-5.7064085	12.4927998	102.7799988
-5.7064085	12.5416002	98.7799988
-5.7064085	12.5904007	103.0000000
-5.7064085	12.6392002	106.0199966
-5.7064085	12.6880007	105.7900009
-5.7064085	12.7368002	104.0999985
-5.7064085	12.7856007	103.6899948
-5.7064085	12.8344002	104.3799973
-5.7064085	12.8832006	99.8399963
-5.7064085	12.9320002	101.7799988
-5.7064085	12.9808006	105.8099976
-5.7064085	13.0296001	106.3099976
-5.7064085	13.0784006	102.3799973
-5.7064085	13.1272001	100.7500000
-5.7064085	13.1760006	102.7500000

-5.7064085	13.2248001	100.8099976
-5.7064085	13.2736006	98.8899994
-5.7064085	13.3224001	100.1999969
-5.7064085	13.3712006	100.8099976
-5.7064085	13.4200001	100.1899948
-5.7064085	13.4688005	100.7399979
-5.7064085	13.5176001	102.4899979
-5.7064085	13.5664005	99.6399994
-5.7064085	13.6152000	101.2399979
-5.7064085	13.6640005	102.2600021
-5.7064085	13.7128000	99.7099991
-5.7064085	13.7616005	95.7900009
-5.7064085	13.8104000	99.4700012
-5.7064085	13.8592005	101.8600006
-5.7064085	13.9080000	103.3499985
-5.7064085	13.9568005	103.2399979
-5.7064085	14.0056000	103.0499954
-5.7064085	14.0544004	98.1299973
-5.7064085	14.1032000	101.2299957
-5.7064085	14.1520004	100.7600021
-5.7064085	14.2007999	99.7699966
-5.7064085	14.2496004	99.5899963
-5.7064085	14.2983999	100.3699951
-5.7064085	14.3472004	97.1100006
-5.7064085	14.3959999	100.6999969
-5.7064085	14.4448004	99.5699997
-5.7064085	14.4935999	101.5299988
-5.7064085	14.5424004	100.6899948
-5.7064085	14.5911999	101.1199951
-5.7064085	14.6400003	100.6800003
-5.7064085	14.6887999	102.7099991
-5.7064085	14.7376003	103.6199951
-5.7064085	14.7863998	106.4099960
-5.7064085	14.8352003	104.5899963
-5.7064085	14.8839998	103.2399979
-5.7064085	14.9328003	103.4399948
-5.7064085	14.9815998	103.9700012
-5.7064085	15.0304003	101.9599991
-5.7002726	0.3416000	106.6100006
-5.7002726	0.3904000	104.0299988
-5.7002726	0.4392000	106.9799957
-5.7002726	0.4880000	105.6599960
-5.7002726	0.5368000	105.9799957
-5.7002726	0.5856000	103.2500000
-5.7002726	0.6344000	103.5800018
-5.7002726	0.6832000	106.7399979
-5.7002726	0.7320000	107.8099976
-5.7002726	0.7808000	105.9199982
-5.7002726	0.8296000	107.7699966
-5.7002726	0.8784000	106.2799988
-5.7002726	0.9272000	106.0800018
-5.7002726	0.9760000	108.0499954
-5.7002726	1.0247999	106.5599976
-5.7002726	1.0735999	104.6800003
-5.7002726	1.1223999	104.1599960
-5.7002726	1.1711999	100.5400009
-5.7002726	1.2200000	106.7900009
-5.7002726	1.2688000	106.3199997

-5.7002726	1.3176000	105.9099960
-5.7002726	1.3664000	107.1299973
-5.7002726	1.4152000	109.5199966
-5.7002726	1.4640000	108.0100021
-5.7002726	1.5128000	108.4399948
-5.7002726	1.5616000	106.5699997
-5.7002726	1.6104000	104.2099991
-5.7002726	1.6592000	102.8899994
-5.7002726	1.7079999	101.7699966
-5.7002726	1.7567999	103.7900009
-5.7002726	1.8055999	105.9799957
-5.7002726	1.8543999	109.1299973
-5.7002726	1.9031999	111.2699966
-5.7002726	1.9519999	108.9499969
-5.7002726	2.0007999	106.3699951
-5.7002726	2.0495999	106.6599960
-5.7002726	2.0983999	106.9000015
-5.7002726	2.1471999	107.6899948
-5.7002726	2.1959999	106.0400009
-5.7002726	2.2447999	108.0800018
-5.7002726	2.2935998	109.7799988
-5.7002726	2.3423998	108.0299988
-5.7002726	2.3912001	105.6100006
-5.7002726	2.4400001	102.9300003
-5.7002726	2.4888000	105.6100006
-5.7002726	2.5376000	104.6999969
-5.7002726	2.5864000	106.3099976
-5.7002726	2.6352000	105.3699951
-5.7002726	2.6840000	106.8899994
-5.7002726	2.7328000	104.6800003
-5.7002726	2.7816000	102.4099960
-5.7002726	2.8304000	103.1800003
-5.7002726	2.8792000	103.1599960
-5.7002726	2.9280000	104.2299957
-5.7002726	2.9768000	105.6999969
-5.7002726	3.0256000	104.8199997
-5.7002726	3.0743999	105.5499954
-5.7002726	3.1231999	102.0800018
-5.7002726	3.1719999	103.0899963
-5.7002726	3.2207999	102.9099960
-5.7002726	3.2695999	105.2900009
-5.7002726	3.3183999	105.8399963
-5.7002726	3.3671999	103.4000015
-5.7002726	3.4159999	103.0499954
-5.7002726	3.4647999	103.3199997
-5.7002726	3.5135999	101.5800018
-5.7002726	3.5623999	100.8399963
-5.7002726	3.6111999	103.7900009
-5.7002726	3.6599998	102.7399979
-5.7002726	3.7087998	103.6399994
-5.7002726	3.7575998	103.7799988
-5.7002726	3.8063998	103.5000000
-5.7002726	3.8551998	104.2099991
-5.7002726	3.9039998	105.5599976
-5.7002726	3.9527998	104.8499985
-5.7002726	4.0016003	105.7600021
-5.7002726	4.0503998	106.9000015
-5.7002726	4.0991998	105.2500000

-5.7002726	4.1479998	102.5000000
-5.7002726	4.1967998	102.5000000
-5.7002726	4.2455997	102.1599960
-5.7002726	4.2943997	106.6299973
-5.7002726	4.3431997	108.9599991
-5.7002726	4.3919997	108.0800018
-5.7002726	4.4407997	103.9899979
-5.7002726	4.4895997	104.5899963
-5.7002726	4.5383997	107.5100021
-5.7002726	4.5871997	110.1899948
-5.7002726	4.6359997	113.5400009
-5.7002726	4.6847997	115.6299973
-5.7002726	4.7336001	114.3799973
-5.7002726	4.7824001	108.6899948
-5.7002726	4.8312001	106.3199997
-5.7002726	4.8800001	107.7200012
-5.7002726	4.9288001	103.4199982
-5.7002726	4.9776001	99.7299957
-5.7002726	5.0264001	104.0999985
-5.7002726	5.0752001	103.8300018
-5.7002726	5.1240001	101.3399963
-5.7002726	5.1728001	103.7900009
-5.7002726	5.2216001	106.2999954
-5.7002726	5.2704000	104.6999969
-5.7002726	5.3192000	105.5899963
-5.7002726	5.3680000	105.7099991
-5.7002726	5.4168000	105.5199966
-5.7002726	5.4656000	101.4599991
-5.7002726	5.5144000	102.2399979
-5.7002726	5.5632000	102.7099991
-5.7002726	5.6120000	103.7200012
-5.7002726	5.6608000	104.5599976
-5.7002726	5.7096000	105.8600006
-5.7002726	5.7584000	104.3799973
-5.7002726	5.8072000	101.2900009
-5.7002726	5.8559999	99.2999954
-5.7002726	5.9047999	102.9399948
-5.7002726	5.9535999	103.4700012
-5.7002726	6.0023999	103.0899963
-5.7002726	6.0511999	104.9599991
-5.7002726	6.0999999	104.3099976
-5.7002726	6.1487999	97.1399994
-5.7002726	6.1975999	104.2999954
-5.7002726	6.2463999	105.5299988
-5.7002726	6.2951999	106.5699997
-5.7002726	6.3439999	104.1699982
-5.7002726	6.3927999	105.0499954
-5.7002726	6.4415998	107.0699997
-5.7002726	6.4903998	108.0800018
-5.7002726	6.5391998	106.7799988
-5.7002726	6.5879998	104.7200012
-5.7002726	6.6367998	104.6299973
-5.7002726	6.6855998	103.8199997
-5.7002726	6.7343998	106.1100006
-5.7002726	6.7831998	104.8399963
-5.7002726	6.8319998	103.1699982
-5.7002726	6.8807998	102.8300018
-5.7002726	6.9295998	101.6599960

-5.7002726	6.9783998	103.9799957
-5.7002726	7.0271997	105.5899963
-5.7002726	7.0759997	103.4799957
-5.7002726	7.1247997	102.4300003
-5.7002726	7.1735997	98.9399948
-5.7002726	7.2223997	105.0100021
-5.7002726	7.2711997	107.0400009
-5.7002726	7.3199997	103.8499985
-5.7002726	7.3687997	105.4300003
-5.7002726	7.4175997	107.5299988
-5.7002726	7.4663997	105.3600006
-5.7002726	7.5151997	102.7699966
-5.7002726	7.5639997	100.2099991
-5.7002726	7.6127996	98.2699966
-5.7002726	7.6615996	98.4799957
-5.7002726	7.7103996	100.8600006
-5.7002726	7.7591996	102.8300018
-5.7002726	7.8079996	103.4899979
-5.7002726	7.8567996	102.5100021
-5.7002726	7.9056001	100.7699966
-5.7002726	7.9544001	103.5899963
-5.7002726	8.0032005	104.7600021
-5.7002726	8.0520000	106.0599976
-5.7002726	8.1008005	104.7600021
-5.7002726	8.1496000	101.7999954
-5.7002726	8.1984005	103.5599976
-5.7002726	8.2472000	98.6899948
-5.7002726	8.2960005	103.1399994
-5.7002726	8.3448000	104.1199951
-5.7002726	8.3936005	100.6500015
-5.7002726	8.4424000	98.7299957
-5.7002726	8.4912004	100.7299957
-5.7002726	8.5400000	101.5299988
-5.7002726	8.5888004	103.8199997
-5.7002726	8.6375999	101.0299988
-5.7002726	8.6864004	104.6299973
-5.7002726	8.7351999	106.4799957
-5.7002726	8.7840004	103.7299957
-5.7002726	8.8327999	103.5499954
-5.7002726	8.8816004	100.1299973
-5.7002726	8.9303999	100.2699966
-5.7002726	8.9792004	102.0400009
-5.7002726	9.0279999	103.5699997
-5.7002726	9.0768003	103.3699951
-5.7002726	9.1255999	101.5699997
-5.7002726	9.1744003	104.0699997
-5.7002726	9.2231998	103.0100021
-5.7002726	9.2720003	102.8499985
-5.7002726	9.3207998	100.2299957
-5.7002726	9.3696003	100.6599960
-5.7002726	9.4184008	105.2200012
-5.7002726	9.4672003	105.9199982
-5.7002726	9.5160007	104.3899994
-5.7002726	9.5648003	100.7600021
-5.7002726	9.6136007	105.0400009
-5.7002726	9.6624002	105.5199966
-5.7002726	9.7112007	105.5599976
-5.7002726	9.7600002	102.4099960

-5.7002726	9.8088007	103.0000000
-5.7002726	9.8576002	102.0199966
-5.7002726	9.9064007	102.5699997
-5.7002726	9.9552002	99.4700012
-5.7002726	10.0040007	99.4000015
-5.7002726	10.0528002	103.4599991
-5.7002726	10.1016006	102.2699966
-5.7002726	10.1504002	99.5499954
-5.7002726	10.1992006	101.3799973
-5.7002726	10.2480001	103.1100006
-5.7002726	10.2968006	104.3099976
-5.7002726	10.3456001	104.5499954
-5.7002726	10.3944006	105.4199982
-5.7002726	10.4432001	105.2299957
-5.7002726	10.4920006	104.5199966
-5.7002726	10.5408001	101.9199982
-5.7002726	10.5896006	103.1699982
-5.7002726	10.6384001	101.2299957
-5.7002726	10.6872005	100.7500000
-5.7002726	10.7360001	102.6599960
-5.7002726	10.7848005	103.1599960
-5.7002726	10.8336000	104.9399948
-5.7002726	10.8824005	103.3899994
-5.7002726	10.9312000	100.6199951
-5.7002726	10.9800005	100.4499969
-5.7002726	11.0288000	101.9199982
-5.7002726	11.0776005	103.8699951
-5.7002726	11.1264000	105.2799988
-5.7002726	11.1752005	102.0899963
-5.7002726	11.2240000	103.0499954
-5.7002726	11.2728004	101.8699951
-5.7002726	11.3216000	102.2600021
-5.7002726	11.3704004	104.0999985
-5.7002726	11.4191999	102.8699951
-5.7002726	11.4680004	104.5499954
-5.7002726	11.5167999	102.4199982
-5.7002726	11.5656004	102.9899979
-5.7002726	11.6143999	100.9000015
-5.7002726	11.6632004	100.1899948
-5.7002726	11.7119999	102.0100021
-5.7002726	11.7608004	104.5299988
-5.7002726	11.8095999	102.6800003
-5.7002726	11.8584003	101.7799988
-5.7002726	11.9071999	101.7099991
-5.7002726	11.9560003	103.7299957
-5.7002726	12.0047998	104.4300003
-5.7002726	12.0536003	106.0400009
-5.7002726	12.1023998	101.9799957
-5.7002726	12.1512003	102.3899994
-5.7002726	12.1999998	101.7500000
-5.7002726	12.2488003	99.9199982
-5.7002726	12.2975998	104.4499969
-5.7002726	12.3464003	101.7900009
-5.7002726	12.3951998	105.7299957
-5.7002726	12.4440002	105.7799988
-5.7002726	12.4927998	102.4099960
-5.7002726	12.5416002	97.2699966
-5.7002726	12.5904007	103.4700012

-5.7002726	12.6392002	106.6500015
-5.7002726	12.6880007	106.6199951
-5.7002726	12.7368002	104.6299973
-5.7002726	12.7856007	103.4700012
-5.7002726	12.8344002	103.6100006
-5.7002726	12.8832006	100.7099991
-5.7002726	12.9320002	101.8699951
-5.7002726	12.9808006	105.7600021
-5.7002726	13.0296001	106.3399963
-5.7002726	13.0784006	102.2099991
-5.7002726	13.1272001	100.9199982
-5.7002726	13.1760006	103.1299973
-5.7002726	13.2248001	100.6800003
-5.7002726	13.2736006	99.1299973
-5.7002726	13.3224001	99.6299973
-5.7002726	13.3712006	100.9399948
-5.7002726	13.4200001	101.3199997
-5.7002726	13.4688005	99.2099991
-5.7002726	13.5176001	102.7699966
-5.7002726	13.5664005	100.3799973
-5.7002726	13.6152000	101.5400009
-5.7002726	13.6640005	101.5400009
-5.7002726	13.7128000	100.2500000
-5.7002726	13.7616005	97.5999985
-5.7002726	13.8104000	99.5000000
-5.7002726	13.8592005	101.7399979
-5.7002726	13.9080000	103.8499985
-5.7002726	13.9568005	103.7699966
-5.7002726	14.0056000	102.6899948
-5.7002726	14.0544004	99.6100006
-5.7002726	14.1032000	101.9499969
-5.7002726	14.1520004	101.1100006
-5.7002726	14.2007999	99.6999969
-5.7002726	14.2496004	98.4199982
-5.7002726	14.2983999	99.8799973
-5.7002726	14.3472004	98.5100021
-5.7002726	14.3959999	101.6699982
-5.7002726	14.4448004	100.3399963
-5.7002726	14.4935999	102.4300003
-5.7002726	14.5424004	102.3799973
-5.7002726	14.5911999	101.6999969
-5.7002726	14.6400003	100.1699982
-5.7002726	14.6887999	102.4000015
-5.7002726	14.7376003	103.9700012
-5.7002726	14.7863998	106.5100021
-5.7002726	14.8352003	104.5299988
-5.7002726	14.8839998	103.1299973
-5.7002726	14.9328003	104.1800003
-5.7002726	14.9815998	104.6199951
-5.7002726	15.0304003	102.0199966
-5.6941366	0.3416000	106.4000015
-5.6941366	0.3904000	101.8799973
-5.6941366	0.4392000	106.5000000
-5.6941366	0.4880000	105.3499985
-5.6941366	0.5368000	105.0899963
-5.6941366	0.5856000	103.0999985
-5.6941366	0.6344000	103.2399979
-5.6941366	0.6832000	105.8399963

-5.6941366	0.7320000	107.4499969
-5.6941366	0.7808000	106.1399994
-5.6941366	0.8296000	108.4700012
-5.6941366	0.8784000	107.0199966
-5.6941366	0.9272000	106.6299973
-5.6941366	0.9760000	107.9499969
-5.6941366	1.0247999	106.0999985
-5.6941366	1.0735999	104.1399994
-5.6941366	1.1223999	104.5000000
-5.6941366	1.1711999	102.1699982
-5.6941366	1.2200000	106.2699966
-5.6941366	1.2688000	105.5699997
-5.6941366	1.3176000	105.4499969
-5.6941366	1.3664000	107.2399979
-5.6941366	1.4152000	109.7600021
-5.6941366	1.4640000	108.3699951
-5.6941366	1.5128000	108.4000015
-5.6941366	1.5616000	106.6399994
-5.6941366	1.6104000	105.7299957
-5.6941366	1.6592000	103.9799957
-5.6941366	1.7079999	102.3699951
-5.6941366	1.7567999	104.0000000
-5.6941366	1.8055999	105.9599991
-5.6941366	1.8543999	109.4000015
-5.6941366	1.9031999	111.1999969
-5.6941366	1.9519999	108.7299957
-5.6941366	2.0007999	106.3300018
-5.6941366	2.0495999	106.7799988
-5.6941366	2.0983999	107.0400009
-5.6941366	2.1471999	107.1199951
-5.6941366	2.1959999	106.2200012
-5.6941366	2.2447999	107.7200012
-5.6941366	2.2935998	109.1100006
-5.6941366	2.3423998	107.6899948
-5.6941366	2.3912001	105.5499954
-5.6941366	2.4400001	103.6399994
-5.6941366	2.4888000	106.2799988
-5.6941366	2.5376000	106.3300018
-5.6941366	2.5864000	106.3399963
-5.6941366	2.6352000	104.4499969
-5.6941366	2.6840000	106.9599991
-5.6941366	2.7328000	104.4499969
-5.6941366	2.7816000	101.3699951
-5.6941366	2.8304000	102.8499985
-5.6941366	2.8792000	102.7500000
-5.6941366	2.9280000	104.1199951
-5.6941366	2.9768000	106.1299973
-5.6941366	3.0256000	104.3199997
-5.6941366	3.0743999	105.5599976
-5.6941366	3.1231999	102.7399979
-5.6941366	3.1719999	103.8199997
-5.6941366	3.2207999	103.3399963
-5.6941366	3.2695999	105.0199966
-5.6941366	3.3183999	105.0000000
-5.6941366	3.3671999	102.5599976
-5.6941366	3.4159999	101.7099991
-5.6941366	3.4647999	103.6399994
-5.6941366	3.5135999	102.3699951

-5.6941366	3.5623999	101.4199982
-5.6941366	3.6111999	103.6699982
-5.6941366	3.6599998	103.8199997
-5.6941366	3.7087998	104.5999985
-5.6941366	3.7575998	103.5100021
-5.6941366	3.8063998	102.9000015
-5.6941366	3.8551998	104.4000015
-5.6941366	3.9039998	106.1199951
-5.6941366	3.9527998	105.0299988
-5.6941366	4.0016003	105.3199997
-5.6941366	4.0503998	106.5400009
-5.6941366	4.0991998	104.6699982
-5.6941366	4.1479998	102.6699982
-5.6941366	4.1967998	102.3499985
-5.6941366	4.2455997	102.9199982
-5.6941366	4.2943997	107.2500000
-5.6941366	4.3431997	109.8799973
-5.6941366	4.3919997	108.2799988
-5.6941366	4.4407997	104.9700012
-5.6941366	4.4895997	104.8899994
-5.6941366	4.5383997	108.1199951
-5.6941366	4.5871997	110.3199997
-5.6941366	4.6359997	113.3499985
-5.6941366	4.6847997	115.5000000
-5.6941366	4.7336001	114.5599976
-5.6941366	4.7824001	109.3799973
-5.6941366	4.8312001	106.9499969
-5.6941366	4.8800001	107.8300018
-5.6941366	4.9288001	104.5999985
-5.6941366	4.9776001	100.5400009
-5.6941366	5.0264001	103.6299973
-5.6941366	5.0752001	103.4700012
-5.6941366	5.1240001	102.5899963
-5.6941366	5.1728001	104.6500015
-5.6941366	5.2216001	106.2999954
-5.6941366	5.2704000	104.2799988
-5.6941366	5.3192000	104.4000015
-5.6941366	5.3680000	105.9599991
-5.6941366	5.4168000	105.6100006
-5.6941366	5.4656000	102.7699966
-5.6941366	5.5144000	102.7099991
-5.6941366	5.5632000	102.4000015
-5.6941366	5.6120000	103.8899994
-5.6941366	5.6608000	104.8399963
-5.6941366	5.7096000	106.3600006
-5.6941366	5.7584000	104.9199982
-5.6941366	5.8072000	101.8399963
-5.6941366	5.8559999	100.5999985
-5.6941366	5.9047999	102.2299957
-5.6941366	5.9535999	103.3199997
-5.6941366	6.0023999	103.9000015
-5.6941366	6.0511999	105.4199982
-5.6941366	6.0999999	104.9399948
-5.6941366	6.1487999	97.2399979
-5.6941366	6.1975999	105.2299957
-5.6941366	6.2463999	105.9300003
-5.6941366	6.2951999	106.0499954
-5.6941366	6.3439999	103.0199966

-5.6941366	6.3927999	104.6899948
-5.6941366	6.4415998	107.0499954
-5.6941366	6.4903998	108.2200012
-5.6941366	6.5391998	107.1399994
-5.6941366	6.5879998	104.6599960
-5.6941366	6.6367998	104.1199951
-5.6941366	6.6855998	104.0400009
-5.6941366	6.7343998	106.3099976
-5.6941366	6.7831998	104.5899963
-5.6941366	6.8319998	102.7799988
-5.6941366	6.8807998	102.8300018
-5.6941366	6.9295998	102.5100021
-5.6941366	6.9783998	105.2600021
-5.6941366	7.0271997	106.5499954
-5.6941366	7.0759997	103.7500000
-5.6941366	7.1247997	103.2200012
-5.6941366	7.1735997	100.8099976
-5.6941366	7.2223997	105.8300018
-5.6941366	7.2711997	107.3099976
-5.6941366	7.3199997	103.9000015
-5.6941366	7.3687997	104.7799988
-5.6941366	7.4175997	107.6699982
-5.6941366	7.4663997	105.6699982
-5.6941366	7.5151997	103.3699951
-5.6941366	7.5639997	100.0199966
-5.6941366	7.6127996	99.7500000
-5.6941366	7.6615996	100.8399963
-5.6941366	7.7103996	102.0199966
-5.6941366	7.7591996	102.3099976
-5.6941366	7.8079996	103.3099976
-5.6941366	7.8567996	102.5999985
-5.6941366	7.9056001	100.5499954
-5.6941366	7.9544001	103.9399948
-5.6941366	8.0032005	105.5499954
-5.6941366	8.0520000	106.4799957
-5.6941366	8.1008005	105.4399948
-5.6941366	8.1496000	103.2200012
-5.6941366	8.1984005	103.7399979
-5.6941366	8.2472000	98.0699997
-5.6941366	8.2960005	102.2900009
-5.6941366	8.3448000	103.5699997
-5.6941366	8.3936005	100.6500015
-5.6941366	8.4424000	99.0899963
-5.6941366	8.4912004	100.0699997
-5.6941366	8.5400000	101.8300018
-5.6941366	8.5888004	103.8099976
-5.6941366	8.6375999	101.9499969
-5.6941366	8.6864004	105.0000000
-5.6941366	8.7351999	106.9700012
-5.6941366	8.7840004	104.1999969
-5.6941366	8.8327999	103.7699966
-5.6941366	8.8816004	101.5800018
-5.6941366	8.9303999	101.1699982
-5.6941366	8.9792004	102.5599976
-5.6941366	9.0279999	103.7799988
-5.6941366	9.0768003	102.7399979
-5.6941366	9.1255999	99.6100006
-5.6941366	9.1744003	103.4499969

-5.6941366	9.2231998	102.8600006
-5.6941366	9.2720003	102.7500000
-5.6941366	9.3207998	99.0599976
-5.6941366	9.3696003	99.9399948
-5.6941366	9.4184008	105.5999985
-5.6941366	9.4672003	106.4700012
-5.6941366	9.5160007	104.9099960
-5.6941366	9.5648003	100.4499969
-5.6941366	9.6136007	105.1299973
-5.6941366	9.6624002	104.5999985
-5.6941366	9.7112007	105.3300018
-5.6941366	9.7600002	102.0699997
-5.6941366	9.8088007	103.0100021
-5.6941366	9.8576002	101.7999954
-5.6941366	9.9064007	102.8699951
-5.6941366	9.9552002	100.1999969
-5.6941366	10.0040007	99.8199997
-5.6941366	10.0528002	104.1500015
-5.6941366	10.1016006	103.5699997
-5.6941366	10.1504002	100.4399948
-5.6941366	10.1992006	102.0999985
-5.6941366	10.2480001	103.5800018
-5.6941366	10.2968006	104.6999969
-5.6941366	10.3456001	104.3199997
-5.6941366	10.3944006	105.0199966
-5.6941366	10.4432001	104.9099960
-5.6941366	10.4920006	104.0800018
-5.6941366	10.5408001	101.7200012
-5.6941366	10.5896006	102.2999954
-5.6941366	10.6384001	100.5299988
-5.6941366	10.6872005	100.9000015
-5.6941366	10.7360001	102.4300003
-5.6941366	10.7848005	103.6199951
-5.6941366	10.8336000	104.6399994
-5.6941366	10.8824005	103.1699982
-5.6941366	10.9312000	101.2999954
-5.6941366	10.9800005	101.2200012
-5.6941366	11.0288000	102.6299973
-5.6941366	11.0776005	104.0299988
-5.6941366	11.1264000	105.4499969
-5.6941366	11.1752005	102.0999985
-5.6941366	11.2240000	102.9499969
-5.6941366	11.2728004	102.3699951
-5.6941366	11.3216000	102.9300003
-5.6941366	11.3704004	104.7099991
-5.6941366	11.4191999	101.9000015
-5.6941366	11.4680004	104.0100021
-5.6941366	11.5167999	101.8300018
-5.6941366	11.5656004	102.2299957
-5.6941366	11.6143999	100.9199982
-5.6941366	11.6632004	99.8099976
-5.6941366	11.7119999	100.9300003
-5.6941366	11.7608004	103.2299957
-5.6941366	11.8095999	102.1100006
-5.6941366	11.8584003	101.9499969
-5.6941366	11.9071999	102.7200012
-5.6941366	11.9560003	104.4399948
-5.6941366	12.0047998	104.2099991

-5.6941366	12.0536003	106.0400009
-5.6941366	12.1023998	102.7500000
-5.6941366	12.1512003	102.0599976
-5.6941366	12.1999998	101.3199997
-5.6941366	12.2488003	100.3199997
-5.6941366	12.2975998	104.6199951
-5.6941366	12.3464003	101.5599976
-5.6941366	12.3951998	105.7099991
-5.6941366	12.4440002	106.0599976
-5.6941366	12.4927998	103.0199966
-5.6941366	12.5416002	95.8699951
-5.6941366	12.5904007	103.4799957
-5.6941366	12.6392002	106.8300018
-5.6941366	12.6880007	107.1800003
-5.6941366	12.7368002	104.6999969
-5.6941366	12.7856007	102.7699966
-5.6941366	12.8344002	102.6800003
-5.6941366	12.8832006	101.9300003
-5.6941366	12.9320002	102.0000000
-5.6941366	12.9808006	105.1299973
-5.6941366	13.0296001	106.2299957
-5.6941366	13.0784006	102.6500015
-5.6941366	13.1272001	100.9700012
-5.6941366	13.1760006	103.3399963
-5.6941366	13.2248001	100.5899963
-5.6941366	13.2736006	99.2799988
-5.6941366	13.3224001	99.9399948
-5.6941366	13.3712006	101.1599960
-5.6941366	13.4200001	102.3499985
-5.6941366	13.4688005	97.7699966
-5.6941366	13.5176001	103.3099976
-5.6941366	13.5664005	100.9499969
-5.6941366	13.6152000	102.1500015
-5.6941366	13.6640005	101.5699997
-5.6941366	13.7128000	100.3300018
-5.6941366	13.7616005	99.1899948
-5.6941366	13.8104000	99.6599960
-5.6941366	13.8592005	101.9799957
-5.6941366	13.9080000	104.9799957
-5.6941366	13.9568005	104.4599991
-5.6941366	14.0056000	102.0499954
-5.6941366	14.0544004	100.5899963
-5.6941366	14.1032000	102.3499985
-5.6941366	14.1520004	101.6899948
-5.6941366	14.2007999	100.1199951
-5.6941366	14.2496004	97.5000000
-5.6941366	14.2983999	99.1699982
-5.6941366	14.3472004	99.1399994
-5.6941366	14.3959999	102.5400009
-5.6941366	14.4448004	101.5100021
-5.6941366	14.4935999	103.2799988
-5.6941366	14.5424004	103.6599960
-5.6941366	14.5911999	102.1299973
-5.6941366	14.6400003	100.0299988
-5.6941366	14.6887999	102.1500015
-5.6941366	14.7376003	104.2600021
-5.6941366	14.7863998	106.2200012
-5.6941366	14.8352003	103.7500000

-5.6941366	14.8839998	102.9499969
-5.6941366	14.9328003	104.5699997
-5.6941366	14.9815998	104.8300018
-5.6941366	15.0304003	101.4399948
-5.6880007	0.3416000	105.9300003
-5.6880007	0.3904000	99.5299988
-5.6880007	0.4392000	106.2999954
-5.6880007	0.4880000	105.4499969
-5.6880007	0.5368000	104.8499985
-5.6880007	0.5856000	103.9399948
-5.6880007	0.6344000	103.8699951
-5.6880007	0.6832000	104.9399948
-5.6880007	0.7320000	107.2200012
-5.6880007	0.7808000	107.1599960
-5.6880007	0.8296000	109.0899963
-5.6880007	0.8784000	108.0400009
-5.6880007	0.9272000	107.2999954
-5.6880007	0.9760000	107.8199997
-5.6880007	1.0247999	105.5100021
-5.6880007	1.0735999	103.7799988
-5.6880007	1.1223999	104.6699982
-5.6880007	1.1711999	103.1599960
-5.6880007	1.2200000	105.5299988
-5.6880007	1.2688000	104.9599991
-5.6880007	1.3176000	105.1899948
-5.6880007	1.3664000	106.7600021
-5.6880007	1.4152000	109.0599976
-5.6880007	1.4640000	108.4799957
-5.6880007	1.5128000	108.3899994
-5.6880007	1.5616000	106.8199997
-5.6880007	1.6104000	107.1100006
-5.6880007	1.6592000	105.6399994
-5.6880007	1.7079999	103.3899994
-5.6880007	1.7567999	104.2600021
-5.6880007	1.8055999	105.8699951
-5.6880007	1.8543999	109.3499985
-5.6880007	1.9031999	110.8199997
-5.6880007	1.9519999	108.3699951
-5.6880007	2.0007999	105.9300003
-5.6880007	2.0495999	105.6800003
-5.6880007	2.0983999	106.1299973
-5.6880007	2.1471999	105.8399963
-5.6880007	2.1959999	105.7600021
-5.6880007	2.2447999	106.5699997
-5.6880007	2.2935998	107.8499985
-5.6880007	2.3423998	107.2600021
-5.6880007	2.3912001	105.2200012
-5.6880007	2.4400001	103.6999969
-5.6880007	2.4888000	106.2699966
-5.6880007	2.5376000	106.7699966
-5.6880007	2.5864000	105.7299957
-5.6880007	2.6352000	104.6100006
-5.6880007	2.6840000	107.1399994
-5.6880007	2.7328000	104.2600021
-5.6880007	2.7816000	101.0999985
-5.6880007	2.8304000	102.9099960
-5.6880007	2.8792000	102.4899979
-5.6880007	2.9280000	103.5199966

-5.6880007	2.9768000	106.0599976
-5.6880007	3.0256000	103.8499985
-5.6880007	3.0743999	105.7999954
-5.6880007	3.1231999	103.6500015
-5.6880007	3.1719999	104.6500015
-5.6880007	3.2207999	103.7799988
-5.6880007	3.2695999	104.8799973
-5.6880007	3.3183999	104.3600006
-5.6880007	3.3671999	102.0599976
-5.6880007	3.4159999	100.7099991
-5.6880007	3.4647999	104.1500015
-5.6880007	3.5135999	103.0999985
-5.6880007	3.5623999	101.7399979
-5.6880007	3.6111999	103.9000015
-5.6880007	3.6599998	105.3600006
-5.6880007	3.7087998	105.0199966
-5.6880007	3.7575998	103.0599976
-5.6880007	3.8063998	102.0299988
-5.6880007	3.8551998	104.0599976
-5.6880007	3.9039998	106.5599976
-5.6880007	3.9527998	105.8099976
-5.6880007	4.0016003	104.8300018
-5.6880007	4.0503998	105.3699951
-5.6880007	4.0991998	103.4099960
-5.6880007	4.1479998	102.6500015
-5.6880007	4.1967998	102.1199951
-5.6880007	4.2455997	103.7900009
-5.6880007	4.2943997	108.1199951
-5.6880007	4.3431997	110.3899994
-5.6880007	4.3919997	107.4099960
-5.6880007	4.4407997	104.4599991
-5.6880007	4.4895997	105.2699966
-5.6880007	4.5383997	108.2299957
-5.6880007	4.5871997	109.9599991
-5.6880007	4.6359997	112.8099976
-5.6880007	4.6847997	115.2900009
-5.6880007	4.7336001	114.6500015
-5.6880007	4.7824001	109.7799988
-5.6880007	4.8312001	107.3399963
-5.6880007	4.8800001	108.2399979
-5.6880007	4.9288001	106.4099960
-5.6880007	4.9776001	101.2200012
-5.6880007	5.0264001	102.8600006
-5.6880007	5.0752001	103.1699982
-5.6880007	5.1240001	103.6500015
-5.6880007	5.1728001	105.8399963
-5.6880007	5.2216001	106.7799988
-5.6880007	5.2704000	104.3799973
-5.6880007	5.3192000	103.5100021
-5.6880007	5.3680000	106.1299973
-5.6880007	5.4168000	105.3199997
-5.6880007	5.4656000	103.4499969
-5.6880007	5.5144000	103.5100021
-5.6880007	5.5632000	102.7600021
-5.6880007	5.6120000	104.7099991
-5.6880007	5.6608000	105.2999954
-5.6880007	5.7096000	106.4799957
-5.6880007	5.7584000	105.2099991

-5.6880007	5.8072000	102.9199982
-5.6880007	5.8559999	101.9700012
-5.6880007	5.9047999	101.3300018
-5.6880007	5.9535999	103.3499985
-5.6880007	6.0023999	105.0899963
-5.6880007	6.0511999	106.0800018
-5.6880007	6.0999999	105.9399948
-5.6880007	6.1487999	98.6800003
-5.6880007	6.1975999	106.1500015
-5.6880007	6.2463999	105.9499969
-5.6880007	6.2951999	105.1100006
-5.6880007	6.3439999	102.0199966
-5.6880007	6.3927999	104.5699997
-5.6880007	6.4415998	106.6699982
-5.6880007	6.4903998	108.0499954
-5.6880007	6.5391998	107.3600006
-5.6880007	6.5879998	104.7399979
-5.6880007	6.6367998	103.9599991
-5.6880007	6.6855998	104.3300018
-5.6880007	6.7343998	106.3099976
-5.6880007	6.7831998	104.4099960
-5.6880007	6.8319998	102.5699997
-5.6880007	6.8807998	102.7600021
-5.6880007	6.9295998	102.8799973
-5.6880007	6.9783998	106.2600021
-5.6880007	7.0271997	106.7399979
-5.6880007	7.0759997	103.6399994
-5.6880007	7.1247997	103.9700012
-5.6880007	7.1735997	101.8099976
-5.6880007	7.2223997	106.0199966
-5.6880007	7.2711997	107.0999985
-5.6880007	7.3199997	104.4099960
-5.6880007	7.3687997	104.3799973
-5.6880007	7.4175997	107.2299957
-5.6880007	7.4663997	105.8799973
-5.6880007	7.5151997	104.5899963
-5.6880007	7.5639997	101.9899979
-5.6880007	7.6127996	101.3300018
-5.6880007	7.6615996	102.2299957
-5.6880007	7.7103996	102.2999954
-5.6880007	7.7591996	102.0599976
-5.6880007	7.8079996	104.1299973
-5.6880007	7.8567996	103.5999985
-5.6880007	7.9056001	100.6699982
-5.6880007	7.9544001	103.5299988
-5.6880007	8.0032005	105.3199997
-5.6880007	8.0520000	105.9599991
-5.6880007	8.1008005	104.9399948
-5.6880007	8.1496000	103.4899979
-5.6880007	8.1984005	103.7099991
-5.6880007	8.2472000	98.2399979
-5.6880007	8.2960005	101.5299988
-5.6880007	8.3448000	102.7999954
-5.6880007	8.3936005	100.9300003
-5.6880007	8.4424000	100.1199951
-5.6880007	8.4912004	100.4300003
-5.6880007	8.5400000	102.4199982
-5.6880007	8.5888004	103.8799973

-5.6880007	8.6375999	101.6699982
-5.6880007	8.6864004	104.8600006
-5.6880007	8.7351999	106.7900009
-5.6880007	8.7840004	104.2500000
-5.6880007	8.8327999	104.0199966
-5.6880007	8.8816004	102.9899979
-5.6880007	8.9303999	102.3799973
-5.6880007	8.9792004	103.4199982
-5.6880007	9.0279999	103.9899979
-5.6880007	9.0768003	101.9700012
-5.6880007	9.1255999	99.8300018
-5.6880007	9.1744003	103.5299988
-5.6880007	9.2231998	102.8699951
-5.6880007	9.2720003	102.4199982
-5.6880007	9.3207998	96.8799973
-5.6880007	9.3696003	100.0699997
-5.6880007	9.4184008	106.1100006
-5.6880007	9.4672003	106.9000015
-5.6880007	9.5160007	105.4499969
-5.6880007	9.5648003	101.9799957
-5.6880007	9.6136007	104.9499969
-5.6880007	9.6624002	103.6599960
-5.6880007	9.7112007	104.8699951
-5.6880007	9.7600002	101.5999985
-5.6880007	9.8088007	103.4099960
-5.6880007	9.8576002	102.7799988
-5.6880007	9.9064007	103.3399963
-5.6880007	9.9552002	101.5299988
-5.6880007	10.0040007	101.5499954
-5.6880007	10.0528002	104.6699982
-5.6880007	10.1016006	104.5299988
-5.6880007	10.1504002	101.4799957
-5.6880007	10.1992006	103.0299988
-5.6880007	10.2480001	104.0999985
-5.6880007	10.2968006	104.6399994
-5.6880007	10.3456001	103.1800003
-5.6880007	10.3944006	103.3799973
-5.6880007	10.4432001	103.7799988
-5.6880007	10.4920006	103.5699997
-5.6880007	10.5408001	102.0599976
-5.6880007	10.5896006	101.8899994
-5.6880007	10.6384001	100.2999954
-5.6880007	10.6872005	100.5400009
-5.6880007	10.7360001	101.0000000
-5.6880007	10.7848005	102.6999969
-5.6880007	10.8336000	104.0699997
-5.6880007	10.8824005	103.6500015
-5.6880007	10.9312000	102.3600006
-5.6880007	10.9800005	101.6399994
-5.6880007	11.0288000	101.8499985
-5.6880007	11.0776005	103.7600021
-5.6880007	11.1264000	105.3899994
-5.6880007	11.1752005	101.7399979
-5.6880007	11.2240000	102.2999954
-5.6880007	11.2728004	102.7900009
-5.6880007	11.3216000	103.4799957
-5.6880007	11.3704004	104.6800003
-5.6880007	11.4191999	100.4399948

-5.6880007	11.4680004	103.5999985
-5.6880007	11.5167999	102.1599960
-5.6880007	11.5656004	102.0499954
-5.6880007	11.6143999	101.6699982
-5.6880007	11.6632004	100.5599976
-5.6880007	11.7119999	100.9799957
-5.6880007	11.7608004	102.1599960
-5.6880007	11.8095999	101.8499985
-5.6880007	11.8584003	103.1699982
-5.6880007	11.9071999	104.6100006
-5.6880007	11.9560003	104.7600021
-5.6880007	12.0047998	103.6199951
-5.6880007	12.0536003	105.7900009
-5.6880007	12.1023998	102.9000015
-5.6880007	12.1512003	101.0599976
-5.6880007	12.1999998	100.2600021
-5.6880007	12.2488003	100.6599960
-5.6880007	12.2975998	104.3699951
-5.6880007	12.3464003	100.9300003
-5.6880007	12.3951998	105.3699951
-5.6880007	12.4440002	105.9000015
-5.6880007	12.4927998	103.3899994
-5.6880007	12.5416002	94.7799988
-5.6880007	12.5904007	103.8600006
-5.6880007	12.6392002	106.9000015
-5.6880007	12.6880007	107.0800018
-5.6880007	12.7368002	104.8799973
-5.6880007	12.7856007	102.8300018
-5.6880007	12.8344002	101.8300018
-5.6880007	12.8832006	101.7900009
-5.6880007	12.9320002	102.0999985
-5.6880007	12.9808006	105.4799957
-5.6880007	13.0296001	106.9899979
-5.6880007	13.0784006	103.9000015
-5.6880007	13.1272001	101.3499985
-5.6880007	13.1760006	103.1599960
-5.6880007	13.2248001	99.9199982
-5.6880007	13.2736006	98.4300003
-5.6880007	13.3224001	100.4300003
-5.6880007	13.3712006	101.8699951
-5.6880007	13.4200001	102.9099960
-5.6880007	13.4688005	96.2099991
-5.6880007	13.5176001	103.4300003
-5.6880007	13.5664005	101.1199951
-5.6880007	13.6152000	103.2900009
-5.6880007	13.6640005	102.7900009
-5.6880007	13.7128000	101.5100021
-5.6880007	13.7616005	100.9499969
-5.6880007	13.8104000	100.6100006
-5.6880007	13.8592005	101.7099991
-5.6880007	13.9080000	105.7799988
-5.6880007	13.9568005	105.4000015
-5.6880007	14.0056000	101.2699966
-5.6880007	14.0544004	101.8499985
-5.6880007	14.1032000	102.7600021
-5.6880007	14.1520004	100.5499954
-5.6880007	14.2007999	100.8799973
-5.6880007	14.2496004	97.7399979

-5.6880007	14.2983999	99.7399979
-5.6880007	14.3472004	100.9700012
-5.6880007	14.3959999	103.9399948
-5.6880007	14.4448004	102.5599976
-5.6880007	14.4935999	104.0199966
-5.6880007	14.5424004	104.1899948
-5.6880007	14.5911999	102.2299957
-5.6880007	14.6400003	100.1599960
-5.6880007	14.6887999	102.3799973
-5.6880007	14.7376003	105.2699966
-5.6880007	14.7863998	106.2099991
-5.6880007	14.8352003	103.0800018
-5.6880007	14.8839998	102.5499954
-5.6880007	14.9328003	104.3499985
-5.6880007	14.9815998	104.8799973
-5.6880007	15.0304003	100.9899979
-5.6818647	0.3416000	105.5699997
-5.6818647	0.3904000	99.7200012
-5.6818647	0.4392000	106.5199966
-5.6818647	0.4880000	105.8399963
-5.6818647	0.5368000	105.0299988
-5.6818647	0.5856000	104.5599976
-5.6818647	0.6344000	104.2600021
-5.6818647	0.6832000	103.9099960
-5.6818647	0.7320000	106.8399963
-5.6818647	0.7808000	107.9099960
-5.6818647	0.8296000	109.3499985
-5.6818647	0.8784000	108.9499969
-5.6818647	0.9272000	108.2699966
-5.6818647	0.9760000	107.8600006
-5.6818647	1.0247999	105.4399948
-5.6818647	1.0735999	103.9899979
-5.6818647	1.1223999	104.7699966
-5.6818647	1.1711999	103.2999954
-5.6818647	1.2200000	104.7299957
-5.6818647	1.2688000	104.9700012
-5.6818647	1.3176000	104.8899994
-5.6818647	1.3664000	105.7399979
-5.6818647	1.4152000	108.3499985
-5.6818647	1.4640000	108.7699966
-5.6818647	1.5128000	108.5999985
-5.6818647	1.5616000	107.5899963
-5.6818647	1.6104000	108.2500000
-5.6818647	1.6592000	106.5199966
-5.6818647	1.7079999	104.1199951
-5.6818647	1.7567999	105.1800003
-5.6818647	1.8055999	105.8899994
-5.6818647	1.8543999	108.6100006
-5.6818647	1.9031999	109.8899994
-5.6818647	1.9519999	108.0299988
-5.6818647	2.0007999	105.3199997
-5.6818647	2.0495999	103.8499985
-5.6818647	2.0983999	104.5400009
-5.6818647	2.1471999	104.5100021
-5.6818647	2.1959999	105.0299988
-5.6818647	2.2447999	105.0499954
-5.6818647	2.2935998	106.0999985
-5.6818647	2.3423998	106.3099976

-5.6818647	2.3912001	104.7600021
-5.6818647	2.4400001	103.9199982
-5.6818647	2.4888000	106.4099960
-5.6818647	2.5376000	107.0899963
-5.6818647	2.5864000	104.9199982
-5.6818647	2.6352000	104.6399994
-5.6818647	2.6840000	107.6999969
-5.6818647	2.7328000	105.1399994
-5.6818647	2.7816000	101.5400009
-5.6818647	2.8304000	103.1599960
-5.6818647	2.8792000	102.3099976
-5.6818647	2.9280000	103.5899963
-5.6818647	2.9768000	106.5599976
-5.6818647	3.0256000	104.6599960
-5.6818647	3.0743999	105.7699966
-5.6818647	3.1231999	103.8799973
-5.6818647	3.1719999	104.9099960
-5.6818647	3.2207999	104.3600006
-5.6818647	3.2695999	104.8199997
-5.6818647	3.3183999	104.2099991
-5.6818647	3.3671999	102.4799957
-5.6818647	3.4159999	100.6699982
-5.6818647	3.4647999	105.1399994
-5.6818647	3.5135999	104.0800018
-5.6818647	3.5623999	101.8099976
-5.6818647	3.6111999	103.5299988
-5.6818647	3.6599998	105.7500000
-5.6818647	3.7087998	104.2399979
-5.6818647	3.7575998	102.0100021
-5.6818647	3.8063998	101.2299957
-5.6818647	3.8551998	104.3199997
-5.6818647	3.9039998	107.0699997
-5.6818647	3.9527998	106.3099976
-5.6818647	4.0016003	104.6599960
-5.6818647	4.0503998	104.5299988
-5.6818647	4.0991998	102.0800018
-5.6818647	4.1479998	102.4599991
-5.6818647	4.1967998	101.7600021
-5.6818647	4.2455997	104.2500000
-5.6818647	4.2943997	108.2399979
-5.6818647	4.3431997	110.3600006
-5.6818647	4.3919997	107.2299957
-5.6818647	4.4407997	103.8899994
-5.6818647	4.4895997	105.5599976
-5.6818647	4.5383997	107.8799973
-5.6818647	4.5871997	109.5699997
-5.6818647	4.6359997	111.9399948
-5.6818647	4.6847997	114.5999985
-5.6818647	4.7336001	114.2600021
-5.6818647	4.7824001	109.5699997
-5.6818647	4.8312001	107.7500000
-5.6818647	4.8800001	108.9899979
-5.6818647	4.9288001	107.7600021
-5.6818647	4.9776001	101.5299988
-5.6818647	5.0264001	102.3600006
-5.6818647	5.0752001	102.9399948
-5.6818647	5.1240001	103.5899963
-5.6818647	5.1728001	106.1999969

-5.6818647	5.2216001	106.4399948
-5.6818647	5.2704000	104.5899963
-5.6818647	5.3192000	102.6100006
-5.6818647	5.3680000	105.7600021
-5.6818647	5.4168000	104.5199966
-5.6818647	5.4656000	103.3399963
-5.6818647	5.5144000	103.5299988
-5.6818647	5.5632000	102.8300018
-5.6818647	5.6120000	105.0100021
-5.6818647	5.6608000	105.6299973
-5.6818647	5.7096000	107.1500015
-5.6818647	5.7584000	105.7500000
-5.6818647	5.8072000	104.3300018
-5.6818647	5.8559999	103.4700012
-5.6818647	5.9047999	100.8799973
-5.6818647	5.9535999	103.5299988
-5.6818647	6.0023999	105.9099960
-5.6818647	6.0511999	106.5499954
-5.6818647	6.0999999	106.3899994
-5.6818647	6.1487999	100.9300003
-5.6818647	6.1975999	106.5800018
-5.6818647	6.2463999	105.5000000
-5.6818647	6.2951999	104.2999954
-5.6818647	6.3439999	102.1500015
-5.6818647	6.3927999	104.3300018
-5.6818647	6.4415998	106.5800018
-5.6818647	6.4903998	108.0400009
-5.6818647	6.5391998	107.0899963
-5.6818647	6.5879998	104.4599991
-5.6818647	6.6367998	104.0999985
-5.6818647	6.6855998	105.0999985
-5.6818647	6.7343998	105.8499985
-5.6818647	6.7831998	104.0499954
-5.6818647	6.8319998	102.2600021
-5.6818647	6.8807998	102.4199982
-5.6818647	6.9295998	102.9399948
-5.6818647	6.9783998	106.7999954
-5.6818647	7.0271997	106.4300003
-5.6818647	7.0759997	104.4700012
-5.6818647	7.1247997	104.6500015
-5.6818647	7.1735997	102.7099991
-5.6818647	7.2223997	106.6399994
-5.6818647	7.2711997	106.9099960
-5.6818647	7.3199997	104.5100021
-5.6818647	7.3687997	104.2399979
-5.6818647	7.4175997	106.5100021
-5.6818647	7.4663997	105.2900009
-5.6818647	7.5151997	104.8499985
-5.6818647	7.5639997	103.0899963
-5.6818647	7.6127996	102.7299957
-5.6818647	7.6615996	103.2999954
-5.6818647	7.7103996	102.1299973
-5.6818647	7.7591996	101.9300003
-5.6818647	7.8079996	104.6100006
-5.6818647	7.8567996	104.6999969
-5.6818647	7.9056001	101.3300018
-5.6818647	7.9544001	103.1800003
-5.6818647	8.0032005	104.7999954

-5.6818647	8.0520000	105.3799973
-5.6818647	8.1008005	104.8099976
-5.6818647	8.1496000	103.9399948
-5.6818647	8.1984005	103.4799957
-5.6818647	8.2472000	98.0899963
-5.6818647	8.2960005	101.1299973
-5.6818647	8.3448000	102.0199966
-5.6818647	8.3936005	101.6800003
-5.6818647	8.4424000	101.5599976
-5.6818647	8.4912004	101.6299973
-5.6818647	8.5400000	102.4000015
-5.6818647	8.5888004	103.4899979
-5.6818647	8.6375999	101.8600006
-5.6818647	8.6864004	104.1500015
-5.6818647	8.7351999	105.9499969
-5.6818647	8.7840004	104.2099991
-5.6818647	8.8327999	104.7799988
-5.6818647	8.8816004	104.0499954
-5.6818647	8.9303999	103.5100021
-5.6818647	8.9792004	103.8600006
-5.6818647	9.0279999	104.2900009
-5.6818647	9.0768003	101.3899994
-5.6818647	9.1255999	100.2399979
-5.6818647	9.1744003	103.5299988
-5.6818647	9.2231998	103.0599976
-5.6818647	9.2720003	101.9499969
-5.6818647	9.3207998	95.0599976
-5.6818647	9.3696003	101.1199951
-5.6818647	9.4184008	107.0400009
-5.6818647	9.4672003	107.5800018
-5.6818647	9.5160007	106.0499954
-5.6818647	9.5648003	103.1199951
-5.6818647	9.6136007	104.8799973
-5.6818647	9.6624002	103.5699997
-5.6818647	9.7112007	104.2799988
-5.6818647	9.7600002	100.6500015
-5.6818647	9.8088007	103.0599976
-5.6818647	9.8576002	103.6199951
-5.6818647	9.9064007	103.9000015
-5.6818647	9.9552002	102.1500015
-5.6818647	10.0040007	102.4300003
-5.6818647	10.0528002	104.5599976
-5.6818647	10.1016006	104.7999954
-5.6818647	10.1504002	102.1599960
-5.6818647	10.1992006	103.7399979
-5.6818647	10.2480001	104.2999954
-5.6818647	10.2968006	103.8699951
-5.6818647	10.3456001	101.2999954
-5.6818647	10.3944006	101.2299957
-5.6818647	10.4432001	102.2399979
-5.6818647	10.4920006	103.0299988
-5.6818647	10.5408001	102.2200012
-5.6818647	10.5896006	101.5400009
-5.6818647	10.6384001	100.5299988
-5.6818647	10.6872005	100.9599991
-5.6818647	10.7360001	100.7200012
-5.6818647	10.7848005	102.2200012
-5.6818647	10.8336000	103.2999954

-5.6818647	10.8824005	103.8300018
-5.6818647	10.9312000	103.3399963
-5.6818647	10.9800005	102.5400009
-5.6818647	11.0288000	101.0100021
-5.6818647	11.0776005	103.0100021
-5.6818647	11.1264000	104.9899979
-5.6818647	11.1752005	101.3099976
-5.6818647	11.2240000	101.3099976
-5.6818647	11.2728004	102.7900009
-5.6818647	11.3216000	104.0999985
-5.6818647	11.3704004	104.9300003
-5.6818647	11.4191999	101.4099960
-5.6818647	11.4680004	104.0199966
-5.6818647	11.5167999	102.9499969
-5.6818647	11.5656004	101.9199982
-5.6818647	11.6143999	102.0000000
-5.6818647	11.6632004	101.3600006
-5.6818647	11.7119999	101.0499954
-5.6818647	11.7608004	101.1599960
-5.6818647	11.8095999	102.4700012
-5.6818647	11.8584003	104.4899979
-5.6818647	11.9071999	105.6399994
-5.6818647	11.9560003	104.8499985
-5.6818647	12.0047998	102.9000015
-5.6818647	12.0536003	105.1800003
-5.6818647	12.1023998	102.4399948
-5.6818647	12.1512003	99.5699997
-5.6818647	12.1999998	98.6399994
-5.6818647	12.2488003	100.6299973
-5.6818647	12.2975998	103.9099960
-5.6818647	12.3464003	100.3699951
-5.6818647	12.3951998	104.7200012
-5.6818647	12.4440002	105.4300003
-5.6818647	12.4927998	104.0100021
-5.6818647	12.5416002	94.7500000
-5.6818647	12.5904007	103.5899963
-5.6818647	12.6392002	106.3199997
-5.6818647	12.6880007	106.4799957
-5.6818647	12.7368002	104.8600006
-5.6818647	12.7856007	103.2299957
-5.6818647	12.8344002	102.2299957
-5.6818647	12.8832006	102.3799973
-5.6818647	12.9320002	102.7399979
-5.6818647	12.9808006	105.5100021
-5.6818647	13.0296001	106.9700012
-5.6818647	13.0784006	104.5299988
-5.6818647	13.1272001	101.6500015
-5.6818647	13.1760006	102.9399948
-5.6818647	13.2248001	100.0199966
-5.6818647	13.2736006	99.4099960
-5.6818647	13.3224001	101.0699997
-5.6818647	13.3712006	102.1899948
-5.6818647	13.4200001	103.1599960
-5.6818647	13.4688005	95.1800003
-5.6818647	13.5176001	103.6500015
-5.6818647	13.5664005	102.5699997
-5.6818647	13.6152000	104.4300003
-5.6818647	13.6640005	103.8099976

-5.6818647	13.7128000	102.2600021
-5.6818647	13.7616005	101.7999954
-5.6818647	13.8104000	101.8899994
-5.6818647	13.8592005	102.2299957
-5.6818647	13.9080000	106.6399994
-5.6818647	13.9568005	105.5599976
-5.6818647	14.0056000	100.1100006
-5.6818647	14.0544004	102.4399948
-5.6818647	14.1032000	103.2500000
-5.6818647	14.1520004	100.1800003
-5.6818647	14.2007999	102.2500000
-5.6818647	14.2496004	99.3099976
-5.6818647	14.2983999	101.3199997
-5.6818647	14.3472004	103.3399963
-5.6818647	14.3959999	105.0999985
-5.6818647	14.4448004	102.8399963
-5.6818647	14.4935999	103.8099976
-5.6818647	14.5424004	104.4599991
-5.6818647	14.5911999	102.3799973
-5.6818647	14.6400003	100.5999985
-5.6818647	14.6887999	103.2299957
-5.6818647	14.7376003	105.9599991
-5.6818647	14.7863998	105.9499969
-5.6818647	14.8352003	103.0400009
-5.6818647	14.8839998	102.0699997
-5.6818647	14.9328003	103.6800003
-5.6818647	14.9815998	104.9399948
-5.6818647	15.0304003	102.0599976
-5.6757293	0.3416000	105.3099976
-5.6757293	0.3904000	100.2999954
-5.6757293	0.4392000	107.0100021
-5.6757293	0.4880000	106.1999969
-5.6757293	0.5368000	105.2200012
-5.6757293	0.5856000	105.0100021
-5.6757293	0.6344000	103.9499969
-5.6757293	0.6832000	102.7799988
-5.6757293	0.7320000	105.7999954
-5.6757293	0.7808000	107.7999954
-5.6757293	0.8296000	109.0299988
-5.6757293	0.8784000	109.0299988
-5.6757293	0.9272000	108.3899994
-5.6757293	0.9760000	107.4099960
-5.6757293	1.0247999	105.0599976
-5.6757293	1.0735999	103.2299957
-5.6757293	1.1223999	104.0800018
-5.6757293	1.1711999	103.0100021
-5.6757293	1.2200000	103.6399994
-5.6757293	1.2688000	104.6899948
-5.6757293	1.3176000	105.0000000
-5.6757293	1.3664000	105.2699966
-5.6757293	1.4152000	107.9899979
-5.6757293	1.4640000	109.6800003
-5.6757293	1.5128000	109.5199966
-5.6757293	1.5616000	108.2799988
-5.6757293	1.6104000	109.3300018
-5.6757293	1.6592000	107.7200012
-5.6757293	1.7079999	104.2900009
-5.6757293	1.7567999	105.3300018

-5.6757293	1.8055999	105.2299957
-5.6757293	1.8543999	107.3300018
-5.6757293	1.9031999	108.9300003
-5.6757293	1.9519999	107.5400009
-5.6757293	2.0007999	103.7299957
-5.6757293	2.0495999	101.7600021
-5.6757293	2.0983999	103.2999954
-5.6757293	2.1471999	103.6100006
-5.6757293	2.1959999	103.9300003
-5.6757293	2.2447999	103.6699982
-5.6757293	2.2935998	104.6500015
-5.6757293	2.3423998	105.0699997
-5.6757293	2.3912001	103.5199966
-5.6757293	2.4400001	103.6299973
-5.6757293	2.4888000	106.3600006
-5.6757293	2.5376000	106.5400009
-5.6757293	2.5864000	104.8199997
-5.6757293	2.6352000	104.8300018
-5.6757293	2.6840000	107.9499969
-5.6757293	2.7328000	105.8600006
-5.6757293	2.7816000	102.1800003
-5.6757293	2.8304000	103.4099960
-5.6757293	2.8792000	102.5000000
-5.6757293	2.9280000	103.6299973
-5.6757293	2.9768000	106.5199966
-5.6757293	3.0256000	105.2399979
-5.6757293	3.0743999	105.6899948
-5.6757293	3.1231999	104.0199966
-5.6757293	3.1719999	104.7799988
-5.6757293	3.2207999	104.2299957
-5.6757293	3.2695999	104.5699997
-5.6757293	3.3183999	104.0199966
-5.6757293	3.3671999	102.2799988
-5.6757293	3.4159999	100.9700012
-5.6757293	3.4647999	105.8300018
-5.6757293	3.5135999	104.5800018
-5.6757293	3.5623999	101.7099991
-5.6757293	3.6111999	103.0000000
-5.6757293	3.6599998	105.0800018
-5.6757293	3.7087998	103.4000015
-5.6757293	3.7575998	101.5199966
-5.6757293	3.8063998	101.4899979
-5.6757293	3.8551998	104.6899948
-5.6757293	3.9039998	107.0800018
-5.6757293	3.9527998	106.4899979
-5.6757293	4.0016003	104.2099991
-5.6757293	4.0503998	103.4599991
-5.6757293	4.0991998	101.2900009
-5.6757293	4.1479998	103.1800003
-5.6757293	4.1967998	102.4700012
-5.6757293	4.2455997	104.8399963
-5.6757293	4.2943997	107.9700012
-5.6757293	4.3431997	109.7999954
-5.6757293	4.3919997	106.9000015
-5.6757293	4.4407997	103.6399994
-5.6757293	4.4895997	106.1299973
-5.6757293	4.5383997	107.8300018
-5.6757293	4.5871997	109.6299973

-5.6757293	4.6359997	111.8799973
-5.6757293	4.6847997	114.5999985
-5.6757293	4.7336001	113.8399963
-5.6757293	4.7824001	109.5499954
-5.6757293	4.8312001	107.8699951
-5.6757293	4.8800001	109.1199951
-5.6757293	4.9288001	107.8899994
-5.6757293	4.9776001	101.8699951
-5.6757293	5.0264001	102.4000015
-5.6757293	5.0752001	103.3899994
-5.6757293	5.1240001	103.1800003
-5.6757293	5.1728001	106.2099991
-5.6757293	5.2216001	105.4899979
-5.6757293	5.2704000	103.6999969
-5.6757293	5.3192000	101.2999954
-5.6757293	5.3680000	105.2099991
-5.6757293	5.4168000	104.5400009
-5.6757293	5.4656000	103.2399979
-5.6757293	5.5144000	103.5100021
-5.6757293	5.5632000	103.6999969
-5.6757293	5.6120000	105.3399963
-5.6757293	5.6608000	105.5999985
-5.6757293	5.7096000	107.3399963
-5.6757293	5.7584000	105.5599976
-5.6757293	5.8072000	105.5599976
-5.6757293	5.8559999	105.1299973
-5.6757293	5.9047999	101.5499954
-5.6757293	5.9535999	104.3799973
-5.6757293	6.0023999	106.8099976
-5.6757293	6.0511999	107.0100021
-5.6757293	6.0999999	107.0599976
-5.6757293	6.1487999	103.0100021
-5.6757293	6.1975999	106.8399963
-5.6757293	6.2463999	105.4499969
-5.6757293	6.2951999	104.2600021
-5.6757293	6.3439999	103.1399994
-5.6757293	6.3927999	104.9300003
-5.6757293	6.4415998	106.2399979
-5.6757293	6.4903998	106.5800018
-5.6757293	6.5391998	106.0000000
-5.6757293	6.5879998	103.7799988
-5.6757293	6.6367998	104.0000000
-5.6757293	6.6855998	105.7799988
-5.6757293	6.7343998	105.2799988
-5.6757293	6.7831998	103.6500015
-5.6757293	6.8319998	102.4399948
-5.6757293	6.8807998	102.2099991
-5.6757293	6.9295998	102.1899948
-5.6757293	6.9783998	106.1899948
-5.6757293	7.0271997	105.1500015
-5.6757293	7.0759997	104.9000015
-5.6757293	7.1247997	104.8199997
-5.6757293	7.1735997	102.7999954
-5.6757293	7.2223997	107.0299988
-5.6757293	7.2711997	106.5299988
-5.6757293	7.3199997	103.3499985
-5.6757293	7.3687997	103.6199951
-5.6757293	7.4175997	105.7399979

-5.6757293	7.4663997	105.1100006
-5.6757293	7.5151997	104.9000015
-5.6757293	7.5639997	103.9799957
-5.6757293	7.6127996	103.7999954
-5.6757293	7.6615996	103.3099976
-5.6757293	7.7103996	101.7699966
-5.6757293	7.7591996	102.0999985
-5.6757293	7.8079996	105.3399963
-5.6757293	7.8567996	105.7299957
-5.6757293	7.9056001	102.5199966
-5.6757293	7.9544001	103.6299973
-5.6757293	8.0032005	104.3799973
-5.6757293	8.0520000	104.4399948
-5.6757293	8.1008005	104.3199997
-5.6757293	8.1496000	104.1100006
-5.6757293	8.1984005	102.9899979
-5.6757293	8.2472000	97.9899979
-5.6757293	8.2960005	101.1399994
-5.6757293	8.3448000	101.7299957
-5.6757293	8.3936005	102.5599976
-5.6757293	8.4424000	103.1899948
-5.6757293	8.4912004	103.2799988
-5.6757293	8.5400000	102.8699951
-5.6757293	8.5888004	103.8199997
-5.6757293	8.6375999	102.0899963
-5.6757293	8.6864004	103.1500015
-5.6757293	8.7351999	105.5199966
-5.6757293	8.7840004	104.1699982
-5.6757293	8.8327999	105.0299988
-5.6757293	8.8816004	104.6899948
-5.6757293	8.9303999	104.4599991
-5.6757293	8.9792004	103.2799988
-5.6757293	9.0279999	104.7399979
-5.6757293	9.0768003	101.9899979
-5.6757293	9.1255999	101.4899979
-5.6757293	9.1744003	103.2200012
-5.6757293	9.2231998	102.1299973
-5.6757293	9.2720003	100.8600006
-5.6757293	9.3207998	93.1299973
-5.6757293	9.3696003	101.8699951
-5.6757293	9.4184008	107.3600006
-5.6757293	9.4672003	107.6899948
-5.6757293	9.5160007	106.6399994
-5.6757293	9.5648003	104.0199966
-5.6757293	9.6136007	104.6999969
-5.6757293	9.6624002	103.6399994
-5.6757293	9.7112007	103.9300003
-5.6757293	9.7600002	101.3199997
-5.6757293	9.8088007	103.2099991
-5.6757293	9.8576002	104.4700012
-5.6757293	9.9064007	104.4499969
-5.6757293	9.9552002	103.0100021
-5.6757293	10.0040007	103.5400009
-5.6757293	10.0528002	104.3300018
-5.6757293	10.1016006	104.6599960
-5.6757293	10.1504002	103.4499969
-5.6757293	10.1992006	104.5699997
-5.6757293	10.2480001	104.4899979

-5.6757293	10.2968006	103.2600021
-5.6757293	10.3456001	100.5000000
-5.6757293	10.3944006	99.8300018
-5.6757293	10.4432001	101.1800003
-5.6757293	10.4920006	102.9000015
-5.6757293	10.5408001	102.4000015
-5.6757293	10.5896006	101.7200012
-5.6757293	10.6384001	101.3199997
-5.6757293	10.6872005	100.9700012
-5.6757293	10.7360001	100.3600006
-5.6757293	10.7848005	101.5000000
-5.6757293	10.8336000	103.1800003
-5.6757293	10.8824005	104.4199982
-5.6757293	10.9312000	104.7500000
-5.6757293	10.9800005	103.7500000
-5.6757293	11.0288000	99.7099991
-5.6757293	11.0776005	101.5000000
-5.6757293	11.1264000	104.2399979
-5.6757293	11.1752005	100.4899979
-5.6757293	11.2240000	100.2999954
-5.6757293	11.2728004	102.0999985
-5.6757293	11.3216000	104.2299957
-5.6757293	11.3704004	104.9300003
-5.6757293	11.4191999	102.6800003
-5.6757293	11.4680004	104.1100006
-5.6757293	11.5167999	103.2099991
-5.6757293	11.5656004	101.4799957
-5.6757293	11.6143999	102.2399979
-5.6757293	11.6632004	102.6500015
-5.6757293	11.7119999	102.1299973
-5.6757293	11.7608004	101.2999954
-5.6757293	11.8095999	103.6399994
-5.6757293	11.8584003	105.7600021
-5.6757293	11.9071999	106.2099991
-5.6757293	11.9560003	104.3499985
-5.6757293	12.0047998	101.7699966
-5.6757293	12.0536003	104.6500015
-5.6757293	12.1023998	102.0699997
-5.6757293	12.1512003	98.5999985
-5.6757293	12.1999998	97.4599991
-5.6757293	12.2488003	100.3799973
-5.6757293	12.2975998	103.0999985
-5.6757293	12.3464003	100.2600021
-5.6757293	12.3951998	104.6100006
-5.6757293	12.4440002	105.5000000
-5.6757293	12.4927998	104.8499985
-5.6757293	12.5416002	96.4499969
-5.6757293	12.5904007	102.9300003
-5.6757293	12.6392002	105.0899963
-5.6757293	12.6880007	105.1800003
-5.6757293	12.7368002	104.0699997
-5.6757293	12.7856007	104.0999985
-5.6757293	12.8344002	103.0400009
-5.6757293	12.8832006	103.1899948
-5.6757293	12.9320002	102.8300018
-5.6757293	12.9808006	105.2900009
-5.6757293	13.0296001	106.6399994
-5.6757293	13.0784006	104.5699997

-5.6757293	13.1272001	101.4499969
-5.6757293	13.1760006	102.7999954
-5.6757293	13.2248001	100.7200012
-5.6757293	13.2736006	100.0000000
-5.6757293	13.3224001	101.5699997
-5.6757293	13.3712006	102.6800003
-5.6757293	13.4200001	103.0499954
-5.6757293	13.4688005	95.2500000
-5.6757293	13.5176001	103.8399963
-5.6757293	13.5664005	103.9199982
-5.6757293	13.6152000	105.4199982
-5.6757293	13.6640005	104.6199951
-5.6757293	13.7128000	102.3399963
-5.6757293	13.7616005	101.9599991
-5.6757293	13.8104000	102.9700012
-5.6757293	13.8592005	102.5999985
-5.6757293	13.9080000	106.9300003
-5.6757293	13.9568005	106.1199951
-5.6757293	14.0056000	100.1699982
-5.6757293	14.0544004	103.1299973
-5.6757293	14.1032000	103.8499985
-5.6757293	14.1520004	99.2900009
-5.6757293	14.2007999	102.9899979
-5.6757293	14.2496004	100.0800018
-5.6757293	14.2983999	102.2099991
-5.6757293	14.3472004	104.1999969
-5.6757293	14.3959999	105.1199951
-5.6757293	14.4448004	103.0400009
-5.6757293	14.4935999	103.7299957
-5.6757293	14.5424004	104.2799988
-5.6757293	14.5911999	101.9700012
-5.6757293	14.6400003	100.9799957
-5.6757293	14.6887999	103.7999954
-5.6757293	14.7376003	106.3199997
-5.6757293	14.7863998	105.8899994
-5.6757293	14.8352003	103.3499985
-5.6757293	14.8839998	102.7200012
-5.6757293	14.9328003	103.5999985
-5.6757293	14.9815998	104.6500015
-5.6757293	15.0304003	102.4399948
-5.6695933	0.3416000	104.7500000
-5.6695933	0.3904000	100.2900009
-5.6695933	0.4392000	107.1399994
-5.6695933	0.4880000	106.5800018
-5.6695933	0.5368000	104.8699951
-5.6695933	0.5856000	105.3799973
-5.6695933	0.6344000	104.0000000
-5.6695933	0.6832000	101.8199997
-5.6695933	0.7320000	104.7900009
-5.6695933	0.7808000	107.1899948
-5.6695933	0.8296000	108.4199982
-5.6695933	0.8784000	108.8099976
-5.6695933	0.9272000	108.3699951
-5.6695933	0.9760000	106.6800003
-5.6695933	1.0247999	104.8099976
-5.6695933	1.0735999	102.7900009
-5.6695933	1.1223999	102.4700012
-5.6695933	1.1711999	102.6500015

-5.6695933	1.2200000	102.9399948
-5.6695933	1.2688000	104.4199982
-5.6695933	1.3176000	104.9300003
-5.6695933	1.3664000	104.6699982
-5.6695933	1.4152000	107.3699951
-5.6695933	1.4640000	110.0599976
-5.6695933	1.5128000	110.4399948
-5.6695933	1.5616000	109.3899994
-5.6695933	1.6104000	110.1199951
-5.6695933	1.6592000	109.0000000
-5.6695933	1.7079999	105.6599960
-5.6695933	1.7567999	105.7999954
-5.6695933	1.8055999	104.9199982
-5.6695933	1.8543999	106.5999985
-5.6695933	1.9031999	108.7399979
-5.6695933	1.9519999	107.4099960
-5.6695933	2.0007999	102.1800003
-5.6695933	2.0495999	99.6299973
-5.6695933	2.0983999	101.8399963
-5.6695933	2.1471999	102.3600006
-5.6695933	2.1959999	102.2500000
-5.6695933	2.2447999	102.3399963
-5.6695933	2.2935998	103.2399979
-5.6695933	2.3423998	104.1100006
-5.6695933	2.3912001	103.1800003
-5.6695933	2.4400001	103.5999985
-5.6695933	2.4888000	106.1299973
-5.6695933	2.5376000	105.7200012
-5.6695933	2.5864000	104.2699966
-5.6695933	2.6352000	104.7099991
-5.6695933	2.6840000	107.4599991
-5.6695933	2.7328000	105.4599991
-5.6695933	2.7816000	101.6999969
-5.6695933	2.8304000	103.8600006
-5.6695933	2.8792000	103.4000015
-5.6695933	2.9280000	103.7699966
-5.6695933	2.9768000	106.4799957
-5.6695933	3.0256000	106.0899963
-5.6695933	3.0743999	106.0899963
-5.6695933	3.1231999	104.4899979
-5.6695933	3.1719999	104.7699966
-5.6695933	3.2207999	103.9899979
-5.6695933	3.2695999	104.0599976
-5.6695933	3.3183999	103.6699982
-5.6695933	3.3671999	102.5000000
-5.6695933	3.4159999	102.2099991
-5.6695933	3.4647999	105.6299973
-5.6695933	3.5135999	104.9899979
-5.6695933	3.5623999	102.1399994
-5.6695933	3.6111999	102.7799988
-5.6695933	3.6599998	104.6199951
-5.6695933	3.7087998	103.0599976
-5.6695933	3.7575998	101.1100006
-5.6695933	3.8063998	102.0299988
-5.6695933	3.8551998	104.8199997
-5.6695933	3.9039998	106.1599960
-5.6695933	3.9527998	106.7200012
-5.6695933	4.0016003	104.8799973

-5.6695933	4.0503998	103.0599976
-5.6695933	4.0991998	100.9799957
-5.6695933	4.1479998	104.3799973
-5.6695933	4.1967998	103.9099960
-5.6695933	4.2455997	105.7099991
-5.6695933	4.2943997	107.5100021
-5.6695933	4.3431997	109.0400009
-5.6695933	4.3919997	106.6100006
-5.6695933	4.4407997	103.6699982
-5.6695933	4.4895997	106.3199997
-5.6695933	4.5383997	107.2399979
-5.6695933	4.5871997	109.0499954
-5.6695933	4.6359997	111.3799973
-5.6695933	4.6847997	114.0599976
-5.6695933	4.7336001	112.9799957
-5.6695933	4.7824001	108.8099976
-5.6695933	4.8312001	107.1899948
-5.6695933	4.8800001	108.8699951
-5.6695933	4.9288001	107.8899994
-5.6695933	4.9776001	102.2799988
-5.6695933	5.0264001	102.5800018
-5.6695933	5.0752001	103.2799988
-5.6695933	5.1240001	101.4499969
-5.6695933	5.1728001	104.8300018
-5.6695933	5.2216001	104.6899948
-5.6695933	5.2704000	103.9300003
-5.6695933	5.3192000	100.7699966
-5.6695933	5.3680000	105.0499954
-5.6695933	5.4168000	104.5400009
-5.6695933	5.4656000	103.0199966
-5.6695933	5.5144000	103.0899963
-5.6695933	5.5632000	103.8600006
-5.6695933	5.6120000	104.9899979
-5.6695933	5.6608000	104.4399948
-5.6695933	5.7096000	106.7399979
-5.6695933	5.7584000	105.5000000
-5.6695933	5.8072000	106.2200012
-5.6695933	5.8559999	105.5400009
-5.6695933	5.9047999	101.9099960
-5.6695933	5.9535999	105.0400009
-5.6695933	6.0023999	106.6800003
-5.6695933	6.0511999	106.2500000
-5.6695933	6.0999999	107.1599960
-5.6695933	6.1487999	104.5000000
-5.6695933	6.1975999	106.5000000
-5.6695933	6.2463999	105.4199982
-5.6695933	6.2951999	104.4700012
-5.6695933	6.3439999	103.9199982
-5.6695933	6.3927999	105.5699997
-5.6695933	6.4415998	106.0000000
-5.6695933	6.4903998	105.0299988
-5.6695933	6.5391998	105.0699997
-5.6695933	6.5879998	103.2099991
-5.6695933	6.6367998	103.7299957
-5.6695933	6.6855998	105.7900009
-5.6695933	6.7343998	104.5999985
-5.6695933	6.7831998	103.4099960
-5.6695933	6.8319998	102.1299973

-5.6695933	6.8807998	101.4899979
-5.6695933	6.9295998	101.2399979
-5.6695933	6.9783998	105.1399994
-5.6695933	7.0271997	103.3300018
-5.6695933	7.0759997	104.7399979
-5.6695933	7.1247997	105.1199951
-5.6695933	7.1735997	103.0199966
-5.6695933	7.2223997	107.3199997
-5.6695933	7.2711997	106.1100006
-5.6695933	7.3199997	102.3699951
-5.6695933	7.3687997	103.6100006
-5.6695933	7.4175997	105.0800018
-5.6695933	7.4663997	105.0299988
-5.6695933	7.5151997	105.5499954
-5.6695933	7.5639997	105.3499985
-5.6695933	7.6127996	104.6199951
-5.6695933	7.6615996	103.3199997
-5.6695933	7.7103996	101.8399963
-5.6695933	7.7591996	102.6500015
-5.6695933	7.8079996	105.8799973
-5.6695933	7.8567996	106.3399963
-5.6695933	7.9056001	103.3099976
-5.6695933	7.9544001	103.4000015
-5.6695933	8.0032005	103.1299973
-5.6695933	8.0520000	103.7999954
-5.6695933	8.1008005	103.6899948
-5.6695933	8.1496000	103.0899963
-5.6695933	8.1984005	102.4499969
-5.6695933	8.2472000	98.5899963
-5.6695933	8.2960005	101.6899948
-5.6695933	8.3448000	101.1599960
-5.6695933	8.3936005	103.1500015
-5.6695933	8.4424000	104.0199966
-5.6695933	8.4912004	104.0899963
-5.6695933	8.5400000	103.8499985
-5.6695933	8.5888004	104.0000000
-5.6695933	8.6375999	101.7699966
-5.6695933	8.6864004	103.5400009
-5.6695933	8.7351999	105.0800018
-5.6695933	8.7840004	103.5599976
-5.6695933	8.8327999	105.2200012
-5.6695933	8.8816004	104.8600006
-5.6695933	8.9303999	103.9899979
-5.6695933	8.9792004	102.2600021
-5.6695933	9.0279999	105.4700012
-5.6695933	9.0768003	103.2799988
-5.6695933	9.1255999	102.5299988
-5.6695933	9.1744003	103.1699982
-5.6695933	9.2231998	102.1999969
-5.6695933	9.2720003	100.9799957
-5.6695933	9.3207998	92.9300003
-5.6695933	9.3696003	102.9000015
-5.6695933	9.4184008	107.4700012
-5.6695933	9.4672003	107.2600021
-5.6695933	9.5160007	106.2200012
-5.6695933	9.5648003	104.6599960
-5.6695933	9.6136007	104.6100006
-5.6695933	9.6624002	103.4700012

-5.6695933	9.7112007	103.0899963
-5.6695933	9.7600002	101.4700012
-5.6695933	9.8088007	103.0000000
-5.6695933	9.8576002	105.1699982
-5.6695933	9.9064007	105.2200012
-5.6695933	9.9552002	103.6599960
-5.6695933	10.0040007	104.2500000
-5.6695933	10.0528002	104.7299957
-5.6695933	10.1016006	104.1199951
-5.6695933	10.1504002	103.9499969
-5.6695933	10.1992006	104.6699982
-5.6695933	10.2480001	104.6500015
-5.6695933	10.2968006	103.0599976
-5.6695933	10.3456001	100.4599991
-5.6695933	10.3944006	99.0899963
-5.6695933	10.4432001	100.8399963
-5.6695933	10.4920006	103.4399948
-5.6695933	10.5408001	103.3499985
-5.6695933	10.5896006	101.7099991
-5.6695933	10.6384001	102.0100021
-5.6695933	10.6872005	100.9300003
-5.6695933	10.7360001	100.4899979
-5.6695933	10.7848005	101.3899994
-5.6695933	10.8336000	102.9899979
-5.6695933	10.8824005	104.5699997
-5.6695933	10.9312000	105.1599960
-5.6695933	10.9800005	103.9599991
-5.6695933	11.0288000	98.5100021
-5.6695933	11.0776005	101.4700012
-5.6695933	11.1264000	103.9099960
-5.6695933	11.1752005	100.4300003
-5.6695933	11.2240000	99.6100006
-5.6695933	11.2728004	101.2799988
-5.6695933	11.3216000	103.5199966
-5.6695933	11.3704004	104.6199951
-5.6695933	11.4191999	103.9599991
-5.6695933	11.4680004	104.1500015
-5.6695933	11.5167999	103.6999969
-5.6695933	11.5656004	101.2099991
-5.6695933	11.6143999	102.2799988
-5.6695933	11.6632004	103.6500015
-5.6695933	11.7119999	102.8199997
-5.6695933	11.7608004	101.1100006
-5.6695933	11.8095999	104.7500000
-5.6695933	11.8584003	106.3300018
-5.6695933	11.9071999	105.7699966
-5.6695933	11.9560003	103.3799973
-5.6695933	12.0047998	100.0199966
-5.6695933	12.0536003	104.2099991
-5.6695933	12.1023998	102.3699951
-5.6695933	12.1512003	99.2999954
-5.6695933	12.1999998	97.0800018
-5.6695933	12.2488003	100.1100006
-5.6695933	12.2975998	101.7999954
-5.6695933	12.3464003	99.7600021
-5.6695933	12.3951998	104.2900009
-5.6695933	12.4440002	105.5999985
-5.6695933	12.4927998	105.5499954

-5.6695933	12.5416002	99.0299988
-5.6695933	12.5904007	102.8499985
-5.6695933	12.6392002	104.8699951
-5.6695933	12.6880007	104.6999969
-5.6695933	12.7368002	103.6999969
-5.6695933	12.7856007	104.6800003
-5.6695933	12.8344002	103.3799973
-5.6695933	12.8832006	103.2999954
-5.6695933	12.9320002	102.9199982
-5.6695933	12.9808006	105.3399963
-5.6695933	13.0296001	105.6100006
-5.6695933	13.0784006	104.4599991
-5.6695933	13.1272001	101.3199997
-5.6695933	13.1760006	102.1699982
-5.6695933	13.2248001	100.9300003
-5.6695933	13.2736006	100.3600006
-5.6695933	13.3224001	102.0599976
-5.6695933	13.3712006	103.5299988
-5.6695933	13.4200001	103.3699951
-5.6695933	13.4688005	96.7900009
-5.6695933	13.5176001	103.7200012
-5.6695933	13.5664005	104.3600006
-5.6695933	13.6152000	105.5999985
-5.6695933	13.6640005	105.5199966
-5.6695933	13.7128000	102.9799957
-5.6695933	13.7616005	102.6399994
-5.6695933	13.8104000	103.9799957
-5.6695933	13.8592005	102.0299988
-5.6695933	13.9080000	106.9599991
-5.6695933	13.9568005	106.8699951
-5.6695933	14.0056000	100.6199951
-5.6695933	14.0544004	103.3499985
-5.6695933	14.1032000	104.1299973
-5.6695933	14.1520004	97.7600021
-5.6695933	14.2007999	103.3600006
-5.6695933	14.2496004	100.9599991
-5.6695933	14.2983999	102.9700012
-5.6695933	14.3472004	104.9499969
-5.6695933	14.3959999	105.1299973
-5.6695933	14.4448004	103.0499954
-5.6695933	14.4935999	103.3300018
-5.6695933	14.5424004	103.6999969
-5.6695933	14.5911999	102.0599976
-5.6695933	14.6400003	101.5800018
-5.6695933	14.6887999	104.0800018
-5.6695933	14.7376003	106.4799957
-5.6695933	14.7863998	105.9000015
-5.6695933	14.8352003	103.4599991
-5.6695933	14.8839998	102.6599960
-5.6695933	14.9328003	103.4899979
-5.6695933	14.9815998	104.5899963
-5.6695933	15.0304003	102.7900009
-5.6634574	0.3416000	104.7699966
-5.6634574	0.3904000	102.4199982
-5.6634574	0.4392000	107.4199982
-5.6634574	0.4880000	107.1899948
-5.6634574	0.5368000	104.4599991
-5.6634574	0.5856000	105.4899979

-5.6634574	0.6344000	104.4700012
-5.6634574	0.6832000	102.4499969
-5.6634574	0.7320000	104.8600006
-5.6634574	0.7808000	106.7999954
-5.6634574	0.8296000	107.6500015
-5.6634574	0.8784000	108.0199966
-5.6634574	0.9272000	108.4599991
-5.6634574	0.9760000	106.9099960
-5.6634574	1.0247999	104.5699997
-5.6634574	1.0735999	101.8300018
-5.6634574	1.1223999	101.6800003
-5.6634574	1.1711999	101.7699966
-5.6634574	1.2200000	102.7500000
-5.6634574	1.2688000	103.9199982
-5.6634574	1.3176000	104.4499969
-5.6634574	1.3664000	103.8899994
-5.6634574	1.4152000	106.8099976
-5.6634574	1.4640000	110.3399963
-5.6634574	1.5128000	111.0599976
-5.6634574	1.5616000	110.0699997
-5.6634574	1.6104000	110.3899994
-5.6634574	1.6592000	109.6800003
-5.6634574	1.7079999	106.4300003
-5.6634574	1.7567999	106.0599976
-5.6634574	1.8055999	105.0100021
-5.6634574	1.8543999	105.7399979
-5.6634574	1.9031999	108.2299957
-5.6634574	1.9519999	107.0599976
-5.6634574	2.0007999	101.4499969
-5.6634574	2.0495999	99.1399994
-5.6634574	2.0983999	100.8699951
-5.6634574	2.1471999	101.7200012
-5.6634574	2.1959999	101.3399963
-5.6634574	2.2447999	101.6199951
-5.6634574	2.2935998	102.6299973
-5.6634574	2.3423998	103.3799973
-5.6634574	2.3912001	102.5100021
-5.6634574	2.4400001	102.8899994
-5.6634574	2.4888000	105.8399963
-5.6634574	2.5376000	104.6800003
-5.6634574	2.5864000	103.3799973
-5.6634574	2.6352000	104.5699997
-5.6634574	2.6840000	107.2500000
-5.6634574	2.7328000	105.7299957
-5.6634574	2.7816000	102.9300003
-5.6634574	2.8304000	104.8499985
-5.6634574	2.8792000	104.0400009
-5.6634574	2.9280000	103.5000000
-5.6634574	2.9768000	106.6599960
-5.6634574	3.0256000	107.3499985
-5.6634574	3.0743999	106.6399994
-5.6634574	3.1231999	104.3300018
-5.6634574	3.1719999	104.5100021
-5.6634574	3.2207999	104.3600006
-5.6634574	3.2695999	104.5100021
-5.6634574	3.3183999	104.2200012
-5.6634574	3.3671999	103.3300018
-5.6634574	3.4159999	103.4399948

-5.6634574	3.4647999	104.8600006
-5.6634574	3.5135999	104.5100021
-5.6634574	3.5623999	101.8499985
-5.6634574	3.6111999	102.2999954
-5.6634574	3.6599998	104.0000000
-5.6634574	3.7087998	102.5299988
-5.6634574	3.7575998	101.0400009
-5.6634574	3.8063998	102.6100006
-5.6634574	3.8551998	104.7699966
-5.6634574	3.9039998	105.0899963
-5.6634574	3.9527998	106.6800003
-5.6634574	4.0016003	105.1100006
-5.6634574	4.0503998	102.6599960
-5.6634574	4.0991998	101.6199951
-5.6634574	4.1479998	105.2999954
-5.6634574	4.1967998	104.9899979
-5.6634574	4.2455997	105.7099991
-5.6634574	4.2943997	106.8099976
-5.6634574	4.3431997	108.6699982
-5.6634574	4.3919997	106.9000015
-5.6634574	4.4407997	103.5499954
-5.6634574	4.4895997	106.0699997
-5.6634574	4.5383997	107.5100021
-5.6634574	4.5871997	108.8899994
-5.6634574	4.6359997	111.1500015
-5.6634574	4.6847997	113.7299957
-5.6634574	4.7336001	112.2299957
-5.6634574	4.7824001	108.0599976
-5.6634574	4.8312001	106.3899994
-5.6634574	4.8800001	108.5999985
-5.6634574	4.9288001	107.8199997
-5.6634574	4.9776001	102.9899979
-5.6634574	5.0264001	103.0999985
-5.6634574	5.0752001	103.4599991
-5.6634574	5.1240001	99.9099960
-5.6634574	5.1728001	103.6199951
-5.6634574	5.2216001	103.7099991
-5.6634574	5.2704000	103.9000015
-5.6634574	5.3192000	100.6699982
-5.6634574	5.3680000	104.4300003
-5.6634574	5.4168000	104.5400009
-5.6634574	5.4656000	102.4099960
-5.6634574	5.5144000	102.1500015
-5.6634574	5.5632000	103.5899963
-5.6634574	5.6120000	104.8399963
-5.6634574	5.6608000	103.7399979
-5.6634574	5.7096000	106.1399994
-5.6634574	5.7584000	105.7500000
-5.6634574	5.8072000	106.5699997
-5.6634574	5.8559999	105.2200012
-5.6634574	5.9047999	102.0999985
-5.6634574	5.9535999	105.1699982
-5.6634574	6.0023999	105.9700012
-5.6634574	6.0511999	104.9300003
-5.6634574	6.0999999	106.4099960
-5.6634574	6.1487999	105.2699966
-5.6634574	6.1975999	105.7299957
-5.6634574	6.2463999	104.5100021

-5.6634574	6.2951999	104.2200012
-5.6634574	6.3439999	104.2799988
-5.6634574	6.3927999	106.0199966
-5.6634574	6.4415998	105.8099976
-5.6634574	6.4903998	103.2099991
-5.6634574	6.5391998	103.4599991
-5.6634574	6.5879998	102.5800018
-5.6634574	6.6367998	104.0199966
-5.6634574	6.6855998	105.3499985
-5.6634574	6.7343998	103.5000000
-5.6634574	6.7831998	102.9199982
-5.6634574	6.8319998	101.7399979
-5.6634574	6.8807998	100.7699966
-5.6634574	6.9295998	100.2699966
-5.6634574	6.9783998	103.9099960
-5.6634574	7.0271997	101.5599976
-5.6634574	7.0759997	104.6399994
-5.6634574	7.1247997	105.1399994
-5.6634574	7.1735997	102.4199982
-5.6634574	7.2223997	107.4000015
-5.6634574	7.2711997	105.9799957
-5.6634574	7.3199997	101.5100021
-5.6634574	7.3687997	103.2500000
-5.6634574	7.4175997	104.3899994
-5.6634574	7.4663997	104.7500000
-5.6634574	7.5151997	105.5699997
-5.6634574	7.5639997	106.3699951
-5.6634574	7.6127996	104.6699982
-5.6634574	7.6615996	102.8300018
-5.6634574	7.7103996	102.0000000
-5.6634574	7.7591996	103.6800003
-5.6634574	7.8079996	106.0199966
-5.6634574	7.8567996	106.2699966
-5.6634574	7.9056001	103.5599976
-5.6634574	7.9544001	103.1500015
-5.6634574	8.0032005	101.7099991
-5.6634574	8.0520000	102.5199966
-5.6634574	8.1008005	102.1599960
-5.6634574	8.1496000	101.7600021
-5.6634574	8.1984005	101.9000015
-5.6634574	8.2472000	98.8300018
-5.6634574	8.2960005	102.5499954
-5.6634574	8.3448000	101.1699982
-5.6634574	8.3936005	104.0699997
-5.6634574	8.4424000	104.5800018
-5.6634574	8.4912004	105.0999985
-5.6634574	8.5400000	104.9000015
-5.6634574	8.5888004	104.0299988
-5.6634574	8.6375999	101.8799973
-5.6634574	8.6864004	104.3399963
-5.6634574	8.7351999	105.1999969
-5.6634574	8.7840004	103.6599960
-5.6634574	8.8327999	105.1699982
-5.6634574	8.8816004	103.7999954
-5.6634574	8.9303999	102.9899979
-5.6634574	8.9792004	100.9399948
-5.6634574	9.0279999	105.7200012
-5.6634574	9.0768003	104.7299957

-5.6634574	9.1255999	104.5999985
-5.6634574	9.1744003	104.1599960
-5.6634574	9.2231998	102.6599960
-5.6634574	9.2720003	101.2900009
-5.6634574	9.3207998	93.8099976
-5.6634574	9.3696003	104.2600021
-5.6634574	9.4184008	107.1199951
-5.6634574	9.4672003	106.2399979
-5.6634574	9.5160007	105.2699966
-5.6634574	9.5648003	104.8099976
-5.6634574	9.6136007	104.6999969
-5.6634574	9.6624002	103.6599960
-5.6634574	9.7112007	102.3399963
-5.6634574	9.7600002	101.4899979
-5.6634574	9.8088007	102.9000015
-5.6634574	9.8576002	105.7299957
-5.6634574	9.9064007	105.9000015
-5.6634574	9.9552002	104.2299957
-5.6634574	10.0040007	105.3499985
-5.6634574	10.0528002	104.8099976
-5.6634574	10.1016006	103.1800003
-5.6634574	10.1504002	103.6899948
-5.6634574	10.1992006	104.4899979
-5.6634574	10.2480001	104.7200012
-5.6634574	10.2968006	103.2699966
-5.6634574	10.3456001	101.2200012
-5.6634574	10.3944006	99.6299973
-5.6634574	10.4432001	100.7399979
-5.6634574	10.4920006	103.6899948
-5.6634574	10.5408001	104.0699997
-5.6634574	10.5896006	102.2799988
-5.6634574	10.6384001	102.9099960
-5.6634574	10.6872005	101.0999985
-5.6634574	10.7360001	100.6299973
-5.6634574	10.7848005	101.3899994
-5.6634574	10.8336000	102.6399994
-5.6634574	10.8824005	104.4899979
-5.6634574	10.9312000	105.1500015
-5.6634574	10.9800005	103.6599960
-5.6634574	11.0288000	97.8600006
-5.6634574	11.0776005	101.1399994
-5.6634574	11.1264000	103.2099991
-5.6634574	11.1752005	100.3600006
-5.6634574	11.2240000	99.8699951
-5.6634574	11.2728004	100.9899979
-5.6634574	11.3216000	103.0899963
-5.6634574	11.3704004	104.1999969
-5.6634574	11.4191999	105.2500000
-5.6634574	11.4680004	104.4199982
-5.6634574	11.5167999	104.0599976
-5.6634574	11.5656004	101.6399994
-5.6634574	11.6143999	102.6800003
-5.6634574	11.6632004	104.2699966
-5.6634574	11.7119999	103.6800003
-5.6634574	11.7608004	101.5800018
-5.6634574	11.8095999	105.6599960
-5.6634574	11.8584003	106.8499985
-5.6634574	11.9071999	105.4799957

-5.6634574	11.9560003	102.3699951
-5.6634574	12.0047998	97.9899979
-5.6634574	12.0536003	103.6899948
-5.6634574	12.1023998	102.1299973
-5.6634574	12.1512003	100.3600006
-5.6634574	12.1999998	96.3899994
-5.6634574	12.2488003	100.1199951
-5.6634574	12.2975998	101.2900009
-5.6634574	12.3464003	100.1699982
-5.6634574	12.3951998	104.3499985
-5.6634574	12.4440002	105.4799957
-5.6634574	12.4927998	105.0199966
-5.6634574	12.5416002	101.0800018
-5.6634574	12.5904007	102.9399948
-5.6634574	12.6392002	104.8499985
-5.6634574	12.6880007	104.4799957
-5.6634574	12.7368002	103.9799957
-5.6634574	12.7856007	105.0299988
-5.6634574	12.8344002	103.6399994
-5.6634574	12.8832006	103.3199997
-5.6634574	12.9320002	103.4799957
-5.6634574	12.9808006	105.9000015
-5.6634574	13.0296001	104.8899994
-5.6634574	13.0784006	104.4399948
-5.6634574	13.1272001	101.2399979
-5.6634574	13.1760006	101.9700012
-5.6634574	13.2248001	101.5499954
-5.6634574	13.2736006	101.3399963
-5.6634574	13.3224001	102.4700012
-5.6634574	13.3712006	104.4199982
-5.6634574	13.4200001	103.9599991
-5.6634574	13.4688005	98.5299988
-5.6634574	13.5176001	103.3499985
-5.6634574	13.5664005	104.6899948
-5.6634574	13.6152000	105.4300003
-5.6634574	13.6640005	105.5899963
-5.6634574	13.7128000	103.6699982
-5.6634574	13.7616005	103.7999954
-5.6634574	13.8104000	105.0999985
-5.6634574	13.8592005	100.9899979
-5.6634574	13.9080000	106.1500015
-5.6634574	13.9568005	107.1899948
-5.6634574	14.0056000	101.2099991
-5.6634574	14.0544004	103.9599991
-5.6634574	14.1032000	104.8899994
-5.6634574	14.1520004	96.0599976
-5.6634574	14.2007999	103.6699982
-5.6634574	14.2496004	102.1899948
-5.6634574	14.2983999	103.9499969
-5.6634574	14.3472004	105.2999954
-5.6634574	14.3959999	104.5899963
-5.6634574	14.4448004	102.8899994
-5.6634574	14.4935999	103.1199951
-5.6634574	14.5424004	103.0999985
-5.6634574	14.5911999	102.2099991
-5.6634574	14.6400003	102.0899963
-5.6634574	14.6887999	104.3499985
-5.6634574	14.7376003	106.6500015

-5.6634574	14.7863998	105.7699966
-5.6634574	14.8352003	103.9099960
-5.6634574	14.8839998	102.4000015
-5.6634574	14.9328003	102.9700012
-5.6634574	14.9815998	104.2299957
-5.6634574	15.0304003	103.1100006
-5.6573215	0.3416000	105.3399963
-5.6573215	0.3904000	104.9000015
-5.6573215	0.4392000	107.1800003
-5.6573215	0.4880000	106.7299957
-5.6573215	0.5368000	103.1699982
-5.6573215	0.5856000	104.8799973
-5.6573215	0.6344000	104.5699997
-5.6573215	0.6832000	103.8399963
-5.6573215	0.7320000	104.2299957
-5.6573215	0.7808000	106.3099976
-5.6573215	0.8296000	107.0999985
-5.6573215	0.8784000	107.0400009
-5.6573215	0.9272000	108.0499954
-5.6573215	0.9760000	107.0000000
-5.6573215	1.0247999	104.4399948
-5.6573215	1.0735999	100.5000000
-5.6573215	1.1223999	101.2999954
-5.6573215	1.1711999	101.6399994
-5.6573215	1.2200000	102.9000015
-5.6573215	1.2688000	103.5800018
-5.6573215	1.3176000	103.9099960
-5.6573215	1.3664000	104.1100006
-5.6573215	1.4152000	107.6199951
-5.6573215	1.4640000	110.9599991
-5.6573215	1.5128000	111.5699997
-5.6573215	1.5616000	110.3300018
-5.6573215	1.6104000	109.8499985
-5.6573215	1.6592000	109.6500015
-5.6573215	1.7079999	106.9499969
-5.6573215	1.7567999	106.2299957
-5.6573215	1.8055999	105.1299973
-5.6573215	1.8543999	105.1500015
-5.6573215	1.9031999	107.7999954
-5.6573215	1.9519999	106.7299957
-5.6573215	2.0007999	101.0100021
-5.6573215	2.0495999	99.3699951
-5.6573215	2.0983999	100.8499985
-5.6573215	2.1471999	101.7099991
-5.6573215	2.1959999	101.0100021
-5.6573215	2.2447999	101.6100006
-5.6573215	2.2935998	102.3099976
-5.6573215	2.3423998	102.8799973
-5.6573215	2.3912001	102.3600006
-5.6573215	2.4400001	103.0199966
-5.6573215	2.4888000	106.2900009
-5.6573215	2.5376000	104.7900009
-5.6573215	2.5864000	102.6500015
-5.6573215	2.6352000	103.9199982
-5.6573215	2.6840000	107.1199951
-5.6573215	2.7328000	106.0800018
-5.6573215	2.7816000	104.1699982
-5.6573215	2.8304000	105.7500000

-5.6573215	2.8792000	104.4599991
-5.6573215	2.9280000	103.3799973
-5.6573215	2.9768000	106.5599976
-5.6573215	3.0256000	108.4199982
-5.6573215	3.0743999	107.2099991
-5.6573215	3.1231999	104.0000000
-5.6573215	3.1719999	104.3600006
-5.6573215	3.2207999	105.1399994
-5.6573215	3.2695999	105.2200012
-5.6573215	3.3183999	104.5800018
-5.6573215	3.3671999	103.7099991
-5.6573215	3.4159999	104.3399963
-5.6573215	3.4647999	104.2299957
-5.6573215	3.5135999	104.2699966
-5.6573215	3.5623999	101.9499969
-5.6573215	3.6111999	101.7600021
-5.6573215	3.6599998	103.0999985
-5.6573215	3.7087998	102.5800018
-5.6573215	3.7575998	101.7799988
-5.6573215	3.8063998	103.4000015
-5.6573215	3.8551998	104.6699982
-5.6573215	3.9039998	103.5699997
-5.6573215	3.9527998	105.9199982
-5.6573215	4.0016003	105.1399994
-5.6573215	4.0503998	102.9099960
-5.6573215	4.0991998	102.6199951
-5.6573215	4.1479998	106.2200012
-5.6573215	4.1967998	105.4899979
-5.6573215	4.2455997	106.2999954
-5.6573215	4.2943997	106.4599991
-5.6573215	4.3431997	108.4799957
-5.6573215	4.3919997	107.3099976
-5.6573215	4.4407997	103.9399948
-5.6573215	4.4895997	105.8300018
-5.6573215	4.5383997	108.4399948
-5.6573215	4.5871997	109.2799988
-5.6573215	4.6359997	110.7200012
-5.6573215	4.6847997	112.7999954
-5.6573215	4.7336001	111.0100021
-5.6573215	4.7824001	107.6100006
-5.6573215	4.8312001	105.8600006
-5.6573215	4.8800001	108.1899948
-5.6573215	4.9288001	107.4199982
-5.6573215	4.9776001	103.7500000
-5.6573215	5.0264001	103.7500000
-5.6573215	5.0752001	103.5800018
-5.6573215	5.1240001	98.0899963
-5.6573215	5.1728001	101.8899994
-5.6573215	5.2216001	101.9399948
-5.6573215	5.2704000	103.1899948
-5.6573215	5.3192000	100.1899948
-5.6573215	5.3680000	103.6999969
-5.6573215	5.4168000	104.7500000
-5.6573215	5.4656000	102.2699966
-5.6573215	5.5144000	101.4799957
-5.6573215	5.5632000	103.3399963
-5.6573215	5.6120000	104.1899948
-5.6573215	5.6608000	102.7299957

-5.6573215	5.7096000	105.4199982
-5.6573215	5.7584000	105.4300003
-5.6573215	5.8072000	106.1899948
-5.6573215	5.8559999	104.9599991
-5.6573215	5.9047999	102.8099976
-5.6573215	5.9535999	105.1999969
-5.6573215	6.0023999	104.9799957
-5.6573215	6.0511999	103.5699997
-5.6573215	6.0999999	105.3699951
-5.6573215	6.1487999	104.7299957
-5.6573215	6.1975999	105.3199997
-5.6573215	6.2463999	104.3799973
-5.6573215	6.2951999	104.5800018
-5.6573215	6.3439999	104.9599991
-5.6573215	6.3927999	106.0999985
-5.6573215	6.4415998	105.4300003
-5.6573215	6.4903998	100.7900009
-5.6573215	6.5391998	101.5999985
-5.6573215	6.5879998	102.1800003
-5.6573215	6.6367998	104.2799988
-5.6573215	6.6855998	104.1999969
-5.6573215	6.7343998	102.4599991
-5.6573215	6.7831998	102.3499985
-5.6573215	6.8319998	101.0800018
-5.6573215	6.8807998	100.3499985
-5.6573215	6.9295998	100.7399979
-5.6573215	6.9783998	103.3300018
-5.6573215	7.0271997	100.7799988
-5.6573215	7.0759997	104.7999954
-5.6573215	7.1247997	105.2900009
-5.6573215	7.1735997	103.4099960
-5.6573215	7.2223997	107.7399979
-5.6573215	7.2711997	106.0800018
-5.6573215	7.3199997	100.0699997
-5.6573215	7.3687997	102.4799957
-5.6573215	7.4175997	103.5999985
-5.6573215	7.4663997	104.0100021
-5.6573215	7.5151997	104.9899979
-5.6573215	7.5639997	106.0699997
-5.6573215	7.6127996	104.8799973
-5.6573215	7.6615996	103.4000015
-5.6573215	7.7103996	102.3300018
-5.6573215	7.7591996	104.2200012
-5.6573215	7.8079996	105.5199966
-5.6573215	7.8567996	105.8199997
-5.6573215	7.9056001	104.0199966
-5.6573215	7.9544001	103.9099960
-5.6573215	8.0032005	101.5100021
-5.6573215	8.0520000	102.2900009
-5.6573215	8.1008005	101.8399963
-5.6573215	8.1496000	100.8899994
-5.6573215	8.1984005	101.2500000
-5.6573215	8.2472000	98.4499969
-5.6573215	8.2960005	103.4300003
-5.6573215	8.3448000	101.5599976
-5.6573215	8.3936005	104.6599960
-5.6573215	8.4424000	104.7999954
-5.6573215	8.4912004	105.0400009

-5.6573215	8.5400000	105.0899963
-5.6573215	8.5888004	104.0000000
-5.6573215	8.6375999	102.0999985
-5.6573215	8.6864004	105.2099991
-5.6573215	8.7351999	105.9799957
-5.6573215	8.7840004	104.4099960
-5.6573215	8.8327999	105.1699982
-5.6573215	8.8816004	102.2799988
-5.6573215	8.9303999	100.9399948
-5.6573215	8.9792004	98.9799957
-5.6573215	9.0279999	105.7299957
-5.6573215	9.0768003	105.7099991
-5.6573215	9.1255999	105.1100006
-5.6573215	9.1744003	104.5400009
-5.6573215	9.2231998	102.8600006
-5.6573215	9.2720003	101.1399994
-5.6573215	9.3207998	97.0299988
-5.6573215	9.3696003	105.3699951
-5.6573215	9.4184008	106.8600006
-5.6573215	9.4672003	105.4799957
-5.6573215	9.5160007	104.3199997
-5.6573215	9.5648003	103.9099960
-5.6573215	9.6136007	104.9199982
-5.6573215	9.6624002	104.4899979
-5.6573215	9.7112007	102.4799957
-5.6573215	9.7600002	101.9199982
-5.6573215	9.8088007	102.9700012
-5.6573215	9.8576002	105.2999954
-5.6573215	9.9064007	105.7600021
-5.6573215	9.9552002	103.8600006
-5.6573215	10.0040007	105.1100006
-5.6573215	10.0528002	104.2999954
-5.6573215	10.1016006	102.0100021
-5.6573215	10.1504002	103.2699966
-5.6573215	10.1992006	104.2099991
-5.6573215	10.2480001	104.7799988
-5.6573215	10.2968006	103.8300018
-5.6573215	10.3456001	102.2399979
-5.6573215	10.3944006	99.9799957
-5.6573215	10.4432001	101.2799988
-5.6573215	10.4920006	104.2200012
-5.6573215	10.5408001	104.6100006
-5.6573215	10.5896006	102.7500000
-5.6573215	10.6384001	103.5999985
-5.6573215	10.6872005	101.3399963
-5.6573215	10.7360001	101.6999969
-5.6573215	10.7848005	101.7600021
-5.6573215	10.8336000	102.1299973
-5.6573215	10.8824005	103.9399948
-5.6573215	10.9312000	104.6399994
-5.6573215	10.9800005	103.4000015
-5.6573215	11.0288000	98.9199982
-5.6573215	11.0776005	101.0699997
-5.6573215	11.1264000	102.6100006
-5.6573215	11.1752005	100.3600006
-5.6573215	11.2240000	100.6999969
-5.6573215	11.2728004	100.9700012
-5.6573215	11.3216000	102.8699951

-5.6573215	11.3704004	104.7200012
-5.6573215	11.4191999	106.5999985
-5.6573215	11.4680004	104.3300018
-5.6573215	11.5167999	103.8899994
-5.6573215	11.5656004	101.9700012
-5.6573215	11.6143999	102.7399979
-5.6573215	11.6632004	104.3499985
-5.6573215	11.7119999	104.1699982
-5.6573215	11.7608004	102.6299973
-5.6573215	11.8095999	106.3099976
-5.6573215	11.8584003	106.4199982
-5.6573215	11.9071999	104.4700012
-5.6573215	11.9560003	101.5999985
-5.6573215	12.0047998	96.7500000
-5.6573215	12.0536003	103.7600021
-5.6573215	12.1023998	102.5000000
-5.6573215	12.1512003	101.9199982
-5.6573215	12.1999998	97.2600021
-5.6573215	12.2488003	100.5100021
-5.6573215	12.2975998	101.2600021
-5.6573215	12.3464003	100.9799957
-5.6573215	12.3951998	104.4000015
-5.6573215	12.4440002	105.2099991
-5.6573215	12.4927998	104.4099960
-5.6573215	12.5416002	102.7200012
-5.6573215	12.5904007	102.4499969
-5.6573215	12.6392002	104.7500000
-5.6573215	12.6880007	104.3499985
-5.6573215	12.7368002	104.0899963
-5.6573215	12.7856007	105.2900009
-5.6573215	12.8344002	104.2600021
-5.6573215	12.8832006	103.9000015
-5.6573215	12.9320002	104.1899948
-5.6573215	12.9808006	106.0199966
-5.6573215	13.0296001	103.7999954
-5.6573215	13.0784006	104.0400009
-5.6573215	13.1272001	100.9899979
-5.6573215	13.1760006	102.3799973
-5.6573215	13.2248001	102.7999954
-5.6573215	13.2736006	102.9300003
-5.6573215	13.3224001	101.9899979
-5.6573215	13.3712006	104.6999969
-5.6573215	13.4200001	104.6599960
-5.6573215	13.4688005	100.7900009
-5.6573215	13.5176001	103.1800003
-5.6573215	13.5664005	105.0599976
-5.6573215	13.6152000	104.9899979
-5.6573215	13.6640005	104.8499985
-5.6573215	13.7128000	102.9899979
-5.6573215	13.7616005	103.8199997
-5.6573215	13.8104000	105.2699966
-5.6573215	13.8592005	101.0899963
-5.6573215	13.9080000	104.9399948
-5.6573215	13.9568005	107.1899948
-5.6573215	14.0056000	102.4700012
-5.6573215	14.0544004	103.6399994
-5.6573215	14.1032000	105.1399994
-5.6573215	14.1520004	97.5400009

-5.6573215	14.2007999	103.8600006
-5.6573215	14.2496004	102.9099960
-5.6573215	14.2983999	104.4099960
-5.6573215	14.3472004	105.2200012
-5.6573215	14.3959999	103.5899963
-5.6573215	14.4448004	101.9899979
-5.6573215	14.4935999	102.5699997
-5.6573215	14.5424004	102.6699982
-5.6573215	14.5911999	102.2099991
-5.6573215	14.6400003	102.1999969
-5.6573215	14.6887999	103.5899963
-5.6573215	14.7376003	105.3199997
-5.6573215	14.7863998	104.7500000
-5.6573215	14.8352003	104.1500015
-5.6573215	14.8839998	102.5800018
-5.6573215	14.9328003	103.3600006
-5.6573215	14.9815998	103.9399948
-5.6573215	15.0304003	103.2799988
-5.6511855	0.3416000	106.0499954
-5.6511855	0.3904000	106.2299957
-5.6511855	0.4392000	106.8799973
-5.6511855	0.4880000	106.2099991
-5.6511855	0.5368000	101.4300003
-5.6511855	0.5856000	105.0999985
-5.6511855	0.6344000	105.0599976
-5.6511855	0.6832000	105.1899948
-5.6511855	0.7320000	103.0699997
-5.6511855	0.7808000	105.6800003
-5.6511855	0.8296000	106.5000000
-5.6511855	0.8784000	105.9799957
-5.6511855	0.9272000	107.3300018
-5.6511855	0.9760000	106.6299973
-5.6511855	1.0247999	103.6899948
-5.6511855	1.0735999	98.6800003
-5.6511855	1.1223999	100.6999969
-5.6511855	1.1711999	101.8199997
-5.6511855	1.2200000	103.3499985
-5.6511855	1.2688000	102.4899979
-5.6511855	1.3176000	103.3399963
-5.6511855	1.3664000	104.7500000
-5.6511855	1.4152000	108.2600021
-5.6511855	1.4640000	111.3499985
-5.6511855	1.5128000	111.7699966
-5.6511855	1.5616000	109.9700012
-5.6511855	1.6104000	109.0999985
-5.6511855	1.6592000	109.5199966
-5.6511855	1.7079999	107.3499985
-5.6511855	1.7567999	105.9300003
-5.6511855	1.8055999	105.4700012
-5.6511855	1.8543999	105.7600021
-5.6511855	1.9031999	108.6699982
-5.6511855	1.9519999	106.6800003
-5.6511855	2.0007999	100.7299957
-5.6511855	2.0495999	100.6399994
-5.6511855	2.0983999	102.3899994
-5.6511855	2.1471999	102.4199982
-5.6511855	2.1959999	100.3499985
-5.6511855	2.2447999	101.9499969

-5.6511855	2.2935998	102.8799973
-5.6511855	2.3423998	103.3199997
-5.6511855	2.3912001	102.7600021
-5.6511855	2.4400001	103.2299957
-5.6511855	2.4888000	106.3399963
-5.6511855	2.5376000	103.9599991
-5.6511855	2.5864000	101.7099991
-5.6511855	2.6352000	103.5199966
-5.6511855	2.6840000	106.6100006
-5.6511855	2.7328000	105.8099976
-5.6511855	2.7816000	105.0499954
-5.6511855	2.8304000	106.3899994
-5.6511855	2.8792000	105.2699966
-5.6511855	2.9280000	103.8199997
-5.6511855	2.9768000	106.6399994
-5.6511855	3.0256000	109.0100021
-5.6511855	3.0743999	108.1199951
-5.6511855	3.1231999	104.4399948
-5.6511855	3.1719999	104.8499985
-5.6511855	3.2207999	105.4099960
-5.6511855	3.2695999	105.3600006
-5.6511855	3.3183999	104.3600006
-5.6511855	3.3671999	103.3199997
-5.6511855	3.4159999	104.3499985
-5.6511855	3.4647999	103.2600021
-5.6511855	3.5135999	104.3799973
-5.6511855	3.5623999	102.7799988
-5.6511855	3.6111999	102.5699997
-5.6511855	3.6599998	103.5000000
-5.6511855	3.7087998	103.5599976
-5.6511855	3.7575998	103.1899948
-5.6511855	3.8063998	104.5400009
-5.6511855	3.8551998	104.4399948
-5.6511855	3.9039998	101.8399963
-5.6511855	3.9527998	104.9599991
-5.6511855	4.0016003	104.9099960
-5.6511855	4.0503998	103.0400009
-5.6511855	4.0991998	103.5699997
-5.6511855	4.1479998	107.1999969
-5.6511855	4.1967998	105.8499985
-5.6511855	4.2455997	106.3199997
-5.6511855	4.2943997	106.0899963
-5.6511855	4.3431997	107.9199982
-5.6511855	4.3919997	106.8499985
-5.6511855	4.4407997	104.0000000
-5.6511855	4.4895997	105.1800003
-5.6511855	4.5383997	108.9300003
-5.6511855	4.5871997	109.4300003
-5.6511855	4.6359997	110.9099960
-5.6511855	4.6847997	112.4300003
-5.6511855	4.7336001	110.0400009
-5.6511855	4.7824001	107.8099976
-5.6511855	4.8312001	105.4399948
-5.6511855	4.8800001	107.7299957
-5.6511855	4.9288001	107.1800003
-5.6511855	4.9776001	104.6299973
-5.6511855	5.0264001	104.6299973
-5.6511855	5.0752001	104.1899948

-5.6511855	5.1240001	98.5299988
-5.6511855	5.1728001	100.2799988
-5.6511855	5.2216001	101.0899963
-5.6511855	5.2704000	102.5100021
-5.6511855	5.3192000	99.5100021
-5.6511855	5.3680000	103.7099991
-5.6511855	5.4168000	105.7399979
-5.6511855	5.4656000	103.2900009
-5.6511855	5.5144000	101.9700012
-5.6511855	5.5632000	103.2200012
-5.6511855	5.6120000	103.8799973
-5.6511855	5.6608000	101.6199951
-5.6511855	5.7096000	103.8099976
-5.6511855	5.7584000	104.4700012
-5.6511855	5.8072000	105.1800003
-5.6511855	5.8559999	104.6199951
-5.6511855	5.9047999	103.2299957
-5.6511855	5.9535999	104.5000000
-5.6511855	6.0023999	103.5899963
-5.6511855	6.0511999	101.8300018
-5.6511855	6.0999999	104.2600021
-5.6511855	6.1487999	103.4300003
-5.6511855	6.1975999	104.9700012
-5.6511855	6.2463999	104.5100021
-5.6511855	6.2951999	104.7799988
-5.6511855	6.3439999	104.8899994
-5.6511855	6.3927999	105.8199997
-5.6511855	6.4415998	104.8600006
-5.6511855	6.4903998	98.6699982
-5.6511855	6.5391998	100.3399963
-5.6511855	6.5879998	102.2399979
-5.6511855	6.6367998	104.5999985
-5.6511855	6.6855998	102.9099960
-5.6511855	6.7343998	102.3699951
-5.6511855	6.7831998	102.2399979
-5.6511855	6.8319998	100.7600021
-5.6511855	6.8807998	100.1899948
-5.6511855	6.9295998	101.3699951
-5.6511855	6.9783998	103.4199982
-5.6511855	7.0271997	100.7099991
-5.6511855	7.0759997	104.3300018
-5.6511855	7.1247997	105.2600021
-5.6511855	7.1735997	103.9799957
-5.6511855	7.2223997	107.2699966
-5.6511855	7.2711997	106.3699951
-5.6511855	7.3199997	99.6899948
-5.6511855	7.3687997	101.7500000
-5.6511855	7.4175997	102.3899994
-5.6511855	7.4663997	103.2600021
-5.6511855	7.5151997	105.0800018
-5.6511855	7.5639997	106.1299973
-5.6511855	7.6127996	105.6199951
-5.6511855	7.6615996	104.5899963
-5.6511855	7.7103996	103.4000015
-5.6511855	7.7591996	105.3099976
-5.6511855	7.8079996	105.7399979
-5.6511855	7.8567996	105.2299957
-5.6511855	7.9056001	103.8199997

-5.6511855	7.9544001	104.1599960
-5.6511855	8.0032005	101.9199982
-5.6511855	8.0520000	102.3300018
-5.6511855	8.1008005	101.2399979
-5.6511855	8.1496000	99.7699966
-5.6511855	8.1984005	101.1299973
-5.6511855	8.2472000	99.8600006
-5.6511855	8.2960005	104.2600021
-5.6511855	8.3448000	101.7299957
-5.6511855	8.3936005	104.8699951
-5.6511855	8.4424000	104.8199997
-5.6511855	8.4912004	104.4399948
-5.6511855	8.5400000	105.2600021
-5.6511855	8.5888004	104.4799957
-5.6511855	8.6375999	102.4000015
-5.6511855	8.6864004	106.4399948
-5.6511855	8.7351999	106.6699982
-5.6511855	8.7840004	104.6899948
-5.6511855	8.8327999	104.1999969
-5.6511855	8.8816004	100.3199997
-5.6511855	8.9303999	98.9099960
-5.6511855	8.9792004	97.9899979
-5.6511855	9.0279999	105.8099976
-5.6511855	9.0768003	106.7799988
-5.6511855	9.1255999	105.3199997
-5.6511855	9.1744003	105.3899994
-5.6511855	9.2231998	103.4399948
-5.6511855	9.2720003	101.4700012
-5.6511855	9.3207998	99.2900009
-5.6511855	9.3696003	105.4499969
-5.6511855	9.4184008	106.1299973
-5.6511855	9.4672003	104.4700012
-5.6511855	9.5160007	103.5800018
-5.6511855	9.5648003	104.0199966
-5.6511855	9.6136007	105.3899994
-5.6511855	9.6624002	105.0000000
-5.6511855	9.7112007	102.8699951
-5.6511855	9.7600002	102.7200012
-5.6511855	9.8088007	103.4700012
-5.6511855	9.8576002	105.0299988
-5.6511855	9.9064007	105.6699982
-5.6511855	9.9552002	104.2999954
-5.6511855	10.0040007	105.0699997
-5.6511855	10.0528002	104.3399963
-5.6511855	10.1016006	101.8199997
-5.6511855	10.1504002	102.7699966
-5.6511855	10.1992006	103.7299957
-5.6511855	10.2480001	104.6899948
-5.6511855	10.2968006	104.3499985
-5.6511855	10.3456001	103.4199982
-5.6511855	10.3944006	101.1800003
-5.6511855	10.4432001	102.6999969
-5.6511855	10.4920006	105.0100021
-5.6511855	10.5408001	105.1899948
-5.6511855	10.5896006	103.3499985
-5.6511855	10.6384001	104.2699966
-5.6511855	10.6872005	101.0199966
-5.6511855	10.7360001	102.1399994

-5.6511855	10.7848005	102.0100021
-5.6511855	10.8336000	102.3099976
-5.6511855	10.8824005	104.2900009
-5.6511855	10.9312000	104.4300003
-5.6511855	10.9800005	102.7799988
-5.6511855	11.0288000	99.9000015
-5.6511855	11.0776005	101.0400009
-5.6511855	11.1264000	101.6599960
-5.6511855	11.1752005	100.4300003
-5.6511855	11.2240000	101.5699997
-5.6511855	11.2728004	100.4499969
-5.6511855	11.3216000	102.5000000
-5.6511855	11.3704004	105.2299957
-5.6511855	11.4191999	106.6599960
-5.6511855	11.4680004	103.6899948
-5.6511855	11.5167999	103.6999969
-5.6511855	11.5656004	102.1599960
-5.6511855	11.6143999	102.5400009
-5.6511855	11.6632004	104.5400009
-5.6511855	11.7119999	104.9899979
-5.6511855	11.7608004	103.8699951
-5.6511855	11.8095999	106.0400009
-5.6511855	11.8584003	105.7600021
-5.6511855	11.9071999	103.6899948
-5.6511855	11.9560003	101.2799988
-5.6511855	12.0047998	95.8600006
-5.6511855	12.0536003	103.8300018
-5.6511855	12.1023998	102.6299973
-5.6511855	12.1512003	102.9199982
-5.6511855	12.1999998	98.7299957
-5.6511855	12.2488003	101.6500015
-5.6511855	12.2975998	101.4599991
-5.6511855	12.3464003	101.9599991
-5.6511855	12.3951998	104.7099991
-5.6511855	12.4440002	104.7500000
-5.6511855	12.4927998	103.2399979
-5.6511855	12.5416002	103.1899948
-5.6511855	12.5904007	102.6999969
-5.6511855	12.6392002	104.9899979
-5.6511855	12.6880007	104.5499954
-5.6511855	12.7368002	104.1899948
-5.6511855	12.7856007	105.3600006
-5.6511855	12.8344002	104.1599960
-5.6511855	12.8832006	103.5800018
-5.6511855	12.9320002	104.2200012
-5.6511855	12.9808006	106.2500000
-5.6511855	13.0296001	103.6399994
-5.6511855	13.0784006	104.5000000
-5.6511855	13.1272001	101.1299973
-5.6511855	13.1760006	103.2500000
-5.6511855	13.2248001	103.8799973
-5.6511855	13.2736006	103.2699966
-5.6511855	13.3224001	101.5199966
-5.6511855	13.3712006	105.0199966
-5.6511855	13.4200001	105.3399963
-5.6511855	13.4688005	102.7999954
-5.6511855	13.5176001	103.0299988
-5.6511855	13.5664005	104.7900009

-5.6511855	13.6152000	104.6699982
-5.6511855	13.6640005	104.0499954
-5.6511855	13.7128000	102.3099976
-5.6511855	13.7616005	103.5599976
-5.6511855	13.8104000	105.0000000
-5.6511855	13.8592005	101.0999985
-5.6511855	13.9080000	103.8600006
-5.6511855	13.9568005	107.1699982
-5.6511855	14.0056000	103.9599991
-5.6511855	14.0544004	103.0100021
-5.6511855	14.1032000	105.2799988
-5.6511855	14.1520004	98.8799973
-5.6511855	14.2007999	103.7600021
-5.6511855	14.2496004	102.9099960
-5.6511855	14.2983999	104.2799988
-5.6511855	14.3472004	104.5800018
-5.6511855	14.3959999	101.8099976
-5.6511855	14.4448004	101.8799973
-5.6511855	14.4935999	102.9000015
-5.6511855	14.5424004	102.7399979
-5.6511855	14.5911999	102.2999954
-5.6511855	14.6400003	102.0800018
-5.6511855	14.6887999	102.7299957
-5.6511855	14.7376003	104.5299988
-5.6511855	14.7863998	104.0299988
-5.6511855	14.8352003	104.0899963
-5.6511855	14.8839998	102.6399994
-5.6511855	14.9328003	103.4799957
-5.6511855	14.9815998	103.1599960
-5.6511855	15.0304003	102.4700012
-5.6450496	0.3416000	106.4899979
-5.6450496	0.3904000	107.3099976
-5.6450496	0.4392000	106.3600006
-5.6450496	0.4880000	105.6500015
-5.6450496	0.5368000	99.7099991
-5.6450496	0.5856000	105.4899979
-5.6450496	0.6344000	106.4300003
-5.6450496	0.6832000	106.6500015
-5.6450496	0.7320000	102.2799988
-5.6450496	0.7808000	105.9700012
-5.6450496	0.8296000	105.8799973
-5.6450496	0.8784000	104.9000015
-5.6450496	0.9272000	106.5999985
-5.6450496	0.9760000	106.3499985
-5.6450496	1.0247999	103.5499954
-5.6450496	1.0735999	98.2200012
-5.6450496	1.1223999	100.7999954
-5.6450496	1.1711999	102.7399979
-5.6450496	1.2200000	104.4700012
-5.6450496	1.2688000	102.4199982
-5.6450496	1.3176000	103.1399994
-5.6450496	1.3664000	105.1100006
-5.6450496	1.4152000	108.4700012
-5.6450496	1.4640000	111.2500000
-5.6450496	1.5128000	111.4300003
-5.6450496	1.5616000	109.7200012
-5.6450496	1.6104000	108.4700012
-5.6450496	1.6592000	109.2699966

-5.6450496	1.7079999	108.1100006
-5.6450496	1.7567999	106.3199997
-5.6450496	1.8055999	106.4799957
-5.6450496	1.8543999	107.2900009
-5.6450496	1.9031999	109.5499954
-5.6450496	1.9519999	106.9599991
-5.6450496	2.0007999	102.6899948
-5.6450496	2.0495999	102.5299988
-5.6450496	2.0983999	103.8199997
-5.6450496	2.1471999	103.1500015
-5.6450496	2.1959999	100.1999969
-5.6450496	2.2447999	102.0800018
-5.6450496	2.2935998	103.4599991
-5.6450496	2.3423998	104.3199997
-5.6450496	2.3912001	104.2200012
-5.6450496	2.4400001	104.7900009
-5.6450496	2.4888000	106.9700012
-5.6450496	2.5376000	103.6599960
-5.6450496	2.5864000	100.5100021
-5.6450496	2.6352000	103.1399994
-5.6450496	2.6840000	106.3399963
-5.6450496	2.7328000	105.6299973
-5.6450496	2.7816000	105.0400009
-5.6450496	2.8304000	106.2799988
-5.6450496	2.8792000	105.0899963
-5.6450496	2.9280000	104.1500015
-5.6450496	2.9768000	107.0299988
-5.6450496	3.0256000	109.5999985
-5.6450496	3.0743999	108.9799957
-5.6450496	3.1231999	105.0899963
-5.6450496	3.1719999	105.7399979
-5.6450496	3.2207999	106.2399979
-5.6450496	3.2695999	105.7999954
-5.6450496	3.3183999	104.0699997
-5.6450496	3.3671999	102.9700012
-5.6450496	3.4159999	104.2299957
-5.6450496	3.4647999	102.1500015
-5.6450496	3.5135999	104.1899948
-5.6450496	3.5623999	103.6100006
-5.6450496	3.6111999	103.4099960
-5.6450496	3.6599998	103.8199997
-5.6450496	3.7087998	104.2299957
-5.6450496	3.7575998	103.7500000
-5.6450496	3.8063998	104.5899963
-5.6450496	3.8551998	104.1100006
-5.6450496	3.9039998	101.0599976
-5.6450496	3.9527998	104.9399948
-5.6450496	4.0016003	105.4599991
-5.6450496	4.0503998	103.2600021
-5.6450496	4.0991998	104.0000000
-5.6450496	4.1479998	107.3199997
-5.6450496	4.1967998	105.3499985
-5.6450496	4.2455997	105.9499969
-5.6450496	4.2943997	105.7799988
-5.6450496	4.3431997	107.6100006
-5.6450496	4.3919997	106.3300018
-5.6450496	4.4407997	103.3600006
-5.6450496	4.4895997	103.9300003

-5.6450496	4.5383997	108.4199982
-5.6450496	4.5871997	108.5800018
-5.6450496	4.6359997	110.4000015
-5.6450496	4.6847997	112.0299988
-5.6450496	4.7336001	110.0899963
-5.6450496	4.7824001	108.0100021
-5.6450496	4.8312001	104.8899994
-5.6450496	4.8800001	107.3499985
-5.6450496	4.9288001	106.8600006
-5.6450496	4.9776001	105.0899963
-5.6450496	5.0264001	105.2799988
-5.6450496	5.0752001	104.7200012
-5.6450496	5.1240001	99.0899963
-5.6450496	5.1728001	98.9499969
-5.6450496	5.2216001	100.7600021
-5.6450496	5.2704000	102.5400009
-5.6450496	5.3192000	99.2399979
-5.6450496	5.3680000	104.5199966
-5.6450496	5.4168000	106.8399963
-5.6450496	5.4656000	104.7699966
-5.6450496	5.5144000	102.8099976
-5.6450496	5.5632000	102.7799988
-5.6450496	5.6120000	103.7200012
-5.6450496	5.6608000	100.8099976
-5.6450496	5.7096000	102.1599960
-5.6450496	5.7584000	104.1599960
-5.6450496	5.8072000	104.0999985
-5.6450496	5.8559999	102.9099960
-5.6450496	5.9047999	102.3600006
-5.6450496	5.9535999	103.3699951
-5.6450496	6.0023999	102.5299988
-5.6450496	6.0511999	100.3099976
-5.6450496	6.0999999	103.1800003
-5.6450496	6.1487999	102.3799973
-5.6450496	6.1975999	104.5400009
-5.6450496	6.2463999	104.1100006
-5.6450496	6.2951999	104.3699951
-5.6450496	6.3439999	104.4700012
-5.6450496	6.3927999	104.9899979
-5.6450496	6.4415998	104.0299988
-5.6450496	6.4903998	99.1199951
-5.6450496	6.5391998	100.5899963
-5.6450496	6.5879998	102.7099991
-5.6450496	6.6367998	104.9399948
-5.6450496	6.6855998	101.7500000
-5.6450496	6.7343998	102.5800018
-5.6450496	6.7831998	102.3600006
-5.6450496	6.8319998	100.5100021
-5.6450496	6.8807998	100.7799988
-5.6450496	6.9295998	102.8300018
-5.6450496	6.9783998	104.4799957
-5.6450496	7.0271997	102.0899963
-5.6450496	7.0759997	104.1199951
-5.6450496	7.1247997	104.4399948
-5.6450496	7.1735997	104.1899948
-5.6450496	7.2223997	106.5000000
-5.6450496	7.2711997	106.6599960
-5.6450496	7.3199997	100.6399994

-5.6450496	7.3687997	101.8399963
-5.6450496	7.4175997	101.5199966
-5.6450496	7.4663997	102.2799988
-5.6450496	7.5151997	104.9300003
-5.6450496	7.5639997	105.9000015
-5.6450496	7.6127996	105.7600021
-5.6450496	7.6615996	105.4700012
-5.6450496	7.7103996	104.5800018
-5.6450496	7.7591996	105.8699951
-5.6450496	7.8079996	105.5499954
-5.6450496	7.8567996	104.8399963
-5.6450496	7.9056001	103.2799988
-5.6450496	7.9544001	103.7399979
-5.6450496	8.0032005	102.2200012
-5.6450496	8.0520000	102.5400009
-5.6450496	8.1008005	101.1299973
-5.6450496	8.1496000	98.9799957
-5.6450496	8.1984005	101.2600021
-5.6450496	8.2472000	101.1500015
-5.6450496	8.2960005	104.2900009
-5.6450496	8.3448000	101.2399979
-5.6450496	8.3936005	104.5999985
-5.6450496	8.4424000	104.4599991
-5.6450496	8.4912004	103.6599960
-5.6450496	8.5400000	105.4399948
-5.6450496	8.5888004	105.0299988
-5.6450496	8.6375999	103.2900009
-5.6450496	8.6864004	107.1199951
-5.6450496	8.7351999	106.5000000
-5.6450496	8.7840004	104.4399948
-5.6450496	8.8327999	103.2200012
-5.6450496	8.8816004	98.4799957
-5.6450496	8.9303999	97.1199951
-5.6450496	8.9792004	98.5599976
-5.6450496	9.0279999	104.5000000
-5.6450496	9.0768003	106.6299973
-5.6450496	9.1255999	104.3199997
-5.6450496	9.1744003	106.2600021
-5.6450496	9.2231998	105.1800003
-5.6450496	9.2720003	102.0999985
-5.6450496	9.3207998	100.7600021
-5.6450496	9.3696003	104.8499985
-5.6450496	9.4184008	105.3499985
-5.6450496	9.4672003	103.9300003
-5.6450496	9.5160007	102.9300003
-5.6450496	9.5648003	103.9000015
-5.6450496	9.6136007	105.1599960
-5.6450496	9.6624002	104.7399979
-5.6450496	9.7112007	103.2399979
-5.6450496	9.7600002	102.8600006
-5.6450496	9.8088007	103.4300003
-5.6450496	9.8576002	103.8699951
-5.6450496	9.9064007	105.3099976
-5.6450496	9.9552002	104.6800003
-5.6450496	10.0040007	104.9000015
-5.6450496	10.0528002	103.9399948
-5.6450496	10.1016006	100.8899994
-5.6450496	10.1504002	101.8600006

-5.6450496	10.1992006	103.3699951
-5.6450496	10.2480001	104.4599991
-5.6450496	10.2968006	104.5499954
-5.6450496	10.3456001	103.9700012
-5.6450496	10.3944006	101.9300003
-5.6450496	10.4432001	104.0599976
-5.6450496	10.4920006	105.8699951
-5.6450496	10.5408001	106.0100021
-5.6450496	10.5896006	103.7299957
-5.6450496	10.6384001	104.9099960
-5.6450496	10.6872005	101.5299988
-5.6450496	10.7360001	102.9199982
-5.6450496	10.7848005	102.4099960
-5.6450496	10.8336000	102.3699951
-5.6450496	10.8824005	104.5000000
-5.6450496	10.9312000	103.7799988
-5.6450496	10.9800005	102.0800018
-5.6450496	11.0288000	101.4199982
-5.6450496	11.0776005	101.9399948
-5.6450496	11.1264000	101.1899948
-5.6450496	11.1752005	101.4199982
-5.6450496	11.2240000	103.0800018
-5.6450496	11.2728004	101.0299988
-5.6450496	11.3216000	102.6199951
-5.6450496	11.3704004	105.9199982
-5.6450496	11.4191999	106.7399979
-5.6450496	11.4680004	103.8300018
-5.6450496	11.5167999	103.9199982
-5.6450496	11.5656004	102.4000015
-5.6450496	11.6143999	102.1500015
-5.6450496	11.6632004	103.9599991
-5.6450496	11.7119999	104.8199997
-5.6450496	11.7608004	104.1299973
-5.6450496	11.8095999	105.1599960
-5.6450496	11.8584003	105.0000000
-5.6450496	11.9071999	103.1299973
-5.6450496	11.9560003	101.1500015
-5.6450496	12.0047998	96.3199997
-5.6450496	12.0536003	104.4000015
-5.6450496	12.1023998	103.7999954
-5.6450496	12.1512003	103.8899994
-5.6450496	12.1999998	100.0699997
-5.6450496	12.2488003	102.9499969
-5.6450496	12.2975998	102.4599991
-5.6450496	12.3464003	103.4700012
-5.6450496	12.3951998	105.1100006
-5.6450496	12.4440002	103.8799973
-5.6450496	12.4927998	101.6800003
-5.6450496	12.5416002	102.7200012
-5.6450496	12.5904007	103.0599976
-5.6450496	12.6392002	105.6299973
-5.6450496	12.6880007	105.4199982
-5.6450496	12.7368002	104.8399963
-5.6450496	12.7856007	105.8399963
-5.6450496	12.8344002	104.5100021
-5.6450496	12.8832006	103.7200012
-5.6450496	12.9320002	104.9899979
-5.6450496	12.9808006	106.2200012

-5.6450496	13.0296001	102.9899979
-5.6450496	13.0784006	104.6500015
-5.6450496	13.1272001	100.9000015
-5.6450496	13.1760006	103.4799957
-5.6450496	13.2248001	104.2200012
-5.6450496	13.2736006	103.1999969
-5.6450496	13.3224001	100.9799957
-5.6450496	13.3712006	105.1699982
-5.6450496	13.4200001	105.7299957
-5.6450496	13.4688005	104.0000000
-5.6450496	13.5176001	102.7999954
-5.6450496	13.5664005	103.6899948
-5.6450496	13.6152000	103.9599991
-5.6450496	13.6640005	103.5599976
-5.6450496	13.7128000	102.2399979
-5.6450496	13.7616005	103.3199997
-5.6450496	13.8104000	104.1399994
-5.6450496	13.8592005	101.1299973
-5.6450496	13.9080000	103.7699966
-5.6450496	13.9568005	106.8799973
-5.6450496	14.0056000	104.6800003
-5.6450496	14.0544004	102.6199951
-5.6450496	14.1032000	105.7500000
-5.6450496	14.1520004	101.2900009
-5.6450496	14.2007999	104.0999985
-5.6450496	14.2496004	103.5899963
-5.6450496	14.2983999	104.5499954
-5.6450496	14.3472004	104.2500000
-5.6450496	14.3959999	100.2900009
-5.6450496	14.4448004	101.6100006
-5.6450496	14.4935999	102.8399963
-5.6450496	14.5424004	102.7900009
-5.6450496	14.5911999	102.6800003
-5.6450496	14.6400003	102.1999969
-5.6450496	14.6887999	102.7299957
-5.6450496	14.7376003	104.2999954
-5.6450496	14.7863998	102.8300018
-5.6450496	14.8352003	103.9199982
-5.6450496	14.8839998	102.0000000
-5.6450496	14.9328003	103.3499985
-5.6450496	14.9815998	103.0100021
-5.6450496	15.0304003	102.7099991
-5.6389136	0.3416000	106.5899963
-5.6389136	0.3904000	108.2099991
-5.6389136	0.4392000	105.6899948
-5.6389136	0.4880000	105.2799988
-5.6389136	0.5368000	99.6800003
-5.6389136	0.5856000	106.2600021
-5.6389136	0.6344000	108.0100021
-5.6389136	0.6832000	108.1599960
-5.6389136	0.7320000	101.5100021
-5.6389136	0.7808000	106.1699982
-5.6389136	0.8296000	104.4199982
-5.6389136	0.8784000	103.1199951
-5.6389136	0.9272000	105.0299988
-5.6389136	0.9760000	106.2399979
-5.6389136	1.0247999	103.4000015
-5.6389136	1.0735999	97.5999985

-5.6389136	1.1223999	101.2299957
-5.6389136	1.1711999	103.7200012
-5.6389136	1.2200000	105.3699951
-5.6389136	1.2688000	102.6199951
-5.6389136	1.3176000	102.9700012
-5.6389136	1.3664000	105.2999954
-5.6389136	1.4152000	108.7200012
-5.6389136	1.4640000	111.1999969
-5.6389136	1.5128000	110.9700012
-5.6389136	1.5616000	108.7600021
-5.6389136	1.6104000	107.5299988
-5.6389136	1.6592000	109.0800018
-5.6389136	1.7079999	108.5000000
-5.6389136	1.7567999	106.9000015
-5.6389136	1.8055999	107.5100021
-5.6389136	1.8543999	108.7200012
-5.6389136	1.9031999	110.3399963
-5.6389136	1.9519999	107.2399979
-5.6389136	2.0007999	104.6500015
-5.6389136	2.0495999	104.6100006
-5.6389136	2.0983999	104.5499954
-5.6389136	2.1471999	103.9399948
-5.6389136	2.1959999	100.6500015
-5.6389136	2.2447999	102.6599960
-5.6389136	2.2935998	103.7200012
-5.6389136	2.3423998	104.9099960
-5.6389136	2.3912001	104.7399979
-5.6389136	2.4400001	105.6500015
-5.6389136	2.4888000	107.0299988
-5.6389136	2.5376000	102.9599991
-5.6389136	2.5864000	99.5000000
-5.6389136	2.6352000	102.9199982
-5.6389136	2.6840000	106.1500015
-5.6389136	2.7328000	105.7600021
-5.6389136	2.7816000	105.7900009
-5.6389136	2.8304000	106.4599991
-5.6389136	2.8792000	104.7999954
-5.6389136	2.9280000	104.5999985
-5.6389136	2.9768000	107.4899979
-5.6389136	3.0256000	109.8799973
-5.6389136	3.0743999	109.2299957
-5.6389136	3.1231999	105.0199966
-5.6389136	3.1719999	105.9499969
-5.6389136	3.2207999	106.9199982
-5.6389136	3.2695999	106.7099991
-5.6389136	3.3183999	104.3199997
-5.6389136	3.3671999	102.3899994
-5.6389136	3.4159999	103.6199951
-5.6389136	3.4647999	102.1999969
-5.6389136	3.5135999	104.2799988
-5.6389136	3.5623999	103.8099976
-5.6389136	3.6111999	103.7299957
-5.6389136	3.6599998	103.6199951
-5.6389136	3.7087998	104.3600006
-5.6389136	3.7575998	104.2600021
-5.6389136	3.8063998	104.6100006
-5.6389136	3.8551998	104.4899979
-5.6389136	3.9039998	101.9399948

-5.6389136	3.9527998	104.9199982
-5.6389136	4.0016003	105.1500015
-5.6389136	4.0503998	102.7299957
-5.6389136	4.0991998	103.6399994
-5.6389136	4.1479998	106.5100021
-5.6389136	4.1967998	103.6100006
-5.6389136	4.2455997	105.2900009
-5.6389136	4.2943997	105.9000015
-5.6389136	4.3431997	107.4199982
-5.6389136	4.3919997	106.0299988
-5.6389136	4.4407997	103.9599991
-5.6389136	4.4895997	103.5100021
-5.6389136	4.5383997	108.6599960
-5.6389136	4.5871997	109.0299988
-5.6389136	4.6359997	110.7399979
-5.6389136	4.6847997	112.1599960
-5.6389136	4.7336001	110.3899994
-5.6389136	4.7824001	108.4700012
-5.6389136	4.8312001	104.7500000
-5.6389136	4.8800001	107.5899963
-5.6389136	4.9288001	106.9499969
-5.6389136	4.9776001	105.5199966
-5.6389136	5.0264001	105.9700012
-5.6389136	5.0752001	105.1599960
-5.6389136	5.1240001	100.6800003
-5.6389136	5.1728001	99.7399979
-5.6389136	5.2216001	101.1899948
-5.6389136	5.2704000	102.5499954
-5.6389136	5.3192000	99.8899994
-5.6389136	5.3680000	105.0199966
-5.6389136	5.4168000	106.8899994
-5.6389136	5.4656000	105.2299957
-5.6389136	5.5144000	103.2399979
-5.6389136	5.5632000	102.7399979
-5.6389136	5.6120000	103.7500000
-5.6389136	5.6608000	100.7600021
-5.6389136	5.7096000	101.1100006
-5.6389136	5.7584000	103.7600021
-5.6389136	5.8072000	103.5800018
-5.6389136	5.8559999	101.8499985
-5.6389136	5.9047999	101.5800018
-5.6389136	5.9535999	102.3399963
-5.6389136	6.0023999	102.1899948
-5.6389136	6.0511999	100.2500000
-5.6389136	6.0999999	102.4499969
-5.6389136	6.1487999	101.5400009
-5.6389136	6.1975999	104.5699997
-5.6389136	6.2463999	103.8399963
-5.6389136	6.2951999	103.9199982
-5.6389136	6.3439999	104.4499969
-5.6389136	6.3927999	104.2799988
-5.6389136	6.4415998	103.2099991
-5.6389136	6.4903998	99.7600021
-5.6389136	6.5391998	101.2099991
-5.6389136	6.5879998	102.9300003
-5.6389136	6.6367998	104.6699982
-5.6389136	6.6855998	101.1299973
-5.6389136	6.7343998	102.8399963

-5.6389136	6.7831998	102.3199997
-5.6389136	6.8319998	100.8099976
-5.6389136	6.8807998	102.0999985
-5.6389136	6.9295998	104.4300003
-5.6389136	6.9783998	105.5999985
-5.6389136	7.0271997	103.7299957
-5.6389136	7.0759997	104.4300003
-5.6389136	7.1247997	103.4199982
-5.6389136	7.1735997	104.3199997
-5.6389136	7.2223997	105.9199982
-5.6389136	7.2711997	106.7999954
-5.6389136	7.3199997	101.4000015
-5.6389136	7.3687997	101.8199997
-5.6389136	7.4175997	101.1899948
-5.6389136	7.4663997	102.0400009
-5.6389136	7.5151997	104.6800003
-5.6389136	7.5639997	105.0199966
-5.6389136	7.6127996	105.5599976
-5.6389136	7.6615996	105.8600006
-5.6389136	7.7103996	104.9799957
-5.6389136	7.7591996	106.1699982
-5.6389136	7.8079996	105.7099991
-5.6389136	7.8567996	104.2799988
-5.6389136	7.9056001	103.3699951
-5.6389136	7.9544001	103.9700012
-5.6389136	8.0032005	102.9399948
-5.6389136	8.0520000	102.4199982
-5.6389136	8.1008005	101.3699951
-5.6389136	8.1496000	99.1199951
-5.6389136	8.1984005	101.4399948
-5.6389136	8.2472000	102.1100006
-5.6389136	8.2960005	104.6500015
-5.6389136	8.3448000	102.6999969
-5.6389136	8.3936005	105.0199966
-5.6389136	8.4424000	104.8300018
-5.6389136	8.4912004	103.4300003
-5.6389136	8.5400000	105.4700012
-5.6389136	8.5888004	105.7999954
-5.6389136	8.6375999	104.2500000
-5.6389136	8.6864004	107.0100021
-5.6389136	8.7351999	106.2500000
-5.6389136	8.7840004	104.6399994
-5.6389136	8.8327999	102.0199966
-5.6389136	8.8816004	96.7500000
-5.6389136	8.9303999	97.2200012
-5.6389136	8.9792004	99.1999969
-5.6389136	9.0279999	102.7900009
-5.6389136	9.0768003	106.1100006
-5.6389136	9.1255999	103.8699951
-5.6389136	9.1744003	107.0000000
-5.6389136	9.2231998	106.3099976
-5.6389136	9.2720003	102.2799988
-5.6389136	9.3207998	101.4899979
-5.6389136	9.3696003	103.7799988
-5.6389136	9.4184008	103.9700012
-5.6389136	9.4672003	103.4000015
-5.6389136	9.5160007	102.9799957
-5.6389136	9.5648003	103.6899948

-5.6389136	9.6136007	104.8499985
-5.6389136	9.6624002	104.5800018
-5.6389136	9.7112007	103.7299957
-5.6389136	9.7600002	102.8799973
-5.6389136	9.8088007	103.4000015
-5.6389136	9.8576002	102.5899963
-5.6389136	9.9064007	104.6199951
-5.6389136	9.9552002	104.7299957
-5.6389136	10.0040007	104.3399963
-5.6389136	10.0528002	103.1100006
-5.6389136	10.1016006	101.0299988
-5.6389136	10.1504002	101.6500015
-5.6389136	10.1992006	103.8199997
-5.6389136	10.2480001	104.4899979
-5.6389136	10.2968006	104.5899963
-5.6389136	10.3456001	104.4099960
-5.6389136	10.3944006	102.8499985
-5.6389136	10.4432001	104.3399963
-5.6389136	10.4920006	105.7999954
-5.6389136	10.5408001	106.1599960
-5.6389136	10.5896006	104.5999985
-5.6389136	10.6384001	104.9599991
-5.6389136	10.6872005	101.3399963
-5.6389136	10.7360001	103.2900009
-5.6389136	10.7848005	103.2500000
-5.6389136	10.8336000	103.0499954
-5.6389136	10.8824005	105.1999969
-5.6389136	10.9312000	103.9700012
-5.6389136	10.9800005	102.8600006
-5.6389136	11.0288000	103.3099976
-5.6389136	11.0776005	102.3499985
-5.6389136	11.1264000	100.8099976
-5.6389136	11.1752005	101.9399948
-5.6389136	11.2240000	104.3499985
-5.6389136	11.2728004	101.3099976
-5.6389136	11.3216000	102.7999954
-5.6389136	11.3704004	106.5599976
-5.6389136	11.4191999	106.7600021
-5.6389136	11.4680004	103.9799957
-5.6389136	11.5167999	104.2200012
-5.6389136	11.5656004	103.1199951
-5.6389136	11.6143999	102.0299988
-5.6389136	11.6632004	102.8499985
-5.6389136	11.7119999	104.5499954
-5.6389136	11.7608004	104.7200012
-5.6389136	11.8095999	104.5400009
-5.6389136	11.8584003	104.7399979
-5.6389136	11.9071999	103.2299957
-5.6389136	11.9560003	101.8399963
-5.6389136	12.0047998	98.2699966
-5.6389136	12.0536003	105.1199951
-5.6389136	12.1023998	104.3799973
-5.6389136	12.1512003	103.4399948
-5.6389136	12.1999998	100.1999969
-5.6389136	12.2488003	103.7699966
-5.6389136	12.2975998	103.8399963
-5.6389136	12.3464003	105.1699982
-5.6389136	12.3951998	105.3899994

-5.6389136	12.4440002	102.9000015
-5.6389136	12.4927998	100.5800018
-5.6389136	12.5416002	101.8600006
-5.6389136	12.5904007	103.1100006
-5.6389136	12.6392002	105.6299973
-5.6389136	12.6880007	105.6399994
-5.6389136	12.7368002	104.7200012
-5.6389136	12.7856007	105.9499969
-5.6389136	12.8344002	105.1199951
-5.6389136	12.8832006	103.8899994
-5.6389136	12.9320002	105.3399963
-5.6389136	12.9808006	106.2099991
-5.6389136	13.0296001	103.9499969
-5.6389136	13.0784006	105.3399963
-5.6389136	13.1272001	100.4300003
-5.6389136	13.1760006	103.9000015
-5.6389136	13.2248001	104.9300003
-5.6389136	13.2736006	103.3099976
-5.6389136	13.3224001	99.4099960
-5.6389136	13.3712006	104.5199966
-5.6389136	13.4200001	105.5100021
-5.6389136	13.4688005	104.2699966
-5.6389136	13.5176001	102.4199982
-5.6389136	13.5664005	103.1199951
-5.6389136	13.6152000	103.7699966
-5.6389136	13.6640005	102.6999969
-5.6389136	13.7128000	101.6100006
-5.6389136	13.7616005	102.6599960
-5.6389136	13.8104000	103.2500000
-5.6389136	13.8592005	102.0000000
-5.6389136	13.9080000	103.4899979
-5.6389136	13.9568005	106.2099991
-5.6389136	14.0056000	104.8099976
-5.6389136	14.0544004	101.7200012
-5.6389136	14.1032000	105.6999969
-5.6389136	14.1520004	103.3300018
-5.6389136	14.2007999	104.6199951
-5.6389136	14.2496004	104.3699951
-5.6389136	14.2983999	105.0599976
-5.6389136	14.3472004	104.4099960
-5.6389136	14.3959999	99.5999985
-5.6389136	14.4448004	101.6199951
-5.6389136	14.4935999	102.7999954
-5.6389136	14.5424004	102.7799988
-5.6389136	14.5911999	102.8199997
-5.6389136	14.6400003	102.7799988
-5.6389136	14.6887999	103.0100021
-5.6389136	14.7376003	104.4099960
-5.6389136	14.7863998	101.9499969
-5.6389136	14.8352003	104.1500015
-5.6389136	14.8839998	102.6199951
-5.6389136	14.9328003	103.2500000
-5.6389136	14.9815998	102.0499954
-5.6389136	15.0304003	102.5499954
-5.6327777	0.3416000	106.6399994
-5.6327777	0.3904000	108.3799973
-5.6327777	0.4392000	105.5999985
-5.6327777	0.4880000	105.0699997

-5.6327777	0.5368000	99.9700012
-5.6327777	0.5856000	107.1100006
-5.6327777	0.6344000	109.0400009
-5.6327777	0.6832000	108.2699966
-5.6327777	0.7320000	99.8899994
-5.6327777	0.7808000	105.9199982
-5.6327777	0.8296000	102.6999969
-5.6327777	0.8784000	102.1100006
-5.6327777	0.9272000	102.7699966
-5.6327777	0.9760000	105.3600006
-5.6327777	1.0247999	103.5800018
-5.6327777	1.0735999	98.2699966
-5.6327777	1.1223999	101.4000015
-5.6327777	1.1711999	104.5299988
-5.6327777	1.2200000	106.0000000
-5.6327777	1.2688000	102.7900009
-5.6327777	1.3176000	102.9000015
-5.6327777	1.3664000	105.4899979
-5.6327777	1.4152000	108.9399948
-5.6327777	1.4640000	110.0000000
-5.6327777	1.5128000	110.5000000
-5.6327777	1.5616000	107.8799973
-5.6327777	1.6104000	106.0899963
-5.6327777	1.6592000	108.6699982
-5.6327777	1.7079999	108.7399979
-5.6327777	1.7567999	107.2600021
-5.6327777	1.8055999	107.9599991
-5.6327777	1.8543999	109.7299957
-5.6327777	1.9031999	110.9300003
-5.6327777	1.9519999	107.3799973
-5.6327777	2.0007999	105.6999969
-5.6327777	2.0495999	105.6699982
-5.6327777	2.0983999	105.1899948
-5.6327777	2.1471999	104.6999969
-5.6327777	2.1959999	100.5100021
-5.6327777	2.2447999	102.3600006
-5.6327777	2.2935998	103.4399948
-5.6327777	2.3423998	105.9099960
-5.6327777	2.3912001	106.0599976
-5.6327777	2.4400001	106.6699982
-5.6327777	2.4888000	106.6699982
-5.6327777	2.5376000	102.0400009
-5.6327777	2.5864000	98.7299957
-5.6327777	2.6352000	102.7299957
-5.6327777	2.6840000	105.4300003
-5.6327777	2.7328000	105.0400009
-5.6327777	2.7816000	105.3600006
-5.6327777	2.8304000	106.1199951
-5.6327777	2.8792000	104.6399994
-5.6327777	2.9280000	105.9099960
-5.6327777	2.9768000	107.8600006
-5.6327777	3.0256000	109.4599991
-5.6327777	3.0743999	108.5199966
-5.6327777	3.1231999	104.0199966
-5.6327777	3.1719999	105.6599960
-5.6327777	3.2207999	107.0100021
-5.6327777	3.2695999	106.9399948
-5.6327777	3.3183999	104.1199951

-5.6327777	3.3671999	102.1399994
-5.6327777	3.4159999	103.4599991
-5.6327777	3.4647999	102.8499985
-5.6327777	3.5135999	104.6500015
-5.6327777	3.5623999	104.3799973
-5.6327777	3.6111999	104.0899963
-5.6327777	3.6599998	103.4599991
-5.6327777	3.7087998	104.9499969
-5.6327777	3.7575998	105.2099991
-5.6327777	3.8063998	105.1299973
-5.6327777	3.8551998	104.7999954
-5.6327777	3.9039998	102.0400009
-5.6327777	3.9527998	104.7200012
-5.6327777	4.0016003	105.2799988
-5.6327777	4.0503998	102.4199982
-5.6327777	4.0991998	102.4899979
-5.6327777	4.1479998	105.3099976
-5.6327777	4.1967998	101.6899948
-5.6327777	4.2455997	104.8099976
-5.6327777	4.2943997	105.6699982
-5.6327777	4.3431997	107.2799988
-5.6327777	4.3919997	106.1999969
-5.6327777	4.4407997	104.8199997
-5.6327777	4.4895997	104.1899948
-5.6327777	4.5383997	108.3699951
-5.6327777	4.5871997	109.1699982
-5.6327777	4.6359997	110.5499954
-5.6327777	4.6847997	111.6999969
-5.6327777	4.7336001	110.1500015
-5.6327777	4.7824001	108.8799973
-5.6327777	4.8312001	105.6899948
-5.6327777	4.8800001	107.9899979
-5.6327777	4.9288001	107.0999985
-5.6327777	4.9776001	105.6999969
-5.6327777	5.0264001	106.9199982
-5.6327777	5.0752001	105.9199982
-5.6327777	5.1240001	103.0699997
-5.6327777	5.1728001	100.8600006
-5.6327777	5.2216001	102.1199951
-5.6327777	5.2704000	103.1100006
-5.6327777	5.3192000	101.0100021
-5.6327777	5.3680000	105.3099976
-5.6327777	5.4168000	106.6899948
-5.6327777	5.4656000	105.8499985
-5.6327777	5.5144000	104.4499969
-5.6327777	5.5632000	103.4499969
-5.6327777	5.6120000	103.7099991
-5.6327777	5.6608000	100.6800003
-5.6327777	5.7096000	100.2299957
-5.6327777	5.7584000	103.1899948
-5.6327777	5.8072000	103.1399994
-5.6327777	5.8559999	101.0100021
-5.6327777	5.9047999	100.7799988
-5.6327777	5.9535999	101.4300003
-5.6327777	6.0023999	101.9199982
-5.6327777	6.0511999	100.3399963
-5.6327777	6.0999999	102.5499954
-5.6327777	6.1487999	100.9899979

-5.6327777	6.1975999	104.8199997
-5.6327777	6.2463999	103.6299973
-5.6327777	6.2951999	103.1100006
-5.6327777	6.3439999	104.4899979
-5.6327777	6.3927999	103.5599976
-5.6327777	6.4415998	102.1599960
-5.6327777	6.4903998	100.4700012
-5.6327777	6.5391998	101.8899994
-5.6327777	6.5879998	103.0100021
-5.6327777	6.6367998	103.9899979
-5.6327777	6.6855998	100.7399979
-5.6327777	6.7343998	103.6399994
-5.6327777	6.7831998	102.6299973
-5.6327777	6.8319998	101.0100021
-5.6327777	6.8807998	103.1999969
-5.6327777	6.9295998	105.6500015
-5.6327777	6.9783998	106.0499954
-5.6327777	7.0271997	104.8300018
-5.6327777	7.0759997	104.4499969
-5.6327777	7.1247997	102.5400009
-5.6327777	7.1735997	104.6199951
-5.6327777	7.2223997	105.2999954
-5.6327777	7.2711997	106.6199951
-5.6327777	7.3199997	101.9399948
-5.6327777	7.3687997	102.1199951
-5.6327777	7.4175997	101.3399963
-5.6327777	7.4663997	102.3899994
-5.6327777	7.5151997	104.6699982
-5.6327777	7.5639997	104.1199951
-5.6327777	7.6127996	104.7699966
-5.6327777	7.6615996	106.3699951
-5.6327777	7.7103996	105.2200012
-5.6327777	7.7591996	106.1299973
-5.6327777	7.8079996	105.9899979
-5.6327777	7.8567996	103.9300003
-5.6327777	7.9056001	103.3399963
-5.6327777	7.9544001	104.1899948
-5.6327777	8.0032005	103.6899948
-5.6327777	8.0520000	102.2500000
-5.6327777	8.1008005	102.3600006
-5.6327777	8.1496000	99.4300003
-5.6327777	8.1984005	101.5599976
-5.6327777	8.2472000	103.3499985
-5.6327777	8.2960005	105.2699966
-5.6327777	8.3448000	104.0299988
-5.6327777	8.3936005	105.0000000
-5.6327777	8.4424000	104.6500015
-5.6327777	8.4912004	102.7699966
-5.6327777	8.5400000	105.3699951
-5.6327777	8.5888004	106.6399994
-5.6327777	8.6375999	105.0100021
-5.6327777	8.6864004	106.6199951
-5.6327777	8.7351999	105.3699951
-5.6327777	8.7840004	104.4799957
-5.6327777	8.8327999	101.0800018
-5.6327777	8.8816004	96.4099960
-5.6327777	8.9303999	97.5899963
-5.6327777	8.9792004	100.0199966

-5.6327777	9.0279999	101.4899979
-5.6327777	9.0768003	105.5800018
-5.6327777	9.1255999	102.7999954
-5.6327777	9.1744003	107.1199951
-5.6327777	9.2231998	107.2699966
-5.6327777	9.2720003	102.6399994
-5.6327777	9.3207998	101.8399963
-5.6327777	9.3696003	102.6999969
-5.6327777	9.4184008	103.0199966
-5.6327777	9.4672003	103.1999969
-5.6327777	9.5160007	103.5599976
-5.6327777	9.5648003	104.0699997
-5.6327777	9.6136007	104.2999954
-5.6327777	9.6624002	104.2999954
-5.6327777	9.7112007	104.5000000
-5.6327777	9.7600002	103.4499969
-5.6327777	9.8088007	103.4000015
-5.6327777	9.8576002	101.6399994
-5.6327777	9.9064007	104.0499954
-5.6327777	9.9552002	104.4399948
-5.6327777	10.0040007	103.5400009
-5.6327777	10.0528002	102.5800018
-5.6327777	10.1016006	101.0000000
-5.6327777	10.1504002	101.6899948
-5.6327777	10.1992006	104.2999954
-5.6327777	10.2480001	104.2299957
-5.6327777	10.2968006	104.7200012
-5.6327777	10.3456001	104.7099991
-5.6327777	10.3944006	103.3499985
-5.6327777	10.4432001	104.8799973
-5.6327777	10.4920006	106.3399963
-5.6327777	10.5408001	106.5400009
-5.6327777	10.5896006	105.2900009
-5.6327777	10.6384001	104.8799973
-5.6327777	10.6872005	101.2299957
-5.6327777	10.7360001	103.6800003
-5.6327777	10.7848005	104.0299988
-5.6327777	10.8336000	104.1299973
-5.6327777	10.8824005	105.9700012
-5.6327777	10.9312000	103.7399979
-5.6327777	10.9800005	103.6399994
-5.6327777	11.0288000	105.2099991
-5.6327777	11.0776005	103.5299988
-5.6327777	11.1264000	101.0499954
-5.6327777	11.1752005	101.9199982
-5.6327777	11.2240000	104.8899994
-5.6327777	11.2728004	101.4000015
-5.6327777	11.3216000	102.6999969
-5.6327777	11.3704004	106.7099991
-5.6327777	11.4191999	106.0599976
-5.6327777	11.4680004	103.1399994
-5.6327777	11.5167999	103.2099991
-5.6327777	11.5656004	102.7099991
-5.6327777	11.6143999	101.4399948
-5.6327777	11.6632004	102.5699997
-5.6327777	11.7119999	104.3600006
-5.6327777	11.7608004	104.9700012
-5.6327777	11.8095999	104.0100021

-5.6327777	11.8584003	104.3899994
-5.6327777	11.9071999	102.9499969
-5.6327777	11.9560003	102.2699966
-5.6327777	12.0047998	100.5999985
-5.6327777	12.0536003	105.6500015
-5.6327777	12.1023998	104.3899994
-5.6327777	12.1512003	102.2299957
-5.6327777	12.1999998	100.9599991
-5.6327777	12.2488003	104.3499985
-5.6327777	12.2975998	104.7600021
-5.6327777	12.3464003	106.0899963
-5.6327777	12.3951998	105.3899994
-5.6327777	12.4440002	102.1800003
-5.6327777	12.4927998	100.8899994
-5.6327777	12.5416002	101.9499969
-5.6327777	12.5904007	103.3600006
-5.6327777	12.6392002	105.1999969
-5.6327777	12.6880007	105.2799988
-5.6327777	12.7368002	104.1899948
-5.6327777	12.7856007	106.3499985
-5.6327777	12.8344002	105.5400009
-5.6327777	12.8832006	103.6699982
-5.6327777	12.9320002	105.0699997
-5.6327777	12.9808006	105.5999985
-5.6327777	13.0296001	104.6100006
-5.6327777	13.0784006	105.8399963
-5.6327777	13.1272001	101.2900009
-5.6327777	13.1760006	104.2500000
-5.6327777	13.2248001	104.9099960
-5.6327777	13.2736006	102.4000015
-5.6327777	13.3224001	97.3399963
-5.6327777	13.3712006	103.4499969
-5.6327777	13.4200001	104.9499969
-5.6327777	13.4688005	104.3399963
-5.6327777	13.5176001	102.3399963
-5.6327777	13.5664005	102.5000000
-5.6327777	13.6152000	103.7699966
-5.6327777	13.6640005	102.6100006
-5.6327777	13.7128000	100.8699951
-5.6327777	13.7616005	101.5400009
-5.6327777	13.8104000	101.7900009
-5.6327777	13.8592005	102.4899979
-5.6327777	13.9080000	103.2600021
-5.6327777	13.9568005	105.9300003
-5.6327777	14.0056000	105.0899963
-5.6327777	14.0544004	100.8199997
-5.6327777	14.1032000	105.2500000
-5.6327777	14.1520004	105.0599976
-5.6327777	14.2007999	104.1299973
-5.6327777	14.2496004	104.3699951
-5.6327777	14.2983999	105.4899979
-5.6327777	14.3472004	105.0199966
-5.6327777	14.3959999	99.4700012
-5.6327777	14.4448004	101.2600021
-5.6327777	14.4935999	102.3300018
-5.6327777	14.5424004	102.7999954
-5.6327777	14.5911999	102.9099960
-5.6327777	14.6400003	103.2600021

-5.6327777	14.6887999	102.9000015
-5.6327777	14.7376003	103.8799973
-5.6327777	14.7863998	100.8099976
-5.6327777	14.8352003	104.0999985
-5.6327777	14.8839998	103.1500015
-5.6327777	14.9328003	103.5800018
-5.6327777	14.9815998	101.6100006
-5.6327777	15.0304003	102.5400009
-5.6266418	0.3416000	105.9300003
-5.6266418	0.3904000	108.2399979
-5.6266418	0.4392000	106.2699966
-5.6266418	0.4880000	105.7299957
-5.6266418	0.5368000	100.7799988
-5.6266418	0.5856000	108.1800003
-5.6266418	0.6344000	109.8899994
-5.6266418	0.6832000	107.5800018
-5.6266418	0.7320000	100.2200012
-5.6266418	0.7808000	105.6500015
-5.6266418	0.8296000	100.7500000
-5.6266418	0.8784000	101.3799973
-5.6266418	0.9272000	101.5999985
-5.6266418	0.9760000	105.0499954
-5.6266418	1.0247999	104.2999954
-5.6266418	1.0735999	99.5199966
-5.6266418	1.1223999	101.0400009
-5.6266418	1.1711999	105.2099991
-5.6266418	1.2200000	106.7099991
-5.6266418	1.2688000	102.4799957
-5.6266418	1.3176000	103.3799973
-5.6266418	1.3664000	105.6599960
-5.6266418	1.4152000	108.7399979
-5.6266418	1.4640000	108.7399979
-5.6266418	1.5128000	110.0499954
-5.6266418	1.5616000	106.9499969
-5.6266418	1.6104000	105.5199966
-5.6266418	1.6592000	108.5299988
-5.6266418	1.7079999	108.7999954
-5.6266418	1.7567999	107.3199997
-5.6266418	1.8055999	108.1699982
-5.6266418	1.8543999	110.1800003
-5.6266418	1.9031999	110.5899963
-5.6266418	1.9519999	106.9499969
-5.6266418	2.0007999	106.6599960
-5.6266418	2.0495999	107.3300018
-5.6266418	2.0983999	105.8699951
-5.6266418	2.1471999	105.2799988
-5.6266418	2.1959999	100.5100021
-5.6266418	2.2447999	102.2799988
-5.6266418	2.2935998	103.8600006
-5.6266418	2.3423998	106.5199966
-5.6266418	2.3912001	107.0100021
-5.6266418	2.4400001	107.1899948
-5.6266418	2.4888000	105.6599960
-5.6266418	2.5376000	100.9399948
-5.6266418	2.5864000	99.4899979
-5.6266418	2.6352000	102.9000015
-5.6266418	2.6840000	104.7299957
-5.6266418	2.7328000	104.0100021

-5.6266418	2.7816000	104.6500015
-5.6266418	2.8304000	105.7099991
-5.6266418	2.8792000	104.8600006
-5.6266418	2.9280000	107.2299957
-5.6266418	2.9768000	108.5999985
-5.6266418	3.0256000	109.0499954
-5.6266418	3.0743999	107.1399994
-5.6266418	3.1231999	103.0400009
-5.6266418	3.1719999	105.0000000
-5.6266418	3.2207999	106.3300018
-5.6266418	3.2695999	106.1500015
-5.6266418	3.3183999	103.9399948
-5.6266418	3.3671999	102.0100021
-5.6266418	3.4159999	103.3600006
-5.6266418	3.4647999	102.9700012
-5.6266418	3.5135999	104.4700012
-5.6266418	3.5623999	104.3899994
-5.6266418	3.6111999	104.4700012
-5.6266418	3.6599998	104.1699982
-5.6266418	3.7087998	105.1899948
-5.6266418	3.7575998	105.7600021
-5.6266418	3.8063998	105.6899948
-5.6266418	3.8551998	105.2699966
-5.6266418	3.9039998	102.6599960
-5.6266418	3.9527998	104.7200012
-5.6266418	4.0016003	105.2500000
-5.6266418	4.0503998	101.8499985
-5.6266418	4.0991998	101.6100006
-5.6266418	4.1479998	104.0499954
-5.6266418	4.1967998	101.1899948
-5.6266418	4.2455997	103.8300018
-5.6266418	4.2943997	104.7900009
-5.6266418	4.3431997	107.0400009
-5.6266418	4.3919997	106.5499954
-5.6266418	4.4407997	105.5299988
-5.6266418	4.4895997	104.3399963
-5.6266418	4.5383997	107.6199951
-5.6266418	4.5871997	108.9599991
-5.6266418	4.6359997	110.7999954
-5.6266418	4.6847997	112.5999985
-5.6266418	4.7336001	111.2999954
-5.6266418	4.7824001	109.5299988
-5.6266418	4.8312001	106.6100006
-5.6266418	4.8800001	108.3300018
-5.6266418	4.9288001	107.0999985
-5.6266418	4.9776001	105.7799988
-5.6266418	5.0264001	107.5000000
-5.6266418	5.0752001	106.1599960
-5.6266418	5.1240001	104.9599991
-5.6266418	5.1728001	101.2600021
-5.6266418	5.2216001	103.3799973
-5.6266418	5.2704000	103.9799957
-5.6266418	5.3192000	101.9700012
-5.6266418	5.3680000	105.0100021
-5.6266418	5.4168000	105.8499985
-5.6266418	5.4656000	105.4700012
-5.6266418	5.5144000	104.5000000
-5.6266418	5.5632000	103.2799988

-5.6266418	5.6120000	103.2799988
-5.6266418	5.6608000	100.8099976
-5.6266418	5.7096000	99.6500015
-5.6266418	5.7584000	103.2200012
-5.6266418	5.8072000	103.3799973
-5.6266418	5.8559999	100.7999954
-5.6266418	5.9047999	100.4000015
-5.6266418	5.9535999	101.0400009
-5.6266418	6.0023999	101.9099960
-5.6266418	6.0511999	100.1599960
-5.6266418	6.0999999	102.8600006
-5.6266418	6.1487999	101.5800018
-5.6266418	6.1975999	104.5299988
-5.6266418	6.2463999	103.1399994
-5.6266418	6.2951999	102.3799973
-5.6266418	6.3439999	104.5800018
-5.6266418	6.3927999	103.4799957
-5.6266418	6.4415998	101.8099976
-5.6266418	6.4903998	102.2600021
-5.6266418	6.5391998	102.9199982
-5.6266418	6.5879998	102.7299957
-5.6266418	6.6367998	102.9700012
-5.6266418	6.6855998	101.4499969
-5.6266418	6.7343998	103.8600006
-5.6266418	6.7831998	103.1800003
-5.6266418	6.8319998	101.7299957
-5.6266418	6.8807998	103.8499985
-5.6266418	6.9295998	105.5499954
-5.6266418	6.9783998	106.0599976
-5.6266418	7.0271997	105.3600006
-5.6266418	7.0759997	104.2200012
-5.6266418	7.1247997	101.4899979
-5.6266418	7.1735997	103.8799973
-5.6266418	7.2223997	104.3099976
-5.6266418	7.2711997	106.3300018
-5.6266418	7.3199997	103.0599976
-5.6266418	7.3687997	102.1199951
-5.6266418	7.4175997	101.5299988
-5.6266418	7.4663997	103.5000000
-5.6266418	7.5151997	105.2600021
-5.6266418	7.5639997	103.5499954
-5.6266418	7.6127996	103.3199997
-5.6266418	7.6615996	105.4099960
-5.6266418	7.7103996	104.5999985
-5.6266418	7.7591996	105.6299973
-5.6266418	7.8079996	106.0400009
-5.6266418	7.8567996	103.4899979
-5.6266418	7.9056001	103.0699997
-5.6266418	7.9544001	103.8399963
-5.6266418	8.0032005	104.0400009
-5.6266418	8.0520000	102.2600021
-5.6266418	8.1008005	103.0299988
-5.6266418	8.1496000	99.9599991
-5.6266418	8.1984005	101.7299957
-5.6266418	8.2472000	104.5899963
-5.6266418	8.2960005	105.6899948
-5.6266418	8.3448000	105.3399963
-5.6266418	8.3936005	105.2399979

-5.6266418	8.4424000	104.5400009
-5.6266418	8.4912004	103.1299973
-5.6266418	8.5400000	105.5899963
-5.6266418	8.5888004	107.3099976
-5.6266418	8.6375999	106.1399994
-5.6266418	8.6864004	106.3799973
-5.6266418	8.7351999	104.2900009
-5.6266418	8.7840004	104.1299973
-5.6266418	8.8327999	100.1899948
-5.6266418	8.8816004	96.4700012
-5.6266418	8.9303999	98.8499985
-5.6266418	8.9792004	101.3099976
-5.6266418	9.0279999	100.5499954
-5.6266418	9.0768003	105.2999954
-5.6266418	9.1255999	101.7200012
-5.6266418	9.1744003	107.3099976
-5.6266418	9.2231998	108.1599960
-5.6266418	9.2720003	103.9300003
-5.6266418	9.3207998	101.1699982
-5.6266418	9.3696003	101.8699951
-5.6266418	9.4184008	102.0199966
-5.6266418	9.4672003	102.6599960
-5.6266418	9.5160007	104.3099976
-5.6266418	9.5648003	104.5000000
-5.6266418	9.6136007	103.5800018
-5.6266418	9.6624002	103.4599991
-5.6266418	9.7112007	104.6599960
-5.6266418	9.7600002	103.8499985
-5.6266418	9.8088007	103.2900009
-5.6266418	9.8576002	100.9799957
-5.6266418	9.9064007	104.1899948
-5.6266418	9.9552002	105.1899948
-5.6266418	10.0040007	103.9099960
-5.6266418	10.0528002	102.9000015
-5.6266418	10.1016006	101.6500015
-5.6266418	10.1504002	101.7600021
-5.6266418	10.1992006	104.4599991
-5.6266418	10.2480001	103.5000000
-5.6266418	10.2968006	104.6299973
-5.6266418	10.3456001	105.2299957
-5.6266418	10.3944006	104.5999985
-5.6266418	10.4432001	105.2699966
-5.6266418	10.4920006	106.5000000
-5.6266418	10.5408001	106.5400009
-5.6266418	10.5896006	105.7600021
-5.6266418	10.6384001	104.9199982
-5.6266418	10.6872005	101.3499985
-5.6266418	10.7360001	103.9000015
-5.6266418	10.7848005	105.0199966
-5.6266418	10.8336000	105.1800003
-5.6266418	10.8824005	106.6899948
-5.6266418	10.9312000	103.7799988
-5.6266418	10.9800005	104.1100006
-5.6266418	11.0288000	106.0699997
-5.6266418	11.0776005	104.8799973
-5.6266418	11.1264000	101.2399979
-5.6266418	11.1752005	101.5999985
-5.6266418	11.2240000	105.0599976

-5.6266418	11.2728004	101.5800018
-5.6266418	11.3216000	102.9899979
-5.6266418	11.3704004	106.4000015
-5.6266418	11.4191999	105.5299988
-5.6266418	11.4680004	102.5800018
-5.6266418	11.5167999	102.3699951
-5.6266418	11.5656004	102.6699982
-5.6266418	11.6143999	101.5899963
-5.6266418	11.6632004	102.2399979
-5.6266418	11.7119999	103.7600021
-5.6266418	11.7608004	104.5699997
-5.6266418	11.8095999	103.5299988
-5.6266418	11.8584003	104.3099976
-5.6266418	11.9071999	102.8300018
-5.6266418	11.9560003	102.5100021
-5.6266418	12.0047998	102.4700012
-5.6266418	12.0536003	105.9499969
-5.6266418	12.1023998	104.1299973
-5.6266418	12.1512003	100.7500000
-5.6266418	12.1999998	100.7500000
-5.6266418	12.2488003	104.7699966
-5.6266418	12.2975998	105.6699982
-5.6266418	12.3464003	106.9899979
-5.6266418	12.3951998	105.5000000
-5.6266418	12.4440002	101.6199951
-5.6266418	12.4927998	101.0599976
-5.6266418	12.5416002	102.1299973
-5.6266418	12.5904007	104.0100021
-5.6266418	12.6392002	104.7299957
-5.6266418	12.6880007	104.7600021
-5.6266418	12.7368002	104.1399994
-5.6266418	12.7856007	106.3799973
-5.6266418	12.8344002	105.2600021
-5.6266418	12.8832006	102.6399994
-5.6266418	12.9320002	104.7999954
-5.6266418	12.9808006	105.2200012
-5.6266418	13.0296001	105.3600006
-5.6266418	13.0784006	106.2900009
-5.6266418	13.1272001	102.0100021
-5.6266418	13.1760006	104.3600006
-5.6266418	13.2248001	104.1999969
-5.6266418	13.2736006	101.3300018
-5.6266418	13.3224001	96.4099960
-5.6266418	13.3712006	102.4300003
-5.6266418	13.4200001	104.0999985
-5.6266418	13.4688005	104.1599960
-5.6266418	13.5176001	102.7500000
-5.6266418	13.5664005	102.1299973
-5.6266418	13.6152000	103.6999969
-5.6266418	13.6640005	102.6699982
-5.6266418	13.7128000	101.2299957
-5.6266418	13.7616005	101.7600021
-5.6266418	13.8104000	100.9300003
-5.6266418	13.8592005	102.3399963
-5.6266418	13.9080000	103.5999985
-5.6266418	13.9568005	105.5400009
-5.6266418	14.0056000	104.9499969
-5.6266418	14.0544004	100.9599991

-5.6266418	14.1032000	105.0599976
-5.6266418	14.1520004	105.3099976
-5.6266418	14.2007999	103.6899948
-5.6266418	14.2496004	104.1800003
-5.6266418	14.2983999	105.6599960
-5.6266418	14.3472004	104.9599991
-5.6266418	14.3959999	99.1699982
-5.6266418	14.4448004	101.5100021
-5.6266418	14.4935999	102.5599976
-5.6266418	14.5424004	103.1299973
-5.6266418	14.5911999	103.5299988
-5.6266418	14.6400003	104.2799988
-5.6266418	14.6887999	104.1500015
-5.6266418	14.7376003	104.1999969
-5.6266418	14.7863998	100.2999954
-5.6266418	14.8352003	104.0100021
-5.6266418	14.8839998	102.8600006
-5.6266418	14.9328003	103.4799957
-5.6266418	14.9815998	101.6199951
-5.6266418	15.0304003	102.7699966
-5.6205058	0.3416000	105.1899948
-5.6205058	0.3904000	108.0299988
-5.6205058	0.4392000	106.5400009
-5.6205058	0.4880000	105.9700012
-5.6205058	0.5368000	102.6899948
-5.6205058	0.5856000	108.5999985
-5.6205058	0.6344000	109.8600006
-5.6205058	0.6832000	106.6199951
-5.6205058	0.7320000	101.1599960
-5.6205058	0.7808000	105.8799973
-5.6205058	0.8296000	99.5299988
-5.6205058	0.8784000	100.9300003
-5.6205058	0.9272000	99.8899994
-5.6205058	0.9760000	104.4499969
-5.6205058	1.0247999	104.9199982
-5.6205058	1.0735999	101.0800018
-5.6205058	1.1223999	101.0999985
-5.6205058	1.1711999	105.9399948
-5.6205058	1.2200000	107.2399979
-5.6205058	1.2688000	102.9399948
-5.6205058	1.3176000	104.0599976
-5.6205058	1.3664000	105.9499969
-5.6205058	1.4152000	108.4300003
-5.6205058	1.4640000	106.6500015
-5.6205058	1.5128000	109.3399963
-5.6205058	1.5616000	106.3300018
-5.6205058	1.6104000	105.1100006
-5.6205058	1.6592000	108.2200012
-5.6205058	1.7079999	109.1599960
-5.6205058	1.7567999	107.2900009
-5.6205058	1.8055999	108.1199951
-5.6205058	1.8543999	109.6899948
-5.6205058	1.9031999	109.0800018
-5.6205058	1.9519999	106.3399963
-5.6205058	2.0007999	107.5100021
-5.6205058	2.0495999	108.0800018
-5.6205058	2.0983999	104.9499969
-5.6205058	2.1471999	104.9700012

-5.6205058	2.1959999	101.4599991
-5.6205058	2.2447999	102.2900009
-5.6205058	2.2935998	103.9000015
-5.6205058	2.3423998	106.7299957
-5.6205058	2.3912001	107.7099991
-5.6205058	2.4400001	107.7299957
-5.6205058	2.4888000	105.2799988
-5.6205058	2.5376000	101.4300003
-5.6205058	2.5864000	100.4399948
-5.6205058	2.6352000	103.0400009
-5.6205058	2.6840000	104.1599960
-5.6205058	2.7328000	102.6899948
-5.6205058	2.7816000	103.5699997
-5.6205058	2.8304000	105.4899979
-5.6205058	2.8792000	104.8199997
-5.6205058	2.9280000	107.4499969
-5.6205058	2.9768000	108.2099991
-5.6205058	3.0256000	107.7999954
-5.6205058	3.0743999	104.4399948
-5.6205058	3.1231999	101.6500015
-5.6205058	3.1719999	104.2500000
-5.6205058	3.2207999	106.1500015
-5.6205058	3.2695999	105.9199982
-5.6205058	3.3183999	104.3899994
-5.6205058	3.3671999	102.6500015
-5.6205058	3.4159999	103.6899948
-5.6205058	3.4647999	103.0299988
-5.6205058	3.5135999	104.0699997
-5.6205058	3.5623999	104.4799957
-5.6205058	3.6111999	104.5199966
-5.6205058	3.6599998	104.2999954
-5.6205058	3.7087998	105.0199966
-5.6205058	3.7575998	105.9799957
-5.6205058	3.8063998	105.8799973
-5.6205058	3.8551998	105.2999954
-5.6205058	3.9039998	103.2600021
-5.6205058	3.9527998	104.8699951
-5.6205058	4.0016003	105.1100006
-5.6205058	4.0503998	101.3499985
-5.6205058	4.0991998	101.3099976
-5.6205058	4.1479998	102.9799957
-5.6205058	4.1967998	101.0000000
-5.6205058	4.2455997	103.7999954
-5.6205058	4.2943997	104.1299973
-5.6205058	4.3431997	107.1100006
-5.6205058	4.3919997	107.0400009
-5.6205058	4.4407997	106.6899948
-5.6205058	4.4895997	104.9599991
-5.6205058	4.5383997	106.6299973
-5.6205058	4.5871997	108.5299988
-5.6205058	4.6359997	110.8099976
-5.6205058	4.6847997	113.6699982
-5.6205058	4.7336001	112.7799988
-5.6205058	4.7824001	109.9700012
-5.6205058	4.8312001	107.3699951
-5.6205058	4.8800001	108.5699997
-5.6205058	4.9288001	107.1399994
-5.6205058	4.9776001	105.9499969

-5.6205058	5.0264001	107.3600006
-5.6205058	5.0752001	105.9300003
-5.6205058	5.1240001	105.9399948
-5.6205058	5.1728001	102.3399963
-5.6205058	5.2216001	104.4700012
-5.6205058	5.2704000	104.6999969
-5.6205058	5.3192000	102.6800003
-5.6205058	5.3680000	104.5100021
-5.6205058	5.4168000	104.5999985
-5.6205058	5.4656000	104.0800018
-5.6205058	5.5144000	103.9899979
-5.6205058	5.5632000	103.2299957
-5.6205058	5.6120000	102.3799973
-5.6205058	5.6608000	100.9099960
-5.6205058	5.7096000	99.2600021
-5.6205058	5.7584000	102.8300018
-5.6205058	5.8072000	103.5199966
-5.6205058	5.8559999	100.6500015
-5.6205058	5.9047999	100.4899979
-5.6205058	5.9535999	101.7500000
-5.6205058	6.0023999	102.2600021
-5.6205058	6.0511999	100.3600006
-5.6205058	6.0999999	103.5000000
-5.6205058	6.1487999	102.4000015
-5.6205058	6.1975999	104.4099960
-5.6205058	6.2463999	102.9399948
-5.6205058	6.2951999	102.1599960
-5.6205058	6.3439999	104.8600006
-5.6205058	6.3927999	103.4700012
-5.6205058	6.4415998	101.4399948
-5.6205058	6.4903998	103.4099960
-5.6205058	6.5391998	103.7399979
-5.6205058	6.5879998	102.2900009
-5.6205058	6.6367998	101.4000015
-5.6205058	6.6855998	101.9599991
-5.6205058	6.7343998	103.7699966
-5.6205058	6.7831998	103.6800003
-5.6205058	6.8319998	102.3600006
-5.6205058	6.8807998	104.4300003
-5.6205058	6.9295998	105.2699966
-5.6205058	6.9783998	105.3899994
-5.6205058	7.0271997	105.0999985
-5.6205058	7.0759997	104.1899948
-5.6205058	7.1247997	100.7299957
-5.6205058	7.1735997	103.4799957
-5.6205058	7.2223997	103.7900009
-5.6205058	7.2711997	105.5800018
-5.6205058	7.3199997	103.4899979
-5.6205058	7.3687997	102.2099991
-5.6205058	7.4175997	102.1500015
-5.6205058	7.4663997	104.6100006
-5.6205058	7.5151997	105.5800018
-5.6205058	7.5639997	103.3499985
-5.6205058	7.6127996	101.3899994
-5.6205058	7.6615996	103.2099991
-5.6205058	7.7103996	103.1899948
-5.6205058	7.7591996	104.6399994
-5.6205058	7.8079996	105.6599960

-5.6205058	7.8567996	103.1500015
-5.6205058	7.9056001	103.2399979
-5.6205058	7.9544001	103.7999954
-5.6205058	8.0032005	103.9899979
-5.6205058	8.0520000	102.2399979
-5.6205058	8.1008005	103.8099976
-5.6205058	8.1496000	100.7999954
-5.6205058	8.1984005	101.7600021
-5.6205058	8.2472000	104.8300018
-5.6205058	8.2960005	106.1199951
-5.6205058	8.3448000	106.7500000
-5.6205058	8.3936005	105.2500000
-5.6205058	8.4424000	103.9499969
-5.6205058	8.4912004	103.2999954
-5.6205058	8.5400000	105.7999954
-5.6205058	8.5888004	107.5499954
-5.6205058	8.6375999	106.5100021
-5.6205058	8.6864004	105.7299957
-5.6205058	8.7351999	103.4099960
-5.6205058	8.7840004	103.9700012
-5.6205058	8.8327999	100.0299988
-5.6205058	8.8816004	97.2699966
-5.6205058	8.9303999	100.1399994
-5.6205058	8.9792004	102.5400009
-5.6205058	9.0279999	99.5299988
-5.6205058	9.0768003	104.7699966
-5.6205058	9.1255999	101.0899963
-5.6205058	9.1744003	107.1199951
-5.6205058	9.2231998	108.5800018
-5.6205058	9.2720003	104.8699951
-5.6205058	9.3207998	101.4099960
-5.6205058	9.3696003	101.8499985
-5.6205058	9.4184008	101.5100021
-5.6205058	9.4672003	102.5999985
-5.6205058	9.5160007	105.0800018
-5.6205058	9.5648003	104.5299988
-5.6205058	9.6136007	102.8799973
-5.6205058	9.6624002	102.4899979
-5.6205058	9.7112007	104.4300003
-5.6205058	9.7600002	103.9099960
-5.6205058	9.8088007	102.8799973
-5.6205058	9.8576002	100.1800003
-5.6205058	9.9064007	104.4300003
-5.6205058	9.9552002	105.9199982
-5.6205058	10.0040007	104.6199951
-5.6205058	10.0528002	103.0199966
-5.6205058	10.1016006	102.0100021
-5.6205058	10.1504002	102.4499969
-5.6205058	10.1992006	104.2999954
-5.6205058	10.2480001	102.6999969
-5.6205058	10.2968006	104.6100006
-5.6205058	10.3456001	106.1999969
-5.6205058	10.3944006	105.6800003
-5.6205058	10.4432001	105.0699997
-5.6205058	10.4920006	106.5599976
-5.6205058	10.5408001	106.6299973
-5.6205058	10.5896006	106.1599960
-5.6205058	10.6384001	104.7699966

-5.6205058	10.6872005	101.3499985
-5.6205058	10.7360001	103.8600006
-5.6205058	10.7848005	105.5699997
-5.6205058	10.8336000	105.4700012
-5.6205058	10.8824005	106.3699951
-5.6205058	10.9312000	103.2999954
-5.6205058	10.9800005	104.4099960
-5.6205058	11.0288000	106.5299988
-5.6205058	11.0776005	106.1100006
-5.6205058	11.1264000	101.8600006
-5.6205058	11.1752005	101.3399963
-5.6205058	11.2240000	105.4399948
-5.6205058	11.2728004	102.1899948
-5.6205058	11.3216000	103.0299988
-5.6205058	11.3704004	105.4499969
-5.6205058	11.4191999	104.9599991
-5.6205058	11.4680004	102.0199966
-5.6205058	11.5167999	101.0499954
-5.6205058	11.5656004	102.0100021
-5.6205058	11.6143999	101.4499969
-5.6205058	11.6632004	101.7299957
-5.6205058	11.7119999	103.1299973
-5.6205058	11.7608004	104.2699966
-5.6205058	11.8095999	104.1399994
-5.6205058	11.8584003	104.5599976
-5.6205058	11.9071999	102.6699982
-5.6205058	11.9560003	102.4599991
-5.6205058	12.0047998	104.3199997
-5.6205058	12.0536003	106.5499954
-5.6205058	12.1023998	104.5800018
-5.6205058	12.1512003	100.7500000
-5.6205058	12.1999998	100.4399948
-5.6205058	12.2488003	104.9399948
-5.6205058	12.2975998	106.3600006
-5.6205058	12.3464003	107.2500000
-5.6205058	12.3951998	105.3199997
-5.6205058	12.4440002	101.7799988
-5.6205058	12.4927998	101.9300003
-5.6205058	12.5416002	103.2900009
-5.6205058	12.5904007	104.4300003
-5.6205058	12.6392002	103.0899963
-5.6205058	12.6880007	104.0800018
-5.6205058	12.7368002	104.6599960
-5.6205058	12.7856007	106.7099991
-5.6205058	12.8344002	105.3600006
-5.6205058	12.8832006	102.6800003
-5.6205058	12.9320002	104.8099976
-5.6205058	12.9808006	104.7999954
-5.6205058	13.0296001	105.1899948
-5.6205058	13.0784006	105.5699997
-5.6205058	13.1272001	101.7399979
-5.6205058	13.1760006	103.6899948
-5.6205058	13.2248001	103.2699966
-5.6205058	13.2736006	101.1699982
-5.6205058	13.3224001	95.9399948
-5.6205058	13.3712006	101.6999969
-5.6205058	13.4200001	103.3399963
-5.6205058	13.4688005	103.2299957

-5.6205058	13.5176001	102.6299973
-5.6205058	13.5664005	101.4499969
-5.6205058	13.6152000	103.4599991
-5.6205058	13.6640005	102.6199951
-5.6205058	13.7128000	101.0599976
-5.6205058	13.7616005	101.9599991
-5.6205058	13.8104000	100.2699966
-5.6205058	13.8592005	101.9700012
-5.6205058	13.9080000	103.6199951
-5.6205058	13.9568005	104.8899994
-5.6205058	14.0056000	104.0499954
-5.6205058	14.0544004	101.6800003
-5.6205058	14.1032000	104.6299973
-5.6205058	14.1520004	104.5100021
-5.6205058	14.2007999	102.1800003
-5.6205058	14.2496004	103.6999969
-5.6205058	14.2983999	105.6500015
-5.6205058	14.3472004	104.8199997
-5.6205058	14.3959999	100.4099960
-5.6205058	14.4448004	101.5499954
-5.6205058	14.4935999	102.3799973
-5.6205058	14.5424004	102.8199997
-5.6205058	14.5911999	103.5599976
-5.6205058	14.6400003	105.3899994
-5.6205058	14.6887999	105.3499985
-5.6205058	14.7376003	104.1299973
-5.6205058	14.7863998	99.4799957
-5.6205058	14.8352003	103.7500000
-5.6205058	14.8839998	102.3099976
-5.6205058	14.9328003	103.4300003
-5.6205058	14.9815998	101.8199997
-5.6205058	15.0304003	102.8600006
-5.6143699	0.3416000	105.2799988
-5.6143699	0.3904000	106.9700012
-5.6143699	0.4392000	106.5699997
-5.6143699	0.4880000	105.3799973
-5.6143699	0.5368000	104.6899948
-5.6143699	0.5856000	108.7399979
-5.6143699	0.6344000	108.9499969
-5.6143699	0.6832000	105.4799957
-5.6143699	0.7320000	102.3799973
-5.6143699	0.7808000	105.5999985
-5.6143699	0.8296000	98.9899979
-5.6143699	0.8784000	101.3799973
-5.6143699	0.9272000	98.7900009
-5.6143699	0.9760000	104.0199966
-5.6143699	1.0247999	104.9499969
-5.6143699	1.0735999	101.8199997
-5.6143699	1.1223999	101.1899948
-5.6143699	1.1711999	105.8300018
-5.6143699	1.2200000	106.8799973
-5.6143699	1.2688000	102.7600021
-5.6143699	1.3176000	104.3600006
-5.6143699	1.3664000	106.2500000
-5.6143699	1.4152000	108.3899994
-5.6143699	1.4640000	104.6699982
-5.6143699	1.5128000	108.7699966
-5.6143699	1.5616000	105.3600006

-5.6143699	1.6104000	104.4000015
-5.6143699	1.6592000	107.8899994
-5.6143699	1.7079999	109.4799957
-5.6143699	1.7567999	107.2799988
-5.6143699	1.8055999	107.7799988
-5.6143699	1.8543999	108.5699997
-5.6143699	1.9031999	107.7900009
-5.6143699	1.9519999	105.5400009
-5.6143699	2.0007999	107.0499954
-5.6143699	2.0495999	108.6100006
-5.6143699	2.0983999	104.4899979
-5.6143699	2.1471999	104.5599976
-5.6143699	2.1959999	102.1299973
-5.6143699	2.2447999	103.5400009
-5.6143699	2.2935998	104.2699966
-5.6143699	2.3423998	106.5400009
-5.6143699	2.3912001	107.8300018
-5.6143699	2.4400001	107.5599976
-5.6143699	2.4888000	104.6299973
-5.6143699	2.5376000	101.7200012
-5.6143699	2.5864000	101.2900009
-5.6143699	2.6352000	103.1100006
-5.6143699	2.6840000	103.9300003
-5.6143699	2.7328000	102.0199966
-5.6143699	2.7816000	103.9000015
-5.6143699	2.8304000	105.9199982
-5.6143699	2.8792000	105.8099976
-5.6143699	2.9280000	107.1299973
-5.6143699	2.9768000	107.4899979
-5.6143699	3.0256000	106.9300003
-5.6143699	3.0743999	102.4199982
-5.6143699	3.1231999	100.4599991
-5.6143699	3.1719999	102.7500000
-5.6143699	3.2207999	105.2399979
-5.6143699	3.2695999	105.1599960
-5.6143699	3.3183999	104.7600021
-5.6143699	3.3671999	103.4199982
-5.6143699	3.4159999	104.2099991
-5.6143699	3.4647999	103.7299957
-5.6143699	3.5135999	104.3099976
-5.6143699	3.5623999	104.6399994
-5.6143699	3.6111999	104.5000000
-5.6143699	3.6599998	104.3399963
-5.6143699	3.7087998	105.4700012
-5.6143699	3.7575998	106.8300018
-5.6143699	3.8063998	106.4300003
-5.6143699	3.8551998	105.3499985
-5.6143699	3.9039998	103.5299988
-5.6143699	3.9527998	104.4899979
-5.6143699	4.0016003	104.7600021
-5.6143699	4.0503998	101.7999954
-5.6143699	4.0991998	101.1299973
-5.6143699	4.1479998	101.9099960
-5.6143699	4.1967998	100.9199982
-5.6143699	4.2455997	103.9000015
-5.6143699	4.2943997	103.5499954
-5.6143699	4.3431997	107.3899994
-5.6143699	4.3919997	107.9300003

-5.6143699	4.4407997	108.5400009
-5.6143699	4.4895997	106.3300018
-5.6143699	4.5383997	107.0800018
-5.6143699	4.5871997	109.4399948
-5.6143699	4.6359997	111.5299988
-5.6143699	4.6847997	114.6999969
-5.6143699	4.7336001	113.6399994
-5.6143699	4.7824001	110.4199982
-5.6143699	4.8312001	108.3600006
-5.6143699	4.8800001	108.6599960
-5.6143699	4.9288001	107.1399994
-5.6143699	4.9776001	106.4099960
-5.6143699	5.0264001	107.0299988
-5.6143699	5.0752001	105.4399948
-5.6143699	5.1240001	106.9799957
-5.6143699	5.1728001	103.6800003
-5.6143699	5.2216001	105.5599976
-5.6143699	5.2704000	105.5599976
-5.6143699	5.3192000	103.4300003
-5.6143699	5.3680000	103.2099991
-5.6143699	5.4168000	102.6599960
-5.6143699	5.4656000	102.0599976
-5.6143699	5.5144000	103.0199966
-5.6143699	5.5632000	103.1299973
-5.6143699	5.6120000	101.7900009
-5.6143699	5.6608000	101.2299957
-5.6143699	5.7096000	99.4599991
-5.6143699	5.7584000	102.1699982
-5.6143699	5.8072000	103.9399948
-5.6143699	5.8559999	102.1199951
-5.6143699	5.9047999	100.6800003
-5.6143699	5.9535999	102.4300003
-5.6143699	6.0023999	102.5000000
-5.6143699	6.0511999	100.9700012
-5.6143699	6.0999999	104.1999969
-5.6143699	6.1487999	103.6800003
-5.6143699	6.1975999	104.4899979
-5.6143699	6.2463999	103.2799988
-5.6143699	6.2951999	102.5599976
-5.6143699	6.3439999	105.2399979
-5.6143699	6.3927999	103.5699997
-5.6143699	6.4415998	101.2799988
-5.6143699	6.4903998	103.0800018
-5.6143699	6.5391998	103.5999985
-5.6143699	6.5879998	101.7399979
-5.6143699	6.6367998	100.5199966
-5.6143699	6.6855998	102.6599960
-5.6143699	6.7343998	103.4599991
-5.6143699	6.7831998	103.7500000
-5.6143699	6.8319998	102.9000015
-5.6143699	6.8807998	104.6800003
-5.6143699	6.9295998	104.7200012
-5.6143699	6.9783998	104.2500000
-5.6143699	7.0271997	103.8099976
-5.6143699	7.0759997	104.0800018
-5.6143699	7.1247997	102.0699997
-5.6143699	7.1735997	103.9099960
-5.6143699	7.2223997	103.6199951

-5.6143699	7.2711997	104.8300018
-5.6143699	7.3199997	103.1500015
-5.6143699	7.3687997	101.5299988
-5.6143699	7.4175997	102.6699982
-5.6143699	7.4663997	105.7399979
-5.6143699	7.5151997	105.9599991
-5.6143699	7.5639997	102.9199982
-5.6143699	7.6127996	99.5699997
-5.6143699	7.6615996	101.4000015
-5.6143699	7.7103996	102.2399979
-5.6143699	7.7591996	104.3600006
-5.6143699	7.8079996	105.8199997
-5.6143699	7.8567996	102.9799957
-5.6143699	7.9056001	104.0100021
-5.6143699	7.9544001	104.5299988
-5.6143699	8.0032005	104.1100006
-5.6143699	8.0520000	101.6800003
-5.6143699	8.1008005	103.9000015
-5.6143699	8.1496000	101.6699982
-5.6143699	8.1984005	101.6100006
-5.6143699	8.2472000	104.8300018
-5.6143699	8.2960005	106.9599991
-5.6143699	8.3448000	108.4799957
-5.6143699	8.3936005	105.6699982
-5.6143699	8.4424000	103.5000000
-5.6143699	8.4912004	103.0800018
-5.6143699	8.5400000	105.3300018
-5.6143699	8.5888004	107.3799973
-5.6143699	8.6375999	106.6299973
-5.6143699	8.6864004	104.5499954
-5.6143699	8.7351999	103.3799973
-5.6143699	8.7840004	104.4300003
-5.6143699	8.8327999	99.5999985
-5.6143699	8.8816004	98.6199951
-5.6143699	8.9303999	101.3600006
-5.6143699	8.9792004	103.0800018
-5.6143699	9.0279999	101.0499954
-5.6143699	9.0768003	104.8199997
-5.6143699	9.1255999	100.9899979
-5.6143699	9.1744003	106.7299957
-5.6143699	9.2231998	108.0699997
-5.6143699	9.2720003	105.4899979
-5.6143699	9.3207998	103.0999985
-5.6143699	9.3696003	102.9199982
-5.6143699	9.4184008	101.1699982
-5.6143699	9.4672003	102.2200012
-5.6143699	9.5160007	104.7600021
-5.6143699	9.5648003	104.7500000
-5.6143699	9.6136007	103.0000000
-5.6143699	9.6624002	102.5599976
-5.6143699	9.7112007	104.3199997
-5.6143699	9.7600002	103.7799988
-5.6143699	9.8088007	102.5699997
-5.6143699	9.8576002	100.4300003
-5.6143699	9.9064007	104.8699951
-5.6143699	9.9552002	106.6100006
-5.6143699	10.0040007	105.3899994
-5.6143699	10.0528002	103.5400009

-5.6143699	10.1016006	102.9399948
-5.6143699	10.1504002	103.1699982
-5.6143699	10.1992006	103.9499969
-5.6143699	10.2480001	102.2999954
-5.6143699	10.2968006	105.1100006
-5.6143699	10.3456001	106.9099960
-5.6143699	10.3944006	106.6500015
-5.6143699	10.4432001	104.6899948
-5.6143699	10.4920006	106.5800018
-5.6143699	10.5408001	106.7299957
-5.6143699	10.5896006	106.2999954
-5.6143699	10.6384001	103.9799957
-5.6143699	10.6872005	100.0999985
-5.6143699	10.7360001	103.5400009
-5.6143699	10.7848005	106.2200012
-5.6143699	10.8336000	105.9799957
-5.6143699	10.8824005	106.1899948
-5.6143699	10.9312000	103.0100021
-5.6143699	10.9800005	104.1199951
-5.6143699	11.0288000	106.3899994
-5.6143699	11.0776005	106.6599960
-5.6143699	11.1264000	102.1100006
-5.6143699	11.1752005	101.0199966
-5.6143699	11.2240000	105.2999954
-5.6143699	11.2728004	102.2999954
-5.6143699	11.3216000	102.9099960
-5.6143699	11.3704004	104.1800003
-5.6143699	11.4191999	104.0699997
-5.6143699	11.4680004	100.5100021
-5.6143699	11.5167999	99.3399963
-5.6143699	11.5656004	101.2699966
-5.6143699	11.6143999	101.5899963
-5.6143699	11.6632004	101.6800003
-5.6143699	11.7119999	103.2299957
-5.6143699	11.7608004	104.4599991
-5.6143699	11.8095999	104.8099976
-5.6143699	11.8584003	104.2600021
-5.6143699	11.9071999	102.0299988
-5.6143699	11.9560003	102.2500000
-5.6143699	12.0047998	105.3799973
-5.6143699	12.0536003	106.7200012
-5.6143699	12.1023998	104.1999969
-5.6143699	12.1512003	100.4000015
-5.6143699	12.1999998	100.0400009
-5.6143699	12.2488003	104.8399963
-5.6143699	12.2975998	106.6100006
-5.6143699	12.3464003	107.0400009
-5.6143699	12.3951998	105.0000000
-5.6143699	12.4440002	102.2600021
-5.6143699	12.4927998	103.1299973
-5.6143699	12.5416002	104.4799957
-5.6143699	12.5904007	104.7399979
-5.6143699	12.6392002	101.0499954
-5.6143699	12.6880007	103.0000000
-5.6143699	12.7368002	104.5499954
-5.6143699	12.7856007	106.4700012
-5.6143699	12.8344002	105.4700012
-5.6143699	12.8832006	102.4799957

-5.6143699	12.9320002	104.4700012
-5.6143699	12.9808006	105.0000000
-5.6143699	13.0296001	105.9300003
-5.6143699	13.0784006	105.6800003
-5.6143699	13.1272001	102.1800003
-5.6143699	13.1760006	103.6800003
-5.6143699	13.2248001	102.2699966
-5.6143699	13.2736006	100.8099976
-5.6143699	13.3224001	95.2299957
-5.6143699	13.3712006	100.4899979
-5.6143699	13.4200001	102.7099991
-5.6143699	13.4688005	102.6100006
-5.6143699	13.5176001	102.6199951
-5.6143699	13.5664005	102.3099976
-5.6143699	13.6152000	103.6599960
-5.6143699	13.6640005	102.3499985
-5.6143699	13.7128000	100.6999969
-5.6143699	13.7616005	102.1800003
-5.6143699	13.8104000	100.8499985
-5.6143699	13.8592005	102.0199966
-5.6143699	13.9080000	103.2399979
-5.6143699	13.9568005	104.2500000
-5.6143699	14.0056000	103.4399948
-5.6143699	14.0544004	102.3600006
-5.6143699	14.1032000	103.9499969
-5.6143699	14.1520004	102.7999954
-5.6143699	14.2007999	100.4700012
-5.6143699	14.2496004	102.8399963
-5.6143699	14.2983999	105.1999969
-5.6143699	14.3472004	104.3699951
-5.6143699	14.3959999	101.3600006
-5.6143699	14.4448004	101.6199951
-5.6143699	14.4935999	102.4499969
-5.6143699	14.5424004	102.6800003
-5.6143699	14.5911999	103.0699997
-5.6143699	14.6400003	105.0999985
-5.6143699	14.6887999	105.5000000
-5.6143699	14.7376003	103.5299988
-5.6143699	14.7863998	99.6800003
-5.6143699	14.8352003	103.6199951
-5.6143699	14.8839998	101.7299957
-5.6143699	14.9328003	103.2099991
-5.6143699	14.9815998	101.4099960
-5.6143699	15.0304003	102.3899994
-5.6082339	0.3416000	105.4899979
-5.6082339	0.3904000	106.2399979
-5.6082339	0.4392000	107.0499954
-5.6082339	0.4880000	104.6500015
-5.6082339	0.5368000	105.6399994
-5.6082339	0.5856000	108.4799957
-5.6082339	0.6344000	107.7099991
-5.6082339	0.6832000	104.6999969
-5.6082339	0.7320000	104.0100021
-5.6082339	0.7808000	105.9499969
-5.6082339	0.8296000	100.0499954
-5.6082339	0.8784000	102.9799957
-5.6082339	0.9272000	99.6599960
-5.6082339	0.9760000	103.9300003

-5.6082339	1.0247999	105.0599976
-5.6082339	1.0735999	102.9199982
-5.6082339	1.1223999	101.6399994
-5.6082339	1.1711999	105.9700012
-5.6082339	1.2200000	106.4499969
-5.6082339	1.2688000	102.1699982
-5.6082339	1.3176000	104.5499954
-5.6082339	1.3664000	106.7099991
-5.6082339	1.4152000	108.0699997
-5.6082339	1.4640000	102.2799988
-5.6082339	1.5128000	108.1100006
-5.6082339	1.5616000	105.2799988
-5.6082339	1.6104000	104.1500015
-5.6082339	1.6592000	107.3799973
-5.6082339	1.7079999	109.4199982
-5.6082339	1.7567999	106.6999969
-5.6082339	1.8055999	107.0599976
-5.6082339	1.8543999	107.1399994
-5.6082339	1.9031999	105.6999969
-5.6082339	1.9519999	104.5899963
-5.6082339	2.0007999	106.7600021
-5.6082339	2.0495999	109.3099976
-5.6082339	2.0983999	105.6699982
-5.6082339	2.1471999	104.6299973
-5.6082339	2.1959999	102.5400009
-5.6082339	2.2447999	104.4300003
-5.6082339	2.2935998	105.2399979
-5.6082339	2.3423998	106.7799988
-5.6082339	2.3912001	108.5100021
-5.6082339	2.4400001	107.8699951
-5.6082339	2.4888000	104.3099976
-5.6082339	2.5376000	102.5400009
-5.6082339	2.5864000	102.8300018
-5.6082339	2.6352000	104.0000000
-5.6082339	2.6840000	104.1199951
-5.6082339	2.7328000	101.3600006
-5.6082339	2.7816000	103.6699982
-5.6082339	2.8304000	105.8300018
-5.6082339	2.8792000	106.6599960
-5.6082339	2.9280000	106.7600021
-5.6082339	2.9768000	106.6500015
-5.6082339	3.0256000	106.0199966
-5.6082339	3.0743999	100.7299957
-5.6082339	3.1231999	100.0400009
-5.6082339	3.1719999	102.0699997
-5.6082339	3.2207999	104.8199997
-5.6082339	3.2695999	104.2099991
-5.6082339	3.3183999	104.6199951
-5.6082339	3.3671999	104.2600021
-5.6082339	3.4159999	105.0299988
-5.6082339	3.4647999	103.8699951
-5.6082339	3.5135999	104.3899994
-5.6082339	3.5623999	105.1299973
-5.6082339	3.6111999	104.9499969
-5.6082339	3.6599998	104.5699997
-5.6082339	3.7087998	106.0100021
-5.6082339	3.7575998	107.4799957
-5.6082339	3.8063998	106.4399948

-5.6082339	3.8551998	104.7299957
-5.6082339	3.9039998	103.1800003
-5.6082339	3.9527998	103.5699997
-5.6082339	4.0016003	104.6500015
-5.6082339	4.0503998	102.7999954
-5.6082339	4.0991998	101.7299957
-5.6082339	4.1479998	101.4899979
-5.6082339	4.1967998	101.8300018
-5.6082339	4.2455997	104.0800018
-5.6082339	4.2943997	102.7299957
-5.6082339	4.3431997	107.8099976
-5.6082339	4.3919997	108.8399963
-5.6082339	4.4407997	109.2399979
-5.6082339	4.4895997	106.6699982
-5.6082339	4.5383997	106.7900009
-5.6082339	4.5871997	108.9300003
-5.6082339	4.6359997	111.7399979
-5.6082339	4.6847997	115.5999985
-5.6082339	4.7336001	114.5299988
-5.6082339	4.7824001	110.7600021
-5.6082339	4.8312001	109.0199966
-5.6082339	4.8800001	108.2600021
-5.6082339	4.9288001	106.8399963
-5.6082339	4.9776001	106.7200012
-5.6082339	5.0264001	106.5299988
-5.6082339	5.0752001	105.6899948
-5.6082339	5.1240001	107.7099991
-5.6082339	5.1728001	104.2600021
-5.6082339	5.2216001	105.8799973
-5.6082339	5.2704000	105.8399963
-5.6082339	5.3192000	103.3499985
-5.6082339	5.3680000	102.2600021
-5.6082339	5.4168000	102.1699982
-5.6082339	5.4656000	101.5999985
-5.6082339	5.5144000	102.9300003
-5.6082339	5.5632000	103.0599976
-5.6082339	5.6120000	101.3799973
-5.6082339	5.6608000	101.2600021
-5.6082339	5.7096000	99.9499969
-5.6082339	5.7584000	101.8899994
-5.6082339	5.8072000	103.8399963
-5.6082339	5.8559999	103.2200012
-5.6082339	5.9047999	100.4399948
-5.6082339	5.9535999	102.5199966
-5.6082339	6.0023999	102.8499985
-5.6082339	6.0511999	101.5299988
-5.6082339	6.0999999	105.5699997
-5.6082339	6.1487999	105.0499954
-5.6082339	6.1975999	104.0999985
-5.6082339	6.2463999	103.2699966
-5.6082339	6.2951999	102.8600006
-5.6082339	6.3439999	105.4700012
-5.6082339	6.3927999	103.5100021
-5.6082339	6.4415998	100.9300003
-5.6082339	6.4903998	102.1699982
-5.6082339	6.5391998	102.6599960
-5.6082339	6.5879998	101.1500015
-5.6082339	6.6367998	101.0000000

-5.6082339	6.6855998	103.8099976
-5.6082339	6.7343998	103.6399994
-5.6082339	6.7831998	104.1399994
-5.6082339	6.8319998	102.9199982
-5.6082339	6.8807998	104.8099976
-5.6082339	6.9295998	104.8099976
-5.6082339	6.9783998	104.0000000
-5.6082339	7.0271997	103.0400009
-5.6082339	7.0759997	103.5400009
-5.6082339	7.1247997	103.0299988
-5.6082339	7.1735997	104.1800003
-5.6082339	7.2223997	103.3799973
-5.6082339	7.2711997	104.4499969
-5.6082339	7.3199997	103.2200012
-5.6082339	7.3687997	100.5899963
-5.6082339	7.4175997	102.3399963
-5.6082339	7.4663997	105.9399948
-5.6082339	7.5151997	105.7399979
-5.6082339	7.5639997	102.7900009
-5.6082339	7.6127996	98.2799988
-5.6082339	7.6615996	99.9300003
-5.6082339	7.7103996	101.8499985
-5.6082339	7.7591996	104.3600006
-5.6082339	7.8079996	106.0400009
-5.6082339	7.8567996	103.9599991
-5.6082339	7.9056001	104.9700012
-5.6082339	7.9544001	105.2399979
-5.6082339	8.0032005	104.5199966
-5.6082339	8.0520000	101.5000000
-5.6082339	8.1008005	103.7699966
-5.6082339	8.1496000	102.0199966
-5.6082339	8.1984005	101.4199982
-5.6082339	8.2472000	105.0100021
-5.6082339	8.2960005	107.4000015
-5.6082339	8.3448000	108.7999954
-5.6082339	8.3936005	105.6500015
-5.6082339	8.4424000	103.0800018
-5.6082339	8.4912004	102.9000015
-5.6082339	8.5400000	105.0999985
-5.6082339	8.5888004	107.1100006
-5.6082339	8.6375999	106.5499954
-5.6082339	8.6864004	103.1199951
-5.6082339	8.7351999	103.4099960
-5.6082339	8.7840004	104.8199997
-5.6082339	8.8327999	99.8099976
-5.6082339	8.8816004	100.0800018
-5.6082339	8.9303999	101.7600021
-5.6082339	8.9792004	103.5899963
-5.6082339	9.0279999	102.4099960
-5.6082339	9.0768003	104.2900009
-5.6082339	9.1255999	100.5000000
-5.6082339	9.1744003	106.4499969
-5.6082339	9.2231998	107.4899979
-5.6082339	9.2720003	105.0499954
-5.6082339	9.3207998	104.4599991
-5.6082339	9.3696003	104.2699966
-5.6082339	9.4184008	101.9599991
-5.6082339	9.4672003	102.4399948

-5.6082339	9.5160007	104.3499985
-5.6082339	9.5648003	105.0299988
-5.6082339	9.6136007	102.8899994
-5.6082339	9.6624002	102.4000015
-5.6082339	9.7112007	104.4000015
-5.6082339	9.7600002	104.5000000
-5.6082339	9.8088007	102.6299973
-5.6082339	9.8576002	100.8199997
-5.6082339	9.9064007	105.4700012
-5.6082339	9.9552002	107.4899979
-5.6082339	10.0040007	106.4399948
-5.6082339	10.0528002	104.0100021
-5.6082339	10.1016006	102.9000015
-5.6082339	10.1504002	103.3699951
-5.6082339	10.1992006	102.9899979
-5.6082339	10.2480001	101.5100021
-5.6082339	10.2968006	105.4199982
-5.6082339	10.3456001	107.0299988
-5.6082339	10.3944006	107.0599976
-5.6082339	10.4432001	105.4300003
-5.6082339	10.4920006	106.9700012
-5.6082339	10.5408001	107.0800018
-5.6082339	10.5896006	106.2999954
-5.6082339	10.6384001	103.5000000
-5.6082339	10.6872005	99.1699982
-5.6082339	10.7360001	102.6399994
-5.6082339	10.7848005	106.0100021
-5.6082339	10.8336000	105.8300018
-5.6082339	10.8824005	105.3899994
-5.6082339	10.9312000	101.8199997
-5.6082339	10.9800005	102.3099976
-5.6082339	11.0288000	105.2099991
-5.6082339	11.0776005	106.4399948
-5.6082339	11.1264000	101.9300003
-5.6082339	11.1752005	100.6800003
-5.6082339	11.2240000	105.0699997
-5.6082339	11.2728004	102.0599976
-5.6082339	11.3216000	102.3399963
-5.6082339	11.3704004	102.3799973
-5.6082339	11.4191999	103.4000015
-5.6082339	11.4680004	100.1199951
-5.6082339	11.5167999	98.4499969
-5.6082339	11.5656004	100.6299973
-5.6082339	11.6143999	102.0499954
-5.6082339	11.6632004	102.5000000
-5.6082339	11.7119999	103.5400009
-5.6082339	11.7608004	104.2999954
-5.6082339	11.8095999	104.8199997
-5.6082339	11.8584003	103.4199982
-5.6082339	11.9071999	101.1299973
-5.6082339	11.9560003	101.4700012
-5.6082339	12.0047998	105.3499985
-5.6082339	12.0536003	106.5299988
-5.6082339	12.1023998	104.1500015
-5.6082339	12.1512003	100.6599960
-5.6082339	12.1999998	100.5100021
-5.6082339	12.2488003	104.8699951
-5.6082339	12.2975998	106.6899948

-5.6082339	12.3464003	106.8499985
-5.6082339	12.3951998	104.7799988
-5.6082339	12.4440002	102.4599991
-5.6082339	12.4927998	103.8099976
-5.6082339	12.5416002	105.3799973
-5.6082339	12.5904007	104.9199982
-5.6082339	12.6392002	99.0100021
-5.6082339	12.6880007	102.0199966
-5.6082339	12.7368002	104.7999954
-5.6082339	12.7856007	106.2799988
-5.6082339	12.8344002	105.4799957
-5.6082339	12.8832006	102.5599976
-5.6082339	12.9320002	104.7699966
-5.6082339	12.9808006	104.8699951
-5.6082339	13.0296001	105.4700012
-5.6082339	13.0784006	105.2200012
-5.6082339	13.1272001	103.3799973
-5.6082339	13.1760006	103.6999969
-5.6082339	13.2248001	100.8899994
-5.6082339	13.2736006	100.2999954
-5.6082339	13.3224001	96.7399979
-5.6082339	13.3712006	100.0299988
-5.6082339	13.4200001	102.4799957
-5.6082339	13.4688005	102.5400009
-5.6082339	13.5176001	103.2699966
-5.6082339	13.5664005	102.9499969
-5.6082339	13.6152000	103.5800018
-5.6082339	13.6640005	102.4899979
-5.6082339	13.7128000	101.0299988
-5.6082339	13.7616005	102.5199966
-5.6082339	13.8104000	101.3799973
-5.6082339	13.8592005	101.9799957
-5.6082339	13.9080000	103.2600021
-5.6082339	13.9568005	103.3600006
-5.6082339	14.0056000	102.5400009
-5.6082339	14.0544004	103.3499985
-5.6082339	14.1032000	103.8499985
-5.6082339	14.1520004	101.5299988
-5.6082339	14.2007999	98.9899979
-5.6082339	14.2496004	101.7099991
-5.6082339	14.2983999	103.8799973
-5.6082339	14.3472004	102.9399948
-5.6082339	14.3959999	101.3600006
-5.6082339	14.4448004	101.2099991
-5.6082339	14.4935999	102.6699982
-5.6082339	14.5424004	102.9599991
-5.6082339	14.5911999	103.0100021
-5.6082339	14.6400003	104.1699982
-5.6082339	14.6887999	105.3300018
-5.6082339	14.7376003	102.5199966
-5.6082339	14.7863998	99.4000015
-5.6082339	14.8352003	103.0000000
-5.6082339	14.8839998	99.6599960
-5.6082339	14.9328003	102.4799957
-5.6082339	14.9815998	101.9899979
-5.6082339	15.0304003	102.4000015
-5.6020980	0.3416000	105.3699951
-5.6020980	0.3904000	105.2099991

-5.6020980	0.4392000	107.1500015
-5.6020980	0.4880000	103.4599991
-5.6020980	0.5368000	106.4499969
-5.6020980	0.5856000	108.0499954
-5.6020980	0.6344000	106.0499954
-5.6020980	0.6832000	104.0499954
-5.6020980	0.7320000	105.8300018
-5.6020980	0.7808000	106.8600006
-5.6020980	0.8296000	102.0100021
-5.6020980	0.8784000	104.4199982
-5.6020980	0.9272000	101.3300018
-5.6020980	0.9760000	104.2399979
-5.6020980	1.0247999	105.0499954
-5.6020980	1.0735999	103.6500015
-5.6020980	1.1223999	102.2900009
-5.6020980	1.1711999	105.7999954
-5.6020980	1.2200000	105.8600006
-5.6020980	1.2688000	101.6100006
-5.6020980	1.3176000	104.8099976
-5.6020980	1.3664000	106.7299957
-5.6020980	1.4152000	107.8099976
-5.6020980	1.4640000	102.3699951
-5.6020980	1.5128000	107.6999969
-5.6020980	1.5616000	105.3499985
-5.6020980	1.6104000	105.0699997
-5.6020980	1.6592000	107.1800003
-5.6020980	1.7079999	109.4399948
-5.6020980	1.7567999	106.0100021
-5.6020980	1.8055999	106.2999954
-5.6020980	1.8543999	106.1800003
-5.6020980	1.9031999	104.1800003
-5.6020980	1.9519999	103.9399948
-5.6020980	2.0007999	106.5499954
-5.6020980	2.0495999	110.0100021
-5.6020980	2.0983999	106.6599960
-5.6020980	2.1471999	104.5699997
-5.6020980	2.1959999	103.4399948
-5.6020980	2.2447999	105.7999954
-5.6020980	2.2935998	105.8799973
-5.6020980	2.3423998	106.1899948
-5.6020980	2.3912001	108.2799988
-5.6020980	2.4400001	107.6899948
-5.6020980	2.4888000	104.2699966
-5.6020980	2.5376000	103.7500000
-5.6020980	2.5864000	104.6500015
-5.6020980	2.6352000	105.0000000
-5.6020980	2.6840000	104.4599991
-5.6020980	2.7328000	101.4799957
-5.6020980	2.7816000	103.7500000
-5.6020980	2.8304000	105.7900009
-5.6020980	2.8792000	107.0699997
-5.6020980	2.9280000	105.2999954
-5.6020980	2.9768000	105.5400009
-5.6020980	3.0256000	105.4700012
-5.6020980	3.0743999	99.5100021
-5.6020980	3.1231999	99.9799957
-5.6020980	3.1719999	100.8600006
-5.6020980	3.2207999	104.6299973

-5.6020980	3.2695999	103.4799957
-5.6020980	3.3183999	104.5999985
-5.6020980	3.3671999	104.5999985
-5.6020980	3.4159999	105.4700012
-5.6020980	3.4647999	103.6399994
-5.6020980	3.5135999	104.2699966
-5.6020980	3.5623999	105.3300018
-5.6020980	3.6111999	105.1899948
-5.6020980	3.6599998	104.5699997
-5.6020980	3.7087998	106.2299957
-5.6020980	3.7575998	107.8199997
-5.6020980	3.8063998	105.8799973
-5.6020980	3.8551998	104.0499954
-5.6020980	3.9039998	103.9499969
-5.6020980	3.9527998	102.6699982
-5.6020980	4.0016003	104.1199951
-5.6020980	4.0503998	103.3399963
-5.6020980	4.0991998	102.6899948
-5.6020980	4.1479998	101.7999954
-5.6020980	4.1967998	103.1399994
-5.6020980	4.2455997	104.3499985
-5.6020980	4.2943997	103.1299973
-5.6020980	4.3431997	108.0800018
-5.6020980	4.3919997	108.9599991
-5.6020980	4.4407997	109.7099991
-5.6020980	4.4895997	106.6800003
-5.6020980	4.5383997	106.6899948
-5.6020980	4.5871997	108.5499954
-5.6020980	4.6359997	112.6800003
-5.6020980	4.6847997	117.1899948
-5.6020980	4.7336001	116.0999985
-5.6020980	4.7824001	111.4300003
-5.6020980	4.8312001	109.2699966
-5.6020980	4.8800001	108.4399948
-5.6020980	4.9288001	106.9799957
-5.6020980	4.9776001	107.1199951
-5.6020980	5.0264001	105.7900009
-5.6020980	5.0752001	105.3699951
-5.6020980	5.1240001	107.3399963
-5.6020980	5.1728001	104.0499954
-5.6020980	5.2216001	105.5400009
-5.6020980	5.2704000	105.1599960
-5.6020980	5.3192000	102.8899994
-5.6020980	5.3680000	102.0999985
-5.6020980	5.4168000	101.8600006
-5.6020980	5.4656000	100.7299957
-5.6020980	5.5144000	102.1999969
-5.6020980	5.5632000	102.5899963
-5.6020980	5.6120000	101.0199966
-5.6020980	5.6608000	101.7099991
-5.6020980	5.7096000	100.7600021
-5.6020980	5.7584000	101.6899948
-5.6020980	5.8072000	103.6800003
-5.6020980	5.8559999	104.1699982
-5.6020980	5.9047999	100.5899963
-5.6020980	5.9535999	103.0499954
-5.6020980	6.0023999	103.1899948
-5.6020980	6.0511999	102.6699982

-5.6020980	6.0999999	106.6100006
-5.6020980	6.1487999	106.0699997
-5.6020980	6.1975999	103.5199966
-5.6020980	6.2463999	103.8600006
-5.6020980	6.2951999	104.4599991
-5.6020980	6.3439999	106.2299957
-5.6020980	6.3927999	103.8499985
-5.6020980	6.4415998	101.2699966
-5.6020980	6.4903998	101.6399994
-5.6020980	6.5391998	101.9499969
-5.6020980	6.5879998	100.8099976
-5.6020980	6.6367998	101.6800003
-5.6020980	6.6855998	104.8300018
-5.6020980	6.7343998	103.4599991
-5.6020980	6.7831998	104.5000000
-5.6020980	6.8319998	103.4300003
-5.6020980	6.8807998	105.0400009
-5.6020980	6.9295998	104.7699966
-5.6020980	6.9783998	104.0599976
-5.6020980	7.0271997	102.3300018
-5.6020980	7.0759997	103.0299988
-5.6020980	7.1247997	103.4099960
-5.6020980	7.1735997	103.5899963
-5.6020980	7.2223997	103.1399994
-5.6020980	7.2711997	104.4099960
-5.6020980	7.3199997	103.7500000
-5.6020980	7.3687997	99.7200012
-5.6020980	7.4175997	101.7600021
-5.6020980	7.4663997	105.6800003
-5.6020980	7.5151997	105.6100006
-5.6020980	7.5639997	103.0899963
-5.6020980	7.6127996	98.4899979
-5.6020980	7.6615996	98.4099960
-5.6020980	7.7103996	101.2200012
-5.6020980	7.7591996	104.1299973
-5.6020980	7.8079996	105.9099960
-5.6020980	7.8567996	104.8499985
-5.6020980	7.9056001	105.9599991
-5.6020980	7.9544001	105.5899963
-5.6020980	8.0032005	104.6500015
-5.6020980	8.0520000	101.7600021
-5.6020980	8.1008005	102.8799973
-5.6020980	8.1496000	102.0299988
-5.6020980	8.1984005	101.2699966
-5.6020980	8.2472000	104.9700012
-5.6020980	8.2960005	107.5000000
-5.6020980	8.3448000	109.0299988
-5.6020980	8.3936005	105.8899994
-5.6020980	8.4424000	102.5400009
-5.6020980	8.4912004	103.5199966
-5.6020980	8.5400000	105.2699966
-5.6020980	8.5888004	106.6100006
-5.6020980	8.6375999	106.2099991
-5.6020980	8.6864004	101.9599991
-5.6020980	8.7351999	104.2399979
-5.6020980	8.7840004	105.1899948
-5.6020980	8.8327999	99.6100006
-5.6020980	8.8816004	100.7299957

-5.6020980	8.9303999	102.0899963
-5.6020980	8.9792004	104.0999985
-5.6020980	9.0279999	103.5899963
-5.6020980	9.0768003	103.8300018
-5.6020980	9.1255999	100.3699951
-5.6020980	9.1744003	106.1999969
-5.6020980	9.2231998	106.2399979
-5.6020980	9.2720003	104.3199997
-5.6020980	9.3207998	105.7200012
-5.6020980	9.3696003	105.5199966
-5.6020980	9.4184008	102.3199997
-5.6020980	9.4672003	102.7900009
-5.6020980	9.5160007	103.3199997
-5.6020980	9.5648003	104.8899994
-5.6020980	9.6136007	103.0699997
-5.6020980	9.6624002	102.6100006
-5.6020980	9.7112007	104.7999954
-5.6020980	9.7600002	105.3399963
-5.6020980	9.8088007	103.2999954
-5.6020980	9.8576002	101.4300003
-5.6020980	9.9064007	105.6899948
-5.6020980	9.9552002	107.6800003
-5.6020980	10.0040007	107.2500000
-5.6020980	10.0528002	104.9199982
-5.6020980	10.1016006	103.8600006
-5.6020980	10.1504002	104.2500000
-5.6020980	10.1992006	102.1800003
-5.6020980	10.2480001	101.2299957
-5.6020980	10.2968006	105.4700012
-5.6020980	10.3456001	106.7900009
-5.6020980	10.3944006	107.3099976
-5.6020980	10.4432001	105.9899979
-5.6020980	10.4920006	107.0100021
-5.6020980	10.5408001	107.1399994
-5.6020980	10.5896006	105.8899994
-5.6020980	10.6384001	102.6299973
-5.6020980	10.6872005	97.6999969
-5.6020980	10.7360001	101.4799957
-5.6020980	10.7848005	105.4399948
-5.6020980	10.8336000	105.1299973
-5.6020980	10.8824005	104.4300003
-5.6020980	10.9312000	100.9799957
-5.6020980	10.9800005	100.3499985
-5.6020980	11.0288000	103.2299957
-5.6020980	11.0776005	105.3399963
-5.6020980	11.1264000	101.1100006
-5.6020980	11.1752005	100.3499985
-5.6020980	11.2240000	104.8099976
-5.6020980	11.2728004	102.5000000
-5.6020980	11.3216000	102.6599960
-5.6020980	11.3704004	101.3699951
-5.6020980	11.4191999	103.5199966
-5.6020980	11.4680004	100.7399979
-5.6020980	11.5167999	99.4099960
-5.6020980	11.5656004	100.8399963
-5.6020980	11.6143999	102.5299988
-5.6020980	11.6632004	103.0899963
-5.6020980	11.7119999	104.2500000

-5.6020980	11.7608004	104.6999969
-5.6020980	11.8095999	104.9000015
-5.6020980	11.8584003	102.2999954
-5.6020980	11.9071999	100.6399994
-5.6020980	11.9560003	101.0299988
-5.6020980	12.0047998	104.8799973
-5.6020980	12.0536003	106.2200012
-5.6020980	12.1023998	104.2900009
-5.6020980	12.1512003	101.4000015
-5.6020980	12.1999998	101.0400009
-5.6020980	12.2488003	104.3600006
-5.6020980	12.2975998	106.7200012
-5.6020980	12.3464003	106.7600021
-5.6020980	12.3951998	104.9300003
-5.6020980	12.4440002	102.8699951
-5.6020980	12.4927998	103.1999969
-5.6020980	12.5416002	105.7200012
-5.6020980	12.5904007	104.9199982
-5.6020980	12.6392002	97.6500015
-5.6020980	12.6880007	101.6699982
-5.6020980	12.7368002	105.1699982
-5.6020980	12.7856007	106.0800018
-5.6020980	12.8344002	105.7399979
-5.6020980	12.8832006	103.0499954
-5.6020980	12.9320002	105.4599991
-5.6020980	12.9808006	105.2600021
-5.6020980	13.0296001	104.8300018
-5.6020980	13.0784006	104.8399963
-5.6020980	13.1272001	103.6699982
-5.6020980	13.1760006	103.4300003
-5.6020980	13.2248001	99.7500000
-5.6020980	13.2736006	100.3699951
-5.6020980	13.3224001	98.1299973
-5.6020980	13.3712006	99.6999969
-5.6020980	13.4200001	102.3099976
-5.6020980	13.4688005	102.4700012
-5.6020980	13.5176001	103.6599960
-5.6020980	13.5664005	103.7399979
-5.6020980	13.6152000	103.4199982
-5.6020980	13.6640005	102.3099976
-5.6020980	13.7128000	101.6699982
-5.6020980	13.7616005	103.2500000
-5.6020980	13.8104000	102.8899994
-5.6020980	13.8592005	102.9499969
-5.6020980	13.9080000	103.5999985
-5.6020980	13.9568005	102.2999954
-5.6020980	14.0056000	101.3300018
-5.6020980	14.0544004	104.1999969
-5.6020980	14.1032000	103.7299957
-5.6020980	14.1520004	100.6199951
-5.6020980	14.2007999	99.3099976
-5.6020980	14.2496004	101.5999985
-5.6020980	14.2983999	102.8499985
-5.6020980	14.3472004	101.4300003
-5.6020980	14.3959999	101.0499954
-5.6020980	14.4448004	100.9300003
-5.6020980	14.4935999	103.0800018
-5.6020980	14.5424004	103.3499985

-5.6020980	14.5911999	103.1899948
-5.6020980	14.6400003	103.0699997
-5.6020980	14.6887999	105.3399963
-5.6020980	14.7376003	102.1299973
-5.6020980	14.7863998	100.3899994
-5.6020980	14.8352003	103.0499954
-5.6020980	14.8839998	98.3399963
-5.6020980	14.9328003	101.8399963
-5.6020980	14.9815998	102.1800003
-5.6020980	15.0304003	102.1800003
-5.5959620	0.3416000	106.2299957
-5.5959620	0.3904000	104.6299973
-5.5959620	0.4392000	106.9099960
-5.5959620	0.4880000	101.6599960
-5.5959620	0.5368000	106.4099960
-5.5959620	0.5856000	106.6500015
-5.5959620	0.6344000	104.0400009
-5.5959620	0.6832000	103.7099991
-5.5959620	0.7320000	106.1500015
-5.5959620	0.7808000	106.8399963
-5.5959620	0.8296000	104.2900009
-5.5959620	0.8784000	105.6699982
-5.5959620	0.9272000	102.0800018
-5.5959620	0.9760000	104.2799988
-5.5959620	1.0247999	104.9199982
-5.5959620	1.0735999	104.6800003
-5.5959620	1.1223999	104.0000000
-5.5959620	1.1711999	105.4399948
-5.5959620	1.2200000	104.8300018
-5.5959620	1.2688000	101.6599960
-5.5959620	1.3176000	104.4599991
-5.5959620	1.3664000	105.8399963
-5.5959620	1.4152000	107.0599976
-5.5959620	1.4640000	102.2799988
-5.5959620	1.5128000	107.3399963
-5.5959620	1.5616000	105.8799973
-5.5959620	1.6104000	104.8199997
-5.5959620	1.6592000	106.6199951
-5.5959620	1.7079999	109.3699951
-5.5959620	1.7567999	105.2999954
-5.5959620	1.8055999	105.2600021
-5.5959620	1.8543999	106.0100021
-5.5959620	1.9031999	104.2099991
-5.5959620	1.9519999	103.9300003
-5.5959620	2.0007999	106.0400009
-5.5959620	2.0495999	109.6899948
-5.5959620	2.0983999	107.2099991
-5.5959620	2.1471999	105.0499954
-5.5959620	2.1959999	104.1800003
-5.5959620	2.2447999	106.5100021
-5.5959620	2.2935998	105.5199966
-5.5959620	2.3423998	105.9899979
-5.5959620	2.3912001	107.7699966
-5.5959620	2.4400001	107.0100021
-5.5959620	2.4888000	104.6599960
-5.5959620	2.5376000	104.4000015
-5.5959620	2.5864000	105.0999985
-5.5959620	2.6352000	105.8499985

-5.5959620	2.6840000	104.9499969
-5.5959620	2.7328000	102.7999954
-5.5959620	2.7816000	104.1100006
-5.5959620	2.8304000	105.6999969
-5.5959620	2.8792000	106.6899948
-5.5959620	2.9280000	103.5499954
-5.5959620	2.9768000	104.4300003
-5.5959620	3.0256000	105.2399979
-5.5959620	3.0743999	99.4099960
-5.5959620	3.1231999	99.6299973
-5.5959620	3.1719999	99.7699966
-5.5959620	3.2207999	104.6899948
-5.5959620	3.2695999	103.5599976
-5.5959620	3.3183999	103.8499985
-5.5959620	3.3671999	104.6299973
-5.5959620	3.4159999	105.6899948
-5.5959620	3.4647999	103.7999954
-5.5959620	3.5135999	104.5499954
-5.5959620	3.5623999	106.0999985
-5.5959620	3.6111999	105.6699982
-5.5959620	3.6599998	103.9499969
-5.5959620	3.7087998	106.3899994
-5.5959620	3.7575998	108.0199966
-5.5959620	3.8063998	105.4599991
-5.5959620	3.8551998	103.8199997
-5.5959620	3.9039998	104.5899963
-5.5959620	3.9527998	102.0499954
-5.5959620	4.0016003	104.2099991
-5.5959620	4.0503998	104.0000000
-5.5959620	4.0991998	103.5999985
-5.5959620	4.1479998	102.6399994
-5.5959620	4.1967998	103.6199951
-5.5959620	4.2455997	104.7999954
-5.5959620	4.2943997	104.2099991
-5.5959620	4.3431997	108.4399948
-5.5959620	4.3919997	108.4199982
-5.5959620	4.4407997	109.8799973
-5.5959620	4.4895997	107.2699966
-5.5959620	4.5383997	107.1199951
-5.5959620	4.5871997	108.2699966
-5.5959620	4.6359997	113.3199997
-5.5959620	4.6847997	117.7799988
-5.5959620	4.7336001	116.4799957
-5.5959620	4.7824001	111.5100021
-5.5959620	4.8312001	109.0299988
-5.5959620	4.8800001	108.5999985
-5.5959620	4.9288001	107.2299957
-5.5959620	4.9776001	107.2399979
-5.5959620	5.0264001	105.5400009
-5.5959620	5.0752001	105.7299957
-5.5959620	5.1240001	107.5400009
-5.5959620	5.1728001	104.8799973
-5.5959620	5.2216001	105.2900009
-5.5959620	5.2704000	104.1999969
-5.5959620	5.3192000	102.5499954
-5.5959620	5.3680000	102.7500000
-5.5959620	5.4168000	102.3300018
-5.5959620	5.4656000	101.4300003

-5.5959620	5.5144000	103.3499985
-5.5959620	5.5632000	103.5599976
-5.5959620	5.6120000	101.9899979
-5.5959620	5.6608000	102.3199997
-5.5959620	5.7096000	101.4300003
-5.5959620	5.7584000	101.4099960
-5.5959620	5.8072000	103.3699951
-5.5959620	5.8559999	104.4199982
-5.5959620	5.9047999	100.0199966
-5.5959620	5.9535999	102.9099960
-5.5959620	6.0023999	103.3499985
-5.5959620	6.0511999	104.3699951
-5.5959620	6.0999999	107.7600021
-5.5959620	6.1487999	106.8699951
-5.5959620	6.1975999	103.7900009
-5.5959620	6.2463999	104.4300003
-5.5959620	6.2951999	105.6599960
-5.5959620	6.3439999	106.5199966
-5.5959620	6.3927999	103.7799988
-5.5959620	6.4415998	101.4300003
-5.5959620	6.4903998	100.9199982
-5.5959620	6.5391998	101.2099991
-5.5959620	6.5879998	100.3099976
-5.5959620	6.6367998	102.4399948
-5.5959620	6.6855998	105.4399948
-5.5959620	6.7343998	104.1399994
-5.5959620	6.7831998	104.6199951
-5.5959620	6.8319998	103.5699997
-5.5959620	6.8807998	105.5199966
-5.5959620	6.9295998	104.7299957
-5.5959620	6.9783998	103.6199951
-5.5959620	7.0271997	101.5499954
-5.5959620	7.0759997	102.7600021
-5.5959620	7.1247997	103.8499985
-5.5959620	7.1735997	103.2999954
-5.5959620	7.2223997	103.3099976
-5.5959620	7.2711997	104.2099991
-5.5959620	7.3199997	103.5899963
-5.5959620	7.3687997	99.7099991
-5.5959620	7.4175997	101.1299973
-5.5959620	7.4663997	104.8699951
-5.5959620	7.5151997	105.0999985
-5.5959620	7.5639997	103.0499954
-5.5959620	7.6127996	99.4799957
-5.5959620	7.6615996	98.5400009
-5.5959620	7.7103996	101.7500000
-5.5959620	7.7591996	104.2999954
-5.5959620	7.8079996	105.7900009
-5.5959620	7.8567996	105.8099976
-5.5959620	7.9056001	107.0699997
-5.5959620	7.9544001	106.2699966
-5.5959620	8.0032005	104.9099960
-5.5959620	8.0520000	101.4199982
-5.5959620	8.1008005	101.6500015
-5.5959620	8.1496000	101.7999954
-5.5959620	8.1984005	101.7500000
-5.5959620	8.2472000	105.4399948
-5.5959620	8.2960005	108.1800003

-5.5959620	8.3448000	109.1800003
-5.5959620	8.3936005	106.1599960
-5.5959620	8.4424000	102.0499954
-5.5959620	8.4912004	103.5400009
-5.5959620	8.5400000	105.5499954
-5.5959620	8.5888004	106.5899963
-5.5959620	8.6375999	105.9099960
-5.5959620	8.6864004	100.6399994
-5.5959620	8.7351999	104.6199951
-5.5959620	8.7840004	105.1100006
-5.5959620	8.8327999	99.6399994
-5.5959620	8.8816004	101.8399963
-5.5959620	8.9303999	102.5499954
-5.5959620	8.9792004	104.5499954
-5.5959620	9.0279999	104.6199951
-5.5959620	9.0768003	103.5100021
-5.5959620	9.1255999	100.9599991
-5.5959620	9.1744003	105.9499969
-5.5959620	9.2231998	105.1599960
-5.5959620	9.2720003	103.1999969
-5.5959620	9.3207998	106.5199966
-5.5959620	9.3696003	105.6500015
-5.5959620	9.4184008	102.3399963
-5.5959620	9.4672003	103.4300003
-5.5959620	9.5160007	102.1699982
-5.5959620	9.5648003	105.0199966
-5.5959620	9.6136007	103.2900009
-5.5959620	9.6624002	102.9799957
-5.5959620	9.7112007	105.2799988
-5.5959620	9.7600002	106.0000000
-5.5959620	9.8088007	104.1299973
-5.5959620	9.8576002	102.7099991
-5.5959620	9.9064007	105.6599960
-5.5959620	9.9552002	107.3099976
-5.5959620	10.0040007	107.4499969
-5.5959620	10.0528002	104.9700012
-5.5959620	10.1016006	103.7299957
-5.5959620	10.1504002	105.0199966
-5.5959620	10.1992006	101.8499985
-5.5959620	10.2480001	101.6599960
-5.5959620	10.2968006	105.1100006
-5.5959620	10.3456001	105.6999969
-5.5959620	10.3944006	106.2299957
-5.5959620	10.4432001	106.2600021
-5.5959620	10.4920006	107.1999969
-5.5959620	10.5408001	107.3899994
-5.5959620	10.5896006	105.2799988
-5.5959620	10.6384001	101.3099976
-5.5959620	10.6872005	96.6100006
-5.5959620	10.7360001	100.2600021
-5.5959620	10.7848005	104.7999954
-5.5959620	10.8336000	105.0999985
-5.5959620	10.8824005	103.4899979
-5.5959620	10.9312000	100.1500015
-5.5959620	10.9800005	99.0899963
-5.5959620	11.0288000	101.8199997
-5.5959620	11.0776005	103.6399994
-5.5959620	11.1264000	100.1399994

-5.5959620	11.1752005	100.5699997
-5.5959620	11.2240000	104.4700012
-5.5959620	11.2728004	102.4300003
-5.5959620	11.3216000	102.5499954
-5.5959620	11.3704004	100.4599991
-5.5959620	11.4191999	103.5699997
-5.5959620	11.4680004	101.2500000
-5.5959620	11.5167999	100.2099991
-5.5959620	11.5656004	100.2099991
-5.5959620	11.6143999	102.3199997
-5.5959620	11.6632004	103.7999954
-5.5959620	11.7119999	105.1999969
-5.5959620	11.7608004	104.8799973
-5.5959620	11.8095999	104.8899994
-5.5959620	11.8584003	101.3199997
-5.5959620	11.9071999	100.1699982
-5.5959620	11.9560003	100.8300018
-5.5959620	12.0047998	104.6699982
-5.5959620	12.0536003	106.1199951
-5.5959620	12.1023998	104.8099976
-5.5959620	12.1512003	102.2299957
-5.5959620	12.1999998	101.6999969
-5.5959620	12.2488003	103.7900009
-5.5959620	12.2975998	106.7500000
-5.5959620	12.3464003	106.7900009
-5.5959620	12.3951998	105.1500015
-5.5959620	12.4440002	103.1399994
-5.5959620	12.4927998	102.6299973
-5.5959620	12.5416002	106.0400009
-5.5959620	12.5904007	104.9399948
-5.5959620	12.6392002	96.9700012
-5.5959620	12.6880007	102.0999985
-5.5959620	12.7368002	105.0699997
-5.5959620	12.7856007	105.8300018
-5.5959620	12.8344002	106.4499969
-5.5959620	12.8832006	104.6299973
-5.5959620	12.9320002	105.8099976
-5.5959620	12.9808006	104.9700012
-5.5959620	13.0296001	103.6999969
-5.5959620	13.0784006	104.2099991
-5.5959620	13.1272001	103.7399979
-5.5959620	13.1760006	103.0499954
-5.5959620	13.2248001	99.6500015
-5.5959620	13.2736006	100.5599976
-5.5959620	13.3224001	99.1199951
-5.5959620	13.3712006	99.9099960
-5.5959620	13.4200001	102.5800018
-5.5959620	13.4688005	102.1500015
-5.5959620	13.5176001	103.5100021
-5.5959620	13.5664005	104.0599976
-5.5959620	13.6152000	102.9599991
-5.5959620	13.6640005	102.1199951
-5.5959620	13.7128000	101.8199997
-5.5959620	13.7616005	103.2600021
-5.5959620	13.8104000	104.2200012
-5.5959620	13.8592005	104.0199966
-5.5959620	13.9080000	103.8300018
-5.5959620	13.9568005	101.8300018

-5.5959620	14.0056000	100.0699997
-5.5959620	14.0544004	104.4599991
-5.5959620	14.1032000	103.7600021
-5.5959620	14.1520004	99.8699951
-5.5959620	14.2007999	99.3699951
-5.5959620	14.2496004	101.5999985
-5.5959620	14.2983999	102.1299973
-5.5959620	14.3472004	100.9399948
-5.5959620	14.3959999	101.3600006
-5.5959620	14.4448004	101.1399994
-5.5959620	14.4935999	103.1899948
-5.5959620	14.5424004	103.3099976
-5.5959620	14.5911999	102.6999969
-5.5959620	14.6400003	101.6699982
-5.5959620	14.6887999	104.8199997
-5.5959620	14.7376003	101.8699951
-5.5959620	14.7863998	101.4899979
-5.5959620	14.8352003	103.7099991
-5.5959620	14.8839998	98.5499954
-5.5959620	14.9328003	101.7399979
-5.5959620	14.9815998	102.2200012
-5.5959620	15.0304003	101.9300003
-5.5898261	0.3416000	106.5299988
-5.5898261	0.3904000	103.0800018
-5.5898261	0.4392000	106.6399994
-5.5898261	0.4880000	100.2099991
-5.5898261	0.5368000	105.1100006
-5.5898261	0.5856000	105.2799988
-5.5898261	0.6344000	102.9599991
-5.5898261	0.6832000	103.8300018
-5.5898261	0.7320000	106.8699951
-5.5898261	0.7808000	106.7200012
-5.5898261	0.8296000	106.2399979
-5.5898261	0.8784000	106.9499969
-5.5898261	0.9272000	103.9899979
-5.5898261	0.9760000	105.2399979
-5.5898261	1.0247999	105.5999985
-5.5898261	1.0735999	105.7900009
-5.5898261	1.1223999	105.4700012
-5.5898261	1.1711999	104.7900009
-5.5898261	1.2200000	103.6599960
-5.5898261	1.2688000	101.9199982
-5.5898261	1.3176000	104.3799973
-5.5898261	1.3664000	105.1500015
-5.5898261	1.4152000	107.1800003
-5.5898261	1.4640000	104.1899948
-5.5898261	1.5128000	107.9700012
-5.5898261	1.5616000	106.5199966
-5.5898261	1.6104000	104.1500015
-5.5898261	1.6592000	106.9099960
-5.5898261	1.7079999	109.3600006
-5.5898261	1.7567999	105.5499954
-5.5898261	1.8055999	104.4099960
-5.5898261	1.8543999	106.0299988
-5.5898261	1.9031999	104.2699966
-5.5898261	1.9519999	103.4499969
-5.5898261	2.0007999	105.1399994
-5.5898261	2.0495999	109.3499985

-5.5898261	2.0983999	108.4000015
-5.5898261	2.1471999	106.3600006
-5.5898261	2.1959999	104.8499985
-5.5898261	2.2447999	107.0999985
-5.5898261	2.2935998	105.0199966
-5.5898261	2.3423998	105.2900009
-5.5898261	2.3912001	107.0699997
-5.5898261	2.4400001	106.3199997
-5.5898261	2.4888000	104.2500000
-5.5898261	2.5376000	104.4599991
-5.5898261	2.5864000	105.4599991
-5.5898261	2.6352000	106.5100021
-5.5898261	2.6840000	105.2399979
-5.5898261	2.7328000	104.2999954
-5.5898261	2.7816000	104.7699966
-5.5898261	2.8304000	105.5299988
-5.5898261	2.8792000	106.2699966
-5.5898261	2.9280000	101.5899963
-5.5898261	2.9768000	104.7399979
-5.5898261	3.0256000	106.2200012
-5.5898261	3.0743999	101.5599976
-5.5898261	3.1231999	100.0299988
-5.5898261	3.1719999	98.4599991
-5.5898261	3.2207999	104.0400009
-5.5898261	3.2695999	103.1599960
-5.5898261	3.3183999	102.7500000
-5.5898261	3.3671999	103.9000015
-5.5898261	3.4159999	106.0699997
-5.5898261	3.4647999	104.5400009
-5.5898261	3.5135999	104.8799973
-5.5898261	3.5623999	106.5599976
-5.5898261	3.6111999	105.9499969
-5.5898261	3.6599998	102.8399963
-5.5898261	3.7087998	106.4899979
-5.5898261	3.7575998	107.9899979
-5.5898261	3.8063998	104.9599991
-5.5898261	3.8551998	103.9899979
-5.5898261	3.9039998	105.0400009
-5.5898261	3.9527998	101.4399948
-5.5898261	4.0016003	104.7200012
-5.5898261	4.0503998	104.9700012
-5.5898261	4.0991998	104.2799988
-5.5898261	4.1479998	103.3600006
-5.5898261	4.1967998	103.5899963
-5.5898261	4.2455997	105.0199966
-5.5898261	4.2943997	105.6399994
-5.5898261	4.3431997	108.9799957
-5.5898261	4.3919997	108.0800018
-5.5898261	4.4407997	109.9199982
-5.5898261	4.4895997	107.9499969
-5.5898261	4.5383997	108.2699966
-5.5898261	4.5871997	108.2200012
-5.5898261	4.6359997	113.6599960
-5.5898261	4.6847997	117.8499985
-5.5898261	4.7336001	116.5299988
-5.5898261	4.7824001	111.5999985
-5.5898261	4.8312001	108.4700012
-5.5898261	4.8800001	107.8699951

-5.5898261	4.9288001	106.6199951
-5.5898261	4.9776001	106.9899979
-5.5898261	5.0264001	105.8600006
-5.5898261	5.0752001	105.7600021
-5.5898261	5.1240001	107.0699997
-5.5898261	5.1728001	105.0000000
-5.5898261	5.2216001	104.8499985
-5.5898261	5.2704000	103.5000000
-5.5898261	5.3192000	103.1999969
-5.5898261	5.3680000	103.2299957
-5.5898261	5.4168000	102.2799988
-5.5898261	5.4656000	102.7500000
-5.5898261	5.5144000	104.6899948
-5.5898261	5.5632000	104.8099976
-5.5898261	5.6120000	103.9499969
-5.5898261	5.6608000	103.6699982
-5.5898261	5.7096000	102.8499985
-5.5898261	5.7584000	102.2999954
-5.5898261	5.8072000	103.7200012
-5.5898261	5.8559999	104.4899979
-5.5898261	5.9047999	98.7799988
-5.5898261	5.9535999	102.1299973
-5.5898261	6.0023999	103.4499969
-5.5898261	6.0511999	105.8099976
-5.5898261	6.0999999	108.5699997
-5.5898261	6.1487999	107.6399994
-5.5898261	6.1975999	104.2200012
-5.5898261	6.2463999	104.9700012
-5.5898261	6.2951999	106.7399979
-5.5898261	6.3439999	106.8699951
-5.5898261	6.3927999	103.5100021
-5.5898261	6.4415998	101.5599976
-5.5898261	6.4903998	100.8499985
-5.5898261	6.5391998	100.2600021
-5.5898261	6.5879998	99.6599960
-5.5898261	6.6367998	103.0999985
-5.5898261	6.6855998	105.9399948
-5.5898261	6.7343998	104.4300003
-5.5898261	6.7831998	104.4399948
-5.5898261	6.8319998	103.5299988
-5.5898261	6.8807998	105.7200012
-5.5898261	6.9295998	104.7399979
-5.5898261	6.9783998	102.2699966
-5.5898261	7.0271997	101.2699966
-5.5898261	7.0759997	102.6100006
-5.5898261	7.1247997	104.4599991
-5.5898261	7.1735997	103.3799973
-5.5898261	7.2223997	104.0699997
-5.5898261	7.2711997	104.7099991
-5.5898261	7.3199997	104.0899963
-5.5898261	7.3687997	100.0599976
-5.5898261	7.4175997	101.0499954
-5.5898261	7.4663997	104.3899994
-5.5898261	7.5151997	104.6500015
-5.5898261	7.5639997	102.6399994
-5.5898261	7.6127996	100.4700012
-5.5898261	7.6615996	100.5100021
-5.5898261	7.7103996	103.1199951

-5.5898261	7.7591996	103.8799973
-5.5898261	7.8079996	105.4899979
-5.5898261	7.8567996	106.3399963
-5.5898261	7.9056001	108.0299988
-5.5898261	7.9544001	107.3300018
-5.5898261	8.0032005	105.4199982
-5.5898261	8.0520000	100.7699966
-5.5898261	8.1008005	100.4399948
-5.5898261	8.1496000	101.6899948
-5.5898261	8.1984005	101.8099976
-5.5898261	8.2472000	105.6800003
-5.5898261	8.2960005	108.6999969
-5.5898261	8.3448000	109.2099991
-5.5898261	8.3936005	106.6500015
-5.5898261	8.4424000	102.3399963
-5.5898261	8.4912004	104.0499954
-5.5898261	8.5400000	105.7200012
-5.5898261	8.5888004	106.6399994
-5.5898261	8.6375999	105.9199982
-5.5898261	8.6864004	99.1500015
-5.5898261	8.7351999	104.9399948
-5.5898261	8.7840004	105.1299973
-5.5898261	8.8327999	99.7900009
-5.5898261	8.8816004	102.9599991
-5.5898261	8.9303999	103.2500000
-5.5898261	8.9792004	104.9499969
-5.5898261	9.0279999	105.8699951
-5.5898261	9.0768003	103.4000015
-5.5898261	9.1255999	101.7500000
-5.5898261	9.1744003	105.7500000
-5.5898261	9.2231998	104.0599976
-5.5898261	9.2720003	102.1500015
-5.5898261	9.3207998	107.1699982
-5.5898261	9.3696003	105.4000015
-5.5898261	9.4184008	102.0299988
-5.5898261	9.4672003	103.4700012
-5.5898261	9.5160007	100.1899948
-5.5898261	9.5648003	104.5400009
-5.5898261	9.6136007	103.2900009
-5.5898261	9.6624002	103.5299988
-5.5898261	9.7112007	105.9799957
-5.5898261	9.7600002	106.6899948
-5.5898261	9.8088007	104.7999954
-5.5898261	9.8576002	103.3799973
-5.5898261	9.9064007	104.6599960
-5.5898261	9.9552002	106.2200012
-5.5898261	10.0040007	107.2699966
-5.5898261	10.0528002	105.0800018
-5.5898261	10.1016006	103.3699951
-5.5898261	10.1504002	105.2799988
-5.5898261	10.1992006	102.4000015
-5.5898261	10.2480001	102.2399979
-5.5898261	10.2968006	104.6599960
-5.5898261	10.3456001	103.7799988
-5.5898261	10.3944006	104.4899979
-5.5898261	10.4432001	106.0199966
-5.5898261	10.4920006	107.1199951
-5.5898261	10.5408001	107.2999954

-5.5898261	10.5896006	104.3899994
-5.5898261	10.6384001	100.4099960
-5.5898261	10.6872005	96.5299988
-5.5898261	10.7360001	99.9099960
-5.5898261	10.7848005	104.7600021
-5.5898261	10.8336000	105.3399963
-5.5898261	10.8824005	103.0100021
-5.5898261	10.9312000	99.5000000
-5.5898261	10.9800005	98.1999969
-5.5898261	11.0288000	101.0100021
-5.5898261	11.0776005	102.3499985
-5.5898261	11.1264000	99.8600006
-5.5898261	11.1752005	100.8799973
-5.5898261	11.2240000	103.9300003
-5.5898261	11.2728004	102.6399994
-5.5898261	11.3216000	102.5800018
-5.5898261	11.3704004	100.1100006
-5.5898261	11.4191999	103.4599991
-5.5898261	11.4680004	101.8499985
-5.5898261	11.5167999	101.5999985
-5.5898261	11.5656004	100.6100006
-5.5898261	11.6143999	102.4599991
-5.5898261	11.6632004	104.4499969
-5.5898261	11.7119999	105.7500000
-5.5898261	11.7608004	104.6299973
-5.5898261	11.8095999	104.0100021
-5.5898261	11.8584003	99.9000015
-5.5898261	11.9071999	99.7200012
-5.5898261	11.9560003	101.0599976
-5.5898261	12.0047998	104.1899948
-5.5898261	12.0536003	105.4899979
-5.5898261	12.1023998	104.7500000
-5.5898261	12.1512003	102.0299988
-5.5898261	12.1999998	101.9599991
-5.5898261	12.2488003	102.9099960
-5.5898261	12.2975998	106.9599991
-5.5898261	12.3464003	107.4300003
-5.5898261	12.3951998	105.4499969
-5.5898261	12.4440002	102.5199966
-5.5898261	12.4927998	101.6800003
-5.5898261	12.5416002	105.7999954
-5.5898261	12.5904007	104.6599960
-5.5898261	12.6392002	98.2900009
-5.5898261	12.6880007	102.8899994
-5.5898261	12.7368002	104.5199966
-5.5898261	12.7856007	104.8499985
-5.5898261	12.8344002	105.9899979
-5.5898261	12.8832006	105.2099991
-5.5898261	12.9320002	106.0299988
-5.5898261	12.9808006	104.8499985
-5.5898261	13.0296001	103.2299957
-5.5898261	13.0784006	104.2600021
-5.5898261	13.1272001	104.7799988
-5.5898261	13.1760006	103.7999954
-5.5898261	13.2248001	99.3099976
-5.5898261	13.2736006	100.6899948
-5.5898261	13.3224001	99.9499969
-5.5898261	13.3712006	99.9000015

-5.5898261	13.4200001	102.9300003
-5.5898261	13.4688005	103.0100021
-5.5898261	13.5176001	103.7600021
-5.5898261	13.5664005	104.6599960
-5.5898261	13.6152000	102.7200012
-5.5898261	13.6640005	102.4899979
-5.5898261	13.7128000	103.0299988
-5.5898261	13.7616005	103.3300018
-5.5898261	13.8104000	105.0499954
-5.5898261	13.8592005	104.9199982
-5.5898261	13.9080000	104.3399963
-5.5898261	13.9568005	101.6999969
-5.5898261	14.0056000	99.7999954
-5.5898261	14.0544004	103.9700012
-5.5898261	14.1032000	103.9099960
-5.5898261	14.1520004	100.1999969
-5.5898261	14.2007999	100.4399948
-5.5898261	14.2496004	101.3099976
-5.5898261	14.2983999	101.4399948
-5.5898261	14.3472004	100.8699951
-5.5898261	14.3959999	101.2699966
-5.5898261	14.4448004	101.4199982
-5.5898261	14.4935999	103.1599960
-5.5898261	14.5424004	103.2999954
-5.5898261	14.5911999	102.9499969
-5.5898261	14.6400003	100.1699982
-5.5898261	14.6887999	103.8199997
-5.5898261	14.7376003	101.1800003
-5.5898261	14.7863998	102.4899979
-5.5898261	14.8352003	104.5999985
-5.5898261	14.8839998	99.3699951
-5.5898261	14.9328003	101.7299957
-5.5898261	14.9815998	102.4000015
-5.5898261	15.0304003	102.2399979
-5.5836902	0.3416000	106.5299988
-5.5836902	0.3904000	103.4700012
-5.5836902	0.4392000	106.5999985
-5.5836902	0.4880000	99.8300018
-5.5836902	0.5368000	103.7500000
-5.5836902	0.5856000	103.9899979
-5.5836902	0.6344000	101.9899979
-5.5836902	0.6832000	103.7600021
-5.5836902	0.7320000	106.9000015
-5.5836902	0.7808000	106.4399948
-5.5836902	0.8296000	107.8899994
-5.5836902	0.8784000	107.9099960
-5.5836902	0.9272000	106.0000000
-5.5836902	0.9760000	106.8099976
-5.5836902	1.0247999	107.0899963
-5.5836902	1.0735999	107.3699951
-5.5836902	1.1223999	106.8199997
-5.5836902	1.1711999	104.7799988
-5.5836902	1.2200000	103.0599976
-5.5836902	1.2688000	102.3399963
-5.5836902	1.3176000	104.5599976
-5.5836902	1.3664000	104.6800003
-5.5836902	1.4152000	107.2500000
-5.5836902	1.4640000	105.8499985

-5.5836902	1.5128000	107.8799973
-5.5836902	1.5616000	107.1100006
-5.5836902	1.6104000	103.2699966
-5.5836902	1.6592000	106.6800003
-5.5836902	1.7079999	108.7099991
-5.5836902	1.7567999	105.1100006
-5.5836902	1.8055999	103.6299973
-5.5836902	1.8543999	106.4300003
-5.5836902	1.9031999	104.0000000
-5.5836902	1.9519999	102.9599991
-5.5836902	2.0007999	104.2099991
-5.5836902	2.0495999	108.7299957
-5.5836902	2.0983999	109.1699982
-5.5836902	2.1471999	107.6500015
-5.5836902	2.1959999	105.6100006
-5.5836902	2.2447999	105.9899979
-5.5836902	2.2935998	103.9599991
-5.5836902	2.3423998	104.9899979
-5.5836902	2.3912001	106.5800018
-5.5836902	2.4400001	105.4700012
-5.5836902	2.4888000	103.5499954
-5.5836902	2.5376000	103.4399948
-5.5836902	2.5864000	105.1899948
-5.5836902	2.6352000	106.6699982
-5.5836902	2.6840000	104.6699982
-5.5836902	2.7328000	105.0400009
-5.5836902	2.7816000	104.5899963
-5.5836902	2.8304000	105.0400009
-5.5836902	2.8792000	105.5699997
-5.5836902	2.9280000	100.8499985
-5.5836902	2.9768000	105.1199951
-5.5836902	3.0256000	106.3300018
-5.5836902	3.0743999	102.6199951
-5.5836902	3.1231999	100.0199966
-5.5836902	3.1719999	98.2999954
-5.5836902	3.2207999	104.2600021
-5.5836902	3.2695999	103.5000000
-5.5836902	3.3183999	101.9599991
-5.5836902	3.3671999	102.7799988
-5.5836902	3.4159999	105.9399948
-5.5836902	3.4647999	104.6399994
-5.5836902	3.5135999	104.9000015
-5.5836902	3.5623999	106.5699997
-5.5836902	3.6111999	106.2099991
-5.5836902	3.6599998	102.3099976
-5.5836902	3.7087998	106.0499954
-5.5836902	3.7575998	107.3799973
-5.5836902	3.8063998	104.1899948
-5.5836902	3.8551998	103.9399948
-5.5836902	3.9039998	105.1800003
-5.5836902	3.9527998	100.8899994
-5.5836902	4.0016003	104.9300003
-5.5836902	4.0503998	105.0599976
-5.5836902	4.0991998	104.6199951
-5.5836902	4.1479998	104.1699982
-5.5836902	4.1967998	103.6599960
-5.5836902	4.2455997	104.2699966
-5.5836902	4.2943997	107.0299988

-5.5836902	4.3431997	109.1299973
-5.5836902	4.3919997	107.3099976
-5.5836902	4.4407997	109.3099976
-5.5836902	4.4895997	107.7999954
-5.5836902	4.5383997	108.0899963
-5.5836902	4.5871997	107.1800003
-5.5836902	4.6359997	113.5299988
-5.5836902	4.6847997	117.7299957
-5.5836902	4.7336001	116.4499969
-5.5836902	4.7824001	111.5000000
-5.5836902	4.8312001	107.6100006
-5.5836902	4.8800001	107.2099991
-5.5836902	4.9288001	106.0699997
-5.5836902	4.9776001	106.5400009
-5.5836902	5.0264001	106.0599976
-5.5836902	5.0752001	105.6100006
-5.5836902	5.1240001	106.1299973
-5.5836902	5.1728001	103.6599960
-5.5836902	5.2216001	104.1299973
-5.5836902	5.2704000	102.7200012
-5.5836902	5.3192000	103.9300003
-5.5836902	5.3680000	104.1899948
-5.5836902	5.4168000	102.6299973
-5.5836902	5.4656000	104.2999954
-5.5836902	5.5144000	106.0400009
-5.5836902	5.5632000	105.7500000
-5.5836902	5.6120000	105.1999969
-5.5836902	5.6608000	104.7099991
-5.5836902	5.7096000	104.4199982
-5.5836902	5.7584000	103.1999969
-5.5836902	5.8072000	103.0999985
-5.5836902	5.8559999	103.3199997
-5.5836902	5.9047999	98.5199966
-5.5836902	5.9535999	101.4799957
-5.5836902	6.0023999	103.5499954
-5.5836902	6.0511999	106.8300018
-5.5836902	6.0999999	108.7099991
-5.5836902	6.1487999	107.5800018
-5.5836902	6.1975999	104.2600021
-5.5836902	6.2463999	104.9199982
-5.5836902	6.2951999	106.9000015
-5.5836902	6.3439999	106.5000000
-5.5836902	6.3927999	102.9399948
-5.5836902	6.4415998	102.3600006
-5.5836902	6.4903998	101.2699966
-5.5836902	6.5391998	99.7099991
-5.5836902	6.5879998	98.9399948
-5.5836902	6.6367998	102.9499969
-5.5836902	6.6855998	105.9599991
-5.5836902	6.7343998	104.5100021
-5.5836902	6.7831998	104.5299988
-5.5836902	6.8319998	103.4300003
-5.5836902	6.8807998	105.5400009
-5.5836902	6.9295998	104.2999954
-5.5836902	6.9783998	100.6899948
-5.5836902	7.0271997	101.0100021
-5.5836902	7.0759997	102.7299957
-5.5836902	7.1247997	103.9899979

-5.5836902	7.1735997	103.0100021
-5.5836902	7.2223997	104.7900009
-5.5836902	7.2711997	105.1699982
-5.5836902	7.3199997	104.2099991
-5.5836902	7.3687997	100.9099960
-5.5836902	7.4175997	101.1800003
-5.5836902	7.4663997	104.1599960
-5.5836902	7.5151997	104.7099991
-5.5836902	7.5639997	103.1500015
-5.5836902	7.6127996	101.9599991
-5.5836902	7.6615996	102.1699982
-5.5836902	7.7103996	103.6999969
-5.5836902	7.7591996	103.0999985
-5.5836902	7.8079996	104.9000015
-5.5836902	7.8567996	106.5499954
-5.5836902	7.9056001	108.2600021
-5.5836902	7.9544001	107.0100021
-5.5836902	8.0032005	105.5699997
-5.5836902	8.0520000	100.6699982
-5.5836902	8.1008005	99.8300018
-5.5836902	8.1496000	101.6100006
-5.5836902	8.1984005	102.3499985
-5.5836902	8.2472000	106.3799973
-5.5836902	8.2960005	108.6999969
-5.5836902	8.3448000	108.5999985
-5.5836902	8.3936005	106.5499954
-5.5836902	8.4424000	102.4300003
-5.5836902	8.4912004	105.0800018
-5.5836902	8.5400000	106.7799988
-5.5836902	8.5888004	106.8199997
-5.5836902	8.6375999	105.4899979
-5.5836902	8.6864004	99.4199982
-5.5836902	8.7351999	104.5299988
-5.5836902	8.7840004	104.7500000
-5.5836902	8.8327999	101.1999969
-5.5836902	8.8816004	103.7900009
-5.5836902	8.9303999	103.7900009
-5.5836902	8.9792004	104.7900009
-5.5836902	9.0279999	105.2999954
-5.5836902	9.0768003	102.8799973
-5.5836902	9.1255999	102.6399994
-5.5836902	9.1744003	105.4700012
-5.5836902	9.2231998	102.8499985
-5.5836902	9.2720003	101.4599991
-5.5836902	9.3207998	106.8600006
-5.5836902	9.3696003	104.3199997
-5.5836902	9.4184008	101.5000000
-5.5836902	9.4672003	102.9199982
-5.5836902	9.5160007	99.8499985
-5.5836902	9.5648003	103.9899979
-5.5836902	9.6136007	103.2399979
-5.5836902	9.6624002	103.6500015
-5.5836902	9.7112007	106.3499985
-5.5836902	9.7600002	107.2200012
-5.5836902	9.8088007	104.8300018
-5.5836902	9.8576002	103.5100021
-5.5836902	9.9064007	103.2600021
-5.5836902	9.9552002	104.9099960

-5.5836902	10.0040007	107.0400009
-5.5836902	10.0528002	105.1399994
-5.5836902	10.1016006	102.7299957
-5.5836902	10.1504002	105.3899994
-5.5836902	10.1992006	102.7699966
-5.5836902	10.2480001	102.3099976
-5.5836902	10.2968006	103.8499985
-5.5836902	10.3456001	102.0899963
-5.5836902	10.3944006	102.2200012
-5.5836902	10.4432001	105.5299988
-5.5836902	10.4920006	106.6899948
-5.5836902	10.5408001	106.8699951
-5.5836902	10.5896006	103.8600006
-5.5836902	10.6384001	100.0800018
-5.5836902	10.6872005	97.6500015
-5.5836902	10.7360001	99.8300018
-5.5836902	10.7848005	104.4799957
-5.5836902	10.8336000	105.1999969
-5.5836902	10.8824005	102.2699966
-5.5836902	10.9312000	98.9599991
-5.5836902	10.9800005	98.2799988
-5.5836902	11.0288000	100.5100021
-5.5836902	11.0776005	101.5899963
-5.5836902	11.1264000	100.3099976
-5.5836902	11.1752005	101.2799988
-5.5836902	11.2240000	103.1800003
-5.5836902	11.2728004	102.6199951
-5.5836902	11.3216000	102.1599960
-5.5836902	11.3704004	100.1800003
-5.5836902	11.4191999	103.6100006
-5.5836902	11.4680004	103.4599991
-5.5836902	11.5167999	103.5699997
-5.5836902	11.5656004	101.5499954
-5.5836902	11.6143999	102.7399979
-5.5836902	11.6632004	105.1800003
-5.5836902	11.7119999	105.8300018
-5.5836902	11.7608004	104.1699982
-5.5836902	11.8095999	103.1899948
-5.5836902	11.8584003	99.4099960
-5.5836902	11.9071999	99.6299973
-5.5836902	11.9560003	101.3499985
-5.5836902	12.0047998	103.5000000
-5.5836902	12.0536003	104.8399963
-5.5836902	12.1023998	105.0000000
-5.5836902	12.1512003	102.6899948
-5.5836902	12.1999998	102.5999985
-5.5836902	12.2488003	102.4300003
-5.5836902	12.2975998	107.1599960
-5.5836902	12.3464003	107.7600021
-5.5836902	12.3951998	105.4000015
-5.5836902	12.4440002	101.8199997
-5.5836902	12.4927998	101.0599976
-5.5836902	12.5416002	105.8899994
-5.5836902	12.5904007	104.6699982
-5.5836902	12.6392002	100.4700012
-5.5836902	12.6880007	104.1599960
-5.5836902	12.7368002	104.3899994
-5.5836902	12.7856007	103.9599991

-5.5836902	12.8344002	105.0800018
-5.5836902	12.8832006	105.4199982
-5.5836902	12.9320002	105.7699966
-5.5836902	12.9808006	104.1199951
-5.5836902	13.0296001	101.9899979
-5.5836902	13.0784006	103.5100021
-5.5836902	13.1272001	105.1999969
-5.5836902	13.1760006	104.3600006
-5.5836902	13.2248001	99.1100006
-5.5836902	13.2736006	101.1299973
-5.5836902	13.3224001	100.7799988
-5.5836902	13.3712006	100.2099991
-5.5836902	13.4200001	103.4300003
-5.5836902	13.4688005	103.7699966
-5.5836902	13.5176001	103.6899948
-5.5836902	13.5664005	104.1899948
-5.5836902	13.6152000	102.0400009
-5.5836902	13.6640005	103.0400009
-5.5836902	13.7128000	104.3099976
-5.5836902	13.7616005	103.7200012
-5.5836902	13.8104000	104.6899948
-5.5836902	13.8592005	104.9799957
-5.5836902	13.9080000	104.6999969
-5.5836902	13.9568005	101.9700012
-5.5836902	14.0056000	99.5199966
-5.5836902	14.0544004	103.1899948
-5.5836902	14.1032000	104.0400009
-5.5836902	14.1520004	101.1899948
-5.5836902	14.2007999	101.6199951
-5.5836902	14.2496004	101.0100021
-5.5836902	14.2983999	101.1800003
-5.5836902	14.3472004	101.1800003
-5.5836902	14.3959999	101.4700012
-5.5836902	14.4448004	101.7600021
-5.5836902	14.4935999	103.5199966
-5.5836902	14.5424004	103.5100021
-5.5836902	14.5911999	103.5499954
-5.5836902	14.6400003	100.1999969
-5.5836902	14.6887999	103.4399948
-5.5836902	14.7376003	101.0699997
-5.5836902	14.7863998	103.0800018
-5.5836902	14.8352003	105.3199997
-5.5836902	14.8839998	100.2600021
-5.5836902	14.9328003	101.7900009
-5.5836902	14.9815998	103.2900009
-5.5836902	15.0304003	102.7999954
-5.5775542	0.3416000	106.4599991
-5.5775542	0.3904000	103.9300003
-5.5775542	0.4392000	106.5800018
-5.5775542	0.4880000	99.7299957
-5.5775542	0.5368000	102.5299988
-5.5775542	0.5856000	102.5999985
-5.5775542	0.6344000	100.7500000
-5.5775542	0.6832000	103.3199997
-5.5775542	0.7320000	106.6399994
-5.5775542	0.7808000	105.5699997
-5.5775542	0.8296000	108.3199997
-5.5775542	0.8784000	108.2099991

-5.5775542	0.9272000	106.9399948
-5.5775542	0.9760000	107.9899979
-5.5775542	1.0247999	108.3499985
-5.5775542	1.0735999	108.5599976
-5.5775542	1.1223999	107.5800018
-5.5775542	1.1711999	104.2999954
-5.5775542	1.2200000	102.5699997
-5.5775542	1.2688000	102.9799957
-5.5775542	1.3176000	104.7399979
-5.5775542	1.3664000	103.4700012
-5.5775542	1.4152000	106.7699966
-5.5775542	1.4640000	107.8199997
-5.5775542	1.5128000	107.8899994
-5.5775542	1.5616000	106.4599991
-5.5775542	1.6104000	102.0499954
-5.5775542	1.6592000	106.3199997
-5.5775542	1.7079999	107.9599991
-5.5775542	1.7567999	105.0499954
-5.5775542	1.8055999	103.0899963
-5.5775542	1.8543999	106.9000015
-5.5775542	1.9031999	104.4599991
-5.5775542	1.9519999	102.4300003
-5.5775542	2.0007999	102.9000015
-5.5775542	2.0495999	107.5999985
-5.5775542	2.0983999	108.7299957
-5.5775542	2.1471999	108.2699966
-5.5775542	2.1959999	105.8799973
-5.5775542	2.2447999	104.7299957
-5.5775542	2.2935998	103.0699997
-5.5775542	2.3423998	104.5000000
-5.5775542	2.3912001	105.7600021
-5.5775542	2.4400001	104.5899963
-5.5775542	2.4888000	103.1599960
-5.5775542	2.5376000	102.6999969
-5.5775542	2.5864000	104.5999985
-5.5775542	2.6352000	106.1599960
-5.5775542	2.6840000	103.7799988
-5.5775542	2.7328000	105.8399963
-5.5775542	2.7816000	105.0199966
-5.5775542	2.8304000	104.2699966
-5.5775542	2.8792000	104.5699997
-5.5775542	2.9280000	99.7900009
-5.5775542	2.9768000	105.2500000
-5.5775542	3.0256000	106.1599960
-5.5775542	3.0743999	103.4799957
-5.5775542	3.1231999	98.9599991
-5.5775542	3.1719999	97.9300003
-5.5775542	3.2207999	104.2799988
-5.5775542	3.2695999	103.7399979
-5.5775542	3.3183999	101.1399994
-5.5775542	3.3671999	101.5599976
-5.5775542	3.4159999	105.2799988
-5.5775542	3.4647999	104.4199982
-5.5775542	3.5135999	105.1199951
-5.5775542	3.5623999	106.5800018
-5.5775542	3.6111999	106.2399979
-5.5775542	3.6599998	101.9499969
-5.5775542	3.7087998	105.6599960

-5.5775542	3.7575998	106.8499985
-5.5775542	3.8063998	103.5899963
-5.5775542	3.8551998	103.6199951
-5.5775542	3.9039998	104.9000015
-5.5775542	3.9527998	101.7500000
-5.5775542	4.0016003	105.1500015
-5.5775542	4.0503998	104.7200012
-5.5775542	4.0991998	104.8199997
-5.5775542	4.1479998	104.9499969
-5.5775542	4.1967998	103.1699982
-5.5775542	4.2455997	103.2399979
-5.5775542	4.2943997	108.1800003
-5.5775542	4.3431997	109.1299973
-5.5775542	4.3919997	106.4899979
-5.5775542	4.4407997	108.7600021
-5.5775542	4.4895997	107.8899994
-5.5775542	4.5383997	107.7600021
-5.5775542	4.5871997	106.8899994
-5.5775542	4.6359997	113.1100006
-5.5775542	4.6847997	117.3899994
-5.5775542	4.7336001	116.2900009
-5.5775542	4.7824001	111.3699951
-5.5775542	4.8312001	106.9499969
-5.5775542	4.8800001	106.8600006
-5.5775542	4.9288001	106.0400009
-5.5775542	4.9776001	106.4300003
-5.5775542	5.0264001	106.6199951
-5.5775542	5.0752001	105.2500000
-5.5775542	5.1240001	104.7799988
-5.5775542	5.1728001	102.0499954
-5.5775542	5.2216001	103.4700012
-5.5775542	5.2704000	102.0599976
-5.5775542	5.3192000	104.5000000
-5.5775542	5.3680000	104.8199997
-5.5775542	5.4168000	102.7900009
-5.5775542	5.4656000	105.0800018
-5.5775542	5.5144000	106.9099960
-5.5775542	5.5632000	106.7999954
-5.5775542	5.6120000	106.3499985
-5.5775542	5.6608000	105.6500015
-5.5775542	5.7096000	105.5400009
-5.5775542	5.7584000	103.7200012
-5.5775542	5.8072000	102.6399994
-5.5775542	5.8559999	102.1299973
-5.5775542	5.9047999	98.7399979
-5.5775542	5.9535999	101.5599976
-5.5775542	6.0023999	103.3600006
-5.5775542	6.0511999	107.1999969
-5.5775542	6.0999999	108.1599960
-5.5775542	6.1487999	107.6999969
-5.5775542	6.1975999	105.0499954
-5.5775542	6.2463999	105.4799957
-5.5775542	6.2951999	107.0100021
-5.5775542	6.3439999	106.0599976
-5.5775542	6.3927999	102.9599991
-5.5775542	6.4415998	103.3099976
-5.5775542	6.4903998	101.9899979
-5.5775542	6.5391998	100.1899948

-5.5775542	6.5879998	98.8699951
-5.5775542	6.6367998	102.2299957
-5.5775542	6.6855998	105.6599960
-5.5775542	6.7343998	104.3699951
-5.5775542	6.7831998	104.4000015
-5.5775542	6.8319998	103.8600006
-5.5775542	6.8807998	105.0599976
-5.5775542	6.9295998	102.6999969
-5.5775542	6.9783998	98.3799973
-5.5775542	7.0271997	100.6100006
-5.5775542	7.0759997	103.0400009
-5.5775542	7.1247997	103.6699982
-5.5775542	7.1735997	102.5299988
-5.5775542	7.2223997	105.2099991
-5.5775542	7.2711997	104.9799957
-5.5775542	7.3199997	103.7099991
-5.5775542	7.3687997	102.1899948
-5.5775542	7.4175997	102.2600021
-5.5775542	7.4663997	104.6800003
-5.5775542	7.5151997	105.6100006
-5.5775542	7.5639997	103.5499954
-5.5775542	7.6127996	102.9700012
-5.5775542	7.6615996	103.8899994
-5.5775542	7.7103996	104.3199997
-5.5775542	7.7591996	102.2299957
-5.5775542	7.8079996	104.2699966
-5.5775542	7.8567996	106.0000000
-5.5775542	7.9056001	107.8300018
-5.5775542	7.9544001	106.2900009
-5.5775542	8.0032005	105.1100006
-5.5775542	8.0520000	100.5199966
-5.5775542	8.1008005	99.6500015
-5.5775542	8.1496000	102.0199966
-5.5775542	8.1984005	102.9199982
-5.5775542	8.2472000	106.4700012
-5.5775542	8.2960005	108.3399963
-5.5775542	8.3448000	107.9300003
-5.5775542	8.3936005	106.1800003
-5.5775542	8.4424000	101.9499969
-5.5775542	8.4912004	105.1500015
-5.5775542	8.5400000	107.3699951
-5.5775542	8.5888004	106.9399948
-5.5775542	8.6375999	105.2600021
-5.5775542	8.6864004	100.1299973
-5.5775542	8.7351999	104.2200012
-5.5775542	8.7840004	103.9000015
-5.5775542	8.8327999	101.9000015
-5.5775542	8.8816004	104.1100006
-5.5775542	8.9303999	104.0999985
-5.5775542	8.9792004	104.7699966
-5.5775542	9.0279999	105.0999985
-5.5775542	9.0768003	103.3899994
-5.5775542	9.1255999	103.7500000
-5.5775542	9.1744003	104.5699997
-5.5775542	9.2231998	101.7099991
-5.5775542	9.2720003	102.2600021
-5.5775542	9.3207998	106.4700012
-5.5775542	9.3696003	103.0999985

-5.5775542	9.4184008	100.5599976
-5.5775542	9.4672003	102.2799988
-5.5775542	9.5160007	99.6699982
-5.5775542	9.5648003	103.5999985
-5.5775542	9.6136007	103.3300018
-5.5775542	9.6624002	103.8899994
-5.5775542	9.7112007	106.5199966
-5.5775542	9.7600002	107.2399979
-5.5775542	9.8088007	104.6199951
-5.5775542	9.8576002	103.6399994
-5.5775542	9.9064007	102.0000000
-5.5775542	9.9552002	103.8499985
-5.5775542	10.0040007	107.1199951
-5.5775542	10.0528002	105.6299973
-5.5775542	10.1016006	102.8399963
-5.5775542	10.1504002	105.5400009
-5.5775542	10.1992006	103.5100021
-5.5775542	10.2480001	102.9099960
-5.5775542	10.2968006	103.2799988
-5.5775542	10.3456001	100.9899979
-5.5775542	10.3944006	100.4000015
-5.5775542	10.4432001	104.7200012
-5.5775542	10.4920006	106.5299988
-5.5775542	10.5408001	106.5199966
-5.5775542	10.5896006	103.3699951
-5.5775542	10.6384001	100.3399963
-5.5775542	10.6872005	98.6899948
-5.5775542	10.7360001	100.8499985
-5.5775542	10.7848005	104.8300018
-5.5775542	10.8336000	105.4700012
-5.5775542	10.8824005	102.2699966
-5.5775542	10.9312000	99.9199982
-5.5775542	10.9800005	100.0199966
-5.5775542	11.0288000	101.0499954
-5.5775542	11.0776005	101.4199982
-5.5775542	11.1264000	101.3499985
-5.5775542	11.1752005	102.5000000
-5.5775542	11.2240000	102.7200012
-5.5775542	11.2728004	103.0000000
-5.5775542	11.3216000	102.2799988
-5.5775542	11.3704004	101.4399948
-5.5775542	11.4191999	103.8099976
-5.5775542	11.4680004	104.2299957
-5.5775542	11.5167999	104.6599960
-5.5775542	11.5656004	101.8099976
-5.5775542	11.6143999	102.5999985
-5.5775542	11.6632004	105.7999954
-5.5775542	11.7119999	106.0800018
-5.5775542	11.7608004	104.2399979
-5.5775542	11.8095999	102.9000015
-5.5775542	11.8584003	99.2099991
-5.5775542	11.9071999	99.4799957
-5.5775542	11.9560003	101.7099991
-5.5775542	12.0047998	103.3799973
-5.5775542	12.0536003	104.7200012
-5.5775542	12.1023998	105.2600021
-5.5775542	12.1512003	103.0899963
-5.5775542	12.1999998	103.2200012

-5.5775542	12.2488003	102.3799973
-5.5775542	12.2975998	107.5299988
-5.5775542	12.3464003	108.1199951
-5.5775542	12.3951998	105.4399948
-5.5775542	12.4440002	101.7200012
-5.5775542	12.4927998	100.6199951
-5.5775542	12.5416002	105.9300003
-5.5775542	12.5904007	104.9300003
-5.5775542	12.6392002	102.9799957
-5.5775542	12.6880007	105.6299973
-5.5775542	12.7368002	104.6100006
-5.5775542	12.7856007	103.7299957
-5.5775542	12.8344002	103.9700012
-5.5775542	12.8832006	104.5899963
-5.5775542	12.9320002	104.6399994
-5.5775542	12.9808006	103.3199997
-5.5775542	13.0296001	100.8899994
-5.5775542	13.0784006	103.1899948
-5.5775542	13.1272001	104.8499985
-5.5775542	13.1760006	104.7200012
-5.5775542	13.2248001	99.4199982
-5.5775542	13.2736006	102.1599960
-5.5775542	13.3224001	100.4599991
-5.5775542	13.3712006	100.3300018
-5.5775542	13.4200001	103.8099976
-5.5775542	13.4688005	104.1500015
-5.5775542	13.5176001	103.1999969
-5.5775542	13.5664005	103.7699966
-5.5775542	13.6152000	101.3300018
-5.5775542	13.6640005	103.7500000
-5.5775542	13.7128000	105.0599976
-5.5775542	13.7616005	102.9700012
-5.5775542	13.8104000	103.9000015
-5.5775542	13.8592005	104.3899994
-5.5775542	13.9080000	104.8300018
-5.5775542	13.9568005	102.9199982
-5.5775542	14.0056000	99.7999954
-5.5775542	14.0544004	102.0699997
-5.5775542	14.1032000	103.9199982
-5.5775542	14.1520004	102.0499954
-5.5775542	14.2007999	102.2399979
-5.5775542	14.2496004	100.9000015
-5.5775542	14.2983999	101.9000015
-5.5775542	14.3472004	102.4000015
-5.5775542	14.3959999	102.1599960
-5.5775542	14.4448004	102.4199982
-5.5775542	14.4935999	103.7799988
-5.5775542	14.5424004	103.3899994
-5.5775542	14.5911999	103.6999969
-5.5775542	14.6400003	101.0000000
-5.5775542	14.6887999	103.0000000
-5.5775542	14.7376003	100.6999969
-5.5775542	14.7863998	103.6699982
-5.5775542	14.8352003	106.2099991
-5.5775542	14.8839998	102.4700012
-5.5775542	14.9328003	102.4099960
-5.5775542	14.9815998	103.7999954
-5.5775542	15.0304003	102.9199982

-5.5714183	0.3416000	107.0000000
-5.5714183	0.3904000	104.5899963
-5.5714183	0.4392000	106.5699997
-5.5714183	0.4880000	100.4899979
-5.5714183	0.5368000	101.4799957
-5.5714183	0.5856000	101.3600006
-5.5714183	0.6344000	100.1699982
-5.5714183	0.6832000	103.5199966
-5.5714183	0.7320000	106.5400009
-5.5714183	0.7808000	104.5400009
-5.5714183	0.8296000	108.4700012
-5.5714183	0.8784000	108.7099991
-5.5714183	0.9272000	107.8399963
-5.5714183	0.9760000	109.3099976
-5.5714183	1.0247999	109.6199951
-5.5714183	1.0735999	109.5000000
-5.5714183	1.1223999	108.1399994
-5.5714183	1.1711999	104.1399994
-5.5714183	1.2200000	102.5899963
-5.5714183	1.2688000	104.1899948
-5.5714183	1.3176000	105.1500015
-5.5714183	1.3664000	102.9300003
-5.5714183	1.4152000	105.9899979
-5.5714183	1.4640000	107.7399979
-5.5714183	1.5128000	106.6999969
-5.5714183	1.5616000	105.2799988
-5.5714183	1.6104000	100.4700012
-5.5714183	1.6592000	106.4499969
-5.5714183	1.7079999	107.6999969
-5.5714183	1.7567999	105.4199982
-5.5714183	1.8055999	102.8799973
-5.5714183	1.8543999	107.0000000
-5.5714183	1.9031999	104.4300003
-5.5714183	1.9519999	101.8199997
-5.5714183	2.0007999	102.5699997
-5.5714183	2.0495999	106.7799988
-5.5714183	2.0983999	108.3199997
-5.5714183	2.1471999	108.8899994
-5.5714183	2.1959999	105.8600006
-5.5714183	2.2447999	103.3099976
-5.5714183	2.2935998	102.7900009
-5.5714183	2.3423998	104.5299988
-5.5714183	2.3912001	105.2600021
-5.5714183	2.4400001	103.9899979
-5.5714183	2.4888000	102.7999954
-5.5714183	2.5376000	102.0999985
-5.5714183	2.5864000	104.0499954
-5.5714183	2.6352000	105.7600021
-5.5714183	2.6840000	103.7799988
-5.5714183	2.7328000	106.4499969
-5.5714183	2.7816000	105.2799988
-5.5714183	2.8304000	103.7399979
-5.5714183	2.8792000	104.2299957
-5.5714183	2.9280000	99.6299973
-5.5714183	2.9768000	105.5599976
-5.5714183	3.0256000	105.7999954
-5.5714183	3.0743999	104.5800018
-5.5714183	3.1231999	98.6299973

-5.5714183	3.1719999	99.5400009
-5.5714183	3.2207999	104.4000015
-5.5714183	3.2695999	103.5699997
-5.5714183	3.3183999	99.9499969
-5.5714183	3.3671999	100.6399994
-5.5714183	3.4159999	104.7399979
-5.5714183	3.4647999	104.1199951
-5.5714183	3.5135999	105.0499954
-5.5714183	3.5623999	106.8799973
-5.5714183	3.6111999	106.6999969
-5.5714183	3.6599998	101.9199982
-5.5714183	3.7087998	105.4499969
-5.5714183	3.7575998	106.2200012
-5.5714183	3.8063998	103.0599976
-5.5714183	3.8551998	103.2399979
-5.5714183	3.9039998	103.9300003
-5.5714183	3.9527998	102.9300003
-5.5714183	4.0016003	105.8499985
-5.5714183	4.0503998	104.9700012
-5.5714183	4.0991998	104.5899963
-5.5714183	4.1479998	104.7299957
-5.5714183	4.1967998	101.7799988
-5.5714183	4.2455997	101.5299988
-5.5714183	4.2943997	108.2399979
-5.5714183	4.3431997	108.9000015
-5.5714183	4.3919997	105.7699966
-5.5714183	4.4407997	107.7699966
-5.5714183	4.4895997	107.1299973
-5.5714183	4.5383997	106.7699966
-5.5714183	4.5871997	106.3199997
-5.5714183	4.6359997	112.3899994
-5.5714183	4.6847997	116.8300018
-5.5714183	4.7336001	115.7799988
-5.5714183	4.7824001	110.6699982
-5.5714183	4.8312001	106.0899963
-5.5714183	4.8800001	106.0499954
-5.5714183	4.9288001	105.5699997
-5.5714183	4.9776001	105.7900009
-5.5714183	5.0264001	106.8799973
-5.5714183	5.0752001	105.3199997
-5.5714183	5.1240001	103.9399948
-5.5714183	5.1728001	100.0000000
-5.5714183	5.2216001	102.6399994
-5.5714183	5.2704000	102.2799988
-5.5714183	5.3192000	104.9300003
-5.5714183	5.3680000	104.9000015
-5.5714183	5.4168000	103.0400009
-5.5714183	5.4656000	106.4099960
-5.5714183	5.5144000	108.0100021
-5.5714183	5.5632000	107.9700012
-5.5714183	5.6120000	107.6599960
-5.5714183	5.6608000	106.4499969
-5.5714183	5.7096000	106.3799973
-5.5714183	5.7584000	103.6100006
-5.5714183	5.8072000	101.8899994
-5.5714183	5.8559999	101.2999954
-5.5714183	5.9047999	99.5400009
-5.5714183	5.9535999	101.8899994

-5.5714183	6.0023999	103.7900009
-5.5714183	6.0511999	107.0199966
-5.5714183	6.0999999	107.1800003
-5.5714183	6.1487999	107.7099991
-5.5714183	6.1975999	105.6500015
-5.5714183	6.2463999	104.1899948
-5.5714183	6.2951999	106.1699982
-5.5714183	6.3439999	105.1699982
-5.5714183	6.3927999	102.6899948
-5.5714183	6.4415998	103.8600006
-5.5714183	6.4903998	102.3600006
-5.5714183	6.5391998	100.5999985
-5.5714183	6.5879998	98.7600021
-5.5714183	6.6367998	101.2099991
-5.5714183	6.6855998	105.1899948
-5.5714183	6.7343998	104.2799988
-5.5714183	6.7831998	104.3099976
-5.5714183	6.8319998	104.1199951
-5.5714183	6.8807998	104.6899948
-5.5714183	6.9295998	101.7699966
-5.5714183	6.9783998	96.4499969
-5.5714183	7.0271997	100.8699951
-5.5714183	7.0759997	103.4700012
-5.5714183	7.1247997	103.5000000
-5.5714183	7.1735997	102.4599991
-5.5714183	7.2223997	105.4799957
-5.5714183	7.2711997	104.5000000
-5.5714183	7.3199997	102.7200012
-5.5714183	7.3687997	103.0199966
-5.5714183	7.4175997	103.4099960
-5.5714183	7.4663997	105.4399948
-5.5714183	7.5151997	106.3300018
-5.5714183	7.5639997	103.4799957
-5.5714183	7.6127996	102.6500015
-5.5714183	7.6615996	104.6599960
-5.5714183	7.7103996	104.7500000
-5.5714183	7.7591996	101.4799957
-5.5714183	7.8079996	103.7999954
-5.5714183	7.8567996	105.6100006
-5.5714183	7.9056001	107.2399979
-5.5714183	7.9544001	105.4300003
-5.5714183	8.0032005	105.0899963
-5.5714183	8.0520000	100.9599991
-5.5714183	8.1008005	100.4300003
-5.5714183	8.1496000	102.6500015
-5.5714183	8.1984005	103.1500015
-5.5714183	8.2472000	106.1500015
-5.5714183	8.2960005	107.3600006
-5.5714183	8.3448000	106.7500000
-5.5714183	8.3936005	105.7399979
-5.5714183	8.4424000	102.6999969
-5.5714183	8.4912004	104.8099976
-5.5714183	8.5400000	107.2099991
-5.5714183	8.5888004	106.5199966
-5.5714183	8.6375999	104.8799973
-5.5714183	8.6864004	100.5299988
-5.5714183	8.7351999	103.5100021
-5.5714183	8.7840004	103.3699951

-5.5714183	8.8327999	103.1199951
-5.5714183	8.8816004	104.3099976
-5.5714183	8.9303999	103.8600006
-5.5714183	8.9792004	104.2999954
-5.5714183	9.0279999	104.8199997
-5.5714183	9.0768003	103.8899994
-5.5714183	9.1255999	104.6299973
-5.5714183	9.1744003	103.8199997
-5.5714183	9.2231998	101.7299957
-5.5714183	9.2720003	103.0400009
-5.5714183	9.3207998	105.6699982
-5.5714183	9.3696003	101.5000000
-5.5714183	9.4184008	100.0400009
-5.5714183	9.4672003	101.9399948
-5.5714183	9.5160007	100.7200012
-5.5714183	9.5648003	103.9799957
-5.5714183	9.6136007	103.7099991
-5.5714183	9.6624002	103.8099976
-5.5714183	9.7112007	106.3099976
-5.5714183	9.7600002	106.6899948
-5.5714183	9.8088007	103.5800018
-5.5714183	9.8576002	103.1399994
-5.5714183	9.9064007	101.2200012
-5.5714183	9.9552002	104.0199966
-5.5714183	10.0040007	107.2699966
-5.5714183	10.0528002	105.0699997
-5.5714183	10.1016006	102.1100006
-5.5714183	10.1504002	105.0699997
-5.5714183	10.1992006	104.1899948
-5.5714183	10.2480001	103.6599960
-5.5714183	10.2968006	103.1699982
-5.5714183	10.3456001	100.0499954
-5.5714183	10.3944006	98.3199997
-5.5714183	10.4432001	103.7699966
-5.5714183	10.4920006	106.0199966
-5.5714183	10.5408001	106.0800018
-5.5714183	10.5896006	103.2399979
-5.5714183	10.6384001	101.1199951
-5.5714183	10.6872005	100.5499954
-5.5714183	10.7360001	102.5899963
-5.5714183	10.7848005	105.8899994
-5.5714183	10.8336000	106.3099976
-5.5714183	10.8824005	102.5999985
-5.5714183	10.9312000	100.9799957
-5.5714183	10.9800005	102.1299973
-5.5714183	11.0288000	102.1800003
-5.5714183	11.0776005	101.5599976
-5.5714183	11.1264000	103.1100006
-5.5714183	11.1752005	104.2299957
-5.5714183	11.2240000	102.8099976
-5.5714183	11.2728004	102.4199982
-5.5714183	11.3216000	101.6399994
-5.5714183	11.3704004	102.4899979
-5.5714183	11.4191999	103.9799957
-5.5714183	11.4680004	104.9199982
-5.5714183	11.5167999	105.6299973
-5.5714183	11.5656004	102.3300018
-5.5714183	11.6143999	102.6199951

-5.5714183	11.6632004	105.7799988
-5.5714183	11.7119999	105.7699966
-5.5714183	11.7608004	103.8199997
-5.5714183	11.8095999	102.6299973
-5.5714183	11.8584003	99.7799988
-5.5714183	11.9071999	99.2399979
-5.5714183	11.9560003	101.7900009
-5.5714183	12.0047998	103.4300003
-5.5714183	12.0536003	104.8499985
-5.5714183	12.1023998	105.5199966
-5.5714183	12.1512003	103.4000015
-5.5714183	12.1999998	103.3699951
-5.5714183	12.2488003	103.0299988
-5.5714183	12.2975998	107.6800003
-5.5714183	12.3464003	107.9899979
-5.5714183	12.3951998	104.6800003
-5.5714183	12.4440002	100.9000015
-5.5714183	12.4927998	100.7900009
-5.5714183	12.5416002	105.3199997
-5.5714183	12.5904007	104.9700012
-5.5714183	12.6392002	104.8199997
-5.5714183	12.6880007	106.4799957
-5.5714183	12.7368002	104.6800003
-5.5714183	12.7856007	103.4300003
-5.5714183	12.8344002	102.5599976
-5.5714183	12.8832006	103.4199982
-5.5714183	12.9320002	103.3600006
-5.5714183	12.9808006	102.6100006
-5.5714183	13.0296001	100.7500000
-5.5714183	13.0784006	102.9700012
-5.5714183	13.1272001	104.4599991
-5.5714183	13.1760006	105.3899994
-5.5714183	13.2248001	100.1299973
-5.5714183	13.2736006	103.0400009
-5.5714183	13.3224001	100.8099976
-5.5714183	13.3712006	101.0800018
-5.5714183	13.4200001	104.5100021
-5.5714183	13.4688005	104.7099991
-5.5714183	13.5176001	102.8799973
-5.5714183	13.5664005	102.8499985
-5.5714183	13.6152000	99.9099960
-5.5714183	13.6640005	103.5199966
-5.5714183	13.7128000	105.1899948
-5.5714183	13.7616005	101.6899948
-5.5714183	13.8104000	102.3600006
-5.5714183	13.8592005	103.0599976
-5.5714183	13.9080000	104.1699982
-5.5714183	13.9568005	103.7999954
-5.5714183	14.0056000	100.2799988
-5.5714183	14.0544004	100.9000015
-5.5714183	14.1032000	103.5299988
-5.5714183	14.1520004	102.6699982
-5.5714183	14.2007999	101.6299973
-5.5714183	14.2496004	100.3099976
-5.5714183	14.2983999	102.5699997
-5.5714183	14.3472004	103.7299957
-5.5714183	14.3959999	103.0999985
-5.5714183	14.4448004	103.0199966

-5.5714183	14.4935999	103.5599976
-5.5714183	14.5424004	102.8099976
-5.5714183	14.5911999	103.2999954
-5.5714183	14.6400003	101.5899963
-5.5714183	14.6887999	102.6199951
-5.5714183	14.7376003	100.5299988
-5.5714183	14.7863998	103.6899948
-5.5714183	14.8352003	106.4499969
-5.5714183	14.8839998	103.8899994
-5.5714183	14.9328003	102.6899948
-5.5714183	14.9815998	104.6500015
-5.5714183	15.0304003	103.7399979
-5.5652823	0.3416000	106.3799973
-5.5652823	0.3904000	104.5899963
-5.5652823	0.4392000	106.5100021
-5.5652823	0.4880000	102.0199966
-5.5652823	0.5368000	101.8799973
-5.5652823	0.5856000	101.7299957
-5.5652823	0.6344000	100.3399963
-5.5652823	0.6832000	103.7299957
-5.5652823	0.7320000	106.9000015
-5.5652823	0.7808000	104.9399948
-5.5652823	0.8296000	108.3899994
-5.5652823	0.8784000	108.9399948
-5.5652823	0.9272000	109.3600006
-5.5652823	0.9760000	110.7699966
-5.5652823	1.0247999	110.5000000
-5.5652823	1.0735999	109.3699951
-5.5652823	1.1223999	107.7500000
-5.5652823	1.1711999	104.0699997
-5.5652823	1.2200000	103.1199951
-5.5652823	1.2688000	105.2999954
-5.5652823	1.3176000	105.6199951
-5.5652823	1.3664000	102.9499969
-5.5652823	1.4152000	105.3600006
-5.5652823	1.4640000	107.7399979
-5.5652823	1.5128000	105.5299988
-5.5652823	1.5616000	103.8699951
-5.5652823	1.6104000	100.5899963
-5.5652823	1.6592000	106.6399994
-5.5652823	1.7079999	107.5000000
-5.5652823	1.7567999	106.0800018
-5.5652823	1.8055999	103.8300018
-5.5652823	1.8543999	106.8799973
-5.5652823	1.9031999	103.8499985
-5.5652823	1.9519999	101.3199997
-5.5652823	2.0007999	102.3499985
-5.5652823	2.0495999	106.2099991
-5.5652823	2.0983999	107.7799988
-5.5652823	2.1471999	108.7299957
-5.5652823	2.1959999	105.2299957
-5.5652823	2.2447999	101.9899979
-5.5652823	2.2935998	103.2299957
-5.5652823	2.3423998	104.5100021
-5.5652823	2.3912001	105.0899963
-5.5652823	2.4400001	103.9499969
-5.5652823	2.4888000	102.2299957
-5.5652823	2.5376000	101.7399979

-5.5652823	2.5864000	103.8499985
-5.5652823	2.6352000	104.9700012
-5.5652823	2.6840000	103.3799973
-5.5652823	2.7328000	106.0000000
-5.5652823	2.7816000	104.7399979
-5.5652823	2.8304000	102.9599991
-5.5652823	2.8792000	104.1999969
-5.5652823	2.9280000	100.0000000
-5.5652823	2.9768000	106.0599976
-5.5652823	3.0256000	105.2200012
-5.5652823	3.0743999	105.2099991
-5.5652823	3.1231999	99.3699951
-5.5652823	3.1719999	101.1899948
-5.5652823	3.2207999	104.7500000
-5.5652823	3.2695999	103.9099960
-5.5652823	3.3183999	100.2200012
-5.5652823	3.3671999	100.1500015
-5.5652823	3.4159999	104.6199951
-5.5652823	3.4647999	104.3499985
-5.5652823	3.5135999	105.1399994
-5.5652823	3.5623999	106.7099991
-5.5652823	3.6111999	106.7999954
-5.5652823	3.6599998	102.0299988
-5.5652823	3.7087998	105.3099976
-5.5652823	3.7575998	105.5199966
-5.5652823	3.8063998	101.9099960
-5.5652823	3.8551998	102.3099976
-5.5652823	3.9039998	103.1599960
-5.5652823	3.9527998	104.1800003
-5.5652823	4.0016003	106.2900009
-5.5652823	4.0503998	105.1199951
-5.5652823	4.0991998	103.9199982
-5.5652823	4.1479998	103.9099960
-5.5652823	4.1967998	100.5899963
-5.5652823	4.2455997	99.4599991
-5.5652823	4.2943997	107.0400009
-5.5652823	4.3431997	108.2900009
-5.5652823	4.3919997	105.5999985
-5.5652823	4.4407997	106.9799957
-5.5652823	4.4895997	105.9499969
-5.5652823	4.5383997	105.2200012
-5.5652823	4.5871997	105.8300018
-5.5652823	4.6359997	111.5400009
-5.5652823	4.6847997	116.6399994
-5.5652823	4.7336001	115.9000015
-5.5652823	4.7824001	110.4399948
-5.5652823	4.8312001	105.9000015
-5.5652823	4.8800001	105.4399948
-5.5652823	4.9288001	105.1399994
-5.5652823	4.9776001	105.1699982
-5.5652823	5.0264001	106.6199951
-5.5652823	5.0752001	104.8899994
-5.5652823	5.1240001	102.9499969
-5.5652823	5.1728001	98.1899948
-5.5652823	5.2216001	100.8300018
-5.5652823	5.2704000	102.6899948
-5.5652823	5.3192000	105.5599976
-5.5652823	5.3680000	104.9300003

-5.5652823	5.4168000	103.1299973
-5.5652823	5.4656000	106.6999969
-5.5652823	5.5144000	107.9700012
-5.5652823	5.5632000	108.2200012
-5.5652823	5.6120000	108.0100021
-5.5652823	5.6608000	106.8899994
-5.5652823	5.7096000	106.9300003
-5.5652823	5.7584000	103.4399948
-5.5652823	5.8072000	101.2600021
-5.5652823	5.8559999	100.7999954
-5.5652823	5.9047999	100.2600021
-5.5652823	5.9535999	102.6599960
-5.5652823	6.0023999	104.4700012
-5.5652823	6.0511999	106.5100021
-5.5652823	6.0999999	105.7099991
-5.5652823	6.1487999	106.9799957
-5.5652823	6.1975999	105.3799973
-5.5652823	6.2463999	102.5599976
-5.5652823	6.2951999	105.2900009
-5.5652823	6.3439999	104.6500015
-5.5652823	6.3927999	102.6800003
-5.5652823	6.4415998	104.2500000
-5.5652823	6.4903998	102.8899994
-5.5652823	6.5391998	101.6299973
-5.5652823	6.5879998	99.6800003
-5.5652823	6.6367998	100.1599960
-5.5652823	6.6855998	104.3600006
-5.5652823	6.7343998	103.7900009
-5.5652823	6.7831998	104.4700012
-5.5652823	6.8319998	104.5499954
-5.5652823	6.8807998	104.0299988
-5.5652823	6.9295998	101.4000015
-5.5652823	6.9783998	95.5999985
-5.5652823	7.0271997	101.5699997
-5.5652823	7.0759997	103.9399948
-5.5652823	7.1247997	103.4000015
-5.5652823	7.1735997	102.3300018
-5.5652823	7.2223997	105.6299973
-5.5652823	7.2711997	104.3499985
-5.5652823	7.3199997	102.2900009
-5.5652823	7.3687997	103.5400009
-5.5652823	7.4175997	104.9899979
-5.5652823	7.4663997	106.5599976
-5.5652823	7.5151997	107.1399994
-5.5652823	7.5639997	103.7900009
-5.5652823	7.6127996	101.8499985
-5.5652823	7.6615996	104.2699966
-5.5652823	7.7103996	104.4300003
-5.5652823	7.7591996	100.8600006
-5.5652823	7.8079996	103.7600021
-5.5652823	7.8567996	104.8699951
-5.5652823	7.9056001	106.1299973
-5.5652823	7.9544001	103.8399963
-5.5652823	8.0032005	104.2200012
-5.5652823	8.0520000	101.1999969
-5.5652823	8.1008005	101.4000015
-5.5652823	8.1496000	103.1999969
-5.5652823	8.1984005	102.6199951

-5.5652823	8.2472000	105.2999954
-5.5652823	8.2960005	105.9499969
-5.5652823	8.3448000	105.5100021
-5.5652823	8.3936005	104.8399963
-5.5652823	8.4424000	102.9199982
-5.5652823	8.4912004	104.6899948
-5.5652823	8.5400000	106.8199997
-5.5652823	8.5888004	105.4499969
-5.5652823	8.6375999	103.3499985
-5.5652823	8.6864004	100.8399963
-5.5652823	8.7351999	103.1100006
-5.5652823	8.7840004	103.5299988
-5.5652823	8.8327999	104.1800003
-5.5652823	8.8816004	103.9399948
-5.5652823	8.9303999	102.9300003
-5.5652823	8.9792004	103.4700012
-5.5652823	9.0279999	104.1899948
-5.5652823	9.0768003	103.1100006
-5.5652823	9.1255999	105.2799988
-5.5652823	9.1744003	103.5100021
-5.5652823	9.2231998	101.8300018
-5.5652823	9.2720003	103.6100006
-5.5652823	9.3207998	105.0800018
-5.5652823	9.3696003	101.2900009
-5.5652823	9.4184008	100.2600021
-5.5652823	9.4672003	101.5599976
-5.5652823	9.5160007	101.4099960
-5.5652823	9.5648003	103.7799988
-5.5652823	9.6136007	103.7500000
-5.5652823	9.6624002	103.4099960
-5.5652823	9.7112007	105.9599991
-5.5652823	9.7600002	105.9700012
-5.5652823	9.8088007	102.2799988
-5.5652823	9.8576002	102.2399979
-5.5652823	9.9064007	101.0599976
-5.5652823	9.9552002	104.7399979
-5.5652823	10.0040007	107.6699982
-5.5652823	10.0528002	105.2200012
-5.5652823	10.1016006	102.4700012
-5.5652823	10.1504002	105.0199966
-5.5652823	10.1992006	104.8499985
-5.5652823	10.2480001	104.4099960
-5.5652823	10.2968006	104.1699982
-5.5652823	10.3456001	100.3099976
-5.5652823	10.3944006	98.0499954
-5.5652823	10.4432001	102.4300003
-5.5652823	10.4920006	105.4399948
-5.5652823	10.5408001	105.6500015
-5.5652823	10.5896006	103.5400009
-5.5652823	10.6384001	102.5499954
-5.5652823	10.6872005	102.1299973
-5.5652823	10.7360001	104.0000000
-5.5652823	10.7848005	106.4799957
-5.5652823	10.8336000	106.5800018
-5.5652823	10.8824005	103.1100006
-5.5652823	10.9312000	101.9300003
-5.5652823	10.9800005	103.4000015
-5.5652823	11.0288000	102.5299988

-5.5652823	11.0776005	102.2500000
-5.5652823	11.1264000	105.0800018
-5.5652823	11.1752005	105.6500015
-5.5652823	11.2240000	102.8699951
-5.5652823	11.2728004	101.7299957
-5.5652823	11.3216000	101.0800018
-5.5652823	11.3704004	103.3799973
-5.5652823	11.4191999	104.0400009
-5.5652823	11.4680004	105.5999985
-5.5652823	11.5167999	106.4599991
-5.5652823	11.5656004	103.4399948
-5.5652823	11.6143999	102.7600021
-5.5652823	11.6632004	105.4199982
-5.5652823	11.7119999	105.1299973
-5.5652823	11.7608004	104.1100006
-5.5652823	11.8095999	102.7699966
-5.5652823	11.8584003	100.5800018
-5.5652823	11.9071999	99.2500000
-5.5652823	11.9560003	101.9300003
-5.5652823	12.0047998	103.2799988
-5.5652823	12.0536003	103.9499969
-5.5652823	12.1023998	105.3399963
-5.5652823	12.1512003	103.4599991
-5.5652823	12.1999998	103.0999985
-5.5652823	12.2488003	103.3199997
-5.5652823	12.2975998	107.4300003
-5.5652823	12.3464003	107.3899994
-5.5652823	12.3951998	103.7399979
-5.5652823	12.4440002	100.3399963
-5.5652823	12.4927998	101.1999969
-5.5652823	12.5416002	104.8899994
-5.5652823	12.5904007	104.7900009
-5.5652823	12.6392002	105.7999954
-5.5652823	12.6880007	106.6299973
-5.5652823	12.7368002	105.1899948
-5.5652823	12.7856007	103.3799973
-5.5652823	12.8344002	100.9399948
-5.5652823	12.8832006	101.5499954
-5.5652823	12.9320002	102.4499969
-5.5652823	12.9808006	102.3300018
-5.5652823	13.0296001	100.3799973
-5.5652823	13.0784006	102.7699966
-5.5652823	13.1272001	104.6500015
-5.5652823	13.1760006	106.1899948
-5.5652823	13.2248001	100.0000000
-5.5652823	13.2736006	103.5299988
-5.5652823	13.3224001	101.1500015
-5.5652823	13.3712006	101.6699982
-5.5652823	13.4200001	105.3199997
-5.5652823	13.4688005	105.5299988
-5.5652823	13.5176001	102.6100006
-5.5652823	13.5664005	102.3600006
-5.5652823	13.6152000	99.1199951
-5.5652823	13.6640005	103.3799973
-5.5652823	13.7128000	105.2600021
-5.5652823	13.7616005	100.5599976
-5.5652823	13.8104000	101.0199966
-5.5652823	13.8592005	101.8099976

-5.5652823	13.9080000	103.0800018
-5.5652823	13.9568005	104.2200012
-5.5652823	14.0056000	101.0699997
-5.5652823	14.0544004	99.7699966
-5.5652823	14.1032000	102.9000015
-5.5652823	14.1520004	102.8199997
-5.5652823	14.2007999	101.6100006
-5.5652823	14.2496004	99.8699951
-5.5652823	14.2983999	102.6599960
-5.5652823	14.3472004	104.2099991
-5.5652823	14.3959999	103.3399963
-5.5652823	14.4448004	102.9000015
-5.5652823	14.4935999	103.3699951
-5.5652823	14.5424004	102.3799973
-5.5652823	14.5911999	103.1999969
-5.5652823	14.6400003	102.2099991
-5.5652823	14.6887999	102.2299957
-5.5652823	14.7376003	100.2200012
-5.5652823	14.7863998	103.5400009
-5.5652823	14.8352003	106.1199951
-5.5652823	14.8839998	103.9700012
-5.5652823	14.9328003	101.6100006
-5.5652823	14.9815998	104.9199982
-5.5652823	15.0304003	104.6500015
-5.5591464	0.3416000	106.3099976
-5.5591464	0.3904000	105.7900009
-5.5591464	0.4392000	106.9799957
-5.5591464	0.4880000	103.9000015
-5.5591464	0.5368000	103.4399948
-5.5591464	0.5856000	102.2900009
-5.5591464	0.6344000	99.9000015
-5.5591464	0.6832000	103.7699966
-5.5591464	0.7320000	106.2999954
-5.5591464	0.7808000	104.1800003
-5.5591464	0.8296000	107.6800003
-5.5591464	0.8784000	109.0299988
-5.5591464	0.9272000	110.4199982
-5.5591464	0.9760000	111.7799988
-5.5591464	1.0247999	110.9399948
-5.5591464	1.0735999	109.0699997
-5.5591464	1.1223999	107.3199997
-5.5591464	1.1711999	104.1999969
-5.5591464	1.2200000	104.0199966
-5.5591464	1.2688000	105.8499985
-5.5591464	1.3176000	105.4599991
-5.5591464	1.3664000	102.1800003
-5.5591464	1.4152000	103.6399994
-5.5591464	1.4640000	106.0899963
-5.5591464	1.5128000	103.5199966
-5.5591464	1.5616000	102.3099976
-5.5591464	1.6104000	101.7699966
-5.5591464	1.6592000	106.1599960
-5.5591464	1.7079999	106.3399963
-5.5591464	1.7567999	105.6999969
-5.5591464	1.8055999	104.5899963
-5.5591464	1.8543999	106.7299957
-5.5591464	1.9031999	104.1299973
-5.5591464	1.9519999	101.4399948

-5.5591464	2.0007999	102.2900009
-5.5591464	2.0495999	105.5400009
-5.5591464	2.0983999	107.0599976
-5.5591464	2.1471999	108.1500015
-5.5591464	2.1959999	104.6599960
-5.5591464	2.2447999	100.7600021
-5.5591464	2.2935998	103.9199982
-5.5591464	2.3423998	105.4099960
-5.5591464	2.3912001	105.3499985
-5.5591464	2.4400001	103.7699966
-5.5591464	2.4888000	102.8300018
-5.5591464	2.5376000	101.3899994
-5.5591464	2.5864000	103.8199997
-5.5591464	2.6352000	104.3300018
-5.5591464	2.6840000	103.1800003
-5.5591464	2.7328000	105.7600021
-5.5591464	2.7816000	104.3099976
-5.5591464	2.8304000	102.1399994
-5.5591464	2.8792000	104.3300018
-5.5591464	2.9280000	102.2099991
-5.5591464	2.9768000	106.1399994
-5.5591464	3.0256000	104.0400009
-5.5591464	3.0743999	104.5299988
-5.5591464	3.1231999	99.4000015
-5.5591464	3.1719999	102.9399948
-5.5591464	3.2207999	105.7699966
-5.5591464	3.2695999	104.5299988
-5.5591464	3.3183999	100.4700012
-5.5591464	3.3671999	100.3899994
-5.5591464	3.4159999	104.4099960
-5.5591464	3.4647999	104.4499969
-5.5591464	3.5135999	105.3300018
-5.5591464	3.5623999	106.3799973
-5.5591464	3.6111999	106.0599976
-5.5591464	3.6599998	103.0499954
-5.5591464	3.7087998	105.3399963
-5.5591464	3.7575998	105.1399994
-5.5591464	3.8063998	100.6599960
-5.5591464	3.8551998	100.5899963
-5.5591464	3.9039998	102.9199982
-5.5591464	3.9527998	105.3199997
-5.5591464	4.0016003	106.3600006
-5.5591464	4.0503998	104.7399979
-5.5591464	4.0991998	102.6599960
-5.5591464	4.1479998	102.2900009
-5.5591464	4.1967998	99.7299957
-5.5591464	4.2455997	97.1100006
-5.5591464	4.2943997	105.3799973
-5.5591464	4.3431997	107.3300018
-5.5591464	4.3919997	105.2600021
-5.5591464	4.4407997	105.9399948
-5.5591464	4.4895997	104.3300018
-5.5591464	4.5383997	103.2799988
-5.5591464	4.5871997	104.8699951
-5.5591464	4.6359997	110.8399963
-5.5591464	4.6847997	116.6899948
-5.5591464	4.7336001	115.8699951
-5.5591464	4.7824001	109.6699982

-5.5591464	4.8312001	106.1599960
-5.5591464	4.8800001	106.3600006
-5.5591464	4.9288001	105.9300003
-5.5591464	4.9776001	104.8499985
-5.5591464	5.0264001	106.3099976
-5.5591464	5.0752001	104.9300003
-5.5591464	5.1240001	102.2399979
-5.5591464	5.1728001	96.4599991
-5.5591464	5.2216001	99.2699966
-5.5591464	5.2704000	102.5999985
-5.5591464	5.3192000	105.4399948
-5.5591464	5.3680000	104.9000015
-5.5591464	5.4168000	104.0000000
-5.5591464	5.4656000	106.6699982
-5.5591464	5.5144000	107.4700012
-5.5591464	5.5632000	107.6199951
-5.5591464	5.6120000	106.7200012
-5.5591464	5.6608000	106.5199966
-5.5591464	5.7096000	106.9899979
-5.5591464	5.7584000	102.9300003
-5.5591464	5.8072000	100.4199982
-5.5591464	5.8559999	100.6999969
-5.5591464	5.9047999	101.6599960
-5.5591464	5.9535999	103.7200012
-5.5591464	6.0023999	105.2999954
-5.5591464	6.0511999	106.4099960
-5.5591464	6.0999999	104.5100021
-5.5591464	6.1487999	106.1199951
-5.5591464	6.1975999	104.7900009
-5.5591464	6.2463999	100.5800018
-5.5591464	6.2951999	103.9899979
-5.5591464	6.3439999	104.0899963
-5.5591464	6.3927999	103.1299973
-5.5591464	6.4415998	104.8600006
-5.5591464	6.4903998	103.3600006
-5.5591464	6.5391998	103.1800003
-5.5591464	6.5879998	100.5999985
-5.5591464	6.6367998	100.1599960
-5.5591464	6.6855998	103.9300003
-5.5591464	6.7343998	103.4899979
-5.5591464	6.7831998	104.3499985
-5.5591464	6.8319998	104.6199951
-5.5591464	6.8807998	103.7999954
-5.5591464	6.9295998	101.3099976
-5.5591464	6.9783998	97.2999954
-5.5591464	7.0271997	102.0699997
-5.5591464	7.0759997	104.0400009
-5.5591464	7.1247997	102.9399948
-5.5591464	7.1735997	101.8199997
-5.5591464	7.2223997	105.3699951
-5.5591464	7.2711997	104.0899963
-5.5591464	7.3199997	101.2699966
-5.5591464	7.3687997	102.5999985
-5.5591464	7.4175997	105.7200012
-5.5591464	7.4663997	107.1199951
-5.5591464	7.5151997	107.5999985
-5.5591464	7.5639997	103.6500015
-5.5591464	7.6127996	101.7500000

-5.5591464	7.6615996	103.0599976
-5.5591464	7.7103996	103.7900009
-5.5591464	7.7591996	101.7900009
-5.5591464	7.8079996	104.2099991
-5.5591464	7.8567996	104.6100006
-5.5591464	7.9056001	105.0100021
-5.5591464	7.9544001	101.9499969
-5.5591464	8.0032005	103.3799973
-5.5591464	8.0520000	101.5899963
-5.5591464	8.1008005	102.4399948
-5.5591464	8.1496000	103.7099991
-5.5591464	8.1984005	102.7200012
-5.5591464	8.2472000	104.5999985
-5.5591464	8.2960005	104.2399979
-5.5591464	8.3448000	103.9000015
-5.5591464	8.3936005	103.5000000
-5.5591464	8.4424000	102.4300003
-5.5591464	8.4912004	104.3699951
-5.5591464	8.5400000	106.8600006
-5.5591464	8.5888004	104.8399963
-5.5591464	8.6375999	102.2299957
-5.5591464	8.6864004	101.7799988
-5.5591464	8.7351999	102.9099960
-5.5591464	8.7840004	104.0599976
-5.5591464	8.8327999	105.2699966
-5.5591464	8.8816004	103.3199997
-5.5591464	8.9303999	101.6899948
-5.5591464	8.9792004	102.1399994
-5.5591464	9.0279999	103.1399994
-5.5591464	9.0768003	102.3799973
-5.5591464	9.1255999	105.6699982
-5.5591464	9.1744003	103.0800018
-5.5591464	9.2231998	101.1100006
-5.5591464	9.2720003	104.1800003
-5.5591464	9.3207998	104.4599991
-5.5591464	9.3696003	101.4700012
-5.5591464	9.4184008	100.7699966
-5.5591464	9.4672003	101.5800018
-5.5591464	9.5160007	103.1699982
-5.5591464	9.5648003	104.4099960
-5.5591464	9.6136007	103.8099976
-5.5591464	9.6624002	102.0199966
-5.5591464	9.7112007	105.4199982
-5.5591464	9.7600002	105.5000000
-5.5591464	9.8088007	101.5199966
-5.5591464	9.8576002	102.6899948
-5.5591464	9.9064007	102.4499969
-5.5591464	9.9552002	106.1899948
-5.5591464	10.0040007	108.0699997
-5.5591464	10.0528002	105.0599976
-5.5591464	10.1016006	102.8600006
-5.5591464	10.1504002	105.2299957
-5.5591464	10.1992006	105.3099976
-5.5591464	10.2480001	105.2600021
-5.5591464	10.2968006	105.2699966
-5.5591464	10.3456001	101.4799957
-5.5591464	10.3944006	98.5999985
-5.5591464	10.4432001	101.8099976

-5.5591464	10.4920006	104.8600006
-5.5591464	10.5408001	105.4799957
-5.5591464	10.5896006	104.2999954
-5.5591464	10.6384001	103.5199966
-5.5591464	10.6872005	102.5800018
-5.5591464	10.7360001	104.7999954
-5.5591464	10.7848005	106.7600021
-5.5591464	10.8336000	106.6899948
-5.5591464	10.8824005	103.5299988
-5.5591464	10.9312000	103.1800003
-5.5591464	10.9800005	104.5499954
-5.5591464	11.0288000	102.7600021
-5.5591464	11.0776005	102.7299957
-5.5591464	11.1264000	106.0800018
-5.5591464	11.1752005	106.4899979
-5.5591464	11.2240000	102.9899979
-5.5591464	11.2728004	100.7099991
-5.5591464	11.3216000	100.4899979
-5.5591464	11.3704004	103.5299988
-5.5591464	11.4191999	104.4799957
-5.5591464	11.4680004	106.2500000
-5.5591464	11.5167999	106.7999954
-5.5591464	11.5656004	104.0100021
-5.5591464	11.6143999	103.0400009
-5.5591464	11.6632004	105.1299973
-5.5591464	11.7119999	104.8099976
-5.5591464	11.7608004	104.7799988
-5.5591464	11.8095999	103.5299988
-5.5591464	11.8584003	102.3600006
-5.5591464	11.9071999	100.0000000
-5.5591464	11.9560003	102.0899963
-5.5591464	12.0047998	103.3600006
-5.5591464	12.0536003	103.4099960
-5.5591464	12.1023998	105.3699951
-5.5591464	12.1512003	103.8600006
-5.5591464	12.1999998	103.0699997
-5.5591464	12.2488003	103.8399963
-5.5591464	12.2975998	107.1599960
-5.5591464	12.3464003	106.1100006
-5.5591464	12.3951998	102.8199997
-5.5591464	12.4440002	100.6500015
-5.5591464	12.4927998	99.9300003
-5.5591464	12.5416002	104.3199997
-5.5591464	12.5904007	105.1500015
-5.5591464	12.6392002	105.8499985
-5.5591464	12.6880007	106.3399963
-5.5591464	12.7368002	105.6800003
-5.5591464	12.7856007	103.6999969
-5.5591464	12.8344002	100.6800003
-5.5591464	12.8832006	100.4099960
-5.5591464	12.9320002	101.7999954
-5.5591464	12.9808006	102.0299988
-5.5591464	13.0296001	99.3099976
-5.5591464	13.0784006	102.8300018
-5.5591464	13.1272001	104.8199997
-5.5591464	13.1760006	106.5199966
-5.5591464	13.2248001	101.0899963
-5.5591464	13.2736006	103.5100021

-5.5591464	13.3224001	101.4000015
-5.5591464	13.3712006	102.6199951
-5.5591464	13.4200001	106.4099960
-5.5591464	13.4688005	106.0400009
-5.5591464	13.5176001	102.2799988
-5.5591464	13.5664005	102.0800018
-5.5591464	13.6152000	98.7399979
-5.5591464	13.6640005	102.9799957
-5.5591464	13.7128000	104.1800003
-5.5591464	13.7616005	99.8899994
-5.5591464	13.8104000	100.7999954
-5.5591464	13.8592005	101.0400009
-5.5591464	13.9080000	101.3399963
-5.5591464	13.9568005	104.4199982
-5.5591464	14.0056000	101.4300003
-5.5591464	14.0544004	99.3399963
-5.5591464	14.1032000	102.2399979
-5.5591464	14.1520004	102.4599991
-5.5591464	14.2007999	101.7600021
-5.5591464	14.2496004	100.3499985
-5.5591464	14.2983999	102.9399948
-5.5591464	14.3472004	104.3300018
-5.5591464	14.3959999	103.3699951
-5.5591464	14.4448004	102.8899994
-5.5591464	14.4935999	103.5400009
-5.5591464	14.5424004	101.9300003
-5.5591464	14.5911999	102.4300003
-5.5591464	14.6400003	102.5899963
-5.5591464	14.6887999	102.1199951
-5.5591464	14.7376003	100.0599976
-5.5591464	14.7863998	103.6199951
-5.5591464	14.8352003	105.6500015
-5.5591464	14.8839998	103.4199982
-5.5591464	14.9328003	100.3799973
-5.5591464	14.9815998	105.1199951
-5.5591464	15.0304003	105.4300003
-5.5530105	0.3416000	105.8099976
-5.5530105	0.3904000	105.9799957
-5.5530105	0.4392000	107.7099991
-5.5530105	0.4880000	105.5699997
-5.5530105	0.5368000	104.5800018
-5.5530105	0.5856000	102.4599991
-5.5530105	0.6344000	99.7200012
-5.5530105	0.6832000	104.1399994
-5.5530105	0.7320000	106.4399948
-5.5530105	0.7808000	104.3300018
-5.5530105	0.8296000	107.1199951
-5.5530105	0.8784000	108.6299973
-5.5530105	0.9272000	110.7900009
-5.5530105	0.9760000	111.8699951
-5.5530105	1.0247999	110.6199951
-5.5530105	1.0735999	108.4000015
-5.5530105	1.1223999	106.7799988
-5.5530105	1.1711999	103.9000015
-5.5530105	1.2200000	104.5699997
-5.5530105	1.2688000	106.0499954
-5.5530105	1.3176000	104.5999985
-5.5530105	1.3664000	100.6299973

-5.5530105	1.4152000	101.8899994
-5.5530105	1.4640000	105.0100021
-5.5530105	1.5128000	103.0899963
-5.5530105	1.5616000	101.7099991
-5.5530105	1.6104000	103.3499985
-5.5530105	1.6592000	106.2299957
-5.5530105	1.7079999	105.7399979
-5.5530105	1.7567999	106.1500015
-5.5530105	1.8055999	105.5699997
-5.5530105	1.8543999	106.0599976
-5.5530105	1.9031999	104.6399994
-5.5530105	1.9519999	102.0800018
-5.5530105	2.0007999	102.5199966
-5.5530105	2.0495999	104.9300003
-5.5530105	2.0983999	106.1500015
-5.5530105	2.1471999	107.1599960
-5.5530105	2.1959999	103.6999969
-5.5530105	2.2447999	101.0299988
-5.5530105	2.2935998	105.1399994
-5.5530105	2.3423998	106.2999954
-5.5530105	2.3912001	105.5699997
-5.5530105	2.4400001	103.5899963
-5.5530105	2.4888000	103.8799973
-5.5530105	2.5376000	103.2200012
-5.5530105	2.5864000	105.2900009
-5.5530105	2.6352000	104.9599991
-5.5530105	2.6840000	103.9799957
-5.5530105	2.7328000	105.3600006
-5.5530105	2.7816000	104.0299988
-5.5530105	2.8304000	101.7099991
-5.5530105	2.8792000	104.5400009
-5.5530105	2.9280000	103.9499969
-5.5530105	2.9768000	106.5100021
-5.5530105	3.0256000	103.4899979
-5.5530105	3.0743999	104.8099976
-5.5530105	3.1231999	99.8799973
-5.5530105	3.1719999	104.1800003
-5.5530105	3.2207999	106.3199997
-5.5530105	3.2695999	104.9700012
-5.5530105	3.3183999	101.0800018
-5.5530105	3.3671999	100.9199982
-5.5530105	3.4159999	104.6299973
-5.5530105	3.4647999	105.0599976
-5.5530105	3.5135999	105.8099976
-5.5530105	3.5623999	106.7900009
-5.5530105	3.6111999	105.3099976
-5.5530105	3.6599998	103.5000000
-5.5530105	3.7087998	105.4300003
-5.5530105	3.7575998	105.1999969
-5.5530105	3.8063998	100.1199951
-5.5530105	3.8551998	99.4300003
-5.5530105	3.9039998	102.7799988
-5.5530105	3.9527998	105.7600021
-5.5530105	4.0016003	105.3300018
-5.5530105	4.0503998	104.4700012
-5.5530105	4.0991998	102.2600021
-5.5530105	4.1479998	100.5599976
-5.5530105	4.1967998	98.9399948

-5.5530105	4.2455997	97.0599976
-5.5530105	4.2943997	103.5999985
-5.5530105	4.3431997	106.3099976
-5.5530105	4.3919997	104.8399963
-5.5530105	4.4407997	104.7099991
-5.5530105	4.4895997	102.8699951
-5.5530105	4.5383997	102.3600006
-5.5530105	4.5871997	104.5299988
-5.5530105	4.6359997	110.1899948
-5.5530105	4.6847997	116.8499985
-5.5530105	4.7336001	116.0000000
-5.5530105	4.7824001	108.9199982
-5.5530105	4.8312001	106.7299957
-5.5530105	4.8800001	107.6800003
-5.5530105	4.9288001	106.7299957
-5.5530105	4.9776001	104.5100021
-5.5530105	5.0264001	106.2900009
-5.5530105	5.0752001	105.0899963
-5.5530105	5.1240001	101.7500000
-5.5530105	5.1728001	96.8399963
-5.5530105	5.2216001	98.2600021
-5.5530105	5.2704000	102.0899963
-5.5530105	5.3192000	104.9599991
-5.5530105	5.3680000	104.9199982
-5.5530105	5.4168000	104.9000015
-5.5530105	5.4656000	106.7399979
-5.5530105	5.5144000	107.4300003
-5.5530105	5.5632000	107.6299973
-5.5530105	5.6120000	105.8499985
-5.5530105	5.6608000	106.2099991
-5.5530105	5.7096000	106.8300018
-5.5530105	5.7584000	102.9099960
-5.5530105	5.8072000	101.2200012
-5.5530105	5.8559999	101.7299957
-5.5530105	5.9047999	102.5000000
-5.5530105	5.9535999	104.7500000
-5.5530105	6.0023999	106.1100006
-5.5530105	6.0511999	106.4899979
-5.5530105	6.0999999	104.3399963
-5.5530105	6.1487999	105.6199951
-5.5530105	6.1975999	104.2299957
-5.5530105	6.2463999	99.2299957
-5.5530105	6.2951999	103.5699997
-5.5530105	6.3439999	103.9899979
-5.5530105	6.3927999	103.6399994
-5.5530105	6.4415998	104.6699982
-5.5530105	6.4903998	103.4199982
-5.5530105	6.5391998	104.3600006
-5.5530105	6.5879998	101.9000015
-5.5530105	6.6367998	100.9099960
-5.5530105	6.6855998	104.1299973
-5.5530105	6.7343998	103.7600021
-5.5530105	6.7831998	104.3399963
-5.5530105	6.8319998	104.5899963
-5.5530105	6.8807998	103.6100006
-5.5530105	6.9295998	101.9099960
-5.5530105	6.9783998	99.6800003
-5.5530105	7.0271997	103.5100021

-5.5530105	7.0759997	103.9399948
-5.5530105	7.1247997	102.3399963
-5.5530105	7.1735997	101.3199997
-5.5530105	7.2223997	105.0000000
-5.5530105	7.2711997	103.9099960
-5.5530105	7.3199997	101.2099991
-5.5530105	7.3687997	101.7500000
-5.5530105	7.4175997	106.2200012
-5.5530105	7.4663997	107.3199997
-5.5530105	7.5151997	107.5299988
-5.5530105	7.5639997	102.7999954
-5.5530105	7.6127996	101.4399948
-5.5530105	7.6615996	102.5100021
-5.5530105	7.7103996	103.3699951
-5.5530105	7.7591996	103.0000000
-5.5530105	7.8079996	104.7900009
-5.5530105	7.8567996	104.3600006
-5.5530105	7.9056001	104.4499969
-5.5530105	7.9544001	101.7999954
-5.5530105	8.0032005	102.8300018
-5.5530105	8.0520000	101.4300003
-5.5530105	8.1008005	103.4700012
-5.5530105	8.1496000	104.2399979
-5.5530105	8.1984005	102.5499954
-5.5530105	8.2472000	103.5999985
-5.5530105	8.2960005	103.0400009
-5.5530105	8.3448000	103.0000000
-5.5530105	8.3936005	102.2099991
-5.5530105	8.4424000	101.7500000
-5.5530105	8.4912004	103.2099991
-5.5530105	8.5400000	106.3300018
-5.5530105	8.5888004	104.2600021
-5.5530105	8.6375999	101.5400009
-5.5530105	8.6864004	101.9199982
-5.5530105	8.7351999	103.2299957
-5.5530105	8.7840004	104.8499985
-5.5530105	8.8327999	105.6800003
-5.5530105	8.8816004	102.8799973
-5.5530105	8.9303999	101.0000000
-5.5530105	8.9792004	102.0199966
-5.5530105	9.0279999	103.1299973
-5.5530105	9.0768003	102.1500015
-5.5530105	9.1255999	106.0499954
-5.5530105	9.1744003	103.0499954
-5.5530105	9.2231998	101.4799957
-5.5530105	9.2720003	104.9099960
-5.5530105	9.3207998	104.3199997
-5.5530105	9.3696003	102.2900009
-5.5530105	9.4184008	101.7399979
-5.5530105	9.4672003	102.8699951
-5.5530105	9.5160007	104.3899994
-5.5530105	9.5648003	104.7699966
-5.5530105	9.6136007	103.3899994
-5.5530105	9.6624002	100.5199966
-5.5530105	9.7112007	103.9499969
-5.5530105	9.7600002	105.0599976
-5.5530105	9.8088007	101.7600021
-5.5530105	9.8576002	103.2600021

-5.5530105	9.9064007	104.4700012
-5.5530105	9.9552002	107.8600006
-5.5530105	10.0040007	108.4199982
-5.5530105	10.0528002	105.1299973
-5.5530105	10.1016006	103.8399963
-5.5530105	10.1504002	105.4700012
-5.5530105	10.1992006	105.8399963
-5.5530105	10.2480001	106.3399963
-5.5530105	10.2968006	106.5699997
-5.5530105	10.3456001	102.9199982
-5.5530105	10.3944006	100.2699966
-5.5530105	10.4432001	101.6699982
-5.5530105	10.4920006	104.4499969
-5.5530105	10.5408001	105.4899979
-5.5530105	10.5896006	105.0499954
-5.5530105	10.6384001	104.6999969
-5.5530105	10.6872005	102.8199997
-5.5530105	10.7360001	105.2999954
-5.5530105	10.7848005	106.9099960
-5.5530105	10.8336000	106.7600021
-5.5530105	10.8824005	104.1999969
-5.5530105	10.9312000	104.6999969
-5.5530105	10.9800005	105.2200012
-5.5530105	11.0288000	102.7299957
-5.5530105	11.0776005	102.7600021
-5.5530105	11.1264000	106.2999954
-5.5530105	11.1752005	106.7999954
-5.5530105	11.2240000	103.8600006
-5.5530105	11.2728004	100.1899948
-5.5530105	11.3216000	101.0699997
-5.5530105	11.3704004	103.8399963
-5.5530105	11.4191999	104.9099960
-5.5530105	11.4680004	106.0699997
-5.5530105	11.5167999	106.6699982
-5.5530105	11.5656004	104.1399994
-5.5530105	11.6143999	102.9499969
-5.5530105	11.6632004	104.8799973
-5.5530105	11.7119999	104.5400009
-5.5530105	11.7608004	105.3699951
-5.5530105	11.8095999	104.5899963
-5.5530105	11.8584003	103.9099960
-5.5530105	11.9071999	101.3699951
-5.5530105	11.9560003	102.8799973
-5.5530105	12.0047998	103.9700012
-5.5530105	12.0536003	102.6399994
-5.5530105	12.1023998	105.1699982
-5.5530105	12.1512003	103.7200012
-5.5530105	12.1999998	102.7600021
-5.5530105	12.2488003	104.0199966
-5.5530105	12.2975998	106.5599976
-5.5530105	12.3464003	104.6199951
-5.5530105	12.3951998	102.0400009
-5.5530105	12.4440002	101.0000000
-5.5530105	12.4927998	98.4700012
-5.5530105	12.5416002	103.3099976
-5.5530105	12.5904007	104.6199951
-5.5530105	12.6392002	105.1299973
-5.5530105	12.6880007	105.4000015

-5.5530105	12.7368002	105.4499969
-5.5530105	12.7856007	103.9399948
-5.5530105	12.8344002	100.5199966
-5.5530105	12.8832006	99.4799957
-5.5530105	12.9320002	101.7999954
-5.5530105	12.9808006	102.0400009
-5.5530105	13.0296001	98.6100006
-5.5530105	13.0784006	103.2399979
-5.5530105	13.1272001	105.1699982
-5.5530105	13.1760006	106.6399994
-5.5530105	13.2248001	102.0299988
-5.5530105	13.2736006	103.4499969
-5.5530105	13.3224001	102.0299988
-5.5530105	13.3712006	103.7600021
-5.5530105	13.4200001	107.0400009
-5.5530105	13.4688005	106.4199982
-5.5530105	13.5176001	102.5599976
-5.5530105	13.5664005	102.0999985
-5.5530105	13.6152000	98.8799973
-5.5530105	13.6640005	102.5000000
-5.5530105	13.7128000	102.8600006
-5.5530105	13.7616005	98.7999954
-5.5530105	13.8104000	101.1999969
-5.5530105	13.8592005	100.8300018
-5.5530105	13.9080000	99.5999985
-5.5530105	13.9568005	104.2299957
-5.5530105	14.0056000	101.5599976
-5.5530105	14.0544004	98.7299957
-5.5530105	14.1032000	101.4599991
-5.5530105	14.1520004	101.6100006
-5.5530105	14.2007999	101.6899948
-5.5530105	14.2496004	101.1199951
-5.5530105	14.2983999	103.1999969
-5.5530105	14.3472004	104.6800003
-5.5530105	14.3959999	104.0100021
-5.5530105	14.4448004	103.1999969
-5.5530105	14.4935999	103.1100006
-5.5530105	14.5424004	100.5899963
-5.5530105	14.5911999	101.5499954
-5.5530105	14.6400003	102.9599991
-5.5530105	14.6887999	102.2399979
-5.5530105	14.7376003	99.4199982
-5.5530105	14.7863998	102.8099976
-5.5530105	14.8352003	104.7600021
-5.5530105	14.8839998	102.8799973
-5.5530105	14.9328003	99.6100006
-5.5530105	14.9815998	104.9300003
-5.5530105	15.0304003	105.9599991
-5.5468745	0.3416000	105.3699951
-5.5468745	0.3904000	106.1100006
-5.5468745	0.4392000	108.3399963
-5.5468745	0.4880000	107.0599976
-5.5468745	0.5368000	105.6599960
-5.5468745	0.5856000	102.6199951
-5.5468745	0.6344000	100.0499954
-5.5468745	0.6832000	104.5599976
-5.5468745	0.7320000	106.3199997
-5.5468745	0.7808000	104.3600006

-5.5468745	0.8296000	107.2799988
-5.5468745	0.8784000	108.5199966
-5.5468745	0.9272000	111.0199966
-5.5468745	0.9760000	111.3499985
-5.5468745	1.0247999	110.0899963
-5.5468745	1.0735999	107.7799988
-5.5468745	1.1223999	106.2399979
-5.5468745	1.1711999	103.6500015
-5.5468745	1.2200000	105.0400009
-5.5468745	1.2688000	106.2099991
-5.5468745	1.3176000	104.1199951
-5.5468745	1.3664000	100.8399963
-5.5468745	1.4152000	101.8199997
-5.5468745	1.4640000	104.1899948
-5.5468745	1.5128000	102.8199997
-5.5468745	1.5616000	102.5800018
-5.5468745	1.6104000	105.0000000
-5.5468745	1.6592000	106.4799957
-5.5468745	1.7079999	105.4899979
-5.5468745	1.7567999	106.5599976
-5.5468745	1.8055999	106.5599976
-5.5468745	1.8543999	105.4000015
-5.5468745	1.9031999	104.7600021
-5.5468745	1.9519999	102.2600021
-5.5468745	2.0007999	102.6599960
-5.5468745	2.0495999	104.5800018
-5.5468745	2.0983999	105.6299973
-5.5468745	2.1471999	106.6899948
-5.5468745	2.1959999	103.5800018
-5.5468745	2.2447999	101.9099960
-5.5468745	2.2935998	105.7500000
-5.5468745	2.3423998	106.8600006
-5.5468745	2.3912001	104.9599991
-5.5468745	2.4400001	103.3600006
-5.5468745	2.4888000	104.6599960
-5.5468745	2.5376000	104.4799957
-5.5468745	2.5864000	106.3099976
-5.5468745	2.6352000	105.1800003
-5.5468745	2.6840000	104.7099991
-5.5468745	2.7328000	104.8799973
-5.5468745	2.7816000	103.4399948
-5.5468745	2.8304000	102.1399994
-5.5468745	2.8792000	104.9000015
-5.5468745	2.9280000	106.0100021
-5.5468745	2.9768000	107.4099960
-5.5468745	3.0256000	103.8199997
-5.5468745	3.0743999	105.4399948
-5.5468745	3.1231999	101.9799957
-5.5468745	3.1719999	105.3099976
-5.5468745	3.2207999	106.5400009
-5.5468745	3.2695999	105.4700012
-5.5468745	3.3183999	102.2500000
-5.5468745	3.3671999	101.7299957
-5.5468745	3.4159999	104.5299988
-5.5468745	3.4647999	105.7200012
-5.5468745	3.5135999	105.5899963
-5.5468745	3.5623999	106.7099991
-5.5468745	3.6111999	104.0400009

-5.5468745	3.6599998	103.9499969
-5.5468745	3.7087998	105.4700012
-5.5468745	3.7575998	104.9799957
-5.5468745	3.8063998	99.7900009
-5.5468745	3.8551998	99.1800003
-5.5468745	3.9039998	103.3399963
-5.5468745	3.9527998	105.5400009
-5.5468745	4.0016003	104.4399948
-5.5468745	4.0503998	104.7600021
-5.5468745	4.0991998	101.6800003
-5.5468745	4.1479998	99.0299988
-5.5468745	4.1967998	98.5100021
-5.5468745	4.2455997	97.7600021
-5.5468745	4.2943997	102.3600006
-5.5468745	4.3431997	105.3799973
-5.5468745	4.3919997	104.1199951
-5.5468745	4.4407997	103.0400009
-5.5468745	4.4895997	101.8300018
-5.5468745	4.5383997	102.3699951
-5.5468745	4.5871997	104.7099991
-5.5468745	4.6359997	110.3699951
-5.5468745	4.6847997	117.1899948
-5.5468745	4.7336001	115.9499969
-5.5468745	4.7824001	108.2699966
-5.5468745	4.8312001	107.3199997
-5.5468745	4.8800001	108.7699966
-5.5468745	4.9288001	106.9899979
-5.5468745	4.9776001	103.6500015
-5.5468745	5.0264001	105.6299973
-5.5468745	5.0752001	104.8799973
-5.5468745	5.1240001	101.8499985
-5.5468745	5.1728001	96.7600021
-5.5468745	5.2216001	97.1500015
-5.5468745	5.2704000	101.6199951
-5.5468745	5.3192000	104.5599976
-5.5468745	5.3680000	104.8199997
-5.5468745	5.4168000	105.2399979
-5.5468745	5.4656000	106.3899994
-5.5468745	5.5144000	106.9399948
-5.5468745	5.5632000	107.4599991
-5.5468745	5.6120000	105.3499985
-5.5468745	5.6608000	106.6500015
-5.5468745	5.7096000	106.7600021
-5.5468745	5.7584000	102.7399979
-5.5468745	5.8072000	102.0800018
-5.5468745	5.8559999	103.1100006
-5.5468745	5.9047999	103.5599976
-5.5468745	5.9535999	105.9300003
-5.5468745	6.0023999	107.0499954
-5.5468745	6.0511999	106.6699982
-5.5468745	6.0999999	104.4499969
-5.5468745	6.1487999	105.1399994
-5.5468745	6.1975999	103.5199966
-5.5468745	6.2463999	97.8600006
-5.5468745	6.2951999	103.2600021
-5.5468745	6.3439999	103.9700012
-5.5468745	6.3927999	104.0599976
-5.5468745	6.4415998	104.7600021

-5.5468745	6.4903998	104.0899963
-5.5468745	6.5391998	104.7399979
-5.5468745	6.5879998	102.1999969
-5.5468745	6.6367998	101.3099976
-5.5468745	6.6855998	104.0199966
-5.5468745	6.7343998	103.4300003
-5.5468745	6.7831998	104.1599960
-5.5468745	6.8319998	103.9499969
-5.5468745	6.8807998	103.4199982
-5.5468745	6.9295998	103.4700012
-5.5468745	6.9783998	101.6899948
-5.5468745	7.0271997	104.0599976
-5.5468745	7.0759997	103.3399963
-5.5468745	7.1247997	101.7999954
-5.5468745	7.1735997	100.8699951
-5.5468745	7.2223997	105.0199966
-5.5468745	7.2711997	104.5299988
-5.5468745	7.3199997	101.2699966
-5.5468745	7.3687997	100.6999969
-5.5468745	7.4175997	106.0299988
-5.5468745	7.4663997	106.8799973
-5.5468745	7.5151997	106.9799957
-5.5468745	7.5639997	102.0800018
-5.5468745	7.6127996	101.4899979
-5.5468745	7.6615996	101.6999969
-5.5468745	7.7103996	101.8399963
-5.5468745	7.7591996	103.9899979
-5.5468745	7.8079996	105.0100021
-5.5468745	7.8567996	104.2900009
-5.5468745	7.9056001	104.4799957
-5.5468745	7.9544001	102.1899948
-5.5468745	8.0032005	103.0899963
-5.5468745	8.0520000	101.5800018
-5.5468745	8.1008005	103.9700012
-5.5468745	8.1496000	104.2600021
-5.5468745	8.1984005	102.7099991
-5.5468745	8.2472000	103.3499985
-5.5468745	8.2960005	102.1999969
-5.5468745	8.3448000	102.2799988
-5.5468745	8.3936005	101.5100021
-5.5468745	8.4424000	101.5899963
-5.5468745	8.4912004	103.1800003
-5.5468745	8.5400000	105.4899979
-5.5468745	8.5888004	103.4700012
-5.5468745	8.6375999	100.6199951
-5.5468745	8.6864004	101.7699966
-5.5468745	8.7351999	103.5000000
-5.5468745	8.7840004	106.0499954
-5.5468745	8.8327999	106.5199966
-5.5468745	8.8816004	103.0899963
-5.5468745	8.9303999	100.6699982
-5.5468745	8.9792004	101.7099991
-5.5468745	9.0279999	102.4899979
-5.5468745	9.0768003	101.4899979
-5.5468745	9.1255999	106.4499969
-5.5468745	9.1744003	103.8899994
-5.5468745	9.2231998	101.8300018
-5.5468745	9.2720003	104.9799957

-5.5468745	9.3207998	104.1199951
-5.5468745	9.3696003	103.0899963
-5.5468745	9.4184008	102.2799988
-5.5468745	9.4672003	103.7600021
-5.5468745	9.5160007	105.5400009
-5.5468745	9.5648003	105.4399948
-5.5468745	9.6136007	103.4300003
-5.5468745	9.6624002	99.1599960
-5.5468745	9.7112007	102.3300018
-5.5468745	9.7600002	104.2299957
-5.5468745	9.8088007	101.8399963
-5.5468745	9.8576002	103.6399994
-5.5468745	9.9064007	105.9700012
-5.5468745	9.9552002	108.9599991
-5.5468745	10.0040007	108.2900009
-5.5468745	10.0528002	104.7900009
-5.5468745	10.1016006	104.4799957
-5.5468745	10.1504002	106.0400009
-5.5468745	10.1992006	105.8300018
-5.5468745	10.2480001	106.7399979
-5.5468745	10.2968006	107.5800018
-5.5468745	10.3456001	104.0499954
-5.5468745	10.3944006	101.3699951
-5.5468745	10.4432001	101.1199951
-5.5468745	10.4920006	103.5299988
-5.5468745	10.5408001	105.1299973
-5.5468745	10.5896006	105.7200012
-5.5468745	10.6384001	105.7900009
-5.5468745	10.6872005	103.7200012
-5.5468745	10.7360001	105.0199966
-5.5468745	10.7848005	106.7600021
-5.5468745	10.8336000	106.6999969
-5.5468745	10.8824005	104.4499969
-5.5468745	10.9312000	105.2500000
-5.5468745	10.9800005	104.8099976
-5.5468745	11.0288000	102.1999969
-5.5468745	11.0776005	102.6399994
-5.5468745	11.1264000	106.0800018
-5.5468745	11.1752005	105.9899979
-5.5468745	11.2240000	104.5000000
-5.5468745	11.2728004	99.9399948
-5.5468745	11.3216000	101.1999969
-5.5468745	11.3704004	103.1599960
-5.5468745	11.4191999	104.8499985
-5.5468745	11.4680004	105.4700012
-5.5468745	11.5167999	106.5800018
-5.5468745	11.5656004	104.5800018
-5.5468745	11.6143999	102.3699951
-5.5468745	11.6632004	104.3099976
-5.5468745	11.7119999	104.3199997
-5.5468745	11.7608004	105.7799988
-5.5468745	11.8095999	105.4899979
-5.5468745	11.8584003	105.4300003
-5.5468745	11.9071999	102.9599991
-5.5468745	11.9560003	103.5999985
-5.5468745	12.0047998	104.2299957
-5.5468745	12.0536003	101.8300018
-5.5468745	12.1023998	105.0199966

-5.5468745	12.1512003	103.5999985
-5.5468745	12.1999998	102.1999969
-5.5468745	12.2488003	103.6100006
-5.5468745	12.2975998	105.3899994
-5.5468745	12.3464003	102.9799957
-5.5468745	12.3951998	101.4599991
-5.5468745	12.4440002	101.3499985
-5.5468745	12.4927998	97.2999954
-5.5468745	12.5416002	102.7200012
-5.5468745	12.5904007	104.0999985
-5.5468745	12.6392002	104.0499954
-5.5468745	12.6880007	103.9899979
-5.5468745	12.7368002	104.8899994
-5.5468745	12.7856007	103.8899994
-5.5468745	12.8344002	100.7600021
-5.5468745	12.8832006	99.6800003
-5.5468745	12.9320002	102.3499985
-5.5468745	12.9808006	102.6399994
-5.5468745	13.0296001	98.4499969
-5.5468745	13.0784006	103.4599991
-5.5468745	13.1272001	105.6500015
-5.5468745	13.1760006	106.4199982
-5.5468745	13.2248001	101.9199982
-5.5468745	13.2736006	102.6999969
-5.5468745	13.3224001	103.0800018
-5.5468745	13.3712006	105.1699982
-5.5468745	13.4200001	107.7200012
-5.5468745	13.4688005	106.8699951
-5.5468745	13.5176001	103.0599976
-5.5468745	13.5664005	102.5800018
-5.5468745	13.6152000	99.5800018
-5.5468745	13.6640005	101.5699997
-5.5468745	13.7128000	101.7699966
-5.5468745	13.7616005	99.3300018
-5.5468745	13.8104000	102.5299988
-5.5468745	13.8592005	101.8499985
-5.5468745	13.9080000	98.3199997
-5.5468745	13.9568005	103.8199997
-5.5468745	14.0056000	101.9700012
-5.5468745	14.0544004	98.6899948
-5.5468745	14.1032000	100.7500000
-5.5468745	14.1520004	101.1100006
-5.5468745	14.2007999	102.1800003
-5.5468745	14.2496004	102.0800018
-5.5468745	14.2983999	103.4599991
-5.5468745	14.3472004	104.8199997
-5.5468745	14.3959999	104.4799957
-5.5468745	14.4448004	103.5299988
-5.5468745	14.4935999	102.8899994
-5.5468745	14.5424004	99.4000015
-5.5468745	14.5911999	100.9300003
-5.5468745	14.6400003	102.9099960
-5.5468745	14.6887999	102.8399963
-5.5468745	14.7376003	99.6399994
-5.5468745	14.7863998	102.5400009
-5.5468745	14.8352003	104.1800003
-5.5468745	14.8839998	101.9799957
-5.5468745	14.9328003	97.7900009

-5.5468745	14.9815998	104.6299973
-5.5468745	15.0304003	106.5299988
-5.5407386	0.3416000	104.3099976
-5.5407386	0.3904000	105.8799973
-5.5407386	0.4392000	108.9300003
-5.5407386	0.4880000	108.3399963
-5.5407386	0.5368000	106.7200012
-5.5407386	0.5856000	102.9099960
-5.5407386	0.6344000	100.7600021
-5.5407386	0.6832000	104.8300018
-5.5407386	0.7320000	105.8300018
-5.5407386	0.7808000	103.7099991
-5.5407386	0.8296000	106.7299957
-5.5407386	0.8784000	107.6199951
-5.5407386	0.9272000	110.3600006
-5.5407386	0.9760000	109.8600006
-5.5407386	1.0247999	109.2500000
-5.5407386	1.0735999	106.9300003
-5.5407386	1.1223999	105.5400009
-5.5407386	1.1711999	103.7399979
-5.5407386	1.2200000	105.1999969
-5.5407386	1.2688000	105.5999985
-5.5407386	1.3176000	102.6299973
-5.5407386	1.3664000	100.0599976
-5.5407386	1.4152000	101.7399979
-5.5407386	1.4640000	104.2900009
-5.5407386	1.5128000	104.0100021
-5.5407386	1.5616000	103.0999985
-5.5407386	1.6104000	106.2999954
-5.5407386	1.6592000	106.1899948
-5.5407386	1.7079999	104.8600006
-5.5407386	1.7567999	106.6100006
-5.5407386	1.8055999	107.0499954
-5.5407386	1.8543999	104.1500015
-5.5407386	1.9031999	104.6899948
-5.5407386	1.9519999	102.2900009
-5.5407386	2.0007999	101.8600006
-5.5407386	2.0495999	104.0299988
-5.5407386	2.0983999	105.0000000
-5.5407386	2.1471999	105.7900009
-5.5407386	2.1959999	103.4399948
-5.5407386	2.2447999	102.9000015
-5.5407386	2.2935998	105.9799957
-5.5407386	2.3423998	107.4000015
-5.5407386	2.3912001	104.6299973
-5.5407386	2.4400001	103.1299973
-5.5407386	2.4888000	104.7900009
-5.5407386	2.5376000	105.2500000
-5.5407386	2.5864000	106.9499969
-5.5407386	2.6352000	105.3399963
-5.5407386	2.6840000	104.7799988
-5.5407386	2.7328000	104.2600021
-5.5407386	2.7816000	102.8499985
-5.5407386	2.8304000	102.3799973
-5.5407386	2.8792000	104.7799988
-5.5407386	2.9280000	107.3099976
-5.5407386	2.9768000	107.9599991
-5.5407386	3.0256000	104.0699997

-5.5407386	3.0743999	105.2999954
-5.5407386	3.1231999	103.1599960
-5.5407386	3.1719999	105.1699982
-5.5407386	3.2207999	106.5100021
-5.5407386	3.2695999	106.2900009
-5.5407386	3.3183999	103.3600006
-5.5407386	3.3671999	101.8699951
-5.5407386	3.4159999	103.8300018
-5.5407386	3.4647999	105.9399948
-5.5407386	3.5135999	105.7699966
-5.5407386	3.5623999	106.1800003
-5.5407386	3.6111999	102.3099976
-5.5407386	3.6599998	104.2500000
-5.5407386	3.7087998	105.2500000
-5.5407386	3.7575998	104.7099991
-5.5407386	3.8063998	99.7200012
-5.5407386	3.8551998	98.4000015
-5.5407386	3.9039998	103.7799988
-5.5407386	3.9527998	105.0699997
-5.5407386	4.0016003	102.9899979
-5.5407386	4.0503998	104.4399948
-5.5407386	4.0991998	101.6899948
-5.5407386	4.1479998	98.5499954
-5.5407386	4.1967998	99.2500000
-5.5407386	4.2455997	99.0999985
-5.5407386	4.2943997	101.3600006
-5.5407386	4.3431997	104.7999954
-5.5407386	4.3919997	103.6100006
-5.5407386	4.4407997	101.8199997
-5.5407386	4.4895997	101.6699982
-5.5407386	4.5383997	103.1699982
-5.5407386	4.5871997	104.7600021
-5.5407386	4.6359997	110.3199997
-5.5407386	4.6847997	117.2799988
-5.5407386	4.7336001	115.7500000
-5.5407386	4.7824001	108.0100021
-5.5407386	4.8312001	107.8600006
-5.5407386	4.8800001	109.6800003
-5.5407386	4.9288001	107.2399979
-5.5407386	4.9776001	102.6699982
-5.5407386	5.0264001	105.1599960
-5.5407386	5.0752001	104.5599976
-5.5407386	5.1240001	102.0400009
-5.5407386	5.1728001	97.8199997
-5.5407386	5.2216001	96.6199951
-5.5407386	5.2704000	100.6599960
-5.5407386	5.3192000	104.0599976
-5.5407386	5.3680000	104.2699966
-5.5407386	5.4168000	105.1299973
-5.5407386	5.4656000	105.6699982
-5.5407386	5.5144000	106.5000000
-5.5407386	5.5632000	107.2099991
-5.5407386	5.6120000	104.1299973
-5.5407386	5.6608000	106.6800003
-5.5407386	5.7096000	106.7099991
-5.5407386	5.7584000	103.4799957
-5.5407386	5.8072000	103.0100021
-5.5407386	5.8559999	103.7600021

-5.5407386	5.9047999	103.6699982
-5.5407386	5.9535999	106.5199966
-5.5407386	6.0023999	107.5800018
-5.5407386	6.0511999	107.1199951
-5.5407386	6.0999999	105.1800003
-5.5407386	6.1487999	105.0800018
-5.5407386	6.1975999	103.2299957
-5.5407386	6.2463999	98.8499985
-5.5407386	6.2951999	103.1199951
-5.5407386	6.3439999	104.0999985
-5.5407386	6.3927999	104.5800018
-5.5407386	6.4415998	104.6899948
-5.5407386	6.4903998	104.8199997
-5.5407386	6.5391998	104.9799957
-5.5407386	6.5879998	102.2299957
-5.5407386	6.6367998	101.7699966
-5.5407386	6.6855998	103.8899994
-5.5407386	6.7343998	102.4000015
-5.5407386	6.7831998	103.3699951
-5.5407386	6.8319998	102.6999969
-5.5407386	6.8807998	103.1500015
-5.5407386	6.9295998	104.9499969
-5.5407386	6.9783998	103.8199997
-5.5407386	7.0271997	104.4399948
-5.5407386	7.0759997	102.9899979
-5.5407386	7.1247997	102.0899963
-5.5407386	7.1735997	101.6699982
-5.5407386	7.2223997	105.2999954
-5.5407386	7.2711997	104.7200012
-5.5407386	7.3199997	101.4199982
-5.5407386	7.3687997	99.9599991
-5.5407386	7.4175997	105.6999969
-5.5407386	7.4663997	106.0999985
-5.5407386	7.5151997	106.0499954
-5.5407386	7.5639997	101.4799957
-5.5407386	7.6127996	102.3699951
-5.5407386	7.6615996	102.5199966
-5.5407386	7.7103996	100.8699951
-5.5407386	7.7591996	105.0999985
-5.5407386	7.8079996	105.1699982
-5.5407386	7.8567996	103.9899979
-5.5407386	7.9056001	104.9300003
-5.5407386	7.9544001	103.3499985
-5.5407386	8.0032005	102.9599991
-5.5407386	8.0520000	100.5499954
-5.5407386	8.1008005	103.7799988
-5.5407386	8.1496000	104.4700012
-5.5407386	8.1984005	103.2200012
-5.5407386	8.2472000	103.2999954
-5.5407386	8.2960005	102.0699997
-5.5407386	8.3448000	102.2299957
-5.5407386	8.3936005	100.8300018
-5.5407386	8.4424000	100.6100006
-5.5407386	8.4912004	103.1199951
-5.5407386	8.5400000	105.3799973
-5.5407386	8.5888004	104.0699997
-5.5407386	8.6375999	100.1299973
-5.5407386	8.6864004	101.4499969

-5.5407386	8.7351999	103.6999969
-5.5407386	8.7840004	106.8699951
-5.5407386	8.8327999	106.6899948
-5.5407386	8.8816004	103.5699997
-5.5407386	8.9303999	101.3300018
-5.5407386	8.9792004	102.1500015
-5.5407386	9.0279999	102.2399979
-5.5407386	9.0768003	100.8699951
-5.5407386	9.1255999	106.8300018
-5.5407386	9.1744003	104.4399948
-5.5407386	9.2231998	101.3699951
-5.5407386	9.2720003	104.7900009
-5.5407386	9.3207998	103.8600006
-5.5407386	9.3696003	104.2399979
-5.5407386	9.4184008	103.0999985
-5.5407386	9.4672003	103.7999954
-5.5407386	9.5160007	105.1299973
-5.5407386	9.5648003	105.1699982
-5.5407386	9.6136007	103.1199951
-5.5407386	9.6624002	98.2500000
-5.5407386	9.7112007	100.7399979
-5.5407386	9.7600002	103.4199982
-5.5407386	9.8088007	102.3099976
-5.5407386	9.8576002	104.4599991
-5.5407386	9.9064007	107.1999969
-5.5407386	9.9552002	109.1699982
-5.5407386	10.0040007	107.7299957
-5.5407386	10.0528002	105.2799988
-5.5407386	10.1016006	105.2200012
-5.5407386	10.1504002	106.4899979
-5.5407386	10.1992006	105.5599976
-5.5407386	10.2480001	106.5699997
-5.5407386	10.2968006	108.0199966
-5.5407386	10.3456001	105.2699966
-5.5407386	10.3944006	102.8699951
-5.5407386	10.4432001	100.5599976
-5.5407386	10.4920006	103.1100006
-5.5407386	10.5408001	105.1599960
-5.5407386	10.5896006	106.2200012
-5.5407386	10.6384001	106.2799988
-5.5407386	10.6872005	103.4799957
-5.5407386	10.7360001	103.8300018
-5.5407386	10.7848005	105.9700012
-5.5407386	10.8336000	105.9799957
-5.5407386	10.8824005	104.5299988
-5.5407386	10.9312000	105.7399979
-5.5407386	10.9800005	104.0499954
-5.5407386	11.0288000	101.3300018
-5.5407386	11.0776005	102.9799957
-5.5407386	11.1264000	105.7699966
-5.5407386	11.1752005	105.0299988
-5.5407386	11.2240000	104.9199982
-5.5407386	11.2728004	100.2900009
-5.5407386	11.3216000	100.8499985
-5.5407386	11.3704004	102.1899948
-5.5407386	11.4191999	104.6100006
-5.5407386	11.4680004	104.8099976
-5.5407386	11.5167999	105.9099960

-5.5407386	11.5656004	104.4499969
-5.5407386	11.6143999	101.7299957
-5.5407386	11.6632004	104.3300018
-5.5407386	11.7119999	104.3300018
-5.5407386	11.7608004	106.3399963
-5.5407386	11.8095999	106.2500000
-5.5407386	11.8584003	106.3199997
-5.5407386	11.9071999	104.4300003
-5.5407386	11.9560003	104.5800018
-5.5407386	12.0047998	104.5499954
-5.5407386	12.0536003	100.9899979
-5.5407386	12.1023998	105.0199966
-5.5407386	12.1512003	103.6199951
-5.5407386	12.1999998	102.3099976
-5.5407386	12.2488003	103.3399963
-5.5407386	12.2975998	104.4899979
-5.5407386	12.3464003	101.8600006
-5.5407386	12.3951998	101.7500000
-5.5407386	12.4440002	102.5699997
-5.5407386	12.4927998	96.3499985
-5.5407386	12.5416002	103.0499954
-5.5407386	12.5904007	103.9599991
-5.5407386	12.6392002	103.3799973
-5.5407386	12.6880007	102.9300003
-5.5407386	12.7368002	104.1599960
-5.5407386	12.7856007	103.6500015
-5.5407386	12.8344002	101.5199966
-5.5407386	12.8832006	99.7900009
-5.5407386	12.9320002	102.7999954
-5.5407386	12.9808006	102.8499985
-5.5407386	13.0296001	97.5400009
-5.5407386	13.0784006	103.3300018
-5.5407386	13.1272001	105.9399948
-5.5407386	13.1760006	106.1699982
-5.5407386	13.2248001	101.9199982
-5.5407386	13.2736006	102.3799973
-5.5407386	13.3224001	103.4000015
-5.5407386	13.3712006	105.5800018
-5.5407386	13.4200001	107.7399979
-5.5407386	13.4688005	106.9700012
-5.5407386	13.5176001	103.4099960
-5.5407386	13.5664005	103.4700012
-5.5407386	13.6152000	101.3699951
-5.5407386	13.6640005	101.9499969
-5.5407386	13.7128000	100.7500000
-5.5407386	13.7616005	99.5699997
-5.5407386	13.8104000	103.5100021
-5.5407386	13.8592005	102.6500015
-5.5407386	13.9080000	97.7099991
-5.5407386	13.9568005	103.5499954
-5.5407386	14.0056000	102.6199951
-5.5407386	14.0544004	98.7900009
-5.5407386	14.1032000	100.6899948
-5.5407386	14.1520004	100.9000015
-5.5407386	14.2007999	102.9099960
-5.5407386	14.2496004	102.3199997
-5.5407386	14.2983999	103.1699982
-5.5407386	14.3472004	104.4899979

-5.5407386	14.3959999	104.2500000
-5.5407386	14.4448004	104.0499954
-5.5407386	14.4935999	103.5899963
-5.5407386	14.5424004	99.0800018
-5.5407386	14.5911999	100.8600006
-5.5407386	14.6400003	103.1500015
-5.5407386	14.6887999	103.4499969
-5.5407386	14.7376003	99.1500015
-5.5407386	14.7863998	101.6599960
-5.5407386	14.8352003	103.6100006
-5.5407386	14.8839998	101.3699951
-5.5407386	14.9328003	96.0199966
-5.5407386	14.9815998	103.9099960
-5.5407386	15.0304003	106.2099991
-5.5346026	0.3416000	104.5800018
-5.5346026	0.3904000	106.4199982
-5.5346026	0.4392000	109.7299957
-5.5346026	0.4880000	109.2200012
-5.5346026	0.5368000	107.0199966
-5.5346026	0.5856000	102.5699997
-5.5346026	0.6344000	101.2099991
-5.5346026	0.6832000	104.8300018
-5.5346026	0.7320000	105.1299973
-5.5346026	0.7808000	102.2699966
-5.5346026	0.8296000	105.7799988
-5.5346026	0.8784000	107.3199997
-5.5346026	0.9272000	109.3799973
-5.5346026	0.9760000	107.7999954
-5.5346026	1.0247999	108.1599960
-5.5346026	1.0735999	106.9000015
-5.5346026	1.1223999	105.3799973
-5.5346026	1.1711999	103.8899994
-5.5346026	1.2200000	104.8799973
-5.5346026	1.2688000	105.0899963
-5.5346026	1.3176000	101.8300018
-5.5346026	1.3664000	99.5699997
-5.5346026	1.4152000	101.5699997
-5.5346026	1.4640000	104.3199997
-5.5346026	1.5128000	105.1800003
-5.5346026	1.5616000	103.7900009
-5.5346026	1.6104000	106.6800003
-5.5346026	1.6592000	106.0800018
-5.5346026	1.7079999	103.8300018
-5.5346026	1.7567999	106.0100021
-5.5346026	1.8055999	106.8099976
-5.5346026	1.8543999	103.0400009
-5.5346026	1.9031999	104.3399963
-5.5346026	1.9519999	101.7900009
-5.5346026	2.0007999	101.5000000
-5.5346026	2.0495999	104.0000000
-5.5346026	2.0983999	105.0199966
-5.5346026	2.1471999	105.3799973
-5.5346026	2.1959999	104.1699982
-5.5346026	2.2447999	104.4399948
-5.5346026	2.2935998	105.8899994
-5.5346026	2.3423998	108.0000000
-5.5346026	2.3912001	104.5899963
-5.5346026	2.4400001	103.4199982

-5.5346026	2.4888000	105.2699966
-5.5346026	2.5376000	106.5100021
-5.5346026	2.5864000	107.6399994
-5.5346026	2.6352000	105.8699951
-5.5346026	2.6840000	104.8399963
-5.5346026	2.7328000	104.1599960
-5.5346026	2.7816000	103.4199982
-5.5346026	2.8304000	102.9799957
-5.5346026	2.8792000	105.4000015
-5.5346026	2.9280000	108.6899948
-5.5346026	2.9768000	108.4099960
-5.5346026	3.0256000	104.1800003
-5.5346026	3.0743999	104.7500000
-5.5346026	3.1231999	103.3799973
-5.5346026	3.1719999	104.8399963
-5.5346026	3.2207999	106.2900009
-5.5346026	3.2695999	106.2200012
-5.5346026	3.3183999	103.5000000
-5.5346026	3.3671999	101.8699951
-5.5346026	3.4159999	102.2999954
-5.5346026	3.4647999	105.2399979
-5.5346026	3.5135999	105.3199997
-5.5346026	3.5623999	105.7399979
-5.5346026	3.6111999	100.7699966
-5.5346026	3.6599998	104.5499954
-5.5346026	3.7087998	105.0299988
-5.5346026	3.7575998	104.4499969
-5.5346026	3.8063998	100.0899963
-5.5346026	3.8551998	97.5599976
-5.5346026	3.9039998	103.1599960
-5.5346026	3.9527998	103.9300003
-5.5346026	4.0016003	101.3499985
-5.5346026	4.0503998	104.2200012
-5.5346026	4.0991998	102.1500015
-5.5346026	4.1479998	98.3899994
-5.5346026	4.1967998	100.0699997
-5.5346026	4.2455997	101.2399979
-5.5346026	4.2943997	102.1500015
-5.5346026	4.3431997	104.9899979
-5.5346026	4.3919997	103.3199997
-5.5346026	4.4407997	100.7399979
-5.5346026	4.4895997	101.4499969
-5.5346026	4.5383997	104.1100006
-5.5346026	4.5871997	104.8799973
-5.5346026	4.6359997	110.6800003
-5.5346026	4.6847997	117.0299988
-5.5346026	4.7336001	115.0599976
-5.5346026	4.7824001	107.3199997
-5.5346026	4.8312001	107.9399948
-5.5346026	4.8800001	110.2299957
-5.5346026	4.9288001	107.2200012
-5.5346026	4.9776001	102.4700012
-5.5346026	5.0264001	105.3799973
-5.5346026	5.0752001	104.8300018
-5.5346026	5.1240001	102.5699997
-5.5346026	5.1728001	98.9700012
-5.5346026	5.2216001	98.3300018
-5.5346026	5.2704000	100.2299957

-5.5346026	5.3192000	103.5100021
-5.5346026	5.3680000	103.9099960
-5.5346026	5.4168000	105.2299957
-5.5346026	5.4656000	105.3199997
-5.5346026	5.5144000	105.9000015
-5.5346026	5.5632000	106.8099976
-5.5346026	5.6120000	103.0499954
-5.5346026	5.6608000	106.9499969
-5.5346026	5.7096000	107.2999954
-5.5346026	5.7584000	104.3499985
-5.5346026	5.8072000	103.4700012
-5.5346026	5.8559999	104.2299957
-5.5346026	5.9047999	104.2200012
-5.5346026	5.9535999	107.0899963
-5.5346026	6.0023999	107.8199997
-5.5346026	6.0511999	107.3399963
-5.5346026	6.0999999	106.1399994
-5.5346026	6.1487999	105.7099991
-5.5346026	6.1975999	103.6699982
-5.5346026	6.2463999	100.3399963
-5.5346026	6.2951999	103.1199951
-5.5346026	6.3439999	103.7799988
-5.5346026	6.3927999	105.0899963
-5.5346026	6.4415998	105.0100021
-5.5346026	6.4903998	105.4099960
-5.5346026	6.5391998	104.6199951
-5.5346026	6.5879998	101.3399963
-5.5346026	6.6367998	102.6500015
-5.5346026	6.6855998	104.4199982
-5.5346026	6.7343998	101.9700012
-5.5346026	6.7831998	102.2600021
-5.5346026	6.8319998	101.1199951
-5.5346026	6.8807998	103.3499985
-5.5346026	6.9295998	106.0899963
-5.5346026	6.9783998	105.2799988
-5.5346026	7.0271997	104.3799973
-5.5346026	7.0759997	102.3899994
-5.5346026	7.1247997	102.2399979
-5.5346026	7.1735997	102.3199997
-5.5346026	7.2223997	105.4300003
-5.5346026	7.2711997	104.7799988
-5.5346026	7.3199997	101.6599960
-5.5346026	7.3687997	99.3799973
-5.5346026	7.4175997	104.9599991
-5.5346026	7.4663997	104.6199951
-5.5346026	7.5151997	104.2299957
-5.5346026	7.5639997	100.6299973
-5.5346026	7.6127996	103.4499969
-5.5346026	7.6615996	103.6500015
-5.5346026	7.7103996	100.0899963
-5.5346026	7.7591996	106.1800003
-5.5346026	7.8079996	105.3499985
-5.5346026	7.8567996	104.3600006
-5.5346026	7.9056001	105.9199982
-5.5346026	7.9544001	105.1800003
-5.5346026	8.0032005	103.9099960
-5.5346026	8.0520000	100.0400009
-5.5346026	8.1008005	103.6100006

-5.5346026	8.1496000	104.9899979
-5.5346026	8.1984005	104.3099976
-5.5346026	8.2472000	103.6399994
-5.5346026	8.2960005	102.2299957
-5.5346026	8.3448000	102.5000000
-5.5346026	8.3936005	100.9499969
-5.5346026	8.4424000	100.7399979
-5.5346026	8.4912004	102.5800018
-5.5346026	8.5400000	104.7999954
-5.5346026	8.5888004	104.7799988
-5.5346026	8.6375999	101.0599976
-5.5346026	8.6864004	101.6299973
-5.5346026	8.7351999	104.3600006
-5.5346026	8.7840004	107.3399963
-5.5346026	8.8327999	106.7699966
-5.5346026	8.8816004	104.4799957
-5.5346026	8.9303999	102.9799957
-5.5346026	8.9792004	103.3199997
-5.5346026	9.0279999	102.6299973
-5.5346026	9.0768003	102.4099960
-5.5346026	9.1255999	106.8600006
-5.5346026	9.1744003	104.0499954
-5.5346026	9.2231998	100.5999985
-5.5346026	9.2720003	104.7200012
-5.5346026	9.3207998	103.8300018
-5.5346026	9.3696003	105.0199966
-5.5346026	9.4184008	103.4399948
-5.5346026	9.4672003	103.7299957
-5.5346026	9.5160007	105.2099991
-5.5346026	9.5648003	105.3799973
-5.5346026	9.6136007	103.3300018
-5.5346026	9.6624002	97.9599991
-5.5346026	9.7112007	99.4700012
-5.5346026	9.7600002	103.0599976
-5.5346026	9.8088007	102.7200012
-5.5346026	9.8576002	104.8399963
-5.5346026	9.9064007	107.5100021
-5.5346026	9.9552002	108.5100021
-5.5346026	10.0040007	106.8399963
-5.5346026	10.0528002	105.6100006
-5.5346026	10.1016006	105.8199997
-5.5346026	10.1504002	106.7299957
-5.5346026	10.1992006	105.2099991
-5.5346026	10.2480001	106.1899948
-5.5346026	10.2968006	107.5199966
-5.5346026	10.3456001	105.8300018
-5.5346026	10.3944006	103.6399994
-5.5346026	10.4432001	101.3600006
-5.5346026	10.4920006	102.5800018
-5.5346026	10.5408001	104.9300003
-5.5346026	10.5896006	106.2600021
-5.5346026	10.6384001	105.7699966
-5.5346026	10.6872005	102.5100021
-5.5346026	10.7360001	102.6699982
-5.5346026	10.7848005	105.6100006
-5.5346026	10.8336000	105.4700012
-5.5346026	10.8824005	104.4700012
-5.5346026	10.9312000	106.0000000

-5.5346026	10.9800005	103.9099960
-5.5346026	11.0288000	101.3099976
-5.5346026	11.0776005	103.2200012
-5.5346026	11.1264000	105.2500000
-5.5346026	11.1752005	103.6599960
-5.5346026	11.2240000	105.2999954
-5.5346026	11.2728004	101.5699997
-5.5346026	11.3216000	100.4499969
-5.5346026	11.3704004	101.1699982
-5.5346026	11.4191999	104.5199966
-5.5346026	11.4680004	104.3799973
-5.5346026	11.5167999	105.8399963
-5.5346026	11.5656004	104.4899979
-5.5346026	11.6143999	100.9799957
-5.5346026	11.6632004	104.4700012
-5.5346026	11.7119999	104.6599960
-5.5346026	11.7608004	106.3799973
-5.5346026	11.8095999	106.4399948
-5.5346026	11.8584003	106.5800018
-5.5346026	11.9071999	105.2600021
-5.5346026	11.9560003	105.0999985
-5.5346026	12.0047998	105.0899963
-5.5346026	12.0536003	102.0100021
-5.5346026	12.1023998	105.5100021
-5.5346026	12.1512003	104.1299973
-5.5346026	12.1999998	103.1800003
-5.5346026	12.2488003	103.6100006
-5.5346026	12.2975998	104.0599976
-5.5346026	12.3464003	101.2699966
-5.5346026	12.3951998	102.4499969
-5.5346026	12.4440002	103.9899979
-5.5346026	12.4927998	95.6199951
-5.5346026	12.5416002	103.1100006
-5.5346026	12.5904007	103.2500000
-5.5346026	12.6392002	103.0899963
-5.5346026	12.6880007	102.1399994
-5.5346026	12.7368002	102.8499985
-5.5346026	12.7856007	103.1199951
-5.5346026	12.8344002	102.3099976
-5.5346026	12.8832006	100.1899948
-5.5346026	12.9320002	103.3899994
-5.5346026	12.9808006	103.5299988
-5.5346026	13.0296001	97.8899994
-5.5346026	13.0784006	103.4499969
-5.5346026	13.1272001	105.7099991
-5.5346026	13.1760006	105.6299973
-5.5346026	13.2248001	102.0100021
-5.5346026	13.2736006	102.2299957
-5.5346026	13.3224001	103.6299973
-5.5346026	13.3712006	105.6599960
-5.5346026	13.4200001	107.2099991
-5.5346026	13.4688005	106.6100006
-5.5346026	13.5176001	103.7999954
-5.5346026	13.5664005	104.2200012
-5.5346026	13.6152000	102.7799988
-5.5346026	13.6640005	101.7600021
-5.5346026	13.7128000	100.2200012
-5.5346026	13.7616005	100.2500000

-5.5346026	13.8104000	104.5000000
-5.5346026	13.8592005	103.1599960
-5.5346026	13.9080000	97.5499954
-5.5346026	13.9568005	102.9000015
-5.5346026	14.0056000	102.7500000
-5.5346026	14.0544004	99.3600006
-5.5346026	14.1032000	101.0599976
-5.5346026	14.1520004	101.4700012
-5.5346026	14.2007999	103.3600006
-5.5346026	14.2496004	102.4000015
-5.5346026	14.2983999	103.5199966
-5.5346026	14.3472004	104.5299988
-5.5346026	14.3959999	104.2900009
-5.5346026	14.4448004	104.1999969
-5.5346026	14.4935999	103.7799988
-5.5346026	14.5424004	98.8799973
-5.5346026	14.5911999	101.2500000
-5.5346026	14.6400003	103.4599991
-5.5346026	14.6887999	103.5699997
-5.5346026	14.7376003	98.6599960
-5.5346026	14.7863998	100.9000015
-5.5346026	14.8352003	103.4000015
-5.5346026	14.8839998	101.5299988
-5.5346026	14.9328003	95.8199997
-5.5346026	14.9815998	103.9799957
-5.5346026	15.0304003	105.8699951
-5.5284672	0.3416000	104.8300018
-5.5284672	0.3904000	107.1199951
-5.5284672	0.4392000	110.0699997
-5.5284672	0.4880000	109.2900009
-5.5284672	0.5368000	106.1500015
-5.5284672	0.5856000	102.1299973
-5.5284672	0.6344000	102.4300003
-5.5284672	0.6832000	105.0899963
-5.5284672	0.7320000	104.7600021
-5.5284672	0.7808000	102.4799957
-5.5284672	0.8296000	105.8799973
-5.5284672	0.8784000	107.3799973
-5.5284672	0.9272000	108.3600006
-5.5284672	0.9760000	105.4700012
-5.5284672	1.0247999	106.5499954
-5.5284672	1.0735999	106.3600006
-5.5284672	1.1223999	105.3399963
-5.5284672	1.1711999	104.0000000
-5.5284672	1.2200000	103.7399979
-5.5284672	1.2688000	104.5899963
-5.5284672	1.3176000	101.5999985
-5.5284672	1.3664000	99.9599991
-5.5284672	1.4152000	102.3600006
-5.5284672	1.4640000	105.4700012
-5.5284672	1.5128000	106.8199997
-5.5284672	1.5616000	104.4199982
-5.5284672	1.6104000	106.2500000
-5.5284672	1.6592000	106.1899948
-5.5284672	1.7079999	104.2999954
-5.5284672	1.7567999	106.0999985
-5.5284672	1.8055999	106.7799988
-5.5284672	1.8543999	102.5100021

-5.5284672	1.9031999	103.8399963
-5.5284672	1.9519999	101.1800003
-5.5284672	2.0007999	101.4399948
-5.5284672	2.0495999	104.3300018
-5.5284672	2.0983999	104.9399948
-5.5284672	2.1471999	104.4899979
-5.5284672	2.1959999	104.3099976
-5.5284672	2.2447999	105.7299957
-5.5284672	2.2935998	105.2399979
-5.5284672	2.3423998	107.5999985
-5.5284672	2.3912001	104.2900009
-5.5284672	2.4400001	104.0699997
-5.5284672	2.4888000	104.7799988
-5.5284672	2.5376000	107.2200012
-5.5284672	2.5864000	107.7900009
-5.5284672	2.6352000	106.3499985
-5.5284672	2.6840000	105.0400009
-5.5284672	2.7328000	103.8099976
-5.5284672	2.7816000	103.5599976
-5.5284672	2.8304000	103.5299988
-5.5284672	2.8792000	105.8600006
-5.5284672	2.9280000	108.9399948
-5.5284672	2.9768000	108.6899948
-5.5284672	3.0256000	104.2699966
-5.5284672	3.0743999	104.2399979
-5.5284672	3.1231999	103.4700012
-5.5284672	3.1719999	104.3899994
-5.5284672	3.2207999	105.5699997
-5.5284672	3.2695999	105.8499985
-5.5284672	3.3183999	103.7399979
-5.5284672	3.3671999	101.4399948
-5.5284672	3.4159999	100.4899979
-5.5284672	3.4647999	103.9700012
-5.5284672	3.5135999	104.0899963
-5.5284672	3.5623999	105.3300018
-5.5284672	3.6111999	100.8899994
-5.5284672	3.6599998	104.6899948
-5.5284672	3.7087998	104.8099976
-5.5284672	3.7575998	104.1800003
-5.5284672	3.8063998	101.1999969
-5.5284672	3.8551998	98.6299973
-5.5284672	3.9039998	102.7500000
-5.5284672	3.9527998	102.9799957
-5.5284672	4.0016003	99.8600006
-5.5284672	4.0503998	104.3199997
-5.5284672	4.0991998	103.3199997
-5.5284672	4.1479998	99.5400009
-5.5284672	4.1967998	100.9399948
-5.5284672	4.2455997	103.3799973
-5.5284672	4.2943997	103.0199966
-5.5284672	4.3431997	105.5199966
-5.5284672	4.3919997	103.4000015
-5.5284672	4.4407997	99.9700012
-5.5284672	4.4895997	101.3699951
-5.5284672	4.5383997	104.7299957
-5.5284672	4.5871997	105.5100021
-5.5284672	4.6359997	111.0599976
-5.5284672	4.6847997	116.8099976

-5.5284672	4.7336001	114.8300018
-5.5284672	4.7824001	107.4799957
-5.5284672	4.8312001	107.9700012
-5.5284672	4.8800001	110.1399994
-5.5284672	4.9288001	106.9700012
-5.5284672	4.9776001	102.1399994
-5.5284672	5.0264001	105.4499969
-5.5284672	5.0752001	105.3399963
-5.5284672	5.1240001	103.4300003
-5.5284672	5.1728001	100.5499954
-5.5284672	5.2216001	100.1399994
-5.5284672	5.2704000	100.4199982
-5.5284672	5.3192000	102.6199951
-5.5284672	5.3680000	103.7900009
-5.5284672	5.4168000	105.4700012
-5.5284672	5.4656000	105.3600006
-5.5284672	5.5144000	105.7999954
-5.5284672	5.5632000	107.0899963
-5.5284672	5.6120000	103.5199966
-5.5284672	5.6608000	107.0899963
-5.5284672	5.7096000	107.3099976
-5.5284672	5.7584000	104.6599960
-5.5284672	5.8072000	103.5400009
-5.5284672	5.8559999	104.1699982
-5.5284672	5.9047999	104.1999969
-5.5284672	5.9535999	107.3399963
-5.5284672	6.0023999	107.7900009
-5.5284672	6.0511999	107.0000000
-5.5284672	6.0999999	106.8799973
-5.5284672	6.1487999	106.4300003
-5.5284672	6.1975999	104.4899979
-5.5284672	6.2463999	102.4300003
-5.5284672	6.2951999	103.7500000
-5.5284672	6.3439999	103.4000015
-5.5284672	6.3927999	105.2699966
-5.5284672	6.4415998	105.2200012
-5.5284672	6.4903998	105.9700012
-5.5284672	6.5391998	104.1299973
-5.5284672	6.5879998	101.0800018
-5.5284672	6.6367998	102.8199997
-5.5284672	6.6855998	104.3199997
-5.5284672	6.7343998	101.1500015
-5.5284672	6.7831998	101.6399994
-5.5284672	6.8319998	100.3600006
-5.5284672	6.8807998	103.3300018
-5.5284672	6.9295998	106.7399979
-5.5284672	6.9783998	105.1899948
-5.5284672	7.0271997	104.3600006
-5.5284672	7.0759997	101.6199951
-5.5284672	7.1247997	102.5499954
-5.5284672	7.1735997	102.7799988
-5.5284672	7.2223997	105.1699982
-5.5284672	7.2711997	104.5800018
-5.5284672	7.3199997	101.9300003
-5.5284672	7.3687997	100.5299988
-5.5284672	7.4175997	104.7099991
-5.5284672	7.4663997	103.8699951
-5.5284672	7.5151997	102.9099960

-5.5284672	7.5639997	100.9199982
-5.5284672	7.6127996	104.0100021
-5.5284672	7.6615996	104.3799973
-5.5284672	7.7103996	99.0000000
-5.5284672	7.7591996	106.5000000
-5.5284672	7.8079996	105.4000015
-5.5284672	7.8567996	104.4700012
-5.5284672	7.9056001	106.6100006
-5.5284672	7.9544001	106.7299957
-5.5284672	8.0032005	104.5400009
-5.5284672	8.0520000	99.4300003
-5.5284672	8.1008005	103.5000000
-5.5284672	8.1496000	105.5599976
-5.5284672	8.1984005	105.2299957
-5.5284672	8.2472000	104.1800003
-5.5284672	8.2960005	102.9300003
-5.5284672	8.3448000	103.1299973
-5.5284672	8.3936005	101.2099991
-5.5284672	8.4424000	101.1999969
-5.5284672	8.4912004	101.5000000
-5.5284672	8.5400000	103.6399994
-5.5284672	8.5888004	104.7699966
-5.5284672	8.6375999	100.7699966
-5.5284672	8.6864004	101.1899948
-5.5284672	8.7351999	104.8899994
-5.5284672	8.7840004	107.6500015
-5.5284672	8.8327999	106.7299957
-5.5284672	8.8816004	105.3199997
-5.5284672	8.9303999	104.6199951
-5.5284672	8.9792004	104.5999985
-5.5284672	9.0279999	103.1399994
-5.5284672	9.0768003	103.9499969
-5.5284672	9.1255999	107.0299988
-5.5284672	9.1744003	104.1100006
-5.5284672	9.2231998	101.6199951
-5.5284672	9.2720003	104.5999985
-5.5284672	9.3207998	103.3600006
-5.5284672	9.3696003	105.5599976
-5.5284672	9.4184008	104.0100021
-5.5284672	9.4672003	103.6999969
-5.5284672	9.5160007	104.3799973
-5.5284672	9.5648003	104.7500000
-5.5284672	9.6136007	103.2900009
-5.5284672	9.6624002	99.2799988
-5.5284672	9.7112007	98.6599960
-5.5284672	9.7600002	102.7399979
-5.5284672	9.8088007	102.9099960
-5.5284672	9.8576002	104.4599991
-5.5284672	9.9064007	106.5599976
-5.5284672	9.9552002	106.9499969
-5.5284672	10.0040007	105.7200012
-5.5284672	10.0528002	106.2900009
-5.5284672	10.1016006	106.5400009
-5.5284672	10.1504002	106.6100006
-5.5284672	10.1992006	104.3300018
-5.5284672	10.2480001	105.1599960
-5.5284672	10.2968006	106.6100006
-5.5284672	10.3456001	105.7500000

-5.5284672	10.3944006	103.9000015
-5.5284672	10.4432001	102.0999985
-5.5284672	10.4920006	102.4599991
-5.5284672	10.5408001	104.6299973
-5.5284672	10.5896006	106.1100006
-5.5284672	10.6384001	105.2799988
-5.5284672	10.6872005	102.3199997
-5.5284672	10.7360001	101.8600006
-5.5284672	10.7848005	105.2399979
-5.5284672	10.8336000	105.4099960
-5.5284672	10.8824005	104.7600021
-5.5284672	10.9312000	105.9300003
-5.5284672	10.9800005	103.5699997
-5.5284672	11.0288000	100.9599991
-5.5284672	11.0776005	103.2999954
-5.5284672	11.1264000	105.0100021
-5.5284672	11.1752005	102.8099976
-5.5284672	11.2240000	105.5400009
-5.5284672	11.2728004	102.8399963
-5.5284672	11.3216000	100.2799988
-5.5284672	11.3704004	101.4000015
-5.5284672	11.4191999	104.6500015
-5.5284672	11.4680004	104.1399994
-5.5284672	11.5167999	105.9599991
-5.5284672	11.5656004	104.5499954
-5.5284672	11.6143999	99.9599991
-5.5284672	11.6632004	104.6100006
-5.5284672	11.7119999	104.9000015
-5.5284672	11.7608004	106.0100021
-5.5284672	11.8095999	105.6399994
-5.5284672	11.8584003	105.7399979
-5.5284672	11.9071999	105.0299988
-5.5284672	11.9560003	105.0999985
-5.5284672	12.0047998	105.5100021
-5.5284672	12.0536003	103.1199951
-5.5284672	12.1023998	106.0499954
-5.5284672	12.1512003	103.9799957
-5.5284672	12.1999998	104.1999969
-5.5284672	12.2488003	103.9300003
-5.5284672	12.2975998	103.4000015
-5.5284672	12.3464003	101.0899963
-5.5284672	12.3951998	102.4899979
-5.5284672	12.4440002	104.9899979
-5.5284672	12.4927998	98.4399948
-5.5284672	12.5416002	103.4899979
-5.5284672	12.5904007	102.0100021
-5.5284672	12.6392002	102.4700012
-5.5284672	12.6880007	101.3699951
-5.5284672	12.7368002	101.5899963
-5.5284672	12.7856007	103.1899948
-5.5284672	12.8344002	102.9499969
-5.5284672	12.8832006	100.1699982
-5.5284672	12.9320002	103.2900009
-5.5284672	12.9808006	103.8799973
-5.5284672	13.0296001	99.4000015
-5.5284672	13.0784006	103.1500015
-5.5284672	13.1272001	104.7099991
-5.5284672	13.1760006	104.0400009

-5.5284672	13.2248001	101.1699982
-5.5284672	13.2736006	102.0499954
-5.5284672	13.3224001	103.6500015
-5.5284672	13.3712006	105.3699951
-5.5284672	13.4200001	106.1999969
-5.5284672	13.4688005	105.8099976
-5.5284672	13.5176001	104.1599960
-5.5284672	13.5664005	104.7799988
-5.5284672	13.6152000	103.7299957
-5.5284672	13.6640005	101.4499969
-5.5284672	13.7128000	100.9899979
-5.5284672	13.7616005	100.8199997
-5.5284672	13.8104000	104.7900009
-5.5284672	13.8592005	103.3600006
-5.5284672	13.9080000	98.1699982
-5.5284672	13.9568005	102.2999954
-5.5284672	14.0056000	103.2200012
-5.5284672	14.0544004	100.1500015
-5.5284672	14.1032000	101.7299957
-5.5284672	14.1520004	102.5199966
-5.5284672	14.2007999	103.6299973
-5.5284672	14.2496004	102.1399994
-5.5284672	14.2983999	103.8499985
-5.5284672	14.3472004	104.7299957
-5.5284672	14.3959999	104.0599976
-5.5284672	14.4448004	103.9899979
-5.5284672	14.4935999	103.8600006
-5.5284672	14.5424004	99.7500000
-5.5284672	14.5911999	102.2099991
-5.5284672	14.6400003	104.1599960
-5.5284672	14.6887999	104.1699982
-5.5284672	14.7376003	99.4799957
-5.5284672	14.7863998	100.2099991
-5.5284672	14.8352003	102.9000015
-5.5284672	14.8839998	102.3499985
-5.5284672	14.9328003	95.9300003
-5.5284672	14.9815998	103.6800003
-5.5284672	15.0304003	104.9199982
-5.5223312	0.3416000	105.6100006
-5.5223312	0.3904000	107.8499985
-5.5223312	0.4392000	110.0000000
-5.5223312	0.4880000	109.2500000
-5.5223312	0.5368000	105.7200012
-5.5223312	0.5856000	102.2799988
-5.5223312	0.6344000	103.6599960
-5.5223312	0.6832000	105.0199966
-5.5223312	0.7320000	103.5400009
-5.5223312	0.7808000	101.4700012
-5.5223312	0.8296000	105.6399994
-5.5223312	0.8784000	107.8099976
-5.5223312	0.9272000	107.3199997
-5.5223312	0.9760000	103.1800003
-5.5223312	1.0247999	104.4300003
-5.5223312	1.0735999	105.8300018
-5.5223312	1.1223999	105.5999985
-5.5223312	1.1711999	104.7999954
-5.5223312	1.2200000	103.1500015
-5.5223312	1.2688000	104.5100021

-5.5223312	1.3176000	102.1299973
-5.5223312	1.3664000	101.3499985
-5.5223312	1.4152000	103.0499954
-5.5223312	1.4640000	106.1800003
-5.5223312	1.5128000	107.6100006
-5.5223312	1.5616000	104.3499985
-5.5223312	1.6104000	106.1299973
-5.5223312	1.6592000	106.4199982
-5.5223312	1.7079999	104.8099976
-5.5223312	1.7567999	105.8600006
-5.5223312	1.8055999	106.3799973
-5.5223312	1.8543999	102.2399979
-5.5223312	1.9031999	102.8399963
-5.5223312	1.9519999	99.8699951
-5.5223312	2.0007999	100.6599960
-5.5223312	2.0495999	103.9799957
-5.5223312	2.0983999	104.4300003
-5.5223312	2.1471999	103.8899994
-5.5223312	2.1959999	105.0199966
-5.5223312	2.2447999	106.5199966
-5.5223312	2.2935998	104.2600021
-5.5223312	2.3423998	106.7799988
-5.5223312	2.3912001	103.8799973
-5.5223312	2.4400001	104.9499969
-5.5223312	2.4888000	103.8600006
-5.5223312	2.5376000	106.5800018
-5.5223312	2.5864000	107.0000000
-5.5223312	2.6352000	106.0800018
-5.5223312	2.6840000	104.9899979
-5.5223312	2.7328000	104.4300003
-5.5223312	2.7816000	103.5299988
-5.5223312	2.8304000	103.0699997
-5.5223312	2.8792000	105.5699997
-5.5223312	2.9280000	108.4099960
-5.5223312	2.9768000	108.2399979
-5.5223312	3.0256000	104.4599991
-5.5223312	3.0743999	103.5800018
-5.5223312	3.1231999	103.4700012
-5.5223312	3.1719999	103.9399948
-5.5223312	3.2207999	105.0699997
-5.5223312	3.2695999	105.4300003
-5.5223312	3.3183999	103.6500015
-5.5223312	3.3671999	101.4499969
-5.5223312	3.4159999	98.7699966
-5.5223312	3.4647999	102.3099976
-5.5223312	3.5135999	102.3799973
-5.5223312	3.5623999	104.5899963
-5.5223312	3.6111999	101.1100006
-5.5223312	3.6599998	104.9000015
-5.5223312	3.7087998	104.6299973
-5.5223312	3.7575998	103.7799988
-5.5223312	3.8063998	101.9799957
-5.5223312	3.8551998	99.4000015
-5.5223312	3.9039998	102.2299957
-5.5223312	3.9527998	103.0400009
-5.5223312	4.0016003	100.7699966
-5.5223312	4.0503998	104.5499954
-5.5223312	4.0991998	104.0100021

-5.5223312	4.1479998	101.0199966
-5.5223312	4.1967998	101.6399994
-5.5223312	4.2455997	104.0100021
-5.5223312	4.2943997	103.7600021
-5.5223312	4.3431997	106.0199966
-5.5223312	4.3919997	102.9799957
-5.5223312	4.4407997	99.9899979
-5.5223312	4.4895997	102.1299973
-5.5223312	4.5383997	105.1500015
-5.5223312	4.5871997	106.0100021
-5.5223312	4.6359997	111.6899948
-5.5223312	4.6847997	116.6399994
-5.5223312	4.7336001	114.5100021
-5.5223312	4.7824001	107.6199951
-5.5223312	4.8312001	108.0699997
-5.5223312	4.8800001	109.9899979
-5.5223312	4.9288001	106.8899994
-5.5223312	4.9776001	101.5999985
-5.5223312	5.0264001	105.1800003
-5.5223312	5.0752001	105.8699951
-5.5223312	5.1240001	104.2900009
-5.5223312	5.1728001	100.5100021
-5.5223312	5.2216001	101.0499954
-5.5223312	5.2704000	100.7500000
-5.5223312	5.3192000	101.8799973
-5.5223312	5.3680000	103.4300003
-5.5223312	5.4168000	105.2999954
-5.5223312	5.4656000	104.4300003
-5.5223312	5.5144000	104.6999969
-5.5223312	5.5632000	106.7699966
-5.5223312	5.6120000	103.9199982
-5.5223312	5.6608000	106.9899979
-5.5223312	5.7096000	107.0599976
-5.5223312	5.7584000	104.1999969
-5.5223312	5.8072000	102.3099976
-5.5223312	5.8559999	103.5599976
-5.5223312	5.9047999	104.4599991
-5.5223312	5.9535999	107.4300003
-5.5223312	6.0023999	107.4700012
-5.5223312	6.0511999	106.1199951
-5.5223312	6.0999999	106.9599991
-5.5223312	6.1487999	107.4300003
-5.5223312	6.1975999	106.1699982
-5.5223312	6.2463999	104.2699966
-5.5223312	6.2951999	104.1699982
-5.5223312	6.3439999	103.7099991
-5.5223312	6.3927999	105.9899979
-5.5223312	6.4415998	105.7500000
-5.5223312	6.4903998	106.7099991
-5.5223312	6.5391998	103.9399948
-5.5223312	6.5879998	100.7699966
-5.5223312	6.6367998	102.4499969
-5.5223312	6.6855998	103.3399963
-5.5223312	6.7343998	99.8799973
-5.5223312	6.7831998	101.0899963
-5.5223312	6.8319998	99.9300003
-5.5223312	6.8807998	103.6699982
-5.5223312	6.9295998	106.9000015

-5.5223312	6.9783998	104.5699997
-5.5223312	7.0271997	103.7099991
-5.5223312	7.0759997	101.2999954
-5.5223312	7.1247997	103.2600021
-5.5223312	7.1735997	103.1399994
-5.5223312	7.2223997	104.2500000
-5.5223312	7.2711997	103.9000015
-5.5223312	7.3199997	101.5499954
-5.5223312	7.3687997	101.0400009
-5.5223312	7.4175997	104.5100021
-5.5223312	7.4663997	103.2799988
-5.5223312	7.5151997	101.6599960
-5.5223312	7.5639997	101.0899963
-5.5223312	7.6127996	104.4399948
-5.5223312	7.6615996	104.3300018
-5.5223312	7.7103996	98.7999954
-5.5223312	7.7591996	106.5299988
-5.5223312	7.8079996	105.2999954
-5.5223312	7.8567996	104.7699966
-5.5223312	7.9056001	107.0100021
-5.5223312	7.9544001	107.4000015
-5.5223312	8.0032005	104.7200012
-5.5223312	8.0520000	99.5599976
-5.5223312	8.1008005	102.9399948
-5.5223312	8.1496000	105.3899994
-5.5223312	8.1984005	105.7399979
-5.5223312	8.2472000	104.7299957
-5.5223312	8.2960005	103.5699997
-5.5223312	8.3448000	103.5999985
-5.5223312	8.3936005	102.4300003
-5.5223312	8.4424000	102.4700012
-5.5223312	8.4912004	101.4399948
-5.5223312	8.5400000	102.6999969
-5.5223312	8.5888004	104.7799988
-5.5223312	8.6375999	100.5199966
-5.5223312	8.6864004	100.7900009
-5.5223312	8.7351999	105.0299988
-5.5223312	8.7840004	107.4599991
-5.5223312	8.8327999	106.5699997
-5.5223312	8.8816004	106.0999985
-5.5223312	8.9303999	106.0599976
-5.5223312	8.9792004	105.2099991
-5.5223312	9.0279999	102.8699951
-5.5223312	9.0768003	105.0100021
-5.5223312	9.1255999	106.8899994
-5.5223312	9.1744003	103.7600021
-5.5223312	9.2231998	101.7399979
-5.5223312	9.2720003	103.9399948
-5.5223312	9.3207998	102.4099960
-5.5223312	9.3696003	105.6699982
-5.5223312	9.4184008	104.2500000
-5.5223312	9.4672003	102.8600006
-5.5223312	9.5160007	103.2600021
-5.5223312	9.5648003	104.4300003
-5.5223312	9.6136007	103.6299973
-5.5223312	9.6624002	100.8600006
-5.5223312	9.7112007	99.9700012
-5.5223312	9.7600002	102.6999969

-5.5223312	9.8088007	102.9300003
-5.5223312	9.8576002	103.4499969
-5.5223312	9.9064007	105.1399994
-5.5223312	9.9552002	105.3300018
-5.5223312	10.0040007	104.5499954
-5.5223312	10.0528002	106.1299973
-5.5223312	10.1016006	105.9199982
-5.5223312	10.1504002	106.1800003
-5.5223312	10.1992006	103.4300003
-5.5223312	10.2480001	103.9700012
-5.5223312	10.2968006	105.4000015
-5.5223312	10.3456001	105.3499985
-5.5223312	10.3944006	103.6399994
-5.5223312	10.4432001	102.5899963
-5.5223312	10.4920006	102.5000000
-5.5223312	10.5408001	104.4499969
-5.5223312	10.5896006	105.9000015
-5.5223312	10.6384001	104.5000000
-5.5223312	10.6872005	102.0299988
-5.5223312	10.7360001	101.2500000
-5.5223312	10.7848005	105.0299988
-5.5223312	10.8336000	105.1199951
-5.5223312	10.8824005	104.6399994
-5.5223312	10.9312000	105.3099976
-5.5223312	10.9800005	102.6199951
-5.5223312	11.0288000	101.2299957
-5.5223312	11.0776005	103.6800003
-5.5223312	11.1264000	105.1100006
-5.5223312	11.1752005	102.2399979
-5.5223312	11.2240000	104.9099960
-5.5223312	11.2728004	103.2999954
-5.5223312	11.3216000	98.7500000
-5.5223312	11.3704004	101.0400009
-5.5223312	11.4191999	104.5899963
-5.5223312	11.4680004	104.6299973
-5.5223312	11.5167999	106.3899994
-5.5223312	11.5656004	104.7799988
-5.5223312	11.6143999	99.2399979
-5.5223312	11.6632004	104.1399994
-5.5223312	11.7119999	105.0999985
-5.5223312	11.7608004	105.6299973
-5.5223312	11.8095999	104.3699951
-5.5223312	11.8584003	104.8799973
-5.5223312	11.9071999	104.2799988
-5.5223312	11.9560003	104.5100021
-5.5223312	12.0047998	105.5800018
-5.5223312	12.0536003	104.7999954
-5.5223312	12.1023998	106.5899963
-5.5223312	12.1512003	103.5299988
-5.5223312	12.1999998	104.5199966
-5.5223312	12.2488003	104.1599960
-5.5223312	12.2975998	103.2600021
-5.5223312	12.3464003	101.7799988
-5.5223312	12.3951998	102.7500000
-5.5223312	12.4440002	105.5899963
-5.5223312	12.4927998	100.4899979
-5.5223312	12.5416002	103.6100006
-5.5223312	12.5904007	102.0000000

-5.5223312	12.6392002	102.5800018
-5.5223312	12.6880007	101.4599991
-5.5223312	12.7368002	101.4300003
-5.5223312	12.7856007	103.3300018
-5.5223312	12.8344002	103.7399979
-5.5223312	12.8832006	100.6800003
-5.5223312	12.9320002	103.0999985
-5.5223312	12.9808006	104.2900009
-5.5223312	13.0296001	100.7600021
-5.5223312	13.0784006	102.9700012
-5.5223312	13.1272001	103.8899994
-5.5223312	13.1760006	102.7399979
-5.5223312	13.2248001	100.5400009
-5.5223312	13.2736006	101.5999985
-5.5223312	13.3224001	102.9700012
-5.5223312	13.3712006	104.8600006
-5.5223312	13.4200001	105.5299988
-5.5223312	13.4688005	104.9099960
-5.5223312	13.5176001	104.2399979
-5.5223312	13.5664005	105.2600021
-5.5223312	13.6152000	104.1899948
-5.5223312	13.6640005	100.8600006
-5.5223312	13.7128000	101.8799973
-5.5223312	13.7616005	101.6599960
-5.5223312	13.8104000	104.9000015
-5.5223312	13.8592005	103.3899994
-5.5223312	13.9080000	98.7600021
-5.5223312	13.9568005	101.6599960
-5.5223312	14.0056000	103.9099960
-5.5223312	14.0544004	101.7699966
-5.5223312	14.1032000	102.5000000
-5.5223312	14.1520004	103.4899979
-5.5223312	14.2007999	104.0000000
-5.5223312	14.2496004	101.3799973
-5.5223312	14.2983999	103.5499954
-5.5223312	14.3472004	104.1399994
-5.5223312	14.3959999	102.9300003
-5.5223312	14.4448004	103.5299988
-5.5223312	14.4935999	104.3099976
-5.5223312	14.5424004	101.3399963
-5.5223312	14.5911999	103.0899963
-5.5223312	14.6400003	103.7999954
-5.5223312	14.6887999	104.1500015
-5.5223312	14.7376003	100.7900009
-5.5223312	14.7863998	100.9799957
-5.5223312	14.8352003	103.0199966
-5.5223312	14.8839998	103.2900009
-5.5223312	14.9328003	96.0999985
-5.5223312	14.9815998	103.7099991
-5.5223312	15.0304003	104.2699966
-5.5161953	0.3416000	106.1699982
-5.5161953	0.3904000	108.4000015
-5.5161953	0.4392000	109.6899948
-5.5161953	0.4880000	108.9799957
-5.5161953	0.5368000	105.3799973
-5.5161953	0.5856000	103.1599960
-5.5161953	0.6344000	105.1299973
-5.5161953	0.6832000	105.3300018

-5.5161953	0.7320000	102.9300003
-5.5161953	0.7808000	101.4300003
-5.5161953	0.8296000	105.1999969
-5.5161953	0.8784000	107.7500000
-5.5161953	0.9272000	106.0400009
-5.5161953	0.9760000	102.1500015
-5.5161953	1.0247999	103.0999985
-5.5161953	1.0735999	105.5299988
-5.5161953	1.1223999	106.2099991
-5.5161953	1.1711999	105.8300018
-5.5161953	1.2200000	102.4599991
-5.5161953	1.2688000	103.8199997
-5.5161953	1.3176000	101.9199982
-5.5161953	1.3664000	101.3699951
-5.5161953	1.4152000	102.8699951
-5.5161953	1.4640000	106.4000015
-5.5161953	1.5128000	108.2399979
-5.5161953	1.5616000	103.6999969
-5.5161953	1.6104000	105.8799973
-5.5161953	1.6592000	105.6399994
-5.5161953	1.7079999	105.0199966
-5.5161953	1.7567999	105.6100006
-5.5161953	1.8055999	106.1500015
-5.5161953	1.8543999	102.3199997
-5.5161953	1.9031999	102.2799988
-5.5161953	1.9519999	99.4899979
-5.5161953	2.0007999	100.6100006
-5.5161953	2.0495999	103.5299988
-5.5161953	2.0983999	103.4199982
-5.5161953	2.1471999	102.6800003
-5.5161953	2.1959999	105.0599976
-5.5161953	2.2447999	106.7099991
-5.5161953	2.2935998	103.3099976
-5.5161953	2.3423998	106.3300018
-5.5161953	2.3912001	105.1399994
-5.5161953	2.4400001	105.7600021
-5.5161953	2.4888000	102.9199982
-5.5161953	2.5376000	105.9799957
-5.5161953	2.5864000	106.2999954
-5.5161953	2.6352000	106.5100021
-5.5161953	2.6840000	105.0899963
-5.5161953	2.7328000	104.7999954
-5.5161953	2.7816000	103.2799988
-5.5161953	2.8304000	101.9799957
-5.5161953	2.8792000	105.2799988
-5.5161953	2.9280000	107.3899994
-5.5161953	2.9768000	107.2200012
-5.5161953	3.0256000	103.9399948
-5.5161953	3.0743999	102.7799988
-5.5161953	3.1231999	102.7399979
-5.5161953	3.1719999	103.5299988
-5.5161953	3.2207999	104.9799957
-5.5161953	3.2695999	105.1500015
-5.5161953	3.3183999	103.2099991
-5.5161953	3.3671999	100.7399979
-5.5161953	3.4159999	98.2200012
-5.5161953	3.4647999	101.2399979
-5.5161953	3.5135999	101.3699951

-5.5161953	3.5623999	103.9000015
-5.5161953	3.6111999	102.2600021
-5.5161953	3.6599998	104.7299957
-5.5161953	3.7087998	104.0299988
-5.5161953	3.7575998	103.7299957
-5.5161953	3.8063998	102.7600021
-5.5161953	3.8551998	99.8199997
-5.5161953	3.9039998	101.2799988
-5.5161953	3.9527998	102.5199966
-5.5161953	4.0016003	101.2799988
-5.5161953	4.0503998	102.9799957
-5.5161953	4.0991998	104.7799988
-5.5161953	4.1479998	102.2600021
-5.5161953	4.1967998	102.4799957
-5.5161953	4.2455997	104.0499954
-5.5161953	4.2943997	104.0299988
-5.5161953	4.3431997	106.4300003
-5.5161953	4.3919997	103.0100021
-5.5161953	4.4407997	100.7699966
-5.5161953	4.4895997	102.7299957
-5.5161953	4.5383997	105.0800018
-5.5161953	4.5871997	105.9799957
-5.5161953	4.6359997	111.0899963
-5.5161953	4.6847997	116.2999954
-5.5161953	4.7336001	114.5899963
-5.5161953	4.7824001	107.9599991
-5.5161953	4.8312001	108.0000000
-5.5161953	4.8800001	109.7500000
-5.5161953	4.9288001	106.9599991
-5.5161953	4.9776001	101.6299973
-5.5161953	5.0264001	105.4799957
-5.5161953	5.0752001	106.2600021
-5.5161953	5.1240001	104.9799957
-5.5161953	5.1728001	101.6399994
-5.5161953	5.2216001	102.4300003
-5.5161953	5.2704000	101.4199982
-5.5161953	5.3192000	101.5299988
-5.5161953	5.3680000	103.3699951
-5.5161953	5.4168000	105.3899994
-5.5161953	5.4656000	104.3099976
-5.5161953	5.5144000	104.3399963
-5.5161953	5.5632000	106.1100006
-5.5161953	5.6120000	103.2099991
-5.5161953	5.6608000	105.5100021
-5.5161953	5.7096000	106.5699997
-5.5161953	5.7584000	104.1899948
-5.5161953	5.8072000	101.2399979
-5.5161953	5.8559999	102.6100006
-5.5161953	5.9047999	104.0699997
-5.5161953	5.9535999	106.4399948
-5.5161953	6.0023999	106.5699997
-5.5161953	6.0511999	105.0499954
-5.5161953	6.0999999	106.7299957
-5.5161953	6.1487999	107.9499969
-5.5161953	6.1975999	107.4000015
-5.5161953	6.2463999	105.7299957
-5.5161953	6.2951999	104.6599960
-5.5161953	6.3439999	104.1100006

-5.5161953	6.3927999	106.1599960
-5.5161953	6.4415998	105.6699982
-5.5161953	6.4903998	106.9099960
-5.5161953	6.5391998	104.2999954
-5.5161953	6.5879998	101.4700012
-5.5161953	6.6367998	102.6800003
-5.5161953	6.6855998	102.5800018
-5.5161953	6.7343998	99.5100021
-5.5161953	6.7831998	101.0299988
-5.5161953	6.8319998	100.2099991
-5.5161953	6.8807998	103.8300018
-5.5161953	6.9295998	106.7200012
-5.5161953	6.9783998	105.0999985
-5.5161953	7.0271997	104.3399963
-5.5161953	7.0759997	101.9700012
-5.5161953	7.1247997	104.1399994
-5.5161953	7.1735997	103.6100006
-5.5161953	7.2223997	102.9899979
-5.5161953	7.2711997	102.9000015
-5.5161953	7.3199997	102.1500015
-5.5161953	7.3687997	101.6100006
-5.5161953	7.4175997	104.2900009
-5.5161953	7.4663997	103.2299957
-5.5161953	7.5151997	100.8399963
-5.5161953	7.5639997	101.6599960
-5.5161953	7.6127996	104.7099991
-5.5161953	7.6615996	104.4700012
-5.5161953	7.7103996	100.1100006
-5.5161953	7.7591996	106.4799957
-5.5161953	7.8079996	105.5000000
-5.5161953	7.8567996	105.0499954
-5.5161953	7.9056001	106.9700012
-5.5161953	7.9544001	107.4399948
-5.5161953	8.0032005	104.0199966
-5.5161953	8.0520000	98.8300018
-5.5161953	8.1008005	102.6199951
-5.5161953	8.1496000	105.3399963
-5.5161953	8.1984005	106.0000000
-5.5161953	8.2472000	105.2500000
-5.5161953	8.2960005	104.4599991
-5.5161953	8.3448000	103.7399979
-5.5161953	8.3936005	103.3799973
-5.5161953	8.4424000	103.4000015
-5.5161953	8.4912004	100.9599991
-5.5161953	8.5400000	102.6599960
-5.5161953	8.5888004	104.8300018
-5.5161953	8.6375999	99.5199966
-5.5161953	8.6864004	100.1500015
-5.5161953	8.7351999	104.6100006
-5.5161953	8.7840004	106.7500000
-5.5161953	8.8327999	106.1199951
-5.5161953	8.8816004	106.3499985
-5.5161953	8.9303999	106.8600006
-5.5161953	8.9792004	105.3699951
-5.5161953	9.0279999	102.8899994
-5.5161953	9.0768003	105.8300018
-5.5161953	9.1255999	106.5199966
-5.5161953	9.1744003	103.0499954

-5.5161953	9.2231998	101.7900009
-5.5161953	9.2720003	103.5400009
-5.5161953	9.3207998	102.2600021
-5.5161953	9.3696003	105.8499985
-5.5161953	9.4184008	104.4300003
-5.5161953	9.4672003	101.8600006
-5.5161953	9.5160007	101.5000000
-5.5161953	9.5648003	103.3600006
-5.5161953	9.6136007	103.4499969
-5.5161953	9.6624002	102.3899994
-5.5161953	9.7112007	101.4399948
-5.5161953	9.7600002	103.1999969
-5.5161953	9.8088007	103.4199982
-5.5161953	9.8576002	102.8499985
-5.5161953	9.9064007	104.0199966
-5.5161953	9.9552002	103.8399963
-5.5161953	10.0040007	103.4099960
-5.5161953	10.0528002	105.9499969
-5.5161953	10.1016006	105.2299957
-5.5161953	10.1504002	105.6800003
-5.5161953	10.1992006	103.2500000
-5.5161953	10.2480001	102.9099960
-5.5161953	10.2968006	104.2399979
-5.5161953	10.3456001	105.1199951
-5.5161953	10.3944006	103.7999954
-5.5161953	10.4432001	103.0899963
-5.5161953	10.4920006	102.8699951
-5.5161953	10.5408001	104.7999954
-5.5161953	10.5896006	105.8300018
-5.5161953	10.6384001	103.1800003
-5.5161953	10.6872005	100.4199982
-5.5161953	10.7360001	101.3699951
-5.5161953	10.7848005	104.7600021
-5.5161953	10.8336000	104.6800003
-5.5161953	10.8824005	104.7099991
-5.5161953	10.9312000	104.8600006
-5.5161953	10.9800005	101.9399948
-5.5161953	11.0288000	101.3499985
-5.5161953	11.0776005	104.2399979
-5.5161953	11.1264000	105.1199951
-5.5161953	11.1752005	102.1299973
-5.5161953	11.2240000	104.1399994
-5.5161953	11.2728004	103.2999954
-5.5161953	11.3216000	97.7399979
-5.5161953	11.3704004	101.0199966
-5.5161953	11.4191999	104.5899963
-5.5161953	11.4680004	105.5100021
-5.5161953	11.5167999	106.8899994
-5.5161953	11.5656004	105.0599976
-5.5161953	11.6143999	99.4099960
-5.5161953	11.6632004	103.4899979
-5.5161953	11.7119999	104.3099976
-5.5161953	11.7608004	104.8499985
-5.5161953	11.8095999	103.2699966
-5.5161953	11.8584003	104.0999985
-5.5161953	11.9071999	103.2999954
-5.5161953	11.9560003	103.8899994
-5.5161953	12.0047998	105.3799973

-5.5161953	12.0536003	105.9499969
-5.5161953	12.1023998	106.8300018
-5.5161953	12.1512003	102.8899994
-5.5161953	12.1999998	104.5100021
-5.5161953	12.2488003	104.2799988
-5.5161953	12.2975998	103.6199951
-5.5161953	12.3464003	102.9599991
-5.5161953	12.3951998	103.2799988
-5.5161953	12.4440002	105.8499985
-5.5161953	12.4927998	101.5899963
-5.5161953	12.5416002	103.3199997
-5.5161953	12.5904007	101.7500000
-5.5161953	12.6392002	102.4700012
-5.5161953	12.6880007	102.0699997
-5.5161953	12.7368002	101.3099976
-5.5161953	12.7856007	103.1299973
-5.5161953	12.8344002	103.9599991
-5.5161953	12.8832006	101.0800018
-5.5161953	12.9320002	103.2600021
-5.5161953	12.9808006	104.4099960
-5.5161953	13.0296001	101.6800003
-5.5161953	13.0784006	102.9599991
-5.5161953	13.1272001	103.3899994
-5.5161953	13.1760006	102.2099991
-5.5161953	13.2248001	101.2200012
-5.5161953	13.2736006	101.5000000
-5.5161953	13.3224001	101.7999954
-5.5161953	13.3712006	103.7299957
-5.5161953	13.4200001	103.9899979
-5.5161953	13.4688005	103.0899963
-5.5161953	13.5176001	103.7399979
-5.5161953	13.5664005	104.9899979
-5.5161953	13.6152000	104.4599991
-5.5161953	13.6640005	100.8799973
-5.5161953	13.7128000	102.8499985
-5.5161953	13.7616005	102.3799973
-5.5161953	13.8104000	104.3499985
-5.5161953	13.8592005	102.0499954
-5.5161953	13.9080000	99.1100006
-5.5161953	13.9568005	101.0100021
-5.5161953	14.0056000	104.2299957
-5.5161953	14.0544004	103.0899963
-5.5161953	14.1032000	103.4199982
-5.5161953	14.1520004	104.2399979
-5.5161953	14.2007999	104.0599976
-5.5161953	14.2496004	100.1800003
-5.5161953	14.2983999	102.4199982
-5.5161953	14.3472004	103.2999954
-5.5161953	14.3959999	101.4899979
-5.5161953	14.4448004	102.4499969
-5.5161953	14.4935999	103.9799957
-5.5161953	14.5424004	102.6800003
-5.5161953	14.5911999	103.8600006
-5.5161953	14.6400003	103.1199951
-5.5161953	14.6887999	103.2699966
-5.5161953	14.7376003	101.3399963
-5.5161953	14.7863998	100.9300003
-5.5161953	14.8352003	102.5100021

-5.5161953	14.8839998	103.9700012
-5.5161953	14.9328003	98.3699951
-5.5161953	14.9815998	103.9499969
-5.5161953	15.0304003	104.1800003
-5.5100594	0.3416000	106.5899963
-5.5100594	0.3904000	108.9899979
-5.5100594	0.4392000	109.2699966
-5.5100594	0.4880000	108.2799988
-5.5100594	0.5368000	104.9700012
-5.5100594	0.5856000	104.0800018
-5.5100594	0.6344000	106.2399979
-5.5100594	0.6832000	105.4899979
-5.5100594	0.7320000	102.5800018
-5.5100594	0.7808000	101.4399948
-5.5100594	0.8296000	105.1599960
-5.5100594	0.8784000	108.0599976
-5.5100594	0.9272000	105.5000000
-5.5100594	0.9760000	102.4599991
-5.5100594	1.0247999	102.0400009
-5.5100594	1.0735999	105.4300003
-5.5100594	1.1223999	107.1500015
-5.5100594	1.1711999	106.2999954
-5.5100594	1.2200000	101.6299973
-5.5100594	1.2688000	103.6399994
-5.5100594	1.3176000	103.1999969
-5.5100594	1.3664000	102.6599960
-5.5100594	1.4152000	102.8300018
-5.5100594	1.4640000	105.9499969
-5.5100594	1.5128000	108.3899994
-5.5100594	1.5616000	104.6299973
-5.5100594	1.6104000	105.9000015
-5.5100594	1.6592000	104.9000015
-5.5100594	1.7079999	105.1999969
-5.5100594	1.7567999	105.4300003
-5.5100594	1.8055999	106.2900009
-5.5100594	1.8543999	103.6899948
-5.5100594	1.9031999	102.4000015
-5.5100594	1.9519999	99.4099960
-5.5100594	2.0007999	101.0100021
-5.5100594	2.0495999	103.2200012
-5.5100594	2.0983999	102.8600006
-5.5100594	2.1471999	102.2099991
-5.5100594	2.1959999	105.3600006
-5.5100594	2.2447999	106.9199982
-5.5100594	2.2935998	102.5000000
-5.5100594	2.3423998	105.6899948
-5.5100594	2.3912001	106.1699982
-5.5100594	2.4400001	106.8799973
-5.5100594	2.4888000	103.3300018
-5.5100594	2.5376000	105.7099991
-5.5100594	2.5864000	105.6599960
-5.5100594	2.6352000	106.8799973
-5.5100594	2.6840000	105.2999954
-5.5100594	2.7328000	105.1399994
-5.5100594	2.7816000	103.7600021
-5.5100594	2.8304000	101.5000000
-5.5100594	2.8792000	105.3399963
-5.5100594	2.9280000	106.9599991

-5.5100594	2.9768000	106.6899948
-5.5100594	3.0256000	103.8699951
-5.5100594	3.0743999	103.1299973
-5.5100594	3.1231999	102.9599991
-5.5100594	3.1719999	103.7500000
-5.5100594	3.2207999	105.2799988
-5.5100594	3.2695999	105.1699982
-5.5100594	3.3183999	103.3499985
-5.5100594	3.3671999	100.7299957
-5.5100594	3.4159999	98.6199951
-5.5100594	3.4647999	101.0800018
-5.5100594	3.5135999	100.1599960
-5.5100594	3.5623999	103.4199982
-5.5100594	3.6111999	103.5100021
-5.5100594	3.6599998	104.5499954
-5.5100594	3.7087998	103.6100006
-5.5100594	3.7575998	104.0299988
-5.5100594	3.8063998	103.8399963
-5.5100594	3.8551998	101.9300003
-5.5100594	3.9039998	100.9899979
-5.5100594	3.9527998	102.3799973
-5.5100594	4.0016003	102.5299988
-5.5100594	4.0503998	102.0699997
-5.5100594	4.0991998	105.2999954
-5.5100594	4.1479998	102.9399948
-5.5100594	4.1967998	102.8399963
-5.5100594	4.2455997	104.2999954
-5.5100594	4.2943997	104.4799957
-5.5100594	4.3431997	106.5100021
-5.5100594	4.3919997	103.0299988
-5.5100594	4.4407997	101.3399963
-5.5100594	4.4895997	102.7900009
-5.5100594	4.5383997	104.6800003
-5.5100594	4.5871997	106.3199997
-5.5100594	4.6359997	110.6999969
-5.5100594	4.6847997	116.2500000
-5.5100594	4.7336001	114.9799957
-5.5100594	4.7824001	108.1899948
-5.5100594	4.8312001	107.4000015
-5.5100594	4.8800001	109.2500000
-5.5100594	4.9288001	106.8600006
-5.5100594	4.9776001	102.2399979
-5.5100594	5.0264001	105.9599991
-5.5100594	5.0752001	106.7099991
-5.5100594	5.1240001	105.6800003
-5.5100594	5.1728001	102.6800003
-5.5100594	5.2216001	104.0499954
-5.5100594	5.2704000	102.9700012
-5.5100594	5.3192000	101.7099991
-5.5100594	5.3680000	104.1599960
-5.5100594	5.4168000	105.8699951
-5.5100594	5.4656000	104.3799973
-5.5100594	5.5144000	104.0699997
-5.5100594	5.5632000	105.4799957
-5.5100594	5.6120000	103.4599991
-5.5100594	5.6608000	104.1800003
-5.5100594	5.7096000	105.0100021
-5.5100594	5.7584000	103.8399963

-5.5100594	5.8072000	100.9899979
-5.5100594	5.8559999	102.8099976
-5.5100594	5.9047999	104.2299957
-5.5100594	5.9535999	105.6999969
-5.5100594	6.0023999	105.3899994
-5.5100594	6.0511999	103.2200012
-5.5100594	6.0999999	105.8699951
-5.5100594	6.1487999	107.6100006
-5.5100594	6.1975999	108.0800018
-5.5100594	6.2463999	106.6399994
-5.5100594	6.2951999	105.6999969
-5.5100594	6.3439999	104.7299957
-5.5100594	6.3927999	106.3799973
-5.5100594	6.4415998	105.8899994
-5.5100594	6.4903998	107.3399963
-5.5100594	6.5391998	105.2200012
-5.5100594	6.5879998	102.7099991
-5.5100594	6.6367998	103.2799988
-5.5100594	6.6855998	102.2900009
-5.5100594	6.7343998	100.6299973
-5.5100594	6.7831998	101.8799973
-5.5100594	6.8319998	101.1999969
-5.5100594	6.8807998	103.7099991
-5.5100594	6.9295998	105.8099976
-5.5100594	6.9783998	104.4700012
-5.5100594	7.0271997	104.7500000
-5.5100594	7.0759997	102.3199997
-5.5100594	7.1247997	104.2200012
-5.5100594	7.1735997	102.7200012
-5.5100594	7.2223997	101.6199951
-5.5100594	7.2711997	102.7900009
-5.5100594	7.3199997	102.2999954
-5.5100594	7.3687997	102.1199951
-5.5100594	7.4175997	104.2699966
-5.5100594	7.4663997	103.6500015
-5.5100594	7.5151997	100.7299957
-5.5100594	7.5639997	102.2099991
-5.5100594	7.6127996	104.6500015
-5.5100594	7.6615996	103.9799957
-5.5100594	7.7103996	101.1899948
-5.5100594	7.7591996	106.2500000
-5.5100594	7.8079996	105.6899948
-5.5100594	7.8567996	105.6399994
-5.5100594	7.9056001	106.7399979
-5.5100594	7.9544001	106.7799988
-5.5100594	8.0032005	102.9399948
-5.5100594	8.0520000	98.5100021
-5.5100594	8.1008005	102.7200012
-5.5100594	8.1496000	105.2299957
-5.5100594	8.1984005	106.3399963
-5.5100594	8.2472000	106.2299957
-5.5100594	8.2960005	105.5499954
-5.5100594	8.3448000	103.7999954
-5.5100594	8.3936005	104.2099991
-5.5100594	8.4424000	104.3300018
-5.5100594	8.4912004	100.6199951
-5.5100594	8.5400000	102.5199966
-5.5100594	8.5888004	104.2399979

-5.5100594	8.6375999	98.4599991
-5.5100594	8.6864004	100.3600006
-5.5100594	8.7351999	104.3799973
-5.5100594	8.7840004	106.1500015
-5.5100594	8.8327999	106.1800003
-5.5100594	8.8816004	106.4899979
-5.5100594	8.9303999	107.1199951
-5.5100594	8.9792004	105.2900009
-5.5100594	9.0279999	102.5800018
-5.5100594	9.0768003	106.3499985
-5.5100594	9.1255999	106.2399979
-5.5100594	9.1744003	102.7099991
-5.5100594	9.2231998	102.2900009
-5.5100594	9.2720003	103.9700012
-5.5100594	9.3207998	103.3399963
-5.5100594	9.3696003	106.5000000
-5.5100594	9.4184008	104.4599991
-5.5100594	9.4672003	101.7299957
-5.5100594	9.5160007	100.9799957
-5.5100594	9.5648003	102.6299973
-5.5100594	9.6136007	103.2399979
-5.5100594	9.6624002	103.4300003
-5.5100594	9.7112007	102.1500015
-5.5100594	9.7600002	103.9099960
-5.5100594	9.8088007	103.7399979
-5.5100594	9.8576002	101.9499969
-5.5100594	9.9064007	103.2200012
-5.5100594	9.9552002	103.3199997
-5.5100594	10.0040007	103.7399979
-5.5100594	10.0528002	105.8899994
-5.5100594	10.1016006	104.6800003
-5.5100594	10.1504002	105.3399963
-5.5100594	10.1992006	103.4199982
-5.5100594	10.2480001	102.1399994
-5.5100594	10.2968006	103.4599991
-5.5100594	10.3456001	104.9499969
-5.5100594	10.3944006	103.8199997
-5.5100594	10.4432001	103.6299973
-5.5100594	10.4920006	103.1699982
-5.5100594	10.5408001	104.5499954
-5.5100594	10.5896006	105.4799957
-5.5100594	10.6384001	102.1199951
-5.5100594	10.6872005	99.7799988
-5.5100594	10.7360001	102.1899948
-5.5100594	10.7848005	104.8899994
-5.5100594	10.8336000	104.9700012
-5.5100594	10.8824005	104.8399963
-5.5100594	10.9312000	104.1199951
-5.5100594	10.9800005	101.2299957
-5.5100594	11.0288000	101.7900009
-5.5100594	11.0776005	104.3300018
-5.5100594	11.1264000	104.5000000
-5.5100594	11.1752005	101.3699951
-5.5100594	11.2240000	102.8799973
-5.5100594	11.2728004	102.6899948
-5.5100594	11.3216000	97.7699966
-5.5100594	11.3704004	101.4700012
-5.5100594	11.4191999	104.8499985

-5.5100594	11.4680004	106.0100021
-5.5100594	11.5167999	107.0199966
-5.5100594	11.5656004	105.5199966
-5.5100594	11.6143999	100.0000000
-5.5100594	11.6632004	102.7900009
-5.5100594	11.7119999	103.1500015
-5.5100594	11.7608004	103.8199997
-5.5100594	11.8095999	102.1299973
-5.5100594	11.8584003	103.6299973
-5.5100594	11.9071999	102.8099976
-5.5100594	11.9560003	103.4499969
-5.5100594	12.0047998	105.3799973
-5.5100594	12.0536003	106.5400009
-5.5100594	12.1023998	106.8399963
-5.5100594	12.1512003	102.2500000
-5.5100594	12.1999998	103.7600021
-5.5100594	12.2488003	103.8499985
-5.5100594	12.2975998	103.5400009
-5.5100594	12.3464003	103.6399994
-5.5100594	12.3951998	103.2500000
-5.5100594	12.4440002	105.5899963
-5.5100594	12.4927998	102.6599960
-5.5100594	12.5416002	103.0800018
-5.5100594	12.5904007	100.7099991
-5.5100594	12.6392002	101.8099976
-5.5100594	12.6880007	102.3399963
-5.5100594	12.7368002	101.6599960
-5.5100594	12.7856007	103.3399963
-5.5100594	12.8344002	103.7799988
-5.5100594	12.8832006	102.2699966
-5.5100594	12.9320002	103.5599976
-5.5100594	12.9808006	104.4099960
-5.5100594	13.0296001	102.7200012
-5.5100594	13.0784006	103.2500000
-5.5100594	13.1272001	103.1199951
-5.5100594	13.1760006	101.5800018
-5.5100594	13.2248001	101.6299973
-5.5100594	13.2736006	101.1699982
-5.5100594	13.3224001	100.8399963
-5.5100594	13.3712006	103.4700012
-5.5100594	13.4200001	103.1999969
-5.5100594	13.4688005	101.6100006
-5.5100594	13.5176001	103.1800003
-5.5100594	13.5664005	105.0499954
-5.5100594	13.6152000	104.4899979
-5.5100594	13.6640005	100.4199982
-5.5100594	13.7128000	103.9700012
-5.5100594	13.7616005	103.9399948
-5.5100594	13.8104000	104.6800003
-5.5100594	13.8592005	101.6100006
-5.5100594	13.9080000	99.8399963
-5.5100594	13.9568005	100.0699997
-5.5100594	14.0056000	103.6500015
-5.5100594	14.0544004	104.0999985
-5.5100594	14.1032000	104.0800018
-5.5100594	14.1520004	104.9700012
-5.5100594	14.2007999	104.6999969
-5.5100594	14.2496004	100.5699997

-5.5100594	14.2983999	101.9899979
-5.5100594	14.3472004	102.5899963
-5.5100594	14.3959999	100.6100006
-5.5100594	14.4448004	101.2600021
-5.5100594	14.4935999	102.7799988
-5.5100594	14.5424004	102.7299957
-5.5100594	14.5911999	104.2399979
-5.5100594	14.6400003	102.8699951
-5.5100594	14.6887999	102.6800003
-5.5100594	14.7376003	103.0599976
-5.5100594	14.7863998	101.9499969
-5.5100594	14.8352003	102.4099960
-5.5100594	14.8839998	104.4799957
-5.5100594	14.9328003	100.5100021
-5.5100594	14.9815998	103.9899979
-5.5100594	15.0304003	104.1899948
-5.5039234	0.3416000	106.0800018
-5.5039234	0.3904000	109.1500015
-5.5039234	0.4392000	108.2999954
-5.5039234	0.4880000	107.2699966
-5.5039234	0.5368000	104.3399963
-5.5039234	0.5856000	104.4499969
-5.5039234	0.6344000	106.9599991
-5.5039234	0.6832000	105.4700012
-5.5039234	0.7320000	101.8899994
-5.5039234	0.7808000	101.6199951
-5.5039234	0.8296000	106.0699997
-5.5039234	0.8784000	108.2299957
-5.5039234	0.9272000	105.2299957
-5.5039234	0.9760000	103.6599960
-5.5039234	1.0247999	101.9199982
-5.5039234	1.0735999	105.0400009
-5.5039234	1.1223999	107.8199997
-5.5039234	1.1711999	106.9300003
-5.5039234	1.2200000	101.4599991
-5.5039234	1.2688000	103.3099976
-5.5039234	1.3176000	103.6999969
-5.5039234	1.3664000	103.7999954
-5.5039234	1.4152000	103.5199966
-5.5039234	1.4640000	105.6299973
-5.5039234	1.5128000	108.4799957
-5.5039234	1.5616000	105.4099960
-5.5039234	1.6104000	106.1299973
-5.5039234	1.6592000	104.2299957
-5.5039234	1.7079999	106.2900009
-5.5039234	1.7567999	106.0400009
-5.5039234	1.8055999	105.9700012
-5.5039234	1.8543999	104.4199982
-5.5039234	1.9031999	102.4199982
-5.5039234	1.9519999	99.2299957
-5.5039234	2.0007999	101.1100006
-5.5039234	2.0495999	102.3499985
-5.5039234	2.0983999	101.6599960
-5.5039234	2.1471999	101.4899979
-5.5039234	2.1959999	105.1299973
-5.5039234	2.2447999	106.7500000
-5.5039234	2.2935998	102.0400009
-5.5039234	2.3423998	105.0800018

-5.5039234	2.3912001	106.9099960
-5.5039234	2.4400001	107.3799973
-5.5039234	2.4888000	103.2999954
-5.5039234	2.5376000	104.9899979
-5.5039234	2.5864000	104.7699966
-5.5039234	2.6352000	106.9000015
-5.5039234	2.6840000	106.1100006
-5.5039234	2.7328000	104.7799988
-5.5039234	2.7816000	103.6299973
-5.5039234	2.8304000	101.1399994
-5.5039234	2.8792000	104.6199951
-5.5039234	2.9280000	105.7399979
-5.5039234	2.9768000	105.6399994
-5.5039234	3.0256000	103.9399948
-5.5039234	3.0743999	103.9499969
-5.5039234	3.1231999	103.5100021
-5.5039234	3.1719999	104.5599976
-5.5039234	3.2207999	105.9700012
-5.5039234	3.2695999	105.8199997
-5.5039234	3.3183999	104.1800003
-5.5039234	3.3671999	101.1599960
-5.5039234	3.4159999	100.2900009
-5.5039234	3.4647999	101.5299988
-5.5039234	3.5135999	99.9199982
-5.5039234	3.5623999	103.4899979
-5.5039234	3.6111999	104.6800003
-5.5039234	3.6599998	103.5699997
-5.5039234	3.7087998	102.9300003
-5.5039234	3.7575998	104.4599991
-5.5039234	3.8063998	104.4499969
-5.5039234	3.8551998	104.1199951
-5.5039234	3.9039998	102.0299988
-5.5039234	3.9527998	102.7399979
-5.5039234	4.0016003	103.7999954
-5.5039234	4.0503998	100.9099960
-5.5039234	4.0991998	105.4499969
-5.5039234	4.1479998	103.4499969
-5.5039234	4.1967998	103.0299988
-5.5039234	4.2455997	103.9599991
-5.5039234	4.2943997	103.8099976
-5.5039234	4.3431997	105.7500000
-5.5039234	4.3919997	102.5199966
-5.5039234	4.4407997	101.6999969
-5.5039234	4.4895997	103.2699966
-5.5039234	4.5383997	104.6999969
-5.5039234	4.5871997	106.6500015
-5.5039234	4.6359997	109.8499985
-5.5039234	4.6847997	116.4700012
-5.5039234	4.7336001	115.6699982
-5.5039234	4.7824001	108.7299957
-5.5039234	4.8312001	106.7099991
-5.5039234	4.8800001	108.7500000
-5.5039234	4.9288001	106.8699951
-5.5039234	4.9776001	102.0199966
-5.5039234	5.0264001	105.4300003
-5.5039234	5.0752001	106.2099991
-5.5039234	5.1240001	105.4799957
-5.5039234	5.1728001	103.1299973

-5.5039234	5.2216001	104.3899994
-5.5039234	5.2704000	104.1299973
-5.5039234	5.3192000	102.0000000
-5.5039234	5.3680000	104.6699982
-5.5039234	5.4168000	106.0199966
-5.5039234	5.4656000	103.8799973
-5.5039234	5.5144000	104.0800018
-5.5039234	5.5632000	105.1500015
-5.5039234	5.6120000	103.2500000
-5.5039234	5.6608000	102.5800018
-5.5039234	5.7096000	102.9399948
-5.5039234	5.7584000	103.2999954
-5.5039234	5.8072000	101.0499954
-5.5039234	5.8559999	103.1500015
-5.5039234	5.9047999	104.1500015
-5.5039234	5.9535999	104.6299973
-5.5039234	6.0023999	104.0699997
-5.5039234	6.0511999	101.4399948
-5.5039234	6.0999999	105.1899948
-5.5039234	6.1487999	107.0299988
-5.5039234	6.1975999	108.4599991
-5.5039234	6.2463999	107.5000000
-5.5039234	6.2951999	106.8799973
-5.5039234	6.3439999	105.6899948
-5.5039234	6.3927999	105.4000015
-5.5039234	6.4415998	105.0599976
-5.5039234	6.4903998	106.6399994
-5.5039234	6.5391998	105.8600006
-5.5039234	6.5879998	104.3300018
-5.5039234	6.6367998	103.4499969
-5.5039234	6.6855998	101.0599976
-5.5039234	6.7343998	100.9099960
-5.5039234	6.7831998	102.8600006
-5.5039234	6.8319998	102.5000000
-5.5039234	6.8807998	103.2900009
-5.5039234	6.9295998	104.6599960
-5.5039234	6.9783998	103.7699966
-5.5039234	7.0271997	105.2699966
-5.5039234	7.0759997	102.7900009
-5.5039234	7.1247997	104.3799973
-5.5039234	7.1735997	101.8600006
-5.5039234	7.2223997	100.5400009
-5.5039234	7.2711997	102.6699982
-5.5039234	7.3199997	102.0299988
-5.5039234	7.3687997	102.4300003
-5.5039234	7.4175997	104.3499985
-5.5039234	7.4663997	104.6299973
-5.5039234	7.5151997	102.2500000
-5.5039234	7.5639997	102.7099991
-5.5039234	7.6127996	103.9499969
-5.5039234	7.6615996	103.4399948
-5.5039234	7.7103996	102.3199997
-5.5039234	7.7591996	105.7999954
-5.5039234	7.8079996	105.5299988
-5.5039234	7.8567996	105.6699982
-5.5039234	7.9056001	105.6599960
-5.5039234	7.9544001	105.2799988
-5.5039234	8.0032005	101.0400009

-5.5039234	8.0520000	97.8899994
-5.5039234	8.1008005	102.3099976
-5.5039234	8.1496000	104.5800018
-5.5039234	8.1984005	106.0699997
-5.5039234	8.2472000	106.4300003
-5.5039234	8.2960005	105.9899979
-5.5039234	8.3448000	103.6500015
-5.5039234	8.3936005	104.5599976
-5.5039234	8.4424000	104.7099991
-5.5039234	8.4912004	101.1100006
-5.5039234	8.5400000	103.3899994
-5.5039234	8.5888004	104.0899963
-5.5039234	8.6375999	97.2200012
-5.5039234	8.6864004	100.2200012
-5.5039234	8.7351999	103.5999985
-5.5039234	8.7840004	105.1599960
-5.5039234	8.8327999	105.4700012
-5.5039234	8.8816004	105.6100006
-5.5039234	8.9303999	106.1899948
-5.5039234	8.9792004	103.7799988
-5.5039234	9.0279999	101.4199982
-5.5039234	9.0768003	105.5599976
-5.5039234	9.1255999	106.0800018
-5.5039234	9.1744003	103.1299973
-5.5039234	9.2231998	102.8099976
-5.5039234	9.2720003	104.3399963
-5.5039234	9.3207998	104.0699997
-5.5039234	9.3696003	106.7399979
-5.5039234	9.4184008	104.4799957
-5.5039234	9.4672003	101.4700012
-5.5039234	9.5160007	99.4199982
-5.5039234	9.5648003	101.5599976
-5.5039234	9.6136007	102.9599991
-5.5039234	9.6624002	104.2099991
-5.5039234	9.7112007	102.4399948
-5.5039234	9.7600002	104.1500015
-5.5039234	9.8088007	104.5499954
-5.5039234	9.8576002	101.5199966
-5.5039234	9.9064007	102.6299973
-5.5039234	9.9552002	102.9799957
-5.5039234	10.0040007	103.6599960
-5.5039234	10.0528002	105.1899948
-5.5039234	10.1016006	103.7200012
-5.5039234	10.1504002	104.7299957
-5.5039234	10.1992006	103.2399979
-5.5039234	10.2480001	101.1100006
-5.5039234	10.2968006	103.2699966
-5.5039234	10.3456001	105.0299988
-5.5039234	10.3944006	103.7399979
-5.5039234	10.4432001	103.5100021
-5.5039234	10.4920006	103.8399963
-5.5039234	10.5408001	104.5199966
-5.5039234	10.5896006	104.4000015
-5.5039234	10.6384001	101.6599960
-5.5039234	10.6872005	100.0699997
-5.5039234	10.7360001	103.4700012
-5.5039234	10.7848005	104.2200012
-5.5039234	10.8336000	104.6800003

-5.5039234	10.8824005	105.0899963
-5.5039234	10.9312000	104.0100021
-5.5039234	10.9800005	100.4599991
-5.5039234	11.0288000	101.9199982
-5.5039234	11.0776005	104.5999985
-5.5039234	11.1264000	104.1800003
-5.5039234	11.1752005	101.3300018
-5.5039234	11.2240000	101.7699966
-5.5039234	11.2728004	101.5999985
-5.5039234	11.3216000	98.0599976
-5.5039234	11.3704004	102.0599976
-5.5039234	11.4191999	105.0499954
-5.5039234	11.4680004	106.1699982
-5.5039234	11.5167999	106.2799988
-5.5039234	11.5656004	104.8499985
-5.5039234	11.6143999	100.2600021
-5.5039234	11.6632004	101.9000015
-5.5039234	11.7119999	102.0800018
-5.5039234	11.7608004	102.7999954
-5.5039234	11.8095999	101.1100006
-5.5039234	11.8584003	103.6100006
-5.5039234	11.9071999	102.9099960
-5.5039234	11.9560003	103.1599960
-5.5039234	12.0047998	104.5699997
-5.5039234	12.0536003	105.5499954
-5.5039234	12.1023998	106.2900009
-5.5039234	12.1512003	101.3099976
-5.5039234	12.1999998	102.5400009
-5.5039234	12.2488003	103.2999954
-5.5039234	12.2975998	103.0499954
-5.5039234	12.3464003	103.2200012
-5.5039234	12.3951998	102.1500015
-5.5039234	12.4440002	104.5299988
-5.5039234	12.4927998	102.8699951
-5.5039234	12.5416002	102.6599960
-5.5039234	12.5904007	100.6800003
-5.5039234	12.6392002	101.1599960
-5.5039234	12.6880007	102.5000000
-5.5039234	12.7368002	102.1199951
-5.5039234	12.7856007	103.7900009
-5.5039234	12.8344002	103.7799988
-5.5039234	12.8832006	103.4199982
-5.5039234	12.9320002	103.3899994
-5.5039234	12.9808006	103.2900009
-5.5039234	13.0296001	102.9599991
-5.5039234	13.0784006	103.3699951
-5.5039234	13.1272001	103.1899948
-5.5039234	13.1760006	101.1299973
-5.5039234	13.2248001	101.9700012
-5.5039234	13.2736006	100.8399963
-5.5039234	13.3224001	99.9300003
-5.5039234	13.3712006	103.3199997
-5.5039234	13.4200001	102.3399963
-5.5039234	13.4688005	100.5599976
-5.5039234	13.5176001	102.3899994
-5.5039234	13.5664005	104.5199966
-5.5039234	13.6152000	104.1800003
-5.5039234	13.6640005	100.9000015

-5.5039234	13.7128000	104.6299973
-5.5039234	13.7616005	104.6199951
-5.5039234	13.8104000	104.0599976
-5.5039234	13.8592005	101.0499954
-5.5039234	13.9080000	100.4799957
-5.5039234	13.9568005	99.9499969
-5.5039234	14.0056000	103.4000015
-5.5039234	14.0544004	104.7900009
-5.5039234	14.1032000	104.1899948
-5.5039234	14.1520004	104.9399948
-5.5039234	14.2007999	105.1800003
-5.5039234	14.2496004	101.1800003
-5.5039234	14.2983999	101.3099976
-5.5039234	14.3472004	101.5599976
-5.5039234	14.3959999	99.5899963
-5.5039234	14.4448004	100.7299957
-5.5039234	14.4935999	102.0899963
-5.5039234	14.5424004	102.5800018
-5.5039234	14.5911999	103.9599991
-5.5039234	14.6400003	102.3799973
-5.5039234	14.6887999	102.9300003
-5.5039234	14.7376003	104.7099991
-5.5039234	14.7863998	102.7200012
-5.5039234	14.8352003	101.8699951
-5.5039234	14.8839998	104.5999985
-5.5039234	14.9328003	102.0199966
-5.5039234	14.9815998	103.3099976
-5.5039234	15.0304003	103.4300003
-5.4977875	0.3416000	104.4700012
-5.4977875	0.3904000	108.4399948
-5.4977875	0.4392000	107.0000000
-5.4977875	0.4880000	106.7699966
-5.4977875	0.5368000	104.6100006
-5.4977875	0.5856000	104.8300018
-5.4977875	0.6344000	107.0199966
-5.4977875	0.6832000	105.0299988
-5.4977875	0.7320000	102.0899963
-5.4977875	0.7808000	101.5100021
-5.4977875	0.8296000	106.3899994
-5.4977875	0.8784000	107.7399979
-5.4977875	0.9272000	104.5699997
-5.4977875	0.9760000	105.0899963
-5.4977875	1.0247999	102.4499969
-5.4977875	1.0735999	105.2900009
-5.4977875	1.1223999	108.0999985
-5.4977875	1.1711999	107.4899979
-5.4977875	1.2200000	102.8600006
-5.4977875	1.2688000	103.5800018
-5.4977875	1.3176000	104.2999954
-5.4977875	1.3664000	104.6899948
-5.4977875	1.4152000	103.5699997
-5.4977875	1.4640000	104.3399963
-5.4977875	1.5128000	108.5000000
-5.4977875	1.5616000	106.5299988
-5.4977875	1.6104000	106.7699966
-5.4977875	1.6592000	103.3600006
-5.4977875	1.7079999	106.6699982
-5.4977875	1.7567999	106.4700012

-5.4977875	1.8055999	105.5299988
-5.4977875	1.8543999	104.3300018
-5.4977875	1.9031999	102.1999969
-5.4977875	1.9519999	99.8799973
-5.4977875	2.0007999	101.5999985
-5.4977875	2.0495999	101.5800018
-5.4977875	2.0983999	101.1399994
-5.4977875	2.1471999	101.4199982
-5.4977875	2.1959999	104.7099991
-5.4977875	2.2447999	106.4899979
-5.4977875	2.2935998	101.6299973
-5.4977875	2.3423998	104.9899979
-5.4977875	2.3912001	107.3499985
-5.4977875	2.4400001	107.4899979
-5.4977875	2.4888000	104.0800018
-5.4977875	2.5376000	104.5100021
-5.4977875	2.5864000	104.7500000
-5.4977875	2.6352000	107.1500015
-5.4977875	2.6840000	106.6199951
-5.4977875	2.7328000	104.4799957
-5.4977875	2.7816000	104.0800018
-5.4977875	2.8304000	100.8099976
-5.4977875	2.8792000	103.3499985
-5.4977875	2.9280000	103.4099960
-5.4977875	2.9768000	104.3300018
-5.4977875	3.0256000	104.1100006
-5.4977875	3.0743999	105.1399994
-5.4977875	3.1231999	104.5899963
-5.4977875	3.1719999	104.5800018
-5.4977875	3.2207999	106.3899994
-5.4977875	3.2695999	106.3699951
-5.4977875	3.3183999	104.6599960
-5.4977875	3.3671999	101.4499969
-5.4977875	3.4159999	102.0599976
-5.4977875	3.4647999	102.4399948
-5.4977875	3.5135999	100.5800018
-5.4977875	3.5623999	103.6899948
-5.4977875	3.6111999	104.6299973
-5.4977875	3.6599998	102.0899963
-5.4977875	3.7087998	101.8199997
-5.4977875	3.7575998	104.3099976
-5.4977875	3.8063998	104.6999969
-5.4977875	3.8551998	104.8799973
-5.4977875	3.9039998	102.3699951
-5.4977875	3.9527998	102.7099991
-5.4977875	4.0016003	104.7399979
-5.4977875	4.0503998	99.6299973
-5.4977875	4.0991998	105.5199966
-5.4977875	4.1479998	103.7399979
-5.4977875	4.1967998	103.6899948
-5.4977875	4.2455997	104.0699997
-5.4977875	4.2943997	103.3099976
-5.4977875	4.3431997	104.7600021
-5.4977875	4.3919997	101.5599976
-5.4977875	4.4407997	101.6199951
-5.4977875	4.4895997	103.3399963
-5.4977875	4.5383997	105.3199997
-5.4977875	4.5871997	107.1100006

-5.4977875	4.6359997	108.7399979
-5.4977875	4.6847997	116.3300018
-5.4977875	4.7336001	115.9300003
-5.4977875	4.7824001	108.9700012
-5.4977875	4.8312001	106.0800018
-5.4977875	4.8800001	108.1500015
-5.4977875	4.9288001	106.5400009
-5.4977875	4.9776001	102.0599976
-5.4977875	5.0264001	104.3399963
-5.4977875	5.0752001	105.3799973
-5.4977875	5.1240001	105.2200012
-5.4977875	5.1728001	103.2399979
-5.4977875	5.2216001	104.6399994
-5.4977875	5.2704000	105.4899979
-5.4977875	5.3192000	103.7600021
-5.4977875	5.3680000	105.2399979
-5.4977875	5.4168000	105.4199982
-5.4977875	5.4656000	103.1399994
-5.4977875	5.5144000	104.1800003
-5.4977875	5.5632000	104.8699951
-5.4977875	5.6120000	102.2999954
-5.4977875	5.6608000	101.8899994
-5.4977875	5.7096000	102.1899948
-5.4977875	5.7584000	103.7600021
-5.4977875	5.8072000	101.9499969
-5.4977875	5.8559999	104.1699982
-5.4977875	5.9047999	104.1199951
-5.4977875	5.9535999	103.5499954
-5.4977875	6.0023999	102.9700012
-5.4977875	6.0511999	100.2699966
-5.4977875	6.0999999	104.6899948
-5.4977875	6.1487999	106.2200012
-5.4977875	6.1975999	108.2099991
-5.4977875	6.2463999	107.3199997
-5.4977875	6.2951999	106.8399963
-5.4977875	6.3439999	106.0699997
-5.4977875	6.3927999	104.1100006
-5.4977875	6.4415998	104.0800018
-5.4977875	6.4903998	105.6100006
-5.4977875	6.5391998	106.1999969
-5.4977875	6.5879998	105.5100021
-5.4977875	6.6367998	103.5499954
-5.4977875	6.6855998	100.6399994
-5.4977875	6.7343998	102.1299973
-5.4977875	6.7831998	103.9300003
-5.4977875	6.8319998	103.5599976
-5.4977875	6.8807998	103.0199966
-5.4977875	6.9295998	103.7099991
-5.4977875	6.9783998	103.2999954
-5.4977875	7.0271997	105.8199997
-5.4977875	7.0759997	103.6899948
-5.4977875	7.1247997	104.1100006
-5.4977875	7.1735997	101.1100006
-5.4977875	7.2223997	99.7999954
-5.4977875	7.2711997	102.2399979
-5.4977875	7.3199997	102.1199951
-5.4977875	7.3687997	102.3799973
-5.4977875	7.4175997	104.1899948

-5.4977875	7.4663997	105.0800018
-5.4977875	7.5151997	103.4300003
-5.4977875	7.5639997	102.1100006
-5.4977875	7.6127996	102.4599991
-5.4977875	7.6615996	102.9399948
-5.4977875	7.7103996	103.6599960
-5.4977875	7.7591996	105.2600021
-5.4977875	7.8079996	105.1599960
-5.4977875	7.8567996	105.7600021
-5.4977875	7.9056001	105.0400009
-5.4977875	7.9544001	104.1800003
-5.4977875	8.0032005	99.5499954
-5.4977875	8.0520000	98.0899963
-5.4977875	8.1008005	101.7900009
-5.4977875	8.1496000	103.5199966
-5.4977875	8.1984005	105.5000000
-5.4977875	8.2472000	106.3199997
-5.4977875	8.2960005	106.0499954
-5.4977875	8.3448000	103.4399948
-5.4977875	8.3936005	105.2399979
-5.4977875	8.4424000	105.2699966
-5.4977875	8.4912004	102.5999985
-5.4977875	8.5400000	104.6299973
-5.4977875	8.5888004	104.5299988
-5.4977875	8.6375999	97.5499954
-5.4977875	8.6864004	100.3699951
-5.4977875	8.7351999	102.5000000
-5.4977875	8.7840004	103.6699982
-5.4977875	8.8327999	104.4300003
-5.4977875	8.8816004	104.4799957
-5.4977875	8.9303999	104.4499969
-5.4977875	8.9792004	102.2900009
-5.4977875	9.0279999	100.6999969
-5.4977875	9.0768003	104.8699951
-5.4977875	9.1255999	105.7299957
-5.4977875	9.1744003	103.1100006
-5.4977875	9.2231998	102.6399994
-5.4977875	9.2720003	104.9300003
-5.4977875	9.3207998	105.2600021
-5.4977875	9.3696003	106.7500000
-5.4977875	9.4184008	103.7699966
-5.4977875	9.4672003	101.0599976
-5.4977875	9.5160007	98.6800003
-5.4977875	9.5648003	101.1100006
-5.4977875	9.6136007	103.1500015
-5.4977875	9.6624002	104.1800003
-5.4977875	9.7112007	102.2999954
-5.4977875	9.7600002	103.7200012
-5.4977875	9.8088007	104.3600006
-5.4977875	9.8576002	100.9199982
-5.4977875	9.9064007	102.6999969
-5.4977875	9.9552002	103.1899948
-5.4977875	10.0040007	103.8600006
-5.4977875	10.0528002	104.3099976
-5.4977875	10.1016006	102.6100006
-5.4977875	10.1504002	104.4399948
-5.4977875	10.1992006	103.4399948
-5.4977875	10.2480001	100.8499985

-5.4977875	10.2968006	103.6500015
-5.4977875	10.3456001	105.2699966
-5.4977875	10.3944006	103.7699966
-5.4977875	10.4432001	103.0999985
-5.4977875	10.4920006	104.0699997
-5.4977875	10.5408001	104.4000015
-5.4977875	10.5896006	103.1999969
-5.4977875	10.6384001	101.6500015
-5.4977875	10.6872005	100.2699966
-5.4977875	10.7360001	104.6899948
-5.4977875	10.7848005	104.0299988
-5.4977875	10.8336000	104.0100021
-5.4977875	10.8824005	104.5000000
-5.4977875	10.9312000	103.9799957
-5.4977875	10.9800005	100.0299988
-5.4977875	11.0288000	102.5800018
-5.4977875	11.0776005	105.1299973
-5.4977875	11.1264000	104.1299973
-5.4977875	11.1752005	101.5299988
-5.4977875	11.2240000	101.0699997
-5.4977875	11.2728004	100.5699997
-5.4977875	11.3216000	97.9300003
-5.4977875	11.3704004	101.7399979
-5.4977875	11.4191999	104.3899994
-5.4977875	11.4680004	105.7699966
-5.4977875	11.5167999	105.1699982
-5.4977875	11.5656004	103.9499969
-5.4977875	11.6143999	100.7099991
-5.4977875	11.6632004	100.9499969
-5.4977875	11.7119999	100.9899979
-5.4977875	11.7608004	102.0199966
-5.4977875	11.8095999	101.0800018
-5.4977875	11.8584003	104.4300003
-5.4977875	11.9071999	103.8199997
-5.4977875	11.9560003	103.3300018
-5.4977875	12.0047998	103.2999954
-5.4977875	12.0536003	104.0199966
-5.4977875	12.1023998	105.6399994
-5.4977875	12.1512003	101.3300018
-5.4977875	12.1999998	100.8799973
-5.4977875	12.2488003	102.4300003
-5.4977875	12.2975998	102.6199951
-5.4977875	12.3464003	102.7399979
-5.4977875	12.3951998	102.5000000
-5.4977875	12.4440002	103.7200012
-5.4977875	12.4927998	101.1800003
-5.4977875	12.5416002	102.0800018
-5.4977875	12.5904007	100.9700012
-5.4977875	12.6392002	101.0299988
-5.4977875	12.6880007	102.9099960
-5.4977875	12.7368002	102.8199997
-5.4977875	12.7856007	103.8799973
-5.4977875	12.8344002	103.5100021
-5.4977875	12.8832006	104.6199951
-5.4977875	12.9320002	103.9399948
-5.4977875	12.9808006	102.5599976
-5.4977875	13.0296001	102.4499969
-5.4977875	13.0784006	103.5299988

-5.4977875	13.1272001	103.9499969
-5.4977875	13.1760006	102.4300003
-5.4977875	13.2248001	102.8499985
-5.4977875	13.2736006	100.8699951
-5.4977875	13.3224001	98.9300003
-5.4977875	13.3712006	102.6800003
-5.4977875	13.4200001	101.0199966
-5.4977875	13.4688005	99.6800003
-5.4977875	13.5176001	101.7500000
-5.4977875	13.5664005	103.8199997
-5.4977875	13.6152000	103.7200012
-5.4977875	13.6640005	101.1199951
-5.4977875	13.7128000	104.6899948
-5.4977875	13.7616005	105.1299973
-5.4977875	13.8104000	103.5499954
-5.4977875	13.8592005	100.5800018
-5.4977875	13.9080000	101.0899963
-5.4977875	13.9568005	99.8899994
-5.4977875	14.0056000	102.6999969
-5.4977875	14.0544004	104.9000015
-5.4977875	14.1032000	104.1399994
-5.4977875	14.1520004	104.4199982
-5.4977875	14.2007999	105.0299988
-5.4977875	14.2496004	101.0499954
-5.4977875	14.2983999	100.2399979
-5.4977875	14.3472004	100.3699951
-5.4977875	14.3959999	98.9899979
-5.4977875	14.4448004	100.5499954
-5.4977875	14.4935999	101.2500000
-5.4977875	14.5424004	101.0999985
-5.4977875	14.5911999	103.5100021
-5.4977875	14.6400003	101.7099991
-5.4977875	14.6887999	102.7500000
-5.4977875	14.7376003	105.6399994
-5.4977875	14.7863998	103.7500000
-5.4977875	14.8352003	102.0400009
-5.4977875	14.8839998	103.6299973
-5.4977875	14.9328003	103.1699982
-5.4977875	14.9815998	102.8799973
-5.4977875	15.0304003	102.7900009
-5.4916515	0.3416000	102.6399994
-5.4916515	0.3904000	107.6199951
-5.4916515	0.4392000	106.0299988
-5.4916515	0.4880000	106.1999969
-5.4916515	0.5368000	104.5699997
-5.4916515	0.5856000	104.8600006
-5.4916515	0.6344000	107.0000000
-5.4916515	0.6832000	105.1199951
-5.4916515	0.7320000	103.1500015
-5.4916515	0.7808000	102.4399948
-5.4916515	0.8296000	106.6100006
-5.4916515	0.8784000	107.3499985
-5.4916515	0.9272000	104.9099960
-5.4916515	0.9760000	107.4899979
-5.4916515	1.0247999	104.7999954
-5.4916515	1.0735999	105.7900009
-5.4916515	1.1223999	107.7799988
-5.4916515	1.1711999	106.7500000

-5.4916515	1.2200000	103.6299973
-5.4916515	1.2688000	103.6500015
-5.4916515	1.3176000	104.5000000
-5.4916515	1.3664000	105.1100006
-5.4916515	1.4152000	104.1299973
-5.4916515	1.4640000	103.7099991
-5.4916515	1.5128000	108.8799973
-5.4916515	1.5616000	108.0000000
-5.4916515	1.6104000	106.9599991
-5.4916515	1.6592000	101.6699982
-5.4916515	1.7079999	106.5800018
-5.4916515	1.7567999	106.6899948
-5.4916515	1.8055999	105.3499985
-5.4916515	1.8543999	104.4899979
-5.4916515	1.9031999	103.1500015
-5.4916515	1.9519999	101.4700012
-5.4916515	2.0007999	103.1899948
-5.4916515	2.0495999	102.2699966
-5.4916515	2.0983999	102.0199966
-5.4916515	2.1471999	101.9599991
-5.4916515	2.1959999	104.4300003
-5.4916515	2.2447999	106.3099976
-5.4916515	2.2935998	101.0899963
-5.4916515	2.3423998	104.2600021
-5.4916515	2.3912001	107.3699951
-5.4916515	2.4400001	107.1399994
-5.4916515	2.4888000	104.8499985
-5.4916515	2.5376000	103.7799988
-5.4916515	2.5864000	104.4499969
-5.4916515	2.6352000	106.5999985
-5.4916515	2.6840000	106.6899948
-5.4916515	2.7328000	103.6100006
-5.4916515	2.7816000	104.9099960
-5.4916515	2.8304000	101.6899948
-5.4916515	2.8792000	102.7600021
-5.4916515	2.9280000	101.7299957
-5.4916515	2.9768000	103.8699951
-5.4916515	3.0256000	104.9399948
-5.4916515	3.0743999	106.5000000
-5.4916515	3.1231999	105.7500000
-5.4916515	3.1719999	104.8899994
-5.4916515	3.2207999	106.7799988
-5.4916515	3.2695999	106.5800018
-5.4916515	3.3183999	104.4199982
-5.4916515	3.3671999	100.7699966
-5.4916515	3.4159999	103.3899994
-5.4916515	3.4647999	103.4700012
-5.4916515	3.5135999	101.4300003
-5.4916515	3.5623999	103.5699997
-5.4916515	3.6111999	104.5699997
-5.4916515	3.6599998	100.9799957
-5.4916515	3.7087998	100.6699982
-5.4916515	3.7575998	103.9300003
-5.4916515	3.8063998	104.6699982
-5.4916515	3.8551998	105.5299988
-5.4916515	3.9039998	102.9199982
-5.4916515	3.9527998	102.2600021
-5.4916515	4.0016003	104.3199997

-5.4916515	4.0503998	97.9599991
-5.4916515	4.0991998	105.0000000
-5.4916515	4.1479998	103.9000015
-5.4916515	4.1967998	104.7799988
-5.4916515	4.2455997	105.1199951
-5.4916515	4.2943997	104.0499954
-5.4916515	4.3431997	104.6899948
-5.4916515	4.3919997	102.2399979
-5.4916515	4.4407997	101.0100021
-5.4916515	4.4895997	102.4899979
-5.4916515	4.5383997	105.8600006
-5.4916515	4.5871997	107.2699966
-5.4916515	4.6359997	107.2600021
-5.4916515	4.6847997	116.0000000
-5.4916515	4.7336001	116.1999969
-5.4916515	4.7824001	109.4899979
-5.4916515	4.8312001	105.2299957
-5.4916515	4.8800001	107.0699997
-5.4916515	4.9288001	106.0400009
-5.4916515	4.9776001	102.8699951
-5.4916515	5.0264001	103.7999954
-5.4916515	5.0752001	104.5800018
-5.4916515	5.1240001	105.0800018
-5.4916515	5.1728001	104.6500015
-5.4916515	5.2216001	106.0699997
-5.4916515	5.2704000	106.9799957
-5.4916515	5.3192000	104.7699966
-5.4916515	5.3680000	105.1899948
-5.4916515	5.4168000	104.9399948
-5.4916515	5.4656000	103.4499969
-5.4916515	5.5144000	105.1399994
-5.4916515	5.5632000	104.7900009
-5.4916515	5.6120000	101.3799973
-5.4916515	5.6608000	101.7200012
-5.4916515	5.7096000	101.2099991
-5.4916515	5.7584000	104.4899979
-5.4916515	5.8072000	103.3099976
-5.4916515	5.8559999	104.9199982
-5.4916515	5.9047999	103.6699982
-5.4916515	5.9535999	102.3399963
-5.4916515	6.0023999	102.1199951
-5.4916515	6.0511999	99.9000015
-5.4916515	6.0999999	104.6299973
-5.4916515	6.1487999	104.8399963
-5.4916515	6.1975999	107.4599991
-5.4916515	6.2463999	107.5499954
-5.4916515	6.2951999	106.9099960
-5.4916515	6.3439999	105.8799973
-5.4916515	6.3927999	102.3399963
-5.4916515	6.4415998	102.9199982
-5.4916515	6.4903998	104.2500000
-5.4916515	6.5391998	106.4099960
-5.4916515	6.5879998	106.5199966
-5.4916515	6.6367998	103.8300018
-5.4916515	6.6855998	100.9599991
-5.4916515	6.7343998	103.4300003
-5.4916515	6.7831998	104.7799988
-5.4916515	6.8319998	103.9700012

-5. 4916515	6. 8807998	102. 5100021
-5. 4916515	6. 9295998	102. 5000000
-5. 4916515	6. 9783998	102. 4499969
-5. 4916515	7. 0271997	106. 1199951
-5. 4916515	7. 0759997	103. 9700012
-5. 4916515	7. 1247997	103. 5999985
-5. 4916515	7. 1735997	100. 9399948
-5. 4916515	7. 2223997	100. 5599976
-5. 4916515	7. 2711997	102. 5000000
-5. 4916515	7. 3199997	102. 1800003
-5. 4916515	7. 3687997	103. 1599960
-5. 4916515	7. 4175997	104. 0100021
-5. 4916515	7. 4663997	105. 3099976
-5. 4916515	7. 5151997	104. 5199966
-5. 4916515	7. 5639997	101. 5899963
-5. 4916515	7. 6127996	101. 1399994
-5. 4916515	7. 6615996	103. 2299957
-5. 4916515	7. 7103996	104. 5499954
-5. 4916515	7. 7591996	104. 8199997
-5. 4916515	7. 8079996	104. 6699982
-5. 4916515	7. 8567996	105. 6500015
-5. 4916515	7. 9056001	104. 2500000
-5. 4916515	7. 9544001	103. 1899948
-5. 4916515	8. 0032005	99. 1299973
-5. 4916515	8. 0520000	97. 9700012
-5. 4916515	8. 1008005	101. 0199966
-5. 4916515	8. 1496000	103. 3799973
-5. 4916515	8. 1984005	105. 3300018
-5. 4916515	8. 2472000	105. 5599976
-5. 4916515	8. 2960005	106. 2200012
-5. 4916515	8. 3448000	103. 4300003
-5. 4916515	8. 3936005	105. 3399963
-5. 4916515	8. 4424000	105. 5800018
-5. 4916515	8. 4912004	103. 7900009
-5. 4916515	8. 5400000	105. 7699966
-5. 4916515	8. 5888004	104. 7999954
-5. 4916515	8. 6375999	97. 5400009
-5. 4916515	8. 6864004	100. 6899948
-5. 4916515	8. 7351999	102. 1399994
-5. 4916515	8. 7840004	102. 9799957
-5. 4916515	8. 8327999	103. 4399948
-5. 4916515	8. 8816004	103. 3300018
-5. 4916515	8. 9303999	102. 7600021
-5. 4916515	8. 9792004	101. 0000000
-5. 4916515	9. 0279999	100. 4499969
-5. 4916515	9. 0768003	104. 4399948
-5. 4916515	9. 1255999	105. 2200012
-5. 4916515	9. 1744003	103. 1599960
-5. 4916515	9. 2231998	102. 4199982
-5. 4916515	9. 2720003	105. 5599976
-5. 4916515	9. 3207998	106. 6699982
-5. 4916515	9. 3696003	106. 7600021
-5. 4916515	9. 4184008	103. 0400009
-5. 4916515	9. 4672003	101. 2299957
-5. 4916515	9. 5160007	98. 4399948
-5. 4916515	9. 5648003	100. 8699951
-5. 4916515	9. 6136007	103. 1500015
-5. 4916515	9. 6624002	103. 9700012

-5.4916515	9.7112007	101.0000000
-5.4916515	9.7600002	103.0000000
-5.4916515	9.8088007	103.8799973
-5.4916515	9.8576002	101.4799957
-5.4916515	9.9064007	103.1899948
-5.4916515	9.9552002	103.5499954
-5.4916515	10.0040007	104.2799988
-5.4916515	10.0528002	104.4499969
-5.4916515	10.1016006	102.8300018
-5.4916515	10.1504002	104.4399948
-5.4916515	10.1992006	103.4199982
-5.4916515	10.2480001	100.6500015
-5.4916515	10.2968006	104.1699982
-5.4916515	10.3456001	105.3600006
-5.4916515	10.3944006	103.1399994
-5.4916515	10.4432001	102.9499969
-5.4916515	10.4920006	104.2900009
-5.4916515	10.5408001	104.0299988
-5.4916515	10.5896006	101.7600021
-5.4916515	10.6384001	101.7200012
-5.4916515	10.6872005	100.5400009
-5.4916515	10.7360001	105.5899963
-5.4916515	10.7848005	103.6800003
-5.4916515	10.8336000	103.8199997
-5.4916515	10.8824005	104.3399963
-5.4916515	10.9312000	104.2600021
-5.4916515	10.9800005	100.9700012
-5.4916515	11.0288000	102.7200012
-5.4916515	11.0776005	105.2799988
-5.4916515	11.1264000	103.6399994
-5.4916515	11.1752005	101.4700012
-5.4916515	11.2240000	101.1800003
-5.4916515	11.2728004	100.4700012
-5.4916515	11.3216000	99.0000000
-5.4916515	11.3704004	101.6599960
-5.4916515	11.4191999	103.7799988
-5.4916515	11.4680004	104.6899948
-5.4916515	11.5167999	103.5699997
-5.4916515	11.5656004	103.0000000
-5.4916515	11.6143999	101.6699982
-5.4916515	11.6632004	101.1199951
-5.4916515	11.7119999	100.2399979
-5.4916515	11.7608004	101.3199997
-5.4916515	11.8095999	101.4899979
-5.4916515	11.8584003	105.2600021
-5.4916515	11.9071999	104.8399963
-5.4916515	11.9560003	103.6800003
-5.4916515	12.0047998	102.3799973
-5.4916515	12.0536003	102.6899948
-5.4916515	12.1023998	105.2099991
-5.4916515	12.1512003	101.6100006
-5.4916515	12.1999998	99.9199982
-5.4916515	12.2488003	101.5699997
-5.4916515	12.2975998	101.9199982
-5.4916515	12.3464003	101.7999954
-5.4916515	12.3951998	103.0400009
-5.4916515	12.4440002	103.7200012
-5.4916515	12.4927998	100.6999969

-5.4916515	12.5416002	102.4300003
-5.4916515	12.5904007	100.9300003
-5.4916515	12.6392002	100.5699997
-5.4916515	12.6880007	103.1100006
-5.4916515	12.7368002	103.4199982
-5.4916515	12.7856007	104.2999954
-5.4916515	12.8344002	103.1899948
-5.4916515	12.8832006	104.8300018
-5.4916515	12.9320002	104.1999969
-5.4916515	12.9808006	101.7299957
-5.4916515	13.0296001	101.8699951
-5.4916515	13.0784006	103.8099976
-5.4916515	13.1272001	104.5100021
-5.4916515	13.1760006	103.1899948
-5.4916515	13.2248001	103.5400009
-5.4916515	13.2736006	101.3300018
-5.4916515	13.3224001	98.8799973
-5.4916515	13.3712006	102.3899994
-5.4916515	13.4200001	99.7500000
-5.4916515	13.4688005	99.0400009
-5.4916515	13.5176001	100.7500000
-5.4916515	13.5664005	102.8699951
-5.4916515	13.6152000	103.6800003
-5.4916515	13.6640005	101.6800003
-5.4916515	13.7128000	104.7500000
-5.4916515	13.7616005	105.6500015
-5.4916515	13.8104000	103.1899948
-5.4916515	13.8592005	100.2900009
-5.4916515	13.9080000	102.1399994
-5.4916515	13.9568005	100.3300018
-5.4916515	14.0056000	101.9000015
-5.4916515	14.0544004	104.0999985
-5.4916515	14.1032000	103.9599991
-5.4916515	14.1520004	104.0299988
-5.4916515	14.2007999	105.0599976
-5.4916515	14.2496004	101.8199997
-5.4916515	14.2983999	99.9000015
-5.4916515	14.3472004	99.6299973
-5.4916515	14.3959999	98.4899979
-5.4916515	14.4448004	100.0299988
-5.4916515	14.4935999	100.1699982
-5.4916515	14.5424004	99.6699982
-5.4916515	14.5911999	103.3399963
-5.4916515	14.6400003	101.9499969
-5.4916515	14.6887999	102.5199966
-5.4916515	14.7376003	106.0299988
-5.4916515	14.7863998	104.0699997
-5.4916515	14.8352003	102.2900009
-5.4916515	14.8839998	102.7900009
-5.4916515	14.9328003	103.8499985
-5.4916515	14.9815998	101.7500000
-5.4916515	15.0304003	101.4099960
-5.4855156	0.3416000	101.0199966
-5.4855156	0.3904000	106.4700012
-5.4855156	0.4392000	104.7799988
-5.4855156	0.4880000	105.5100021
-5.4855156	0.5368000	104.2399979
-5.4855156	0.5856000	104.3499985

-5.4855156	0.6344000	106.5800018
-5.4855156	0.6832000	104.8300018
-5.4855156	0.7320000	103.6199951
-5.4855156	0.7808000	102.3600006
-5.4855156	0.8296000	106.6500015
-5.4855156	0.8784000	107.1599960
-5.4855156	0.9272000	106.0100021
-5.4855156	0.9760000	109.3399963
-5.4855156	1.0247999	106.5599976
-5.4855156	1.0735999	105.7799988
-5.4855156	1.1223999	106.9899979
-5.4855156	1.1711999	106.0599976
-5.4855156	1.2200000	104.8799973
-5.4855156	1.2688000	104.7200012
-5.4855156	1.3176000	105.3499985
-5.4855156	1.3664000	106.2099991
-5.4855156	1.4152000	104.1199951
-5.4855156	1.4640000	103.2900009
-5.4855156	1.5128000	109.1500015
-5.4855156	1.5616000	109.3300018
-5.4855156	1.6104000	107.2600021
-5.4855156	1.6592000	101.3899994
-5.4855156	1.7079999	106.5000000
-5.4855156	1.7567999	106.8399963
-5.4855156	1.8055999	104.6100006
-5.4855156	1.8543999	104.3600006
-5.4855156	1.9031999	104.1800003
-5.4855156	1.9519999	103.0499954
-5.4855156	2.0007999	104.1999969
-5.4855156	2.0495999	102.7500000
-5.4855156	2.0983999	102.9899979
-5.4855156	2.1471999	102.5499954
-5.4855156	2.1959999	104.1999969
-5.4855156	2.2447999	106.1999969
-5.4855156	2.2935998	101.0100021
-5.4855156	2.3423998	103.2999954
-5.4855156	2.3912001	106.5499954
-5.4855156	2.4400001	106.6399994
-5.4855156	2.4888000	105.2799988
-5.4855156	2.5376000	103.1500015
-5.4855156	2.5864000	104.4300003
-5.4855156	2.6352000	105.1699982
-5.4855156	2.6840000	106.5899963
-5.4855156	2.7328000	103.2099991
-5.4855156	2.7816000	106.1399994
-5.4855156	2.8304000	103.3499985
-5.4855156	2.8792000	102.1999969
-5.4855156	2.9280000	100.1399994
-5.4855156	2.9768000	103.5699997
-5.4855156	3.0256000	105.8899994
-5.4855156	3.0743999	107.5999985
-5.4855156	3.1231999	106.4199982
-5.4855156	3.1719999	104.5400009
-5.4855156	3.2207999	106.3600006
-5.4855156	3.2695999	106.4099960
-5.4855156	3.3183999	104.1500015
-5.4855156	3.3671999	101.1800003
-5.4855156	3.4159999	104.2099991

-5.4855156	3.4647999	103.7699966
-5.4855156	3.5135999	101.7099991
-5.4855156	3.5623999	103.9499969
-5.4855156	3.6111999	104.1100006
-5.4855156	3.6599998	100.2799988
-5.4855156	3.7087998	100.6100006
-5.4855156	3.7575998	103.8799973
-5.4855156	3.8063998	104.8699951
-5.4855156	3.8551998	106.3199997
-5.4855156	3.9039998	103.7099991
-5.4855156	3.9527998	102.4799957
-5.4855156	4.0016003	104.5199966
-5.4855156	4.0503998	99.5699997
-5.4855156	4.0991998	104.5199966
-5.4855156	4.1479998	104.4300003
-5.4855156	4.1967998	106.0699997
-5.4855156	4.2455997	106.5800018
-5.4855156	4.2943997	104.1699982
-5.4855156	4.3431997	104.5100021
-5.4855156	4.3919997	102.5699997
-5.4855156	4.4407997	100.1100006
-5.4855156	4.4895997	101.7299957
-5.4855156	4.5383997	105.9799957
-5.4855156	4.5871997	107.0800018
-5.4855156	4.6359997	107.0499954
-5.4855156	4.6847997	115.7200012
-5.4855156	4.7336001	116.1299973
-5.4855156	4.7824001	109.8300018
-5.4855156	4.8312001	104.6999969
-5.4855156	4.8800001	106.6399994
-5.4855156	4.9288001	105.9499969
-5.4855156	4.9776001	103.0599976
-5.4855156	5.0264001	102.3600006
-5.4855156	5.0752001	104.3099976
-5.4855156	5.1240001	104.9199982
-5.4855156	5.1728001	105.3499985
-5.4855156	5.2216001	107.0499954
-5.4855156	5.2704000	107.7099991
-5.4855156	5.3192000	105.3899994
-5.4855156	5.3680000	105.3799973
-5.4855156	5.4168000	104.5199966
-5.4855156	5.4656000	103.3199997
-5.4855156	5.5144000	105.7600021
-5.4855156	5.5632000	105.0899963
-5.4855156	5.6120000	101.6399994
-5.4855156	5.6608000	102.7799988
-5.4855156	5.7096000	100.0199966
-5.4855156	5.7584000	104.4499969
-5.4855156	5.8072000	104.3099976
-5.4855156	5.8559999	105.7600021
-5.4855156	5.9047999	103.5299988
-5.4855156	5.9535999	102.3199997
-5.4855156	6.0023999	102.4799957
-5.4855156	6.0511999	100.4599991
-5.4855156	6.0999999	104.8099976
-5.4855156	6.1487999	103.4099960
-5.4855156	6.1975999	106.2900009
-5.4855156	6.2463999	107.6399994

-5.4855156	6.2951999	106.9000015
-5.4855156	6.3439999	105.6500015
-5.4855156	6.3927999	101.0100021
-5.4855156	6.4415998	102.2099991
-5.4855156	6.4903998	102.6800003
-5.4855156	6.5391998	106.0000000
-5.4855156	6.5879998	106.8199997
-5.4855156	6.6367998	103.7099991
-5.4855156	6.6855998	101.4499969
-5.4855156	6.7343998	104.4899979
-5.4855156	6.7831998	105.4899979
-5.4855156	6.8319998	104.0899963
-5.4855156	6.8807998	102.7999954
-5.4855156	6.9295998	101.5400009
-5.4855156	6.9783998	100.7500000
-5.4855156	7.0271997	105.3699951
-5.4855156	7.0759997	103.8899994
-5.4855156	7.1247997	103.2999954
-5.4855156	7.1735997	101.0000000
-5.4855156	7.2223997	101.5699997
-5.4855156	7.2711997	102.4599991
-5.4855156	7.3199997	101.3600006
-5.4855156	7.3687997	103.9099960
-5.4855156	7.4175997	104.4199982
-5.4855156	7.4663997	105.0100021
-5.4855156	7.5151997	105.2399979
-5.4855156	7.5639997	101.0000000
-5.4855156	7.6127996	99.7900009
-5.4855156	7.6615996	103.4799957
-5.4855156	7.7103996	105.1899948
-5.4855156	7.7591996	104.9899979
-5.4855156	7.8079996	104.3199997
-5.4855156	7.8567996	105.3899994
-5.4855156	7.9056001	103.3499985
-5.4855156	7.9544001	102.6699982
-5.4855156	8.0032005	99.4499969
-5.4855156	8.0520000	98.6500015
-5.4855156	8.1008005	100.1599960
-5.4855156	8.1496000	103.5899963
-5.4855156	8.1984005	105.4499969
-5.4855156	8.2472000	104.5499954
-5.4855156	8.2960005	105.9899979
-5.4855156	8.3448000	104.0400009
-5.4855156	8.3936005	104.5299988
-5.4855156	8.4424000	105.3899994
-5.4855156	8.4912004	104.6199951
-5.4855156	8.5400000	106.3300018
-5.4855156	8.5888004	105.0899963
-5.4855156	8.6375999	99.1599960
-5.4855156	8.6864004	101.6899948
-5.4855156	8.7351999	102.5000000
-5.4855156	8.7840004	102.8399963
-5.4855156	8.8327999	102.7099991
-5.4855156	8.8816004	102.6100006
-5.4855156	8.9303999	101.6299973
-5.4855156	8.9792004	100.5899963
-5.4855156	9.0279999	100.8199997
-5.4855156	9.0768003	103.9700012

-5.4855156	9.1255999	104.7900009
-5.4855156	9.1744003	103.0299988
-5.4855156	9.2231998	102.6500015
-5.4855156	9.2720003	106.4000015
-5.4855156	9.3207998	107.8099976
-5.4855156	9.3696003	107.0299988
-5.4855156	9.4184008	103.2999954
-5.4855156	9.4672003	102.1199951
-5.4855156	9.5160007	99.1199951
-5.4855156	9.5648003	101.0299988
-5.4855156	9.6136007	103.1100006
-5.4855156	9.6624002	103.4899979
-5.4855156	9.7112007	99.5199966
-5.4855156	9.7600002	101.9000015
-5.4855156	9.8088007	102.7099991
-5.4855156	9.8576002	101.3300018
-5.4855156	9.9064007	103.7500000
-5.4855156	9.9552002	104.2999954
-5.4855156	10.0040007	105.0599976
-5.4855156	10.0528002	104.7500000
-5.4855156	10.1016006	102.8399963
-5.4855156	10.1504002	104.4000015
-5.4855156	10.1992006	103.3600006
-5.4855156	10.2480001	101.1399994
-5.4855156	10.2968006	104.7600021
-5.4855156	10.3456001	105.2200012
-5.4855156	10.3944006	102.5000000
-5.4855156	10.4432001	102.8799973
-5.4855156	10.4920006	104.0400009
-5.4855156	10.5408001	103.3300018
-5.4855156	10.5896006	100.0899963
-5.4855156	10.6384001	102.1399994
-5.4855156	10.6872005	102.4300003
-5.4855156	10.7360001	106.3600006
-5.4855156	10.7848005	103.6599960
-5.4855156	10.8336000	103.9499969
-5.4855156	10.8824005	104.0499954
-5.4855156	10.9312000	104.2600021
-5.4855156	10.9800005	101.8499985
-5.4855156	11.0288000	103.8300018
-5.4855156	11.0776005	105.5599976
-5.4855156	11.1264000	103.4199982
-5.4855156	11.1752005	101.4899979
-5.4855156	11.2240000	101.4199982
-5.4855156	11.2728004	100.5100021
-5.4855156	11.3216000	99.8899994
-5.4855156	11.3704004	101.6599960
-5.4855156	11.4191999	103.4799957
-5.4855156	11.4680004	103.5699997
-5.4855156	11.5167999	101.9599991
-5.4855156	11.5656004	102.0299988
-5.4855156	11.6143999	102.4000015
-5.4855156	11.6632004	101.9599991
-5.4855156	11.7119999	101.0100021
-5.4855156	11.7608004	100.7500000
-5.4855156	11.8095999	101.8899994
-5.4855156	11.8584003	106.0100021
-5.4855156	11.9071999	105.9300003

-5.4855156	11.9560003	104.1599960
-5.4855156	12.0047998	101.7500000
-5.4855156	12.0536003	101.6999969
-5.4855156	12.1023998	105.0800018
-5.4855156	12.1512003	102.2500000
-5.4855156	12.1999998	99.5599976
-5.4855156	12.2488003	101.4000015
-5.4855156	12.2975998	101.3699951
-5.4855156	12.3464003	100.4199982
-5.4855156	12.3951998	102.5699997
-5.4855156	12.4440002	103.6500015
-5.4855156	12.4927998	101.1999969
-5.4855156	12.5416002	102.6599960
-5.4855156	12.5904007	100.9300003
-5.4855156	12.6392002	100.9899979
-5.4855156	12.6880007	103.4099960
-5.4855156	12.7368002	104.1100006
-5.4855156	12.7856007	105.2099991
-5.4855156	12.8344002	103.9000015
-5.4855156	12.8832006	105.0499954
-5.4855156	12.9320002	104.2500000
-5.4855156	12.9808006	101.2399979
-5.4855156	13.0296001	101.5499954
-5.4855156	13.0784006	103.3199997
-5.4855156	13.1272001	104.5800018
-5.4855156	13.1760006	103.8399963
-5.4855156	13.2248001	104.0299988
-5.4855156	13.2736006	101.8499985
-5.4855156	13.3224001	99.1399994
-5.4855156	13.3712006	102.9099960
-5.4855156	13.4200001	100.0699997
-5.4855156	13.4688005	99.9899979
-5.4855156	13.5176001	100.5299988
-5.4855156	13.5664005	102.4899979
-5.4855156	13.6152000	103.4199982
-5.4855156	13.6640005	101.7699966
-5.4855156	13.7128000	104.4099960
-5.4855156	13.7616005	105.5199966
-5.4855156	13.8104000	103.0699997
-5.4855156	13.8592005	101.0999985
-5.4855156	13.9080000	103.0999985
-5.4855156	13.9568005	100.9199982
-5.4855156	14.0056000	101.1699982
-5.4855156	14.0544004	103.5100021
-5.4855156	14.1032000	103.6500015
-5.4855156	14.1520004	103.6100006
-5.4855156	14.2007999	105.2099991
-5.4855156	14.2496004	103.0999985
-5.4855156	14.2983999	100.7600021
-5.4855156	14.3472004	99.5000000
-5.4855156	14.3959999	98.8899994
-5.4855156	14.4448004	100.4599991
-5.4855156	14.4935999	100.0000000
-5.4855156	14.5424004	98.2600021
-5.4855156	14.5911999	103.3399963
-5.4855156	14.6400003	102.8399963
-5.4855156	14.6887999	102.9000015
-5.4855156	14.7376003	106.4499969

-5.4855156	14.7863998	105.1399994
-5.4855156	14.8352003	103.2399979
-5.4855156	14.8839998	102.2799988
-5.4855156	14.9328003	103.5199966
-5.4855156	14.9815998	100.9099960
-5.4855156	15.0304003	99.8799973
-5.4793797	0.3416000	99.6800003
-5.4793797	0.3904000	105.2999954
-5.4793797	0.4392000	103.2999954
-5.4793797	0.4880000	104.2699966
-5.4793797	0.5368000	103.5499954
-5.4793797	0.5856000	103.2399979
-5.4793797	0.6344000	105.6599960
-5.4793797	0.6832000	104.3099976
-5.4793797	0.7320000	104.2799988
-5.4793797	0.7808000	103.7999954
-5.4793797	0.8296000	107.0299988
-5.4793797	0.8784000	107.1599960
-5.4793797	0.9272000	106.9499969
-5.4793797	0.9760000	110.4099960
-5.4793797	1.0247999	108.3899994
-5.4793797	1.0735999	106.0800018
-5.4793797	1.1223999	106.1500015
-5.4793797	1.1711999	105.6399994
-5.4793797	1.2200000	106.0999985
-5.4793797	1.2688000	105.2900009
-5.4793797	1.3176000	105.2500000
-5.4793797	1.3664000	107.0000000
-5.4793797	1.4152000	104.8899994
-5.4793797	1.4640000	104.1100006
-5.4793797	1.5128000	109.2600021
-5.4793797	1.5616000	110.0599976
-5.4793797	1.6104000	107.4899979
-5.4793797	1.6592000	100.8799973
-5.4793797	1.7079999	105.5499954
-5.4793797	1.7567999	106.8099976
-5.4793797	1.8055999	103.9199982
-5.4793797	1.8543999	103.7799988
-5.4793797	1.9031999	104.7999954
-5.4793797	1.9519999	104.1699982
-5.4793797	2.0007999	104.5699997
-5.4793797	2.0495999	102.3499985
-5.4793797	2.0983999	103.1100006
-5.4793797	2.1471999	102.3699951
-5.4793797	2.1959999	103.3600006
-5.4793797	2.2447999	105.7200012
-5.4793797	2.2935998	100.8499985
-5.4793797	2.3423998	102.5800018
-5.4793797	2.3912001	105.7500000
-5.4793797	2.4400001	105.6199951
-5.4793797	2.4888000	104.6399994
-5.4793797	2.5376000	102.4000015
-5.4793797	2.5864000	104.6800003
-5.4793797	2.6352000	103.7200012
-5.4793797	2.6840000	106.5100021
-5.4793797	2.7328000	103.2399979
-5.4793797	2.7816000	106.5499954
-5.4793797	2.8304000	103.9499969

-5.4793797	2.8792000	101.3399963
-5.4793797	2.9280000	98.0800018
-5.4793797	2.9768000	102.9399948
-5.4793797	3.0256000	106.5000000
-5.4793797	3.0743999	107.6999969
-5.4793797	3.1231999	106.3600006
-5.4793797	3.1719999	103.5899963
-5.4793797	3.2207999	105.3600006
-5.4793797	3.2695999	105.5800018
-5.4793797	3.3183999	103.6100006
-5.4793797	3.3671999	101.2399979
-5.4793797	3.4159999	104.1100006
-5.4793797	3.4647999	103.9499969
-5.4793797	3.5135999	102.6800003
-5.4793797	3.5623999	104.3399963
-5.4793797	3.6111999	103.7099991
-5.4793797	3.6599998	99.9000015
-5.4793797	3.7087998	100.9599991
-5.4793797	3.7575998	103.7600021
-5.4793797	3.8063998	104.7399979
-5.4793797	3.8551998	106.1100006
-5.4793797	3.9039998	104.1100006
-5.4793797	3.9527998	102.1100006
-5.4793797	4.0016003	103.7900009
-5.4793797	4.0503998	100.5199966
-5.4793797	4.0991998	103.9300003
-5.4793797	4.1479998	104.9700012
-5.4793797	4.1967998	107.1599960
-5.4793797	4.2455997	107.6800003
-5.4793797	4.2943997	104.5100021
-5.4793797	4.3431997	104.9300003
-5.4793797	4.3919997	103.0800018
-5.4793797	4.4407997	99.9799957
-5.4793797	4.4895997	101.4199982
-5.4793797	4.5383997	105.9300003
-5.4793797	4.5871997	107.1899948
-5.4793797	4.6359997	106.7999954
-5.4793797	4.6847997	115.1299973
-5.4793797	4.7336001	115.6299973
-5.4793797	4.7824001	110.0199966
-5.4793797	4.8312001	104.7799988
-5.4793797	4.8800001	106.9399948
-5.4793797	4.9288001	106.4099960
-5.4793797	4.9776001	103.6299973
-5.4793797	5.0264001	101.0199966
-5.4793797	5.0752001	103.9599991
-5.4793797	5.1240001	104.5599976
-5.4793797	5.1728001	105.4000015
-5.4793797	5.2216001	107.3699951
-5.4793797	5.2704000	107.2299957
-5.4793797	5.3192000	105.2399979
-5.4793797	5.3680000	104.9899979
-5.4793797	5.4168000	103.6100006
-5.4793797	5.4656000	103.0000000
-5.4793797	5.5144000	106.2699966
-5.4793797	5.5632000	105.5000000
-5.4793797	5.6120000	101.5499954
-5.4793797	5.6608000	103.8799973

-5.4793797	5.7096000	100.6100006
-5.4793797	5.7584000	104.2799988
-5.4793797	5.8072000	104.3099976
-5.4793797	5.8559999	105.4799957
-5.4793797	5.9047999	102.4199982
-5.4793797	5.9535999	101.9700012
-5.4793797	6.0023999	103.2699966
-5.4793797	6.0511999	101.0899963
-5.4793797	6.0999999	105.5499954
-5.4793797	6.1487999	103.1100006
-5.4793797	6.1975999	105.5199966
-5.4793797	6.2463999	107.2299957
-5.4793797	6.2951999	106.1800003
-5.4793797	6.3439999	104.5800018
-5.4793797	6.3927999	99.2500000
-5.4793797	6.4415998	100.7900009
-5.4793797	6.4903998	100.7299957
-5.4793797	6.5391998	104.7299957
-5.4793797	6.5879998	106.5699997
-5.4793797	6.6367998	103.1500015
-5.4793797	6.6855998	101.3399963
-5.4793797	6.7343998	104.8300018
-5.4793797	6.7831998	105.9499969
-5.4793797	6.8319998	104.1699982
-5.4793797	6.8807998	103.5199966
-5.4793797	6.9295998	101.3699951
-5.4793797	6.9783998	100.7699966
-5.4793797	7.0271997	104.8399963
-5.4793797	7.0759997	103.8499985
-5.4793797	7.1247997	103.0100021
-5.4793797	7.1735997	101.5499954
-5.4793797	7.2223997	102.6999969
-5.4793797	7.2711997	102.1399994
-5.4793797	7.3199997	101.6899948
-5.4793797	7.3687997	104.7500000
-5.4793797	7.4175997	104.6299973
-5.4793797	7.4663997	104.4300003
-5.4793797	7.5151997	105.6899948
-5.4793797	7.5639997	100.5400009
-5.4793797	7.6127996	98.4199982
-5.4793797	7.6615996	103.5000000
-5.4793797	7.7103996	105.5400009
-5.4793797	7.7591996	104.9599991
-5.4793797	7.8079996	104.2699966
-5.4793797	7.8567996	105.1699982
-5.4793797	7.9056001	102.4000015
-5.4793797	7.9544001	102.6399994
-5.4793797	8.0032005	100.9599991
-5.4793797	8.0520000	99.6500015
-5.4793797	8.1008005	98.9700012
-5.4793797	8.1496000	103.6699982
-5.4793797	8.1984005	105.5499954
-5.4793797	8.2472000	103.4700012
-5.4793797	8.2960005	105.5100021
-5.4793797	8.3448000	104.5800018
-5.4793797	8.3936005	104.0999985
-5.4793797	8.4424000	105.4199982
-5.4793797	8.4912004	105.3099976

-5.4793797	8.5400000	106.6599960
-5.4793797	8.5888004	105.2200012
-5.4793797	8.6375999	100.2999954
-5.4793797	8.6864004	102.0599976
-5.4793797	8.7351999	102.6599960
-5.4793797	8.7840004	102.7999954
-5.4793797	8.8327999	102.0199966
-5.4793797	8.8816004	101.9899979
-5.4793797	8.9303999	100.4499969
-5.4793797	8.9792004	100.1500015
-5.4793797	9.0279999	101.4599991
-5.4793797	9.0768003	103.7999954
-5.4793797	9.1255999	104.1199951
-5.4793797	9.1744003	102.7500000
-5.4793797	9.2231998	102.8099976
-5.4793797	9.2720003	106.6999969
-5.4793797	9.3207998	108.0999985
-5.4793797	9.3696003	106.6599960
-5.4793797	9.4184008	102.8399963
-5.4793797	9.4672003	102.4399948
-5.4793797	9.5160007	99.3399963
-5.4793797	9.5648003	101.3799973
-5.4793797	9.6136007	103.2500000
-5.4793797	9.6624002	103.4000015
-5.4793797	9.7112007	99.5299988
-5.4793797	9.7600002	100.9700012
-5.4793797	9.8088007	102.0199966
-5.4793797	9.8576002	101.9099960
-5.4793797	9.9064007	104.2099991
-5.4793797	9.9552002	104.1999969
-5.4793797	10.0040007	104.3399963
-5.4793797	10.0528002	104.5599976
-5.4793797	10.1016006	102.8600006
-5.4793797	10.1504002	104.1800003
-5.4793797	10.1992006	102.9599991
-5.4793797	10.2480001	101.2699966
-5.4793797	10.2968006	104.5199966
-5.4793797	10.3456001	104.8300018
-5.4793797	10.3944006	101.9799957
-5.4793797	10.4432001	103.0899963
-5.4793797	10.4920006	103.8699951
-5.4793797	10.5408001	103.2699966
-5.4793797	10.5896006	99.0199966
-5.4793797	10.6384001	102.9000015
-5.4793797	10.6872005	104.3099976
-5.4793797	10.7360001	106.9199982
-5.4793797	10.7848005	103.1899948
-5.4793797	10.8336000	103.7099991
-5.4793797	10.8824005	103.7299957
-5.4793797	10.9312000	104.0400009
-5.4793797	10.9800005	102.9700012
-5.4793797	11.0288000	104.5000000
-5.4793797	11.0776005	105.8600006
-5.4793797	11.1264000	103.9300003
-5.4793797	11.1752005	102.3600006
-5.4793797	11.2240000	102.1699982
-5.4793797	11.2728004	101.2799988
-5.4793797	11.3216000	100.5599976

-5.4793797	11.3704004	101.2999954
-5.4793797	11.4191999	102.6299973
-5.4793797	11.4680004	102.5199966
-5.4793797	11.5167999	100.8300018
-5.4793797	11.5656004	100.6100006
-5.4793797	11.6143999	102.2999954
-5.4793797	11.6632004	102.6399994
-5.4793797	11.7119999	102.3600006
-5.4793797	11.7608004	100.5100021
-5.4793797	11.8095999	102.2500000
-5.4793797	11.8584003	105.9700012
-5.4793797	11.9071999	106.1299973
-5.4793797	11.9560003	103.7500000
-5.4793797	12.0047998	100.6999969
-5.4793797	12.0536003	101.2799988
-5.4793797	12.1023998	104.9300003
-5.4793797	12.1512003	102.8499985
-5.4793797	12.1999998	99.7799988
-5.4793797	12.2488003	101.6899948
-5.4793797	12.2975998	101.2200012
-5.4793797	12.3464003	99.4700012
-5.4793797	12.3951998	102.1699982
-5.4793797	12.4440002	103.9199982
-5.4793797	12.4927998	101.7200012
-5.4793797	12.5416002	102.8099976
-5.4793797	12.5904007	101.7600021
-5.4793797	12.6392002	102.0000000
-5.4793797	12.6880007	103.8600006
-5.4793797	12.7368002	104.2999954
-5.4793797	12.7856007	105.8600006
-5.4793797	12.8344002	104.9599991
-5.4793797	12.8832006	104.4799957
-5.4793797	12.9320002	103.8600006
-5.4793797	12.9808006	101.0999985
-5.4793797	13.0296001	101.0899963
-5.4793797	13.0784006	102.3899994
-5.4793797	13.1272001	104.7299957
-5.4793797	13.1760006	104.4599991
-5.4793797	13.2248001	104.2200012
-5.4793797	13.2736006	101.9899979
-5.4793797	13.3224001	100.0999985
-5.4793797	13.3712006	103.1399994
-5.4793797	13.4200001	99.9700012
-5.4793797	13.4688005	100.4599991
-5.4793797	13.5176001	100.3399963
-5.4793797	13.5664005	101.5100021
-5.4793797	13.6152000	102.7299957
-5.4793797	13.6640005	101.7999954
-5.4793797	13.7128000	103.9599991
-5.4793797	13.7616005	104.7500000
-5.4793797	13.8104000	102.5000000
-5.4793797	13.8592005	101.4899979
-5.4793797	13.9080000	103.7600021
-5.4793797	13.9568005	101.7999954
-5.4793797	14.0056000	101.6299973
-5.4793797	14.0544004	103.1299973
-5.4793797	14.1032000	103.4199982
-5.4793797	14.1520004	103.0400009

-5.4793797	14.2007999	104.7099991
-5.4793797	14.2496004	103.4099960
-5.4793797	14.2983999	101.4000015
-5.4793797	14.3472004	99.9899979
-5.4793797	14.3959999	99.9399948
-5.4793797	14.4448004	101.4899979
-5.4793797	14.4935999	100.8799973
-5.4793797	14.5424004	98.5499954
-5.4793797	14.5911999	103.7399979
-5.4793797	14.6400003	102.9799957
-5.4793797	14.6887999	102.5499954
-5.4793797	14.7376003	105.9300003
-5.4793797	14.7863998	105.6299973
-5.4793797	14.8352003	103.9700012
-5.4793797	14.8839998	101.5699997
-5.4793797	14.9328003	103.2999954
-5.4793797	14.9815998	100.4499969
-5.4793797	15.0304003	98.7399979
-5.4732437	0.3416000	98.8300018
-5.4732437	0.3904000	103.9899979
-5.4732437	0.4392000	101.9099960
-5.4732437	0.4880000	103.3399963
-5.4732437	0.5368000	103.2399979
-5.4732437	0.5856000	102.9000015
-5.4732437	0.6344000	105.3399963
-5.4732437	0.6832000	104.4099960
-5.4732437	0.7320000	105.7299957
-5.4732437	0.7808000	105.1299973
-5.4732437	0.8296000	106.4000015
-5.4732437	0.8784000	106.6299973
-5.4732437	0.9272000	107.2799988
-5.4732437	0.9760000	110.5899963
-5.4732437	1.0247999	109.2999954
-5.4732437	1.0735999	106.5299988
-5.4732437	1.1223999	105.2500000
-5.4732437	1.1711999	104.8399963
-5.4732437	1.2200000	106.7900009
-5.4732437	1.2688000	105.8799973
-5.4732437	1.3176000	105.4599991
-5.4732437	1.3664000	107.4599991
-5.4732437	1.4152000	105.0699997
-5.4732437	1.4640000	104.9199982
-5.4732437	1.5128000	109.2999954
-5.4732437	1.5616000	110.5000000
-5.4732437	1.6104000	107.8099976
-5.4732437	1.6592000	100.7600021
-5.4732437	1.7079999	104.1800003
-5.4732437	1.7567999	106.2600021
-5.4732437	1.8055999	104.3799973
-5.4732437	1.8543999	104.1599960
-5.4732437	1.9031999	105.9599991
-5.4732437	1.9519999	106.1199951
-5.4732437	2.0007999	104.6899948
-5.4732437	2.0495999	102.1399994
-5.4732437	2.0983999	103.6299973
-5.4732437	2.1471999	102.5599976
-5.4732437	2.1959999	103.0599976
-5.4732437	2.2447999	105.1699982

-5.4732437	2.2935998	100.1899948
-5.4732437	2.3423998	101.3799973
-5.4732437	2.3912001	104.6899948
-5.4732437	2.4400001	104.8099976
-5.4732437	2.4888000	104.3099976
-5.4732437	2.5376000	101.7399979
-5.4732437	2.5864000	105.1500015
-5.4732437	2.6352000	102.8399963
-5.4732437	2.6840000	106.1800003
-5.4732437	2.7328000	104.3399963
-5.4732437	2.7816000	107.0999985
-5.4732437	2.8304000	104.1199951
-5.4732437	2.8792000	101.0100021
-5.4732437	2.9280000	96.9300003
-5.4732437	2.9768000	103.1699982
-5.4732437	3.0256000	107.3399963
-5.4732437	3.0743999	107.8399963
-5.4732437	3.1231999	106.3899994
-5.4732437	3.1719999	102.2999954
-5.4732437	3.2207999	103.7500000
-5.4732437	3.2695999	104.0999985
-5.4732437	3.3183999	102.2500000
-5.4732437	3.3671999	100.5800018
-5.4732437	3.4159999	103.2799988
-5.4732437	3.4647999	104.0499954
-5.4732437	3.5135999	102.8099976
-5.4732437	3.5623999	103.7600021
-5.4732437	3.6111999	103.4199982
-5.4732437	3.6599998	100.9099960
-5.4732437	3.7087998	101.5199966
-5.4732437	3.7575998	103.5499954
-5.4732437	3.8063998	105.5899963
-5.4732437	3.8551998	106.2600021
-5.4732437	3.9039998	104.4000015
-5.4732437	3.9527998	101.9499969
-5.4732437	4.0016003	103.0199966
-5.4732437	4.0503998	101.6100006
-5.4732437	4.0991998	103.5199966
-5.4732437	4.1479998	105.7399979
-5.4732437	4.1967998	108.3899994
-5.4732437	4.2455997	108.9099960
-5.4732437	4.2943997	104.7799988
-5.4732437	4.3431997	105.8799973
-5.4732437	4.3919997	104.0299988
-5.4732437	4.4407997	99.9000015
-5.4732437	4.4895997	100.4899979
-5.4732437	4.5383997	105.3099976
-5.4732437	4.5871997	107.2299957
-5.4732437	4.6359997	107.3499985
-5.4732437	4.6847997	114.4700012
-5.4732437	4.7336001	114.8199997
-5.4732437	4.7824001	110.0999985
-5.4732437	4.8312001	105.5999985
-5.4732437	4.8800001	107.8199997
-5.4732437	4.9288001	107.2500000
-5.4732437	4.9776001	104.7099991
-5.4732437	5.0264001	100.4599991
-5.4732437	5.0752001	104.1699982

-5.4732437	5.1240001	104.8799973
-5.4732437	5.1728001	105.3600006
-5.4732437	5.2216001	107.2600021
-5.4732437	5.2704000	105.2900009
-5.4732437	5.3192000	104.8899994
-5.4732437	5.3680000	104.4199982
-5.4732437	5.4168000	102.5499954
-5.4732437	5.4656000	103.4799957
-5.4732437	5.5144000	106.9700012
-5.4732437	5.5632000	105.9199982
-5.4732437	5.6120000	102.0400009
-5.4732437	5.6608000	105.0599976
-5.4732437	5.7096000	101.3099976
-5.4732437	5.7584000	104.3399963
-5.4732437	5.8072000	104.5800018
-5.4732437	5.8559999	105.2299957
-5.4732437	5.9047999	101.8099976
-5.4732437	5.9535999	101.6999969
-5.4732437	6.0023999	104.1699982
-5.4732437	6.0511999	102.1899948
-5.4732437	6.0999999	106.0899963
-5.4732437	6.1487999	102.5899963
-5.4732437	6.1975999	104.3799973
-5.4732437	6.2463999	106.3099976
-5.4732437	6.2951999	105.1699982
-5.4732437	6.3439999	103.6100006
-5.4732437	6.3927999	99.4499969
-5.4732437	6.4415998	100.4599991
-5.4732437	6.4903998	100.3699951
-5.4732437	6.5391998	103.8399963
-5.4732437	6.5879998	106.4099960
-5.4732437	6.6367998	103.1199951
-5.4732437	6.6855998	102.0999985
-5.4732437	6.7343998	105.6999969
-5.4732437	6.7831998	106.4000015
-5.4732437	6.8319998	104.0400009
-5.4732437	6.8807998	104.1399994
-5.4732437	6.9295998	101.6599960
-5.4732437	6.9783998	101.1699982
-5.4732437	7.0271997	104.4799957
-5.4732437	7.0759997	103.8600006
-5.4732437	7.1247997	102.6800003
-5.4732437	7.1735997	102.2299957
-5.4732437	7.2223997	103.8300018
-5.4732437	7.2711997	102.2099991
-5.4732437	7.3199997	102.5299988
-5.4732437	7.3687997	105.4499969
-5.4732437	7.4175997	104.5800018
-5.4732437	7.4663997	103.9799957
-5.4732437	7.5151997	105.7200012
-5.4732437	7.5639997	100.7600021
-5.4732437	7.6127996	98.3600006
-5.4732437	7.6615996	103.6199951
-5.4732437	7.7103996	105.7799988
-5.4732437	7.7591996	105.0899963
-5.4732437	7.8079996	104.3799973
-5.4732437	7.8567996	105.3799973
-5.4732437	7.9056001	102.7699966

-5.4732437	7.9544001	103.7500000
-5.4732437	8.0032005	103.2500000
-5.4732437	8.0520000	100.9700012
-5.4732437	8.1008005	98.7099991
-5.4732437	8.1496000	104.1399994
-5.4732437	8.1984005	105.9099960
-5.4732437	8.2472000	103.1699982
-5.4732437	8.2960005	105.7099991
-5.4732437	8.3448000	104.9700012
-5.4732437	8.3936005	103.6199951
-5.4732437	8.4424000	105.4000015
-5.4732437	8.4912004	105.3300018
-5.4732437	8.5400000	106.2600021
-5.4732437	8.5888004	105.0599976
-5.4732437	8.6375999	101.2500000
-5.4732437	8.6864004	102.6699982
-5.4732437	8.7351999	103.0999985
-5.4732437	8.7840004	103.1100006
-5.4732437	8.8327999	102.6299973
-5.4732437	8.8816004	102.4199982
-5.4732437	8.9303999	99.8199997
-5.4732437	8.9792004	101.4899979
-5.4732437	9.0279999	102.9199982
-5.4732437	9.0768003	104.0499954
-5.4732437	9.1255999	103.6199951
-5.4732437	9.1744003	102.4599991
-5.4732437	9.2231998	103.1199951
-5.4732437	9.2720003	107.1100006
-5.4732437	9.3207998	108.6399994
-5.4732437	9.3696003	106.4499969
-5.4732437	9.4184008	102.0299988
-5.4732437	9.4672003	103.1599960
-5.4732437	9.5160007	101.4300003
-5.4732437	9.5648003	102.3300018
-5.4732437	9.6136007	103.2999954
-5.4732437	9.6624002	103.2900009
-5.4732437	9.7112007	100.1500015
-5.4732437	9.7600002	100.8099976
-5.4732437	9.8088007	100.8600006
-5.4732437	9.8576002	102.3499985
-5.4732437	9.9064007	104.8600006
-5.4732437	9.9552002	104.3499985
-5.4732437	10.0040007	104.9099960
-5.4732437	10.0528002	105.4099960
-5.4732437	10.1016006	103.4499969
-5.4732437	10.1504002	104.3199997
-5.4732437	10.1992006	102.7299957
-5.4732437	10.2480001	101.4199982
-5.4732437	10.2968006	103.8300018
-5.4732437	10.3456001	104.2600021
-5.4732437	10.3944006	101.3799973
-5.4732437	10.4432001	102.8099976
-5.4732437	10.4920006	103.1399994
-5.4732437	10.5408001	103.5100021
-5.4732437	10.5896006	100.0400009
-5.4732437	10.6384001	104.1199951
-5.4732437	10.6872005	105.8300018
-5.4732437	10.7360001	107.1399994

-5.4732437	10.7848005	104.0999985
-5.4732437	10.8336000	104.3499985
-5.4732437	10.8824005	103.5100021
-5.4732437	10.9312000	103.5000000
-5.4732437	10.9800005	104.0299988
-5.4732437	11.0288000	105.1899948
-5.4732437	11.0776005	106.0599976
-5.4732437	11.1264000	104.5599976
-5.4732437	11.1752005	103.1599960
-5.4732437	11.2240000	102.8799973
-5.4732437	11.2728004	102.2099991
-5.4732437	11.3216000	101.2500000
-5.4732437	11.3704004	100.7299957
-5.4732437	11.4191999	100.9099960
-5.4732437	11.4680004	101.5499954
-5.4732437	11.5167999	100.6699982
-5.4732437	11.5656004	100.8300018
-5.4732437	11.6143999	102.9199982
-5.4732437	11.6632004	103.5100021
-5.4732437	11.7119999	103.5699997
-5.4732437	11.7608004	100.5599976
-5.4732437	11.8095999	102.6399994
-5.4732437	11.8584003	105.5599976
-5.4732437	11.9071999	105.8399963
-5.4732437	11.9560003	103.2299957
-5.4732437	12.0047998	99.5199966
-5.4732437	12.0536003	101.5400009
-5.4732437	12.1023998	105.0299988
-5.4732437	12.1512003	103.4599991
-5.4732437	12.1999998	100.1800003
-5.4732437	12.2488003	101.6999969
-5.4732437	12.2975998	101.1399994
-5.4732437	12.3464003	99.6399994
-5.4732437	12.3951998	102.6199951
-5.4732437	12.4440002	104.7900009
-5.4732437	12.4927998	102.9700012
-5.4732437	12.5416002	103.3699951
-5.4732437	12.5904007	102.4799957
-5.4732437	12.6392002	103.2200012
-5.4732437	12.6880007	104.4599991
-5.4732437	12.7368002	104.9499969
-5.4732437	12.7856007	106.5000000
-5.4732437	12.8344002	105.7600021
-5.4732437	12.8832006	103.6199951
-5.4732437	12.9320002	103.5299988
-5.4732437	12.9808006	102.0800018
-5.4732437	13.0296001	100.6999969
-5.4732437	13.0784006	101.6899948
-5.4732437	13.1272001	104.8699951
-5.4732437	13.1760006	105.3300018
-5.4732437	13.2248001	104.8499985
-5.4732437	13.2736006	102.2200012
-5.4732437	13.3224001	100.9300003
-5.4732437	13.3712006	103.3600006
-5.4732437	13.4200001	100.3899994
-5.4732437	13.4688005	100.6399994
-5.4732437	13.5176001	100.5499954
-5.4732437	13.5664005	101.6399994

-5.4732437	13.6152000	103.0899963
-5.4732437	13.6640005	101.7900009
-5.4732437	13.7128000	103.7799988
-5.4732437	13.7616005	104.6299973
-5.4732437	13.8104000	103.4000015
-5.4732437	13.8592005	102.9199982
-5.4732437	13.9080000	104.6199951
-5.4732437	13.9568005	102.1699982
-5.4732437	14.0056000	102.0999985
-5.4732437	14.0544004	102.7399979
-5.4732437	14.1032000	103.2999954
-5.4732437	14.1520004	102.3300018
-5.4732437	14.2007999	103.7999954
-5.4732437	14.2496004	102.9499969
-5.4732437	14.2983999	102.1500015
-5.4732437	14.3472004	101.1199951
-5.4732437	14.3959999	101.3399963
-5.4732437	14.4448004	102.4399948
-5.4732437	14.4935999	101.8399963
-5.4732437	14.5424004	99.2600021
-5.4732437	14.5911999	104.7299957
-5.4732437	14.6400003	103.1599960
-5.4732437	14.6887999	100.8300018
-5.4732437	14.7376003	104.8300018
-5.4732437	14.7863998	106.1599960
-5.4732437	14.8352003	105.1199951
-5.4732437	14.8839998	100.8300018
-5.4732437	14.9328003	102.4099960
-5.4732437	14.9815998	99.6899948
-5.4732437	15.0304003	98.6199951
-5.4671078	0.3416000	99.9399948
-5.4671078	0.3904000	103.7699966
-5.4671078	0.4392000	101.9199982
-5.4671078	0.4880000	102.7999954
-5.4671078	0.5368000	102.7099991
-5.4671078	0.5856000	102.5299988
-5.4671078	0.6344000	105.5199966
-5.4671078	0.6832000	105.2799988
-5.4671078	0.7320000	106.3199997
-5.4671078	0.7808000	105.3600006
-5.4671078	0.8296000	105.4899979
-5.4671078	0.8784000	106.3899994
-5.4671078	0.9272000	108.0599976
-5.4671078	0.9760000	110.7399979
-5.4671078	1.0247999	110.0299988
-5.4671078	1.0735999	106.6100006
-5.4671078	1.1223999	104.8899994
-5.4671078	1.1711999	104.1399994
-5.4671078	1.2200000	106.3099976
-5.4671078	1.2688000	105.4399948
-5.4671078	1.3176000	104.6699982
-5.4671078	1.3664000	107.4300003
-5.4671078	1.4152000	105.3399963
-5.4671078	1.4640000	106.0199966
-5.4671078	1.5128000	108.7200012
-5.4671078	1.5616000	110.2600021
-5.4671078	1.6104000	107.7099991
-5.4671078	1.6592000	100.9399948

-5.4671078	1.7079999	102.6299973
-5.4671078	1.7567999	105.1699982
-5.4671078	1.8055999	104.2299957
-5.4671078	1.8543999	104.7799988
-5.4671078	1.9031999	107.2900009
-5.4671078	1.9519999	107.3199997
-5.4671078	2.0007999	104.3799973
-5.4671078	2.0495999	102.3499985
-5.4671078	2.0983999	104.1599960
-5.4671078	2.1471999	103.0999985
-5.4671078	2.1959999	103.9000015
-5.4671078	2.2447999	105.4099960
-5.4671078	2.2935998	100.6199951
-5.4671078	2.3423998	100.2600021
-5.4671078	2.3912001	104.2699966
-5.4671078	2.4400001	104.3699951
-5.4671078	2.4888000	103.8300018
-5.4671078	2.5376000	101.9799957
-5.4671078	2.5864000	104.4599991
-5.4671078	2.6352000	101.4300003
-5.4671078	2.6840000	105.4700012
-5.4671078	2.7328000	105.3600006
-5.4671078	2.7816000	107.3099976
-5.4671078	2.8304000	104.5599976
-5.4671078	2.8792000	101.7799988
-5.4671078	2.9280000	97.6999969
-5.4671078	2.9768000	103.7600021
-5.4671078	3.0256000	107.9399948
-5.4671078	3.0743999	107.6100006
-5.4671078	3.1231999	106.4899979
-5.4671078	3.1719999	102.8600006
-5.4671078	3.2207999	103.3099976
-5.4671078	3.2695999	103.6199951
-5.4671078	3.3183999	101.5499954
-5.4671078	3.3671999	100.3099976
-5.4671078	3.4159999	102.4199982
-5.4671078	3.4647999	104.0699997
-5.4671078	3.5135999	102.9499969
-5.4671078	3.5623999	103.5299988
-5.4671078	3.6111999	103.9199982
-5.4671078	3.6599998	102.3600006
-5.4671078	3.7087998	102.7900009
-5.4671078	3.7575998	104.2699966
-5.4671078	3.8063998	106.1399994
-5.4671078	3.8551998	106.6299973
-5.4671078	3.9039998	104.6800003
-5.4671078	3.9527998	101.8099976
-5.4671078	4.0016003	102.4599991
-5.4671078	4.0503998	102.2900009
-5.4671078	4.0991998	102.6800003
-5.4671078	4.1479998	105.9199982
-5.4671078	4.1967998	109.0499954
-5.4671078	4.2455997	109.7099991
-5.4671078	4.2943997	104.7600021
-5.4671078	4.3431997	107.1299973
-5.4671078	4.3919997	105.4399948
-5.4671078	4.4407997	100.4300003
-5.4671078	4.4895997	100.7099991

-5.4671078	4.5383997	104.7900009
-5.4671078	4.5871997	107.3699951
-5.4671078	4.6359997	108.6599960
-5.4671078	4.6847997	114.4599991
-5.4671078	4.7336001	114.2999954
-5.4671078	4.7824001	109.4700012
-5.4671078	4.8312001	106.0299988
-5.4671078	4.8800001	108.4099960
-5.4671078	4.9288001	107.9099960
-5.4671078	4.9776001	105.2900009
-5.4671078	5.0264001	101.0699997
-5.4671078	5.0752001	104.4300003
-5.4671078	5.1240001	105.1699982
-5.4671078	5.1728001	105.4700012
-5.4671078	5.2216001	106.9899979
-5.4671078	5.2704000	102.8399963
-5.4671078	5.3192000	103.3899994
-5.4671078	5.3680000	103.5100021
-5.4671078	5.4168000	102.2399979
-5.4671078	5.4656000	103.8899994
-5.4671078	5.5144000	107.0899963
-5.4671078	5.5632000	106.0299988
-5.4671078	5.6120000	103.2399979
-5.4671078	5.6608000	105.5699997
-5.4671078	5.7096000	100.9799957
-5.4671078	5.7584000	103.6100006
-5.4671078	5.8072000	103.5000000
-5.4671078	5.8559999	104.2900009
-5.4671078	5.9047999	101.5199966
-5.4671078	5.9535999	101.6699982
-5.4671078	6.0023999	104.9099960
-5.4671078	6.0511999	103.0100021
-5.4671078	6.0999999	106.7500000
-5.4671078	6.1487999	102.6999969
-5.4671078	6.1975999	103.7900009
-5.4671078	6.2463999	106.2900009
-5.4671078	6.2951999	105.6599960
-5.4671078	6.3439999	103.6100006
-5.4671078	6.3927999	100.1699982
-5.4671078	6.4415998	100.3199997
-5.4671078	6.4903998	100.2799988
-5.4671078	6.5391998	102.9799957
-5.4671078	6.5879998	105.9399948
-5.4671078	6.6367998	103.5400009
-5.4671078	6.6855998	102.3899994
-5.4671078	6.7343998	105.9799957
-5.4671078	6.7831998	106.8099976
-5.4671078	6.8319998	103.8600006
-5.4671078	6.8807998	104.5599976
-5.4671078	6.9295998	102.1100006
-5.4671078	6.9783998	101.5400009
-5.4671078	7.0271997	104.0400009
-5.4671078	7.0759997	104.0599976
-5.4671078	7.1247997	103.0499954
-5.4671078	7.1735997	103.1100006
-5.4671078	7.2223997	105.1500015
-5.4671078	7.2711997	103.4199982
-5.4671078	7.3199997	103.3600006

-5.4671078	7.3687997	105.7500000
-5.4671078	7.4175997	104.0800018
-5.4671078	7.4663997	103.2799988
-5.4671078	7.5151997	105.4300003
-5.4671078	7.5639997	101.5100021
-5.4671078	7.6127996	99.7799988
-5.4671078	7.6615996	104.2799988
-5.4671078	7.7103996	105.9599991
-5.4671078	7.7591996	104.9300003
-5.4671078	7.8079996	104.2299957
-5.4671078	7.8567996	105.4599991
-5.4671078	7.9056001	102.8099976
-5.4671078	7.9544001	104.5000000
-5.4671078	8.0032005	104.7200012
-5.4671078	8.0520000	101.5100021
-5.4671078	8.1008005	98.6299973
-5.4671078	8.1496000	105.0699997
-5.4671078	8.1984005	106.5699997
-5.4671078	8.2472000	103.0999985
-5.4671078	8.2960005	105.9799957
-5.4671078	8.3448000	105.3399963
-5.4671078	8.3936005	102.8399963
-5.4671078	8.4424000	104.9399948
-5.4671078	8.4912004	104.7500000
-5.4671078	8.5400000	105.6599960
-5.4671078	8.5888004	104.4300003
-5.4671078	8.6375999	101.0800018
-5.4671078	8.6864004	103.6199951
-5.4671078	8.7351999	104.0299988
-5.4671078	8.7840004	103.6699982
-5.4671078	8.8327999	103.5000000
-5.4671078	8.8816004	103.5299988
-5.4671078	8.9303999	101.0899963
-5.4671078	8.9792004	103.2099991
-5.4671078	9.0279999	103.8300018
-5.4671078	9.0768003	103.8199997
-5.4671078	9.1255999	102.8399963
-5.4671078	9.1744003	102.1800003
-5.4671078	9.2231998	103.1500015
-5.4671078	9.2720003	107.1599960
-5.4671078	9.3207998	108.9399948
-5.4671078	9.3696003	106.5299988
-5.4671078	9.4184008	101.7099991
-5.4671078	9.4672003	103.6899948
-5.4671078	9.5160007	102.6800003
-5.4671078	9.5648003	103.3199997
-5.4671078	9.6136007	103.4499969
-5.4671078	9.6624002	103.2299957
-5.4671078	9.7112007	100.7099991
-5.4671078	9.7600002	101.2900009
-5.4671078	9.8088007	101.2699966
-5.4671078	9.8576002	103.1800003
-5.4671078	9.9064007	105.5000000
-5.4671078	9.9552002	104.1800003
-5.4671078	10.0040007	104.8699951
-5.4671078	10.0528002	105.8799973
-5.4671078	10.1016006	103.7299957
-5.4671078	10.1504002	104.1100006

-5.4671078	10.1992006	102.2999954
-5.4671078	10.2480001	101.1500015
-5.4671078	10.2968006	102.9399948
-5.4671078	10.3456001	103.7999954
-5.4671078	10.3944006	100.8300018
-5.4671078	10.4432001	102.6999969
-5.4671078	10.4920006	102.3699951
-5.4671078	10.5408001	103.9499969
-5.4671078	10.5896006	101.3699951
-5.4671078	10.6384001	105.1100006
-5.4671078	10.6872005	106.9599991
-5.4671078	10.7360001	106.9700012
-5.4671078	10.7848005	104.4399948
-5.4671078	10.8336000	104.9399948
-5.4671078	10.8824005	104.4899979
-5.4671078	10.9312000	103.5699997
-5.4671078	10.9800005	105.0999985
-5.4671078	11.0288000	105.3699951
-5.4671078	11.0776005	105.2500000
-5.4671078	11.1264000	104.6199951
-5.4671078	11.1752005	103.4799957
-5.4671078	11.2240000	103.0000000
-5.4671078	11.2728004	102.7799988
-5.4671078	11.3216000	102.5599976
-5.4671078	11.3704004	101.0800018
-5.4671078	11.4191999	100.4199982
-5.4671078	11.4680004	101.3300018
-5.4671078	11.5167999	100.9899979
-5.4671078	11.5656004	101.5100021
-5.4671078	11.6143999	103.3899994
-5.4671078	11.6632004	104.3799973
-5.4671078	11.7119999	104.6999969
-5.4671078	11.7608004	100.8699951
-5.4671078	11.8095999	102.9300003
-5.4671078	11.8584003	104.5000000
-5.4671078	11.9071999	104.5299988
-5.4671078	11.9560003	102.2699966
-5.4671078	12.0047998	99.2799988
-5.4671078	12.0536003	102.5400009
-5.4671078	12.1023998	105.2299957
-5.4671078	12.1512003	103.5800018
-5.4671078	12.1999998	101.0400009
-5.4671078	12.2488003	102.1199951
-5.4671078	12.2975998	101.4300003
-5.4671078	12.3464003	100.4199982
-5.4671078	12.3951998	102.2299957
-5.4671078	12.4440002	104.5999985
-5.4671078	12.4927998	103.5199966
-5.4671078	12.5416002	103.7500000
-5.4671078	12.5904007	103.1800003
-5.4671078	12.6392002	104.3300018
-5.4671078	12.6880007	104.8099976
-5.4671078	12.7368002	105.1999969
-5.4671078	12.7856007	106.9300003
-5.4671078	12.8344002	106.2999954
-5.4671078	12.8832006	102.6699982
-5.4671078	12.9320002	103.1299973
-5.4671078	12.9808006	102.8899994

-5.4671078	13.0296001	100.2299957
-5.4671078	13.0784006	100.5699997
-5.4671078	13.1272001	104.4399948
-5.4671078	13.1760006	105.2200012
-5.4671078	13.2248001	105.1399994
-5.4671078	13.2736006	102.4000015
-5.4671078	13.3224001	101.8799973
-5.4671078	13.3712006	104.4599991
-5.4671078	13.4200001	101.4700012
-5.4671078	13.4688005	100.8399963
-5.4671078	13.5176001	100.5800018
-5.4671078	13.5664005	101.8099976
-5.4671078	13.6152000	103.5899963
-5.4671078	13.6640005	102.7500000
-5.4671078	13.7128000	103.6599960
-5.4671078	13.7616005	104.4499969
-5.4671078	13.8104000	104.0100021
-5.4671078	13.8592005	104.0999985
-5.4671078	13.9080000	105.4099960
-5.4671078	13.9568005	102.4899979
-5.4671078	14.0056000	102.4799957
-5.4671078	14.0544004	102.1999969
-5.4671078	14.1032000	103.0000000
-5.4671078	14.1520004	101.9899979
-5.4671078	14.2007999	103.6399994
-5.4671078	14.2496004	103.3899994
-5.4671078	14.2983999	102.9799957
-5.4671078	14.3472004	102.0999985
-5.4671078	14.3959999	102.3399963
-5.4671078	14.4448004	102.9599991
-5.4671078	14.4935999	102.6999969
-5.4671078	14.5424004	100.8499985
-5.4671078	14.5911999	105.2200012
-5.4671078	14.6400003	103.3699951
-5.4671078	14.6887999	99.2600021
-5.4671078	14.7376003	103.3399963
-5.4671078	14.7863998	106.1299973
-5.4671078	14.8352003	105.8699951
-5.4671078	14.8839998	101.5999985
-5.4671078	14.9328003	101.9099960
-5.4671078	14.9815998	98.8799973
-5.4671078	15.0304003	98.9000015
-5.4609718	0.3416000	101.1500015
-5.4609718	0.3904000	103.8099976
-5.4609718	0.4392000	101.8499985
-5.4609718	0.4880000	101.9899979
-5.4609718	0.5368000	102.8899994
-5.4609718	0.5856000	102.8300018
-5.4609718	0.6344000	105.5899963
-5.4609718	0.6832000	105.5899963
-5.4609718	0.7320000	106.6599960
-5.4609718	0.7808000	105.3699951
-5.4609718	0.8296000	105.2399979
-5.4609718	0.8784000	105.6899948
-5.4609718	0.9272000	107.9199982
-5.4609718	0.9760000	110.0699997
-5.4609718	1.0247999	109.5000000
-5.4609718	1.0735999	106.0999985

-5.4609718	1.1223999	105.0699997
-5.4609718	1.1711999	105.0499954
-5.4609718	1.2200000	106.4099960
-5.4609718	1.2688000	105.0899963
-5.4609718	1.3176000	103.7900009
-5.4609718	1.3664000	107.5499954
-5.4609718	1.4152000	105.2099991
-5.4609718	1.4640000	106.0699997
-5.4609718	1.5128000	107.2600021
-5.4609718	1.5616000	109.5000000
-5.4609718	1.6104000	107.8099976
-5.4609718	1.6592000	102.2799988
-5.4609718	1.7079999	101.5999985
-5.4609718	1.7567999	104.1999969
-5.4609718	1.8055999	103.6699982
-5.4609718	1.8543999	105.2399979
-5.4609718	1.9031999	107.5699997
-5.4609718	1.9519999	107.5400009
-5.4609718	2.0007999	103.5599976
-5.4609718	2.0495999	101.8199997
-5.4609718	2.0983999	103.5899963
-5.4609718	2.1471999	102.7299957
-5.4609718	2.1959999	104.0999985
-5.4609718	2.2447999	105.4399948
-5.4609718	2.2935998	101.7799988
-5.4609718	2.3423998	100.9199982
-5.4609718	2.3912001	104.6199951
-5.4609718	2.4400001	104.6399994
-5.4609718	2.4888000	104.1299973
-5.4609718	2.5376000	103.4899979
-5.4609718	2.5864000	104.7600021
-5.4609718	2.6352000	101.5800018
-5.4609718	2.6840000	105.0499954
-5.4609718	2.7328000	106.2600021
-5.4609718	2.7816000	107.1100006
-5.4609718	2.8304000	104.2999954
-5.4609718	2.8792000	102.3199997
-5.4609718	2.9280000	98.5000000
-5.4609718	2.9768000	104.1899948
-5.4609718	3.0256000	107.7299957
-5.4609718	3.0743999	106.9199982
-5.4609718	3.1231999	106.1699982
-5.4609718	3.1719999	103.0000000
-5.4609718	3.2207999	102.8399963
-5.4609718	3.2695999	103.6199951
-5.4609718	3.3183999	102.1699982
-5.4609718	3.3671999	101.2799988
-5.4609718	3.4159999	101.7900009
-5.4609718	3.4647999	103.5499954
-5.4609718	3.5135999	103.0400009
-5.4609718	3.5623999	103.8099976
-5.4609718	3.6111999	104.3600006
-5.4609718	3.6599998	103.5599976
-5.4609718	3.7087998	104.2399979
-5.4609718	3.7575998	105.1800003
-5.4609718	3.8063998	106.8099976
-5.4609718	3.8551998	107.0899963
-5.4609718	3.9039998	104.9700012

-5.4609718	3.9527998	101.9399948
-5.4609718	4.0016003	102.3300018
-5.4609718	4.0503998	103.4000015
-5.4609718	4.0991998	101.6599960
-5.4609718	4.1479998	105.7999954
-5.4609718	4.1967998	109.0599976
-5.4609718	4.2455997	109.2699966
-5.4609718	4.2943997	103.0999985
-5.4609718	4.3431997	107.3499985
-5.4609718	4.3919997	105.3899994
-5.4609718	4.4407997	101.2099991
-5.4609718	4.4895997	101.6100006
-5.4609718	4.5383997	104.2999954
-5.4609718	4.5871997	107.5999985
-5.4609718	4.6359997	109.6899948
-5.4609718	4.6847997	114.1599960
-5.4609718	4.7336001	113.5599976
-5.4609718	4.7824001	108.3199997
-5.4609718	4.8312001	106.1100006
-5.4609718	4.8800001	109.2799988
-5.4609718	4.9288001	108.5000000
-5.4609718	4.9776001	105.0800018
-5.4609718	5.0264001	100.9499969
-5.4609718	5.0752001	104.5599976
-5.4609718	5.1240001	105.4199982
-5.4609718	5.1728001	105.0199966
-5.4609718	5.2216001	106.0100021
-5.4609718	5.2704000	99.8600006
-5.4609718	5.3192000	102.9799957
-5.4609718	5.3680000	103.7699966
-5.4609718	5.4168000	102.1899948
-5.4609718	5.4656000	104.4700012
-5.4609718	5.5144000	107.3300018
-5.4609718	5.5632000	106.5299988
-5.4609718	5.6120000	104.6500015
-5.4609718	5.6608000	105.8199997
-5.4609718	5.7096000	101.1599960
-5.4609718	5.7584000	103.2299957
-5.4609718	5.8072000	102.6699982
-5.4609718	5.8559999	103.6399994
-5.4609718	5.9047999	101.4700012
-5.4609718	5.9535999	102.1999969
-5.4609718	6.0023999	104.9899979
-5.4609718	6.0511999	102.7799988
-5.4609718	6.0999999	106.9499969
-5.4609718	6.1487999	103.0299988
-5.4609718	6.1975999	102.6399994
-5.4609718	6.2463999	105.5400009
-5.4609718	6.2951999	105.8199997
-5.4609718	6.3439999	103.6899948
-5.4609718	6.3927999	101.1800003
-5.4609718	6.4415998	100.7200012
-5.4609718	6.4903998	101.0400009
-5.4609718	6.5391998	102.5199966
-5.4609718	6.5879998	105.6399994
-5.4609718	6.6367998	103.7900009
-5.4609718	6.6855998	102.6599960
-5.4609718	6.7343998	105.9799957

-5.4609718	6.7831998	107.3199997
-5.4609718	6.8319998	104.7299957
-5.4609718	6.8807998	105.3199997
-5.4609718	6.9295998	102.9799957
-5.4609718	6.9783998	102.5000000
-5.4609718	7.0271997	103.5400009
-5.4609718	7.0759997	104.5100021
-5.4609718	7.1247997	103.3099976
-5.4609718	7.1735997	103.0199966
-5.4609718	7.2223997	105.4399948
-5.4609718	7.2711997	104.5599976
-5.4609718	7.3199997	104.7900009
-5.4609718	7.3687997	105.5899963
-5.4609718	7.4175997	103.2900009
-5.4609718	7.4663997	102.6599960
-5.4609718	7.5151997	105.3899994
-5.4609718	7.5639997	102.7900009
-5.4609718	7.6127996	100.9700012
-5.4609718	7.6615996	104.0499954
-5.4609718	7.7103996	105.8099976
-5.4609718	7.7591996	104.8699951
-5.4609718	7.8079996	104.6399994
-5.4609718	7.8567996	105.7999954
-5.4609718	7.9056001	103.3600006
-5.4609718	7.9544001	105.3199997
-5.4609718	8.0032005	105.5499954
-5.4609718	8.0520000	102.1500015
-5.4609718	8.1008005	99.8899994
-5.4609718	8.1496000	105.7600021
-5.4609718	8.1984005	107.1100006
-5.4609718	8.2472000	104.2500000
-5.4609718	8.2960005	106.0299988
-5.4609718	8.3448000	105.3600006
-5.4609718	8.3936005	101.6899948
-5.4609718	8.4424000	103.6699982
-5.4609718	8.4912004	103.9899979
-5.4609718	8.5400000	104.9099960
-5.4609718	8.5888004	103.7500000
-5.4609718	8.6375999	101.2799988
-5.4609718	8.6864004	103.9499969
-5.4609718	8.7351999	104.1599960
-5.4609718	8.7840004	103.7900009
-5.4609718	8.8327999	104.2999954
-5.4609718	8.8816004	104.2699966
-5.4609718	8.9303999	101.9399948
-5.4609718	8.9792004	104.2699966
-5.4609718	9.0279999	104.0199966
-5.4609718	9.0768003	103.0699997
-5.4609718	9.1255999	102.7200012
-5.4609718	9.1744003	102.4000015
-5.4609718	9.2231998	104.0199966
-5.4609718	9.2720003	107.7200012
-5.4609718	9.3207998	109.2600021
-5.4609718	9.3696003	106.6399994
-5.4609718	9.4184008	101.2099991
-5.4609718	9.4672003	103.5999985
-5.4609718	9.5160007	103.4899979
-5.4609718	9.5648003	104.0000000

-5.4609718	9.6136007	103.6399994
-5.4609718	9.6624002	102.5100021
-5.4609718	9.7112007	101.6299973
-5.4609718	9.7600002	102.3600006
-5.4609718	9.8088007	102.2500000
-5.4609718	9.8576002	103.6899948
-5.4609718	9.9064007	105.7900009
-5.4609718	9.9552002	103.4799957
-5.4609718	10.0040007	104.2500000
-5.4609718	10.0528002	105.6299973
-5.4609718	10.1016006	103.6399994
-5.4609718	10.1504002	104.2200012
-5.4609718	10.1992006	102.6399994
-5.4609718	10.2480001	101.3799973
-5.4609718	10.2968006	102.5199966
-5.4609718	10.3456001	104.1699982
-5.4609718	10.3944006	101.9799957
-5.4609718	10.4432001	102.3699951
-5.4609718	10.4920006	100.7999954
-5.4609718	10.5408001	104.5699997
-5.4609718	10.5896006	103.1500015
-5.4609718	10.6384001	106.0199966
-5.4609718	10.6872005	107.9099960
-5.4609718	10.7360001	107.1299973
-5.4609718	10.7848005	105.0299988
-5.4609718	10.8336000	105.6500015
-5.4609718	10.8824005	105.3699951
-5.4609718	10.9312000	103.5000000
-5.4609718	10.9800005	105.6699982
-5.4609718	11.0288000	105.9000015
-5.4609718	11.0776005	104.6500015
-5.4609718	11.1264000	105.1800003
-5.4609718	11.1752005	104.0400009
-5.4609718	11.2240000	102.7500000
-5.4609718	11.2728004	102.8600006
-5.4609718	11.3216000	103.1699982
-5.4609718	11.3704004	101.1500015
-5.4609718	11.4191999	99.9399948
-5.4609718	11.4680004	101.4799957
-5.4609718	11.5167999	101.7600021
-5.4609718	11.5656004	102.0800018
-5.4609718	11.6143999	103.1199951
-5.4609718	11.6632004	104.4199982
-5.4609718	11.7119999	105.3300018
-5.4609718	11.7608004	101.2699966
-5.4609718	11.8095999	103.3399963
-5.4609718	11.8584003	103.3799973
-5.4609718	11.9071999	102.9499969
-5.4609718	11.9560003	101.0000000
-5.4609718	12.0047998	98.5000000
-5.4609718	12.0536003	102.6800003
-5.4609718	12.1023998	104.5899963
-5.4609718	12.1512003	103.4799957
-5.4609718	12.1999998	101.9199982
-5.4609718	12.2488003	102.7900009
-5.4609718	12.2975998	101.8199997
-5.4609718	12.3464003	100.8699951
-5.4609718	12.3951998	101.0699997

-5.4609718	12.4440002	103.3799973
-5.4609718	12.4927998	102.7999954
-5.4609718	12.5416002	103.5000000
-5.4609718	12.5904007	103.6199951
-5.4609718	12.6392002	105.0999985
-5.4609718	12.6880007	104.0899963
-5.4609718	12.7368002	104.7099991
-5.4609718	12.7856007	106.9099960
-5.4609718	12.8344002	106.7699966
-5.4609718	12.8832006	102.8199997
-5.4609718	12.9320002	103.0800018
-5.4609718	12.9808006	103.8300018
-5.4609718	13.0296001	100.4300003
-5.4609718	13.0784006	99.0699997
-5.4609718	13.1272001	104.1100006
-5.4609718	13.1760006	105.7900009
-5.4609718	13.2248001	105.4599991
-5.4609718	13.2736006	102.3300018
-5.4609718	13.3224001	102.4000015
-5.4609718	13.3712006	105.1100006
-5.4609718	13.4200001	102.2600021
-5.4609718	13.4688005	101.1800003
-5.4609718	13.5176001	101.2600021
-5.4609718	13.5664005	102.2699966
-5.4609718	13.6152000	103.6699982
-5.4609718	13.6640005	103.5899963
-5.4609718	13.7128000	103.2600021
-5.4609718	13.7616005	103.8799973
-5.4609718	13.8104000	104.3099976
-5.4609718	13.8592005	104.9499969
-5.4609718	13.9080000	105.7999954
-5.4609718	13.9568005	102.3699951
-5.4609718	14.0056000	102.8600006
-5.4609718	14.0544004	102.5199966
-5.4609718	14.1032000	103.4099960
-5.4609718	14.1520004	102.4000015
-5.4609718	14.2007999	103.4300003
-5.4609718	14.2496004	102.9899979
-5.4609718	14.2983999	103.4499969
-5.4609718	14.3472004	103.0599976
-5.4609718	14.3959999	103.1999969
-5.4609718	14.4448004	103.7200012
-5.4609718	14.4935999	103.5599976
-5.4609718	14.5424004	102.3099976
-5.4609718	14.5911999	105.3399963
-5.4609718	14.6400003	102.9799957
-5.4609718	14.6887999	98.2099991
-5.4609718	14.7376003	102.2399979
-5.4609718	14.7863998	106.1999969
-5.4609718	14.8352003	106.3300018
-5.4609718	14.8839998	102.0499954
-5.4609718	14.9328003	101.4199982
-5.4609718	14.9815998	98.5100021
-5.4609718	15.0304003	100.2299957
-5.4548359	0.3416000	102.8399963
-5.4548359	0.3904000	104.6800003
-5.4548359	0.4392000	102.5699997
-5.4548359	0.4880000	101.8099976

-5.4548359	0.5368000	103.3899994
-5.4548359	0.5856000	103.0599976
-5.4548359	0.6344000	105.5800018
-5.4548359	0.6832000	105.8300018
-5.4548359	0.7320000	107.1999969
-5.4548359	0.7808000	105.9300003
-5.4548359	0.8296000	105.0400009
-5.4548359	0.8784000	105.3300018
-5.4548359	0.9272000	107.5199966
-5.4548359	0.9760000	109.4399948
-5.4548359	1.0247999	109.1500015
-5.4548359	1.0735999	106.0999985
-5.4548359	1.1223999	105.9599991
-5.4548359	1.1711999	105.9700012
-5.4548359	1.2200000	106.2200012
-5.4548359	1.2688000	104.4300003
-5.4548359	1.3176000	102.5299988
-5.4548359	1.3664000	107.4799957
-5.4548359	1.4152000	106.6299973
-5.4548359	1.4640000	106.5899963
-5.4548359	1.5128000	105.6199951
-5.4548359	1.5616000	108.5299988
-5.4548359	1.6104000	108.0400009
-5.4548359	1.6592000	103.7399979
-5.4548359	1.7079999	101.1699982
-5.4548359	1.7567999	103.4099960
-5.4548359	1.8055999	103.6999969
-5.4548359	1.8543999	105.5299988
-5.4548359	1.9031999	107.3899994
-5.4548359	1.9519999	107.2500000
-5.4548359	2.0007999	102.5800018
-5.4548359	2.0495999	101.0899963
-5.4548359	2.0983999	103.3199997
-5.4548359	2.1471999	103.0199966
-5.4548359	2.1959999	104.5999985
-5.4548359	2.2447999	105.7200012
-5.4548359	2.2935998	102.8499985
-5.4548359	2.3423998	101.9300003
-5.4548359	2.3912001	105.3799973
-5.4548359	2.4400001	105.5499954
-5.4548359	2.4888000	105.1999969
-5.4548359	2.5376000	105.0299988
-5.4548359	2.5864000	104.6999969
-5.4548359	2.6352000	102.3499985
-5.4548359	2.6840000	104.4499969
-5.4548359	2.7328000	106.7999954
-5.4548359	2.7816000	106.5800018
-5.4548359	2.8304000	103.4099960
-5.4548359	2.8792000	103.3300018
-5.4548359	2.9280000	99.9000015
-5.4548359	2.9768000	104.8300018
-5.4548359	3.0256000	107.7200012
-5.4548359	3.0743999	106.4700012
-5.4548359	3.1231999	106.2799988
-5.4548359	3.1719999	104.1299973
-5.4548359	3.2207999	103.5400009
-5.4548359	3.2695999	103.8799973
-5.4548359	3.3183999	102.6199951

-5.4548359	3.3671999	101.8099976
-5.4548359	3.4159999	101.3600006
-5.4548359	3.4647999	103.7399979
-5.4548359	3.5135999	103.0100021
-5.4548359	3.5623999	103.9499969
-5.4548359	3.6111999	104.8799973
-5.4548359	3.6599998	104.8799973
-5.4548359	3.7087998	104.5800018
-5.4548359	3.7575998	105.3399963
-5.4548359	3.8063998	106.7999954
-5.4548359	3.8551998	106.9399948
-5.4548359	3.9039998	105.0400009
-5.4548359	3.9527998	101.9700012
-5.4548359	4.0016003	101.6699982
-5.4548359	4.0503998	102.6699982
-5.4548359	4.0991998	100.5699997
-5.4548359	4.1479998	105.2900009
-5.4548359	4.1967998	108.4399948
-5.4548359	4.2455997	108.2099991
-5.4548359	4.2943997	102.8399963
-5.4548359	4.3431997	107.6800003
-5.4548359	4.3919997	105.3300018
-5.4548359	4.4407997	101.8499985
-5.4548359	4.4895997	102.4799957
-5.4548359	4.5383997	104.6800003
-5.4548359	4.5871997	108.2299957
-5.4548359	4.6359997	109.9599991
-5.4548359	4.6847997	113.5999985
-5.4548359	4.7336001	112.4599991
-5.4548359	4.7824001	106.9700012
-5.4548359	4.8312001	106.0499954
-5.4548359	4.8800001	109.5400009
-5.4548359	4.9288001	108.4899979
-5.4548359	4.9776001	104.7399979
-5.4548359	5.0264001	101.9599991
-5.4548359	5.0752001	104.5800018
-5.4548359	5.1240001	105.7699966
-5.4548359	5.1728001	104.9199982
-5.4548359	5.2216001	105.3600006
-5.4548359	5.2704000	97.6599960
-5.4548359	5.3192000	102.5899963
-5.4548359	5.3680000	103.5400009
-5.4548359	5.4168000	102.0299988
-5.4548359	5.4656000	105.1500015
-5.4548359	5.5144000	107.5599976
-5.4548359	5.5632000	106.7799988
-5.4548359	5.6120000	105.4099960
-5.4548359	5.6608000	105.5800018
-5.4548359	5.7096000	102.0699997
-5.4548359	5.7584000	103.6899948
-5.4548359	5.8072000	102.3300018
-5.4548359	5.8559999	103.3199997
-5.4548359	5.9047999	101.7200012
-5.4548359	5.9535999	102.1599960
-5.4548359	6.0023999	103.9199982
-5.4548359	6.0511999	101.8499985
-5.4548359	6.0999999	107.2999954
-5.4548359	6.1487999	104.0899963

-5.4548359	6.1975999	102.5499954
-5.4548359	6.2463999	105.0699997
-5.4548359	6.2951999	105.6999969
-5.4548359	6.3439999	103.6599960
-5.4548359	6.3927999	102.2200012
-5.4548359	6.4415998	101.1999969
-5.4548359	6.4903998	102.5000000
-5.4548359	6.5391998	102.6899948
-5.4548359	6.5879998	105.3799973
-5.4548359	6.6367998	104.0299988
-5.4548359	6.6855998	102.8399963
-5.4548359	6.7343998	105.7099991
-5.4548359	6.7831998	107.4199982
-5.4548359	6.8319998	105.7500000
-5.4548359	6.8807998	105.6299973
-5.4548359	6.9295998	103.5100021
-5.4548359	6.9783998	103.6899948
-5.4548359	7.0271997	103.8099976
-5.4548359	7.0759997	104.7999954
-5.4548359	7.1247997	103.5000000
-5.4548359	7.1735997	103.1800003
-5.4548359	7.2223997	105.7299957
-5.4548359	7.2711997	105.4399948
-5.4548359	7.3199997	106.0599976
-5.4548359	7.3687997	105.2799988
-5.4548359	7.4175997	102.1699982
-5.4548359	7.4663997	102.6199951
-5.4548359	7.5151997	105.4599991
-5.4548359	7.5639997	103.7299957
-5.4548359	7.6127996	102.2299957
-5.4548359	7.6615996	103.7500000
-5.4548359	7.7103996	105.1500015
-5.4548359	7.7591996	104.1800003
-5.4548359	7.8079996	104.4499969
-5.4548359	7.8567996	106.0599976
-5.4548359	7.9056001	104.1999969
-5.4548359	7.9544001	105.9099960
-5.4548359	8.0032005	106.0299988
-5.4548359	8.0520000	102.8799973
-5.4548359	8.1008005	101.6199951
-5.4548359	8.1496000	106.1699982
-5.4548359	8.1984005	107.0000000
-5.4548359	8.2472000	104.8499985
-5.4548359	8.2960005	105.9099960
-5.4548359	8.3448000	104.9499969
-5.4548359	8.3936005	101.9799957
-5.4548359	8.4424000	103.3799973
-5.4548359	8.4912004	103.9499969
-5.4548359	8.5400000	104.4000015
-5.4548359	8.5888004	103.2099991
-5.4548359	8.6375999	100.6899948
-5.4548359	8.6864004	103.7900009
-5.4548359	8.7351999	103.8799973
-5.4548359	8.7840004	103.4899979
-5.4548359	8.8327999	104.7200012
-5.4548359	8.8816004	104.4300003
-5.4548359	8.9303999	102.7999954
-5.4548359	8.9792004	105.2099991

-5.4548359	9.0279999	103.9499969
-5.4548359	9.0768003	102.8399963
-5.4548359	9.1255999	102.0699997
-5.4548359	9.1744003	103.0699997
-5.4548359	9.2231998	104.5800018
-5.4548359	9.2720003	107.7900009
-5.4548359	9.3207998	109.1100006
-5.4548359	9.3696003	106.2600021
-5.4548359	9.4184008	100.1699982
-5.4548359	9.4672003	103.1399994
-5.4548359	9.5160007	103.1599960
-5.4548359	9.5648003	104.2799988
-5.4548359	9.6136007	104.0999985
-5.4548359	9.6624002	102.2999954
-5.4548359	9.7112007	103.0299988
-5.4548359	9.7600002	103.8099976
-5.4548359	9.8088007	103.9099960
-5.4548359	9.8576002	105.0000000
-5.4548359	9.9064007	105.7399979
-5.4548359	9.9552002	102.0199966
-5.4548359	10.0040007	103.1899948
-5.4548359	10.0528002	105.4399948
-5.4548359	10.1016006	104.1800003
-5.4548359	10.1504002	104.3199997
-5.4548359	10.1992006	102.7999954
-5.4548359	10.2480001	101.1699982
-5.4548359	10.2968006	102.1500015
-5.4548359	10.3456001	104.7200012
-5.4548359	10.3944006	103.3300018
-5.4548359	10.4432001	102.5499954
-5.4548359	10.4920006	99.9199982
-5.4548359	10.5408001	105.3399963
-5.4548359	10.5896006	104.9099960
-5.4548359	10.6384001	106.2600021
-5.4548359	10.6872005	107.4799957
-5.4548359	10.7360001	106.6500015
-5.4548359	10.7848005	105.2500000
-5.4548359	10.8336000	106.1199951
-5.4548359	10.8824005	105.8799973
-5.4548359	10.9312000	103.5000000
-5.4548359	10.9800005	106.1100006
-5.4548359	11.0288000	104.9599991
-5.4548359	11.0776005	103.5199966
-5.4548359	11.1264000	105.7699966
-5.4548359	11.1752005	104.8799973
-5.4548359	11.2240000	102.8300018
-5.4548359	11.2728004	103.0599976
-5.4548359	11.3216000	103.9300003
-5.4548359	11.3704004	101.3499985
-5.4548359	11.4191999	99.3499985
-5.4548359	11.4680004	101.9199982
-5.4548359	11.5167999	102.8600006
-5.4548359	11.5656004	102.7600021
-5.4548359	11.6143999	103.1699982
-5.4548359	11.6632004	104.2600021
-5.4548359	11.7119999	104.8499985
-5.4548359	11.7608004	102.2699966
-5.4548359	11.8095999	103.9899979

-5.4548359	11.8584003	102.6199951
-5.4548359	11.9071999	101.4199982
-5.4548359	11.9560003	99.6800003
-5.4548359	12.0047998	97.8799973
-5.4548359	12.0536003	102.9799957
-5.4548359	12.1023998	103.9300003
-5.4548359	12.1512003	103.2399979
-5.4548359	12.1999998	102.6100006
-5.4548359	12.2488003	103.2099991
-5.4548359	12.2975998	101.7200012
-5.4548359	12.3464003	101.1899948
-5.4548359	12.3951998	100.9199982
-5.4548359	12.4440002	102.5299988
-5.4548359	12.4927998	101.7900009
-5.4548359	12.5416002	103.5100021
-5.4548359	12.5904007	104.2399979
-5.4548359	12.6392002	105.2799988
-5.4548359	12.6880007	102.6299973
-5.4548359	12.7368002	103.7900009
-5.4548359	12.7856007	106.2500000
-5.4548359	12.8344002	106.1299973
-5.4548359	12.8832006	102.5499954
-5.4548359	12.9320002	102.7500000
-5.4548359	12.9808006	104.1599960
-5.4548359	13.0296001	100.2699966
-5.4548359	13.0784006	98.2699966
-5.4548359	13.1272001	104.0999985
-5.4548359	13.1760006	106.0000000
-5.4548359	13.2248001	105.3300018
-5.4548359	13.2736006	102.3099976
-5.4548359	13.3224001	102.6299973
-5.4548359	13.3712006	105.1500015
-5.4548359	13.4200001	101.9599991
-5.4548359	13.4688005	100.6299973
-5.4548359	13.5176001	101.4300003
-5.4548359	13.5664005	102.0800018
-5.4548359	13.6152000	103.4700012
-5.4548359	13.6640005	104.2299957
-5.4548359	13.7128000	103.1800003
-5.4548359	13.7616005	103.4599991
-5.4548359	13.8104000	104.5499954
-5.4548359	13.8592005	105.4499969
-5.4548359	13.9080000	106.3399963
-5.4548359	13.9568005	102.0400009
-5.4548359	14.0056000	102.9700012
-5.4548359	14.0544004	102.4799957
-5.4548359	14.1032000	103.6999969
-5.4548359	14.1520004	102.2799988
-5.4548359	14.2007999	102.9700012
-5.4548359	14.2496004	102.3799973
-5.4548359	14.2983999	103.3699951
-5.4548359	14.3472004	103.6100006
-5.4548359	14.3959999	103.4899979
-5.4548359	14.4448004	104.1399994
-5.4548359	14.4935999	104.4199982
-5.4548359	14.5424004	104.2600021
-5.4548359	14.5911999	105.2999954
-5.4548359	14.6400003	102.4000015

-5.4548359	14.6887999	97.6299973
-5.4548359	14.7376003	101.8600006
-5.4548359	14.7863998	106.0400009
-5.4548359	14.8352003	106.2699966
-5.4548359	14.8839998	102.0299988
-5.4548359	14.9328003	100.8600006
-5.4548359	14.9815998	97.8499985
-5.4548359	15.0304003	101.1800003
-5.4487000	0.3416000	105.0699997
-5.4487000	0.3904000	106.0599976
-5.4487000	0.4392000	103.7799988
-5.4487000	0.4880000	102.9399948
-5.4487000	0.5368000	104.1899948
-5.4487000	0.5856000	103.6399994
-5.4487000	0.6344000	105.8499985
-5.4487000	0.6832000	106.3399963
-5.4487000	0.7320000	107.7200012
-5.4487000	0.7808000	105.5299988
-5.4487000	0.8296000	104.8499985
-5.4487000	0.8784000	105.1999969
-5.4487000	0.9272000	107.0400009
-5.4487000	0.9760000	108.3399963
-5.4487000	1.0247999	108.1599960
-5.4487000	1.0735999	105.6299973
-5.4487000	1.1223999	106.6500015
-5.4487000	1.1711999	106.7900009
-5.4487000	1.2200000	106.0899963
-5.4487000	1.2688000	104.1199951
-5.4487000	1.3176000	101.7500000
-5.4487000	1.3664000	107.1999969
-5.4487000	1.4152000	107.2999954
-5.4487000	1.4640000	106.3399963
-5.4487000	1.5128000	104.6399994
-5.4487000	1.5616000	107.1199951
-5.4487000	1.6104000	107.6500015
-5.4487000	1.6592000	104.9000015
-5.4487000	1.7079999	101.9599991
-5.4487000	1.7567999	102.7399979
-5.4487000	1.8055999	103.6100006
-5.4487000	1.8543999	105.6699982
-5.4487000	1.9031999	107.2799988
-5.4487000	1.9519999	107.1199951
-5.4487000	2.0007999	102.1100006
-5.4487000	2.0495999	101.7900009
-5.4487000	2.0983999	104.0899963
-5.4487000	2.1471999	104.1899948
-5.4487000	2.1959999	105.7900009
-5.4487000	2.2447999	106.5100021
-5.4487000	2.2935998	104.0400009
-5.4487000	2.3423998	102.8199997
-5.4487000	2.3912001	106.0899963
-5.4487000	2.4400001	105.9799957
-5.4487000	2.4888000	106.7999954
-5.4487000	2.5376000	106.7999954
-5.4487000	2.5864000	104.4799957
-5.4487000	2.6352000	102.8300018
-5.4487000	2.6840000	103.9399948
-5.4487000	2.7328000	107.2399979

-5.4487000	2.7816000	106.6599960
-5.4487000	2.8304000	103.5100021
-5.4487000	2.8792000	104.0800018
-5.4487000	2.9280000	101.2500000
-5.4487000	2.9768000	105.5400009
-5.4487000	3.0256000	107.5800018
-5.4487000	3.0743999	106.7900009
-5.4487000	3.1231999	106.7399979
-5.4487000	3.1719999	105.4000015
-5.4487000	3.2207999	104.6399994
-5.4487000	3.2695999	104.5899963
-5.4487000	3.3183999	103.0299988
-5.4487000	3.3671999	102.0699997
-5.4487000	3.4159999	101.7799988
-5.4487000	3.4647999	103.5299988
-5.4487000	3.5135999	102.2699966
-5.4487000	3.5623999	103.9000015
-5.4487000	3.6111999	105.1899948
-5.4487000	3.6599998	105.8399963
-5.4487000	3.7087998	104.3300018
-5.4487000	3.7575998	104.9700012
-5.4487000	3.8063998	106.2299957
-5.4487000	3.8551998	106.0000000
-5.4487000	3.9039998	104.9300003
-5.4487000	3.9527998	102.3499985
-5.4487000	4.0016003	101.8399963
-5.4487000	4.0503998	102.5000000
-5.4487000	4.0991998	99.6999969
-5.4487000	4.1479998	105.0699997
-5.4487000	4.1967998	108.2600021
-5.4487000	4.2455997	107.2600021
-5.4487000	4.2943997	102.0499954
-5.4487000	4.3431997	107.2099991
-5.4487000	4.3919997	104.6800003
-5.4487000	4.4407997	101.9799957
-5.4487000	4.4895997	103.2399979
-5.4487000	4.5383997	105.4599991
-5.4487000	4.5871997	108.1800003
-5.4487000	4.6359997	109.7399979
-5.4487000	4.6847997	113.0299988
-5.4487000	4.7336001	111.5000000
-5.4487000	4.7824001	105.4899979
-5.4487000	4.8312001	106.2399979
-5.4487000	4.8800001	109.4199982
-5.4487000	4.9288001	108.0299988
-5.4487000	4.9776001	103.7099991
-5.4487000	5.0264001	102.5699997
-5.4487000	5.0752001	104.4899979
-5.4487000	5.1240001	106.1299973
-5.4487000	5.1728001	105.0699997
-5.4487000	5.2216001	104.9099960
-5.4487000	5.2704000	96.3199997
-5.4487000	5.3192000	102.5100021
-5.4487000	5.3680000	103.2799988
-5.4487000	5.4168000	102.5599976
-5.4487000	5.4656000	105.9099960
-5.4487000	5.5144000	107.5999985
-5.4487000	5.5632000	106.8199997

-5.4487000	5.6120000	105.9799957
-5.4487000	5.6608000	105.2299957
-5.4487000	5.7096000	102.4399948
-5.4487000	5.7584000	104.3399963
-5.4487000	5.8072000	102.5699997
-5.4487000	5.8559999	103.6199951
-5.4487000	5.9047999	103.1399994
-5.4487000	5.9535999	102.3799973
-5.4487000	6.0023999	102.3199997
-5.4487000	6.0511999	102.3499985
-5.4487000	6.0999999	107.5599976
-5.4487000	6.1487999	104.9799957
-5.4487000	6.1975999	102.9799957
-5.4487000	6.2463999	105.0000000
-5.4487000	6.2951999	105.9399948
-5.4487000	6.3439999	104.4199982
-5.4487000	6.3927999	103.5199966
-5.4487000	6.4415998	102.1899948
-5.4487000	6.4903998	103.2799988
-5.4487000	6.5391998	102.9799957
-5.4487000	6.5879998	105.2399979
-5.4487000	6.6367998	104.4899979
-5.4487000	6.6855998	103.4799957
-5.4487000	6.7343998	105.0299988
-5.4487000	6.7831998	106.5899963
-5.4487000	6.8319998	105.9799957
-5.4487000	6.8807998	105.5800018
-5.4487000	6.9295998	103.6299973
-5.4487000	6.9783998	103.8399963
-5.4487000	7.0271997	103.5599976
-5.4487000	7.0759997	104.6100006
-5.4487000	7.1247997	103.7500000
-5.4487000	7.1735997	103.3799973
-5.4487000	7.2223997	106.1299973
-5.4487000	7.2711997	106.3699951
-5.4487000	7.3199997	106.6100006
-5.4487000	7.3687997	104.6399994
-5.4487000	7.4175997	101.1399994
-5.4487000	7.4663997	102.5599976
-5.4487000	7.5151997	105.2299957
-5.4487000	7.5639997	104.2099991
-5.4487000	7.6127996	103.1199951
-5.4487000	7.6615996	103.6500015
-5.4487000	7.7103996	105.3099976
-5.4487000	7.7591996	104.6699982
-5.4487000	7.8079996	104.4199982
-5.4487000	7.8567996	106.0899963
-5.4487000	7.9056001	104.8099976
-5.4487000	7.9544001	105.8899994
-5.4487000	8.0032005	105.3699951
-5.4487000	8.0520000	103.2999954
-5.4487000	8.1008005	103.1399994
-5.4487000	8.1496000	106.2900009
-5.4487000	8.1984005	106.5100021
-5.4487000	8.2472000	105.1699982
-5.4487000	8.2960005	105.5899963
-5.4487000	8.3448000	104.0800018
-5.4487000	8.3936005	101.6999969

-5.4487000	8.4424000	102.7699966
-5.4487000	8.4912004	103.8499985
-5.4487000	8.5400000	103.7500000
-5.4487000	8.5888004	102.8399963
-5.4487000	8.6375999	100.9399948
-5.4487000	8.6864004	103.7099991
-5.4487000	8.7351999	103.5400009
-5.4487000	8.7840004	102.8199997
-5.4487000	8.8327999	104.6500015
-5.4487000	8.8816004	104.5400009
-5.4487000	8.9303999	103.8300018
-5.4487000	8.9792004	105.9799957
-5.4487000	9.0279999	103.5999985
-5.4487000	9.0768003	102.4899979
-5.4487000	9.1255999	101.4499969
-5.4487000	9.1744003	103.6800003
-5.4487000	9.2231998	105.0800018
-5.4487000	9.2720003	107.8600006
-5.4487000	9.3207998	108.8600006
-5.4487000	9.3696003	106.1699982
-5.4487000	9.4184008	100.2299957
-5.4487000	9.4672003	103.0199966
-5.4487000	9.5160007	102.5699997
-5.4487000	9.5648003	104.1399994
-5.4487000	9.6136007	103.7900009
-5.4487000	9.6624002	101.5400009
-5.4487000	9.7112007	103.7099991
-5.4487000	9.7600002	105.1399994
-5.4487000	9.8088007	105.0999985
-5.4487000	9.8576002	105.6399994
-5.4487000	9.9064007	105.4599991
-5.4487000	9.9552002	100.4799957
-5.4487000	10.0040007	102.3300018
-5.4487000	10.0528002	104.9499969
-5.4487000	10.1016006	104.1500015
-5.4487000	10.1504002	104.2799988
-5.4487000	10.1992006	102.6299973
-5.4487000	10.2480001	100.7699966
-5.4487000	10.2968006	102.2500000
-5.4487000	10.3456001	104.6800003
-5.4487000	10.3944006	104.0699997
-5.4487000	10.4432001	102.3499985
-5.4487000	10.4920006	98.6899948
-5.4487000	10.5408001	105.2799988
-5.4487000	10.5896006	106.4199982
-5.4487000	10.6384001	106.4000015
-5.4487000	10.6872005	106.6699982
-5.4487000	10.7360001	105.4399948
-5.4487000	10.7848005	105.9099960
-5.4487000	10.8336000	106.5400009
-5.4487000	10.8824005	106.2099991
-5.4487000	10.9312000	104.2399979
-5.4487000	10.9800005	106.3600006
-5.4487000	11.0288000	104.4399948
-5.4487000	11.0776005	102.4199982
-5.4487000	11.1264000	105.9300003
-5.4487000	11.1752005	105.3899994
-5.4487000	11.2240000	102.8899994

-5.4487000	11.2728004	102.9199982
-5.4487000	11.3216000	104.3399963
-5.4487000	11.3704004	101.7200012
-5.4487000	11.4191999	99.3300018
-5.4487000	11.4680004	102.7200012
-5.4487000	11.5167999	103.9899979
-5.4487000	11.5656004	104.0499954
-5.4487000	11.6143999	103.9599991
-5.4487000	11.6632004	104.0199966
-5.4487000	11.7119999	103.9399948
-5.4487000	11.7608004	102.6500015
-5.4487000	11.8095999	104.6800003
-5.4487000	11.8584003	103.0599976
-5.4487000	11.9071999	100.9399948
-5.4487000	11.9560003	99.6299973
-5.4487000	12.0047998	97.6100006
-5.4487000	12.0536003	103.4899979
-5.4487000	12.1023998	103.4300003
-5.4487000	12.1512003	102.8600006
-5.4487000	12.1999998	102.8899994
-5.4487000	12.2488003	103.1800003
-5.4487000	12.2975998	101.9300003
-5.4487000	12.3464003	101.5800018
-5.4487000	12.3951998	101.1599960
-5.4487000	12.4440002	101.4300003
-5.4487000	12.4927998	100.2699966
-5.4487000	12.5416002	103.3499985
-5.4487000	12.5904007	105.3300018
-5.4487000	12.6392002	105.7900009
-5.4487000	12.6880007	101.6399994
-5.4487000	12.7368002	103.1299973
-5.4487000	12.7856007	105.9099960
-5.4487000	12.8344002	105.4899979
-5.4487000	12.8832006	102.7900009
-5.4487000	12.9320002	102.7200012
-5.4487000	12.9808006	104.5400009
-5.4487000	13.0296001	100.4899979
-5.4487000	13.0784006	98.3799973
-5.4487000	13.1272001	104.0299988
-5.4487000	13.1760006	105.9300003
-5.4487000	13.2248001	105.4199982
-5.4487000	13.2736006	102.9099960
-5.4487000	13.3224001	102.8699951
-5.4487000	13.3712006	105.0400009
-5.4487000	13.4200001	101.5499954
-5.4487000	13.4688005	100.7699966
-5.4487000	13.5176001	102.2299957
-5.4487000	13.5664005	102.2299957
-5.4487000	13.6152000	103.6399994
-5.4487000	13.6640005	105.2399979
-5.4487000	13.7128000	103.7699966
-5.4487000	13.7616005	103.2299957
-5.4487000	13.8104000	104.4199982
-5.4487000	13.8592005	105.4399948
-5.4487000	13.9080000	106.8600006
-5.4487000	13.9568005	102.2999954
-5.4487000	14.0056000	102.8099976
-5.4487000	14.0544004	102.6699982

-5.4487000	14.1032000	103.3099976
-5.4487000	14.1520004	102.1199951
-5.4487000	14.2007999	102.8699951
-5.4487000	14.2496004	102.0100021
-5.4487000	14.2983999	103.1999969
-5.4487000	14.3472004	103.1100006
-5.4487000	14.3959999	103.1500015
-5.4487000	14.4448004	104.1800003
-5.4487000	14.4935999	104.5299988
-5.4487000	14.5424004	105.0800018
-5.4487000	14.5911999	104.9399948
-5.4487000	14.6400003	102.4599991
-5.4487000	14.6887999	97.9899979
-5.4487000	14.7376003	102.0800018
-5.4487000	14.7863998	105.9700012
-5.4487000	14.8352003	106.1999969
-5.4487000	14.8839998	102.6100006
-5.4487000	14.9328003	100.3399963
-5.4487000	14.9815998	97.2099991
-5.4487000	15.0304003	102.1399994
-5.4425640	0.3416000	107.0599976
-5.4425640	0.3904000	107.7600021
-5.4425640	0.4392000	105.7200012
-5.4425640	0.4880000	104.1500015
-5.4425640	0.5368000	104.9599991
-5.4425640	0.5856000	103.9099960
-5.4425640	0.6344000	105.4899979
-5.4425640	0.6832000	106.4799957
-5.4425640	0.7320000	107.5299988
-5.4425640	0.7808000	105.0299988
-5.4425640	0.8296000	105.1399994
-5.4425640	0.8784000	105.2099991
-5.4425640	0.9272000	106.9199982
-5.4425640	0.9760000	107.7099991
-5.4425640	1.0247999	107.3799973
-5.4425640	1.0735999	105.1299973
-5.4425640	1.1223999	107.5699997
-5.4425640	1.1711999	107.9300003
-5.4425640	1.2200000	106.2399979
-5.4425640	1.2688000	103.9000015
-5.4425640	1.3176000	101.1100006
-5.4425640	1.3664000	106.9899979
-5.4425640	1.4152000	107.7999954
-5.4425640	1.4640000	105.8899994
-5.4425640	1.5128000	103.6599960
-5.4425640	1.5616000	106.1599960
-5.4425640	1.6104000	106.9899979
-5.4425640	1.6592000	105.4300003
-5.4425640	1.7079999	102.7699966
-5.4425640	1.7567999	102.7299957
-5.4425640	1.8055999	102.7500000
-5.4425640	1.8543999	104.9799957
-5.4425640	1.9031999	106.7500000
-5.4425640	1.9519999	106.3300018
-5.4425640	2.0007999	102.0899963
-5.4425640	2.0495999	102.2900009
-5.4425640	2.0983999	104.4700012
-5.4425640	2.1471999	104.7799988

-5.4425640	2.1959999	106.4300003
-5.4425640	2.2447999	107.4300003
-5.4425640	2.2935998	105.4199982
-5.4425640	2.3423998	103.9599991
-5.4425640	2.3912001	106.9300003
-5.4425640	2.4400001	106.1699982
-5.4425640	2.4888000	107.9300003
-5.4425640	2.5376000	108.0199966
-5.4425640	2.5864000	103.9799957
-5.4425640	2.6352000	102.8600006
-5.4425640	2.6840000	102.7200012
-5.4425640	2.7328000	106.7900009
-5.4425640	2.7816000	106.4700012
-5.4425640	2.8304000	104.0400009
-5.4425640	2.8792000	104.4599991
-5.4425640	2.9280000	101.7600021
-5.4425640	2.9768000	105.2500000
-5.4425640	3.0256000	106.8600006
-5.4425640	3.0743999	106.7699966
-5.4425640	3.1231999	106.8600006
-5.4425640	3.1719999	106.4899979
-5.4425640	3.2207999	105.9199982
-5.4425640	3.2695999	105.5699997
-5.4425640	3.3183999	104.2200012
-5.4425640	3.3671999	102.8699951
-5.4425640	3.4159999	101.9599991
-5.4425640	3.4647999	103.2200012
-5.4425640	3.5135999	102.0699997
-5.4425640	3.5623999	104.6999969
-5.4425640	3.6111999	105.3699951
-5.4425640	3.6599998	105.9799957
-5.4425640	3.7087998	104.2900009
-5.4425640	3.7575998	104.9199982
-5.4425640	3.8063998	104.7999954
-5.4425640	3.8551998	105.0199966
-5.4425640	3.9039998	104.2799988
-5.4425640	3.9527998	102.4700012
-5.4425640	4.0016003	102.2699966
-5.4425640	4.0503998	102.3099976
-5.4425640	4.0991998	98.9300003
-5.4425640	4.1479998	104.5999985
-5.4425640	4.1967998	107.4599991
-5.4425640	4.2455997	106.1399994
-5.4425640	4.2943997	101.9099960
-5.4425640	4.3431997	105.9399948
-5.4425640	4.3919997	103.8399963
-5.4425640	4.4407997	102.4399948
-5.4425640	4.4895997	104.8199997
-5.4425640	4.5383997	106.4799957
-5.4425640	4.5871997	107.8199997
-5.4425640	4.6359997	109.1100006
-5.4425640	4.6847997	112.5800018
-5.4425640	4.7336001	110.8300018
-5.4425640	4.7824001	103.7600021
-5.4425640	4.8312001	105.6999969
-5.4425640	4.8800001	109.0100021
-5.4425640	4.9288001	107.4799957
-5.4425640	4.9776001	102.3799973

-5.4425640	5.0264001	103.0499954
-5.4425640	5.0752001	104.8600006
-5.4425640	5.1240001	106.7699966
-5.4425640	5.1728001	105.6299973
-5.4425640	5.2216001	104.7099991
-5.4425640	5.2704000	97.3399963
-5.4425640	5.3192000	102.4700012
-5.4425640	5.3680000	103.0100021
-5.4425640	5.4168000	103.2200012
-5.4425640	5.4656000	106.2900009
-5.4425640	5.5144000	107.4199982
-5.4425640	5.5632000	107.0899963
-5.4425640	5.6120000	106.9399948
-5.4425640	5.6608000	105.0800018
-5.4425640	5.7096000	102.5000000
-5.4425640	5.7584000	104.4599991
-5.4425640	5.8072000	102.2299957
-5.4425640	5.8559999	103.9099960
-5.4425640	5.9047999	104.2900009
-5.4425640	5.9535999	102.8799973
-5.4425640	6.0023999	100.8399963
-5.4425640	6.0511999	102.4799957
-5.4425640	6.0999999	107.9099960
-5.4425640	6.1487999	106.2099991
-5.4425640	6.1975999	103.8499985
-5.4425640	6.2463999	105.3099976
-5.4425640	6.2951999	106.3300018
-5.4425640	6.3439999	104.9300003
-5.4425640	6.3927999	103.5999985
-5.4425640	6.4415998	102.7200012
-5.4425640	6.4903998	103.6199951
-5.4425640	6.5391998	103.3799973
-5.4425640	6.5879998	104.7600021
-5.4425640	6.6367998	104.3600006
-5.4425640	6.6855998	102.6100006
-5.4425640	6.7343998	104.2099991
-5.4425640	6.7831998	105.8699951
-5.4425640	6.8319998	106.4700012
-5.4425640	6.8807998	105.9799957
-5.4425640	6.9295998	103.3600006
-5.4425640	6.9783998	103.2699966
-5.4425640	7.0271997	102.8699951
-5.4425640	7.0759997	104.2200012
-5.4425640	7.1247997	104.3499985
-5.4425640	7.1735997	103.8199997
-5.4425640	7.2223997	106.3699951
-5.4425640	7.2711997	106.8399963
-5.4425640	7.3199997	106.8499985
-5.4425640	7.3687997	104.3499985
-5.4425640	7.4175997	100.7699966
-5.4425640	7.4663997	102.4000015
-5.4425640	7.5151997	103.9099960
-5.4425640	7.5639997	103.7099991
-5.4425640	7.6127996	102.7500000
-5.4425640	7.6615996	103.6999969
-5.4425640	7.7103996	105.5999985
-5.4425640	7.7591996	104.8399963
-5.4425640	7.8079996	104.2699966

-5.4425640	7.8567996	105.6899948
-5.4425640	7.9056001	104.4799957
-5.4425640	7.9544001	105.0199966
-5.4425640	8.0032005	103.9499969
-5.4425640	8.0520000	103.4599991
-5.4425640	8.1008005	103.7900009
-5.4425640	8.1496000	106.2699966
-5.4425640	8.1984005	106.1599960
-5.4425640	8.2472000	105.1599960
-5.4425640	8.2960005	104.6800003
-5.4425640	8.3448000	103.2799988
-5.4425640	8.3936005	101.4099960
-5.4425640	8.4424000	101.8300018
-5.4425640	8.4912004	104.1299973
-5.4425640	8.5400000	103.7299957
-5.4425640	8.5888004	102.6100006
-5.4425640	8.6375999	100.6399994
-5.4425640	8.6864004	103.2900009
-5.4425640	8.7351999	102.9199982
-5.4425640	8.7840004	102.1599960
-5.4425640	8.8327999	104.3699951
-5.4425640	8.8816004	104.5100021
-5.4425640	8.9303999	104.7399979
-5.4425640	8.9792004	105.6599960
-5.4425640	9.0279999	102.3199997
-5.4425640	9.0768003	101.0299988
-5.4425640	9.1255999	100.2799988
-5.4425640	9.1744003	103.6999969
-5.4425640	9.2231998	105.1599960
-5.4425640	9.2720003	107.5000000
-5.4425640	9.3207998	107.9099960
-5.4425640	9.3696003	105.7799988
-5.4425640	9.4184008	101.1500015
-5.4425640	9.4672003	102.7299957
-5.4425640	9.5160007	100.7699966
-5.4425640	9.5648003	103.4300003
-5.4425640	9.6136007	103.5400009
-5.4425640	9.6624002	100.8099976
-5.4425640	9.7112007	103.5599976
-5.4425640	9.7600002	105.4099960
-5.4425640	9.8088007	106.2799988
-5.4425640	9.8576002	106.3199997
-5.4425640	9.9064007	105.4899979
-5.4425640	9.9552002	99.5699997
-5.4425640	10.0040007	100.6100006
-5.4425640	10.0528002	103.6199951
-5.4425640	10.1016006	103.4899979
-5.4425640	10.1504002	103.7900009
-5.4425640	10.1992006	102.5899963
-5.4425640	10.2480001	100.7699966
-5.4425640	10.2968006	102.9199982
-5.4425640	10.3456001	104.9700012
-5.4425640	10.3944006	104.9099960
-5.4425640	10.4432001	102.4700012
-5.4425640	10.4920006	98.0599976
-5.4425640	10.5408001	105.0299988
-5.4425640	10.5896006	106.7699966
-5.4425640	10.6384001	105.5699997

-5.4425640	10.6872005	105.7299957
-5.4425640	10.7360001	104.4499969
-5.4425640	10.7848005	106.1899948
-5.4425640	10.8336000	106.6999969
-5.4425640	10.8824005	106.2399979
-5.4425640	10.9312000	104.6399994
-5.4425640	10.9800005	106.4099960
-5.4425640	11.0288000	103.6299973
-5.4425640	11.0776005	100.8499985
-5.4425640	11.1264000	105.5299988
-5.4425640	11.1752005	105.8099976
-5.4425640	11.2240000	103.4599991
-5.4425640	11.2728004	102.5299988
-5.4425640	11.3216000	104.2600021
-5.4425640	11.3704004	102.0299988
-5.4425640	11.4191999	100.8300018
-5.4425640	11.4680004	103.5800018
-5.4425640	11.5167999	104.4099960
-5.4425640	11.5656004	104.4499969
-5.4425640	11.6143999	104.2200012
-5.4425640	11.6632004	104.3099976
-5.4425640	11.7119999	103.3300018
-5.4425640	11.7608004	103.0400009
-5.4425640	11.8095999	105.6100006
-5.4425640	11.8584003	103.6800003
-5.4425640	11.9071999	100.6899948
-5.4425640	11.9560003	99.8799973
-5.4425640	12.0047998	99.3399963
-5.4425640	12.0536003	104.1500015
-5.4425640	12.1023998	103.1299973
-5.4425640	12.1512003	103.0299988
-5.4425640	12.1999998	103.4599991
-5.4425640	12.2488003	103.4799957
-5.4425640	12.2975998	102.2299957
-5.4425640	12.3464003	101.6800003
-5.4425640	12.3951998	101.4499969
-5.4425640	12.4440002	99.6399994
-5.4425640	12.4927998	98.7999954
-5.4425640	12.5416002	103.2900009
-5.4425640	12.5904007	106.0699997
-5.4425640	12.6392002	106.0199966
-5.4425640	12.6880007	100.5899963
-5.4425640	12.7368002	102.2999954
-5.4425640	12.7856007	105.5199966
-5.4425640	12.8344002	104.5599976
-5.4425640	12.8832006	102.8300018
-5.4425640	12.9320002	103.0299988
-5.4425640	12.9808006	104.6199951
-5.4425640	13.0296001	100.9499969
-5.4425640	13.0784006	98.6100006
-5.4425640	13.1272001	103.8399963
-5.4425640	13.1760006	105.4399948
-5.4425640	13.2248001	105.0800018
-5.4425640	13.2736006	103.2999954
-5.4425640	13.3224001	103.1899948
-5.4425640	13.3712006	104.9199982
-5.4425640	13.4200001	101.3099976
-5.4425640	13.4688005	101.6500015

-5.4425640	13.5176001	102.8499985
-5.4425640	13.5664005	101.3600006
-5.4425640	13.6152000	102.9399948
-5.4425640	13.6640005	105.7500000
-5.4425640	13.7128000	104.1899948
-5.4425640	13.7616005	103.0100021
-5.4425640	13.8104000	103.1199951
-5.4425640	13.8592005	104.2999954
-5.4425640	13.9080000	106.6899948
-5.4425640	13.9568005	103.0699997
-5.4425640	14.0056000	102.5400009
-5.4425640	14.0544004	102.7200012
-5.4425640	14.1032000	102.9899979
-5.4425640	14.1520004	102.9099960
-5.4425640	14.2007999	103.8799973
-5.4425640	14.2496004	102.9700012
-5.4425640	14.2983999	102.8600006
-5.4425640	14.3472004	101.9799957
-5.4425640	14.3959999	102.5199966
-5.4425640	14.4448004	104.2799988
-5.4425640	14.4935999	104.6500015
-5.4425640	14.5424004	105.4700012
-5.4425640	14.5911999	103.6500015
-5.4425640	14.6400003	102.7500000
-5.4425640	14.6887999	100.1399994
-5.4425640	14.7376003	103.2500000
-5.4425640	14.7863998	106.0800018
-5.4425640	14.8352003	105.9399948
-5.4425640	14.8839998	103.4799957
-5.4425640	14.9328003	101.0699997
-5.4425640	14.9815998	97.9599991
-5.4425640	15.0304003	102.6999969
-5.4364281	0.3416000	108.2500000
-5.4364281	0.3904000	108.5299988
-5.4364281	0.4392000	106.6100006
-5.4364281	0.4880000	104.7200012
-5.4364281	0.5368000	105.5699997
-5.4364281	0.5856000	104.4700012
-5.4364281	0.6344000	104.6399994
-5.4364281	0.6832000	105.7900009
-5.4364281	0.7320000	107.4599991
-5.4364281	0.7808000	105.2699966
-5.4364281	0.8296000	105.9599991
-5.4364281	0.8784000	105.2900009
-5.4364281	0.9272000	106.5800018
-5.4364281	0.9760000	107.0999985
-5.4364281	1.0247999	106.5199966
-5.4364281	1.0735999	104.7600021
-5.4364281	1.1223999	108.2900009
-5.4364281	1.1711999	108.9399948
-5.4364281	1.2200000	106.7500000
-5.4364281	1.2688000	104.5199966
-5.4364281	1.3176000	101.4799957
-5.4364281	1.3664000	107.3099976
-5.4364281	1.4152000	108.6599960
-5.4364281	1.4640000	106.1500015
-5.4364281	1.5128000	103.6399994
-5.4364281	1.5616000	105.8399963

-5.4364281	1.6104000	106.1800003
-5.4364281	1.6592000	105.7799988
-5.4364281	1.7079999	103.8499985
-5.4364281	1.7567999	103.2399979
-5.4364281	1.8055999	103.1500015
-5.4364281	1.8543999	105.0999985
-5.4364281	1.9031999	106.0800018
-5.4364281	1.9519999	105.1299973
-5.4364281	2.0007999	101.4000015
-5.4364281	2.0495999	101.6999969
-5.4364281	2.0983999	104.2500000
-5.4364281	2.1471999	105.1199951
-5.4364281	2.1959999	106.7299957
-5.4364281	2.2447999	107.6800003
-5.4364281	2.2935998	105.5000000
-5.4364281	2.3423998	104.9799957
-5.4364281	2.3912001	107.4599991
-5.4364281	2.4400001	106.2699966
-5.4364281	2.4888000	108.7799988
-5.4364281	2.5376000	108.5999985
-5.4364281	2.5864000	105.0599976
-5.4364281	2.6352000	103.6800003
-5.4364281	2.6840000	102.8300018
-5.4364281	2.7328000	106.9099960
-5.4364281	2.7816000	106.4899979
-5.4364281	2.8304000	104.0400009
-5.4364281	2.8792000	103.8699951
-5.4364281	2.9280000	101.2999954
-5.4364281	2.9768000	104.8600006
-5.4364281	3.0256000	106.4899979
-5.4364281	3.0743999	107.0800018
-5.4364281	3.1231999	106.8300018
-5.4364281	3.1719999	106.7299957
-5.4364281	3.2207999	106.6599960
-5.4364281	3.2695999	105.7200012
-5.4364281	3.3183999	104.7900009
-5.4364281	3.3671999	103.6699982
-5.4364281	3.4159999	102.4899979
-5.4364281	3.4647999	102.8600006
-5.4364281	3.5135999	101.2900009
-5.4364281	3.5623999	104.8899994
-5.4364281	3.6111999	105.0100021
-5.4364281	3.6599998	105.8699951
-5.4364281	3.7087998	103.8499985
-5.4364281	3.7575998	104.4599991
-5.4364281	3.8063998	103.7500000
-5.4364281	3.8551998	104.0599976
-5.4364281	3.9039998	104.1399994
-5.4364281	3.9527998	102.8399963
-5.4364281	4.0016003	102.6699982
-5.4364281	4.0503998	102.8499985
-5.4364281	4.0991998	98.7500000
-5.4364281	4.1479998	104.7500000
-5.4364281	4.1967998	106.7399979
-5.4364281	4.2455997	105.0100021
-5.4364281	4.2943997	101.2200012
-5.4364281	4.3431997	104.2099991
-5.4364281	4.3919997	102.5899963

-5.4364281	4.4407997	101.8399963
-5.4364281	4.4895997	105.6899948
-5.4364281	4.5383997	107.0599976
-5.4364281	4.5871997	107.0299988
-5.4364281	4.6359997	107.9899979
-5.4364281	4.6847997	111.8600006
-5.4364281	4.7336001	110.1599960
-5.4364281	4.7824001	103.2799988
-5.4364281	4.8312001	105.4499969
-5.4364281	4.8800001	109.0400009
-5.4364281	4.9288001	107.3799973
-5.4364281	4.9776001	101.9099960
-5.4364281	5.0264001	103.1500015
-5.4364281	5.0752001	105.2299957
-5.4364281	5.1240001	107.3199997
-5.4364281	5.1728001	106.3499985
-5.4364281	5.2216001	104.7500000
-5.4364281	5.2704000	98.6100006
-5.4364281	5.3192000	102.9499969
-5.4364281	5.3680000	103.4000015
-5.4364281	5.4168000	103.9300003
-5.4364281	5.4656000	107.0199966
-5.4364281	5.5144000	107.7399979
-5.4364281	5.5632000	107.5499954
-5.4364281	5.6120000	106.5499954
-5.4364281	5.6608000	105.1999969
-5.4364281	5.7096000	103.1500015
-5.4364281	5.7584000	104.7999954
-5.4364281	5.8072000	103.0699997
-5.4364281	5.8559999	104.4799957
-5.4364281	5.9047999	105.4099960
-5.4364281	5.9535999	103.4499969
-5.4364281	6.0023999	99.4799957
-5.4364281	6.0511999	102.6500015
-5.4364281	6.0999999	108.0000000
-5.4364281	6.1487999	105.9700012
-5.4364281	6.1975999	104.6699982
-5.4364281	6.2463999	105.5800018
-5.4364281	6.2951999	106.0299988
-5.4364281	6.3439999	105.1100006
-5.4364281	6.3927999	104.0000000
-5.4364281	6.4415998	103.3699951
-5.4364281	6.4903998	103.6299973
-5.4364281	6.5391998	103.5999985
-5.4364281	6.5879998	104.8899994
-5.4364281	6.6367998	104.2399979
-5.4364281	6.6855998	102.0499954
-5.4364281	6.7343998	103.3199997
-5.4364281	6.7831998	104.6699982
-5.4364281	6.8319998	106.7999954
-5.4364281	6.8807998	106.7200012
-5.4364281	6.9295998	103.2200012
-5.4364281	6.9783998	102.6299973
-5.4364281	7.0271997	102.3300018
-5.4364281	7.0759997	103.4099960
-5.4364281	7.1247997	104.5299988
-5.4364281	7.1735997	103.8699951
-5.4364281	7.2223997	106.2399979

-5.4364281	7.2711997	106.3600006
-5.4364281	7.3199997	106.4000015
-5.4364281	7.3687997	103.9899979
-5.4364281	7.4175997	101.1699982
-5.4364281	7.4663997	103.0199966
-5.4364281	7.5151997	103.3499985
-5.4364281	7.5639997	103.1999969
-5.4364281	7.6127996	101.5299988
-5.4364281	7.6615996	103.2900009
-5.4364281	7.7103996	105.9899979
-5.4364281	7.7591996	105.6199951
-5.4364281	7.8079996	104.8799973
-5.4364281	7.8567996	105.3600006
-5.4364281	7.9056001	103.4099960
-5.4364281	7.9544001	104.0999985
-5.4364281	8.0032005	103.3499985
-5.4364281	8.0520000	103.4199982
-5.4364281	8.1008005	104.1699982
-5.4364281	8.1496000	105.9799957
-5.4364281	8.1984005	105.6199951
-5.4364281	8.2472000	105.1899948
-5.4364281	8.2960005	104.1599960
-5.4364281	8.3448000	102.8199997
-5.4364281	8.3936005	101.5199966
-5.4364281	8.4424000	101.3799973
-5.4364281	8.4912004	104.6800003
-5.4364281	8.5400000	103.9000015
-5.4364281	8.5888004	102.9099960
-5.4364281	8.6375999	101.4300003
-5.4364281	8.6864004	102.4499969
-5.4364281	8.7351999	102.0000000
-5.4364281	8.7840004	101.8099976
-5.4364281	8.8327999	104.1999969
-5.4364281	8.8816004	103.9599991
-5.4364281	8.9303999	104.3099976
-5.4364281	8.9792004	104.1599960
-5.4364281	9.0279999	101.6399994
-5.4364281	9.0768003	100.2699966
-5.4364281	9.1255999	99.6299973
-5.4364281	9.1744003	104.0400009
-5.4364281	9.2231998	105.6399994
-5.4364281	9.2720003	107.3600006
-5.4364281	9.3207998	106.6399994
-5.4364281	9.3696003	105.2600021
-5.4364281	9.4184008	102.1999969
-5.4364281	9.4672003	102.9300003
-5.4364281	9.5160007	99.7299957
-5.4364281	9.5648003	102.5699997
-5.4364281	9.6136007	103.0800018
-5.4364281	9.6624002	100.3899994
-5.4364281	9.7112007	103.8099976
-5.4364281	9.7600002	105.4199982
-5.4364281	9.8088007	106.4899979
-5.4364281	9.8576002	106.4000015
-5.4364281	9.9064007	105.0999985
-5.4364281	9.9552002	99.6100006
-5.4364281	10.0040007	99.9799957
-5.4364281	10.0528002	102.7900009

-5.4364281	10.1016006	103.3399963
-5.4364281	10.1504002	103.4399948
-5.4364281	10.1992006	102.0000000
-5.4364281	10.2480001	100.7699966
-5.4364281	10.2968006	103.4300003
-5.4364281	10.3456001	105.0199966
-5.4364281	10.3944006	105.5000000
-5.4364281	10.4432001	102.5499954
-5.4364281	10.4920006	97.4199982
-5.4364281	10.5408001	104.4700012
-5.4364281	10.5896006	106.5299988
-5.4364281	10.6384001	104.3799973
-5.4364281	10.6872005	104.8099976
-5.4364281	10.7360001	103.4199982
-5.4364281	10.7848005	106.5800018
-5.4364281	10.8336000	107.3099976
-5.4364281	10.8824005	105.5000000
-5.4364281	10.9312000	103.7999954
-5.4364281	10.9800005	106.0100021
-5.4364281	11.0288000	103.2999954
-5.4364281	11.0776005	101.1199951
-5.4364281	11.1264000	105.6800003
-5.4364281	11.1752005	106.1100006
-5.4364281	11.2240000	104.2500000
-5.4364281	11.2728004	102.5199966
-5.4364281	11.3216000	103.0699997
-5.4364281	11.3704004	101.5699997
-5.4364281	11.4191999	101.4899979
-5.4364281	11.4680004	104.1699982
-5.4364281	11.5167999	105.0400009
-5.4364281	11.5656004	104.8199997
-5.4364281	11.6143999	104.4700012
-5.4364281	11.6632004	104.7099991
-5.4364281	11.7119999	102.8600006
-5.4364281	11.7608004	102.4000015
-5.4364281	11.8095999	105.9399948
-5.4364281	11.8584003	103.9300003
-5.4364281	11.9071999	101.0100021
-5.4364281	11.9560003	99.8099976
-5.4364281	12.0047998	100.1800003
-5.4364281	12.0536003	104.2900009
-5.4364281	12.1023998	102.7500000
-5.4364281	12.1512003	103.5400009
-5.4364281	12.1999998	104.2399979
-5.4364281	12.2488003	103.7399979
-5.4364281	12.2975998	101.9499969
-5.4364281	12.3464003	101.3600006
-5.4364281	12.3951998	102.0299988
-5.4364281	12.4440002	99.5899963
-5.4364281	12.4927998	98.8199997
-5.4364281	12.5416002	103.6399994
-5.4364281	12.5904007	106.5000000
-5.4364281	12.6392002	106.0699997
-5.4364281	12.6880007	100.1100006
-5.4364281	12.7368002	102.0000000
-5.4364281	12.7856007	105.1699982
-5.4364281	12.8344002	103.6599960
-5.4364281	12.8832006	103.1399994

-5.4364281	12.9320002	103.0699997
-5.4364281	12.9808006	104.5000000
-5.4364281	13.0296001	101.7200012
-5.4364281	13.0784006	99.7900009
-5.4364281	13.1272001	104.2299957
-5.4364281	13.1760006	105.2500000
-5.4364281	13.2248001	104.9300003
-5.4364281	13.2736006	103.8699951
-5.4364281	13.3224001	103.9300003
-5.4364281	13.3712006	104.5400009
-5.4364281	13.4200001	101.5000000
-5.4364281	13.4688005	102.9000015
-5.4364281	13.5176001	103.6199951
-5.4364281	13.5664005	100.7099991
-5.4364281	13.6152000	102.3399963
-5.4364281	13.6640005	105.6699982
-5.4364281	13.7128000	104.4300003
-5.4364281	13.7616005	102.5899963
-5.4364281	13.8104000	101.6500015
-5.4364281	13.8592005	103.5599976
-5.4364281	13.9080000	106.4300003
-5.4364281	13.9568005	103.8600006
-5.4364281	14.0056000	102.3099976
-5.4364281	14.0544004	103.0199966
-5.4364281	14.1032000	102.6399994
-5.4364281	14.1520004	103.2600021
-5.4364281	14.2007999	104.1699982
-5.4364281	14.2496004	103.4499969
-5.4364281	14.2983999	102.9599991
-5.4364281	14.3472004	101.5400009
-5.4364281	14.3959999	102.5400009
-5.4364281	14.4448004	104.9700012
-5.4364281	14.4935999	104.9300003
-5.4364281	14.5424004	105.5999985
-5.4364281	14.5911999	103.2999954
-5.4364281	14.6400003	103.1500015
-5.4364281	14.6887999	101.6199951
-5.4364281	14.7376003	104.2600021
-5.4364281	14.7863998	106.3300018
-5.4364281	14.8352003	105.5800018
-5.4364281	14.8839998	103.3799973
-5.4364281	14.9328003	101.5599976
-5.4364281	14.9815998	99.2200012
-5.4364281	15.0304003	103.6100006
-5.4302921	0.3416000	109.3399963
-5.4302921	0.3904000	109.1100006
-5.4302921	0.4392000	107.5199966
-5.4302921	0.4880000	106.0800018
-5.4302921	0.5368000	105.7900009
-5.4302921	0.5856000	104.4899979
-5.4302921	0.6344000	103.9300003
-5.4302921	0.6832000	104.7699966
-5.4302921	0.7320000	106.5699997
-5.4302921	0.7808000	105.1100006
-5.4302921	0.8296000	106.4499969
-5.4302921	0.8784000	105.7099991
-5.4302921	0.9272000	106.0400009
-5.4302921	0.9760000	106.2600021

-5.4302921	1.0247999	105.3300018
-5.4302921	1.0735999	104.9799957
-5.4302921	1.1223999	108.9199982
-5.4302921	1.1711999	109.2699966
-5.4302921	1.2200000	106.9499969
-5.4302921	1.2688000	105.0000000
-5.4302921	1.3176000	102.2900009
-5.4302921	1.3664000	106.9899979
-5.4302921	1.4152000	109.2399979
-5.4302921	1.4640000	107.1500015
-5.4302921	1.5128000	104.3600006
-5.4302921	1.5616000	105.9099960
-5.4302921	1.6104000	105.3300018
-5.4302921	1.6592000	105.4000015
-5.4302921	1.7079999	104.6999969
-5.4302921	1.7567999	104.0299988
-5.4302921	1.8055999	104.1500015
-5.4302921	1.8543999	105.0100021
-5.4302921	1.9031999	105.9399948
-5.4302921	1.9519999	104.0199966
-5.4302921	2.0007999	100.0400009
-5.4302921	2.0495999	100.9199982
-5.4302921	2.0983999	104.4799957
-5.4302921	2.1471999	105.7999954
-5.4302921	2.1959999	106.9399948
-5.4302921	2.2447999	107.5000000
-5.4302921	2.2935998	104.6399994
-5.4302921	2.3423998	104.6599960
-5.4302921	2.3912001	107.4599991
-5.4302921	2.4400001	105.9499969
-5.4302921	2.4888000	108.8799973
-5.4302921	2.5376000	107.9700012
-5.4302921	2.5864000	105.0800018
-5.4302921	2.6352000	103.4399948
-5.4302921	2.6840000	102.8300018
-5.4302921	2.7328000	107.0800018
-5.4302921	2.7816000	106.6299973
-5.4302921	2.8304000	104.0599976
-5.4302921	2.8792000	103.0599976
-5.4302921	2.9280000	100.4499969
-5.4302921	2.9768000	104.0899963
-5.4302921	3.0256000	105.9899979
-5.4302921	3.0743999	107.3399963
-5.4302921	3.1231999	106.7299957
-5.4302921	3.1719999	106.7699966
-5.4302921	3.2207999	106.8600006
-5.4302921	3.2695999	105.4199982
-5.4302921	3.3183999	104.5800018
-5.4302921	3.3671999	103.6100006
-5.4302921	3.4159999	102.7900009
-5.4302921	3.4647999	103.2600021
-5.4302921	3.5135999	100.7399979
-5.4302921	3.5623999	104.8199997
-5.4302921	3.6111999	104.5699997
-5.4302921	3.6599998	105.5899963
-5.4302921	3.7087998	103.3099976
-5.4302921	3.7575998	104.1800003
-5.4302921	3.8063998	102.4000015

-5.4302921	3.8551998	102.8099976
-5.4302921	3.9039998	104.1100006
-5.4302921	3.9527998	103.2500000
-5.4302921	4.0016003	103.0000000
-5.4302921	4.0503998	103.6500015
-5.4302921	4.0991998	100.6399994
-5.4302921	4.1479998	105.0199966
-5.4302921	4.1967998	106.1299973
-5.4302921	4.2455997	104.7600021
-5.4302921	4.2943997	101.5899963
-5.4302921	4.3431997	102.7799988
-5.4302921	4.3919997	102.0599976
-5.4302921	4.4407997	101.0899963
-5.4302921	4.4895997	106.0499954
-5.4302921	4.5383997	107.2900009
-5.4302921	4.5871997	106.3199997
-5.4302921	4.6359997	107.5299988
-5.4302921	4.6847997	111.5499954
-5.4302921	4.7336001	109.3699951
-5.4302921	4.7824001	102.8099976
-5.4302921	4.8312001	104.8899994
-5.4302921	4.8800001	108.5100021
-5.4302921	4.9288001	107.1500015
-5.4302921	4.9776001	101.5800018
-5.4302921	5.0264001	103.1800003
-5.4302921	5.0752001	105.4599991
-5.4302921	5.1240001	107.4499969
-5.4302921	5.1728001	106.3699951
-5.4302921	5.2216001	104.7600021
-5.4302921	5.2704000	100.6399994
-5.4302921	5.3192000	102.7799988
-5.4302921	5.3680000	103.1199951
-5.4302921	5.4168000	104.6899948
-5.4302921	5.4656000	107.6800003
-5.4302921	5.5144000	107.8099976
-5.4302921	5.5632000	107.3600006
-5.4302921	5.6120000	105.4499969
-5.4302921	5.6608000	105.1699982
-5.4302921	5.7096000	103.5800018
-5.4302921	5.7584000	104.6699982
-5.4302921	5.8072000	103.4499969
-5.4302921	5.8559999	105.1500015
-5.4302921	5.9047999	105.9700012
-5.4302921	5.9535999	103.7900009
-5.4302921	6.0023999	99.6299973
-5.4302921	6.0511999	104.1100006
-5.4302921	6.0999999	108.4599991
-5.4302921	6.1487999	105.9799957
-5.4302921	6.1975999	106.2699966
-5.4302921	6.2463999	106.2600021
-5.4302921	6.2951999	105.5100021
-5.4302921	6.3439999	104.8600006
-5.4302921	6.3927999	104.1399994
-5.4302921	6.4415998	103.5499954
-5.4302921	6.4903998	103.0299988
-5.4302921	6.5391998	103.3799973
-5.4302921	6.5879998	104.4000015
-5.4302921	6.6367998	104.4099960

-5.4302921	6.6855998	101.3199997
-5.4302921	6.7343998	103.2999954
-5.4302921	6.7831998	103.4199982
-5.4302921	6.8319998	106.3300018
-5.4302921	6.8807998	107.1100006
-5.4302921	6.9295998	103.5100021
-5.4302921	6.9783998	102.3499985
-5.4302921	7.0271997	102.5699997
-5.4302921	7.0759997	102.5800018
-5.4302921	7.1247997	104.7099991
-5.4302921	7.1735997	104.7699966
-5.4302921	7.2223997	106.1399994
-5.4302921	7.2711997	105.4300003
-5.4302921	7.3199997	105.5599976
-5.4302921	7.3687997	104.0400009
-5.4302921	7.4175997	102.0299988
-5.4302921	7.4663997	103.5800018
-5.4302921	7.5151997	102.5899963
-5.4302921	7.5639997	102.0899963
-5.4302921	7.6127996	100.5299988
-5.4302921	7.6615996	103.6100006
-5.4302921	7.7103996	106.3099976
-5.4302921	7.7591996	105.7200012
-5.4302921	7.8079996	104.9300003
-5.4302921	7.8567996	104.9199982
-5.4302921	7.9056001	102.0599976
-5.4302921	7.9544001	102.8699951
-5.4302921	8.0032005	102.6999969
-5.4302921	8.0520000	103.0899963
-5.4302921	8.1008005	103.3699951
-5.4302921	8.1496000	105.3699951
-5.4302921	8.1984005	105.0899963
-5.4302921	8.2472000	104.6599960
-5.4302921	8.2960005	103.6399994
-5.4302921	8.3448000	102.6199951
-5.4302921	8.3936005	102.0999985
-5.4302921	8.4424000	101.9599991
-5.4302921	8.4912004	105.5299988
-5.4302921	8.5400000	104.6100006
-5.4302921	8.5888004	103.4899979
-5.4302921	8.6375999	101.7099991
-5.4302921	8.6864004	101.7099991
-5.4302921	8.7351999	100.9300003
-5.4302921	8.7840004	101.4099960
-5.4302921	8.8327999	104.2900009
-5.4302921	8.8816004	103.9099960
-5.4302921	8.9303999	104.1699982
-5.4302921	8.9792004	103.5100021
-5.4302921	9.0279999	102.0499954
-5.4302921	9.0768003	99.9000015
-5.4302921	9.1255999	98.5100021
-5.4302921	9.1744003	103.7900009
-5.4302921	9.2231998	105.2600021
-5.4302921	9.2720003	106.8799973
-5.4302921	9.3207998	105.4599991
-5.4302921	9.3696003	104.8499985
-5.4302921	9.4184008	103.3899994
-5.4302921	9.4672003	103.2600021

-5.4302921	9.5160007	98.6399994
-5.4302921	9.5648003	101.7299957
-5.4302921	9.6136007	102.0599976
-5.4302921	9.6624002	101.3399963
-5.4302921	9.7112007	103.6399994
-5.4302921	9.7600002	104.6199951
-5.4302921	9.8088007	106.0100021
-5.4302921	9.8576002	106.2699966
-5.4302921	9.9064007	104.5899963
-5.4302921	9.9552002	100.1500015
-5.4302921	10.0040007	99.8899994
-5.4302921	10.0528002	102.3699951
-5.4302921	10.1016006	103.5199966
-5.4302921	10.1504002	103.1100006
-5.4302921	10.1992006	100.8499985
-5.4302921	10.2480001	100.2799988
-5.4302921	10.2968006	103.3300018
-5.4302921	10.3456001	104.4700012
-5.4302921	10.3944006	105.0499954
-5.4302921	10.4432001	102.0599976
-5.4302921	10.4920006	97.7500000
-5.4302921	10.5408001	103.5299988
-5.4302921	10.5896006	105.7299957
-5.4302921	10.6384001	103.0699997
-5.4302921	10.6872005	103.8399963
-5.4302921	10.7360001	101.9799957
-5.4302921	10.7848005	106.4199982
-5.4302921	10.8336000	107.2699966
-5.4302921	10.8824005	104.3899994
-5.4302921	10.9312000	102.8099976
-5.4302921	10.9800005	105.3499985
-5.4302921	11.0288000	102.7399979
-5.4302921	11.0776005	101.5100021
-5.4302921	11.1264000	105.6999969
-5.4302921	11.1752005	106.6100006
-5.4302921	11.2240000	105.3799973
-5.4302921	11.2728004	102.9099960
-5.4302921	11.3216000	102.4099960
-5.4302921	11.3704004	101.9199982
-5.4302921	11.4191999	102.1899948
-5.4302921	11.4680004	104.4899979
-5.4302921	11.5167999	104.9199982
-5.4302921	11.5656004	104.9199982
-5.4302921	11.6143999	104.5800018
-5.4302921	11.6632004	104.8899994
-5.4302921	11.7119999	102.6599960
-5.4302921	11.7608004	101.8899994
-5.4302921	11.8095999	105.5999985
-5.4302921	11.8584003	104.0400009
-5.4302921	11.9071999	101.6500015
-5.4302921	11.9560003	100.0599976
-5.4302921	12.0047998	101.1999969
-5.4302921	12.0536003	104.7999954
-5.4302921	12.1023998	103.2600021
-5.4302921	12.1512003	104.0499954
-5.4302921	12.1999998	105.1100006
-5.4302921	12.2488003	103.7699966
-5.4302921	12.2975998	101.5999985

-5.4302921	12.3464003	101.9300003
-5.4302921	12.3951998	103.3099976
-5.4302921	12.4440002	100.3699951
-5.4302921	12.4927998	100.3699951
-5.4302921	12.5416002	104.3300018
-5.4302921	12.5904007	106.7999954
-5.4302921	12.6392002	106.5000000
-5.4302921	12.6880007	101.1899948
-5.4302921	12.7368002	102.4000015
-5.4302921	12.7856007	105.1100006
-5.4302921	12.8344002	102.8699951
-5.4302921	12.8832006	102.9000015
-5.4302921	12.9320002	102.7799988
-5.4302921	12.9808006	104.2600021
-5.4302921	13.0296001	102.5499954
-5.4302921	13.0784006	101.5899963
-5.4302921	13.1272001	104.9099960
-5.4302921	13.1760006	104.9399948
-5.4302921	13.2248001	104.8399963
-5.4302921	13.2736006	103.8899994
-5.4302921	13.3224001	104.1100006
-5.4302921	13.3712006	104.4199982
-5.4302921	13.4200001	102.3600006
-5.4302921	13.4688005	104.0999985
-5.4302921	13.5176001	104.0599976
-5.4302921	13.5664005	100.0000000
-5.4302921	13.6152000	101.8699951
-5.4302921	13.6640005	105.4499969
-5.4302921	13.7128000	105.1199951
-5.4302921	13.7616005	102.6199951
-5.4302921	13.8104000	100.5499954
-5.4302921	13.8592005	103.2600021
-5.4302921	13.9080000	106.2299957
-5.4302921	13.9568005	104.5800018
-5.4302921	14.0056000	101.9300003
-5.4302921	14.0544004	102.3499985
-5.4302921	14.1032000	101.1800003
-5.4302921	14.1520004	102.6500015
-5.4302921	14.2007999	104.3799973
-5.4302921	14.2496004	104.3600006
-5.4302921	14.2983999	102.4399948
-5.4302921	14.3472004	100.6399994
-5.4302921	14.3959999	103.1299973
-5.4302921	14.4448004	105.9300003
-5.4302921	14.4935999	105.5199966
-5.4302921	14.5424004	105.7299957
-5.4302921	14.5911999	103.1399994
-5.4302921	14.6400003	104.0699997
-5.4302921	14.6887999	103.0800018
-5.4302921	14.7376003	105.1100006
-5.4302921	14.7863998	106.3099976
-5.4302921	14.8352003	104.7699966
-5.4302921	14.8839998	103.2600021
-5.4302921	14.9328003	101.6800003
-5.4302921	14.9815998	100.3499985
-5.4302921	15.0304003	103.9700012
-5.4241562	0.3416000	110.1100006
-5.4241562	0.3904000	109.4099960

-5.4241562	0.4392000	108.0599976
-5.4241562	0.4880000	107.0699997
-5.4241562	0.5368000	106.0299988
-5.4241562	0.5856000	104.6399994
-5.4241562	0.6344000	103.1999969
-5.4241562	0.6832000	103.6699982
-5.4241562	0.7320000	105.4700012
-5.4241562	0.7808000	104.6899948
-5.4241562	0.8296000	106.8399963
-5.4241562	0.8784000	105.8600006
-5.4241562	0.9272000	105.1399994
-5.4241562	0.9760000	104.7900009
-5.4241562	1.0247999	103.7099991
-5.4241562	1.0735999	104.7699966
-5.4241562	1.1223999	108.7500000
-5.4241562	1.1711999	109.2900009
-5.4241562	1.2200000	107.1599960
-5.4241562	1.2688000	105.4199982
-5.4241562	1.3176000	103.4099960
-5.4241562	1.3664000	106.0999985
-5.4241562	1.4152000	107.8899994
-5.4241562	1.4640000	106.0400009
-5.4241562	1.5128000	105.5800018
-5.4241562	1.5616000	106.4000015
-5.4241562	1.6104000	104.2799988
-5.4241562	1.6592000	104.2399979
-5.4241562	1.7079999	104.9599991
-5.4241562	1.7567999	104.7200012
-5.4241562	1.8055999	104.9399948
-5.4241562	1.8543999	104.9300003
-5.4241562	1.9031999	105.8300018
-5.4241562	1.9519999	103.0000000
-5.4241562	2.0007999	99.2699966
-5.4241562	2.0495999	101.0100021
-5.4241562	2.0983999	104.5299988
-5.4241562	2.1471999	106.1100006
-5.4241562	2.1959999	107.0599976
-5.4241562	2.2447999	106.9499969
-5.4241562	2.2935998	103.4599991
-5.4241562	2.3423998	103.8600006
-5.4241562	2.3912001	106.0899963
-5.4241562	2.4400001	104.7399979
-5.4241562	2.4888000	107.7699966
-5.4241562	2.5376000	107.4899979
-5.4241562	2.5864000	105.7999954
-5.4241562	2.6352000	103.5199966
-5.4241562	2.6840000	103.4499969
-5.4241562	2.7328000	107.5199966
-5.4241562	2.7816000	107.1399994
-5.4241562	2.8304000	104.6299973
-5.4241562	2.8792000	101.8399963
-5.4241562	2.9280000	99.5699997
-5.4241562	2.9768000	103.4799957
-5.4241562	3.0256000	105.4199982
-5.4241562	3.0743999	107.4199982
-5.4241562	3.1231999	106.4300003
-5.4241562	3.1719999	106.0800018
-5.4241562	3.2207999	106.1399994

-5.4241562	3.2695999	105.1399994
-5.4241562	3.3183999	104.9799957
-5.4241562	3.3671999	103.6500015
-5.4241562	3.4159999	102.6199951
-5.4241562	3.4647999	102.4799957
-5.4241562	3.5135999	101.2399979
-5.4241562	3.5623999	104.5499954
-5.4241562	3.6111999	104.1399994
-5.4241562	3.6599998	105.2500000
-5.4241562	3.7087998	103.7600021
-5.4241562	3.7575998	104.2799988
-5.4241562	3.8063998	101.4599991
-5.4241562	3.8551998	102.0199966
-5.4241562	3.9039998	104.0199966
-5.4241562	3.9527998	103.3799973
-5.4241562	4.0016003	103.6500015
-5.4241562	4.0503998	104.4799957
-5.4241562	4.0991998	102.5199966
-5.4241562	4.1479998	105.4300003
-5.4241562	4.1967998	105.9399948
-5.4241562	4.2455997	105.3799973
-5.4241562	4.2943997	101.7399979
-5.4241562	4.3431997	101.5299988
-5.4241562	4.3919997	101.9700012
-5.4241562	4.4407997	101.0000000
-5.4241562	4.4895997	106.3300018
-5.4241562	4.5383997	106.9399948
-5.4241562	4.5871997	104.9099960
-5.4241562	4.6359997	107.6699982
-5.4241562	4.6847997	111.9499969
-5.4241562	4.7336001	109.6199951
-5.4241562	4.7824001	102.7200012
-5.4241562	4.8312001	104.2099991
-5.4241562	4.8800001	108.3300018
-5.4241562	4.9288001	107.5000000
-5.4241562	4.9776001	102.3399963
-5.4241562	5.0264001	102.7900009
-5.4241562	5.0752001	105.4799957
-5.4241562	5.1240001	107.0599976
-5.4241562	5.1728001	105.6500015
-5.4241562	5.2216001	104.3099976
-5.4241562	5.2704000	101.9599991
-5.4241562	5.3192000	102.8899994
-5.4241562	5.3680000	103.5499954
-5.4241562	5.4168000	105.8300018
-5.4241562	5.4656000	108.2999954
-5.4241562	5.5144000	107.7399979
-5.4241562	5.5632000	107.3600006
-5.4241562	5.6120000	104.6199951
-5.4241562	5.6608000	105.1100006
-5.4241562	5.7096000	103.2500000
-5.4241562	5.7584000	104.2399979
-5.4241562	5.8072000	103.1199951
-5.4241562	5.8559999	104.9599991
-5.4241562	5.9047999	105.8899994
-5.4241562	5.9535999	104.0899963
-5.4241562	6.0023999	100.3199997
-5.4241562	6.0511999	105.3899994

-5.4241562	6.0999999	108.2600021
-5.4241562	6.1487999	105.4099960
-5.4241562	6.1975999	107.4300003
-5.4241562	6.2463999	107.0800018
-5.4241562	6.2951999	105.7200012
-5.4241562	6.3439999	104.7099991
-5.4241562	6.3927999	103.8499985
-5.4241562	6.4415998	103.4199982
-5.4241562	6.4903998	101.7099991
-5.4241562	6.5391998	102.4899979
-5.4241562	6.5879998	104.2200012
-5.4241562	6.6367998	105.0000000
-5.4241562	6.6855998	100.3799973
-5.4241562	6.7343998	103.4099960
-5.4241562	6.7831998	102.9599991
-5.4241562	6.8319998	105.7799988
-5.4241562	6.8807998	107.3499985
-5.4241562	6.9295998	103.7500000
-5.4241562	6.9783998	102.0800018
-5.4241562	7.0271997	102.8199997
-5.4241562	7.0759997	102.1399994
-5.4241562	7.1247997	104.8300018
-5.4241562	7.1735997	105.1999969
-5.4241562	7.2223997	106.0800018
-5.4241562	7.2711997	105.6100006
-5.4241562	7.3199997	105.0199966
-5.4241562	7.3687997	103.9599991
-5.4241562	7.4175997	102.9000015
-5.4241562	7.4663997	104.0400009
-5.4241562	7.5151997	101.7299957
-5.4241562	7.5639997	100.6100006
-5.4241562	7.6127996	99.5699997
-5.4241562	7.6615996	104.0899963
-5.4241562	7.7103996	106.5499954
-5.4241562	7.7591996	106.0499954
-5.4241562	7.8079996	105.6399994
-5.4241562	7.8567996	104.5599976
-5.4241562	7.9056001	100.3499985
-5.4241562	7.9544001	101.3600006
-5.4241562	8.0032005	101.6599960
-5.4241562	8.0520000	102.0800018
-5.4241562	8.1008005	101.7600021
-5.4241562	8.1496000	104.6599960
-5.4241562	8.1984005	104.9300003
-5.4241562	8.2472000	104.8199997
-5.4241562	8.2960005	103.6299973
-5.4241562	8.3448000	102.7900009
-5.4241562	8.3936005	102.3399963
-5.4241562	8.4424000	102.2600021
-5.4241562	8.4912004	105.7600021
-5.4241562	8.5400000	104.6999969
-5.4241562	8.5888004	104.3099976
-5.4241562	8.6375999	102.2799988
-5.4241562	8.6864004	101.4499969
-5.4241562	8.7351999	100.5299988
-5.4241562	8.7840004	101.2099991
-5.4241562	8.8327999	104.5100021
-5.4241562	8.8816004	104.4300003

-5.4241562	8.9303999	103.9700012
-5.4241562	8.9792004	102.5599976
-5.4241562	9.0279999	101.6999969
-5.4241562	9.0768003	98.9399948
-5.4241562	9.1255999	98.0299988
-5.4241562	9.1744003	102.6299973
-5.4241562	9.2231998	104.5299988
-5.4241562	9.2720003	106.4300003
-5.4241562	9.3207998	104.3600006
-5.4241562	9.3696003	104.2399979
-5.4241562	9.4184008	104.1899948
-5.4241562	9.4672003	103.8099976
-5.4241562	9.5160007	99.2600021
-5.4241562	9.5648003	102.0599976
-5.4241562	9.6136007	101.2799988
-5.4241562	9.6624002	102.1800003
-5.4241562	9.7112007	103.4799957
-5.4241562	9.7600002	103.6299973
-5.4241562	9.8088007	104.9000015
-5.4241562	9.8576002	105.8499985
-5.4241562	9.9064007	104.5000000
-5.4241562	9.9552002	101.5199966
-5.4241562	10.0040007	100.1899948
-5.4241562	10.0528002	101.8300018
-5.4241562	10.1016006	103.2900009
-5.4241562	10.1504002	102.4899979
-5.4241562	10.1992006	99.1100006
-5.4241562	10.2480001	99.7600021
-5.4241562	10.2968006	103.1500015
-5.4241562	10.3456001	103.6399994
-5.4241562	10.3944006	103.9199982
-5.4241562	10.4432001	100.6999969
-5.4241562	10.4920006	97.4700012
-5.4241562	10.5408001	102.3600006
-5.4241562	10.5896006	105.3199997
-5.4241562	10.6384001	102.9899979
-5.4241562	10.6872005	103.8300018
-5.4241562	10.7360001	100.8399963
-5.4241562	10.7848005	106.3499985
-5.4241562	10.8336000	107.3699951
-5.4241562	10.8824005	104.1599960
-5.4241562	10.9312000	102.0699997
-5.4241562	10.9800005	104.8399963
-5.4241562	11.0288000	103.5599976
-5.4241562	11.0776005	101.9399948
-5.4241562	11.1264000	105.5299988
-5.4241562	11.1752005	106.8699951
-5.4241562	11.2240000	106.2600021
-5.4241562	11.2728004	103.2500000
-5.4241562	11.3216000	101.3600006
-5.4241562	11.3704004	101.7799988
-5.4241562	11.4191999	102.8300018
-5.4241562	11.4680004	104.4899979
-5.4241562	11.5167999	104.5699997
-5.4241562	11.5656004	104.9099960
-5.4241562	11.6143999	103.9599991
-5.4241562	11.6632004	104.2999954
-5.4241562	11.7119999	102.8499985

-5.4241562	11.7608004	100.5400009
-5.4241562	11.8095999	104.5199966
-5.4241562	11.8584003	103.5100021
-5.4241562	11.9071999	101.8300018
-5.4241562	11.9560003	100.4399948
-5.4241562	12.0047998	102.1899948
-5.4241562	12.0536003	105.1999969
-5.4241562	12.1023998	103.6599960
-5.4241562	12.1512003	104.3699951
-5.4241562	12.1999998	105.2399979
-5.4241562	12.2488003	103.6999969
-5.4241562	12.2975998	101.6899948
-5.4241562	12.3464003	102.8199997
-5.4241562	12.3951998	104.1800003
-5.4241562	12.4440002	100.6399994
-5.4241562	12.4927998	101.7699966
-5.4241562	12.5416002	105.0100021
-5.4241562	12.5904007	106.4099960
-5.4241562	12.6392002	107.0000000
-5.4241562	12.6880007	103.3099976
-5.4241562	12.7368002	103.4700012
-5.4241562	12.7856007	105.5599976
-5.4241562	12.8344002	102.6699982
-5.4241562	12.8832006	103.1100006
-5.4241562	12.9320002	102.9399948
-5.4241562	12.9808006	104.5100021
-5.4241562	13.0296001	103.6100006
-5.4241562	13.0784006	103.5599976
-5.4241562	13.1272001	105.5199966
-5.4241562	13.1760006	104.0000000
-5.4241562	13.2248001	104.6399994
-5.4241562	13.2736006	103.9700012
-5.4241562	13.3224001	104.4599991
-5.4241562	13.3712006	103.8499985
-5.4241562	13.4200001	103.1500015
-5.4241562	13.4688005	105.3399963
-5.4241562	13.5176001	104.1899948
-5.4241562	13.5664005	100.1899948
-5.4241562	13.6152000	102.4099960
-5.4241562	13.6640005	105.7500000
-5.4241562	13.7128000	105.5999985
-5.4241562	13.7616005	102.8600006
-5.4241562	13.8104000	98.9000015
-5.4241562	13.8592005	102.4899979
-5.4241562	13.9080000	105.5100021
-5.4241562	13.9568005	104.8300018
-5.4241562	14.0056000	101.8099976
-5.4241562	14.0544004	101.9399948
-5.4241562	14.1032000	99.7299957
-5.4241562	14.1520004	102.7600021
-5.4241562	14.2007999	104.7299957
-5.4241562	14.2496004	104.7500000
-5.4241562	14.2983999	100.4899979
-5.4241562	14.3472004	98.8199997
-5.4241562	14.3959999	103.4599991
-5.4241562	14.4448004	106.6199951
-5.4241562	14.4935999	105.4300003
-5.4241562	14.5424004	105.4099960

-5.4241562	14.5911999	103.0199966
-5.4241562	14.6400003	104.5199966
-5.4241562	14.6887999	103.7399979
-5.4241562	14.7376003	105.3199997
-5.4241562	14.7863998	105.7799988
-5.4241562	14.8352003	103.0999985
-5.4241562	14.8839998	102.9300003
-5.4241562	14.9328003	101.0699997
-5.4241562	14.9815998	101.2299957
-5.4241562	15.0304003	104.5100021
-5.4180202	0.3416000	110.0199966
-5.4180202	0.3904000	109.3600006
-5.4180202	0.4392000	108.5699997
-5.4180202	0.4880000	107.4099960
-5.4180202	0.5368000	106.1999969
-5.4180202	0.5856000	104.9199982
-5.4180202	0.6344000	102.4899979
-5.4180202	0.6832000	102.2500000
-5.4180202	0.7320000	104.0400009
-5.4180202	0.7808000	104.0199966
-5.4180202	0.8296000	106.5499954
-5.4180202	0.8784000	105.3600006
-5.4180202	0.9272000	104.1500015
-5.4180202	0.9760000	104.1599960
-5.4180202	1.0247999	102.7900009
-5.4180202	1.0735999	104.5499954
-5.4180202	1.1223999	108.3699951
-5.4180202	1.1711999	108.8699951
-5.4180202	1.2200000	107.1599960
-5.4180202	1.2688000	105.4499969
-5.4180202	1.3176000	103.9700012
-5.4180202	1.3664000	104.8199997
-5.4180202	1.4152000	106.2999954
-5.4180202	1.4640000	104.5599976
-5.4180202	1.5128000	106.0400009
-5.4180202	1.5616000	107.2699966
-5.4180202	1.6104000	104.3899994
-5.4180202	1.6592000	104.2200012
-5.4180202	1.7079999	104.7799988
-5.4180202	1.7567999	105.6500015
-5.4180202	1.8055999	105.9000015
-5.4180202	1.8543999	105.0599976
-5.4180202	1.9031999	105.8199997
-5.4180202	1.9519999	102.2900009
-5.4180202	2.0007999	98.6299973
-5.4180202	2.0495999	99.8499985
-5.4180202	2.0983999	104.0100021
-5.4180202	2.1471999	105.9000015
-5.4180202	2.1959999	106.7099991
-5.4180202	2.2447999	106.0000000
-5.4180202	2.2935998	102.5199966
-5.4180202	2.3423998	103.0199966
-5.4180202	2.3912001	104.6100006
-5.4180202	2.4400001	104.0299988
-5.4180202	2.4888000	106.3699951
-5.4180202	2.5376000	106.7600021
-5.4180202	2.5864000	106.3399963
-5.4180202	2.6352000	104.1899948

-5.4180202	2.6840000	103.5999985
-5.4180202	2.7328000	107.5599976
-5.4180202	2.7816000	107.1899948
-5.4180202	2.8304000	104.7500000
-5.4180202	2.8792000	101.5299988
-5.4180202	2.9280000	99.7699966
-5.4180202	2.9768000	103.4000015
-5.4180202	3.0256000	105.1599960
-5.4180202	3.0743999	106.8899994
-5.4180202	3.1231999	106.0699997
-5.4180202	3.1719999	105.5000000
-5.4180202	3.2207999	105.0699997
-5.4180202	3.2695999	104.7099991
-5.4180202	3.3183999	105.6699982
-5.4180202	3.3671999	104.6999969
-5.4180202	3.4159999	102.4099960
-5.4180202	3.4647999	102.1399994
-5.4180202	3.5135999	102.0199966
-5.4180202	3.5623999	104.8199997
-5.4180202	3.6111999	104.3399963
-5.4180202	3.6599998	105.0000000
-5.4180202	3.7087998	104.1599960
-5.4180202	3.7575998	104.7500000
-5.4180202	3.8063998	101.2500000
-5.4180202	3.8551998	102.7600021
-5.4180202	3.9039998	104.5199966
-5.4180202	3.9527998	104.0100021
-5.4180202	4.0016003	104.3699951
-5.4180202	4.0503998	105.2500000
-5.4180202	4.0991998	104.2600021
-5.4180202	4.1479998	105.2200012
-5.4180202	4.1967998	105.0100021
-5.4180202	4.2455997	105.6500015
-5.4180202	4.2943997	101.8799973
-5.4180202	4.3431997	100.8199997
-5.4180202	4.3919997	102.3699951
-5.4180202	4.4407997	100.4399948
-5.4180202	4.4895997	106.5899963
-5.4180202	4.5383997	106.8399963
-5.4180202	4.5871997	104.4399948
-5.4180202	4.6359997	108.8399963
-5.4180202	4.6847997	112.7799988
-5.4180202	4.7336001	110.1199951
-5.4180202	4.7824001	103.2099991
-5.4180202	4.8312001	103.1599960
-5.4180202	4.8800001	108.0599976
-5.4180202	4.9288001	108.2799988
-5.4180202	4.9776001	103.6899948
-5.4180202	5.0264001	102.8799973
-5.4180202	5.0752001	105.7600021
-5.4180202	5.1240001	106.8499985
-5.4180202	5.1728001	105.1999969
-5.4180202	5.2216001	104.1599960
-5.4180202	5.2704000	103.0299988
-5.4180202	5.3192000	103.1800003
-5.4180202	5.3680000	104.2600021
-5.4180202	5.4168000	105.9799957
-5.4180202	5.4656000	108.7600021

-5.4180202	5.5144000	107.6599960
-5.4180202	5.5632000	107.1599960
-5.4180202	5.6120000	103.9499969
-5.4180202	5.6608000	105.2399979
-5.4180202	5.7096000	102.6899948
-5.4180202	5.7584000	103.5800018
-5.4180202	5.8072000	102.6399994
-5.4180202	5.8559999	104.5699997
-5.4180202	5.9047999	105.1100006
-5.4180202	5.9535999	104.4700012
-5.4180202	6.0023999	101.5599976
-5.4180202	6.0511999	105.6999969
-5.4180202	6.0999999	107.7999954
-5.4180202	6.1487999	104.1999969
-5.4180202	6.1975999	107.6599960
-5.4180202	6.2463999	107.4499969
-5.4180202	6.2951999	105.8399963
-5.4180202	6.3439999	104.5899963
-5.4180202	6.3927999	103.8600006
-5.4180202	6.4415998	103.7399979
-5.4180202	6.4903998	102.4799957
-5.4180202	6.5391998	102.8199997
-5.4180202	6.5879998	104.4700012
-5.4180202	6.6367998	105.4300003
-5.4180202	6.6855998	99.3399963
-5.4180202	6.7343998	104.2099991
-5.4180202	6.7831998	103.9799957
-5.4180202	6.8319998	106.2099991
-5.4180202	6.8807998	107.1299973
-5.4180202	6.9295998	104.0599976
-5.4180202	6.9783998	102.1899948
-5.4180202	7.0271997	103.8300018
-5.4180202	7.0759997	102.7900009
-5.4180202	7.1247997	105.1399994
-5.4180202	7.1735997	105.2999954
-5.4180202	7.2223997	105.6999969
-5.4180202	7.2711997	105.5800018
-5.4180202	7.3199997	104.6199951
-5.4180202	7.3687997	104.2699966
-5.4180202	7.4175997	103.6899948
-5.4180202	7.4663997	104.3300018
-5.4180202	7.5151997	100.9799957
-5.4180202	7.5639997	99.4899979
-5.4180202	7.6127996	99.2299957
-5.4180202	7.6615996	104.3099976
-5.4180202	7.7103996	105.7900009
-5.4180202	7.7591996	105.2399979
-5.4180202	7.8079996	105.5599976
-5.4180202	7.8567996	103.8799973
-5.4180202	7.9056001	98.8899994
-5.4180202	7.9544001	100.5999985
-5.4180202	8.0032005	101.7999954
-5.4180202	8.0520000	101.7600021
-5.4180202	8.1008005	100.7600021
-5.4180202	8.1496000	103.7799988
-5.4180202	8.1984005	104.9099960
-5.4180202	8.2472000	104.8099976
-5.4180202	8.2960005	103.4899979

-5.4180202	8.3448000	103.1800003
-5.4180202	8.3936005	102.9099960
-5.4180202	8.4424000	102.7900009
-5.4180202	8.4912004	106.1999969
-5.4180202	8.5400000	105.2200012
-5.4180202	8.5888004	104.9399948
-5.4180202	8.6375999	102.0599976
-5.4180202	8.6864004	100.5100021
-5.4180202	8.7351999	99.8199997
-5.4180202	8.7840004	101.0800018
-5.4180202	8.8327999	105.0199966
-5.4180202	8.8816004	105.0000000
-5.4180202	8.9303999	103.7999954
-5.4180202	8.9792004	101.5000000
-5.4180202	9.0279999	100.9300003
-5.4180202	9.0768003	97.7999954
-5.4180202	9.1255999	97.9799957
-5.4180202	9.1744003	101.6599960
-5.4180202	9.2231998	103.6999969
-5.4180202	9.2720003	105.8399963
-5.4180202	9.3207998	102.9700012
-5.4180202	9.3696003	103.7099991
-5.4180202	9.4184008	104.6100006
-5.4180202	9.4672003	104.2200012
-5.4180202	9.5160007	99.9000015
-5.4180202	9.5648003	101.9099960
-5.4180202	9.6136007	101.3300018
-5.4180202	9.6624002	103.4300003
-5.4180202	9.7112007	102.9300003
-5.4180202	9.7600002	101.9899979
-5.4180202	9.8088007	103.4199982
-5.4180202	9.8576002	105.2699966
-5.4180202	9.9064007	104.4499969
-5.4180202	9.9552002	103.5499954
-5.4180202	10.0040007	101.5800018
-5.4180202	10.0528002	102.3199997
-5.4180202	10.1016006	103.9300003
-5.4180202	10.1504002	102.2099991
-5.4180202	10.1992006	98.3199997
-5.4180202	10.2480001	100.2200012
-5.4180202	10.2968006	103.7500000
-5.4180202	10.3456001	104.0699997
-5.4180202	10.3944006	103.3899994
-5.4180202	10.4432001	99.5699997
-5.4180202	10.4920006	97.6500015
-5.4180202	10.5408001	102.3099976
-5.4180202	10.5896006	105.0400009
-5.4180202	10.6384001	102.7600021
-5.4180202	10.6872005	103.8699951
-5.4180202	10.7360001	100.8099976
-5.4180202	10.7848005	106.0299988
-5.4180202	10.8336000	107.4300003
-5.4180202	10.8824005	104.1299973
-5.4180202	10.9312000	101.3099976
-5.4180202	10.9800005	104.3799973
-5.4180202	11.0288000	103.6899948
-5.4180202	11.0776005	102.1299973
-5.4180202	11.1264000	105.4399948

-5.4180202	11.1752005	107.1699982
-5.4180202	11.2240000	107.2200012
-5.4180202	11.2728004	103.8399963
-5.4180202	11.3216000	100.7500000
-5.4180202	11.3704004	101.6699982
-5.4180202	11.4191999	102.9899979
-5.4180202	11.4680004	104.1199951
-5.4180202	11.5167999	104.0499954
-5.4180202	11.5656004	104.5699997
-5.4180202	11.6143999	103.0199966
-5.4180202	11.6632004	104.1500015
-5.4180202	11.7119999	103.5299988
-5.4180202	11.7608004	100.0199966
-5.4180202	11.8095999	103.0499954
-5.4180202	11.8584003	102.0299988
-5.4180202	11.9071999	101.2500000
-5.4180202	11.9560003	100.1999969
-5.4180202	12.0047998	103.2099991
-5.4180202	12.0536003	105.4899979
-5.4180202	12.1023998	104.3699951
-5.4180202	12.1512003	104.6800003
-5.4180202	12.1999998	105.0599976
-5.4180202	12.2488003	103.5100021
-5.4180202	12.2975998	101.3699951
-5.4180202	12.3464003	103.5199966
-5.4180202	12.3951998	104.4799957
-5.4180202	12.4440002	100.7600021
-5.4180202	12.4927998	103.5699997
-5.4180202	12.5416002	105.6699982
-5.4180202	12.5904007	105.0400009
-5.4180202	12.6392002	106.7600021
-5.4180202	12.6880007	104.7399979
-5.4180202	12.7368002	104.5199966
-5.4180202	12.7856007	105.6599960
-5.4180202	12.8344002	102.2999954
-5.4180202	12.8832006	103.0800018
-5.4180202	12.9320002	103.2699966
-5.4180202	12.9808006	104.0899963
-5.4180202	13.0296001	103.8699951
-5.4180202	13.0784006	104.6899948
-5.4180202	13.1272001	105.8799973
-5.4180202	13.1760006	103.4899979
-5.4180202	13.2248001	104.3199997
-5.4180202	13.2736006	103.3099976
-5.4180202	13.3224001	104.0400009
-5.4180202	13.3712006	103.5400009
-5.4180202	13.4200001	104.2099991
-5.4180202	13.4688005	105.8499985
-5.4180202	13.5176001	103.5299988
-5.4180202	13.5664005	100.1999969
-5.4180202	13.6152000	102.4700012
-5.4180202	13.6640005	105.4899979
-5.4180202	13.7128000	105.6299973
-5.4180202	13.7616005	103.4700012
-5.4180202	13.8104000	98.0199966
-5.4180202	13.8592005	102.2900009
-5.4180202	13.9080000	103.9499969
-5.4180202	13.9568005	104.2600021

-5.4180202	14.0056000	101.4000015
-5.4180202	14.0544004	101.5999985
-5.4180202	14.1032000	99.6299973
-5.4180202	14.1520004	102.9000015
-5.4180202	14.2007999	104.3899994
-5.4180202	14.2496004	104.4499969
-5.4180202	14.2983999	98.2900009
-5.4180202	14.3472004	98.0599976
-5.4180202	14.3959999	103.1599960
-5.4180202	14.4448004	106.3600006
-5.4180202	14.4935999	105.2799988
-5.4180202	14.5424004	105.4099960
-5.4180202	14.5911999	103.0400009
-5.4180202	14.6400003	104.7500000
-5.4180202	14.6887999	103.8499985
-5.4180202	14.7376003	105.0000000
-5.4180202	14.7863998	104.8699951
-5.4180202	14.8352003	100.5199966
-5.4180202	14.8839998	102.2900009
-5.4180202	14.9328003	100.9000015
-5.4180202	14.9815998	102.5499954
-5.4180202	15.0304003	104.5899963
-5.4118843	0.3416000	109.9399948
-5.4118843	0.3904000	108.8499985
-5.4118843	0.4392000	108.3499985
-5.4118843	0.4880000	107.6100006
-5.4118843	0.5368000	106.2799988
-5.4118843	0.5856000	105.3399963
-5.4118843	0.6344000	102.3899994
-5.4118843	0.6832000	100.9899979
-5.4118843	0.7320000	102.7999954
-5.4118843	0.7808000	103.0400009
-5.4118843	0.8296000	105.7699966
-5.4118843	0.8784000	104.8099976
-5.4118843	0.9272000	103.1999969
-5.4118843	0.9760000	103.5899963
-5.4118843	1.0247999	102.5299988
-5.4118843	1.0735999	104.3899994
-5.4118843	1.1223999	107.7799988
-5.4118843	1.1711999	108.2699966
-5.4118843	1.2200000	106.7799988
-5.4118843	1.2688000	104.7799988
-5.4118843	1.3176000	104.2500000
-5.4118843	1.3664000	103.7099991
-5.4118843	1.4152000	104.7500000
-5.4118843	1.4640000	103.0100021
-5.4118843	1.5128000	106.5299988
-5.4118843	1.5616000	107.9799957
-5.4118843	1.6104000	104.6999969
-5.4118843	1.6592000	104.5800018
-5.4118843	1.7079999	104.6399994
-5.4118843	1.7567999	106.1599960
-5.4118843	1.8055999	107.0000000
-5.4118843	1.8543999	105.7699966
-5.4118843	1.9031999	106.2799988
-5.4118843	1.9519999	102.1399994
-5.4118843	2.0007999	97.8699951
-5.4118843	2.0495999	99.0000000

-5.4118843	2.0983999	104.1199951
-5.4118843	2.1471999	106.1899948
-5.4118843	2.1959999	106.8300018
-5.4118843	2.2447999	105.4199982
-5.4118843	2.2935998	101.7500000
-5.4118843	2.3423998	102.3199997
-5.4118843	2.3912001	103.4899979
-5.4118843	2.4400001	103.6599960
-5.4118843	2.4888000	105.6399994
-5.4118843	2.5376000	107.0800018
-5.4118843	2.5864000	107.1299973
-5.4118843	2.6352000	104.5199966
-5.4118843	2.6840000	104.8300018
-5.4118843	2.7328000	108.0999985
-5.4118843	2.7816000	107.5100021
-5.4118843	2.8304000	104.5699997
-5.4118843	2.8792000	101.2299957
-5.4118843	2.9280000	100.5699997
-5.4118843	2.9768000	104.2900009
-5.4118843	3.0256000	105.4499969
-5.4118843	3.0743999	106.2799988
-5.4118843	3.1231999	105.8600006
-5.4118843	3.1719999	105.1399994
-5.4118843	3.2207999	103.6199951
-5.4118843	3.2695999	104.3300018
-5.4118843	3.3183999	105.4000015
-5.4118843	3.3671999	105.4199982
-5.4118843	3.4159999	102.7999954
-5.4118843	3.4647999	101.8799973
-5.4118843	3.5135999	102.7799988
-5.4118843	3.5623999	105.0299988
-5.4118843	3.6111999	104.7999954
-5.4118843	3.6599998	105.0999985
-5.4118843	3.7087998	104.4000015
-5.4118843	3.7575998	105.0299988
-5.4118843	3.8063998	101.6899948
-5.4118843	3.8551998	103.1100006
-5.4118843	3.9039998	105.0199966
-5.4118843	3.9527998	104.2299957
-5.4118843	4.0016003	104.5999985
-5.4118843	4.0503998	106.1299973
-5.4118843	4.0991998	105.9899979
-5.4118843	4.1479998	105.2900009
-5.4118843	4.1967998	104.3499985
-5.4118843	4.2455997	105.9599991
-5.4118843	4.2943997	101.7600021
-5.4118843	4.3431997	101.9000015
-5.4118843	4.3919997	103.0899963
-5.4118843	4.4407997	99.3699951
-5.4118843	4.4895997	106.1500015
-5.4118843	4.5383997	106.6199951
-5.4118843	4.5871997	103.9599991
-5.4118843	4.6359997	109.8600006
-5.4118843	4.6847997	113.5299988
-5.4118843	4.7336001	110.8199997
-5.4118843	4.7824001	104.3600006
-5.4118843	4.8312001	102.2999954
-5.4118843	4.8800001	107.6999969

-5.4118843	4.9288001	108.7699966
-5.4118843	4.9776001	104.9700012
-5.4118843	5.0264001	102.7900009
-5.4118843	5.0752001	105.7099991
-5.4118843	5.1240001	106.4700012
-5.4118843	5.1728001	104.6999969
-5.4118843	5.2216001	104.6999969
-5.4118843	5.2704000	103.4399948
-5.4118843	5.3192000	103.7600021
-5.4118843	5.3680000	105.6599960
-5.4118843	5.4168000	106.3600006
-5.4118843	5.4656000	109.0000000
-5.4118843	5.5144000	107.5199966
-5.4118843	5.5632000	106.3799973
-5.4118843	5.6120000	102.3300018
-5.4118843	5.6608000	105.2099991
-5.4118843	5.7096000	102.5599976
-5.4118843	5.7584000	103.4099960
-5.4118843	5.8072000	102.3199997
-5.4118843	5.8559999	103.4399948
-5.4118843	5.9047999	103.7299957
-5.4118843	5.9535999	104.4499969
-5.4118843	6.0023999	102.7200012
-5.4118843	6.0511999	105.8699951
-5.4118843	6.0999999	107.0899963
-5.4118843	6.1487999	102.6100006
-5.4118843	6.1975999	107.6199951
-5.4118843	6.2463999	107.5599976
-5.4118843	6.2951999	105.6500015
-5.4118843	6.3439999	104.6800003
-5.4118843	6.3927999	104.6100006
-5.4118843	6.4415998	104.3099976
-5.4118843	6.4903998	102.6299973
-5.4118843	6.5391998	102.7699966
-5.4118843	6.5879998	104.8699951
-5.4118843	6.6367998	106.0999985
-5.4118843	6.6855998	100.3600006
-5.4118843	6.7343998	104.4099960
-5.4118843	6.7831998	104.5800018
-5.4118843	6.8319998	106.4199982
-5.4118843	6.8807998	106.7099991
-5.4118843	6.9295998	104.9000015
-5.4118843	6.9783998	103.1899948
-5.4118843	7.0271997	105.5000000
-5.4118843	7.0759997	103.9399948
-5.4118843	7.1247997	105.1500015
-5.4118843	7.1735997	104.8499985
-5.4118843	7.2223997	105.1699982
-5.4118843	7.2711997	105.4799957
-5.4118843	7.3199997	104.0400009
-5.4118843	7.3687997	104.3499985
-5.4118843	7.4175997	104.0899963
-5.4118843	7.4663997	104.5199966
-5.4118843	7.5151997	101.2799988
-5.4118843	7.5639997	98.6299973
-5.4118843	7.6127996	99.5299988
-5.4118843	7.6615996	104.0699997
-5.4118843	7.7103996	105.1999969

-5.4118843	7.7591996	104.9799957
-5.4118843	7.8079996	105.3399963
-5.4118843	7.8567996	103.2799988
-5.4118843	7.9056001	97.9000015
-5.4118843	7.9544001	100.3499985
-5.4118843	8.0032005	101.9599991
-5.4118843	8.0520000	101.5699997
-5.4118843	8.1008005	100.6100006
-5.4118843	8.1496000	102.2200012
-5.4118843	8.1984005	104.4300003
-5.4118843	8.2472000	104.8600006
-5.4118843	8.2960005	103.9799957
-5.4118843	8.3448000	103.6999969
-5.4118843	8.3936005	103.5299988
-5.4118843	8.4424000	103.9300003
-5.4118843	8.4912004	106.5699997
-5.4118843	8.5400000	105.1999969
-5.4118843	8.5888004	105.2799988
-5.4118843	8.6375999	102.6399994
-5.4118843	8.6864004	100.0699997
-5.4118843	8.7351999	99.5999985
-5.4118843	8.7840004	101.5999985
-5.4118843	8.8327999	105.2099991
-5.4118843	8.8816004	105.4399948
-5.4118843	8.9303999	104.2399979
-5.4118843	8.9792004	100.9300003
-5.4118843	9.0279999	101.2600021
-5.4118843	9.0768003	98.5899963
-5.4118843	9.1255999	99.2600021
-5.4118843	9.1744003	101.8399963
-5.4118843	9.2231998	103.6699982
-5.4118843	9.2720003	105.5000000
-5.4118843	9.3207998	102.4700012
-5.4118843	9.3696003	103.9000015
-5.4118843	9.4184008	104.3099976
-5.4118843	9.4672003	104.4099960
-5.4118843	9.5160007	100.6899948
-5.4118843	9.5648003	101.9499969
-5.4118843	9.6136007	101.1999969
-5.4118843	9.6624002	104.3899994
-5.4118843	9.7112007	102.8499985
-5.4118843	9.7600002	101.0599976
-5.4118843	9.8088007	101.9000015
-5.4118843	9.8576002	104.3499985
-5.4118843	9.9064007	103.6100006
-5.4118843	9.9552002	104.6800003
-5.4118843	10.0040007	103.2799988
-5.4118843	10.0528002	103.4199982
-5.4118843	10.1016006	104.9799957
-5.4118843	10.1504002	102.3300018
-5.4118843	10.1992006	97.6399994
-5.4118843	10.2480001	100.6899948
-5.4118843	10.2968006	103.7299957
-5.4118843	10.3456001	104.0599976
-5.4118843	10.3944006	102.8899994
-5.4118843	10.4432001	98.6999969
-5.4118843	10.4920006	97.4700012
-5.4118843	10.5408001	101.8699951

-5.4118843	10.5896006	104.7299957
-5.4118843	10.6384001	103.4499969
-5.4118843	10.6872005	104.0699997
-5.4118843	10.7360001	100.5499954
-5.4118843	10.7848005	105.3199997
-5.4118843	10.8336000	107.1399994
-5.4118843	10.8824005	104.0599976
-5.4118843	10.9312000	100.7799988
-5.4118843	10.9800005	103.7600021
-5.4118843	11.0288000	103.9199982
-5.4118843	11.0776005	102.7600021
-5.4118843	11.1264000	105.7299957
-5.4118843	11.1752005	106.9799957
-5.4118843	11.2240000	107.1800003
-5.4118843	11.2728004	104.1899948
-5.4118843	11.3216000	100.5000000
-5.4118843	11.3704004	102.1199951
-5.4118843	11.4191999	102.2299957
-5.4118843	11.4680004	103.6800003
-5.4118843	11.5167999	103.5000000
-5.4118843	11.5656004	104.1800003
-5.4118843	11.6143999	102.6999969
-5.4118843	11.6632004	103.5199966
-5.4118843	11.7119999	103.6299973
-5.4118843	11.7608004	98.9000015
-5.4118843	11.8095999	101.3699951
-5.4118843	11.8584003	100.8799973
-5.4118843	11.9071999	101.0899963
-5.4118843	11.9560003	100.1999969
-5.4118843	12.0047998	102.8699951
-5.4118843	12.0536003	105.0499954
-5.4118843	12.1023998	104.5499954
-5.4118843	12.1512003	104.2099991
-5.4118843	12.1999998	104.3099976
-5.4118843	12.2488003	103.1199951
-5.4118843	12.2975998	101.2099991
-5.4118843	12.3464003	103.9300003
-5.4118843	12.3951998	103.8899994
-5.4118843	12.4440002	101.7299957
-5.4118843	12.4927998	104.8399963
-5.4118843	12.5416002	106.0299988
-5.4118843	12.5904007	103.7200012
-5.4118843	12.6392002	106.3699951
-5.4118843	12.6880007	105.8399963
-5.4118843	12.7368002	104.9599991
-5.4118843	12.7856007	105.5299988
-5.4118843	12.8344002	102.3499985
-5.4118843	12.8832006	103.9599991
-5.4118843	12.9320002	103.7399979
-5.4118843	12.9808006	103.7299957
-5.4118843	13.0296001	104.0800018
-5.4118843	13.0784006	105.1399994
-5.4118843	13.1272001	106.2500000
-5.4118843	13.1760006	103.5800018
-5.4118843	13.2248001	104.7299957
-5.4118843	13.2736006	103.1599960
-5.4118843	13.3224001	103.7799988
-5.4118843	13.3712006	102.7399979

-5.4118843	13.4200001	104.8199997
-5.4118843	13.4688005	105.8399963
-5.4118843	13.5176001	103.1599960
-5.4118843	13.5664005	101.3099976
-5.4118843	13.6152000	103.0800018
-5.4118843	13.6640005	105.3300018
-5.4118843	13.7128000	105.4599991
-5.4118843	13.7616005	103.8099976
-5.4118843	13.8104000	98.0100021
-5.4118843	13.8592005	102.5299988
-5.4118843	13.9080000	103.3799973
-5.4118843	13.9568005	103.5599976
-5.4118843	14.0056000	101.0299988
-5.4118843	14.0544004	101.4499969
-5.4118843	14.1032000	99.8799973
-5.4118843	14.1520004	102.6899948
-5.4118843	14.2007999	103.0400009
-5.4118843	14.2496004	103.3300018
-5.4118843	14.2983999	96.4700012
-5.4118843	14.3472004	97.7299957
-5.4118843	14.3959999	103.0199966
-5.4118843	14.4448004	105.9300003
-5.4118843	14.4935999	104.8600006
-5.4118843	14.5424004	105.5800018
-5.4118843	14.5911999	104.0899963
-5.4118843	14.6400003	104.0499954
-5.4118843	14.6887999	103.0699997
-5.4118843	14.7376003	104.2999954
-5.4118843	14.7863998	104.0800018
-5.4118843	14.8352003	98.3199997
-5.4118843	14.8839998	101.5100021
-5.4118843	14.9328003	99.8199997
-5.4118843	14.9815998	102.9499969
-5.4118843	15.0304003	104.6899948
-5.4057484	0.3416000	109.4799957
-5.4057484	0.3904000	108.6100006
-5.4057484	0.4392000	108.2900009
-5.4057484	0.4880000	108.1899948
-5.4057484	0.5368000	106.5899963
-5.4057484	0.5856000	105.2699966
-5.4057484	0.6344000	102.5299988
-5.4057484	0.6832000	100.1800003
-5.4057484	0.7320000	101.1699982
-5.4057484	0.7808000	101.9799957
-5.4057484	0.8296000	104.6299973
-5.4057484	0.8784000	104.4499969
-5.4057484	0.9272000	102.4899979
-5.4057484	0.9760000	102.3799973
-5.4057484	1.0247999	101.6899948
-5.4057484	1.0735999	103.6999969
-5.4057484	1.1223999	106.7200012
-5.4057484	1.1711999	107.1599960
-5.4057484	1.2200000	105.9499969
-5.4057484	1.2688000	103.8799973
-5.4057484	1.3176000	104.3799973
-5.4057484	1.3664000	102.3899994
-5.4057484	1.4152000	102.6599960
-5.4057484	1.4640000	100.8399963

-5.4057484	1.5128000	106.6399994
-5.4057484	1.5616000	108.4899979
-5.4057484	1.6104000	105.9799957
-5.4057484	1.6592000	105.3099976
-5.4057484	1.7079999	104.5899963
-5.4057484	1.7567999	106.7600021
-5.4057484	1.8055999	107.1299973
-5.4057484	1.8543999	105.4899979
-5.4057484	1.9031999	106.2200012
-5.4057484	1.9519999	102.5499954
-5.4057484	2.0007999	98.3899994
-5.4057484	2.0495999	99.4399948
-5.4057484	2.0983999	104.6500015
-5.4057484	2.1471999	106.1399994
-5.4057484	2.1959999	106.4499969
-5.4057484	2.2447999	104.8499985
-5.4057484	2.2935998	101.5299988
-5.4057484	2.3423998	101.5100021
-5.4057484	2.3912001	102.2900009
-5.4057484	2.4400001	103.1399994
-5.4057484	2.4888000	104.7299957
-5.4057484	2.5376000	107.0499954
-5.4057484	2.5864000	106.6399994
-5.4057484	2.6352000	104.0199966
-5.4057484	2.6840000	104.8600006
-5.4057484	2.7328000	107.6299973
-5.4057484	2.7816000	107.2600021
-5.4057484	2.8304000	103.9499969
-5.4057484	2.8792000	101.6699982
-5.4057484	2.9280000	101.7099991
-5.4057484	2.9768000	104.8699951
-5.4057484	3.0256000	105.2799988
-5.4057484	3.0743999	105.2500000
-5.4057484	3.1231999	105.2699966
-5.4057484	3.1719999	104.3899994
-5.4057484	3.2207999	101.9899979
-5.4057484	3.2695999	104.1199951
-5.4057484	3.3183999	105.2299957
-5.4057484	3.3671999	105.6899948
-5.4057484	3.4159999	102.7999954
-5.4057484	3.4647999	101.6199951
-5.4057484	3.5135999	103.6299973
-5.4057484	3.5623999	105.3499985
-5.4057484	3.6111999	105.5100021
-5.4057484	3.6599998	105.4000015
-5.4057484	3.7087998	104.6199951
-5.4057484	3.7575998	105.2900009
-5.4057484	3.8063998	102.2600021
-5.4057484	3.8551998	103.2799988
-5.4057484	3.9039998	105.1599960
-5.4057484	3.9527998	104.2799988
-5.4057484	4.0016003	104.9899979
-5.4057484	4.0503998	106.8199997
-5.4057484	4.0991998	107.4300003
-5.4057484	4.1479998	104.9099960
-5.4057484	4.1967998	103.4599991
-5.4057484	4.2455997	106.3600006
-5.4057484	4.2943997	103.0800018

-5.4057484	4.3431997	103.3399963
-5.4057484	4.3919997	103.9700012
-5.4057484	4.4407997	99.6500015
-5.4057484	4.4895997	105.1800003
-5.4057484	4.5383997	106.0899963
-5.4057484	4.5871997	103.0999985
-5.4057484	4.6359997	110.6599960
-5.4057484	4.6847997	114.0000000
-5.4057484	4.7336001	111.2200012
-5.4057484	4.7824001	104.7799988
-5.4057484	4.8312001	101.4099960
-5.4057484	4.8800001	107.2999954
-5.4057484	4.9288001	109.2699966
-5.4057484	4.9776001	105.9599991
-5.4057484	5.0264001	102.6899948
-5.4057484	5.0752001	105.6999969
-5.4057484	5.1240001	105.9199982
-5.4057484	5.1728001	103.4099960
-5.4057484	5.2216001	104.5000000
-5.4057484	5.2704000	103.2699966
-5.4057484	5.3192000	103.8199997
-5.4057484	5.3680000	106.2099991
-5.4057484	5.4168000	105.7200012
-5.4057484	5.4656000	108.6699982
-5.4057484	5.5144000	107.1599960
-5.4057484	5.5632000	105.5499954
-5.4057484	5.6120000	102.4599991
-5.4057484	5.6608000	105.0100021
-5.4057484	5.7096000	101.9700012
-5.4057484	5.7584000	103.1500015
-5.4057484	5.8072000	101.3399963
-5.4057484	5.8559999	102.3899994
-5.4057484	5.9047999	102.1399994
-5.4057484	5.9535999	104.0000000
-5.4057484	6.0023999	103.7799988
-5.4057484	6.0511999	105.8300018
-5.4057484	6.0999999	106.1599960
-5.4057484	6.1487999	102.5999985
-5.4057484	6.1975999	107.3399963
-5.4057484	6.2463999	107.4300003
-5.4057484	6.2951999	105.5699997
-5.4057484	6.3439999	104.5899963
-5.4057484	6.3927999	104.7900009
-5.4057484	6.4415998	104.6199951
-5.4057484	6.4903998	103.1899948
-5.4057484	6.5391998	103.5599976
-5.4057484	6.5879998	105.0599976
-5.4057484	6.6367998	106.3499985
-5.4057484	6.6855998	100.9799957
-5.4057484	6.7343998	104.4899979
-5.4057484	6.7831998	105.6299973
-5.4057484	6.8319998	106.6199951
-5.4057484	6.8807998	105.7299957
-5.4057484	6.9295998	105.3499985
-5.4057484	6.9783998	104.3399963
-5.4057484	7.0271997	106.9599991
-5.4057484	7.0759997	105.1100006
-5.4057484	7.1247997	104.9300003

-5.4057484	7.1735997	104.7600021
-5.4057484	7.2223997	104.4899979
-5.4057484	7.2711997	105.2500000
-5.4057484	7.3199997	103.7799988
-5.4057484	7.3687997	104.2200012
-5.4057484	7.4175997	104.5100021
-5.4057484	7.4663997	104.5800018
-5.4057484	7.5151997	101.5100021
-5.4057484	7.5639997	97.8799973
-5.4057484	7.6127996	100.0100021
-5.4057484	7.6615996	103.7900009
-5.4057484	7.7103996	104.3199997
-5.4057484	7.7591996	104.3300018
-5.4057484	7.8079996	104.3399963
-5.4057484	7.8567996	102.9300003
-5.4057484	7.9056001	98.8799973
-5.4057484	7.9544001	100.7999954
-5.4057484	8.0032005	102.1899948
-5.4057484	8.0520000	102.2299957
-5.4057484	8.1008005	100.3300018
-5.4057484	8.1496000	100.5199966
-5.4057484	8.1984005	104.0000000
-5.4057484	8.2472000	104.6599960
-5.4057484	8.2960005	104.0100021
-5.4057484	8.3448000	103.4899979
-5.4057484	8.3936005	104.1599960
-5.4057484	8.4424000	104.6899948
-5.4057484	8.4912004	106.0699997
-5.4057484	8.5400000	104.2200012
-5.4057484	8.5888004	104.7900009
-5.4057484	8.6375999	101.8499985
-5.4057484	8.6864004	99.5899963
-5.4057484	8.7351999	99.4399948
-5.4057484	8.7840004	101.6699982
-5.4057484	8.8327999	104.7999954
-5.4057484	8.8816004	105.8099976
-5.4057484	8.9303999	105.0400009
-5.4057484	8.9792004	101.9499969
-5.4057484	9.0279999	101.1100006
-5.4057484	9.0768003	98.6999969
-5.4057484	9.1255999	100.2900009
-5.4057484	9.1744003	101.8499985
-5.4057484	9.2231998	103.0100021
-5.4057484	9.2720003	104.8600006
-5.4057484	9.3207998	102.4000015
-5.4057484	9.3696003	104.0999985
-5.4057484	9.4184008	103.7799988
-5.4057484	9.4672003	104.5999985
-5.4057484	9.5160007	101.8399963
-5.4057484	9.5648003	102.1899948
-5.4057484	9.6136007	100.7799988
-5.4057484	9.6624002	104.9899979
-5.4057484	9.7112007	101.9399948
-5.4057484	9.7600002	100.3499985
-5.4057484	9.8088007	101.0400009
-5.4057484	9.8576002	104.3099976
-5.4057484	9.9064007	103.5499954
-5.4057484	9.9552002	104.7299957

-5.4057484	10.0040007	104.1399994
-5.4057484	10.0528002	104.3499985
-5.4057484	10.1016006	106.0000000
-5.4057484	10.1504002	102.8899994
-5.4057484	10.1992006	98.0199966
-5.4057484	10.2480001	101.4199982
-5.4057484	10.2968006	103.8399963
-5.4057484	10.3456001	103.8399963
-5.4057484	10.3944006	102.4700012
-5.4057484	10.4432001	98.0199966
-5.4057484	10.4920006	98.4700012
-5.4057484	10.5408001	102.0299988
-5.4057484	10.5896006	104.7299957
-5.4057484	10.6384001	104.4000015
-5.4057484	10.6872005	104.2900009
-5.4057484	10.7360001	100.2399979
-5.4057484	10.7848005	104.6699982
-5.4057484	10.8336000	106.5999985
-5.4057484	10.8824005	104.3199997
-5.4057484	10.9312000	101.4399948
-5.4057484	10.9800005	103.2999954
-5.4057484	11.0288000	103.1999969
-5.4057484	11.0776005	102.0000000
-5.4057484	11.1264000	105.3300018
-5.4057484	11.1752005	107.2600021
-5.4057484	11.2240000	107.4499969
-5.4057484	11.2728004	104.8199997
-5.4057484	11.3216000	101.7799988
-5.4057484	11.3704004	103.0299988
-5.4057484	11.4191999	101.8099976
-5.4057484	11.4680004	103.0599976
-5.4057484	11.5167999	102.1399994
-5.4057484	11.5656004	103.0000000
-5.4057484	11.6143999	102.3699951
-5.4057484	11.6632004	102.7399979
-5.4057484	11.7119999	103.6100006
-5.4057484	11.7608004	99.1399994
-5.4057484	11.8095999	100.3899994
-5.4057484	11.8584003	101.2500000
-5.4057484	11.9071999	101.6899948
-5.4057484	11.9560003	101.6999969
-5.4057484	12.0047998	103.4899979
-5.4057484	12.0536003	104.7399979
-5.4057484	12.1023998	104.8699951
-5.4057484	12.1512003	103.6699982
-5.4057484	12.1999998	103.2799988
-5.4057484	12.2488003	102.5199966
-5.4057484	12.2975998	101.4899979
-5.4057484	12.3464003	103.6699982
-5.4057484	12.3951998	103.0000000
-5.4057484	12.4440002	101.6800003
-5.4057484	12.4927998	105.2600021
-5.4057484	12.5416002	106.1699982
-5.4057484	12.5904007	102.6599960
-5.4057484	12.6392002	105.8199997
-5.4057484	12.6880007	106.1299973
-5.4057484	12.7368002	104.9000015
-5.4057484	12.7856007	105.6100006

-5.4057484	12.8344002	103.0000000
-5.4057484	12.8832006	104.6399994
-5.4057484	12.9320002	104.2799988
-5.4057484	12.9808006	103.8600006
-5.4057484	13.0296001	104.0299988
-5.4057484	13.0784006	104.8699951
-5.4057484	13.1272001	106.4199982
-5.4057484	13.1760006	103.4899979
-5.4057484	13.2248001	104.3199997
-5.4057484	13.2736006	102.7099991
-5.4057484	13.3224001	103.3799973
-5.4057484	13.3712006	101.9899979
-5.4057484	13.4200001	104.8799973
-5.4057484	13.4688005	105.3499985
-5.4057484	13.5176001	102.5000000
-5.4057484	13.5664005	102.2299957
-5.4057484	13.6152000	103.4899979
-5.4057484	13.6640005	104.9099960
-5.4057484	13.7128000	104.6800003
-5.4057484	13.7616005	103.5199966
-5.4057484	13.8104000	98.5100021
-5.4057484	13.8592005	102.5000000
-5.4057484	13.9080000	102.5499954
-5.4057484	13.9568005	102.8199997
-5.4057484	14.0056000	101.1500015
-5.4057484	14.0544004	101.5000000
-5.4057484	14.1032000	100.5999985
-5.4057484	14.1520004	103.0499954
-5.4057484	14.2007999	102.5800018
-5.4057484	14.2496004	102.5599976
-5.4057484	14.2983999	94.8099976
-5.4057484	14.3472004	97.1100006
-5.4057484	14.3959999	102.1399994
-5.4057484	14.4448004	104.5999985
-5.4057484	14.4935999	104.0400009
-5.4057484	14.5424004	105.2900009
-5.4057484	14.5911999	104.3099976
-5.4057484	14.6400003	103.4599991
-5.4057484	14.6887999	103.5800018
-5.4057484	14.7376003	104.2799988
-5.4057484	14.7863998	103.5599976
-5.4057484	14.8352003	96.4300003
-5.4057484	14.8839998	101.0499954
-5.4057484	14.9328003	98.6199951
-5.4057484	14.9815998	102.7099991
-5.4057484	15.0304003	104.0499954
-5.3996124	0.3416000	108.8899994
-5.3996124	0.3904000	108.0100021
-5.3996124	0.4392000	107.6100006
-5.3996124	0.4880000	107.7600021
-5.3996124	0.5368000	106.5899963
-5.3996124	0.5856000	105.1500015
-5.3996124	0.6344000	102.9300003
-5.3996124	0.6832000	100.5400009
-5.3996124	0.7320000	99.7500000
-5.3996124	0.7808000	100.9499969
-5.3996124	0.8296000	103.1100006
-5.3996124	0.8784000	103.9099960

-5.3996124	0.9272000	102.7399979
-5.3996124	0.9760000	101.7399979
-5.3996124	1.0247999	101.5699997
-5.3996124	1.0735999	103.0400009
-5.3996124	1.1223999	105.6899948
-5.3996124	1.1711999	106.0000000
-5.3996124	1.2200000	105.2699966
-5.3996124	1.2688000	103.4599991
-5.3996124	1.3176000	104.5400009
-5.3996124	1.3664000	102.1599960
-5.3996124	1.4152000	101.1199951
-5.3996124	1.4640000	98.6500015
-5.3996124	1.5128000	106.7299957
-5.3996124	1.5616000	108.8300018
-5.3996124	1.6104000	107.1299973
-5.3996124	1.6592000	106.4099960
-5.3996124	1.7079999	104.6399994
-5.3996124	1.7567999	106.7699966
-5.3996124	1.8055999	106.7200012
-5.3996124	1.8543999	105.5599976
-5.3996124	1.9031999	105.4199982
-5.3996124	1.9519999	103.5400009
-5.3996124	2.0007999	100.2200012
-5.3996124	2.0495999	100.3899994
-5.3996124	2.0983999	105.2500000
-5.3996124	2.1471999	106.4199982
-5.3996124	2.1959999	106.5999985
-5.3996124	2.2447999	104.7500000
-5.3996124	2.2935998	101.9300003
-5.3996124	2.3423998	101.4899979
-5.3996124	2.3912001	101.4399948
-5.3996124	2.4400001	102.3799973
-5.3996124	2.4888000	103.5400009
-5.3996124	2.5376000	107.1100006
-5.3996124	2.5864000	106.7999954
-5.3996124	2.6352000	104.0599976
-5.3996124	2.6840000	104.1299973
-5.3996124	2.7328000	107.0699997
-5.3996124	2.7816000	106.9399948
-5.3996124	2.8304000	103.5299988
-5.3996124	2.8792000	102.4199982
-5.3996124	2.9280000	103.4000015
-5.3996124	2.9768000	105.7999954
-5.3996124	3.0256000	105.6800003
-5.3996124	3.0743999	104.7299957
-5.3996124	3.1231999	104.8199997
-5.3996124	3.1719999	104.2699966
-5.3996124	3.2207999	101.1699982
-5.3996124	3.2695999	103.9799957
-5.3996124	3.3183999	104.9199982
-5.3996124	3.3671999	105.6399994
-5.3996124	3.4159999	103.2799988
-5.3996124	3.4647999	101.4799957
-5.3996124	3.5135999	104.4000015
-5.3996124	3.5623999	105.7399979
-5.3996124	3.6111999	106.3499985
-5.3996124	3.6599998	105.9599991
-5.3996124	3.7087998	104.7699966

-5.3996124	3.7575998	105.1399994
-5.3996124	3.8063998	103.0000000
-5.3996124	3.8551998	103.0499954
-5.3996124	3.9039998	104.6500015
-5.3996124	3.9527998	103.8799973
-5.3996124	4.0016003	105.1199951
-5.3996124	4.0503998	107.1399994
-5.3996124	4.0991998	107.6100006
-5.3996124	4.1479998	105.0299988
-5.3996124	4.1967998	103.0400009
-5.3996124	4.2455997	106.0599976
-5.3996124	4.2943997	103.7500000
-5.3996124	4.3431997	104.8099976
-5.3996124	4.3919997	104.1399994
-5.3996124	4.4407997	100.0599976
-5.3996124	4.4895997	104.6999969
-5.3996124	4.5383997	106.3499985
-5.3996124	4.5871997	102.6199951
-5.3996124	4.6359997	111.3600006
-5.3996124	4.6847997	114.8499985
-5.3996124	4.7336001	112.5599976
-5.3996124	4.7824001	105.8099976
-5.3996124	4.8312001	102.4599991
-5.3996124	4.8800001	108.2900009
-5.3996124	4.9288001	110.3499985
-5.3996124	4.9776001	107.0400009
-5.3996124	5.0264001	103.5599976
-5.3996124	5.0752001	105.9199982
-5.3996124	5.1240001	105.3600006
-5.3996124	5.1728001	103.2299957
-5.3996124	5.2216001	104.5699997
-5.3996124	5.2704000	102.3499985
-5.3996124	5.3192000	104.1899948
-5.3996124	5.3680000	106.6999969
-5.3996124	5.4168000	105.0699997
-5.3996124	5.4656000	107.7799988
-5.3996124	5.5144000	106.5499954
-5.3996124	5.5632000	105.0699997
-5.3996124	5.6120000	102.5400009
-5.3996124	5.6608000	105.2399979
-5.3996124	5.7096000	101.7200012
-5.3996124	5.7584000	102.8899994
-5.3996124	5.8072000	100.4499969
-5.3996124	5.8559999	101.0899963
-5.3996124	5.9047999	101.3699951
-5.3996124	5.9535999	103.7099991
-5.3996124	6.0023999	104.1500015
-5.3996124	6.0511999	105.2399979
-5.3996124	6.0999999	105.0000000
-5.3996124	6.1487999	101.8899994
-5.3996124	6.1975999	106.6599960
-5.3996124	6.2463999	107.1599960
-5.3996124	6.2951999	105.7399979
-5.3996124	6.3439999	104.8099976
-5.3996124	6.3927999	104.9700012
-5.3996124	6.4415998	104.8300018
-5.3996124	6.4903998	103.7999954
-5.3996124	6.5391998	104.5299988

-5.3996124	6.5879998	106.0199966
-5.3996124	6.6367998	106.4899979
-5.3996124	6.6855998	101.7900009
-5.3996124	6.7343998	104.1899948
-5.3996124	6.7831998	106.2699966
-5.3996124	6.8319998	106.6999969
-5.3996124	6.8807998	104.2500000
-5.3996124	6.9295998	105.0299988
-5.3996124	6.9783998	104.8699951
-5.3996124	7.0271997	107.7099991
-5.3996124	7.0759997	106.4099960
-5.3996124	7.1247997	105.2500000
-5.3996124	7.1735997	104.6999969
-5.3996124	7.2223997	104.6500015
-5.3996124	7.2711997	105.3799973
-5.3996124	7.3199997	103.2500000
-5.3996124	7.3687997	103.4700012
-5.3996124	7.4175997	104.4099960
-5.3996124	7.4663997	104.6100006
-5.3996124	7.5151997	101.6999969
-5.3996124	7.5639997	97.4700012
-5.3996124	7.6127996	100.5299988
-5.3996124	7.6615996	103.2299957
-5.3996124	7.7103996	103.6899948
-5.3996124	7.7591996	104.4799957
-5.3996124	7.8079996	103.5899963
-5.3996124	7.8567996	102.5499954
-5.3996124	7.9056001	99.8499985
-5.3996124	7.9544001	101.3600006
-5.3996124	8.0032005	102.3399963
-5.3996124	8.0520000	103.0800018
-5.3996124	8.1008005	100.6899948
-5.3996124	8.1496000	99.2200012
-5.3996124	8.1984005	103.7299957
-5.3996124	8.2472000	104.6800003
-5.3996124	8.2960005	104.0299988
-5.3996124	8.3448000	102.9599991
-5.3996124	8.3936005	104.3399963
-5.3996124	8.4424000	104.6800003
-5.3996124	8.4912004	105.0599976
-5.3996124	8.5400000	102.8399963
-5.3996124	8.5888004	104.4399948
-5.3996124	8.6375999	101.7099991
-5.3996124	8.6864004	99.2999954
-5.3996124	8.7351999	100.0400009
-5.3996124	8.7840004	101.7200012
-5.3996124	8.8327999	103.9799957
-5.3996124	8.8816004	105.9700012
-5.3996124	8.9303999	105.8600006
-5.3996124	8.9792004	102.2799988
-5.3996124	9.0279999	100.1299973
-5.3996124	9.0768003	99.1800003
-5.3996124	9.1255999	101.5699997
-5.3996124	9.1744003	101.9700012
-5.3996124	9.2231998	102.5400009
-5.3996124	9.2720003	103.9099960
-5.3996124	9.3207998	101.8899994
-5.3996124	9.3696003	104.0499954

-5.3996124	9.4184008	103.0999985
-5.3996124	9.4672003	104.7900009
-5.3996124	9.5160007	103.0899963
-5.3996124	9.5648003	102.5999985
-5.3996124	9.6136007	101.4700012
-5.3996124	9.6624002	105.1399994
-5.3996124	9.7112007	101.3499985
-5.3996124	9.7600002	99.5599976
-5.3996124	9.8088007	100.3600006
-5.3996124	9.8576002	104.4700012
-5.3996124	9.9064007	104.0299988
-5.3996124	9.9552002	104.8300018
-5.3996124	10.0040007	105.1399994
-5.3996124	10.0528002	105.2099991
-5.3996124	10.1016006	105.0999985
-5.3996124	10.1504002	102.9000015
-5.3996124	10.1992006	98.5400009
-5.3996124	10.2480001	102.2200012
-5.3996124	10.2968006	104.4099960
-5.3996124	10.3456001	103.7799988
-5.3996124	10.3944006	101.7600021
-5.3996124	10.4432001	98.5699997
-5.3996124	10.4920006	99.4099960
-5.3996124	10.5408001	102.8199997
-5.3996124	10.5896006	105.2099991
-5.3996124	10.6384001	105.1800003
-5.3996124	10.6872005	104.4700012
-5.3996124	10.7360001	100.1199951
-5.3996124	10.7848005	104.7500000
-5.3996124	10.8336000	106.7799988
-5.3996124	10.8824005	105.1999969
-5.3996124	10.9312000	102.4799957
-5.3996124	10.9800005	102.8399963
-5.3996124	11.0288000	102.4300003
-5.3996124	11.0776005	101.0400009
-5.3996124	11.1264000	104.7900009
-5.3996124	11.1752005	107.1399994
-5.3996124	11.2240000	107.2900009
-5.3996124	11.2728004	105.0299988
-5.3996124	11.3216000	102.5499954
-5.3996124	11.3704004	103.3600006
-5.3996124	11.4191999	101.5000000
-5.3996124	11.4680004	102.7999954
-5.3996124	11.5167999	101.6899948
-5.3996124	11.5656004	102.0599976
-5.3996124	11.6143999	101.6800003
-5.3996124	11.6632004	102.3600006
-5.3996124	11.7119999	103.6999969
-5.3996124	11.7608004	99.1299973
-5.3996124	11.8095999	99.6500015
-5.3996124	11.8584003	101.8099976
-5.3996124	11.9071999	102.2799988
-5.3996124	11.9560003	103.4599991
-5.3996124	12.0047998	104.4300003
-5.3996124	12.0536003	104.6599960
-5.3996124	12.1023998	105.1699982
-5.3996124	12.1512003	103.7399979
-5.3996124	12.1999998	102.2600021

-5.3996124	12.2488003	102.3699951
-5.3996124	12.2975998	102.1399994
-5.3996124	12.3464003	103.1500015
-5.3996124	12.3951998	102.0000000
-5.3996124	12.4440002	100.8899994
-5.3996124	12.4927998	104.2600021
-5.3996124	12.5416002	105.7099991
-5.3996124	12.5904007	101.8499985
-5.3996124	12.6392002	105.2399979
-5.3996124	12.6880007	105.6199951
-5.3996124	12.7368002	104.4199982
-5.3996124	12.7856007	105.4599991
-5.3996124	12.8344002	103.4199982
-5.3996124	12.8832006	105.0400009
-5.3996124	12.9320002	104.7799988
-5.3996124	12.9808006	104.0000000
-5.3996124	13.0296001	103.4899979
-5.3996124	13.0784006	103.9899979
-5.3996124	13.1272001	106.0899963
-5.3996124	13.1760006	103.0299988
-5.3996124	13.2248001	103.2399979
-5.3996124	13.2736006	103.3499985
-5.3996124	13.3224001	103.8699951
-5.3996124	13.3712006	101.0100021
-5.3996124	13.4200001	104.1500015
-5.3996124	13.4688005	104.3899994
-5.3996124	13.5176001	101.6500015
-5.3996124	13.5664005	102.9700012
-5.3996124	13.6152000	104.1899948
-5.3996124	13.6640005	105.0800018
-5.3996124	13.7128000	104.0800018
-5.3996124	13.7616005	103.2399979
-5.3996124	13.8104000	98.8300018
-5.3996124	13.8592005	102.3699951
-5.3996124	13.9080000	101.8499985
-5.3996124	13.9568005	102.3399963
-5.3996124	14.0056000	101.4799957
-5.3996124	14.0544004	102.3499985
-5.3996124	14.1032000	102.2600021
-5.3996124	14.1520004	103.7900009
-5.3996124	14.2007999	101.6299973
-5.3996124	14.2496004	101.3399963
-5.3996124	14.2983999	94.6999969
-5.3996124	14.3472004	97.6100006
-5.3996124	14.3959999	101.2399979
-5.3996124	14.4448004	102.4300003
-5.3996124	14.4935999	102.8199997
-5.3996124	14.5424004	104.9899979
-5.3996124	14.5911999	104.1399994
-5.3996124	14.6400003	102.3799973
-5.3996124	14.6887999	103.7500000
-5.3996124	14.7376003	104.1199951
-5.3996124	14.7863998	103.0499954
-5.3996124	14.8352003	95.2200012
-5.3996124	14.8839998	100.8099976
-5.3996124	14.9328003	98.5699997
-5.3996124	14.9815998	102.8300018
-5.3996124	15.0304003	103.2600021

-5.3934765	0.3416000	107.7500000
-5.3934765	0.3904000	107.0499954
-5.3934765	0.4392000	106.7500000
-5.3934765	0.4880000	107.3799973
-5.3934765	0.5368000	106.7500000
-5.3934765	0.5856000	105.3600006
-5.3934765	0.6344000	103.7200012
-5.3934765	0.6832000	101.3300018
-5.3934765	0.7320000	98.8899994
-5.3934765	0.7808000	100.3899994
-5.3934765	0.8296000	102.3399963
-5.3934765	0.8784000	103.9000015
-5.3934765	0.9272000	103.4300003
-5.3934765	0.9760000	101.9300003
-5.3934765	1.0247999	102.0699997
-5.3934765	1.0735999	102.8099976
-5.3934765	1.1223999	105.1999969
-5.3934765	1.1711999	104.4799957
-5.3934765	1.2200000	104.4399948
-5.3934765	1.2688000	103.1399994
-5.3934765	1.3176000	104.8699951
-5.3934765	1.3664000	103.0100021
-5.3934765	1.4152000	101.4399948
-5.3934765	1.4640000	99.6800003
-5.3934765	1.5128000	106.7900009
-5.3934765	1.5616000	108.8399963
-5.3934765	1.6104000	107.9499969
-5.3934765	1.6592000	107.7799988
-5.3934765	1.7079999	104.7999954
-5.3934765	1.7567999	106.4499969
-5.3934765	1.8055999	106.3899994
-5.3934765	1.8543999	106.1999969
-5.3934765	1.9031999	104.9499969
-5.3934765	1.9519999	104.4399948
-5.3934765	2.0007999	101.5499954
-5.3934765	2.0495999	101.1999969
-5.3934765	2.0983999	106.2299957
-5.3934765	2.1471999	106.2799988
-5.3934765	2.1959999	106.5499954
-5.3934765	2.2447999	105.1299973
-5.3934765	2.2935998	102.9899979
-5.3934765	2.3423998	102.2900009
-5.3934765	2.3912001	102.2699966
-5.3934765	2.4400001	101.9899979
-5.3934765	2.4888000	103.6599960
-5.3934765	2.5376000	106.6299973
-5.3934765	2.5864000	106.1299973
-5.3934765	2.6352000	103.6599960
-5.3934765	2.6840000	102.8600006
-5.3934765	2.7328000	106.0599976
-5.3934765	2.7816000	105.9599991
-5.3934765	2.8304000	102.3699951
-5.3934765	2.8792000	103.2099991
-5.3934765	2.9280000	104.9599991
-5.3934765	2.9768000	106.4099960
-5.3934765	3.0256000	105.6699982
-5.3934765	3.0743999	104.2399979
-5.3934765	3.1231999	104.3099976

-5.3934765	3.1719999	104.4099960
-5.3934765	3.2207999	100.9099960
-5.3934765	3.2695999	103.8600006
-5.3934765	3.3183999	104.1199951
-5.3934765	3.3671999	105.0299988
-5.3934765	3.4159999	103.5000000
-5.3934765	3.4647999	102.3799973
-5.3934765	3.5135999	104.5199966
-5.3934765	3.5623999	106.1199951
-5.3934765	3.6111999	106.1899948
-5.3934765	3.6599998	106.1199951
-5.3934765	3.7087998	104.4799957
-5.3934765	3.7575998	105.0599976
-5.3934765	3.8063998	103.3899994
-5.3934765	3.8551998	102.7600021
-5.3934765	3.9039998	104.2699966
-5.3934765	3.9527998	103.6800003
-5.3934765	4.0016003	104.9599991
-5.3934765	4.0503998	107.4700012
-5.3934765	4.0991998	107.5800018
-5.3934765	4.1479998	104.6399994
-5.3934765	4.1967998	102.0299988
-5.3934765	4.2455997	105.1500015
-5.3934765	4.2943997	103.9499969
-5.3934765	4.3431997	105.9300003
-5.3934765	4.3919997	103.9199982
-5.3934765	4.4407997	100.2200012
-5.3934765	4.4895997	104.4700012
-5.3934765	4.5383997	106.5999985
-5.3934765	4.5871997	103.0499954
-5.3934765	4.6359997	111.4300003
-5.3934765	4.6847997	115.1299973
-5.3934765	4.7336001	113.2799988
-5.3934765	4.7824001	106.6699982
-5.3934765	4.8312001	103.3499985
-5.3934765	4.8800001	108.7900009
-5.3934765	4.9288001	110.9799957
-5.3934765	4.9776001	107.4799957
-5.3934765	5.0264001	104.2699966
-5.3934765	5.0752001	105.9300003
-5.3934765	5.1240001	105.2600021
-5.3934765	5.1728001	103.8300018
-5.3934765	5.2216001	104.9000015
-5.3934765	5.2704000	102.2399979
-5.3934765	5.3192000	104.6299973
-5.3934765	5.3680000	106.9099960
-5.3934765	5.4168000	104.2699966
-5.3934765	5.4656000	107.0299988
-5.3934765	5.5144000	106.1999969
-5.3934765	5.5632000	104.2900009
-5.3934765	5.6120000	102.4499969
-5.3934765	5.6608000	105.6399994
-5.3934765	5.7096000	102.4099960
-5.3934765	5.7584000	102.5400009
-5.3934765	5.8072000	100.2600021
-5.3934765	5.8559999	100.8300018
-5.3934765	5.9047999	101.2399979
-5.3934765	5.9535999	103.6100006

-5.3934765	6.0023999	104.2500000
-5.3934765	6.0511999	105.0499954
-5.3934765	6.0999999	104.5999985
-5.3934765	6.1487999	101.3899994
-5.3934765	6.1975999	105.8899994
-5.3934765	6.2463999	106.6699982
-5.3934765	6.2951999	105.4899979
-5.3934765	6.3439999	105.1800003
-5.3934765	6.3927999	105.5999985
-5.3934765	6.4415998	105.3099976
-5.3934765	6.4903998	105.0599976
-5.3934765	6.5391998	106.3300018
-5.3934765	6.5879998	106.7799988
-5.3934765	6.6367998	105.7699966
-5.3934765	6.6855998	102.4099960
-5.3934765	6.7343998	103.7699966
-5.3934765	6.7831998	106.1199951
-5.3934765	6.8319998	106.0699997
-5.3934765	6.8807998	102.5699997
-5.3934765	6.9295998	104.6999969
-5.3934765	6.9783998	105.5100021
-5.3934765	7.0271997	108.0800018
-5.3934765	7.0759997	107.0999985
-5.3934765	7.1247997	105.0000000
-5.3934765	7.1735997	103.4199982
-5.3934765	7.2223997	104.4000015
-5.3934765	7.2711997	104.9700012
-5.3934765	7.3199997	103.0899963
-5.3934765	7.3687997	103.2200012
-5.3934765	7.4175997	104.3199997
-5.3934765	7.4663997	104.4300003
-5.3934765	7.5151997	101.9899979
-5.3934765	7.5639997	97.8600006
-5.3934765	7.6127996	100.8099976
-5.3934765	7.6615996	102.4399948
-5.3934765	7.7103996	103.5299988
-5.3934765	7.7591996	104.8600006
-5.3934765	7.8079996	102.7099991
-5.3934765	7.8567996	102.3199997
-5.3934765	7.9056001	101.2099991
-5.3934765	7.9544001	102.1899948
-5.3934765	8.0032005	102.9099960
-5.3934765	8.0520000	104.5199966
-5.3934765	8.1008005	102.0499954
-5.3934765	8.1496000	98.2099991
-5.3934765	8.1984005	103.4799957
-5.3934765	8.2472000	104.4799957
-5.3934765	8.2960005	103.1599960
-5.3934765	8.3448000	101.9700012
-5.3934765	8.3936005	104.0599976
-5.3934765	8.4424000	104.0699997
-5.3934765	8.4912004	103.5699997
-5.3934765	8.5400000	102.1100006
-5.3934765	8.5888004	103.7399979
-5.3934765	8.6375999	101.0400009
-5.3934765	8.6864004	98.3399963
-5.3934765	8.7351999	100.0400009
-5.3934765	8.7840004	101.9399948

-5.3934765	8.8327999	103.4899979
-5.3934765	8.8816004	105.8300018
-5.3934765	8.9303999	106.3399963
-5.3934765	8.9792004	102.6899948
-5.3934765	9.0279999	100.0899963
-5.3934765	9.0768003	100.1299973
-5.3934765	9.1255999	103.0000000
-5.3934765	9.1744003	103.2500000
-5.3934765	9.2231998	102.5100021
-5.3934765	9.2720003	102.6599960
-5.3934765	9.3207998	101.4000015
-5.3934765	9.3696003	104.5999985
-5.3934765	9.4184008	103.0400009
-5.3934765	9.4672003	104.8399963
-5.3934765	9.5160007	103.3499985
-5.3934765	9.5648003	102.0100021
-5.3934765	9.6136007	101.5899963
-5.3934765	9.6624002	104.6699982
-5.3934765	9.7112007	100.2999954
-5.3934765	9.7600002	98.9599991
-5.3934765	9.8088007	100.0100021
-5.3934765	9.8576002	104.2500000
-5.3934765	9.9064007	104.0400009
-5.3934765	9.9552002	104.6800003
-5.3934765	10.0040007	105.9300003
-5.3934765	10.0528002	106.0000000
-5.3934765	10.1016006	103.7600021
-5.3934765	10.1504002	102.8699951
-5.3934765	10.1992006	99.6199951
-5.3934765	10.2480001	102.8199997
-5.3934765	10.2968006	104.7200012
-5.3934765	10.3456001	103.9700012
-5.3934765	10.3944006	101.8399963
-5.3934765	10.4432001	100.1999969
-5.3934765	10.4920006	101.2699966
-5.3934765	10.5408001	103.4199982
-5.3934765	10.5896006	105.1599960
-5.3934765	10.6384001	105.0400009
-5.3934765	10.6872005	104.0499954
-5.3934765	10.7360001	99.9799957
-5.3934765	10.7848005	104.5499954
-5.3934765	10.8336000	106.5400009
-5.3934765	10.8824005	105.5899963
-5.3934765	10.9312000	103.5800018
-5.3934765	10.9800005	102.3499985
-5.3934765	11.0288000	101.1199951
-5.3934765	11.0776005	100.7200012
-5.3934765	11.1264000	104.1399994
-5.3934765	11.1752005	106.5599976
-5.3934765	11.2240000	106.9300003
-5.3934765	11.2728004	105.1599960
-5.3934765	11.3216000	103.5100021
-5.3934765	11.3704004	104.2099991
-5.3934765	11.4191999	101.4199982
-5.3934765	11.4680004	102.5699997
-5.3934765	11.5167999	100.9899979
-5.3934765	11.5656004	101.0400009
-5.3934765	11.6143999	101.4300003

-5.3934765	11.6632004	102.6299973
-5.3934765	11.7119999	103.9499969
-5.3934765	11.7608004	99.7600021
-5.3934765	11.8095999	99.3600006
-5.3934765	11.8584003	102.8899994
-5.3934765	11.9071999	103.9899979
-5.3934765	11.9560003	105.2500000
-5.3934765	12.0047998	104.8699951
-5.3934765	12.0536003	104.3899994
-5.3934765	12.1023998	105.3600006
-5.3934765	12.1512003	103.9300003
-5.3934765	12.1999998	102.5199966
-5.3934765	12.2488003	102.1800003
-5.3934765	12.2975998	102.2299957
-5.3934765	12.3464003	102.1699982
-5.3934765	12.3951998	100.6800003
-5.3934765	12.4440002	100.4399948
-5.3934765	12.4927998	103.1899948
-5.3934765	12.5416002	105.0499954
-5.3934765	12.5904007	102.0000000
-5.3934765	12.6392002	104.6599960
-5.3934765	12.6880007	104.3199997
-5.3934765	12.7368002	103.5199966
-5.3934765	12.7856007	105.2500000
-5.3934765	12.8344002	103.7500000
-5.3934765	12.8832006	105.1500015
-5.3934765	12.9320002	104.8799973
-5.3934765	12.9808006	103.6999969
-5.3934765	13.0296001	102.6699982
-5.3934765	13.0784006	102.5899963
-5.3934765	13.1272001	105.8600006
-5.3934765	13.1760006	104.2099991
-5.3934765	13.2248001	102.4700012
-5.3934765	13.2736006	103.3799973
-5.3934765	13.3224001	104.1800003
-5.3934765	13.3712006	101.1399994
-5.3934765	13.4200001	104.0800018
-5.3934765	13.4688005	103.7999954
-5.3934765	13.5176001	101.9099960
-5.3934765	13.5664005	103.0599976
-5.3934765	13.6152000	103.7500000
-5.3934765	13.6640005	104.7999954
-5.3934765	13.7128000	103.4099960
-5.3934765	13.7616005	102.7299957
-5.3934765	13.8104000	100.2399979
-5.3934765	13.8592005	102.8300018
-5.3934765	13.9080000	101.9000015
-5.3934765	13.9568005	102.9700012
-5.3934765	14.0056000	101.6500015
-5.3934765	14.0544004	103.1599960
-5.3934765	14.1032000	104.2900009
-5.3934765	14.1520004	103.9399948
-5.3934765	14.2007999	100.7299957
-5.3934765	14.2496004	100.9099960
-5.3934765	14.2983999	97.0400009
-5.3934765	14.3472004	99.8099976
-5.3934765	14.3959999	100.9300003
-5.3934765	14.4448004	100.3499985

-5.3934765	14.4935999	102.1999969
-5.3934765	14.5424004	104.4599991
-5.3934765	14.5911999	103.7999954
-5.3934765	14.6400003	101.8699951
-5.3934765	14.6887999	104.4099960
-5.3934765	14.7376003	104.4199982
-5.3934765	14.7863998	103.4700012
-5.3934765	14.8352003	95.9799957
-5.3934765	14.8839998	101.1800003
-5.3934765	14.9328003	99.0299988
-5.3934765	14.9815998	102.5999985
-5.3934765	15.0304003	102.2399979
-5.3873405	0.3416000	107.0499954
-5.3873405	0.3904000	105.8600006
-5.3873405	0.4392000	104.9599991
-5.3873405	0.4880000	106.8799973
-5.3873405	0.5368000	106.8600006
-5.3873405	0.5856000	105.4300003
-5.3873405	0.6344000	104.8699951
-5.3873405	0.6832000	102.7399979
-5.3873405	0.7320000	98.4099960
-5.3873405	0.7808000	100.1599960
-5.3873405	0.8296000	101.7799988
-5.3873405	0.8784000	103.6800003
-5.3873405	0.9272000	104.4700012
-5.3873405	0.9760000	102.2500000
-5.3873405	1.0247999	102.4300003
-5.3873405	1.0735999	102.6299973
-5.3873405	1.1223999	104.8399963
-5.3873405	1.1711999	103.4899979
-5.3873405	1.2200000	104.2600021
-5.3873405	1.2688000	103.5800018
-5.3873405	1.3176000	106.0000000
-5.3873405	1.3664000	104.3899994
-5.3873405	1.4152000	102.2699966
-5.3873405	1.4640000	101.2799988
-5.3873405	1.5128000	107.0299988
-5.3873405	1.5616000	108.2900009
-5.3873405	1.6104000	108.0599976
-5.3873405	1.6592000	108.6999969
-5.3873405	1.7079999	105.7699966
-5.3873405	1.7567999	105.9399948
-5.3873405	1.8055999	105.3300018
-5.3873405	1.8543999	106.3399963
-5.3873405	1.9031999	104.2299957
-5.3873405	1.9519999	105.0400009
-5.3873405	2.0007999	102.7699966
-5.3873405	2.0495999	103.5699997
-5.3873405	2.0983999	107.5899963
-5.3873405	2.1471999	106.3699951
-5.3873405	2.1959999	106.6199951
-5.3873405	2.2447999	105.4599991
-5.3873405	2.2935998	103.6800003
-5.3873405	2.3423998	102.8199997
-5.3873405	2.3912001	102.8699951
-5.3873405	2.4400001	102.3699951
-5.3873405	2.4888000	103.4700012
-5.3873405	2.5376000	105.9700012

-5.3873405	2.5864000	105.8499985
-5.3873405	2.6352000	103.1699982
-5.3873405	2.6840000	102.2900009
-5.3873405	2.7328000	105.1299973
-5.3873405	2.7816000	104.7200012
-5.3873405	2.8304000	101.3099976
-5.3873405	2.8792000	103.5599976
-5.3873405	2.9280000	105.7099991
-5.3873405	2.9768000	106.6100006
-5.3873405	3.0256000	105.4399948
-5.3873405	3.0743999	104.1999969
-5.3873405	3.1231999	103.5499954
-5.3873405	3.1719999	104.1100006
-5.3873405	3.2207999	100.7900009
-5.3873405	3.2695999	103.3499985
-5.3873405	3.3183999	102.0599976
-5.3873405	3.3671999	103.3699951
-5.3873405	3.4159999	103.4099960
-5.3873405	3.4647999	102.7799988
-5.3873405	3.5135999	104.5199966
-5.3873405	3.5623999	106.2099991
-5.3873405	3.6111999	105.6100006
-5.3873405	3.6599998	106.3899994
-5.3873405	3.7087998	104.7200012
-5.3873405	3.7575998	105.0100021
-5.3873405	3.8063998	103.4799957
-5.3873405	3.8551998	101.4499969
-5.3873405	3.9039998	103.0999985
-5.3873405	3.9527998	102.6100006
-5.3873405	4.0016003	104.1800003
-5.3873405	4.0503998	107.3699951
-5.3873405	4.0991998	106.7699966
-5.3873405	4.1479998	104.7500000
-5.3873405	4.1967998	101.1899948
-5.3873405	4.2455997	104.5499954
-5.3873405	4.2943997	104.6399994
-5.3873405	4.3431997	107.0999985
-5.3873405	4.3919997	103.9700012
-5.3873405	4.4407997	101.1500015
-5.3873405	4.4895997	103.6399994
-5.3873405	4.5383997	106.0299988
-5.3873405	4.5871997	102.1599960
-5.3873405	4.6359997	110.6999969
-5.3873405	4.6847997	115.1500015
-5.3873405	4.7336001	113.9499969
-5.3873405	4.7824001	107.4199982
-5.3873405	4.8312001	104.6100006
-5.3873405	4.8800001	109.3600006
-5.3873405	4.9288001	110.8600006
-5.3873405	4.9776001	107.0800018
-5.3873405	5.0264001	104.0000000
-5.3873405	5.0752001	105.3899994
-5.3873405	5.1240001	104.8300018
-5.3873405	5.1728001	103.9199982
-5.3873405	5.2216001	104.4899979
-5.3873405	5.2704000	102.0299988
-5.3873405	5.3192000	105.1699982
-5.3873405	5.3680000	107.2200012

-5.3873405	5.4168000	104.5699997
-5.3873405	5.4656000	106.3499985
-5.3873405	5.5144000	106.2600021
-5.3873405	5.5632000	104.1299973
-5.3873405	5.6120000	102.2099991
-5.3873405	5.6608000	105.6599960
-5.3873405	5.7096000	103.0400009
-5.3873405	5.7584000	102.5400009
-5.3873405	5.8072000	100.1699982
-5.3873405	5.8559999	100.8300018
-5.3873405	5.9047999	101.5000000
-5.3873405	5.9535999	103.2399979
-5.3873405	6.0023999	104.4599991
-5.3873405	6.0511999	105.2600021
-5.3873405	6.0999999	104.5199966
-5.3873405	6.1487999	101.4199982
-5.3873405	6.1975999	105.6399994
-5.3873405	6.2463999	106.5800018
-5.3873405	6.2951999	105.4399948
-5.3873405	6.3439999	105.6100006
-5.3873405	6.3927999	106.2299957
-5.3873405	6.4415998	105.3300018
-5.3873405	6.4903998	104.4099960
-5.3873405	6.5391998	106.8399963
-5.3873405	6.5879998	106.7999954
-5.3873405	6.6367998	105.1899948
-5.3873405	6.6855998	103.1299973
-5.3873405	6.7343998	102.9899979
-5.3873405	6.7831998	105.0000000
-5.3873405	6.8319998	105.0800018
-5.3873405	6.8807998	102.4499969
-5.3873405	6.9295998	104.3300018
-5.3873405	6.9783998	105.7399979
-5.3873405	7.0271997	107.8499985
-5.3873405	7.0759997	106.9499969
-5.3873405	7.1247997	104.5400009
-5.3873405	7.1735997	101.8699951
-5.3873405	7.2223997	103.9199982
-5.3873405	7.2711997	104.4300003
-5.3873405	7.3199997	102.2200012
-5.3873405	7.3687997	102.8499985
-5.3873405	7.4175997	104.3399963
-5.3873405	7.4663997	104.4700012
-5.3873405	7.5151997	102.5699997
-5.3873405	7.5639997	98.5599976
-5.3873405	7.6127996	100.9700012
-5.3873405	7.6615996	102.0800018
-5.3873405	7.7103996	104.0599976
-5.3873405	7.7591996	105.4099960
-5.3873405	7.8079996	101.9000015
-5.3873405	7.8567996	102.3199997
-5.3873405	7.9056001	102.7500000
-5.3873405	7.9544001	102.8099976
-5.3873405	8.0032005	102.7699966
-5.3873405	8.0520000	104.9799957
-5.3873405	8.1008005	103.1699982
-5.3873405	8.1496000	98.2099991
-5.3873405	8.1984005	103.1899948

-5.3873405	8.2472000	104.4899979
-5.3873405	8.2960005	101.7299957
-5.3873405	8.3448000	101.3799973
-5.3873405	8.3936005	103.7399979
-5.3873405	8.4424000	102.9799957
-5.3873405	8.4912004	101.3699951
-5.3873405	8.5400000	100.6699982
-5.3873405	8.5888004	103.0599976
-5.3873405	8.6375999	101.0899963
-5.3873405	8.6864004	98.9300003
-5.3873405	8.7351999	101.0599976
-5.3873405	8.7840004	102.9899979
-5.3873405	8.8327999	103.3399963
-5.3873405	8.8816004	105.3799973
-5.3873405	8.9303999	106.5000000
-5.3873405	8.9792004	103.1800003
-5.3873405	9.0279999	100.4599991
-5.3873405	9.0768003	101.2600021
-5.3873405	9.1255999	104.2200012
-5.3873405	9.1744003	104.2399979
-5.3873405	9.2231998	102.1500015
-5.3873405	9.2720003	101.6500015
-5.3873405	9.3207998	101.7200012
-5.3873405	9.3696003	104.7799988
-5.3873405	9.4184008	102.6500015
-5.3873405	9.4672003	104.9700012
-5.3873405	9.5160007	104.4499969
-5.3873405	9.5648003	102.9899979
-5.3873405	9.6136007	101.6299973
-5.3873405	9.6624002	104.0199966
-5.3873405	9.7112007	99.5899963
-5.3873405	9.7600002	99.3600006
-5.3873405	9.8088007	100.8399963
-5.3873405	9.8576002	104.6999969
-5.3873405	9.9064007	104.1199951
-5.3873405	9.9552002	103.9899979
-5.3873405	10.0040007	105.7999954
-5.3873405	10.0528002	106.0999985
-5.3873405	10.1016006	102.0599976
-5.3873405	10.1504002	103.3199997
-5.3873405	10.1992006	100.8600006
-5.3873405	10.2480001	103.2399979
-5.3873405	10.2968006	104.5000000
-5.3873405	10.3456001	103.5899963
-5.3873405	10.3944006	101.7999954
-5.3873405	10.4432001	101.6599960
-5.3873405	10.4920006	102.6999969
-5.3873405	10.5408001	103.2799988
-5.3873405	10.5896006	104.8099976
-5.3873405	10.6384001	104.4899979
-5.3873405	10.6872005	103.5999985
-5.3873405	10.7360001	99.4300003
-5.3873405	10.7848005	104.5999985
-5.3873405	10.8336000	106.1999969
-5.3873405	10.8824005	105.6100006
-5.3873405	10.9312000	104.5000000
-5.3873405	10.9800005	102.2399979
-5.3873405	11.0288000	100.2999954

-5.3873405	11.0776005	100.8499985
-5.3873405	11.1264000	103.4300003
-5.3873405	11.1752005	105.7500000
-5.3873405	11.2240000	106.1899948
-5.3873405	11.2728004	104.9700012
-5.3873405	11.3216000	103.8600006
-5.3873405	11.3704004	104.2299957
-5.3873405	11.4191999	101.5699997
-5.3873405	11.4680004	102.6399994
-5.3873405	11.5167999	100.1800003
-5.3873405	11.5656004	100.3399963
-5.3873405	11.6143999	101.4499969
-5.3873405	11.6632004	102.5699997
-5.3873405	11.7119999	103.6999969
-5.3873405	11.7608004	100.1299973
-5.3873405	11.8095999	100.1800003
-5.3873405	11.8584003	104.6199951
-5.3873405	11.9071999	105.6500015
-5.3873405	11.9560003	106.5299988
-5.3873405	12.0047998	104.8699951
-5.3873405	12.0536003	103.6599960
-5.3873405	12.1023998	105.0100021
-5.3873405	12.1512003	104.2099991
-5.3873405	12.1999998	103.5400009
-5.3873405	12.2488003	103.0800018
-5.3873405	12.2975998	102.5400009
-5.3873405	12.3464003	100.9399948
-5.3873405	12.3951998	99.6299973
-5.3873405	12.4440002	100.1599960
-5.3873405	12.4927998	101.8399963
-5.3873405	12.5416002	104.3799973
-5.3873405	12.5904007	102.7500000
-5.3873405	12.6392002	104.5499954
-5.3873405	12.6880007	103.5899963
-5.3873405	12.7368002	102.9199982
-5.3873405	12.7856007	105.2799988
-5.3873405	12.8344002	104.0499954
-5.3873405	12.8832006	105.1800003
-5.3873405	12.9320002	105.0400009
-5.3873405	12.9808006	104.4099960
-5.3873405	13.0296001	102.6500015
-5.3873405	13.0784006	101.5000000
-5.3873405	13.1272001	105.9300003
-5.3873405	13.1760006	105.1599960
-5.3873405	13.2248001	101.7399979
-5.3873405	13.2736006	103.9000015
-5.3873405	13.3224001	104.6199951
-5.3873405	13.3712006	101.0400009
-5.3873405	13.4200001	103.9899979
-5.3873405	13.4688005	103.8899994
-5.3873405	13.5176001	102.5100021
-5.3873405	13.5664005	103.3199997
-5.3873405	13.6152000	103.9799957
-5.3873405	13.6640005	104.8199997
-5.3873405	13.7128000	102.3099976
-5.3873405	13.7616005	101.1999969
-5.3873405	13.8104000	100.9799957
-5.3873405	13.8592005	103.0499954

-5.3873405	13.9080000	103.0499954
-5.3873405	13.9568005	103.6999969
-5.3873405	14.0056000	101.8600006
-5.3873405	14.0544004	103.6800003
-5.3873405	14.1032000	105.6100006
-5.3873405	14.1520004	104.1999969
-5.3873405	14.2007999	100.4599991
-5.3873405	14.2496004	101.3799973
-5.3873405	14.2983999	99.8399963
-5.3873405	14.3472004	101.5100021
-5.3873405	14.3959999	101.6299973
-5.3873405	14.4448004	99.6899948
-5.3873405	14.4935999	101.7699966
-5.3873405	14.5424004	103.2900009
-5.3873405	14.5911999	102.9799957
-5.3873405	14.6400003	101.0800018
-5.3873405	14.6887999	105.1100006
-5.3873405	14.7376003	104.9499969
-5.3873405	14.7863998	104.3699951
-5.3873405	14.8352003	98.3600006
-5.3873405	14.8839998	101.5599976
-5.3873405	14.9328003	99.1500015
-5.3873405	14.9815998	102.2399979
-5.3873405	15.0304003	102.0299988
-5.3812046	0.3416000	106.1100006
-5.3812046	0.3904000	104.7299957
-5.3812046	0.4392000	103.4700012
-5.3812046	0.4880000	106.1999969
-5.3812046	0.5368000	106.8899994
-5.3812046	0.5856000	105.4899979
-5.3812046	0.6344000	106.0800018
-5.3812046	0.6832000	104.5899963
-5.3812046	0.7320000	98.4599991
-5.3812046	0.7808000	101.0899963
-5.3812046	0.8296000	102.5199966
-5.3812046	0.8784000	104.5699997
-5.3812046	0.9272000	105.8099976
-5.3812046	0.9760000	102.6399994
-5.3812046	1.0247999	102.2900009
-5.3812046	1.0735999	102.1100006
-5.3812046	1.1223999	104.5800018
-5.3812046	1.1711999	102.4099960
-5.3812046	1.2200000	103.9499969
-5.3812046	1.2688000	104.5699997
-5.3812046	1.3176000	107.4199982
-5.3812046	1.3664000	106.0400009
-5.3812046	1.4152000	103.9700012
-5.3812046	1.4640000	103.5699997
-5.3812046	1.5128000	107.4899979
-5.3812046	1.5616000	107.7299957
-5.3812046	1.6104000	107.2500000
-5.3812046	1.6592000	109.0899963
-5.3812046	1.7079999	106.4300003
-5.3812046	1.7567999	105.6500015
-5.3812046	1.8055999	104.6500015
-5.3812046	1.8543999	106.5100021
-5.3812046	1.9031999	103.3799973
-5.3812046	1.9519999	105.4899979

-5.3812046	2.0007999	104.3199997
-5.3812046	2.0495999	105.7600021
-5.3812046	2.0983999	107.9799957
-5.3812046	2.1471999	106.1199951
-5.3812046	2.1959999	106.2399979
-5.3812046	2.2447999	104.7600021
-5.3812046	2.2935998	103.7099991
-5.3812046	2.3423998	102.6999969
-5.3812046	2.3912001	102.6800003
-5.3812046	2.4400001	102.2399979
-5.3812046	2.4888000	102.7699966
-5.3812046	2.5376000	104.7299957
-5.3812046	2.5864000	105.4599991
-5.3812046	2.6352000	103.4399948
-5.3812046	2.6840000	100.8300018
-5.3812046	2.7328000	103.6899948
-5.3812046	2.7816000	103.2699966
-5.3812046	2.8304000	100.2200012
-5.3812046	2.8792000	104.0400009
-5.3812046	2.9280000	106.5199966
-5.3812046	2.9768000	106.5100021
-5.3812046	3.0256000	105.1199951
-5.3812046	3.0743999	104.7699966
-5.3812046	3.1231999	102.6800003
-5.3812046	3.1719999	103.5100021
-5.3812046	3.2207999	101.9099960
-5.3812046	3.2695999	103.1500015
-5.3812046	3.3183999	101.0400009
-5.3812046	3.3671999	101.7600021
-5.3812046	3.4159999	102.8300018
-5.3812046	3.4647999	102.9499969
-5.3812046	3.5135999	103.9000015
-5.3812046	3.5623999	105.6899948
-5.3812046	3.6111999	104.5699997
-5.3812046	3.6599998	106.3699951
-5.3812046	3.7087998	104.8600006
-5.3812046	3.7575998	104.7699966
-5.3812046	3.8063998	102.8099976
-5.3812046	3.8551998	100.4499969
-5.3812046	3.9039998	101.5499954
-5.3812046	3.9527998	102.3699951
-5.3812046	4.0016003	104.2099991
-5.3812046	4.0503998	106.8499985
-5.3812046	4.0991998	105.5000000
-5.3812046	4.1479998	104.5899963
-5.3812046	4.1967998	100.9599991
-5.3812046	4.2455997	103.9399948
-5.3812046	4.2943997	105.2200012
-5.3812046	4.3431997	106.9799957
-5.3812046	4.3919997	103.4000015
-5.3812046	4.4407997	102.6399994
-5.3812046	4.4895997	103.4099960
-5.3812046	4.5383997	105.6100006
-5.3812046	4.5871997	101.7099991
-5.3812046	4.6359997	109.9000015
-5.3812046	4.6847997	114.6299973
-5.3812046	4.7336001	113.9399948
-5.3812046	4.7824001	107.7500000

-5.3812046	4.8312001	106.0499954
-5.3812046	4.8800001	109.8600006
-5.3812046	4.9288001	110.6800003
-5.3812046	4.9776001	106.9099960
-5.3812046	5.0264001	103.9799957
-5.3812046	5.0752001	104.6199951
-5.3812046	5.1240001	103.9799957
-5.3812046	5.1728001	103.5000000
-5.3812046	5.2216001	103.1999969
-5.3812046	5.2704000	101.5199966
-5.3812046	5.3192000	105.3799973
-5.3812046	5.3680000	106.8600006
-5.3812046	5.4168000	104.7699966
-5.3812046	5.4656000	106.2600021
-5.3812046	5.5144000	106.7299957
-5.3812046	5.5632000	104.5299988
-5.3812046	5.6120000	102.2299957
-5.3812046	5.6608000	105.4300003
-5.3812046	5.7096000	102.7900009
-5.3812046	5.7584000	102.0499954
-5.3812046	5.8072000	99.7500000
-5.3812046	5.8559999	101.2099991
-5.3812046	5.9047999	102.2699966
-5.3812046	5.9535999	103.2200012
-5.3812046	6.0023999	104.9599991
-5.3812046	6.0511999	105.9000015
-5.3812046	6.0999999	104.7200012
-5.3812046	6.1487999	101.1800003
-5.3812046	6.1975999	105.3300018
-5.3812046	6.2463999	106.7799988
-5.3812046	6.2951999	105.7799988
-5.3812046	6.3439999	105.8499985
-5.3812046	6.3927999	106.4199982
-5.3812046	6.4415998	105.0999985
-5.3812046	6.4903998	104.6100006
-5.3812046	6.5391998	107.6999969
-5.3812046	6.5879998	106.5000000
-5.3812046	6.6367998	104.1599960
-5.3812046	6.6855998	103.1100006
-5.3812046	6.7343998	102.9499969
-5.3812046	6.7831998	103.8899994
-5.3812046	6.8319998	103.9300003
-5.3812046	6.8807998	102.0800018
-5.3812046	6.9295998	103.4599991
-5.3812046	6.9783998	105.1599960
-5.3812046	7.0271997	106.9000015
-5.3812046	7.0759997	106.0899963
-5.3812046	7.1247997	103.9000015
-5.3812046	7.1735997	99.5800018
-5.3812046	7.2223997	103.8399963
-5.3812046	7.2711997	104.3600006
-5.3812046	7.3199997	101.6500015
-5.3812046	7.3687997	102.2900009
-5.3812046	7.4175997	104.3899994
-5.3812046	7.4663997	104.7900009
-5.3812046	7.5151997	103.4599991
-5.3812046	7.5639997	99.9199982
-5.3812046	7.6127996	101.4499969

-5.3812046	7.6615996	102.2600021
-5.3812046	7.7103996	104.1399994
-5.3812046	7.7591996	105.5299988
-5.3812046	7.8079996	101.4599991
-5.3812046	7.8567996	102.7099991
-5.3812046	7.9056001	104.4899979
-5.3812046	7.9544001	103.5899963
-5.3812046	8.0032005	103.3199997
-5.3812046	8.0520000	105.3499985
-5.3812046	8.1008005	103.9799957
-5.3812046	8.1496000	99.7299957
-5.3812046	8.1984005	103.3600006
-5.3812046	8.2472000	104.5299988
-5.3812046	8.2960005	100.0499954
-5.3812046	8.3448000	100.7999954
-5.3812046	8.3936005	103.7099991
-5.3812046	8.4424000	101.7500000
-5.3812046	8.4912004	98.7500000
-5.3812046	8.5400000	99.4899979
-5.3812046	8.5888004	102.7200012
-5.3812046	8.6375999	100.5100021
-5.3812046	8.6864004	99.1599960
-5.3812046	8.7351999	101.7500000
-5.3812046	8.7840004	102.9700012
-5.3812046	8.8327999	103.1899948
-5.3812046	8.8816004	105.0000000
-5.3812046	8.9303999	106.3600006
-5.3812046	8.9792004	103.5800018
-5.3812046	9.0279999	100.1899948
-5.3812046	9.0768003	101.4399948
-5.3812046	9.1255999	104.3699951
-5.3812046	9.1744003	104.2500000
-5.3812046	9.2231998	101.5499954
-5.3812046	9.2720003	101.0499954
-5.3812046	9.3207998	101.8300018
-5.3812046	9.3696003	104.1199951
-5.3812046	9.4184008	102.7200012
-5.3812046	9.4672003	105.3499985
-5.3812046	9.5160007	105.6299973
-5.3812046	9.5648003	103.9300003
-5.3812046	9.6136007	101.6100006
-5.3812046	9.6624002	103.4700012
-5.3812046	9.7112007	98.7799988
-5.3812046	9.7600002	99.7699966
-5.3812046	9.8088007	101.4399948
-5.3812046	9.8576002	105.5899963
-5.3812046	9.9064007	104.5299988
-5.3812046	9.9552002	103.5599976
-5.3812046	10.0040007	105.1100006
-5.3812046	10.0528002	105.9599991
-5.3812046	10.1016006	100.4599991
-5.3812046	10.1504002	103.9199982
-5.3812046	10.1992006	102.0999985
-5.3812046	10.2480001	103.4199982
-5.3812046	10.2968006	104.3399963
-5.3812046	10.3456001	103.1899948
-5.3812046	10.3944006	101.6199951
-5.3812046	10.4432001	102.5800018

-5.3812046	10.4920006	104.1599960
-5.3812046	10.5408001	103.6899948
-5.3812046	10.5896006	104.7299957
-5.3812046	10.6384001	103.6800003
-5.3812046	10.6872005	103.2500000
-5.3812046	10.7360001	98.9499969
-5.3812046	10.7848005	104.7900009
-5.3812046	10.8336000	106.1599960
-5.3812046	10.8824005	105.9700012
-5.3812046	10.9312000	105.5299988
-5.3812046	10.9800005	102.5999985
-5.3812046	11.0288000	99.7200012
-5.3812046	11.0776005	101.0000000
-5.3812046	11.1264000	102.6699982
-5.3812046	11.1752005	105.4799957
-5.3812046	11.2240000	106.4300003
-5.3812046	11.2728004	105.4300003
-5.3812046	11.3216000	104.2099991
-5.3812046	11.3704004	103.5800018
-5.3812046	11.4191999	102.3899994
-5.3812046	11.4680004	103.1899948
-5.3812046	11.5167999	100.0199966
-5.3812046	11.5656004	99.9899979
-5.3812046	11.6143999	100.9899979
-5.3812046	11.6632004	102.4000015
-5.3812046	11.7119999	103.7799988
-5.3812046	11.7608004	100.9399948
-5.3812046	11.8095999	101.1199951
-5.3812046	11.8584003	106.1299973
-5.3812046	11.9071999	106.9099960
-5.3812046	11.9560003	107.2399979
-5.3812046	12.0047998	105.0100021
-5.3812046	12.0536003	103.5400009
-5.3812046	12.1023998	104.5699997
-5.3812046	12.1512003	104.6100006
-5.3812046	12.1999998	104.9099960
-5.3812046	12.2488003	104.1999969
-5.3812046	12.2975998	102.9499969
-5.3812046	12.3464003	100.4799957
-5.3812046	12.3951998	100.2500000
-5.3812046	12.4440002	100.1699982
-5.3812046	12.4927998	101.1699982
-5.3812046	12.5416002	103.7500000
-5.3812046	12.5904007	103.5400009
-5.3812046	12.6392002	104.5100021
-5.3812046	12.6880007	102.8300018
-5.3812046	12.7368002	103.0800018
-5.3812046	12.7856007	105.5699997
-5.3812046	12.8344002	104.4199982
-5.3812046	12.8832006	104.5000000
-5.3812046	12.9320002	104.6800003
-5.3812046	12.9808006	104.5599976
-5.3812046	13.0296001	102.3099976
-5.3812046	13.0784006	101.6800003
-5.3812046	13.1272001	106.2500000
-5.3812046	13.1760006	105.4599991
-5.3812046	13.2248001	100.0400009
-5.3812046	13.2736006	103.9700012

-5.3812046	13.3224001	104.9399948
-5.3812046	13.3712006	101.7200012
-5.3812046	13.4200001	103.9799957
-5.3812046	13.4688005	104.0199966
-5.3812046	13.5176001	102.9199982
-5.3812046	13.5664005	102.9899979
-5.3812046	13.6152000	103.8799973
-5.3812046	13.6640005	104.9700012
-5.3812046	13.7128000	101.7799988
-5.3812046	13.7616005	100.0699997
-5.3812046	13.8104000	101.8499985
-5.3812046	13.8592005	103.4399948
-5.3812046	13.9080000	103.3499985
-5.3812046	13.9568005	104.1299973
-5.3812046	14.0056000	102.1100006
-5.3812046	14.0544004	104.0199966
-5.3812046	14.1032000	106.3799973
-5.3812046	14.1520004	104.1999969
-5.3812046	14.2007999	101.0699997
-5.3812046	14.2496004	102.0699997
-5.3812046	14.2983999	101.9599991
-5.3812046	14.3472004	102.9899979
-5.3812046	14.3959999	102.1800003
-5.3812046	14.4448004	98.6199951
-5.3812046	14.4935999	101.1500015
-5.3812046	14.5424004	102.1899948
-5.3812046	14.5911999	102.0000000
-5.3812046	14.6400003	101.7600021
-5.3812046	14.6887999	105.7299957
-5.3812046	14.7376003	105.4899979
-5.3812046	14.7863998	104.9300003
-5.3812046	14.8352003	100.2600021
-5.3812046	14.8839998	102.0199966
-5.3812046	14.9328003	99.8099976
-5.3812046	14.9815998	101.7500000
-5.3812046	15.0304003	101.8600006
-5.3750687	0.3416000	105.5499954
-5.3750687	0.3904000	103.9000015
-5.3750687	0.4392000	102.1599960
-5.3750687	0.4880000	105.3600006
-5.3750687	0.5368000	106.7299957
-5.3750687	0.5856000	106.2799988
-5.3750687	0.6344000	107.4399948
-5.3750687	0.6832000	106.3099976
-5.3750687	0.7320000	99.4399948
-5.3750687	0.7808000	102.7999954
-5.3750687	0.8296000	103.6999969
-5.3750687	0.8784000	105.1899948
-5.3750687	0.9272000	107.2200012
-5.3750687	0.9760000	104.4000015
-5.3750687	1.0247999	103.4099960
-5.3750687	1.0735999	103.0000000
-5.3750687	1.1223999	105.1399994
-5.3750687	1.1711999	102.1599960
-5.3750687	1.2200000	103.5499954
-5.3750687	1.2688000	105.4599991
-5.3750687	1.3176000	108.6299973
-5.3750687	1.3664000	107.7600021

-5.3750687	1.4152000	106.1399994
-5.3750687	1.4640000	106.0100021
-5.3750687	1.5128000	107.9399948
-5.3750687	1.5616000	107.1899948
-5.3750687	1.6104000	105.6199951
-5.3750687	1.6592000	108.7900009
-5.3750687	1.7079999	106.3899994
-5.3750687	1.7567999	105.0299988
-5.3750687	1.8055999	104.6299973
-5.3750687	1.8543999	107.1299973
-5.3750687	1.9031999	103.1399994
-5.3750687	1.9519999	105.0100021
-5.3750687	2.0007999	104.6100006
-5.3750687	2.0495999	106.6199951
-5.3750687	2.0983999	107.7799988
-5.3750687	2.1471999	105.9599991
-5.3750687	2.1959999	106.0499954
-5.3750687	2.2447999	104.1500015
-5.3750687	2.2935998	103.9199982
-5.3750687	2.3423998	103.0299988
-5.3750687	2.3912001	102.4599991
-5.3750687	2.4400001	101.9700012
-5.3750687	2.4888000	102.4300003
-5.3750687	2.5376000	104.0199966
-5.3750687	2.5864000	105.5100021
-5.3750687	2.6352000	104.0199966
-5.3750687	2.6840000	99.7699966
-5.3750687	2.7328000	102.9499969
-5.3750687	2.7816000	102.4199982
-5.3750687	2.8304000	100.7799988
-5.3750687	2.8792000	104.5599976
-5.3750687	2.9280000	107.3699951
-5.3750687	2.9768000	107.0599976
-5.3750687	3.0256000	105.0699997
-5.3750687	3.0743999	105.2200012
-5.3750687	3.1231999	101.9199982
-5.3750687	3.1719999	103.0599976
-5.3750687	3.2207999	102.9799957
-5.3750687	3.2695999	102.6800003
-5.3750687	3.3183999	100.1399994
-5.3750687	3.3671999	100.4000015
-5.3750687	3.4159999	102.4899979
-5.3750687	3.4647999	103.1299973
-5.3750687	3.5135999	103.6100006
-5.3750687	3.5623999	105.2900009
-5.3750687	3.6111999	103.0100021
-5.3750687	3.6599998	105.7699966
-5.3750687	3.7087998	104.5699997
-5.3750687	3.7575998	104.1399994
-5.3750687	3.8063998	101.5899963
-5.3750687	3.8551998	99.4899979
-5.3750687	3.9039998	99.9099960
-5.3750687	3.9527998	102.4499969
-5.3750687	4.0016003	104.3099976
-5.3750687	4.0503998	105.6100006
-5.3750687	4.0991998	104.0499954
-5.3750687	4.1479998	104.5299988
-5.3750687	4.1967998	101.2099991

-5.3750687	4.2455997	103.3399963
-5.3750687	4.2943997	104.3600006
-5.3750687	4.3431997	106.5899963
-5.3750687	4.3919997	102.8399963
-5.3750687	4.4407997	103.6699982
-5.3750687	4.4895997	104.2999954
-5.3750687	4.5383997	105.7500000
-5.3750687	4.5871997	101.8699951
-5.3750687	4.6359997	109.5299988
-5.3750687	4.6847997	114.6100006
-5.3750687	4.7336001	114.3899994
-5.3750687	4.7824001	108.3099976
-5.3750687	4.8312001	107.5400009
-5.3750687	4.8800001	110.1599960
-5.3750687	4.9288001	109.7900009
-5.3750687	4.9776001	106.1500015
-5.3750687	5.0264001	103.5199966
-5.3750687	5.0752001	103.5299988
-5.3750687	5.1240001	102.7999954
-5.3750687	5.1728001	103.8600006
-5.3750687	5.2216001	102.2999954
-5.3750687	5.2704000	101.4300003
-5.3750687	5.3192000	105.7799988
-5.3750687	5.3680000	106.9099960
-5.3750687	5.4168000	105.6999969
-5.3750687	5.4656000	106.1599960
-5.3750687	5.5144000	106.8099976
-5.3750687	5.5632000	104.7600021
-5.3750687	5.6120000	101.2500000
-5.3750687	5.6608000	105.1800003
-5.3750687	5.7096000	102.8699951
-5.3750687	5.7584000	101.8199997
-5.3750687	5.8072000	100.3499985
-5.3750687	5.8559999	101.6599960
-5.3750687	5.9047999	103.3499985
-5.3750687	5.9535999	103.8899994
-5.3750687	6.0023999	105.4300003
-5.3750687	6.0511999	105.8499985
-5.3750687	6.0999999	105.1199951
-5.3750687	6.1487999	100.7799988
-5.3750687	6.1975999	105.2299957
-5.3750687	6.2463999	106.9499969
-5.3750687	6.2951999	106.0100021
-5.3750687	6.3439999	106.2200012
-5.3750687	6.3927999	106.6299973
-5.3750687	6.4415998	104.7399979
-5.3750687	6.4903998	104.3399963
-5.3750687	6.5391998	107.5100021
-5.3750687	6.5879998	105.5599976
-5.3750687	6.6367998	102.9399948
-5.3750687	6.6855998	103.5000000
-5.3750687	6.7343998	103.5400009
-5.3750687	6.7831998	102.2600021
-5.3750687	6.8319998	102.4199982
-5.3750687	6.8807998	101.9099960
-5.3750687	6.9295998	102.6699982
-5.3750687	6.9783998	104.5100021
-5.3750687	7.0271997	106.1599960

-5.3750687	7.0759997	105.5499954
-5.3750687	7.1247997	103.9700012
-5.3750687	7.1735997	97.3799973
-5.3750687	7.2223997	104.1699982
-5.3750687	7.2711997	104.9700012
-5.3750687	7.3199997	101.7399979
-5.3750687	7.3687997	102.5999985
-5.3750687	7.4175997	103.8899994
-5.3750687	7.4663997	104.6999969
-5.3750687	7.5151997	104.1399994
-5.3750687	7.5639997	101.4099960
-5.3750687	7.6127996	102.1199951
-5.3750687	7.6615996	102.9899979
-5.3750687	7.7103996	104.6699982
-5.3750687	7.7591996	105.2999954
-5.3750687	7.8079996	101.6699982
-5.3750687	7.8567996	102.9000015
-5.3750687	7.9056001	104.3399963
-5.3750687	7.9544001	103.5000000
-5.3750687	8.0032005	103.3899994
-5.3750687	8.0520000	105.2900009
-5.3750687	8.1008005	105.0800018
-5.3750687	8.1496000	101.7999954
-5.3750687	8.1984005	103.7900009
-5.3750687	8.2472000	104.6699982
-5.3750687	8.2960005	98.2200012
-5.3750687	8.3448000	101.3899994
-5.3750687	8.3936005	103.5599976
-5.3750687	8.4424000	100.7699966
-5.3750687	8.4912004	97.3699951
-5.3750687	8.5400000	99.8799973
-5.3750687	8.5888004	103.0100021
-5.3750687	8.6375999	101.3399963
-5.3750687	8.6864004	100.0999985
-5.3750687	8.7351999	102.9000015
-5.3750687	8.7840004	103.4099960
-5.3750687	8.8327999	103.5199966
-5.3750687	8.8816004	104.5999985
-5.3750687	8.9303999	105.9300003
-5.3750687	8.9792004	103.2299957
-5.3750687	9.0279999	100.1500015
-5.3750687	9.0768003	102.1800003
-5.3750687	9.1255999	104.6699982
-5.3750687	9.1744003	104.2200012
-5.3750687	9.2231998	101.2200012
-5.3750687	9.2720003	100.7699966
-5.3750687	9.3207998	102.0599976
-5.3750687	9.3696003	103.5400009
-5.3750687	9.4184008	102.8399963
-5.3750687	9.4672003	105.4399948
-5.3750687	9.5160007	106.1100006
-5.3750687	9.5648003	104.6100006
-5.3750687	9.6136007	101.6500015
-5.3750687	9.6624002	103.0400009
-5.3750687	9.7112007	99.4899979
-5.3750687	9.7600002	100.1699982
-5.3750687	9.8088007	101.5699997
-5.3750687	9.8576002	105.7900009

-5.3750687	9.9064007	104.2200012
-5.3750687	9.9552002	103.7200012
-5.3750687	10.0040007	105.1100006
-5.3750687	10.0528002	106.1599960
-5.3750687	10.1016006	98.4799957
-5.3750687	10.1504002	104.0899963
-5.3750687	10.1992006	103.2200012
-5.3750687	10.2480001	103.5699997
-5.3750687	10.2968006	104.1500015
-5.3750687	10.3456001	103.5699997
-5.3750687	10.3944006	101.8799973
-5.3750687	10.4432001	103.3699951
-5.3750687	10.4920006	104.3099976
-5.3750687	10.5408001	102.9599991
-5.3750687	10.5896006	104.8099976
-5.3750687	10.6384001	103.1599960
-5.3750687	10.6872005	103.0999985
-5.3750687	10.7360001	99.3699951
-5.3750687	10.7848005	105.1299973
-5.3750687	10.8336000	105.7099991
-5.3750687	10.8824005	106.1199951
-5.3750687	10.9312000	106.0699997
-5.3750687	10.9800005	103.2799988
-5.3750687	11.0288000	100.4899979
-5.3750687	11.0776005	101.9599991
-5.3750687	11.1264000	102.6999969
-5.3750687	11.1752005	104.8499985
-5.3750687	11.2240000	106.3499985
-5.3750687	11.2728004	105.5699997
-5.3750687	11.3216000	103.8199997
-5.3750687	11.3704004	102.7999954
-5.3750687	11.4191999	103.1800003
-5.3750687	11.4680004	103.9300003
-5.3750687	11.5167999	100.7399979
-5.3750687	11.5656004	100.9599991
-5.3750687	11.6143999	100.5499954
-5.3750687	11.6632004	102.2099991
-5.3750687	11.7119999	104.0499954
-5.3750687	11.7608004	101.5100021
-5.3750687	11.8095999	101.7900009
-5.3750687	11.8584003	106.5499954
-5.3750687	11.9071999	107.3600006
-5.3750687	11.9560003	107.0000000
-5.3750687	12.0047998	104.7399979
-5.3750687	12.0536003	103.6699982
-5.3750687	12.1023998	103.5400009
-5.3750687	12.1512003	104.5899963
-5.3750687	12.1999998	106.1599960
-5.3750687	12.2488003	105.5899963
-5.3750687	12.2975998	103.3799973
-5.3750687	12.3464003	100.5000000
-5.3750687	12.3951998	100.7099991
-5.3750687	12.4440002	100.1299973
-5.3750687	12.4927998	100.2799988
-5.3750687	12.5416002	102.9300003
-5.3750687	12.5904007	104.1299973
-5.3750687	12.6392002	104.7699966
-5.3750687	12.6880007	102.7200012

-5.3750687	12.7368002	103.3799973
-5.3750687	12.7856007	105.5899963
-5.3750687	12.8344002	104.5800018
-5.3750687	12.8832006	104.1800003
-5.3750687	12.9320002	104.5999985
-5.3750687	12.9808006	104.3600006
-5.3750687	13.0296001	102.1399994
-5.3750687	13.0784006	102.1299973
-5.3750687	13.1272001	106.2999954
-5.3750687	13.1760006	105.8600006
-5.3750687	13.2248001	99.6399994
-5.3750687	13.2736006	104.1999969
-5.3750687	13.3224001	104.8799973
-5.3750687	13.3712006	101.8399963
-5.3750687	13.4200001	103.8399963
-5.3750687	13.4688005	104.2900009
-5.3750687	13.5176001	103.8899994
-5.3750687	13.5664005	102.9599991
-5.3750687	13.6152000	103.9899979
-5.3750687	13.6640005	104.6100006
-5.3750687	13.7128000	102.1100006
-5.3750687	13.7616005	100.3600006
-5.3750687	13.8104000	102.5400009
-5.3750687	13.8592005	103.8199997
-5.3750687	13.9080000	103.8199997
-5.3750687	13.9568005	104.2099991
-5.3750687	14.0056000	102.1299973
-5.3750687	14.0544004	103.8499985
-5.3750687	14.1032000	106.4599991
-5.3750687	14.1520004	103.4599991
-5.3750687	14.2007999	100.9700012
-5.3750687	14.2496004	102.5400009
-5.3750687	14.2983999	103.3600006
-5.3750687	14.3472004	104.2500000
-5.3750687	14.3959999	103.2600021
-5.3750687	14.4448004	98.5999985
-5.3750687	14.4935999	101.2500000
-5.3750687	14.5424004	101.7900009
-5.3750687	14.5911999	101.5999985
-5.3750687	14.6400003	102.3699951
-5.3750687	14.6887999	105.5999985
-5.3750687	14.7376003	105.6500015
-5.3750687	14.7863998	105.2099991
-5.3750687	14.8352003	101.7900009
-5.3750687	14.8839998	102.3399963
-5.3750687	14.9328003	100.8699951
-5.3750687	14.9815998	101.7200012
-5.3750687	15.0304003	102.3499985
-5.3689327	0.3416000	104.6899948
-5.3689327	0.3904000	103.2900009
-5.3689327	0.4392000	101.4399948
-5.3689327	0.4880000	105.1699982
-5.3689327	0.5368000	106.3199997
-5.3689327	0.5856000	107.0599976
-5.3689327	0.6344000	108.5299988
-5.3689327	0.6832000	107.3099976
-5.3689327	0.7320000	101.8699951
-5.3689327	0.7808000	104.8699951

-5.3689327	0.8296000	105.3399963
-5.3689327	0.8784000	106.0299988
-5.3689327	0.9272000	108.3799973
-5.3689327	0.9760000	106.3199997
-5.3689327	1.0247999	104.6599960
-5.3689327	1.0735999	103.7399979
-5.3689327	1.1223999	105.6199951
-5.3689327	1.1711999	101.8600006
-5.3689327	1.2200000	102.9700012
-5.3689327	1.2688000	105.8399963
-5.3689327	1.3176000	109.3799973
-5.3689327	1.3664000	108.5199966
-5.3689327	1.4152000	107.7900009
-5.3689327	1.4640000	108.2900009
-5.3689327	1.5128000	108.2699966
-5.3689327	1.5616000	106.6800003
-5.3689327	1.6104000	103.8600006
-5.3689327	1.6592000	108.2099991
-5.3689327	1.7079999	106.2099991
-5.3689327	1.7567999	104.8099976
-5.3689327	1.8055999	104.5400009
-5.3689327	1.8543999	107.3499985
-5.3689327	1.9031999	104.1800003
-5.3689327	1.9519999	104.4700012
-5.3689327	2.0007999	104.5100021
-5.3689327	2.0495999	107.4599991
-5.3689327	2.0983999	107.4000015
-5.3689327	2.1471999	105.4899979
-5.3689327	2.1959999	105.9300003
-5.3689327	2.2447999	103.9099960
-5.3689327	2.2935998	104.1500015
-5.3689327	2.3423998	103.1100006
-5.3689327	2.3912001	102.2999954
-5.3689327	2.4400001	102.0800018
-5.3689327	2.4888000	102.4499969
-5.3689327	2.5376000	102.8399963
-5.3689327	2.5864000	105.1500015
-5.3689327	2.6352000	104.1999969
-5.3689327	2.6840000	100.0699997
-5.3689327	2.7328000	102.5100021
-5.3689327	2.7816000	101.6599960
-5.3689327	2.8304000	100.9300003
-5.3689327	2.8792000	105.2399979
-5.3689327	2.9280000	108.0100021
-5.3689327	2.9768000	107.4499969
-5.3689327	3.0256000	104.4700012
-5.3689327	3.0743999	105.2699966
-5.3689327	3.1231999	101.7399979
-5.3689327	3.1719999	102.0999985
-5.3689327	3.2207999	103.5000000
-5.3689327	3.2695999	102.5400009
-5.3689327	3.3183999	99.2900009
-5.3689327	3.3671999	99.5400009
-5.3689327	3.4159999	101.9300003
-5.3689327	3.4647999	103.0000000
-5.3689327	3.5135999	103.1899948
-5.3689327	3.5623999	104.4399948
-5.3689327	3.6111999	101.1800003

-5.3689327	3.6599998	104.7299957
-5.3689327	3.7087998	104.2900009
-5.3689327	3.7575998	103.4899979
-5.3689327	3.8063998	100.9499969
-5.3689327	3.8551998	100.3499985
-5.3689327	3.9039998	99.4000015
-5.3689327	3.9527998	102.9399948
-5.3689327	4.0016003	104.3699951
-5.3689327	4.0503998	103.9700012
-5.3689327	4.0991998	101.9700012
-5.3689327	4.1479998	103.6199951
-5.3689327	4.1967998	99.9000015
-5.3689327	4.2455997	103.5699997
-5.3689327	4.2943997	104.2500000
-5.3689327	4.3431997	106.4499969
-5.3689327	4.3919997	103.3699951
-5.3689327	4.4407997	104.5999985
-5.3689327	4.4895997	104.5400009
-5.3689327	4.5383997	105.1100006
-5.3689327	4.5871997	101.4499969
-5.3689327	4.6359997	108.8099976
-5.3689327	4.6847997	113.8699951
-5.3689327	4.7336001	113.7200012
-5.3689327	4.7824001	108.4799957
-5.3689327	4.8312001	107.4300003
-5.3689327	4.8800001	109.1399994
-5.3689327	4.9288001	108.3600006
-5.3689327	4.9776001	105.5100021
-5.3689327	5.0264001	102.4700012
-5.3689327	5.0752001	102.3300018
-5.3689327	5.1240001	102.0499954
-5.3689327	5.1728001	103.5800018
-5.3689327	5.2216001	101.3699951
-5.3689327	5.2704000	102.5400009
-5.3689327	5.3192000	106.6699982
-5.3689327	5.3680000	107.5800018
-5.3689327	5.4168000	106.5699997
-5.3689327	5.4656000	106.5699997
-5.3689327	5.5144000	106.9799957
-5.3689327	5.5632000	104.9599991
-5.3689327	5.6120000	101.3399963
-5.3689327	5.6608000	104.3899994
-5.3689327	5.7096000	102.5100021
-5.3689327	5.7584000	101.9099960
-5.3689327	5.8072000	100.8399963
-5.3689327	5.8559999	101.9799957
-5.3689327	5.9047999	103.5299988
-5.3689327	5.9535999	104.2099991
-5.3689327	6.0023999	105.5899963
-5.3689327	6.0511999	105.6699982
-5.3689327	6.0999999	105.9499969
-5.3689327	6.1487999	101.5000000
-5.3689327	6.1975999	104.9799957
-5.3689327	6.2463999	107.2200012
-5.3689327	6.2951999	106.6500015
-5.3689327	6.3439999	106.9399948
-5.3689327	6.3927999	106.9599991
-5.3689327	6.4415998	104.2799988

-5.3689327	6.4903998	103.8899994
-5.3689327	6.5391998	107.0199966
-5.3689327	6.5879998	103.8300018
-5.3689327	6.6367998	101.2299957
-5.3689327	6.6855998	102.7600021
-5.3689327	6.7343998	103.5899963
-5.3689327	6.7831998	101.0400009
-5.3689327	6.8319998	101.2900009
-5.3689327	6.8807998	102.9399948
-5.3689327	6.9295998	102.5400009
-5.3689327	6.9783998	103.9700012
-5.3689327	7.0271997	105.7600021
-5.3689327	7.0759997	104.9700012
-5.3689327	7.1247997	103.2099991
-5.3689327	7.1735997	93.9300003
-5.3689327	7.2223997	103.0400009
-5.3689327	7.2711997	105.2999954
-5.3689327	7.3199997	102.0000000
-5.3689327	7.3687997	102.7799988
-5.3689327	7.4175997	103.7699966
-5.3689327	7.4663997	104.9399948
-5.3689327	7.5151997	105.1899948
-5.3689327	7.5639997	103.1199951
-5.3689327	7.6127996	102.6699982
-5.3689327	7.6615996	103.7099991
-5.3689327	7.7103996	105.3699951
-5.3689327	7.7591996	105.1699982
-5.3689327	7.8079996	102.0699997
-5.3689327	7.8567996	102.9399948
-5.3689327	7.9056001	103.7900009
-5.3689327	7.9544001	103.7500000
-5.3689327	8.0032005	103.8399963
-5.3689327	8.0520000	105.4000015
-5.3689327	8.1008005	106.0100021
-5.3689327	8.1496000	103.7999954
-5.3689327	8.1984005	104.0999985
-5.3689327	8.2472000	104.4899979
-5.3689327	8.2960005	96.4499969
-5.3689327	8.3448000	102.0999985
-5.3689327	8.3936005	103.5299988
-5.3689327	8.4424000	100.5699997
-5.3689327	8.4912004	96.2099991
-5.3689327	8.5400000	100.0999985
-5.3689327	8.5888004	102.5499954
-5.3689327	8.6375999	101.6800003
-5.3689327	8.6864004	101.6599960
-5.3689327	8.7351999	103.8399963
-5.3689327	8.7840004	104.0599976
-5.3689327	8.8327999	104.0999985
-5.3689327	8.8816004	103.6899948
-5.3689327	8.9303999	104.7699966
-5.3689327	8.9792004	103.2900009
-5.3689327	9.0279999	100.8600006
-5.3689327	9.0768003	102.6899948
-5.3689327	9.1255999	104.8099976
-5.3689327	9.1744003	104.3600006
-5.3689327	9.2231998	100.8099976
-5.3689327	9.2720003	100.7399979

-5.3689327	9.3207998	102.5699997
-5.3689327	9.3696003	102.5699997
-5.3689327	9.4184008	103.1299973
-5.3689327	9.4672003	105.8199997
-5.3689327	9.5160007	106.5100021
-5.3689327	9.5648003	104.9000015
-5.3689327	9.6136007	101.4499969
-5.3689327	9.6624002	102.4499969
-5.3689327	9.7112007	100.0000000
-5.3689327	9.7600002	101.4399948
-5.3689327	9.8088007	101.7900009
-5.3689327	9.8576002	105.6599960
-5.3689327	9.9064007	103.5599976
-5.3689327	9.9552002	103.7699966
-5.3689327	10.0040007	104.8699951
-5.3689327	10.0528002	106.3399963
-5.3689327	10.1016006	99.4700012
-5.3689327	10.1504002	104.6999969
-5.3689327	10.1992006	104.5899963
-5.3689327	10.2480001	104.0599976
-5.3689327	10.2968006	103.9899979
-5.3689327	10.3456001	104.1199951
-5.3689327	10.3944006	103.0199966
-5.3689327	10.4432001	103.5299988
-5.3689327	10.4920006	104.1599960
-5.3689327	10.5408001	101.6599960
-5.3689327	10.5896006	104.3499985
-5.3689327	10.6384001	102.8300018
-5.3689327	10.6872005	103.2999954
-5.3689327	10.7360001	100.4599991
-5.3689327	10.7848005	104.8300018
-5.3689327	10.8336000	104.5000000
-5.3689327	10.8824005	105.5400009
-5.3689327	10.9312000	106.0499954
-5.3689327	10.9800005	103.9199982
-5.3689327	11.0288000	101.3799973
-5.3689327	11.0776005	103.0499954
-5.3689327	11.1264000	102.2699966
-5.3689327	11.1752005	104.0899963
-5.3689327	11.2240000	106.2600021
-5.3689327	11.2728004	105.6399994
-5.3689327	11.3216000	103.3099976
-5.3689327	11.3704004	101.8799973
-5.3689327	11.4191999	103.7699966
-5.3689327	11.4680004	104.5599976
-5.3689327	11.5167999	101.0599976
-5.3689327	11.5656004	101.9499969
-5.3689327	11.6143999	101.5899963
-5.3689327	11.6632004	102.2200012
-5.3689327	11.7119999	104.0899963
-5.3689327	11.7608004	102.3699951
-5.3689327	11.8095999	103.2600021
-5.3689327	11.8584003	106.9700012
-5.3689327	11.9071999	107.3199997
-5.3689327	11.9560003	105.7699966
-5.3689327	12.0047998	104.1500015
-5.3689327	12.0536003	103.6500015
-5.3689327	12.1023998	101.9099960

-5.3689327	12.1512003	104.1699982
-5.3689327	12.1999998	107.1800003
-5.3689327	12.2488003	106.5299988
-5.3689327	12.2975998	103.2699966
-5.3689327	12.3464003	100.0999985
-5.3689327	12.3951998	101.0899963
-5.3689327	12.4440002	101.2099991
-5.3689327	12.4927998	100.7299957
-5.3689327	12.5416002	102.9499969
-5.3689327	12.5904007	104.9599991
-5.3689327	12.6392002	104.8399963
-5.3689327	12.6880007	102.6500015
-5.3689327	12.7368002	103.6599960
-5.3689327	12.7856007	105.2799988
-5.3689327	12.8344002	104.7399979
-5.3689327	12.8832006	104.3699951
-5.3689327	12.9320002	104.8899994
-5.3689327	12.9808006	104.0199966
-5.3689327	13.0296001	101.5100021
-5.3689327	13.0784006	102.0100021
-5.3689327	13.1272001	106.1899948
-5.3689327	13.1760006	106.2299957
-5.3689327	13.2248001	100.4599991
-5.3689327	13.2736006	103.8799973
-5.3689327	13.3224001	104.1399994
-5.3689327	13.3712006	101.7399979
-5.3689327	13.4200001	103.6999969
-5.3689327	13.4688005	105.0299988
-5.3689327	13.5176001	105.2699966
-5.3689327	13.5664005	103.6800003
-5.3689327	13.6152000	104.1299973
-5.3689327	13.6640005	103.6800003
-5.3689327	13.7128000	101.6599960
-5.3689327	13.7616005	100.2699966
-5.3689327	13.8104000	103.0999985
-5.3689327	13.8592005	103.7399979
-5.3689327	13.9080000	103.9799957
-5.3689327	13.9568005	104.0400009
-5.3689327	14.0056000	102.1199951
-5.3689327	14.0544004	103.4000015
-5.3689327	14.1032000	105.9599991
-5.3689327	14.1520004	102.6699982
-5.3689327	14.2007999	101.9000015
-5.3689327	14.2496004	103.7399979
-5.3689327	14.2983999	103.9099960
-5.3689327	14.3472004	104.3300018
-5.3689327	14.3959999	103.4399948
-5.3689327	14.4448004	98.9099960
-5.3689327	14.4935999	101.1999969
-5.3689327	14.5424004	101.4000015
-5.3689327	14.5911999	102.2299957
-5.3689327	14.6400003	103.1800003
-5.3689327	14.6887999	105.4899979
-5.3689327	14.7376003	105.9499969
-5.3689327	14.7863998	105.6299973
-5.3689327	14.8352003	103.1100006
-5.3689327	14.8839998	102.5100021
-5.3689327	14.9328003	100.5199966

-5.3689327	14.9815998	101.2900009
-5.3689327	15.0304003	103.0100021
-5.3627968	0.3416000	104.6199951
-5.3627968	0.3904000	103.0499954
-5.3627968	0.4392000	101.0000000
-5.3627968	0.4880000	104.7699966
-5.3627968	0.5368000	105.1199951
-5.3627968	0.5856000	106.8699951
-5.3627968	0.6344000	109.0100021
-5.3627968	0.6832000	108.0199966
-5.3627968	0.7320000	103.9899979
-5.3627968	0.7808000	106.5199966
-5.3627968	0.8296000	106.1599960
-5.3627968	0.8784000	106.0400009
-5.3627968	0.9272000	108.9799957
-5.3627968	0.9760000	107.8300018
-5.3627968	1.0247999	105.4300003
-5.3627968	1.0735999	104.0000000
-5.3627968	1.1223999	105.9399948
-5.3627968	1.1711999	102.4499969
-5.3627968	1.2200000	102.2099991
-5.3627968	1.2688000	105.6199951
-5.3627968	1.3176000	109.7200012
-5.3627968	1.3664000	108.7099991
-5.3627968	1.4152000	108.4799957
-5.3627968	1.4640000	108.7799988
-5.3627968	1.5128000	107.9499969
-5.3627968	1.5616000	105.8699951
-5.3627968	1.6104000	102.8099976
-5.3627968	1.6592000	107.5800018
-5.3627968	1.7079999	106.0899963
-5.3627968	1.7567999	105.2399979
-5.3627968	1.8055999	104.4899979
-5.3627968	1.8543999	107.1899948
-5.3627968	1.9031999	105.0299988
-5.3627968	1.9519999	104.1399994
-5.3627968	2.0007999	104.1999969
-5.3627968	2.0495999	107.9199982
-5.3627968	2.0983999	106.9799957
-5.3627968	2.1471999	106.1299973
-5.3627968	2.1959999	106.3099976
-5.3627968	2.2447999	103.2999954
-5.3627968	2.2935998	103.6399994
-5.3627968	2.3423998	102.5199966
-5.3627968	2.3912001	101.5599976
-5.3627968	2.4400001	102.7200012
-5.3627968	2.4888000	102.3300018
-5.3627968	2.5376000	102.3899994
-5.3627968	2.5864000	105.2299957
-5.3627968	2.6352000	104.4799957
-5.3627968	2.6840000	100.6299973
-5.3627968	2.7328000	102.4099960
-5.3627968	2.7816000	101.4000015
-5.3627968	2.8304000	101.7600021
-5.3627968	2.8792000	106.1999969
-5.3627968	2.9280000	108.7399979
-5.3627968	2.9768000	108.1299973
-5.3627968	3.0256000	104.5199966

-5.3627968	3.0743999	105.6500015
-5.3627968	3.1231999	102.2399979
-5.3627968	3.1719999	101.4000015
-5.3627968	3.2207999	103.8399963
-5.3627968	3.2695999	102.4599991
-5.3627968	3.3183999	98.6800003
-5.3627968	3.3671999	99.1100006
-5.3627968	3.4159999	101.9599991
-5.3627968	3.4647999	102.9599991
-5.3627968	3.5135999	103.1100006
-5.3627968	3.5623999	103.8199997
-5.3627968	3.6111999	100.9899979
-5.3627968	3.6599998	103.8799973
-5.3627968	3.7087998	103.5000000
-5.3627968	3.7575998	102.5400009
-5.3627968	3.8063998	101.0999985
-5.3627968	3.8551998	101.0400009
-5.3627968	3.9039998	98.7299957
-5.3627968	3.9527998	103.6500015
-5.3627968	4.0016003	104.9899979
-5.3627968	4.0503998	102.5999985
-5.3627968	4.0991998	100.1899948
-5.3627968	4.1479998	103.0899963
-5.3627968	4.1967998	98.4499969
-5.3627968	4.2455997	103.6899948
-5.3627968	4.2943997	104.1899948
-5.3627968	4.3431997	106.4000015
-5.3627968	4.3919997	103.6100006
-5.3627968	4.4407997	104.9300003
-5.3627968	4.4895997	104.0800018
-5.3627968	4.5383997	104.8099976
-5.3627968	4.5871997	101.0999985
-5.3627968	4.6359997	108.2900009
-5.3627968	4.6847997	113.3600006
-5.3627968	4.7336001	113.0800018
-5.3627968	4.7824001	108.4300003
-5.3627968	4.8312001	107.5599976
-5.3627968	4.8800001	108.3699951
-5.3627968	4.9288001	106.7699966
-5.3627968	4.9776001	104.6399994
-5.3627968	5.0264001	101.9099960
-5.3627968	5.0752001	101.7200012
-5.3627968	5.1240001	100.9499969
-5.3627968	5.1728001	102.5400009
-5.3627968	5.2216001	99.3799973
-5.3627968	5.2704000	103.0199966
-5.3627968	5.3192000	106.8799973
-5.3627968	5.3680000	107.4099960
-5.3627968	5.4168000	106.3600006
-5.3627968	5.4656000	106.4300003
-5.3627968	5.5144000	106.4399948
-5.3627968	5.5632000	105.7500000
-5.3627968	5.6120000	101.7099991
-5.3627968	5.6608000	103.2299957
-5.3627968	5.7096000	101.3799973
-5.3627968	5.7584000	102.0999985
-5.3627968	5.8072000	100.8699951
-5.3627968	5.8559999	101.5400009

-5.3627968	5.9047999	103.1899948
-5.3627968	5.9535999	104.5100021
-5.3627968	6.0023999	106.2500000
-5.3627968	6.0511999	105.3699951
-5.3627968	6.0999999	106.5100021
-5.3627968	6.1487999	102.2900009
-5.3627968	6.1975999	104.5699997
-5.3627968	6.2463999	107.0800018
-5.3627968	6.2951999	106.9199982
-5.3627968	6.3439999	107.1800003
-5.3627968	6.3927999	106.8300018
-5.3627968	6.4415998	103.3399963
-5.3627968	6.4903998	102.7399979
-5.3627968	6.5391998	105.5899963
-5.3627968	6.5879998	101.6399994
-5.3627968	6.6367998	100.2799988
-5.3627968	6.6855998	102.7999954
-5.3627968	6.7343998	103.7600021
-5.3627968	6.7831998	100.1599960
-5.3627968	6.8319998	101.2299957
-5.3627968	6.8807998	104.3699951
-5.3627968	6.9295998	102.8499985
-5.3627968	6.9783998	103.2099991
-5.3627968	7.0271997	104.9499969
-5.3627968	7.0759997	104.2799988
-5.3627968	7.1247997	103.1199951
-5.3627968	7.1735997	93.3099976
-5.3627968	7.2223997	102.8300018
-5.3627968	7.2711997	105.2299957
-5.3627968	7.3199997	101.6299973
-5.3627968	7.3687997	102.3199997
-5.3627968	7.4175997	103.2099991
-5.3627968	7.4663997	105.1299973
-5.3627968	7.5151997	106.2900009
-5.3627968	7.5639997	104.3099976
-5.3627968	7.6127996	103.4799957
-5.3627968	7.6615996	104.8300018
-5.3627968	7.7103996	105.9399948
-5.3627968	7.7591996	104.7399979
-5.3627968	7.8079996	102.5100021
-5.3627968	7.8567996	103.0899963
-5.3627968	7.9056001	102.9499969
-5.3627968	7.9544001	104.2299957
-5.3627968	8.0032005	104.3600006
-5.3627968	8.0520000	104.7500000
-5.3627968	8.1008005	105.9599991
-5.3627968	8.1496000	105.5400009
-5.3627968	8.1984005	104.3899994
-5.3627968	8.2472000	104.4199982
-5.3627968	8.2960005	95.9799957
-5.3627968	8.3448000	103.0999985
-5.3627968	8.3936005	104.0499954
-5.3627968	8.4424000	101.5800018
-5.3627968	8.4912004	96.6899948
-5.3627968	8.5400000	100.3099976
-5.3627968	8.5888004	102.1199951
-5.3627968	8.6375999	102.3199997
-5.3627968	8.6864004	103.3199997

-5.3627968	8.7351999	104.7600021
-5.3627968	8.7840004	104.4599991
-5.3627968	8.8327999	104.5100021
-5.3627968	8.8816004	103.1800003
-5.3627968	8.9303999	104.0699997
-5.3627968	8.9792004	103.2699966
-5.3627968	9.0279999	101.0400009
-5.3627968	9.0768003	103.1100006
-5.3627968	9.1255999	104.8600006
-5.3627968	9.1744003	104.0100021
-5.3627968	9.2231998	100.3399963
-5.3627968	9.2720003	101.4099960
-5.3627968	9.3207998	103.0800018
-5.3627968	9.3696003	100.9000015
-5.3627968	9.4184008	102.8699951
-5.3627968	9.4672003	106.0899963
-5.3627968	9.5160007	107.1199951
-5.3627968	9.5648003	105.7399979
-5.3627968	9.6136007	101.6999969
-5.3627968	9.6624002	102.4599991
-5.3627968	9.7112007	100.4599991
-5.3627968	9.7600002	102.4099960
-5.3627968	9.8088007	102.3300018
-5.3627968	9.8576002	106.0100021
-5.3627968	9.9064007	103.3799973
-5.3627968	9.9552002	103.6199951
-5.3627968	10.0040007	104.3300018
-5.3627968	10.0528002	106.5800018
-5.3627968	10.1016006	101.4499969
-5.3627968	10.1504002	106.0000000
-5.3627968	10.1992006	106.0800018
-5.3627968	10.2480001	104.7999954
-5.3627968	10.2968006	103.9700012
-5.3627968	10.3456001	104.6199951
-5.3627968	10.3944006	103.7600021
-5.3627968	10.4432001	103.0400009
-5.3627968	10.4920006	103.3600006
-5.3627968	10.5408001	100.7600021
-5.3627968	10.5896006	103.8600006
-5.3627968	10.6384001	102.7299957
-5.3627968	10.6872005	103.6199951
-5.3627968	10.7360001	101.3699951
-5.3627968	10.7848005	104.5400009
-5.3627968	10.8336000	103.6899948
-5.3627968	10.8824005	104.9599991
-5.3627968	10.9312000	105.8199997
-5.3627968	10.9800005	104.7900009
-5.3627968	11.0288000	102.2099991
-5.3627968	11.0776005	103.7500000
-5.3627968	11.1264000	102.3199997
-5.3627968	11.1752005	104.0199966
-5.3627968	11.2240000	106.4700012
-5.3627968	11.2728004	105.8899994
-5.3627968	11.3216000	102.9199982
-5.3627968	11.3704004	100.6100006
-5.3627968	11.4191999	103.9599991
-5.3627968	11.4680004	105.4199982
-5.3627968	11.5167999	102.0999985

-5.3627968	11.5656004	103.2099991
-5.3627968	11.6143999	102.6399994
-5.3627968	11.6632004	102.5800018
-5.3627968	11.7119999	103.9399948
-5.3627968	11.7608004	103.0000000
-5.3627968	11.8095999	104.9199982
-5.3627968	11.8584003	107.2600021
-5.3627968	11.9071999	106.6199951
-5.3627968	11.9560003	104.3699951
-5.3627968	12.0047998	104.0699997
-5.3627968	12.0536003	103.8600006
-5.3627968	12.1023998	100.0999985
-5.3627968	12.1512003	103.3399963
-5.3627968	12.1999998	107.5100021
-5.3627968	12.2488003	107.5499954
-5.3627968	12.2975998	104.0800018
-5.3627968	12.3464003	100.6199951
-5.3627968	12.3951998	102.1999969
-5.3627968	12.4440002	102.6100006
-5.3627968	12.4927998	101.9099960
-5.3627968	12.5416002	103.5100021
-5.3627968	12.5904007	105.1699982
-5.3627968	12.6392002	104.9700012
-5.3627968	12.6880007	102.8699951
-5.3627968	12.7368002	103.8899994
-5.3627968	12.7856007	104.2799988
-5.3627968	12.8344002	104.9399948
-5.3627968	12.8832006	104.6599960
-5.3627968	12.9320002	105.0199966
-5.3627968	12.9808006	103.0499954
-5.3627968	13.0296001	101.3199997
-5.3627968	13.0784006	102.2399979
-5.3627968	13.1272001	105.1299973
-5.3627968	13.1760006	105.2200012
-5.3627968	13.2248001	101.4499969
-5.3627968	13.2736006	104.5899963
-5.3627968	13.3224001	103.8300018
-5.3627968	13.3712006	100.9899979
-5.3627968	13.4200001	102.6699982
-5.3627968	13.4688005	105.0199966
-5.3627968	13.5176001	105.7099991
-5.3627968	13.5664005	104.3899994
-5.3627968	13.6152000	104.9000015
-5.3627968	13.6640005	103.3300018
-5.3627968	13.7128000	101.8899994
-5.3627968	13.7616005	100.4399948
-5.3627968	13.8104000	103.0100021
-5.3627968	13.8592005	102.9899979
-5.3627968	13.9080000	103.5599976
-5.3627968	13.9568005	103.2600021
-5.3627968	14.0056000	102.5100021
-5.3627968	14.0544004	102.6899948
-5.3627968	14.1032000	104.5400009
-5.3627968	14.1520004	101.9499969
-5.3627968	14.2007999	102.6899948
-5.3627968	14.2496004	104.4899979
-5.3627968	14.2983999	103.3399963
-5.3627968	14.3472004	103.7299957

-5.3627968	14.3959999	103.4799957
-5.3627968	14.4448004	99.6999969
-5.3627968	14.4935999	101.1800003
-5.3627968	14.5424004	101.5999985
-5.3627968	14.5911999	102.8899994
-5.3627968	14.6400003	103.9300003
-5.3627968	14.6887999	105.3600006
-5.3627968	14.7376003	105.7900009
-5.3627968	14.7863998	105.1100006
-5.3627968	14.8352003	103.4000015
-5.3627968	14.8839998	102.5899963
-5.3627968	14.9328003	99.9899979
-5.3627968	14.9815998	101.5899963
-5.3627968	15.0304003	103.6399994
-5.3566608	0.3416000	104.4199982
-5.3566608	0.3904000	103.1699982
-5.3566608	0.4392000	102.0499954
-5.3566608	0.4880000	104.6699982
-5.3566608	0.5368000	104.3600006
-5.3566608	0.5856000	106.8699951
-5.3566608	0.6344000	108.9399948
-5.3566608	0.6832000	108.1100006
-5.3566608	0.7320000	105.6899948
-5.3566608	0.7808000	107.9399948
-5.3566608	0.8296000	106.8899994
-5.3566608	0.8784000	105.8199997
-5.3566608	0.9272000	109.2900009
-5.3566608	0.9760000	108.9799957
-5.3566608	1.0247999	106.1500015
-5.3566608	1.0735999	104.2699966
-5.3566608	1.1223999	106.5999985
-5.3566608	1.1711999	103.0800018
-5.3566608	1.2200000	101.0499954
-5.3566608	1.2688000	104.7999954
-5.3566608	1.3176000	109.6299973
-5.3566608	1.3664000	109.0000000
-5.3566608	1.4152000	109.0699997
-5.3566608	1.4640000	109.0299988
-5.3566608	1.5128000	107.9399948
-5.3566608	1.5616000	105.4899979
-5.3566608	1.6104000	101.7900009
-5.3566608	1.6592000	106.7799988
-5.3566608	1.7079999	105.3899994
-5.3566608	1.7567999	105.7500000
-5.3566608	1.8055999	105.1599960
-5.3566608	1.8543999	107.2699966
-5.3566608	1.9031999	105.8399963
-5.3566608	1.9519999	103.8699951
-5.3566608	2.0007999	103.0899963
-5.3566608	2.0495999	106.9399948
-5.3566608	2.0983999	105.9399948
-5.3566608	2.1471999	106.2399979
-5.3566608	2.1959999	106.5000000
-5.3566608	2.2447999	102.6699982
-5.3566608	2.2935998	103.0000000
-5.3566608	2.3423998	102.0899963
-5.3566608	2.3912001	101.2999954
-5.3566608	2.4400001	102.8499985

-5.3566608	2.4888000	103.0100021
-5.3566608	2.5376000	102.2500000
-5.3566608	2.5864000	104.9300003
-5.3566608	2.6352000	104.3099976
-5.3566608	2.6840000	100.5999985
-5.3566608	2.7328000	102.4000015
-5.3566608	2.7816000	101.3600006
-5.3566608	2.8304000	101.8499985
-5.3566608	2.8792000	106.8099976
-5.3566608	2.9280000	109.3499985
-5.3566608	2.9768000	108.4399948
-5.3566608	3.0256000	105.0000000
-5.3566608	3.0743999	105.5299988
-5.3566608	3.1231999	102.5999985
-5.3566608	3.1719999	100.9399948
-5.3566608	3.2207999	104.2200012
-5.3566608	3.2695999	103.1699982
-5.3566608	3.3183999	99.8399963
-5.3566608	3.3671999	99.4799957
-5.3566608	3.4159999	102.3499985
-5.3566608	3.4647999	103.5499954
-5.3566608	3.5135999	103.0599976
-5.3566608	3.5623999	102.9899979
-5.3566608	3.6111999	100.6299973
-5.3566608	3.6599998	102.5899963
-5.3566608	3.7087998	102.4899979
-5.3566608	3.7575998	101.8899994
-5.3566608	3.8063998	101.4599991
-5.3566608	3.8551998	101.9899979
-5.3566608	3.9039998	98.4700012
-5.3566608	3.9527998	104.4799957
-5.3566608	4.0016003	105.6999969
-5.3566608	4.0503998	101.6699982
-5.3566608	4.0991998	100.1199951
-5.3566608	4.1479998	102.6899948
-5.3566608	4.1967998	97.9099960
-5.3566608	4.2455997	103.9499969
-5.3566608	4.2943997	104.0499954
-5.3566608	4.3431997	106.2299957
-5.3566608	4.3919997	103.3699951
-5.3566608	4.4407997	104.8799973
-5.3566608	4.4895997	104.4300003
-5.3566608	4.5383997	105.0400009
-5.3566608	4.5871997	101.8399963
-5.3566608	4.6359997	108.0299988
-5.3566608	4.6847997	112.7200012
-5.3566608	4.7336001	112.0499954
-5.3566608	4.7824001	107.5999985
-5.3566608	4.8312001	106.7999954
-5.3566608	4.8800001	107.6299973
-5.3566608	4.9288001	105.3499985
-5.3566608	4.9776001	104.1399994
-5.3566608	5.0264001	102.3899994
-5.3566608	5.0752001	101.5000000
-5.3566608	5.1240001	99.9399948
-5.3566608	5.1728001	101.9499969
-5.3566608	5.2216001	98.0999985
-5.3566608	5.2704000	103.3699951

-5.3566608	5.3192000	106.7799988
-5.3566608	5.3680000	107.0499954
-5.3566608	5.4168000	105.8899994
-5.3566608	5.4656000	106.6199951
-5.3566608	5.5144000	105.5299988
-5.3566608	5.5632000	105.9899979
-5.3566608	5.6120000	102.3099976
-5.3566608	5.6608000	102.5599976
-5.3566608	5.7096000	100.1199951
-5.3566608	5.7584000	101.5599976
-5.3566608	5.8072000	100.2600021
-5.3566608	5.8559999	100.7099991
-5.3566608	5.9047999	102.7399979
-5.3566608	5.9535999	105.1899948
-5.3566608	6.0023999	105.9799957
-5.3566608	6.0511999	104.5499954
-5.3566608	6.0999999	106.8799973
-5.3566608	6.1487999	102.9700012
-5.3566608	6.1975999	103.8300018
-5.3566608	6.2463999	106.0999985
-5.3566608	6.2951999	106.4199982
-5.3566608	6.3439999	106.3699951
-5.3566608	6.3927999	106.2099991
-5.3566608	6.4415998	102.9899979
-5.3566608	6.4903998	102.9700012
-5.3566608	6.5391998	104.7600021
-5.3566608	6.5879998	100.1500015
-5.3566608	6.6367998	99.4099960
-5.3566608	6.6855998	102.6100006
-5.3566608	6.7343998	104.0599976
-5.3566608	6.7831998	99.8099976
-5.3566608	6.8319998	101.1599960
-5.3566608	6.8807998	104.6500015
-5.3566608	6.9295998	103.3600006
-5.3566608	6.9783998	102.7200012
-5.3566608	7.0271997	104.3499985
-5.3566608	7.0759997	103.9799957
-5.3566608	7.1247997	103.4000015
-5.3566608	7.1735997	93.7600021
-5.3566608	7.2223997	103.4799957
-5.3566608	7.2711997	105.6800003
-5.3566608	7.3199997	102.7500000
-5.3566608	7.3687997	101.6899948
-5.3566608	7.4175997	102.5800018
-5.3566608	7.4663997	105.3799973
-5.3566608	7.5151997	106.8300018
-5.3566608	7.5639997	105.0199966
-5.3566608	7.6127996	104.3499985
-5.3566608	7.6615996	105.9000015
-5.3566608	7.7103996	106.0999985
-5.3566608	7.7591996	104.4700012
-5.3566608	7.8079996	103.9099960
-5.3566608	7.8567996	103.9799957
-5.3566608	7.9056001	102.2600021
-5.3566608	7.9544001	104.7200012
-5.3566608	8.0032005	104.9000015
-5.3566608	8.0520000	104.7500000
-5.3566608	8.1008005	106.0699997

-5.3566608	8.1496000	106.9099960
-5.3566608	8.1984005	105.2900009
-5.3566608	8.2472000	104.6399994
-5.3566608	8.2960005	97.4000015
-5.3566608	8.3448000	104.3799973
-5.3566608	8.3936005	104.9099960
-5.3566608	8.4424000	102.8899994
-5.3566608	8.4912004	98.4000015
-5.3566608	8.5400000	101.7200012
-5.3566608	8.5888004	102.0800018
-5.3566608	8.6375999	102.5000000
-5.3566608	8.6864004	103.8099976
-5.3566608	8.7351999	104.0699997
-5.3566608	8.7840004	104.0199966
-5.3566608	8.8327999	104.6199951
-5.3566608	8.8816004	103.0199966
-5.3566608	8.9303999	103.2900009
-5.3566608	8.9792004	103.6699982
-5.3566608	9.0279999	101.9700012
-5.3566608	9.0768003	103.5400009
-5.3566608	9.1255999	104.7200012
-5.3566608	9.1744003	103.7399979
-5.3566608	9.2231998	100.5699997
-5.3566608	9.2720003	102.1999969
-5.3566608	9.3207998	103.5899963
-5.3566608	9.3696003	99.6599960
-5.3566608	9.4184008	102.6999969
-5.3566608	9.4672003	106.0299988
-5.3566608	9.5160007	106.9899979
-5.3566608	9.5648003	105.4799957
-5.3566608	9.6136007	101.4700012
-5.3566608	9.6624002	102.4899979
-5.3566608	9.7112007	101.2900009
-5.3566608	9.7600002	103.0299988
-5.3566608	9.8088007	102.0499954
-5.3566608	9.8576002	105.8799973
-5.3566608	9.9064007	103.2399979
-5.3566608	9.9552002	103.5100021
-5.3566608	10.0040007	103.8699951
-5.3566608	10.0528002	106.8499985
-5.3566608	10.1016006	103.3600006
-5.3566608	10.1504002	106.8099976
-5.3566608	10.1992006	106.6999969
-5.3566608	10.2480001	104.2799988
-5.3566608	10.2968006	103.4000015
-5.3566608	10.3456001	104.7299957
-5.3566608	10.3944006	104.1199951
-5.3566608	10.4432001	102.5699997
-5.3566608	10.4920006	102.0599976
-5.3566608	10.5408001	100.0299988
-5.3566608	10.5896006	103.7099991
-5.3566608	10.6384001	103.5499954
-5.3566608	10.6872005	104.2799988
-5.3566608	10.7360001	102.4599991
-5.3566608	10.7848005	103.5999985
-5.3566608	10.8336000	103.0800018
-5.3566608	10.8824005	104.9300003
-5.3566608	10.9312000	105.4199982

-5.3566608	10.9800005	104.8600006
-5.3566608	11.0288000	103.5400009
-5.3566608	11.0776005	104.4000015
-5.3566608	11.1264000	102.5100021
-5.3566608	11.1752005	104.2299957
-5.3566608	11.2240000	106.9899979
-5.3566608	11.2728004	106.1699982
-5.3566608	11.3216000	103.2699966
-5.3566608	11.3704004	100.7200012
-5.3566608	11.4191999	103.8799973
-5.3566608	11.4680004	105.9099960
-5.3566608	11.5167999	103.5699997
-5.3566608	11.5656004	104.1999969
-5.3566608	11.6143999	103.0899963
-5.3566608	11.6632004	102.7399979
-5.3566608	11.7119999	104.0800018
-5.3566608	11.7608004	103.7500000
-5.3566608	11.8095999	106.0800018
-5.3566608	11.8584003	107.2600021
-5.3566608	11.9071999	106.3199997
-5.3566608	11.9560003	103.7799988
-5.3566608	12.0047998	104.3799973
-5.3566608	12.0536003	104.1699982
-5.3566608	12.1023998	98.3300018
-5.3566608	12.1512003	102.2699966
-5.3566608	12.1999998	107.1500015
-5.3566608	12.2488003	107.7099991
-5.3566608	12.2975998	104.6500015
-5.3566608	12.3464003	101.6100006
-5.3566608	12.3951998	103.7500000
-5.3566608	12.4440002	104.2200012
-5.3566608	12.4927998	103.2200012
-5.3566608	12.5416002	104.1100006
-5.3566608	12.5904007	105.0199966
-5.3566608	12.6392002	104.9000015
-5.3566608	12.6880007	103.1100006
-5.3566608	12.7368002	104.0400009
-5.3566608	12.7856007	102.9499969
-5.3566608	12.8344002	104.7900009
-5.3566608	12.8832006	105.3300018
-5.3566608	12.9320002	105.3899994
-5.3566608	12.9808006	101.8099976
-5.3566608	13.0296001	100.7999954
-5.3566608	13.0784006	102.2900009
-5.3566608	13.1272001	103.9599991
-5.3566608	13.1760006	104.4700012
-5.3566608	13.2248001	102.3199997
-5.3566608	13.2736006	105.2900009
-5.3566608	13.3224001	103.9099960
-5.3566608	13.3712006	100.2999954
-5.3566608	13.4200001	101.5800018
-5.3566608	13.4688005	104.3099976
-5.3566608	13.5176001	105.9899979
-5.3566608	13.5664005	105.0000000
-5.3566608	13.6152000	104.9000015
-5.3566608	13.6640005	102.6199951
-5.3566608	13.7128000	102.5299988
-5.3566608	13.7616005	101.5499954

-5.3566608	13.8104000	103.1899948
-5.3566608	13.8592005	102.5199966
-5.3566608	13.9080000	102.7600021
-5.3566608	13.9568005	101.5699997
-5.3566608	14.0056000	102.8899994
-5.3566608	14.0544004	102.1699982
-5.3566608	14.1032000	103.1199951
-5.3566608	14.1520004	101.2999954
-5.3566608	14.2007999	103.6299973
-5.3566608	14.2496004	104.9099960
-5.3566608	14.2983999	102.3099976
-5.3566608	14.3472004	103.2699966
-5.3566608	14.3959999	103.1500015
-5.3566608	14.4448004	99.7099991
-5.3566608	14.4935999	101.1699982
-5.3566608	14.5424004	102.7399979
-5.3566608	14.5911999	103.7500000
-5.3566608	14.6400003	104.9199982
-5.3566608	14.6887999	105.1599960
-5.3566608	14.7376003	105.3099976
-5.3566608	14.7863998	104.3899994
-5.3566608	14.8352003	103.1800003
-5.3566608	14.8839998	102.7399979
-5.3566608	14.9328003	99.1100006
-5.3566608	14.9815998	101.4399948
-5.3566608	15.0304003	103.4700012
-5.3505249	0.3416000	104.8099976
-5.3505249	0.3904000	104.0899963
-5.3505249	0.4392000	103.4300003
-5.3505249	0.4880000	105.1399994
-5.3505249	0.5368000	103.7699966
-5.3505249	0.5856000	106.5000000
-5.3505249	0.6344000	107.6299973
-5.3505249	0.6832000	107.1500015
-5.3505249	0.7320000	106.2699966
-5.3505249	0.7808000	108.1800003
-5.3505249	0.8296000	106.3099976
-5.3505249	0.8784000	104.3499985
-5.3505249	0.9272000	108.9199982
-5.3505249	0.9760000	108.9700012
-5.3505249	1.0247999	106.9099960
-5.3505249	1.0735999	105.2099991
-5.3505249	1.1223999	107.1199951
-5.3505249	1.1711999	103.7999954
-5.3505249	1.2200000	101.0499954
-5.3505249	1.2688000	104.7900009
-5.3505249	1.3176000	109.2399979
-5.3505249	1.3664000	108.6299973
-5.3505249	1.4152000	108.4799957
-5.3505249	1.4640000	108.4799957
-5.3505249	1.5128000	107.8799973
-5.3505249	1.5616000	105.3799973
-5.3505249	1.6104000	102.3399963
-5.3505249	1.6592000	106.5299988
-5.3505249	1.7079999	105.2900009
-5.3505249	1.7567999	105.9499969
-5.3505249	1.8055999	105.0499954
-5.3505249	1.8543999	106.5499954

-5.3505249	1.9031999	106.2500000
-5.3505249	1.9519999	103.2500000
-5.3505249	2.0007999	101.4099960
-5.3505249	2.0495999	106.1299973
-5.3505249	2.0983999	106.0800018
-5.3505249	2.1471999	106.7900009
-5.3505249	2.1959999	107.0599976
-5.3505249	2.2447999	103.0000000
-5.3505249	2.2935998	102.6299973
-5.3505249	2.3423998	102.8199997
-5.3505249	2.3912001	101.9700012
-5.3505249	2.4400001	103.3399963
-5.3505249	2.4888000	103.5499954
-5.3505249	2.5376000	101.9799957
-5.3505249	2.5864000	104.6699982
-5.3505249	2.6352000	103.9499969
-5.3505249	2.6840000	101.6299973
-5.3505249	2.7328000	103.1899948
-5.3505249	2.7816000	101.8600006
-5.3505249	2.8304000	102.2099991
-5.3505249	2.8792000	106.7500000
-5.3505249	2.9280000	109.2799988
-5.3505249	2.9768000	107.9599991
-5.3505249	3.0256000	105.0699997
-5.3505249	3.0743999	105.0400009
-5.3505249	3.1231999	103.1800003
-5.3505249	3.1719999	100.5299988
-5.3505249	3.2207999	103.5999985
-5.3505249	3.2695999	103.4499969
-5.3505249	3.3183999	100.5499954
-5.3505249	3.3671999	99.9700012
-5.3505249	3.4159999	103.3199997
-5.3505249	3.4647999	104.5800018
-5.3505249	3.5135999	103.6800003
-5.3505249	3.5623999	102.7399979
-5.3505249	3.6111999	100.6899948
-5.3505249	3.6599998	101.3399963
-5.3505249	3.7087998	101.5499954
-5.3505249	3.7575998	101.2200012
-5.3505249	3.8063998	102.7500000
-5.3505249	3.8551998	103.1299973
-5.3505249	3.9039998	99.5000000
-5.3505249	3.9527998	104.5599976
-5.3505249	4.0016003	105.5299988
-5.3505249	4.0503998	100.7500000
-5.3505249	4.0991998	100.1999969
-5.3505249	4.1479998	102.2900009
-5.3505249	4.1967998	97.0499954
-5.3505249	4.2455997	104.6500015
-5.3505249	4.2943997	104.1299973
-5.3505249	4.3431997	106.4099960
-5.3505249	4.3919997	103.2099991
-5.3505249	4.4407997	104.4000015
-5.3505249	4.4895997	104.1999969
-5.3505249	4.5383997	105.2699966
-5.3505249	4.5871997	102.3399963
-5.3505249	4.6359997	108.2399979
-5.3505249	4.6847997	113.2999954

-5.3505249	4.7336001	112.0599976
-5.3505249	4.7824001	107.1299973
-5.3505249	4.8312001	105.7099991
-5.3505249	4.8800001	106.6399994
-5.3505249	4.9288001	104.1399994
-5.3505249	4.9776001	103.7200012
-5.3505249	5.0264001	102.8499985
-5.3505249	5.0752001	101.6500015
-5.3505249	5.1240001	99.2299957
-5.3505249	5.1728001	101.5100021
-5.3505249	5.2216001	96.7799988
-5.3505249	5.2704000	103.3699951
-5.3505249	5.3192000	106.7099991
-5.3505249	5.3680000	106.9499969
-5.3505249	5.4168000	105.5199966
-5.3505249	5.4656000	106.4899979
-5.3505249	5.5144000	103.9899979
-5.3505249	5.5632000	104.7999954
-5.3505249	5.6120000	103.1699982
-5.3505249	5.6608000	102.4599991
-5.3505249	5.7096000	100.5499954
-5.3505249	5.7584000	101.8499985
-5.3505249	5.8072000	100.9000015
-5.3505249	5.8559999	100.9599991
-5.3505249	5.9047999	101.6599960
-5.3505249	5.9535999	105.9599991
-5.3505249	6.0023999	105.7500000
-5.3505249	6.0511999	103.6999969
-5.3505249	6.0999999	107.3199997
-5.3505249	6.1487999	104.3899994
-5.3505249	6.1975999	103.0999985
-5.3505249	6.2463999	104.8300018
-5.3505249	6.2951999	105.2999954
-5.3505249	6.3439999	105.0999985
-5.3505249	6.3927999	105.0800018
-5.3505249	6.4415998	102.3099976
-5.3505249	6.4903998	102.5400009
-5.3505249	6.5391998	103.5000000
-5.3505249	6.5879998	99.0000000
-5.3505249	6.6367998	99.2699966
-5.3505249	6.6855998	102.6199951
-5.3505249	6.7343998	104.5699997
-5.3505249	6.7831998	100.1800003
-5.3505249	6.8319998	101.0400009
-5.3505249	6.8807998	104.7699966
-5.3505249	6.9295998	104.1100006
-5.3505249	6.9783998	103.0299988
-5.3505249	7.0271997	104.0199966
-5.3505249	7.0759997	103.9499969
-5.3505249	7.1247997	104.0299988
-5.3505249	7.1735997	96.3799973
-5.3505249	7.2223997	103.5999985
-5.3505249	7.2711997	105.2600021
-5.3505249	7.3199997	103.2099991
-5.3505249	7.3687997	100.9300003
-5.3505249	7.4175997	101.7999954
-5.3505249	7.4663997	105.3199997
-5.3505249	7.5151997	106.7399979

-5.3505249	7.5639997	105.0999985
-5.3505249	7.6127996	104.6800003
-5.3505249	7.6615996	106.6899948
-5.3505249	7.7103996	106.4300003
-5.3505249	7.7591996	104.1399994
-5.3505249	7.8079996	105.0100021
-5.3505249	7.8567996	104.4399948
-5.3505249	7.9056001	100.9499969
-5.3505249	7.9544001	104.6500015
-5.3505249	8.0032005	105.1399994
-5.3505249	8.0520000	105.0000000
-5.3505249	8.1008005	106.2900009
-5.3505249	8.1496000	107.5899963
-5.3505249	8.1984005	106.2099991
-5.3505249	8.2472000	104.5800018
-5.3505249	8.2960005	98.6100006
-5.3505249	8.3448000	105.5599976
-5.3505249	8.3936005	105.3699951
-5.3505249	8.4424000	103.6699982
-5.3505249	8.4912004	100.7399979
-5.3505249	8.5400000	103.0699997
-5.3505249	8.5888004	101.8300018
-5.3505249	8.6375999	103.3099976
-5.3505249	8.6864004	104.3099976
-5.3505249	8.7351999	103.2200012
-5.3505249	8.7840004	104.0299988
-5.3505249	8.8327999	104.5899963
-5.3505249	8.8816004	102.6899948
-5.3505249	8.9303999	102.2099991
-5.3505249	8.9792004	103.7900009
-5.3505249	9.0279999	103.1500015
-5.3505249	9.0768003	103.9499969
-5.3505249	9.1255999	104.5999985
-5.3505249	9.1744003	103.5100021
-5.3505249	9.2231998	100.2799988
-5.3505249	9.2720003	102.0899963
-5.3505249	9.3207998	103.2299957
-5.3505249	9.3696003	98.9499969
-5.3505249	9.4184008	102.3499985
-5.3505249	9.4672003	105.8600006
-5.3505249	9.5160007	106.7200012
-5.3505249	9.5648003	105.1500015
-5.3505249	9.6136007	101.3699951
-5.3505249	9.6624002	102.7699966
-5.3505249	9.7112007	102.5000000
-5.3505249	9.7600002	103.8499985
-5.3505249	9.8088007	102.9399948
-5.3505249	9.8576002	105.6199951
-5.3505249	9.9064007	103.5499954
-5.3505249	9.9552002	103.5299988
-5.3505249	10.0040007	103.8600006
-5.3505249	10.0528002	106.4099960
-5.3505249	10.1016006	104.5499954
-5.3505249	10.1504002	107.0400009
-5.3505249	10.1992006	106.9199982
-5.3505249	10.2480001	103.6399994
-5.3505249	10.2968006	103.0400009
-5.3505249	10.3456001	104.9599991

-5.3505249	10.3944006	104.5999985
-5.3505249	10.4432001	102.9000015
-5.3505249	10.4920006	101.1399994
-5.3505249	10.5408001	99.0599976
-5.3505249	10.5896006	103.1100006
-5.3505249	10.6384001	103.9799957
-5.3505249	10.6872005	104.6699982
-5.3505249	10.7360001	103.3600006
-5.3505249	10.7848005	102.5699997
-5.3505249	10.8336000	102.3799973
-5.3505249	10.8824005	104.6100006
-5.3505249	10.9312000	104.3099976
-5.3505249	10.9800005	104.4499969
-5.3505249	11.0288000	104.0499954
-5.3505249	11.0776005	104.5400009
-5.3505249	11.1264000	102.6899948
-5.3505249	11.1752005	104.5000000
-5.3505249	11.2240000	107.0599976
-5.3505249	11.2728004	105.9300003
-5.3505249	11.3216000	103.4799957
-5.3505249	11.3704004	101.2500000
-5.3505249	11.4191999	102.6699982
-5.3505249	11.4680004	105.5800018
-5.3505249	11.5167999	103.8300018
-5.3505249	11.5656004	104.0800018
-5.3505249	11.6143999	103.3699951
-5.3505249	11.6632004	102.9700012
-5.3505249	11.7119999	104.2799988
-5.3505249	11.7608004	104.7200012
-5.3505249	11.8095999	107.0100021
-5.3505249	11.8584003	107.0199966
-5.3505249	11.9071999	106.0400009
-5.3505249	11.9560003	103.8499985
-5.3505249	12.0047998	105.0100021
-5.3505249	12.0536003	104.5100021
-5.3505249	12.1023998	97.4199982
-5.3505249	12.1512003	101.4599991
-5.3505249	12.1999998	106.3399963
-5.3505249	12.2488003	107.5699997
-5.3505249	12.2975998	104.8300018
-5.3505249	12.3464003	101.7799988
-5.3505249	12.3951998	104.4300003
-5.3505249	12.4440002	105.4599991
-5.3505249	12.4927998	104.4199982
-5.3505249	12.5416002	105.0999985
-5.3505249	12.5904007	104.9000015
-5.3505249	12.6392002	104.7500000
-5.3505249	12.6880007	103.9399948
-5.3505249	12.7368002	104.2299957
-5.3505249	12.7856007	101.7500000
-5.3505249	12.8344002	104.6299973
-5.3505249	12.8832006	105.7600021
-5.3505249	12.9320002	106.0199966
-5.3505249	12.9808006	101.2699966
-5.3505249	13.0296001	100.8499985
-5.3505249	13.0784006	101.4499969
-5.3505249	13.1272001	102.0400009
-5.3505249	13.1760006	104.1399994

-5.3505249	13.2248001	104.0699997
-5.3505249	13.2736006	106.3399963
-5.3505249	13.3224001	104.0800018
-5.3505249	13.3712006	99.6100006
-5.3505249	13.4200001	100.3199997
-5.3505249	13.4688005	103.6399994
-5.3505249	13.5176001	106.3799973
-5.3505249	13.5664005	105.6999969
-5.3505249	13.6152000	105.0299988
-5.3505249	13.6640005	101.9399948
-5.3505249	13.7128000	102.7200012
-5.3505249	13.7616005	101.4099960
-5.3505249	13.8104000	102.5800018
-5.3505249	13.8592005	101.7500000
-5.3505249	13.9080000	102.4399948
-5.3505249	13.9568005	99.7099991
-5.3505249	14.0056000	102.9300003
-5.3505249	14.0544004	101.6500015
-5.3505249	14.1032000	101.5999985
-5.3505249	14.1520004	100.4199982
-5.3505249	14.2007999	103.9700012
-5.3505249	14.2496004	105.2999954
-5.3505249	14.2983999	102.1599960
-5.3505249	14.3472004	102.9099960
-5.3505249	14.3959999	102.6899948
-5.3505249	14.4448004	100.0599976
-5.3505249	14.4935999	101.4899979
-5.3505249	14.5424004	103.7099991
-5.3505249	14.5911999	104.2999954
-5.3505249	14.6400003	105.4799957
-5.3505249	14.6887999	105.4700012
-5.3505249	14.7376003	104.9799957
-5.3505249	14.7863998	103.8799973
-5.3505249	14.8352003	102.7399979
-5.3505249	14.8839998	102.6299973
-5.3505249	14.9328003	97.9199982
-5.3505249	14.9815998	101.8300018
-5.3505249	15.0304003	103.4599991
-5.3443890	0.3416000	105.1699982
-5.3443890	0.3904000	104.7600021
-5.3443890	0.4392000	104.5400009
-5.3443890	0.4880000	105.5100021
-5.3443890	0.5368000	103.3199997
-5.3443890	0.5856000	105.7699966
-5.3443890	0.6344000	105.9099960
-5.3443890	0.6832000	106.1800003
-5.3443890	0.7320000	106.1899948
-5.3443890	0.7808000	107.1599960
-5.3443890	0.8296000	105.0800018
-5.3443890	0.8784000	102.7699966
-5.3443890	0.9272000	108.3899994
-5.3443890	0.9760000	108.2600021
-5.3443890	1.0247999	107.1999969
-5.3443890	1.0735999	105.6299973
-5.3443890	1.1223999	107.5000000
-5.3443890	1.1711999	104.3399963
-5.3443890	1.2200000	101.6800003
-5.3443890	1.2688000	104.5100021

-5.3443890	1.3176000	108.3899994
-5.3443890	1.3664000	107.7500000
-5.3443890	1.4152000	107.7099991
-5.3443890	1.4640000	107.8099976
-5.3443890	1.5128000	107.5299988
-5.3443890	1.5616000	104.9300003
-5.3443890	1.6104000	102.8099976
-5.3443890	1.6592000	106.4300003
-5.3443890	1.7079999	105.8499985
-5.3443890	1.7567999	106.3899994
-5.3443890	1.8055999	104.5899963
-5.3443890	1.8543999	105.9499969
-5.3443890	1.9031999	107.0199966
-5.3443890	1.9519999	104.1199951
-5.3443890	2.0007999	100.7900009
-5.3443890	2.0495999	106.5499954
-5.3443890	2.0983999	106.8799973
-5.3443890	2.1471999	107.1899948
-5.3443890	2.1959999	107.2699966
-5.3443890	2.2447999	103.5000000
-5.3443890	2.2935998	102.2299957
-5.3443890	2.3423998	102.7699966
-5.3443890	2.3912001	102.5499954
-5.3443890	2.4400001	103.9399948
-5.3443890	2.4888000	103.3199997
-5.3443890	2.5376000	101.3099976
-5.3443890	2.5864000	104.6199951
-5.3443890	2.6352000	103.7600021
-5.3443890	2.6840000	102.1599960
-5.3443890	2.7328000	103.9399948
-5.3443890	2.7816000	102.1899948
-5.3443890	2.8304000	101.4000015
-5.3443890	2.8792000	106.4099960
-5.3443890	2.9280000	108.9899979
-5.3443890	2.9768000	107.0699997
-5.3443890	3.0256000	105.1899948
-5.3443890	3.0743999	105.0000000
-5.3443890	3.1231999	103.7500000
-5.3443890	3.1719999	100.0499954
-5.3443890	3.2207999	102.9700012
-5.3443890	3.2695999	104.5599976
-5.3443890	3.3183999	101.8899994
-5.3443890	3.3671999	100.8199997
-5.3443890	3.4159999	104.1899948
-5.3443890	3.4647999	105.7600021
-5.3443890	3.5135999	104.4599991
-5.3443890	3.5623999	102.7699966
-5.3443890	3.6111999	101.2099991
-5.3443890	3.6599998	100.7200012
-5.3443890	3.7087998	101.3899994
-5.3443890	3.7575998	100.5499954
-5.3443890	3.8063998	103.6500015
-5.3443890	3.8551998	104.4199982
-5.3443890	3.9039998	100.6100006
-5.3443890	3.9527998	104.0999985
-5.3443890	4.0016003	105.1199951
-5.3443890	4.0503998	100.9000015
-5.3443890	4.0991998	100.5499954

-5.3443890	4.1479998	101.9799957
-5.3443890	4.1967998	95.5299988
-5.3443890	4.2455997	105.1699982
-5.3443890	4.2943997	105.4799957
-5.3443890	4.3431997	106.6899948
-5.3443890	4.3919997	102.9499969
-5.3443890	4.4407997	103.8399963
-5.3443890	4.4895997	103.1500015
-5.3443890	4.5383997	104.7699966
-5.3443890	4.5871997	102.9799957
-5.3443890	4.6359997	108.2299957
-5.3443890	4.6847997	113.3600006
-5.3443890	4.7336001	111.4300003
-5.3443890	4.7824001	106.2699966
-5.3443890	4.8312001	104.4499969
-5.3443890	4.8800001	105.6399994
-5.3443890	4.9288001	103.1500015
-5.3443890	4.9776001	103.7299957
-5.3443890	5.0264001	103.8199997
-5.3443890	5.0752001	102.4199982
-5.3443890	5.1240001	99.6500015
-5.3443890	5.1728001	100.9700012
-5.3443890	5.2216001	95.7500000
-5.3443890	5.2704000	103.0199966
-5.3443890	5.3192000	106.5299988
-5.3443890	5.3680000	106.8199997
-5.3443890	5.4168000	105.0599976
-5.3443890	5.4656000	106.6100006
-5.3443890	5.5144000	103.0699997
-5.3443890	5.5632000	103.7500000
-5.3443890	5.6120000	104.0800018
-5.3443890	5.6608000	102.0199966
-5.3443890	5.7096000	100.6899948
-5.3443890	5.7584000	101.8300018
-5.3443890	5.8072000	100.3799973
-5.3443890	5.8559999	101.0299988
-5.3443890	5.9047999	100.0899963
-5.3443890	5.9535999	106.3600006
-5.3443890	6.0023999	105.7099991
-5.3443890	6.0511999	104.2399979
-5.3443890	6.0999999	108.0400009
-5.3443890	6.1487999	105.5400009
-5.3443890	6.1975999	102.3300018
-5.3443890	6.2463999	103.5699997
-5.3443890	6.2951999	103.8699951
-5.3443890	6.3439999	103.3499985
-5.3443890	6.3927999	103.6399994
-5.3443890	6.4415998	101.3699951
-5.3443890	6.4903998	102.4700012
-5.3443890	6.5391998	103.0000000
-5.3443890	6.5879998	98.7799988
-5.3443890	6.6367998	99.9000015
-5.3443890	6.6855998	102.3099976
-5.3443890	6.7343998	104.5699997
-5.3443890	6.7831998	101.1500015
-5.3443890	6.8319998	101.8600006
-5.3443890	6.8807998	105.2200012
-5.3443890	6.9295998	104.8799973

-5.3443890	6.9783998	102.9799957
-5.3443890	7.0271997	103.0999985
-5.3443890	7.0759997	103.4599991
-5.3443890	7.1247997	103.8899994
-5.3443890	7.1735997	98.5899963
-5.3443890	7.2223997	103.4099960
-5.3443890	7.2711997	104.3099976
-5.3443890	7.3199997	102.9399948
-5.3443890	7.3687997	99.5899963
-5.3443890	7.4175997	101.4399948
-5.3443890	7.4663997	105.2399979
-5.3443890	7.5151997	106.2999954
-5.3443890	7.5639997	104.4499969
-5.3443890	7.6127996	104.4399948
-5.3443890	7.6615996	107.0000000
-5.3443890	7.7103996	106.4000015
-5.3443890	7.7591996	104.4000015
-5.3443890	7.8079996	105.0899963
-5.3443890	7.8567996	104.7399979
-5.3443890	7.9056001	101.0999985
-5.3443890	7.9544001	105.3199997
-5.3443890	8.0032005	105.6800003
-5.3443890	8.0520000	105.1899948
-5.3443890	8.1008005	106.4899979
-5.3443890	8.1496000	107.7200012
-5.3443890	8.1984005	106.6800003
-5.3443890	8.2472000	104.5899963
-5.3443890	8.2960005	100.5299988
-5.3443890	8.3448000	106.0199966
-5.3443890	8.3936005	105.6800003
-5.3443890	8.4424000	104.3899994
-5.3443890	8.4912004	102.4799957
-5.3443890	8.5400000	103.4599991
-5.3443890	8.5888004	101.6500015
-5.3443890	8.6375999	103.5400009
-5.3443890	8.6864004	104.1899948
-5.3443890	8.7351999	101.5499954
-5.3443890	8.7840004	103.2699966
-5.3443890	8.8327999	104.5000000
-5.3443890	8.8816004	102.9000015
-5.3443890	8.9303999	101.5800018
-5.3443890	8.9792004	103.5999985
-5.3443890	9.0279999	103.8799973
-5.3443890	9.0768003	103.4399948
-5.3443890	9.1255999	103.9300003
-5.3443890	9.1744003	103.4099960
-5.3443890	9.2231998	100.7299957
-5.3443890	9.2720003	102.4899979
-5.3443890	9.3207998	102.7999954
-5.3443890	9.3696003	97.9899979
-5.3443890	9.4184008	101.6199951
-5.3443890	9.4672003	105.4799957
-5.3443890	9.5160007	106.3600006
-5.3443890	9.5648003	104.6100006
-5.3443890	9.6136007	101.8399963
-5.3443890	9.6624002	102.9700012
-5.3443890	9.7112007	102.7999954
-5.3443890	9.7600002	104.6100006

-5.3443890	9.8088007	103.9700012
-5.3443890	9.8576002	105.5499954
-5.3443890	9.9064007	104.1100006
-5.3443890	9.9552002	103.3099976
-5.3443890	10.0040007	103.2799988
-5.3443890	10.0528002	105.6299973
-5.3443890	10.1016006	106.2900009
-5.3443890	10.1504002	107.4599991
-5.3443890	10.1992006	106.6399994
-5.3443890	10.2480001	102.6199951
-5.3443890	10.2968006	102.6599960
-5.3443890	10.3456001	104.5100021
-5.3443890	10.3944006	104.3300018
-5.3443890	10.4432001	103.1500015
-5.3443890	10.4920006	100.9199982
-5.3443890	10.5408001	99.5999985
-5.3443890	10.5896006	102.4700012
-5.3443890	10.6384001	104.0100021
-5.3443890	10.6872005	105.0999985
-5.3443890	10.7360001	103.6800003
-5.3443890	10.7848005	100.7799988
-5.3443890	10.8336000	102.2699966
-5.3443890	10.8824005	103.9899979
-5.3443890	10.9312000	103.0699997
-5.3443890	10.9800005	103.5699997
-5.3443890	11.0288000	103.6299973
-5.3443890	11.0776005	104.1599960
-5.3443890	11.1264000	102.7099991
-5.3443890	11.1752005	105.1999969
-5.3443890	11.2240000	107.2799988
-5.3443890	11.2728004	105.5100021
-5.3443890	11.3216000	103.3600006
-5.3443890	11.3704004	101.3899994
-5.3443890	11.4191999	101.1699982
-5.3443890	11.4680004	105.0800018
-5.3443890	11.5167999	103.4899979
-5.3443890	11.5656004	102.9700012
-5.3443890	11.6143999	103.2099991
-5.3443890	11.6632004	103.2699966
-5.3443890	11.7119999	104.2299957
-5.3443890	11.7608004	105.2799988
-5.3443890	11.8095999	107.7500000
-5.3443890	11.8584003	106.7600021
-5.3443890	11.9071999	105.7099991
-5.3443890	11.9560003	103.4099960
-5.3443890	12.0047998	105.2200012
-5.3443890	12.0536003	104.5699997
-5.3443890	12.1023998	97.5000000
-5.3443890	12.1512003	101.0299988
-5.3443890	12.1999998	105.1699982
-5.3443890	12.2488003	106.8099976
-5.3443890	12.2975998	104.9899979
-5.3443890	12.3464003	102.7099991
-5.3443890	12.3951998	105.5699997
-5.3443890	12.4440002	107.1199951
-5.3443890	12.4927998	106.1299973
-5.3443890	12.5416002	106.2900009
-5.3443890	12.5904007	104.9499969

-5.3443890	12.6392002	104.6299973
-5.3443890	12.6880007	104.5000000
-5.3443890	12.7368002	104.0499954
-5.3443890	12.7856007	100.9099960
-5.3443890	12.8344002	104.2299957
-5.3443890	12.8832006	105.7099991
-5.3443890	12.9320002	106.1899948
-5.3443890	12.9808006	100.6999969
-5.3443890	13.0296001	101.0299988
-5.3443890	13.0784006	100.8699951
-5.3443890	13.1272001	100.1899948
-5.3443890	13.1760006	103.4099960
-5.3443890	13.2248001	104.8300018
-5.3443890	13.2736006	107.0699997
-5.3443890	13.3224001	104.3899994
-5.3443890	13.3712006	99.5800018
-5.3443890	13.4200001	99.2699966
-5.3443890	13.4688005	103.0100021
-5.3443890	13.5176001	106.0000000
-5.3443890	13.5664005	105.6800003
-5.3443890	13.6152000	105.0499954
-5.3443890	13.6640005	102.9499969
-5.3443890	13.7128000	103.0499954
-5.3443890	13.7616005	100.8699951
-5.3443890	13.8104000	102.1899948
-5.3443890	13.8592005	100.9300003
-5.3443890	13.9080000	102.0400009
-5.3443890	13.9568005	98.7200012
-5.3443890	14.0056000	103.0400009
-5.3443890	14.0544004	101.9199982
-5.3443890	14.1032000	100.8600006
-5.3443890	14.1520004	100.2500000
-5.3443890	14.2007999	104.9199982
-5.3443890	14.2496004	106.2699966
-5.3443890	14.2983999	103.0599976
-5.3443890	14.3472004	102.9799957
-5.3443890	14.3959999	101.7399979
-5.3443890	14.4448004	99.6100006
-5.3443890	14.4935999	101.9799957
-5.3443890	14.5424004	104.3399963
-5.3443890	14.5911999	104.3399963
-5.3443890	14.6400003	105.6999969
-5.3443890	14.6887999	105.5000000
-5.3443890	14.7376003	104.3699951
-5.3443890	14.7863998	103.2099991
-5.3443890	14.8352003	102.7500000
-5.3443890	14.8839998	102.9000015
-5.3443890	14.9328003	96.3300018
-5.3443890	14.9815998	101.9000015
-5.3443890	15.0304003	103.0400009
-5.3382535	0.3416000	106.4300003
-5.3382535	0.3904000	106.0599976
-5.3382535	0.4392000	106.0499954
-5.3382535	0.4880000	106.2399979
-5.3382535	0.5368000	104.2299957
-5.3382535	0.5856000	105.4899979
-5.3382535	0.6344000	104.2600021
-5.3382535	0.6832000	105.6100006

-5.3382535	0.7320000	105.3699951
-5.3382535	0.7808000	105.9599991
-5.3382535	0.8296000	103.7399979
-5.3382535	0.8784000	101.0199966
-5.3382535	0.9272000	107.2900009
-5.3382535	0.9760000	107.0400009
-5.3382535	1.0247999	107.4599991
-5.3382535	1.0735999	105.9599991
-5.3382535	1.1223999	107.1800003
-5.3382535	1.1711999	104.8199997
-5.3382535	1.2200000	102.4499969
-5.3382535	1.2688000	104.3899994
-5.3382535	1.3176000	107.7900009
-5.3382535	1.3664000	107.6899948
-5.3382535	1.4152000	107.6100006
-5.3382535	1.4640000	107.1800003
-5.3382535	1.5128000	106.8399963
-5.3382535	1.5616000	104.4399948
-5.3382535	1.6104000	103.7699966
-5.3382535	1.6592000	106.4199982
-5.3382535	1.7079999	106.2399979
-5.3382535	1.7567999	106.8799973
-5.3382535	1.8055999	104.8699951
-5.3382535	1.8543999	105.4899979
-5.3382535	1.9031999	106.9799957
-5.3382535	1.9519999	104.6500015
-5.3382535	2.0007999	99.8099976
-5.3382535	2.0495999	106.6100006
-5.3382535	2.0983999	107.6699982
-5.3382535	2.1471999	107.6500015
-5.3382535	2.1959999	106.9899979
-5.3382535	2.2447999	102.9799957
-5.3382535	2.2935998	101.1599960
-5.3382535	2.3423998	102.4599991
-5.3382535	2.3912001	103.0000000
-5.3382535	2.4400001	104.7600021
-5.3382535	2.4888000	103.8399963
-5.3382535	2.5376000	102.6699982
-5.3382535	2.5864000	105.0299988
-5.3382535	2.6352000	103.5400009
-5.3382535	2.6840000	102.5199966
-5.3382535	2.7328000	104.6899948
-5.3382535	2.7816000	102.6599960
-5.3382535	2.8304000	101.1699982
-5.3382535	2.8792000	105.7600021
-5.3382535	2.9280000	108.0000000
-5.3382535	2.9768000	106.1299973
-5.3382535	3.0256000	105.6299973
-5.3382535	3.0743999	105.1199951
-5.3382535	3.1231999	103.7600021
-5.3382535	3.1719999	99.8099976
-5.3382535	3.2207999	102.6100006
-5.3382535	3.2695999	105.1800003
-5.3382535	3.3183999	103.4099960
-5.3382535	3.3671999	101.3899994
-5.3382535	3.4159999	104.9199982
-5.3382535	3.4647999	106.5400009
-5.3382535	3.5135999	105.0999985

-5.3382535	3.5623999	103.5299988
-5.3382535	3.6111999	102.1999969
-5.3382535	3.6599998	100.6899948
-5.3382535	3.7087998	101.0199966
-5.3382535	3.7575998	100.5699997
-5.3382535	3.8063998	104.1500015
-5.3382535	3.8551998	104.3899994
-5.3382535	3.9039998	100.6800003
-5.3382535	3.9527998	103.3099976
-5.3382535	4.0016003	105.0199966
-5.3382535	4.0503998	102.0000000
-5.3382535	4.0991998	102.0699997
-5.3382535	4.1479998	102.3699951
-5.3382535	4.1967998	96.7399979
-5.3382535	4.2455997	105.3399963
-5.3382535	4.2943997	106.3099976
-5.3382535	4.3431997	106.7799988
-5.3382535	4.3919997	102.1899948
-5.3382535	4.4407997	102.9899979
-5.3382535	4.4895997	102.4700012
-5.3382535	4.5383997	104.5499954
-5.3382535	4.5871997	103.9199982
-5.3382535	4.6359997	108.7900009
-5.3382535	4.6847997	114.0199966
-5.3382535	4.7336001	111.7900009
-5.3382535	4.7824001	105.6599960
-5.3382535	4.8312001	103.7699966
-5.3382535	4.8800001	105.5199966
-5.3382535	4.9288001	102.5299988
-5.3382535	4.9776001	103.8499985
-5.3382535	5.0264001	105.0400009
-5.3382535	5.0752001	103.1899948
-5.3382535	5.1240001	100.2699966
-5.3382535	5.1728001	100.7399979
-5.3382535	5.2216001	94.4499969
-5.3382535	5.2704000	101.2299957
-5.3382535	5.3192000	105.3300018
-5.3382535	5.3680000	106.0199966
-5.3382535	5.4168000	104.3300018
-5.3382535	5.4656000	106.3300018
-5.3382535	5.5144000	102.4399948
-5.3382535	5.5632000	102.7200012
-5.3382535	5.6120000	104.4199982
-5.3382535	5.6608000	101.9300003
-5.3382535	5.7096000	100.9300003
-5.3382535	5.7584000	101.8399963
-5.3382535	5.8072000	100.3099976
-5.3382535	5.8559999	101.6399994
-5.3382535	5.9047999	99.7099991
-5.3382535	5.9535999	106.8899994
-5.3382535	6.0023999	105.6100006
-5.3382535	6.0511999	104.3699951
-5.3382535	6.0999999	108.2200012
-5.3382535	6.1487999	106.0800018
-5.3382535	6.1975999	102.0299988
-5.3382535	6.2463999	102.0400009
-5.3382535	6.2951999	102.2099991
-5.3382535	6.3439999	101.3499985

-5.3382535	6.3927999	102.1699982
-5.3382535	6.4415998	100.9899979
-5.3382535	6.4903998	102.7999954
-5.3382535	6.5391998	102.8099976
-5.3382535	6.5879998	99.6199951
-5.3382535	6.6367998	101.5800018
-5.3382535	6.6855998	103.7900009
-5.3382535	6.7343998	105.1599960
-5.3382535	6.7831998	101.6599960
-5.3382535	6.8319998	102.5000000
-5.3382535	6.8807998	104.6500015
-5.3382535	6.9295998	105.2399979
-5.3382535	6.9783998	103.0100021
-5.3382535	7.0271997	101.9899979
-5.3382535	7.0759997	102.8600006
-5.3382535	7.1247997	104.3199997
-5.3382535	7.1735997	102.3199997
-5.3382535	7.2223997	104.7799988
-5.3382535	7.2711997	103.7900009
-5.3382535	7.3199997	103.0199966
-5.3382535	7.3687997	99.2099991
-5.3382535	7.4175997	101.3199997
-5.3382535	7.4663997	104.8300018
-5.3382535	7.5151997	105.5899963
-5.3382535	7.5639997	103.4899979
-5.3382535	7.6127996	104.2799988
-5.3382535	7.6615996	107.1999969
-5.3382535	7.7103996	105.7699966
-5.3382535	7.7591996	103.5999985
-5.3382535	7.8079996	104.6199951
-5.3382535	7.8567996	104.9899979
-5.3382535	7.9056001	101.6899948
-5.3382535	7.9544001	105.3099976
-5.3382535	8.0032005	105.7799988
-5.3382535	8.0520000	105.3600006
-5.3382535	8.1008005	106.6999969
-5.3382535	8.1496000	107.7299957
-5.3382535	8.1984005	107.1299973
-5.3382535	8.2472000	104.8099976
-5.3382535	8.2960005	102.7900009
-5.3382535	8.3448000	106.6299973
-5.3382535	8.3936005	106.5199966
-5.3382535	8.4424000	104.6500015
-5.3382535	8.4912004	104.3499985
-5.3382535	8.5400000	103.7699966
-5.3382535	8.5888004	102.0599976
-5.3382535	8.6375999	104.1100006
-5.3382535	8.6864004	103.6599960
-5.3382535	8.7351999	100.1500015
-5.3382535	8.7840004	102.4499969
-5.3382535	8.8327999	104.1399994
-5.3382535	8.8816004	103.6399994
-5.3382535	8.9303999	101.8799973
-5.3382535	8.9792004	102.9399948
-5.3382535	9.0279999	104.0400009
-5.3382535	9.0768003	103.1899948
-5.3382535	9.1255999	103.4700012
-5.3382535	9.1744003	103.2200012

-5.3382535	9.2231998	101.7500000
-5.3382535	9.2720003	103.0800018
-5.3382535	9.3207998	102.6800003
-5.3382535	9.3696003	98.5299988
-5.3382535	9.4184008	101.5899963
-5.3382535	9.4672003	105.1699982
-5.3382535	9.5160007	106.4399948
-5.3382535	9.5648003	104.9300003
-5.3382535	9.6136007	102.5199966
-5.3382535	9.6624002	103.2200012
-5.3382535	9.7112007	103.0699997
-5.3382535	9.7600002	104.7900009
-5.3382535	9.8088007	104.9000015
-5.3382535	9.8576002	105.6899948
-5.3382535	9.9064007	104.5899963
-5.3382535	9.9552002	102.9199982
-5.3382535	10.0040007	102.8699951
-5.3382535	10.0528002	105.1299973
-5.3382535	10.1016006	106.8300018
-5.3382535	10.1504002	107.5400009
-5.3382535	10.1992006	105.8199997
-5.3382535	10.2480001	100.8399963
-5.3382535	10.2968006	101.5400009
-5.3382535	10.3456001	103.5499954
-5.3382535	10.3944006	103.2600021
-5.3382535	10.4432001	103.2900009
-5.3382535	10.4920006	100.9700012
-5.3382535	10.5408001	100.7399979
-5.3382535	10.5896006	102.2399979
-5.3382535	10.6384001	104.0999985
-5.3382535	10.6872005	105.3399963
-5.3382535	10.7360001	103.5800018
-5.3382535	10.7848005	99.8499985
-5.3382535	10.8336000	102.8099976
-5.3382535	10.8824005	103.8799973
-5.3382535	10.9312000	102.1299973
-5.3382535	10.9800005	102.5899963
-5.3382535	11.0288000	103.1699982
-5.3382535	11.0776005	104.2399979
-5.3382535	11.1264000	104.0499954
-5.3382535	11.1752005	106.3499985
-5.3382535	11.2240000	107.5000000
-5.3382535	11.2728004	105.4499969
-5.3382535	11.3216000	103.8499985
-5.3382535	11.3704004	101.9300003
-5.3382535	11.4191999	99.9000015
-5.3382535	11.4680004	104.6399994
-5.3382535	11.5167999	103.5299988
-5.3382535	11.5656004	102.2500000
-5.3382535	11.6143999	102.0499954
-5.3382535	11.6632004	103.1699982
-5.3382535	11.7119999	104.2999954
-5.3382535	11.7608004	105.2399979
-5.3382535	11.8095999	107.7200012
-5.3382535	11.8584003	106.4499969
-5.3382535	11.9071999	105.8399963
-5.3382535	11.9560003	103.9199982
-5.3382535	12.0047998	105.1999969

-5.3382535	12.0536003	104.4399948
-5.3382535	12.1023998	98.5400009
-5.3382535	12.1512003	100.8099976
-5.3382535	12.1999998	103.7999954
-5.3382535	12.2488003	105.9300003
-5.3382535	12.2975998	105.3899994
-5.3382535	12.3464003	103.7099991
-5.3382535	12.3951998	106.3499985
-5.3382535	12.4440002	107.9700012
-5.3382535	12.4927998	106.7999954
-5.3382535	12.5416002	106.6999969
-5.3382535	12.5904007	104.7600021
-5.3382535	12.6392002	104.6199951
-5.3382535	12.6880007	104.9300003
-5.3382535	12.7368002	104.0199966
-5.3382535	12.7856007	100.6599960
-5.3382535	12.8344002	103.4799957
-5.3382535	12.8832006	105.2500000
-5.3382535	12.9320002	105.9199982
-5.3382535	12.9808006	100.3899994
-5.3382535	13.0296001	102.1500015
-5.3382535	13.0784006	101.6899948
-5.3382535	13.1272001	98.5899963
-5.3382535	13.1760006	102.4700012
-5.3382535	13.2248001	104.7200012
-5.3382535	13.2736006	107.4199982
-5.3382535	13.3224001	105.0100021
-5.3382535	13.3712006	100.2699966
-5.3382535	13.4200001	99.0499954
-5.3382535	13.4688005	102.2500000
-5.3382535	13.5176001	105.2600021
-5.3382535	13.5664005	104.6599960
-5.3382535	13.6152000	104.5699997
-5.3382535	13.6640005	103.8699951
-5.3382535	13.7128000	103.9099960
-5.3382535	13.7616005	100.8699951
-5.3382535	13.8104000	102.2099991
-5.3382535	13.8592005	100.1999969
-5.3382535	13.9080000	101.9499969
-5.3382535	13.9568005	98.0899963
-5.3382535	14.0056000	103.4099960
-5.3382535	14.0544004	102.2900009
-5.3382535	14.1032000	100.5800018
-5.3382535	14.1520004	100.2799988
-5.3382535	14.2007999	105.1100006
-5.3382535	14.2496004	106.4799957
-5.3382535	14.2983999	103.8199997
-5.3382535	14.3472004	103.7500000
-5.3382535	14.3959999	101.9199982
-5.3382535	14.4448004	100.0299988
-5.3382535	14.4935999	102.3600006
-5.3382535	14.5424004	104.7299957
-5.3382535	14.5911999	102.9199982
-5.3382535	14.6400003	105.6800003
-5.3382535	14.6887999	105.4300003
-5.3382535	14.7376003	103.3799973
-5.3382535	14.7863998	102.6699982
-5.3382535	14.8352003	102.9199982

-5.3382535	14.8839998	103.3699951
-5.3382535	14.9328003	96.3199997
-5.3382535	14.9815998	102.1100006
-5.3382535	15.0304003	102.9199982
-5.3321176	0.3416000	106.7999954
-5.3321176	0.3904000	106.9799957
-5.3321176	0.4392000	107.3499985
-5.3321176	0.4880000	107.1599960
-5.3321176	0.5368000	105.9700012
-5.3321176	0.5856000	106.1299973
-5.3321176	0.6344000	103.1299973
-5.3321176	0.6832000	105.1199951
-5.3321176	0.7320000	104.3499985
-5.3321176	0.7808000	104.6199951
-5.3321176	0.8296000	102.3499985
-5.3321176	0.8784000	99.4499969
-5.3321176	0.9272000	105.8199997
-5.3321176	0.9760000	105.9899979
-5.3321176	1.0247999	107.8799973
-5.3321176	1.0735999	106.3199997
-5.3321176	1.1223999	106.7500000
-5.3321176	1.1711999	105.0999985
-5.3321176	1.2200000	104.0599976
-5.3321176	1.2688000	105.2699966
-5.3321176	1.3176000	107.2600021
-5.3321176	1.3664000	107.1199951
-5.3321176	1.4152000	107.2200012
-5.3321176	1.4640000	106.8199997
-5.3321176	1.5128000	107.0199966
-5.3321176	1.5616000	104.9399948
-5.3321176	1.6104000	104.4799957
-5.3321176	1.6592000	106.2699966
-5.3321176	1.7079999	106.5000000
-5.3321176	1.7567999	106.9499969
-5.3321176	1.8055999	104.3499985
-5.3321176	1.8543999	104.6199951
-5.3321176	1.9031999	106.5800018
-5.3321176	1.9519999	105.0699997
-5.3321176	2.0007999	99.8699951
-5.3321176	2.0495999	106.8499985
-5.3321176	2.0983999	108.2200012
-5.3321176	2.1471999	107.1999969
-5.3321176	2.1959999	106.2099991
-5.3321176	2.2447999	102.7999954
-5.3321176	2.2935998	100.3499985
-5.3321176	2.3423998	102.2299957
-5.3321176	2.3912001	103.5000000
-5.3321176	2.4400001	104.9099960
-5.3321176	2.4888000	103.6100006
-5.3321176	2.5376000	103.2900009
-5.3321176	2.5864000	105.3099976
-5.3321176	2.6352000	102.7699966
-5.3321176	2.6840000	102.5999985
-5.3321176	2.7328000	105.4300003
-5.3321176	2.7816000	103.1999969
-5.3321176	2.8304000	100.5899963
-5.3321176	2.8792000	104.6299973
-5.3321176	2.9280000	106.5899963

-5.3321176	2.9768000	104.8799973
-5.3321176	3.0256000	105.4599991
-5.3321176	3.0743999	104.8300018
-5.3321176	3.1231999	103.3399963
-5.3321176	3.1719999	99.6399994
-5.3321176	3.2207999	102.2500000
-5.3321176	3.2695999	105.2900009
-5.3321176	3.3183999	103.8199997
-5.3321176	3.3671999	101.8399963
-5.3321176	3.4159999	105.7600021
-5.3321176	3.4647999	106.9799957
-5.3321176	3.5135999	105.4099960
-5.3321176	3.5623999	104.2900009
-5.3321176	3.6111999	102.6999969
-5.3321176	3.6599998	100.2099991
-5.3321176	3.7087998	101.4399948
-5.3321176	3.7575998	100.7999954
-5.3321176	3.8063998	104.3300018
-5.3321176	3.8551998	104.7500000
-5.3321176	3.9039998	100.9399948
-5.3321176	3.9527998	101.9199982
-5.3321176	4.0016003	104.0899963
-5.3321176	4.0503998	102.6399994
-5.3321176	4.0991998	103.4799957
-5.3321176	4.1479998	103.0699997
-5.3321176	4.1967998	99.2200012
-5.3321176	4.2455997	105.8399963
-5.3321176	4.2943997	106.7699966
-5.3321176	4.3431997	107.0899963
-5.3321176	4.3919997	102.2999954
-5.3321176	4.4407997	102.8799973
-5.3321176	4.4895997	102.3399963
-5.3321176	4.5383997	103.3099976
-5.3321176	4.5871997	104.0199966
-5.3321176	4.6359997	109.4000015
-5.3321176	4.6847997	114.2600021
-5.3321176	4.7336001	111.9899979
-5.3321176	4.7824001	105.3899994
-5.3321176	4.8312001	103.5000000
-5.3321176	4.8800001	105.4799957
-5.3321176	4.9288001	102.3399963
-5.3321176	4.9776001	104.4499969
-5.3321176	5.0264001	105.8399963
-5.3321176	5.0752001	103.5699997
-5.3321176	5.1240001	101.4399948
-5.3321176	5.1728001	101.3399963
-5.3321176	5.2216001	95.5199966
-5.3321176	5.2704000	100.0100021
-5.3321176	5.3192000	104.5599976
-5.3321176	5.3680000	106.1599960
-5.3321176	5.4168000	105.0199966
-5.3321176	5.4656000	106.3699951
-5.3321176	5.5144000	102.5000000
-5.3321176	5.5632000	101.3799973
-5.3321176	5.6120000	104.4799957
-5.3321176	5.6608000	102.3199997
-5.3321176	5.7096000	101.8099976
-5.3321176	5.7584000	102.0199966

-5.3321176	5.8072000	100.5899963
-5.3321176	5.8559999	102.5899963
-5.3321176	5.9047999	99.3600006
-5.3321176	5.9535999	106.4599991
-5.3321176	6.0023999	104.8799973
-5.3321176	6.0511999	104.3399963
-5.3321176	6.0999999	107.9199982
-5.3321176	6.1487999	106.1800003
-5.3321176	6.1975999	101.8199997
-5.3321176	6.2463999	101.1699982
-5.3321176	6.2951999	101.0400009
-5.3321176	6.3439999	99.7699966
-5.3321176	6.3927999	101.1199951
-5.3321176	6.4415998	101.0199966
-5.3321176	6.4903998	103.3399963
-5.3321176	6.5391998	103.2900009
-5.3321176	6.5879998	101.0400009
-5.3321176	6.6367998	102.9199982
-5.3321176	6.6855998	104.2399979
-5.3321176	6.7343998	105.4399948
-5.3321176	6.7831998	102.0699997
-5.3321176	6.8319998	102.7200012
-5.3321176	6.8807998	104.0299988
-5.3321176	6.9295998	105.3199997
-5.3321176	6.9783998	103.2099991
-5.3321176	7.0271997	101.8600006
-5.3321176	7.0759997	102.9300003
-5.3321176	7.1247997	104.1899948
-5.3321176	7.1735997	103.6599960
-5.3321176	7.2223997	104.9399948
-5.3321176	7.2711997	103.7600021
-5.3321176	7.3199997	103.2600021
-5.3321176	7.3687997	98.8899994
-5.3321176	7.4175997	101.8499985
-5.3321176	7.4663997	104.8199997
-5.3321176	7.5151997	104.7799988
-5.3321176	7.5639997	102.9899979
-5.3321176	7.6127996	104.0599976
-5.3321176	7.6615996	106.5999985
-5.3321176	7.7103996	104.7999954
-5.3321176	7.7591996	103.0000000
-5.3321176	7.8079996	104.0499954
-5.3321176	7.8567996	105.1100006
-5.3321176	7.9056001	102.6899948
-5.3321176	7.9544001	105.4000015
-5.3321176	8.0032005	105.7900009
-5.3321176	8.0520000	105.9399948
-5.3321176	8.1008005	107.5400009
-5.3321176	8.1496000	107.8399963
-5.3321176	8.1984005	107.5699997
-5.3321176	8.2472000	105.1299973
-5.3321176	8.2960005	104.6399994
-5.3321176	8.3448000	107.3199997
-5.3321176	8.3936005	106.6199951
-5.3321176	8.4424000	103.5499954
-5.3321176	8.4912004	105.1800003
-5.3321176	8.5400000	104.2299957
-5.3321176	8.5888004	102.6500015

-5.3321176	8.6375999	104.5400009
-5.3321176	8.6864004	103.4099960
-5.3321176	8.7351999	98.7200012
-5.3321176	8.7840004	101.7099991
-5.3321176	8.8327999	103.7699966
-5.3321176	8.8816004	103.9899979
-5.3321176	8.9303999	101.8399963
-5.3321176	8.9792004	102.2799988
-5.3321176	9.0279999	104.0699997
-5.3321176	9.0768003	102.6399994
-5.3321176	9.1255999	102.7099991
-5.3321176	9.1744003	102.9899979
-5.3321176	9.2231998	102.6999969
-5.3321176	9.2720003	103.2999954
-5.3321176	9.3207998	102.4399948
-5.3321176	9.3696003	99.6299973
-5.3321176	9.4184008	101.8300018
-5.3321176	9.4672003	104.7399979
-5.3321176	9.5160007	106.0199966
-5.3321176	9.5648003	104.6899948
-5.3321176	9.6136007	102.8300018
-5.3321176	9.6624002	103.1100006
-5.3321176	9.7112007	103.8499985
-5.3321176	9.7600002	105.2299957
-5.3321176	9.8088007	105.3499985
-5.3321176	9.8576002	105.3699951
-5.3321176	9.9064007	104.4199982
-5.3321176	9.9552002	102.5499954
-5.3321176	10.0040007	102.6500015
-5.3321176	10.0528002	104.1100006
-5.3321176	10.1016006	106.5599976
-5.3321176	10.1504002	107.2600021
-5.3321176	10.1992006	105.0599976
-5.3321176	10.2480001	99.3199997
-5.3321176	10.2968006	100.7799988
-5.3321176	10.3456001	102.5299988
-5.3321176	10.3944006	102.3399963
-5.3321176	10.4432001	103.6299973
-5.3321176	10.4920006	101.1100006
-5.3321176	10.5408001	102.0100021
-5.3321176	10.5896006	102.4399948
-5.3321176	10.6384001	103.9700012
-5.3321176	10.6872005	105.3799973
-5.3321176	10.7360001	103.6699982
-5.3321176	10.7848005	98.8699951
-5.3321176	10.8336000	102.8799973
-5.3321176	10.8824005	103.6399994
-5.3321176	10.9312000	101.1299973
-5.3321176	10.9800005	101.2500000
-5.3321176	11.0288000	102.0800018
-5.3321176	11.0776005	104.0800018
-5.3321176	11.1264000	105.0400009
-5.3321176	11.1752005	106.8499985
-5.3321176	11.2240000	107.2600021
-5.3321176	11.2728004	105.0800018
-5.3321176	11.3216000	103.9000015
-5.3321176	11.3704004	102.7799988
-5.3321176	11.4191999	99.5299988

-5.3321176	11.4680004	104.2299957
-5.3321176	11.5167999	103.0499954
-5.3321176	11.5656004	101.1699982
-5.3321176	11.6143999	100.6800003
-5.3321176	11.6632004	102.4700012
-5.3321176	11.7119999	104.4000015
-5.3321176	11.7608004	105.3499985
-5.3321176	11.8095999	107.4099960
-5.3321176	11.8584003	105.8799973
-5.3321176	11.9071999	105.6999969
-5.3321176	11.9560003	104.3699951
-5.3321176	12.0047998	105.0999985
-5.3321176	12.0536003	103.5499954
-5.3321176	12.1023998	100.0999985
-5.3321176	12.1512003	101.5100021
-5.3321176	12.1999998	102.5899963
-5.3321176	12.2488003	104.6299973
-5.3321176	12.2975998	105.3300018
-5.3321176	12.3464003	103.8799973
-5.3321176	12.3951998	106.4499969
-5.3321176	12.4440002	108.2600021
-5.3321176	12.4927998	106.6599960
-5.3321176	12.5416002	106.5699997
-5.3321176	12.5904007	105.0000000
-5.3321176	12.6392002	104.6199951
-5.3321176	12.6880007	105.0899963
-5.3321176	12.7368002	103.3499985
-5.3321176	12.7856007	101.1399994
-5.3321176	12.8344002	102.5100021
-5.3321176	12.8832006	104.5999985
-5.3321176	12.9320002	105.9000015
-5.3321176	12.9808006	101.6800003
-5.3321176	13.0296001	103.2999954
-5.3321176	13.0784006	102.5299988
-5.3321176	13.1272001	98.4899979
-5.3321176	13.1760006	102.2399979
-5.3321176	13.2248001	103.7399979
-5.3321176	13.2736006	106.8300018
-5.3321176	13.3224001	105.0599976
-5.3321176	13.3712006	100.7900009
-5.3321176	13.4200001	99.5899963
-5.3321176	13.4688005	102.2900009
-5.3321176	13.5176001	104.9499969
-5.3321176	13.5664005	104.0199966
-5.3321176	13.6152000	104.0000000
-5.3321176	13.6640005	104.0400009
-5.3321176	13.7128000	103.9099960
-5.3321176	13.7616005	100.7200012
-5.3321176	13.8104000	102.4700012
-5.3321176	13.8592005	100.4599991
-5.3321176	13.9080000	102.4499969
-5.3321176	13.9568005	97.4799957
-5.3321176	14.0056000	103.2099991
-5.3321176	14.0544004	103.1299973
-5.3321176	14.1032000	101.4499969
-5.3321176	14.1520004	100.8699951
-5.3321176	14.2007999	104.8899994
-5.3321176	14.2496004	106.3399963

-5.3321176	14.2983999	104.0999985
-5.3321176	14.3472004	103.9899979
-5.3321176	14.3959999	101.8300018
-5.3321176	14.4448004	100.0699997
-5.3321176	14.4935999	102.4099960
-5.3321176	14.5424004	104.6399994
-5.3321176	14.5911999	101.6100006
-5.3321176	14.6400003	105.1299973
-5.3321176	14.6887999	104.9099960
-5.3321176	14.7376003	102.4799957
-5.3321176	14.7863998	102.8899994
-5.3321176	14.8352003	103.0199966
-5.3321176	14.8839998	104.0499954
-5.3321176	14.9328003	97.8199997
-5.3321176	14.9815998	102.3899994
-5.3321176	15.0304003	103.4300003
-5.3259816	0.3416000	107.3699951
-5.3259816	0.3904000	108.0499954
-5.3259816	0.4392000	108.1500015
-5.3259816	0.4880000	107.9799957
-5.3259816	0.5368000	107.2799988
-5.3259816	0.5856000	105.9799957
-5.3259816	0.6344000	102.2399979
-5.3259816	0.6832000	104.8600006
-5.3259816	0.7320000	102.9099960
-5.3259816	0.7808000	103.0999985
-5.3259816	0.8296000	101.3899994
-5.3259816	0.8784000	99.3499985
-5.3259816	0.9272000	104.1999969
-5.3259816	0.9760000	104.6999969
-5.3259816	1.0247999	108.3399963
-5.3259816	1.0735999	106.9300003
-5.3259816	1.1223999	105.9499969
-5.3259816	1.1711999	105.3899994
-5.3259816	1.2200000	105.6800003
-5.3259816	1.2688000	105.2799988
-5.3259816	1.3176000	106.6699982
-5.3259816	1.3664000	106.0999985
-5.3259816	1.4152000	106.5899963
-5.3259816	1.4640000	106.5599976
-5.3259816	1.5128000	107.0000000
-5.3259816	1.5616000	105.5199966
-5.3259816	1.6104000	105.5899963
-5.3259816	1.6592000	106.3600006
-5.3259816	1.7079999	106.4599991
-5.3259816	1.7567999	107.1100006
-5.3259816	1.8055999	103.3899994
-5.3259816	1.8543999	103.9599991
-5.3259816	1.9031999	106.5499954
-5.3259816	1.9519999	105.6100006
-5.3259816	2.0007999	101.5100021
-5.3259816	2.0495999	107.6800003
-5.3259816	2.0983999	109.0899963
-5.3259816	2.1471999	107.4199982
-5.3259816	2.1959999	106.1500015
-5.3259816	2.2447999	103.5400009
-5.3259816	2.2935998	100.1299973
-5.3259816	2.3423998	102.2500000

-5.3259816	2.3912001	103.8399963
-5.3259816	2.4400001	105.1399994
-5.3259816	2.4888000	103.2500000
-5.3259816	2.5376000	104.1299973
-5.3259816	2.5864000	106.0999985
-5.3259816	2.6352000	102.5100021
-5.3259816	2.6840000	102.9799957
-5.3259816	2.7328000	106.5199966
-5.3259816	2.7816000	104.2699966
-5.3259816	2.8304000	101.0899963
-5.3259816	2.8792000	103.7600021
-5.3259816	2.9280000	105.1299973
-5.3259816	2.9768000	104.2299957
-5.3259816	3.0256000	105.2799988
-5.3259816	3.0743999	104.8899994
-5.3259816	3.1231999	103.3799973
-5.3259816	3.1719999	99.8199997
-5.3259816	3.2207999	102.2500000
-5.3259816	3.2695999	105.0699997
-5.3259816	3.3183999	103.4300003
-5.3259816	3.3671999	101.8699951
-5.3259816	3.4159999	105.6999969
-5.3259816	3.4647999	106.8099976
-5.3259816	3.5135999	106.0400009
-5.3259816	3.5623999	105.4399948
-5.3259816	3.6111999	102.9799957
-5.3259816	3.6599998	100.5599976
-5.3259816	3.7087998	101.9899979
-5.3259816	3.7575998	100.4399948
-5.3259816	3.8063998	104.1999969
-5.3259816	3.8551998	105.3600006
-5.3259816	3.9039998	101.4300003
-5.3259816	3.9527998	101.0599976
-5.3259816	4.0016003	103.7099991
-5.3259816	4.0503998	103.2900009
-5.3259816	4.0991998	103.9199982
-5.3259816	4.1479998	103.2600021
-5.3259816	4.1967998	100.6500015
-5.3259816	4.2455997	105.7799988
-5.3259816	4.2943997	107.0199966
-5.3259816	4.3431997	107.5000000
-5.3259816	4.3919997	102.6899948
-5.3259816	4.4407997	103.0000000
-5.3259816	4.4895997	102.0800018
-5.3259816	4.5383997	101.9099960
-5.3259816	4.5871997	103.6899948
-5.3259816	4.6359997	110.0899963
-5.3259816	4.6847997	114.6899948
-5.3259816	4.7336001	112.5699997
-5.3259816	4.7824001	105.8899994
-5.3259816	4.8312001	104.2999954
-5.3259816	4.8800001	105.6500015
-5.3259816	4.9288001	102.8199997
-5.3259816	4.9776001	105.1199951
-5.3259816	5.0264001	105.7900009
-5.3259816	5.0752001	103.7399979
-5.3259816	5.1240001	102.7399979
-5.3259816	5.1728001	101.8600006

-5.3259816	5.2216001	96.6199951
-5.3259816	5.2704000	98.7500000
-5.3259816	5.3192000	103.3099976
-5.3259816	5.3680000	106.1299973
-5.3259816	5.4168000	105.4799957
-5.3259816	5.4656000	106.0199966
-5.3259816	5.5144000	102.0400009
-5.3259816	5.5632000	100.2399979
-5.3259816	5.6120000	104.4099960
-5.3259816	5.6608000	102.4300003
-5.3259816	5.7096000	103.1299973
-5.3259816	5.7584000	103.1399994
-5.3259816	5.8072000	101.6800003
-5.3259816	5.8559999	103.7799988
-5.3259816	5.9047999	98.9499969
-5.3259816	5.9535999	105.9599991
-5.3259816	6.0023999	105.5000000
-5.3259816	6.0511999	104.9000015
-5.3259816	6.0999999	107.7600021
-5.3259816	6.1487999	106.2200012
-5.3259816	6.1975999	102.1399994
-5.3259816	6.2463999	101.4300003
-5.3259816	6.2951999	100.5800018
-5.3259816	6.3439999	99.6100006
-5.3259816	6.3927999	100.5999985
-5.3259816	6.4415998	100.0299988
-5.3259816	6.4903998	103.1899948
-5.3259816	6.5391998	103.7699966
-5.3259816	6.5879998	102.2399979
-5.3259816	6.6367998	104.0100021
-5.3259816	6.6855998	104.5899963
-5.3259816	6.7343998	105.2500000
-5.3259816	6.7831998	101.6699982
-5.3259816	6.8319998	102.7799988
-5.3259816	6.8807998	103.6999969
-5.3259816	6.9295998	105.3499985
-5.3259816	6.9783998	103.4199982
-5.3259816	7.0271997	101.6100006
-5.3259816	7.0759997	102.6100006
-5.3259816	7.1247997	103.5499954
-5.3259816	7.1735997	104.1599960
-5.3259816	7.2223997	104.5000000
-5.3259816	7.2711997	103.2299957
-5.3259816	7.3199997	102.1699982
-5.3259816	7.3687997	98.9599991
-5.3259816	7.4175997	102.5499954
-5.3259816	7.4663997	104.7600021
-5.3259816	7.5151997	104.7500000
-5.3259816	7.5639997	102.1899948
-5.3259816	7.6127996	103.4599991
-5.3259816	7.6615996	104.9399948
-5.3259816	7.7103996	104.3600006
-5.3259816	7.7591996	102.6899948
-5.3259816	7.8079996	102.9300003
-5.3259816	7.8567996	104.0999985
-5.3259816	7.9056001	102.7900009
-5.3259816	7.9544001	105.0999985
-5.3259816	8.0032005	105.9199982

-5.3259816	8.0520000	106.4599991
-5.3259816	8.1008005	107.7399979
-5.3259816	8.1496000	107.6899948
-5.3259816	8.1984005	107.4700012
-5.3259816	8.2472000	104.7200012
-5.3259816	8.2960005	105.5299988
-5.3259816	8.3448000	107.3799973
-5.3259816	8.3936005	106.3699951
-5.3259816	8.4424000	103.1100006
-5.3259816	8.4912004	105.3399963
-5.3259816	8.5400000	103.4099960
-5.3259816	8.5888004	102.3899994
-5.3259816	8.6375999	104.7600021
-5.3259816	8.6864004	103.5800018
-5.3259816	8.7351999	99.2999954
-5.3259816	8.7840004	101.4300003
-5.3259816	8.8327999	103.3099976
-5.3259816	8.8816004	104.4199982
-5.3259816	8.9303999	102.6899948
-5.3259816	8.9792004	101.5199966
-5.3259816	9.0279999	103.4199982
-5.3259816	9.0768003	102.0699997
-5.3259816	9.1255999	102.0999985
-5.3259816	9.1744003	102.9000015
-5.3259816	9.2231998	102.6500015
-5.3259816	9.2720003	103.0299988
-5.3259816	9.3207998	101.4799957
-5.3259816	9.3696003	99.7900009
-5.3259816	9.4184008	101.8300018
-5.3259816	9.4672003	104.5199966
-5.3259816	9.5160007	105.5999985
-5.3259816	9.5648003	104.4599991
-5.3259816	9.6136007	103.8799973
-5.3259816	9.6624002	103.4399948
-5.3259816	9.7112007	104.5599976
-5.3259816	9.7600002	105.5699997
-5.3259816	9.8088007	105.9099960
-5.3259816	9.8576002	105.0400009
-5.3259816	9.9064007	104.0199966
-5.3259816	9.9552002	102.1899948
-5.3259816	10.0040007	102.4000015
-5.3259816	10.0528002	103.0199966
-5.3259816	10.1016006	106.2099991
-5.3259816	10.1504002	106.7500000
-5.3259816	10.1992006	104.6699982
-5.3259816	10.2480001	99.5999985
-5.3259816	10.2968006	101.0699997
-5.3259816	10.3456001	102.5199966
-5.3259816	10.3944006	102.4499969
-5.3259816	10.4432001	104.5000000
-5.3259816	10.4920006	102.5299988
-5.3259816	10.5408001	103.1399994
-5.3259816	10.5896006	102.4099960
-5.3259816	10.6384001	103.4399948
-5.3259816	10.6872005	105.1399994
-5.3259816	10.7360001	103.3199997
-5.3259816	10.7848005	98.5699997
-5.3259816	10.8336000	103.1699982

-5.3259816	10.8824005	103.2699966
-5.3259816	10.9312000	100.3600006
-5.3259816	10.9800005	100.9399948
-5.3259816	11.0288000	100.5699997
-5.3259816	11.0776005	103.2900009
-5.3259816	11.1264000	105.5499954
-5.3259816	11.1752005	107.0299988
-5.3259816	11.2240000	106.9599991
-5.3259816	11.2728004	105.1899948
-5.3259816	11.3216000	103.6399994
-5.3259816	11.3704004	102.7600021
-5.3259816	11.4191999	99.8899994
-5.3259816	11.4680004	104.0400009
-5.3259816	11.5167999	102.4000015
-5.3259816	11.5656004	100.2600021
-5.3259816	11.6143999	99.4599991
-5.3259816	11.6632004	101.8199997
-5.3259816	11.7119999	104.2999954
-5.3259816	11.7608004	104.9700012
-5.3259816	11.8095999	106.0800018
-5.3259816	11.8584003	105.2900009
-5.3259816	11.9071999	105.4899979
-5.3259816	11.9560003	104.3399963
-5.3259816	12.0047998	104.3799973
-5.3259816	12.0536003	102.6399994
-5.3259816	12.1023998	101.8699951
-5.3259816	12.1512003	102.7200012
-5.3259816	12.1999998	102.1599960
-5.3259816	12.2488003	103.4799957
-5.3259816	12.2975998	105.1199951
-5.3259816	12.3464003	103.7099991
-5.3259816	12.3951998	106.0499954
-5.3259816	12.4440002	108.2099991
-5.3259816	12.4927998	106.3600006
-5.3259816	12.5416002	105.9599991
-5.3259816	12.5904007	104.8399963
-5.3259816	12.6392002	104.1599960
-5.3259816	12.6880007	104.4799957
-5.3259816	12.7368002	102.1999969
-5.3259816	12.7856007	101.9799957
-5.3259816	12.8344002	102.3399963
-5.3259816	12.8832006	103.7799988
-5.3259816	12.9320002	105.4499969
-5.3259816	12.9808006	102.7699966
-5.3259816	13.0296001	104.7799988
-5.3259816	13.0784006	103.6199951
-5.3259816	13.1272001	98.1699982
-5.3259816	13.1760006	101.7699966
-5.3259816	13.2248001	103.3199997
-5.3259816	13.2736006	106.0899963
-5.3259816	13.3224001	104.8300018
-5.3259816	13.3712006	101.4399948
-5.3259816	13.4200001	100.3199997
-5.3259816	13.4688005	103.1999969
-5.3259816	13.5176001	104.9000015
-5.3259816	13.5664005	103.1999969
-5.3259816	13.6152000	103.8399963
-5.3259816	13.6640005	104.6100006

-5.3259816	13.7128000	103.9899979
-5.3259816	13.7616005	100.3199997
-5.3259816	13.8104000	102.7500000
-5.3259816	13.8592005	100.5699997
-5.3259816	13.9080000	103.0899963
-5.3259816	13.9568005	98.8799973
-5.3259816	14.0056000	102.7099991
-5.3259816	14.0544004	103.0299988
-5.3259816	14.1032000	101.7600021
-5.3259816	14.1520004	101.0800018
-5.3259816	14.2007999	104.2299957
-5.3259816	14.2496004	106.1999969
-5.3259816	14.2983999	104.9300003
-5.3259816	14.3472004	104.0899963
-5.3259816	14.3959999	101.6599960
-5.3259816	14.4448004	100.5100021
-5.3259816	14.4935999	102.4899979
-5.3259816	14.5424004	104.2799988
-5.3259816	14.5911999	100.7600021
-5.3259816	14.6400003	104.6500015
-5.3259816	14.6887999	104.5100021
-5.3259816	14.7376003	101.8699951
-5.3259816	14.7863998	102.9499969
-5.3259816	14.8352003	102.7999954
-5.3259816	14.8839998	104.7799988
-5.3259816	14.9328003	99.7699966
-5.3259816	14.9815998	102.6399994
-5.3259816	15.0304003	104.3399963
-5.3198457	0.3416000	106.4300003
-5.3198457	0.3904000	108.3600006
-5.3198457	0.4392000	108.4899979
-5.3198457	0.4880000	108.2900009
-5.3198457	0.5368000	108.3899994
-5.3198457	0.5856000	106.3199997
-5.3198457	0.6344000	103.0400009
-5.3198457	0.6832000	105.4599991
-5.3198457	0.7320000	102.4099960
-5.3198457	0.7808000	102.4799957
-5.3198457	0.8296000	101.4700012
-5.3198457	0.8784000	99.7500000
-5.3198457	0.9272000	103.0000000
-5.3198457	0.9760000	105.2699966
-5.3198457	1.0247999	108.6699982
-5.3198457	1.0735999	106.7900009
-5.3198457	1.1223999	105.4499969
-5.3198457	1.1711999	106.0100021
-5.3198457	1.2200000	106.2500000
-5.3198457	1.2688000	103.8899994
-5.3198457	1.3176000	105.9700012
-5.3198457	1.3664000	105.3899994
-5.3198457	1.4152000	106.7999954
-5.3198457	1.4640000	106.9499969
-5.3198457	1.5128000	106.8099976
-5.3198457	1.5616000	105.5899963
-5.3198457	1.6104000	105.3699951
-5.3198457	1.6592000	106.2299957
-5.3198457	1.7079999	105.9700012
-5.3198457	1.7567999	107.1399994

-5.3198457	1.8055999	102.9499969
-5.3198457	1.8543999	104.0699997
-5.3198457	1.9031999	106.4499969
-5.3198457	1.9519999	105.9899979
-5.3198457	2.0007999	103.2600021
-5.3198457	2.0495999	107.6999969
-5.3198457	2.0983999	109.2799988
-5.3198457	2.1471999	107.4799957
-5.3198457	2.1959999	106.1199951
-5.3198457	2.2447999	104.1800003
-5.3198457	2.2935998	100.9000015
-5.3198457	2.3423998	101.9300003
-5.3198457	2.3912001	103.8300018
-5.3198457	2.4400001	105.4199982
-5.3198457	2.4888000	103.4000015
-5.3198457	2.5376000	105.1699982
-5.3198457	2.5864000	106.8099976
-5.3198457	2.6352000	102.0400009
-5.3198457	2.6840000	102.5400009
-5.3198457	2.7328000	106.8699951
-5.3198457	2.7816000	104.9000015
-5.3198457	2.8304000	101.3600006
-5.3198457	2.8792000	103.7399979
-5.3198457	2.9280000	104.2500000
-5.3198457	2.9768000	104.6100006
-5.3198457	3.0256000	105.4799957
-5.3198457	3.0743999	104.7999954
-5.3198457	3.1231999	103.1500015
-5.3198457	3.1719999	100.0699997
-5.3198457	3.2207999	102.6199951
-5.3198457	3.2695999	104.9899979
-5.3198457	3.3183999	103.0800018
-5.3198457	3.3671999	101.5599976
-5.3198457	3.4159999	104.9099960
-5.3198457	3.4647999	105.9700012
-5.3198457	3.5135999	105.9199982
-5.3198457	3.5623999	106.0299988
-5.3198457	3.6111999	103.0199966
-5.3198457	3.6599998	100.9199982
-5.3198457	3.7087998	102.2299957
-5.3198457	3.7575998	100.2900009
-5.3198457	3.8063998	103.6199951
-5.3198457	3.8551998	105.7999954
-5.3198457	3.9039998	101.4599991
-5.3198457	3.9527998	101.0599976
-5.3198457	4.0016003	104.1999969
-5.3198457	4.0503998	104.9199982
-5.3198457	4.0991998	104.8899994
-5.3198457	4.1479998	102.7999954
-5.3198457	4.1967998	101.8600006
-5.3198457	4.2455997	105.2200012
-5.3198457	4.2943997	106.8799973
-5.3198457	4.3431997	107.6599960
-5.3198457	4.3919997	103.8099976
-5.3198457	4.4407997	103.2399979
-5.3198457	4.4895997	101.9000015
-5.3198457	4.5383997	100.1699982
-5.3198457	4.5871997	103.8300018

-5.3198457	4.6359997	110.3099976
-5.3198457	4.6847997	114.1599960
-5.3198457	4.7336001	112.3899994
-5.3198457	4.7824001	106.0499954
-5.3198457	4.8312001	105.4099960
-5.3198457	4.8800001	106.3600006
-5.3198457	4.9288001	103.3499985
-5.3198457	4.9776001	105.5000000
-5.3198457	5.0264001	105.2799988
-5.3198457	5.0752001	103.2500000
-5.3198457	5.1240001	103.9000015
-5.3198457	5.1728001	102.9399948
-5.3198457	5.2216001	98.4399948
-5.3198457	5.2704000	98.0800018
-5.3198457	5.3192000	102.5199966
-5.3198457	5.3680000	106.1599960
-5.3198457	5.4168000	105.3199997
-5.3198457	5.4656000	104.9899979
-5.3198457	5.5144000	101.4899979
-5.3198457	5.5632000	100.8099976
-5.3198457	5.6120000	104.0899963
-5.3198457	5.6608000	102.1100006
-5.3198457	5.7096000	103.0999985
-5.3198457	5.7584000	103.4700012
-5.3198457	5.8072000	101.8099976
-5.3198457	5.8559999	104.1599960
-5.3198457	5.9047999	99.6599960
-5.3198457	5.9535999	104.7600021
-5.3198457	6.0023999	105.2500000
-5.3198457	6.0511999	104.6399994
-5.3198457	6.0999999	107.2799988
-5.3198457	6.1487999	106.3099976
-5.3198457	6.1975999	103.0499954
-5.3198457	6.2463999	101.9199982
-5.3198457	6.2951999	101.2900009
-5.3198457	6.3439999	100.6800003
-5.3198457	6.3927999	101.2200012
-5.3198457	6.4415998	99.7299957
-5.3198457	6.4903998	103.3300018
-5.3198457	6.5391998	104.9799957
-5.3198457	6.5879998	103.9399948
-5.3198457	6.6367998	105.1299973
-5.3198457	6.6855998	104.3699951
-5.3198457	6.7343998	104.5199966
-5.3198457	6.7831998	101.0499954
-5.3198457	6.8319998	103.6800003
-5.3198457	6.8807998	104.0400009
-5.3198457	6.9295998	105.2900009
-5.3198457	6.9783998	103.1899948
-5.3198457	7.0271997	101.7299957
-5.3198457	7.0759997	102.5000000
-5.3198457	7.1247997	102.7099991
-5.3198457	7.1735997	103.3199997
-5.3198457	7.2223997	104.2399979
-5.3198457	7.2711997	103.5299988
-5.3198457	7.3199997	101.7799988
-5.3198457	7.3687997	99.5699997
-5.3198457	7.4175997	103.5400009

-5.3198457	7.4663997	104.6800003
-5.3198457	7.5151997	104.4099960
-5.3198457	7.5639997	101.6999969
-5.3198457	7.6127996	103.2399979
-5.3198457	7.6615996	103.5100021
-5.3198457	7.7103996	104.1500015
-5.3198457	7.7591996	102.1500015
-5.3198457	7.8079996	101.7500000
-5.3198457	7.8567996	103.0199966
-5.3198457	7.9056001	103.1599960
-5.3198457	7.9544001	104.9700012
-5.3198457	8.0032005	105.5599976
-5.3198457	8.0520000	106.1899948
-5.3198457	8.1008005	107.3899994
-5.3198457	8.1496000	107.2399979
-5.3198457	8.1984005	107.3099976
-5.3198457	8.2472000	104.9399948
-5.3198457	8.2960005	106.5199966
-5.3198457	8.3448000	107.2600021
-5.3198457	8.3936005	106.2900009
-5.3198457	8.4424000	103.4700012
-5.3198457	8.4912004	105.1500015
-5.3198457	8.5400000	102.3799973
-5.3198457	8.5888004	101.9899979
-5.3198457	8.6375999	104.0999985
-5.3198457	8.6864004	103.5000000
-5.3198457	8.7351999	100.2500000
-5.3198457	8.7840004	101.7699966
-5.3198457	8.8327999	103.3699951
-5.3198457	8.8816004	104.8899994
-5.3198457	8.9303999	103.5999985
-5.3198457	8.9792004	100.9000015
-5.3198457	9.0279999	102.7099991
-5.3198457	9.0768003	101.4599991
-5.3198457	9.1255999	101.8499985
-5.3198457	9.1744003	103.4899979
-5.3198457	9.2231998	103.2600021
-5.3198457	9.2720003	103.5599976
-5.3198457	9.3207998	102.1199951
-5.3198457	9.3696003	100.2200012
-5.3198457	9.4184008	101.4099960
-5.3198457	9.4672003	104.0800018
-5.3198457	9.5160007	105.2200012
-5.3198457	9.5648003	104.0400009
-5.3198457	9.6136007	104.8300018
-5.3198457	9.6624002	103.7099991
-5.3198457	9.7112007	104.5499954
-5.3198457	9.7600002	105.5400009
-5.3198457	9.8088007	105.6599960
-5.3198457	9.8576002	104.7900009
-5.3198457	9.9064007	103.2699966
-5.3198457	9.9552002	101.7299957
-5.3198457	10.0040007	102.3399963
-5.3198457	10.0528002	102.9700012
-5.3198457	10.1016006	106.3799973
-5.3198457	10.1504002	106.5000000
-5.3198457	10.1992006	104.6800003
-5.3198457	10.2480001	100.3799973

-5.3198457	10.2968006	101.6199951
-5.3198457	10.3456001	102.9399948
-5.3198457	10.3944006	102.7200012
-5.3198457	10.4432001	104.7699966
-5.3198457	10.4920006	103.3099976
-5.3198457	10.5408001	103.8799973
-5.3198457	10.5896006	102.9199982
-5.3198457	10.6384001	103.3699951
-5.3198457	10.6872005	105.1599960
-5.3198457	10.7360001	103.2799988
-5.3198457	10.7848005	98.1500015
-5.3198457	10.8336000	103.3799973
-5.3198457	10.8824005	103.0699997
-5.3198457	10.9312000	100.6399994
-5.3198457	10.9800005	101.1199951
-5.3198457	11.0288000	100.2699966
-5.3198457	11.0776005	103.2299957
-5.3198457	11.1264000	105.9700012
-5.3198457	11.1752005	106.6800003
-5.3198457	11.2240000	106.5899963
-5.3198457	11.2728004	105.3899994
-5.3198457	11.3216000	103.6299973
-5.3198457	11.3704004	102.9700012
-5.3198457	11.4191999	101.1599960
-5.3198457	11.4680004	104.5499954
-5.3198457	11.5167999	103.3399963
-5.3198457	11.5656004	101.1100006
-5.3198457	11.6143999	98.8799973
-5.3198457	11.6632004	101.6599960
-5.3198457	11.7119999	104.4300003
-5.3198457	11.7608004	104.6899948
-5.3198457	11.8095999	105.1100006
-5.3198457	11.8584003	104.9599991
-5.3198457	11.9071999	105.3300018
-5.3198457	11.9560003	103.8399963
-5.3198457	12.0047998	103.4000015
-5.3198457	12.0536003	101.7299957
-5.3198457	12.1023998	103.3600006
-5.3198457	12.1512003	103.8300018
-5.3198457	12.1999998	101.9300003
-5.3198457	12.2488003	102.4300003
-5.3198457	12.2975998	105.0100021
-5.3198457	12.3464003	104.0599976
-5.3198457	12.3951998	105.5199966
-5.3198457	12.4440002	107.8499985
-5.3198457	12.4927998	105.7999954
-5.3198457	12.5416002	105.0100021
-5.3198457	12.5904007	104.5699997
-5.3198457	12.6392002	104.2299957
-5.3198457	12.6880007	103.8799973
-5.3198457	12.7368002	101.0199966
-5.3198457	12.7856007	102.9099960
-5.3198457	12.8344002	101.8899994
-5.3198457	12.8832006	103.7200012
-5.3198457	12.9320002	104.7999954
-5.3198457	12.9808006	103.1599960
-5.3198457	13.0296001	105.3799973
-5.3198457	13.0784006	104.2799988

-5.3198457	13.1272001	98.5599976
-5.3198457	13.1760006	101.1199951
-5.3198457	13.2248001	102.2999954
-5.3198457	13.2736006	104.9199982
-5.3198457	13.3224001	104.4499969
-5.3198457	13.3712006	102.0499954
-5.3198457	13.4200001	101.6500015
-5.3198457	13.4688005	104.1100006
-5.3198457	13.5176001	104.6399994
-5.3198457	13.5664005	102.0599976
-5.3198457	13.6152000	103.1699982
-5.3198457	13.6640005	104.8199997
-5.3198457	13.7128000	103.7399979
-5.3198457	13.7616005	101.6500015
-5.3198457	13.8104000	103.5999985
-5.3198457	13.8592005	100.7999954
-5.3198457	13.9080000	103.3099976
-5.3198457	13.9568005	100.3899994
-5.3198457	14.0056000	101.9300003
-5.3198457	14.0544004	103.0199966
-5.3198457	14.1032000	102.4300003
-5.3198457	14.1520004	101.5599976
-5.3198457	14.2007999	103.4000015
-5.3198457	14.2496004	105.5100021
-5.3198457	14.2983999	104.7999954
-5.3198457	14.3472004	104.0599976
-5.3198457	14.3959999	102.0899963
-5.3198457	14.4448004	101.5400009
-5.3198457	14.4935999	102.5000000
-5.3198457	14.5424004	103.5800018
-5.3198457	14.5911999	99.7099991
-5.3198457	14.6400003	103.8899994
-5.3198457	14.6887999	103.6500015
-5.3198457	14.7376003	100.9199982
-5.3198457	14.7863998	102.7799988
-5.3198457	14.8352003	103.0699997
-5.3198457	14.8839998	105.6299973
-5.3198457	14.9328003	101.5000000
-5.3198457	14.9815998	102.8799973
-5.3198457	15.0304003	105.2200012
-5.3137097	0.3416000	104.9899979
-5.3137097	0.3904000	108.5499954
-5.3137097	0.4392000	108.8300018
-5.3137097	0.4880000	108.6699982
-5.3137097	0.5368000	109.2099991
-5.3137097	0.5856000	106.1100006
-5.3137097	0.6344000	104.1599960
-5.3137097	0.6832000	106.1199951
-5.3137097	0.7320000	102.1699982
-5.3137097	0.7808000	102.7299957
-5.3137097	0.8296000	102.2200012
-5.3137097	0.8784000	100.4499969
-5.3137097	0.9272000	101.2999954
-5.3137097	0.9760000	105.3699951
-5.3137097	1.0247999	108.5400009
-5.3137097	1.0735999	105.5499954
-5.3137097	1.1223999	104.5299988
-5.3137097	1.1711999	106.6199951

-5.3137097	1.2200000	107.0499954
-5.3137097	1.2688000	103.0899963
-5.3137097	1.3176000	106.0599976
-5.3137097	1.3664000	105.0999985
-5.3137097	1.4152000	106.9000015
-5.3137097	1.4640000	107.3199997
-5.3137097	1.5128000	106.9399948
-5.3137097	1.5616000	106.0100021
-5.3137097	1.6104000	105.7099991
-5.3137097	1.6592000	106.6199951
-5.3137097	1.7079999	105.5800018
-5.3137097	1.7567999	107.1100006
-5.3137097	1.8055999	102.2399979
-5.3137097	1.8543999	103.4499969
-5.3137097	1.9031999	105.5199966
-5.3137097	1.9519999	105.4099960
-5.3137097	2.0007999	104.4300003
-5.3137097	2.0495999	107.0499954
-5.3137097	2.0983999	108.9499969
-5.3137097	2.1471999	107.5699997
-5.3137097	2.1959999	106.6899948
-5.3137097	2.2447999	104.8399963
-5.3137097	2.2935998	101.6199951
-5.3137097	2.3423998	102.2799988
-5.3137097	2.3912001	103.9499969
-5.3137097	2.4400001	106.0100021
-5.3137097	2.4888000	104.9199982
-5.3137097	2.5376000	106.6699982
-5.3137097	2.5864000	107.0199966
-5.3137097	2.6352000	101.3600006
-5.3137097	2.6840000	103.0299988
-5.3137097	2.7328000	106.6399994
-5.3137097	2.7816000	105.4799957
-5.3137097	2.8304000	102.1399994
-5.3137097	2.8792000	103.6299973
-5.3137097	2.9280000	103.2399979
-5.3137097	2.9768000	105.0000000
-5.3137097	3.0256000	105.5899963
-5.3137097	3.0743999	104.7799988
-5.3137097	3.1231999	103.4300003
-5.3137097	3.1719999	100.6299973
-5.3137097	3.2207999	103.1199951
-5.3137097	3.2695999	104.1599960
-5.3137097	3.3183999	101.9899979
-5.3137097	3.3671999	100.9199982
-5.3137097	3.4159999	104.2200012
-5.3137097	3.4647999	104.8399963
-5.3137097	3.5135999	105.1800003
-5.3137097	3.5623999	105.9099960
-5.3137097	3.6111999	102.9399948
-5.3137097	3.6599998	101.0699997
-5.3137097	3.7087998	102.2999954
-5.3137097	3.7575998	101.0899963
-5.3137097	3.8063998	104.5800018
-5.3137097	3.8551998	106.5599976
-5.3137097	3.9039998	101.8099976
-5.3137097	3.9527998	101.5299988
-5.3137097	4.0016003	104.8899994

-5.3137097	4.0503998	106.0899963
-5.3137097	4.0991998	105.7799988
-5.3137097	4.1479998	102.4399948
-5.3137097	4.1967998	103.9700012
-5.3137097	4.2455997	105.0100021
-5.3137097	4.2943997	105.6699982
-5.3137097	4.3431997	107.6500015
-5.3137097	4.3919997	104.5199966
-5.3137097	4.4407997	103.3499985
-5.3137097	4.4895997	102.4300003
-5.3137097	4.5383997	99.9099960
-5.3137097	4.5871997	103.4799957
-5.3137097	4.6359997	110.5800018
-5.3137097	4.6847997	113.9700012
-5.3137097	4.7336001	112.6299973
-5.3137097	4.7824001	106.5899963
-5.3137097	4.8312001	106.5899963
-5.3137097	4.8800001	107.3699951
-5.3137097	4.9288001	104.8199997
-5.3137097	4.9776001	105.9899979
-5.3137097	5.0264001	104.4199982
-5.3137097	5.0752001	102.2200012
-5.3137097	5.1240001	104.2699966
-5.3137097	5.1728001	103.6899948
-5.3137097	5.2216001	100.3099976
-5.3137097	5.2704000	98.2099991
-5.3137097	5.3192000	101.4199982
-5.3137097	5.3680000	106.0699997
-5.3137097	5.4168000	105.2999954
-5.3137097	5.4656000	104.1100006
-5.3137097	5.5144000	100.5999985
-5.3137097	5.5632000	101.4300003
-5.3137097	5.6120000	103.8099976
-5.3137097	5.6608000	102.5299988
-5.3137097	5.7096000	103.4399948
-5.3137097	5.7584000	104.2299957
-5.3137097	5.8072000	103.0699997
-5.3137097	5.8559999	104.7900009
-5.3137097	5.9047999	100.8099976
-5.3137097	5.9535999	102.9199982
-5.3137097	6.0023999	104.7900009
-5.3137097	6.0511999	103.9599991
-5.3137097	6.0999999	106.0800018
-5.3137097	6.1487999	105.9099960
-5.3137097	6.1975999	103.4099960
-5.3137097	6.2463999	101.9899979
-5.3137097	6.2951999	102.4599991
-5.3137097	6.3439999	102.7200012
-5.3137097	6.3927999	102.7299957
-5.3137097	6.4415998	100.4399948
-5.3137097	6.4903998	103.5499954
-5.3137097	6.5391998	105.7699966
-5.3137097	6.5879998	104.8300018
-5.3137097	6.6367998	105.4099960
-5.3137097	6.6855998	104.1299973
-5.3137097	6.7343998	103.7799988
-5.3137097	6.7831998	100.0100021
-5.3137097	6.8319998	104.0400009

-5.3137097	6.8807998	104.0699997
-5.3137097	6.9295998	105.3099976
-5.3137097	6.9783998	103.5999985
-5.3137097	7.0271997	102.7299957
-5.3137097	7.0759997	102.9000015
-5.3137097	7.1247997	102.5499954
-5.3137097	7.1735997	102.6599960
-5.3137097	7.2223997	104.1999969
-5.3137097	7.2711997	104.2099991
-5.3137097	7.3199997	101.3600006
-5.3137097	7.3687997	100.3099976
-5.3137097	7.4175997	104.4599991
-5.3137097	7.4663997	105.0699997
-5.3137097	7.5151997	104.5699997
-5.3137097	7.5639997	101.7399979
-5.3137097	7.6127996	102.8699951
-5.3137097	7.6615996	101.0699997
-5.3137097	7.7103996	103.7299957
-5.3137097	7.7591996	101.5100021
-5.3137097	7.8079996	101.9700012
-5.3137097	7.8567996	102.1299973
-5.3137097	7.9056001	102.8099976
-5.3137097	7.9544001	104.5000000
-5.3137097	8.0032005	104.9300003
-5.3137097	8.0520000	105.3300018
-5.3137097	8.1008005	106.7200012
-5.3137097	8.1496000	106.9700012
-5.3137097	8.1984005	107.3300018
-5.3137097	8.2472000	104.9599991
-5.3137097	8.2960005	106.9499969
-5.3137097	8.3448000	107.0699997
-5.3137097	8.3936005	105.9700012
-5.3137097	8.4424000	103.6299973
-5.3137097	8.4912004	104.7099991
-5.3137097	8.5400000	101.6100006
-5.3137097	8.5888004	101.4199982
-5.3137097	8.6375999	102.7699966
-5.3137097	8.6864004	103.1299973
-5.3137097	8.7351999	101.8199997
-5.3137097	8.7840004	103.2799988
-5.3137097	8.8327999	103.8399963
-5.3137097	8.8816004	105.0599976
-5.3137097	8.9303999	104.2099991
-5.3137097	8.9792004	100.5699997
-5.3137097	9.0279999	102.0899963
-5.3137097	9.0768003	101.3300018
-5.3137097	9.1255999	102.1199951
-5.3137097	9.1744003	103.8499985
-5.3137097	9.2231998	103.1699982
-5.3137097	9.2720003	103.6399994
-5.3137097	9.3207998	102.7500000
-5.3137097	9.3696003	101.0100021
-5.3137097	9.4184008	101.3199997
-5.3137097	9.4672003	103.6699982
-5.3137097	9.5160007	104.9499969
-5.3137097	9.5648003	104.0499954
-5.3137097	9.6136007	105.3600006
-5.3137097	9.6624002	104.0899963

-5.3137097	9.7112007	104.0499954
-5.3137097	9.7600002	105.2900009
-5.3137097	9.8088007	105.3199997
-5.3137097	9.8576002	104.3499985
-5.3137097	9.9064007	102.1599960
-5.3137097	9.9552002	101.3099976
-5.3137097	10.0040007	102.5199966
-5.3137097	10.0528002	103.1299973
-5.3137097	10.1016006	106.0999985
-5.3137097	10.1504002	105.2500000
-5.3137097	10.1992006	104.6599960
-5.3137097	10.2480001	101.5400009
-5.3137097	10.2968006	102.0800018
-5.3137097	10.3456001	103.4000015
-5.3137097	10.3944006	103.6999969
-5.3137097	10.4432001	105.2399979
-5.3137097	10.4920006	103.7500000
-5.3137097	10.5408001	103.8600006
-5.3137097	10.5896006	103.3499985
-5.3137097	10.6384001	103.2999954
-5.3137097	10.6872005	104.8600006
-5.3137097	10.7360001	103.2699966
-5.3137097	10.7848005	98.7999954
-5.3137097	10.8336000	103.5100021
-5.3137097	10.8824005	103.5699997
-5.3137097	10.9312000	101.2799988
-5.3137097	10.9800005	101.9799957
-5.3137097	11.0288000	100.4700012
-5.3137097	11.0776005	103.4499969
-5.3137097	11.1264000	106.2900009
-5.3137097	11.1752005	106.0100021
-5.3137097	11.2240000	106.1699982
-5.3137097	11.2728004	105.9799957
-5.3137097	11.3216000	103.9799957
-5.3137097	11.3704004	103.4199982
-5.3137097	11.4191999	102.3499985
-5.3137097	11.4680004	104.7999954
-5.3137097	11.5167999	104.6599960
-5.3137097	11.5656004	102.9000015
-5.3137097	11.6143999	99.6199951
-5.3137097	11.6632004	101.3799973
-5.3137097	11.7119999	104.3499985
-5.3137097	11.7608004	104.2299957
-5.3137097	11.8095999	104.1599960
-5.3137097	11.8584003	105.0100021
-5.3137097	11.9071999	105.3499985
-5.3137097	11.9560003	104.0499954
-5.3137097	12.0047998	102.7299957
[truncated: 467,505 more chars]
